# The Conserved YPX_3_L Motif in the BK Polyomavirus VP1 Protein Is Important for Viral Particle Assembly but Not for Its Secretion into Extracellular Vesicles

**DOI:** 10.3390/v16071124

**Published:** 2024-07-13

**Authors:** Marine Bentz, Louison Collet, Virginie Morel, Véronique Descamps, Emmanuelle Blanchard, Caroline Lambert, Baptiste Demey, Etienne Brochot, Francois Helle

**Affiliations:** 1 UR UPJV4294, Agents Infectieux, Résistance et Chimiothérapie (AGIR), Centre Universitaire de Recherche en Santé, Université de Picardie Jules Verne, 80000 Amiens, Francelouison.collet@u-picardie.fr (L.C.); morel.virginie@chu-amiens.fr (V.M.); descamps.veronique@chu-amiens.fr (V.D.); demey.baptiste@chu-amiens.fr (B.D.); brochot.etienne@chu-amiens.fr (E.B.); 2 Laboratoire de Virologie, Centre Hospitalier Universitaire, 80054 Amiens, France; 3 INSERM U1259, Université de Tours et CHU de Tours, 37032 Tours, France; emmanuelle.blanchard@univ-tours.fr; 4 Plateforme IBiSA de Microscopie Electronique, Université de Tours et CHU de Tours, 37032 Tours, France

**Keywords:** BKPyV, extracellular vesicles, late domain, ESCRT, capsid assembly

## Abstract

The BK polyomavirus (BKPyV) is a small DNA non-enveloped virus whose infection is asymptomatic in most of the world’s adult population. However, in cases of immunosuppression, the reactivation of the virus can cause various complications, and in particular, nephropathies in kidney transplant recipients or hemorrhagic cystitis in bone marrow transplant recipients. Recently, it was demonstrated that BKPyV virions can use extracellular vesicles to collectively traffic in and out of cells, thus exiting producing cells without cell lysis and entering target cells by diversified entry routes. By a comparison to other naked viruses, we investigated the possibility that BKPyV virions recruit the Endosomal-Sorting Complexes Required for Transport (ESCRT) machinery through late domains in order to hijack extracellular vesicles. We identified a single potential late domain in the BKPyV structural proteins, a YPX_3_L motif in the VP1 protein, and used pseudovirions to study the effect of point mutations found in a BKPyV clinical isolate or known to ablate the interaction of such a domain with the ESCRT machinery. Our results suggest that this domain is not involved in BKPyV association with extracellular vesicles but is crucial for capsomere interaction and thus viral particle assembly.

## 1. Introduction

The BK polyomavirus (BKPyV) is a widely distributed ubiquitous virus [1,2]. The seroprevalence is more than 80% of the world adult population over the age of 21 [3]. The primary infection asymptomatically occurs during childhood and infection quietly persists in the kidney and urogenital tract epithelial cells. To date, mechanisms of persistence and immune evasion remain poorly understood. In immunocompromised patients, particularly in cases of transplants, the virus can be reactivated and cause various complications. Up to 10% of kidney transplant recipients will be affected by BK virus nephropathy in the first two years after transplantation, which frequently leads to graft loss [4,5]. Furthermore, hemorrhagic cystitis occurs in up to 15% of bone marrow transplant recipients. Unfortunately, no specific antiviral against BKPyV has been approved yet and the only therapeutic option is the modulation of the immunosuppressive drug regimen to improve immune control and reduce BKPyV disease though it may increase the inherent risk of rejection [4].

BKPyV is a small DNA non-enveloped virus with a diameter of ~45 nm [6,7] and a density of 1.34 or 1.18 g/mL in cesium chloride or iodixanol gradients, respectively [8,9]. The genome is enclosed in a capsid composed of the major viral protein (VP1) associated with the minor viral proteins (VP2 and VP3) in the inner part of the particles. BKPyV icosahedral capsid consists of 360 VP1 molecules organized into 72 pentamers stabilized by intra- and inter-pentameric disulphide bonds as well as Ca^2+^ cations [6,7]. Sixty of the seventy-two pentamers are coordinated with six adjacent pentamers and the other twelve pentamers are located on the icosahedral vertices of the capsid, coordinated with five surrounding pentamers. Neighboring pentamers are linked by invading arms at the C-terminal of VP1, with each capsomere donating five arms and likewise receiving five arms from five neighboring pentamers [6,7]. It has long been known that naked virions are released after cell lysis, but recently, several studies showed that the BKPyV, as well as the JC Polyomavirus (JCPyV, another member of the *Polyomaviridae* family), could be released non-lytically within extracellular vesicles (EVs) [9,10,11]. However, the secretory pathway by which the BKPyV associates with EVs remains to be elucidated.

EVs are lipid bilayer-delimited particles that are physiologically released from cells and carry proteins, nucleic acids, lipids, or metabolites to recipient cells [12,13,14,15]. They are classified according to their cellular origin: (i) microvesicles directly bud from the plasma membrane, (ii) exosomes come from the fusion of multivesicular bodies with the plasma membrane, (iii) large vesicles can also be released after fusion of double-membraned autophagosomes with the plasma membrane [12,13,14,15]. EVs usually require the Endosomal-Sorting Complexes Required for Transport (ESCRT) to be formed [16,17,18]. This machinery consists of the ESCRT-I, ESCRT-II and ESCRT-III complexes that bud membranes and sever membrane necks from their inner face. ESCRT-I in concert with ESCRT-II initiates membrane bud formation. Eventually, the newly formed vesicle is pinched off by the action of ESCRT-III filaments which assemble through activation by membrane-associated ESCRT-II. The ALIX protein (also known as PDCD6IP) forms an alternative pathway to that of ESCRT-I–II for the recruitment and activation of ESCRT-III.

In addition to their roles in communication and cell transport, EVs can promote the transmission of infection or facilitate the spread of pathogens such as viruses. In particular, EVs enable several naked viruses to exit host cells through a non-lytic pathway, to diversify their transmission routes and enhance their fitness while evading the immune system, as shown for some members of the *Picornaviridae*, *Hepeviridae*, *Reoviridae* or *Caliciviridae* families [19,20,21,22,23,24,25,26,27,28]. Some of them, for instance Hepatitis A and E Viruses (HAV and HEV), directly hijack the ESCRT machinery. As for enveloped virus budding, they recruit ESCRT proteins by means of “late domain” motifs (with reference to late steps of the viral cycle) located in viral structural proteins [29,30,31,32,33,34,35,36]. Three classes of late motif have been identified: PPXY, P(S/T)AP and YPXL or YPX_3_L [29,30,31].

Here, we investigated the possible recruitment of the ESCRT machinery by BKPyV virions to hijack EVs. To this end, we examined BKPyV structural protein sequences looking for potential late domains and identified a single YPX_3_L motif located in the VP1 protein. Since this motif is used by several viruses to recruit the ESCRT machinery through the ALIX protein [30], we studied the effect of several mutations on BKPyV pseudovirion production and secretion. Our results suggest that this domain is not involved in ESCRT machinery recruitment for BKPyV association with EVs but is crucial for BKPyV viral particle assembly.

## 2. Materials and Methods

### 2.1. Cell Cultures, Antibodies and Plasmids

The human embryonic kidney HEK293TT cells, stably transfected with pTIH plasmid containing the coding cDNA for SV40 TAg, were kindly provided by C.B. Buck (National Cancer Institute, Bethesda, MD, USA). Cells were cultured in Dulbecco’s modified Eagle’s medium (Invitrogen, Carlsbad, CA, USA) supplemented with 10% fetal bovine serum (Invitrogen). Cells were cultured in a humidified environment with 5% CO_2_ at 37 °C. The 3B2 monoclonal anti-BKPyV VP1 antibody was purchased from Abnova (1:1000; Taipei, Taïwan). The anti-SV40 VP2/3 antibody was purchased from Abcam (1:1000; Cambridge, UK). The 6C5 monoclonal anti-GAPDH antibody was purchased from Santa Cruz Biotechnology (1:1000; Dallas, TX, USA). The rabbit anti-mouse IgG (whole molecule) peroxidase-labeled and the goat anti-rabbit IgG (whole molecule) peroxidase-labeled antibodies were obtained from Sigma (1:10,000; Saint-Louis, MO, USA). The pIaw, ph2b and ph3b plasmids, respectively encoding the genotype Ia VP1, genotype IV VP2 and genotype IV VP3 proteins, were kindly provided by C.B. Buck (National Cancer Institute, Bethesda, MD, USA). The pGL4.13 vector encoding the luciferase reporter gene *luc2* (*Photinus pyralis*) was obtained from Promega (Promega, Madison, WI, USA).

### 2.2. Site-Directed Mutagenesis

Y300A and L305A mutations were introduced in the pIaw plasmid using the QuickChange II XL Site-Directed Mutagenesis Kit (Agilent Technologies, Santa Clara, CA, USA) following the manufacturer’s protocol with the following mutagenic primers: Y300A-Fwd 5′-CGCAGCGTGAAGAATCCCGCTCCAATCAGTTTCCTGCT-3′, Y300A-Rev 5′-AGCAGGAAACTGATTGGAGCGGGATTCTTCACGCTGCG-3′, L305A-Fwd 5′-ATCCCTATCCAATCAGTTTCGCGCTGTCCGATTTGATCAATC-3′, L305A-Rev 5′-GATTGATCAAATCGGACAGCGCGAAACTGATTGG-ATAGGGAT-3′. VP1 nucleotide sequences were verified for each mutant. The plasmid pIaw-P301L, thus encoding the MM strain VP1 protein (accession number CAA24307.1), was obtained from Invitrogen GeneArt services (Invitrogen GeneArt services, Waltham, MA, USA).

### 2.3. Pseudovirion Production and Infection

Twenty-four hours before transfection, 2.10^6^ HEK293TT cells were plated on 75 cm^2^ flasks. pIaw (with or without mutation), ph2b, ph3b and pGL4.13 plasmids were co-transfected (ratio 2:1:1:1) into cells using JetOptimus^®^ (Polyplus, Ilkirch, France) transfection reagent, according to the supplier’s recommendations. Supernatants (10 mL) were harvested 72 h after transfection, centrifuged at 1500 g for 5 min and stored at 4 °C while cells were resuspended with trypsin (Sigma, Saint-Louis, MO, USA). Afterwards, cells were pelleted by centrifugation, resuspended in 10 mL sterile Phosphate-Buffered Saline (PBS) and lysed by 3 freeze/thaw cycles. Cell debris was removed by centrifugation and the supernatants were recovered to evaluate the presence of intracellular infectious pseudovirions. Extracellular and intracellular infectivities were evaluated by measuring Luciferase activity 72 h after inoculation of naive HEK293TT cells with cell supernatants or cell lysates, using the Luciferase Assay System kit (Promega, Madison, WI, USA) and a Centro XS^3^ LB 960 (Berthold Technologies, Bad Wildbad, Germany) luminometer, following the instructions of manufacturers.

### 2.4. Reporter Plasmid Quantification by Real-Time PCR

The number of Luciferase plasmid copies was quantified using a real-time PCR with Taqman reagents (Applied Biosystems^®^, Waltham, MA, USA). Briefly, PCR assays were performed with a final volume of 25 μL containing 12.5 μL of 2x Taqman universal PCR Master Mix and 5 μL of intracellular DNA or extracellular pseudovirions treated with DNase I following the supplier’s instructions (Thermo Fisher Scientific, Waltham, MA, USA), in order to remove non-encapsidated DNA. Forward primer (5′-CATCTTCGGCAACCAGATCAT-3′; 600 nM), reverse primer (5′-CGAAAGCCGCAGATCAAGTAG-3′; 600 nM) and Luc-QSY probe (6FAM-ACCGCTATCCTCAGCGTGGTGCC-3′; 300 nM) were also included in the PCR mix reaction. Amplification and detection were performed on the ABI PRISM 7900 real-time PCR machine (Applied Biosystems^®^, Waltham, MA, USA) using the following protocol: 2 min at 50 °C, 15 min at 95 °C followed by 40 cycles (95 °C for 15 s and 60 °C for 60 s). Signals were measured and analyzed with SDS 2.4.1 software. Reporter plasmid copy numbers were calculated using a standard curve established with serial dilutions of the purified pGL4.13 plasmid and are expressed as copies per reactions.

### 2.5. Buoyant Density Iodixanol Gradient Ultracentrifugation

The supernatants of transfected cells were overlaid on iodixanol gradients formed by equal-volume (2.4 mL) steps of 20, 25, 30, 35 and 40% (wt/vol) iodixanol (Visipaque, 320 mg/mL, GE Healthcare, Chicago, IL, USA) solutions in PBS. Equilibrium was reached by ultracentrifugation for 24 h at 130,000× *g* in a SW32.1 Ti rotor at 4 °C in a Beckman Optima L-100 K BioSafe ultracentrifuge. Seventeen fractions (1 mL) were collected from the top. The density (g/mL) of each fraction was calculated according to the optical density at 340 nm. The infectivity of each fraction was assessed as described above.

### 2.6. Neuraminidase Treatment

Cells were resuspended and treated with increasing concentrations of type V Neuraminidase from *Clostridum perfringens* (Sigma, Saint-Louis, MO, USA) for 30 min at 37 °C under agitation. After washing, cells were incubated for 48 h with EV-associated or naked pseudovirions obtained from iodixanol gradient (fractions 5–6 or 12–13, respectively). Infectivity was evaluated by measuring Luciferase activity, as described above.

### 2.7. Disruption of Pseudovirions into Pentamers

Pseudovirions were disrupted into pentamers by EGTA and dithiothreitol (DTT) treatment [37]. Five hundred microliters of purified pseudovirions were incubated in 50 mM Tris–HCl buffer (pH 7.5) containing 150 mM NaCl, 1 mM EGTA and 20 mM DTT in a final volume of 250 µL, at room temperature for 30 min. Then, pentamers were separated in iodixanol gradients as described above.

### 2.8. Western Blotting

All samples used were stored in Laemmli buffer (Sigma, Saint-Louis, MO, USA) and boiled at 95 °C for 5 min. Proteins were separated by SDS-PAGE and transferred to nitrocellulose membranes (Bio-Rad, Hercules, CA, USA) by the semi-dry transfer method. Blots were blocked in PBS containing 0.1% Tween 20 (VWR Chemicals, Radnor, PA, USA) and 5% dried milk for 1 h at room temperature. The primary antibodies were diluted in PBS containing 0.1% Tween 20, 5% Bovine Serum Albumin (VWR Chemicals, Radnor, PA, USA) and 0.05% azide and incubated overnight at 4 °C. After three washes with PBS containing 0.1% Tween 20, blots were incubated for 1 h with the peroxidase-labeled secondary antibody diluted in PBS containing 0.1% Tween 20 and 5% dried milk. The peroxidase-positive signals were visualized after a 5 min incubation in 0.1 M Tris buffer (pH 8.5) containing 225 µM P-coumaric acid, 1.25 mM Luminol and 0.009% H_2_O_2_. Band intensities were quantified using ImageJ (https://imagej.net/ij/ (accessed on 19 February 2022)).

### 2.9. Electron Microscopy

Pseudoviral particles contained in the supernatants of infected cells were concentrated 30× using PEG precipitation. Briefly, 150 mL of cell supernatant was mixed with 37.5 mL of a PEG 6000 solution (40% in PBS). The mixtures were incubated overnight at 4 °C and centrifuged at 1500× *g*, 4 °C, for 30 min. The pellets were resuspended in 5 mL of PBS and fractionated by iodixanol gradient as described above. Cells were trypsinized and incubated with the fractions of interest for 2 h at 37 °C, under gentle shaking. Then, they were washed with PBS, fixed in 4% paraformaldehyde and 1% glutaraldehyde in 0.1 M phosphate buffer (pH 7.2) for 48 h, and postfixed with 2% osmium tetroxide (Electron Microscopy Sciences, Hatfield, PA, USA) for 1 h. They were then dehydrated in a graded series of ethanol solutions, and cell pellets were embedded in Epon resin, which was allowed to polymerize for 48 h at 60 °C. Ultrathin sections were cut on a UC7 ultramicrotome (Leica Microsystems, Vienna, Austria), collected on a 200 mesh gold grid, and stained with 2.5% uranyl acetate—5% lead citrate. The grids were observed with a JEOL JEM-1011 electron microscope (JEOL, Tokyo, Japan) connected to a Gatan Rio 9 digital camera driven by Digital Micrograph software (Gatan, Pleasanton, CA, USA).

### 2.10. Statistical Analyses

Statistical analyses were performed using GraphPad Prism version 8.0.0 for Windows (GraphPad Software, San Diego, CA, USA). Student’s unpaired *t*-test was carried out to compare results between wild type and mutant models. The threshold for statistical significance was set to *p* < 0.05.

## 3. Results

### 3.1. A YPX_3_L Motif Is Conserved in the VP1 Protein Sequence of Not Only the BK Polyomavirus but Also Most Other Human Polyomaviruses

**Figure 1:**
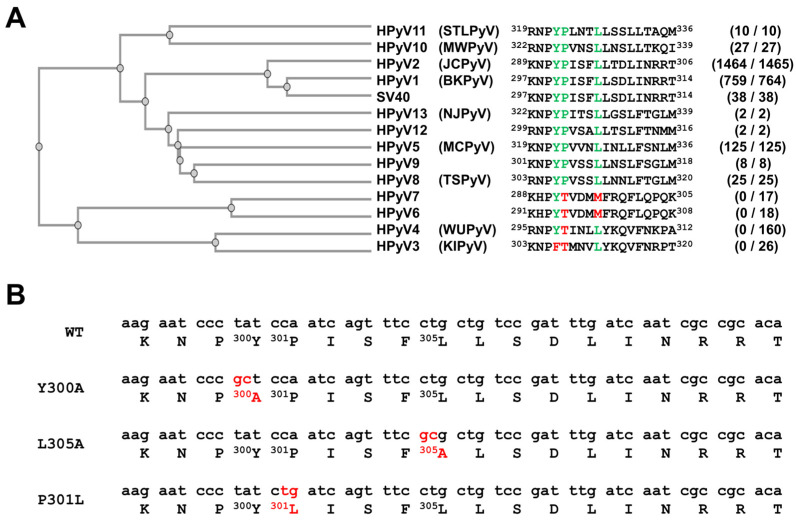
Conservation of the YPX_3_L in the VP1 sequence of human polyomaviruses and mutagenesis of this motif in the BKPyV VP1 sequence of the pIaw plasmid. (**A**) A phylogenetic tree obtained after alignment of human polyomavirus VP1 protein consensus sequences, using Clustal Omega (https://www.ebi.ac.uk/Tools/msa/clustalo/ accessed on 5 April 2024), is presented on the left. The alignment of human polyomavirus VP1 protein consensus sequences next to the motif is shown in the middle. The amino acids of the YPX_3_L motif are shown in green or red depending on whether they are present or not in the consensus sequence, respectively. For each human polyomavirus, the conservation of YPX_3_L is indicated in parentheses (“number of sequences in which the motif is present/number of analyzed sequences”). (**B**) Three plasmids encoding VP1 with mutation Y300A, L305A or P301L were used in this study. For each construct, the nucleotide and protein sequences are shown on the top and the bottom, respectively. Nucleotides that have been mutated are shown in red.

In order to identify potential late domains in the BKPyV capsid proteins, we analyzed their sequences and looked for PPXY, P(S/T)AP, YPXL or YPX_3_L motifs. No PPXY, P(S/T)AP and YPXL motifs were identified but we found a YPX_3_L motif between residues 300 and 305 of the BKPyV VP1 protein. More interestingly, we observed that this motif was highly conserved, not only in BKPyV (759 out of 764 sequences) but also in JCPyV (1464 out of 1465 sequences), HPyV5, HPyV8, HPyV9, HPyV10, HPyV11, HPyV12, and HPyV13, as well as SV40 VP1 sequences (Figure 1A and Supplementary Data S1). In contrast, this motif was not present in HPyV3, HPyV4, HPyV6 and HPyV7 VP1 protein sequences. To investigate whether this motif is involved in the secretion of infectious particles into EVs, we decided to produce pseudovirions harboring mutations in this motif. Two different mutations, Y300A and L305A (Figure 1B), were introduced into the VP1-expressing plasmid used for pseudovirion production, since similar mutations were shown to be associated with reduced binding of HAV particles to the ALIX ESCRT protein and thus reduced envelopment into EVs [32]. We also studied the effect of the P301L mutation, which is found in the BKPyV MM strain (accession number CAA24307.1).

### 3.2. Mutations of the BKPyV VP1 YPX_3_L Motif Impair the Assembly of Viral Particles

**Figure 2:**
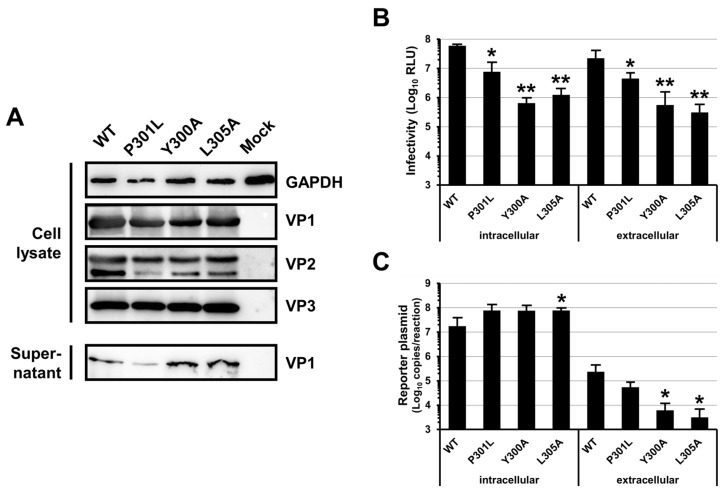
Production of WT, P301L, Y300A and L305A pseudovirions. HEK293TT cells have been transfected with WT, P301L, Y300A and L305A VP1 constructs, as well as plasmids expressing the VP2, VP3 and Luciferase proteins. (**A**) The presence of the VP1 (42 kDa), VP2 (38 kDa) and VP3 (27 kDa) proteins in cell lysates and supernatants was verified by Western blot using the 3B2 monoclonal antibody and a polyclonal anti-SV40 VP2 and VP3 antibody. The GAPDH (36 kDa) was used as a loading control. A representative experiment is shown. (**B**) Naive HEK293TT cells were inoculated with pseudovirions present in the supernatant (extracellular) or into the producing cells (intracellular). Inoculated cells were incubated for 72 h before measuring Luciferase activity. Results are expressed as Relative Light Units (RLUs) and reported as means ± standard deviations of at least three independent experiments. (**C**) The amount of extracellular encapsidated reporter plasmid was quantified by real-time PCR. The amounts of reporter plasmid found intracellularly were determined to ensure that the transfection efficiency was similar in each condition. Results are expressed as copies per reaction and reported as means ± standard deviations of three independent experiments. * *p* < 0.05, ** *p* < 0.01.

HEK293TT cells were co-transfected with VP1-, VP2-, VP3- and Luciferase-expressing plasmids in order to produce pseudovirions. As shown in Figure 2A, the Y300A, L305A and P301L mutations did not have a dramatic effect on the VP1, VP2 and VP3 expression and stability since similar amounts of the proteins were detected for the WT and the mutants in the cell lysates. In contrast, we observed that the Y300A and L305A mutations strongly reduced the extracellular infectivity with 1.6 and 1.9 log reductions, respectively, while P301L showed an intermediate decrease with a 0.7 log reduction (Figure 2B). These results indicated that the three mutations affected the assembly, the secretion and/or the specific infectivity of the viral particles. However, our results also suggested that a defect in secretion was unlikely since similar amounts of VP1 were detected in the cell supernatants for the WT and the mutants with the exception of a slight decrease for P301L (Figure 2A). We also tested the infectivity of the viral particles present inside the transfected cells and observed a 0.9, 2 and 1.7 log reduction for the P301L, Y300A and L305A mutations as compared to the WT, respectively (Figure 2B). This result further argued against a defect in viral particle secretion but did not permit the discrimination between a defect in the viral particle assembly or a decrease in the viral particle specific infectivity. Unfortunately, we could not confirm that VP2/VP3 were correctly incorporated into the extracellular capsids because of a lack of Western blot sensitivity for these proteins in the cell supernatants. However, we quantified the amount of extracellular encapsidated reporter plasmid (Figure 2C) and observed that it strongly correlated with the extracellular infectivity (Figure 2B), showing a 0.6, 1.6 and 1.9 log reduction for P301L, Y300A and L305A as compared to the WT, respectively. Similar amounts of the reporter plasmid were found intracellularly, confirming that the transfection was performed similarly for the WT and each mutant (Figure 2C). Altogether, these results suggested that the specific infectivity of the pseudovirions was not affected by the different mutations and pointed to an effect on particle assembly. They also suggested that VP1 might be secreted without being incorporated into infectious pseudoviral particles, particularly for the Y300A and L305A mutants, which showed similar amounts of extracellular VP1 but decreased amounts of encapsidated reporter plasmid as compared to the WT.

### 3.3. Mutations of the BKPyV VP1 YPX_3_L Motif Do Not Impair the Association of Infectious Particles with EVs

**Figure 3:**
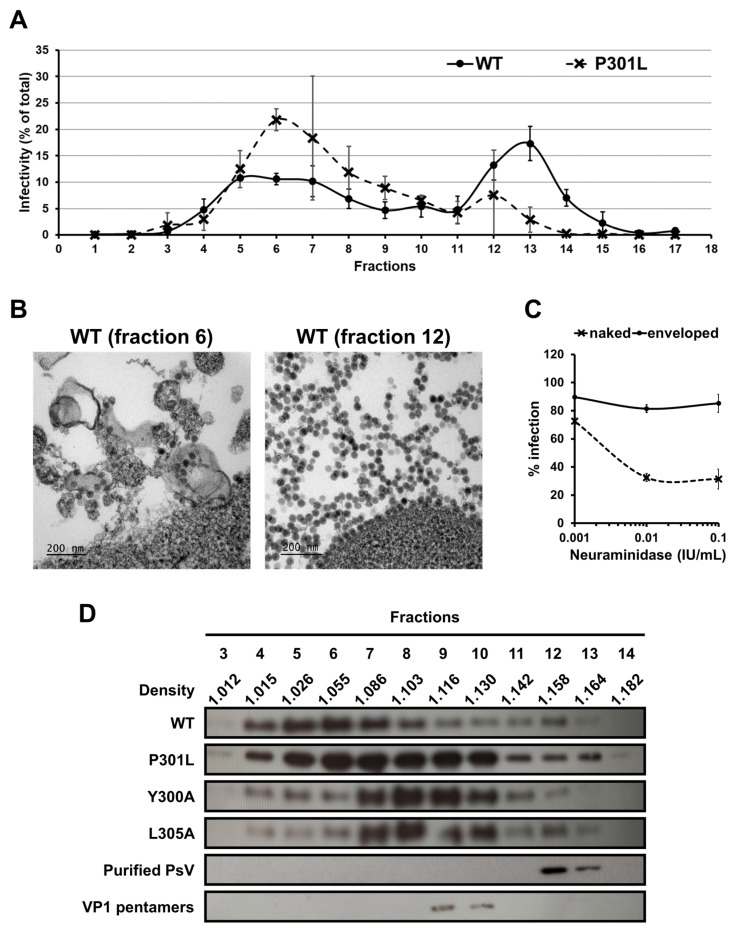
Association of infectious pseudovirions with EVs. (**A**) Supernatants of pseudovirions producing cells were harvested 3 days post-transfection, centrifuged to remove cell debris, and overlaid on a 20% to 40% (wt/vol) iodixanol gradient. After a 24 h ultracentrifugation, 17 fractions were collected. Presence of infectious pseudovirions in each fraction was assessed by measuring Luciferase activity 3 days after inoculation of naive HEK293TT cells. This is expressed as percentage of total infectivity (RLU in the fraction/sum of RLU in all fractions) and reported as means ± standard deviations of three independent experiments. The results are shown only for WT and P301L pseudovirions since Y300A and L305A pseudovirion infectivity was too low to obtain reproducible measurements. (**B**) HEK293TT cells were incubated for 2 h with fractions containing EV-associated or naked pseudovirions, fixed, and processed for electron microscopy. Electron micrographs of EV-associated and naked pseudovirion particles are shown on the left and right, respectively. (**C**) Naive HEK293TT cells were resuspended and treated with increasing concentrations of type V Neuraminidase for 30 min. After washing, they were inoculated with enveloped (fractions 5 and 6) or naked (fraction 12 and 13) WT pseudovirions. Infectivity was assessed by measuring Luciferase activity 2 days after inoculation. Results are expressed as percentage of infection as compared to infection of untreated cells and reported as means ± standard deviations of three independent experiments. (**D**) The presence of the VP1 protein in the different fractions was evaluated by Western blot using the 3B2 monoclonal antibody. Three independent experiments have been performed and a representative one is shown. The density (g/mL) of each fraction was calculated according to the optical density at 340 nm and is reported as means of three independent experiments. Purified naked pseudovirions and VP1 pentamers were also separated in iodixanol gradients to identify the fractions in which they respectively sedimented.

To investigate whether the P301L, Y300A and L305A mutations impaired the association of infectious particles with EVs, we separated the viral particles according to their densities using iodixanol gradients. As described for native BKPyV virions [9], we observed two populations of infectious particles for the WT and P301L pseudovirions (Figure 3A), the first one with a density ranging between 1.01 and 1.13 g/mL (fractions 4 to 10) and the second with a density around 1.16 g/mL (fractions 12 and 13). In addition, though the Y300A and L305A pseudovirion infectivity was too low to obtain reproducible measurements, we sometimes detected the presence of infectious pseudovirions, in particular in the low-density fractions. Through electron microscopy using the WT samples, we confirmed that naked particles were found in fraction 12 whereas fraction 6 contained EV-associated pseudovirions, either within the EVs or stuck at their surface (Figure 3B). In contrast, it was extremely difficult to detect viral particles by electron microscopy using the Y300A mutant, further suggesting that mutated capsid proteins could be secreted without being incorporated into capsids. We also confirmed that the EV-associated pseudovirion infectivity was only slightly affected by target cell Neuraminidase treatment, contrary to the naked pseudovirions, which were strongly affected (Figure 3C). Together, these results indicated that the mutations do not prevent the association of pseudovirions with EVs and further suggested that the mutations are responsible for a viral particle assembly defect.

### 3.4. The P301L, Y300A and L305A Mutations in the BKPyV VP1 Protein May Impair the Assembly of VP1 Pentamers into Capsid

We also sought to detect the presence of the VP1 protein in the fractions obtained after fractionation in iodixanol gradients (Figure 3D). As shown for the native BKPyV virions [9], we observed that most of the VP1 proteins were present in the low-density fractions (77% of the total VP1 in fractions 4 to 8) when using the WT pseudovirion-producing cell supernatant. The amount of VP1 in fractions 12 and 13 was very low as compared to fractions 4 to 10, suggesting that the specific infectivity of the naked particles is higher than that of the EV-associated particles. Alternatively, it is possible that a certain amount of VP1 in fractions 4 to 10 was not incorporated into full particles. In contrast, when using the Y300A and L305A mutants, we evidenced that the VP1 proteins were mainly in fractions 7 to 10 (68 and 70% of total VP1 for the Y300A and L305A, respectively). The P301L mutant showed a mixed profile with the VP1 proteins mainly distributed in fractions 4 to 10 (12 ± 3% of total VP1 in each of these fractions).

Since our results suggested that the mutations impair capsid assembly and cause the secretion of capsid proteins not incorporated into particles, we hypothesized that the shift of VP1 proteins observed in the iodixanol gradient with the mutants as compared to the WT may correspond to the secretion of VP1 as pentamers. To test this hypothesis, we concentrated the naked pseudovirions and treated them with EGTA and DTT in order to disrupt the viral capsids into pentamers, as previously described for several polyomaviruses [37,38,39]. Then, we carried out iodixanol gradients with purified naked pseudovirions or pentamers. As shown in Figure 3D, the purified pseudovirions peaked in fractions 12 and 13 whereas the pentamers peaked in fractions 9 and 10. These results supported our hypothesis that the Y300A and L305A mutants were secreted mainly as pentamers and rarely as infectious particles while the P301L mutation led to the secretion of VP1 pentamers as well as infectious particles. However, these results must be interpreted very cautiously because of the multiple parameters that influence the density, such as the incorporation into particles which can encapsidate a reporter plasmid or not, as well as the association with EVs with variable amounts of proteins and lipids.

**Figure 4:**
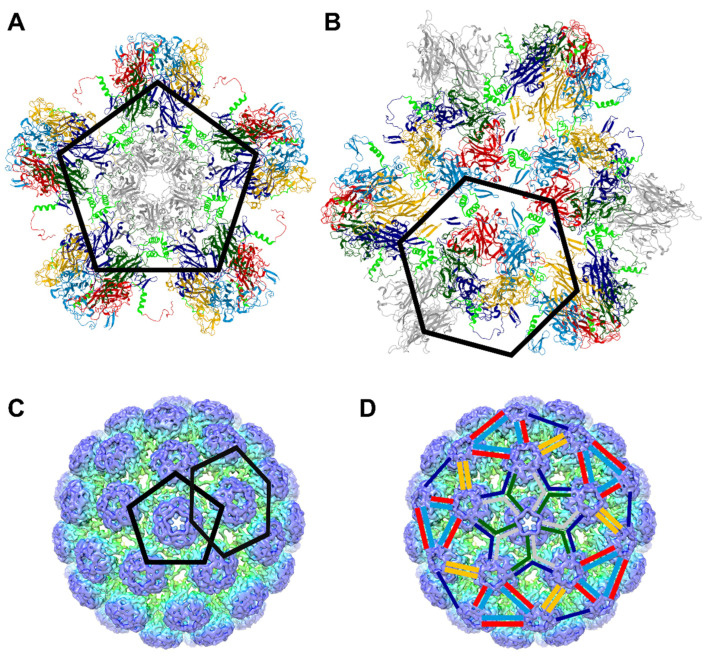
Location of the Y300, P301 and L305 amino acids in the structure of BKPyV viral capsid. (**A**,**B**) VP1 monomers of pentavalent pentamers are shown in gray whereas VP1 monomers of hexavalent pentamers are shown in red, green, blue, cyan and yellow. The amino acids 297 to 314 of each VP1 monomer, encompassing the “pentapeptide hinge” (297–301) and the C helix (302–314) are highlighted in light green. Images were created using Mol* Viewer [42] and obtained from the Research Collaboratory for Structural Bioinformatics Protein Data Bank (rcsb.org) [43] (PDB ID: 6ESB [6]). (**C**) The respective positions of pentavalent and hexavalent pentamers on the viral capsid structure are highlighted at the center of a pentamer and a hexamer, respectively. (**D**) The C-terminal arms of VP1 monomers are highlighted using the color code used in panels A and B. Panels C and D have been produced from https://pdbj.org/emnavi/data/emdb/media/3283/images/emd_3283.tif.jpg (accessed on 19 February 2022) [7,44].

To better understand the effect of these mutations on viral particle assembly, we explored the location of the corresponding amino acids in the BKPyV viral particle structure (Figure 4). We observed that the YPX_3_L motif is present in the region that connects the core of VP1 to its invading arm. In particular, the Y300 and P301 amino acids are present in the motif KNPYP, which has been called the “pentapeptide hinge” [40,41] and the L305 amino acid is present in a sequence referred to as the C helix that can form α-helices (Figure 4A,B; see gray, blue, green, red and cyan VP1 monomers) or be disordered on some subunits (Figure 4A,B; see yellow VP1 monomers). This region is involved in trimerization (Figure 4A; see interactions between blue, green and gray monomers) or dimerization (Figure 4B; see interactions between red and cyan VP1 monomers as well as between yellow VP1 monomers). These observations argue for a major role of Y300, P301 and L305 in the assembly of pentamers as viral capsids (Figure 4C,D).

## 4. Discussion

In this study, we investigated the potential recruitment of the ESCRT machinery by BKPyV viral particles to be enveloped in EVs. To this end, we looked at BKPyV structural proteins for the amino acid motifs PPXY, P(S/T)AP, YPXL or YPX_3_L, which are known to recruit the ESCRT machinery [29,30,31,32,33,34,35]. We identified in the VP1 capsid protein a single YPX_3_L motif that is known to enable the binding of cellular and viral proteins to the ALIX ESCRT protein [16,29,30,31,32,45,46]. Though this motif is expected to be structurally buried, it could be accessible on a precursor particle and/or before major conformational rearrangements, as proposed for HAV [47]. Interestingly, this motif was highly conserved not only in BKPyV VP1 sequences but also in JCPyV and other HPyV sequences, suggesting a crucial role of this domain. Nevertheless, we also identified five BKPyV sequences where the motif is not present (e.g., MM strain [48]), demonstrating that it is not indispensable for the virus. We decided to introduce Tyr-to-Ala and Leu-to-Ala mutations in this YPX_3_L motif since they have been shown to ablate interactions with ALIX in other studies [32] and also studied the effect of the Pro-to-Leu mutation which is found in the MM strain.

Our first hypothesis was that the YPX_3_L motif could be involved in the recruitment of ALIX in order to bud into EVs. However, our results showed that the P301L mutation does not prevent the production of EV-associated pseudovirions and, even though the Y300A and L305A mutant pseudovirion infectivity was too low to obtain reproducible measurements, we could also detect the presence of infectious pseudovirions in fractions corresponding to EV-associated pseudovirions with these mutations. This result proves that the YPX_3_L motif is not indispensable for the association of BKPyV with EV and strongly suggest that it does not recruit the ALIX protein to carry out this process. In line with this result, it has recently been shown that the knockdown of not only ALIX but also HGS, TSG101, VPS25, VPS20, CHMP4A, and VPS4A ESCRT-related proteins did not significantly reduce the association of JCPyV with EVs, demonstrating that the ESCRT machinery is not involved in the production of EV-associated JCPyV [49]. This study also suggests that the biogenesis of EV-associated JCPyV depends on exosomes and secretory autophagosomes and, since BKPyV and JCPyV share 75% sequence homology, it is likely that BKPyV uses similar mechanisms, but this remains to be demonstrated [49].

Altogether, our results strongly suggest that the Y300, P301 and L305 amino acids are crucial for the assembly of VP1 pentamers into capsids. This result is in line with data obtained with the SV40 model [40,41,50]. Indeed, Y300 and P301 are localized in a region that may be regarded as an extended hinged link between the core of VP1 and its C-terminal arm that invades a surrounding pentamer. During assembly, this hinge appears to function in orienting the arm away from its parent pentamer, facilitating contacts with other pentamers. Interestingly, in the SV40 native virus model, it was shown that the mutations Y299T and Y299A (corresponding to Y300A in our study) were more defective in viability than Y299F and that the aromatic ring contacts the C-arms of surrounding pentamers [41]. Furthermore, they observed that the mutation of P300 (corresponding to P301 in our study) to Glycine was more unstable than a mutation to Alanine, indicating a requirement for the rigidity of the pentapeptide hinge [41]. It was also shown in the SV40 model that L304 (L305 in our study) is present in a peptide referred to as the C helix that can be structured in an α-helix or disordered on some subunits and that is involved in dimerization or trimerization [40,50]. Using SV40 pseudovirions produced in insect cells, it has also been shown that an L304W mutation in VP1 (corresponding to L305 in our study) leads to the formation of spherical particles with a smaller diameter than normal [51]. In these studies, the interactions between the C helices were shown to be mediated by a range of hydrophobic residues, including this leucine residue [40,41,51]. Altogether, our results suggest that the YPX_3_L motif in the BK polyomavirus VP1 protein is important for viral particle assembly and argue against the potential involvement of this motif in the recruitment of the ALIX protein for the secretion of BKPyV viral particles into EVs. Interestingly, these results support the study of Osipov et al., who identified this region as a druggable pocket to develop capsid assembly inhibitors [52].

## Data Availability

Raw data are available on request.

## Supplementary Materials

The following supporting information can be downloaded at: https://www.mdpi.com/article/10.3390/v16071124/s1, Supplementary Data S1: HPyV VP1 alignments (NCBI Virus—April 2024).
