# Supplementary material for: The Conserved YPX3L Motif in the BK Polyomavirus VP1 Protein Is Important for Viral Particle Assembly but Not for Its Secretion into Extracellular Vesicles
Source: Viruses. 2024 Jul 13;16(7):1124. doi: 10.3390/v16071124 (PMC11281352; doi:10.3390/v16071124)
Supplement: Supplementary file 1 [file viruses-16-01124-s001.zip › HPyV 01 alignment.pdf]

CLUSTAL O(1.2.4) multiple sequence alignment

```
ALF06344.1          MAPTKRKGECPGAAPKKPKEPVQVPKLLIKGGVEVLEVKTGVDAITEVECFLNPEMGDPD  60
QTJ15012.1          MAPTKRKGECPGAAPKKPKEPVQVPKLLIKGGVEVLEVKTGVDAITEVECFLNPEMGDPE  60
QTJ15120.1          MAPTKRKGECPGAAPKKPKEPVQVPKLLIKGGVEVLEVKTGVDAITEVECFLNPEMGDPE  60
QHI34657.1          MAPTKRKGECPGAAPKKPKEPVQVPKLLIKGGVEVLEVKTGVDAITEVECFLNPEMGDPD  60
ARE72390.1          MAPTKRKGECPGAAPKKPKEPVQVPKLLIKGGVEVLEVKTGVDAITEVECFLNPEMGDPD  60
QTJ14970.1          MAPTKRKGECPGAAPKKPKEPVQVPKLLIKGGVEVLEVKTGLDAITEVECFLNPEMGDPD  60
QTJ14928.1          MAPTKRKGECPGAAPKKPKEPVQVPKLLIKGGVEVLEVKTGVDAITEVECFLNPEMGDPE  60
AAT47419.1          MAPTKRKGECPGAAPKKPKEPVQVPKLLIKGGVEVLEVKTGVDAITQVECFLNPEMGDPD  60
AAT47413.1          MAPTKRKGECPGAAPKKPKEPVQVPKLLIKGGVEVLEVKTGVDAITEVECFLNPEMGDPD  60
AFQ31661.1          MAPTKRKGECPGAAPKKPKEPVQVPKLLIKGGVEVLEVKTGVDAITEVECFLNPEMGGPD  60
YP_717939.1         MAPTKRKGECPGAAPKKPKEPVQVPKLLIKGGVEVLEVKTGVDAITEVECFLNPEMGDPD  60
AFK08545.1          MAPTKRKGECPGAAPKKPKEPVQVPKLLIKGGVEVLEVKTGVDAITEVECFLNPEMGDPD  60
P03088.2            MAPTKRKGECPGAAPKKPKEPVQVPKLLIKGGVEVLEVKTGVDAITEVECFLNPEMGDPD  60
CAA24299.1          MAPTKRKGECPGAAPKKPKEPVQVPKLLIKGGVEVLEVKTGVDAITEVECFLNPEMGDPD  60
QTJ14922.1          MAPTKRKGECPGAAPKKPKEPVQVPKLLIKGGVEVLEVKTGVDAITEVECFLNPEMGDPD  60
AFA41877.1          MAPTKRKGECPGAAPKKPKEPVQVPKLLIKGGVEVLEVKTGVDAITEVECFLNPEMGDPN  60
AAT47371.1          MAPAKRKGECPGAAPKKPKEPVQVPKLLIKGGVEVLEVKTGVDAITEVECFLNPEMGDPD  60
AYO46658.1          MAPTKRKGECPGAAPKKPKEPVQVPKLLIKGGVEVLEVKTGVDAITEVECFLNPEMGDPD  60
AYO46610.1          MAPTKRKGECPGAAPKKPKEPVQVPKLLIKGGVEVLEVKTGVDAITEVECFLNPEMGDPD  60
AYO46689.1          MAPTKRKGECPGAAPKKPKEPVQVPKLLIKGGVEVLEVKTGVDAITEVECFLNPEMGDAD  60
AYO46693.1          MAPTKRKGECPGAAPKKPKEPVQVPKLLIKGGVEVLEVKTGVDAITEVECFLNPEMGDPD  60
BAG75283.1          MAPTKRKGECPGAAPKKPKEPVQVPKLLIKGGVEVLEVKTGVDAITEVECFLNPEMGDPD  60
BAG75313.1          MAPTKRKGECPGAAPKKPKEPVQVPKLLIKGGVEVLEVKTGVDAITEVECFLNPEMGDPD  60
BAG75373.1          MAPTKRKGECPGAAPKKPKEPVQVPKLLIKGGVEVLEVKTGVDAITEVECFLNPEMGDPD  60
BCV68914.1          MAPTKRKGECPGAAPKKPKDPVQVPKLLIKGGVEVLEVKTGVDAITEVECFLNPEMGDPD  60
BCV68917.1          MAPTKRKGECPGAAPKKPKDPVQVPKLLIKGGVEVLEVKTGVDAITEVECFLNPEMGDPD  60
BCV68923.1          MAPTKRKGECPGAAPKKPKDPVQVPKLLIKGGVEVLEVKTGVDAITEVECFLNPEMGDPD  60
AYO46620.1          MAPTKRKGECPGAAPKKPKDPVQVPKLLIKGGVEVLEVKTGVDAITEVECFLNPEMGDPD  60
AYO46646.1          MAPTKRKGECPGAAPKKPKDPVQVPKLLIKGGVEVLEVKTGVDAITEVECFLNPEMGDPD  60
AYO46663.1          MAPTKRKGECPGAAPKKPKDPVQVPKLLIKGGVEVLEVKTGVDAITEVECFLNPEMGDPD  60
AYO46694.1          MAPTKRKGECPGAAPKKPKDPVQVPKLLIKGGVEVLEVKTGVDAITEVECFLNPEMGDPD  60
BAZ99906.1          MAPTKRKGECPGAAPKKPKDPVQVPKLLIKGGVEVLEVKTGVDAITEVECFLNPEMGDPD  60
BAZ99912.1          MAPTKRKGECPGAAPKKPKDPVQVPKLLIKGGVEVLEVKTGVDAITEVECFLNPEMGDPD  60
BAI43576.1          MAPTKRKGECPGAAPKKPKDPVQVPKLLIKGGVEVLEVKTGVDAITEVECFLNPEMGDPD  60
BAI43594.1          MAPTKRKGECPGAAPKKPKDPVQVPKLLIKGGVEVLEVKTGVDAITEVECFLNPEMGDPD  60
BAI43606.1          MAPTKRKGECPGAAPKKPKDPVQVPKLLIKGGVEVLEVKTGVDAITEVECFLNPEMGDPD  60
BAI43624.1          MAPTKRKGECPGAAPKKPKDPVQVPKLLIKGGVEVLEVKTGVDAITEVECFLNPEMGDPD  60
BAH23450.1          MAPTKRKGECPGAAPKKPKDPVQVPKLLIKGGVEVLEVKTGVDAITEVECFLNPEMGDPD  60
BAH23456.1          MAPTKRKGECPGAAPKKPKDPVQVPKLLIKGGVEVLEVKTGVDAITEVECFLNPEMGDPD  60
BAH23462.1          MAPTKRKGECPGAAPKKPKDPVQVPKLLIKGGVEVLEVKTGVDAITEVECFLNPEMGDPD  60
BAH23468.1          MAPTKRKGECPGAAPKKPKDPVQVPKLLIKGGVEVLEVKTGVDAITEVECFLNPEMGDPD  60
BAH23474.1          MAPTKRKGECPGAAPKKPKDPVQVPKLLIKGGVEVLEVKTGVDAITEVECFLNPEMGDPD  60
BAH23480.1          MAPTKRKGECPGAAPKKPKDPVQVPKLLIKGGVEVLEVKTGVDAITEVECFLNPEMGDPD  60
BAH23486.1          MAPTKRKGECPGAAPKKPKDPVQVPKLLIKGGVEVLEVKTGVDAITEVECFLNPEMGDPD  60
BAH23492.1          MAPTKRKGECPGAAPKKPKDPVQVPKLLIKGGVEVLEVKTGVDAITEVECFLNPEMGDPD  60
BAH23498.1          MAPTKRKGECPGAAPKKPKDPVQVPKLLIKGGVEVLEVKTGVDAITEVECFLNPEMGDPD  60
BAH23504.1          MAPTKRKGECPGAAPKKPKDPVQVPKLLIKGGVEVLEVKTGVDAITEVECFLNPEMGDPD  60
BAH23510.1          MAPTKRKGECPGAAPKKPKDPVQVPKLLIKGGVEVLEVKTGVDAITEVECFLNPEMGDPD  60
BAH23516.1          MAPTKRKGECPGAAPKKPKDPVQVPKLLIKGGVEVLEVKTGVDAITEVECFLNPEMGDPD  60
BAH23522.1          MAPTKRKGECPGAAPKKPKDPVQVPKLLIKGGVEVLEVKTGVDAITEVECFLNPEMGDPD  60
BAH23528.1          MAPTKRKGECPGAAPKKPKDPVQVPKLLIKGGVEVLEVKTGVDAITEVECFLNPEMGDPD  60
BAH23534.1          MAPTKRKGECPGAAPKKPKDPVQVPKLLIKGGVEVLEVKTGVDAITEVECFLNPEMGDPD  60
BAH23540.1          MAPTKRKGECPGAAPKKPKDPVQVPKLLIKGGVEVLEVKTGVDAITEVECFLNPEMGDPD  60
BAH23546.1          MAPTKRKGECPGAAPKKPKDPVQVPKLLIKGGVEVLEVKTGVDAITEVECFLNPEMGDPD  60
BAH23552.1          MAPTKRKGECPGAAPKKPKDPVQVPKLLIKGGVEVLEVKTGVDAITEVECFLNPEMGDPD  60
BAH23558.1          MAPTKRKGECPGAAPKKPKDPVQVPKLLIKGGVEVLEVKTGVDAITEVECFLNPEMGDPD  60
BAG84488.1          MAPTKRKGECPGAAPKKPKDPVQVPKLLIKGGVEVLEVKTGVDAITEVECFLNPEMGDPD  60
BAG84494.1          MAPTKRKGECPGAAPKKPKDPVQVPKLLIKGGVEVLEVKTGVDAITEVECFLNPEMGDPD  60
BAG84500.1          MAPTKRKGECPGAAPKKPKDPVQVPKLLIKGGVEVLEVKTGVDAITEVECFLNPEMGDPD  60
BAG84506.1          MAPTKRKGECPGAAPKKPKDPVQVPKLLIKGGVEVLEVKTGVDAITEVECFLNPEMGDPD  60
BAG84512.1          MAPTKRKGECPGAAPKKPKDPVQVPKLLIKGGVEVLEVKTGVDAITEVECFLNPEMGDPD  60
```

BAG84518.1	MAPTKRKGECPGAAPKKPKDPVQVPKLLIKGGVEVLEVKTGVDAITEVECFLNPEMGDPD	60
BAG84524.1	MAPTKRKGECPGAAPKKPKDPVQVPKLLIKGGVEVLEVKTGVDAITEVECFLNPEMGDPD	60
BAG75163.1	MAPTKRKGECPGAAPKKPKDPVQVPKLLIKGGVEVLEVKTGVDAITEVECFLNPEMGDPD	60
BAG75169.1	MAPTKRKGECPGAAPKKPKDPVQVPKLLIKGGVEVLEVKTGVDAITEVECFLNPEMGDPD	60
BAG75175.1	MAPTKRKGECPGAAPKKPKDPVQVPKLLIKGGVEVLEVKTGVDAITEVECFLNPEMGDPD	60
BAG75181.1	MAPTKRKGECPGAAPKKPKDPVQVPKLLIKGGVEVLEVKTGVDAITEVECFLNPEMGDPD	60
BAG75187.1	MAPTKRKGECPGAAPKKPKDPVQVPKLLIKGGVEVLEVKTGVDAITEVECFLNPEMGDPD	60
BAG75193.1	MAPTKRKGECPGAAPKKPKDPVQVPKLLIKGGVEVLEVKTGVDAITEVECFLNPEMGDPD	60
BAG75199.1	MAPTKRKGECPGAAPKKPKDPVQVPKLLIKGGVEVLEVKTGVDAITEVECFLNPEMGDPD	60
BAG75241.1	MAPTKRKGECPGAAPKKPKDPVQVPKLLIKGGVEVLEVKTGVDAITEVECFLNPEMGDPD	60
BAG75247.1	MAPTKRKGECPGAAPKKPKDPVQVPKLLIKGGVEVLEVKTGVDAITEVECFLNPEMGDPD	60
BAG75253.1	MAPTKRKGECPGAAPKKPKDPVQVPKLLIKGGVEVLEVKTGVDAITEVECFLNPEMGDPD	60
BAG75265.1	MAPTKRKGECPGAAPKKPKDPVQVPKLLIKGGVEVLEVKTGVDAITEVECFLNPEMGDPD	60
BAG75289.1	MAPTKRKGECPGAAPKKPKDPVQVPKLLIKGGVEVLEVKTGVDAITEVECFLNPEMGDPD	60
BAG75295.1	MAPTKRKGECPGAAPKKPKDPVQVPKLLIKGGVEVLEVKTGVDAITEVECFLNPEMGDPD	60
BAG75307.1	MAPTKRKGECPGAAPKKPKDPVQVPKLLIKGGVEVLEVKTGVDAITEVECFLNPEMGDPD	60
BAG75325.1	MAPTKRKGECPGAAPKKPKDPVQVPKLLIKGGVEVLEVKTGVDAITEVECFLNPEMGDPD	60
BAG75331.1	MAPTKRKGECPGAAPKKPKDPVQVPKLLIKGGVEVLEVKTGVDAITEVECFLNPEMGDPD	60
BAG75337.1	MAPTKRKGECPGAAPKKPKDPVQVPKLLIKGGVEVLEVKTGVDAITEVECFLNPEMGDPD	60
BAG75343.1	MAPTKRKGECPGAAPKKPKDPVQVPKLLIKGGVEVLEVKTGVDAITEVECFLNPEMGDPD	60
BAG75397.1	MAPTKRKGECPGAAPKKPKDPVQVPKLLIKGGVEVLEVKTGVDAITEVECFLNPEMGDPD	60
BAG75421.1	MAPTKRKGECPGAAPKKPKDPVQVPKLLIKGGVEVLEVKTGVDAITEVECFLNPEMGDPD	60
BAG75427.1	MAPTKRKGECPGAAPKKPKDPVQVPKLLIKGGVEVLEVKTGVDAITEVECFLNPEMGDPD	60
BAG75433.1	MAPTKRKGECPGAAPKKPKDPVQVPKLLIKGGVEVLEVKTGVDAITEVECFLNPEMGDPD	60
BAF93349.1	MAPTKRKGECPGAAPKKPKDPVQVPKLLIKGGVEVLEVKTGVDAITEVECFLNPEMGDPD	60
BAF76166.1	MAPTKRKGECPGAAPKKPKDPVQVPKLLIKGGVEVLEVKTGVDAITEVECFLNPEMGDPD	60
BAF76172.1	MAPTKRKGECPGAAPKKPKDPVQVPKLLIKGGVEVLEVKTGVDAITEVECFLNPEMGDPD	60
BAF76178.1	MAPTKRKGECPGAAPKKPKDPVQVPKLLIKGGVEVLEVKTGVDAITEVECFLNPEMGDPD	60
BAF76184.1	MAPTKRKGECPGAAPKKPKDPVQVPKLLIKGGVEVLEVKTGVDAITEVECFLNPEMGDPD	60
BAF76190.1	MAPTKRKGECPGAAPKKPKDPVQVPKLLIKGGVEVLEVKTGVDAITEVECFLNPEMGDPD	60
BAF76202.1	MAPTKRKGECPGAAPKKPKDPVQVPKLLIKGGVEVLEVKTGVDAITEVECFLNPEMGDPD	60
BAF42985.1	MAPTKRKGECPGAAPKKPKDPVQVPKLLIKGGVEVLEVKTGVDAITEVECFLNPEMGDPD	60
BAF42991.1	MAPTKRKGECPGAAPKKPKDPVQVPKLLIKGGVEVLEVKTGVDAITEVECFLNPEMGDPD	60
BAF02951.1	MAPTKRKGECPGAAPKKPKDPVQVPKLLIKGGVEVLEVKTGVDAITEVECFLNPEMGDPD	60
BAF02963.1	MAPTKRKGECPGAAPKKPKDPVQVPKLLIKGGVEVLEVKTGVDAITEVECFLNPEMGDPD	60
BAF02969.1	MAPTKRKGECPGAAPKKPKDPVQVPKLLIKGGVEVLEVKTGVDAITEVECFLNPEMGDPD	60
BAF02981.1	MAPTKRKGECPGAAPKKPKDPVQVPKLLIKGGVEVLEVKTGVDAITEVECFLNPEMGDPD	60
BAF02987.1	MAPTKRKGECPGAAPKKPKDPVQVPKLLIKGGVEVLEVKTGVDAITEVECFLNPEMGDPD	60
BAF02993.1	MAPTKRKGECPGAAPKKPKDPVQVPKLLIKGGVEVLEVKTGVDAITEVECFLNPEMGDPD	60
BAF02999.1	MAPTKRKGECPGAAPKKPKDPVQVPKLLIKGGVEVLEVKTGVDAITEVECFLNPEMGDPD	60
BAF03005.1	MAPTKRKGECPGAAPKKPKDPVQVPKLLIKGGVEVLEVKTGVDAITEVECFLNPEMGDPD	60
BAF03011.1	MAPTKRKGECPGAAPKKPKDPVQVPKLLIKGGVEVLEVKTGVDAITEVECFLNPEMGDPD	60
BAE96065.1	MAPTKRKGECPGAAPKKPKDPVQVPKLLIKGGVEVLEVKTGVDAITEVECFLNPEMGDPD	60
BAE53666.1	MAPTKRKGECPGAAPKKPKDPVQVPKLLIKGGVEVLEVKTGVDAITEVECFLNPEMGDPD	60
BAE46623.1	MAPTKRKGECPGAAPKKPKDPVQVPKLLIKGGVEVLEVKTGVDAITEVECFLNPEMGDPD	60
CAA40235.1	MAPTKRKGECPGAAPKKPKDPVQVPKLLIKGGVEVLEVKTGVDAITEVECFLNPEMGDPD	60
BAI43570.1	MAPTKRKGECPGAAPKKPKDPVQVPKLLIKGGVEVLEVKTGVDAITEVECFLNPEMGDPD	60
BAI43582.1	MAPTKRKGECPGAAPKKPKDPVQVPKLLIKGGVEVLEVKTGVDAITEVECFLNPEMGDPD	60
BAI43612.1	MAPTKRKGECPGAAPKKPKDPVQVPKLLIKGGVEVLEVKTGVDAITEVECFLNPEMGDPD	60
BAI43618.1	MAPTKRKGECPGAAPKKPKDPVQVPKLLIKGGVEVLEVKTGVDAITEVECFLNPEMGDPD	60
BAI43630.1	MAPTKRKGECPGAAPKKPKDPVQVPKLLIKGGVEVLEVKTGVDAITEVECFLNPEMGDPD	60
BAG75301.1	MAPTKRKGECPGAAPKKPKDPVQVPKLLIKGGVEVLEVKTGVDAITEVECFLNPEMGDPD	60
BAG75349.1	MAPTKRKGECPGAAPKKPKDPVQVPKLLIKGGVEVLEVKTGVDAITEVECFLNPEMGDPD	60
BAG75367.1	MAPTKRKGECPGAAPKKPKDPVQVPKLLIKGGVEVLEVKTGVDAITEVECFLNPEMGDPD	60
BAF76196.1	MAPTKRKGECPGAAPKKPKDPVQVPKLLIKGGVEVLEVKTGVDAITEVECFLNPEMGDPD	60
BAF02975.1	MAPTKRKGECPGAAPKKPKEPVQVPKLLIKGGVEVLEVKTGVDAITEVECFLNPEMGDPD	60
BAI43600.1	MAPTKRKGECPGAAPKKPKEPVQVPKLLIKGGVEVLEVKTGVDAITEVECFLNPEMGDPD	60
BAE96071.1	MAPTKRKGECPGAAPKKPKEPVQVPKLLIKGGVEVLEVKTGVDAITEVECFLNPEMGDPD	60
BAE53660.1	MAPTKRKGECPGAAPKKPKEPVQVPKLLIKGGVEVLEVKTGVDAITEVECFLNPEMGDPD	60
BCV68926.1	MAPTKRKGECPGAAPKKPKDPVQVPKLLIKGGVEVLEVKTGVDAITEVECFLNPEMGDPD	60
AYO46647.1	MAPTKRKGECPGAAPKKPKDPVQVPKLLIKGGVEVLEVKTGVDAITEVECFLNPEMGDPD	60
BAR80134.1	MAPTKRKGECPGAAPKKPKDPVQVPKLLIKGGVEVLEVKTGVDAITEVECFLNPEMGDPD	60
BAR80140.1	MAPTKRKGECPGAAPKKPKDPVQVPKLLIKGGVEVLEVKTGVDAITEVECFLNPEMGDPD	60
BAI43588.1	MAPTKRKGECPGAAPKKPKDPVQVPKLLIKGGVEVLEVKTGVDAITEVECFLNPEMGDPN	60

```
BAG75361.1      MAPTKRKGECPGAAPKKPKDPVQVPKLLIKGGVEVLEVKTGVDAITEVECFLNPEMGDPD  60
BAE53642.1      MAPTKRKGECPGAAPKKPKDPVQVPKLLIKGGVEVLEVKTGVDAITEVECFLNPEMGDPD  60
BAE53648.1      MAPTKRKGECPGAAPKKPKDPVQVPKLLIKGGVEVLEVKTGVDAITEVECFLNPEMGDPD  60
AFA41903.1      MAPTKRKGECPGAAPKKPKEPVQVPKLLIKGGVEVLEVKTGVDAITEVECFLNPEMGDPD  60
AFA41918.1      MAPTKRKGECPGAAPKKPKEPVQVPKLLIKGGVEVLEVKTGVDAITEVECFLNPEMGDPD  60
BAF42937.1      MAPTKRKGECPGAAPKKPKEPVQVPKLLIKGGVEVLEVKTGVDAITEVECFLNPEMGDPD  60
BAF42997.1      MAPTKRKGECPGAAPKKPKEPVQVPKLLIKGGVEVLEVKTGVDAITEVECFLNPEMGDPD  60
WLD47902.1      MAPTKRKGECPGAAPKKPKEPVQVPKLLIKGGVEVLEVKTGVDAITEVECFLNPEMGDPD  60
WLD47938.1      MAPTKRKGECPGAAPKKPKEPVQVPKLLIKGGVEVLEVKTGVDAITEVECFLNPEMGDPD  60
WLD47980.1      MAPTKRKGECPGAAPKKPKEPVQVPKLLIKGGVEVLEVKTGVDAITEVECFLNPEMGDPD  60
UTM61891.1      MAPTKRKGECPGAAPKKPKEPVQVPKLLIKGGVEVLEVKTGVDAITEVECFLNPEMGDPD  60
UTM61897.1      MAPTKRKGECPGAAPKKPKEPVQVPKLLIKGGVEVLEVKTGVDAITEVECFLNPEMGDPD  60
UTM61903.1      MAPTKRKGECPGAAPKKPKEPVQVPKLLIKGGVEVLEVKTGVDAITEVECFLNPEMGDPD  60
UTM62215.1      MAPTKRKGECPGAAPKKPKEPVQVPKLLIKGGVEVLEVKTGVDAITEVECFLNPEMGDPD  60
UTM62221.1      MAPTKRKGECPGAAPKKPKEPVQVPKLLIKGGVEVLEVKTGVDAITEVECFLNPEMGDPD  60
UTM62275.1      MAPTKRKGECPGAAPKKPKEPVQVPKLLIKGGVEVLEVKTGVDAITEVECFLNPEMGDPD  60
UTM62281.1      MAPTKRKGECPGAAPKKPKEPVQVPKLLIKGGVEVLEVKTGVDAITEVECFLNPEMGDPD  60
UTM62329.1      MAPTKRKGECPGAAPKKPKEPVQVPKLLIKGGVEVLEVKTGVDAITEVECFLNPEMGDPD  60
UTM62335.1      MAPTKRKGECPGAAPKKPKEPVQVPKLLIKGGVEVLEVKTGVDAITEVECFLNPEMGDPD  60
UTM62383.1      MAPTKRKGECPGAAPKKPKEPVQVPKLLIKGGVEVLEVKTGVDAITEVECFLNPEMGDPD  60
UTM62395.1      MAPTKRKGECPGAAPKKPKEPVQVPKLLIKGGVEVLEVKTGVDAITEVECFLNPEMGDPD  60
UTM62443.1      MAPTKRKGECPGAAPKKPKEPVQVPKLLIKGGVEVLEVKTGVDAITEVECFLNPEMGDPD  60
UTM62449.1      MAPTKRKGECPGAAPKKPKEPVQVPKLLIKGGVEVLEVKTGVDAITEVECFLNPEMGDPD  60
UTM62467.1      MAPTKRKGECPGAAPKKPKEPVQVPKLLIKGGVEVLEVKTGVDAITEVECFLNPEMGDPD  60
UTM62545.1      MAPTKRKGECPGAAPKKPKEPVQVPKLLIKGGVEVLEVKTGVDAITEVECFLNPEMGDPD  60
UTM62551.1      MAPTKRKGECPGAAPKKPKEPVQVPKLLIKGGVEVLEVKTGVDAITEVECFLNPEMGDPD  60
UTM62587.1      MAPTKRKGECPGAAPKKPKEPVQVPKLLIKGGVEVLEVKTGVDAITEVECFLNPEMGDPD  60
UTM62593.1      MAPTKRKGECPGAAPKKPKEPVQVPKLLIKGGVEVLEVKTGVDAITEVECFLNPEMGDPD  60
UTM62599.1      MAPTKRKGECPGAAPKKPKEPVQVPKLLIKGGVEVLEVKTGVDAITEVECFLNPEMGDPD  60
QUW04850.1      MAPTKRKGECPGAAPKKPKEPVQVPKLLIKGGVEVLEVKTGVDAITEVECFLNPEMGDPD  60
QTJ14976.1      MAPTKRKGECPGAAPKKPKEPVQVPKLLIKGGVEVLEVKTGVDAITEVECFLNPEMGDPD  60
QTJ15000.1      MAPTKRKGECPGAAPKKPKEPVQVPKLLIKGGVEVLEVKTGVDAITEVECFLNPEMGDPD  60
QTJ15054.1      MAPTKRKGECPGAAPKKPKEPVQVPKLLIKGGVEVLEVKTGVDAITEVECFLNPEMGDPD  60
QTJ15072.1      MAPTKRKGECPGAAPKKPKEPVQVPKLLIKGGVEVLEVKTGVDAITEVECFLNPEMGDPD  60
QTJ15084.1      MAPTKRKGECPGAAPKKPKEPVQVPKLLIKGGVEVLEVKTGVDAITEVECFLNPEMGDPD  60
QTJ15090.1      MAPTKRKGECPGAAPKKPKEPVQVPKLLIKGGVEVLEVKTGVDAITEVECFLNPEMGDPD  60
QTJ15108.1      MAPTKRKGECPGAAPKKPKEPVQVPKLLIKGGVEVLEVKTGVDAITEVECFLNPEMGDPD  60
QTJ15114.1      MAPTKRKGECPGAAPKKPKEPVQVPKLLIKGGVEVLEVKTGVDAITEVECFLNPEMGDPD  60
QTJ15132.1      MAPTKRKGECPGAAPKKPKEPVQVPKLLIKGGVEVLEVKTGVDAITEVECFLNPEMGDPD  60
QTJ15138.1      MAPTKRKGECPGAAPKKPKEPVQVPKLLIKGGVEVLEVKTGVDAITEVECFLNPEMGDPD  60
AYO46612.1      MAPTKRKGECPGAAPKKPKEPVQVPKLLIKGGVEVLEVKTGVDAITEVECFLNPEMGDPD  60
AYO46613.1      MAPTKRKGECPGAAPKKPKEPVQVPKLLIKGGVEVLEVKTGVDAITEVECFLNPEMGDPD  60
AYO46617.1      MAPTKRKGECPGAAPKKPKEPVQVPKLLIKGGVEVLEVKTGVDAITEVECFLNPEMGDPD  60
AYO46621.1      MAPTKRKGECPGAAPKKPKEPVQVPKLLIKGGVEVLEVKTGVDAITEVECFLNPEMGDPD  60
AYO46622.1      MAPTKRKGECPGAAPKKPKEPVQVPKLLIKGGVEVLEVKTGVDAITEVECFLNPEMGDPD  60
AYO46623.1      MAPTKRKGECPGAAPKKPKEPVQVPKLLIKGGVEVLEVKTGVDAITEVECFLNPEMGDPD  60
AYO46624.1      MAPTKRKGECPGAAPKKPKEPVQVPKLLIKGGVEVLEVKTGVDAITEVECFLNPEMGDPD  60
AYO46629.1      MAPTKRKGECPGAAPKKPKEPVQVPKLLIKGGVEVLEVKTGVDAITEVECFLNPEMGDPD  60
AYO46632.1      MAPTKRKGECPGAAPKKPKEPVQVPKLLIKGGVEVLEVKTGVDAITEVECFLNPEMGDPD  60
AYO46635.1      MAPTKRKGECPGAAPKKPKEPVQVPKLLIKGGVEVLEVKTGVDAITEVECFLNPEMGDPD  60
AYO46638.1      MAPTKRKGECPGAAPKKPKEPVQVPKLLIKGGVEVLEVKTGVDAITEVECFLNPEMGDPD  60
AYO46641.1      MAPTKRKGECPGAAPKKPKEPVQVPKLLIKGGVEVLEVKTGVDAITEVECFLNPEMGDPD  60
AYO46642.1      MAPTKRKGECPGAAPKKPKEPVQVPKLLIKGGVEVLEVKTGVDAITEVECFLNPEMGDPD  60
AYO46644.1      MAPTKRKGECPGAAPKKPKEPVQVPKLLIKGGVEVLEVKTGVDAITEVECFLNPEMGDPD  60
AYO46651.1      MAPTKRKGECPGAAPKKPKEPVQVPKLLIKGGVEVLEVKTGVDAITEVECFLNPEMGDPD  60
AYO46652.1      MAPTKRKGECPGAAPKKPKEPVQVPKLLIKGGVEVLEVKTGVDAITEVECFLNPEMGDPD  60
AYO46659.1      MAPTKRKGECPGAAPKKPKEPVQVPKLLIKGGVEVLEVKTGVDAITEVECFLNPEMGDPD  60
AYO46660.1      MAPTKRKGECPGAAPKKPKEPVQVPKLLIKGGVEVLEVKTGVDAITEVECFLNPEMGDPD  60
AYO46662.1      MAPTKRKGECPGAAPKKPKEPVQVPKLLIKGGVEVLEVKTGVDAITEVECFLNPEMGDPD  60
AYO46665.1      MAPTKRKGECPGAAPKKPKEPVQVPKLLIKGGVEVLEVKTGVDAITEVECFLNPEMGDPD  60
AYO46670.1      MAPTKRKGECPGAAPKKPKEPVQVPKLLIKGGVEVLEVKTGVDAITEVECFLNPEMGDPD  60
AYO46672.1      MAPTKRKGECPGAAPKKPKEPVQVPKLLIKGGVEVLEVKTGVDAITEVECFLNPEMGDPD  60
AYO46676.1      MAPTKRKGECPGAAPKKPKEPVQVPKLLIKGGVEVLEVKTGVDAITEVECFLNPEMGDPD  60
AYO46677.1      MAPTKRKGECPGAAPKKPKEPVQVPKLLIKGGVEVLEVKTGVDAITEVECFLNPEMGDPD  60
```

AYO46679.1	MAPTKRKGECPGAAPKKPKEPVQVPKLLIKGGVEVLEVKTGVDAITEVECFLNPEMGDPD	60
AYO46682.1	MAPTKRKGECPGAAPKKPKEPVQVPKLLIKGGVEVLEVKTGVDAITEVECFLNPEMGDPD	60
AYO46691.1	MAPTKRKGECPGAAPKKPKEPVQVPKLLIKGGVEVLEVKTGVDAITEVECFLNPEMGDPD	60
AYO46692.1	MAPTKRKGECPGAAPKKPKEPVQVPKLLIKGGVEVLEVKTGVDAITEVECFLNPEMGDPD	60
AUF70040.1	MAPTKRKGECPGAAPKKPKEPVQVPKLLIKGGVEVLEVKTGVDAITEVECFLNPEMGDPD	60
SOH95722.1	MAPTKRKGECPGAAPKKPKEPVQVPKLLIKGGVEVLEVKTGVDAITEVECFLNPEMGDPD	60
SOI09711.1	MAPTKRKGECPGAAPKKPKEPVQVPKLLIKGGVEVLEVKTGVDAITEVECFLNPEMGDPD	60
APG38208.1	MAPTKRKGECPGAAPKKPKEPVQVPKLLIKGGVEVLEVKTGVDAITEVECFLNPEMGDPD	60
ALF06332.1	MAPTKRKGECPGAAPKKPKEPVQVPKLLIKGGVEVLEVKTGVDAITEVECFLNPEMGDPD	60
ALF06341.1	MAPTKRKGECPGAAPKKPKEPVQVPKLLIKGGVEVLEVKTGVDAITEVECFLNPEMGDPD	60
BAR80122.1	MAPTKRKGECPGAAPKKPKEPVQVPKLLIKGGVEVLEVKTGVDAITEVECFLNPEMGDPD	60
BAR80128.1	MAPTKRKGECPGAAPKKPKEPVQVPKLLIKGGVEVLEVKTGVDAITEVECFLNPEMGDPD	60
AJG42164.1	MAPTKRKGECPGAAPKKPKEPVQVPKLLIKGGVEVLEVKTGVDAITEVECFLNPEMGDPD	60
AHU87340.1	MAPTKRKGECPGAAPKKPKEPVQVPKLLIKGGVEVLEVKTGVDAITEVECFLNPEMGDPD	60
AHU87341.1	MAPTKRKGECPGAAPKKPKEPVQVPKLLIKGGVEVLEVKTGVDAITEVECFLNPEMGDPD	60
AHU87368.1	MAPTKRKGECPGAAPKKPKEPVQVPKLLIKGGVEVLEVKTGVDAITEVECFLNPEMGDPD	60
AHU87369.1	MAPTKRKGECPGAAPKKPKEPVQVPKLLIKGGVEVLEVKTGVDAITEVECFLNPEMGDPD	60
AFQ31636.1	MAPTKRKGECPGAAPKKPKEPVQVPKLLIKGGVEVLEVKTGVDAITEVECFLNPEMGDPD	60
AFQ31637.1	MAPTKRKGECPGAAPKKPKEPVQVPKLLIKGGVEVLEVKTGVDAITEVECFLNPEMGDPD	60
AFQ31639.1	MAPTKRKGECPGAAPKKPKEPVQVPKLLIKGGVEVLEVKTGVDAITEVECFLNPEMGDPD	60
AFQ31640.1	MAPTKRKGECPGAAPKKPKEPVQVPKLLIKGGVEVLEVKTGVDAITEVECFLNPEMGDPD	60
AFQ31641.1	MAPTKRKGECPGAAPKKPKEPVQVPKLLIKGGVEVLEVKTGVDAITEVECFLNPEMGDPD	60
AFQ31644.1	MAPTKRKGECPGAAPKKPKEPVQVPKLLIKGGVEVLEVKTGVDAITEVECFLNPEMGDPD	60
AFQ31648.1	MAPTKRKGECPGAAPKKPKEPVQVPKLLIKGGVEVLEVKTGVDAITEVECFLNPEMGDPD	60
AFQ31657.1	MAPTKRKGECPGAAPKKPKEPVQVPKLLIKGGVEVLEVKTGVDAITEVECFLNPEMGDPD	60
AFA41882.1	MAPTKRKGECPGAAPKKPKEPVQVPKLLIKGGVEVLEVKTGVDAITEVECFLNPEMGDPD	60
AFA41887.1	MAPTKRKGECPGAAPKKPKEPVQVPKLLIKGGVEVLEVKTGVDAITEVECFLNPEMGDPD	60
AFA41890.1	MAPTKRKGECPGAAPKKPKEPVQVPKLLIKGGVEVLEVKTGVDAITEVECFLNPEMGDPD	60
AFA41891.1	MAPTKRKGECPGAAPKKPKEPVQVPKLLIKGGVEVLEVKTGVDAITEVECFLNPEMGDPD	60
AFA41895.1	MAPTKRKGECPGAAPKKPKEPVQVPKLLIKGGVEVLEVKTGVDAITEVECFLNPEMGDPD	60
AFA41898.1	MAPTKRKGECPGAAPKKPKEPVQVPKLLIKGGVEVLEVKTGVDAITEVECFLNPEMGDPD	60
AFA41901.1	MAPTKRKGECPGAAPKKPKEPVQVPKLLIKGGVEVLEVKTGVDAITEVECFLNPEMGDPD	60
AFA41902.1	MAPTKRKGECPGAAPKKPKEPVQVPKLLIKGGVEVLEVKTGVDAITEVECFLNPEMGDPD	60
AFA41910.1	MAPTKRKGECPGAAPKKPKEPVQVPKLLIKGGVEVLEVKTGVDAITEVECFLNPEMGDPD	60
AFA41914.1	MAPTKRKGECPGAAPKKPKEPVQVPKLLIKGGVEVLEVKTGVDAITEVECFLNPEMGDPD	60
AFA41917.1	MAPTKRKGECPGAAPKKPKEPVQVPKLLIKGGVEVLEVKTGVDAITEVECFLNPEMGDPD	60
AEO89562.1	MAPTKRKGECPGAAPKKPKEPVQVPKLLIKGGVEVLEVKTGVDAITEVECFLNPEMGDPD	60
AEO89568.1	MAPTKRKGECPGAAPKKPKEPVQVPKLLIKGGVEVLEVKTGVDAITEVECFLNPEMGDPD	60
AEO89586.1	MAPTKRKGECPGAAPKKPKEPVQVPKLLIKGGVEVLEVKTGVDAITEVECFLNPEMGDPD	60
AEO89598.1	MAPTKRKGECPGAAPKKPKEPVQVPKLLIKGGVEVLEVKTGVDAITEVECFLNPEMGDPD	60
AEO89621.1	MAPTKRKGECPGAAPKKPKEPVQVPKLLIKGGVEVLEVKTGVDAITEVECFLNPEMGDPD	60
AEK21505.1	MAPTKRKGECPGAAPKKPKEPVQVPKLLIKGGVEVLEVKTGVDAITEVECFLNPEMGDPD	60
BAG84458.1	MAPTKRKGECPGAAPKKPKEPVQVPKLLIKGGVEVLEVKTGVDAITEVECFLNPEMGDPD	60
BAG84470.1	MAPTKRKGECPGAAPKKPKEPVQVPKLLIKGGVEVLEVKTGVDAITEVECFLNPEMGDPD	60
BAG84482.1	MAPTKRKGECPGAAPKKPKEPVQVPKLLIKGGVEVLEVKTGVDAITEVECFLNPEMGDPD	60
BAG75319.1	MAPTKRKGECPGAAPKKPKEPVQVPKLLIKGGVEVLEVKTGVDAITEVECFLNPEMGDPD	60
BAG75355.1	MAPTKRKGECPGAAPKKPKEPVQVPKLLIKGGVEVLEVKTGVDAITEVECFLNPEMGDPD	60
BAG75391.1	MAPTKRKGECPGAAPKKPKEPVQVPKLLIKGGVEVLEVKTGVDAITEVECFLNPEMGDPD	60
BAG75439.1	MAPTKRKGECPGAAPKKPKEPVQVPKLLIKGGVEVLEVKTGVDAITEVECFLNPEMGDPD	60
BAF93301.1	MAPTKRKGECPGAAPKKPKEPVQVPKLLIKGGVEVLEVKTGVDAITEVECFLNPEMGDPD	60
BAF93331.1	MAPTKRKGECPGAAPKKPKEPVQVPKLLIKGGVEVLEVKTGVDAITEVECFLNPEMGDPD	60
BAF93355.1	MAPTKRKGECPGAAPKKPKEPVQVPKLLIKGGVEVLEVKTGVDAITEVECFLNPEMGDPD	60
BAF76208.1	MAPTKRKGECPGAAPKKPKEPVQVPKLLIKGGVEVLEVKTGVDAITEVECFLNPEMGDPD	60
BAF42877.1	MAPTKRKGECPGAAPKKPKEPVQVPKLLIKGGVEVLEVKTGVDAITEVECFLNPEMGDPD	60
BAF42883.1	MAPTKRKGECPGAAPKKPKEPVQVPKLLIKGGVEVLEVKTGVDAITEVECFLNPEMGDPD	60
BAF42889.1	MAPTKRKGECPGAAPKKPKEPVQVPKLLIKGGVEVLEVKTGVDAITEVECFLNPEMGDPD	60
BAF42961.1	MAPTKRKGECPGAAPKKPKEPVQVPKLLIKGGVEVLEVKTGVDAITEVECFLNPEMGDPD	60
BAF42967.1	MAPTKRKGECPGAAPKKPKEPVQVPKLLIKGGVEVLEVKTGVDAITEVECFLNPEMGDPD	60
BAF42973.1	MAPTKRKGECPGAAPKKPKEPVQVPKLLIKGGVEVLEVKTGVDAITEVECFLNPEMGDPD	60
BAF43003.1	MAPTKRKGECPGAAPKKPKEPVQVPKLLIKGGVEVLEVKTGVDAITEVECFLNPEMGDPD	60
BAF43027.1	MAPTKRKGECPGAAPKKPKEPVQVPKLLIKGGVEVLEVKTGVDAITEVECFLNPEMGDPD	60
BAF43033.1	MAPTKRKGECPGAAPKKPKEPVQVPKLLIKGGVEVLEVKTGVDAITEVECFLNPEMGDPD	60
ABI94659.1	MAPTKRKGECPGAAPKKPKEPVQVPKLLIKGGVEVLEVKTGVDAITEVECFLNPEMGDPD	60
ABI94683.1	MAPTKRKGECPGAAPKKPKEPVQVPKLLIKGGVEVLEVKTGVDAITEVECFLNPEMGDPD	60

ABI94719.1	MAPTKRKGECPGAAPKKPKEPVQVPKLLIKGGVEVLEVKTGVDAITEVECFLNPEMGDPD	60
BAE96053.1	MAPTKRKGECPGAAPKKPKEPVQVPKLLIKGGVEVLEVKTGVDAITEVECFLNPEMGDPD	60
BAE96059.1	MAPTKRKGECPGAAPKKPKEPVQVPKLLIKGGVEVLEVKTGVDAITEVECFLNPEMGDPD	60
ABC18005.1	MAPTKRKGECPGAAPKKPKEPVQVPKLLIKGGVEVLEVKTGVDAITEVECFLNPEMGDPD	60
AAT47377.1	MAPTKRKGECPGAAPKKPKEPVQVPKLLIKGGVEVLEVKTGVDAITEVECFLNPEMGDPD	60
AAT47383.1	MAPTKRKGECPGAAPKKPKEPVQVPKLLIKGGVEVLEVKTGVDAITEVECFLNPEMGDPD	60
AAT47405.1	MAPTKRKGECPGAAPKKPKEPVQVPKLLIKGGVEVLEVKTGVDAITEVECFLNPEMGDPD	60
CAA40239.1	MAPTKRKGECPGAAPKKPKEPVQVPKLLIKGGVEVLEVKTGVDAITEVECFLNPEMGDPD	60
QTJ14940.1	MAPTKRKGECPGAAPKKPKEPVQVPKLLIKGGVEVLEVKTGVDAITEVECFLNPEMGDPD	60
ALF06317.1	MAPTKRKGECPGAAPKKPKEPVQVPKLLIKGGVEVLEVKTGLDAITEVECFLNPEMGDPD	60
ALF06307.1	MAPTKRKGECPGAAPKKPKEPVQVPKLLIKGGVEVLEVKTGVDAITEVECFLNPEMGDPD	60
ALF06297.1	MAPTKRKGECPGAAPKKPKEPVQVPKLLIKGGVEVLEVKTGXDAITEVECFLNPEMGDPD	60
ALF06350.1	MAPTKRKGECPGAAPKKPKEPVQVPKLLIKGGVEVLEVKTGVDAITEVECFLNPEMGDPD	60
AAT47431.1	MAPTKRKGECPGAAPKKPKEPVQVPKLLIKGGVEVLEVKTGVDAITEVECFLNPEMGDPD	60
QTJ15006.1	MAPTKRKGECPGAAPKKPKEPVQVPKLLIKGGVEVLEVKTGVDAITEVECFLNPEMGDPD	60
AYO46667.1	MAPTKRKGECPGAAPKKPKEPVQVPKLLIKGGVEVLEVKTGVDAITEVECFLNPEMGDPD	60
WLD47956.1	MAPTKRKGECPGAAPKKPKEPVQVPKLLIKGGVEVLEVKTGVDAITEVECFLNPEMGDPD	60
QTJ15150.1	MAPTKRKGECPGAAPKKPKEPVQVPKLLIKGGVEVLEVKTGVDAITEVECFLNPEMGDPD	60
UTM62389.1	MAPTKRKGECPGAAPKKPKEPVQVPKLLIKGGVEVLEVKTGVDAITEVECFLNPEMGDPD	60
ABI94701.1	MAPTKRKGECPGAAPKKPKEPVQVPKLLIKGGVEVLEVKTGVDAITEVECFLNPEMGDPD	60
BAF02945.1	MAPTKRKGECPGAAPKKPKEPVQVPKLLIKGGVEVLEVKTGVDAITEVECFLNPEMGDPD	60
CAA40243.1	MAPTKRKGECPGAAPKKPKEPVQVPKLLIKGGVEVLEVKTGVDAITEVECFLNPEMGDPD	60
ABI94725.1	MAPTKRKGECPGAAPKKPKEPVQVPKLLIKGGVEVLEVKTGVDAITEVECFLNPEMGDPD	60
AFQ31660.1	MAPTKRKGECPGAAPKKPKEPVQVPKLLIKGGVEVLEVKTGVDAITEVECFLNPEMGDPD	60
ALF06353.1	MAPTQRKGECPGAAPKKPKEPVQVPKLLIKGGVEVLEVKTGVDAITEVECFLNPEMGDPD	60
ALF06312.1	MAPTKRKGECPGAAPKKPKEPVQVPKLLIKGGVEVLEVKTGVDAITEVECFLNPEMGDPD	60
QEJ80721.1	MAPTKRKGECPGAAPKKPKEPVQVPKLLIKGGVEVLEVKTGVDAITEVECFLNPEMGDPD	60
AYO46614.1	MAPTKRKGECPGAAPKKPKEPVQVPKLLIKGGVEVLEVKTGVDAITEVECFLNPEMGDPD	60
AYO46619.1	MAPTKRKGECPGAAPKKPKEPVQVPKLLIKGGVEVLEVKTGVDAITEVECFLNPEMGDPD	60
AYO46625.1	MAPTKRKGECPGAAPKKPKEPVQVPKLLIKGGVEVLEVKTGVDAITEVECFLNPEMGDPD	60
AYO46626.1	MAPTKRKGECPGAAPKKPKEPVQVPKLLIKGGVEVLEVKTGVDAITEVECFLNPEMGDPD	60
AYO46628.1	MAPTKRKGECPGAAPKKPKEPVQVPKLLIKGGVEVLEVKTGVDAITEVECFLNPEMGDPD	60
AYO46633.1	MAPTKRKGECPGAAPKKPKEPVQVPKLLIKGGVEVLEVKTGVDAITEVECFLNPEMGDPD	60
AYO46636.1	MAPTKRKGECPGAAPKKPKEPVQVPKLLIKGGVEVLEVKTGVDAITEVECFLNPEMGDPD	60
AYO46637.1	MAPTKRKGECPGAAPKKPKEPVQVPKLLIKGGVEVLEVKTGVDAITEVECFLNPEMGDPD	60
AYO46640.1	MAPTKRKGECPGAAPKKPKEPVQVPKLLIKGGVEVLEVKTGVDAITEVECFLNPEMGDPD	60
AYO46648.1	MAPTKRKGECPGAAPKKPKEPVQVPKLLIKGGVEVLEVKTGVDAITEVECFLNPEMGDPD	60
AYO46650.1	MAPTKRKGECPGAAPKKPKEPVQVPKLLIKGGVEVLEVKTGVDAITEVECFLNPEMGDPD	60
AYO46653.1	MAPTKRKGECPGAAPKKPKEPVQVPKLLIKGGVEVLEVKTGVDAITEVECFLNPEMGDPD	60
AYO46654.1	MAPTKRKGECPGAAPKKPKEPVQVPKLLIKGGVEVLEVKTGVDAITEVECFLNPEMGDPD	60
AYO46671.1	MAPTKRKGECPGAAPKKPKEPVQVPKLLIKGGVEVLEVKTGVDAITEVECFLNPEMGDPD	60
AYO46673.1	MAPTKRKGECPGAAPKKPKEPVQVPKLLIKGGVEVLEVKTGVDAITEVECFLNPEMGDPD	60
AYO46674.1	MAPTKRKGECPGAAPKKPKEPVQVPKLLIKGGVEVLEVKTGVDAITEVECFLNPEMGDPD	60
AYO46675.1	MAPTKRKGECPGAAPKKPKEPVQVPKLLIKGGVEVLEVKTGVDAITEVECFLNPEMGDPD	60
AYO46680.1	MAPTKRKGECPGAAPKKPKEPVQVPKLLIKGGVEVLEVKTGVDAITEVECFLNPEMGDPD	60
AYO46681.1	MAPTKRKGECPGAAPKKPKEPVQVPKLLIKGGVEVLEVKTGVDAITEVECFLNPEMGDPD	60
AYO46684.1	MAPTKRKGECPGAAPKKPKEPVQVPKLLIKGGVEVLEVKTGVDAITEVECFLNPEMGDPD	60
AYO46687.1	MAPTKRKGECPGAAPKKPKEPVQVPKLLIKGGVEVLEVKTGVDAITEVECFLNPEMGDPD	60
AEO89574.1	MAPTKRKGECPGAAPKKPKEPVQVPKLLIKGGVEVLEVKTGVDAITEVECFLNPEMGDPD	60
BAF42979.1	MAPTKRKGECPGAAPKKPKEPVQVPKLLIKGGVEVLEVKTGVDAITEVECFLNPEMGDPD	60
BAF43009.1	MAPTKRKGECPGAAPKKPKEPVQVPKLLIKGGVEVLEVKTGVDAITEVECFLNPEMGDPD	60
AYO46678.1	MAPTKRKGECPGAAPKKPKEPVQVPKLLIKGGVEVLEVKTGVDAITEVECFLNPEMGDPD	60
UTM62479.1	MAPTKRKGECPGAAPKKPKEPVQVPKLLIKGGVEVLEVKTGVDAITEVECFLNPEMGDPD	60
QTJ14964.1	MAPTKRKGECPGAAPKKPKEPVQVPKLLIKGGVEVLEVKTGVDAITEVECFLNPEMGDPD	60
QTJ15036.1	MAPTKRKGECPGAAPKKPKEPVQVPKLLIKGGVEVLEVKTGVDAITEVECFLNPEMGDPD	60
QTJ15066.1	MAPTKRKGECPGAAPKKPKEPVQVPKLLIKGGVEVLEVKTGVDAITEVECFLNPEMGDPD	60
QTJ15078.1	MAPTKRKGECPGAAPKKPKEPVQVPKLLIKGGVEVLEVKTGVDAITEVECFLNPEMGDPD	60
QTJ15096.1	MAPTKRKGECPGAAPKKPKEPVQVPKLLIKGGVEVLEVKTGVDAITEVECFLNPEMGDPD	60
AQM40276.1	MAPTKRKGECPGAAPKKPKEPVQVPKLLIKGGVEVLEVKTGVDAITEVECFLNPEMGDPD	60
ABI94689.1	MAPTKRKGECPGAAPKKPKEPVQVPKLLIKGGVEVLEVKTGVDAITEVECFLNPEMGDPD	60
ABI94671.1	MAPTKRKGECPGAAPKKPKEPVQVPKLLIKGGVEVLEVKTGVDAITEVECFLNPEMGDSD	60
AYO46688.1	MAPTKRKGECPGAAPKKPKEPVQVPKLLIKGGVEVLEVKTGVDAITEVECFLNPEMGDPD	60
QTJ15060.1	MAPTKRKGECPGAAPKKPKEPVQVPKLLIKGGVEVLEVKTGVDAITEVECFLNPEMGDPD	60
AFQ31642.1	MAPTKRKGECPGAAPKKPKEPVQVPKLLIKGGVEVLEVKTGVDAITEVECFLNPEMGDPD	60

AYO46616.1	MAPTKRKGECPGAAPKKPKEPVQVPKLLIKGGVEVLEFKTGVDAITEVECFLNPEMGDPD	60
CAA79594.1	MAPTKRKGECPGAAPKKPKEPVQVPKLLIKGGVEVLEVKTGVDAITEVECFLNPEMGDPD	60
WLD47818.1	MAPTKRKGECPGAAPKKPKEPVQVPKLLIKGGVEVLEVKTGVDAITEVECFLNPEMGDPN	60
UTM62155.1	MAPTKRKGECPGAAPKKPKEPVQVPKLLIKGGVEVLEVKTGVDAITEVECFLNPEMGDPN	60
AFA41907.1	MAPTKRKGECPGAAPKKPKEPVQVPKLLIKGGVEVLEVKTGVDAITEVECFLNPEMGDPN	60
WLD47944.1	MAPTKRKGECPGAAPKKPKEPVQVPKLLIKGGVEVLEVKTGVDAITEVECFLNPEMGDPD	60
AYO46668.1	MAPTKRKGECPGAAPKKPKEPVQVPKLLIKGGVEVLEVKTGVDAITEVECFLNPEMGDPD	60
AFQ31651.1	MAPTKRKGECPGAAPKKPKEPVQVPKLLIKGGVEVLEVKTGVDAITEVECFLNPEMGDPD	60
QTJ14958.1	MAPTKRKGECPGAAPKKPKEPVQVPKLLIKGGVEVLEVKTGVDAITEVECFLNPEMGDPD	60
AYO46618.1	MAPTKRKGECPGAAPKKPKEPVQVPKLLIKGGVEVLEVKTGVDAITEVECFLNPEMGDPD	60
QTJ15018.1	MAPTKRKGECPGAAPKKPKEPVQVPKLLIKGGVEVLEVKTGVDAITEVECFLNPEMGDPD	60
AYO46609.1	MAPTKRKGECPGAAPKKPKEPVQVPKLLIKGGVEVLEVKTGVDAITEVECFLNPEMGDPD	60
ALF06356.1	MAPTKRKGECPGAAPKKPKEPVQVPKLLIKGGVEVLEVKTGVDAITEVECFLNPEMGDPD	60
AFQ31646.1	MAPTKRKGECPGAAPKKPKEPVQVPKLLIKGGVEVLEVKTGVDAITEVECFLNPEMGDPD	60
AFQ31659.1	MAPTKRKGECPGAAPKKPKEPVQVPKLLIKGGVEVLEVKTGVDAITEVECFLNPEMGDPD	60
BAF02957.1	MAPTKRKGECPGAAPKKPKEPVQVPKLLIKGGVEVLEVKTGVDAITEVECFLNPEIGDPD	60
AAT47365.1	MAPTKRKGECPGAAPKKPKEPVQVPKLLIKGGVEVLEVKTGVDAITEVECFLNPEMGDPD	60
AAT47389.1	MAPTKRKGECPGAAPKKPKEPVQVPKLLIKGGVEVLEVKTGVDAITEVECFLNPEMGDPD	60
AAT47395.1	MAPTKRKGECPGAAPKKPKEPVQVPKLLIKGGVEVLEVKTGVDAITEVECFLNPEMGDPD	60
AAT47401.1	MAPTKRKGECPGAAPKKPEEPVQVPKLLIKGGVEVLEVKTGVDAITEVECFLNPEMGDPD	60
CAA24307.1	MAPTKRKGECPGAAPKKPKEPVQVPKLLIKGGVEVLEVKTGVDAITEVECFLNPEMGDPD	60
AAT47425.1	MAPTKRKGECPGAAPKKPKEPVQVPKLLIKGGVEVLEVKTGVDAITEVECFLNPEMGDPD	60
AFA41880.1	MAPTKRKGECPGAAPKKPKEPVQVPKLLIKGGVEVLEVKTGLDAITEVECFLNPEMGDPD	60
WLD47968.1	MAPTKRKGECPGAAPKKPKEPVQVPKLLIKGGVEVLEVKTGLDAITEVECFLNPEMGDPD	60
AFA41920.1	MAPTKRKGECPGAAPKKPKEPVQVPKLLIKGGVEVLEVKTGLDAITEVECFLNPEMGDPD	60
CBX88302.1	MAPTKRKGECPGAAPKKPKEPVQVPKLLIKGGVEVLEVKTGLDAITEVECFLNPEMGDPD	60
AUF70036.1	MAPTKRKGECPGAAPKKPKELVQVPKLLIKGGVEVLEVKTGLDAITEVECFLNPEMGDPD	60
ALF06323.1	MAPTKRKGECPGAAPKKPKEPVQVPKLLIKGGVEVLEAKTGLDAITEVECFLNPEMGDPD	60
WLD47890.1	MAPTKRKGECPGAAPKKPKEPVQVPKLLIKGGVEVLEVKTGLDAITEVECFLNPEMGDPD	60
BAF03097.1	MAPTKRKGECPGAAPKKPKEPVQVPKLLIKGGVEVLEVKTGLDAITEVECFLNPEMGDPD	60
ABI94695.1	MAPTKKKGECPGAAPKKPKEPVQVPKLLIKGGVEVLEVKTGLDAITEVECFLNPEMGDPD	60
ABI94623.1	MAPTKRKGECPGAAPKKPKEPVQVPKLLIKGGVEVLEVKTGLDAITEVECFLNPEMGDPD	60
UTM62533.1	MAPTKRKGECPGAAPKKPKEPVQVPKLLIKGGVEVLEVKTGLDAITEVECFLNPEMGDPD	60
QOS45058.1	MAPTKRKGECPGAAPKKPKEPVQVPKLLIKGGVEVLEVKTGLDAITEVECFLNPEMGDPD	60
AHU87362.1	MAPTKRKGECPGAAPKKPKEPVQVPKLLIKGGVEVLEVKTGLDAITEVECFLNPEMGDPD	60
AHU87363.1	MAPTKRKGECPGAAPKKPKEPVQVPKLLIKGGVEVLEVKTGLDAITEVECFLNPEMGDPD	60
AFQ31658.1	MAPTKRKGECPGAAPKKPKEPVQVPKLLIKGGVEVLEVKTGLDAITEVECFLNPEMGDPD	60
AFA41876.1	MAPTKRKGECPGAAPKKPKEPVQVPKLLIKGGVEVLEVKTGLDAITEVECFLNPEMGDPD	60
AFA41906.1	MAPTKRKGECPGAAPKKPKEPVQVPKLLIKGGVEVLEVKTGLDAITEVECFLNPEMGDPD	60
AEO89604.1	MAPTKRKGECPGAAPKKPKEPVQVPKLLIKGGVEVLEVKTGLDAITEVECFLNPEMGDPD	60
CBX88350.1	MAPTKRKGECPGAAPKKPKEPVQVPKLLIKGGVEVLEVKTGLDAITEVECFLNPEMGDPD	60
BAG84446.1	MAPTKRKGECPGAAPKKPKEPVQVPKLLIKGGVEVLEVKTGLDAITEVECFLNPEMGDPD	60
BAG84464.1	MAPTKRKGECPGAAPKKPKEPVQVPKLLIKGGVEVLEVKTGLDAITEVECFLNPEMGDPD	60
BAF93295.1	MAPTKRKGECPGAAPKKPKEPVQVPKLLIKGGVEVLEVKTGLDAITEVECFLNPEMGDPD	60
BAF93373.1	MAPTKRKGECPGAAPKKPKEPVQVPKLLIKGGVEVLEVKTGLDAITEVECFLNPEMGDPD	60
BAF93379.1	MAPTKRKGECPGAAPKKPKEPVQVPKLLIKGGVEVLEVKTGLDAITEVECFLNPEMGDPD	60
BAF42913.1	MAPTKRKGECPGAAPKKPKEPVQVPKLLIKGGVEVLEVKTGLDAITEVECFLNPEMGDPD	60
BAF42931.1	MAPTKRKGECPGAAPKKPKEPVQVPKLLIKGGVEVLEVKTGLDAITEVECFLNPEMGDPD	60
BAF43015.1	MAPTKRKGECPGAAPKKPKEPVQVPKLLIKGGVEVLEVKTGLDAITEVECFLNPEMGDPD	60
ABI94629.1	MAPTKRKGECPGAAPKKPKEPVQVPKLLIKGGVEVLEVKTGLDAITEVECFLNPEMGDPD	60
ABI94653.1	MAPTKRKGECPGAAPKKPKEPVQVPKLLIKGGVEVLEVKTGLDAITEVECFLNPEMGDPD	60
ABI94707.1	MAPTKRKGECPGAAPKKPKEPVQVPKLLIKGGVEVLEVKTGLDAITEVECFLNPEMGDPD	60
BAF03085.1	MAPTKRKGECPGAAPKKPKEPVQVPKLLIKGGVEVLEVKTGLDAITEVECFLNPEMGDPD	60
BAF03091.1	MAPTKRKGECPGAAPKKPKEPVQVPKLLIKGGVEVLEVKTGLDAITEVECFLNPEMGDPD	60
BAF03103.1	MAPTKRKGECPGAAPKKPKEPVQVPKLLIKGGVEVLEVKTGLDAITEVECFLNPEMGDPD	60
BAF03109.1	MAPTKRKGECPGAAPKKPKEPVQVPKLLIKGGVEVLEVKTGLDAITEVECFLNPEMGDPD	60
BAF93325.1	MAPTKRKGECPGAAPKKPKEPVQVPKLLIKGGVEVLEVKTGLDAITEVECFLNPEMGDPD	60
QTJ14934.1	MAPTKRKGECPGAAPKKPKEPVQVPKLLIKGGVEVLEVKTGLDAITEVECFLNPEMGDPD	60
QTJ14994.1	MAPTKRKGECPGAAPKKPKEPVQVPKLLIKGGVEVLEVKTGLDAITEVECFLNPEMGDPD	60
AFA41886.1	MAPTKRKGECPGAAPKKPKEPVQVPKLLIKGGVEVLEVKTGLDAITEVECFLNPEMGDPB	60
AFQ31650.1	MAPTKRKGECPGAAPKKPKEPVQVPKLLIKGGVEVLEVKTGLDAITEVECFLNPEMGDPD	60
AAT47347.1	MAPTKRKGECPGAAPKKPKEPVQVPKLLIKGGVEVLEVKTGLDAITEVECFLNPEMGDPD	60
ABI94617.1	MAPTKRKGECPGAAPKKPKEPMQVPKLLIKGGVEVLEVKTGLDAITEVECFLNPEMGDPD	60
BAF93319.1	MAPTKRKGECPGAAPKKPKEPVQVPKLLIKGGVEVLEVKTGLDAITEVECFLNPEMGDPD	60

CBX88314.1	MAPTKRKGECPGAAPKKPKEPVQVPKLLIKGGVEVLEVKTGLDAITEVECFLNPEMGDPD	60
ALF06320.1	MAPTKRKGECPGAAPKKPKEPVQVPKLLIKGGVEVLEVKTGLDAITEVECFLNPEMGDPD	60
WLD47830.1	MAPTKRKGECPGAAPKKPKEPVQVPKLLIKGGVEVLEVKTGLDAITEVECFLNPEMGDPD	60
WLD47866.1	MAPTKRKGECPGAAPKKPKEPVQVPKLLIKGGVEVLEVKTGLDAITEVECFLNPEMGDPD	60
UTM61939.1	MAPTKRKGECPGAAPKKPKEPVQVPKLLIKGGVEVLEVKTGLDAITEVECFLNPEMGDPD	60
UTM62023.1	MAPTKRKGECPGAAPKKPKEPVQVPKLLIKGGVEVLEVKTGLDAITEVECFLNPEMGDPD	60
UTM62029.1	MAPTKRKGECPGAAPKKPKEPVQVPKLLIKGGVEVLEVKTGLDAITEVECFLNPEMGDPD	60
UTM62053.1	MAPTKRKGECPGAAPKKPKEPVQVPKLLIKGGVEVLEVKTGLDAITEVECFLNPEMGDPD	60
UTM62059.1	MAPTKRKGECPGAAPKKPKEPVQVPKLLIKGGVEVLEVKTGLDAITEVECFLNPEMGDPD	60
UTM62065.1	MAPTKRKGECPGAAPKKPKEPVQVPKLLIKGGVEVLEVKTGLDAITEVECFLNPEMGDPD	60
UTM62317.1	MAPTKRKGECPGAAPKKPKEPVQVPKLLIKGGVEVLEVKTGLDAITEVECFLNPEMGDPD	60
UTM62323.1	MAPTKRKGECPGAAPKKPKEPVQVPKLLIKGGVEVLEVKTGLDAITEVECFLNPEMGDPD	60
UTM62353.1	MAPTKRKGECPGAAPKKPKEPVQVPKLLIKGGVEVLEVKTGLDAITEVECFLNPEMGDPD	60
UTM62473.1	MAPTKRKGECPGAAPKKPKEPVQVPKLLIKGGVEVLEVKTGLDAITEVECFLNPEMGDPD	60
UTM62491.1	MAPTKRKGECPGAAPKKPKEPVQVPKLLIKGGVEVLEVKTGLDAITEVECFLNPEMGDPD	60
UTM62497.1	MAPTKRKGECPGAAPKKPKEPVQVPKLLIKGGVEVLEVKTGLDAITEVECFLNPEMGDPD	60
UTM62503.1	MAPTKRKGECPGAAPKKPKEPVQVPKLLIKGGVEVLEVKTGLDAITEVECFLNPEMGDPD	60
QTJ14988.1	MAPTKRKGECPGAAPKKPKEPVQVPKLLIKGGVEVLEVKTGLDAITEVECFLNPEMGDPD	60
QTJ15024.1	MAPTKRKGECPGAAPKKPKEPVQVPKLLIKGGVEVLEVKTGLDAITEVECFLNPEMGDPD	60
QTJ15030.1	MAPTKRKGECPGAAPKKPKEPVQVPKLLIKGGVEVLEVKTGLDAITEVECFLNPEMGDPD	60
QTJ15102.1	MAPTKRKGECPGAAPKKPKEPVQVPKLLIKGGVEVLEVKTGLDAITEVECFLNPEMGDPD	60
ALF06300.1	MAPTKRKGECPGAAPKKPKEPVQVPKLLIKGGVEVLEVKTGLDAITEVECFLNPEMGDPD	60
AHU87342.1	MAPTKRKGECPGAAPKKPKEPVQVPKLLIKGGVEVLEVKTGLDAITEVECFLNPEMGDPD	60
AHU87343.1	MAPTKRKGECPGAAPKKPKEPVQVPKLLIKGGVEVLEVKTGLDAITEVECFLNPEMGDPD	60
AHU87346.1	MAPTKRKGECPGAAPKKPKEPVQVPKLLIKGGVEVLEVKTGLDAITEVECFLNPEMGDPD	60
AHU87347.1	MAPTKRKGECPGAAPKKPKEPVQVPKLLIKGGVEVLEVKTGLDAITEVECFLNPEMGDPD	60
AHU87349.1	MAPTKRKGECPGAAPKKPKEPVQVPKLLIKGGVEVLEVKTGLDAITEVECFLNPEMGDPD	60
AHU87356.1	MAPTKRKGECPGAAPKKPKEPVQVPKLLIKGGVEVLEVKTGLDAITEVECFLNPEMGDPD	60
AHU87357.1	MAPTKRKGECPGAAPKKPKEPVQVPKLLIKGGVEVLEVKTGLDAITEVECFLNPEMGDPD	60
AHU87358.1	MAPTKRKGECPGAAPKKPKEPVQVPKLLIKGGVEVLEVKTGLDAITEVECFLNPEMGDPD	60
AHU87359.1	MAPTKRKGECPGAAPKKPKEPVQVPKLLIKGGVEVLEVKTGLDAITEVECFLNPEMGDPD	60
AFQ31643.1	MAPTKRKGECPGAAPKKPKEPVQVPKLLIKGGVEVLEVKTGLDAITEVECFLNPEMGDPD	60
AFQ31656.1	MAPTKRKGECPGAAPKKPKEPVQVPKLLIKGGVEVLEVKTGLDAITEVECFLNPEMGDPD	60
AFA41879.1	MAPTKRKGECPGAAPKKPKEPVQVPKLLIKGGVEVLEVKTGLDAITEVECFLNPEMGDPD	60
AFA41897.1	MAPTKRKGECPGAAPKKPKEPVQVPKLLIKGGVEVLEVKTGLDAITEVECFLNPEMGDPD	60
AFA41900.1	MAPTKRKGECPGAAPKKPKEPVQVPKLLIKGGVEVLEVKTGLDAITEVECFLNPEMGDPD	60
AEO89580.1	MAPTKRKGECPGAAPKKPKEPVQVPKLLIKGGVEVLEVKTGLDAITEVECFLNPEMGDPD	60
AEO89592.1	MAPTKRKGECPGAAPKKPKEPVQVPKLLIKGGVEVLEVKTGLDAITEVECFLNPEMGDPD	60
BAF93277.1	MAPTKRKGECPGAAPKKPKEPVQVPKLLIKGGVEVLEVKTGLDAITEVECFLNPEMGDPD	60
BAF93289.1	MAPTKRKGECPGAAPKKPKEPVQVPKLLIKGGVEVLEVKTGLDAITEVECFLNPEMGDPD	60
BAF93307.1	MAPTKRKGECPGAAPKKPKEPVQVPKLLIKGGVEVLEVKTGLDAITEVECFLNPEMGDPD	60
BAF93337.1	MAPTKRKGECPGAAPKKPKEPVQVPKLLIKGGVEVLEVKTGLDAITEVECFLNPEMGDPD	60
BAF42895.1	MAPTKRKGECPGAAPKKPKEPVQVPKLLIKGGVEVLEVKTGLDAITEVECFLNPEMGDPD	60
BAF42919.1	MAPTKRKGECPGAAPKKPKEPVQVPKLLIKGGVEVLEVKTGLDAITEVECFLNPEMGDPD	60
BAF42943.1	MAPTKRKGECPGAAPKKPKEPVQVPKLLIKGGVEVLEVKTGLDAITEVECFLNPEMGDPD	60
BAF42949.1	MAPTKRKGECPGAAPKKPKEPVQVPKLLIKGGVEVLEVKTGLDAITEVECFLNPEMGDPD	60
ABI94665.1	MAPTKRKGECPGAAPKKPKEPVQVPKLLIKGGVEVLEVKTGLDAITEVECFLNPEMGDPD	60
ABI94677.1	MAPTKRKGECPGAAPKKPKEPVQVPKLLIKGGVEVLEVKTGLDAITEVECFLNPEMGDPD	60
BAF02939.1	MAPTKRKGECPGAAPKKPKEPVQVPKLLIKGGVEVLEVKTGLDAITEVECFLNPEMGDPD	60
AAT47353.1	MAPTKRKGECPGAAPKKPKEPVQVPKLLIKGGVEVLEVKTGLDAITEVECFLNPEMGDPD	60
AAT47359.1	MAPTKRKGECPGAAPKKPKEPVQVPKLLIKGGVEVLEVKTGLDAITEVECFLNPEMGDPD	60
CAA40247.1	MAPTKRKGECPGAAPKKPKEPVQVPKLLIKGGVEVLEVKTGLDAITEVECFLNPEMGDPD	60
QTJ14982.1	MAPTKRKGECPGAAPKKPKEPVQVPKLLIKGGVEVLEVKTGLDAITEVECFLNPEMGDPD	60
QTJ15126.1	MAPTKRKGECPGAAPKKPKEPVQVPKLLIKGGVEVLEVKTGLDAITEVECFLNPEMGDPD	60
ABI94635.1	MAPTKRKGECPGAAPKKPKEPVQVPKLLIKGGVEVLEVKTGVDAITEVECFLNPEMGDPD	60
ALF06335.1	MAPTKRKGECPGAAPKKPKEPVQVPKLLIKGGVEVLEVKTGLDAITEVECFLNPEMGDPD	60
AHU87348.1	MAPTKRKGECPGAAPKKPKEPVQVPKLLIKGGVEVLEVKTGLDAITEVECFLNPEMGDPD	60
AFQ31649.1	MAPTKRKGECPGAAPKKPKEPVQVPKLLIKGGVEVLEVKTGLDAITEVECFLNPEMGDPD	60
AFQ31654.1	MAPTKRKGECPGAAPKKPKEPVQVPKLLIKGGVEVLEVKTGLDAITEVECFLNPEMGDPD	60
BAF42907.1	MAPTKRKGECPGAAPKKPKEPVQVPKLLIKGGVEVLEVKTGLDAITEVECFLNPEMGDPD	60
WLD47824.1	MAPTKRKGECPGAAPKKPKEPVQVPKLLIKGGVEVLEVKTGLDAITEVECFLNPEMGDPD	60
WLD47848.1	MAPTKRKGECPGAAPKKPKEPVQVPKLLIKGGVEVLEVKTGLDAITEVECFLNPEMGDPD	60
WLD47854.1	MAPTKRKGECPGAAPKKPKEPVQVPKLLIKGGVEVLEVKTGLDAITEVECFLNPEMGDPD	60
WLD47860.1	MAPTKRKGECPGAAPKKPKEPVQVPKLLIKGGVEVLEVKTGLDAITEVECFLNPEMGDPD	60

```
WLD47878.1      MAPTKRKGECPGAAPKKPKEPVQVPKLLIKGGVEVLEVKTGLDAITEVECFLNPEMGDPD 60
WLD47908.1      MAPTKRKGECPGAAPKKPKEPVQVPKLLIKGGVEVLEVKTGLDAITEVECFLNPEMGDPD 60
WLD47914.1      MAPTKRKGECPGAAPKKPKEPVQVPKLLIKGGVEVLEVKTGLDAITEVECFLNPEMGDPD 60
WLD47920.1      MAPTKRKGECPGAAPKKPKEPVQVPKLLIKGGVEVLEVKTGLDAITEVECFLNPEMGDPD 60
WLD47926.1      MAPTKRKGECPGAAPKKPKEPVQVPKLLIKGGVEVLEVKTGLDAITEVECFLNPEMGDPD 60
WLD47932.1      MAPTKRKGECPGAAPKKPKEPVQVPKLLIKGGVEVLEVKTGLDAITEVECFLNPEMGDPD 60
WLD47950.1      MAPTKRKGECPGAAPKKPKEPVQVPKLLIKGGVEVLEVKTGLDAITEVECFLNPEMGDPD 60
WLD47962.1      MAPTKRKGECPGAAPKKPKEPVQVPKLLIKGGVEVLEVKTGLDAITEVECFLNPEMGDPD 60
WLD47974.1      MAPTKRKGECPGAAPKKPKEPVQVPKLLIKGGVEVLEVKTGLDAITEVECFLNPEMGDPD 60
WLD47998.1      MAPTKRKGECPGAAPKKPKEPVQVPKLLIKGGVEVLEVKTGLDAITEVECFLNPEMGDPD 60
WLD48004.1      MAPTKRKGECPGAAPKKPKEPVQVPKLLIKGGVEVLEVKTGLDAITEVECFLNPEMGDPD 60
WLD48016.1      MAPTKRKGECPGAAPKKPKEPVQVPKLLIKGGVEVLEVKTGLDAITEVECFLNPEMGDPD 60
WLD48022.1      MAPTKRKGECPGAAPKKPKEPVQVPKLLIKGGVEVLEVKTGLDAITEVECFLNPEMGDPD 60
WLD48034.1      MAPTKRKGECPGAAPKKPKEPVQVPKLLIKGGVEVLEVKTGLDAITEVECFLNPEMGDPD 60
UTM61909.1      MAPTKRKGECPGAAPKKPKEPVQVPKLLIKGGVEVLEVKTGLDAITEVECFLNPEMGDPD 60
UTM61915.1      MAPTKRKGECPGAAPKKPKEPVQVPKLLIKGGVEVLEVKTGLDAITEVECFLNPEMGDPD 60
UTM61921.1      MAPTKRKGECPGAAPKKPKEPVQVPKLLIKGGVEVLEVKTGLDAITEVECFLNPEMGDPD 60
UTM61927.1      MAPTKRKGECPGAAPKKPKEPVQVPKLLIKGGVEVLEVKTGLDAITEVECFLNPEMGDPD 60
UTM61933.1      MAPTKRKGECPGAAPKKPKEPVQVPKLLIKGGVEVLEVKTGLDAITEVECFLNPEMGDPD 60
UTM61963.1      MAPTKRKGECPGAAPKKPKEPVQVPKLLIKGGVEVLEVKTGLDAITEVECFLNPEMGDPD 60
UTM61969.1      MAPTKRKGECPGAAPKKPKEPVQVPKLLIKGGVEVLEVKTGLDAITEVECFLNPEMGDPD 60
UTM61975.1      MAPTKRKGECPGAAPKKPKEPVQVPKLLIKGGVEVLEVKTGLDAITEVECFLNPEMGDPD 60
UTM61981.1      MAPTKRKGECPGAAPKKPKEPVQVPKLLIKGGVEVLEVKTGLDAITEVECFLNPEMGDPD 60
UTM62005.1      MAPTKRKGECPGAAPKKPKEPVQVPKLLIKGGVEVLEVKTGLDAITEVECFLNPEMGDPD 60
UTM62017.1      MAPTKRKGECPGAAPKKPKEPVQVPKLLIKGGVEVLEVKTGLDAITEVECFLNPEMGDPD 60
UTM62035.1      MAPTKRKGECPGAAPKKPKEPVQVPKLLIKGGVEVLEVKTGLDAITEVECFLNPEMGDPD 60
UTM62041.1      MAPTKRKGECPGAAPKKPKEPVQVPKLLIKGGVEVLEVKTGLDAITEVECFLNPEMGDPD 60
UTM62047.1      MAPTKRKGECPGAAPKKPKEPVQVPKLLIKGGVEVLEVKTGLDAITEVECFLNPEMGDPD 60
UTM62083.1      MAPTKRKGECPGAAPKKPKEPVQVPKLLIKGGVEVLEVKTGLDAITEVECFLNPEMGDPD 60
UTM62089.1      MAPTKRKGECPGAAPKKPKEPVQVPKLLIKGGVEVLEVKTGLDAITEVECFLNPEMGDPD 60
UTM62095.1      MAPTKRKGECPGAAPKKPKEPVQVPKLLIKGGVEVLEVKTGLDAITEVECFLNPEMGDPD 60
UTM62149.1      MAPTKRKGECPGAAPKKPKEPVQVPKLLIKGGVEVLEVKTGLDAITEVECFLNPEMGDPD 60
UTM62161.1      MAPTKRKGECPGAAPKKPKEPVQVPKLLIKGGVEVLEVKTGLDAITEVECFLNPEMGDPD 60
UTM62167.1      MAPTKRKGECPGAAPKKPKEPVQVPKLLIKGGVEVLEVKTGLDAITEVECFLNPEMGDPD 60
UTM62173.1      MAPTKRKGECPGAAPKKPKEPVQVPKLLIKGGVEVLEVKTGLDAITEVECFLNPEMGDPD 60
UTM62179.1      MAPTKRKGECPGAAPKKPKEPVQVPKLLIKGGVEVLEVKTGLDAITEVECFLNPEMGDPD 60
UTM62185.1      MAPTKRKGECPGAAPKKPKEPVQVPKLLIKGGVEVLEVKTGLDAITEVECFLNPEMGDPD 60
UTM62191.1      MAPTKRKGECPGAAPKKPKEPVQVPKLLIKGGVEVLEVKTGLDAITEVECFLNPEMGDPD 60
UTM62197.1      MAPTKRKGECPGAAPKKPKEPVQVPKLLIKGGVEVLEVKTGLDAITEVECFLNPEMGDPD 60
UTM62203.1      MAPTKRKGECPGAAPKKPKEPVQVPKLLIKGGVEVLEVKTGLDAITEVECFLNPEMGDPD 60
UTM62209.1      MAPTKRKGECPGAAPKKPKEPVQVPKLLIKGGVEVLEVKTGLDAITEVECFLNPEMGDPD 60
UTM62227.1      MAPTKRKGECPGAAPKKPKEPVQVPKLLIKGGVEVLEVKTGLDAITEVECFLNPEMGDPD 60
UTM62239.1      MAPTKRKGECPGAAPKKPKEPVQVPKLLIKGGVEVLEVKTGLDAITEVECFLNPEMGDPD 60
UTM62245.1      MAPTKRKGECPGAAPKKPKEPVQVPKLLIKGGVEVLEVKTGLDAITEVECFLNPEMGDPD 60
UTM62251.1      MAPTKRKGECPGAAPKKPKEPVQVPKLLIKGGVEVLEVKTGLDAITEVECFLNPEMGDPD 60
UTM62257.1      MAPTKRKGECPGAAPKKPKEPVQVPKLLIKGGVEVLEVKTGLDAITEVECFLNPEMGDPD 60
UTM62263.1      MAPTKRKGECPGAAPKKPKEPVQVPKLLIKGGVEVLEVKTGLDAITEVECFLNPEMGDPD 60
UTM62269.1      MAPTKRKGECPGAAPKKPKEPVQVPKLLIKGGVEVLEVKTGLDAITEVECFLNPEMGDPD 60
UTM62287.1      MAPTKRKGECPGAAPKKPKEPVQVPKLLIKGGVEVLEVKTGLDAITEVECFLNPEMGDPD 60
UTM62293.1      MAPTKRKGECPGAAPKKPKEPVQVPKLLIKGGVEVLEVKTGLDAITEVECFLNPEMGDPD 60
UTM62299.1      MAPTKRKGECPGAAPKKPKEPVQVPKLLIKGGVEVLEVKTGLDAITEVECFLNPEMGDPD 60
UTM62305.1      MAPTKRKGECPGAAPKKPKEPVQVPKLLIKGGVEVLEVKTGLDAITEVECFLNPEMGDPD 60
UTM62311.1      MAPTKRKGECPGAAPKKPKEPVQVPKLLIKGGVEVLEVKTGLDAITEVECFLNPEMGDPD 60
UTM62359.1      MAPTKRKGECPGAAPKKPKEPVQVPKLLIKGGVEVLEVKTGLDAITEVECFLNPEMGDPD 60
UTM62365.1      MAPTKRKGECPGAAPKKPKEPVQVPKLLIKGGVEVLEVKTGLDAITEVECFLNPEMGDPD 60
UTM62371.1      MAPTKRKGECPGAAPKKPKEPVQVPKLLIKGGVEVLEVKTGLDAITEVECFLNPEMGDPD 60
UTM62377.1      MAPTKRKGECPGAAPKKPKEPVQVPKLLIKGGVEVLEVKTGLDAITEVECFLNPEMGDPD 60
UTM62455.1      MAPTKRKGECPGAAPKKPKEPVQVPKLLIKGGVEVLEVKTGLDAITEVECFLNPEMGDPD 60
UTM62461.1      MAPTKRKGECPGAAPKKPKEPVQVPKLLIKGGVEVLEVKTGLDAITEVECFLNPEMGDPD 60
UTM62485.1      MAPTKRKGECPGAAPKKPKEPVQVPKLLIKGGVEVLEVKTGLDAITEVECFLNPEMGDPD 60
UTM62509.1      MAPTKRKGECPGAAPKKPKEPVQVPKLLIKGGVEVLEVKTGLDAITEVECFLNPEMGDPD 60
UTM62515.1      MAPTKRKGECPGAAPKKPKEPVQVPKLLIKGGVEVLEVKTGLDAITEVECFLNPEMGDPD 60
UTM62521.1      MAPTKRKGECPGAAPKKPKEPVQVPKLLIKGGVEVLEVKTGLDAITEVECFLNPEMGDPD 60
UTM62527.1      MAPTKRKGECPGAAPKKPKEPVQVPKLLIKGGVEVLEVKTGLDAITEVECFLNPEMGDPD 60
```

```
UTM62539.1        MAPTKRKGECPGAAPKKPKEPVQVPKLLIKGGVEVLEVKTGLDAITEVECFLNPEMGDPD  60
UTM62557.1        MAPTKRKGECPGAAPKKPKEPVQVPKLLIKGGVEVLEVKTGLDAITEVECFLNPEMGDPD  60
UTM62563.1        MAPTKRKGECPGAAPKKPKEPVQVPKLLIKGGVEVLEVKTGLDAITEVECFLNPEMGDPD  60
UTM62569.1        MAPTKRKGECPGAAPKKPKEPVQVPKLLIKGGVEVLEVKTGLDAITEVECFLNPEMGDPD  60
UTM62575.1        MAPTKRKGECPGAAPKKPKEPVQVPKLLIKGGVEVLEVKTGLDAITEVECFLNPEMGDPD  60
UTM62581.1        MAPTKRKGECPGAAPKKPKEPVQVPKLLIKGGVEVLEVKTGLDAITEVECFLNPEMGDPD  60
UTM62605.1        MAPTKRKGECPGAAPKKPKEPVQVPKLLIKGGVEVLEVKTGLDAITEVECFLNPEMGDPD  60
UTM62617.1        MAPTKRKGECPGAAPKKPKEPVQVPKLLIKGGVEVLEVKTGLDAITEVECFLNPEMGDPD  60
UTM62623.1        MAPTKRKGECPGAAPKKPKEPVQVPKLLIKGGVEVLEVKTGLDAITEVECFLNPEMGDPD  60
UTM62629.1        MAPTKRKGECPGAAPKKPKEPVQVPKLLIKGGVEVLEVKTGLDAITEVECFLNPEMGDPD  60
UTM62635.1        MAPTKRKGECPGAAPKKPKEPVQVPKLLIKGGVEVLEVKTGLDAITEVECFLNPEMGDPD  60
QTJ15048.1        MAPTKRKGECPGAAPKKPKEPVQVPKLLIKGGVEVLEVKTGLDAITEVECFLNPEMGDPD  60
QTJ15144.1        MAPTKRKGECPGAAPKKPKEPVQVPKLLIKGGVEVLEVKTGLDAITEVECFLNPEMGDPD  60
AHU87360.1        MAPTKRKGECPGAAPKKPKEPVQVPKLLIKGGVEVLEVKTGLDAITEVECFLNPEMGDPD  60
AHU87361.1        MAPTKRKGECPGAAPKKPKEPVQVPKLLIKGGVEVLEVKTGLDAITEVECFLNPEMGDPD  60
AHU87366.1        MAPTKRKGECPGAAPKKPKEPVQVPKLLIKGGVEVLEVKTGLDAITEVECFLNPEMGDPD  60
AHU87367.1        MAPTKRKGECPGAAPKKPKEPVQVPKLLIKGGVEVLEVKTGLDAITEVECFLNPEMGDPD  60
AFQ31652.1        MAPTKRKGECPGAAPKKPKEPVQVPKLLIKGGVEVLEVKTGLDAITEVECFLNPEMGDPD  60
AFA41888.1        MAPTKRKGECPGAAPKKPKEPVQVPKLLIKGGVEVLEVKTGLDAITEVECFLNPEMGDPD  60
AFA41893.1        MAPTKRKGECPGAAPKKPKEPVQVPKLLIKGGVEVLEVKTGLDAITEVECFLNPEMGDPD  60
AFA41894.1        MAPTKRKGECPGAAPKKPKEPVQVPKLLIKGGVEVLEVKTGLDAITEVECFLNPEMGDPD  60
AFA41899.1        MAPTKRKGECPGAAPKKPKEPVQVPKLLIKGGVEVLEVKTGLDAITEVECFLNPEMGDPD  60
AFA41904.1        MAPTKRKGECPGAAPKKPKEPVQVPKLLIKGGVEVLEVKTGLDAITEVECFLNPEMGDPD  60
AFA41905.1        MAPTKRKGECPGAAPKKPKEPVQVPKLLIKGGVEVLEVKTGLDAITEVECFLNPEMGDPD  60
AFA41908.1        MAPTKRKGECPGAAPKKPKEPVQVPKLLIKGGVEVLEVKTGLDAITEVECFLNPEMGDPD  60
AFA41911.1        MAPTKRKGECPGAAPKKPKEPVQVPKLLIKGGVEVLEVKTGLDAITEVECFLNPEMGDPD  60
AFA41912.1        MAPTKRKGECPGAAPKKPKEPVQVPKLLIKGGVEVLEVKTGLDAITEVECFLNPEMGDPD  60
AFA41913.1        MAPTKRKGECPGAAPKKPKEPVQVPKLLIKGGVEVLEVKTGLDAITEVECFLNPEMGDPD  60
AFA41915.1        MAPTKRKGECPGAAPKKPKEPVQVPKLLIKGGVEVLEVKTGLDAITEVECFLNPEMGDPD  60
AFA41916.1        MAPTKRKGECPGAAPKKPKEPVQVPKLLIKGGVEVLEVKTGLDAITEVECFLNPEMGDPD  60
AFA41919.1        MAPTKRKGECPGAAPKKPKEPVQVPKLLIKGGVEVLEVKTGLDAITEVECFLNPEMGDPD  60
AFA41921.1        MAPTKRKGECPGAAPKKPKEPVQVPKLLIKGGVEVLEVKTGLDAITEVECFLNPEMGDPD  60
AFA41922.1        MAPTKRKGECPGAAPKKPKEPVQVPKLLIKGGVEVLEVKTGLDAITEVECFLNPEMGDPD  60
AFA41923.1        MAPTKRKGECPGAAPKKPKEPVQVPKLLIKGGVEVLEVKTGLDAITEVECFLNPEMGDPD  60
AFA41924.1        MAPTKRKGECPGAAPKKPKEPVQVPKLLIKGGVEVLEVKTGLDAITEVECFLNPEMGDPD  60
AFA41925.1        MAPTKRKGECPGAAPKKPKEPVQVPKLLIKGGVEVLEVKTGLDAITEVECFLNPEMGDPD  60
CBX88272.1        MAPTKRKGECPGAAPKKPKEPVQVPKLLIKGGVEVLEVKTGLDAITEVECFLNPEMGDPD  60
CBX88278.1        MAPTKRKGECPGAAPKKPKEPVQVPKLLIKGGVEVLEVKTGLDAITEVECFLNPEMGDPD  60
CBX88284.1        MAPTKRKGECPGAAPKKPKEPVQVPKLLIKGGVEVLEVKTGLDAITEVECFLNPEMGDPD  60
CBX88290.1        MAPTKRKGECPGAAPKKPKEPVQVPKLLIKGGVEVLEVKTGLDAITEVECFLNPEMGDPD  60
CBX88296.1        MAPTKRKGECPGAAPKKPKEPVQVPKLLIKGGVEVLEVKTGLDAITEVECFLNPEMGDPD  60
CBX88308.1        MAPTKRKGECPGAAPKKPKEPVQVPKLLIKGGVEVLEVKTGLDAITEVECFLNPEMGDPD  60
CBX88332.1        MAPTKRKGECPGAAPKKPKEPVQVPKLLIKGGVEVLEVKTGLDAITEVECFLNPEMGDPD  60
CBX88356.1        MAPTKRKGECPGAAPKKPKEPVQVPKLLIKGGVEVLEVKTGLDAITEVECFLNPEMGDPD  60
CBX88362.1        MAPTKRKGECPGAAPKKPKEPVQVPKLLIKGGVEVLEVKTGLDAITEVECFLNPEMGDPD  60
BAF93313.1        MAPTKRKGECPGAAPKKPKEPVQVPKLLIKGGVEVLEVKTGLDAITEVECFLNPEMGDPD  60
BAF42955.1        MAPTKRKGECPGAAPKKPKEPVQVPKLLIKGGVEVLEVKTGLDAITEVECFLNPEMGDPD  60
BAF43021.1        MAPTKRKGECPGAAPKKPKEPVQVPKLLIKGGVEVLEVKTGLDAITEVECFLNPEMGDPD  60
ABI94611.1        MAPTKRKGECPGAAPKKPKEPVQVPKLLIKGGVEVLEVKTGLDAITEVECFLNPEMGDPD  60
ABI94641.1        MAPTKRKGECPGAAPKKPKEPVQVPKLLIKGGVEVLEVKTGLDAITEVECFLNPEMGDPD  60
ABI94647.1        MAPTKRKGECPGAAPKKPKEPVQVPKLLIKGGVEVLEVKTGLDAITEVECFLNPEMGDPD  60
WLD47896.1        MAPTKRKGECPGAAPKKPKEPVQVPKLLIKGGVEVLEVKTGLDAITEVECFLNPEMGDPD  60
WLD48028.1        MAPTKRKGECPGAAPKKPKEPVQVPKLLIKGGVEVLEVKTGLDAITEVECFLNPEMGDPD  60
BAG84440.1        MAPTKRKGECPGAAPKKPKEPVQVPKLLIKGGVEVLEVKTGLDAITEVECFLNPEMGDPD  60
BAF93283.1        MAPTKRKGECPGAAPKKPKEPVQVPKLLIKGGVEVLEVKTGLDAITEVECFLNPEMGDPD  60
BAF93361.1        MAPTKRKGECPGAAPKKPKEPVQVPKLLIKGGVEVLEVKTGLDAITEVECFLNPEMGDPD  60
WLD48040.1        MAPTKRKGECPGAAPKKPKEPVQVPKLLIKGGVEVLEVKTGLDAITEVECFLNPEMGDPD  60
UTM62611.1        MAPTKRKGECPGAAPKKPKEPVQVPKLLIKGGVEVLEVKTGLDAITEVECFLNPEMGDPD  60
AFQ31645.1        MAPTKRKGECPGAAPKKPKEPVQVPKLLIKGGVEVLEVKTGLDAITEVECFLNPEMGDPD  60
AFA41909.1        MAPTKRKGECPGAAPKKPKEPVQVPKLLIKGGVEVLEVKTGLDAITEVECFLNPEMGDPD  60
ABI94713.1        MAPTKRKGECPGAAPKKPKEPVQVPKLLIKGGVEVLEVKTGLDAITEVECFLNPEMGDPD  60
ABD04662.1        MAPTKRKGECPGAAPKKPKEPVQVPKLLIKGGVEVLEVKTGLDAITEVECFLNPEMGDPN  60
QTJ15042.1        MAPTKRKGECPGAAPKKPKEPVQVPKLLIKGGVEVLEVKTGVDAITEVECFLNPEMGNPE  60
AGX89705.1        MAPTKRKGECPGAAPKKPKEPVQVPKLLIKGGVEVLEVKTGVDAITEVECFLNPEDQVSA  60
```

AGX89710.1	MAPTKRKGECPGAAPKKPKEPVQVPKLLIKGGVEVLEVKTGVDAITEVECFLNPEDQVSA	60
AGX89717.1	MAPTKRKGECPGAAPKKPKEPVQVPKLLIKGGVEVLEVKTGVDAITEVECFLNPEDQVSA	60
AFQ31655.1	MAPTKRKGECPGAAPKKPKEPVQVPKLLIKGGVEVLEVKTGVDAITEVECFLNPEMGDPD	60
AAA46882.1	MAPTKRKGECPGAAPKKPKEPVQVPKLLIKGGVEVLEVKTGVDAITEVECFLNPEMGDPD	60
P14996.1	MAPTKRKGECPGAAPKKPKEPVQVPKLLIKGGVEVLEVKTGVDAITEVECFLNPEMGDPD	60
AEO89615.1	MAPTKRKGECPGAAPKKPKEPVQVPKLLIKGGVEVLEVKTGVDAITEVECFLNPEMGDPD	60
BCV68929.1	MAPTKRKGECPGAAPKKPKEPVQVPKLLIKGGVEVLEVKTGVDAITEVECFLNPEMGDPD	60
QTJ14952.1	MAPTKRKGECPGAAPKKPKEPVQVPKLLIKGGVEVLEVKTGVDAITEVECFLNPEMGDPD	60
BAG75157.1	MAPTKRKGECPGAAPKKPKEPVQVPKLLIKGGVEVLEVKTGVDAITEVECFLNPEMGDPD	60
BAG75211.1	MAPTKRKGECPGAAPKKPKEPVQVPKLLIKGGVEVLEVKTGVDAITEVECFLNPEMGDPD	60
BAF03017.1	MAPTKRKGECPGAAPKKPKEPVQVPKLLIKGGVEVLEVKTGVDAITEVECFLNPEMGDPD	60
BAF42925.1	MAPTKRKGECPGAAPKKPKEPVQVPKLLIKGGVEVLEVKTGVDAITEVECFLNPEMGDPD	60
WLD47836.1	MAPTKRKGECPGAAPKKPKEPVQVPKLLIKGGVEVLEVKTGVDAITEVECFLNPEMGDPD	60
UEL36354.1	MAPTKRKGECPGAAPKKPKEPVQVPKLLIKGGVEVLEVKTGVDAITEVECFLNPEMGDPD	60
BAF42901.1	MAPTKRKGECPGAAPKKPKEPVQVPKLLIKGGVEVLEVKTGVDAITEVECFLNPEMGDPD	60
ABP87973.1	MAPTKRKGECPGAAPKKPKEPVQVPKLLIKGGVEVLEVKTGVDAITEVECFLNPEMGDPD	60
AFA41878.1	MAPTKRKGECPGAAPKKPKEPVQVPKLLIKGGVEVLEVKTGVDAITEVECFLNPEMGDPD	60
QHI34655.1	MAPTKRKGECPGAAPRKPKEPVQVPKLLIKGGVEVLEVKTGVDAITEVECFLNPEMGDPD	60
UTM61945.1	MAPTKRKGECPGAAPKKPKEPVQVPKLLIKGGVEVLEVKTGVDAITEVECFLNPEMGDPD	60
UTM61987.1	MAPTKRKGECPGAAPKKPKEPVQVPKLLIKGGVEVLEVKTGVDAITEVECFLNPEMGDPD	60
UTM62233.1	MAPTKRKGECPGAAPKKPKEPVQVPKLLIKGGVEVLEVKTGVDAITEVECFLNPEMGDPD	60
UTM62341.1	MAPTKRKGECPGAAPKKPKEPVQVPKLLIKGGVEVLEVKTGVDAITEVECFLNPEMGDPD	60
UTM62347.1	MAPTKRKGECPGAAPKKPKEPVQVPKLLIKGGVEVLEVKTGVDAITEVECFLNPEMGDPD	60
AHU87354.1	MAPTKRKGECPGAAPKKPKEPVQVPKLLIKGGVEVLEVKTGVDAITEVECFLNPEMGDPD	60
AHU87355.1	MAPTKRKGECPGAAPKKPKEPVQVPKLLIKGGVEVLEVKTGVDAITEVECFLNPEMGDPD	60
AHU87364.1	MAPTKRKGECPGAAPKKPKEPVQVPKLLIKGGVEVLEVKTGVDAITEVECFLNPEMGDPD	60
AHU87365.1	MAPTKRKGECPGAAPKKPKEPVQVPKLLIKGGVEVLEVKTGVDAITEVECFLNPEMGDPD	60
AFQ31638.1	MAPTKRKGECPGAAPKKPKEPVQVPKLLIKGGVEVLEVKTGVDAITEVECFLNPEMGDPD	60
AFA41874.1	MAPTKRKGECPGAAPKKPKEPVQVPKLLIKGGVEVLEVKTGVDAITEVECFLNPEMGDPD	60
AFA41875.1	MAPTKRKGECPGAAPKKPKEPVQVPKLLIKGGVEVLEVKTGVDAITEVECFLNPEMGDPD	60
AFA41896.1	MAPTKRKGECPGAAPKKPKEPVQVPKLLIKGGVEVLEVKTGVDAITEVECFLNPEMGDPD	60
BAF93367.1	MAPTKRKGECPGAAPKKPKEPVQVPKLLIKGGVEVLEVKTGVDAITEVECFLNPEMGDPD	60
CAA79596.1	MAPTKRKGECPGAAPKKPKEPVQVPKLLIKGGVEVLEVKTGVDAITEVECFLNPEMGDPD	60
AFA41883.1	MAPTKRKGECPGAAPKKPKEPVQVPKLLIKGGVEVLEVKTGVDAITEVECFLNPEMGDPD	60
WLD47812.1	MAPTKRKGECPGAAPKKPKEPVQVPKLLIKGGVEVLEVKTGVDAITEVECFLNPEMGNSD	60
BAG75277.1	MAPTKRKGECPGAAPKKPKEPVQVPKLLIKGGVEVLEVKTGVDAITEVECFLNPEMGDPD	60
BAF75138.1	MAPTKRKGECPGAAPKKPKEPVQVPKLLIKGGVEVLEVKTGVDAITEVECFLNPEMGDPD	60
BAF75102.1	MAPTKRKGECPGAAPKKPKEPVQVPKLLIKGGVEVLEVKTGVDAITEVECFLNPEMGDPD	60
AYO46643.1	MAPTKRKGECPGAAPRKPKDPVQVPKLLIKGGVEVLEVKTGVDAITEVECFLNPEMGDPD	60
QUW04844.1	MAPTKRKGECPGAAPKKPKEPVQVPKLLIKGGVEVLEVKTGVDAITEVECFLNPEMGDPD	60
AYO46683.1	MAPTKRKGECPGAAPKKPKDPVQVPKLLIKGGVEVLEVKTGVDAITEVECFLNPEMGDPD	60
AYO46645.1	MAPTKRKGECPGAAPKKPKDPVQVPKLLIKGGVEVLEVKTGVDAITEVECFLNPEMGDPY	60
BAF75180.1	MAPTKRKGECPGAAPKKPKDPVQVPKLLIKGGVEVLEVKTGVDAITEVECFLNPEMGDPD	60
BAF75204.1	MAPTKRKGECPGAAPKKPKDPVQVPKLLIKGGVEVLEVKTGVDAITEVECFLNPEMGDPD	60
AYO46631.1	MAPTKRKGECPGAAPKKPKDPVQVPKLLIKGGVEVLEVKTGVDAITEVECFLNPEMGDPD	60
AYO46608.1	MAPTKRKGECPGAAPKKPKDPVQVPKLLIKGGVEVLEVKTGVDAITEVECFLNPEMGDPD	60
AYO46611.1	MAPTKRKGECPGAAPKKPKDPVQVPKLLIKGGVEVLEVKTGVDAITEVECFLNPEMGDPD	60
AYO46615.1	MAPTKRKGECPGAAPKKPKDPVQVPKLLIKGGVEVLEVKTGVDAITEVECFLNPEMGDPD	60
AYO46639.1	MAPTKRKGECPGAAPKKPKDPVQVPKLLIKGGVEVLEVKTGVDAITEVECFLNPEMGDPD	60
AYO46649.1	MAPTKRKGECPGAAPKKPKDPVQVPKLLIKGGVEVLEVKTGVDAITEVECFLNPEMGDPD	60
AYO46655.1	MAPTKRKGECPGAAPKKPKDPVQVPKLLIKGGVEVLEVKTGVDAITEVECFLNPEMGDPD	60
AYO46664.1	MAPTKRKGECPGAAPKKPKDPVQVPKLLIKGGVEVLEVKTGVDAITEVECFLNPEMGDPD	60
AYO46669.1	MAPTKRKGECPGAAPKKPKDPVQVPKLLIKGGVEVLEVKTGVDAITEVECFLNPEMGDPD	60
AYO46685.1	MAPTKRKGECPGAAPKKPKDPVQVPKLLIKGGVEVLEVKTGVDAITEVECFLNPEMGDPD	60
AYO46686.1	MAPTKRKGECPGAAPKKPKDPVQVPKLLIKGGVEVLEVKTGVDAITEVECFLNPEMGDPD	60
AYO46690.1	MAPTKRKGECPGAAPKKPKDPVQVPKLLIKGGVEVLEVKTGVDAITEVECFLNPEMGDPD	60
AYO46627.1	MAPTKRKGECPGAAPKKPKDPVQVPKLLIKGGVEVLEVKTGVDAITEVECFLNPEMGDPD	60
AYO46630.1	MAPTKRKGECPGAAPKKPKDPVQVPKLLIKGGVEVLEVKTGVDAITEVECFLNPEMGDPD	60
AYO46656.1	MAPTKRKGECPGAAPKKPKDPVQVPKLLIKGGVEVLEVKTGVDAITEVECFLNPEMGDPD	60
BAG75217.1	MAPTKRKGECPGAAPKKPKEPVQVPKLLIKGGVEVLEVKTGVDAITEVECFLNPEMGDPD	60
BAG75229.1	MAPTKRKGECPGAAPKKPKEPVQVPKLLIKGGVEVLEVKTGVDAITEVECFLNPEMGDPD	60
BAG75235.1	MAPTKRKGECPGAAPKKPKEPVQVPKLLIKGGVEVLEVKTGVDAITEVECFLNPEMGDPD	60
BAG75259.1	MAPTKRKGECPGAAPKKPKEPVQVPKLLIKGGVEVLEVKTGVDAITEVECFLNPEMGDPD	60
BAF75150.1	MAPTKRKGECPGAAPKKPKEPVQVPKLLIKGGVEVLEVKTGVDAITEVECFLNPEMGDPD	60

```
BAF75156.1      MAPTKRKGECPGAAPKKPKEPVQVPKLLIKGGVEVLEVKTGVDAITEVECFLNPEMGDPD  60
BAF75174.1      MAPTKRKGECPGAAPKKPKEPVQVPKLLIKGGVEVLEVKTGVDAITEVECFLNPEMGDPD  60
BAE96083.1      MAPTKRKGECPGAAPKKPKEPVQVPKLLIKGGVEVLEVKTGVDAITEVECFLNPEMGDPD  60
BAE53654.1      MAPTKRKGECPGAAPKKPKEPVQVPKLLIKGGVEVLEVKTGVDAITEVECFLNPEMGDPD  60
BAF03035.1      MAPTKRKGECPGAAPKKPKEPVQVPKLLIKGGVEVLEVKTGVDAITEVECFLNPEMGDPD  60
QHI34656.1      MAPTKRKGECPGAAPKKPKEPVQVPKLLIKGGVEVLEVKTGLDAITEVECFLNPEMGDPD  60
ARE72396.1      MAPTKRKGECPGAAPKKPKEPVQVPKLLIKGGVEVLEVKTGVDAITEVECFLNPEMGDPD  60
AFA41885.1      MAPTKRKGECPGAAPKKPKEPVQVPKLLIKGGVEVLEVKTGVDAITEVECFLNPEMGDPD  60
BAG75271.1      MAPTKRKGECPGAAPKKPKDPVQVPKLLIKGGVEVLEVKTGVDAITEVECFLNPEMGDPD  60
BAF75090.1      MAPTKRKGECPGAAPKKPKDPVQVPKLLIKGGVEVLEVKTGVDAITEVECFLNPEMGDPD  60
BAF75306.1      MAPTKRKGECPGAAPKKPKDPVQVPKLLIKGGVEVLEVKTGVDAITEVECFLNPEMGDPD  60
BAF75342.1      MAPTKRKGECPGAAPKKPKDPVQVPKLLIKGGVEVLEVKTGVDAITEVECFLNPEMGDPD  60
BAF03029.1      MAPTKRKGECPGAAPKKPKDPVQVPKLLIKGGVEVLEVKTGVDAITEVECFLNPEMGDPD  60
BAG75205.1      MAPTKRKGECPGAAPKKPKEPVQVPKLLIKGGVEVLEVKTGVDAITEVECFLNPEMGDPD  60
BAG75223.1      MAPTKRKGECPGAAPKKPKEPVQVPKLLIKGGVEVLEVKTGVDAITEVECFLNPEMGDPD  60
BAF75144.1      MAPTKRKGECPGAAPKKPKEPVQVPKLLIKGGVEVLEVKTGVDAITEVECFLNPEMGDPD  60
BAF75162.1      MAPTKRKGECPGAAPKKPKEPVQVPKLLIKGGVEVLEVKTGVDAITEVECFLNPEMGDPD  60
BAF75168.1      MAPTKRKGECPGAAPKKPKEPVQVPKLLIKGGVEVLEVKTGVDAITEVECFLNPEMGDPD  60
BAF75240.1      MAPTKRKGECPGAAPKKPKEPVQVPKLLIKGGVEVLEVKTGVDAITEVECFLNPEMGDPD  60
BAF03023.1      MAPTKRKGECPGAAPKKPKEPVQVPKLLIKGGVEVLEVKTGVDAITEVECFLNPEMGDPD  60
BAE96077.1      MAPTKRKGECPGAAPKKPKEPVQVPKLLIKGGVEVLEVKTGVDAITEVECFLNPEMGDPD  60
BAG84476.1      MAPTKRKGECPGAAPKKPKEPVQVPKLLIKGGVEVLEVKTGVDAITEVECFLNPEMGDPD  60
AYO46661.1      MAPTKRKGECPGAAPKKPKEPVQVPKLLIKGGVEVLEVKTGVDAITEVECFLNPEMGDPD  60
AYO46634.1      MAPTKRKGECPGAAPKKPKEPVQVPKLLIKGGVEVLEVKTGVDAITEVECFLNPEMGDPD  60
AYO46657.1      MAPTKRKGECPGAAPKKPKEPVQVPKLLIKGGVEVLEVKTGVDAITEVECFLNPEMGDPD  60
BAG75379.1      MAPTKRKGECPGAAPKKPKEPVQVPKLLIKGGVEVLEVKTGVDAITEVECFLNPEMGDPD  60
BAG75409.1      MAPTKRKGECPGAAPKKPKEPVQVPKLLIKGGVEVLEVKTGVDAITEVECFLNPEMGDPD  60
BAG75415.1      MAPTKRKGECPGAAPKKPKEPVQVPKLLIKGGVEVLEVKTGVDAITEVECFLNPEMGDPD  60
BAF75096.1      MAPTKRKGECPGAAPKKPKEPVQVPKLLIKGGVEVLEVKTGVDAITEVECFLNPEMGDPD  60
BAF75192.1      MAPTKRKGECPGAAPKKPKEPVQVPKLLIKGGVEVLEVKTGVDAITEVECFLNPEMGDPD  60
BAF75198.1      MAPTKRKGECPGAAPKKPKEPVQVPKLLIKGGVEVLEVKTGVDAITEVECFLNPEMGDPD  60
BAF75210.1      MAPTKRKGECPGAAPKKPKEPVQVPKLLIKGGVEVLEVKTGVDAITEVECFLNPEMGDPD  60
BAF75234.1      MAPTKRKGECPGAAPKKPKEPVQVPKLLIKGGVEVLEVKTGVDAITEVECFLNPEMGDPD  60
BAF75264.1      MAPTKRKGECPGAAPKKPKEPVQVPKLLIKGGVEVLEVKTGVDAITEVECFLNPEMGDPD  60
BAF75270.1      MAPTKRKGECPGAAPKKPKEPVQVPKLLIKGGVEVLEVKTGVDAITEVECFLNPEMGDPD  60
BAF75282.1      MAPTKRKGECPGAAPKKPKEPVQVPKLLIKGGVEVLEVKTGVDAITEVECFLNPEMGDPD  60
BAF75300.1      MAPTKRKGECPGAAPKKPKEPVQVPKLLIKGGVEVLEVKTGVDAITEVECFLNPEMGDPD  60
BAF75312.1      MAPTKRKGECPGAAPKKPKEPVQVPKLLIKGGVEVLEVKTGVDAITEVECFLNPEMGDPD  60
BAF75318.1      MAPTKRKGECPGAAPKKPKEPVQVPKLLIKGGVEVLEVKTGVDAITEVECFLNPEMGDPD  60
BAF75324.1      MAPTKRKGECPGAAPKKPKEPVQVPKLLIKGGVEVLEVKTGVDAITEVECFLNPEMGDPD  60
BAF75336.1      MAPTKRKGECPGAAPKKPKEPVQVPKLLIKGGVEVLEVKTGVDAITEVECFLNPEMGDPD  60
AFA41889.1      MAPTKRKGECPGAAPKKPKEPVQVPKLLIKGGVEVLEVKTGVDAITEVECFLNPEMGDPD  60
UEL36357.1      MAPTKRKGECPGAAPKKPKEPVQVPKLLIKGGVEVLEVKTGVDAITEVECFLNPEMGDPD  60
BCV68920.1      MAPTKRKGECPGAAPKKPKEPVQVPKLLIKGGVEVLEVKTGVDAITEVECFLNPEMGDPD  60
WLD47992.1      MAPTKRKGECPGAAPKKPKEPVQVPKLLIKGGVEVLEVKTGVDAITEVECFLNPEMGDPD  60
UTM62413.1      MAPTKRKGECPGAAPKKPKEPVQVPKLLIKGGVEVLEVKTGVDAITEVECFLNPEMGDPD  60
AHU87344.1      MAPTKRKGECPGAAPKKPKEPVQVPKLLIKGGVEVLEVKTGVDAITEVECFLNPEMGDPD  60
AHU87345.1      MAPTKRKGECPGAAPKKPKEPVQVPKLLIKGGVEVLEVKTGVDAITEVECFLNPEMGDPD  60
QTJ14946.1      MAPTKRKGECPGAAPKKPKEPVQVPKLLIKGGVEVLEVKTGVDAITEVECFLNPEMGDPD  60
AYO46666.1      MAPTKRKGECPGAAPKKPKEPVQVPKLLIKGGVEVLEVKTGVDAITEVECFLNPEMGDPD  60
WLD47788.1      MAPTKRKGECPGAAPKKPKEPVQVPKLLIKGGVEVLEVKTGVDAITEVECFLNPEMGDPD  60
WLD47794.1      MAPTKRKGECPGAAPKKPKEPVQVPKLLIKGGVEVLEVKTGVDAITEVECFLNPEMGDPD  60
WLD47800.1      MAPTKRKGECPGAAPKKPKEPVQVPKLLIKGGVEVLEVKTGVDAITEVECFLNPEMGDPD  60
WLD47806.1      MAPTKRKGECPGAAPKKPKEPVQVPKLLIKGGVEVLEVKTGVDAITEVECFLNPEMGDPD  60
WLD47872.1      MAPTKRKGECPGAAPKKPKEPVQVPKLLIKGGVEVLEVKTGVDAITEVECFLNPEMGDPD  60
WLD47986.1      MAPTKRKGECPGAAPKKPKEPVQVPKLLIKGGVEVLEVKTGVDAITEVECFLNPEMGDPD  60
WLD48010.1      MAPTKRKGECPGAAPKKPKEPVQVPKLLIKGGVEVLEVKTGVDAITEVECFLNPEMGDPD  60
UTM61951.1      MAPTKRKGECPGAAPKKPKEPVQVPKLLIKGGVEVLEVKTGVDAITEVECFLNPEMGDPD  60
UTM61957.1      MAPTKRKGECPGAAPKKPKEPVQVPKLLIKGGVEVLEVKTGVDAITEVECFLNPEMGDPD  60
UTM61993.1      MAPTKRKGECPGAAPKKPKEPVQVPKLLIKGGVEVLEVKTGVDAITEVECFLNPEMGDPD  60
UTM61999.1      MAPTKRKGECPGAAPKKPKEPVQVPKLLIKGGVEVLEVKTGVDAITEVECFLNPEMGDPD  60
UTM62011.1      MAPTKRKGECPGAAPKKPKEPVQVPKLLIKGGVEVLEVKTGVDAITEVECFLNPEMGDPD  60
UTM62071.1      MAPTKRKGECPGAAPKKPKEPVQVPKLLIKGGVEVLEVKTGVDAITEVECFLNPEMGDPD  60
UTM62077.1      MAPTKRKGECPGAAPKKPKEPVQVPKLLIKGGVEVLEVKTGVDAITEVECFLNPEMGDPD  60
```

```
UTM62119.1          MAPTKRKGECPGAAPKKPKEPVQVPKLLIKGGVEVLEVKTGVDAITEVECFLNPEMGDPD  60
UTM62125.1          MAPTKRKGECPGAAPKKPKEPVQVPKLLIKGGVEVLEVKTGVDAITEVECFLNPEMGDPD  60
UTM62131.1          MAPTKRKGECPGAAPKKPKEPVQVPKLLIKGGVEVLEVKTGVDAITEVECFLNPEMGDPD  60
UTM62137.1          MAPTKRKGECPGAAPKKPKEPVQVPKLLIKGGVEVLEVKTGVDAITEVECFLNPEMGDPD  60
UTM62143.1          MAPTKRKGECPGAAPKKPKEPVQVPKLLIKGGVEVLEVKTGVDAITEVECFLNPEMGDPD  60
UTM62401.1          MAPTKRKGECPGAAPKKPKEPVQVPKLLIKGGVEVLEVKTGVDAITEVECFLNPEMGDPD  60
UTM62407.1          MAPTKRKGECPGAAPKKPKEPVQVPKLLIKGGVEVLEVKTGVDAITEVECFLNPEMGDPD  60
UTM62419.1          MAPTKRKGECPGAAPKKPKEPVQVPKLLIKGGVEVLEVKTGVDAITEVECFLNPEMGDPD  60
UTM62425.1          MAPTKRKGECPGAAPKKPKEPVQVPKLLIKGGVEVLEVKTGVDAITEVECFLNPEMGDPD  60
UTM62431.1          MAPTKRKGECPGAAPKKPKEPVQVPKLLIKGGVEVLEVKTGVDAITEVECFLNPEMGDPD  60
UTM62437.1          MAPTKRKGECPGAAPKKPKEPVQVPKLLIKGGVEVLEVKTGVDAITEVECFLNPEMGDPD  60
UTM62641.1          MAPTKRKGECPGAAPKKPKEPVQVPKLLIKGGVEVLEVKTGVDAITEVECFLNPEMGDPD  60
UTM62647.1          MAPTKRKGECPGAAPKKPKEPVQVPKLLIKGGVEVLEVKTGVDAITEVECFLNPEMGDPD  60
ALF06326.1          MAPTKRKGECPGAAPKKPKEPVQVPKLLIKGGVEVLEVKTGVDAITEVECFLNPEMGDPD  60
ALF06329.1          MAPTKRKGECPGAAPKKPKEPVQVPKLLIKGGVEVLEVKTGVDAITEVECFLNPEMGDPD  60
AHU87338.1          MAPTKRKGECPGAAPKKPKEPVQVPKLLIKGGVEVLEVKTGVDAITEVECFLNPEMGDPD  60
AHU87339.1          MAPTKRKGECPGAAPKKPKEPVQVPKLLIKGGVEVLEVKTGVDAITEVECFLNPEMGDPD  60
AHU87350.1          MAPTKRKGECPGAAPKKPKEPVQVPKLLIKGGVEVLEVKTGVDAITEVECFLNPEMGDPD  60
AHU87351.1          MAPTKRKGECPGAAPKKPKEPVQVPKLLIKGGVEVLEVKTGVDAITEVECFLNPEMGDPD  60
AHU87352.1          MAPTKRKGECPGAAPKKPKEPVQVPKLLIKGGVEVLEVKTGVDAITEVECFLNPEMGDPD  60
AHU87353.1          MAPTKRKGECPGAAPKKPKEPVQVPKLLIKGGVEVLEVKTGVDAITEVECFLNPEMGDPD  60
AFQ31653.1          MAPTKRKGECPGAAPKKPKEPVQVPKLLIKGGVEVLEVKTGVDAITEVECFLNPEMGDPD  60
AFA41884.1          MAPTKRKGECPGAAPKKPKEPVQVPKLLIKGGVEVLEVKTGVDAITEVECFLNPEMGDPD  60
CBX88320.1          MAPTKRKGECPGAAPKKPKEPVQVPKLLIKGGVEVLEVKTGVDAITEVECFLNPEMGDPD  60
CBX88326.1          MAPTKRKGECPGAAPKKPKEPVQVPKLLIKGGVEVLEVKTGVDAITEVECFLNPEMGDPD  60
CBX88338.1          MAPTKRKGECPGAAPKKPKEPVQVPKLLIKGGVEVLEVKTGVDAITEVECFLNPEMGDPD  60
CBX88344.1          MAPTKRKGECPGAAPKKPKEPVQVPKLLIKGGVEVLEVKTGVDAITEVECFLNPEMGDPD  60
BAG84452.1          MAPTKRKGECPGAAPKKPKEPVQVPKLLIKGGVEVLEVKTGVDAITEVECFLNPEMGDPD  60
BAG75385.1          MAPTKRKGECPGAAPKKPKEPVQVPKLLIKGGVEVLEVKTGVDAITEVECFLNPEMGDPD  60
BAG75445.1          MAPTKRKGECPGAAPKKPKEPVQVPKLLIKGGVEVLEVKTGVDAITEVECFLNPEMGDPD  60
BAF93343.1          MAPTKRKGECPGAAPKKPKEPVQVPKLLIKGGVEVLEVKTGVDAITEVECFLNPEMGDPD  60
BAF75066.1          MAPTKRKGECPGAAPKKPKEPVQVPKLLIKGGVEVLEVKTGVDAITEVECFLNPEMGDPD  60
BAF75072.1          MAPTKRKGECPGAAPKKPKEPVQVPKLLIKGGVEVLEVKTGVDAITEVECFLNPEMGDPD  60
BAF75078.1          MAPTKRKGECPGAAPKKPKEPVQVPKLLIKGGVEVLEVKTGVDAITEVECFLNPEMGDPD  60
BAF75084.1          MAPTKRKGECPGAAPKKPKEPVQVPKLLIKGGVEVLEVKTGVDAITEVECFLNPEMGDPD  60
BAF75108.1          MAPTKRKGECPGAAPKKPKEPVQVPKLLIKGGVEVLEVKTGVDAITEVECFLNPEMGDPD  60
BAF75132.1          MAPTKRKGECPGAAPKKPKEPVQVPKLLIKGGVEVLEVKTGVDAITEVECFLNPEMGDPD  60
BAF75216.1          MAPTKRKGECPGAAPKKPKEPVQVPKLLIKGGVEVLEVKTGVDAITEVECFLNPEMGDPD  60
BAF75222.1          MAPTKRKGECPGAAPKKPKEPVQVPKLLIKGGVEVLEVKTGVDAITEVECFLNPEMGDPD  60
BAF75228.1          MAPTKRKGECPGAAPKKPKEPVQVPKLLIKGGVEVLEVKTGVDAITEVECFLNPEMGDPD  60
BAF75246.1          MAPTKRKGECPGAAPKKPKEPVQVPKLLIKGGVEVLEVKTGVDAITEVECFLNPEMGDPD  60
BAF75252.1          MAPTKRKGECPGAAPKKPKEPVQVPKLLIKGGVEVLEVKTGVDAITEVECFLNPEMGDPD  60
BAF75258.1          MAPTKRKGECPGAAPKKPKEPVQVPKLLIKGGVEVLEVKTGVDAITEVECFLNPEMGDPD  60
BAF75276.1          MAPTKRKGECPGAAPKKPKEPVQVPKLLIKGGVEVLEVKTGVDAITEVECFLNPEMGDPD  60
BAF75330.1          MAPTKRKGECPGAAPKKPKEPVQVPKLLIKGGVEVLEVKTGVDAITEVECFLNPEMGDPD  60
BAF03115.1          MAPTKRKGECPGAAPKKPKEPVQVPKLLIKGGVEVLEVKTGVDAITEVECFLNPEMGDPD  60
BAF03120.1          MAPTKRKGECPGAAPKKPKEPVQVPKLLIKGGVEVLEVKTGVDAITEVECFLNPEMGDPD  60
WLD47842.1          MAPTKRKGECPGAAPKKPKEPVQVPKLLIKGGVEVLEVKTGVDAITEVECFLNPEMGDPD  60
WLD47884.1          MAPTKRKGECPGAAPKKPKEPVQVPKLLIKGGVEVLEVKTGVDAITEVECFLNPEMGDPD  60
UTM62101.1          MAPTKRKGECPGAAPKKPKEPVQVPKLLIKGGVEVLEVKTGVDAITEVECFLNPEMGDPD  60
UTM62107.1          MAPTKRKGECPGAAPKKPKEPVQVPKLLIKGGVEVLEVKTGVDAITEVECFLNPEMGDPD  60
UTM62113.1          MAPTKRKGECPGAAPKKPKEPVQVPKLLIKGGVEVLEVKTGVDAITEVECFLNPEMGDPD  60
AFQ31647.1          MAPTKRKGECPGAAPKKPKEPVQVPKLLIKGGVEVLEVKTGVDAITEVECFLNPEMGDPD  60
AFA41892.1          MAPTKRKGECPGAAPKKPKEPVQVPKLLIKGGVEVLEVKTGVDAITEVECFLNPEMGDPD  60
BAG75403.1          MAPTKRKGECPGAAPKKPKEPVQVPKLLIKGGVEVLEVKTGVDAITEVECFLNPEMGDPD  60
BAF75114.1          MAPTKRKGECPGAAPKKPKEPVQVPKLLIKGGVEVLEVKTGVDAITEVECFLNPEMGDPD  60
BAF75120.1          MAPTKRKGECPGAAPKKPKEPVQVPKLLIKGGVEVLEVKTGVDAITEVECFLNPEMGDPD  60
BAF75126.1          MAPTKRKGECPGAAPKKPKEPVQVPKLLIKGGVEVLEVKTGVDAITEVECFLNPEMGDPD  60
BAF75186.1          MAPTKRKGECPGAAPKKPKEPVQVPKLLIKGGVEVLEVKTGVDAITEVECFLNPEMGDPD  60
BAF75288.1          MAPTKRKGECPGAAPKKPKEPVQVPKLLIKGGVEVLEVKTGVDAITEVECFLNPEMGDPD  60
BAF75294.1          MAPTKRKGECPGAAPKKPKEPVQVPKLLIKGGVEVLEVKTGVDAITEVECFLNPEMGDPD  60
BAF75348.1          MAPTKRKGECPGAAPKKPKEPVQVPKLLIKGGVEVLEVKTGVDAITEVECFLNPEMGDPD  60
AFA41881.1          MAPTKRKGECPGAAPKKPKEPVQVPKLLIKGGVEVLEVKTGVDAITEVECFLNPEMGDPD  60
                    ***:::*********:**:: :*************** *** ****:********
```

```
ALF06344.1       ENLRGFSL--KLXAENDFXSDSPXXKMLPCYSTARIPLPNLNEDLTCGNLLMWEAVTVQT  118
QTJ15012.1       ENLRGFSL--KLSVGNNFSSDSPQRQMLPCYSTARIPLPNLNEDLTCGNLLMWEAVTVQT  118
QTJ15120.1       ENLRGFSL--KLSVGNNFSSDSPQRQMLPCYSTARIPLPNLNEDLTCGNLLMWEAVTVQT  118
QHI34657.1       ENLRGFSL--KLSVQNDFSSDSPQRKMLPCYSTARIPLPNLNEDLTCGNLLMWEAVTVQT  118
ARE72390.1       ENLRGFSL--KLTAETDFSSDSPDRKMLPCYSTARIPLPNLNEDLTCGNLLMWEAVTVKT  118
QTJ14970.1       ENLRGFSL--QLSAENDFSSDSPERKMLPCYSTARIPLPNLNEDLTCGNLLMWEAVTVQT  118
QTJ14928.1       ENLRGFSLSLKLSAKNDFNSDSPDRKMLPCYSTARIPLPNLNEDLTCGNLLMWEAVTVQT  120
AAT47419.1       ENLRGFSL--KLSAENDFSSDSPERKMLPCYSTARIPLPNLNEDLTCGNLLMWEAVTVQT  118
AAT47413.1       ENLRGFSL--KLSAENDFSSDSPERKMLPCYSTARIPLPNLNEDLTCGNLLMWEAVTVQT  118
AFQ31661.1       ENLRGFSL--KLSAENDFSSDSPERKMLPCYSTAGIPLPNLNEDLTCGNLLMWGAVTVQT  118
YP_717939.1      ENLRGFSL--KLSAENDFSSDSPERKMLPCYSTARIPLPNLNEDLTCGNLLMWEAVTVQT  118
AFK08545.1       ENLRGFSL--KLSAENDFSSDSPERKMLPCYSTARIPLPNLNEDLTCGNLLMWEAVTVQT  118
P03088.2         ENLRGFSL--KLSAENDFSSDSPERKMLPCYSTARIPLPNLNEDLTCGNLLMWEAVTVQT  118
CAA24299.1       ENLRGFSL--KLSAENDFSSDSPERKMLPCYSTARIPLPNLNEDLTCGNLLMWEAVTVQT  118
QTJ14922.1       ENLRGFSL--KLSAENDFSSDSPEKKMLPCYSTARIPLPNLNEDLTCGNLLMWEAVTVQT  118
AFA41877.1       ENLRGFSL--MLSAENDFNSDSPERKMLPCYSTARIPLPNLNEDLTCGNLLMWEAVTVQT  118
AAT47371.1       ENLRGFSL--KLSAENDFSSDSPERKMLPCYSTARIPLPNLNEDLTCGNLLMWEAVTVQT  118
AYO46658.1       ENLRGFSL--KLSAENDFSSDSPERKMLPCYSTARIPLPNLNEDLTCGNLLMWEAVTVQT  118
AYO46610.1       ENLRGFSV--KLSVQNDFSSDSPERKMLPCYSTARIPLPNLNEDLTCGNLLMWEAVTVQT  118
AYO46689.1       ENLRGFSV--KLSGQNDFSSDSPERKMLPCYSTARIPLPNLNEDLTCGNLLMWEAVTVQT  118
AYO46693.1       ENLRGFSL--KLSGEKDFSSDSPERKMLPCYSTARIPLPNLNEDLTCGNLLMWEAVTVQT  118
BAG75283.1       ENLRGFSL--KLSAENDFSSDSPERKMLPCYSTARIPLPNLNEDLTCGNLLMWEAVTVQT  118
BAG75313.1       ENLRGFSL--KLSAENDFSSDSPERKMLPCYSTARIPLPNLNEDLTCGNLLMWEAVTVQT  118
BAG75373.1       ENLRGFSL--KLSAENDFSSDSPERKMLPCYSTARIPLPNLNEDLTCGNLLMWEAVTVQT  118
BCV68914.1       ENLRGFSL--KLSAENDFSSDSPERKMLPCYSTARIPLPNLNEDLTCGNLLMWEAVTVQT  118
BCV68917.1       ENLRGFSL--KLSAENDFSSDSPERKMLPCYSTARIPLPNLNEDLTCGNLLMWEAVTVQT  118
BCV68923.1       ENLRGFSL--KLSAENDFSSDSPERKMLPCYSTARIPLPNLNEDLTCGNLLMWEAVTVQT  118
AYO46620.1       ENLRGFSL--KLSAENDFSSDSPERKMLPCYSTARIPLPNLNEDLTCGNLLMWEAVTVQT  118
AYO46646.1       ENLRGFSL--KLSAENDFSSDSPERKMLPCYSTARIPLPNLNEDLTCGNLLMWEAVTVQT  118
AYO46663.1       ENLRGFSL--KLSAENDFSSDSPERKMLPCYSTARIPLPNLNEDLTCGNLLMWEAVTVQT  118
AYO46694.1       ENLRGFSL--KLSAENDFSSDSPERKMLPCYSTARIPLPNLNEDLTCGNLLMWEAVTVQT  118
BAZ99906.1       ENLRGFSL--KLSAENDFSSDSPERKMLPCYSTARIPLPNLNEDLTCGNLLMWEAVTVQT  118
BAZ99912.1       ENLRGFSL--KLSAENDFSSDSPERKMLPCYSTARIPLPNLNEDLTCGNLLMWEAVTVQT  118
BAI43576.1       ENLRGFSL--KLSAENDFSSDSPERKMLPCYSTARIPLPNLNEDLTCGNLLMWEAVTVQT  118
BAI43594.1       ENLRGFSL--KLSAENDFSSDSPERKMLPCYSTARIPLPNLNEDLTCGNLLMWEAVTVQT  118
BAI43606.1       ENLRGFSL--KLSAENDFSSDSPERKMLPCYSTARIPLPNLNEDLTCGNLLMWEAVTVQT  118
BAI43624.1       ENLRGFSL--KLSAENDFSSDSPERKMLPCYSTARIPLPNLNEDLTCGNLLMWEAVTVQT  118
BAH23450.1       ENLRGFSL--KLSAENDFSSDSPERKMLPCYSTARIPLPNLNEDLTCGNLLMWEAVTVQT  118
BAH23456.1       ENLRGFSL--KLSAENDFSSDSPERKMLPCYSTARIPLPNLNEDLTCGNLLMWEAVTVQT  118
BAH23462.1       ENLRGFSL--KLTAENDFSSDSPERKMLPCYSTARIPLPNLNEDLTCGNLLMWEAVTVQT  118
BAH23468.1       ENLRGFSL--KLSAENDFSSDSPERKMLPCYSTARIPLPNLNEDLTCGNLLMWEAVTVQT  118
BAH23474.1       ENLRGFSL--KLSAENDFSSDSPERKMLPCYSTARIPLPNLNEDLTCGNLLMWEAVTVQT  118
BAH23480.1       ENLRGFSL--KLSAENDFSSDSPERKMLPCYSTARIPLPNLNEDLTCGNLLMWEAVTVQT  118
BAH23486.1       ENLRGFSL--KLSAENDFSSDSPERKMLPCYSTARIPLPNLNEDLTCGNLLMWEAVTVQT  118
BAH23492.1       ENLRGFSL--KLSAENDFSSDSPERKMLPCYSTARIPLPNLNEDLTCGNLLMWEAVTVQT  118
BAH23498.1       ENLRGFSL--KLSAENDFSSDSPERKMLPCYSTARIPLPNLNEDLTCGNLLMWEAVTVQT  118
BAH23504.1       ENLRGFSL--KLSAENDFSSDSPERKMLPCYSTARIPLPNLNEDLTCGNLLMWEAVTVQT  118
BAH23510.1       ENLRGFSL--KLSAENDFSSDSPERKMLPCYSTARIPLPNLNEDLTCGNLLMWEAVTVQT  118
BAH23516.1       ENLRGFSL--KLSAENDFSSDSPERKMLPCYSTARIPLPNLNEDLTCGNLLMWEAVTVQT  118
BAH23522.1       ENLRGFSL--KLSAENDFSSDSPERKMLPCYSTARIPLPNLNEDLTCGNLLMWEAVTVQT  118
BAH23528.1       ENLRGFSL--KLSAENDFSSDSPERKMLPCYSTARIPLPNLNEDLTCGNLLMWEAVTVQT  118
BAH23534.1       ENLRGFSL--KLSAENDFSSDSPERKMLPCYSTARIPLPNLNEDLTCGNLLMWEAVTVQT  118
BAH23540.1       ENLRGFSL--KLSAENDFSSDSPERKMLPCYSTARIPLPNLNEDLTCGNLLMWEAVTVQT  118
BAH23546.1       ENLRGFSL--KLSAENDFSSDSPERKMLPCYSTARIPLPNLNEDLTCGNLLMWEAVTVQT  118
BAH23552.1       ENLRGFSL--KLSAENDFSSDSPERKMLPCYSTARIPLPNLNEDLTCGNLLMWEAVTVQT  118
BAH23558.1       ENLRGFSL--KLSAENDFSSDSPERKMLPCYSTARIPLPNLNEDLTCGNLLMWEAVTVQT  118
BAG84488.1       ENLRGFSL--KLSAENDFSSDSPERKMLPCYSTARIPLPNLNEDLTCGNLLMWEAVTVQT  118
BAG84494.1       ENLRGFSL--KLSAENDFSSDSPERKMLPCYSTARIPLPNLNEDLTCGNLLMWEAVTVQT  118
BAG84500.1       ENLRGFSL--KLSAENDFSSDSPERKMLPCYSTARIPLPNLNEDLTCGNLLMWEAVTVQT  118
BAG84506.1       ENLRGFSL--KLSAENDFSSDSPERKMLPCYSTARIPLPNLNEDLTCGNLLMWEAVTVQT  118
BAG84512.1       ENLRGFSL--KLSAENDFSSDSPERKMLPCYSTARIPLPNLNEDLTCGNLLMWEAVTVQT  118
BAG84518.1       ENLRGFSL--KLSAENDFSSDSPERKMLPCYSTARIPLPNLNEDLTCGNLLMWEAVTVQT  118
BAG84524.1       ENLRGFSL--KLSAENDFSSDSPERKMLPCYSTARIPLPNLNEDLTCGNLLMWEAVTVQT  118
```

```
BAG75163.1        ENLRGFSL--KLSAENDFSSDSPERKMLPCYSTARIPLPNLNEDLTCGNLLMWEAVTVQT  118
BAG75169.1        ENLRGFSL--KLSAENDFSSDSPERKMLPCYSTARIPLPNLNEDLTCGNLLMWEAVTVQT  118
BAG75175.1        ENLRGFSL--KLSAENDFSSDSPERKMLPCYSTARIPLPNLNEDLTCGNLLMWEAVTVQT  118
BAG75181.1        ENLRGFSL--KLSAENDFSSDSPERKMLPCYSTARIPLPNLNEDLTCGNLLMWEAVTVQT  118
BAG75187.1        ENLRGFSL--KLSAENDFSSDSPERKMLPCYSTARIPLPNLNEDLTCGNLLMWEAVTVQT  118
BAG75193.1        ENLRGFSL--KLSAENDFSSDSPERKMLPCYSTARIPLPNLNEDLTCGNLLMWEAVTVQT  118
BAG75199.1        ENLRGFSL--KLSAENDFSSDSPERKMLPCYSTARIPLPNLNEDLTCGNLLMWEAVTVQT  118
BAG75241.1        ENLRGFSL--KLSAENDFSSDSPERKMLPCYSTARIPLPNLNEDLTCGNLLMWEAVTVQT  118
BAG75247.1        ENLRGFSL--KLSAENDFSSDSPERKMLPCYSTARIPLPNLNEDLTCGNLLMWEAVTVQT  118
BAG75253.1        ENLRGFSL--KLSAENDFSSDSPERKMLPCYSTARIPLPNLNEDLTCGNLLMWEAVTVQT  118
BAG75265.1        ENLRGFSL--KLSAENDFSSDSPERKMLPCYSTARIPLPNLNEDLTCGNLLMWEAVTVQT  118
BAG75289.1        ENLRGFSL--KLSAENDFSSDSPERKMLPCYSTARIPLPNLNEDLTCGNLLMWEAVTVQT  118
BAG75295.1        ENLRGFSL--KLSAENDFSSDSPERKMLPCYSTARIPLPNLNEDLTCGNLLMWEAVTVQT  118
BAG75307.1        ENLRGFSL--KLSAENDFSSDSPERKMLPCYSTARIPLPNLNEDLTCGNLLMWEAVTVQT  118
BAG75325.1        ENLRGFSL--KLSAENDFSSDSPERKMLPCYSTARIPLPNLNEDLTCGNLLMWEAVTVQT  118
BAG75331.1        ENLRGFSL--KLSAENDFSSDSPERKMLPCYSTARIPLPNLNEDLTCGNLLMWEAVTVQT  118
BAG75337.1        ENLRGFSL--KLSAENDFSSDSPERKMLPCYSTARIPLPNLNEDLTCGNLLMWEAVTVQT  118
BAG75343.1        ENLRGFSL--KLSAENDFSSDSPERKMLPCYSTARIPLPNLNEDLTCGNLLMWEAVTVQT  118
BAG75397.1        ENLRGFSL--KLSAENDFSSDSPERKMLPCYSTARIPLPNLNEDLTCGNLLMWEAVTVQT  118
BAG75421.1        ENLRGFSL--KLSAENDFSSDSPERKMLPCYSTARIPLPNLNEDLTCGNLLMWEAVTVQT  118
BAG75427.1        ENLRGFSL--KLSAENDFSSDSPERKMLPCYSTARIPLPNLNEDLTCGNLLMWEAVTVQT  118
BAG75433.1        ENLRGFSL--KLSAENDFSSDSPERKMLPCYSTARIPLPNLNEDLTCGNLLMWEAVTVQT  118
BAF93349.1        ENLRGFSL--KLSAENDFSSDSPERKMLPCYSTARIPLPNLNEDLTCGNLLMWEAVTVQT  118
BAF76166.1        ENLRGFSL--KLSAENDFSSDSPERKMLPCYSTARIPLPNLNEDLTCGNLLMWEAVTVQT  118
BAF76172.1        ENLRGFSL--KLSAENDFSSDSPERKMLPCYSTARIPLPNLNEDLTCGNLLMWEAVTVQT  118
BAF76178.1        ENLRGFSL--KLSAENDFSSDSPERKMLPCYSTARIPLPNLNEDLTCGNLLMWEAVTVQT  118
BAF76184.1        ENLRGFSL--KLSAENDFSSDSPERKMLPCYSTARIPLPNLNEDLTCGNLLMWEAVTVQT  118
BAF76190.1        ENLRGFSL--KLSAENDFSSDSPERKMLPCYSTARIPLPNLNEDLTCGNLLMWEAVTVQT  118
BAF76202.1        ENLRGFSL--KLSAENDFSSDSPERKMLPCYSTARIPLPNLNEDLTCGNLLMWEAVTVQT  118
BAF42985.1        ENLRGFSL--KLSAENDFSSDSPERKMLPCYSTARIPLPNLNEDLTCGNLLMWEAVTVQT  118
BAF42991.1        ENLRGFSL--KLSAENDFSSDSPERKMLPCYSTARIPLPNLNEDLTCGNLLMWEAVTVQT  118
BAF02951.1        ENLRGFSL--KLSAENDFSSDSPERKMLPCYSTARIPLPNLNEDLTCGNLLMWEAVTVQT  118
BAF02963.1        ENLRGFSL--KLSAENDFSSDSPERKMLPCYSTARIPLPNLNEDLTCGNLLMWEAVTVQT  118
BAF02969.1        ENLRGFSL--KLSAENDFSSDSPERKMLPCYSTARIPLPNLNEDLTCGNLLMWEAVTVQT  118
BAF02981.1        ENLRGFSL--KLSAENDFSSDSPERKMLPCYSTARIPLPNLNEDLTCGNLLMWEAVTVQT  118
BAF02987.1        ENLRGFSL--KLSAENDFSSDSPERKMLPCYSTARIPLPNLNEDLTCGNLLMWEAVTVQT  118
BAF02993.1        ENLRGFSL--KLSAENDFSSDSPERKMLPCYSTARIPLPNLNEDLTCGNLLMWEAVTVQT  118
BAF02999.1        ENLRGFSL--KLSAENDFSSDSPERKMLPCYSTARIPLPNLNEDLTCGNLLMWEAVTVQT  118
BAF03005.1        ENLRGFSL--KLSAENDFSSDSPERKMLPCYSTARIPLPNLNEDLTCGNLLMWEAVTVQT  118
BAF03011.1        ENLRGFSL--KLSAENDFSSDSPERKMLPCYSTARIPLPNLNEDLTCGNLLMWEAVTVQT  118
BAE96065.1        ENLRGFSL--KLSAENDFSSDSPERKMLPCYSTARIPLPNLNEDLTCGNLLMWEAVTVQT  118
BAE53666.1        ENLRGFSL--KLSAENDFSSDSPERKMLPCYSTARIPLPNLNEDLTCGNLLMWEAVTVQT  118
BAE46623.1        ENLRGFSL--KLSAENDFSSDSPERKMLPCYSTARIPLPNLNEDLTCGNLLMWEAVTVQT  118
CAA40235.1        ENLRGFSL--KLSAENDFSSDSPERKMLPCYSTARIPLPNLNEDLTCGNLLMWEAVTVQT  118
BAI43570.1        ENLRGFSL--KLSAENDFSSDSPERKMLPCYSTARIPLPNLNEDLTCGNLLMWEAVTVQT  118
BAI43582.1        ENLRGFSL--KLSAENDFSSDSPERKMLPCYSTARIPLPNLNEDLTCGNLLMWEAVTVQT  118
BAI43612.1        ENLRGFSL--KLSAENDFSSDSPERKMLPCYSTARIPLPNLNEDLTCGNLLMWEAVTVQT  118
BAI43618.1        ENLRGFSL--KLSAENDFSSDSPERKMLPCYSTARIPLPNLNEDLTCGNLLMWEAVTVQT  118
BAI43630.1        ENLRGFSL--KLSAENDFSSDSPERKMLPCYSTARIPLPNLNEDLTCGNLLMWEAVTVQT  118
BAG75301.1        ENLRGFSL--KLSAENDFSSDSPERKMLPCYSTARIPLPNLNEDLTCGNLLMWEAVTVQT  118
BAG75349.1        ENLRGFSL--KLSAENDFSSDSPERKMLPCYSTARIPLPNLNEDLTCGNLLMWEAVTVQT  118
BAG75367.1        ENLRGFSL--KLSAENDFSSDSPERKMLPCYSTARIPLPNLNEDLTCGNLLMWEAVTVQT  118
BAF76196.1        ENLRGFSL--KLSAENDFSSDSPERKMLPCYSTARIPLPNLNEDLTCGNLLMWEAVTVQT  118
BAF02975.1        ENLRGFSL--KLSVENDFSSDSPQRKMLPCYSTARIPLPNLNEDLTCGNLLMWEAVTVQT  118
BAI43600.1        ENLRGFSL--KLSAENDFSSDSPDKKMLPCYSTARIPLPNLNEDLTCGNLLMWEAVTVQT  118
BAE96071.1        ENLRGFSL--KLSAENDFSSDSPDKKMLPCYSTARIPLPNLNEDLTCGNLLMWEAVTVQT  118
BAE53660.1        ENLRGFSL--KLSAENDFSSDSPDKKMLPCYSTARIPLPNLNEDLTCGNLLMWEAVTVQT  118
BCV68926.1        ENLRGFSL--KLSAENDFSSDSPERKMLPCYSTARIPLPNLNEDLTCGNLLMWEAVTVQT  118
AYO46647.1        ENLRGFSL--KLSAENDFSSDSPERKMLPCYSTARIPLPNLNEDLTCGNLLMWEAVTVQT  118
BAR80134.1        ENLRGFSL--KLSAENNFSSDSPERKMLPCYSTARIPLPNLNEDLTCGNLLMWEAVTVQT  118
BAR80140.1        ENLRGFSL--KLSAENNFSSDSPERKMLPCYSTARIPLPNLNEDLTCGNLLMWEAVTVQT  118
BAI43588.1        ENLRGFSL--KLSAENDFSSDSPERKMLPCYSTARIPLPNLNEDLTCGNLLMWEAVTVQT  118
BAG75361.1        ENLRGFSL--KLSAENDFSSDSPDRKMLPCYSTARIPLPNLNEDLTCGNLLMWEAVTVQT  118
BAE53642.1        ENLRGFSL--KLSAENDFSSDSPQRKMLPCYSTARIPLPNLNEDLTCGNLLMWEAVTVQT  118
```

BAE53648.1	KNLRGFSL--KLSAENDFSSDSPERKMLPCYSTARIPLPNLNEDLTCGNLLMWEAVTVQT	118
AFA41903.1	ENLRGFSL--KLSAENDFSSDSPERKMLPCYSTARIPLPNLNEDLTCGNLLMWEAVTVQT	118
AFA41918.1	ENLRGFSL--KLSAENDFSSDSPERKMLPCYSTARIPLPNLNEDLTCGNLLMWEAVTVQT	118
BAF42937.1	ENLRGFSL--KLSAENDFSSDSPERKMLPCYSTARIPLPNLNEDLTCGNLLMWEAVTVQT	118
BAF42997.1	ENLRGFSL--KLSAENDFSSDSPERKMLPCYSTARIPLPNLNEDLTCGNLLMWEAVTVQT	118
WLD47902.1	ENLRGFSL--KLSAENDFSSDSPERKMLPCYSTARIPLPNLNEDLTCGNLLMWEAVTVQT	118
WLD47938.1	ENLRGFSL--KLSAENDFSSDSPERKMLPCYSTARIPLPNLNEDLTCGNLLMWEAVTVQT	118
WLD47980.1	ENLRGFSL--KLSAENDFSSDSPERKMLPCYSTARIPLPNLNEDLTCGNLLMWEAVTVQT	118
UTM61891.1	ENLRGFSL--KLSAENDFSSDSPERKMLPCYSTARIPLPNLNEDLTCGNLLMWEAVTVQT	118
UTM61897.1	ENLRGFSL--KLSAENDFSSDSPERKMLPCYSTARIPLPNLNEDLTCGNLLMWEAVTVQT	118
UTM61903.1	ENLRGFSL--KLSAENDFSSDSPERKMLPCYSTARIPLPNLNEDLTCGNLLMWEAVTVQT	118
UTM62215.1	ENLRGFSL--KLSAENDFSSDSPERKMLPCYSTARIPLPNLNEDLTCGNLLMWEAVTVQT	118
UTM62221.1	ENLRGFSL--KLSAENDFSSDSPERKMLPCYSTARIPLPNLNEDLTCGNLLMWEAVTVQT	118
UTM62275.1	ENLRGFSL--KLSAENDFSSDSPERKMLPCYSTARIPLPNLNEDLTCGNLLMWEAVTVQT	118
UTM62281.1	ENLRGFSL--KLSAENDFSSDSPERKMLPCYSTARIPLPNLNEDLTCGNLLMWEAVTVQT	118
UTM62329.1	ENLRGFSL--KLSAENDFSSDSPERKMLPCYSTARIPLPNLNEDLTCGNLLMWEAVTVQT	118
UTM62335.1	ENLRGFSL--KLSAENDFSSDSPERKMLPCYSTARIPLPNLNEDLTCGNLLMWEAVTVQT	118
UTM62383.1	ENLRGFSL--KLSAENDFSSDSPERKMLPCYSTARIPLPNLNEDLTCGNLLMWEAVTVQT	118
UTM62395.1	ENLRGFSL--KLSAENDFSSDSPERKMLPCYSTARIPLPNLNEDLTCGNLLMWEAVTVQT	118
UTM62443.1	ENLRGFSL--KLSAENDFSSDSPERKMLPCYSTARIPLPNLNEDLTCGNLLMWEAVTVQT	118
UTM62449.1	ENLRGFSL--KLSAENDFSSDSPERKMLPCYSTARIPLPNLNEDLTCGNLLMWEAVTVQT	118
UTM62467.1	ENLRGFSL--KLSAENDFSSDSPERKMLPCYSTARIPLPNLNEDLTCGNLLMWEAVTVQT	118
UTM62545.1	ENLRGFSL--KLSAENDFSSDSPERKMLPCYSTARIPLPNLNEDLTCGNLLMWEAVTVQT	118
UTM62551.1	ENLRGFSL--KLSAENDFSSDSPERKMLPCYSTARIPLPNLNEDLTCGNLLMWEAVTVQT	118
UTM62587.1	ENLRGFSL--KLSAENDFSSDSPERKMLPCYSTARIPLPNLNEDLTCGNLLMWEAVTVQT	118
UTM62593.1	ENLRGFSL--KLSAENDFSSDSPERKMLPCYSTARIPLPNLNEDLTCGNLLMWEAVTVQT	118
UTM62599.1	ENLRGFSL--KLSAENDFSSDSPERKMLPCYSTARIPLPNLNEDLTCGNLLMWEAVTVQT	118
QUW04850.1	ENLRGFSL--KLSAENDFSSDSPERKMLPCYSTARIPLPNLNEDLTCGNLLMWEAVTVQT	118
QTJ14976.1	ENLRGFSL--KLSAENDFSSDSPERKMLPCYSTARIPLPNLNEDLTCGNLLMWEAVTVQT	118
QTJ15000.1	ENLRGFSL--KLSAENDFSSDSPERKMLPCYSTARIPLPNLNEDLTCGNLLMWEAVTVQT	118
QTJ15054.1	ENLRGFSL--KLSAENDFSSDSPERKMLPCYSTARIPLPNLNEDLTCGNLLMWEAVTVQT	118
QTJ15072.1	ENLRGFSL--KLSAENDFSSDSPERKMLPCYSTARIPLPNLNEDLTCGNLLMWEAVTVQT	118
QTJ15084.1	ENLRGFSL--KLSAENDFSSDSPERKMLPCYSTARIPLPNLNEDLTCGNLLMWEAVTVQT	118
QTJ15090.1	ENLRGFSL--KLSAENDFSSDSPERKMLPCYSTARIPLPNLNEDLTCGNLLMWEAVTVQT	118
QTJ15108.1	ENLRGFSL--KLSAENDFSSDSPERKMLPCYSTARIPLPNLNEDLTCGNLLMWEAVTVQT	118
QTJ15114.1	ENLRGFSL--KLSAENDFSSDSPERKMLPCYSTARIPLPNLNEDLTCGNLLMWEAVTVQT	118
QTJ15132.1	ENLRGFSL--KLSAENDFSSDSPERKMLPCYSTARIPLPNLNEDLTCGNLLMWEAVTVQT	118
QTJ15138.1	ENLRGFSL--KLSAENDFSSDSPERKMLPCYSTARIPLPNLNEDLTCGNLLMWEAVTVQT	118
AYO46612.1	ENLRGFSL--KLSAENDFSSDSPERKMLPCYSTARIPLPNLNEDLTCGNLLMWEAVTVQT	118
AYO46613.1	ENLRGFSL--KLSAENDFSSDSPERKMLPCYSTARIPLPNLNEDLTCGNLLMWEAVTVQT	118
AYO46617.1	ENLRGFSL--KLSAENDFSSDSPERKMLPCYSTARIPLPNLNEDLTCGNLLMWEAVTVQT	118
AYO46621.1	ENLRGFSL--KLSAENDFSSDSPERKMLPCYSTARIPLPNLNEDLTCGNLLMWEAVTVQT	118
AYO46622.1	ENLRGFSL--KLSAENDFSSDSPERKMLPCYSTARIPLPNLNEDLTCGNLLMWEAVTVQT	118
AYO46623.1	ENLRGFSL--KLSAENDFSSDSPERKMLPCYSTARIPLPNLNEDLTCGNLLMWEAVTVQT	118
AYO46624.1	ENLRGFSL--KLSAENDFSSDSPERKMLPCYSTARIPLPNLNEDLTCGNLLMWEAVTVQT	118
AYO46629.1	ENLRGFSL--KLSAENDFSSDSPERKMLPCYSTARIPLPNLNEDLTCGNLLMWEAVTVQT	118
AYO46632.1	ENLRGFSL--KLSAENDFSSDSPERKMLPCYSTARIPLPNLNEDLTCGNLLMWEAVTVQT	118
AYO46635.1	ENLRGFSL--KLSAENDFSSDSPERKMLPCYSTARIPLPNLNEDLTCGNLLMWEAVTVQT	118
AYO46638.1	ENLRGFSL--KLSAENDFSSDSPERKMLPCYSTARIPLPNLNEDLTCGNLLMWEAVTVQT	118
AYO46641.1	ENLRGFSL--KLSAENDFSSDSPERKMLPCYSTARIPLPNLNEDLTCGNLLMWEAVTVQT	118
AYO46642.1	ENLRGFSL--KLSAENDFSSDSPERKMLPCYSTARIPLPNLNEDLTCGNLLMWEAVTVQT	118
AYO46644.1	ENLRGFSL--KLSAENDFSSDSPERKMLPCYSTARIPLPNLNEDLTCGNLLMWEAVTVQT	118
AYO46651.1	ENLRGFSL--KLSAENDFSSDSPERKMLPCYSTARIPLPNLNEDLTCGNLLMWEAVTVQT	118
AYO46652.1	ENLRGFSL--KLSAENDFSSDSPERKMLPCYSTARIPLPNLNEDLTCGNLLMWEAVTVQT	118
AYO46659.1	ENLRGFSL--KLSAENDFSSDSPERKMLPCYSTARIPLPNLNEDLTCGNLLMWEAVTVQT	118
AYO46660.1	ENLRGFSL--KLSAENDFSSDSPERKMLPCYSTARIPLPNLNEDLTCGNLLMWEAVTVQT	118
AYO46662.1	ENLRGFSL--KLSAENDFSSDSPERKMLPCYSTARIPLPNLNEDLTCGNLLMWEAVTVQT	118
AYO46665.1	ENLRGFSL--KLSAENDFSSDSPERKMLPCYSTARIPLPNLNEDLTCGNLLMWEAVTVQT	118
AYO46670.1	ENLRGFSL--KLSAENDFSSDSPERKMLPCYSTARIPLPNLNEDLTCGNLLMWEAVTVQT	118
AYO46672.1	ENLRGFSL--KLSAENDFSSDSPERKMLPCYSTARIPLPNLNEDLTCGNLLMWEAVTVQT	118
AYO46676.1	ENLRGFSL--KLSAENDFSSDSPERKMLPCYSTARIPLPNLNEDLTCGNLLMWEAVTVQT	118
AYO46677.1	ENLRGFSL--KLSAENDFSSDSPERKMLPCYSTARIPLPNLNEDLTCGNLLMWEAVTVQT	118
AYO46679.1	ENLRGFSL--KLSAENDFSSDSPERKMLPCYSTARIPLPNLNEDLTCGNLLMWEAVTVQT	118
AYO46682.1	ENLRGFSL--KLSAENDFSSDSPERKMLPCYSTARIPLPNLNEDLTCGNLLMWEAVTVQT	118

AYO46691.1	ENLRGFSL--KLSAENDFSSDSPERKMLPCYSTARIPLPNLNEDLTCGNLLMWEAVTVQT	118
AYO46692.1	ENLRGFSL--KLSAENDFSSDSPERKMLPCYSTARIPLPNLNEDLTCGNLLMWEAVTVQT	118
AUF70040.1	ENLRGFSL--KLSAENDFSSDSPERKMLPCYSTARIPLPNLNEDLTCGNLLMWEAVTVQT	118
SOH95722.1	ENLRGFSL--KLSAENDFSSDSPERKMLPCYSTARIPLPNLNEDLTCGNLLMWEAVTVQT	118
SOI09711.1	ENLRGFSL--KLSAENDFSSDSPERKMLPCYSTARIPLPNLNEDLTCGNLLMWEAVTVQT	118
APG38208.1	ENLRGFSL--KLSAENDFSSDSPERKMLPCYSTARIPLPNLNEDLTCGNLLMWEAVTVQT	118
ALF06332.1	ENLRGFSL--KLSAENDFSSDSPERKMLPCYSTARIPLPNLNEDLTCGNLLMWEAVTVQT	118
ALF06341.1	ENLRGFSL--KLSAENDFSSDSPERKMLPCYSTARIPLPNLNEDLTCGNLLMWEAVTVQT	118
BAR80122.1	ENLRGFSL--KLSAENDFSSDSPERKMLPCYSTARIPLPNLNEDLTCGNLLMWEAVTVQT	118
BAR80128.1	ENLRGFSL--KLSAENDFSSDSPERKMLPCYSTARIPLPNLNEDLTCGNLLMWEAVTVQT	118
AJG42164.1	ENLRGFSL--KLSAENDFSSDSPERKMLPCYSTARIPLPNLNEDLTCGNLLMWEAVTVQT	118
AHU87340.1	ENLRGFSL--KLSAENDFSSDSPERKMLPCYSTARIPLPNLNEDLTCGNLLMWEAVTVQT	118
AHU87341.1	ENLRGFSL--KLSAENDFSSDSPERKMLPCYSTARIPLPNLNEDLTCGNLLMWEAVTVQT	118
AHU87368.1	ENLRGFSL--KLSAENDFSSDSPERKMLPCYSTARIPLPNLNEDLTCGNLLMWEAVTVQT	118
AHU87369.1	ENLRGFSL--KLSAENDFSSDSPERKMLPCYSTARIPLPNLNEDLTCGNLLMWEAVTVQT	118
AFQ31636.1	ENLRGFSL--KLSAENDFSSDSPERKMLPCYSTARIPLPNLNEDLTCGNLLMWEAVTVQT	118
AFQ31637.1	ENLRGFSL--KLSAENDFSSDSPERKMLPCYSTARIPLPNLNEDLTCGNLLMWEAVTVQT	118
AFQ31639.1	ENLRGFSL--KLSAENDFSSDSPERKMLPCYSTARIPLPNLNEDLTCGNLLMWEAVTVQT	118
AFQ31640.1	ENLRGFSL--KLSAENDFSSDSPERKMLPCYSTARIPLPNLNEDLTCGNLLMWEAVTVQT	118
AFQ31641.1	ENLRGFSL--KLSAENDFSSDSPERKMLPCYSTARIPLPNLNEDLTCGNLLMWEAVTVQT	118
AFQ31644.1	ENLRGFSL--KLSAENDFSSDSPERKMLPCYSTARIPLPNLNEDLTCGNLLMWEAVTVQT	118
AFQ31648.1	ENLRGFSL--KLSAENDFSSDSPERKMLPCYSTARIPLPNLNEDLTCGNLLMWEAVTVQT	118
AFQ31657.1	ENLRGFSL--KLSAENDFSSDSPERKMLPCYSTARIPLPNLNEDLTCGNLLMWEAVTVQT	118
AFA41882.1	ENLRGFSL--KLSAENDFSSDSPERKMLPCYSTARIPLPNLNEDLTCGNLLMWEAVTVQT	118
AFA41887.1	ENLRGFSL--KLSAENDFSSDSPERKMLPCYSTARIPLPNLNEDLTCGNLLMWEAVTVQT	118
AFA41890.1	ENLRGFSL--KLSAENDFSSDSPERKMLPCYSTARIPLPNLNEDLTCGNLLMWEAVTVQT	118
AFA41891.1	ENLRGFSL--KLSAENDFSSDSPERKMLPCYSTARIPLPNLNEDLTCGNLLMWEAVTVQT	118
AFA41895.1	ENLRGFSL--KLSAENDFSSDSPERKMLPCYSTARIPLPNLNEDLTCGNLLMWEAVTVQT	118
AFA41898.1	ENLRGFSL--KLSAENDFSSDSPERKMLPCYSTARIPLPNLNEDLTCGNLLMWEAVTVQT	118
AFA41901.1	ENLRGFSL--KLSAENDFSSDSPERKMLPCYSTARIPLPNLNEDLTCGNLLMWEAVTVQT	118
AFA41902.1	ENLRGFSL--KLSAENDFSSDSPERKMLPCYSTARIPLPNLNEDLTCGNLLMWEAVTVQT	118
AFA41910.1	ENLRGFSL--KLSAENDFSSDSPERKMLPCYSTARIPLPNLNEDLTCGNLLMWEAVTVQT	118
AFA41914.1	ENLRGFSL--KLSAENDFSSDSPERKMLPCYSTARIPLPNLNEDLTCGNLLMWEAVTVQT	118
AFA41917.1	ENLRGFSL--KLSAENDFSSDSPERKMLPCYSTARIPLPNLNEDLTCGNLLMWEAVTVQT	118
AEO89562.1	ENLRGFSL--KLSAENDFSSDSPERKMLPCYSTARIPLPNLNEDLTCGNLLMWEAVTVQT	118
AEO89568.1	ENLRGFSL--KLSAENDFSSDSPERKMLPCYSTARIPLPNLNEDLTCGNLLMWEAVTVQT	118
AEO89586.1	ENLRGFSL--KLSAENDFSSDSPERKMLPCYSTARIPLPNLNEDLTCGNLLMWEAVTVQT	118
AEO89598.1	ENLRGFSL--KLSAENDFSSDSPERKMLPCYSTARIPLPNLNEDLTCGNLLMWEAVTVQT	118
AEO89621.1	ENLRGFSL--KLSAENDFSSDSPERKMLPCYSTARIPLPNLNEDLTCGNLLMWEAVTVQT	118
AEK21505.1	ENLRGFSL--KLSAENDFSSDSPERKMLPCYSTARIPLPNLNEDLTCGNLLMWEAVTVQT	118
BAG84458.1	ENLRGFSL--KLSAENDFSSDSPERKMLPCYSTARIPLPNLNEDLTCGNLLMWEAVTVQT	118
BAG84470.1	ENLRGFSL--KLSAENDFSSDSPERKMLPCYSTARIPLPNLNEDLTCGNLLMWEAVTVQT	118
BAG84482.1	ENLRGFSL--KLSAENDFSSDSPERKMLPCYSTARIPLPNLNEDLTCGNLLMWEAVTVQT	118
BAG75319.1	ENLRGFSL--KLSAENDFSSDSPERKMLPCYSTARIPLPNLNEDLTCGNLLMWEAVTVQT	118
BAG75355.1	ENLRGFSL--KLSAENDFSSDSPERKMLPCYSTARIPLPNLNEDLTCGNLLMWEAVTVQT	118
BAG75391.1	ENLRGFSL--KLSAENDFSSDSPERKMLPCYSTARIPLPNLNEDLTCGNLLMWEAVTVQT	118
BAG75439.1	ENLRGFSL--KLSAENDFSSDSPERKMLPCYSTARIPLPNLNEDLTCGNLLMWEAVTVQT	118
BAF93301.1	ENLRGFSL--KLSAENDFSSDSPERKMLPCYSTARIPLPNLNEDLTCGNLLMWEAVTVQT	118
BAF93331.1	ENLRGFSL--KLSAENDFSSDSPERKMLPCYSTARIPLPNLNEDLTCGNLLMWEAVTVQT	118
BAF93355.1	ENLRGFSL--KLSAENDFSSDSPERKMLPCYSTARIPLPNLNEDLTCGNLLMWEAVTVQT	118
BAF76208.1	ENLRGFSL--KLSAENDFSSDSPERKMLPCYSTARIPLPNLNEDLTCGNLLMWEAVTVQT	118
BAF42877.1	ENLRGFSL--KLSAENDFSSDSPERKMLPCYSTARIPLPNLNEDLTCGNLLMWEAVTVQT	118
BAF42883.1	ENLRGFSL--KLSAENDFSSDSPERKMLPCYSTARIPLPNLNEDLTCGNLLMWEAVTVQT	118
BAF42889.1	ENLRGFSL--KLSAENDFSSDSPERKMLPCYSTARIPLPNLNEDLTCGNLLMWEAVTVQT	118
BAF42961.1	ENLRGFSL--KLSAENDFSSDSPERKMLPCYSTARIPLPNLNEDLTCGNLLMWEAVTVQT	118
BAF42967.1	ENLRGFSL--KLSAENDFSSDSPERKMLPCYSTARIPLPNLNEDLTCGNLLMWEAVTVQT	118
BAF42973.1	ENLRGFSL--KLSAENDFSSDSPERKMLPCYSTARIPLPNLNEDLTCGNLLMWEAVTVQT	118
BAF43003.1	ENLRGFSL--KLSAENDFSSDSPERKMLPCYSTARIPLPNLNEDLTCGNLLMWEAVTVQT	118
BAF43027.1	ENLRGFSL--KLSAENDFSSDSPERKMLPCYSTARIPLPNLNEDLTCGNLLMWEAVTVQT	118
BAF43033.1	ENLRGFSL--KLSAENDFSSDSPERKMLPCYSTARIPLPNLNEDLTCGNLLMWEAVTVQT	118
ABI94659.1	ENLRGFSL--KLSAENDFSSDSPERKMLPCYSTARIPLPNLNEDLTCGNLLMWEAVTVQT	118
ABI94683.1	ENLRGFSL--KLSAENDFSSDSPERKMLPCYSTARIPLPNLNEDLTCGNLLMWEAVTVQT	118
ABI94719.1	ENLRGFSL--KLSAENDFSSDSPERKMLPCYSTARIPLPNLNEDLTCGNLLMWEAVTVQT	118
BAE96053.1	ENLRGFSL--KLSAENDFSSDSPERKMLPCYSTARIPLPNLNEDLTCGNLLMWEAVTVQT	118

```
BAE96059.1        ENLRGFSL--KLSAENDFSSDSPERKMLPCYSTARIPLPNLNEDLTCGNLLMWEAVTVQT  118
ABC18005.1        ENLRGFSL--KLSAENDFSSDSPERKMLPCYSTARIPLPNLNEDLTCGNLLMWEAVTVQT  118
AAT47377.1        ENLRGFSL--KLSAENDFSSDSPERKMLPCYSTARIPLPNLNEDLTCGNLLMWEAVTVQT  118
AAT47383.1        ENLRGFSL--KLSAENDFSSDSPERKMLPCYSTARIPLPNLNEDLTCGNLLMWEAVTVQT  118
AAT47405.1        ENLRGFSL--KLSAENDFSSDSPERKMLPCYSTARIPLPNLNEDLTCGNLLMWEAVTVQT  118
CAA40239.1        ENLRGFSL--KLSAENDFSSDSPERKMLPCYSTARIPLPNLNEDLTCGNLLMWEAVTVQT  118
QTJ14940.1        ENLRGFSL--KLSAENDFSSDSPERKMLPCYSTARIPLPNLNEDLTCGNLLMWEAVTVQT  118
ALF06317.1        ENLRGFSL--KLSAENDFSSDSPEREMLPCYSTARIPLPNLNEDLTCGNLLMWEAVTVQT  118
ALF06307.1        ENLRGFSL--KLSAENDFSSDSPERKMLPCYSTARIPHPNLNEDLTCGNLLMWEAVTVQT  118
ALF06297.1        ENLRGFSL--KLSAENDFSSDSPERKMLPCYSTARIPLPNLNEDLTCGNLLMWEAVTVQT  118
ALF06350.1        ENLRGFSL--KLSAENDFSSDSPERKMLPCYSTARIPLPNLNEDLTCGNLLMWEAVTVQT  118
AAT47431.1        ENLRGFSL--KLSAQNDFSSDSPERKMLPCYSTARIPLPNLNEDLTCGNLLMWEAVTVQT  118
QTJ15006.1        ENLRGFSL--KLSAQNDFSSDSPERKMLPCYSTARIPLPNLNEDLTCGNLLMWEAVTVQT  118
AYO46667.1        ENLRGFSL--KLSAQNDFSSDSPERKMLPCYSTARIPLPNLNEDLTCGNLLMWEAVTVQT  118
WLD47956.1        ENLRGFSL--KLSAKNDFSSDSPERKMLPCYSTARIPLPNLNEDLTCGNLLMWEAVTVQT  118
QTJ15150.1        ENLRGFSL--KLSAKNDFSSDSPERKMLPCYSTARIPLPNLNEDLTCGNLLMWEAVTVQT  118
UTM62389.1        ENLRGFSL--KLSAKNDFSSDSPERKMLPCYSTARIPLPNLNEDLTCGNLLMWEAVTVQT  118
ABI94701.1        ENLRGFSL--KLSAKNDFSSDSPERKMLPCYSTARIPLPNLNEDLTCGNLLMWEAVTVQT  118
BAF02945.1        ENLRGFSL--KLSAKNDFSSDSPERKMLPCYSTARIPLPNLNEDLTCGNLLMWEAVTVQT  118
CAA40243.1        ENLRGFSL--KLSAKNDFSSDSPERKMLPCYSTARIPLPNLNEDLTCGNLLMWEAVTVQT  118
ABI94725.1        ENLRGFSL--KLSAEYDFSSDSPERKMLPCYSTARIPLPNLYEDLTCGNLLMWEAVTVQT  118
AFQ31660.1        ENLRGFSL--KLSAENDFSSDSPERKMLPCYSTARIPLPNLNEDLTCGNLLMWEAVTVQT  118
ALF06353.1        ENLRGFSL--KLSAENDFSSDSPERKMLPCYSTARIPLPNLNEDLTCGNLLMWEAVTVQT  118
ALF06312.1        ENLRGFSL--KLSAENDFSSDSPERKMLPCYSTARIPLPNLNEDLTCGNLLMWEAVTVQT  118
QEJ80721.1        ENLRGFSL--KLSAENDFSSDSPERKMLPCYSTARIPLPNLNEDLTCGNLLMWEAVTVQT  118
AYO46614.1        ENLRGFSL--KLSAENDFSSDSPERKMLPCYSTARIPLPNLNEDLTCGNLLMWEAVTVQT  118
AYO46619.1        ENLRGFSL--KLSAENDFSSDSPERKMLPCYSTARIPLPNLNEDLTCGNLLMWEAVTVQT  118
AYO46625.1        ENLRGFSL--KLSAENDFSSDSPERKMLPCYSTARIPLPNLNEDLTCGNLLMWEAVTVQT  118
AYO46626.1        ENLRGFSL--KLSAENDFSSDSPERKMLPCYSTARIPLPNLNEDLTCGNLLMWEAVTVQT  118
AYO46628.1        ENLRGFSL--KLSAENDFSSDSPERKMLPCYSTARIPLPNLNEDLTCGNLLMWEAVTVQT  118
AYO46633.1        ENLRGFSL--KLSAENDFSSDSPERKMLPCYSTARIPLPNLNEDLTCGNLLMWEAVTVQT  118
AYO46636.1        ENLRGFSL--KLSAENDFSSDSPERKMLPCYSTARIPLPNLNEDLTCGNLLMWEAVTVQT  118
AYO46637.1        ENLRGFSL--KLSAENDFSSDSPERKMLPCYSTARIPLPNLNEDLTCGNLLMWEAVTVQT  118
AYO46640.1        ENLRGFSL--KLSAENDFSSDSPERKMLPCYSTARIPLPNLNEDLTCGNLLMWEAVTVQT  118
AYO46648.1        ENLRGFSL--KLSAENDFSSDSPERKMLPCYSTARIPLPNLNEDLTCGNLLMWEAVTVQT  118
AYO46650.1        ENLRGFSL--KLSAENDFSSDSPERKMLPCYSTARIPLPNLNEDLTCGNLLMWEAVTVQT  118
AYO46653.1        ENLRGFSL--KLSAENDFSSDSPERKMLPCYSTARIPLPNLNEDLTCGNLLMWEAVTVQT  118
AYO46654.1        ENLRGFSL--KLSAENDFSSDSPERKMLPCYSTARIPLPNLNEDLTCGNLLMWEAVTVQT  118
AYO46671.1        ENLRGFSL--KLSAENDFSSDSPERKMLPCYSTARIPLPNLNEDLTCGNLLMWEAVTVQT  118
AYO46673.1        ENLRGFSL--KLSAENDFSSDSPERKMLPCYSTARIPLPNLNEDLTCGNLLMWEAVTVQT  118
AYO46674.1        ENLRGFSL--KLSAENDFSSDSPERKMLPCYSTARIPLPNLNEDLTCGNLLMWEAVTVQT  118
AYO46675.1        ENLRGFSL--KLSAENDFSSDSPERKMLPCYSTARIPLPNLNEDLTCGNLLMWEAVTVQT  118
AYO46680.1        ENLRGFSL--KLSAENDFSSDSPERKMLPCYSTARIPLPNLNEDLTCGNLLMWEAVTVQT  118
AYO46681.1        ENLRGFSL--KLSAENDFSSDSPERKMLPCYSTARIPLPNLNEDLTCGNLLMWEAVTVQT  118
AYO46684.1        ENLRGFSL--KLSAENDFSSDSPERKMLPCYSTARIPLPNLNEDLTCGNLLMWEAVTVQT  118
AYO46687.1        ENLRGFSL--KLSAENDFSSDSPERKMLPCYSTARIPLPNLNEDLTCGNLLMWEAVTVQT  118
AEO89574.1        ENLRGFSL--KLSAENDFSSDSPERKMLPCYSTARIPLPNLNEDLTCGNLLMWEAVTVQT  118
BAF42979.1        ENLRGFSL--KLSAENDFSSDSPERKMLPCYSTARIPLPNLNEDLTCGNLLMWEAVTVQT  118
BAF43009.1        ENLRGFSL--KLSAENDFSSDSPERKMLPCYSTARIPLPNLNEDLTCGNLLMWEAVTVQT  118
AYO46678.1        ENLRGFSL--KLSAENDFSSDSPERKMLPCYSTARIPLPNLNEDLTCGNLLMWEAVTVQT  118
UTM62479.1        ENLRGFSL--KLSAENDFSSDSPERKMLPCYSTARIPLPNLNEDLTCGNLLMWEAVTVQT  118
QTJ14964.1        ENLRGFSL--KLSAENDFSSDSPERKMLPCYSTARIPLPNLNEDLTCGNLLMWEAVTVQT  118
QTJ15036.1        ENLRGFSL--KLSAENDFSSDSPERKMLPCYSTARIPLPNLNEDLTCGNLLMWEAVTVQT  118
QTJ15066.1        ENLRGFSL--KLSAENDFSSDSPERKMLPCYSTARIPLPNLNEDLTCGNLLMWEAVTVQT  118
QTJ15078.1        ENLRGFSL--KLSAENDFSSDSPERKMLPCYSTARIPLPNLNEDLTCGNLLMWEAVTVQT  118
QTJ15096.1        ENLRGFSL--KLSAENDFSSDSPERKMLPCYSTARIPLPNLNEDLTCGNLLMWEAVTVQT  118
AQM40276.1        ENLRGFSL--KLSAENDFSSDSPERKMLPCYSTARIPLPNLNEDLTCGNLLMWEAVTVQT  118
ABI94689.1        ENLRGFSL--KLSAENDFSSDSPERKMLPCYSTARIPLPNLNEDLTCGNLLMWEAVTVQT  118
ABI94671.1        ENLRGFSL--KLSAENDFSSDSPERKMLPCYSTARIPLPNLNEDLTCGNLLMWEAVTVQT  118
AYO46688.1        ENLRGFSL--KLSAENDFSSDSPKRKMLPCYSTARIPLPNLNEDLTCGNLLMWEAVTVQT  118
QTJ15060.1        ENLRGFSL--KLSAENDFSSDSPQRKMLPCYSTARIPLPNLNEDLTCGNLLMWEAVTVQT  118
AFQ31642.1        ENLRGFSL--KLSAENDFSSDSPQRKMLPCYSTARIPLPNLNEDLTCGNLLMWEAVTVQT  118
AYO46616.1        ENLRGFSL--KLSAENDFSSDSPQRKMLPCYSTARIPLPNLNEDLTCGNLLMWEAVTVQT  118
CAA79594.1        ENLRGFSL--KLSAENDFSSDSPERKMLPCYSTARIPLPNLNEDLTCGNLLMWEAVTVQT  118
```

WLD47818.1	ENLRGFSL--KLSAENDFSSDSPERKMLPCYSTARIPLPNLNEDLTCGNLLMWEAVTVQT	118
UTM62155.1	ENLRGFSL--KLSAENDFSSDSPERKMLPCYSTARIPLPNLNEDLTCGNLLMWEAVTVQT	118
AFA41907.1	ENLRGFSL--KLSAENDFSSDSPERKMLPCYSTARIPLPNLNEDLTCGNLLMWEAVTVQT	118
WLD47944.1	ENLRGFSL--KLSAENNFSSDSPERKMLPCYSTARIPLPNLNEDLTCGNLLMWEAVTVQT	118
AYO46668.1	ENLRGFSL--KLSAENNFSSDSPERKMLPCYSTARIPLPNLNEDLTCGNLLMWEAVTVQT	118
AFQ31651.1	ENLRGFSL--KLSAENNFSSDSPERKMLPCYSTARIPLPNLNEDLTCGNLLMWEAVTVQT	118
QTJ14958.1	ENLRGFSV--KLSAENDFSSDSPERKMLPCYSTARIPLPNLNEDLTCGNLLMWEAVTVQT	118
AYO46618.1	ENLRGFSV--KLSAENDFSSDSPERKMLPCYSTARIPLPNLNEDLTCGNLLMWEAVTVQT	118
QTJ15018.1	ENLRGFSL--KLSAENDFSSDSPERKMLPCYSTARIPLPNLNEDLTCGNLLMWEAVTVQT	118
AYO46609.1	ENLRGFSL--KLSAENDFSSDSPERKMLPCYSTARIPLPNLNEDLTCGNLLMWEAVTVQT	118
ALF06356.1	ENLRGFSL--KLSAENDFSSDSPERKMLPCYSTARIPLPNLNEDLTCGNLLMWEAVTVQT	118
AFQ31646.1	ENLRGFSL--KLSAENDFSSDSPERKMLPCYSTARIPLPNLNEDLTCGNLLMWEAVTVQT	118
AFQ31659.1	ENLRGFSL--KLSAENDFSSDSPERKMLPCYSTARIPLPNLNEDLTCGNLLMWEAVTVQT	118
BAF02957.1	ENLRGFSL--KLSAENDFSSDSPERKMLPCYSTARIPLPNLNEDLTCGNLLMWEAVTVQT	118
AAT47365.1	ENLRGFSL--KLSAENDFSSDSPERKMLPCYSTARIPLPNLNEDLTCGNLLMWEAVTVQT	118
AAT47389.1	ENLRGFSL--KLSAENDFSSDSPERKMLPCYSTARIPLPNLNEDLTCGNLLMWEAVTVQT	118
AAT47395.1	ENLRGFSL--KLSAENDFSSDSPERKMLPCYSTARIPLPNLNEDLTCGNLLMWEAVTVQT	118
AAT47401.1	ENLRGFSL--KLSAENDFSSDSPERKMLPCYSTARIPLPNLNEDLTCGNLLMWEAVTVQT	118
CAA24307.1	ENLRGFSL--KLSAENDFSSDSPERKMLPCYSTARIPLPNLNEDLTCGNLLMWEAVTVQT	118
AAT47425.1	ENLRGFSL--KLSAQNDFSSDSPERKMLPCYSTARIPLPNLNEDLTCGNLLMWEAVTVQT	118
AFA41880.1	ENLRGFSL--KLSAENDFSSDSPERKMLPCYSTARIPLPNLNEDLTCGNLLMWEAVTVQT	118
WLD47968.1	KNLRGFSL--KLSAENDFSSDSPERKMLPCYSTARIPLPNLNEDLTCGNLLMWEAVTVQT	118
AFA41920.1	ENLRGFSL--KLSAQNDFNSDSPERKMLPCYSTARIPLPNLNEDLTCGNLLMWEAVTVQT	118
CBX88302.1	ENLRGFSL--KLSAENDFSSDSPERKMLPCYSTARIPLPNLNEDLTCGNLLMWEAVTVQT	118
AUF70036.1	ENLRGFSL--KLSAENDFSSDSPERKMLPCYSTARIPLPNLNEDLTCGNLLMWEAVTVQT	118
ALF06323.1	ENLRGFSL--KLSAENDFSSDSPERKMLPCYSTARIPLPNLNEDLTCGNLLMWEAVTVQT	118
WLD47890.1	ENLRGFSL--MLSAENNFSSDSPERKMLPCYSTARIPLPNLNEDLTCGNLLMWEAVTVQT	118
BAF03097.1	ENLRGFSL--KVSAENDFSSDSPDRKMLPCYSTARIPLPNLNEDLTCGNLLMWEAVTVQT	118
ABI94695.1	ENLRGFSL--KLSAENDFSSDSPDRKMLPCYSTARIPLPNLNEDLTCGNLLMWEAVTVQT	118
ABI94623.1	ENLRGFSL--KLSAKNDFSSDSPDRKMLPCYSTARIPLPNLNEDLTCGNLLMWEAVTVQT	118
UTM62533.1	ENLRGFSL--KLSAENDFSSDSPDRKMLPCYSTARIPLPNLNEDLTCGNLLMWEAVTVQT	118
QOS45058.1	ENLRGFSL--KLSAENDFSSDSPDRKMLPCYSTARIPLPNLNEDLTCGNLLMWEAVTVQT	118
AHU87362.1	ENLRGFSL--KLSAENDFSSDSPDRKMLPCYSTARIPLPNLNEDLTCGNLLMWEAVTVQT	118
AHU87363.1	ENLRGFSL--KLSAENDFSSDSPDRKMLPCYSTARIPLPNLNEDLTCGNLLMWEAVTVQT	118
AFQ31658.1	ENLRGFSL--KLSAENDFSSDSPDRKMLPCYSTARIPLPNLNEDLTCGNLLMWEAVTVQT	118
AFA41876.1	ENLRGFSL--KLSAENDFSSDSPDRKMLPCYSTARIPLPNLNEDLTCGNLLMWEAVTVQT	118
AFA41906.1	ENLRGFSL--KLSAENDFSSDSPDRKMLPCYSTARIPLPNLNEDLTCGNLLMWEAVTVQT	118
AEO89604.1	ENLRGFSL--KLSAENDFSSDSPDRKMLPCYSTARIPLPNLNEDLTCGNLLMWEAVTVQT	118
CBX88350.1	ENLRGFSL--KLSAENDFSSDSPDRKMLPCYSTARIPLPNLNEDLTCGNLLMWEAVTVQT	118
BAG84446.1	ENLRGFSL--KLSAENDFSSDSPDRKMLPCYSTARIPLPNLNEDLTCGNLLMWEAVTVQT	118
BAG84464.1	ENLRGFSL--KLSAENDFSSDSPDRKMLPCYSTARIPLPNLNEDLTCGNLLMWEAVTVQT	118
BAF93295.1	ENLRGFSL--KLSAENDFSSDSPDRKMLPCYSTARIPLPNLNEDLTCGNLLMWEAVTVQT	118
BAF93373.1	ENLRGFSL--KLSAENDFSSDSPDRKMLPCYSTARIPLPNLNEDLTCGNLLMWEAVTVQT	118
BAF93379.1	ENLRGFSL--KLSAENDFSSDSPDRKMLPCYSTARIPLPNLNEDLTCGNLLMWEAVTVQT	118
BAF42913.1	ENLRGFSL--KLSAENDFSSDSPDRKMLPCYSTARIPLPNLNEDLTCGNLLMWEAVTVQT	118
BAF42931.1	ENLRGFSL--KLSAENDFSSDSPDRKMLPCYSTARIPLPNLNEDLTCGNLLMWEAVTVQT	118
BAF43015.1	ENLRGFSL--KLSAENDFSSDSPDRKMLPCYSTARIPLPNLNEDLTCGNLLMWEAVTVQT	118
ABI94629.1	ENLRGFSL--KLSAENDFSSDSPDRKMLPCYSTARIPLPNLNEDLTCGNLLMWEAVTVQT	118
ABI94653.1	ENLRGFSL--KLSAENDFSSDSPDRKMLPCYSTARIPLPNLNEDLTCGNLLMWEAVTVQT	118
ABI94707.1	ENLRGFSL--KLSAENDFSSDSPDRKMLPCYSTARIPLPNLNEDLTCGNLLMWEAVTVQT	118
BAF03085.1	ENLRGFSL--KLSAENDFSSDSPDRKMLPCYSTARIPLPNLNEDLTCGNLLMWEAVTVQT	118
BAF03091.1	ENLRGFSL--KLSAENDFSSDSPDRKMLPCYSTARIPLPNLNEDLTCGNLLMWEAVTVQT	118
BAF03103.1	ENLRGFSL--KLSAENDFSSDSPDRKMLPCYSTARIPLPNLNEDLTCGNLLMWEAVTVQT	118
BAF03109.1	ENLRGFSL--KLSAENDFSSDSPDRKMLPCYSTARIPLPNLNEDLTCGNLLMWEAVTVQT	118
BAF93325.1	ENLRGFSL--KLSAENDFSSDSPDRKMLPCYSTARIPLPNLNEDLTCGNLLMWEAVTVQT	118
QTJ14934.1	ENLRGFSL--KLSAEKDFSSDSPERKMLPCYSTARIPLPNLNEDLTCGNLLMWEAVTVQT	118
QTJ14994.1	ENLRGFSL--KLSAEKDFSSDSPERKMLPCYSTARIPLPNLNEDLTCGNLLMWEAVTVQT	118
AFA41886.1	ENLRGFSL--KLSAENDFSSDSPERKMLPCYSTARIPLPNLNEDLTCGNLLMWEAVTVQT	118
AFQ31650.1	ENLRGFSL--RLSAENDFSSDSPERKMLPCYSTARIPLPNLNEDLTCGNLLMWEAVTVQT	118
AAT47347.1	ENLRGFSL--KLSAENDFSSDSPERKMLPCYSTARIPLPNLNEDLTCGNLLMWEAVTVQT	118
ABI94617.1	ENLRGFSL--KLSAENDFSSDSPERKMLPCYSTARIPLPNLNEDLTCGNLLMWEAVTVQT	118
BAF93319.1	ENLRGFSL--KLSAENDFSSDSPERKMLPCYSTARIPLPNLNEDLTCGNLLMWEAVTVQT	118
CBX88314.1	ENLRGFSL--KLSAENDFSSDSPERKMLPCYSTARIPLPNLNEDLTCGNLLMWEAVTVQT	118
ALF06320.1	ENLRGFSL--KLSAENDFSSDSPERKMLPCYSTARIPLPNLNEDLTCGNLLMWEAVTVQT	118

WLD47830.1	ENLRGFSL--KLSAENDFSSDSPERKMLPCYSTARIPLPNLNEDLTCGNLLMWEAVTVQT	118
WLD47866.1	ENLRGFSL--KLSAENDFSSDSPERKMLPCYSTARIPLPNLNEDLTCGNLLMWEAVTVQT	118
UTM61939.1	ENLRGFSL--KLSAENDFSSDSPERKMLPCYSTARIPLPNLNEDLTCGNLLMWEAVTVQT	118
UTM62023.1	ENLRGFSL--KLSAENDFSSDSPERKMLPCYSTARIPLPNLNEDLTCGNLLMWEAVTVQT	118
UTM62029.1	ENLRGFSL--KLSAENDFSSDSPERKMLPCYSTARIPLPNLNEDLTCGNLLMWEAVTVQT	118
UTM62053.1	ENLRGFSL--KLSAENDFSSDSPERKMLPCYSTARIPLPNLNEDLTCGNLLMWEAVTVQT	118
UTM62059.1	ENLRGFSL--KLSAENDFSSDSPERKMLPCYSTARIPLPNLNEDLTCGNLLMWEAVTVQT	118
UTM62065.1	ENLRGFSL--KLSAENDFSSDSPERKMLPCYSTARIPLPNLNEDLTCGNLLMWEAVTVQT	118
UTM62317.1	ENLRGFSL--KLSAENDFSSDSPERKMLPCYSTARIPLPNLNEDLTCGNLLMWEAVTVQT	118
UTM62323.1	ENLRGFSL--KLSAENDFSSDSPERKMLPCYSTARIPLPNLNEDLTCGNLLMWEAVTVQT	118
UTM62353.1	ENLRGFSL--KLSAENDFSSDSPERKMLPCYSTARIPLPNLNEDLTCGNLLMWEAVTVQT	118
UTM62473.1	ENLRGFSL--KLSAENDFSSDSPERKMLPCYSTARIPLPNLNEDLTCGNLLMWEAVTVQT	118
UTM62491.1	ENLRGFSL--KLSAENDFSSDSPERKMLPCYSTARIPLPNLNEDLTCGNLLMWEAVTVQT	118
UTM62497.1	ENLRGFSL--KLSAENDFSSDSPERKMLPCYSTARIPLPNLNEDLTCGNLLMWEAVTVQT	118
UTM62503.1	ENLRGFSL--KLSAENDFSSDSPERKMLPCYSTARIPLPNLNEDLTCGNLLMWEAVTVQT	118
QTJ14988.1	ENLRGFSL--KLSAENDFSSDSPERKMLPCYSTARIPLPNLNEDLTCGNLLMWEAVTVQT	118
QTJ15024.1	ENLRGFSL--KLSAENDFSSDSPERKMLPCYSTARIPLPNLNEDLTCGNLLMWEAVTVQT	118
QTJ15030.1	ENLRGFSL--KLSAENDFSSDSPERKMLPCYSTARIPLPNLNEDLTCGNLLMWEAVTVQT	118
QTJ15102.1	ENLRGFSL--KLSAENDFSSDSPERKMLPCYSTARIPLPNLNEDLTCGNLLMWEAVTVQT	118
ALF06300.1	ENLRGFSL--KLSAENDFSSDSPERKMLPCYSTARIPLPNLNEDLTCGNLLMWEAVTVQT	118
AHU87342.1	ENLRGFSL--KLSAENDFSSDSPERKMLPCYSTARIPLPNLNEDLTCGNLLMWEAVTVQT	118
AHU87343.1	ENLRGFSL--KLSAENDFSSDSPERKMLPCYSTARIPLPNLNEDLTCGNLLMWEAVTVQT	118
AHU87346.1	ENLRGFSL--KLSAENDFSSDSPERKMLPCYSTARIPLPNLNEDLTCGNLLMWEAVTVQT	118
AHU87347.1	ENLRGFSL--KLSAENDFSSDSPERKMLPCYSTARIPLPNLNEDLTCGNLLMWEAVTVQT	118
AHU87349.1	ENLRGFSL--KLSAENDFSSDSPERKMLPCYSTARIPLPNLNEDLTCGNLLMWEAVTVQT	118
AHU87356.1	ENLRGFSL--KLSAENDFSSDSPERKMLPCYSTARIPLPNLNEDLTCGNLLMWEAVTVQT	118
AHU87357.1	ENLRGFSL--KLSAENDFSSDSPERKMLPCYSTARIPLPNLNEDLTCGNLLMWEAVTVQT	118
AHU87358.1	ENLRGFSL--KLSAENDFSSDSPERKMLPCYSTARIPLPNLNEDLTCGNLLMWEAVTVQT	118
AHU87359.1	ENLRGFSL--KLSAENDFSSDSPERKMLPCYSTARIPLPNLNEDLTCGNLLMWEAVTVQT	118
AFQ31643.1	ENLRGFSL--KLSAENDFSSDSPERKMLPCYSTARIPLPNLNEDLTCGNLLMWEAVTVQT	118
AFQ31656.1	ENLRGFSL--KLSAENDFSSDSPERKMLPCYSTARIPLPNLNEDLTCGNLLMWEAVTVQT	118
AFA41879.1	ENLRGFSL--KLSAENDFSSDSPERKMLPCYSTARIPLPNLNEDLTCGNLLMWEAVTVQT	118
AFA41897.1	ENLRGFSL--KLSAENDFSSDSPERKMLPCYSTARIPLPNLNEDLTCGNLLMWEAVTVQT	118
AFA41900.1	ENLRGFSL--KLSAENDFSSDSPERKMLPCYSTARIPLPNLNEDLTCGNLLMWEAVTVQT	118
AEO89580.1	ENLRGFSL--KLSAENDFSSDSPERKMLPCYSTARIPLPNLNEDLTCGNLLMWEAVTVQT	118
AEO89592.1	ENLRGFSL--KLSAENDFSSDSPERKMLPCYSTARIPLPNLNEDLTCGNLLMWEAVTVQT	118
BAF93277.1	ENLRGFSL--KLSAENDFSSDSPERKMLPCYSTARIPLPNLNEDLTCGNLLMWEAVTVQT	118
BAF93289.1	ENLRGFSL--KLSAENDFSSDSPERKMLPCYSTARIPLPNLNEDLTCGNLLMWEAVTVQT	118
BAF93307.1	ENLRGFSL--KLSAENDFSSDSPERKMLPCYSTARIPLPNLNEDLTCGNLLMWEAVTVQT	118
BAF93337.1	ENLRGFSL--KLSAENDFSSDSPERKMLPCYSTARIPLPNLNEDLTCGNLLMWEAVTVQT	118
BAF42895.1	ENLRGFSL--KLSAENDFSSDSPERKMLPCYSTARIPLPNLNEDLTCGNLLMWEAVTVQT	118
BAF42919.1	ENLRGFSL--KLSAENDFSSDSPERKMLPCYSTARIPLPNLNEDLTCGNLLMWEAVTVQT	118
BAF42943.1	ENLRGFSL--KLSAENDFSSDSPERKMLPCYSTARIPLPNLNEDLTCGNLLMWEAVTVQT	118
BAF42949.1	ENLRGFSL--KLSAENDFSSDSPERKMLPCYSTARIPLPNLNEDLTCGNLLMWEAVTVQT	118
ABI94665.1	ENLRGFSL--KLSAENDFSSDSPERKMLPCYSTARIPLPNLNEDLTCGNLLMWEAVTVQT	118
ABI94677.1	ENLRGFSL--KLSAENDFSSDSPERKMLPCYSTARIPLPNLNEDLTCGNLLMWEAVTVQT	118
BAF02939.1	ENLRGFSL--KLSAENDFSSDSPERKMLPCYSTARIPLPNLNEDLTCGNLLMWEAVTVQT	118
AAT47353.1	ENLRGFSL--KLSAENDFSSDSPERKMLPCYSTARIPLPNLNEDLTCGNLLMWEAVTVQT	118
AAT47359.1	ENLRGFSL--KLSAENDFSSDSPERKMLPCYSTARIPLPNLNEDLTCGNLLMWEAVTVQT	118
CAA40247.1	ENLRGFSL--KLSAENDFSSDSPERKMLPCYSTARIPLPNLNEDLTCGNLLMWEAVTVQT	118
QTJ14982.1	ENLRGFSL--KLSAENNFSSDSPERKMLPCYSTARIPLPNLNEDLTCGNLLMWEAVTVQT	118
QTJ15126.1	ENLRGFSL--KLSAENNFSSDSPERKMLPCYSTARIPLPNLNEDLTCGNLLMWEAVTVQT	118
ABI94635.1	ENLRGFSL--KLSAENDFSSDSPERKMLPCYSTARIPLPNLNEDLTCGNLLMWEAVTVQT	118
ALF06335.1	ENLRGFSL--KLSAENDFSSDSPERKMLPCYSTARIPLPNLNEDLTCGNLLMWEAVTVQT	118
AHU87348.1	ENLRGFSL--KLSAENDFSSDSPERKMLPCYSTARIPLPNLNEDLTCGNLLMWEAVTVQT	118
AFQ31649.1	ENLRGFSL--KLSAENDFSSDSPERKMLPCYSTARIPLPNLNEDLTCGNLLMWEAVTVQT	118
AFQ31654.1	ENLRGFSL--KLSAENDFSSDSPERKMLPCYSTARIPLPNLNEDLTCGNLLMWEAVTVQT	118
BAF42907.1	ENLRGFSL--KLSAENDFSSDSPERKMLPCYSTARIPLPNLNEDLTCGNLLMWEAVTVQT	118
WLD47824.1	ENLRGFSL--KLSAENDFSSDSPERKMLPCYSTARIPLPNLNEDLTCGNLLMWEAVTVQT	118
WLD47848.1	ENLRGFSL--KLSAENDFSSDSPERKMLPCYSTARIPLPNLNEDLTCGNLLMWEAVTVQT	118
WLD47854.1	ENLRGFSL--KLSAENDFSSDSPERKMLPCYSTARIPLPNLNEDLTCGNLLMWEAVTVQT	118
WLD47860.1	ENLRGFSL--KLSAENDFSSDSPERKMLPCYSTARIPLPNLNEDLTCGNLLMWEAVTVQT	118
WLD47878.1	ENLRGFSL--KLSAENDFSSDSPERKMLPCYSTARIPLPNLNEDLTCGNLLMWEAVTVQT	118
WLD47908.1	ENLRGFSL--KLSAENDFSSDSPERKMLPCYSTARIPLPNLNEDLTCGNLLMWEAVTVQT	118

WLD47914.1	ENLRGFSL--KLSAENDFSSDSPERKMLPCYSTARIPLPNLNEDLTCGNLLMWEAVTVQT	118
WLD47920.1	ENLRGFSL--KLSAENDFSSDSPERKMLPCYSTARIPLPNLNEDLTCGNLLMWEAVTVQT	118
WLD47926.1	ENLRGFSL--KLSAENDFSSDSPERKMLPCYSTARIPLPNLNEDLTCGNLLMWEAVTVQT	118
WLD47932.1	ENLRGFSL--KLSAENDFSSDSPERKMLPCYSTARIPLPNLNEDLTCGNLLMWEAVTVQT	118
WLD47950.1	ENLRGFSL--KLSAENDFSSDSPERKMLPCYSTARIPLPNLNEDLTCGNLLMWEAVTVQT	118
WLD47962.1	ENLRGFSL--KLSAENDFSSDSPERKMLPCYSTARIPLPNLNEDLTCGNLLMWEAVTVQT	118
WLD47974.1	ENLRGFSL--KLSAENDFSSDSPERKMLPCYSTARIPLPNLNEDLTCGNLLMWEAVTVQT	118
WLD47998.1	ENLRGFSL--KLSAENDFSSDSPERKMLPCYSTARIPLPNLNEDLTCGNLLMWEAVTVQT	118
WLD48004.1	ENLRGFSL--KLSAENDFSSDSPERKMLPCYSTARIPLPNLNEDLTCGNLLMWEAVTVQT	118
WLD48016.1	ENLRGFSL--KLSAENDFSSDSPERKMLPCYSTARIPLPNLNEDLTCGNLLMWEAVTVQT	118
WLD48022.1	ENLRGFSL--KLSAENDFSSDSPERKMLPCYSTARIPLPNLNEDLTCGNLLMWEAVTVQT	118
WLD48034.1	ENLRGFSL--KLSAENDFSSDSPERKMLPCYSTARIPLPNLNEDLTCGNLLMWEAVTVQT	118
UTM61909.1	ENLRGFSL--KLSAENDFSSDSPERKMLPCYSTARIPLPNLNEDLTCGNLLMWEAVTVQT	118
UTM61915.1	ENLRGFSL--KLSAENDFSSDSPERKMLPCYSTARIPLPNLNEDLTCGNLLMWEAVTVQT	118
UTM61921.1	ENLRGFSL--KLSAENDFSSDSPERKMLPCYSTARIPLPNLNEDLTCGNLLMWEAVTVQT	118
UTM61927.1	ENLRGFSL--KLSAENDFSSDSPERKMLPCYSTARIPLPNLNEDLTCGNLLMWEAVTVQT	118
UTM61933.1	ENLRGFSL--KLSAENDFSSDSPERKMLPCYSTARIPLPNLNEDLTCGNLLMWEAVTVQT	118
UTM61963.1	ENLRGFSL--KLSAENDFSSDSPERKMLPCYSTARIPLPNLNEDLTCGNLLMWEAVTVQT	118
UTM61969.1	ENLRGFSL--KLSAENDFSSDSPERKMLPCYSTARIPLPNLNEDLTCGNLLMWEAVTVQT	118
UTM61975.1	ENLRGFSL--KLSAENDFSSDSPERKMLPCYSTARIPLPNLNEDLTCGNLLMWEAVTVQT	118
UTM61981.1	ENLRGFSL--KLSAENDFSSDSPERKMLPCYSTARIPLPNLNEDLTCGNLLMWEAVTVQT	118
UTM62005.1	ENLRGFSL--KLSAENDFSSDSPERKMLPCYSTARIPLPNLNEDLTCGNLLMWEAVTVQT	118
UTM62017.1	ENLRGFSL--KLSAENDFSSDSPERKMLPCYSTARIPLPNLNEDLTCGNLLMWEAVTVQT	118
UTM62035.1	ENLRGFSL--KLSAENDFSSDSPERKMLPCYSTARIPLPNLNEDLTCGNLLMWEAVTVQT	118
UTM62041.1	ENLRGFSL--KLSAENDFSSDSPERKMLPCYSTARIPLPNLNEDLTCGNLLMWEAVTVQT	118
UTM62047.1	ENLRGFSL--KLSAENDFSSDSPERKMLPCYSTARIPLPNLNEDLTCGNLLMWEAVTVQT	118
UTM62083.1	ENLRGFSL--KLSAENDFSSDSPERKMLPCYSTARIPLPNLNEDLTCGNLLMWEAVTVQT	118
UTM62089.1	ENLRGFSL--KLSAENDFSSDSPERKMLPCYSTARIPLPNLNEDLTCGNLLMWEAVTVQT	118
UTM62095.1	ENLRGFSL--KLSAENDFSSDSPERKMLPCYSTARIPLPNLNEDLTCGNLLMWEAVTVQT	118
UTM62149.1	ENLRGFSL--KLSAENDFSSDSPERKMLPCYSTARIPLPNLNEDLTCGNLLMWEAVTVQT	118
UTM62161.1	ENLRGFSL--KLSAENDFSSDSPERKMLPCYSTARIPLPNLNEDLTCGNLLMWEAVTVQT	118
UTM62167.1	ENLRGFSL--KLSAENDFSSDSPERKMLPCYSTARIPLPNLNEDLTCGNLLMWEAVTVQT	118
UTM62173.1	ENLRGFSL--KLSAENDFSSDSPERKMLPCYSTARIPLPNLNEDLTCGNLLMWEAVTVQT	118
UTM62179.1	ENLRGFSL--KLSAENDFSSDSPERKMLPCYSTARIPLPNLNEDLTCGNLLMWEAVTVQT	118
UTM62185.1	ENLRGFSL--KLSAENDFSSDSPERKMLPCYSTARIPLPNLNEDLTCGNLLMWEAVTVQT	118
UTM62191.1	ENLRGFSL--KLSAENDFSSDSPERKMLPCYSTARIPLPNLNEDLTCGNLLMWEAVTVQT	118
UTM62197.1	ENLRGFSL--KLSAENDFSSDSPERKMLPCYSTARIPLPNLNEDLTCGNLLMWEAVTVQT	118
UTM62203.1	ENLRGFSL--KLSAENDFSSDSPERKMLPCYSTARIPLPNLNEDLTCGNLLMWEAVTVQT	118
UTM62209.1	ENLRGFSL--KLSAENDFSSDSPERKMLPCYSTARIPLPNLNEDLTCGNLLMWEAVTVQT	118
UTM62227.1	ENLRGFSL--KLSAENDFSSDSPERKMLPCYSTARIPLPNLNEDLTCGNLLMWEAVTVQT	118
UTM62239.1	ENLRGFSL--KLSAENDFSSDSPERKMLPCYSTARIPLPNLNEDLTCGNLLMWEAVTVQT	118
UTM62245.1	ENLRGFSL--KLSAENDFSSDSPERKMLPCYSTARIPLPNLNEDLTCGNLLMWEAVTVQT	118
UTM62251.1	ENLRGFSL--KLSAENDFSSDSPERKMLPCYSTARIPLPNLNEDLTCGNLLMWEAVTVQT	118
UTM62257.1	ENLRGFSL--KLSAENDFSSDSPERKMLPCYSTARIPLPNLNEDLTCGNLLMWEAVTVQT	118
UTM62263.1	ENLRGFSL--KLSAENDFSSDSPERKMLPCYSTARIPLPNLNEDLTCGNLLMWEAVTVQT	118
UTM62269.1	ENLRGFSL--KLSAENDFSSDSPERKMLPCYSTARIPLPNLNEDLTCGNLLMWEAVTVQT	118
UTM62287.1	ENLRGFSL--KLSAENDFSSDSPERKMLPCYSTARIPLPNLNEDLTCGNLLMWEAVTVQT	118
UTM62293.1	ENLRGFSL--KLSAENDFSSDSPERKMLPCYSTARIPLPNLNEDLTCGNLLMWEAVTVQT	118
UTM62299.1	ENLRGFSL--KLSAENDFSSDSPERKMLPCYSTARIPLPNLNEDLTCGNLLMWEAVTVQT	118
UTM62305.1	ENLRGFSL--KLSAENDFSSDSPERKMLPCYSTARIPLPNLNEDLTCGNLLMWEAVTVQT	118
UTM62311.1	ENLRGFSL--KLSAENDFSSDSPERKMLPCYSTARIPLPNLNEDLTCGNLLMWEAVTVQT	118
UTM62359.1	ENLRGFSL--KLSAENDFSSDSPERKMLPCYSTARIPLPNLNEDLTCGNLLMWEAVTVQT	118
UTM62365.1	ENLRGFSL--KLSAENDFSSDSPERKMLPCYSTARIPLPNLNEDLTCGNLLMWEAVTVQT	118
UTM62371.1	ENLRGFSL--KLSAENDFSSDSPERKMLPCYSTARIPLPNLNEDLTCGNLLMWEAVTVQT	118
UTM62377.1	ENLRGFSL--KLSAENDFSSDSPERKMLPCYSTARIPLPNLNEDLTCGNLLMWEAVTVQT	118
UTM62455.1	ENLRGFSL--KLSAENDFSSDSPERKMLPCYSTARIPLPNLNEDLTCGNLLMWEAVTVQT	118
UTM62461.1	ENLRGFSL--KLSAENDFSSDSPERKMLPCYSTARIPLPNLNEDLTCGNLLMWEAVTVQT	118
UTM62485.1	ENLRGFSL--KLSAENDFSSDSPERKMLPCYSTARIPLPNLNEDLTCGNLLMWEAVTVQT	118
UTM62509.1	ENLRGFSL--KLSAENDFSSDSPERKMLPCYSTARIPLPNLNEDLTCGNLLMWEAVTVQT	118
UTM62515.1	ENLRGFSL--KLSAENDFSSDSPERKMLPCYSTARIPLPNLNEDLTCGNLLMWEAVTVQT	118
UTM62521.1	ENLRGFSL--KLSAENDFSSDSPERKMLPCYSTARIPLPNLNEDLTCGNLLMWEAVTVQT	118
UTM62527.1	ENLRGFSL--KLSAENDFSSDSPERKMLPCYSTARIPLPNLNEDLTCGNLLMWEAVTVQT	118
UTM62539.1	ENLRGFSL--KLSAENDFSSDSPERKMLPCYSTARIPLPNLNEDLTCGNLLMWEAVTVQT	118
UTM62557.1	ENLRGFSL--KLSAENDFSSDSPERKMLPCYSTARIPLPNLNEDLTCGNLLMWEAVTVQT	118

UTM62563.1	ENLRGFSL--KLSAENDFSSDSPERKMLPCYSTARIPLPNLNEDLTCGNLLMWEAVTVQT	118
UTM62569.1	ENLRGFSL--KLSAENDFSSDSPERKMLPCYSTARIPLPNLNEDLTCGNLLMWEAVTVQT	118
UTM62575.1	ENLRGFSL--KLSAENDFSSDSPERKMLPCYSTARIPLPNLNEDLTCGNLLMWEAVTVQT	118
UTM62581.1	ENLRGFSL--KLSAENDFSSDSPERKMLPCYSTARIPLPNLNEDLTCGNLLMWEAVTVQT	118
UTM62605.1	ENLRGFSL--KLSAENDFSSDSPERKMLPCYSTARIPLPNLNEDLTCGNLLMWEAVTVQT	118
UTM62617.1	ENLRGFSL--KLSAENDFSSDSPERKMLPCYSTARIPLPNLNEDLTCGNLLMWEAVTVQT	118
UTM62623.1	ENLRGFSL--KLSAENDFSSDSPERKMLPCYSTARIPLPNLNEDLTCGNLLMWEAVTVQT	118
UTM62629.1	ENLRGFSL--KLSAENDFSSDSPERKMLPCYSTARIPLPNLNEDLTCGNLLMWEAVTVQT	118
UTM62635.1	ENLRGFSL--KLSAENDFSSDSPERKMLPCYSTARIPLPNLNEDLTCGNLLMWEAVTVQT	118
QTJ15048.1	ENLRGFSL--KLSAENDFSSDSPERKMLPCYSTARIPLPNLNEDLTCGNLLMWEAVTVQT	118
QTJ15144.1	ENLRGFSL--KLSAENDFSSDSPERKMLPCYSTARIPLPNLNEDLTCGNLLMWEAVTVQT	118
AHU87360.1	ENLRGFSL--KLSAENDFSSDSPERKMLPCYSTARIPLPNLNEDLTCGNLLMWEAVTVQT	118
AHU87361.1	ENLRGFSL--KLSAENDFSSDSPERKMLPCYSTARIPLPNLNEDLTCGNLLMWEAVTVQT	118
AHU87366.1	ENLRGFSL--KLSAENDFSSDSPERKMLPCYSTARIPLPNLNEDLTCGNLLMWEAVTVQT	118
AHU87367.1	ENLRGFSL--KLSAENDFSSDSPERKMLPCYSTARIPLPNLNEDLTCGNLLMWEAVTVQT	118
AFQ31652.1	ENLRGFSL--KLSAENDFSSDSPERKMLPCYSTARIPLPNLNEDLTCGNLLMWEAVTVQT	118
AFA41888.1	ENLRGFSL--KLSAENDFSSDSPERKMLPCYSTARIPLPNLNEDLTCGNLLMWEAVTVQT	118
AFA41893.1	ENLRGFSL--KLSAENDFSSDSPERKMLPCYSTARIPLPNLNEDLTCGNLLMWEAVTVQT	118
AFA41894.1	ENLRGFSL--KLSAENDFSSDSPERKMLPCYSTARIPLPNLNEDLTCGNLLMWEAVTVQT	118
AFA41899.1	ENLRGFSL--KLSAENDFSSDSPERKMLPCYSTARIPLPNLNEDLTCGNLLMWEAVTVQT	118
AFA41904.1	ENLRGFSL--KLSAENDFSSDSPERKMLPCYSTARIPLPNLNEDLTCGNLLMWEAVTVQT	118
AFA41905.1	ENLRGFSL--KLSAENDFSSDSPERKMLPCYSTARIPLPNLNEDLTCGNLLMWEAVTVQT	118
AFA41908.1	ENLRGFSL--KLSAENDFSSDSPERKMLPCYSTARIPLPNLNEDLTCGNLLMWEAVTVQT	118
AFA41911.1	ENLRGFSL--KLSAENDFSSDSPERKMLPCYSTARIPLPNLNEDLTCGNLLMWEAVTVQT	118
AFA41912.1	ENLRGFSL--KLSAENDFSSDSPERKMLPCYSTARIPLPNLNEDLTCGNLLMWEAVTVQT	118
AFA41913.1	ENLRGFSL--KLSAENDFSSDSPERKMLPCYSTARIPLPNLNEDLTCGNLLMWEAVTVQT	118
AFA41915.1	ENLRGFSL--KLSAENDFSSDSPERKMLPCYSTARIPLPNLNEDLTCGNLLMWEAVTVQT	118
AFA41916.1	ENLRGFSL--KLSAENDFSSDSPERKMLPCYSTARIPLPNLNEDLTCGNLLMWEAVTVQT	118
AFA41919.1	ENLRGFSL--KLSAENDFSSDSPERKMLPCYSTARIPLPNLNEDLTCGNLLMWEAVTVQT	118
AFA41921.1	ENLRGFSL--KLSAENDFSSDSPERKMLPCYSTARIPLPNLNEDLTCGNLLMWEAVTVQT	118
AFA41922.1	ENLRGFSL--KLSAENDFSSDSPERKMLPCYSTARIPLPNLNEDLTCGNLLMWEAVTVQT	118
AFA41923.1	ENLRGFSL--KLSAENDFSSDSPERKMLPCYSTARIPLPNLNEDLTCGNLLMWEAVTVQT	118
AFA41924.1	ENLRGFSL--KLSAENDFSSDSPERKMLPCYSTARIPLPNLNEDLTCGNLLMWEAVTVQT	118
AFA41925.1	ENLRGFSL--KLSAENDFSSDSPERKMLPCYSTARIPLPNLNEDLTCGNLLMWEAVTVQT	118
CBX88272.1	ENLRGFSL--KLSAENDFSSDSPERKMLPCYSTARIPLPNLNEDLTCGNLLMWEAVTVQT	118
CBX88278.1	ENLRGFSL--KLSAENDFSSDSPERKMLPCYSTARIPLPNLNEDLTCGNLLMWEAVTVQT	118
CBX88284.1	ENLRGFSL--KLSAENDFSSDSPERKMLPCYSTARIPLPNLNEDLTCGNLLMWEAVTVQT	118
CBX88290.1	ENLRGFSL--KLSAENDFSSDSPERKMLPCYSTARIPLPNLNEDLTCGNLLMWEAVTVQT	118
CBX88296.1	ENLRGFSL--KLSAENDFSSDSPERKMLPCYSTARIPLPNLNEDLTCGNLLMWEAVTVQT	118
CBX88308.1	ENLRGFSL--KLSAENDFSSDSPERKMLPCYSTARIPLPNLNEDLTCGNLLMWEAVTVQT	118
CBX88332.1	ENLRGFSL--KLSAENDFSSDSPERKMLPCYSTARIPLPNLNEDLTCGNLLMWEAVTVQT	118
CBX88356.1	ENLRGFSL--KLSAENDFSSDSPERKMLPCYSTARIPLPNLNEDLTCGNLLMWEAVTVQT	118
CBX88362.1	ENLRGFSL--KLSAENDFSSDSPERKMLPCYSTARIPLPNLNEDLTCGNLLMWEAVTVQT	118
BAF93313.1	ENLRGFSL--KLSAENDFSSDSPERKMLPCYSTARIPLPNLNEDLTCGNLLMWEAVTVQT	118
BAF42955.1	ENLRGFSL--KLSAENDFSSDSPERKMLPCYSTARIPLPNLNEDLTCGNLLMWEAVTVQT	118
BAF43021.1	ENLRGFSL--KLSAENDFSSDSPERKMLPCYSTARIPLPNLNEDLTCGNLLMWEAVTVQT	118
ABI94611.1	ENLRGFSL--KLSAENDFSSDSPERKMLPCYSTARIPLPNLNEDLTCGNLLMWEAVTVQT	118
ABI94641.1	ENLRGFSL--KLSAENDFSSDSPERKMLPCYSTARIPLPNLNEDLTCGNLLMWEAVTVQT	118
ABI94647.1	ENLRGFSL--KLSAENDFSSDSPERKMLPCYSTARIPLPNLNEDLTCGNLLMWEAVTVQT	118
WLD47896.1	ENLRGFSL--KLSAENNFSSDSPERKMLPCYSTARIPLPNLNEDLTCGNLLMWEAVTVQT	118
WLD48028.1	ENLRGFSL--KLSAENDFSSDSPQRKMLPCYSTARIPLPNLNEDLTCGNLLMWEAVTVQT	118
BAG84440.1	ENLRGFSL--KLSAENDFSSDSPDRKMLPCYSTARIPLPNLNEDLTCGNLLMWEAVTVQT	118
BAF93283.1	ENLRGFSL--KLSAENDFSSDSPDRKMLPCYSTARIPLPNLNEDLTCGNLLMWEAVTVQT	118
BAF93361.1	ENLRGFSL--KLSAENDFSSDSPDRKMLPCYSTARIPLPNLNEDLTCGNLLMWEAVTVQT	118
WLD48040.1	ENLRGFSL--KLSAQNDFSSDSPERKMLPCYSTARIPLPNLNEDLTCGNLLMWEAVTVQT	118
UTM62611.1	ENLRGFSL--KLSAQNDFSSDSPERKMLPCYSTARIPLPNLNEDLTCGNLLMWEAVTVQT	118
AFQ31645.1	ENLRGFSL--KLSAENDFSSDSPERKMLPCYSTARIPLPNLNEDLTCGNLLMWEAVTVQT	118
AFA41909.1	ENLRGFSL--RLSAENDFSSDSPERKMLPCYSTARIPLPNLNEDLTCGNLLMWEAVTVQT	118
ABI94713.1	ENLRGFSL--KLSAENDFSSDSPERKMLPCYSTARIPLPNLNEDLTCGNLLMWEAVTVQT	118
ABD04662.1	ENLRGFSL--KLSAENDFSSDSPERKMLPCYSTARIPLPNLNEDLTCGNLLMWEAVTVQT	118
QTJ15042.1	ENLRGFSL--RLSTANDFNSDSPQRQMLPCYSTARIPLPNLNEDLTCGNLLMWEAVTVQT	118
AGX89705.1	TVIRGYSQ--HLSAENAFESDSPDRKMLPCYSTARIPLPNLNEDLTCGNLLMWEAVTVKT	118
AGX89710.1	TVIRGYSQ--HLSAENAFESDSPDRKMLPCYSTARIPLPNLNEDLTCGNLLMWEAVTVKT	118
AGX89717.1	TVIRGYSQ--HLSAENAFESDSPDRKMLPCYSTARIPLPNLNEDLTCGNLLMWEAVTVKT	118

AFQ31655.1	DNLRGYSQ--HLSAENAFESDSPDRKMLPCYSTARIPLPNLNEDLTCGNLLMWEAVTVKT	118
AAA46882.1	DNLRGYSQ--HLSAENAFESDSPDRKMLPCYSTARIPLPNLNEDLTCGNLLMWEAVTVKT	118
P14996.1	DNLRGYSQ--HLSAENAFESDSPDRKMLPCYSTARIPLPNLNEDLTCGNLLMWEAVTVKT	118
AEO89615.1	ENLRGYSQ--HLSAENAFESDSPDRKMLPCYSTARIPLPNLNEDLTCGNLLMWEAVTVKT	118
BCV68929.1	DHLRGYSQ--HLTAENAFDSDSPDKKMLPCYSTARIPLPNLNEDLTCGNLLMWEAVTVKT	118
QTJ14952.1	DHLRGYSQ--HLTAENAFDSDSPDKKMLPCYSTARIPLPNLNEDLTCGNLLMWEAVTVKT	118
BAG75157.1	DHLRGYSQ--HLTAENAFDSDSPDKKMLPCYSTARIPLPNLNEDLTCGNLLMWEAVTVKT	118
BAG75211.1	DHLRGYSQ--HLTAENAFDSDSPDKKMLPCYSTARIPLPNLNEDLTCGNLLMWEAVTVKT	118
BAF03017.1	DHLRGYSQ--HLTAENAFDSDSPDKKMLPCYSTARIPLPNLNEDLTCGNLLMWEAVTVKT	118
BAF42925.1	NDLRGYSL--KLTAENAFDSDSPDKKMLPCYSTARIPLPNLNEDLTCGNLLMWEAVTVKT	118
WLD47836.1	DNLRGYSL--KLTAENAFESDSPDKKMLPCYSTARIPLPNLNEDLTCGNLLMWEAVTVKT	118
UEL36354.1	ENLRGFSL--KLTAENAFDSDSPDKKMLPCYSTARIPLPNLNEDLTCGNLLMWEAVTVKT	118
BAF42901.1	ENLRGYSL--KLTAENAFDSDSPDKKMLPCYSTARIPLPNLNEDLTCGNLLMWEAVTVKT	118
ABP87973.1	DNLRGYSL--KLTAENAFDSDSPDKKMLPCYSTARIPLPNLNEDLTCGNLLMWEAVTVKT	118
AFA41878.1	DNLRGYSL--KLTAENAFDSDSPDKKMLPCYSTARIPLPNLNEDLTCGNLLMWEAVTVKT	118
QHI34655.1	DNLRGYSL--KLTAENAFDSDSPDKKMLPCYSTARIPLPNLNEDLTCGNLLMWEAVTVKT	118
UTM61945.1	DNLRGYSL--KLTAENAFDSDSPDKKMLPCYSTARIPLPNLNEDLTCGNLLMWEAVTVKT	118
UTM61987.1	DNLRGYSL--KLTAENAFDSDSPDKKMLPCYSTARIPLPNLNEDLTCGNLLMWEAVTVKT	118
UTM62233.1	DNLRGYSL--KLTAENAFDSDSPDKKMLPCYSTARIPLPNLNEDLTCGNLLMWEAVTVKT	118
UTM62341.1	DNLRGYSL--KLTAENAFDSDSPDKKMLPCYSTARIPLPNLNEDLTCGNLLMWEAVTVKT	118
UTM62347.1	DNLRGYSL--KLTAENAFDSDSPDKKMLPCYSTARIPLPNLNEDLTCGNLLMWEAVTVKT	118
AHU87354.1	DNLRGYSL--KLTAENAFDSDSPDKKMLPCYSTARIPLPNLNEDLTCGNLLMWEAVTVKT	118
AHU87355.1	DNLRGYSL--KLTAENAFDSDSPDKKMLPCYSTARIPLPNLNEDLTCGNLLMWEAVTVKT	118
AHU87364.1	DNLRGYSL--KLTAENAFDSDSPDKKMLPCYSTARIPLPNLNEDLTCGNLLMWEAVTVKT	118
AHU87365.1	DNLRGYSL--KLTAENAFDSDSPDKKMLPCYSTARIPLPNLNEDLTCGNLLMWEAVTVKT	118
AFQ31638.1	DNLRGYSL--KLTAENAFDSDSPDKKMLPCYSTARIPLPNLNEDLTCGNLLMWEAVTVKT	118
AFA41874.1	DNLRGYSL--KLTAENAFDSDSPDKKMLPCYSTARIPLPNLNEDLTCGNLLMWEAVTVKT	118
AFA41875.1	DNLRGYSL--KLTAENAFDSDSPDKKMLPCYSTARIPLPNLNEDLTCGNLLMWEAVTVKT	118
AFA41896.1	DNLRGYSL--KLTAENAFDSDSPDKKMLPCYSTARIPLPNLNEDLTCGNLLMWEAVTVKT	118
BAF93367.1	DNLRGYSL--KLTAENAFDSDSPDKKMLPCYSTARIPLPNLNEDLTCGNLLMWEAVTVKT	118
CAA79596.1	DNLRGYSL--KLTAENAFDSDSPDKKMLPCYSTARIPLPNLNEDLTCGNLLMWEAVTVKT	118
AFA41883.1	NDLRGYSL--RLTAETAFDSDSPDRKMLPCYSTARIPLPNLNEDLTCGNLLMWEAVTVKT	118
WLD47812.1	NDLRGYSL--KLTAGTAFDSDSPDRKMLPCYSTARIPLPNLNEDLTCGNLLMWEAVTVKT	118
BAG75277.1	NDLRGYSL--RLTAETAFDSDSPDRKMLPCYSTARIPLPNLNEDLTCGNLLMWEAVTVKT	118
BAF75138.1	NDLRGYSL--RLTAETAFDSDSPDRKMLPCYSTARIPLPNLNEDLTCGNLLMWEAVTVKT	118
BAF75102.1	NDLRGYSL--RLTAETAFDSDSPDRKMLPCYSTARIPLPNLNEDLTCGNLLMWEAVTVKT	118
AYO46643.1	SDLRGYSL--RLTAETAFDSDSPDRKMLPCYSTARIPLPNLNEDLTCGNLLMWEAVTVKT	118
QUW04844.1	SDLRGYSL--RLTAETAFDSDSPDRKMLPCYSTARIPLPNLNEDLTCGNLLMWEAVTVKT	118
AYO46683.1	SDLRGYSL--RLTAETAFDSDSPDRKMLPCYSTARIPLPNLNEDLTCGNLLMWEAVTVKT	118
AYO46645.1	SDLRGYSL--RLTAETAFDSDSPDRKMLPCYSTARIPLPNLNEDLTCGNLLMWEAVTVKT	118
BAF75180.1	NDLRGYSL--RLTAETAFDSDSPDRKMLPCYSTARIPLPNLNEDLTCGNLLMWEAVTVKT	118
BAF75204.1	NDLRGYSL--RLTAETAFDSDSPDRKMLPCYSTARIPLPNLNEDLTCGNLLMWEAVTVKT	118
AYO46631.1	SNLRGYSL--RLTAETAFDSDSPDRKMLPCYSTARIPLPNLNEDLTCGNLLMWEAVTVKT	118
AYO46608.1	SDLRGYSL--RLTAETAFDSDSPDRKMLPCYSTARIPLPNLNEDLTCGNLLMWEAVTVKT	118
AYO46611.1	SDLRGYSL--RLTAETAFDSDSPDRKMLPCYSTARIPLPNLNEDLTCGNLLMWEAVTVKT	118
AYO46615.1	SDLRGYSL--RLTAETAFDSDSPDRKMLPCYSTARIPLPNLNEDLTCGNLLMWEAVTVKT	118
AYO46639.1	SDLRGYSL--RLTAETAFDSDSPDRKMLPCYSTARIPLPNLNEDLTCGNLLMWEAVTVKT	118
AYO46649.1	SDLRGYSL--RLTAETAFDSDSPDRKMLPCYSTARIPLPNLNEDLTCGNLLMWEAVTVKT	118
AYO46655.1	SDLRGYSL--RLTAETAFDSDSPDRKMLPCYSTARIPLPNLNEDLTCGNLLMWEAVTVKT	118
AYO46664.1	SDLRGYSL--RLTAETAFDSDSPDRKMLPCYSTARIPLPNLNEDLTCGNLLMWEAVTVKT	118
AYO46669.1	SDLRGYSL--RLTAETAFDSDSPDRKMLPCYSTARIPLPNLNEDLTCGNLLMWEAVTVKT	118
AYO46685.1	SDLRGYSL--RLTAETAFDSDSPDRKMLPCYSTARIPLPNLNEDLTCGNLLMWEAVTVKT	118
AYO46686.1	SDLRGYSL--RLTAETAFDSDSPDRKMLPCYSTARIPLPNLNEDLTCGNLLMWEAVTVKT	118
AYO46690.1	SDLRGYSL--RLTAETAFDSDSPDRKMLPCYSTARIPLPNLNEDLTCGNLLMWEAVTVKT	118
AYO46627.1	SDLRGYSL--RLTAETAFHSDSPDRKMLPCYSTARIPLPNLNEDLTCGNLLMWEAVTVKT	118
AYO46630.1	SDLRGYSL--RLTAETAFHSDSPDRKMLPCYSTARIPLPNLNEDLTCGNLLMWEAVTVKT	118
AYO46656.1	SDLRGYSL--RLTAETAFNSDSPDRKMLPCYSTARIPLPNLNEDLTCGNLLMWEAVTVKT	118
BAG75217.1	NDLRGYSL--RLTAETAFDSDSPDRKMLPCYSTARIPLPNLNEDLTCGNLLMWEAVTVKT	118
BAG75229.1	NDLRGYSL--RLTAETAFDSDSPDRKMLPCYSTARIPLPNLNEDLTCGNLLMWEAVTVKT	118
BAG75235.1	NDLRGYSL--RLTAETAFDSDSPDRKMLPCYSTARIPLPNLNEDLTCGNLLMWEAVTVKT	118
BAG75259.1	NDLRGYSL--RLTAETAFDSDSPDRKMLPCYSTARIPLPNLNEDLTCGNLLMWEAVTVKT	118
BAF75150.1	NDLRGYSL--RLTAETAFDSDSPDRKMLPCYSTARIPLPNLNEDLTCGNLLMWEAVTVKT	118
BAF75156.1	NDLRGYSL--RLTAETAFDSDSPDRKMLPCYSTARIPLPNLNEDLTCGNLLMWEAVTVKT	118
BAF75174.1	NDLRGYSL--RLTAETAFDSDSPDRKMLPCYSTARIPLPNLNEDLTCGNLLMWEAVTVKT	118

BAE96083.1	NDLRGYSL--RLTAETAFDSDSPDRKMLPCYSTARIPLPNLNEDLTCGNLLMWEAVTVKT	118
BAE53654.1	NDLRGYSL--RLTAETAFDSDSPDRKMLPCYSTARIPLPNLNEDLTCGNLLMWEAVTVKT	118
BAF03035.1	NDLRGYSL--RLTAETAFNSDSPDRKMLPCYSTARIPLPNLNEDLTCGNLLMWEAVTVKT	118
QHI34656.1	NDLRGYSL--RLTAETAFESDSPDRKMLPCYSTARIPLPNLNEDLTCGSLLMWEAVTVKT	118
ARE72396.1	NDLRGYSL--RLTAETAFESDSPDRKMLPCYSTARIPLPNLNEDLTCGNLLMWEAVTVKT	118
AFA41885.1	NDLRGYSL--RLTAETAFNSDSPDRKMLPCYSTARIPLPNLNEDLTCGNLLMWEAVTVKT	118
BAG75271.1	NDLRGYSL--RLTAETAFDSDSPDRKMLPCYSTARIPLPNLNEDLTCGNLLMWEAVTVKT	118
BAF75090.1	NDLRGYSL--RLTAETAFDSDSPDRKMLPCYSTARIPLPNLNEDLTCGNLLMWEAVTVKT	118
BAF75306.1	NDLRGYSL--RLTAETAFDSDSPDRKMLPCYSTARIPLPNLNEDLTCGNLLMWEAVTVKT	118
BAF75342.1	NDLRGYSL--RLTAETAFDSDSPDRKMLPCYSTARIPLPNLNEDLTCGNLLMWEAVTVKT	118
BAF03029.1	NDLRGYSL--RLTAETAFDSDSPDRKMLPCYSTARIPLPNLNEDLTCGNLLMWEAVTVKT	118
BAG75205.1	NDLRGYSL--RLTAETAFDSDSPDRKMLPCYSTARIPLPNLNEDLTCGNLLMWEAVTVKT	118
BAG75223.1	NDLRGYSL--RLTAETAFDSDSPDRKMLPCYSTARIPLPNLNEDLTCGNLLMWEAVTVKT	118
BAF75144.1	NDLRGYSL--RLTAETAFDSDSPDRKMLPCYSTARIPLPNLNEDLTCGNLLMWEAVTVKT	118
BAF75162.1	NDLRGYSL--RLTAETAFDSDSPDRKMLPCYSTARIPLPNLNEDLTCGNLLMWEAVTVKT	118
BAF75168.1	NDLRGYSL--RLTAETAFDSDSPDRKMLPCYSTARIPLPNLNEDLTCGNLLMWEAVTVKT	118
BAF75240.1	NDLRGYSL--RLTAETAFDSDSPDRKMLPCYSTARIPLPNLNEDLTCGNLLMWEAVTVKT	118
BAF03023.1	NDLRGYSL--RLTAETAFDSDSPDRKMLPCYSTARIPLPNLNEDLTCGNLLMWEAVTVKT	118
BAE96077.1	NDLRGYSL--RLTAETAFDSDSPDRKMLPCYSTARIPLPNLNEDLTCGNLLMWEAVTVKT	118
BAG84476.1	NDLRGYSL--RLTAETAFDSDSPDRKMLPCYSTARIPLPNLNEDLTCGNLLMWEAVTVKT	118
AYO46661.1	SNLRGYSL--RLTAETAFDSDSPDRKMLPCYSTARIPLPNLNEDLTCGNLLMWEAVTVKT	118
AYO46634.1	NDLRGYSL--RLTAETAFDSDSPDRKMLPCYSTARIPLPNLNEDLTCGNLLMWEAVTVKT	118
AYO46657.1	NDLRGYSL--RLTAETAFDSDSPDRKMLPCYSTARIPLPNLNEDLTCGNLLMWEAVTVKT	118
BAG75379.1	NDLRGYSL--RLTAETAFDSDSPDRKMLPCYSTARIPLPNLNEDLTCGNLLMWEAVTVKT	118
BAG75409.1	NDLRGYSL--RLTAETAFDSDSPDRKMLPCYSTARIPLPNLNEDLTCGNLLMWEAVTVKT	118
BAG75415.1	NDLRGYSL--RLTAETAFDSDSPDRKMLPCYSTARIPLPNLNEDLTCGNLLMWEAVTVKT	118
BAF75096.1	NDLRGYSL--RLTAETAFDSDSPDRKMLPCYSTARIPLPNLNEDLTCGNLLMWEAVTVKT	118
BAF75192.1	NDLRGYSL--RLTAETAFDSDSPDRKMLPCYSTARIPLPNLNEDLTCGNLLMWEAVTVKT	118
BAF75198.1	NDLRGYSL--RLTAETAFDSDSPDRKMLPCYSTARIPLPNLNEDLTCGNLLMWEAVTVKT	118
BAF75210.1	NDLRGYSL--RLTAETAFDSDSPDRKMLPCYSTARIPLPNLNEDLTCGNLLMWEAVTVKT	118
BAF75234.1	NDLRGYSL--RLTAETAFDSDSPDRKMLPCYSTARIPLPNLNEDLTCGNLLMWEAVTVKT	118
BAF75264.1	NDLRGYSL--RLTAETAFDSDSPDRKMLPCYSTARIPLPNLNEDLTCGNLLMWEAVTVKT	118
BAF75270.1	NDLRGYSL--RLTAETAFDSDSPDRKMLPCYSTARIPLPNLNEDLTCGNLLMWEAVTVKT	118
BAF75282.1	NDLRGYSL--RLTAETAFDSDSPDRKMLPCYSTARIPLPNLNEDLTCGNLLMWEAVTVKT	118
BAF75300.1	NDLRGYSL--RLTAETAFDSDSPDRKMLPCYSTARIPLPNLNEDLTCGNLLMWEAVTVKT	118
BAF75312.1	NDLRGYSL--RLTAETAFDSDSPDRKMLPCYSTARIPLPNLNEDLTCGNLLMWEAVTVKT	118
BAF75318.1	NDLRGYSL--RLTAETAFDSDSPDRKMLPCYSTARIPLPNLNEDLTCGNLLMWEAVTVKT	118
BAF75324.1	NDLRGYSL--RLTAETAFDSDSPDRKMLPCYSTARIPLPNLNEDLTCGNLLMWEAVTVKT	118
BAF75336.1	NDLRGYSL--RLTAETAFDSDSPDRKMLPCYSTARIPLPNLNEDLTCGNLLMWEAVTVKT	118
AFA41889.1	SDLRGYSL--RLTAETAFDSDSPDRKMLPCYSTARIPLPNLNEDLTCGNLLMWEAVTVKT	118
UEL36357.1	ENLRGFSL--RLTAETAFDSDSPDRKMLPCYSTARIPLPNLNEDLTCGNLLMWEAVTVKT	118
BCV68920.1	NDLRGYSL--RLTAETAFDSDSPDRKMLPCYSTARIPLPNLNEDLTCGNLLMWEAVTVKT	118
WLD47992.1	NDLRGYSL--KLTAETAFNSDSPDRKMLPCYSTARIPLPNLNEDLTCGNLLMWEAVTVKT	118
UTM62413.1	NDLRGYSL--KLTAETAFNSDSPDRKMLPCYSTARIPLPNLNEDLTCGNLLMWEAVTVKT	118
AHU87344.1	NDLRGYSL--RLTAETAFDSDSPDRKMLPCYSTARIPLPNLNEDLTCGNLLMWEAVTVKT	118
AHU87345.1	NDLRGYSL--RLTAETAFDSDSPDRKMLPCYSTARIPLPNLNEDLTCGNLLMWEAVTVKT	118
QTJ14946.1	SDLRGYSL--RLTAETAFESDSPDRKMLPCYSTARIPLPNLNEDLTCGNLLMWEAVTVKT	118
AYO46666.1	SDLRGYSL--RLTAETAFESDSPDRKMLPCYSTARIPLPNLNEDLTCGNLLMWEAVTVKT	118
WLD47788.1	NDLRGYSL--RLTAETAFDSDSPDRKMLPCYSTARIPLPNLNEDLTCGNLLMWEAVTVKT	118
WLD47794.1	NDLRGYSL--RLTAETAFDSDSPDRKMLPCYSTARIPLPNLNEDLTCGNLLMWEAVTVKT	118
WLD47800.1	NDLRGYSL--RLTAETAFDSDSPDRKMLPCYSTARIPLPNLNEDLTCGNLLMWEAVTVKT	118
WLD47806.1	NDLRGYSL--RLTAETAFDSDSPDRKMLPCYSTARIPLPNLNEDLTCGNLLMWEAVTVKT	118
WLD47872.1	NDLRGYSL--RLTAETAFDSDSPDRKMLPCYSTARIPLPNLNEDLTCGNLLMWEAVTVKT	118
WLD47986.1	NDLRGYSL--RLTAETAFDSDSPDRKMLPCYSTARIPLPNLNEDLTCGNLLMWEAVTVKT	118
WLD48010.1	NDLRGYSL--RLTAETAFDSDSPDRKMLPCYSTARIPLPNLNEDLTCGNLLMWEAVTVKT	118
UTM61951.1	NDLRGYSL--RLTAETAFDSDSPDRKMLPCYSTARIPLPNLNEDLTCGNLLMWEAVTVKT	118
UTM61957.1	NDLRGYSL--RLTAETAFDSDSPDRKMLPCYSTARIPLPNLNEDLTCGNLLMWEAVTVKT	118
UTM61993.1	NDLRGYSL--RLTAETAFDSDSPDRKMLPCYSTARIPLPNLNEDLTCGNLLMWEAVTVKT	118
UTM61999.1	NDLRGYSL--RLTAETAFDSDSPDRKMLPCYSTARIPLPNLNEDLTCGNLLMWEAVTVKT	118
UTM62011.1	NDLRGYSL--RLTAETAFDSDSPDRKMLPCYSTARIPLPNLNEDLTCGNLLMWEAVTVKT	118
UTM62071.1	NDLRGYSL--RLTAETAFDSDSPDRKMLPCYSTARIPLPNLNEDLTCGSLLMWEAVTVKT	118
UTM62077.1	NDLRGYSL--RLTAETAFDSDSPDRKMLPCYSTARIPLPNLNEDLTCGNLLMWEAVTVKT	118
UTM62119.1	NDLRGYSL--RLTAETAFDSDSPDRKMLPCYSTARIPLPNLNEDLTCGNLLMWEAVTVKT	118
UTM62125.1	NDLRGYSL--RLTAETAFDSDSPDRKMLPCYSTARIPLPNLNEDLTCGNLLMWEAVTVKT	118

```
UTM62131.1      NDLRGYSL--RLTAETAFDSDSPDRKMLPCYSTARIPLPNLNEDLTCGNLLMWEAVTVKT  118
UTM62137.1      NDLRGYSL--RLTAETAFDSDSPDRKMLPCYSTARIPLPNLNEDLTCGNLLMWEAVTVKT  118
UTM62143.1      NDLRGYSL--RLTAETAFDSDSPDRKMLPCYSTARIPLPNLNEDLTCGNLLMWEAVTVKT  118
UTM62401.1      NDLRGYSL--RLTAETAFDSDSPDRKMLPCYSTARIPLPNLNEDLTCGNLLMWEAVTVKT  118
UTM62407.1      NDLRGYSL--RLTAETAFDSDSPDRKMLPCYSTARIPLPNLNEDLTCGNLLMWEAVTVKT  118
UTM62419.1      NDLRGYSL--RLTAETAFDSDSPDRKMLPCYSTARIPLPNLNEDLTCGNLLMWEAVTVKT  118
UTM62425.1      NDLRGYSL--RLTAETAFDSDSPDRKMLPCYSTARIPLPNLNEDLTCGNLLMWEAVTVKT  118
UTM62431.1      NDLRGYSL--RLTAETAFDSDSPDRKMLPCYSTARIPLPNLNEDLTCGNLLMWEAVTVKT  118
UTM62437.1      NDLRGYSL--RLTAETAFDSDSPDRKMLPCYSTARIPLPNLNEDLTCGNLLMWEAVTVKT  118
UTM62641.1      NDLRGYSL--RLTAETAFDSDSPDRKMLPCYSTARIPLPNLNEDLTCGNLLMWEAVTVKT  118
UTM62647.1      NDLRGYSL--RLTAETAFDSDSPDRKMLPCYSTARIPLPNLNEDLTCGNLLMWEAVTVKT  118
ALF06326.1      NDLRGYSL--RLTAETAFDSDSPDRKMLPCYSTARIPLPNLNEDLTCGNLLMWEAVTVKT  118
ALF06329.1      NDLRGYSL--RLTAETAFDSDSPDRKMLPCYSTARIPLPNLNEDLTCGNLLMWEAVTVKT  118
AHU87338.1      NDLRGYSL--RLTAETAFDSDSPDRKMLPCYSTARIPLPNLNEDLTCGNLLMWEAVTVKT  118
AHU87339.1      NDLRGYSL--RLTAETAFDSDSPDRKMLPCYSTARIPLPNLNEDLTCGNLLMWEAVTVKT  118
AHU87350.1      NDLRGYSL--RLTAETAFDSDSPDRKMLPCYSTARIPLPNLNEDLTCGNLLMWEAVTVKT  118
AHU87351.1      NDLRGYSL--RLTAETAFDSDSPDRKMLPCYSTARIPLPNLNEDLTCGNLLMWEAVTVKT  118
AHU87352.1      NDLRGYSL--RLTAETAFDSDSPDRKMLPCYSTARIPLPNLNEDLTCGNLLMWEAVTVKT  118
AHU87353.1      NDLRGYSL--RLTAETAFDSDSPDRKMLPCYSTARIPLPNLNEDLTCGNLLMWEAVTVKT  118
AFQ31653.1      NDLRGYSL--RLTAETAFDSDSPDRKMLPCYSTARIPLPNLNEDLTCGNLLMWEAVTVKT  118
AFA41884.1      NDLRGYSL--RLTAETAFDSDSPDRKMLPCYSTARIPLPNLNEDLTCGNLLMWEAVTVKT  118
CBX88320.1      NDLRGYSL--RLTAETAFDSDSPDRKMLPCYSTARIPLPNLNEDLTCGNLLMWEAVTVKT  118
CBX88326.1      NDLRGYSL--RLTAETAFDSDSPDRKMLPCYSTARIPLPNLNEDLTCGNLLMWEAVTVKT  118
CBX88338.1      NDLRGYSL--RLTAETAFDSDSPDRKMLPCYSTARIPLPNLNEDLTCGNLLMWEAVTVKT  118
CBX88344.1      NDLRGYSL--RLTAETAFDSDSPDRKMLPCYSTARIPLPNLNEDLTCGNLLMWEAVTVKT  118
BAG84452.1      NDLRGYSL--RLTAETAFDSDSPDRKMLPCYSTARIPLPNLNEDLTCGNLLMWEAVTVKT  118
BAG75385.1      NDLRGYSL--RLTAETAFDSDSPDRKMLPCYSTARIPLPNLNEDLTCGNLLMWEAVTVKT  118
BAG75445.1      NDLRGYSL--RLTAETAFDSDSPDRKMLPCYSTARIPLPNLNEDLTCGNLLMWEAVTVKT  118
BAF93343.1      NDLRGYSL--RLTAETAFDSDSPDRKMLPCYSTARIPLPNLNEDLTCGNLLMWEAVTVKT  118
BAF75066.1      NDLRGYSL--RLTAETAFDSDSPDRKMLPCYSTARIPLPNLNEDLTCGNLLMWEAVTVKT  118
BAF75072.1      NDLRGYSL--RLTAETAFDSDSPDRKMLPCYSTARIPLPNLNEDLTCGNLLMWEAVTVKT  118
BAF75078.1      NDLRGYSL--RLTAETAFDSDSPDRKMLPCYSTARIPLPNLNEDLTCGNLLMWEAVTVKT  118
BAF75084.1      NDLRGYSL--RLTAETAFDSDSPDRKMLPCYSTARIPLPNLNEDLTCGNLLMWEAVTVKT  118
BAF75108.1      NDLRGYSL--RLTAETAFDSDSPDRKMLPCYSTARIPLPNLNEDLTCGNLLMWEAVTVKT  118
BAF75132.1      NDLRGYSL--RLTAETAFDSDSPDRKMLPCYSTARIPLPNLNEDLTCGNLLMWEAVTVKT  118
BAF75216.1      NDLRGYSL--RLTAETAFDSDSPDRKMLPCYSTARIPLPNLNEDLTCGNLLMWEAVTVKT  118
BAF75222.1      NDLRGYSL--RLTAETAFDSDSPDRKMLPCYSTARIPLPNLNEDLTCGNLLMWEAVTVKT  118
BAF75228.1      NDLRGYSL--RLTAETAFDSDSPDRKMLPCYSTARIPLPNLNEDLTCGNLLMWEAVTVKT  118
BAF75246.1      NDLRGYSL--RLTAETAFDSDSPDRKMLPCYSTARIPLPNLNEDLTCGNLLMWEAVTVKT  118
BAF75252.1      NDLRGYSL--RLTAETAFDSDSPDRKMLPCYSTARIPLPNLNEDLTCGNLLMWEAVTVKT  118
BAF75258.1      NDLRGYSL--RLTAETAFDSDSPDRKMLPCYSTARIPLPNLNEDLTCGNLLMWEAVTVKT  118
BAF75276.1      NDLRGYSL--RLTAETAFDSDSPDRKMLPCYSTARIPLPNLNEDLTCGNLLMWEAVTVKT  118
BAF75330.1      NDLRGYSL--RLTAETAFDSDSPDRKMLPCYSTARIPLPNLNEDLTCGNLLMWEAVTVKT  118
BAF03115.1      NDLRGYSL--RLTAETAFDSDSPDRKMLPCYSTARIPLPNLNEDLTCGNLLMWEAVTVKT  118
BAF03120.1      NDLRGYSL--RLTAETAFDSDSPDRKMLPCYSTARIPLPNLNEDLTCGNLLMWEAVTVKT  118
WLD47842.1      NDLRGYSL--RLTAETAFESDSPDRKMLPCYSTARIPLPNLNEDLTCGNLLMWEAVTVKT  118
WLD47884.1      NDLRGYSL--RLTAETAFESDSPDRKMLPCYSTARIPLPNLNEDLTCGNLLMWEAVTVKT  118
UTM62101.1      NDLRGYSL--RLTAETAFESDSPDRKMLPCYSTARIPLPNLNEDLTCGNLLMWEAVTVKT  118
UTM62107.1      NDLRGYSL--RLTAETAFESDSPDRKMLPCYSTARIPLPNLNEDLTCGNLLMWEAVTVKT  118
UTM62113.1      NDLRGYSL--RLTAETAFESDSPDRKMLPCYSTARIPLPNLNEDLTCGNLLMWEAVTVKT  118
AFQ31647.1      NDLRGYSL--RLTAETAFESDSPDRKMLPCYSTARIPLPNLNEDLTCGNLLMWEAVTVKT  118
AFA41892.1      NDLRGYSL--RLTAETAFESDSPDRKMLPCYSTARIPLPNLNEDLTCGNLLMWEAVTVKT  118
BAG75403.1      NDLRGYSL--RLTAETAFESDSPDRKMLPCYSTARIPLPNLNEDLTCGNLLMWEAVTVKT  118
BAF75114.1      NDLRGYSL--RLTAETAFESDSPDRKMLPCYSTARIPLPNLNEDLTCGNLLMWEAVTVKT  118
BAF75120.1      NDLRGYSL--RLTAETAFESDSPDRKMLPCYSTARIPLPNLNEDLTCGNLLMWEAVTVKT  118
BAF75126.1      NDLRGYSL--RLTAETAFESDSPDRKMLPCYSTARIPLPNLNEDLTCGNLLMWEAVTVKT  118
BAF75186.1      NDLRGYSL--RLTAETAFESDSPDRKMLPCYSTARIPLPNLNEDLTCGNLLMWEAVTVKT  118
BAF75288.1      NDLRGYSL--RLTAETAFESDSPDRKMLPCYSTARIPLPNLNEDLTCGNLLMWEAVTVKT  118
BAF75294.1      NDLRGYSL--RLTAETAFESDSPDRKMLPCYSTARIPLPNLNEDLTCGNLLMWEAVTVKT  118
BAF75348.1      NDLRGYSL--RLTAETAFESDSPDRKMLPCYSTARIPLPNLNEDLTCGNLLMWEAVTVKT  118
AFA41881.1      NDLRGYSL--RLTAETAFKSDSPDRKMLPCYSTARIPLPNLNEDLTCGNLLMWEAVTVKT  118
                  :**:*    :     * ****  :******** ** *** ******.**** ****:*

ALF06344.1      EVIGITSMLNLHAGSQKVHEXGGGKPXQGSNFHFFAVGGDPLEMQGVLMNYRTKYPXGTI  178
```

QTJ15012.1	EVIGITSMLNLHAGSQKVHENGGGKPIQGSNFHFFAVGGDPLEMQGVLMNYKTKYPDGTV	178
QTJ15120.1	EVIGITSMLNLHAGSQKVHENGGGKPIQGSNFHFFAVGGDPLEMQGVLMNYKTKYPDGTV	178
QHI34657.1	EVIGITSMLNLHAGSQKVHENGGGKPIQGSNFHFFAVGGDPLEMQGVLMNYKTKYPDGTV	178
ARE72390.1	EVIGITSMLNLHAGSQKVHEHGGGKPIQGSNFHFFAVGGDPLEMQGVLMNYRTKYPEGTI	178
QTJ14970.1	EVIGITSMLNLHAGSQKVHEHGGGKPIQGSNFHFFAVGGDPLEMQGVLMNYRTKYPQGTI	178
QTJ14928.1	EVIGITSMLNLHAGSQKVHENGGGKPIQGSNFHFFAVGGDPLEMQGVLMNYRTKYPDGTV	180
AAT47419.1	EVIGITSMLNLHAGSQKVHEHGGGKPIRGSNFHFFAVGGDPLEMQGVLMNYRTKYPEGTI	178
AAT47413.1	EVTGITSMLNLHAGSQKVHEHGGGKPIQGSNFHFFAVGGDPLEMQGVLMNYRTKYPDGTI	178
AFQ31661.1	EVIGITSMLNLHAGSQKVHEHGGGKPIQGSNFHFFAVGGDPLEMQGVLMNYRTKYPDGTI	178
YP_717939.1	EVIGITSMLNLHAGSQKVHEHGGGKPIQGSNFHFFAVGGEPLEMQGVLMNYRSKYPDGTI	178
AFK08545.1	EVIGITSMLNLHAGSQKVHEHGGGKPIQGSNFHFFAVGGEPLEMQGVLMNYRSKYPDGTI	178
P03088.2	EVIGITSMLNLHAGSQKVHEHGGGKPIQGSNFHFFAVGGEPLEMQGVLMNYRSKYPDGTI	178
CAA24299.1	EVIGITSMLNLHAGSQKVHEHGGGKPIQGSNFHFFAVGGEPLEMQGVLMNYRSKYPDGTI	178
QTJ14922.1	EVIGITSMLNLHAGSQKVHEHGGGKPIQGSNFHFFAVGGDPLEMQGVLMNYRTKYPDGTV	178
AFA41877.1	EVIGITSMLNLHAGSQKVHEHGGGKPIQGSNFHFFAVGGDPLEMQGVLMNYRTKYPDGTI	178
AAT47371.1	EVIGITSMLNLHAGSQKVHEHGGGKPIQGSNFHFFAVGGDPLEMQGVLMNYRTKYPDGTI	178
AYO46658.1	EVIGITSMLNLHAGSQKVHEHGGGKPIQGSNFHFFAVGGDPLEMQGVLLNYRTKYPDGTI	178
AYO46610.1	EVIGITSMLNLHAGSQKVHEHGGGKPIQGSNFHFFAVGGDPLEMQGVLLNYRTKYPDGTI	178
AYO46689.1	EVIGITSMLNLHAGSQKVHEHGGGKPIQGSNFHFFAVGGDPLEMQGVLLNYRTKYPDGTI	178
AYO46693.1	EVIGITSMLNLHAGSQKVHEHGGGKPIQGSNFHFFAVGGDPLEMQGVLLNYRTKYPDGTI	178
BAG75283.1	EVIGITSMLNLHAGSQKVHEHGGGKPIQGSNFHFFAVGGDPLEMQGVLMNYRTKYPEGTI	178
BAG75313.1	EVIGITSMLNLHAGSQKVHEHGGGKPIQGSNFHFFAVGGDPLEMQGVLMNYRTKYPEGTI	178
BAG75373.1	EVIGITSMLNLHAGSQKVHEHGGGKPIQGSNFHFFAVGGDPLEMQGVLMNYRTKYPEGTI	178
BCV68914.1	EVIGITSMLNLHAGSQKVHEHGGGKPIQGSNFHFFAVGGDPLEMQGVLMNYRTKYPEGTI	178
BCV68917.1	EVIGITSMLNLHAGSQKVHEHGGGKPIQGSNFHFFAVGGDPLEMQGVLMNYRTKYPEGTI	178
BCV68923.1	EVIGITSMLNLHAGSQKVHEHGGGKPIQGSNFHFFAVGGDPLEMQGVLMNYRTKYPEGTI	178
AYO46620.1	EVIGITSMLNLHAGSQKVHEHGGGKPIQGSNFHFFAVGGDPLEMQGVLMNYRTKYPEGTI	178
AYO46646.1	EVIGITSMLNLHAGSQKVHEHGGGKPIQGSNFHFFAVGGDPLEMQGVLMNYRTKYPEGTI	178
AYO46663.1	EVIGITSMLNLHAGSQKVHEHGGGKPIQGSNFHFFAVGGDPLEMQGVLMNYRTKYPEGTI	178
AYO46694.1	EVIGITSMLNLHAGSQKVHEHGGGKPIQGSNFHFFAVGGDPLEMQGVLMNYRTKYPEGTI	178
BAZ99906.1	EVIGITSMLNLHAGSQKVHEHGGGKPIQGSNFHFFAVGGDPLEMQGVLMNYRTKYPEGTI	178
BAZ99912.1	EVIGITSMLNLHAGSQKVHEHGGGKPIQGSNFHFFAVGGDPLEMQGVLMNYRTKYPEGTI	178
BAI43576.1	EVIGITSMLNLHAGSQKVHEHGGGKPIQGSNFHFFAVGGDPLEMQGVLMNYRTKYPEGTI	178
BAI43594.1	EVIGITSMLNLHAGSQKVHEHGGGKPIQGSNFHFFAVGGDPLEMQGVLMNYRTKYPEGTI	178
BAI43606.1	EVIGITSMLNLHAGSQKVHEHGGGKPIQGSNFHFFAVGGDPLEMQGVLMNYRTKYPEGTI	178
BAI43624.1	EVIGITSMLNLHAGSQKVHEHGGGKPIQGSNFHFFAVGGDPLEMQGVLMNYRTKYPEGTI	178
BAH23450.1	EVIGITSMLNLHAGSQKVHEHGGGKPIQGSNFHFFAVGGDPLEMQGVLMNYRTKYPEGTI	178
BAH23456.1	EVIGITSMLNLHAGSQKVHEHGGGKPIQGSNFHFFAVGGDPLEMQGVLMNYRTKYPEGTI	178
BAH23462.1	EVIGITSMLNLHAGSQKVHEHGGGKPIQGSNFHFFAVGGDPLEMQGVLMNYRTKYPEGTI	178
BAH23468.1	EVIGITSMLNLHAGSQKVHEHGGGKPIQGSNFHFFAVGGDPLEMQGVLMNYRTKYPEGTI	178
BAH23474.1	EVIGITSMLNLHAGSQKVHEHGGGKPIQGSNFHFFAVGGDPLEMQGVLMNYRTKYPEGTI	178
BAH23480.1	EVIGITSMLNLHAGSQKVHEHGGGKPIQGSNFHFFAVGGDPLEMQGVLMNYRTKYPEGTI	178
BAH23486.1	EVIGITSMLNLHAGSQKVHEHGGGKPIQGSNFHFFAVGGDPLEMQGVLMNYRTKYPEGTI	178
BAH23492.1	EVIGITSMLNLHAGSQKVHEHGGGKPIQGSNFHFFAVGGDPLEMQGVLMNYRTKYPEGTI	178
BAH23498.1	EVIGITSMLNLHAGSQKVHEHGGGKPIQGSNFHFFAVGGDPLEMQGVLMNYRTKYPEGTI	178
BAH23504.1	EVIGITSMLNLHAGSQKVHEHGGGKPIQGSNFHFFAVGGDPLEMQGVLMNYRTKYPEGTI	178
BAH23510.1	EVIGITSMLNLHAGSQKVHEHGGGKPIQGSNFHFFAVGGDPLEMQGVLMNYRTKYPEGTI	178
BAH23516.1	EVIGITSMLNLHAGSQKVHEHGGGKPIQGSNFHFFAVGGDPLEMQGVLMNYRTKYPEGTI	178
BAH23522.1	EVIGITSMLNLHAGSQKVHEHGGGKPIQGSNFHFFAVGGDPLEMQGVLMNYRTKYPEGTI	178
BAH23528.1	EVIGITSMLNLHAGSQKVHEHGGGKPIQGSNFHFFAVGGDPLEMQGVLMNYRTKYPEGTI	178
BAH23534.1	EVIGITSMLNLHAGSQKVHEHGGGKPIQGSNFHFFAVGGDPLEMQGVLMNYRTKYPEGTI	178
BAH23540.1	EVIGITSMLNLHAGSQKVHEHGGGKPIQGSNFHFFAVGGDPLEMQGVLMNYRTKYPEGTI	178
BAH23546.1	EVIGITSMLNLHAGSQKVHEHGGGKPIQGSNFHFFAVGGDPLEMQGVLMNYRTKYPEGTI	178
BAH23552.1	EVIGITSMLNLHAGSQKVHEHGGGKPIQGSNFHFFAVGGDPLEMQGVLMNYRTKYPEGTI	178
BAH23558.1	EVIGITSMLNLHAGSQKVHEHGGGKPIQGSNFHFFAVGGDPLEMQGVLMNYRTKYPEGTI	178
BAG84488.1	EVIGITSMLNLHAGSQKVHEHGGGKPIQGSNFHFFAVGGDPLEMQGVLMNYRTKYPEGTI	178
BAG84494.1	EVIGITSMLNLHAGSQKVHEHGGGKPIQGSNFHFFAVGGDPLEMQGVLMNYRTKYPEGTI	178
BAG84500.1	EVIGITSMLNLHAGSQKVHEHGGGKPIQGSNFHFFAVGGDPLEMQGVLMNYRTKYPEGTI	178
BAG84506.1	EVIGITSMLNLHAGSQKVHEHGGGKPIQGSNFHFFAVGGDPLEMQGVLMNYRTKYPEGTI	178
BAG84512.1	EVIGITSMLNLHAGSQKVHEHGGGKPIQGSNFHFFAVGGDPLEMQGVLMNYRTKYPEGTI	178
BAG84518.1	EVIGITSMLNLHAGSQKVHEHGGGKPIQGSNFHFFAVGGDPLEMQGVLMNYRTKYPEGTI	178
BAG84524.1	EVIGITSMLNLHAGSQKVHEHGGGKPIQGSNFHFFAVGGDPLEMQGVLMNYRTKYPEGTI	178
BAG75163.1	EVIGITSMLNLHAGSQKVHEHGGGKPIQGSNFHFFAVGGDPLEMQGVLMNYRTKYPEGTI	178
BAG75169.1	EVIGITSMLNLHAGSQKVHEHGGGKPIQGSNFHFFAVGGDPLEMQGVLMNYRTKYPEGTI	178

BAG75175.1	EVIGITSMLNLHAGSQKVHEHGGGKPIQGSNFHFFAVGGDPLEMQGVLMNYRTKYPEGTI	178
BAG75181.1	EVIGITSMLNLHAGSQKVHEHGGGKPIQGSNFHFFAVGGDPLEMQGVLMNYRTKYPEGTI	178
BAG75187.1	EVIGITSMLNLHAGSQKVHEHGGGKPIQGSNFHFFAVGGDPLEMQGVLMNYRTKYPEGTI	178
BAG75193.1	EVIGITSMLNLHAGSQKVHEHGGGKPIQGSNFHFFAVGGDPLEMQGVLMNYRTKYPEGTI	178
BAG75199.1	EVIGITSMLNLHAGSQKVHEHGGGKPIQGSNFHFFAVGGDPLEMQGVLMNYRTKYPEGTI	178
BAG75241.1	EVIGITSMLNLHAGSQKVHEHGGGKPIQGSNFHFFAVGGDPLEMQGVLMNYRTKYPEGTI	178
BAG75247.1	EVIGITSMLNLHAGSQKVHEHGGGKPIQGSNFHFFAVGGDPLEMQGVLMNYRTKYPEGTI	178
BAG75253.1	EVIGITSMLNLHAGSQKVHEHGGGKPIQGSNFHFFAVGGDPLEMQGVLMNYRTKYPEGTI	178
BAG75265.1	EVIGITSMLNLHAGSQKVHEHGGGKPIQGSNFHFFAVGGDPLEMQGVLMNYRTKYPEGTI	178
BAG75289.1	EVIGITSMLNLHAGSQKVHEHGGGKPIQGSNFHFFAVGGDPLEMQGVLMNYRTKYPEGTI	178
BAG75295.1	EVIGITSMLNLHAGSQKVHEHGGGKPIQGSNFHFFAVGGDPLEMQGVLMNYRTKYPEGTI	178
BAG75307.1	EVIGITSMLNLHAGSQKVHEHGGGKPIQGSNFHFFAVGGDPLEMQGVLMNYRTKYPEGTI	178
BAG75325.1	EVIGITSMLNLHAGSQKVHEHGGGKPIQGSNFHFFAVGGDPLEMQGVLMNYRTKYPEGTI	178
BAG75331.1	EVIGITSMLNLHAGSQKVHEHGGGKPIQGSNFHFFAVGGDPLEMQGVLMNYRTKYPEGTI	178
BAG75337.1	EVIGITSMLNLHAGSQKVHEHGGGKPIQGSNFHFFAVGGDPLEMQGVLMNYRTKYPEGTI	178
BAG75343.1	EVIGITSMLNLHAGSQKVHEHGGGKPIQGSNFHFFAVGGDPLEMQGVLMNYRTKYPEGTI	178
BAG75397.1	EVIGITSMLNLHAGSQKVHEHGGGKPIQGSNFHFFAVGGDPLEMQGVLMNYRTKYPEGTI	178
BAG75421.1	EVIGITSMLNLHAGSQKVHEHGGGKPIQGSNFHFFAVGGDPLEMQGVLMNYRTKYPEGTI	178
BAG75427.1	EVIGITSMLNLHAGSQKVHEHGGGKPIQGSNFHFFAVGGDPLEMQGVLMNYRTKYPEGTI	178
BAG75433.1	EVIGITSMLNLHAGSQKVHEHGGGKPIQGSNFHFFAVGGDPLEMQGVLMNYRTKYPEGTI	178
BAF93349.1	EVIGITSMLNLHAGSQKVHEHGGGKPIQGSNFHFFAVGGDPLEMQGVLMNYRTKYPEGTI	178
BAF76166.1	EVIGITSMLNLHAGSQKVHEHGGGKPIQGSNFHFFAVGGDPLEMQGVLMNYRTKYPEGTI	178
BAF76172.1	EVIGITSMLNLHAGSQKVHEHGGGKPIQGSNFHFFAVGGDPLEMQGVLMNYRTKYPEGTI	178
BAF76178.1	EVIGITSMLNLHAGSQKVHEHGGGKPIQGSNFHFFAVGGDPLEMQGVLMNYRTKYPEGTI	178
BAF76184.1	EVIGITSMLNLHAGSQKVHEHGGGKPIQGSNFHFFAVGGDPLEMQGVLMNYRTKYPEGTI	178
BAF76190.1	EVIGITSMLNLHAGSQKVHEHGGGKPIQGSNFHFFAVGGDPLEMQGVLMNYRTKYPEGTI	178
BAF76202.1	EVIGITSMLNLHAGSQKVHEHGGGKPIQGSNFHFFAVGGDPLEMQGVLMNYRTKYPEGTI	178
BAF42985.1	EVIGITSMLNLHAGSQKVHEHGGGKPIQGSNFHFFAVGGDPLEMQGVLMNYRTKYPEGTI	178
BAF42991.1	EVIGITSMLNLHAGSQKVHEHGGGKPIQGSNFHFFAVGGDPLEMQGVLMNYRTKYPEGTI	178
BAF02951.1	EVIGITSMLNLHAGSQKVHEHGGGKPIQGSNFHFFAVGGDPLEMQGVLMNYRTKYPEGTI	178
BAF02963.1	EVIGITSMLNLHAGSQKVHEHGGGKPIQGSNFHFFAVGGDPLEMQGVLMNYRTKYPEGTI	178
BAF02969.1	EVIGITSMLNLHAGSQKVHEHGGGKPIQGSNFHFFAVGGDPLEMQGVLMNYRTKYPEGTI	178
BAF02981.1	EVIGITSMLNLHAGSQKVHEHGGGKPIQGSNFHFFAVGGDPLEMQGVLMNYRTKYPEGTI	178
BAF02987.1	EVIGITSMLNLHAGSQKVHEHGGGKPIQGSNFHFFAVGGDPLEMQGVLMNYRTKYPEGTI	178
BAF02993.1	EVIGITSMLNLHAGSQKVHEHGGGKPIQGSNFHFFAVGGDPLEMQGVLMNYRTKYPEGTI	178
BAF02999.1	EVIGITSMLNLHAGSQKVHEHGGGKPIQGSNFHFFAVGGDPLEMQGVLMNYRTKYPEGTI	178
BAF03005.1	EVIGITSMLNLHAGSQKVHEHGGGKPIQGSNFHFFAVGGDPLEMQGVLMNYRTKYPEGTI	178
BAF03011.1	EVIGITSMLNLHAGSQKVHEHGGGKPIQGSNFHFFAVGGDPLEMQGVLMNYRTKYPEGTI	178
BAE96065.1	EVIGITSMLNLHAGSQKVHEHGGGKPIQGSNFHFFAVGGDPLEMQGVLMNYRTKYPEGTI	178
BAE53666.1	EVIGITSMLNLHAGSQKVHEHGGGKPIQGSNFHFFAVGGDPLEMQGVLMNYRTKYPEGTI	178
BAE46623.1	EVIGITSMLNLHAGSQKVHEHGGGKPIQGSNFHFFAVGGDPLEMQGVLMNYRTKYPEGTI	178
CAA40235.1	EVIGITSMLNLHAGSQKVHEHGGGKPIQGSNFHFFAVGGDPLEMQGVLMNYRTKYPEGTI	178
BAI43570.1	EVIGITSMLNLHAGSQKVHEHGGGKPIQGSNFHFFAVGGDPLEMQGVLMNYRTKYPEGTI	178
BAI43582.1	EVIGITSMLNLHAGSQKVHEHGGGKPIQGSNFHFFAVGGDPLEMQGVLMNYRTKYPEGTI	178
BAI43612.1	EVIGITSMLNLHAGSQKVHEHGGGKPIQGSNFHFFAVGGDPLEMQGVLMNYRTKYPEGTI	178
BAI43618.1	EVIGITSMLNLHAGSQKVHEHGGGKPIQGSNFHFFAVGGDPLEMQGVLMNYRTKYPEGTI	178
BAI43630.1	EVIGITSMLNLHAGSQKVHEHGGGKPIQGSNFHFFAVGGDPLEMQGVLMNYRTKYPEGTI	178
BAG75301.1	EVIGITSMLNLHAGSQKVHEHGGGKPIQGSNFHFFAVGGDPLEMQGVLMNYRTKYPEGTI	178
BAG75349.1	EVIGITSMLNLHAGSQKVHEHGGGKPIQGSNFHFFAVGGDPLEMQGVLMNYRTKYPEGTI	178
BAG75367.1	EVIGITSMLNLHAGSQKVHEHGGGKPIQGSNFHFFAVGGDPLEMQGVLMNYRTKYPEGTI	178
BAF76196.1	EVIGITSMLNLHAGSQKVHEHGGGKPIQGSNFHFFAVGGDPLEMQGVLMNYRTKYPEGTI	178
BAF02975.1	EVIGITSMLNLHAGSQKVHEHGGGKPIQGSNFHFFAVGGDPLEMQGVLMNYRTKYPEGTI	178
BAI43600.1	EVIGITSMLNLHAGSQKVHEHGGGKPIQGSNFHFFAVGGDPLEMQGVLMNYRTKYPEGTI	178
BAE96071.1	EVIGITSMLNLHAGSQKVHEHGGGKPIQGSNFHFFAVGGDPLEMQGVLMNYRTKYPEGTI	178
BAE53660.1	EVIGITSMLNLHAGSQKVHEHGGGKPIQGSNFHFFAVGGDPLEMQGVLMNYRTKYPEGTI	178
BCV68926.1	EVVGITSMLNLHAGSQKVHEHGGGKPIQGSNFHFFAVGGDPLEMQGVLMNYRTKYPEGTI	178
AYO46647.1	EVIGITSMLNLHAGSQKVHEHGGGKPIQGSNFHFFAVGGDPLEMQGVLMNYRTKYPEGTI	178
BAR80134.1	EVIGITSMLNLHAGSQKVHEHGGGKPIQGSNFHFFAVGGDPLEMQGVLMNYRTKYPEGTI	178
BAR80140.1	EVIGITSMLNLHAGSQKVHEHGGGKPIQGSNFHFFAVGGDPLEMQGVLMNYRTKYPEGTI	178
BAI43588.1	EVIGITSMLNLHAGSQKVHEHGGGKPIQGSNFHFFAVGGDPLEMQGVLMNYRTKYPEGTI	178
BAG75361.1	EVIGITSMLNLHAGSQKVHEHGGGKPIQGSNFHFFAVGGDPLEMQGVLMNYRTKYPEGTI	178
BAE53642.1	EVIGITSMLNLHAGSQKVHEHGGGKPIQGSNFHFFAVGGDPLEMQGVLMNYRTKYPEGTI	178
BAE53648.1	EVIGITSMLNLHAGSQKVHEHGGGKPIQGSNFHFFAVGGDPLEMQGVLMNYRTKYPEGTI	178
AFA41903.1	EVIGITSMLNLHAGSQKVHEHGGGKPIQGSNFHFFAVGGDPLEMQGVLMNYRTKYPEGTI	178

AFA41918.1	EVIGITSMLNLHAGSQKVHEHGGGKPIQGSNFHFFAVGGDPLEMQGVLMNYRTKYPEGTI	178
BAF42937.1	EVIGITSMLNLHAGSQKVHEHGGGKPIQGSNFHFFAVGGDPLEMQGVLMNYRTKYPEGTI	178
BAF42997.1	EVIGITSMLNLHAGSQKVHEHGGGKPIQGSNFHFFAVGGDPLEMQGVLMNYRTKYPEGTI	178
WLD47902.1	EVIGITSMLNLHAGSQKVHEHGGGKPIQGSNFHFFAVGGDPLEMQGVLMNYRTKYPDGTI	178
WLD47938.1	EVIGITSMLNLHAGSQKVHEHGGGKPIQGSNFHFFAVGGDPLEMQGVLMNYRTKYPDGTI	178
WLD47980.1	EVIGITSMLNLHAGSQKVHEHGGGKPIQGSNFHFFAVGGDPLEMQGVLMNYRTKYPDGTI	178
UTM61891.1	EVIGITSMLNLHAGSQKVHEHGGGKPIQGSNFHFFAVGGDPLEMQGVLMNYRTKYPDGTI	178
UTM61897.1	EVIGITSMLNLHAGSQKVHEHGGGKPIQGSNFHFFAVGGDPLEMQGVLMNYRTKYPDGTI	178
UTM61903.1	EVIGITSMLNLHAGSQKVHEHGGGKPIQGSNFHFFAVGGDPLEMQGVLMNYRTKYPDGTI	178
UTM62215.1	EVIGITSMLNLHAGSQKVHEHGGGKPIQGSNFHFFAVGGDPLEMQGVLMNYRTKYPDGTI	178
UTM62221.1	EVIGITSMLNLHAGSQKVHEHGGGKPIQGSNFHFFAVGGDPLEMQGVLMNYRTKYPDGTI	178
UTM62275.1	EVIGITSMLNLHAGSQKVHEHGGGKPIQGSNFHFFAVGGDPLEMQGVLMNYRTKYPDGTI	178
UTM62281.1	EVIGITSMLNLHAGSQKVHEHGGGKPIQGSNFHFFAVGGDPLEMQGVLMNYRTKYPDGTI	178
UTM62329.1	EVIGITSMLNLHAGSQKVHEHGGGKPIQGSNFHFFAVGGDPLEMQGVLMNYRTKYPDGTI	178
UTM62335.1	EVIGITSMLNLHAGSQKVHEHGGGKPIQGSNFHFFAVGGDPLEMQGVLMNYRTKYPDGTI	178
UTM62383.1	EVIGITSMLNLHAGSQKVHEHGGGKPIQGSNFHFFAVGGDPLEMQGVLMNYRTKYPDGTI	178
UTM62395.1	EVIGITSMLNLHAGSQKVHEHGGGKPIQGSNFHFFAVGGDPLEMQGVLMNYRTKYPDGTI	178
UTM62443.1	EVIGITSMLNLHAGSQKVHEHGGGKPIQGSNFHFFAVGGDPLEMQGVLMNYRTKYPDGTI	178
UTM62449.1	EVIGITSMLNLHAGSQKVHEHGGGKPIQGSNFHFFAVGGDPLEMQGVLMNYRTKYPDGTI	178
UTM62467.1	EVIGITSMLNLHAGSQKVHEHGGGKPIQGSNFHFFAVGGDPLEMQGVLMNYRTKYPDGTI	178
UTM62545.1	EVIGITSMLNLHAGSQKVHEHGGGKPIQGSNFHFFAVGGDPLEMQGVLMNYRTKYPDGTI	178
UTM62551.1	EVIGITSMLNLHAGSQKVHEHGGGKPIQGSNFHFFAVGGDPLEMQGVLMNYRTKYPDGTI	178
UTM62587.1	EVIGITSMLNLHAGSQKVHEHGGGKPIQGSNFHFFAVGGDPLEMQGVLMNYRTKYPDGTI	178
UTM62593.1	EVIGITSMLNLHAGSQKVHEHGGGKPIQGSNFHFFAVGGDPLEMQGVLMNYRTKYPDGTI	178
UTM62599.1	EVIGITSMLNLHAGSQKVHEHGGGKPIQGSNFHFFAVGGDPLEMQGVLMNYRTKYPDGTI	178
QUW04850.1	EVIGITSMLNLHAGSQKVHEHGGGKPIQGSNFHFFAVGGDPLEMQGVLMNYRTKYPDGTI	178
QTJ14976.1	EVIGITSMLNLHAGSQKVHEHGGGKPIQGSNFHFFAVGGDPLEMQGVLMNYRTKYPDGTI	178
QTJ15000.1	EVIGITSMLNLHAGSQKVHEHGGGKPIQGSNFHFFAVGGDPLEMQGVLMNYRTKYPDGTI	178
QTJ15054.1	EVIGITSMLNLHAGSQKVHEHGGGKPIQGSNFHFFAVGGDPLEMQGVLMNYRTKYPDGTI	178
QTJ15072.1	EVIGITSMLNLHAGSQKVHEHGGGKPIQGSNFHFFAVGGDPLEMQGVLMNYRTKYPDGTI	178
QTJ15084.1	EVIGITSMLNLHAGSQKVHEHGGGKPIQGSNFHFFAVGGDPLEMQGVLMNYRTKYPDGTI	178
QTJ15090.1	EVIGITSMLNLHAGSQKVHEHGGGKPIQGSNFHFFAVGGDPLEMQGVLMNYRTKYPDGTI	178
QTJ15108.1	EVIGITSMLNLHAGSQKVHEHGGGKPIQGSNFHFFAVGGDPLEMQGVLMNYRTKYPDGTI	178
QTJ15114.1	EVIGITSMLNLHAGSQKVHEHGGGKPIQGSNFHFFAVGGDPLEMQGVLMNYRTKYPDGTI	178
QTJ15132.1	EVIGITSMLNLHAGSQKVHEHGGGKPIQGSNFHFFAVGGDPLEMQGVLMNYRTKYPDGTI	178
QTJ15138.1	EVIGITSMLNLHAGSQKVHEHGGGKPIQGSNFHFFAVGGDPLEMQGVLMNYRTKYPDGTI	178
AYO46612.1	EVIGITSMLNLHAGSQKVHEHGGGKPIQGSNFHFFAVGGDPLEMQGVLMNYRTKYPDGTI	178
AYO46613.1	EVIGITSMLNLHAGSQKVHEHGGGKPIQGSNFHFFAVGGDPLEMQGVLMNYRTKYPDGTI	178
AYO46617.1	EVIGITSMLNLHAGSQKVHEHGGGKPIQGSNFHFFAVGGDPLEMQGVLMNYRTKYPDGTI	178
AYO46621.1	EVIGITSMLNLHAGSQKVHEHGGGKPIQGSNFHFFAVGGDPLEMQGVLMNYRTKYPDGTI	178
AYO46622.1	EVIGITSMLNLHAGSQKVHEHGGGKPIQGSNFHFFAVGGDPLEMQGVLMNYRTKYPDGTI	178
AYO46623.1	EVIGITSMLNLHAGSQKVHEHGGGKPIQGSNFHFFAVGGDPLEMQGVLMNYRTKYPDGTI	178
AYO46624.1	EVIGITSMLNLHAGSQKVHEHGGGKPIQGSNFHFFAVGGDPLEMQGVLMNYRTKYPDGTI	178
AYO46629.1	EVIGITSMLNLHAGSQKVHEHGGGKPIQGSNFHFFAVGGDPLEMQGVLMNYRTKYPDGTI	178
AYO46632.1	EVIGITSMLNLHAGSQKVHEHGGGKPIQGSNFHFFAVGGDPLEMQGVLMNYRTKYPDGTI	178
AYO46635.1	EVIGITSMLNLHAGSQKVHEHGGGKPIQGSNFHFFAVGGDPLEMQGVLMNYRTKYPDGTI	178
AYO46638.1	EVIGITSMLNLHAGSQKVHEHGGGKPIQGSNFHFFAVGGDPLEMQGVLMNYRTKYPDGTI	178
AYO46641.1	EVIGITSMLNLHAGSQKVHEHGGGKPIQGSNFHFFAVGGDPLEMQGVLMNYRTKYPDGTI	178
AYO46642.1	EVIGITSMLNLHAGSQKVHEHGGGKPIQGSNFHFFAVGGDPLEMQGVLMNYRTKYPDGTI	178
AYO46644.1	EVIGITSMLNLHAGSQKVHEHGGGKPIQGSNFHFFAVGGDPLEMQGVLMNYRTKYPDGTI	178
AYO46651.1	EVIGITSMLNLHAGSQKVHEHGGGKPIQGSNFHFFAVGGDPLEMQGVLMNYRTKYPDGTI	178
AYO46652.1	EVIGITSMLNLHAGSQKVHEHGGGKPIQGSNFHFFAVGGDPLEMQGVLMNYRTKYPDGTI	178
AYO46659.1	EVIGITSMLNLHAGSQKVHEHGGGKPIQGSNFHFFAVGGDPLEMQGVLMNYRTKYPDGTI	178
AYO46660.1	EVIGITSMLNLHAGSQKVHEHGGGKPIQGSNFHFFAVGGDPLEMQGVLMNYRTKYPDGTI	178
AYO46662.1	EVIGITSMLNLHAGSQKVHEHGGGKPIQGSNFHFFAVGGDPLEMQGVLMNYRTKYPDGTI	178
AYO46665.1	EVIGITSMLNLHAGSQKVHEHGGGKPIQGSNFHFFAVGGDPLEMQGVLMNYRTKYPDGTI	178
AYO46670.1	EVIGITSMLNLHAGSQKVHEHGGGKPIQGSNFHFFAVGGDPLEMQGVLMNYRTKYPDGTI	178
AYO46672.1	EVIGITSMLNLHAGSQKVHEHGGGKPIQGSNFHFFAVGGDPLEMQGVLMNYRTKYPDGTI	178
AYO46676.1	EVIGITSMLNLHAGSQKVHEHGGGKPIQGSNFHFFAVGGDPLEMQGVLMNYRTKYPDGTI	178
AYO46677.1	EVIGITSMLNLHAGSQKVHEHGGGKPIQGSNFHFFAVGGDPLEMQGVLMNYRTKYPDGTI	178
AYO46679.1	EVIGITSMLNLHAGSQKVHEHGGGKPIQGSNFHFFAVGGDPLEMQGVLMNYRTKYPDGTI	178
AYO46682.1	EVIGITSMLNLHAGSQKVHEHGGGKPIQGSNFHFFAVGGDPLEMQGVLMNYRTKYPDGTI	178
AYO46691.1	EVIGITSMLNLHAGSQKVHEHGGGKPIQGSNFHFFAVGGDPLEMQGVLMNYRTKYPDGTI	178
AYO46692.1	EVIGITSMLNLHAGSQKVHEHGGGKPIQGSNFHFFAVGGDPLEMQGVLMNYRTKYPDGTI	178

AUF70040.1	EVIGITSMLNLHAGSQKVHEHGGGKPIQGSNFHFFAVGGDPLEMQGVLMNYRTKYPDGTI	178
SOH95722.1	EVIGITSMLNLHAGSQKVHEHGGGKPIQGSNFHFFAVGGDPLEMQGVLMNYRTKYPDGTI	178
SOI09711.1	EVIGITSMLNLHAGSQKVHEHGGGKPIQGSNFHFFAVGGDPLEMQGVLMNYRTKYPDGTI	178
APG38208.1	EVIGITSMLNLHAGSQKVHEHGGGKPIQGSNFHFFAVGGDPLEMQGVLMNYRTKYPDGTI	178
ALF06332.1	EVIGITSMLNLHAGSQKVHEHGGGKPIQGSNFHFFAVGGDPLEMQGVLMNYRTKYPDGTI	178
ALF06341.1	EVIGITSMLNLHAGSQKVHEHGGGKPIQGSNFHFFAVGGDPLEMQGVLMNYRTKYPDGTI	178
BAR80122.1	EVIGITSMLNLHAGSQKVHEHGGGKPIQGSNFHFFAVGGDPLEMQGVLMNYRTKYPDGTI	178
BAR80128.1	EVIGITSMLNLHAGSQKVHEHGGGKPIQGSNFHFFAVGGDPLEMQGVLMNYRTKYPDGTI	178
AJG42164.1	EVIGITSMLNLHAGSQKVHEHGGGKPIQGSNFHFFAVGGDPLEMQGVLMNYRTKYPDGTI	178
AHU87340.1	EVIGITSMLNLHAGSQKVHEHGGGKPIQGSNFHFFAVGGDPLEMQGVLMNYRTKYPDGTI	178
AHU87341.1	EVIGITSMLNLHAGSQKVHEHGGGKPIQGSNFHFFAVGGDPLEMQGVLMNYRTKYPDGTI	178
AHU87368.1	EVIGITSMLNLHAGSQKVHEHGGGKPIQGSNFHFFAVGGDPLEMQGVLMNYRTKYPDGTI	178
AHU87369.1	EVIGITSMLNLHAGSQKVHEHGGGKPIQGSNFHFFAVGGDPLEMQGVLMNYRTKYPDGTI	178
AFQ31636.1	EVIGITSMLNLHAGSQKVHEHGGGKPIQGSNFHFFAVGGDPLEMQGVLMNYRTKYPDGTI	178
AFQ31637.1	EVIGITSMLNLHAGSQKVHEHGGGKPIQGSNFHFFAVGGDPLEMQGVLMNYRTKYPDGTI	178
AFQ31639.1	EVIGITSMLNLHAGSQKVHEHGGGKPIQGSNFHFFAVGGDPLEMQGVLMNYRTKYPDGTI	178
AFQ31640.1	EVIGITSMLNLHAGSQKVHEHGGGKPIQGSNFHFFAVGGDPLEMQGVLMNYRTKYPDGTI	178
AFQ31641.1	EVIGITSMLNLHAGSQKVHEHGGGKPIQGSNFHFFAVGGDPLEMQGVLMNYRTKYPDGTI	178
AFQ31644.1	EVIGITSMLNLHAGSQKVHEHGGGKPIQGSNFHFFAVGGDPLEMQGVLMNYRTKYPDGTI	178
AFQ31648.1	EVIGITSMLNLHAGSQKVHEHGGGKPIQGSNFHFFAVGGDPLEMQGVLMNYRTKYPDGTI	178
AFQ31657.1	EVIGITSMLNLHAGSQKVHEHGGGKPIQGSNFHFFAVGGDPLEMQGVLMNYRTKYPDGTI	178
AFA41882.1	EVIGITSMLNLHAGSQKVHEHGGGKPIQGSNFHFFAVGGDPLEMQGVLMNYRTKYPDGTI	178
AFA41887.1	EVIGITSMLNLHAGSQKVHEHGGGKPIQGSNFHFFAVGGDPLEMQGVLMNYRTKYPDGTI	178
AFA41890.1	EVIGITSMLNLHAGSQKVHEHGGGKPIQGSNFHFFAVGGDPLEMQGVLMNYRTKYPDGTI	178
AFA41891.1	EVIGITSMLNLHAGSQKVHEHGGGKPIQGSNFHFFAVGGDPLEMQGVLMNYRTKYPDGTI	178
AFA41895.1	EVIGITSMLNLHAGSQKVHEHGGGKPIQGSNFHFFAVGGDPLEMQGVLMNYRTKYPDGTI	178
AFA41898.1	EVIGITSMLNLHAGSQKVHEHGGGKPIQGSNFHFFAVGGDPLEMQGVLMNYRTKYPDGTI	178
AFA41901.1	EVIGITSMLNLHAGSQKVHEHGGGKPIQGSNFHFFAVGGDPLEMQGVLMNYRTKYPDGTI	178
AFA41902.1	EVIGITSMLNLHAGSQKVHEHGGGKPIQGSNFHFFAVGGDPLEMQGVLMNYRTKYPDGTI	178
AFA41910.1	EVIGITSMLNLHAGSQKVHEHGGGKPIQGSNFHFFAVGGDPLEMQGVLMNYRTKYPDGTI	178
AFA41914.1	EVIGITSMLNLHAGSQKVHEHGGGKPIQGSNFHFFAVGGDPLEMQGVLMNYRTKYPDGTI	178
AFA41917.1	EVIGITSMLNLHAGSQKVHEHGGGKPIQGSNFHFFAVGGDPLEMQGVLMNYRTKYPDGTI	178
AEO89562.1	EVIGITSMLNLHAGSQKVHEHGGGKPIQGSNFHFFAVGGDPLEMQGVLMNYRTKYPDGTI	178
AEO89568.1	EVIGITSMLNLHAGSQKVHEHGGGKPIQGSNFHFFAVGGDPLEMQGVLMNYRTKYPDGTI	178
AEO89586.1	EVIGITSMLNLHAGSQKVHEHGGGKPIQGSNFHFFAVGGDPLEMQGVLMNYRTKYPDGTI	178
AEO89598.1	EVIGITSMLNLHAGSQKVHEHGGGKPIQGSNFHFFAVGGDPLEMQGVLMNYRTKYPDGTI	178
AEO89621.1	EVIGITSMLNLHAGSQKVHEHGGGKPIQGSNFHFFAVGGDPLEMQGVLMNYRTKYPDGTI	178
AEK21505.1	EVIGITSMLNLHAGSQKVHEHGGGKPIQGSNFHFFAVGGDPLEMQGVLMNYRTKYPDGTI	178
BAG84458.1	EVIGITSMLNLHAGSQKVHEHGGGKPIQGSNFHFFAVGGDPLEMQGVLMNYRTKYPDGTI	178
BAG84470.1	EVIGITSMLNLHAGSQKVHEHGGGKPIQGSNFHFFAVGGDPLEMQGVLMNYRTKYPDGTI	178
BAG84482.1	EVIGITSMLNLHAGSQKVHEHGGGKPIQGSNFHFFAVGGDPLEMQGVLMNYRTKYPDGTI	178
BAG75319.1	EVIGITSMLNLHAGSQKVHEHGGGKPIQGSNFHFFAVGGDPLEMQGVLMNYRTKYPDGTI	178
BAG75355.1	EVIGITSMLNLHAGSQKVHEHGGGKPIQGSNFHFFAVGGDPLEMQGVLMNYRTKYPDGTI	178
BAG75391.1	EVIGITSMLNLHAGSQKVHEHGGGKPIQGSNFHFFAVGGDPLEMQGVLMNYRTKYPDGTI	178
BAG75439.1	EVIGITSMLNLHAGSQKVHEHGGGKPIQGSNFHFFAVGGDPLEMQGVLMNYRTKYPDGTI	178
BAF93301.1	EVIGITSMLNLHAGSQKVHEHGGGKPIQGSNFHFFAVGGDPLEMQGVLMNYRTKYPDGTI	178
BAF93331.1	EVIGITSMLNLHAGSQKVHEHGGGKPIQGSNFHFFAVGGDPLEMQGVLMNYRTKYPDGTI	178
BAF93355.1	EVIGITSMLNLHAGSQKVHEHGGGKPIQGSNFHFFAVGGDPLEMQGVLMNYRTKYPDGTI	178
BAF76208.1	EVIGITSMLNLHAGSQKVHEHGGGKPIQGSNFHFFAVGGDPLEMQGVLMNYRTKYPDGTI	178
BAF42877.1	EVIGITSMLNLHAGSQKVHEHGGGKPIQGSNFHFFAVGGDPLEMQGVLMNYRTKYPDGTI	178
BAF42883.1	EVIGITSMLNLHAGSQKVHEHGGGKPIQGSNFHFFAVGGDPLEMQGVLMNYRTKYPDGTI	178
BAF42889.1	EVIGITSMLNLHAGSQKVHEHGGGKPIQGSNFHFFAVGGDPLEMQGVLMNYRTKYPDGTI	178
BAF42961.1	EVIGITSMLNLHAGSQKVHEHGGGKPIQGSNFHFFAVGGDPLEMQGVLMNYRTKYPDGTI	178
BAF42967.1	EVIGITSMLNLHAGSQKVHEHGGGKPIQGSNFHFFAVGGDPLEMQGVLMNYRTKYPDGTI	178
BAF42973.1	EVIGITSMLNLHAGSQKVHEHGGGKPIQGSNFHFFAVGGDPLEMQGVLMNYRTKYPDGTI	178
BAF43003.1	EVIGITSMLNLHAGSQKVHEHGGGKPIQGSNFHFFAVGGDPLEMQGVLMNYRTKYPDGTI	178
BAF43027.1	EVIGITSMLNLHAGSQKVHEHGGGKPIQGSNFHFFAVGGDPLEMQGVLMNYRTKYPDGTI	178
BAF43033.1	EVIGITSMLNLHAGSQKVHEHGGGKPIQGSNFHFFAVGGDPLEMQGVLMNYRTKYPDGTI	178
ABI94659.1	EVIGITSMLNLHAGSQKVHEHGGGKPIQGSNFHFFAVGGDPLEMQGVLMNYRTKYPDGTI	178
ABI94683.1	EVIGITSMLNLHAGSQKVHEHGGGKPIQGSNFHFFAVGGDPLEMQGVLMNYRTKYPDGTI	178
ABI94719.1	EVIGITSMLNLHAGSQKVHEHGGGKPIQGSNFHFFAVGGDPLEMQGVLMNYRTKYPDGTI	178
BAE96053.1	EVIGITSMLNLHAGSQKVHEHGGGKPIQGSNFHFFAVGGDPLEMQGVLMNYRTKYPDGTI	178
BAE96059.1	EVIGITSMLNLHAGSQKVHEHGGGKPIQGSNFHFFAVGGDPLEMQGVLMNYRTKYPDGTI	178
ABC18005.1	EVIGITSMLNLHAGSQKVHEHGGGKPIQGSNFHFFAVGGDPLEMQGVLMNYRTKYPDGTI	178

AAT47377.1	EVIGITSMLNLHAGSQKVHEHGGGKPIQGSNFHFFAVGGDPLEMQGVLMNYRTKYPDGTI	178
AAT47383.1	EVIGITSMLNLHAGSQKVHEHGGGKPIQGSNFHFFAVGGDPLEMQGVLMNYRTKYPDGTI	178
AAT47405.1	EVIGITSMLNLHAGSQKVHEHGGGKPIQGSNFHFFAVGGDPLEMQGVLMNYRTKYPDGTI	178
CAA40239.1	EVIGITSMLNLHAGSQKVHEHGGGKPIQGSNFHFFAVGGDPLEMQGVLMNYRTKYPDGTI	178
QTJ14940.1	EVIGITSMLNLHAGSQKVHEHGGGKPIQGSNFHFFAVGGDPLEMQGVLMNYRTKYPDGTI	178
ALF06317.1	EVIGITSMLNLHAGSQEVHEHGGGKPIQGSNFHFFAVGGDPLEMQGVLMNYRTKYPEGTI	178
ALF06307.1	EVIGITSMLNLHAGSQKVHEHGGGKPIQGSNFHFFAVGGDPLEMQGVLMNYRTKYPEGTI	178
ALF06297.1	EVIGITSMLNLHAGSQKVHEHGGGKPIQGSNFHFFAVGGDPLEMQGVLMNYRTKYPXGTI	178
ALF06350.1	EVIGITSMLNLHAGSQKVHEHGGGKPIQGSNFHFFAVGGDPLEMQGVLMNYRTKYPXGTI	178
AAT47431.1	EVIGITSMLNLHAGSQKVHEHGGGKPIQGSNFHFFAVGGDPLEMQGVLMNYWTKYPEGTI	178
QTJ15006.1	EVIGITSMLNLHAGSQKVHEHGGGKPIQGSNFHFFAVGGDPLEMQGVLMNYRTKYPDGTI	178
AYO46667.1	EVIGITSMLNLHAGSQKVHEHGGGKPIQGSNFHFFAVGGDPLEMQGVLMNYRTKYPDGTI	178
WLD47956.1	EVIGITSMLNLHAGSQKVHENGGGKPIQGSNFHFFAVGGDPLEMQGVLMNYRTKYPDGTI	178
QTJ15150.1	EVIGITSMLNLHAGSQKVHENGGGKPIQGSNFHFFAVGGDPLEMQGVLMNYRTKYPDGTI	178
UTM62389.1	EVIGITSMLNLHAGSQKVHEHGGGKPIQGSNFHFFAVGGDPLEMQGVLMNYRTKYPDGTI	178
ABI94701.1	EVIGITSMLNLHAGSQKVHEHGGGKPIQGSNFHFFAVGGDPLEMQGVLMNYRTKYPDGTI	178
BAF02945.1	EVIGITSMLNLHAGSQKVHEHGGGKPIQGSNFHFFAVGGDPLEMQGVLMNYRTKYPDGTI	178
CAA40243.1	EVIGITSMLNLHAGSQKVHEHGGGKPIQGSNFHFFAVGGDPLEMQGVLMNYRTKYPDGTI	178
ABI94725.1	EVIGITSMLNLHAGSQKVHEHGGGKPIQGSNFHFFAVGGDPLEMQGVLMNYRTKYPDGTI	178
AFQ31660.1	EVIGITSMLNLHAGSQKVHEHGGGKPIQGSNFHFFAVGGDPLEMQGVLMNYRTKYPDGTI	178
ALF06353.1	EVIGITSMLNLHAGSQKVHEHGGGKPIQGSNFHFFAVGGDPLEMQGVLMNYRTKYPDGTX	178
ALF06312.1	EVIGITSMLNLHAGSQKVHEHGGGKPIQGSNLHFSAVGGDPLEMQGVLMNYRTKYPDGTI	178
QEJ80721.1	EVIGITSMLNLHAGSQKVHEHGGGKPIQGSNFHFFAVGGDPLEMQGVLLNYRTKYPDGTI	178
AYO46614.1	EVIGITSMLNLHAGSQKVHEHGGGKPIQGSNFHFFAVGGDPLEMQGVLLNYRTKYPDGTI	178
AYO46619.1	EVIGITSMLNLHAGSQKVHEHGGGKPIQGSNFHFFAVGGDPLEMQGVLLNYRTKYPDGTI	178
AYO46625.1	EVIGITSMLNLHAGSQKVHEHGGGKPIQGSNFHFFAVGGDPLEMQGVLLNYRTKYPDGTI	178
AYO46626.1	EVIGITSMLNLHAGSQKVHEHGGGKPIQGSNFHFFAVGGDPLEMQGVLLNYRTKYPDGTI	178
AYO46628.1	EVIGITSMLNLHAGSQKVHEHGGGKPIQGSNFHFFAVGGDPLEMQGVLLNYRTKYPDGTI	178
AYO46633.1	EVIGITSMLNLHAGSQKVHEHGGGKPIQGSNFHFFAVGGDPLEMQGVLLNYRTKYPDGTI	178
AYO46636.1	EVIGITSMLNLHAGSQKVHEHGGGKPIQGSNFHFFAVGGDPLEMQGVLLNYRTKYPDGTI	178
AYO46637.1	EVIGITSMLNLHAGSQKVHEHGGGKPIQGSNFHFFAVGGDPLEMQGVLLNYRTKYPDGTI	178
AYO46640.1	EVIGITSMLNLHAGSQKVHEHGGGKPIQGSNFHFFAVGGDPLEMQGVLLNYRTKYPDGTI	178
AYO46648.1	EVIGITSMLNLHAGSQKVHEHGGGKPIQGSNFHFFAVGGDPLEMQGVLLNYRTKYPDGTI	178
AYO46650.1	EVIGITSMLNLHAGSQKVHEHGGGKPIQGSNFHFFAVGGDPLEMQGVLLNYRTKYPDGTI	178
AYO46653.1	EVIGITSMLNLHAGSQKVHEHGGGKPIQGSNFHFFAVGGDPLEMQGVLLNYRTKYPDGTI	178
AYO46654.1	EVIGITSMLNLHAGSQKVHEHGGGKPIQGSNFHFFAVGGDPLEMQGVLLNYRTKYPDGTI	178
AYO46671.1	EVIGITSMLNLHAGSQKVHEHGGGKPIQGSNFHFFAVGGDPLEMQGVLLNYRTKYPDGTI	178
AYO46673.1	EVIGITSMLNLHAGSQKVHEHGGGKPIQGSNFHFFAVGGDPLEMQGVLLNYRTKYPDGTI	178
AYO46674.1	EVIGITSMLNLHAGSQKVHEHGGGKPIQGSNFHFFAVGGDPLEMQGVLLNYRTKYPDGTI	178
AYO46675.1	EVIGITSMLNLHAGSQKVHEHGGGKPIQGSNFHFFAVGGDPLEMQGVLLNYRTKYPDGTI	178
AYO46680.1	EVIGITSMLNLHAGSQKVHEHGGGKPIQGSNFHFFAVGGDPLEMQGVLLNYRTKYPDGTI	178
AYO46681.1	EVIGITSMLNLHAGSQKVHEHGGGKPIQGSNFHFFAVGGDPLEMQGVLLNYRTKYPDGTI	178
AYO46684.1	EVIGITSMLNLHAGSQKVHEHGGGKPIQGSNFHFFAVGGDPLEMQGVLLNYRTKYPDGTI	178
AYO46687.1	EVIGITSMLNLHAGSQKVHEHGGGKPIQGSNFHFFAVGGDPLEMQGVLLNYRTKYPDGTI	178
AEO89574.1	EVIGITSMLNLHAGSQKVHEHGGGKPIQGSNFHFFAVGGDPLEMQGVLLNYRTKYPDGTI	178
BAF42979.1	EVIGITSMLNLHAGSQKVHEHGGGKPIQGSNFHFFAVGGDPLEMQGVLLNYRTKYPDGTI	178
BAF43009.1	EVIGITSMLNLHAGSQKVHEHGGGKPIQGSNFHFFAVGGDPLEMQGVLLNYRTKYPDGTI	178
AYO46678.1	EVIGITSMLNLHAGSQKVHEHGGGKPIQGSNFHFFAVGGDPLEMQGVLLNYRTKYPDGTI	178
UTM62479.1	EVIGITSMLNLHAGSQKVHEHGGGKPIQGSNFHFFAVGGDPLEMQGVLMNYRTKYPDGTV	178
QTJ14964.1	EVIGITSMLNLHAGSQKVHEHGGGKPIQGSNFHFFAVGGDPLEMQGVLMNYRTKYPDGTV	178
QTJ15036.1	EVIGITSMLNLHAGSQKVHEHGGGKPIQGSNFHFFAVGGDPLEMQGVLMNYRTKYPDGTV	178
QTJ15066.1	EVIGITSMLNLHAGSQKVHEHGGGKPIQGSNFHFFAVGGDPLEMQGVLMNYRTKYPDGTV	178
QTJ15078.1	EVIGITSMLNLHAGSQKVHEHGGGKPIQGSNFHFFAVGGDPLEMQGVLMNYRTKYPDGTV	178
QTJ15096.1	EVIGITSMLNLHAGSQKVHEHGGGKPIQGSNFHFFAVGGDPLEMQGVLMNYRTKYPDGTV	178
AQM40276.1	EVIGITSMLNLHAGSQKVHEHGGGKPIQGSNFHFFAVGGDPLEMQGVLMNYRTKYPDGTV	178
ABI94689.1	EVIGITSMLNLHAGSQKVHEHGGGKPIQGSNFHFFAVGGDPLEMQGVLMNYRTKYPDGTV	178
ABI94671.1	EVIGITSMLNLHAGSQKVHEHGGGKPIQGSNFHFFAVGGDPLEMQGVLMNYRTKYPDGTV	178
AYO46688.1	EVIGITSMLNLHAGSQKVHEHGGGKPIQGSNFHFFAVGGDPLEMQGVLMNYRTKYPDGTI	178
QTJ15060.1	EVIGITSMLNLHAGSQKVHEHGGGKPIQGSNFHFFAVGGDPLEMQGVLMNYRTKYPDGTI	178
AFQ31642.1	EVIGITSMLNLHAGSQKVHEHGGGKPIQGSNFHFFAVGGDPLEMQGVLMNYRTKYPDGTI	178
AYO46616.1	EVIGITSMLNLHAGSQKVHEHGGGKPIQGSNFHFFAVGGDPLEMQGVLMNYRTKYPDGTI	178
CAA79594.1	EVIGITSMLNLHAGSQKVHEHGGGKPIQGSNFHFFAVGGDPLEMQGVLMNYRTKYPDGTI	178
WLD47818.1	EVIGITSMLNLHAGSQKVHEHGGGKPIQGSNFHFFAVGGDPLEMQGVLMNYRTKYPDGTI	178
UTM62155.1	EVIGITSMLNLHAGSQKVHEHGGGKPIQGSNFHFFAVGGDPLEMQGVLMNYRTKYPDGTI	178

AFA41907.1	EVIGITSMLNLHAGSQKVHEHGGGKPIQGSNFHFFAVGGDPLEMQGVLMNYRTKYPDGTI	178
WLD47944.1	EVIGITSMLNLHAGSQKVHEHGGGKPIQGSNFHFFAVGGDPLEMQGVLMNYRTKYPDGTI	178
AYO46668.1	EVIGITSMLNLHAGSQKVHEHGGGKPIQGSNFHFFAVGGDPLEMQGVLMNYRTKYPDGTI	178
AFQ31651.1	EVIGITSMLNLHAGSQKVHEHGGGKPIQGSNFHFFAVGGDPLEMQGVLMNYRTKYPDGTI	178
QTJ14958.1	EVIGITSMLNLHAGSQKVHEHGGGKPIQGSNFHFFAVGGDPLEMQGVLMNYRTKYPDGTI	178
AYO46618.1	EVIGITSMLNLHAGSQKVHEHGGGKPIQGSNFHFFAVGGDPLEMQGVLMNYRTKYPDGTI	178
QTJ15018.1	EVIGITSMLNLHAGSQKVHEHGGGKPIQGSNFHFFAVGGDPLEMQGVLMNYRTKYPDGTI	178
AYO46609.1	EVIGITSMLNLHAGSQKVHEHGGGKPIQGSNFHFFAVGGDPLEMQGVLMNYRTKYPDGTI	178
ALF06356.1	EVIGITSMLNLHAGSQKVHEHGGGKPIQGSNFHFFAVGGDPLEMQGVLMNYRTKYPDGTI	178
AFQ31646.1	EVIGITSMLNLHAGSQKVHEHGGGKPIQGSNFHFFAVGGDPLEMQGVLMNYRTKYPDGTI	178
AFQ31659.1	EVIGITSMLNLHAGSQKVHEHGGGKPIQGSNFHFFAVGGDPLEMQGVLMNYRTKYPDGTI	178
BAF02957.1	EVIGITSMLNLHAGSQKVHEHGGGKPIQGSNFHFFAVGGDPLEMQGVLMNYRTKYPDGTI	178
AAT47365.1	EVIGITSMLNLHAGSQKVHEHGGGKPIQGSNFHFFAVGGDPLEMQGVLMNYRTKYPDGTI	178
AAT47389.1	EVIGITSMLNLHAGSQKVHEHGGGKPIQGSNFHFFAVGGDPLEMQGVLMNYRTKYPDGTI	178
AAT47395.1	EVIGITSMPNLHAGSQKVHEHGGGKPIQGSNFHFFAVGGDPLEMQGVLMNYRTKYPDGTI	178
AAT47401.1	EVIGITSMLNLHAGSQKVHEHGGGKPIQGSNFHFFAVGGDPLEMQGVLMNYRTKYPDGTI	178
CAA24307.1	EVIGITSMLNLHAGSQKVHEHGGGKPIQGSNFHFFAVGGDPLEMQGVLMNYRTKYPDGTI	178
AAT47425.1	EVIGITSMLNLHAGSQKVHEHGGGKPIQGSNFHFFAVGGDPLEMQGVLMNYRTKYPEGTI	178
AFA41880.1	EVIGITSMLNLHAGSQKVHEHGGGKPIQGSNFHFFAVGGDPLEMQGVLMNYRTKYPEGTI	178
WLD47968.1	EVIGITSMLNLHAGSQKVHENGGGKPIQGSNFHFFAVGGDPLEMQGVLMNYRTKYPEGTI	178
AFA41920.1	EVIGITSMLNLHAGSQKVHENGGGKPIQGSNFHFFAVGGDPLEMQGVLMNYRTKYPEGTI	178
CBX88302.1	EVIGITSMLNLHAGSQKVHEHGGGKPIQGSNFHFFAVGGDPLEMQGVLMNYRTKYPEGTI	178
AUF70036.1	EVIGITSMLNLHAGSQKVHEHGGGKPIQGSNFHFFAVGGDPLEMQGVLMNYRTKYPEGTI	178
ALF06323.1	EVIGITSMLNLHAGSQKVHEHGGGKPIQGSNFHFFAVGGDPLEMQGVLMNYRTKYPEGTI	178
WLD47890.1	EVIGITSMLNLHAGSQKVHEHGGGKPIQGSNFHFFAVGGDPLEMQGVLMNYRTKYPEGTI	178
BAF03097.1	EVIGITSMLNLHAGSQKVHEHGGGKPIQGSNFHFFAVGGDPLEMQGVLMNYRTKYPEGTI	178
ABI94695.1	EVIGITSMLNLHAGSQKVHEHGGGKPIQGSNFHFFAVGGDPLEMQGVLMNYRTKYPEGTI	178
ABI94623.1	EVIGITSMLNLHAGSQKVHEHGGGKPIQGSNFHFFAVGGDPLEMQGVLMNYRTKYPEGTI	178
UTM62533.1	EVIGITSMLNLHAGSQKVHEHGGGKPIQGSNFHFFAVGGDPLEMQGVLMNYRTKYPEGTI	178
QOS45058.1	EVIGITSMLNLHAGSQKVHEHGGGKPIQGSNFHFFAVGGDPLEMQGVLMNYRTKYPEGTI	178
AHU87362.1	EVIGITSMLNLHAGSQKVHEHGGGKPIQGSNFHFFAVGGDPLEMQGVLMNYRTKYPEGTI	178
AHU87363.1	EVIGITSMLNLHAGSQKVHEHGGGKPIQGSNFHFFAVGGDPLEMQGVLMNYRTKYPEGTI	178
AFQ31658.1	EVIGITSMLNLHAGSQKVHEHGGGKPIQGSNFHFFAVGGDPLEMQGVLMNYRTKYPEGTI	178
AFA41876.1	EVIGITSMLNLHAGSQKVHEHGGGKPIQGSNFHFFAVGGDPLEMQGVLMNYRTKYPEGTI	178
AFA41906.1	EVIGITSMLNLHAGSQKVHEHGGGKPIQGSNFHFFAVGGDPLEMQGVLMNYRTKYPEGTI	178
AEO89604.1	EVIGITSMLNLHAGSQKVHEHGGGKPIQGSNFHFFAVGGDPLEMQGVLMNYRTKYPEGTI	178
CBX88350.1	EVIGITSMLNLHAGSQKVHEHGGGKPIQGSNFHFFAVGGDPLEMQGVLMNYRTKYPEGTI	178
BAG84446.1	EVIGITSMLNLHAGSQKVHEHGGGKPIQGSNFHFFAVGGDPLEMQGVLMNYRTKYPEGTI	178
BAG84464.1	EVIGITSMLNLHAGSQKVHEHGGGKPIQGSNFHFFAVGGDPLEMQGVLMNYRTKYPEGTI	178
BAF93295.1	EVIGITSMLNLHAGSQKVHEHGGGKPIQGSNFHFFAVGGDPLEMQGVLMNYRTKYPEGTI	178
BAF93373.1	EVIGITSMLNLHAGSQKVHEHGGGKPIQGSNFHFFAVGGDPLEMQGVLMNYRTKYPEGTI	178
BAF93379.1	EVIGITSMLNLHAGSQKVHEHGGGKPIQGSNFHFFAVGGDPLEMQGVLMNYRTKYPEGTI	178
BAF42913.1	EVIGITSMLNLHAGSQKVHEHGGGKPIQGSNFHFFAVGGDPLEMQGVLMNYRTKYPEGTI	178
BAF42931.1	EVIGITSMLNLHAGSQKVHEHGGGKPIQGSNFHFFAVGGDPLEMQGVLMNYRTKYPEGTI	178
BAF43015.1	EVIGITSMLNLHAGSQKVHEHGGGKPIQGSNFHFFAVGGDPLEMQGVLMNYRTKYPEGTI	178
ABI94629.1	EVIGITSMLNLHAGSQKVHEHGGGKPIQGSNFHFFAVGGDPLEMQGVLMNYRTKYPEGTI	178
ABI94653.1	EVIGITSMLNLHAGSQKVHEHGGGKPIQGSNFHFFAVGGDPLEMQGVLMNYRTKYPEGTI	178
ABI94707.1	EVIGITSMLNLHAGSQKVHEHGGGKPIQGSNFHFFAVGGDPLEMQGVLMNYRTKYPEGTI	178
BAF03085.1	EVIGITSMLNLHAGSQKVHEHGGGKPIQGSNFHFFAVGGDPLEMQGVLMNYRTKYPEGTI	178
BAF03091.1	EVIGITSMLNLHAGSQKVHEHGGGKPIQGSNFHFFAVGGDPLEMQGVLMNYRTKYPEGTI	178
BAF03103.1	EVIGITSMLNLHAGSQKVHEHGGGKPIQGSNFHFFAVGGDPLEMQGVLMNYRTKYPEGTI	178
BAF03109.1	EVIGITSMLNLHAGSQKVHEHGGGKPIQGSNFHFFAVGGDPLEMQGVLMNYRTKYPEGTI	178
BAF93325.1	EVIGITSMLNLHAGSQKVHEHGGGKPIQGSNFHFFAVGGDPLEMQGVLMNYRTKYPEGTI	178
QTJ14934.1	EVIGITSMLNLHAGSQKVHEHGGGKPIQGSNFHFFAVGGNPLEMQGVLMNYRTKYPEGTI	178
QTJ14994.1	EVIGITSMLNLHAGSQKVHEHGGGKPIQGSNFHFFAVGGDPLEMQGVLMNYRTKYPEGTI	178
AFA41886.1	EVIGITSMLNLHAGSQKVHEHGGGKPIQGSNFHFFAVGGDPLEMQGVLMNYRTKYPEGTI	178
AFQ31650.1	EVIGITSMLNLHAGSQKVHEHGGGKPIQGSNFHFFAVGGDPLEMQGVLMNYRTKYPEGTI	178
AAT47347.1	EVIGITSMLNLHAGSQKVHEHGGGKPIQGSNFHFFAVGGDPLEMQGVLMNYRTKYPEGTI	178
ABI94617.1	EVIGITSMLNLHAGSQKVHEHGGGKPIQGSNFHFFAVGGDPLEMQGVLMNYRTKYPEGTI	178
BAF93319.1	EVIGITSMLNLHAGSQKVHEHGGGKPIQGSNFHFFAVGGDPLEMQGVLMNYRTKYPEGTI	178
CBX88314.1	EVIGITSMLNLHAGSKKVHEHGGGKPIQGSNFHFFAVGGDPLEMQGVLMNYRTKYPEGTI	178
ALF06320.1	EVIGITSMLNLHAGSQKVHEHGGGKPIQGSNFHFFAVGGDPLEMQGVLMNYRTKYPEGTI	178
WLD47830.1	EVIGITSMLNLHAGSQKVHEHGGGKPIQGSNFHFFAVGGDPLEMQGVLMNYRTKYPEGTI	178
WLD47866.1	EVIGITSMLNLHAGSQKVHEHGGGKPIQGSNFHFFAVGGDPLEMQGVLMNYRTKYPEGTI	178

UTM61939.1	EVIGITSMLNLHAGSQKVHEHGGGKPIQGSNFHFFAVGGDPLEMQGVLMNYRTKYPEGTI	178
UTM62023.1	EVIGITSMLNLHAGSQKVHEHGGGKPIQGSNFHFFAVGGDPLEMQGVLMNYRTKYPEGTI	178
UTM62029.1	EVIGITSMLNLHAGSQKVHEHGGGKPIQGSNFHFFAVGGDPLEMQGVLMNYRTKYPEGTI	178
UTM62053.1	EVIGITSMLNLHAGSQKVHEHGGGKPIQGSNFHFFAVGGDPLEMQGVLMNYRTKYPEGTI	178
UTM62059.1	EVIGITSMLNLHAGSQKVHEHGGGKPIQGSNFHFFAVGGDPLEMQGVLMNYRTKYPEGTI	178
UTM62065.1	EVIGITSMLNLHAGSQKVHEHGGGKPIQGSNFHFFAVGGDPLEMQGVLMNYRTKYPEGTI	178
UTM62317.1	EVIGITSMLNLHAGSQKVHEHGGGKPIQGSNFHFFAVGGDPLEMQGVLMNYRTKYPEGTI	178
UTM62323.1	EVIGITSMLNLHAGSQKVHEHGGGKPIQGSNFHFFAVGGDPLEMQGVLMNYRTKYPEGTI	178
UTM62353.1	EVIGITSMLNLHAGSQKVHEHGGGKPIQGSNFHFFAVGGDPLEMQGVLMNYRTKYPEGTI	178
UTM62473.1	EVIGITSMLNLHAGSQKVHEHGGGKPIQGSNFHFFAVGGDPLEMQGVLMNYRTKYPEGTI	178
UTM62491.1	EVIGITSMLNLHAGSQKVHEHGGGKPIQGSNFHFFAVGGDPLEMQGVLMNYRTKYPEGTI	178
UTM62497.1	EVIGITSMLNLHAGSQKVHEHGGGKPIQGSNFHFFAVGGDPLEMQGVLMNYRTKYPEGTI	178
UTM62503.1	EVIGITSMLNLHAGSQKVHEHGGGKPIQGSNFHFFAVGGDPLEMQGVLMNYRTKYPEGTI	178
QTJ14988.1	EVIGITSMLNLHAGSQKVHEHGGGKPIQGSNFHFFAVGGDPLEMQGVLMNYRTKYPEGTI	178
QTJ15024.1	EVIGITSMLNLHAGSQKVHEHGGGKPIQGSNFHFFAVGGDPLEMQGVLMNYRTKYPEGTI	178
QTJ15030.1	EVIGITSMLNLHAGSQKVHEHGGGKPIQGSNFHFFAVGGDPLEMQGVLMNYRTKYPEGTI	178
QTJ15102.1	EVIGITSMLNLHAGSQKVHEHGGGKPIQGSNFHFFAVGGDPLEMQGVLMNYRTKYPEGTI	178
ALF06300.1	EVIGITSMLNLHAGSQKVHEHGGGKPIQGSNFHFFAVGGDPLEMQGVLMNYRTKYPEGTI	178
AHU87342.1	EVIGITSMLNLHAGSQKVHEHGGGKPIQGSNFHFFAVGGDPLEMQGVLMNYRTKYPEGTI	178
AHU87343.1	EVIGITSMLNLHAGSQKVHEHGGGKPIQGSNFHFFAVGGDPLEMQGVLMNYRTKYPEGTI	178
AHU87346.1	EVIGITSMLNLHAGSQKVHEHGGGKPIQGSNFHFFAVGGDPLEMQGVLMNYRTKYPEGTI	178
AHU87347.1	EVIGITSMLNLHAGSQKVHEHGGGKPIQGSNFHFFAVGGDPLEMQGVLMNYRTKYPEGTI	178
AHU87349.1	EVIGITSMLNLHAGSQKVHEHGGGKPIQGSNFHFFAVGGDPLEMQGVLMNYRTKYPEGTI	178
AHU87356.1	EVIGITSMLNLHAGSQKVHEHGGGKPIQGSNFHFFAVGGDPLEMQGVLMNYRTKYPEGTI	178
AHU87357.1	EVIGITSMLNLHAGSQKVHEHGGGKPIQGSNFHFFAVGGDPLEMQGVLMNYRTKYPEGTI	178
AHU87358.1	EVIGITSMLNLHAGSQKVHEHGGGKPIQGSNFHFFAVGGDPLEMQGVLMNYRTKYPEGTI	178
AHU87359.1	EVIGITSMLNLHAGSQKVHEHGGGKPIQGSNFHFFAVGGDPLEMQGVLMNYRTKYPEGTI	178
AFQ31643.1	EVIGITSMLNLHAGSQKVHEHGGGKPIQGSNFHFFAVGGDPLEMQGVLMNYRTKYPEGTI	178
AFQ31656.1	EVIGITSMLNLHAGSQKVHEHGGGKPIQGSNFHFFAVGGDPLEMQGVLMNYRTKYPEGTI	178
AFA41879.1	EVIGITSMLNLHAGSQKVHEHGGGKPIQGSNFHFFAVGGDPLEMQGVLMNYRTKYPEGTI	178
AFA41897.1	EVIGITSMLNLHAGSQKVHEHGGGKPIQGSNFHFFAVGGDPLEMQGVLMNYRTKYPEGTI	178
AFA41900.1	EVIGITSMLNLHAGSQKVHEHGGGKPIQGSNFHFFAVGGDPLEMQGVLMNYRTKYPEGTI	178
AEO89580.1	EVIGITSMLNLHAGSQKVHEHGGGKPIQGSNFHFFAVGGDPLEMQGVLMNYRTKYPEGTI	178
AEO89592.1	EVIGITSMLNLHAGSQKVHEHGGGKPIQGSNFHFFAVGGDPLEMQGVLMNYRTKYPEGTI	178
BAF93277.1	EVIGITSMLNLHAGSQKVHEHGGGKPIQGSNFHFFAVGGDPLEMQGVLMNYRTKYPEGTI	178
BAF93289.1	EVIGITSMLNLHAGSQKVHEHGGGKPIQGSNFHFFAVGGDPLEMQGVLMNYRTKYPEGTI	178
BAF93307.1	EVIGITSMLNLHAGSQKVHEHGGGKPIQGSNFHFFAVGGDPLEMQGVLMNYRTKYPEGTI	178
BAF93337.1	EVIGITSMLNLHAGSQKVHEHGGGKPIQGSNFHFFAVGGDPLEMQGVLMNYRTKYPEGTI	178
BAF42895.1	EVIGITSMLNLHAGSQKVHEHGGGKPIQGSNFHFFAVGGDPLEMQGVLMNYRTKYPEGTI	178
BAF42919.1	EVIGITSMLNLHAGSQKVHEHGGGKPIQGSNFHFFAVGGDPLEMQGVLMNYRTKYPEGTI	178
BAF42943.1	EVIGITSMLNLHAGSQKVHEHGGGKPIQGSNFHFFAVGGDPLEMQGVLMNYRTKYPEGTI	178
BAF42949.1	EVIGITSMLNLHAGSQKVHEHGGGKPIQGSNFHFFAVGGDPLEMQGVLMNYRTKYPEGTI	178
ABI94665.1	EVIGITSMLNLHAGSQKVHEHGGGKPIQGSNFHFFAVGGDPLEMQGVLMNYRTKYPEGTI	178
ABI94677.1	EVIGITSMLNLHAGSQKVHEHGGGKPIQGSNFHFFAVGGDPLEMQGVLMNYRTKYPEGTI	178
BAF02939.1	EVIGITSMLNLHAGSQKVHEHGGGKPIQGSNFHFFAVGGDPLEMQGVLMNYRTKYPEGTI	178
AAT47353.1	EVIGITSMLNLHAGSQKVHEHGGGKPIQGSNFHFFAVGGDPLEMQGVLMNYRTKYPEGTI	178
AAT47359.1	EVIGITSMLNLHAGSQKVHEHGGGKPIQGSNFHFFAVGGDPLEMQGVLMNYRTKYPEGTI	178
CAA40247.1	EVIGITSMLNLHAGSQKVHEHGGGKPIQGSNFHFFAVGGDPLEMQGVLMNYRTKYPEGTI	178
QTJ14982.1	EVIGITSMLNLHAGSQKVHEHGGGKPIQGSNFHFFAVGGDPLEMQGVLMNYRTKYPEGTI	178
QTJ15126.1	EVIGITSMLNLHAGSQKVHEHGGGKPIQGSNFHFFAVGGDPLEMQGVLMNYRTKYPEGTI	178
ABI94635.1	EVIGITSMLNLHAGSQKVHEHGGGKPIQGSNFHFFAVGGDPLEMQGVLMNYRTKYPEGTI	178
ALF06335.1	EVIGITSMLNLHAGSQKVHEHGGGKPIQGSNFHFFAVGGDPLEMQGVLMNYRTKYPEGTI	178
AHU87348.1	EVIGITSMLNLHAGSQKVHEHGGGKPIQGSNFHFFAVGGDPLEMQGVLMNYRTKYPEGTI	178
AFQ31649.1	EVIGITSMLNLHAGSQKVHEHGGGKPIQGSNFHFFAVGGDPLEMQGVLMNYRTKYPEGTI	178
AFQ31654.1	EVIGITSMLNLHAGSQKVHEHGGGKPIQGSNFHFFAVGGDPLEMQGVLMNYRTKYPEGTI	178
BAF42907.1	EVIGITSMLNLHAGSQKVHEHGGGKPIQGSNFHFFAVGGDPLEMQGVLMNYRTKYPEGTI	178
WLD47824.1	EVIGITSMLNLHAGSQKVHEHGGGKPIQGSNFHFFAVGGDPLEMQGVLMNYRTKYPEGTI	178
WLD47848.1	EVIGITSMLNLHAGSQKVHEHGGGKPIQGSNFHFFAVGGDPLEMQGVLMNYRTKYPEGTI	178
WLD47854.1	EVIGITSMLNLHAGSQKVHEHGGGKPIQGSNFHFFAVGGDPLEMQGVLMNYRTKYPEGTI	178
WLD47860.1	EVIGITSMLNLHAGSQKVHEHGGGKPIQGSNFHFFAVGGDPLEMQGVLMNYRTKYPEGTI	178
WLD47878.1	EVIGITSMLNLHAGSQKVHEHGGGKPIQGSNFHFFAVGGDPLEMQGVLMNYRTKYPEGTI	178
WLD47908.1	EVIGITSMLNLHAGSQKVHEHGGGKPIQGSNFHFFAVGGDPLEMQGVLMNYRTKYPEGTI	178
WLD47914.1	EVIGITSMLNLHAGSQKVHEHGGGKPIQGSNFHFFAVGGDPLEMQGVLMNYRTKYPEGTI	178
WLD47920.1	EVIGITSMLNLHAGSQKVHEHGGGKPIQGSNFHFFAVGGDPLEMQGVLMNYRTKYPEGTI	178

```
WLD47926.1     EVIGITSMLNLHAGSQKVHEHGGGKPIQGSNFHFFAVGGDPLEMQGVLMNYRTKYPEGTI  178
WLD47932.1     EVIGITSMLNLHAGSQKVHEHGGGKPIQGSNFHFFAVGGDPLEMQGVLMNYRTKYPEGTI  178
WLD47950.1     EVIGITSMLNLHAGSQKVHEHGGGKPIQGSNFHFFAVGGDPLEMQGVLMNYRTKYPEGTI  178
WLD47962.1     EVIGITSMLNLHAGSQKVHEHGGGKPIQGSNFHFFAVGGDPLEMQGVLMNYRTKYPEGTI  178
WLD47974.1     EVIGITSMLNLHAGSQKVHEHGGGKPIQGSNFHFFAVGGDPLEMQGVLMNYRTKYPEGTI  178
WLD47998.1     EVIGITSMLNLHAGSQKVHEHGGGKPIQGSNFHFFAVGGDPLEMQGVLMNYRTKYPEGTI  178
WLD48004.1     EVIGITSMLNLHAGSQKVHEHGGGKPIQGSNFHFFAVGGDPLEMQGVLMNYRTKYPEGTI  178
WLD48016.1     EVIGITSMLNLHAGSQKVHEHGGGKPIQGSNFHFFAVGGDPLEMQGVLMNYRTKYPEGTI  178
WLD48022.1     EVIGITSMLNLHAGSQKVHEHGGGKPIQGSNFHFFAVGGDPLEMQGVLMNYRTKYPEGTI  178
WLD48034.1     EVIGITSMLNLHAGSQKVHEHGGGKPIQGSNFHFFAVGGDPLEMQGVLMNYRTKYPEGTI  178
UTM61909.1     EVIGITSMLNLHAGSQKVHEHGGGKPIQGSNFHFFAVGGDPLEMQGVLMNYRTKYPEGTI  178
UTM61915.1     EVIGITSMLNLHAGSQKVHEHGGGKPIQGSNFHFFAVGGDPLEMQGVLMNYRTKYPEGTI  178
UTM61921.1     EVIGITSMLNLHAGSQKVHEHGGGKPIQGSNFHFFAVGGDPLEMQGVLMNYRTKYPEGTI  178
UTM61927.1     EVIGITSMLNLHAGSQKVHEHGGGKPIQGSNFHFFAVGGDPLEMQGVLMNYRTKYPEGTI  178
UTM61933.1     EVIGITSMLNLHAGSQKVHEHGGGKPIQGSNFHFFAVGGDPLEMQGVLMNYRTKYPEGTI  178
UTM61963.1     EVIGITSMLNLHAGSQKVHEHGGGKPIQGSNFHFFAVGGDPLEMQGVLMNYRTKYPEGTI  178
UTM61969.1     EVIGITSMLNLHAGSQKVHEHGGGKPIQGSNFHFFAVGGDPLEMQGVLMNYRTKYPEGTI  178
UTM61975.1     EVIGITSMLNLHAGSQKVHEHGGGKPIQGSNFHFFAVGGDPLEMQGVLMNYRTKYPEGTI  178
UTM61981.1     EVIGITSMLNLHAGSQKVHEHGGGKPIQGSNFHFFAVGGDPLEMQGVLMNYRTKYPEGTI  178
UTM62005.1     EVIGITSMLNLHAGSQKVHEHGGGKPIQGSNFHFFAVGGDPLEMQGVLMNYRTKYPEGTI  178
UTM62017.1     EVIGITSMLNLHAGSQKVHEHGGGKPIQGSNFHFFAVGGDPLEMQGVLMNYRTKYPEGTI  178
UTM62035.1     EVIGITSMLNLHAGSQKVHEHGGGKPIQGSNFHFFAVGGDPLEMQGVLMNYRTKYPEGTI  178
UTM62041.1     EVIGITSMLNLHAGSQKVHEHGGGKPIQGSNFHFFAVGGDPLEMQGVLMNYRTKYPEGTI  178
UTM62047.1     EVIGITSMLNLHAGSQKVHEHGGGKPIQGSNFHFFAVGGDPLEMQGVLMNYRTKYPEGTI  178
UTM62083.1     EVIGITSMLNLHAGSQKVHEHGGGKPIQGSNFHFFAVGGDPLEMQGVLMNYRTKYPEGTI  178
UTM62089.1     EVIGITSMLNLHAGSQKVHEHGGGKPIQGSNFHFFAVGGDPLEMQGVLMNYRTKYPEGTI  178
UTM62095.1     EVIGITSMLNLHAGSQKVHEHGGGKPIQGSNFHFFAVGGDPLEMQGVLMNYRTKYPEGTI  178
UTM62149.1     EVIGITSMLNLHAGSQKVHEHGGGKPIQGSNFHFFAVGGDPLEMQGVLMNYRTKYPEGTI  178
UTM62161.1     EVIGITSMLNLHAGSQKVHEHGGGKPIQGSNFHFFAVGGDPLEMQGVLMNYRTKYPEGTI  178
UTM62167.1     EVIGITSMLNLHAGSQKVHEHGGGKPIQGSNFHFFAVGGDPLEMQGVLMNYRTKYPEGTI  178
UTM62173.1     EVIGITSMLNLHAGSQKVHEHGGGKPIQGSNFHFFAVGGDPLEMQGVLMNYRTKYPEGTI  178
UTM62179.1     EVIGITSMLNLHAGSQKVHEHGGGKPIQGSNFHFFAVGGDPLEMQGVLMNYRTKYPEGTI  178
UTM62185.1     EVIGITSMLNLHAGSQKVHEHGGGKPIQGSNFHFFAVGGDPLEMQGVLMNYRTKYPEGTI  178
UTM62191.1     EVIGITSMLNLHAGSQKVHEHGGGKPIQGSNFHFFAVGGDPLEMQGVLMNYRTKYPEGTI  178
UTM62197.1     EVIGITSMLNLHAGSQKVHEHGGGKPIQGSNFHFFAVGGDPLEMQGVLMNYRTKYPEGTI  178
UTM62203.1     EVIGITSMLNLHAGSQKVHEHGGGKPIQGSNFHFFAVGGDPLEMQGVLMNYRTKYPEGTI  178
UTM62209.1     EVIGITSMLNLHAGSQKVHEHGGGKPIQGSNFHFFAVGGDPLEMQGVLMNYRTKYPEGTI  178
UTM62227.1     EVIGITSMLNLHAGSQKVHEHGGGKPIQGSNFHFFAVGGDPLEMQGVLMNYRTKYPEGTI  178
UTM62239.1     EVIGITSMLNLHAGSQKVHEHGGGKPIQGSNFHFFAVGGDPLEMQGVLMNYRTKYPEGTI  178
UTM62245.1     EVIGITSMLNLHAGSQKVHEHGGGKPIQGSNFHFFAVGGDPLEMQGVLMNYRTKYPEGTI  178
UTM62251.1     EVIGITSMLNLHAGSQKVHEHGGGKPIQGSNFHFFAVGGDPLEMQGVLMNYRTKYPEGTI  178
UTM62257.1     EVIGITSMLNLHAGSQKVHEHGGGKPIQGSNFHFFAVGGDPLEMQGVLMNYRTKYPEGTI  178
UTM62263.1     EVIGITSMLNLHAGSQKVHEHGGGKPIQGSNFHFFAVGGDPLEMQGVLMNYRTKYPEGTI  178
UTM62269.1     EVIGITSMLNLHAGSQKVHEHGGGKPIQGSNFHFFAVGGDPLEMQGVLMNYRTKYPEGTI  178
UTM62287.1     EVIGITSMLNLHAGSQKVHEHGGGKPIQGSNFHFFAVGGDPLEMQGVLMNYRTKYPEGTI  178
UTM62293.1     EVIGITSMLNLHAGSQKVHEHGGGKPIQGSNFHFFAVGGDPLEMQGVLMNYRTKYPEGTI  178
UTM62299.1     EVIGITSMLNLHAGSQKVHEHGGGKPIQGSNFHFFAVGGDPLEMQGVLMNYRTKYPEGTI  178
UTM62305.1     EVIGITSMLNLHAGSQKVHEHGGGKPIQGSNFHFFAVGGDPLEMQGVLMNYRTKYPEGTI  178
UTM62311.1     EVIGITSMLNLHAGSQKVHEHGGGKPIQGSNFHFFAVGGDPLEMQGVLMNYRTKYPEGTI  178
UTM62359.1     EVIGITSMLNLHAGSQKVHEHGGGKPIQGSNFHFFAVGGDPLEMQGVLMNYRTKYPEGTI  178
UTM62365.1     EVIGITSMLNLHAGSQKVHEHGGGKPIQGSNFHFFAVGGDPLEMQGVLMNYRTKYPEGTI  178
UTM62371.1     EVIGITSMLNLHAGSQKVHEHGGGKPIQGSNFHFFAVGGDPLEMQGVLMNYRTKYPEGTI  178
UTM62377.1     EVIGITSMLNLHAGSQKVHEHGGGKPIQGSNFHFFAVGGDPLEMQGVLMNYRTKYPEGTI  178
UTM62455.1     EVIGITSMLNLHAGSQKVHEHGGGKPIQGSNFHFFAVGGDPLEMQGVLMNYRTKYPEGTI  178
UTM62461.1     EVIGITSMLNLHAGSQKVHEHGGGKPIQGSNFHFFAVGGDPLEMQGVLMNYRTKYPEGTI  178
UTM62485.1     EVIGITSMLNLHAGSQKVHEHGGGKPIQGSNFHFFAVGGDPLEMQGVLMNYRTKYPEGTI  178
UTM62509.1     EVIGITSMLNLHAGSQKVHEHGGGKPIQGSNFHFFAVGGDPLEMQGVLMNYRTKYPEGTI  178
UTM62515.1     EVIGITSMLNLHAGSQKVHEHGGGKPIQGSNFHFFAVGGDPLEMQGVLMNYRTKYPEGTI  178
UTM62521.1     EVIGITSMLNLHAGSQKVHEHGGGKPIQGSNFHFFAVGGDPLEMQGVLMNYRTKYPEGTI  178
UTM62527.1     EVIGITSMLNLHAGSQKVHEHGGGKPIQGSNFHFFAVGGDPLEMQGVLMNYRTKYPEGTI  178
UTM62539.1     EVIGITSMLNLHAGSQKVHEHGGGKPIQGSNFHFFAVGGDPLEMQGVLMNYRTKYPEGTI  178
UTM62557.1     EVIGITSMLNLHAGSQKVHEHGGGKPIQGSNFHFFAVGGDPLEMQGVLMNYRTKYPEGTI  178
UTM62563.1     EVIGITSMLNLHAGSQKVHEHGGGKPIQGSNFHFFAVGGDPLEMQGVLMNYRTKYPEGTI  178
UTM62569.1     EVIGITSMLNLHAGSQKVHEHGGGKPIQGSNFHFFAVGGDPLEMQGVLMNYRTKYPEGTI  178
```

UTM62575.1	EVIGITSMLNLHAGSQKVHEHGGGKPIQGSNFHFFAVGGDPLEMQGVLMNYRTKYPEGTI	178
UTM62581.1	EVIGITSMLNLHAGSQKVHEHGGGKPIQGSNFHFFAVGGDPLEMQGVLMNYRTKYPEGTI	178
UTM62605.1	EVIGITSMLNLHAGSQKVHEHGGGKPIQGSNFHFFAVGGDPLEMQGVLMNYRTKYPEGTI	178
UTM62617.1	EVIGITSMLNLHAGSQKVHEHGGGKPIQGSNFHFFAVGGDPLEMQGVLMNYRTKYPEGTI	178
UTM62623.1	EVIGITSMLNLHAGSQKVHEHGGGKPIQGSNFHFFAVGGDPLEMQGVLMNYRTKYPEGTI	178
UTM62629.1	EVIGITSMLNLHAGSQKVHEHGGGKPIQGSNFHFFAVGGDPLEMQGVLMNYRTKYPEGTI	178
UTM62635.1	EVIGITSMLNLHAGSQKVHEHGGGKPIQGSNFHFFAVGGDPLEMQGVLMNYRTKYPEGTI	178
QTJ15048.1	EVIGITSMLNLHAGSQKVHEHGGGKPIQGSNFHFFAVGGDPLEMQGVLMNYRTKYPEGTI	178
QTJ15144.1	EVIGITSMLNLHAGSQKVHEHGGGKPIQGSNFHFFAVGGDPLEMQGVLMNYRTKYPEGTI	178
AHU87360.1	EVIGITSMLNLHAGSQKVHEHGGGKPIQGSNFHFFAVGGDPLEMQGVLMNYRTKYPEGTI	178
AHU87361.1	EVIGITSMLNLHAGSQKVHEHGGGKPIQGSNFHFFAVGGDPLEMQGVLMNYRTKYPEGTI	178
AHU87366.1	EVIGITSMLNLHAGSQKVHEHGGGKPIQGSNFHFFAVGGDPLEMQGVLMNYRTKYPEGTI	178
AHU87367.1	EVIGITSMLNLHAGSQKVHEHGGGKPIQGSNFHFFAVGGDPLEMQGVLMNYRTKYPEGTI	178
AFQ31652.1	EVIGITSMLNLHAGSQKVHEHGGGKPIQGSNFHFFAVGGDPLEMQGVLMNYRTKYPEGTI	178
AFA41888.1	EVIGITSMLNLHAGSQKVHEHGGGKPIQGSNFHFFAVGGDPLEMQGVLMNYRTKYPEGTI	178
AFA41893.1	EVIGITSMLNLHAGSQKVHEHGGGKPIQGSNFHFFAVGGDPLEMQGVLMNYRTKYPEGTI	178
AFA41894.1	EVIGITSMLNLHAGSQKVHEHGGGKPIQGSNFHFFAVGGDPLEMQGVLMNYRTKYPEGTI	178
AFA41899.1	EVIGITSMLNLHAGSQKVHEHGGGKPIQGSNFHFFAVGGDPLEMQGVLMNYRTKYPEGTI	178
AFA41904.1	EVIGITSMLNLHAGSQKVHEHGGGKPIQGSNFHFFAVGGDPLEMQGVLMNYRTKYPEGTI	178
AFA41905.1	EVIGITSMLNLHAGSQKVHEHGGGKPIQGSNFHFFAVGGDPLEMQGVLMNYRTKYPEGTI	178
AFA41908.1	EVIGITSMLNLHAGSQKVHEHGGGKPIQGSNFHFFAVGGDPLEMQGVLMNYRTKYPEGTI	178
AFA41911.1	EVIGITSMLNLHAGSQKVHEHGGGKPIQGSNFHFFAVGGDPLEMQGVLMNYRTKYPEGTI	178
AFA41912.1	EVIGITSMLNLHAGSQKVHEHGGGKPIQGSNFHFFAVGGDPLEMQGVLMNYRTKYPEGTI	178
AFA41913.1	EVIGITSMLNLHAGSQKVHEHGGGKPIQGSNFHFFAVGGDPLEMQGVLMNYRTKYPEGTI	178
AFA41915.1	EVIGITSMLNLHAGSQKVHEHGGGKPIQGSNFHFFAVGGDPLEMQGVLMNYRTKYPEGTI	178
AFA41916.1	EVIGITSMLNLHAGSQKVHEHGGGKPIQGSNFHFFAVGGDPLEMQGVLMNYRTKYPEGTI	178
AFA41919.1	EVIGITSMLNLHAGSQKVHEHGGGKPIQGSNFHFFAVGGDPLEMQGVLMNYRTKYPEGTI	178
AFA41921.1	EVIGITSMLNLHAGSQKVHEHGGGKPIQGSNFHFFAVGGDPLEMQGVLMNYRTKYPEGTI	178
AFA41922.1	EVIGITSMLNLHAGSQKVHEHGGGKPIQGSNFHFFAVGGDPLEMQGVLMNYRTKYPEGTI	178
AFA41923.1	EVIGITSMLNLHAGSQKVHEHGGGKPIQGSNFHFFAVGGDPLEMQGVLMNYRTKYPEGTI	178
AFA41924.1	EVIGITSMLNLHAGSQKVHEHGGGKPIQGSNFHFFAVGGDPLEMQGVLMNYRTKYPEGTI	178
AFA41925.1	EVIGITSMLNLHAGSQKVHEHGGGKPIQGSNFHFFAVGGDPLEMQGVLMNYRTKYPEGTI	178
CBX88272.1	EVIGITSMLNLHAGSQKVHEHGGGKPIQGSNFHFFAVGGDPLEMQGVLMNYRTKYPEGTI	178
CBX88278.1	EVIGITSMLNLHAGSQKVHEHGGGKPIQGSNFHFFAVGGDPLEMQGVLMNYRTKYPEGTI	178
CBX88284.1	EVIGITSMLNLHAGSQKVHEHGGGKPIQGSNFHFFAVGGDPLEMQGVLMNYRTKYPEGTI	178
CBX88290.1	EVIGITSMLNLHAGSQKVHEHGGGKPIQGSNFHFFAVGGDPLEMQGVLMNYRTKYPEGTI	178
CBX88296.1	EVIGITSMLNLHAGSQKVHEHGGGKPIQGSNFHFFAVGGDPLEMQGVLMNYRTKYPEGTI	178
CBX88308.1	EVIGITSMLNLHAGSQKVHEHGGGKPIQGSNFHFFAVGGDPLEMQGVLMNYRTKYPEGTI	178
CBX88332.1	EVIGITSMLNLHAGSQKVHEHGGGKPIQGSNFHFFAVGGDPLEMQGVLMNYRTKYPEGTI	178
CBX88356.1	EVIGITSMLNLHAGSQKVHEHGGGKPIQGSNFHFFAVGGDPLEMQGVLMNYRTKYPEGTI	178
CBX88362.1	EVIGITSMLNLHAGSQKVHEHGGGKPIQGSNFHFFAVGGDPLEMQGVLMNYRTKYPEGTI	178
BAF93313.1	EVIGITSMLNLHAGSQKVHEHGGGKPIQGSNFHFFAVGGDPLEMQGVLMNYRTKYPEGTI	178
BAF42955.1	EVIGITSMLNLHAGSQKVHEHGGGKPIQGSNFHFFAVGGDPLEMQGVLMNYRTKYPEGTI	178
BAF43021.1	EVIGITSMLNLHAGSQKVHEHGGGKPIQGSNFHFFAVGGDPLEMQGVLMNYRTKYPEGTI	178
ABI94611.1	EVIGITSMLNLHAGSQKVHEHGGGKPIQGSNFHFFAVGGDPLEMQGVLMNYRTKYPEGTI	178
ABI94641.1	EVIGITSMLNLHAGSQKVHEHGGGKPIQGSNFHFFAVGGDPLEMQGVLMNYRTKYPEGTI	178
ABI94647.1	EVIGITSMLNLHAGSQKVHEHGGGKPIQGSNFHFFAVGGDPLEMQGVLMNYRTKYPEGTI	178
WLD47896.1	EVIGITSMLNLHAGSQKVHEHGGGKPIQGSNFHFFAVGGDPLEMQGVLMNYRTKYPEGTI	178
WLD48028.1	EVIGITSMLNLHAGSQKVHEHGGGKPIQGSNFHFFAVGGDPLEMQGVLMNYRTKYPEGTI	178
BAG84440.1	EVIGITSMLNLHAGSQKVHEHGGGKPIQGSNFHFFAVGGDPLEMQGVLMNYRTKYPEGTI	178
BAF93283.1	EVIGITSMLNLHAGSQKVHEHGGGKPIQGSNFHFFAVGGDPLEMQGVLMNYRTKYPEGTI	178
BAF93361.1	EVIGITSMLNLHAGSQKVHEHGGGKPIQGSNFHFFAVGGDPLEMQGVLMNYRTKYPEGTI	178
WLD48040.1	EVIGITSMLNLHAGSQKVHEHGGGKPIQGSNFHFFAVGGDPLEMQGVLMNYRTKYPEGTI	178
UTM62611.1	EVIGITSMLNLHAGSQKVHEHGGGKPIQGSNFHFFAVGGDPLEMQGVLMNYRTKYPEGTI	178
AFQ31645.1	EVIGITSMLNLHAGSQKVHEHGGGKPIQGSNFHFFAVGGDPLEMQGVLMNYRTKYPEGTI	178
AFA41909.1	EVIGITSMLNLHAGSQKVHEHGGGKPIQGSNFHFFAVGGDPLEMQGVLMNYRTKYPEGTI	178
ABI94713.1	EVIGITSMLNLHAGSQKVHEHGGGKPIQGSNFHFFAVGGDPLEMQGVLMNYRTKYPEGTI	178
ABD04662.1	EVIGITSMLNLHAGSQKVHEHGGGKPIQGSNFHFFAVGGDPLEMQGVLMNYRTKYPEGTI	178
QTJ15042.1	EVIGITSMLNLHAGSQKVHDQGGGKPIQGSNFHFFAVGGDPLEMQGVLMNYKTKYPDGTV	178
AGX89705.1	EVIGITSMLNLHAGSQKVHENGGGKPVQGSNFHFFAVGGDPLEMQGVLMNYRTKYPQGTI	178
AGX89710.1	EVIGITSMLNLHAGSQKVHENGGGKPVQGSNFHFFAVGGDPLEMQGVLMNYRTKYPQGTI	178
AGX89717.1	EVIGITSMLNLHAGSQKVHENGGGKPVQGSNFHFFAVGGDPLEMQGVLMNYRTKYPQGTI	178
AFQ31655.1	EVIGITSMLNLHAGSQKVHENGGGKPVQGSNFHFFAVGGDPLEMQGVLMNYRTKYPQGTI	178
AAA46882.1	EVIGITSMLNLHAGSQKVHENGGGKPVQGSNFHFFAVGGDPLEMQGVLMNYRTKYPQGTI	178

```
P14996.1        EVIGITSMLNLHAGSQKVHENGGGKPVQGSNFHFFAVGGDPLEMQGVLMNYRTKYPQGTI  178
AEO89615.1      EVIGITSMLNLHAGSQKVHENGGGKPVQGSNFHFFAVGGDPLEMQGVLMNYRTKYPQGTI  178
BCV68929.1      EVIGITSMLNLHAGSQKVHENGGGKPVQGSNFHFFAVGGDPLEMQGVLMNYRTKYPQGTI  178
QTJ14952.1      EVIGITSMLNLHAGSQKVHENGGGKPVQGSNFHFFAVGGDPLEMQGVLMNYRTKYPQGTI  178
BAG75157.1      EVIGITSMLNLHAGSQKVHENGGGKPVQGSNFHFFAVGGDPLEMQGVLMNYRTKYPQGTI  178
BAG75211.1      EVIGITSMLNLHAGSQKVHENGGGKPVQGSNFHFFAVGGDPLEMQGVLMNYRTKYPQGTI  178
BAF03017.1      EVIGITSMLNLHAGSQKVHENGGGKPVQGSNFHFFAVGGDPLEMQGVLMNYRTKYPQGTI  178
BAF42925.1      EVIGITSMLNLHAGSQKVHENGGGKPVQGSNFHFFAVGGDPLEMQGVLMNYRTKYPQGTI  178
WLD47836.1      EVIGITSMLNLHAGSQKVHEKGGGKPVQGSNFHFFAVGGDPLEMQGVLMNYRTKYPQGTI  178
UEL36354.1      EVIGITSMLNLHAGSQKVHENGGGKPVQGSNFHFFAVGGDPLEMQGVLMNYRTKYPQGTI  178
BAF42901.1      EVIGITSMLNLHAGSQKVHENGGGKPVQGSNFHFFAVGGDPLEMQGVLMNYRTKYPQGTI  178
ABP87973.1      EVIGITSMLNLHAGSQKVHENGGGKPVQGSNFHFFAVGGDPLEMQGVLMNYRTKYPQGTI  178
AFA41878.1      EVIGITSMLNLHAGSXKVHENGGGKPVQGSNFHFFAVGGDPLEMQGVLMNYRTKYPQGTI  178
QHI34655.1      EVIGITSMLNLHAGSQKVHENGGGKPVQGSNFHFFAVGGDPLEMQGVLMNYRTKYPQGTI  178
UTM61945.1      EVIGITSMLNLHAGSQKVHQNGGGKPVQGSNFHFFAVGGDPLEMQGVLMNYRTKYPQGTI  178
UTM61987.1      EVIGITSMLNLHAGSQKVHENGGGKPVQGSNFHFFAVGGDPLEMQGVLMNYRTKYPQGTI  178
UTM62233.1      EVIGITSMLNLHAGSQKVHENGGGKPVQGSNFHFFAVGGDPLEMQGVLMNYRTKYPQGTI  178
UTM62341.1      EVIGITSMLNLHAGSQKVHENGGGKPVQGSNFHFFAVGGDPLEMQGVLMNYRTKYPQGTI  178
UTM62347.1      EVIGITSMLNLHAGSQKVHENGGGKPVQGSNFHFFAVGGDPLEMQGVLMNYRTKYPQGTI  178
AHU87354.1      EVIGITSMLNLHAGSQKVHENGGGKPVQGSNFHFFAVGGDPLEMQGVLMNYRTKYPQGTI  178
AHU87355.1      EVIGITSMLNLHAGSQKVHENGGGKPVQGSNFHFFAVGGDPLEMQGVLMNYRTKYPQGTI  178
AHU87364.1      EVIGITSMLNLHAGSQKVHENGGGKPVQGSNFHFFAVGGDPLEMQGVLMNYRTKYPQGTI  178
AHU87365.1      EVIGITSMLNLHAGSQKVHENGGGKPVQGSNFHFFAVGGDPLEMQGVLMNYRTKYPQGTI  178
AFQ31638.1      EVIGITSMLNLHAGSQKVHENGGGKPVQGSNFHFFAVGGDPLEMQGVLMNYRTKYPQGTI  178
AFA41874.1      EVIGITSMLNLHAGSQKVHENGGGKPVQGSNFHFFAVGGDPLEMQGVLMNYRTKYPQGTI  178
AFA41875.1      EVIGITSMLNLHAGSQKVHENGGGKPVQGSNFHFFAVGGDPLEMQGVLMNYRTKYPQGTI  178
AFA41896.1      EVIGITSMLNLHAGSQKVHENGGGKPVQGSNFHFFAVGGDPLEMQGVLMNYRTKYPQGTI  178
BAF93367.1      EVIGITSMLNLHAGSQKVHENGGGKPVQGSNFHFFAVGGDPLEMQGVLMNYRTKYPQGTI  178
CAA79596.1      EVIGITSMLNLHAGSQKVHENGGGKPVQGSNFHFFAVGGDPLEMQGVLMNYRTKYPQGTI  178
AFA41883.1      EVIGITSMLNLHAGSQKVHENGGGKPIQGSNFHFFAVGGDPLEMQGVLMNYRTKYPEGTV  178
WLD47812.1      EVIGITSMLNLHAGSQKVHENGGGKPIQGSNFHFFAVGGDPLEMQGVLMNYRTKYPEGTV  178
BAG75277.1      EVMGITSMLNLHAGSQKVHENGGGKPIQGSNFHFFAVGGDPLEMQGVLMNYRTKYPEGTV  178
BAF75138.1      EVMGITSMLNLHAGSQKVHDNGGGKPIQGSNFHFFAVGGDPLEMQGVLMNYRTKYPEGTV  178
BAF75102.1      EVIGITSMLNLHAGSQKVHQNGGGKPVQGSNFHFFAVGGDPLEMQGVLMNYRTKYPEGTV  178
AYO46643.1      EVIGITSMLNLHAGSQKVHQNGGGKPVQGSNFHFFAVGGDPLEMQGVLMNYRTKYPEGTV  178
QUW04844.1      EVIGITSMLNLHAGSQKVHQNGGGKPVQGSNFHFFAVGGDPLEMQGVLMNYRTKYPEGTV  178
AYO46683.1      EVIGITSMLNLHAGSQKVHQNGGGKPVQGSNFHFFAVGGDPLEMQGVLMNYRTKYPEGTV  178
AYO46645.1      EVIGITSMLNLHAGSQKVHENGGGKPVQGSNFHFFAVGGDPLEMQGVLMNYRTKYPQGTV  178
BAF75180.1      EVIGITSMLNLHAGSQKVHENGGGKPVQGSNFHFFAVGGDPLEMQGVLMNYRTKYPEGTV  178
BAF75204.1      EVIGITSMLNLHAGSQKVHENGGGKPVQGSNFHFFAVGGDPLEMQGVLMNYRTKYPEGTV  178
AYO46631.1      EVIGITSMLNLHAGSQKVHENGGGKPVQGSNFHFFAVGGDPLEMQGVLMNYRTKYPEGTV  178
AYO46608.1      EVIGITSMLNLHAGSQKVHENGGGKPVQGSNFHFFAVGGDPLEMQGVLMNYRTKYPEGTV  178
AYO46611.1      EVIGITSMLNLHAGSQKVHENGGGKPVQGSNFHFFAVGGDPLEMQGVLMNYRTKYPEGTV  178
AYO46615.1      EVIGITSMLNLHAGSQKVHENGGGKPVQGSNFHFFAVGGDPLEMQGVLMNYRTKYPEGTV  178
AYO46639.1      EVIGITSMLNLHAGSQKVHENGGGKPVQGSNFHFFAVGGDPLEMQGVLMNYRTKYPEGTV  178
AYO46649.1      EVIGITSMLNLHAGSQKVHENGGGKPVQGSNFHFFAVGGDPLEMQGVLMNYRTKYPEGTV  178
AYO46655.1      EVIGITSMLNLHAGSQKVHENGGGKPVQGSNFHFFAVGGDPLEMQGVLMNYRTKYPEGTV  178
AYO46664.1      EVIGITSMLNLHAGSQKVHENGGGKPVQGSNFHFFAVGGDPLEMQGVLMNYRTKYPEGTV  178
AYO46669.1      EVIGITSMLNLHAGSQKVHENGGGKPVQGSNFHFFAVGGDPLEMQGVLMNYRTKYPEGTV  178
AYO46685.1      EVIGITSMLNLHAGSQKVHENGGGKPVQGSNFHFFAVGGDPLEMQGVLMNYRTKYPEGTV  178
AYO46686.1      EVIGITSMLNLHAGSQKVHENGGGKPVQGSNFHFFAVGGDPLEMQGVLMNYRTKYPEGTV  178
AYO46690.1      EVIGITSMLNLHAGSQKVHENGGGKPVQGSNFHFFAVGGDPLEMQGVLMNYRTKYPEGTV  178
AYO46627.1      EVIGITSMLNLHAGSQKVHENGGGKPVQGSNFHFFAVGGDPLEMQGVLMNYRTKYPEGTV  178
AYO46630.1      EVIGITSMLNLHAGSQKVHENGGGKPVQGSNFHFFAVGGDPLEMQGVLMNYRTKYPEGTV  178
AYO46656.1      EVIGITSMLNLHAGSQKVHENGGGKPVQGSNFHFFAVGGDPLEMQGVLMNYRTKYPEGTV  178
BAG75217.1      EVIGITSMLNLHAGSQKVHDNGGGKPIQGSNFHFFAVGGDPLEMQGVLMNYRTKYPEGTV  178
BAG75229.1      EVIGITSMLNLHAGSQKVHDNGGGKPIQGSNFHFFAVGGDPLEMQGVLMNYRTKYPEGTV  178
BAG75235.1      EVIGITSMLNLHAGSQKVHDNGGGKPIQGSNFHFFAVGGDPLEMQGVLMNYRTKYPEGTV  178
BAG75259.1      EVIGITSMLNLHAGSQKVHDNGGGKPIQGSNFHFFAVGGDPLEMQGVLMNYRTKYPEGTV  178
BAF75150.1      EVIGITSMLNLHAGSQKVHDNGGGKPIQGSNFHFFAVGGDPLEMQGVLMNYRTKYPEGTV  178
BAF75156.1      EVIGITSMLNLHAGSQKVHDNGGGKPIQGSNFHFFAVGGDPLEMQGVLMNYRTKYPEGTV  178
BAF75174.1      EVIGITSMLNLHAGSQKVHDNGGGKPIQGSNFHFFAVGGDPLEMQGVLMNYRTKYPEGTV  178
BAE96083.1      EVIGITSMLNLHAGSQKVHDNGGGKPIQGSNFHFFAVGGDPLEMQGVLMNYRTKYPEGTV  178
BAE53654.1      EVIGITSMLNLHAGSQKVHDNGGGKPIQGSNFHFFAVGGDPLEMQGVLMNYRTKYPEGTV  178
```

BAF03035.1	EVIGITSMLNLHAGSQKVHDNGGGKPIQGSNFHFFAVGGDPLEMQGVLMNYRTKYPEGTV	178
QHI34656.1	EVIGITSMLNLHAGSQKVHENGGGKPIQGSNFHFFAVGGDPLEMQGVLMNYRTKYPEGTV	178
ARE72396.1	EVIGITSMLNLHAGSQKVHENGGGKPIQGSNFHFFAVGGDPLEMQGVLMNYRTKYPEGTV	178
AFA41885.1	EVIGITSMLNLHAGSQKVHENGGGKPIQGSNFHFFAVGGDPLEMQGVLMNYRTKYPEGTV	178
BAG75271.1	EVIGITSMLNLHAGSQKVHENGGGKPIQGSNFHFFAVGGDPLEMQGVLMNYRTKYPEGTV	178
BAF75090.1	EVIGITSMLNLHAGSQKVHENGGGKPIQGSNFHFFAVGGDPLEMQGVLMNYRTKYPEGTV	178
BAF75306.1	EVIGITSMLNLHAGSQKVHENGGGKPIQGSNFHFFAVGGDPLEMQGVLMNYRTKYPEGTV	178
BAF75342.1	EVIGITSMLNLHAGSQKVHENGGGKPIQGSNFHFFAVGGDPLEMQGVLMNYRTKYPEGTV	178
BAF03029.1	EVIGITSMLNLHAGSQKVHENGGGKPIQGSNFHFFAVGGDPLEMQGVLMNYRTKYPEGTV	178
BAG75205.1	EVMGITSMLNLHAGSQKVHENGGGKPIQGSNFHFFAVGGDPLEMQGVLMNYRTKYPEGTV	178
BAG75223.1	EVMGITSMLNLHAGSQKVHENGGGKPIQGSNFHFFAVGGDPLEMQGVLMNYRTKYPEGTV	178
BAF75144.1	EVMGITSMLNLHAGSQKVHENGGGKPIQGSNFHFFAVGGDPLEMQGVLMNYRTKYPEGTV	178
BAF75162.1	EVMGITSMLNLHAGSQKVHENGGGKPIQGSNFHFFAVGGDPLEMQGVLMNYRTKYPEGTV	178
BAF75168.1	EVMGITSMLNLHAGSQKVHENGGGKPIQGSNFHFFAVGGDPLEMQGVLMNYRTKYPEGTV	178
BAF75240.1	EVMGITSMLNLHAGSQKVHENGGGKPIQGSNFHFFAVGGDPLEMQGVLMNYRTKYPEGTV	178
BAF03023.1	EVMGITSMLNLHAGSQKVHENGGGKPIQGSNFHFFAVGGDPLEMQGVLMNYRTKYPEGTV	178
BAE96077.1	EVMGITSMLNLHAGSQKVHENGGGKPIQGSNFHFFAVGGDPLEMQGVLMNYRTKYPEGTV	178
BAG84476.1	EVIGITSMLNLHAGSQKVHENGGGKPIQGSNFHFFAVGGDPLEMQGVLMNYRTKYPEGTV	178
AYO46661.1	EVIGITSMLNLHAGSQKVHENGGGKPVQGSNFHFFAVGGDPLEMQGVLMNYRTKYPEGTV	178
AYO46634.1	EVIGITSMLNLHAGSQKVHENGGGKPVQGSNFHFFAVGGDPLEMQGVLMNYRTKYPEGTV	178
AYO46657.1	EVIGITSMLNLHAGSQKVHENGGGKPVQGSNFHFFAVGGDPLEMQGVLMNYRTKYPEGTV	178
BAG75379.1	EVIGITSMLNLHAGSQKVHENGGGKPVQGSNFHFFAVGGDPLEMQGVLMNYRTKYPEGTV	178
BAG75409.1	EVIGITSMLNLHAGSQKVHENGGGKPVQGSNFHFFAVGGDPLEMQGVLMNYRTKYPEGTV	178
BAG75415.1	EVIGITSMLNLHAGSQKVHENGGGKPVQGSNFHFFAVGGDPLEMQGVLMNYRTKYPEGTV	178
BAF75096.1	EVIGITSMLNLHAGSQKVHENGGGKPVQGSNFHFFAVGGDPLEMQGVLMNYRTKYPEGTV	178
BAF75192.1	EVIGITSMLNLHAGSQKVHENGGGKPVQGSNFHFFAVGGDPLEMQGVLMNYRTKYPEGTV	178
BAF75198.1	EVIGITSMLNLHAGSQKVHENGGGKPVQGSNFHFFAVGGDPLEMQGVLMNYRTKYPEGTV	178
BAF75210.1	EVIGITSMLNLHAGSQKVHENGGGKPVQGSNFHFFAVGGDPLEMQGVLMNYRTKYPEGTV	178
BAF75234.1	EVIGITSMLNLHAGSQKVHENGGGKPVQGSNFHFFAVGGDPLEMQGVLMNYRTKYPEGTV	178
BAF75264.1	EVIGITSMLNLHAGSQKVHENGGGKPVQGSNFHFFAVGGDPLEMQGVLMNYRTKYPEGTV	178
BAF75270.1	EVIGITSMLNLHAGSQKVHENGGGKPVQGSNFHFFAVGGDPLEMQGVLMNYRTKYPEGTV	178
BAF75282.1	EVIGITSMLNLHAGSQKVHENGGGKPVQGSNFHFFAVGGDPLEMQGVLMNYRTKYPEGTV	178
BAF75300.1	EVIGITSMLNLHAGSQKVHENGGGKPVQGSNFHFFAVGGDPLEMQGVLMNYRTKYPEGTV	178
BAF75312.1	EVIGITSMLNLHAGSQKVHENGGGKPVQGSNFHFFAVGGDPLEMQGVLMNYRTKYPEGTV	178
BAF75318.1	EVIGITSMLNLHAGSQKVHENGGGKPVQGSNFHFFAVGGDPLEMQGVLMNYRTKYPEGTV	178
BAF75324.1	EVIGITSMLNLHAGSQKVHENGGGKPVQGSNFHFFAVGGDPLEMQGVLMNYRTKYPEGTV	178
BAF75336.1	EVIGITSMLNLHAGSQKVHENGGGKPVQGSNFHFFAVGGDPLEMQGVLMNYRTKYPEGTV	178
AFA41889.1	EVIGITSMLNLHAGSQKVHENGGGKPVQGSNFHFFAVGGDPLEMQGVLMNYRTKYPEGTV	178
UEL36357.1	EVIGITSMLNLHAGSQKVHENGGGKPIQGSNFHFFAVGGDPLEMQGVLMNYRTKYPEGTV	178
BCV68920.1	EVIGITSMLNLHAGSQKVHDNGGGKPIQGSNFHFFAVGGDPLEMQGVLMNYRTKYPEGTV	178
WLD47992.1	EVIGITSMLNLHAGSQKVHENGGGKPIQGSNFHFFAVGGDPLEMQGVLMNYRTKYPEGTV	178
UTM62413.1	EVIGITSMLNLHAGSQKVHENGGGKPIQGSNFHFFAVGGDPLEMQGVLMNYRTKYPEGTV	178
AHU87344.1	EVIGITSMLNLHAGSQKVHENGGGKPIQGSNFHFFAVGGDPLEMQGVLMNYRTKYPEGTV	178
AHU87345.1	EVIGITSMLNLHAGSQKVHENGGGKPIQGSNFHFFAVGGDPLEMQGVLMNYRTKYPEGTV	178
QTJ14946.1	EVIGITSMLNLHAGSQKVHENGGGKPIQGSNFHFFAVGGDPLEMQGVLMNYRTKYPEGTV	178
AYO46666.1	EVIGITSMLNLHAGSQKVHENGGGKPIQGSNFHFFAVGGDPLEMQGVLMNYRTKYPEGTV	178
WLD47788.1	EVIGITSMLNLHAGSQKVHENGGGKPIQGSNFHFFAVGGDPLEMQGVLMNYRTKYPEGTV	178
WLD47794.1	EVIGITSMLNLHAGSQKVHENGGGKPIQGSNFHFFAVGGDPLEMQGVLMNYRTKYPEGTV	178
WLD47800.1	EVIGITSMLNLHAGSQKVHENGGGKPIQGSNFHFFAVGGDPLEMQGVLMNYRTKYPEGTV	178
WLD47806.1	EVIGITSMLNLHAGSQKVHENGGGKPIQGSNFHFFAVGGDPLEMQGVLMNYRTKYPEGTV	178
WLD47872.1	EVIGITSMLNLHAGSQKVHENGGGKPIQGSNFHFFAVGGDPLEMQGVLMNYRTKYPEGTV	178
WLD47986.1	EVIGITSMLNLHAGSQKVHENGGGKPIQGSNFHFFAVGGDPLEMQGVLMNYRTKYPEGTV	178
WLD48010.1	EVIGITSMLNLHAGSQKVHENGGGKPIQGSNFHFFAVGGDPLEMQGVLMNYRTKYPEGTV	178
UTM61951.1	EVIGITSMLNLHAGSQKVHENGGGKPIQGSNFHFFAVGGDPLEMQGVLMNYRTKYPEGTV	178
UTM61957.1	EVIGITSMLNLHAGSQKVHENGGGKPIQGSNFHFFAVGGDPLEMQGVLMNYRTKYPEGTV	178
UTM61993.1	EVIGITSMLNLHAGSQKVHENGGGKPIQGSNFHFFAVGGDPLEMQGVLMNYRTKYPEGTV	178
UTM61999.1	EVIGITSMLNLHAGSQKVHENGGGKPIQGSNFHFFAVGGDPLEMQGVLMNYRTKYPEGTV	178
UTM62011.1	EVIGITSMLNLHAGSQKVHENGGGKPIQGSNFHFFAVGGDPLEMQGVLMNYRTKYPEGTV	178
UTM62071.1	EVIGITSMLNLHAGSQKVHENGGGKPIQGSNFHFFAVGGDPLEMQGVLMNYRTKYPEGTV	178
UTM62077.1	EVIGITSMLNLHAGSQKVHENGGGKPIQGSNFHFFAVGGDPLEMQGVLMNYRTKYPEGTV	178
UTM62119.1	EVIGITSMLNLHAGSQKVHENGGGKPIQGSNFHFFAVGGDPLEMQGVLMNYRTKYPEGTV	178
UTM62125.1	EVIGITSMLNLHAGSQKVHENGGGKPIQGSNFHFFAVGGDPLEMQGVLMNYRTKYPEGTV	178
UTM62131.1	EVIGITSMLNLHAGSQKVHENGGGKPIQGSNFHFFAVGGDPLEMQGVLMNYRTKYPEGTV	178
UTM62137.1	EVIGITSMLNLHAGSQKVHENGGGKPIQGSNFHFFAVGGDPLEMQGVLMNYRTKYPEGTV	178

```
UTM62143.1        EVIGITSMLNLHAGSQKVHENGGGKPIQGSNFHFFAVGGDPLEMQGVLMNYRTKYPEGTV  178
UTM62401.1        EVIGITSMLNLHAGSQKVHENGGGKPIQGSNFHFFAVGGDPLEMQGVLMNYRTKYPEGTV  178
UTM62407.1        EVIGITSMLNLHAGSQKVHENGGGKPIQGSNFHFFAVGGDPLEMQGVLMNYRTKYPEGTV  178
UTM62419.1        EVIGITSMLNLHAGSQKVHENGGGKPIQGSNFHFFAVGGDPLEMQGVLMNYRTKYPEGTV  178
UTM62425.1        EVIGITSMLNLHAGSQKVHENGGGKPIQGSNFHFFAVGGDPLEMQGVLMNYRTKYPEGTV  178
UTM62431.1        EVIGITSMLNLHAGSQKVHENGGGKPIQGSNFHFFAVGGDPLEMQGVLMNYRTKYPEGTV  178
UTM62437.1        EVIGITSMLNLHAGSQKVHENGGGKPIQGSNFHFFAVGGDPLEMQGVLMNYRTKYPEGTV  178
UTM62641.1        EVIGITSMLNLHAGSQKVHENGGGKPIQGSNFHFFAVGGDPLEMQGVLMNYRTKYPEGTV  178
UTM62647.1        EVIGITSMLNLHAGSQKVHENGGGKPIQGSNFHFFAVGGDPLEMQGVLMNYRTKYPEGTV  178
ALF06326.1        EVIGITSMLNLHAGSQKVHENGGGKPIQGSNFHFFAVGGDPLEMQGVLMNYRTKYPEGTV  178
ALF06329.1        EVIGITSMLNLHAGSQKVHENGGGKPIQGSNFHFFAVGGDPLEMQGVLMNYRTKYPEGTV  178
AHU87338.1        EVIGITSMLNLHAGSQKVHENGGGKPIQGSNFHFFAVGGDPLEMQGVLMNYRTKYPEGTV  178
AHU87339.1        EVIGITSMLNLHAGSQKVHENGGGKPIQGSNFHFFAVGGDPLEMQGVLMNYRTKYPEGTV  178
AHU87350.1        EVIGITSMLNLHAGSQKVHENGGGKPIQGSNFHFFAVGGDPLEMQGVLMNYRTKYPEGTV  178
AHU87351.1        EVIGITSMLNLHAGSQKVHENGGGKPIQGSNFHFFAVGGDPLEMQGVLMNYRTKYPEGTV  178
AHU87352.1        EVIGITSMLNLHAGSQKVHENGGGKPIQGSNFHFFAVGGDPLEMQGVLMNYRTKYPEGTV  178
AHU87353.1        EVIGITSMLNLHAGSQKVHENGGGKPIQGSNFHFFAVGGDPLEMQGVLMNYRTKYPEGTV  178
AFQ31653.1        EVIGITSMLNLHAGSQKVHENGGGKPIQGSNFHFFAVGGDPLEMQGVLMNYRTKYPEGTV  178
AFA41884.1        EVIGITSMLNLHAGSQKVHENGGGKPIQGSNFHFFAVGGDPLEMQGVLMNYRTKYPEGTV  178
CBX88320.1        EVIGITSMLNLHAGSQKVHENGGGKPIQGSNFHFFAVGGDPLEMQGVLMNYRTKYPEGTV  178
CBX88326.1        EVIGITSMLNLHAGSQKVHENGGGKPIQGSNFHFFAVGGDPLEMQGVLMNYRTKYPEGTV  178
CBX88338.1        EVIGITSMLNLHAGSQKVHENGGGKPIQGSNFHFFAVGGDPLEMQGVLMNYRTKYPEGTV  178
CBX88344.1        EVIGITSMLNLHAGSQKVHENGGGKPIQGSNFHFFAVGGDPLEMQGVLMNYRTKYPEGTV  178
BAG84452.1        EVIGITSMLNLHAGSQKVHENGGGKPIQGSNFHFFAVGGDPLEMQGVLMNYRTKYPEGTV  178
BAG75385.1        EVIGITSMLNLHAGSQKVHENGGGKPIQGSNFHFFAVGGDPLEMQGVLMNYRTKYPEGTV  178
BAG75445.1        EVIGITSMLNLHAGSQKVHENGGGKPIQGSNFHFFAVGGDPLEMQGVLMNYRTKYPEGTV  178
BAF93343.1        EVIGITSMLNLHAGSQKVHENGGGKPIQGSNFHFFAVGGDPLEMQGVLMNYRTKYPEGTV  178
BAF75066.1        EVIGITSMLNLHAGSQKVHENGGGKPIQGSNFHFFAVGGDPLEMQGVLMNYRTKYPEGTV  178
BAF75072.1        EVIGITSMLNLHAGSQKVHENGGGKPIQGSNFHFFAVGGDPLEMQGVLMNYRTKYPEGTV  178
BAF75078.1        EVIGITSMLNLHAGSQKVHENGGGKPIQGSNFHFFAVGGDPLEMQGVLMNYRTKYPEGTV  178
BAF75084.1        EVIGITSMLNLHAGSQKVHENGGGKPIQGSNFHFFAVGGDPLEMQGVLMNYRTKYPEGTV  178
BAF75108.1        EVIGITSMLNLHAGSQKVHENGGGKPIQGSNFHFFAVGGDPLEMQGVLMNYRTKYPEGTV  178
BAF75132.1        EVIGITSMLNLHAGSQKVHENGGGKPIQGSNFHFFAVGGDPLEMQGVLMNYRTKYPEGTV  178
BAF75216.1        EVIGITSMLNLHAGSQKVHENGGGKPIQGSNFHFFAVGGDPLEMQGVLMNYRTKYPEGTV  178
BAF75222.1        EVIGITSMLNLHAGSQKVHENGGGKPIQGSNFHFFAVGGDPLEMQGVLMNYRTKYPEGTV  178
BAF75228.1        EVIGITSMLNLHAGSQKVHENGGGKPIQGSNFHFFAVGGDPLEMQGVLMNYRTKYPEGTV  178
BAF75246.1        EVIGITSMLNLHAGSQKVHENGGGKPIQGSNFHFFAVGGDPLEMQGVLMNYRTKYPEGTV  178
BAF75252.1        EVIGITSMLNLHAGSQKVHENGGGKPIQGSNFHFFAVGGDPLEMQGVLMNYRTKYPEGTV  178
BAF75258.1        EVIGITSMLNLHAGSQKVHENGGGKPIQGSNFHFFAVGGDPLEMQGVLMNYRTKYPEGTV  178
BAF75276.1        EVIGITSMLNLHAGSQKVHENGGGKPIQGSNFHFFAVGGDPLEMQGVLMNYRTKYPEGTV  178
BAF75330.1        EVIGITSMLNLHAGSQKVHENGGGKPIQGSNFHFFAVGGDPLEMQGVLMNYRTKYPEGTV  178
BAF03115.1        EVIGITSMLNLHAGSQKVHENGGGKPIQGSNFHFFAVGGDPLEMQGVLMNYRTKYPEGTV  178
BAF03120.1        EVIGITSMLNLHAGSQKVHENGGGKPIQGSNFHFFAVGGDPLEMQGVLMNYRTKYPEGTV  178
WLD47842.1        EVIGITSMLNLHAGSQKVHENGGGKPIQGSNFHFFAVGGDPLEMQGVLMNYRTKYPEGTV  178
WLD47884.1        EVIGITSMLNLHAGSQKVHENGGGKPIQGSNFHFFAVGGDPLEMQGVLMNYRTKYPEGTV  178
UTM62101.1        EVIGITSMLNLHAGSQKVHENGGGKPIQGSNFHFFAVGGDPLEMQGVLMNYRTKYPEGTV  178
UTM62107.1        EVIGITSMLNLHAGSQKVHENGGGKPIQGSNFHFFAVGGDPLEMQGVLMNYRTKYPEGTV  178
UTM62113.1        EVIGITSMLNLHAGSQKVHENGGGKPIQGSNFHFFAVGGDPLEMQGVLMNYRTKYPEGTV  178
AFQ31647.1        EVIGITSMLNLHAGSQKVHENGGGKPIQGSNFHFFAVGGDPLEMQGVLMNYRTKYPEGTV  178
AFA41892.1        EVIGITSMLNLHAGSQKVHENGGGKPIQGSNFHFFAVGGDPLEMQGVLMNYRTKYPEGTV  178
BAG75403.1        EVIGITSMLNLHAGSQKVHENGGGKPIQGSNFHFFAVGGDPLEMQGVLMNYRTKYPEGTV  178
BAF75114.1        EVIGITSMLNLHAGSQKVHENGGGKPIQGSNFHFFAVGGDPLEMQGVLMNYRTKYPEGTV  178
BAF75120.1        EVIGITSMLNLHAGSQKVHENGGGKPIQGSNFHFFAVGGDPLEMQGVLMNYRTKYPEGTV  178
BAF75126.1        EVIGITSMLNLHAGSQKVHENGGGKPIQGSNFHFFAVGGDPLEMQGVLMNYRTKYPEGTV  178
BAF75186.1        EVIGITSMLNLHAGSQKVHENGGGKPIQGSNFHFFAVGGDPLEMQGVLMNYRTKYPEGTV  178
BAF75288.1        EVIGITSMLNLHAGSQKVHENGGGKPIQGSNFHFFAVGGDPLEMQGVLMNYRTKYPEGTV  178
BAF75294.1        EVIGITSMLNLHAGSQKVHENGGGKPIQGSNFHFFAVGGDPLEMQGVLMNYRTKYPEGTV  178
BAF75348.1        EVIGITSMLNLHAGSQKVHENGGGKPIQGSNFHFFAVGGDPLEMQGVLMNYRTKYPEGTV  178
AFA41881.1        EVIGITSMLNLHAGSQKVHENGGGKPIQGSNFHFFAVGGDPLEMQGVLMNYRTKYPEGTV  178
                  ** ***** ****** :**: ***** :***:** ****:********:** :*** **

ALF06344.1        TPKNPTAQSQVMNTDHKAYLDKNNAYPVECWVPDPSRNENTRYFGTFTGGENVPPVLHVT  238
QTJ15012.1        TPKNPTAQSQVMNTDHKAYLDKNNAYPVECWVPDPSRNENTRYFGTFTGGENVPPVLHVT  238
QTJ15120.1        TPKNPTAQSQVMNTDHKAYLDKNNAYPVECWVPDPSRNENTRYFGTFTGGENVPPVLHVT  238
```

QHI34657.1	TPKNPTAQSQVMNTDHKAYLDKNNAYPVECWVPDPSRNENTRYFGTFTGGENVPPVLHVT	238
ARE72390.1	TPKNPTAQSQVMNTDHKAYLDKNNAYPVECWIPDPSRNENTRYFGTFTGGENVPPVLHVT	238
QTJ14970.1	TPKNPTAQSQVMNTDHKAYLDKNNAYPVECWIPDPSKNENTRYFGTYTGGENVPPVLHVT	238
QTJ14928.1	TPKNPTAQSQVMNTDHKAYLDKNNAYPVECWVPDPSRNENTRYFGTFTGGENVPPVLHVT	240
AAT47419.1	TPKNPTAQSQVMNTDHKAYLDKNNAYPVECWVPDPSRNENTRYFGTFTGGENVPPVLHVT	238
AAT47413.1	TPKNPTAQSQVMNTDHKAYLDKNNAYPVECWVPDPSRNENTRYFGTFTGGENVPPVLHVT	238
AFQ31661.1	TPKNPTAQSQVMNTDHKAYLDKNNAYPVECWVPDPSRNENTRYFGTFTGGENVPPVLHVT	238
YP_717939.1	TPKNPTAQSQVMNTDHKAYLDKNNAYPVECWVPDPSRNENARYFGTFTGGENVPPVLHVT	238
AFK08545.1	TPKNPTAQSQVMNTDHKAYLDKNNAYPVECWVPDPSRNENARYFGTFTGGENVPPVLHVT	238
P03088.2	TPKNPTAQSQVMNTDHKAYLDKNNAYPVECWVPDPSRNENARYFGTFTGGENVPPVLHVT	238
CAA24299.1	TPKNPTAQSQVMNTDHKAYLDKNNAYPVECWVPDPSRNENARYFGTFTGGENVPPVLHVT	238
QTJ14922.1	TPKNPTAQSQVMNTDHKAYLDKNNAYPVECWVPDPSRNENTRYFGTFTGGENVPPVLHVT	238
AFA41877.1	TPKNPTAQSQVMNTDHKAYLDKNNAYPVECWVPDPSRNENTRYFGTFTGGENVPPVLHVT	238
AAT47371.1	TPKNPTAQSQVMNTDHKAYLDKNNAYPVECWVPDPSRNENTRYFGTFTGGENVPPVLHVT	238
AYO46658.1	TPKNPTAQSQVMNTDHKAYLDKNNAYPVECWVPDPSRNENTRYFGTFTGGENVPPVLHVT	238
AYO46610.1	TPKNPTAQSQVMNTDHKAYLDKNNAYPVECWVPDPSRNENTRYFGTFTGGENVPPVLHVT	238
AYO46689.1	TPKNPTAQSQVMNTDHKAYLDKNNAYPVECWVPDPSRNENTRYFGTFTGGENVPPVLHVT	238
AYO46693.1	TPKNPTAQSQVMNTDHKAYLDKNNAYPVECWVPDPSRNENTRYFGTFTGGENVPPVLHVT	238
BAG75283.1	TPKNPTAQSQVMNTDHKAYLDKNNAYPVECWIPDPSRNENTRYFGTLTGGENVPPVLHVT	238
BAG75313.1	TPKNPTAQSQVMNTDHKAYLDKNNAYPVECWIPDPSRNENTRYFGTLTGGENVPPVLHVT	238
BAG75373.1	TPKNPTAQSQVMNTDHKAYLDKNNAYPVECWIPDPSRNENTRYFGTLTGGENVPPVLHVT	238
BCV68914.1	TPKNPTAQSQVMNTDHKAYLDKNNAYPVECWIPDPSRNENTRYFGTLTGGENVPPVLHVT	238
BCV68917.1	TPKNPTAQSQVMNTDHKAYLDKNNAYPVECWIPDPSRNENTRYFGTLTGGENVPPVLHVT	238
BCV68923.1	TPKNPTAQSQVMNTDHKAYLDKNNAYPVECWIPDPSRNENTRYFGTLTGGENVPPVLHVT	238
AYO46620.1	TPKNPTAQSQVMNTDHKAYLDKNNAYPVECWIPDPSRNENTRYFGTLTGGENVPPVLHVT	238
AYO46646.1	TPKNPTAQSQVMNTDHKAYLDKNNAYPVECWIPDPSRNENTRYFGTLTGGENVPPVLHVT	238
AYO46663.1	TPKNPTAQSQVMNTDHKAYLDKNNAYPVECWIPDPSRNENTRYFGTLTGGENVPPVLHVT	238
AYO46694.1	TPKNPTAQSQVMNTDHKAYLDKNNAYPVECWIPDPSRNENTRYFGTLTGGENVPPVLHVT	238
BAZ99906.1	TPKNPTAQSQVMNTDHKAYLDKNNAYPVECWIPDPSRNENTRYFGTLTGGENVPPVLHVT	238
BAZ99912.1	TPKNPTAQSQVMNTDHKAYLDKNNAYPVECWIPDPSRNENTRYFGTLTGGENVPPVLHVT	238
BAI43576.1	TPKNPTAQSQVMNTDHKAYLDKNNAYPVECWIPDPSRNENTRYFGTLTGGENVPPVLHVT	238
BAI43594.1	TPKNPTAQSQVMNTDHKAYLDKNNAYPVECWIPDPSRNENTRYFGTLTGGENVPPVLHVT	238
BAI43606.1	TPKNPTAQSQVMNTDHKAYLDKNNAYPVECWIPDPSRNENTRYFGTLTGGENVPPVLHVT	238
BAI43624.1	TPKNPTAQSQVMNTDHKAYLDKNNAYPVECWIPDPSRNENTRYFGTLTGGENVPPVLHVT	238
BAH23450.1	TPKNPTAQSQVMNTDHKAYLDKNNAYPVECWIPDPSRNENTRYFGTLTGGENVPPVLHVT	238
BAH23456.1	TPKNPTAQSQVMNTDHKAYLDKNNAYPVECWIPDPSRNENTRYFGTLTGGENVPPVLHVT	238
BAH23462.1	TPKNPTAQSQVMNTDHKAYLDKNNAYPVECWIPDPSRNENTRYFGTLTGGENVPPVLHVT	238
BAH23468.1	TPKNPTAQSQVMNTDHKAYLDKNNAYPVECWIPDPSRNENTRYFGTLTGGENVPPVLHVT	238
BAH23474.1	TPKNPTAQSQVMNTDHKAYLDKNNAYPVECWIPDPSRNENTRYFGTLTGGENVPPVLHVT	238
BAH23480.1	TPKNPTAQSQVMNTDHKAYLDKNNAYPVECWIPDPSRNENTRYFGTLTGGENVPPVLHVT	238
BAH23486.1	TPKNPTAQSQVMNTDHKAYLDKNNAYPVECWIPDPSRNENTRYFGTLTGGENVPPVLHVT	238
BAH23492.1	TPKNPTAQSQVMNTDHKAYLDKNNAYPVECWIPDPSRNENTRYFGTLTGGENVPPVLHVT	238
BAH23498.1	TPKNPTAQSQVMNTDHKAYLDKNNAYPVECWIPDPSRNENTRYFGTLTGGENVPPVLHVT	238
BAH23504.1	TPKNPTAQSQVMNTDHKAYLDKNNAYPVECWIPDPSRNENTRYFGTLTGGENVPPVLHVT	238
BAH23510.1	TPKNPTAQSQVMNTDHKAYLDKNNAYPVECWIPDPSRNENTRYFGTLTGGENVPPVLHVT	238
BAH23516.1	TPKNPTAQSQVMNTDHKAYLDKNNAYPVECWIPDPSRNENTRYFGTLTGGENVPPVLHVT	238
BAH23522.1	TPKNPTAQSQVMNTDHKAYLDKNNAYPVECWIPDPSRNENTRYFGTLTGGENVPPVLHVT	238
BAH23528.1	TPKNPTAQSQVMNTDHKAYLDKNNAYPVECWIPDPSRNENTRYFGTLTGGENVPPVLHVT	238
BAH23534.1	TPKNPTAQSQVMNTDHKAYLDKNNAYPVECWIPDPSRNENTRYFGTLTGGENVPPVLHVT	238
BAH23540.1	TPKNPTAQSQVMNTDHKAYLDKNNAYPVECWIPDPSRNENTRYFGTLTGGENVPPVLHVT	238
BAH23546.1	TPKNPTAQSQVMNTDHKAYLDKNNAYPVECWIPDPSRNENTRYFGTLTGGENVPPVLHVT	238
BAH23552.1	TPKNPTAQSQVMNTDHKAYLDKNNAYPVECWIPDPSRNENTRYFGTLTGGENVPPVLHVT	238
BAH23558.1	TPKNPTAQSQVMNTDHKAYLDKNNAYPVECWIPDPSRNENTRYFGTLTGGENVPPVLHVT	238
BAG84488.1	TPKNPTAQSQVMNTDHKAYLDKNNAYPVECWIPDPSRNENTRYFGTLTGGENVPPVLHVT	238
BAG84494.1	TPKNPTAQSQVMNTDHKAYLDKNNAYPVECWIPDPSRNENTRYFGTLTGGENVPPVLHVT	238
BAG84500.1	TPKNPTAQSQVMNTDHKAYLDKNNAYPVECWIPDPSRNENTRYFGTLTGGENVPPVLHVT	238
BAG84506.1	TPKNPTAQSQVMNTDHKAYLDKNNAYPVECWIPDPSRNENTRYFGTLTGGENVPPVLHVT	238
BAG84512.1	TPKNPTAQSQVMNTDHKAYLDKNNAYPVECWIPDPSRNENTRYFGTLTGGENVPPVLHVT	238
BAG84518.1	TPKNPTAQSQVMNTDHKAYLDKNNAYPVECWIPDPSRNENTRYFGTLTGGENVPPVLHVT	238
BAG84524.1	TPKNPTAQSQVMNTDHKAYLDKNNAYPVECWIPDPSRNENTRYFGTLTGGENVPPVLHVT	238
BAG75163.1	TPKNPTAQSQVMNTDHKAYLDKNNAYPVECWIPDPSRNENTRYFGTLTGGENVPPVLHVT	238
BAG75169.1	TPKNPTAQSQVMNTDHKAYLDKNNAYPVECWIPDPSRNENTRYFGTLTGGENVPPVLHVT	238
BAG75175.1	TPKNPTAQSQVMNTDHKAYLDKNNAYPVECWIPDPSRNENTRYFGTLTGGENVPPVLHVT	238
BAG75181.1	TPKNPTAQSQVMNTDHKAYLDKNNAYPVECWIPDPSRNENTRYFGTLTGGENVPPVLHVT	238

BAG75187.1 TPKNPTAQSQVMNTDHKAYLDKNNAYPVECWIPDPSRNENTRYFGTLTGGENVPPVLHVT 238
BAG75193.1 TPKNPTAQSQVMNTDHKAYLDKNNAYPVECWIPDPSRNENTRYFGTLTGGENVPPVLHVT 238
BAG75199.1 TPKNPTAQSQVMNTDHKAYLDKNNAYPVECWIPDPSRNENTRYFGTLTGGENVPPVLHVT 238
BAG75241.1 TPKNPTAQSQVMNTDHKAYLDKNNAYPVECWIPDPSRNENTRYFGTLTGGENVPPVLHVT 238
BAG75247.1 TPKNPTAQSQVMNTDHKAYLDKNNAYPVECWIPDPSRNENTRYFGTLTGGENVPPVLHVT 238
BAG75253.1 TPKNPTAQSQVMNTDHKAYLDKNNAYPVECWIPDPSRNENTRYFGTLTGGENVPPVLHVT 238
BAG75265.1 TPKNPTAQSQVMNTDHKAYLDKNNAYPVECWIPDPSRNENTRYFGTLTGGENVPPVLHVT 238
BAG75289.1 TPKNPTAQSQVMNTDHKAYLDKNNAYPVECWIPDPSRNENTRYFGTLTGGENVPPVLHVT 238
BAG75295.1 TPKNPTAQSQVMNTDHKAYLDKNNAYPVECWIPDPSRNENTRYFGTLTGGENVPPVLHVT 238
BAG75307.1 TPKNPTAQSQVMNTDHKAYLDKNNAYPVECWIPDPSRNENTRYFGTLTGGENVPPVLHVT 238
BAG75325.1 TPKNPTAQSQVMNTDHKAYLDKNNAYPVECWIPDPSRNENTRYFGTLTGGENVPPVLHVT 238
BAG75331.1 TPKNPTAQSQVMNTDHKAYLDKNNAYPVECWIPDPSRNENTRYFGTLTGGENVPPVLHVT 238
BAG75337.1 TPKNPTAQSQVMNTDHKAYLDKNNAYPVECWIPDPSRNENTRYFGTLTGGENVPPVLHVT 238
BAG75343.1 TPKNPTAQSQVMNTDHKAYLDKNNAYPVECWIPDPSRNENTRYFGTLTGGENVPPVLHVT 238
BAG75397.1 TPKNPTAQSQVMNTDHKAYLDKNNAYPVECWIPDPSRNENTRYFGTLTGGENVPPVLHVT 238
BAG75421.1 TPKNPTAQSQVMNTDHKAYLDKNNAYPVECWIPDPSRNENTRYFGTLTGGENVPPVLHVT 238
BAG75427.1 TPKNPTAQSQVMNTDHKAYLDKNNAYPVECWIPDPSRNENTRYFGTLTGGENVPPVLHVT 238
BAG75433.1 TPKNPTAQSQVMNTDHKAYLDKNNAYPVECWIPDPSRNENTRYFGTLTGGENVPPVLHVT 238
BAF93349.1 TPKNPTAQSQVMNTDHKAYLDKNNAYPVECWIPDPSRNENTRYFGTLTGGENVPPVLHVT 238
BAF76166.1 TPKNPTAQSQVMNTDHKAYLDKNNAYPVECWIPDPSRNENTRYFGTLTGGENVPPVLHVT 238
BAF76172.1 TPKNPTAQSQVMNTDHKAYLDKNNAYPVECWIPDPSRNENTRYFGTLTGGENVPPVLHVT 238
BAF76178.1 TPKNPTAQSQVMNTDHKAYLDKNNAYPVECWIPDPSRNENTRYFGTLTGGENVPPVLHVT 238
BAF76184.1 TPKNPTAQSQVMNTDHKAYLDKNNAYPVECWIPDPSRNENTRYFGTLTGGENVPPVLHVT 238
BAF76190.1 TPKNPTAQSQVMNTDHKAYLDKNNAYPVECWIPDPSRNENTRYFGTLTGGENVPPVLHVT 238
BAF76202.1 TPKNPTAQSQVMNTDHKAYLDKNNAYPVECWIPDPSRNENTRYFGTLTGGENVPPVLHVT 238
BAF42985.1 TPKNPTAQSQVMNTDHKAYLDKNNAYPVECWIPDPSRNENTRYFGTLTGGENVPPVLHVT 238
BAF42991.1 TPKNPTAQSQVMNTDHKAYLDKNNAYPVECWIPDPSRNENTRYFGTLTGGENVPPVLHVT 238
BAF02951.1 TPKNPTAQSQVMNTDHKAYLDKNNAYPVECWIPDPSRNENTRYFGTLTGGENVPPVLHVT 238
BAF02963.1 TPKNPTAQSQVMNTDHKAYLDKNNAYPVECWIPDPSRNENTRYFGTLTGGENVPPVLHVT 238
BAF02969.1 TPKNPTAQSQVMNTDHKAYLDKNNAYPVECWIPDPSRNENTRYFGTLTGGENVPPVLHVT 238
BAF02981.1 TPKNPTAQSQVMNTDHKAYLDKNNAYPVECWIPDPSRNENTRYFGTLTGGENVPPVLHVT 238
BAF02987.1 TPKNPTAQSQVMNTDHKAYLDKNNAYPVECWIPDPSRNENTRYFGTLTGGENVPPVLHVT 238
BAF02993.1 TPKNPTAQSQVMNTDHKAYLDKNNAYPVECWIPDPSRNENTRYFGTLTGGENVPPVLHVT 238
BAF02999.1 TPKNPTAQSQVMNTDHKAYLDKNNAYPVECWIPDPSRNENTRYFGTLTGGENVPPVLHVT 238
BAF03005.1 TPKNPTAQSQVMNTDHKAYLDKNNAYPVECWIPDPSRNENTRYFGTLTGGENVPPVLHVT 238
BAF03011.1 TPKNPTAQSQVMNTDHKAYLDKNNAYPVECWIPDPSRNENTRYFGTLTGGENVPPVLHVT 238
BAE96065.1 TPKNPTAQSQVMNTDHKAYLDKNNAYPVECWIPDPSRNENTRYFGTLTGGENVPPVLHVT 238
BAE53666.1 TPKNPTAQSQVMNTDHKAYLDKNNAYPVECWIPDPSRNENTRYFGTLTGGENVPPVLHVT 238
BAE46623.1 TPKNPTAQSQVMNTDHKAYLDKNNAYPVECWIPDPSRNENTRYFGTLTGGENVPPVLHVT 238
CAA40235.1 TPKNPTAQSQVMNTDHKAYLDKNNAYPVECWIPDPSRNENTRYFGTLTGGENVPPVLHVT 238
BAI43570.1 TPKNPTAQSQVMNTDHKAYLDKNNAYPVECWIPDPSRNENTRYFGTFTGGENVPPVLHVT 238
BAI43582.1 TPKNPTAQSQVMNTDHKAYLDKNNAYPVECWIPDPSRNENTRYFGTFTGGENVPPVLHVT 238
BAI43612.1 TPKNPTAQSQVMNTDHKAYLDKNNAYPVECWIPDPSRNENTRYFGTFTGGENVPPVLHVT 238
BAI43618.1 TPKNPTAQSQVMNTDHKAYLDKNNAYPVECWIPDPSRNENTRYFGTFTGGENVPPVLHVT 238
BAI43630.1 TPKNPTAQSQVMNTDHKAYLDKNNAYPVECWIPDPSRNENTRYFGTFTGGENVPPVLHVT 238
BAG75301.1 TPKNPTAQSQVMNTDHKAYLDKNNAYPVECWIPDPSRNENTRYFGTFTGGENVPPVLHVT 238
BAG75349.1 TPKNPTAQSQVMNTDHKAYLDKNNAYPVECWIPDPSRNENTRYFGTFTGGENVPPVLHVT 238
BAG75367.1 TPKNPTAQSQVMNTDHKAYLDKNNAYPVECWIPDPSRNENTRYFGTFTGGENVPPVLHVT 238
BAF76196.1 TPKNPTAQSQVMNTDHKAYLDKNNAYPVECWIPDPSRNENTRYFGTFTGGENVPPVLHVT 238
BAF02975.1 TPKNPTAQSQVMNTDHKAYLDKNNAYPVECWIPDPSRNENTRYFGTLTGGENVPPVLHVT 238
BAI43600.1 TPKNPTAQSQVMNTDHKAYLDKNNAYPVECWIPDPSRNENTRYFGTLTGGENVPPVLHVT 238
BAE96071.1 TPKNPTAQSQVMNTDHKAYLDKNNAYPVECWIPDPSRNENTRYFGTLTGGENVPPVLHVT 238
BAE53660.1 TPKNPTAQSQVMNTDHKAYLDKNNAYPVECWIPDPSRNENTRYFGTLTGGENVPPVLHVT 238
BCV68926.1 TPKNPTAQSQVMNTDHKAYLDKNNAYPVECWIPDPSRNENTRYFGTLTGGENVPPVLHVT 238
AYO46647.1 TPKNPTAQSQVMNTDHKAYLDKNNAYPVECWIPDPSRNENTRYFGTLTGGENVPPVLHVT 238
BAR80134.1 TPKNPTAQSQVMNTDHKAYLDKNNAYPVECWIPDPSRNENTRYFGTLTGGENVPPVLHVT 238
BAR80140.1 TPKNPTAQSQVMNTDHKAYLDKNNAYPVECWIPDPSRNENTRYFGTLTGGENVPPVLHVT 238
BAI43588.1 TPKNPTAQSQVMNTDHKAYLDKNNAYPVECWIPDPSRNENTRYFGTLTGGENVPPVLHVT 238
BAG75361.1 TPKNPTAQSQVMNTDHKAYLDKNNAYPVECWIPDPSRNENTRYFGTLTGGENVPPVLHVT 238
BAE53642.1 TPKNPTAQSQVMNTDHKAYLDKNNAYPVECWIPDPSRNENTRYFGTLTGGENVPPVLHVT 238
BAE53648.1 TPKNPTAQSQVMNTDHKAYLDKNNAYPVECWIPDPSRNENTRYFGTLTGGENVPPVLHVT 238
AFA41903.1 TPKNPTAQSQVMNTDHKAYLDKNNAYPVECWVPDPSRNENTRYFGTFTGGENVPPVLHVT 238
AFA41918.1 TPKNPTAQSQVMNTDHKAYLDKNNAYPVECWVPDPSRNENTRYFGTFTGGENVPPVLHVT 238
BAF42937.1 TPKNPTAQSQVMNTDHKAYLDKNNAYPVECWVPDPSRNENTRYFGTFTGGENVPPVLHVT 238

BAF42997.1	TPKNPTAQSQVMNTDHKAYLDKNNAYPVECWVPDPSRNENTRYFGTFTGGENVPPVLHVT	238
WLD47902.1	TPKNPTAQSQVMNTDHKAYLDKNNAYPVECWVPDPSRNENTRYFGTFTGGENVPPVLHVT	238
WLD47938.1	TPKNPTAQSQVMNTDHKAYLDKNNAYPVECWVPDPSRNENTRYFGTFTGGENVPPVLHVT	238
WLD47980.1	TPKNPTAQSQVMNTDHKAYLDKNNAYPVECWVPDPSRNENTRYFGTFTGGENVPPVLHVT	238
UTM61891.1	TPKNPTAQSQVMNTDHKAYLDKNNAYPVECWVPDPSRNENTRYFGTFTGGENVPPVLHVT	238
UTM61897.1	TPKNPTAQSQVMNTDHKAYLDKNNAYPVECWVPDPSRNENTRYFGTFTGGENVPPVLHVT	238
UTM61903.1	TPKNPTAQSQVMNTDHKAYLDKNNAYPVECWVPDPSRNENTRYFGTFTGGENVPPVLHVT	238
UTM62215.1	TPKNPTAQSQVMNTDHKAYLDKNNAYPVECWVPDPSRNENTRYFGTFTGGENVPPVLHVT	238
UTM62221.1	TPKNPTAQSQVMNTDHKAYLDKNNAYPVECWVPDPSRNENTRYFGTFTGGENVPPVLHVT	238
UTM62275.1	TPKNPTAQSQVMNTDHKAYLDKNNAYPVECWVPDPSRNENTRYFGTFTGGENVPPVLHVT	238
UTM62281.1	TPKNPTAQSQVMNTDHKAYLDKNNAYPVECWVPDPSRNENTRYFGTFTGGENVPPVLHVT	238
UTM62329.1	TPKNPTAQSQVMNTDHKAYLDKNNAYPVECWVPDPSRNENTRYFGTFTGGENVPPVLHVT	238
UTM62335.1	TPKNPTAQSQVMNTDHKAYLDKNNAYPVECWVPDPSRNENTRYFGTFTGGENVPPVLHVT	238
UTM62383.1	TPKNPTAQSQVMNTDHKAYLDKNNAYPVECWVPDPSRNENTRYFGTFTGGENVPPVLHVT	238
UTM62395.1	TPKNPTAQSQVMNTDHKAYLDKNNAYPVECWVPDPSRNENTRYFGTFTGGENVPPVLHVT	238
UTM62443.1	TPKNPTAQSQVMNTDHKAYLDKNNAYPVECWVPDPSRNENTRYFGTFTGGENVPPVLHVT	238
UTM62449.1	TPKNPTAQSQVMNTDHKAYLDKNNAYPVECWVPDPSRNENTRYFGTFTGGENVPPVLHVT	238
UTM62467.1	TPKNPTAQSQVMNTDHKAYLDKNNAYPVECWVPDPSRNENTRYFGTFTGGENVPPVLHVT	238
UTM62545.1	TPKNPTAQSQVMNTDHKAYLDKNNAYPVECWVPDPSRNENTRYFGTFTGGENVPPVLHVT	238
UTM62551.1	TPKNPTAQSQVMNTDHKAYLDKNNAYPVECWVPDPSRNENTRYFGTFTGGENVPPVLHVT	238
UTM62587.1	TPKNPTAQSQVMNTDHKAYLDKNNAYPVECWVPDPSRNENTRYFGTFTGGENVPPVLHVT	238
UTM62593.1	TPKNPTAQSQVMNTDHKAYLDKNNAYPVECWVPDPSRNENTRYFGTFTGGENVPPVLHVT	238
UTM62599.1	TPKNPTAQSQVMNTDHKAYLDKNNAYPVECWVPDPSRNENTRYFGTFTGGENVPPVLHVT	238
QUW04850.1	TPKNPTAQSQVMNTDHKAYLDKNNAYPVECWVPDPSRNENTRYFGTFTGGENVPPVLHVT	238
QTJ14976.1	TPKNPTAQSQVMNTDHKAYLDKNNAYPVECWVPDPSRNENTRYFGTFTGGENVPPVLHVT	238
QTJ15000.1	TPKNPTAQSQVMNTDHKAYLDKNNAYPVECWVPDPSRNENTRYFGTFTGGENVPPVLHVT	238
QTJ15054.1	TPKNPTAQSQVMNTDHKAYLDKNNAYPVECWVPDPSRNENTRYFGTFTGGENVPPVLHVT	238
QTJ15072.1	TPKNPTAQSQVMNTDHKAYLDKNNAYPVECWVPDPSRNENTRYFGTFTGGENVPPVLHVT	238
QTJ15084.1	TPKNPTAQSQVMNTDHKAYLDKNNAYPVECWVPDPSRNENTRYFGTFTGGENVPPVLHVT	238
QTJ15090.1	TPKNPTAQSQVMNTDHKAYLDKNNAYPVECWVPDPSRNENTRYFGTFTGGENVPPVLHVT	238
QTJ15108.1	TPKNPTAQSQVMNTDHKAYLDKNNAYPVECWVPDPSRNENTRYFGTFTGGENVPPVLHVT	238
QTJ15114.1	TPKNPTAQSQVMNTDHKAYLDKNNAYPVECWVPDPSRNENTRYFGTFTGGENVPPVLHVT	238
QTJ15132.1	TPKNPTAQSQVMNTDHKAYLDKNNAYPVECWVPDPSRNENTRYFGTFTGGENVPPVLHVT	238
QTJ15138.1	TPKNPTAQSQVMNTDHKAYLDKNNAYPVECWVPDPSRNENTRYFGTFTGGENVPPVLHVT	238
AYO46612.1	TPKNPTAQSQVMNTDHKAYLDKNNAYPVECWVPDPSRNENTRYFGTFTGGENVPPVLHVT	238
AYO46613.1	TPKNPTAQSQVMNTDHKAYLDKNNAYPVECWVPDPSRNENTRYFGTFTGGENVPPVLHVT	238
AYO46617.1	TPKNPTAQSQVMNTDHKAYLDKNNAYPVECWVPDPSRNENTRYFGTFTGGENVPPVLHVT	238
AYO46621.1	TPKNPTAQSQVMNTDHKAYLDKNNAYPVECWVPDPSRNENTRYFGTFTGGENVPPVLHVT	238
AYO46622.1	TPKNPTAQSQVMNTDHKAYLDKNNAYPVECWVPDPSRNENTRYFGTFTGGENVPPVLHVT	238
AYO46623.1	TPKNPTAQSQVMNTDHKAYLDKNNAYPVECWVPDPSRNENTRYFGTFTGGENVPPVLHVT	238
AYO46624.1	TPKNPTAQSQVMNTDHKAYLDKNNAYPVECWVPDPSRNENTRYFGTFTGGENVPPVLHVT	238
AYO46629.1	TPKNPTAQSQVMNTDHKAYLDKNNAYPVECWVPDPSRNENTRYFGTFTGGENVPPVLHVT	238
AYO46632.1	TPKNPTAQSQVMNTDHKAYLDKNNAYPVECWVPDPSRNENTRYFGTFTGGENVPPVLHVT	238
AYO46635.1	TPKNPTAQSQVMNTDHKAYLDKNNAYPVECWVPDPSRNENTRYFGTFTGGENVPPVLHVT	238
AYO46638.1	TPKNPTAQSQVMNTDHKAYLDKNNAYPVECWVPDPSRNENTRYFGTFTGGENVPPVLHVT	238
AYO46641.1	TPKNPTAQSQVMNTDHKAYLDKNNAYPVECWVPDPSRNENTRYFGTFTGGENVPPVLHVT	238
AYO46642.1	TPKNPTAQSQVMNTDHKAYLDKNNAYPVECWVPDPSRNENTRYFGTFTGGENVPPVLHVT	238
AYO46644.1	TPKNPTAQSQVMNTDHKAYLDKNNAYPVECWVPDPSRNENTRYFGTFTGGENVPPVLHVT	238
AYO46651.1	TPKNPTAQSQVMNTDHKAYLDKNNAYPVECWVPDPSRNENTRYFGTFTGGENVPPVLHVT	238
AYO46652.1	TPKNPTAQSQVMNTDHKAYLDKNNAYPVECWVPDPSRNENTRYFGTFTGGENVPPVLHVT	238
AYO46659.1	TPKNPTAQSQVMNTDHKAYLDKNNAYPVECWVPDPSRNENTRYFGTFTGGENVPPVLHVT	238
AYO46660.1	TPKNPTAQSQVMNTDHKAYLDKNNAYPVECWVPDPSRNENTRYFGTFTGGENVPPVLHVT	238
AYO46662.1	TPKNPTAQSQVMNTDHKAYLDKNNAYPVECWVPDPSRNENTRYFGTFTGGENVPPVLHVT	238
AYO46665.1	TPKNPTAQSQVMNTDHKAYLDKNNAYPVECWVPDPSRNENTRYFGTFTGGENVPPVLHVT	238
AYO46670.1	TPKNPTAQSQVMNTDHKAYLDKNNAYPVECWVPDPSRNENTRYFGTFTGGENVPPVLHVT	238
AYO46672.1	TPKNPTAQSQVMNTDHKAYLDKNNAYPVECWVPDPSRNENTRYFGTFTGGENVPPVLHVT	238
AYO46676.1	TPKNPTAQSQVMNTDHKAYLDKNNAYPVECWVPDPSRNENTRYFGTFTGGENVPPVLHVT	238
AYO46677.1	TPKNPTAQSQVMNTDHKAYLDKNNAYPVECWVPDPSRNENTRYFGTFTGGENVPPVLHVT	238
AYO46679.1	TPKNPTAQSQVMNTDHKAYLDKNNAYPVECWVPDPSRNENTRYFGTFTGGENVPPVLHVT	238
AYO46682.1	TPKNPTAQSQVMNTDHKAYLDKNNAYPVECWVPDPSRNENTRYFGTFTGGENVPPVLHVT	238
AYO46691.1	TPKNPTAQSQVMNTDHKAYLDKNNAYPVECWVPDPSRNENTRYFGTFTGGENVPPVLHVT	238
AYO46692.1	TPKNPTAQSQVMNTDHKAYLDKNNAYPVECWVPDPSRNENTRYFGTFTGGENVPPVLHVT	238
AUF70040.1	TPKNPTAQSQVMNTDHKAYLDKNNAYPVECWVPDPSRNENTRYFGTFTGGENVPPVLHVT	238
SOH95722.1	TPKNPTAQSQVMNTDHKAYLDKNNAYPVECWVPDPSRNENTRYFGTFTGGENVPPVLHVT	238

```
SOI09711.1        TPKNPTAQSQVMNTDHKAYLDKNNAYPVECWVPDPSRNENTRYFGTFTGGENVPPVLHVT  238
APG38208.1        TPKNPTAQSQVMNTDHKAYLDKNNAYPVECWVPDPSRNENTRYFGTFTGGENVPPVLHVT  238
ALF06332.1        TPKNPTAQSQVMNTDHKAYLDKNNAYPVECWVPDPSRNENTRYFGTFTGGENVPPVLHVT  238
ALF06341.1        TPKNPTAQSQVMNTDHKAYLDKNNAYPVECWVPDPSRNENTRYFGTFTGGENVPPVLHVT  238
BAR80122.1        TPKNPTAQSQVMNTDHKAYLDKNNAYPVECWVPDPSRNENTRYFGTFTGGENVPPVLHVT  238
BAR80128.1        TPKNPTAQSQVMNTDHKAYLDKNNAYPVECWVPDPSRNENTRYFGTFTGGENVPPVLHVT  238
AJG42164.1        TPKNPTAQSQVMNTDHKAYLDKNNAYPVECWVPDPSRNENTRYFGTFTGGENVPPVLHVT  238
AHU87340.1        TPKNPTAQSQVMNTDHKAYLDKNNAYPVECWVPDPSRNENTRYFGTFTGGENVPPVLHVT  238
AHU87341.1        TPKNPTAQSQVMNTDHKAYLDKNNAYPVECWVPDPSRNENTRYFGTFTGGENVPPVLHVT  238
AHU87368.1        TPKNPTAQSQVMNTDHKAYLDKNNAYPVECWVPDPSRNENTRYFGTFTGGENVPPVLHVT  238
AHU87369.1        TPKNPTAQSQVMNTDHKAYLDKNNAYPVECWVPDPSRNENTRYFGTFTGGENVPPVLHVT  238
AFQ31636.1        TPKNPTAQSQVMNTDHKAYLDKNNAYPVECWVPDPSRNENTRYFGTFTGGENVPPVLHVT  238
AFQ31637.1        TPKNPTAQSQVMNTDHKAYLDKNNAYPVECWVPDPSRNENTRYFGTFTGGENVPPVLHVT  238
AFQ31639.1        TPKNPTAQSQVMNTDHKAYLDKNNAYPVECWVPDPSRNENTRYFGTFTGGENVPPVLHVT  238
AFQ31640.1        TPKNPTAQSQVMNTDHKAYLDKNNAYPVECWVPDPSRNENTRYFGTFTGGENVPPVLHVT  238
AFQ31641.1        TPKNPTAQSQVMNTDHKAYLDKNNAYPVECWVPDPSRNENTRYFGTFTGGENVPPVLHVT  238
AFQ31644.1        TPKNPTAQSQVMNTDHKAYLDKNNAYPVECWVPDPSRNENTRYFGTFTGGENVPPVLHVT  238
AFQ31648.1        TPKNPTAQSQVMNTDHKAYLDKNNAYPVECWVPDPSRNENTRYFGTFTGGENVPPVLHVT  238
AFQ31657.1        TPKNPTAQSQVMNTDHKAYLDKNNAYPVECWVPDPSRNENTRYFGTFTGGENVPPVLHVT  238
AFA41882.1        TPKNPTAQSQVMNTDHKAYLDKNNAYPVECWVPDPSRNENTRYFGTFTGGENVPPVLHVT  238
AFA41887.1        TPKNPTAQSQVMNTDHKAYLDKNNAYPVECWVPDPSRNENTRYFGTFTGGENVPPVLHVT  238
AFA41890.1        TPKNPTAQSQVMNTDHKAYLDKNNAYPVECWVPDPSRNENTRYFGTFTGGENVPPVLHVT  238
AFA41891.1        TPKNPTAQSQVMNTDHKAYLDKNNAYPVECWVPDPSRNENTRYFGTFTGGENVPPVLHVT  238
AFA41895.1        TPKNPTAQSQVMNTDHKAYLDKNNAYPVECWVPDPSRNENTRYFGTFTGGENVPPVLHVT  238
AFA41898.1        TPKNPTAQSQVMNTDHKAYLDKNNAYPVECWVPDPSRNENTRYFGTFTGGENVPPVLHVT  238
AFA41901.1        TPKNPTAQSQVMNTDHKAYLDKNNAYPVECWVPDPSRNENTRYFGTFTGGENVPPVLHVT  238
AFA41902.1        TPKNPTAQSQVMNTDHKAYLDKNNAYPVECWVPDPSRNENTRYFGTFTGGENVPPVLHVT  238
AFA41910.1        TPKNPTAQSQVMNTDHKAYLDKNNAYPVECWVPDPSRNENTRYFGTFTGGENVPPVLHVT  238
AFA41914.1        TPKNPTAQSQVMNTDHKAYLDKNNAYPVECWVPDPSRNENTRYFGTFTGGENVPPVLHVT  238
AFA41917.1        TPKNPTAQSQVMNTDHKAYLDKNNAYPVECWVPDPSRNENTRYFGTFTGGENVPPVLHVT  238
AEO89562.1        TPKNPTAQSQVMNTDHKAYLDKNNAYPVECWVPDPSRNENTRYFGTFTGGENVPPVLHVT  238
AEO89568.1        TPKNPTAQSQVMNTDHKAYLDKNNAYPVECWVPDPSRNENTRYFGTFTGGENVPPVLHVT  238
AEO89586.1        TPKNPTAQSQVMNTDHKAYLDKNNAYPVECWVPDPSRNENTRYFGTFTGGENVPPVLHVT  238
AEO89598.1        TPKNPTAQSQVMNTDHKAYLDKNNAYPVECWVPDPSRNENTRYFGTFTGGENVPPVLHVT  238
AEO89621.1        TPKNPTAQSQVMNTDHKAYLDKNNAYPVECWVPDPSRNENTRYFGTFTGGENVPPVLHVT  238
AEK21505.1        TPKNPTAQSQVMNTDHKAYLDKNNAYPVECWVPDPSRNENTRYFGTFTGGENVPPVLHVT  238
BAG84458.1        TPKNPTAQSQVMNTDHKAYLDKNNAYPVECWVPDPSRNENTRYFGTFTGGENVPPVLHVT  238
BAG84470.1        TPKNPTAQSQVMNTDHKAYLDKNNAYPVECWVPDPSRNENTRYFGTFTGGENVPPVLHVT  238
BAG84482.1        TPKNPTAQSQVMNTDHKAYLDKNNAYPVECWVPDPSRNENTRYFGTFTGGENVPPVLHVT  238
BAG75319.1        TPKNPTAQSQVMNTDHKAYLDKNNAYPVECWVPDPSRNENTRYFGTFTGGENVPPVLHVT  238
BAG75355.1        TPKNPTAQSQVMNTDHKAYLDKNNAYPVECWVPDPSRNENTRYFGTFTGGENVPPVLHVT  238
BAG75391.1        TPKNPTAQSQVMNTDHKAYLDKNNAYPVECWVPDPSRNENTRYFGTFTGGENVPPVLHVT  238
BAG75439.1        TPKNPTAQSQVMNTDHKAYLDKNNAYPVECWVPDPSRNENTRYFGTFTGGENVPPVLHVT  238
BAF93301.1        TPKNPTAQSQVMNTDHKAYLDKNNAYPVECWVPDPSRNENTRYFGTFTGGENVPPVLHVT  238
BAF93331.1        TPKNPTAQSQVMNTDHKAYLDKNNAYPVECWVPDPSRNENTRYFGTFTGGENVPPVLHVT  238
BAF93355.1        TPKNPTAQSQVMNTDHKAYLDKNNAYPVECWVPDPSRNENTRYFGTFTGGENVPPVLHVT  238
BAF76208.1        TPKNPTAQSQVMNTDHKAYLDKNNAYPVECWVPDPSRNENTRYFGTFTGGENVPPVLHVT  238
BAF42877.1        TPKNPTAQSQVMNTDHKAYLDKNNAYPVECWVPDPSRNENTRYFGTFTGGENVPPVLHVT  238
BAF42883.1        TPKNPTAQSQVMNTDHKAYLDKNNAYPVECWVPDPSRNENTRYFGTFTGGENVPPVLHVT  238
BAF42889.1        TPKNPTAQSQVMNTDHKAYLDKNNAYPVECWVPDPSRNENTRYFGTFTGGENVPPVLHVT  238
BAF42961.1        TPKNPTAQSQVMNTDHKAYLDKNNAYPVECWVPDPSRNENTRYFGTFTGGENVPPVLHVT  238
BAF42967.1        TPKNPTAQSQVMNTDHKAYLDKNNAYPVECWVPDPSRNENTRYFGTFTGGENVPPVLHVT  238
BAF42973.1        TPKNPTAQSQVMNTDHKAYLDKNNAYPVECWVPDPSRNENTRYFGTFTGGENVPPVLHVT  238
BAF43003.1        TPKNPTAQSQVMNTDHKAYLDKNNAYPVECWVPDPSRNENTRYFGTFTGGENVPPVLHVT  238
BAF43027.1        TPKNPTAQSQVMNTDHKAYLDKNNAYPVECWVPDPSRNENTRYFGTFTGGENVPPVLHVT  238
BAF43033.1        TPKNPTAQSQVMNTDHKAYLDKNNAYPVECWVPDPSRNENTRYFGTFTGGENVPPVLHVT  238
ABI94659.1        TPKNPTAQSQVMNTDHKAYLDKNNAYPVECWVPDPSRNENTRYFGTFTGGENVPPVLHVT  238
ABI94683.1        TPKNPTAQSQVMNTDHKAYLDKNNAYPVECWVPDPSRNENTRYFGTFTGGENVPPVLHVT  238
ABI94719.1        TPKNPTAQSQVMNTDHKAYLDKNNAYPVECWVPDPSRNENTRYFGTFTGGENVPPVLHVT  238
BAE96053.1        TPKNPTAQSQVMNTDHKAYLDKNNAYPVECWVPDPSRNENTRYFGTFTGGENVPPVLHVT  238
BAE96059.1        TPKNPTAQSQVMNTDHKAYLDKNNAYPVECWVPDPSRNENTRYFGTFTGGENVPPVLHVT  238
ABC18005.1        TPKNPTAQSQVMNTDHKAYLDKNNAYPVECWVPDPSRNENTRYFGTFTGGENVPPVLHVT  238
AAT47377.1        TPKNPTAQSQVMNTDHKAYLDKNNAYPVECWVPDPSRNENTRYFGTFTGGENVPPVLHVT  238
AAT47383.1        TPKNPTAQSQVMNTDHKAYLDKNNAYPVECWVPDPSRNENTRYFGTFTGGENVPPVLHVT  238
```

```
AAT47405.1        TPKNPTAQSQVMNTDHKAYLDKNNAYPVECWVPDPSRNENTRYFGTFTGGENVPPVLHVT  238
CAA40239.1        TPKNPTAQSQVMNTDHKAYLDKNNAYPVECWVPDPSRNENTRYFGTFTGGENVPPVLHVT  238
QTJ14940.1        TPKNPTAQSQVMNTDHKAYLDKNNAYPVECWVPDPSRNENTRYFGTLTGGENVPPVLHVT  238
ALF06317.1        TPKSPTAQSQVMNTDHKAYLDKNNAYPVECWIPDPSRNENTRYFGTFTGGENVPPVLHVT  238
ALF06307.1        TPKNPTAQSQVMNTDHKAYLGKNNAYPVECWIPDPSRNENTRYFGTFTGGENVPPVLHVT  238
ALF06297.1        TPKNPTAQSQVMNTDHKAYLDKNNAYPVECWIPDPSRNENTRYFGTFTGGENVPPVLHVT  238
ALF06350.1        TPKNPTAQSQVMNTDHKAYLDKNNAYPVECWXPDPSRNENTRYFGTFTGGENVPPVLHVT  238
AAT47431.1        TPKNPTAQSQVMNTDHKAYLDKNNAYPVECWVPDPSRNENTRYFGTFTGGENVPPVLHVT  238
QTJ15006.1        TPKNPTAQSQVMNTDHKAYLDKNNAYPVECWVPDPSRNENTRYFGTFTGGENVPPVLHVT  238
AYO46667.1        TPKNPTAQSQVMNTDHKAYLDKNNAYPVECWVPDPSRNENTRYFGTFTGGENVPPVLHVT  238
WLD47956.1        TPKNPTAQSQVMNTDHKAYLDKNNAYPVECWVPDPSRNENTRYFGTFTGGENVPPVLHVT  238
QTJ15150.1        TPKNPTAQSQVMNTDHKAYLDKNNAYPVECWVPDPSRNENTRYFGTFTGGENVPPVLHVT  238
UTM62389.1        TPKNPTAQSQVMNTDHKAYLDKNNAYPVECWVPDPSRNENTRYFGTFTGGENVPPVLHVT  238
ABI94701.1        TPKNPTAQSQVMNTDHKAYLDKNNAYPVECWVPDPSRNENTRYFGTFTGGENVPPVLHVT  238
BAF02945.1        TPKNPTAQSQVMNTDHKAYLDKNNAYPVECWVPDPSRNENTRYFGTFTGGENVPPVLHVT  238
CAA40243.1        TPKNPTAQSQVMNTDHKAYLDKNNAYPVECWVPDPSRNENTRYFGTFTGGENVPPVLHVT  238
ABI94725.1        TPKNPTAQSQVMNTDHKAYLDKNNAYPVECWVPDPSRNENTRYFGTFTGGENVPPVLHVT  238
AFQ31660.1        TPKNPTAQSQVMNTDHKAYLDKNNAYPVECWVPDPSRNENTRYFGTFTGGENVPPVLHVT  238
ALF06353.1        TPKNPTAQSQVMNTDHKAYLDKNNAYPVECWXPDPSRNENTRYFGTFTGGENVPPVLHVT  238
ALF06312.1        TPKNPTAQSQVMNTDHKAYLDKNNAYPVECWVPDPSRNENTRYFGTFTGGENVPPVLHVT  238
QEJ80721.1        TPKNPTAQSQVMNTDHKAYLDKNNAYPVECWVPDPSRNENTRYFGTFTGGENVPPVLHVT  238
AYO46614.1        TPKNPTAQSQVMNTDHKAYLDKNNAYPVECWVPDPSRNENTRYFGTFTGGENVPPVLHVT  238
AYO46619.1        TPKNPTAQSQVMNTDHKAYLDKNNAYPVECWVPDPSRNENTRYFGTFTGGENVPPVLHVT  238
AYO46625.1        TPKNPTAQSQVMNTDHKAYLDKNNAYPVECWVPDPSRNENTRYFGTFTGGENVPPVLHVT  238
AYO46626.1        TPKNPTAQSQVMNTDHKAYLDKNNAYPVECWVPDPSRNENTRYFGTFTGGENVPPVLHVT  238
AYO46628.1        TPKNPTAQSQVMNTDHKAYLDKNNAYPVECWVPDPSRNENTRYFGTFTGGENVPPVLHVT  238
AYO46633.1        TPKNPTAQSQVMNTDHKAYLDKNNAYPVECWVPDPSRNENTRYFGTFTGGENVPPVLHVT  238
AYO46636.1        TPKNPTAQSQVMNTDHKAYLDKNNAYPVECWVPDPSRNENTRYFGTFTGGENVPPVLHVT  238
AYO46637.1        TPKNPTAQSQVMNTDHKAYLDKNNAYPVECWVPDPSRNENTRYFGTFTGGENVPPVLHVT  238
AYO46640.1        TPKNPTAQSQVMNTDHKAYLDKNNAYPVECWVPDPSRNENTRYFGTFTGGENVPPVLHVT  238
AYO46648.1        TPKNPTAQSQVMNTDHKAYLDKNNAYPVECWVPDPSRNENTRYFGTFTGGENVPPVLHVT  238
AYO46650.1        TPKNPTAQSQVMNTDHKAYLDKNNAYPVECWVPDPSRNENTRYFGTFTGGENVPPVLHVT  238
AYO46653.1        TPKNPTAQSQVMNTDHKAYLDKNNAYPVECWVPDPSRNENTRYFGTFTGGENVPPVLHVT  238
AYO46654.1        TPKNPTAQSQVMNTDHKAYLDKNNAYPVECWVPDPSRNENTRYFGTFTGGENVPPVLHVT  238
AYO46671.1        TPKNPTAQSQVMNTDHKAYLDKNNAYPVECWVPDPSRNENTRYFGTFTGGENVPPVLHVT  238
AYO46673.1        TPKNPTAQSQVMNTDHKAYLDKNNAYPVECWVPDPSRNENTRYFGTFTGGENVPPVLHVT  238
AYO46674.1        TPKNPTAQSQVMNTDHKAYLDKNNAYPVECWVPDPSRNENTRYFGTFTGGENVPPVLHVT  238
AYO46675.1        TPKNPTAQSQVMNTDHKAYLDKNNAYPVECWVPDPSRNENTRYFGTFTGGENVPPVLHVT  238
AYO46680.1        TPKNPTAQSQVMNTDHKAYLDKNNAYPVECWVPDPSRNENTRYFGTFTGGENVPPVLHVT  238
AYO46681.1        TPKNPTAQSQVMNTDHKAYLDKNNAYPVECWVPDPSRNENTRYFGTFTGGENVPPVLHVT  238
AYO46684.1        TPKNPTAQSQVMNTDHKAYLDKNNAYPVECWVPDPSRNENTRYFGTFTGGENVPPVLHVT  238
AYO46687.1        TPKNPTAQSQVMNTDHKAYLDKNNAYPVECWVPDPSRNENTRYFGTFTGGENVPPVLHVT  238
AEO89574.1        TPKNPTAQSQVMNTDHKAYLDKNNAYPVECWVPDPSRNENTRYFGTFTGGENVPPVLHVT  238
BAF42979.1        TPKNPTAQSQVMNTDHKAYLDKNNAYPVECWVPDPSRNENTRYFGTFTGGENVPPVLHVT  238
BAF43009.1        TPKNPTAQSQVMNTDHKAYLDKNNAYPVECWVPDPSRNENTRYFGTFTGGENVPPVLHVT  238
AYO46678.1        TPKNPTAQSQVMNTDHKAYLDKNNAYPVECWVPDPSRNENTRYFGTFTGGENVPPVLHVT  238
UTM62479.1        TPKNPTAQSQVMNTDHKAYLDKNNAYPVECWVPDPSRNENTRYFGTFTGGENVPPVLHVT  238
QTJ14964.1        TPKNPTAQSQVMNTDHKAYLDKNNAYPVECWVPDPSRNENTRYFGTFTGGENVPPVLHVT  238
QTJ15036.1        TPKNPTAQSQVMNTDHKAYLDKNNAYPVECWVPDPSRNENTRYFGTFTGGENVPPVLHVT  238
QTJ15066.1        TPKNPTAQSQVMNTDHKAYLDKNNAYPVECWVPDPSRNENTRYFGTFTGGENVPPVLHVT  238
QTJ15078.1        TPKNPTAQSQVMNTDHKAYLDKNNAYPVECWVPDPSRNENTRYFGTFTGGENVPPVLHVT  238
QTJ15096.1        TPKNPTAQSQVMNTDHKAYLDKNNAYPVECWVPDPSRNENTRYFGTFTGGENVPPVLHVT  238
AQM40276.1        TPKNPTAQSQVMNTDHKAYLDKNNAYPVECWVPDPSRNENTRYFGTFTGGENVPPVLHVT  238
ABI94689.1        TPKNPTAQSQVMNTDHKAYLDKNNAYPVECWVPDPSRNENTRYFGTFTGGENVPPVLHVT  238
ABI94671.1        TPKNPTAQSQVMNTDHKAYLDKNNAYPVECWVPDPSRNENTRYFGTFTGGENVPPVLHVT  238
AYO46688.1        TPKNPTAQSQVMNTDHKAYLDKNNAYPVECWVPDPSRNENTRYFGTFTGGENVPPVLHVT  238
QTJ15060.1        TPKNPTAQSQVMNTDHKAYLDKNNAYPVECWVPDPSRNENTRYFGTFTGGENVPPVLHVT  238
AFQ31642.1        TPKNPTAQSQVMNTDHKAYLDKNNAYPVECWVPDPSRNENTRYFGTFTGGENVPPVLHVT  238
AYO46616.1        TPKNPTAQSQVMNTDHKAYLDKNNAYPVECWVPDPSRNENTRYFGTFTGGENVPPVLHVT  238
CAA79594.1        TPKNPTAQSQVMNTDHKAYLDKNNAYPVECWVPDPSRNENTRYFGTFTGGENVPPVLHVT  238
WLD47818.1        TPKNPTAQSQVMNTDHKAYLDKNNAYPVECWVPDPSRNENTRYFGTFTGGENVPPVLHVT  238
UTM62155.1        TPKNPTAQSQVMNTDHKAYLDKNNAYPVECWVPDPSRNENTRYFGTFTGGENVPPVLHVT  238
AFA41907.1        TPKNPTAQSQVMNTDHKAYLDKNNAYPVECWVPDPSRNENTRYFGTFTGGENVPPVLHVT  238
WLD47944.1        TPKNPTAQSQVMNTDHKAYLDKNNAYPVECWVPDPSRNENTRYFGTFTGGENVPPVLHVT  238
```

AYO46668.1	TPKNPTAQSQVMNTDHKAYLDKNNAYPVECWVPDPSRNENTRYFGTFTGGENVPPVLHVT	238
AFQ31651.1	TPKNPTAQSQVMNTDHKAYLDKNNAYPVECWVPDPSRNENTRYFGTFTGGENVPPVLHVT	238
QTJ14958.1	TPKNPTAQSQVMNTDHKAYLDKNNAYPVECWVPDPSRNENTRYFGTFTGGENVPPVLHVT	238
AYO46618.1	TPKNPTAQSQVMNTDHKAYLDKNNAYPVECWVPDPSRNENTRYFGTFTGGENVPPVLHVT	238
QTJ15018.1	TPKNPTAQSQVMNTDHKAYLDKNNAYPVECWVPDPSRNENTRYFGTFTGGENVPPVLHVT	238
AYO46609.1	TPKNPTAQSQVMNTDHKAYLDKNNAYPVECWVPDPSRNENTRYFGTFTGGENVPPVLHVT	238
ALF06356.1	TPKNPTAQSQVMNTDHKAYLDKNNAYPVECWVPDPSRNENTRYFGTFTGGENVPPVLHVT	238
AFQ31646.1	TPKNPTAQSQVMNTDHKAYLDKNNAYPVECWVPDPSRNENTRYFGTFTGGENVPPVLHVT	238
AFQ31659.1	TPKNPTAQSQVMNTDHKAYLDKNNAYPVECWVPDPSRNENTRYFGTFTGGENVPPVLHVT	238
BAF02957.1	TPKNPTAQSQVMNTDHKAYLDKNNAYPVECWVPDPSRNENTRYFGTFTGGENVPPVLHVT	238
AAT47365.1	TPKNPTAQSQVMNTDHKAYLDKNNAYPVECWVPDPSRNENTRYFGTFTGGENVPPVLHVT	238
AAT47389.1	TPKNPTAQSQVMNTDHKAYLDKNNAYPVECWVPDPSRNENTRYFGTFTGGENVPPVLHVT	238
AAT47395.1	TPKNPTAQSQVMNTDHKAYLDKNNAYPVECWVPDPSRNENTRYFGTFTGGENVPPVLHVT	238
AAT47401.1	TPKNPTAQSQVMNTDHKAYLDKNNAYPVECWVPDPSRNENTRYFGTFTGGENVPPVLHVT	238
CAA24307.1	TPKNPTAQSQVMNTDHKAYLDKNNAYPVECWVPDPSRNENTRYFGTFTGGENVPPVLHVT	238
AAT47425.1	TPKNPTAQSQVMNTDHKAYLDKNNAYPVECWVPDPSRNENTRYFGTFTGGENVPPVLHVT	238
AFA41880.1	TPKNPTAQSQVMNTDHKAYLDKNNAYPVECWIPDPSRNENTRYFGTFTGGENVPPVLHVT	238
WLD47968.1	TPKNPTAQSQVMNTDHKAYLDKNNAYPVECWIPDPSRNENTRYFGTFTGGENVPPVLHVT	238
AFA41920.1	TPKNPTAQSQVMNTDHKAYLDKNNAYPVECWIPDPSRNENTRYFGTFTGGENVPPVLHVT	238
CBX88302.1	TPKNPTAQSQVMNTDHKAYLDKNNAYPVECWIPDPTRNENTRYFGTFTGGENVPPVLHIT	238
AUF70036.1	TPKNPTAQPQVMNTDHKAYLDKNNAYPVECWIPDPSRNENTRYFGTFTGGENVPPVLHVT	238
ALF06323.1	TPKNPTAQSQVMDTDHKAYLDKNNAYPVECWIPDPSRNENTRYFGTFTGGENVPPVLHVT	238
WLD47890.1	TPKNPTAQSQVMNTDHKAYLDKNNAYPVECWIPDPSRNENTRYFGTFTGGENVPPVLHVT	238
BAF03097.1	TPKNPTAQSQVMNTDHKAYLDKNNAYPVECWIPDPSRNENTRYFGTFTGGENVPPVLHVT	238
ABI94695.1	TPKNPTAQSQVMNTDHKAYLDKNNAYPVECWIPDPSRNENTRYFGTFTGGENVPPVLHVT	238
ABI94623.1	TPKNPTAQSQVMNTDHKAYLDKNNAYPVECWIPDPSRNENTRYFGTFTGGENVPPVLHVT	238
UTM62533.1	TPKNPTAQSQVMNTDHKAYLDKNNAYPVECWIPDPSRNENTRYFGTFTGGENVPPVLHVT	238
QOS45058.1	TPKNPTAQSQVMNTDHKAYLDKNNAYPVECWIPDPSRNENTRYFGTFTGGENVPPVLHVT	238
AHU87362.1	TPKNPTAQSQVMNTDHKAYLDKNNAYPVECWIPDPSRNENTRYFGTFTGGENVPPVLHVT	238
AHU87363.1	TPKNPTAQSQVMNTDHKAYLDKNNAYPVECWIPDPSRNENTRYFGTFTGGENVPPVLHVT	238
AFQ31658.1	TPKNPTAQSQVMNTDHKAYLDKNNAYPVECWIPDPSRNENTRYFGTFTGGENVPPVLHVT	238
AFA41876.1	TPKNPTAQSQVMNTDHKAYLDKNNAYPVECWIPDPSRNENTRYFGTFTGGENVPPVLHVT	238
AFA41906.1	TPKNPTAQSQVMNTDHKAYLDKNNAYPVECWIPDPSRNENTRYFGTFTGGENVPPVLHVT	238
AEO89604.1	TPKNPTAQSQVMNTDHKAYLDKNNAYPVECWIPDPSRNENTRYFGTFTGGENVPPVLHVT	238
CBX88350.1	TPKNPTAQSQVMNTDHKAYLDKNNAYPVECWIPDPSRNENTRYFGTFTGGENVPPVLHVT	238
BAG84446.1	TPKNPTAQSQVMNTDHKAYLDKNNAYPVECWIPDPSRNENTRYFGTFTGGENVPPVLHVT	238
BAG84464.1	TPKNPTAQSQVMNTDHKAYLDKNNAYPVECWIPDPSRNENTRYFGTFTGGENVPPVLHVT	238
BAF93295.1	TPKNPTAQSQVMNTDHKAYLDKNNAYPVECWIPDPSRNENTRYFGTFTGGENVPPVLHVT	238
BAF93373.1	TPKNPTAQSQVMNTDHKAYLDKNNAYPVECWIPDPSRNENTRYFGTFTGGENVPPVLHVT	238
BAF93379.1	TPKNPTAQSQVMNTDHKAYLDKNNAYPVECWIPDPSRNENTRYFGTFTGGENVPPVLHVT	238
BAF42913.1	TPKNPTAQSQVMNTDHKAYLDKNNAYPVECWIPDPSRNENTRYFGTFTGGENVPPVLHVT	238
BAF42931.1	TPKNPTAQSQVMNTDHKAYLDKNNAYPVECWIPDPSRNENTRYFGTFTGGENVPPVLHVT	238
BAF43015.1	TPKNPTAQSQVMNTDHKAYLDKNNAYPVECWIPDPSRNENTRYFGTFTGGENVPPVLHVT	238
ABI94629.1	TPKNPTAQSQVMNTDHKAYLDKNNAYPVECWIPDPSRNENTRYFGTFTGGENVPPVLHVT	238
ABI94653.1	TPKNPTAQSQVMNTDHKAYLDKNNAYPVECWIPDPSRNENTRYFGTFTGGENVPPVLHVT	238
ABI94707.1	TPKNPTAQSQVMNTDHKAYLDKNNAYPVECWIPDPSRNENTRYFGTFTGGENVPPVLHVT	238
BAF03085.1	TPKNPTAQSQVMNTDHKAYLDKNNAYPVECWIPDPSRNENTRYFGTFTGGENVPPVLHVT	238
BAF03091.1	TPKNPTAQSQVMNTDHKAYLDKNNAYPVECWIPDPSRNENTRYFGTFTGGENVPPVLHVT	238
BAF03103.1	TPKNPTAQSQVMNTDHKAYLDKNNAYPVECWIPDPSRNENTRYFGTFTGGENVPPVLHVT	238
BAF03109.1	TPKNPTAQSQVMNTDHKAYLDKNNAYPVECWIPDPSRNENTRYFGTFTGGENVPPVLHVT	238
BAF93325.1	TPKNPTAQSQVMNTDHKAYLDKNNAYPVECWIPDPSRNENTRYFGTFTGGENVPPVLHVT	238
QTJ14934.1	TPKNPTAQSQVMNTDHKAYLDKNNAYPVECWIPDPSRNENTRYFGTFTGGENVPPVLHVT	238
QTJ14994.1	TPKNPTAQSQVMNTDHKAYLDKNNAYPVECWIPDPSRNENTRYFGTFTGGENVPPVLHVT	238
AFA41886.1	TPKNPTAQSQVMNTDHKAYLDKNNAYPVECWIPDPSRNENTRYFGTFTGGENVPPVLHVT	238
AFQ31650.1	TPKNPTAQSQVMNTDHKAYLDKNNAYPVECWIPDPSRNENTRYFGTFTGGENVPPVLHVT	238
AAT47347.1	TPKNPTAQSQVMNTDHKAYLDKNNAYPVECWIPDPSRNENTRYFGTFTGGENVPPVLHVT	238
ABI94617.1	TPKNPTAQSQVMNTDHKAYLDKNNAYPVECWIPDPSRNENTRYFGTFTGGENVPPVLHVT	238
BAF93319.1	TPKNPTAQSQVMNTDHKAYLDKNNAYPVECWIPDPSRNENTRYFGTFTGGENVPPVLHVT	238
CBX88314.1	TPKNPTAQSQVMNTDHKAYLDKNNAYPVECWIPDPSRNENTRYFGTFTGGENVPPVLHVT	238
ALF06320.1	TPKNPTAQSQVMNTDHKAYLDKNNAYPVECWIPDPSRNENTRYFGTFTGGENVPPVLHVT	238
WLD47830.1	TPKNPTAQSQVMNTDHKAYLDKNNAYPVECWIPDPSRNENTRYFGTFTGGENVPPVLHVT	238
WLD47866.1	TPKNPTAQSQVMNTDHKAYLDKNNAYPVECWIPDPSRNENTRYFGTFTGGENVPPVLHVT	238
UTM61939.1	TPKNPTAQSQVMNTDHKAYLDKNNAYPVECWIPDPSRNENTRYFGTFTGGENVPPVLHVT	238
UTM62023.1	TPKNPTAQSQVMNTDHKAYLDKNNAYPVECWIPDPSRNENTRYFGTFTGGENVPPVLHVT	238

UTM62029.1	TPKNPTAQSQVMNTDHKAYLDKNNAYPVECWIPDPSRNENTRYFGTFTGGENVPPVLHVT	238
UTM62053.1	TPKNPTAQSQVMNTDHKAYLDKNNAYPVECWIPDPSRNENTRYFGTFTGGENVPPVLHVT	238
UTM62059.1	TPKNPTAQSQVMNTDHKAYLDKNNAYPVECWIPDPSRNENTRYFGTFTGGENVPPVLHVT	238
UTM62065.1	TPKNPTAQSQVMNTDHKAYLDKNNAYPVECWIPDPSRNENTRYFGTFTGGENVPPVLHVT	238
UTM62317.1	TPKNPTAQSQVMNTDHKAYLDKNNAYPVECWIPDPSRNENTRYFGTFTGGENVPPVLHVT	238
UTM62323.1	TPKNPTAQSQVMNTDHKAYLDKNNAYPVECWIPDPSRNENTRYFGTFTGGENVPPVLHVT	238
UTM62353.1	TPKNPTAQSQVMNTDHKAYLDKNNAYPVECWIPDPSRNENTRYFGTFTGGENVPPVLHVT	238
UTM62473.1	TPKNPTAQSQVMNTDHKAYLDKNNAYPVECWIPDPSRNENTRYFGTFTGGENVPPVLHVT	238
UTM62491.1	TPKNPTAQSQVMNTDHKAYLDKNNAYPVECWIPDPSRNENTRYFGTFTGGENVPPVLHVT	238
UTM62497.1	TPKNPTAQSQVMNTDHKAYLDKNNAYPVECWIPDPSRNENTRYFGTFTGGENVPPVLHVT	238
UTM62503.1	TPKNPTAQSQVMNTDHKAYLDKNNAYPVECWIPDPSRNENTRYFGTFTGGENVPPVLHVT	238
QTJ14988.1	TPKNPTAQSQVMNTDHKAYLDKNNAYPVECWIPDPSRNENTRYFGTFTGGENVPPVLHVT	238
QTJ15024.1	TPKNPTAQSQVMNTDHKAYLDKNNAYPVECWIPDPSRNENTRYFGTFTGGENVPPVLHVT	238
QTJ15030.1	TPKNPTAQSQVMNTDHKAYLDKNNAYPVECWIPDPSRNENTRYFGTFTGGENVPPVLHVT	238
QTJ15102.1	TPKNPTAQSQVMNTDHKAYLDKNNAYPVECWIPDPSRNENTRYFGTFTGGENVPPVLHVT	238
ALF06300.1	TPKNPTAQSQVMNTDHKAYLDKNNAYPVECWIPDPSRNENTRYFGTFTGGENVPPVLHVT	238
AHU87342.1	TPKNPTAQSQVMNTDHKAYLDKNNAYPVECWIPDPSRNENTRYFGTFTGGENVPPVLHVT	238
AHU87343.1	TPKNPTAQSQVMNTDHKAYLDKNNAYPVECWIPDPSRNENTRYFGTFTGGENVPPVLHVT	238
AHU87346.1	TPKNPTAQSQVMNTDHKAYLDKNNAYPVECWIPDPSRNENTRYFGTFTGGENVPPVLHVT	238
AHU87347.1	TPKNPTAQSQVMNTDHKAYLDKNNAYPVECWIPDPSRNENTRYFGTFTGGENVPPVLHVT	238
AHU87349.1	TPKNPTAQSQVMNTDHKAYLDKNNAYPVECWIPDPSRNENTRYFGTFTGGENVPPVLHVT	238
AHU87356.1	TPKNPTAQSQVMNTDHKAYLDKNNAYPVECWIPDPSRNENTRYFGTFTGGENVPPVLHVT	238
AHU87357.1	TPKNPTAQSQVMNTDHKAYLDKNNAYPVECWIPDPSRNENTRYFGTFTGGENVPPVLHVT	238
AHU87358.1	TPKNPTAQSQVMNTDHKAYLDKNNAYPVECWIPDPSRNENTRYFGTFTGGENVPPVLHVT	238
AHU87359.1	TPKNPTAQSQVMNTDHKAYLDKNNAYPVECWIPDPSRNENTRYFGTFTGGENVPPVLHVT	238
AFQ31643.1	TPKNPTAQSQVMNTDHKAYLDKNNAYPVECWIPDPSRNENTRYFGTFTGGENVPPVLHVT	238
AFQ31656.1	TPKNPTAQSQVMNTDHKAYLDKNNAYPVECWIPDPSRNENTRYFGTFTGGENVPPVLHVT	238
AFA41879.1	TPKNPTAQSQVMNTDHKAYLDKNNAYPVECWIPDPSRNENTRYFGTFTGGENVPPVLHVT	238
AFA41897.1	TPKNPTAQSQVMNTDHKAYLDKNNAYPVECWIPDPSRNENTRYFGTFTGGENVPPVLHVT	238
AFA41900.1	TPKNPTAQSQVMNTDHKAYLDKNNAYPVECWIPDPSRNENTRYFGTFTGGENVPPVLHVT	238
AEO89580.1	TPKNPTAQSQVMNTDHKAYLDKNNAYPVECWIPDPSRNENTRYFGTFTGGENVPPVLHVT	238
AEO89592.1	TPKNPTAQSQVMNTDHKAYLDKNNAYPVECWIPDPSRNENTRYFGTFTGGENVPPVLHVT	238
BAF93277.1	TPKNPTAQSQVMNTDHKAYLDKNNAYPVECWIPDPSRNENTRYFGTFTGGENVPPVLHVT	238
BAF93289.1	TPKNPTAQSQVMNTDHKAYLDKNNAYPVECWIPDPSRNENTRYFGTFTGGENVPPVLHVT	238
BAF93307.1	TPKNPTAQSQVMNTDHKAYLDKNNAYPVECWIPDPSRNENTRYFGTFTGGENVPPVLHVT	238
BAF93337.1	TPKNPTAQSQVMNTDHKAYLDKNNAYPVECWIPDPSRNENTRYFGTFTGGENVPPVLHVT	238
BAF42895.1	TPKNPTAQSQVMNTDHKAYLDKNNAYPVECWIPDPSRNENTRYFGTFTGGENVPPVLHVT	238
BAF42919.1	TPKNPTAQSQVMNTDHKAYLDKNNAYPVECWIPDPSRNENTRYFGTFTGGENVPPVLHVT	238
BAF42943.1	TPKNPTAQSQVMNTDHKAYLDKNNAYPVECWIPDPSRNENTRYFGTFTGGENVPPVLHVT	238
BAF42949.1	TPKNPTAQSQVMNTDHKAYLDKNNAYPVECWIPDPSRNENTRYFGTFTGGENVPPVLHVT	238
ABI94665.1	TPKNPTAQSQVMNTDHKAYLDKNNAYPVECWIPDPSRNENTRYFGTFTGGENVPPVLHVT	238
ABI94677.1	TPKNPTAQSQVMNTDHKAYLDKNNAYPVECWIPDPSRNENTRYFGTFTGGENVPPVLHVT	238
BAF02939.1	TPKNPTAQSQVMNTDHKAYLDKNNAYPVECWIPDPSRNENTRYFGTFTGGENVPPVLHVT	238
AAT47353.1	TPKNPTAQSQVMNTDHKAYLDKNNAYPVECWIPDPSRNENTRYFGTFTGGENVPPVLHVT	238
AAT47359.1	TPKNPTAQSQVMNTDHKAYLDKNNAYPVECWIPDPSRNENTRYFGTFTGGENVPPVLHVT	238
CAA40247.1	TPKNPTAQSQVMNTDHKAYLDKNNAYPVECWIPDPSRNENTRYFGTFTGGENVPPVLHVT	238
QTJ14982.1	TPKNPTAQSQVMNTDHKAYLDKNNAYPVECWIPDPSRNENTRYFGTFTGGENVPPVLHVT	238
QTJ15126.1	TPKNPTAQSQVMNTDHKAYLDKNNAYPVECWIPDPSRNENTRYFGTFTGGENVPPVLHVT	238
ABI94635.1	TPKNPTAQSQVMNTDHKAYLDKNNAYPVECWIPDPSRNENTRYFGTFTGGENVPPVLHVT	238
ALF06335.1	TPKNPTAQSQVMNTDHKAYLDKNNAYPVECWIPDPSRNENTRYFGTFTGGENVPPVLHVT	238
AHU87348.1	TPKNPTAQSQVMNTDHKAYLDKNNAYPVECWIPDPSRNENTRYFGTFTGGENVPPVLHVT	238
AFQ31649.1	TPKNPTAQSQVMNTDHKAYLDKNNAYPVECWIPDPSRNENTRYFGTFTGGENVPPVLHVT	238
AFQ31654.1	TPKNPTAQSQVMNTDHKAYLDKNNAYPVECWIPDPSRNENTRYFGTFTGGENVPPVLHVT	238
BAF42907.1	TPKNPTAQSQVMNTDHKAYLDKNNAYPVECWIPDPSRNENTRYFGTFTGGENVPPVLHVT	238
WLD47824.1	TPKNPTAQSQVMNTDHKAYLDKNNAYPVECWIPDPSRNENTRYFGTFTGGENVPPVLHVT	238
WLD47848.1	TPKNPTAQSQVMNTDHKAYLDKNNAYPVECWIPDPSRNENTRYFGTFTGGENVPPVLHVT	238
WLD47854.1	TPKNPTAQSQVMNTDHKAYLDKNNAYPVECWIPDPSRNENTRYFGTFTGGENVPPVLHVT	238
WLD47860.1	TPKNPTAQSQVMNTDHKAYLDKNNAYPVECWIPDPSRNENTRYFGTFTGGENVPPVLHVT	238
WLD47878.1	TPKNPTAQSQVMNTDHKAYLDKNNAYPVECWIPDPSRNENTRYFGTFTGGENVPPVLHVT	238
WLD47908.1	TPKNPTAQSQVMNTDHKAYLDKNNAYPVECWIPDPSRNENTRYFGTFTGGENVPPVLHVT	238
WLD47914.1	TPKNPTAQSQVMNTDHKAYLDKNNAYPVECWIPDPSRNENTRYFGTFTGGENVPPVLHVT	238
WLD47920.1	TPKNPTAQSQVMNTDHKAYLDKNNAYPVECWIPDPSRNENTRYFGTFTGGENVPPVLHVT	238
WLD47926.1	TPKNPTAQSQVMNTDHKAYLDKNNAYPVECWIPDPSRNENTRYFGTFTGGENVPPVLHVT	238
WLD47932.1	TPKNPTAQSQVMNTDHKAYLDKNNAYPVECWIPDPSRNENTRYFGTFTGGENVPPVLHVT	238

WLD47950.1 TPKNPTAQSQVMNTDHKAYLDKNNAYPVECWIPDPSRNENTRYFGTFTGGENVPPVLHVT 238
WLD47962.1 TPKNPTAQSQVMNTDHKAYLDKNNAYPVECWIPDPSRNENTRYFGTFTGGENVPPVLHVT 238
WLD47974.1 TPKNPTAQSQVMNTDHKAYLDKNNAYPVECWIPDPSRNENTRYFGTFTGGENVPPVLHVT 238
WLD47998.1 TPKNPTAQSQVMNTDHKAYLDKNNAYPVECWIPDPSRNENTRYFGTFTGGENVPPVLHVT 238
WLD48004.1 TPKNPTAQSQVMNTDHKAYLDKNNAYPVECWIPDPSRNENTRYFGTFTGGENVPPVLHVT 238
WLD48016.1 TPKNPTAQSQVMNTDHKAYLDKNNAYPVECWIPDPSRNENTRYFGTFTGGENVPPVLHVT 238
WLD48022.1 TPKNPTAQSQVMNTDHKAYLDKNNAYPVECWIPDPSRNENTRYFGTFTGGENVPPVLHVT 238
WLD48034.1 TPKNPTAQSQVMNTDHKAYLDKNNAYPVECWIPDPSRNENTRYFGTFTGGENVPPVLHVT 238
UTM61909.1 TPKNPTAQSQVMNTDHKAYLDKNNAYPVECWIPDPSRNENTRYFGTFTGGENVPPVLHVT 238
UTM61915.1 TPKNPTAQSQVMNTDHKAYLDKNNAYPVECWIPDPSRNENTRYFGTFTGGENVPPVLHVT 238
UTM61921.1 TPKNPTAQSQVMNTDHKAYLDKNNAYPVECWIPDPSRNENTRYFGTFTGGENVPPVLHVT 238
UTM61927.1 TPKNPTAQSQVMNTDHKAYLDKNNAYPVECWIPDPSRNENTRYFGTFTGGENVPPVLHVT 238
UTM61933.1 TPKNPTAQSQVMNTDHKAYLDKNNAYPVECWIPDPSRNENTRYFGTFTGGENVPPVLHVT 238
UTM61963.1 TPKNPTAQSQVMNTDHKAYLDKNNAYPVECWIPDPSRNENTRYFGTFTGGENVPPVLHVT 238
UTM61969.1 TPKNPTAQSQVMNTDHKAYLDKNNAYPVECWIPDPSRNENTRYFGTFTGGENVPPVLHVT 238
UTM61975.1 TPKNPTAQSQVMNTDHKAYLDKNNAYPVECWIPDPSRNENTRYFGTFTGGENVPPVLHVT 238
UTM61981.1 TPKNPTAQSQVMNTDHKAYLDKNNAYPVECWIPDPSRNENTRYFGTFTGGENVPPVLHVT 238
UTM62005.1 TPKNPTAQSQVMNTDHKAYLDKNNAYPVECWIPDPSRNENTRYFGTFTGGENVPPVLHVT 238
UTM62017.1 TPKNPTAQSQVMNTDHKAYLDKNNAYPVECWIPDPSRNENTRYFGTFTGGENVPPVLHVT 238
UTM62035.1 TPKNPTAQSQVMNTDHKAYLDKNNAYPVECWIPDPSRNENTRYFGTFTGGENVPPVLHVT 238
UTM62041.1 TPKNPTAQSQVMNTDHKAYLDKNNAYPVECWIPDPSRNENTRYFGTFTGGENVPPVLHVT 238
UTM62047.1 TPKNPTAQSQVMNTDHKAYLDKNNAYPVECWIPDPSRNENTRYFGTFTGGENVPPVLHVT 238
UTM62083.1 TPKNPTAQSQVMNTDHKAYLDKNNAYPVECWIPDPSRNENTRYFGTFTGGENVPPVLHVT 238
UTM62089.1 TPKNPTAQSQVMNTDHKAYLDKNNAYPVECWIPDPSRNENTRYFGTFTGGENVPPVLHVT 238
UTM62095.1 TPKNPTAQSQVMNTDHKAYLDKNNAYPVECWIPDPSRNENTRYFGTFTGGENVPPVLHVT 238
UTM62149.1 TPKNPTAQSQVMNTDHKAYLDKNNAYPVECWIPDPSRNENTRYFGTFTGGENVPPVLHVT 238
UTM62161.1 TPKNPTAQSQVMNTDHKAYLDKNNAYPVECWIPDPSRNENTRYFGTFTGGENVPPVLHVT 238
UTM62167.1 TPKNPTAQSQVMNTDHKAYLDKNNAYPVECWIPDPSRNENTRYFGTFTGGENVPPVLHVT 238
UTM62173.1 TPKNPTAQSQVMNTDHKAYLDKNNAYPVECWIPDPSRNENTRYFGTFTGGENVPPVLHVT 238
UTM62179.1 TPKNPTAQSQVMNTDHKAYLDKNNAYPVECWIPDPSRNENTRYFGTFTGGENVPPVLHVT 238
UTM62185.1 TPKNPTAQSQVMNTDHKAYLDKNNAYPVECWIPDPSRNENTRYFGTFTGGENVPPVLHVT 238
UTM62191.1 TPKNPTAQSQVMNTDHKAYLDKNNAYPVECWIPDPSRNENTRYFGTFTGGENVPPVLHVT 238
UTM62197.1 TPKNPTAQSQVMNTDHKAYLDKNNAYPVECWIPDPSRNENTRYFGTFTGGENVPPVLHVT 238
UTM62203.1 TPKNPTAQSQVMNTDHKAYLDKNNAYPVECWIPDPSRNENTRYFGTFTGGENVPPVLHVT 238
UTM62209.1 TPKNPTAQSQVMNTDHKAYLDKNNAYPVECWIPDPSRNENTRYFGTFTGGENVPPVLHVT 238
UTM62227.1 TPKNPTAQSQVMNTDHKAYLDKNNAYPVECWIPDPSRNENTRYFGTFTGGENVPPVLHVT 238
UTM62239.1 TPKNPTAQSQVMNTDHKAYLDKNNAYPVECWIPDPSRNENTRYFGTFTGGENVPPVLHVT 238
UTM62245.1 TPKNPTAQSQVMNTDHKAYLDKNNAYPVECWIPDPSRNENTRYFGTFTGGENVPPVLHVT 238
UTM62251.1 TPKNPTAQSQVMNTDHKAYLDKNNAYPVECWIPDPSRNENTRYFGTFTGGENVPPVLHVT 238
UTM62257.1 TPKNPTAQSQVMNTDHKAYLDKNNAYPVECWIPDPSRNENTRYFGTFTGGENVPPVLHVT 238
UTM62263.1 TPKNPTAQSQVMNTDHKAYLDKNNAYPVECWIPDPSRNENTRYFGTFTGGENVPPVLHVT 238
UTM62269.1 TPKNPTAQSQVMNTDHKAYLDKNNAYPVECWIPDPSRNENTRYFGTFTGGENVPPVLHVT 238
UTM62287.1 TPKNPTAQSQVMNTDHKAYLDKNNAYPVECWIPDPSRNENTRYFGTFTGGENVPPVLHVT 238
UTM62293.1 TPKNPTAQSQVMNTDHKAYLDKNNAYPVECWIPDPSRNENTRYFGTFTGGENVPPVLHVT 238
UTM62299.1 TPKNPTAQSQVMNTDHKAYLDKNNAYPVECWIPDPSRNENTRYFGTFTGGENVPPVLHVT 238
UTM62305.1 TPKNPTAQSQVMNTDHKAYLDKNNAYPVECWIPDPSRNENTRYFGTFTGGENVPPVLHVT 238
UTM62311.1 TPKNPTAQSQVMNTDHKAYLDKNNAYPVECWIPDPSRNENTRYFGTFTGGENVPPVLHVT 238
UTM62359.1 TPKNPTAQSQVMNTDHKAYLDKNNAYPVECWIPDPSRNENTRYFGTFTGGENVPPVLHVT 238
UTM62365.1 TPKNPTAQSQVMNTDHKAYLDKNNAYPVECWIPDPSRNENTRYFGTFTGGENVPPVLHVT 238
UTM62371.1 TPKNPTAQSQVMNTDHKAYLDKNNAYPVECWIPDPSRNENTRYFGTFTGGENVPPVLHVT 238
UTM62377.1 TPKNPTAQSQVMNTDHKAYLDKNNAYPVECWIPDPSRNENTRYFGTFTGGENVPPVLHVT 238
UTM62455.1 TPKNPTAQSQVMNTDHKAYLDKNNAYPVECWIPDPSRNENTRYFGTFTGGENVPPVLHVT 238
UTM62461.1 TPKNPTAQSQVMNTDHKAYLDKNNAYPVECWIPDPSRNENTRYFGTFTGGENVPPVLHVT 238
UTM62485.1 TPKNPTAQSQVMNTDHKAYLDKNNAYPVECWIPDPSRNENTRYFGTFTGGENVPPVLHVT 238
UTM62509.1 TPKNPTAQSQVMNTDHKAYLDKNNAYPVECWIPDPSRNENTRYFGTFTGGENVPPVLHVT 238
UTM62515.1 TPKNPTAQSQVMNTDHKAYLDKNNAYPVECWIPDPSRNENTRYFGTFTGGENVPPVLHVT 238
UTM62521.1 TPKNPTAQSQVMNTDHKAYLDKNNAYPVECWIPDPSRNENTRYFGTFTGGENVPPVLHVT 238
UTM62527.1 TPKNPTAQSQVMNTDHKAYLDKNNAYPVECWIPDPSRNENTRYFGTFTGGENVPPVLHVT 238
UTM62539.1 TPKNPTAQSQVMNTDHKAYLDKNNAYPVECWIPDPSRNENTRYFGTFTGGENVPPVLHVT 238
UTM62557.1 TPKNPTAQSQVMNTDHKAYLDKNNAYPVECWIPDPSRNENTRYFGTFTGGENVPPVLHVT 238
UTM62563.1 TPKNPTAQSQVMNTDHKAYLDKNNAYPVECWIPDPSRNENTRYFGTFTGGENVPPVLHVT 238
UTM62569.1 TPKNPTAQSQVMNTDHKAYLDKNNAYPVECWIPDPSRNENTRYFGTFTGGENVPPVLHVT 238
UTM62575.1 TPKNPTAQSQVMNTDHKAYLDKNNAYPVECWIPDPSRNENTRYFGTFTGGENVPPVLHVT 238
UTM62581.1 TPKNPTAQSQVMNTDHKAYLDKNNAYPVECWIPDPSRNENTRYFGTFTGGENVPPVLHVT 238

UTM62605.1	TPKNPTAQSQVMNTDHKAYLDKNNAYPVECWIPDPSRNENTRYFGTFTGGENVPPVLHVT	238
UTM62617.1	TPKNPTAQSQVMNTDHKAYLDKNNAYPVECWIPDPSRNENTRYFGTFTGGENVPPVLHVT	238
UTM62623.1	TPKNPTAQSQVMNTDHKAYLDKNNAYPVECWIPDPSRNENTRYFGTFTGGENVPPVLHVT	238
UTM62629.1	TPKNPTAQSQVMNTDHKAYLDKNNAYPVECWIPDPSRNENTRYFGTFTGGENVPPVLHVT	238
UTM62635.1	TPKNPTAQSQVMNTDHKAYLDKNNAYPVECWIPDPSRNENTRYFGTFTGGENVPPVLHVT	238
QTJ15048.1	TPKNPTAQSQVMNTDHKAYLDKNNAYPVECWIPDPSRNENTRYFGTFTGGENVPPVLHVT	238
QTJ15144.1	TPKNPTAQSQVMNTDHKAYLDKNNAYPVECWIPDPSRNENTRYFGTFTGGENVPPVLHVT	238
AHU87360.1	TPKNPTAQSQVMNTDHKAYLDKNNAYPVECWIPDPSRNENTRYFGTFTGGENVPPVLHVT	238
AHU87361.1	TPKNPTAQSQVMNTDHKAYLDKNNAYPVECWIPDPSRNENTRYFGTFTGGENVPPVLHVT	238
AHU87366.1	TPKNPTAQSQVMNTDHKAYLDKNNAYPVECWIPDPSRNENTRYFGTFTGGENVPPVLHVT	238
AHU87367.1	TPKNPTAQSQVMNTDHKAYLDKNNAYPVECWIPDPSRNENTRYFGTFTGGENVPPVLHVT	238
AFQ31652.1	TPKNPTAQSQVMNTDHKAYLDKNNAYPVECWIPDPSRNENTRYFGTFTGGENVPPVLHVT	238
AFA41888.1	TPKNPTAQSQVMNTDHKAYLDKNNAYPVECWIPDPSRNENTRYFGTFTGGENVPPVLHVT	238
AFA41893.1	TPKNPTAQSQVMNTDHKAYLDKNNAYPVECWIPDPSRNENTRYFGTFTGGENVPPVLHVT	238
AFA41894.1	TPKNPTAQSQVMNTDHKAYLDKNNAYPVECWIPDPSRNENTRYFGTFTGGENVPPVLHVT	238
AFA41899.1	TPKNPTAQSQVMNTDHKAYLDKNNAYPVECWIPDPSRNENTRYFGTFTGGENVPPVLHVT	238
AFA41904.1	TPKNPTAQSQVMNTDHKAYLDKNNAYPVECWIPDPSRNENTRYFGTFTGGENVPPVLHVT	238
AFA41905.1	TPKNPTAQSQVMNTDHKAYLDKNNAYPVECWIPDPSRNENTRYFGTFTGGENVPPVLHVT	238
AFA41908.1	TPKNPTAQSQVMNTDHKAYLDKNNAYPVECWIPDPSRNENTRYFGTFTGGENVPPVLHVT	238
AFA41911.1	TPKNPTAQSQVMNTDHKAYLDKNNAYPVECWIPDPSRNENTRYFGTFTGGENVPPVLHVT	238
AFA41912.1	TPKNPTAQSQVMNTDHKAYLDKNNAYPVECWIPDPSRNENTRYFGTFTGGENVPPVLHVT	238
AFA41913.1	TPKNPTAQSQVMNTDHKAYLDKNNAYPVECWIPDPSRNENTRYFGTFTGGENVPPVLHVT	238
AFA41915.1	TPKNPTAQSQVMNTDHKAYLDKNNAYPVECWIPDPSRNENTRYFGTFTGGENVPPVLHVT	238
AFA41916.1	TPKNPTAQSQVMNTDHKAYLDKNNAYPVECWIPDPSRNENTRYFGTFTGGENVPPVLHVT	238
AFA41919.1	TPKNPTAQSQVMNTDHKAYLDKNNAYPVECWIPDPSRNENTRYFGTFTGGENVPPVLHVT	238
AFA41921.1	TPKNPTAQSQVMNTDHKAYLDKNNAYPVECWIPDPSRNENTRYFGTFTGGENVPPVLHVT	238
AFA41922.1	TPKNPTAQSQVMNTDHKAYLDKNNAYPVECWIPDPSRNENTRYFGTFTGGENVPPVLHVT	238
AFA41923.1	TPKNPTAQSQVMNTDHKAYLDKNNAYPVECWIPDPSRNENTRYFGTFTGGENVPPVLHVT	238
AFA41924.1	TPKNPTAQSQVMNTDHKAYLDKNNAYPVECWIPDPSRNENTRYFGTFTGGENVPPVLHVT	238
AFA41925.1	TPKNPTAQSQVMNTDHKAYLDKNNAYPVECWIPDPSRNENTRYFGTFTGGENVPPVLHVT	238
CBX88272.1	TPKNPTAQSQVMNTDHKAYLDKNNAYPVECWIPDPSRNENTRYFGTFTGGENVPPVLHVT	238
CBX88278.1	TPKNPTAQSQVMNTDHKAYLDKNNAYPVECWIPDPSRNENTRYFGTFTGGENVPPVLHVT	238
CBX88284.1	TPKNPTAQSQVMNTDHKAYLDKNNAYPVECWIPDPSRNENTRYFGTFTGGENVPPVLHVT	238
CBX88290.1	TPKNPTAQSQVMNTDHKAYLDKNNAYPVECWIPDPSRNENTRYFGTFTGGENVPPVLHVT	238
CBX88296.1	TPKNPTAQSQVMNTDHKAYLDKNNAYPVECWIPDPSRNENTRYFGTFTGGENVPPVLHVT	238
CBX88308.1	TPKNPTAQSQVMNTDHKAYLDKNNAYPVECWIPDPSRNENTRYFGTFTGGENVPPVLHVT	238
CBX88332.1	TPKNPTAQSQVMNTDHKAYLDKNNAYPVECWIPDPSRNENTRYFGTFTGGENVPPVLHVT	238
CBX88356.1	TPKNPTAQSQVMNTDHKAYLDKNNAYPVECWIPDPSRNENTRYFGTFTGGENVPPVLHVT	238
CBX88362.1	TPKNPTAQSQVMNTDHKAYLDKNNAYPVECWIPDPSRNENTRYFGTFTGGENVPPVLHVT	238
BAF93313.1	TPKNPTAQSQVMNTDHKAYLDKNNAYPVECWIPDPSRNENTRYFGTFTGGENVPPVLHVT	238
BAF42955.1	TPKNPTAQSQVMNTDHKAYLDKNNAYPVECWIPDPSRNENTRYFGTFTGGENVPPVLHVT	238
BAF43021.1	TPKNPTAQSQVMNTDHKAYLDKNNAYPVECWIPDPSRNENTRYFGTFTGGENVPPVLHVT	238
ABI94611.1	TPKNPTAQSQVMNTDHKAYLDKNNAYPVECWIPDPSRNENTRYFGTFTGGENVPPVLHVT	238
ABI94641.1	TPKNPTAQSQVMNTDHKAYLDKNNAYPVECWIPDPSRNENTRYFGTFTGGENVPPVLHVT	238
ABI94647.1	TPKNPTAQSQVMNTDHKAYLDKNNAYPVECWIPDPSRNENTRYFGTFTGGENVPPVLHVT	238
WLD47896.1	TPKNPTAQSQVMNTDHKAYLDKNNAYPVECWIPDPSRNENTRYFGTFTGGENVPPVLHVT	238
WLD48028.1	TPKNPTAQSQVMNTDHKAYLDKNNAYPVECWIPDPSRNENTRYFGTFTGGENVPPVLHVT	238
BAG84440.1	TPKNPTAQSQVMNTDHKAYLDKNNAYPVECWIPDPSRNENTRYFGTFTGGENVPPVLHVT	238
BAF93283.1	TPKNPTAQSQVMNTDHKAYLDKNNAYPVECWIPDPSRNENTRYFGTFTGGENVPPVLHVT	238
BAF93361.1	TPKNPTAQSQVMNTDHKAYLDKNNAYPVECWIPDPSRNENTRYFGTFTGGENVPPVLHVT	238
WLD48040.1	TPKNPTAQSQVMNTDHKAYLDKNNAYPVECWIPDPSRNENTRYFGTFTGGENVPPVLHVT	238
UTM62611.1	TPKNPTAQSQVMNTDHKAYLDKNNAYPVECWIPDPSRNENTRYFGTFTGGENVPPVLHVT	238
AFQ31645.1	TPKNPTAQSQVMNTDHKAYLDKNNAYPVECWIPDPSRNENTRYFGTFTGGENVPPVLHVT	238
AFA41909.1	TPKNPTAQSQVMNTDHKAYLDKNNAYPVECWIPDPSRNENTRYFGTFTGGENVPPVLHVT	238
ABI94713.1	TPKNPTAQSQVMNTDHMAYLDKNNAYPVECWIPDPSRNENTRYFGTFTGGENVPPVLHVT	238
ABD04662.1	TPKNPTAQSQVMNTDHKAYLDKNNAYPVECWIPDPSRNENTRYFGTFTGGENVPPVLHVT	238
QTJ15042.1	TPKNPTAQSQVMNTDHKAYLDKNNAYPVECWVPDPSRNENTRYFGTFTGGENVPPVLHVT	238
AGX89705.1	TPKNPTAQSQVMNTDHKAYLDKNNAYPVECWIPDPSRNENTRYFGTYTGGENVPPVLHVT	238
AGX89710.1	TPKNPTAQSQVMNTDHKAYLDKNNAYPVECWIPDPSRNENTRYFGTYTGGENVPPVLHVT	238
AGX89717.1	TPKNPTAQSQVMNTDHKAYLDKNNAYPVECWIPDPSRNENTRYFGTYTGGENVPPVLHVT	238
AFQ31655.1	TPKNPTAQSQVMNTDHKAYLDKNNAYPVECWIPDPSRNENTRYFGTYTGGENVPPVLHVT	238
AAA46882.1	TPKNPTAQSQVMNTDHKAYLDKNNAYPVECWIPDPSRNENTRYFGTYTGGENVPPVLHVT	238
P14996.1	TPKNPTAQSQVMNTDHKAYLDKNNAYPVECWIPDPSRNENTRYFGTYTGGENVPPVLHVT	238
AEO89615.1	TPKNPTAQSQVMNTDHKAYLDKNNAYPVECWIPDPSRNENTRYFGTYTGGENVPPVLHVT	238

BCV68929.1	TPKNPTAQSQVMNTDHKAYLDKNNAYPVECWIPDPSKNENTRYFGTYTGGENVPPVLHVT	238
QTJ14952.1	TPKNPTAQSQVMNTDHKAYLDKNNAYPVECWIPDPSKNENTRYFGTYTGGENVPPVLHVT	238
BAG75157.1	TPKNPTAQSQVMNTDHKAYLDKNNAYPVECWIPDPSKNENTRYFGTYTGGENVPPVLHVT	238
BAG75211.1	TPKNPTAQSQVMNTDHKAYLDKNNAYPVECWIPDPSKNENTRYFGTYTGGENVPPVLHVT	238
BAF03017.1	TPKNPTAQSQVMNTDHKAYLDKNNAYPVECWIPDPSKNENTRYFGTYTGGENVPPVLHVT	238
BAF42925.1	TPKNPTAQSQVMNTDHKAYLDKNNAYPVECWIPDPSRNENTRYFGTYTGGENVPPVLHVT	238
WLD47836.1	TPKNPTAQSQVMNTDHKAYLDKNNAYPVECWIPDPSRNENTRYFGTYTGGENVPPVLHVT	238
UEL36354.1	TPKNPTAQSQVMNTDHKAYLDKNNAYPVECWIPDPSRNENTRYFGTYTGGENVPPVLHVT	238
BAF42901.1	TPKNPTAQSQVMNTDHKAYLDKNNAYPVECWIPDPSRNENTRYFGTYTGGENVPPVLHVT	238
ABP87973.1	TPKNPTAQSQVMNTDHKAYLDKNNAYPVECWIPDPSRNENTRYFGTYTGGENVPPVLXVT	238
AFA41878.1	TPKNPTAQSQVMNTDHKAYLDKNNAYPVECWIPDPSRNENTRYFGTYTGGENVPPVLHVT	238
QHI34655.1	TPKNPTAQSQVMNTDHKAYLDKNNAYPVECWIPDPSRNENTRYFGTYTGGENVPPVLHVT	238
UTM61945.1	TPKNPTAQSQVMNTDHKAYLDKNNAYPVECWIPDPSRNENTRYFGTYTGGENVPPVLHVT	238
UTM61987.1	TPKNPTAQSQVMNTDHKAYLDKNNAYPVECWIPDPSRNENTRYFGTYTGGENVPPVLHVT	238
UTM62233.1	TPKNPTAQSQVMNTDHKAYLDKNNAYPVECWIPDPSRNENTRYFGTYTGGENVPPVLHVT	238
UTM62341.1	TPKNPTAQSQVMNTDHKAYLDKNNAYPVECWIPDPSRNENTRYFGTYTGGENVPPVLHVT	238
UTM62347.1	TPKNPTAQSQVMNTDHKAYLDKNNAYPVECWIPDPSRNENTRYFGTYTGGENVPPVLHVT	238
AHU87354.1	TPKNPTAQSQVMNTDHKAYLDKNNAYPVECWIPDPSRNENTRYFGTYTGGENVPPVLHVT	238
AHU87355.1	TPKNPTAQSQVMNTDHKAYLDKNNAYPVECWIPDPSRNENTRYFGTYTGGENVPPVLHVT	238
AHU87364.1	TPKNPTAQSQVMNTDHKAYLDKNNAYPVECWIPDPSRNENTRYFGTYTGGENVPPVLHVT	238
AHU87365.1	TPKNPTAQSQVMNTDHKAYLDKNNAYPVECWIPDPSRNENTRYFGTYTGGENVPPVLHVT	238
AFQ31638.1	TPKNPTAQSQVMNTDHKAYLDKNNAYPVECWIPDPSRNENTRYFGTYTGGENVPPVLHVT	238
AFA41874.1	TPKNPTAQSQVMNTDHKAYLDKNNAYPVECWIPDPSRNENTRYFGTYTGGENVPPVLHVT	238
AFA41875.1	TPKNPTAQSQVMNTDHKAYLDKNNAYPVECWIPDPSRNENTRYFGTYTGGENVPPVLHVT	238
AFA41896.1	TPKNPTAQSQVMNTDHKAYLDKNNAYPVECWIPDPSRNENTRYFGTYTGGENVPPVLHVT	238
BAF93367.1	TPKNPTAQSQVMNTDHKAYLDKNNAYPVECWIPDPSRNENTRYFGTYTGGENVPPVLHVT	238
CAA79596.1	TPKNPTAQSQVMNTDHKAYLDKNNAYPVECWIPDPSRNENTRYFGTYTGGENVPPVLHVT	238
AFA41883.1	TPKNPTAQSQVMNTDHKAYLDKNNAYPVECWIPDPSRNENTRYFGTYTGGENVPPVLHVT	238
WLD47812.1	TPKNPTAQSQVMNTDHKAYLDKNNAYPVECWIPDPSRNENTRYFGTYTGGENVPPVLHVT	238
BAG75277.1	TPKNPTAQSQVMNTDHKAYLDKNNAYPVECWIPDPSRNENTRYFGTYTGGENVPPVLHVT	238
BAF75138.1	TPKNPTAQSQVMNTDHKAYLDKNNAYPVECWIPDPSRNENTRYFGTYTGGENVPPVLHVT	238
BAF75102.1	TPKNPTAQSQVMNTDHKAYLDKNNAYPVECWIPDPSRNENTRYFGTYTGGENVPPVLHVT	238
AYO46643.1	TPKNPTAQSQVMNTDHKAYLDKNNAYPVECWIPDPSRNENTRYFGTYTGGENVPPVLHVT	238
QUW04844.1	TPKNPTAQSQVMNTDHKAYLDKNNAYPVECWIPDPSRNENTRYFGTYTGGENVPPVLHVT	238
AYO46683.1	TPKNPTAQSQVMNTDHKAYLDKNNAYPVECWIPDPSRNENTRYFGTYTGGENVPPVLHVT	238
AYO46645.1	TPKNPTAQSQVMNTDHKAYLDKNNAYPVECWIPDPSRNENTRYFGTYTGGENVPPVLHVT	238
BAF75180.1	TPKNPTAQSQVMNTDHKAYLDKNNAYPVECWIPDPSRNENTRYFGTYTGGENVPPVLHVT	238
BAF75204.1	TPKNPTAQSQVMNTDHKAYLDKNNAYPVECWIPDPSRNENTRYFGTYTGGENVPPVLHVT	238
AYO46631.1	TPKNPTAQSQVMNTDHKAYLDKNNAYPVECWIPDPSRNENTRYFGTYTGGENVPPVLHVT	238
AYO46608.1	TPKNPTAQSQVMNTDHKAYLDKNNAYPVECWIPDPSRNENTRYFGTYTGGENVPPVLHVT	238
AYO46611.1	TPKNPTAQSQVMNTDHKAYLDKNNAYPVECWIPDPSRNENTRYFGTYTGGENVPPVLHVT	238
AYO46615.1	TPKNPTAQSQVMNTDHKAYLDKNNAYPVECWIPDPSRNENTRYFGTYTGGENVPPVLHVT	238
AYO46639.1	TPKNPTAQSQVMNTDHKAYLDKNNAYPVECWIPDPSRNENTRYFGTYTGGENVPPVLHVT	238
AYO46649.1	TPKNPTAQSQVMNTDHKAYLDKNNAYPVECWIPDPSRNENTRYFGTYTGGENVPPVLHVT	238
AYO46655.1	TPKNPTAQSQVMNTDHKAYLDKNNAYPVECWIPDPSRNENTRYFGTYTGGENVPPVLHVT	238
AYO46664.1	TPKNPTAQSQVMNTDHKAYLDKNNAYPVECWIPDPSRNENTRYFGTYTGGENVPPVLHVT	238
AYO46669.1	TPKNPTAQSQVMNTDHKAYLDKNNAYPVECWIPDPSRNENTRYFGTYTGGENVPPVLHVT	238
AYO46685.1	TPKNPTAQSQVMNTDHKAYLDKNNAYPVECWIPDPSRNENTRYFGTYTGGENVPPVLHVT	238
AYO46686.1	TPKNPTAQSQVMNTDHKAYLDKNNAYPVECWIPDPSRNENTRYFGTYTGGENVPPVLHVT	238
AYO46690.1	TPKNPTAQSQVMNTDHKAYLDKNNAYPVECWIPDPSRNENTRYFGTYTGGENVPPVLHVT	238
AYO46627.1	TPKNPTAQSQVMNTDHKAYLDKNNAYPVECWIPDPSRNENTRYFGTYTGGENVPPVLHVT	238
AYO46630.1	TPKNPTAQSQVMNTDHKAYLDKNNAYPVECWIPDPSRNENTRYFGTYTGGENVPPVLHVT	238
AYO46656.1	TPKNPTAQSQVMNTDHKAYLDKNNAYPVECWIPDPSRNENTRYFGTYTGGENVPPVLHVT	238
BAG75217.1	TPKNPTAQSQVMNTDHKAYLDKNNAYPVECWIPDPSRNENTRYFGTYTGGENVPPVLHVT	238
BAG75229.1	TPKNPTAQSQVMNTDHKAYLDKNNAYPVECWIPDPSRNENTRYFGTYTGGENVPPVLHVT	238
BAG75235.1	TPKNPTAQSQVMNTDHKAYLDKNNAYPVECWIPDPSRNENTRYFGTYTGGENVPPVLHVT	238
BAG75259.1	TPKNPTAQSQVMNTDHKAYLDKNNAYPVECWIPDPSRNENTRYFGTYTGGENVPPVLHVT	238
BAF75150.1	TPKNPTAQSQVMNTDHKAYLDKNNAYPVECWIPDPSRNENTRYFGTYTGGENVPPVLHVT	238
BAF75156.1	TPKNPTAQSQVMNTDHKAYLDKNNAYPVECWIPDPSRNENTRYFGTYTGGENVPPVLHVT	238
BAF75174.1	TPKNPTAQSQVMNTDHKAYLDKNNAYPVECWIPDPSRNENTRYFGTYTGGENVPPVLHVT	238
BAE96083.1	TPKNPTAQSQVMNTDHKAYLDKNNAYPVECWIPDPSRNENTRYFGTYTGGENVPPVLHVT	238
BAE53654.1	TPKNPTAQSQVMNTDHKAYLDKNNAYPVECWIPDPSRNENTRYFGTYTGGENVPPVLHVT	238
BAF03035.1	TPKNPTAQSQVMNTDHKAYLDKNNAYPVECWIPDPSRNENTRYFGTYTGGENVPPVLHVT	238
QHI34656.1	TPKNPTAQSQVMNTDHKAYLDKNNAYPVECWIPDPSRNENTRYFGTYTGGENVPPVLHVT	238

```
ARE72396.1          TPKNPTAQSQVMNTDHKAYLDKNNAYPVECWIPDPSRNENTRYFGTYTGGENVPPVLHVT  238
AFA41885.1          TPKNPTAXSQVMNTDHKAYLDKNNAYPVECWIPDPSRNENTRYFGTYTGGENVPPVLHVT  238
BAG75271.1          TPKNPTAQSQVMNTDHKAYLDKNNAYPVECWIPDPSRNENTRYFGTYTGGENVPPVLHVT  238
BAF75090.1          TPKNPTAQSQVMNTDHKAYLDKNNAYPVECWIPDPSRNENTRYFGTYTGGENVPPVLHVT  238
BAF75306.1          TPKNPTAQSQVMNTDHKAYLDKNNAYPVECWIPDPSRNENTRYFGTYTGGENVPPVLHVT  238
BAF75342.1          TPKNPTAQSQVMNTDHKAYLDKNNAYPVECWIPDPSRNENTRYFGTYTGGENVPPVLHVT  238
BAF03029.1          TPKNPTAQSQVMNTDHKAYLDKNNAYPVECWIPDPSRNENTRYFGTYTGGENVPPVLHVT  238
BAG75205.1          TPKNPTAQSQVMNTDHKAYLDKNNAYPVECWIPDPSRNENTRYFGTYTGGENVPPVLHVT  238
BAG75223.1          TPKNPTAQSQVMNTDHKAYLDKNNAYPVECWIPDPSRNENTRYFGTYTGGENVPPVLHVT  238
BAF75144.1          TPKNPTAQSQVMNTDHKAYLDKNNAYPVECWIPDPSRNENTRYFGTYTGGENVPPVLHVT  238
BAF75162.1          TPKNPTAQSQVMNTDHKAYLDKNNAYPVECWIPDPSRNENTRYFGTYTGGENVPPVLHVT  238
BAF75168.1          TPKNPTAQSQVMNTDHKAYLDKNNAYPVECWIPDPSRNENTRYFGTYTGGENVPPVLHVT  238
BAF75240.1          TPKNPTAQSQVMNTDHKAYLDKNNAYPVECWIPDPSRNENTRYFGTYTGGENVPPVLHVT  238
BAF03023.1          TPKNPTAQSQVMNTDHKAYLDKNNAYPVECWIPDPSRNENTRYFGTYTGGENVPPVLHVT  238
BAE96077.1          TPKNPTAQSQVMNTDHKAYLDKNNAYPVECWIPDPSRNENTRYFGTYTGGENVPPVLHVT  238
BAG84476.1          TPKNPTAQSQVMNTDHKAYLDKNNAYPVECWIPDPSKNENTRYFGTYTGGENVPPVLHVT  238
AYO46661.1          TPKNPTAQSQVMNTDHKAYLDKNNAYPVECWIPDPSRNENTRYFGTYTGGENVPPVLHVT  238
AYO46634.1          TPKNPTAQSQVMNTDHKAYLDKNNAYPVECWIPDPSRNENTRYFGTYTGGENVPPVLHVT  238
AYO46657.1          TPKNPTAQSQVMNTDHKAYLDKNNAYPVECWIPDPSRNENTRYFGTYTGGENVPPVLHVT  238
BAG75379.1          TPKNPTAQSQVMNTDHKAYLDKNNAYPVECWIPDPSRNENTRYFGTYTGGENVPPVLHVT  238
BAG75409.1          TPKNPTAQSQVMNTDHKAYLDKNNAYPVECWIPDPSRNENTRYFGTYTGGENVPPVLHVT  238
BAG75415.1          TPKNPTAQSQVMNTDHKAYLDKNNAYPVECWIPDPSRNENTRYFGTYTGGENVPPVLHVT  238
BAF75096.1          TPKNPTAQSQVMNTDHKAYLDKNNAYPVECWIPDPSRNENTRYFGTYTGGENVPPVLHVT  238
BAF75192.1          TPKNPTAQSQVMNTDHKAYLDKNNAYPVECWIPDPSRNENTRYFGTYTGGENVPPVLHVT  238
BAF75198.1          TPKNPTAQSQVMNTDHKAYLDKNNAYPVECWIPDPSRNENTRYFGTYTGGENVPPVLHVT  238
BAF75210.1          TPKNPTAQSQVMNTDHKAYLDKNNAYPVECWIPDPSRNENTRYFGTYTGGENVPPVLHVT  238
BAF75234.1          TPKNPTAQSQVMNTDHKAYLDKNNAYPVECWIPDPSRNENTRYFGTYTGGENVPPVLHVT  238
BAF75264.1          TPKNPTAQSQVMNTDHKAYLDKNNAYPVECWIPDPSRNENTRYFGTYTGGENVPPVLHVT  238
BAF75270.1          TPKNPTAQSQVMNTDHKAYLDKNNAYPVECWIPDPSRNENTRYFGTYTGGENVPPVLHVT  238
BAF75282.1          TPKNPTAQSQVMNTDHKAYLDKNNAYPVECWIPDPSRNENTRYFGTYTGGENVPPVLHVT  238
BAF75300.1          TPKNPTAQSQVMNTDHKAYLDKNNAYPVECWIPDPSRNENTRYFGTYTGGENVPPVLHVT  238
BAF75312.1          TPKNPTAQSQVMNTDHKAYLDKNNAYPVECWIPDPSRNENTRYFGTYTGGENVPPVLHVT  238
BAF75318.1          TPKNPTAQSQVMNTDHKAYLDKNNAYPVECWIPDPSRNENTRYFGTYTGGENVPPVLHVT  238
BAF75324.1          TPKNPTAQSQVMNTDHKAYLDKNNAYPVECWIPDPSRNENTRYFGTYTGGENVPPVLHVT  238
BAF75336.1          TPKNPTAQSQVMNTDHKAYLDKNNAYPVECWIPDPSRNENTRYFGTYTGGENVPPVLHVT  238
AFA41889.1          TPKNPTAQSQVMNTDHKAYLDKNNAYPVECWIPDPSRNENTRYFGTYTGGENVPPVLHVT  238
UEL36357.1          TPKNPTAQSQVMNTDHKAYLDKNNAYPVECWIPDPSRNENTRYFGTYTGGENVPPVLHVT  238
BCV68920.1          TPKNPTAQSQVMNTDHKAYLDKNNAYPVECWIPDPSRNENTRYFGTYTGGENVPPVLHVT  238
WLD47992.1          TPKNPTAQSQVMNTDHKAYLDKNNAYPVECWIPDPSRNENTRYFGTYTGGENVPPVLHVT  238
UTM62413.1          TPKNPTAQSQVMNTDHKAYLDKNNAYPVECWIPDPSRNENTRYFGTYTGGENVPPVLHVT  238
AHU87344.1          TPKNPTAQSQVMNTDHKAYLDKNNAYPVECWIPDPSRNENTRYFGTYTGGENVPPVLHVT  238
AHU87345.1          TPKNPTAQSQVMNTDHKAYLDKNNAYPVECWIPDPSRNENTRYFGTYTGGENVPPVLHVT  238
QTJ14946.1          TPKNPTAQSQVMNTDHKAYLDKNNAYPVECWIPDPSRNENTRYFGTYTGGENVPPVLHVT  238
AYO46666.1          TPKNPTAQSQVMNTDHKAYLDKNNAYPVECWIPDPSRNENTRYFGTYTGGENVPPVLHVT  238
WLD47788.1          TPKNPTAQSQVMNTDHKAYLDKNNAYPVECWIPDPSRNENTRYFGTYTGGENVPPVLHVT  238
WLD47794.1          TPKNPTAQSQVMNTDHKAYLDKNNAYPVECWIPDPSRNENTRYFGTYTGGENVPPVLHVT  238
WLD47800.1          TPKNPTAQSQVMNTDHKAYLDKNNAYPVECWIPDPSRNENTRYFGTYTGGENVPPVLHVT  238
WLD47806.1          TPKNPTAQSQVMNTDHKAYLDKNNAYPVECWIPDPSRNENTRYFGTYTGGENVPPVLHVT  238
WLD47872.1          TPKNPTAQSQVMNTDHKAYLDKNNAYPVECWIPDPSRNENTRYFGTYTGGENVPPVLHVT  238
WLD47986.1          TPKNPTAQSQVMNTDHKAYLDKNNAYPVECWIPDPSRNENTRYFGTYTGGENVPPVLHVT  238
WLD48010.1          TPKNPTAQSQVMNTDHKAYLDKNNAYPVECWIPDPSRNENTRYFGTYTGGENVPPVLHVT  238
UTM61951.1          TPKNPTAQSQVMNTDHKAYLDKNNAYPVECWIPDPSRNENTRYFGTYTGGENVPPVLHVT  238
UTM61957.1          TPKNPTAQSQVMNTDHKAYLDKNNAYPVECWIPDPSRNENTRYFGTYTGGENVPPVLHVT  238
UTM61993.1          TPKNPTAQSQVMNTDHKAYLDKNNAYPVECWIPDPSRNENTRYFGTYTGGENVPPVLHVT  238
UTM61999.1          TPKNPTAQSQVMNTDHKAYLDKNNAYPVECWIPDPSRNENTRYFGTYTGGENVPPVLHVT  238
UTM62011.1          TPKNPTAQSQVMNTDHKAYLDKNNAYPVECWIPDPSRNENTRYFGTYTGGENVPPVLHVT  238
UTM62071.1          TPKNPTAQSQVMNTDHKAYLDKNNAYPVECWIPDPSRNENTRYFGTYTGGENVPPVLHVT  238
UTM62077.1          TPKNPTAQSQVMNTDHKAYLDKNNAYPVECWIPDPSRNENTRYFGTYTGGENVPPVLHVT  238
UTM62119.1          TPKNPTAQSQVMNTDHKAYLDKNNAYPVECWIPDPSRNENTRYFGTYTGGENVPPVLHVT  238
UTM62125.1          TPKNPTAQSQVMNTDHKAYLDKNNAYPVECWIPDPSRNENTRYFGTYTGGENVPPVLHVT  238
UTM62131.1          TPKNPTAQSQVMNTDHKAYLDKNNAYPVECWIPDPSRNENTRYFGTYTGGENVPPVLHVT  238
UTM62137.1          TPKNPTAQSQVMNTDHKAYLDKNNAYPVECWIPDPSRNENTRYFGTYTGGENVPPVLHVT  238
UTM62143.1          TPKNPTAQSQVMNTDHKAYLDKNNAYPVECWIPDPSRNENTRYFGTYTGGENVPPVLHVT  238
UTM62401.1          TPKNPTAQSQVMNTDHKAYLDKNNAYPVECWIPDPSRNENTRYFGTYTGGENVPPVLHVT  238
```

```
UTM62407.1      TPKNPTAQSQVMNTDHKAYLDKNNAYPVECWIPDPSRNENTRYFGTYTGGENVPPVLHVT  238
UTM62419.1      TPKNPTAQSQVMNTDHKAYLDKNNAYPVECWIPDPSRNENTRYFGTYTGGENVPPVLHVT  238
UTM62425.1      TPKNPTAQSQVMNTDHKAYLDKNNAYPVECWIPDPSRNENTRYFGTYTGGENVPPVLHVT  238
UTM62431.1      TPKNPTAQSQVMNTDHKAYLDKNNAYPVECWIPDPSRNENTRYFGTYTGGENVPPVLHVT  238
UTM62437.1      TPKNPTAQSQVMNTDHKAYLDKNNAYPVECWIPDPSRNENTRYFGTYTGGENVPPVLHVT  238
UTM62641.1      TPKNPTAQSQVMNTDHKAYLDKNNAYPVECWIPDPSRNENTRYFGTYTGGENVPPVLHVT  238
UTM62647.1      TPKNPTAQSQVMNTDHKAYLDKNNAYPVECWIPDPSRNENTRYFGTYTGGENVPPVLHVT  238
ALF06326.1      TPKNPTAQSQVMNTDHKAYLDKNNAYPVECWIPDPSRNENTRYFGTYTGGENVPPVLHVT  238
ALF06329.1      TPKNPTAQSQVMNTDHKAYLDKNNAYPVECWIPDPSRNENTRYFGTYTGGENVPPVLHVT  238
AHU87338.1      TPKNPTAQSQVMNTDHKAYLDKNNAYPVECWIPDPSRNENTRYFGTYTGGENVPPVLHVT  238
AHU87339.1      TPKNPTAQSQVMNTDHKAYLDKNNAYPVECWIPDPSRNENTRYFGTYTGGENVPPVLHVT  238
AHU87350.1      TPKNPTAQSQVMNTDHKAYLDKNNAYPVECWIPDPSRNENTRYFGTYTGGENVPPVLHVT  238
AHU87351.1      TPKNPTAQSQVMNTDHKAYLDKNNAYPVECWIPDPSRNENTRYFGTYTGGENVPPVLHVT  238
AHU87352.1      TPKNPTAQSQVMNTDHKAYLDKNNAYPVECWIPDPSRNENTRYFGTYTGGENVPPVLHVT  238
AHU87353.1      TPKNPTAQSQVMNTDHKAYLDKNNAYPVECWIPDPSRNENTRYFGTYTGGENVPPVLHVT  238
AFQ31653.1      TPKNPTAQSQVMNTDHKAYLDKNNAYPVECWIPDPSRNENTRYFGTYTGGENVPPVLHVT  238
AFA41884.1      TPKNPTAQSQVMNTDHKAYLDKNNAYPVECWIPDPSRNENTRYFGTYTGGENVPPVLHVT  238
CBX88320.1      TPKNPTAQSQVMNTDHKAYLDKNNAYPVECWIPDPSRNENTRYFGTYTGGENVPPVLHVT  238
CBX88326.1      TPKNPTAQSQVMNTDHKAYLDKNNAYPVECWIPDPSRNENTRYFGTYTGGENVPPVLHVT  238
CBX88338.1      TPKNPTAQSQVMNTDHKAYLDKNNAYPVECWIPDPSRNENTRYFGTYTGGENVPPVLHVT  238
CBX88344.1      TPKNPTAQSQVMNTDHKAYLDKNNAYPVECWIPDPSRNENTRYFGTYTGGENVPPVLHVT  238
BAG84452.1      TPKNPTAQSQVMNTDHKAYLDKNNAYPVECWIPDPSRNENTRYFGTYTGGENVPPVLHVT  238
BAG75385.1      TPKNPTAQSQVMNTDHKAYLDKNNAYPVECWIPDPSRNENTRYFGTYTGGENVPPVLHVT  238
BAG75445.1      TPKNPTAQSQVMNTDHKAYLDKNNAYPVECWIPDPSRNENTRYFGTYTGGENVPPVLHVT  238
BAF93343.1      TPKNPTAQSQVMNTDHKAYLDKNNAYPVECWIPDPSRNENTRYFGTYTGGENVPPVLHVT  238
BAF75066.1      TPKNPTAQSQVMNTDHKAYLDKNNAYPVECWIPDPSRNENTRYFGTYTGGENVPPVLHVT  238
BAF75072.1      TPKNPTAQSQVMNTDHKAYLDKNNAYPVECWIPDPSRNENTRYFGTYTGGENVPPVLHVT  238
BAF75078.1      TPKNPTAQSQVMNTDHKAYLDKNNAYPVECWIPDPSRNENTRYFGTYTGGENVPPVLHVT  238
BAF75084.1      TPKNPTAQSQVMNTDHKAYLDKNNAYPVECWIPDPSRNENTRYFGTYTGGENVPPVLHVT  238
BAF75108.1      TPKNPTAQSQVMNTDHKAYLDKNNAYPVECWIPDPSRNENTRYFGTYTGGENVPPVLHVT  238
BAF75132.1      TPKNPTAQSQVMNTDHKAYLDKNNAYPVECWIPDPSRNENTRYFGTYTGGENVPPVLHVT  238
BAF75216.1      TPKNPTAQSQVMNTDHKAYLDKNNAYPVECWIPDPSRNENTRYFGTYTGGENVPPVLHVT  238
BAF75222.1      TPKNPTAQSQVMNTDHKAYLDKNNAYPVECWIPDPSRNENTRYFGTYTGGENVPPVLHVT  238
BAF75228.1      TPKNPTAQSQVMNTDHKAYLDKNNAYPVECWIPDPSRNENTRYFGTYTGGENVPPVLHVT  238
BAF75246.1      TPKNPTAQSQVMNTDHKAYLDKNNAYPVECWIPDPSRNENTRYFGTYTGGENVPPVLHVT  238
BAF75252.1      TPKNPTAQSQVMNTDHKAYLDKNNAYPVECWIPDPSRNENTRYFGTYTGGENVPPVLHVT  238
BAF75258.1      TPKNPTAQSQVMNTDHKAYLDKNNAYPVECWIPDPSRNENTRYFGTYTGGENVPPVLHVT  238
BAF75276.1      TPKNPTAQSQVMNTDHKAYLDKNNAYPVECWIPDPSRNENTRYFGTYTGGENVPPVLHVT  238
BAF75330.1      TPKNPTAQSQVMNTDHKAYLDKNNAYPVECWIPDPSRNENTRYFGTYTGGENVPPVLHVT  238
BAF03115.1      TPKNPTAQSQVMNTDHKAYLDKNNAYPVECWIPDPSRNENTRYFGTYTGGENVPPVLHVT  238
BAF03120.1      TPKNPTAQSQVMNTDHKAYLDKNNAYPVECWIPDPSRNENTRYFGTYTGGENVPPVLHVT  238
WLD47842.1      TPKNPTAQSQVMNTDHKAYLDKNNAYPVECWIPDPSRNENTRYFGTYTGGENVPPVLHVT  238
WLD47884.1      TPKNPTAQSQVMNTDHKAYLDKNNAYPVECWIPDPSRNENTRYFGTYTGGENVPPVLHVT  238
UTM62101.1      TPKNPTAQSQVMNTDHKAYLDKNNAYPVECWIPDPSRNENTRYFGTYTGGENVPPVLHVT  238
UTM62107.1      TPKNPTAQSQVMNTDHKAYLDKNNAYPVECWIPDPSRNENTRYFGTYTGGENVPPVLHVT  238
UTM62113.1      TPKNPTAQSQVMNTDHKAYLDKNNAYPVECWIPDPSRNENTRYFGTYTGGENVPPVLHVT  238
AFQ31647.1      TPKNPTAQSQVMNTDHKAYLDKNNAYPVECWIPDPSRNENTRYFGTYTGGENVPPVLHVT  238
AFA41892.1      TPKNPTAQSQVMNTDHKAYLDKNNAYPVECWIPDPSRNENTRYFGTYTGGENVPPVLHVT  238
BAG75403.1      TPKNPTAQSQVMNTDHKAYLDKNNAYPVECWIPDPSRNENTRYFGTYTGGENVPPVLHVT  238
BAF75114.1      TPKNPTAQSQVMNTDHKAYLDKNNAYPVECWIPDPSRNENTRYFGTYTGGENVPPVLHVT  238
BAF75120.1      TPKNPTAQSQVMNTDHKAYLDKNNAYPVECWIPDPSRNENTRYFGTYTGGENVPPVLHVT  238
BAF75126.1      TPKNPTAQSQVMNTDHKAYLDKNNAYPVECWIPDPSRNENTRYFGTYTGGENVPPVLHVT  238
BAF75186.1      TPKNPTAQSQVMNTDHKAYLDKNNAYPVECWIPDPSRNENTRYFGTYTGGENVPPVLHVT  238
BAF75288.1      TPKNPTAQSQVMNTDHKAYLDKNNAYPVECWIPDPSRNENTRYFGTYTGGENVPPVLHVT  238
BAF75294.1      TPKNPTAQSQVMNTDHKAYLDKNNAYPVECWIPDPSRNENTRYFGTYTGGENVPPVLHVT  238
BAF75348.1      TPKNPTAQSQVMNTDHKAYLDKNNAYPVECWIPDPSRNENTRYFGTYTGGENVPPVLHVT  238
AFA41881.1      TPKNPTAQSQVMNTDHKAYLDKNNAYPVECWIPDPSRNENTRYFGTYTGGENVPPVLHVT  238
                ***.***  ***:*** ***.********** ***::***:***** ********** :*

ALF06344.1      NTATTVLLDEQGVGPLCKADSLYVSAADICGLFTNSSGTQQWRGLARYFKIRLRKRSVKN  298
QTJ15012.1      NTATTVLLDEQGVGPLCKADSLYVSAADICGLFTNSSGTQQWRGLARYFKIRLRKRSVKN  298
QTJ15120.1      NTATTVLLDEQGVGPLCKADSLYVSAADICGLFTNSSGTQQWRGLARYFKIRLRKRSVKN  298
QHI34657.1      NTATTVLLDEQGVGPLCKADSLYVSAADICGLFTNSSGTQQWRGLARYFKIRLRKRSVKN  298
ARE72390.1      NTATTVLLDEQGVGPLCKADSLYVSAADICGLFTNSSGTQQWRGLARYFKIRLRKRSVKN  298
```

QTJ14970.1	NTATTVLLDEQGVGPLCKADSLYVSAADICGLFTNSSGTQQWRGLARYFKIRLRKRSVKN	298
QTJ14928.1	NTATTVLLDEQGVGPLCKADSLYVSAADICGLFTNSSGTQQWRGLARYFKIRLRKRSVKN	300
AAT47419.1	NTATTVLLDEQGVGPLCKADSLYVSAADICGLFTNSSGTQQWRGLARYFKIRLRKRSVKN	298
AAT47413.1	NTATTVLLDEQGVGPLCKADGLYVSAADICGLFTNSSGTQQWRGLARYFKTRLRKRSVKN	298
AFQ31661.1	NTATTVLLDEQGVGPLCKADSLYVSAADICGLFTNSSGTQQWRGLARYFKIRLRKRSAKN	298
YP_717939.1	NTATTVLLDEQGVGPLCKADSLYVSAADICGLFTNSSGTQQWRGLARYFKIRLRKRSVKN	298
AFK08545.1	NTATTVLLDEQGVGPLCKADSLYVSAADICGLFTNSSGTQQWRGLARYFKIRLRKRSVKN	298
P03088.2	NTATTVLLDEQGVGPLCKADSLYVSAADICGLFTNSSGTQQWRGLARYFKIRLRKRSVKN	298
CAA24299.1	NTATTVLLDEQGVGPLCKADSLYVSAADICGLFTNSSGTQQWRGLARYFKIRLRKRSVKN	298
QTJ14922.1	NTATTVLLDEQGVGPLCKADSLYVSAADICGLFTNSSGTQQWRGLARYFKIRLRKRSVKN	298
AFA41877.1	NTATTVLLDEQGVGPLCKADSLYVSAADICGLFTNSSGTQQWRGLARYFKIRLRKRSVKN	298
AAT47371.1	NTATTVLLDEQGVGPLCKADSLYVSAADICGLFTNSSGTQQWRGLARYFRIRLRKRSVKN	298
AYO46658.1	NTATTVLLDEQGVGPLCKADSLYVSAADICGLFTNSSGTQQWRGLARYFKIRLRKRSVKN	298
AYO46610.1	NTATTVLLDEQGVGPLCKADSLYVSAADICGLFTNSSGTQQWRGLARYFKIRLRKRSVKN	298
AYO46689.1	NTATTVLLDEQGVGPLCKADSLYVSAADICGLFTNSSGTQQWRGLARYFKIRLRKRSVKN	298
AYO46693.1	NTATTVLLDEQGVGPLCKADSLYVSAADICGLFTNSSGTQQWRGLARYFKIRLRKRSVKN	298
BAG75283.1	NTATTVLLDEQGVGPLCKADSLYVSAADICGLFTNSSGTQQWRGLARYFKIRLRKRSVKN	298
BAG75313.1	NTATTVLLDEQGVGPLCKADSLYVSAADICGLFTNSSGTQQWRGLARYFKIRLRKRSVKN	298
BAG75373.1	NTATTVLLDEQGVGPLCKADSLYVSAADICGLFTNSSGTQQWRGLARYFKIRLRKRSVKN	298
BCV68914.1	NTATTVLLDEQGVGPLCKADSLYVSAADICGLFTNSSGTQQWRGLARYFKIRLRKRSVKN	298
BCV68917.1	NTATTVLLDEQGVGPLCKADSLYVSAADICGLFTNSSGTQQWRGLARYFKIRLRKRSVKN	298
BCV68923.1	NTATTVLLDEQGVGPLCKADSLYVSAADICGLFTNSSGTQQWRGLARYFKIRLRKRSVKN	298
AYO46620.1	NTATTVLLDEQGVGPLCKADSLYVSAADICGLFTNSSGTQQWRGLARYFKIRLRKRSVKN	298
AYO46646.1	NTATTVLLDEQGVGPLCKADSLYVSAADICGLFTNSSGTQQWRGLARYFKIRLRKRSVKN	298
AYO46663.1	NTATTVLLDEQGVGPLCKADSLYVSAADICGLFTNSSGTQQWRGLARYFKIRLRKRSVKN	298
AYO46694.1	NTATTVLLDEQGVGPLCKADSLYVSAADICGLFTNSSGTQQWRGLARYFKIRLRKRSVKN	298
BAZ99906.1	NTATTVLLDEQGVGPLCKADSLYVSAADICGLFTNSSGTQQWRGLARYFKIRLRKRSVKN	298
BAZ99912.1	NTATTVLLDEQGVGPLCKADSLYVSAADICGLFTNSSGTQQWRGLARYFKIRLRKRSVKN	298
BAI43576.1	NTATTVLLDEQGVGPLCKADSLYVSAADICGLFTNSSGTQQWRGLARYFKIRLRKRSVKN	298
BAI43594.1	NTATTVLLDEQGVGPLCKADSLYVSAADICGLFTNSSGTQQWRGLARYFKIRLRKRSVKN	298
BAI43606.1	NTATTVLLDEQGVGPLCKADSLYVSAADICGLFTNSSGTQQWRGLARYFKIRLRKRSVKN	298
BAI43624.1	NTATTVLLDEQGVGPLCKADSLYVSAADICGLFTNSSGTQQWRGLARYFKIRLRKRSVKN	298
BAH23450.1	NTATTVLLDEQGVGPLCKADSLYVSAADICGLFTNSSGTQQWRGLARYFKIRLRKRSVKN	298
BAH23456.1	NTATTVLLDEQGVGPLCKADSLYVSAADICGLFTNSSGTQQWRGLARYFKIRLRKRSVKN	298
BAH23462.1	NTATTVLLDEQGVGPLCKADSLYVSAADICGLFTNSSGTQQWRGLARYFKIRLRKRSVKN	298
BAH23468.1	NTATTVLLDEQGVGPLCKADSLYVSAADICGLFTNSSGTQQWRGLARYFKIRLRKRSVKN	298
BAH23474.1	NTATTVLLDEQGVGPLCKADSLYVSAADICGLFTNSSGTQQWRGLARYFKIRLRKRSVKN	298
BAH23480.1	NTATTVLLDEQGVGPLCKADSLYVSAADICGLFTNSSGTQQWRGLARYFKIRLRKRSVKN	298
BAH23486.1	NTATTVLLDEQGVGPLCKADSLYVSAADICGLFTNSSGTQQWRGLARYFKIRLRKRSVKN	298
BAH23492.1	NTATTVLLDEQGVGPLCKADSLYVSAADICGLFTNSSGTQQWRGLARYFKIRLRKRSVKN	298
BAH23498.1	NTATTVLLDEQGVGPLCKADSLYVSAADICGLFTNSSGTQQWRGLARYFKIRLRKRSVKN	298
BAH23504.1	NTATTVLLDEQGVGPLCKADSLYVSAADICGLFTNSSGTQQWRGLARYFKIRLRKRSVKN	298
BAH23510.1	NTATTVLLDEQGVGPLCKADSLYVSAADICGLFTNSSGTQQWRGLARYFKIRLRKRSVKN	298
BAH23516.1	NTATTVLLDEQGVGPLCKADSLYVSAADICGLFTNSSGTQQWRGLARYFKIRLRKRSVKN	298
BAH23522.1	NTATTVLLDEQGVGPLCKADSLYVSAADICGLFTNSSGTQQWRGLARYFKIRLRKRSVKN	298
BAH23528.1	NTATTVLLDEQGVGPLCKADSLYVSAADICGLFTNSSGTQQWRGLARYFKIRLRKRSVKN	298
BAH23534.1	NTATTVLLDEQGVGPLCKADSLYVSAADICGLFTNSSGTQQWRGLARYFKIRLRKRSVKN	298
BAH23540.1	NTATTVLLDEQGVGPLCKADSLYVSAADICGLFTNSSGTQQWRGLARYFKIRLRKRSVKN	298
BAH23546.1	NTATTVLLDEQGVGPLCKADSLYVSAADICGLFTNSSGTQQWRGLARYFKIRLRKRSVKN	298
BAH23552.1	NTATTVLLDEQGVGPLCKADSLYVSAADICGLFTNSSGTQQWRGLARYFKIRLRKRSVKN	298
BAH23558.1	NTATTVLLDEQGVGPLCKADSLYVSAADICGLFTNSSGTQQWRGLARYFKIRLRKRSVKN	298
BAG84488.1	NTATTVLLDEQGVGPLCKADSLYVSAADICGLFTNSSGTQQWRGLARYFKIRLRKRSVKN	298
BAG84494.1	NTATTVLLDEQGVGPLCKADSLYVSAADICGLFTNSSGTQQWRGLARYFKIRLRKRSVKN	298
BAG84500.1	NTATTVLLDEQGVGPLCKADSLYVSAADICGLFTNSSGTQQWRGLARYFKIRLRKRSVKN	298
BAG84506.1	NTATTVLLDEQGVGPLCKADSLYVSAADICGLFTNSSGTQQWRGLARYFKIRLRKRSVKN	298
BAG84512.1	NTATTVLLDEQGVGPLCKADSLYVSAADICGLFTNSSGTQQWRGLARYFKIRLRKRSVKN	298
BAG84518.1	NTATTVLLDEQGVGPLCKADSLYVSAADICGLFTNSSGTQQWRGLARYFKIRLRKRSVKN	298
BAG84524.1	NTATTVLLDEQGVGPLCKADSLYVSAADICGLFTNSSGTQQWRGLARYFKIRLRKRSVKN	298
BAG75163.1	NTATTVLLDEQGVGPLCKADSLYVSAADICGLFTNSSGTQQWRGLARYFKIRLRKRSVKN	298
BAG75169.1	NTATTVLLDEQGVGPLCKADSLYVSAADICGLFTNSSGTQQWRGLARYFKIRLRKRSVKN	298
BAG75175.1	NTATTVLLDEQGVGPLCKADSLYVSAADICGLFTNSSGTQQWRGLARYFKIRLRKRSVKN	298
BAG75181.1	NTATTVLLDEQGVGPLCKADSLYVSAADICGLFTNSSGTQQWRGLARYFKIRLRKRSVKN	298
BAG75187.1	NTATTVLLDEQGVGPLCKADSLYVSAADICGLFTNSSGTQQWRGLARYFKIRLRKRSVKN	298
BAG75193.1	NTATTVLLDEQGVGPLCKADSLYVSAADICGLFTNSSGTQQWRGLARYFKIRLRKRSVKN	298

BAG75199.1 NTATTVLLDEQGVGPLCKADSLYVSAADICGLFTNSSGTQQWRGLARYFKIRLRKRSVKN 298
BAG75241.1 NTATTVLLDEQGVGPLCKADSLYVSAADICGLFTNSSGTQQWRGLARYFKIRLRKRSVKN 298
BAG75247.1 NTATTVLLDEQGVGPLCKADSLYVSAADICGLFTNSSGTQQWRGLARYFKIRLRKRSVKN 298
BAG75253.1 NTATTVLLDEQGVGPLCKADSLYVSAADICGLFTNSSGTQQWRGLARYFKIRLRKRSVKN 298
BAG75265.1 NTATTVLLDEQGVGPLCKADSLYVSAADICGLFTNSSGTQQWRGLARYFKIRLRKRSVKN 298
BAG75289.1 NTATTVLLDEQGVGPLCKADSLYVSAADICGLFTNSSGTQQWRGLARYFKIRLRKRSVKN 298
BAG75295.1 NTATTVLLDEQGVGPLCKADSLYVSAADICGLFTNSSGTQQWRGLARYFKIRLRKRSVKN 298
BAG75307.1 NTATTVLLDEQGVGPLCKADSLYVSAADICGLFTNSSGTQQWRGLARYFKIRLRKRSVKN 298
BAG75325.1 NTATTVLLDEQGVGPLCKADSLYVSAADICGLFTNSSGTQQWRGLARYFKIRLRKRSVKN 298
BAG75331.1 NTATTVLLDEQGVGPLCKADSLYVSAADICGLFTNSSGTQQWRGLARYFKIRLRKRSVKN 298
BAG75337.1 NTATTVLLDEQGVGPLCKADSLYVSAADICGLFTNSSGTQQWRGLARYFKIRLRKRSVKN 298
BAG75343.1 NTATTVLLDEQGVGPLCKADSLYVSAADICGLFTNSSGTQQWRGLARYFKIRLRKRSVKN 298
BAG75397.1 NTATTVLLDEQGVGPLCKADSLYVSAADICGLFTNSSGTQQWRGLARYFKIRLRKRSVKN 298
BAG75421.1 NTATTVLLDEQGVGPLCKADSLYVSAADICGLFTNSSGTQQWRGLARYFKIRLRKRSVKN 298
BAG75427.1 NTATTVLLDEQGVGPLCKADSLYVSAADICGLFTNSSGTQQWRGLARYFKIRLRKRSVKN 298
BAG75433.1 NTATTVLLDEQGVGPLCKADSLYVSAADICGLFTNSSGTQQWRGLARYFKIRLRKRSVKN 298
BAF93349.1 NTATTVLLDEQGVGPLCKADSLYVSAADICGLFTNSSGTQQWRGLARYFKIRLRKRSVKN 298
BAF76166.1 NTATTVLLDEQGVGPLCKADSLYVSAADICGLFTNSSGTQQWRGLARYFKIRLRKRSVKN 298
BAF76172.1 NTATTVLLDEQGVGPLCKADSLYVSAADICGLFTNSSGTQQWRGLARYFKIRLRKRSVKN 298
BAF76178.1 NTATTVLLDEQGVGPLCKADSLYVSAADICGLFTNSSGTQQWRGLARYFKIRLRKRSVKN 298
BAF76184.1 NTATTVLLDEQGVGPLCKADSLYVSAADICGLFTNSSGTQQWRGLARYFKIRLRKRSVKN 298
BAF76190.1 NTATTVLLDEQGVGPLCKADSLYVSAADICGLFTNSSGTQQWRGLARYFKIRLRKRSVKN 298
BAF76202.1 NTATTVLLDEQGVGPLCKADSLYVSAADICGLFTNSSGTQQWRGLARYFKIRLRKRSVKN 298
BAF42985.1 NTATTVLLDEQGVGPLCKADSLYVSAADICGLFTNSSGTQQWRGLARYFKIRLRKRSVKN 298
BAF42991.1 NTATTVLLDEQGVGPLCKADSLYVSAADICGLFTNSSGTQQWRGLARYFKIRLRKRSVKN 298
BAF02951.1 NTATTVLLDEQGVGPLCKADSLYVSAADICGLFTNSSGTQQWRGLARYFKIRLRKRSVKN 298
BAF02963.1 NTATTVLLDEQGVGPLCKADSLYVSAADICGLFTNSSGTQQWRGLARYFKIRLRKRSVKN 298
BAF02969.1 NTATTVLLDEQGVGPLCKADSLYVSAADICGLFTNSSGTQQWRGLARYFKIRLRKRSVKN 298
BAF02981.1 NTATTVLLDEQGVGPLCKADSLYVSAADICGLFTNSSGTQQWRGLARYFKIRLRKRSVKN 298
BAF02987.1 NTATTVLLDEQGVGPLCKADSLYVSAADICGLFTNSSGTQQWRGLARYFKIRLRKRSVKN 298
BAF02993.1 NTATTVLLDEQGVGPLCKADSLYVSAADICGLFTNSSGTQQWRGLARYFKIRLRKRSVKN 298
BAF02999.1 NTATTVLLDEQGVGPLCKADSLYVSAADICGLFTNSSGTQQWRGLARYFKIRLRKRSVKN 298
BAF03005.1 NTATTVLLDEQGVGPLCKADSLYVSAADICGLFTNSSGTQQWRGLARYFKIRLRKRSVKN 298
BAF03011.1 NTATTVLLDEQGVGPLCKADSLYVSAADICGLFTNSSGTQQWRGLARYFKIRLRKRSVKN 298
BAE96065.1 NTATTVLLDEQGVGPLCKADSLYVSAADICGLFTNSSGTQQWRGLARYFKIRLRKRSVKN 298
BAE53666.1 NTATTVLLDEQGVGPLCKADSLYVSAADICGLFTNSSGTQQWRGLARYFKIRLRKRSVKN 298
BAE46623.1 NTATTVLLDEQGVGPLCKADSLYVSAADICGLFTNSSGTQQWRGLARYFKIRLRKRSVKN 298
CAA40235.1 NTATTVLLDEQGVGPLCKADSLYVSAADICGLFTNSSGTQQWRGLARYFKIRLRKRSVKN 298
BAI43570.1 NTATTVLLDEQGVGPLCKADSLYVSAADICGLFTNSSGTQQWRGLARYFKIRLRKRSVKN 298
BAI43582.1 NTATTVLLDEQGVGPLCKADSLYVSAADICGLFTNSSGTQQWRGLARYFKIRLRKRSVKN 298
BAI43612.1 NTATTVLLDEQGVGPLCKADSLYVSAADICGLFTNSSGTQQWRGLARYFKIRLRKRSVKN 298
BAI43618.1 NTATTVLLDEQGVGPLCKADSLYVSAADICGLFTNSSGTQQWRGLARYFKIRLRKRSVKN 298
BAI43630.1 NTATTVLLDEQGVGPLCKADSLYVSAADICGLFTNSSGTQQWRGLARYFKIRLRKRSVKN 298
BAG75301.1 NTATTVLLDEQGVGPLCKADSLYVSAADICGLFTNSSGTQQWRGLARYFKIRLRKRSVKN 298
BAG75349.1 NTATTVLLDEQGVGPLCKADSLYVSAADICGLFTNSSGTQQWRGLARYFKIRLRKRSVKN 298
BAG75367.1 NTATTVLLDEQGVGPLCKADSLYVSAADICGLFTNSSGTQQWRGLARYFKIRLRKRSVKN 298
BAF76196.1 NTATTVLLDEQGVGPLCKADSLYVSAADICGLFTNSSGTQQWRGLARYFKIRLRKRSVKN 298
BAF02975.1 NTATTVLLDEQGVGPLCKADSLYVSAADICGLFTNSSGTQQWRGLARYFKIRLRKRSVKN 298
BAI43600.1 NTATTVLLDEQGVGPLCKADSLYVSAADICGLFTNSSGTQQWRGLARYFKIRLRKRSVKN 298
BAE96071.1 NTATTVLLDEQGVGPLCKADSLYVSAADICGLFTNSSGTQQWRGLARYFKIRLRKRSVKN 298
BAE53660.1 NTATTVLLDEQGVGPLCKADSLYVSAADICGLFTNSSGTQQWRGLARYFKIRLRKRSVKN 298
BCV68926.1 NTATTVLLDEQGVGPLCKADSLYVSAADICGLFTNSSGTQQWRGLARYFKIRLRKRSVKN 298
AYO46647.1 NTATTVLLDEQGVGPLCKADSLYVSAADIWGLFTNSSGTQQWRGLARYFKIRLRKRSVKN 298
BAR80134.1 NTATTVLLDEQGVGPLCKADSLYVSAADICGLFTNSSGTQQWRGLARYFKIRLRKRSVKN 298
BAR80140.1 NTATTVLLDEQGVGPLCKADSLYVSAADICGLFTNSSGTQQWRGLARYFKIRLRKRSVKN 298
BAI43588.1 NTATTVLLDEQGVGPLCKADSLYVSAADICGLFTNSSGTQQWRGLARYFKIRLRKRSVKN 298
BAG75361.1 NTATTVLLDEQGVGPLCKADSLYVSAADICGLFTNSSGTQQWRGLARYFKIRLRKRSVKN 298
BAE53642.1 NTATTVLLDEQGVGPLCKADSLYVSAADICGLFTNSSGTQQWRGLARYFKIRLRKRSVKN 298
BAE53648.1 NTATTVLLDEQGVGPLCKADSLYVSAADICGLFTNSSGTQQWRGLARYFKIRLRKRSVKN 298
AFA41903.1 NTATTVLLDEQGVGPLCKADSLYVSAADICGLFTNSSGTQQWRGLARYFKIRLRKRSVKN 298
AFA41918.1 NTATTVLLDEQGVGPLCKADSLYVSAADICGLFTNSSGTQQWRGLARYFKIRLRKRSVKN 298
BAF42937.1 NTATTVLLDEQGVGPLCKADSLYVSAADICGLFTNSSGTQQWRGLARYFKIRLRKRSVKN 298
BAF42997.1 NTATTVLLDEQGVGPLCKADSLYVSAADICGLFTNSSGTQQWRGLARYFKIRLRKRSVKN 298
WLD47902.1 NTATTVLLDEQGVGPLCKADSLYVSAADICGLFTNSSGTQQWRGLARYFKIRLRKRSVKN 298

WLD47938.1	NTATTVLLDEQGVGPLCKADSLYVSAADICGLFTNSSGTQQWRGLARYFKIRLRKRSVKN	298
WLD47980.1	NTATTVLLDEQGVGPLCKADSLYVSAADICGLFTNSSGTQQWRGLARYFKIRLRKRSVKN	298
UTM61891.1	NTATTVLLDEQGVGPLCKADSLYVSAADICGLFTNSSGTQQWRGLARYFKIRLRKRSVKN	298
UTM61897.1	NTATTVLLDEQGVGPLCKADSLYVSAADICGLFTNSSGTQQWRGLARYFKIRLRKRSVKN	298
UTM61903.1	NTATTVLLDEQGVGPLCKADSLYVSAADICGLFTNSSGTQQWRGLARYFKIRLRKRSVKN	298
UTM62215.1	NTATTVLLDEQGVGPLCKADSLYVSAADICGLFTNSSGTQQWRGLARYFKIRLRKRSVKN	298
UTM62221.1	NTATTVLLDEQGVGPLCKADSLYVSAADICGLFTNSSGTQQWRGLARYFKIRLRKRSVKN	298
UTM62275.1	NTATTVLLDEQGVGPLCKADSLYVSAADICGLFTNSSGTQQWRGLARYFKIRLRKRSVKN	298
UTM62281.1	NTATTVLLDEQGVGPLCKADSLYVSAADICGLFTNSSGTQQWRGLARYFKIRLRKRSVKN	298
UTM62329.1	NTATTVLLDEQGVGPLCKADSLYVSAADICGLFTNSSGTQQWRGLARYFKIRLRKRSVKN	298
UTM62335.1	NTATTVLLDEQGVGPLCKADSLYVSAADICGLFTNSSGTQQWRGLARYFKIRLRKRSVKN	298
UTM62383.1	NTATTVLLDEQGVGPLCKADSLYVSAADICGLFTNSSGTQQWRGLARYFKIRLRKRSVKN	298
UTM62395.1	NTATTVLLDEQGVGPLCKADSLYVSAADICGLFTNSSGTQQWRGLARYFKIRLRKRSVKN	298
UTM62443.1	NTATTVLLDEQGVGPLCKADSLYVSAADICGLFTNSSGTQQWRGLARYFKIRLRKRSVKN	298
UTM62449.1	NTATTVLLDEQGVGPLCKADSLYVSAADICGLFTNSSGTQQWRGLARYFKIRLRKRSVKN	298
UTM62467.1	NTATTVLLDEQGVGPLCKADSLYVSAADICGLFTNSSGTQQWRGLARYFKIRLRKRSVKN	298
UTM62545.1	NTATTVLLDEQGVGPLCKADSLYVSAADICGLFTNSSGTQQWRGLARYFKIRLRKRSVKN	298
UTM62551.1	NTATTVLLDEQGVGPLCKADSLYVSAADICGLFTNSSGTQQWRGLARYFKIRLRKRSVKN	298
UTM62587.1	NTATTVLLDEQGVGPLCKADSLYVSAADICGLFTNSSGTQQWRGLARYFKIRLRKRSVKN	298
UTM62593.1	NTATTVLLDEQGVGPLCKADSLYVSAADICGLFTNSSGTQQWRGLARYFKIRLRKRSVKN	298
UTM62599.1	NTATTVLLDEQGVGPLCKADSLYVSAADICGLFTNSSGTQQWRGLARYFKIRLRKRSVKN	298
QUW04850.1	NTATTVLLDEQGVGPLCKADSLYVSAADICGLFTNSSGTQQWRGLARYFKIRLRKRSVKN	298
QTJ14976.1	NTATTVLLDEQGVGPLCKADSLYVSAADICGLFTNSSGTQQWRGLARYFKIRLRKRSVKN	298
QTJ15000.1	NTATTVLLDEQGVGPLCKADSLYVSAADICGLFTNSSGTQQWRGLARYFKIRLRKRSVKN	298
QTJ15054.1	NTATTVLLDEQGVGPLCKADSLYVSAADICGLFTNSSGTQQWRGLARYFKIRLRKRSVKN	298
QTJ15072.1	NTATTVLLDEQGVGPLCKADSLYVSAADICGLFTNSSGTQQWRGLARYFKIRLRKRSVKN	298
QTJ15084.1	NTATTVLLDEQGVGPLCKADSLYVSAADICGLFTNSSGTQQWRGLARYFKIRLRKRSVKN	298
QTJ15090.1	NTATTVLLDEQGVGPLCKADSLYVSAADICGLFTNSSGTQQWRGLARYFKIRLRKRSVKN	298
QTJ15108.1	NTATTVLLDEQGVGPLCKADSLYVSAADICGLFTNSSGTQQWRGLARYFKIRLRKRSVKN	298
QTJ15114.1	NTATTVLLDEQGVGPLCKADSLYVSAADICGLFTNSSGTQQWRGLARYFKIRLRKRSVKN	298
QTJ15132.1	NTATTVLLDEQGVGPLCKADSLYVSAADICGLFTNSSGTQQWRGLARYFKIRLRKRSVKN	298
QTJ15138.1	NTATTVLLDEQGVGPLCKADSLYVSAADICGLFTNSSGTQQWRGLARYFKIRLRKRSVKN	298
AYO46612.1	NTATTVLLDEQGVGPLCKADSLYVSAADICGLFTNSSGTQQWRGLARYFKIRLRKRSVKN	298
AYO46613.1	NTATTVLLDEQGVGPLCKADSLYVSAADICGLFTNSSGTQQWRGLARYFKIRLRKRSVKN	298
AYO46617.1	NTATTVLLDEQGVGPLCKADSLYVSAADICGLFTNSSGTQQWRGLARYFKIRLRKRSVKN	298
AYO46621.1	NTATTVLLDEQGVGPLCKADSLYVSAADICGLFTNSSGTQQWRGLARYFKIRLRKRSVKN	298
AYO46622.1	NTATTVLLDEQGVGPLCKADSLYVSAADICGLFTNSSGTQQWRGLARYFKIRLRKRSVKN	298
AYO46623.1	NTATTVLLDEQGVGPLCKADSLYVSAADICGLFTNSSGTQQWRGLARYFKIRLRKRSVKN	298
AYO46624.1	NTATTVLLDEQGVGPLCKADSLYVSAADICGLFTNSSGTQQWRGLARYFKIRLRKRSVKN	298
AYO46629.1	NTATTVLLDEQGVGPLCKADSLYVSAADICGLFTNSSGTQQWRGLARYFKIRLRKRSVKN	298
AYO46632.1	NTATTVLLDEQGVGPLCKADSLYVSAADICGLFTNSSGTQQWRGLARYFKIRLRKRSVKN	298
AYO46635.1	NTATTVLLDEQGVGPLCKADSLYVSAADICGLFTNSSGTQQWRGLARYFKIRLRKRSVKN	298
AYO46638.1	NTATTVLLDEQGVGPLCKADSLYVSAADICGLFTNSSGTQQWRGLARYFKIRLRKRSVKN	298
AYO46641.1	NTATTVLLDEQGVGPLCKADSLYVSAADICGLFTNSSGTQQWRGLARYFKIRLRKRSVKN	298
AYO46642.1	NTATTVLLDEQGVGPLCKADSLYVSAADICGLFTNSSGTQQWRGLARYFKIRLRKRSVKN	298
AYO46644.1	NTATTVLLDEQGVGPLCKADSLYVSAADICGLFTNSSGTQQWRGLARYFKIRLRKRSVKN	298
AYO46651.1	NTATTVLLDEQGVGPLCKADSLYVSAADICGLFTNSSGTQQWRGLARYFKIRLRKRSVKN	298
AYO46652.1	NTATTVLLDEQGVGPLCKADSLYVSAADICGLFTNSSGTQQWRGLARYFKIRLRKRSVKN	298
AYO46659.1	NTATTVLLDEQGVGPLCKADSLYVSAADICGLFTNSSGTQQWRGLARYFKIRLRKRSVKN	298
AYO46660.1	NTATTVLLDEQGVGPLCKADSLYVSAADICGLFTNSSGTQQWRGLARYFKIRLRKRSVKN	298
AYO46662.1	NTATTVLLDEQGVGPLCKADSLYVSAADICGLFTNSSGTQQWRGLARYFKIRLRKRSVKN	298
AYO46665.1	NTATTVLLDEQGVGPLCKADSLYVSAADICGLFTNSSGTQQWRGLARYFKIRLRKRSVKN	298
AYO46670.1	NTATTVLLDEQGVGPLCKADSLYVSAADICGLFTNSSGTQQWRGLARYFKIRLRKRSVKN	298
AYO46672.1	NTATTVLLDEQGVGPLCKADSLYVSAADICGLFTNSSGTQQWRGLARYFKIRLRKRSVKN	298
AYO46676.1	NTATTVLLDEQGVGPLCKADSLYVSAADICGLFTNSSGTQQWRGLARYFKIRLRKRSVKN	298
AYO46677.1	NTATTVLLDEQGVGPLCKADSLYVSAADICGLFTNSSGTQQWRGLARYFKIRLRKRSVKN	298
AYO46679.1	NTATTVLLDEQGVGPLCKADSLYVSAADICGLFTNSSGTQQWRGLARYFKIRLRKRSVKN	298
AYO46682.1	NTATTVLLDEQGVGPLCKADSLYVSAADICGLFTNSSGTQQWRGLARYFKIRLRKRSVKN	298
AYO46691.1	NTATTVLLDEQGVGPLCKADSLYVSAADICGLFTNSSGTQQWRGLARYFKIRLRKRSVKN	298
AYO46692.1	NTATTVLLDEQGVGPLCKADSLYVSAADICGLFTNSSGTQQWRGLARYFKIRLRKRSVKN	298
AUF70040.1	NTATTVLLDEQGVGPLCKADSLYVSAADICGLFTNSSGTQQWRGLARYFKIRLRKRSVKN	298
SOH95722.1	NTATTVLLDEQGVGPLCKADSLYVSAADICGLFTNSSGTQQWRGLARYFKIRLRKRSVKN	298
SOI09711.1	NTATTVLLDEQGVGPLCKADSLYVSAADICGLFTNSSGTQQWRGLARYFKIRLRKRSVKN	298
APG38208.1	NTATTVLLDEQGVGPLCKADSLYVSAADICGLFTNSSGTQQWRGLARYFKIRLRKRSVKN	298

ALF06332.1 NTATTVLLDEQGVGPLCKADSLYVSAADICGLFTNSSGTQQWRGLARYFKIRLRKRSVKN 298
ALF06341.1 NTATTVLLDEQGVGPLCKADSLYVSAADICGLFTNSSGTQQWRGLARYFKIRLRKRSVKN 298
BAR80122.1 NTATTVLLDEQGVGPLCKADSLYVSAADICGLFTNSSGTQQWRGLARYFKIRLRKRSVKN 298
BAR80128.1 NTATTVLLDEQGVGPLCKADSLYVSAADICGLFTNSSGTQQWRGLARYFKIRLRKRSVKN 298
AJG42164.1 NTATTVLLDEQGVGPLCKADSLYVSAADICGLFTNSSGTQQWRGLARYFKIRLRKRSVKN 298
AHU87340.1 NTATTVLLDEQGVGPLCKADSLYVSAADICGLFTNSSGTQQWRGLARYFKIRLRKRSVKN 298
AHU87341.1 NTATTVLLDEQGVGPLCKADSLYVSAADICGLFTNSSGTQQWRGLARYFKIRLRKRSVKN 298
AHU87368.1 NTATTVLLDEQGVGPLCKADSLYVSAADICGLFTNSSGTQQWRGLARYFKIRLRKRSVKN 298
AHU87369.1 NTATTVLLDEQGVGPLCKADSLYVSAADICGLFTNSSGTQQWRGLARYFKIRLRKRSVKN 298
AFQ31636.1 NTATTVLLDEQGVGPLCKADSLYVSAADICGLFTNSSGTQQWRGLARYFKIRLRKRSVKN 298
AFQ31637.1 NTATTVLLDEQGVGPLCKADSLYVSAADICGLFTNSSGTQQWRGLARYFKIRLRKRSVKN 298
AFQ31639.1 NTATTVLLDEQGVGPLCKADSLYVSAADICGLFTNSSGTQQWRGLARYFKIRLRKRSVKN 298
AFQ31640.1 NTATTVLLDEQGVGPLCKADSLYVSAADICGLFTNSSGTQQWRGLARYFKIRLRKRSVKN 298
AFQ31641.1 NTATTVLLDEQGVGPLCKADSLYVSAADICGLFTNSSGTQQWRGLARYFKIRLRKRSVKN 298
AFQ31644.1 NTATTVLLDEQGVGPLCKADSLYVSAADICGLFTNSSGTQQWRGLARYFKIRLRKRSVKN 298
AFQ31648.1 NTATTVLLDEQGVGPLCKADSLYVSAADICGLFTNSSGTQQWRGLARYFKIRLRKRSVKN 298
AFQ31657.1 NTATTVLLDEQGVGPLCKADSLYVSAADICGLFTNSSGTQQWRGLARYFKIRLRKRSVKN 298
AFA41882.1 NTATTVLLDEQGVGPLCKADSLYVSAADICGLFTNSSGTQQWRGLARYFKIRLRKRSVKN 298
AFA41887.1 NTATTVLLDEQGVGPLCKADSLYVSAADICGLFTNSSGTQQWRGLARYFKIRLRKRSVKN 298
AFA41890.1 NTATTVLLDEQGVGPLCKADSLYVSAADICGLFTNSSGTQQWRGLARYFKIRLRKRSVKN 298
AFA41891.1 NTATTVLLDEQGVGPLCKADSLYVSAADICGLFTNSSGTQQWRGLARYFKIRLRKRSVKN 298
AFA41895.1 NTATTVLLDEQGVGPLCKADSLYVSAADICGLFTNSSGTQQWRGLARYFKIRLRKRSVKN 298
AFA41898.1 NTATTVLLDEQGVGPLCKADSLYVSAADICGLFTNSSGTQQWRGLARYFKIRLRKRSVKN 298
AFA41901.1 NTATTVLLDEQGVGPLCKADSLYVSAADICGLFTNSSGTQQWRGLARYFKIRLRKRSVKN 298
AFA41902.1 NTATTVLLDEQGVGPLCKADSLYVSAADICGLFTNSSGTQQWRGLARYFKIRLRKRSVKN 298
AFA41910.1 NTATTVLLDEQGVGPLCKADSLYVSAADICGLFTNSSGTQQWRGLARYFKIRLRKRSVKN 298
AFA41914.1 NTATTVLLDEQGVGPLCKADSLYVSAADICGLFTNSSGTQQWRGLARYFKIRLRKRSVKN 298
AFA41917.1 NTATTVLLDEQGVGPLCKADSLYVSAADICGLFTNSSGTQQWRGLARYFKIRLRKRSVKN 298
AEO89562.1 NTATTVLLDEQGVGPLCKADSLYVSAADICGLFTNSSGTQQWRGLARYFKIRLRKRSVKN 298
AEO89568.1 NTATTVLLDEQGVGPLCKADSLYVSAADICGLFTNSSGTQQWRGLARYFKIRLRKRSVKN 298
AEO89586.1 NTATTVLLDEQGVGPLCKADSLYVSAADICGLFTNSSGTQQWRGLARYFKIRLRKRSVKN 298
AEO89598.1 NTATTVLLDEQGVGPLCKADSLYVSAADICGLFTNSSGTQQWRGLARYFKIRLRKRSVKN 298
AEO89621.1 NTATTVLLDEQGVGPLCKADSLYVSAADICGLFTNSSGTQQWRGLARYFKIRLRKRSVKN 298
AEK21505.1 NTATTVLLDEQGVGPLCKADSLYVSAADICGLFTNSSGTQQWRGLARYFKIRLRKRSVKN 298
BAG84458.1 NTATTVLLDEQGVGPLCKADSLYVSAADICGLFTNSSGTQQWRGLARYFKIRLRKRSVKN 298
BAG84470.1 NTATTVLLDEQGVGPLCKADSLYVSAADICGLFTNSSGTQQWRGLARYFKIRLRKRSVKN 298
BAG84482.1 NTATTVLLDEQGVGPLCKADSLYVSAADICGLFTNSSGTQQWRGLARYFKIRLRKRSVKN 298
BAG75319.1 NTATTVLLDEQGVGPLCKADSLYVSAADICGLFTNSSGTQQWRGLARYFKIRLRKRSVKN 298
BAG75355.1 NTATTVLLDEQGVGPLCKADSLYVSAADICGLFTNSSGTQQWRGLARYFKIRLRKRSVKN 298
BAG75391.1 NTATTVLLDEQGVGPLCKADSLYVSAADICGLFTNSSGTQQWRGLARYFKIRLRKRSVKN 298
BAG75439.1 NTATTVLLDEQGVGPLCKADSLYVSAADICGLFTNSSGTQQWRGLARYFKIRLRKRSVKN 298
BAF93301.1 NTATTVLLDEQGVGPLCKADSLYVSAADICGLFTNSSGTQQWRGLARYFKIRLRKRSVKN 298
BAF93331.1 NTATTVLLDEQGVGPLCKADSLYVSAADICGLFTNSSGTQQWRGLARYFKIRLRKRSVKN 298
BAF93355.1 NTATTVLLDEQGVGPLCKADSLYVSAADICGLFTNSSGTQQWRGLARYFKIRLRKRSVKN 298
BAF76208.1 NTATTVLLDEQGVGPLCKADSLYVSAADICGLFTNSSGTQQWRGLARYFKIRLRKRSVKN 298
BAF42877.1 NTATTVLLDEQGVGPLCKADSLYVSAADICGLFTNSSGTQQWRGLARYFKIRLRKRSVKN 298
BAF42883.1 NTATTVLLDEQGVGPLCKADSLYVSAADICGLFTNSSGTQQWRGLARYFKIRLRKRSVKN 298
BAF42889.1 NTATTVLLDEQGVGPLCKADSLYVSAADICGLFTNSSGTQQWRGLARYFKIRLRKRSVKN 298
BAF42961.1 NTATTVLLDEQGVGPLCKADSLYVSAADICGLFTNSSGTQQWRGLARYFKIRLRKRSVKN 298
BAF42967.1 NTATTVLLDEQGVGPLCKADSLYVSAADICGLFTNSSGTQQWRGLARYFKIRLRKRSVKN 298
BAF42973.1 NTATTVLLDEQGVGPLCKADSLYVSAADICGLFTNSSGTQQWRGLARYFKIRLRKRSVKN 298
BAF43003.1 NTATTVLLDEQGVGPLCKADSLYVSAADICGLFTNSSGTQQWRGLARYFKIRLRKRSVKN 298
BAF43027.1 NTATTVLLDEQGVGPLCKADSLYVSAADICGLFTNSSGTQQWRGLARYFKIRLRKRSVKN 298
BAF43033.1 NTATTVLLDEQGVGPLCKADSLYVSAADICGLFTNSSGTQQWRGLARYFKIRLRKRSVKN 298
ABI94659.1 NTATTVLLDEQGVGPLCKADSLYVSAADICGLFTNSSGTQQWRGLARYFKIRLRKRSVKN 298
ABI94683.1 NTATTVLLDEQGVGPLCKADSLYVSAADICGLFTNSSGTQQWRGLARYFKIRLRKRSVKN 298
ABI94719.1 NTATTVLLDEQGVGPLCKADSLYVSAADICGLFTNSSGTQQWRGLARYFKIRLRKRSVKN 298
BAE96053.1 NTATTVLLDEQGVGPLCKADSLYVSAADICGLFTNSSGTQQWRGLARYFKIRLRKRSVKN 298
BAE96059.1 NTATTVLLDEQGVGPLCKADSLYVSAADICGLFTNSSGTQQWRGLARYFKIRLRKRSVKN 298
ABC18005.1 NTATTVLLDEQGVGPLCKADSLYVSAADICGLFTNSSGTQQWRGLARYFKIRLRKRSVKN 298
AAT47377.1 NTATTVLLDEQGVGPLCKADSLYVSAADICGLFTNSSGTQQWRGLARYFKIRLRKRSVKN 298
AAT47383.1 NTATTVLLDEQGVGPLCKADSLYVSAADICGLFTNSSGTQQWRGLARYFKIRLRKRSVKN 298
AAT47405.1 NTATTVLLDEQGVGPLCKADSLYVSAADICGLFTNSSGTQQWRGLARYFKIRLRKRSVKN 298
CAA40239.1 NTATTVLLDEQGVGPLCKADSLYVSAADICGLFTNSSGTQQWRGLARYFKIRLRKRSVKN 298

QTJ14940.1	NTATTVLLDEQGVGPLCKADSLYVSAADICGLFTNSSGTQQWRGLARYFKIRLRKRSVKN	298
ALF06317.1	NTATTVLLDEQGVGPLCKADSLYVSAADICGLFTNSSGTQQWRGLARYFKIRLRKRSVKN	298
ALF06307.1	NTATTVLLDEQGVGPLCKADSLYVSAADICGLFTNSSGTQQWRGLARYFKIRLRKRSVKN	298
ALF06297.1	NTATTVLLDEQGVGPLCKADSLYVSXADICGLFTNSSGTQQWRGLARYFKIRLRKRSVKN	298
ALF06350.1	NTATTVLLDEQGVGPLCKADSLYVSAADICGLFTNSSGTQQWRGLARYFKIRLRKRSVKN	298
AAT47431.1	NTATTVLLDEQGVGPLCKADSLYVSAADICGLFTNSSGTQQWRGLARYFKIRLRKRSVKN	298
QTJ15006.1	NTATTVLLDEQGVGPLCKADSLYVSAADICGLFTNSSGTQQWRGLARYFKIRLRKRSVKN	298
AYO46667.1	NTATTVLLDEQGVGPLCKADSLYVSAADICGLFTNSSGTQQWRGLARYFKIRLRKRSVKN	298
WLD47956.1	NTATTVLLDEQGVGPLCKADSLYVSAADICGLFTNSSGTQQWRGLARYFKIRLRKRSVKN	298
QTJ15150.1	NTATTVLLDEQGVGPLCKADSLYVSAADICGLFTNSSGTQQWRGLARYFKIRLRKRSVKN	298
UTM62389.1	NTATTVLLDEQGVGPLCKADSLYVSAADICGLFTNSSGTQQWRGLARYFKIRLRKRSVKN	298
ABI94701.1	NTATTVLLDEQGVGPLCKADSLYVSAADICGLFTNSSGTQQWRGLARYFKIRLRKRSVKN	298
BAF02945.1	NTATTVLLDEQGVGPLCKADSLYVSAADICGLFTNSSGTQQWRGLARYFKIRLRKRSVKN	298
CAA40243.1	NTATTVLLDEQGVGPLCKADSLYVSAADICGLFTNSSGTQQWRGLARYFKIRLRKRSVKN	298
ABI94725.1	NTATTVLLDEQGVGPLCKADSLYVSAADICGLFTNSSGTQQWRGLARYFKIRLRKRSVKN	298
AFQ31660.1	NTATTVLLDEQGVGPLCKADSLYVSAADICGLFTNSSGTRQWRGLARYFKIRLRKRSVKN	298
ALF06353.1	NTATTVLLDEQGVGPLCKADSLYVSAADICGLFTNSSGTQQWRGLARYFKIRLRKRSVKN	298
ALF06312.1	NTATTVLLDEQGVGPLCKADSLYVSAADICGLFTNSSGTQQWRGLARYFKIRLRKRSVKN	298
QEJ80721.1	NTATTVLLDEQGVGPLCKADSLYVSAADICGLFTNSSGTQQWRGLARYFKIRLRKRSVKN	298
AYO46614.1	NTATTVLLDEQGVGPLCKADSLYVSAADICGLFTNSSGTQQWRGLARYFKIRLRKRSVKN	298
AYO46619.1	NTATTVLLDEQGVGPLCKADSLYVSAADICGLFTNSSGTQQWRGLARYFKIRLRKRSVKN	298
AYO46625.1	NTATTVLLDEQGVGPLCKADSLYVSAADICGLFTNSSGTQQWRGLARYFKIRLRKRSVKN	298
AYO46626.1	NTATTVLLDEQGVGPLCKADSLYVSAADICGLFTNSSGTQQWRGLARYFKIRLRKRSVKN	298
AYO46628.1	NTATTVLLDEQGVGPLCKADSLYVSAADICGLFTNSSGTQQWRGLARYFKIRLRKRSVKN	298
AYO46633.1	NTATTVLLDEQGVGPLCKADSLYVSAADICGLFTNSSGTQQWRGLARYFKIRLRKRSVKN	298
AYO46636.1	NTATTVLLDEQGVGPLCKADSLYVSAADICGLFTNSSGTQQWRGLARYFKIRLRKRSVKN	298
AYO46637.1	NTATTVLLDEQGVGPLCKADSLYVSAADICGLFTNSSGTQQWRGLARYFKIRLRKRSVKN	298
AYO46640.1	NTATTVLLDEQGVGPLCKADSLYVSAADICGLFTNSSGTQQWRGLARYFKIRLRKRSVKN	298
AYO46648.1	NTATTVLLDEQGVGPLCKADSLYVSAADICGLFTNSSGTQQWRGLARYFKIRLRKRSVKN	298
AYO46650.1	NTATTVLLDEQGVGPLCKADSLYVSAADICGLFTNSSGTQQWRGLARYFKIRLRKRSVKN	298
AYO46653.1	NTATTVLLDEQGVGPLCKADSLYVSAADICGLFTNSSGTQQWRGLARYFKIRLRKRSVKN	298
AYO46654.1	NTATTVLLDEQGVGPLCKADSLYVSAADICGLFTNSSGTQQWRGLARYFKIRLRKRSVKN	298
AYO46671.1	NTATTVLLDEQGVGPLCKADSLYVSAADICGLFTNSSGTQQWRGLARYFKIRLRKRSVKN	298
AYO46673.1	NTATTVLLDEQGVGPLCKADSLYVSAADICGLFTNSSGTQQWRGLARYFKIRLRKRSVKN	298
AYO46674.1	NTATTVLLDEQGVGPLCKADSLYVSAADICGLFTNSSGTQQWRGLARYFKIRLRKRSVKN	298
AYO46675.1	NTATTVLLDEQGVGPLCKADSLYVSAADICGLFTNSSGTQQWRGLARYFKIRLRKRSVKN	298
AYO46680.1	NTATTVLLDEQGVGPLCKADSLYVSAADICGLFTNSSGTQQWRGLARYFKIRLRKRSVKN	298
AYO46681.1	NTATTVLLDEQGVGPLCKADSLYVSAADICGLFTNSSGTQQWRGLARYFKIRLRKRSVKN	298
AYO46684.1	NTATTVLLDEQGVGPLCKADSLYVSAADICGLFTNSSGTQQWRGLARYFKIRLRKRSVKN	298
AYO46687.1	NTATTVLLDEQGVGPLCKADSLYVSAADICGLFTNSSGTQQWRGLARYFKIRLRKRSVKN	298
AEO89574.1	NTATTVLLDEQGVGPLCKADSLYVSAADICGLFTNSSGTQQWRGLARYFKIRLRKRSVKN	298
BAF42979.1	NTATTVLLDEQGVGPLCKADSLYVSAADICGLFTNSSGTQQWRGLARYFKIRLRKRSVKN	298
BAF43009.1	NTATTVLLDEQGVGPLCKADSLYVSAADICGLFTNSSGTQQWRGLARYFKIRLRKRSVKN	298
AYO46678.1	NTATTVLLDEQGVGPLCKADSLYVSAADICGLFTNSSGTQQWRGLARYFKIRLRKRSVKN	298
UTM62479.1	NTATTVLLDEQGVGPLCKADSLYVSAADICGLFTNSSGTQQWRGLARYFKIRLRKRSVKN	298
QTJ14964.1	NTATTVLLDEQGVGPLCKADSLYVSAADICGLFTNSSGTQQWRGLARYFKIRLRKRSVKN	298
QTJ15036.1	NTATTVLLDEQGVGPLCKADSLYVSAADICGLFTNSSGTQQWRGLARYFKIRLRKRSVKN	298
QTJ15066.1	NTATTVLLDEQGVGPLCKADSLYVSAADICGLFTNSSGTQQWRGLARYFKIRLRKRSVKN	298
QTJ15078.1	NTATTVLLDEQGVGPLCKADSLYVSAADICGLFTNSSGTQQWRGLARYFKIRLRKRSVKN	298
QTJ15096.1	NTATTVLLDEQGVGPLCKADSLYVSAADICGLFTNSSGTQQWRGLARYFKIRLRKRSVKN	298
AQM40276.1	NTATTVLLDEQGVGPLCKADSLYVSAADICGLFTNSSGTQQWRGLARYFKIRLRKRSVKN	298
ABI94689.1	NTATTVLLDEQGVGPLCKADSLYVSAADICGLFTNSSGTQQWRGLARYFKIRLRKRSVKN	298
ABI94671.1	NTATTVLLDEQGVGPLCKADSLYVSAADICGLFTNSSGTQQWRGLARYFKIRLRKRSVKN	298
AYO46688.1	NTATTVLLDEQGVGPLCKADSLYVSAADICGLFTNSSGTQQWRGLARYFKIRLRKRSVKN	298
QTJ15060.1	NTATTVLLDEQGVGPLCKADSLYVSAADICGLFTNSSGTQQWRGLARYFKIRLRKRSVKN	298
AFQ31642.1	NTATTVLLDEQGVGPLCKADSLYVSAADICGLFTNSSGTQQWRGLARYFKIRLRKRSVKN	298
AYO46616.1	NTATTVLLDEQGVGPLCKADSLYVSAADICGLFTNSSGTQQWRGLARYFKIRLRKRSVKN	298
CAA79594.1	NTATTVLLDEQGVGPLCKADSLYVSAADICGLFTNSSGTQQWRGLARYFKIRLRKRSVKN	298
WLD47818.1	NTATTVLLDEQGVGPLCKADSLYVSAADICGLFTNSSGTQQWRGLARYFKIRLRKRSVKN	298
UTM62155.1	NTATTVLLDEQGVGPLCKADSLYVSAADICGLFTNSSGTQQWRGLARYFKIRLRKRSVKN	298
AFA41907.1	NTATTVLLDEQGVGPLCKADSLYVSAADICGLFTNSSGTQQWRGLARYFKIRLRKRSVKN	298
WLD47944.1	NTATTVLLDEQGVGPLCKADSLYVSAADICGLFTNSSGTQQWRGLARYFKIRLRKRSVKN	298
AYO46668.1	NTATTVLLDEQGVGPLCKADSLYVSAADICGLFTNSSGTQQWRGLARYFKIRLRKRSVKN	298
AFQ31651.1	NTATTVLLDEQGVGPLCKADSLYVSAADICGLFTNSSGTQQWRGLARYFKIRLRKRSVKN	298

```
QTJ14958.1        NTATTVLLDEQGVGPLCKADSLYVSAADICGLFTNSSGTQQWRGLARYFKIRLRKRSVKN  298
AYO46618.1        NTATTVLLDEQGVGPLCKADSLYVSAADICGLFTNSSGTQQWRGLARYFKIRLRKRSVKN  298
QTJ15018.1        NTATTVLLDEQGVGPLCKADSLYVSAADICGLFTNSSGTQQWRGLARYFKIRLRKRSVKN  298
AYO46609.1        NTATTVLLDEQGVGPLCKADSLYVSAADICGLFTNSSGTQQWRGLARYFKIRLRKRSVKN  298
ALF06356.1        NTATTVLLDEQGVGPLCKADSLYVSAADICGLFTNSSGTQQWRGLARYFKIRLKKRSVKN  298
AFQ31646.1        NTATTVLLDEQGVGPLCKADSLYVSAADICGLFTNSSGTQQWRGLARYFKIRLRKRSVKN  298
AFQ31659.1        NAATTVLLDEQGVGPLCKADSLYVSAADICGLFTNSSGTQQWRGLARYFKIRLRKRSVKN  298
BAF02957.1        NTATTVLLDEQGVGPLCKADSLYVSAADICGLFTNSSGTQQWRGLARYFKIRLRKRSVKN  298
AAT47365.1        NTATTVLLDEQGVGPLCKADSLYVSAADICGLFTNSSGTQQWRGLARYFKIRLRKRSVKN  298
AAT47389.1        NTATTVLLDEQGVGPLCKADSLYVSAADICGLFTNSSGTQQWRGLARYFKIRLRKRSVKN  298
AAT47395.1        NTATTVLLDEQGVGPLCKADSLYVSAADICGLFTNSSGTQQWRGLARYFKIRLRKRSVKN  298
AAT47401.1        NTATTVLLDEQGVGPLCKADSLYVSAADICGLFTNSSGTQQWRGLARYFKIRLRKRSVKN  298
CAA24307.1        NTATTVLLDEQGVGPLCKADSLYVSAADICGLFTNSSGTQQWRGLARYFKIRLRKRSVKN  298
AAT47425.1        NTATTVLLDEQGVGPLCKADSLYVSAADICGLFTNSSGTQQWRGLARYFKIRLRKRSVKN  298
AFA41880.1        NTATTVLLDEQGVGPLCKADSLYVSAADICGLFTNSSGTQQWRGLARYFKIRLRKRSVKN  298
WLD47968.1        NTATTVLLDEQGVGPLCKADSLYVSAADICGLFTNSSGTQQWRGLARYFKIRLRKRSVKN  298
AFA41920.1        NTATTVLLDEQGVGPLCKADSLYVSAADICGLFTNSSGTQQWRGLARYFKIRLRKRSVKN  298
CBX88302.1        NTATTVLLDEQGVGPLCKADSLYVSAADICGLFTNSSGTQQWRGLARYFKIRLRKRSVKN  298
AUF70036.1        NTATTVLLDEQGVGPLCKADSLYVSAADICGLFTNSSGTQQWRGLARYFKIRLRKRSVKN  298
ALF06323.1        NTATTVLLDEQGVGPLCKADSLYVSAADICGLFTNSSGTQQWRGLARYFKIRLRKRSVKN  298
WLD47890.1        NTATTVLLDEQGVGPLCKADSLYVSAADICGLFTNSSGTQQWRGLARYFKIRLRKRSVKN  298
BAF03097.1        NTATTVLLDEQGVGPLCKADSLYVSAADICGLFTNSSGTQQWRGLARYFKIRLRKRSVKN  298
ABI94695.1        NTATTVLLDEQGVGPLCKADSLYVSAADICGLFTNSSGTQQWRGLARYFKIRLRKRSVKN  298
ABI94623.1        NTATTVLLDEQGVGPLCKADSLYVSAADICGLFTNSSGTQQWRGLARYFKIRLRKRSVKN  298
UTM62533.1        NTATTVLLDEQGVGPLCKADSLYVSAADICGLFTNSSGTQQWRGLARYFKIRLRKRSVKN  298
QOS45058.1        NTATTVLLDEQGVGPLCKADSLYVSAADICGLFTNSSGTQQWRGLARYFKIRLRKRSVKN  298
AHU87362.1        NTATTVLLDEQGVGPLCKADSLYVSAADICGLFTNSSGTQQWRGLARYFKIRLRKRSVKN  298
AHU87363.1        NTATTVLLDEQGVGPLCKADSLYVSAADICGLFTNSSGTQQWRGLARYFKIRLRKRSVKN  298
AFQ31658.1        NTATTVLLDEQGVGPLCKADSLYVSAADICGLFTNSSGTQQWRGLARYFKIRLRKRSVKN  298
AFA41876.1        NTATTVLLDEQGVGPLCKADSLYVSAADICGLFTNSSGTQQWRGLARYFKIRLRKRSVKN  298
AFA41906.1        NTATTVLLDEQGVGPLCKADSLYVSAADICGLFTNSSGTQQWRGLARYFKIRLRKRSVKN  298
AEO89604.1        NTATTVLLDEQGVGPLCKADSLYVSAADICGLFTNSSGTQQWRGLARYFKIRLRKRSVKN  298
CBX88350.1        NTATTVLLDEQGVGPLCKADSLYVSAADICGLFTNSSGTQQWRGLARYFKIRLRKRSVKN  298
BAG84446.1        NTATTVLLDEQGVGPLCKADSLYVSAADICGLFTNSSGTQQWRGLARYFKIRLRKRSVKN  298
BAG84464.1        NTATTVLLDEQGVGPLCKADSLYVSAADICGLFTNSSGTQQWRGLARYFKIRLRKRSVKN  298
BAF93295.1        NTATTVLLDEQGVGPLCKADSLYVSAADICGLFTNSSGTQQWRGLARYFKIRLRKRSVKN  298
BAF93373.1        NTATTVLLDEQGVGPLCKADSLYVSAADICGLFTNSSGTQQWRGLARYFKIRLRKRSVKN  298
BAF93379.1        NTATTVLLDEQGVGPLCKADSLYVSAADICGLFTNSSGTQQWRGLARYFKIRLRKRSVKN  298
BAF42913.1        NTATTVLLDEQGVGPLCKADSLYVSAADICGLFTNSSGTQQWRGLARYFKIRLRKRSVKN  298
BAF42931.1        NTATTVLLDEQGVGPLCKADSLYVSAADICGLFTNSSGTQQWRGLARYFKIRLRKRSVKN  298
BAF43015.1        NTATTVLLDEQGVGPLCKADSLYVSAADICGLFTNSSGTQQWRGLARYFKIRLRKRSVKN  298
ABI94629.1        NTATTVLLDEQGVGPLCKADSLYVSAADICGLFTNSSGTQQWRGLARYFKIRLRKRSVKN  298
ABI94653.1        NTATTVLLDEQGVGPLCKADSLYVSAADICGLFTNSSGTQQWRGLARYFKIRLRKRSVKN  298
ABI94707.1        NTATTVLLDEQGVGPLCKADSLYVSAADICGLFTNSSGTQQWRGLARYFKIRLRKRSVKN  298
BAF03085.1        NTATTVLLDEQGVGPLCKADSLYVSAADICGLFTNSSGTQQWRGLARYFKIRLRKRSVKN  298
BAF03091.1        NTATTVLLDEQGVGPLCKADSLYVSAADICGLFTNSSGTQQWRGLARYFKIRLRKRSVKN  298
BAF03103.1        NTATTVLLDEQGVGPLCKADSLYVSAADICGLFTNSSGTQQWRGLARYFKIRLRKRSVKN  298
BAF03109.1        NTATTVLLDEQGVGPLCKADSLYVSAADICGLFTNSSGTQQWRGLARYFKIRLRKRSVKN  298
BAF93325.1        NTATTVLLDEQGVGPLCKADSLYVSAADICGLFTNSSGTQQWRGLARYFKIRLRKRSVKN  298
QTJ14934.1        NTATTVLLDEQGVGPLCKADSLYVSAADICGLFTNSSGTQQWRGLARYFKIRLRKRSVKN  298
QTJ14994.1        NTATTVLLDEQGVGPLCKADSLYVSAADICGLFTNSSGTQQWRGLARYFKIRLRKRSVKN  298
AFA41886.1        NTATTVLLDEQGVGPLCKADSLYVSAADICGLFTNSSGTQQWRGLARYFKIRLRKRSVKN  298
AFQ31650.1        NTATTVLLDEQGVGPLCKADSLYVSAADICGLFTNSSGTQQWRGLARYFKIRLRKRSVKN  298
AAT47347.1        NTATTVLLDEQGVGPLCKADSLYVSAADICGLFTNSSGTQQWRGLARYFKIRLRKRSVKN  298
ABI94617.1        NTATTVLLDEQGVGPLCKADSLYVSAADICGLFTNSSGTQQWRGLARYFKIRLRKRSVKN  298
BAF93319.1        NTATTVLLDEQGVGPLCKANSLYVSAADICGLFTNSSGTQQWRGLARYFKIRLRKRSVKN  298
CBX88314.1        NTATTVLLDEQGVGPLCKADSLYVSAADICGLFTNSSGTQQWRGLARYFKIRLRKRSVKN  298
ALF06320.1        NTATTVLLDEQGVGPLCKADSLYVSAADICGLFTNSSGTQQWRGLARYFKIRLRKRSVKN  298
WLD47830.1        NTATTVLLDEQGVGPLCKADSLYVSAADICGLFTNSSGTQQWRGLARYFKIRLRKRSVKN  298
WLD47866.1        NTATTVLLDEQGVGPLCKADSLYVSAADICGLFTNSSGTQQWRGLARYFKIRLRKRSVKN  298
UTM61939.1        NTATTVLLDEQGVGPLCKADSLYVSAADICGLFTNSSGTQQWRGLARYFKIRLRKRSVKN  298
UTM62023.1        NTATTVLLDEQGVGPLCKADSLYVSAADICGLFTNSSGTQQWRGLARYFKIRLRKRSVKN  298
UTM62029.1        NTATTVLLDEQGVGPLCKADSLYVSAADICGLFTNSSGTQQWRGLARYFKIRLRKRSVKN  298
UTM62053.1        NTATTVLLDEQGVGPLCKADSLYVSAADICGLFTNSSGTQQWRGLARYFKIRLRKRSVKN  298
```

UTM62059.1 NTATTVLLDEQGVGPLCKADSLYVSAADICGLFTNSSGTQQWRGLARYFKIRLRKRSVKN 298
UTM62065.1 NTATTVLLDEQGVGPLCKADSLYVSAADICGLFTNSSGTQQWRGLARYFKIRLRKRSVKN 298
UTM62317.1 NTATTVLLDEQGVGPLCKADSLYVSAADICGLFTNSSGTQQWRGLARYFKIRLRKRSVKN 298
UTM62323.1 NTATTVLLDEQGVGPLCKADSLYVSAADICGLFTNSSGTQQWRGLARYFKIRLRKRSVKN 298
UTM62353.1 NTATTVLLDEQGVGPLCKADSLYVSAADICGLFTNSSGTQQWRGLARYFKIRLRKRSVKN 298
UTM62473.1 NTATTVLLDEQGVGPLCKADSLYVSAADICGLFTNSSGTQQWRGLARYFKIRLRKRSVKN 298
UTM62491.1 NTATTVLLDEQGVGPLCKADSLYVSAADICGLFTNSSGTQQWRGLARYFKIRLRKRSVKN 298
UTM62497.1 NTATTVLLDEQGVGPLCKADSLYVSAADICGLFTNSSGTQQWRGLARYFKIRLRKRSVKN 298
UTM62503.1 NTATTVLLDEQGVGPLCKADSLYVSAADICGLFTNSSGTQQWRGLARYFKIRLRKRSVKN 298
QTJ14988.1 NTATTVLLDEQGVGPLCKADSLYVSAADICGLFTNSSGTQQWRGLARYFKIRLRKRSVKN 298
QTJ15024.1 NTATTVLLDEQGVGPLCKADSLYVSAADICGLFTNSSGTQQWRGLARYFKIRLRKRSVKN 298
QTJ15030.1 NTATTVLLDEQGVGPLCKADSLYVSAADICGLFTNSSGTQQWRGLARYFKIRLRKRSVKN 298
QTJ15102.1 NTATTVLLDEQGVGPLCKADSLYVSAADICGLFTNSSGTQQWRGLARYFKIRLRKRSVKN 298
ALF06300.1 NTATTVLLDEQGVGPLCKADSLYVSAADICGLFTNSSGTQQWRGLARYFKIRLRKRSVKN 298
AHU87342.1 NTATTVLLDEQGVGPLCKADSLYVSAADICGLFTNSSGTQQWRGLARYFKIRLRKRSVKN 298
AHU87343.1 NTATTVLLDEQGVGPLCKADSLYVSAADICGLFTNSSGTQQWRGLARYFKIRLRKRSVKN 298
AHU87346.1 NTATTVLLDEQGVGPLCKADSLYVSAADICGLFTNSSGTQQWRGLARYFKIRLRKRSVKN 298
AHU87347.1 NTATTVLLDEQGVGPLCKADSLYVSAADICGLFTNSSGTQQWRGLARYFKIRLRKRSVKN 298
AHU87349.1 NTATTVLLDEQGVGPLCKADSLYVSAADICGLFTNSSGTQQWRGLARYFKIRLRKRSVKN 298
AHU87356.1 NTATTVLLDEQGVGPLCKADSLYVSAADICGLFTNSSGTQQWRGLARYFKIRLRKRSVKN 298
AHU87357.1 NTATTVLLDEQGVGPLCKADSLYVSAADICGLFTNSSGTQQWRGLARYFKIRLRKRSVKN 298
AHU87358.1 NTATTVLLDEQGVGPLCKADSLYVSAADICGLFTNSSGTQQWRGLARYFKIRLRKRSVKN 298
AHU87359.1 NTATTVLLDEQGVGPLCKADSLYVSAADICGLFTNSSGTQQWRGLARYFKIRLRKRSVKN 298
AFQ31643.1 NTATTVLLDEQGVGPLCKADSLYVSAADICGLFTNSSGTQQWRGLARYFKIRLRKRSVKN 298
AFQ31656.1 NTATTVLLDEQGVGPLCKADSLYVSAADICGLFTNSSGTQQWRGLARYFKIRLRKRSVKN 298
AFA41879.1 NTATTVLLDEQGVGPLCKADSLYVSAADICGLFTNSSGTQQWRGLARYFKIRLRKRSVKN 298
AFA41897.1 NTATTVLLDEQGVGPLCKADSLYVSAADICGLFTNSSGTQQWRGLARYFKIRLRKRSVKN 298
AFA41900.1 NTATTVLLDEQGVGPLCKADSLYVSAADICGLFTNSSGTQQWRGLARYFKIRLRKRSVKN 298
AEO89580.1 NTATTVLLDEQGVGPLCKADSLYVSAADICGLFTNSSGTQQWRGLARYFKIRLRKRSVKN 298
AEO89592.1 NTATTVLLDEQGVGPLCKADSLYVSAADICGLFTNSSGTQQWRGLARYFKIRLRKRSVKN 298
BAF93277.1 NTATTVLLDEQGVGPLCKADSLYVSAADICGLFTNSSGTQQWRGLARYFKIRLRKRSVKN 298
BAF93289.1 NTATTVLLDEQGVGPLCKADSLYVSAADICGLFTNSSGTQQWRGLARYFKIRLRKRSVKN 298
BAF93307.1 NTATTVLLDEQGVGPLCKADSLYVSAADICGLFTNSSGTQQWRGLARYFKIRLRKRSVKN 298
BAF93337.1 NTATTVLLDEQGVGPLCKADSLYVSAADICGLFTNSSGTQQWRGLARYFKIRLRKRSVKN 298
BAF42895.1 NTATTVLLDEQGVGPLCKADSLYVSAADICGLFTNSSGTQQWRGLARYFKIRLRKRSVKN 298
BAF42919.1 NTATTVLLDEQGVGPLCKADSLYVSAADICGLFTNSSGTQQWRGLARYFKIRLRKRSVKN 298
BAF42943.1 NTATTVLLDEQGVGPLCKADSLYVSAADICGLFTNSSGTQQWRGLARYFKIRLRKRSVKN 298
BAF42949.1 NTATTVLLDEQGVGPLCKADSLYVSAADICGLFTNSSGTQQWRGLARYFKIRLRKRSVKN 298
ABI94665.1 NTATTVLLDEQGVGPLCKADSLYVSAADICGLFTNSSGTQQWRGLARYFKIRLRKRSVKN 298
ABI94677.1 NTATTVLLDEQGVGPLCKADSLYVSAADICGLFTNSSGTQQWRGLARYFKIRLRKRSVKN 298
BAF02939.1 NTATTVLLDEQGVGPLCKADSLYVSAADICGLFTNSSGTQQWRGLARYFKIRLRKRSVKN 298
AAT47353.1 NTATTVLLDEQGVGPLCKADSLYVSAADICGLFTNSSGTQQWRGLARYFKIRLRKRSVKN 298
AAT47359.1 NTATTVLLDEQGVGPLCKADSLYVSAADICGLFTNSSGTQQWRGLARYFKIRLRKRSVKN 298
CAA40247.1 NTATTVLLDEQGVGPLCKADSLYVSAADICGLFTNSSGTQQWRGLARYFKIRLRKRSVKN 298
QTJ14982.1 NTATTVLLDEQGVGPLCKADSLYVSAADICGLFTNSSGTQQWRGLARYFKIRLRKRSVKN 298
QTJ15126.1 NTATTVLLDEQGVGPLCKADSLYVSAADICGLFTNSSGTQQWRGLARYFKIRLRKRSVKN 298
ABI94635.1 NTATTVLLDEQGVGPLCKADSLYVSAADICGLFTNSSGTQQWRGLARYFKIRLRKRSVKN 298
ALF06335.1 NTATTVLLDEQGVGPLCKADSLYVSAADICGLFTNSSGTQQWRGLARYFKIRLRKRSVKN 298
AHU87348.1 NTATTVLLDEQGVGPLCKADSLYVSAADICGLFTNSSGTQQWRGLARYFKIRLRKRSVKN 298
AFQ31649.1 NTATTVLLDEQGVGPLCKADSLYVSAADICGLFTNSSGTQQWRGLARYFKIRLRKRSVKN 298
AFQ31654.1 NTATTVLLDEQGVGPLCKADSLYVSAADICGLFTNSSGTQQWRGLARYFKIRLRKRSVKN 298
BAF42907.1 NTATTVLLDEQGVGPLCKADSLYVSAADICGLFTNSSGTQQWRGLARYFKIRLRKRSVKN 298
WLD47824.1 NTATTVLLDEQGVGPLCKADSLYVSAADICGLFTNSSGTQQWRGLARYFKIRLRKRSVKN 298
WLD47848.1 NTATTVLLDEQGVGPLCKADSLYVSAADICGLFTNSSGTQQWRGLARYFKIRLRKRSVKN 298
WLD47854.1 NTATTVLLDEQGVGPLCKADSLYVSAADICGLFTNSSGTQQWRGLARYFKIRLRKRSVKN 298
WLD47860.1 NTATTVLLDEQGVGPLCKADSLYVSAADICGLFTNSSGTQQWRGLARYFKIRLRKRSVKN 298
WLD47878.1 NTATTVLLDEQGVGPLCKADSLYVSAADICGLFTNSSGTQQWRGLARYFKIRLRKRSVKN 298
WLD47908.1 NTATTVLLDEQGVGPLCKADSLYVSAADICGLFTNSSGTQQWRGLARYFKIRLRKRSVKN 298
WLD47914.1 NTATTVLLDEQGVGPLCKADSLYVSAADICGLFTNSSGTQQWRGLARYFKIRLRKRSVKN 298
WLD47920.1 NTATTVLLDEQGVGPLCKADSLYVSAADICGLFTNSSGTQQWRGLARYFKIRLRKRSVKN 298
WLD47926.1 NTATTVLLDEQGVGPLCKADSLYVSAADICGLFTNSSGTQQWRGLARYFKIRLRKRSVKN 298
WLD47932.1 NTATTVLLDEQGVGPLCKADSLYVSAADICGLFTNSSGTQQWRGLARYFKIRLRKRSVKN 298
WLD47950.1 NTATTVLLDEQGVGPLCKADSLYVSAADICGLFTNSSGTQQWRGLARYFKIRLRKRSVKN 298
WLD47962.1 NTATTVLLDEQGVGPLCKADSLYVSAADICGLFTNSSGTQQWRGLARYFKIRLRKRSVKN 298

```
WLD47974.1        NTATTVLLDEQGVGPLCKADSLYVSAADICGLFTNSSGTQQWRGLARYFKIRLRKRSVKN  298
WLD47998.1        NTATTVLLDEQGVGPLCKADSLYVSAADICGLFTNSSGTQQWRGLARYFKIRLRKRSVKN  298
WLD48004.1        NTATTVLLDEQGVGPLCKADSLYVSAADICGLFTNSSGTQQWRGLARYFKIRLRKRSVKN  298
WLD48016.1        NTATTVLLDEQGVGPLCKADSLYVSAADICGLFTNSSGTQQWRGLARYFKIRLRKRSVKN  298
WLD48022.1        NTATTVLLDEQGVGPLCKADSLYVSAADICGLFTNSSGTQQWRGLARYFKIRLRKRSVKN  298
WLD48034.1        NTATTVLLDEQGVGPLCKADSLYVSAADICGLFTNSSGTQQWRGLARYFKIRLRKRSVKN  298
UTM61909.1        NTATTVLLDEQGVGPLCKADSLYVSAADICGLFTNSSGTQQWRGLARYFKIRLRKRSVKN  298
UTM61915.1        NTATTVLLDEQGVGPLCKADSLYVSAADICGLFTNSSGTQQWRGLARYFKIRLRKRSVKN  298
UTM61921.1        NTATTVLLDEQGVGPLCKADSLYVSAADICGLFTNSSGTQQWRGLARYFKIRLRKRSVKN  298
UTM61927.1        NTATTVLLDEQGVGPLCKADSLYVSAADICGLFTNSSGTQQWRGLARYFKIRLRKRSVKN  298
UTM61933.1        NTATTVLLDEQGVGPLCKADSLYVSAADICGLFTNSSGTQQWRGLARYFKIRLRKRSVKN  298
UTM61963.1        NTATTVLLDEQGVGPLCKADSLYVSAADICGLFTNSSGTQQWRGLARYFKIRLRKRSVKN  298
UTM61969.1        NTATTVLLDEQGVGPLCKADSLYVSAADICGLFTNSSGTQQWRGLARYFKIRLRKRSVKN  298
UTM61975.1        NTATTVLLDEQGVGPLCKADSLYVSAADICGLFTNSSGTQQWRGLARYFKIRLRKRSVKN  298
UTM61981.1        NTATTVLLDEQGVGPLCKADSLYVSAADICGLFTNSSGTQQWRGLARYFKIRLRKRSVKN  298
UTM62005.1        NTATTVLLDEQGVGPLCKADSLYVSAADICGLFTNSSGTQQWRGLARYFKIRLRKRSVKN  298
UTM62017.1        NTATTVLLDEQGVGPLCKADSLYVSAADICGLFTNSSGTQQWRGLARYFKIRLRKRSVKN  298
UTM62035.1        NTATTVLLDEQGVGPLCKADSLYVSAADICGLFTNSSGTQQWRGLARYFKIRLRKRSVKN  298
UTM62041.1        NTATTVLLDEQGVGPLCKADSLYVSAADICGLFTNSSGTQQWRGLARYFKIRLRKRSVKN  298
UTM62047.1        NTATTVLLDEQGVGPLCKADSLYVSAADICGLFTNSSGTQQWRGLARYFKIRLRKRSVKN  298
UTM62083.1        NTATTVLLDEQGVGPLCKADSLYVSAADICGLFTNSSGTQQWRGLARYFKIRLRKRSVKN  298
UTM62089.1        NTATTVLLDEQGVGPLCKADSLYVSAADICGLFTNSSGTQQWRGLARYFKIRLRKRSVKN  298
UTM62095.1        NTATTVLLDEQGVGPLCKADSLYVSAADICGLFTNSSGTQQWRGLARYFKIRLRKRSVKN  298
UTM62149.1        NTATTVLLDEQGVGPLCKADSLYVSAADICGLFTNSSGTQQWRGLARYFKIRLRKRSVKN  298
UTM62161.1        NTATTVLLDEQGVGPLCKADSLYVSAADICGLFTNSSGTQQWRGLARYFKIRLRKRSVKN  298
UTM62167.1        NTATTVLLDEQGVGPLCKADSLYVSAADICGLFTNSSGTQQWRGLARYFKIRLRKRSVKN  298
UTM62173.1        NTATTVLLDEQGVGPLCKADSLYVSAADICGLFTNSSGTQQWRGLARYFKIRLRKRSVKN  298
UTM62179.1        NTATTVLLDEQGVGPLCKADSLYVSAADICGLFTNSSGTQQWRGLARYFKIRLRKRSVKN  298
UTM62185.1        NTATTVLLDEQGVGPLCKADSLYVSAADICGLFTNSSGTQQWRGLARYFKIRLRKRSVKN  298
UTM62191.1        NTATTVLLDEQGVGPLCKADSLYVSAADICGLFTNSSGTQQWRGLARYFKIRLRKRSVKN  298
UTM62197.1        NTATTVLLDEQGVGPLCKADSLYVSAADICGLFTNSSGTQQWRGLARYFKIRLRKRSVKN  298
UTM62203.1        NTATTVLLDEQGVGPLCKADSLYVSAADICGLFTNSSGTQQWRGLARYFKIRLRKRSVKN  298
UTM62209.1        NTATTVLLDEQGVGPLCKADSLYVSAADICGLFTNSSGTQQWRGLARYFKIRLRKRSVKN  298
UTM62227.1        NTATTVLLDEQGVGPLCKADSLYVSAADICGLFTNSSGTQQWRGLARYFKIRLRKRSVKN  298
UTM62239.1        NTATTVLLDEQGVGPLCKADSLYVSAADICGLFTNSSGTQQWRGLARYFKIRLRKRSVKN  298
UTM62245.1        NTATTVLLDEQGVGPLCKADSLYVSAADICGLFTNSSGTQQWRGLARYFKIRLRKRSVKN  298
UTM62251.1        NTATTVLLDEQGVGPLCKADSLYVSAADICGLFTNSSGTQQWRGLARYFKIRLRKRSVKN  298
UTM62257.1        NTATTVLLDEQGVGPLCKADSLYVSAADICGLFTNSSGTQQWRGLARYFKIRLRKRSVKN  298
UTM62263.1        NTATTVLLDEQGVGPLCKADSLYVSAADICGLFTNSSGTQQWRGLARYFKIRLRKRSVKN  298
UTM62269.1        NTATTVLLDEQGVGPLCKADSLYVSAADICGLFTNSSGTQQWRGLARYFKIRLRKRSVKN  298
UTM62287.1        NTATTVLLDEQGVGPLCKADSLYVSAADICGLFTNSSGTQQWRGLARYFKIRLRKRSVKN  298
UTM62293.1        NTATTVLLDEQGVGPLCKADSLYVSAADICGLFTNSSGTQQWRGLARYFKIRLRKRSVKN  298
UTM62299.1        NTATTVLLDEQGVGPLCKADSLYVSAADICGLFTNSSGTQQWRGLARYFKIRLRKRSVKN  298
UTM62305.1        NTATTVLLDEQGVGPLCKADSLYVSAADICGLFTNSSGTQQWRGLARYFKIRLRKRSVKN  298
UTM62311.1        NTATTVLLDEQGVGPLCKADSLYVSAADICGLFTNSSGTQQWRGLARYFKIRLRKRSVKN  298
UTM62359.1        NTATTVLLDEQGVGPLCKADSLYVSAADICGLFTNSSGTQQWRGLARYFKIRLRKRSVKN  298
UTM62365.1        NTATTVLLDEQGVGPLCKADSLYVSAADICGLFTNSSGTQQWRGLARYFKIRLRKRSVKN  298
UTM62371.1        NTATTVLLDEQGVGPLCKADSLYVSAADICGLFTNSSGTQQWRGLARYFKIRLRKRSVKN  298
UTM62377.1        NTATTVLLDEQGVGPLCKADSLYVSAADICGLFTNSSGTQQWRGLARYFKIRLRKRSVKN  298
UTM62455.1        NTATTVLLDEQGVGPLCKADSLYVSAADICGLFTNSSGTQQWRGLARYFKIRLRKRSVKN  298
UTM62461.1        NTATTVLLDEQGVGPLCKADSLYVSAADICGLFTNSSGTQQWRGLARYFKIRLRKRSVKN  298
UTM62485.1        NTATTVLLDEQGVGPLCKADSLYVSAADICGLFTNSSGTQQWRGLARYFKIRLRKRSVKN  298
UTM62509.1        NTATTVLLDEQGVGPLCKADSLYVSAADICGLFTNSSGTQQWRGLARYFKIRLRKRSVKN  298
UTM62515.1        NTATTVLLDEQGVGPLCKADSLYVSAADICGLFTNSSGTQQWRGLARYFKIRLRKRSVKN  298
UTM62521.1        NTATTVLLDEQGVGPLCKADSLYVSAADICGLFTNSSGTQQWRGLARYFKIRLRKRSVKN  298
UTM62527.1        NTATTVLLDEQGVGPLCKADSLYVSAADICGLFTNSSGTQQWRGLARYFKIRLRKRSVKN  298
UTM62539.1        NTATTVLLDEQGVGPLCKADSLYVSAADICGLFTNSSGTQQWRGLARYFKIRLRKRSVKN  298
UTM62557.1        NTATTVLLDEQGVGPLCKADSLYVSAADICGLFTNSSGTQQWRGLARYFKIRLRKRSVKN  298
UTM62563.1        NTATTVLLDEQGVGPLCKADSLYVSAADICGLFTNSSGTQQWRGLARYFKIRLRKRSVKN  298
UTM62569.1        NTATTVLLDEQGVGPLCKADSLYVSAADICGLFTNSSGTQQWRGLARYFKIRLRKRSVKN  298
UTM62575.1        NTATTVLLDEQGVGPLCKADSLYVSAADICGLFTNSSGTQQWRGLARYFKIRLRKRSVKN  298
UTM62581.1        NTATTVLLDEQGVGPLCKADSLYVSAADICGLFTNSSGTQQWRGLARYFKIRLRKRSVKN  298
UTM62605.1        NTATTVLLDEQGVGPLCKADSLYVSAADICGLFTNSSGTQQWRGLARYFKIRLRKRSVKN  298
UTM62617.1        NTATTVLLDEQGVGPLCKADSLYVSAADICGLFTNSSGTQQWRGLARYFKIRLRKRSVKN  298
```

```
UTM62623.1    NTATTVLLDEQGVGPLCKADSLYVSAADICGLFTNSSGTQQWRGLARYFKIRLRKRSVKN  298
UTM62629.1    NTATTVLLDEQGVGPLCKADSLYVSAADICGLFTNSSGTQQWRGLARYFKIRLRKRSVKN  298
UTM62635.1    NTATTVLLDEQGVGPLCKADSLYVSAADICGLFTNSSGTQQWRGLARYFKIRLRKRSVKN  298
QTJ15048.1    NTATTVLLDEQGVGPLCKADSLYVSAADICGLFTNSSGTQQWRGLARYFKIRLRKRSVKN  298
QTJ15144.1    NTATTVLLDEQGVGPLCKADSLYVSAADICGLFTNSSGTQQWRGLARYFKIRLRKRSVKN  298
AHU87360.1    NTATTVLLDEQGVGPLCKADSLYVSAADICGLFTNSSGTQQWRGLARYFKIRLRKRSVKN  298
AHU87361.1    NTATTVLLDEQGVGPLCKADSLYVSAADICGLFTNSSGTQQWRGLARYFKIRLRKRSVKN  298
AHU87366.1    NTATTVLLDEQGVGPLCKADSLYVSAADICGLFTNSSGTQQWRGLARYFKIRLRKRSVKN  298
AHU87367.1    NTATTVLLDEQGVGPLCKADSLYVSAADICGLFTNSSGTQQWRGLARYFKIRLRKRSVKN  298
AFQ31652.1    NTATTVLLDEQGVGPLCKADSLYVSAADICGLFTNSSGTQQWRGLARYFKIRLRKRSVKN  298
AFA41888.1    NTATTVLLDEQGVGPLCKADSLYVSAADICGLFTNSSGTQQWRGLARYFKIRLRKRSVKN  298
AFA41893.1    NTATTVLLDEQGVGPLCKADSLYVSAADICGLFTNSSGTQQWRGLARYFKIRLRKRSVKN  298
AFA41894.1    NTATTVLLDEQGVGPLCKADSLYVSAADICGLFTNSSGTQQWRGLARYFKIRLRKRSVKN  298
AFA41899.1    NTATTVLLDEQGVGPLCKADSLYVSAADICGLFTNSSGTQQWRGLARYFKIRLRKRSVKN  298
AFA41904.1    NTATTVLLDEQGVGPLCKADSLYVSAADICGLFTNSSGTQQWRGLARYFKIRLRKRSVKN  298
AFA41905.1    NTATTVLLDEQGVGPLCKADSLYVSAADICGLFTNSSGTQQWRGLARYFKIRLRKRSVKN  298
AFA41908.1    NTATTVLLDEQGVGPLCKADSLYVSAADICGLFTNSSGTQQWRGLARYFKIRLRKRSVKN  298
AFA41911.1    NTATTVLLDEQGVGPLCKADSLYVSAADICGLFTNSSGTQQWRGLARYFKIRLRKRSVKN  298
AFA41912.1    NTATTVLLDEQGVGPLCKADSLYVSAADICGLFTNSSGTQQWRGLARYFKIRLRKRSVKN  298
AFA41913.1    NTATTVLLDEQGVGPLCKADSLYVSAADICGLFTNSSGTQQWRGLARYFKIRLRKRSVKN  298
AFA41915.1    NTATTVLLDEQGVGPLCKADSLYVSAADICGLFTNSSGTQQWRGLARYFKIRLRKRSVKN  298
AFA41916.1    NTATTVLLDEQGVGPLCKADSLYVSAADICGLFTNSSGTQQWRGLARYFKIRLRKRSVKN  298
AFA41919.1    NTATTVLLDEQGVGPLCKADSLYVSAADICGLFTNSSGTQQWRGLARYFKIRLRKRSVKN  298
AFA41921.1    NTATTVLLDEQGVGPLCKADSLYVSAADICGLFTNSSGTQQWRGLARYFKIRLRKRSVKN  298
AFA41922.1    NTATTVLLDEQGVGPLCKADSLYVSAADICGLFTNSSGTQQWRGLARYFKIRLRKRSVKN  298
AFA41923.1    NTATTVLLDEQGVGPLCKADSLYVSAADICGLFTNSSGTQQWRGLARYFKIRLRKRSVKN  298
AFA41924.1    NTATTVLLDEQGVGPLCKADSLYVSAADICGLFTNSSGTQQWRGLARYFKIRLRKRSVKN  298
AFA41925.1    NTATTVLLDEQGVGPLCKADSLYVSAADICGLFTNSSGTQQWRGLARYFKIRLRKRSVKN  298
CBX88272.1    NTATTVLLDEQGVGPLCKADSLYVSAADICGLFTNSSGTQQWRGLARYFKIRLRKRSVKN  298
CBX88278.1    NTATTVLLDEQGVGPLCKADSLYVSAADICGLFTNSSGTQQWRGLARYFKIRLRKRSVKN  298
CBX88284.1    NTATTVLLDEQGVGPLCKADSLYVSAADICGLFTNSSGTQQWRGLARYFKIRLRKRSVKN  298
CBX88290.1    NTATTVLLDEQGVGPLCKADSLYVSAADICGLFTNSSGTQQWRGLARYFKIRLRKRSVKN  298
CBX88296.1    NTATTVLLDEQGVGPLCKADSLYVSAADICGLFTNSSGTQQWRGLARYFKIRLRKRSVKN  298
CBX88308.1    NTATTVLLDEQGVGPLCKADSLYVSAADICGLFTNSSGTQQWRGLARYFKIRLRKRSVKN  298
CBX88332.1    NTATTVLLDEQGVGPLCKADSLYVSAADICGLFTNSSGTQQWRGLARYFKIRLRKRSVKN  298
CBX88356.1    NTATTVLLDEQGVGPLCKADSLYVSAADICGLFTNSSGTQQWRGLARYFKIRLRKRSVKN  298
CBX88362.1    NTATTVLLDEQGVGPLCKADSLYVSAADICGLFTNSSGTQQWRGLARYFKIRLRKRSVKN  298
BAF93313.1    NTATTVLLDEQGVGPLCKADSLYVSAADICGLFTNSSGTQQWRGLARYFKIRLRKRSVKN  298
BAF42955.1    NTATTVLLDEQGVGPLCKADSLYVSAADICGLFTNSSGTQQWRGLARYFKIRLRKRSVKN  298
BAF43021.1    NTATTVLLDEQGVGPLCKADSLYVSAADICGLFTNSSGTQQWRGLARYFKIRLRKRSVKN  298
ABI94611.1    NTATTVLLDEQGVGPLCKADSLYVSAADICGLFTNSSGTQQWRGLARYFKIRLRKRSVKN  298
ABI94641.1    NTATTVLLDEQGVGPLCKADSLYVSAADICGLFTNSSGTQQWRGLARYFKIRLRKRSVKN  298
ABI94647.1    NTATTVLLDEQGVGPLCKADSLYVSAADICGLFTNSSGTQQWRGLARYFKIRLRKRSVKN  298
WLD47896.1    NTATTVLLDEQGVGPLCKADSLYVSAADICGLFTNSSGTQQWRGLARYFKIRLRKRSVKN  298
WLD48028.1    NTATTVLLDEQGVGPLCKADSLYVSAADICGLFTNSSGTQQWRGLARYFKIRLRKRSVKN  298
BAG84440.1    NTATTVLLDEQGVGPLCKADSLYVSAADICGLFTNSSGTQQWRGLARYFKIRLRKRSVKN  298
BAF93283.1    NTATTVLLDEQGVGPLCKADSLYVSAADICGLFTNSSGTQQWRGLARYFKIRLRKRSVKN  298
BAF93361.1    NTATTVLLDEQGVGPLCKADSLYVSAADICGLFTNSSGTQQWRGLARYFKIRLRKRSVKN  298
WLD48040.1    NTATTVLLDEQGVGPLCKADSLYVSAADICGLFTNSSGTQQWRGLARYFKIRLRKRSVKN  298
UTM62611.1    NTATTVLLDEQGVGPLCKADSLYVSAADICGLFTNSSGTQQWRGLARYFKIRLRKRSVKN  298
AFQ31645.1    NTATTVLLDEQGVGPLCKADSLYVSATDICGLFTNSSGTQQWRGLARYFKIRLRKRSVKN  298
AFA41909.1    NTATTVLLDEQGVGPLCKADSLYVSAADICGLFTNSSGTQQWRGLARYFKIRLRKRSVKN  298
ABI94713.1    NTATTVLLDEQGVGPLCKADSLYVSAADICGLFTNSSGTQQWRGLARYFKIRLRKRSVKN  298
ABD04662.1    NTATTVLLDEQGVGPLCKADSLYVSAADICGLFTNSSGTQQWRGLARYFKIRLRKRSVKN  298
QTJ15042.1    NTATTVLLDEQGVGPLCKADSLYVSAADICGLFTNSSGTQQWRGLARYFKIRLRKRSVKN  298
AGX89705.1    NTATTVLLDEQGVGPLCKADSLYVSAADICGLFTNSSGTQQWRGLARYFKIRLRKRSVKN  298
AGX89710.1    NTATTVLLDEQGVGPLCKADSLYVSAADICGLFTNSSGTQQWRGLARYFKIRLRKRSVKN  298
AGX89717.1    NTATTVLLDEQGVGPLCKADSLYVSAADICGLFTNSSGTQQWRGLARYFKIRLRKRSVKN  298
AFQ31655.1    NTATTVLLDEQGVGPLCKADSLYVSAADICGLFTNSSGTQQWRGLARYFKIRLRKRSVKN  298
AAA46882.1    NTATTVLLDEQGVGPLCKADSLYVSAADICGLFTNSSGTQQWRGLARYFKIRLRKRSVKN  298
P14996.1      NTATTVLLDEQGVGPLCKADSLYVSAADICGLFTNSSGTQQWRGLARYFKIRLRKRSVKN  298
AEO89615.1    NTATTVLLDEQGVGPLCKADSLYVSAADICGLFTNSSGTQQWRGLARYFKIRLRKRSVKN  298
BCV68929.1    NTATTVLLDEQGVGPLCKADSLYVSAADICGLFTNSSGTQQWRGLARYFKIRLRKRSVKN  298
QTJ14952.1    NTATTVLLDEQGVGPLCKADSLYVSAADICGLFTNSSGTQQWRGLARYFKIRLRKRSVKN  298
```

```
BAG75157.1        NTATTVLLDEQGVGPLCKADSLYVSAADICGLFTNSSGTQQWRGLARYFKIRLRKRSVKN  298
BAG75211.1        NTATTVLLDEQGVGPLCKADSLYVSAADICGLFTNSSGTQQWRGLARYFKIRLRKRSVKN  298
BAF03017.1        NTATTVLLDEQGVGPLCKADSLYVSAADICGLFTNSSGTQQWRGLARYFKIRLRKRSVKN  298
BAF42925.1        NTATTVLLDEQGVGPLCKADSLYVSAADICGLFTNSSGTQQWRGLARYFKIRLRKRSVKN  298
WLD47836.1        NTATTVLLDEQGVGPLCKADSLYVSAADICGLFTNSSGTQQWRGLARYFKIRLRKRSVKN  298
UEL36354.1        NTATTVLLDEQGVGPLCKADSLYVSAADICGLFTNSSGTQQWRGLARYFKIRLRKRSVKN  298
BAF42901.1        NTATTVLLDEQGVGPLCKADSLYVSAADICGLFTNSSGTQQWRGLARYFKIRLRKRSVKN  298
ABP87973.1        NTATTVLLDEQGVGPLCKADSLYVSAADICGLFTNSSGTQQWRGLARYFKIRLRKRSVKN  298
AFA41878.1        NTATTVLLDEQGVGPLCKADSLYVSAADICGLFTNSSGTQQWRGLARYFKIRLRKRSVKN  298
QHI34655.1        NTATTVLLDEQGVGPLCKADSLYVSAADICGLFTNSSGTQQWRGLARYFKIRLRKRSVKN  298
UTM61945.1        NTATTVLLDEQGVGPLCKADSLYVSAADICGLFTNSSGTQQWRGLARYFKIRLRKRSVKN  298
UTM61987.1        NTATTVLLDEQGVGPLCKADSLYVSAADICGLFTNSSGTQQWRGLARYFKIRLRKRSVKN  298
UTM62233.1        NTATTVLLDEQGVGPLCKADSLYVSAADICGLFTNSSGTQQWRGLARYFKIRLRKRSVKN  298
UTM62341.1        NTATTVLLDEQGVGPLCKADSLYVSAADICGLFTNSSGTQQWRGLARYFKIRLRKRSVKN  298
UTM62347.1        NTATTVLLDEQGVGPLCKADSLYVSAADICGLFTNSSGTQQWRGLARYFKIRLRKRSVKN  298
AHU87354.1        NTATTVLLDEQGVGPLCKADSLYVSAADICGLFTNSSGTQQWRGLARYFKIRLRKRSVKN  298
AHU87355.1        NTATTVLLDEQGVGPLCKADSLYVSAADICGLFTNSSGTQQWRGLARYFKIRLRKRSVKN  298
AHU87364.1        NTATTVLLDEQGVGPLCKADSLYVSAADICGLFTNSSGTQQWRGLARYFKIRLRKRSVKN  298
AHU87365.1        NTATTVLLDEQGVGPLCKADSLYVSAADICGLFTNSSGTQQWRGLARYFKIRLRKRSVKN  298
AFQ31638.1        NTATTVLLDEQGVGPLCKADSLYVSAADICGLFTNSSGTQQWRGLARYFKIRLRKRSVKN  298
AFA41874.1        NTATTVLLDEQGVGPLCKADSLYVSAADICGLFTNSSGTQQWRGLARYFKIRLRKRSVKN  298
AFA41875.1        NTATTVLLDEQGVGPLCKADSLYVSAADICGLFTNSSGTQQWRGLARYFKIRLRKRSVKN  298
AFA41896.1        NTATTVLLDEQGVGPLCKADSLYVSAADICGLFTNSSGTQQWRGLARYFKIRLRKRSVKN  298
BAF93367.1        NTATTVLLDEQGVGPLCKADSLYVSAADICGLFTNSSGTQQWRGLARYFKIRLRKRSVKN  298
CAA79596.1        NTATTVLLDEQGVGPLCKADSLYVSAADICGLFTNSSGTQQWRGLARYFKIRLRKRSVKN  298
AFA41883.1        NTATTVLLDEQGVGPLCKADSLYVSAADICGLFTNSSGTQQWRGLPRYFKIRMRKRSVKN  298
WLD47812.1        NTATTVLLDEQGVGPLCKADSLYVSAADICGLFTNSSGTQQWRGLPRYFKIRLRKRSVKN  298
BAG75277.1        NTATTVLLDEQGVGPLCKADSLYVSAADICGLFINSSGTQQWRGLPRYFKIRLRKRSVKN  298
BAF75138.1        NTATTVLLDEQGVGPLCKADSLYVSAADICGLFTNSSGTQQWRGLPRYFKIRLRKRSVKN  298
BAF75102.1        NTATTVLLDEQGVGPLCKADSLYVSAADICGLFTNSSGTQQWRGLPRYFKIRLRKRSVKN  298
AYO46643.1        NTATTVLLDEQGVGPLCKADSLYVSAADICGLFTNSSGTQQWRGLPRYFKIRLRKRSVKN  298
QUW04844.1        NTATTVLLDEQGVGPLCKADSLYVSAADICGLFTNSSGTQQWRGLPRYFKIRLRKRSVKN  298
AYO46683.1        NTATTVLLDEQGVGPLCKADSLYVSAADICGLFTNSSGTQQWRGLPRYFKIRLRKRSVKN  298
AYO46645.1        NTATTVLLDEQGVGPLCKADSLYVSAADICGLFTNSSGTQQWRGLPRYFKIRLRKRSVKN  298
BAF75180.1        NTATTVLLDEQGVGPLCKADSLYVSAADICGLFTNSSGTQQWRGLPRYFKIRLRKRSVKN  298
BAF75204.1        NTATTVLLDEQGVGPLCKADSLYVSAADICGLFTNSSGTQQWRGLPRYFKIRLRKRSVKN  298
AYO46631.1        NTATTVLLDEQGVGPLCKADSLYVSAADICGLFTNSSGTQQWRGLPRYFKIRLRKRSVKN  298
AYO46608.1        NTATTVLLDEQGVGPLCKADSLYVSAADICGLFTNSSGTQQWRGLPRYFKIRLRKRSVKN  298
AYO46611.1        NTATTVLLDEQGVGPLCKADSLYVSAADICGLFTNSSGTQQWRGLPRYFKIRLRKRSVKN  298
AYO46615.1        NTATTVLLDEQGVGPLCKADSLYVSAADICGLFTNSSGTQQWRGLPRYFKIRLRKRSVKN  298
AYO46639.1        NTATTVLLDEQGVGPLCKADSLYVSAADICGLFTNSSGTQQWRGLPRYFKIRLRKRSVKN  298
AYO46649.1        NTATTVLLDEQGVGPLCKADSLYVSAADICGLFTNSSGTQQWRGLPRYFKIRLRKRSVKN  298
AYO46655.1        NTATTVLLDEQGVGPLCKADSLYVSAADICGLFTNSSGTQQWRGLPRYFKIRLRKRSVKN  298
AYO46664.1        NTATTVLLDEQGVGPLCKADSLYVSAADICGLFTNSSGTQQWRGLPRYFKIRLRKRSVKN  298
AYO46669.1        NTATTVLLDEQGVGPLCKADSLYVSAADICGLFTNSSGTQQWRGLPRYFKIRLRKRSVKN  298
AYO46685.1        NTATTVLLDEQGVGPLCKADSLYVSAADICGLFTNSSGTQQWRGLPRYFKIRLRKRSVKN  298
AYO46686.1        NTATTVLLDEQGVGPLCKADSLYVSAADICGLFTNSSGTQQWRGLPRYFKIRLRKRSVKN  298
AYO46690.1        NTATTVLLDEQGVGPLCKADSLYVSAADICGLFTNSSGTQQWRGLPRYFKIRLRKRSVKN  298
AYO46627.1        NTATTVLLDEQGVGPLCKADSLYVSAADICGLFTNSSGTQQWRGLPRYFKIRLRKRSVKN  298
AYO46630.1        NTATTVLLDEQGVGPLCKADSLYVSAADICGLFTNSSGTQQWRGLPRYFKIRLRKRSVKN  298
AYO46656.1        NTATTVLLDEQGVGPLCKADSLYVSAADICGLFTNSSGTQQWRGLPRYFKIRLRKRSVKN  298
BAG75217.1        NTATTVLLDEQGVGPLCKADSLYVSAADICGLFTNSSGTQQWRGLPRYFKIRLRKRSVKN  298
BAG75229.1        NTATTVLLDEQGVGPLCKADSLYVSAADICGLFTNSSGTQQWRGLPRYFKIRLRKRSVKN  298
BAG75235.1        NTATTVLLDEQGVGPLCKADSLYVSAADICGLFTNSSGTQQWRGLPRYFKIRLRKRSVKN  298
BAG75259.1        NTATTVLLDEQGVGPLCKADSLYVSAADICGLFTNSSGTQQWRGLPRYFKIRLRKRSVKN  298
BAF75150.1        NTATTVLLDEQGVGPLCKADSLYVSAADICGLFTNSSGTQQWRGLPRYFKIRLRKRSVKN  298
BAF75156.1        NTATTVLLDEQGVGPLCKADSLYVSAADICGLFTNSSGTQQWRGLPRYFKIRLRKRSVKN  298
BAF75174.1        NTATTVLLDEQGVGPLCKADSLYVSAADICGLFTNSSGTQQWRGLPRYFKIRLRKRSVKN  298
BAE96083.1        NTATTVLLDEQGVGPLCKADSLYVSAADICGLFTNSSGTQQWRGLPRYFKIRLRKRSVKN  298
BAE53654.1        NTATTVLLDEQGVGPLCKADSLYVSAADICGLFTNSSGTQQWRGLPRYFKIRLRKRSVKN  298
BAF03035.1        NTATTVLLDEQGVGPLCKADSLYVSAADICGLFTNSSGTQQWRGLPRYFKIRLRKRSVKN  298
QHI34656.1        NTATTVLLDEQGVGPLCKADSLYVSAADICGLFTNSSGTQQWRGLPRYFKIRLRKRSVKN  298
ARE72396.1        NTATTVLLDEQGVGPLCKADSLYVSAADICGLFTNSSGTQQWRGLPRYFKIRLRKRSVKN  298
AFA41885.1        NTATTVLLDEQGVGPLCKADSLYVSAADICGLFTNSSGTQQWRGLPRYFKIRLRKRSVKN  298
```

BAG75271.1	NTATTVLLDEQGVGPLCKADSLYVSAADICGLFTNSSGTQQWRGLPRYFKIRLRKRSVKN	298
BAF75090.1	NTATTVLLDEQGVGPLCKADSLYVSAADICGLFTNSSGTQQWRGLPRYFKIRLRKRSVKN	298
BAF75306.1	NTATTVLLDEQGVGPLCKADSLYVSAADICGLFTNSSGTQQWRGLPRYFKIRLRKRSVKN	298
BAF75342.1	NTATTVLLDEQGVGPLCKADSLYVSAADICGLFTNSSGTQQWRGLPRYFKIRLRKRSVKN	298
BAF03029.1	NTATTVLLDEQGVGPLCKADSLYVSAADICGLFTNSSGTQQWRGLPRYFKIRLRKRSVKN	298
BAG75205.1	NTATTVLLDEQGVGPLCKADSLYVSAADICGLFTNSSGTQQWRGLPRYFKIRLRKRSVKN	298
BAG75223.1	NTATTVLLDEQGVGPLCKADSLYVSAADICGLFTNSSGTQQWRGLPRYFKIRLRKRSVKN	298
BAF75144.1	NTATTVLLDEQGVGPLCKADSLYVSAADICGLFTNSSGTQQWRGLPRYFKIRLRKRSVKN	298
BAF75162.1	NTATTVLLDEQGVGPLCKADSLYVSAADICGLFTNSSGTQQWRGLPRYFKIRLRKRSVKN	298
BAF75168.1	NTATTVLLDEQGVGPLCKADSLYVSAADICGLFTNSSGTQQWRGLPRYFKIRLRKRSVKN	298
BAF75240.1	NTATTVLLDEQGVGPLCKADSLYVSAADICGLFTNSSGTQQWRGLPRYFKIRLRKRSVKN	298
BAF03023.1	NTATTVLLDEQGVGPLCKADSLYVSAADICGLFTNSSGTQQWRGLPRYFKIRLRKRSVKN	298
BAE96077.1	NTATTVLLDEQGVGPLCKADSLYVSAADICGLFTNSSGTQQWRGLPRYFKIRLRKRSVKN	298
BAG84476.1	NTATTVLLDEQGVGPLCKADSLYVSAADICGLFTNSSGTQQWRGLPRYFKIRLRKRSVKN	298
AYO46661.1	NTATTVLLDEQGVGPLCKADSLYVSAADICGLFTNSSGTQQWRGLPRYFKIRLRKRSVKN	298
AYO46634.1	NTATTVLLDEQGVGPLCKADSLYVSAADICGLFTNSSGTQQWRGLPRYFKIRLRKRSVKN	298
AYO46657.1	NTATTVLLDEQGVGPLCKADSLYVSAADICGLFTNSSGTQQWRGLPRYFKIRLRKRSVKN	298
BAG75379.1	NTATTVLLDEQGVGPLCKADSLYVSAADICGLFTNSSGTQQWRGLPRYFKIRLRKRSVKN	298
BAG75409.1	NTATTVLLDEQGVGPLCKADSLYVSAADICGLFTNSSGTQQWRGLPRYFKIRLRKRSVKN	298
BAG75415.1	NTATTVLLDEQGVGPLCKADSLYVSAADICGLFTNSSGTQQWRGLPRYFKIRLRKRSVKN	298
BAF75096.1	NTATTVLLDEQGVGPLCKADSLYVSAADICGLFTNSSGTQQWRGLPRYFKIRLRKRSVKN	298
BAF75192.1	NTATTVLLDEQGVGPLCKADSLYVSAADICGLFTNSSGTQQWRGLPRYFKIRLRKRSVKN	298
BAF75198.1	NTATTVLLDEQGVGPLCKADSLYVSAADICGLFTNSSGTQQWRGLPRYFKIRLRKRSVKN	298
BAF75210.1	NTATTVLLDEQGVGPLCKADSLYVSAADICGLFTNSSGTQQWRGLPRYFKIRLRKRSVKN	298
BAF75234.1	NTATTVLLDEQGVGPLCKADSLYVSAADICGLFTNSSGTQQWRGLPRYFKIRLRKRSVKN	298
BAF75264.1	NTATTVLLDEQGVGPLCKADSLYVSAADICGLFTNSSGTQQWRGLPRYFKIRLRKRSVKN	298
BAF75270.1	NTATTVLLDEQGVGPLCKADSLYVSAADICGLFTNSSGTQQWRGLPRYFKIRLRKRSVKN	298
BAF75282.1	NTATTVLLDEQGVGPLCKADSLYVSAADICGLFTNSSGTQQWRGLPRYFKIRLRKRSVKN	298
BAF75300.1	NTATTVLLDEQGVGPLCKADSLYVSAADICGLFTNSSGTQQWRGLPRYFKIRLRKRSVKN	298
BAF75312.1	NTATTVLLDEQGVGPLCKADSLYVSAADICGLFTNSSGTQQWRGLPRYFKIRLRKRSVKN	298
BAF75318.1	NTATTVLLDEQGVGPLCKADSLYVSAADICGLFTNSSGTQQWRGLPRYFKIRLRKRSVKN	298
BAF75324.1	NTATTVLLDEQGVGPLCKADSLYVSAADICGLFTNSSGTQQWRGLPRYFKIRLRKRSVKN	298
BAF75336.1	NTATTVLLDEQGVGPLCKADSLYVSAADICGLFTNSSGTQQWRGLPRYFKIRLRKRSVKN	298
AFA41889.1	NTATTVLLDEQGVGPLCKADSLYVSAADICGLFTNSSGTQQWRGLPRYFKIRLRKRSVKN	298
UEL36357.1	NTATTVLLDEQGVGPLCKADSLYVSAADICGLFTNSSGTQQWRGLPRYFKIRLRKRSVKN	298
BCV68920.1	NTATTVLLDEQGVGPLCKADSLYVSAADICGLFTNSSGTQQWRGLPRYFKIRLRKRSVKN	298
WLD47992.1	NTATTVLLDEQGVGPLCKADSLYVSAADICGLFTNSSGTQQWRGLPRYFKIRLRKRSVKN	298
UTM62413.1	NTATTVLLDEQGVGPLCKADSLYVSAADICGLFTNSSGTQQWRGLPRYFKIRLRKRSVKN	298
AHU87344.1	NTATTVLLDEQGVGPLCKADSLYVSAADICGLFTNSSGTQQWRGLPRYFKIRLRKRSVKN	298
AHU87345.1	NTATTVLLDEQGVGPLCKADSLYVSAADICGLFTNSSGTQQWRGLPRYFKIRLRKRSVKN	298
QTJ14946.1	NTATTVLLDEQGVGPLCKADSLYVSAADICGLFTNSSGTQQWRGLPRYFKIRLRKRSVKN	298
AYO46666.1	NTATTVLLDEQGVGPLCKADSLYVSAADICGLFTNSSGTQQWRGLPRYFKIRLRKRSVKN	298
WLD47788.1	NTATTVLLDEQGVGPLCKADSLYVSAADICGLFTNSSGTQQWRGLPRYFKIRLRKRSVKN	298
WLD47794.1	NTATTVLLDEQGVGPLCKADSLYVSAADICGLFTNSSGTQQWRGLPRYFKIRLRKRSVKN	298
WLD47800.1	NTATTVLLDEQGVGPLCKADSLYVSAADICGLFTNSSGTQQWRGLPRYFKIRLRKRSVKN	298
WLD47806.1	NTATTVLLDEQGVGPLCKADSLYVSAADICGLFTNSSGTQQWRGLPRYFKIRLRKRSVKN	298
WLD47872.1	NTATTVLLDEQGVGPLCKADSLYVSAADICGLFTNSSGTQQWRGLPRYFKIRLRKRSVKN	298
WLD47986.1	NTATTVLLDEQGVGPLCKADSLYVSAADICGLFTNSSGTQQWRGLPRYFKIRLRKRSVKN	298
WLD48010.1	NTATTVLLDEQGVGPLCKADSLYVSAADICGLFTNSSGTQQWRGLPRYFKIRLRKRSVKN	298
UTM61951.1	NTATTVLLDEQGVGPLCKADSLYVSAADICGLFTNSSGTQQWRGLPRYFKIRLRKRSVKN	298
UTM61957.1	NTATTVLLDEQGVGPLCKADSLYVSAADICGLFTNSSGTQQWRGLPRYFKIRLRKRSVKN	298
UTM61993.1	NTATTVLLDEQGVGPLCKADSLYVSAADICGLFTNSSGTQQWRGLPRYFKIRLRKRSVKN	298
UTM61999.1	NTATTVLLDEQGVGPLCKADSLYVSAADICGLFTNSSGTQQWRGLPRYFKIRLRKRSVKN	298
UTM62011.1	NTATTVLLDEQGVGPLCKADSLYVSAADICGLFTNSSGTQQWRGLPRYFKIRLRKRSVKN	298
UTM62071.1	NTATTVLLDEQGVGPLCKADSLYVSAADICGLFTNSSGTQQWRGLPRYFKIRLRKRSVKN	298
UTM62077.1	NTATTVLLDEQGVGPLCKADSLYVSAADICGLFTNSSGTQQWRGLPRYFKIRLRKRSVKN	298
UTM62119.1	NTATTVLLDEQGVGPLCKADSLYVSAADICGLFTNSSGTQQWRGLPRYFKIRLRKRSVKN	298
UTM62125.1	NTATTVLLDEQGVGPLCKADSLYVSAADICGLFTNSSGTQQWRGLPRYFKIRLRKRSVKN	298
UTM62131.1	NTATTVLLDEQGVGPLCKADSLYVSAADICGLFTNSSGTQQWRGLPRYFKIRLRKRSVKN	298
UTM62137.1	NTATTVLLDEQGVGPLCKADSLYVSAADICGLFTNSSGTQQWRGLPRYFKIRLRKRSVKN	298
UTM62143.1	NTATTVLLDEQGVGPLCKADSLYVSAADICGLFTNSSGTQQWRGLPRYFKIRLRKRSVKN	298
UTM62401.1	NTATTVLLDEQGVGPLCKADSLYVSAADICGLFTNSSGTQQWRGLPRYFKIRLRKRSVKN	298
UTM62407.1	NTATTVLLDEQGVGPLCKADSLYVSAADICGLFTNSSGTQQWRGLPRYFKIRLRKRSVKN	298
UTM62419.1	NTATTVLLDEQGVGPLCKADSLYVSAADICGLFTNSSGTQQWRGLPRYFKIRLRKRSVKN	298

```
UTM62425.1        NTATTVLLDEQGVGPLCKADSLYVSAADICGLFTNSSGTQQWRGLPRYFKIRLRKRSVKN  298
UTM62431.1        NTATTVLLDEQGVGPLCKADSLYVSAADICGLFTNSSGTQQWRGLPRYFKIRLRKRSVKN  298
UTM62437.1        NTATTVLLDEQGVGPLCKADSLYVSAADICGLFTNSSGTQQWRGLPRYFKIRLRKRSVKN  298
UTM62641.1        NTATTVLLDEQGVGPLCKADSLYVSAADICGLFTNSSGTQQWRGLPRYFKIRLRKRSVKN  298
UTM62647.1        NTATTVLLDEQGVGPLCKADSLYVSAADICGLFTNSSGTQQWRGLPRYFKIRLRKRSVKN  298
ALF06326.1        NTATTVLLDEQGVGPLCKADSLYVSAADICGLFTNSSGTQQWRGLPRYFKIRLRKRSVKN  298
ALF06329.1        NTATTVLLDEQGVGPLCKADSLYVSAADICGLFTNSSGTQQWRGLPRYFKIRLRKRSVKN  298
AHU87338.1        NTATTVLLDEQGVGPLCKADSLYVSAADICGLFTNSSGTQQWRGLPRYFKIRLRKRSVKN  298
AHU87339.1        NTATTVLLDEQGVGPLCKADSLYVSAADICGLFTNSSGTQQWRGLPRYFKIRLRKRSVKN  298
AHU87350.1        NTATTVLLDEQGVGPLCKADSLYVSAADICGLFTNSSGTQQWRGLPRYFKIRLRKRSVKN  298
AHU87351.1        NTATTVLLDEQGVGPLCKADSLYVSAADICGLFTNSSGTQQWRGLPRYFKIRLRKRSVKN  298
AHU87352.1        NTATTVLLDEQGVGPLCKADSLYVSAADICGLFTNSSGTQQWRGLPRYFKIRLRKRSVKN  298
AHU87353.1        NTATTVLLDEQGVGPLCKADSLYVSAADICGLFTNSSGTQQWRGLPRYFKIRLRKRSVKN  298
AFQ31653.1        NTATTVLLDEQGVGPLCKADSLYVSAADICGLFTNSSGTQQWRGLPRYFKIRLRKRSVKN  298
AFA41884.1        NTATTVLLDEQGVGPLCKADSLYVSAADICGLFTNSSGTQQWRGLPRYFKIRLRKRSVKN  298
CBX88320.1        NTATTVLLDEQGVGPLCKADSLYVSAADICGLFTNSSGTQQWRGLPRYFKIRLRKRSVKN  298
CBX88326.1        NTATTVLLDEQGVGPLCKADSLYVSAADICGLFTNSSGTQQWRGLPRYFKIRLRKRSVKN  298
CBX88338.1        NTATTVLLDEQGVGPLCKADSLYVSAADICGLFTNSSGTQQWRGLPRYFKIRLRKRSVKN  298
CBX88344.1        NTATTVLLDEQGVGPLCKADSLYVSAADICGLFTNSSGTQQWRGLPRYFKIRLRKRSVKN  298
BAG84452.1        NTATTVLLDEQGVGPLCKADSLYVSAADICGLFTNSSGTQQWRGLPRYFKIRLRKRSVKN  298
BAG75385.1        NTATTVLLDEQGVGPLCKADSLYVSAADICGLFTNSSGTQQWRGLPRYFKIRLRKRSVKN  298
BAG75445.1        NTATTVLLDEQGVGPLCKADSLYVSAADICGLFTNSSGTQQWRGLPRYFKIRLRKRSVKN  298
BAF93343.1        NTATTVLLDEQGVGPLCKADSLYVSAADICGLFTNSSGTQQWRGLPRYFKIRLRKRSVKN  298
BAF75066.1        NTATTVLLDEQGVGPLCKADSLYVSAADICGLFTNSSGTQQWRGLPRYFKIRLRKRSVKN  298
BAF75072.1        NTATTVLLDEQGVGPLCKADSLYVSAADICGLFTNSSGTQQWRGLPRYFKIRLRKRSVKN  298
BAF75078.1        NTATTVLLDEQGVGPLCKADSLYVSAADICGLFTNSSGTQQWRGLPRYFKIRLRKRSVKN  298
BAF75084.1        NTATTVLLDEQGVGPLCKADSLYVSAADICGLFTNSSGTQQWRGLPRYFKIRLRKRSVKN  298
BAF75108.1        NTATTVLLDEQGVGPLCKADSLYVSAADICGLFTNSSGTQQWRGLPRYFKIRLRKRSVKN  298
BAF75132.1        NTATTVLLDEQGVGPLCKADSLYVSAADICGLFTNSSGTQQWRGLPRYFKIRLRKRSVKN  298
BAF75216.1        NTATTVLLDEQGVGPLCKADSLYVSAADICGLFTNSSGTQQWRGLPRYFKIRLRKRSVKN  298
BAF75222.1        NTATTVLLDEQGVGPLCKADSLYVSAADICGLFTNSSGTQQWRGLPRYFKIRLRKRSVKN  298
BAF75228.1        NTATTVLLDEQGVGPLCKADSLYVSAADICGLFTNSSGTQQWRGLPRYFKIRLRKRSVKN  298
BAF75246.1        NTATTVLLDEQGVGPLCKADSLYVSAADICGLFTNSSGTQQWRGLPRYFKIRLRKRSVKN  298
BAF75252.1        NTATTVLLDEQGVGPLCKADSLYVSAADICGLFTNSSGTQQWRGLPRYFKIRLRKRSVKN  298
BAF75258.1        NTATTVLLDEQGVGPLCKADSLYVSAADICGLFTNSSGTQQWRGLPRYFKIRLRKRSVKN  298
BAF75276.1        NTATTVLLDEQGVGPLCKADSLYVSAADICGLFTNSSGTQQWRGLPRYFKIRLRKRSVKN  298
BAF75330.1        NTATTVLLDEQGVGPLCKADSLYVSAADICGLFTNSSGTQQWRGLPRYFKIRLRKRSVKN  298
BAF03115.1        NTATTVLLDEQGVGPLCKADSLYVSAADICGLFTNSSGTQQWRGLPRYFKIRLRKRSVKN  298
BAF03120.1        NTATTVLLDEQGVGPLCKADSLYVSAADICGLFTNSSGTQQWRGLPRYFKIRLRKRSVKN  298
WLD47842.1        NTATTVLLDEQGVGPLCKADSLYVSAADICGLFTNSSGTQQWRGLPRYFKIRLRKRSVKN  298
WLD47884.1        NTATTVLLDEQGVGPLCKADSLYVSAADICGLFTNSSGTQQWRGLPRYFKIRLRKRSVKN  298
UTM62101.1        NTATTVLLDEQGVGPLCKADSLYVSAADICGLFTNSSGTQQWRGLPRYFKIRLRKRSVKN  298
UTM62107.1        NTATTVLLDEQGVGPLCKADSLYVSAADICGLFTNSSGTQQWRGLPRYFKIRLRKRSVKN  298
UTM62113.1        NTATTVLLDEQGVGPLCKADSLYVSAADICGLFTNSSGTQQWRGLPRYFKIRLRKRSVKN  298
AFQ31647.1        NTATTVLLDEQGVGPLCKADSLYVSAADICGLFTNSSGTQQWRGLPRYFKIRLRKRSVKN  298
AFA41892.1        NTATTVLLDEQGVGPLCKADSLYVSAADICGLFTNSSGTQQWRGLPRYFKIRLRKRSVKN  298
BAG75403.1        NTATTVLLDEQGVGPLCKADSLYVSAADICGLFTNSSGTQQWRGLPRYFKIRLRKRSVKN  298
BAF75114.1        NTATTVLLDEQGVGPLCKADSLYVSAADICGLFTNSSGTQQWRGLPRYFKIRLRKRSVKN  298
BAF75120.1        NTATTVLLDEQGVGPLCKADSLYVSAADICGLFTNSSGTQQWRGLPRYFKIRLRKRSVKN  298
BAF75126.1        NTATTVLLDEQGVGPLCKADSLYVSAADICGLFTNSSGTQQWRGLPRYFKIRLRKRSVKN  298
BAF75186.1        NTATTVLLDEQGVGPLCKADSLYVSAADICGLFTNSSGTQQWRGLPRYFKIRLRKRSVKN  298
BAF75288.1        NTATTVLLDEQGVGPLCKADSLYVSAADICGLFTNSSGTQQWRGLPRYFKIRLRKRSVKN  298
BAF75294.1        NTATTVLLDEQGVGPLCKADSLYVSAADICGLFTNSSGTQQWRGLPRYFKIRLRKRSVKN  298
BAF75348.1        NTATTVLLDEQGVGPLCKADSLYVSAADICGLFTNSSGTQQWRGLPRYFKIRLRKRSVKN  298
AFA41881.1        NTATTVLLDEQGVGPLCKADSLYVSAADICGLFTNSSGTQQWRGLPRYFKIRLRKRSVKN  298
                  *:*****************:.**** :** *** *****:***** ***: *::***.**

ALF06344.1        PYPISFLLSDLINRRTQXVDGQPMYGMESQVEEVRVFDGTEXLPGDPDMIRYIDKQGQLQ  358
QTJ15012.1        PYPISFLLSDLINRRTQRVDGQPMYGMESQVEEVRVFDGTERLPGDPDMIRYIDKQGQLQ  358
QTJ15120.1        PYPISFLLSDLINRRTQRVDGQPMYGMESQVEEVRVFDGTERLPGDPDMIRYIDKQGQLQ  358
QHI34657.1        PYPISFLLSDLINRRTQRVDGQPMYGMESQVEEVRVFDGTERLPGDPDMIRYIDKQGQLQ  358
ARE72390.1        PYPISFLLSDLINRRTQRVDGQPMYGMESQVEEVRVFDCTERLPGDPDMIRYIDKQGQLQ  358
QTJ14970.1        PYPISFLLSDLINRRTQRVDGQPMYGMESQVEEVRVFDGTERLPGDPDMIRYIDKQGQLQ  358
QTJ14928.1        PYPISFLLSDLINRRTQRVDGQPMYGMESQVEEVRVFDGTERLPGDPDMIRYIDKQGQLQ  360
```

```
AAT47419.1      PYPISFSLSDLINRRTQRVDGQPMYGMESQVEEVRVFDGTERLPGDPDMIRYIDKQGQLQ  358
AAT47413.1      PYPISSLLSDLINRRTQRVDGQPMYGMESQVEEVRVFDGTERLPGDPDMIRYIDKQGQLQ  358
AFQ31661.1      PYPISFLLSDLINRRTQRVDGQPMYGMESQVEEVRVFDGTERLPGDPDMIRYIDKQGQLQ  358
YP_717939.1     PYPISFLLSDLINRRTQRVDGQPMYGMESQVEEVRVFDGTERLPGDPDMIRYIDKQGQLQ  358
AFK08545.1      PYPISFLLSDLINRRTQRVDGQPMYGMESQVEEVRVFDGTERLPGDPDMIRYIDKQGQLQ  358
P03088.2        PYPISFLLSDLINRRTQRVDGQPMYGMESQVEEVRVFDGTERLPGDPDMIRYIDKQGQLQ  358
CAA24299.1      PYPISFLLSDLINRRTQRVDGQPMYGMESQVEEVRVFDGTERLPGDPDMIRYIDKQGQLQ  358
QTJ14922.1      PYQISFLLSDLINRRTQRVDGQPMYGMESQVEEVRVFDGTERLPGDPDMIRYIDKQGQLQ  358
AFA41877.1      PYPISFLLSDLINRRTQRVDGQPMYGMESQVEEVRVFDGTERLPGDPDMIRYIDKQGQLQ  358
AAT47371.1      PYPISFLLSDLINRRTQRVDGQPMYGMESQVGEVRVFDGTERLPGDPDMIRYIDKQGQLQ  358
AYO46658.1      PYPISFLLSDLINRRTQRVDGQPMYGMESQVEEVRVFDGTERLPGDPDMIRYIDKKGQLQ  358
AYO46610.1      PYPISFLLSDLINRRTQRVDGQPMYGMESQVEEVRVFDGTERLPGDPDMIRYIDKQGQLQ  358
AYO46689.1      PYPISFLLSDLINRRTQRVDGQPMYGMESQVEEVRVFDGTERLPGDPDMIRYIDKQGQLQ  358
AYO46693.1      PYPISFLLSDLINRRTQRVDGQPMYGMESQVEEVRVFDGTERLPGDPDMIRYIDKQGQLQ  358
BAG75283.1      PYPISFLLSDLINRRTQRVDGQPMYGMESQVEEVRVFDGTEKLPGDPDMIRYIDKQGQLQ  358
BAG75313.1      PYPISFLLSDLINRRTQRVDGQPMYGMESQVEEVRVFDGTEKLPGDPDMIRYIDKQGQLQ  358
BAG75373.1      PYPISFLLSDLINRRTQRVDGQPMYGMESQVEEVRVFDGTEKLPGDPDMIRYIDKQGQLQ  358
BCV68914.1      PYPISFLLSDLINRRTQRVDGQPMYGMESQVEEVRVFDGTEKLPGDPDMIRYIDKQGQLQ  358
BCV68917.1      PYPISFLLSDLINRRTQRVDGQPMYGMESQVEEVRVFDGTEKLPGDPDMIRYIDKQGQLQ  358
BCV68923.1      PYPISFLLSDLINRRTQRVDGQPMYGMESQVEEVRVFDGTEKLPGDPDMIRYIDKQGQLQ  358
AYO46620.1      PYPISFLLSDLINRRTQRVDGQPMYGMESQVEEVRVFDGTEKLPGDPDMIRYIDKQGQLQ  358
AYO46646.1      PYPISFLLSDLINRRTQRVDGQPMYGMESQVEEVRVFDGTEKLPGDPDMIRYIDKQGQLQ  358
AYO46663.1      PYPISFLLSDLINRRTQRVDGQPMYGMESQVEEVRVFDGTEKLPGDPDMIRYIDKQGQLQ  358
AYO46694.1      PYPISFLLSDLINRRTQRVDGQPMYGMESQVEEVRVFDGTEKLPGDPDMIRYIDKQGQLQ  358
BAZ99906.1      PYPISFLLSDLINRRTQRVDGQPMYGMESQVEEVRVFDGTEKLPGDPDMIRYIDKQGQLQ  358
BAZ99912.1      PYPISFLLSDLINRRTQRVDGQPMYGMESQVEEVRVFDGTEKLPGDPDMIRYIDKQGQLQ  358
BAI43576.1      PYPISFLLSDLINRRTQRVDGQPMYGMESQVEEVRVFDGTEKLPGDPDMIRYIDKQGQLQ  358
BAI43594.1      PYPISFLLSDLINRRTQRVDGQPMYGMESQVEEVRVFDGTEKLPGDPDMIRYIDKQGQLQ  358
BAI43606.1      PYPISFLLSDLINRRTQRVDGQPMYGMESQVEEVRVFDGTEKLPGDPDMIRYIDKQGQLQ  358
BAI43624.1      PYPISFLLSDLINRRTQRVDGQPMYGMESQVEEVRVFDGTEKLPGDPDMIRYIDKQGQLQ  358
BAH23450.1      PYPISFLLSDLINRRTQRVDGQPMYGMESQVEEVRVFDGTEKLPGDPDMIRYIDKQGQLQ  358
BAH23456.1      PYPISFLLSDLINRRTQRVDGQPMYGMESQVEEVRVFDGTEKLPGDPDMIRYIDKQGQLQ  358
BAH23462.1      PYPISFLLSDLINRRTQRVDGQPMYGMESQVEEVRVFDGTEKLPGDPDMIRYIDKQGQLQ  358
BAH23468.1      PYPISFLLSDLINRRTQRVDGQPMYGMESQVEEVRVFDGTEKLPGDPDMIRYIDKQGQLQ  358
BAH23474.1      PYPISFLLSDLINRRTQRVDGQPMYGMESQVEEVRVFDGTEKLPGDPDMIRYIDKQGQLQ  358
BAH23480.1      PYPISFLLSDLINRRTQRVDGQPMYGMESQVEEVRVFDGTEKLPGDPDMIRYIDKQGQLQ  358
BAH23486.1      PYPISFLLSDLINRRTQRVDGQPMYGMESQVEEVRVFDGTEKLPGDPDMIRYIDKQGQLQ  358
BAH23492.1      PYPISFLLSDLINRRTQRVDGQPMYGMESQVEEVRVFDGTEKLPGDPDMIRYIDKQGQLQ  358
BAH23498.1      PYPISFLLSDLINRRTQRVDGQPMYGMESQVEEVRVFDGTEKLPGDPDMIRYIDKQGQLQ  358
BAH23504.1      PYPISFLLSDLINRRTQRVDGQPMYGMESQVEEVRVFDGTEKLPGDPDMIRYIDKQGQLQ  358
BAH23510.1      PYPISFLLSDLINRRTQRVDGQPMYGMESQVEEVRVFDGTEKLPGDPDMIRYIDKQGQLQ  358
BAH23516.1      PYPISFLLSDLINRRTQRVDGQPMYGMESQVEEVRVFDGTEKLPGDPDMIRYIDKQGQLQ  358
BAH23522.1      PYPISFLLSDLINRRTQRVDGQPMYGMESQVEEVRVFDGTEKLPGDPDMIRYIDKQGQLQ  358
BAH23528.1      PYPISFLLSDLINRRTQRVDGQPMYGMESQVEEVRVFDGTEKLPGDPDMIRYIDKQGQLQ  358
BAH23534.1      PYPISFLLSDLINRRTQRVDGQPMYGMESQVEEVRVFDGTEKLPGDPDMIRYIDKQGQLQ  358
BAH23540.1      PYPISFLLSDLINRRTQRVDGQPMYGMESQVEEVRVFDGTEKLPGDPDMIRYIDKQGQLQ  358
BAH23546.1      PYPISFLLSDLINRRTQRVDGQPMYGMESQVEEVRVFDGTEKLPGDPDMIRYIDKQGQLQ  358
BAH23552.1      PYPISFLLSDLINRRTQRVDGQPMYGMESQVEEVRVFDGTEKLPGDPDMIRYIDKQGQLQ  358
BAH23558.1      PYPISFLLSDLINRRTQRVDGQPMYGMESQVEEVRVFDGTEKLPGDPDMIRYIDKQGQLQ  358
BAG84488.1      PYPISFLLSDLINRRTQRVDGQPMYGMESQVEEVRVFDGTEKLPGDPDMIRYIDKQGQLQ  358
BAG84494.1      PYPISFLLSDLINRRTQRVDGQPMYGMESQVEEVRVFDGTEKLPGDPDMIRYIDKQGQLQ  358
BAG84500.1      PYPISFLLSDLINRRTQRVDGQPMYGMESQVEEVRVFDGTEKLPGDPDMIRYIDKQGQLQ  358
BAG84506.1      PYPISFLLSDLINRRTQRVDGQPMYGMESQVEEVRVFDGTEKLPGDPDMIRYIDKQGQLQ  358
BAG84512.1      PYPISFLLSDLINRRTQRVDGQPMYGMESQVEEVRVFDGTEKLPGDPDMIRYIDKQGQLQ  358
BAG84518.1      PYPISFLLSDLINRRTQRVDGQPMYGMESQVEEVRVFDGTEKLPGDPDMIRYIDKQGQLQ  358
BAG84524.1      PYPISFLLSDLINRRTQRVDGQPMYGMESQVEEVRVFDGTEKLPGDPDMIRYIDKQGQLQ  358
BAG75163.1      PYPISFLLSDLINRRTQRVDGQPMYGMESQVEEVRVFDGTEKLPGDPDMIRYIDKQGQLQ  358
BAG75169.1      PYPISFLLSDLINRRTQRVDGQPMYGMESQVEEVRVFDGTEKLPGDPDMIRYIDKQGQLQ  358
BAG75175.1      PYPISFLLSDLINRRTQRVDGQPMYGMESQVEEVRVFDGTEKLPGDPDMIRYIDKQGQLQ  358
BAG75181.1      PYPISFLLSDLINRRTQRVDGQPMYGMESQVEEVRVFDGTEKLPGDPDMIRYIDKQGQLQ  358
BAG75187.1      PYPISFLLSDLINRRTQRVDGQPMYGMESQVEEVRVFDGTEKLPGDPDMIRYIDKQGQLQ  358
BAG75193.1      PYPISFLLSDLINRRTQRVDGQPMYGMESQVEEVRVFDGTEKLPGDPDMIRYIDKQGQLQ  358
BAG75199.1      PYPISFLLSDLINRRTQRVDGQPMYGMESQVEEVRVFDGTEKLPGDPDMIRYIDKQGQLQ  358
BAG75241.1      PYPISFLLSDLINRRTQRVDGQPMYGMESQVEEVRVFDGTEKLPGDPDMIRYIDKQGQLQ  358
```

```
BAG75247.1          PYPISFLLSDLINRRTQRVDGQPMYGMESQVEEVRVFDGTEKLPGDPDMIRYIDKQGQLQ  358
BAG75253.1          PYPISFLLSDLINRRTQRVDGQPMYGMESQVEEVRVFDGTEKLPGDPDMIRYIDKQGQLQ  358
BAG75265.1          PYPISFLLSDLINRRTQRVDGQPMYGMESQVEEVRVFDGTEKLPGDPDMIRYIDKQGQLQ  358
BAG75289.1          PYPISFLLSDLINRRTQRVDGQPMYGMESQVEEVRVFDGTEKLPGDPDMIRYIDKQGQLQ  358
BAG75295.1          PYPISFLLSDLINRRTQRVDGQPMYGMESQVEEVRVFDGTEKLPGDPDMIRYIDKQGQLQ  358
BAG75307.1          PYPISFLLSDLINRRTQRVDGQPMYGMESQVEEVRVFDGTEKLPGDPDMIRYIDKQGQLQ  358
BAG75325.1          PYPISFLLSDLINRRTQRVDGQPMYGMESQVEEVRVFDGTEKLPGDPDMIRYIDKQGQLQ  358
BAG75331.1          PYPISFLLSDLINRRTQRVDGQPMYGMESQVEEVRVFDGTEKLPGDPDMIRYIDKQGQLQ  358
BAG75337.1          PYPISFLLSDLINRRTQRVDGQPMYGMESQVEEVRVFDGTEKLPGDPDMIRYIDKQGQLQ  358
BAG75343.1          PYPISFLLSDLINRRTQRVDGQPMYGMESQVEEVRVFDGTEKLPGDPDMIRYIDKQGQLQ  358
BAG75397.1          PYPISFLLSDLINRRTQRVDGQPMYGMESQVEEVRVFDGTEKLPGDPDMIRYIDKQGQLQ  358
BAG75421.1          PYPISFLLSDLINRRTQRVDGQPMYGMESQVEEVRVFDGTEKLPGDPDMIRYIDKQGQLQ  358
BAG75427.1          PYPISFLLSDLINRRTQRVDGQPMYGMESQVEEVRVFDGTEKLPGDPDMIRYIDKQGQLQ  358
BAG75433.1          PYPISFLLSDLINRRTQRVDGQPMYGMESQVEEVRVFDGTEKLPGDPDMIRYIDKQGQLQ  358
BAF93349.1          PYPISFLLSDLINRRTQRVDGQPMYGMESQVEEVRVFDGTEKLPGDPDMIRYIDKQGQLQ  358
BAF76166.1          PYPISFLLSDLINRRTQRVDGQPMYGMESQVEEVRVFDGTEKLPGDPDMIRYIDKQGQLQ  358
BAF76172.1          PYPISFLLSDLINRRTQRVDGQPMYGMESQVEEVRVFDGTEKLPGDPDMIRYIDKQGQLQ  358
BAF76178.1          PYPISFLLSDLINRRTQRVDGQPMYGMESQVEEVRVFDGTEKLPGDPDMIRYIDKQGQLQ  358
BAF76184.1          PYPISFLLSDLINRRTQRVDGQPMYGMESQVEEVRVFDGTEKLPGDPDMIRYIDKQGQLQ  358
BAF76190.1          PYPISFLLSDLINRRTQRVDGQPMYGMESQVEEVRVFDGTEKLPGDPDMIRYIDKQGQLQ  358
BAF76202.1          PYPISFLLSDLINRRTQRVDGQPMYGMESQVEEVRVFDGTEKLPGDPDMIRYIDKQGQLQ  358
BAF42985.1          PYPISFLLSDLINRRTQRVDGQPMYGMESQVEEVRVFDGTEKLPGDPDMIRYIDKQGQLQ  358
BAF42991.1          PYPISFLLSDLINRRTQRVDGQPMYGMESQVEEVRVFDGTEKLPGDPDMIRYIDKQGQLQ  358
BAF02951.1          PYPISFLLSDLINRRTQRVDGQPMYGMESQVEEVRVFDGTEKLPGDPDMIRYIDKQGQLQ  358
BAF02963.1          PYPISFLLSDLINRRTQRVDGQPMYGMESQVEEVRVFDGTEKLPGDPDMIRYIDKQGQLQ  358
BAF02969.1          PYPISFLLSDLINRRTQRVDGQPMYGMESQVEEVRVFDGTEKLPGDPDMIRYIDKQGQLQ  358
BAF02981.1          PYPISFLLSDLINRRTQRVDGQPMYGMESQVEEVRVFDGTEKLPGDPDMIRYIDKQGQLQ  358
BAF02987.1          PYPISFLLSDLINRRTQRVDGQPMYGMESQVEEVRVFDGTEKLPGDPDMIRYIDKQGQLQ  358
BAF02993.1          PYPISFLLSDLINRRTQRVDGQPMYGMESQVEEVRVFDGTEKLPGDPDMIRYIDKQGQLQ  358
BAF02999.1          PYPISFLLSDLINRRTQRVDGQPMYGMESQVEEVRVFDGTEKLPGDPDMIRYIDKQGQLQ  358
BAF03005.1          PYPISFLLSDLINRRTQRVDGQPMYGMESQVEEVRVFDGTEKLPGDPDMIRYIDKQGQLQ  358
BAF03011.1          PYPISFLLSDLINRRTQRVDGQPMYGMESQVEEVRVFDGTEKLPGDPDMIRYIDKQGQLQ  358
BAE96065.1          PYPISFLLSDLINRRTQRVDGQPMYGMESQVEEVRVFDGTEKLPGDPDMIRYIDKQGQLQ  358
BAE53666.1          PYPISFLLSDLINRRTQRVDGQPMYGMESQVEEVRVFDGTEKLPGDPDMIRYIDKQGQLQ  358
BAE46623.1          PYPISFLLSDLINRRTQRVDGQPMYGMESQVEEVRVFDGTEKLPGDPDMIRYIDKQGQLQ  358
CAA40235.1          PYPISFLLSDLINRRTQRVDGQPMYGMESQVEEVRVFDGTEKLPGDPDMIRYIDKQGQLQ  358
BAI43570.1          PYPISFLLSDLINRRTQRVDGQPMYGMESQVEEVRVFDGTEKLPGDPDMIRYIDKQGQLQ  358
BAI43582.1          PYPISFLLSDLINRRTQRVDGQPMYGMESQVEEVRVFDGTEKLPGDPDMIRYIDKQGQLQ  358
BAI43612.1          PYPISFLLSDLINRRTQRVDGQPMYGMESQVEEVRVFDGTEKLPGDPDMIRYIDKQGQLQ  358
BAI43618.1          PYPISFLLSDLINRRTQRVDGQPMYGMESQVEEVRVFDGTEKLPGDPDMIRYIDKQGQLQ  358
BAI43630.1          PYPISFLLSDLINRRTQRVDGQPMYGMESQVEEVRVFDGTEKLPGDPDMIRYIDKQGQLQ  358
BAG75301.1          PYPISFLLSDLINRRTQRVDGQPMYGMESQVEEVRVFDGTEKLPGDPDMIRYIDKQGQLQ  358
BAG75349.1          PYPISFLLSDLINRRTQRVDGQPMYGMESQVEEVRVFDGTEKLPGDPDMIRYIDKQGQLQ  358
BAG75367.1          PYPISFLLSDLINRRTQRVDGQPMYGMESQVEEVRVFDGTEKLPGDPDMIRYIDKQGQLQ  358
BAF76196.1          PYPISFLLSDLINRRTQRVDGQPMYGMESQVEEVRVFDGTEKLPGDPDMIRYIDKQGQLQ  358
BAF02975.1          PYPISFLLSDLINRRTQRVDGQPMYGMESQVEEVRVFDGTEKLPGDPDMIRYIDKQGQLQ  358
BAI43600.1          PYPISFLLSDLINRRTQRVDGQPMYGMESQVEEVRVFDGTEKLPGDPDMIRYIDKQGQLQ  358
BAE96071.1          PYPISFLLSDLINRRTQRVDGQPMYGMESQVEEVRVFDGTEKLPGDPDMIRYIDKQGQLQ  358
BAE53660.1          PYPISFLLSDLINRRTQRVDGQPMYGMESQVEEVRVFDGTEKLPGDPDMIRYIDKQGQLQ  358
BCV68926.1          PYPISFLLSDLINRRTQRVDGQPMYGMESQVEEVRVFDGTEKLPGDPDMIRYIDKQGQLQ  358
AYO46647.1          PYPISFLLSDLINRRTQRVDGQPMYGMESQVEEVRVFDGTEKLPGDPDMIRYIDKQGQLQ  358
BAR80134.1          PYPISFLLSDLINRRTQRVDGQPMYGMESQVEEVRVFDGTEKLPGDPDMIRYIDKQGQLQ  358
BAR80140.1          PYPISFLLSDLINRRTQRVDGQPMYGMESQVEEVRVFDGTEKLPGDPDMIRYIDKQGQLQ  358
BAI43588.1          PYPISFLLSDLINRRTQRVDGQPMYGMESQVEEVRVFDGTEKLPGDPDMIRYIDKQGQLQ  358
BAG75361.1          PYPISFLLSDLINRRTQRVDGQPMYGMESQVEEVRVFDGTEKLPGDPDMIRYIDKQGQLQ  358
BAE53642.1          PYPISFLLSDLINRRTQRVDGQPMYGMESQVEEVRVFDGTEKLPGDPDMIRYIDKQGQLQ  358
BAE53648.1          PYPISFLLSDLINRRTQRVDGQPMYGMESQVEEVRVFDGTEKLPGDPDMIRYIDKQGQLQ  358
AFA41903.1          PYPISFLLSDLINRRTQRVDGQPMYGMESQVEEVRVFDGTERLPGDPDMIRYIDKQGQLQ  358
AFA41918.1          PYPISFLLSDLINRRTQRVDGQPMYGMESQVEEVRVFDGTERLPGDPDMIRYIDKQGQLQ  358
BAF42937.1          PYPISFLLSDLINRRTQRVDGQPMYGMESQVEEVRVFDGTERLPGDPDMIRYIDKQGQLQ  358
BAF42997.1          PYPISFLLSDLINRRTQRVDGQPMYGMESQVEEVRVFDGTERLPGDPDMIRYIDKQGQLQ  358
WLD47902.1          PYPISFLLSDLINRRTQRVDGQPMYGMESQVEEVRVFDGTERLPGDPDMIRYIDKQGQLQ  358
WLD47938.1          PYPISFLLSDLINRRTQRVDGQPMYGMESQVEEVRVFDGTERLPGDPDMIRYIDKQGQLQ  358
WLD47980.1          PYPISFLLSDLINRRTQRVDGQPMYGMESQVEEVRVFDGTERLPGDPDMIRYIDKQGQLQ  358
```

UTM61891.1	PYPISFLLSDLINRRTQRVDGQPMYGMESQVEEVRVFDGTERLPGDPDMIRYIDKQGQLQ	358
UTM61897.1	PYPISFLLSDLINRRTQRVDGQPMYGMESQVEEVRVFDGTERLPGDPDMIRYIDKQGQLQ	358
UTM61903.1	PYPISFLLSDLINRRTQRVDGQPMYGMESQVEEVRVFDGTERLPGDPDMIRYIDKQGQLQ	358
UTM62215.1	PYPISFLLSDLINRRTQRVDGQPMYGMESQVEEVRVFDGTERLPGDPDMIRYIDKQGQLQ	358
UTM62221.1	PYPISFLLSDLINRRTQRVDGQPMYGMESQVEEVRVFDGTERLPGDPDMIRYIDKQGQLQ	358
UTM62275.1	PYPISFLLSDLINRRTQRVDGQPMYGMESQVEEVRVFDGTERLPGDPDMIRYIDKQGQLQ	358
UTM62281.1	PYPISFLLSDLINRRTQRVDGQPMYGMESQVEEVRVFDGTERLPGDPDMIRYIDKQGQLQ	358
UTM62329.1	PYPISFLLSDLINRRTQRVDGQPMYGMESQVEEVRVFDGTERLPGDPDMIRYIDKQGQLQ	358
UTM62335.1	PYPISFLLSDLINRRTQRVDGQPMYGMESQVEEVRVFDGTERLPGDPDMIRYIDKQGQLQ	358
UTM62383.1	PYPISFLLSDLINRRTQRVDGQPMYGMESQVEEVRVFDGTERLPGDPDMIRYIDKQGQLQ	358
UTM62395.1	PYPISFLLSDLINRRTQRVDGQPMYGMESQVEEVRVFDGTERLPGDPDMIRYIDKQGQLQ	358
UTM62443.1	PYPISFLLSDLINRRTQRVDGQPMYGMESQVEEVRVFDGTERLPGDPDMIRYIDKQGQLQ	358
UTM62449.1	PYPISFLLSDLINRRTQRVDGQPMYGMESQVEEVRVFDGTERLPGDPDMIRYIDKQGQLQ	358
UTM62467.1	PYPISFLLSDLINRRTQRVDGQPMYGMESQVEEVRVFDGTERLPGDPDMIRYIDKQGQLQ	358
UTM62545.1	PYPISFLLSDLINRRTQRVDGQPMYGMESQVEEVRVFDGTERLPGDPDMIRYIDKQGQLQ	358
UTM62551.1	PYPISFLLSDLINRRTQRVDGQPMYGMESQVEEVRVFDGTERLPGDPDMIRYIDKQGQLQ	358
UTM62587.1	PYPISFLLSDLINRRTQRVDGQPMYGMESQVEEVRVFDGTERLPGDPDMIRYIDKQGQLQ	358
UTM62593.1	PYPISFLLSDLINRRTQRVDGQPMYGMESQVEEVRVFDGTERLPGDPDMIRYIDKQGQLQ	358
UTM62599.1	PYPISFLLSDLINRRTQRVDGQPMYGMESQVEEVRVFDGTERLPGDPDMIRYIDKQGQLQ	358
QUW04850.1	PYPISFLLSDLINRRTQRVDGQPMYGMESQVEEVRVFDGTERLPGDPDMIRYIDKQGQLQ	358
QTJ14976.1	PYPISFLLSDLINRRTQRVDGQPMYGMESQVEEVRVFDGTERLPGDPDMIRYIDKQGQLQ	358
QTJ15000.1	PYPISFLLSDLINRRTQRVDGQPMYGMESQVEEVRVFDGTERLPGDPDMIRYIDKQGQLQ	358
QTJ15054.1	PYPISFLLSDLINRRTQRVDGQPMYGMESQVEEVRVFDGTERLPGDPDMIRYIDKQGQLQ	358
QTJ15072.1	PYPISFLLSDLINRRTQRVDGQPMYGMESQVEEVRVFDGTERLPGDPDMIRYIDKQGQLQ	358
QTJ15084.1	PYPISFLLSDLINRRTQRVDGQPMYGMESQVEEVRVFDGTERLPGDPDMIRYIDKQGQLQ	358
QTJ15090.1	PYPISFLLSDLINRRTQRVDGQPMYGMESQVEEVRVFDGTERLPGDPDMIRYIDKQGQLQ	358
QTJ15108.1	PYPISFLLSDLINRRTQRVDGQPMYGMESQVEEVRVFDGTERLPGDPDMIRYIDKQGQLQ	358
QTJ15114.1	PYPISFLLSDLINRRTQRVDGQPMYGMESQVEEVRVFDGTERLPGDPDMIRYIDKQGQLQ	358
QTJ15132.1	PYPISFLLSDLINRRTQRVDGQPMYGMESQVEEVRVFDGTERLPGDPDMIRYIDKQGQLQ	358
QTJ15138.1	PYPISFLLSDLINRRTQRVDGQPMYGMESQVEEVRVFDGTERLPGDPDMIRYIDKQGQLQ	358
AYO46612.1	PYPISFLLSDLINRRTQRVDGQPMYGMESQVEEVRVFDGTERLPGDPDMIRYIDKQGQLQ	358
AYO46613.1	PYPISFLLSDLINRRTQRVDGQPMYGMESQVEEVRVFDGTERLPGDPDMIRYIDKQGQLQ	358
AYO46617.1	PYPISFLLSDLINRRTQRVDGQPMYGMESQVEEVRVFDGTERLPGDPDMIRYIDKQGQLQ	358
AYO46621.1	PYPISFLLSDLINRRTQRVDGQPMYGMESQVEEVRVFDGTERLPGDPDMIRYIDKQGQLQ	358
AYO46622.1	PYPISFLLSDLINRRTQRVDGQPMYGMESQVEEVRVFDGTERLPGDPDMIRYIDKQGQLQ	358
AYO46623.1	PYPISFLLSDLINRRTQRVDGQPMYGMESQVEEVRVFDGTERLPGDPDMIRYIDKQGQLQ	358
AYO46624.1	PYPISFLLSDLINRRTQRVDGQPMYGMESQVEEVRVFDGTERLPGDPDMIRYIDKQGQLQ	358
AYO46629.1	PYPISFLLSDLINRRTQRVDGQPMYGMESQVEEVRVFDGTERLPGDPDMIRYIDKQGQLQ	358
AYO46632.1	PYPISFLLSDLINRRTQRVDGQPMYGMESQVEEVRVFDGTERLPGDPDMIRYIDKQGQLQ	358
AYO46635.1	PYPISFLLSDLINRRTQRVDGQPMYGMESQVEEVRVFDGTERLPGDPDMIRYIDKQGQLQ	358
AYO46638.1	PYPISFLLSDLINRRTQRVDGQPMYGMESQVEEVRVFDGTERLPGDPDMIRYIDKQGQLQ	358
AYO46641.1	PYPISFLLSDLINRRTQRVDGQPMYGMESQVEEVRVFDGTERLPGDPDMIRYIDKQGQLQ	358
AYO46642.1	PYPISFLLSDLINRRTQRVDGQPMYGMESQVEEVRVFDGTERLPGDPDMIRYIDKQGQLQ	358
AYO46644.1	PYPISFLLSDLINRRTQRVDGQPMYGMESQVEEVRVFDGTERLPGDPDMIRYIDKQGQLQ	358
AYO46651.1	PYPISFLLSDLINRRTQRVDGQPMYGMESQVEEVRVFDGTERLPGDPDMIRYIDKQGQLQ	358
AYO46652.1	PYPISFLLSDLINRRTQRVDGQPMYGMESQVEEVRVFDGTERLPGDPDMIRYIDKQGQLQ	358
AYO46659.1	PYPISFLLSDLINRRTQRVDGQPMYGMESQVEEVRVFDGTERLPGDPDMIRYIDKQGQLQ	358
AYO46660.1	PYPISFLLSDLINRRTQRVDGQPMYGMESQVEEVRVFDGTERLPGDPDMIRYIDKQGQLQ	358
AYO46662.1	PYPISFLLSDLINRRTQRVDGQPMYGMESQVEEVRVFDGTERLPGDPDMIRYIDKQGQLQ	358
AYO46665.1	PYPISFLLSDLINRRTQRVDGQPMYGMESQVEEVRVFDGTERLPGDPDMIRYIDKQGQLQ	358
AYO46670.1	PYPISFLLSDLINRRTQRVDGQPMYGMESQVEEVRVFDGTERLPGDPDMIRYIDKQGQLQ	358
AYO46672.1	PYPISFLLSDLINRRTQRVDGQPMYGMESQVEEVRVFDGTERLPGDPDMIRYIDKQGQLQ	358
AYO46676.1	PYPISFLLSDLINRRTQRVDGQPMYGMESQVEEVRVFDGTERLPGDPDMIRYIDKQGQLQ	358
AYO46677.1	PYPISFLLSDLINRRTQRVDGQPMYGMESQVEEVRVFDGTERLPGDPDMIRYIDKQGQLQ	358
AYO46679.1	PYPISFLLSDLINRRTQRVDGQPMYGMESQVEEVRVFDGTERLPGDPDMIRYIDKQGQLQ	358
AYO46682.1	PYPISFLLSDLINRRTQRVDGQPMYGMESQVEEVRVFDGTERLPGDPDMIRYIDKQGQLQ	358
AYO46691.1	PYPISFLLSDLINRRTQRVDGQPMYGMESQVEEVRVFDGTERLPGDPDMIRYIDKQGQLQ	358
AYO46692.1	PYPISFLLSDLINRRTQRVDGQPMYGMESQVEEVRVFDGTERLPGDPDMIRYIDKQGQLQ	358
AUF70040.1	PYPISFLLSDLINRRTQRVDGQPMYGMESQVEEVRVFDGTERLPGDPDMIRYIDKQGQLQ	358
SOH95722.1	PYPISFLLSDLINRRTQRVDGQPMYGMESQVEEVRVFDGTERLPGDPDMIRYIDKQGQLQ	358
SOI09711.1	PYPISFLLSDLINRRTQRVDGQPMYGMESQVEEVRVFDGTERLPGDPDMIRYIDKQGQLQ	358
APG38208.1	PYPISFLLSDLINRRTQRVDGQPMYGMESQVEEVRVFDGTERLPGDPDMIRYIDKQGQLQ	358
ALF06332.1	PYPISFLLSDLINRRTQRVDGQPMYGMESQVEEVRVFDGTERLPGDPDMIRYIDKQGQLQ	358
ALF06341.1	PYPISFLLSDLINRRTQRVDGQPMYGMESQVEEVRVFDGTERLPGDPDMIRYIDKQGQLQ	358

```
BAR80122.1        PYPISFLLSDLINRRTQRVDGQPMYGMESQVEEVRVFDGTERLPGDPDMIRYIDKQGQLQ  358
BAR80128.1        PYPISFLLSDLINRRTQRVDGQPMYGMESQVEEVRVFDGTERLPGDPDMIRYIDKQGQLQ  358
AJG42164.1        PYPISFLLSDLINRRTQRVDGQPMYGMESQVEEVRVFDGTERLPGDPDMIRYIDKQGQLQ  358
AHU87340.1        PYPISFLLSDLINRRTQRVDGQPMYGMESQVEEVRVFDGTERLPGDPDMIRYIDKQGQLQ  358
AHU87341.1        PYPISFLLSDLINRRTQRVDGQPMYGMESQVEEVRVFDGTERLPGDPDMIRYIDKQGQLQ  358
AHU87368.1        PYPISFLLSDLINRRTQRVDGQPMYGMESQVEEVRVFDGTERLPGDPDMIRYIDKQGQLQ  358
AHU87369.1        PYPISFLLSDLINRRTQRVDGQPMYGMESQVEEVRVFDGTERLPGDPDMIRYIDKQGQLQ  358
AFQ31636.1        PYPISFLLSDLINRRTQRVDGQPMYGMESQVEEVRVFDGTERLPGDPDMIRYIDKQGQLQ  358
AFQ31637.1        PYPISFLLSDLINRRTQRVDGQPMYGMESQVEEVRVFDGTERLPGDPDMIRYIDKQGQLQ  358
AFQ31639.1        PYPISFLLSDLINRRTQRVDGQPMYGMESQVEEVRVFDGTERLPGDPDMIRYIDKQGQLQ  358
AFQ31640.1        PYPISFLLSDLINRRTQRVDGQPMYGMESQVEEVRVFDGTERLPGDPDMIRYIDKQGQLQ  358
AFQ31641.1        PYPISFLLSDLINRRTQRVDGQPMYGMESQVEEVRVFDGTERLPGDPDMIRYIDKQGQLQ  358
AFQ31644.1        PYPISFLLSDLINRRTQRVDGQPMYGMESQVEEVRVFDGTERLPGDPDMIRYIDKQGQLQ  358
AFQ31648.1        PYPISFLLSDLINRRTQRVDGQPMYGMESQVEEVRVFDGTERLPGDPDMIRYIDKQGQLQ  358
AFQ31657.1        PYPISFLLSDLINRRTQRVDGQPMYGMESQVEEVRVFDGTERLPGDPDMIRYIDKQGQLQ  358
AFA41882.1        PYPISFLLSDLINRRTQRVDGQPMYGMESQVEEVRVFDGTERLPGDPDMIRYIDKQGQLQ  358
AFA41887.1        PYPISFLLSDLINRRTQRVDGQPMYGMESQVEEVRVFDGTERLPGDPDMIRYIDKQGQLQ  358
AFA41890.1        PYPISFLLSDLINRRTQRVDGQPMYGMESQVEEVRVFDGTERLPGDPDMIRYIDKQGQLQ  358
AFA41891.1        PYPISFLLSDLINRRTQRVDGQPMYGMESQVEEVRVFDGTERLPGDPDMIRYIDKQGQLQ  358
AFA41895.1        PYPISFLLSDLINRRTQRVDGQPMYGMESQVEEVRVFDGTERLPGDPDMIRYIDKQGQLQ  358
AFA41898.1        PYPISFLLSDLINRRTQRVDGQPMYGMESQVEEVRVFDGTERLPGDPDMIRYIDKQGQLQ  358
AFA41901.1        PYPISFLLSDLINRRTQRVDGQPMYGMESQVEEVRVFDGTERLPGDPDMIRYIDKQGQLQ  358
AFA41902.1        PYPISFLLSDLINRRTQRVDGQPMYGMESQVEEVRVFDGTERLPGDPDMIRYIDKQGQLQ  358
AFA41910.1        PYPISFLLSDLINRRTQRVDGQPMYGMESQVEEVRVFDGTERLPGDPDMIRYIDKQGQLQ  358
AFA41914.1        PYPISFLLSDLINRRTQRVDGQPMYGMESQVEEVRVFDGTERLPGDPDMIRYIDKQGQLQ  358
AFA41917.1        PYPISFLLSDLINRRTQRVDGQPMYGMESQVEEVRVFDGTERLPGDPDMIRYIDKQGQLQ  358
AEO89562.1        PYPISFLLSDLINRRTQRVDGQPMYGMESQVEEVRVFDGTERLPGDPDMIRYIDKQGQLQ  358
AEO89568.1        PYPISFLLSDLINRRTQRVDGQPMYGMESQVEEVRVFDGTERLPGDPDMIRYIDKQGQLQ  358
AEO89586.1        PYPISFLLSDLINRRTQRVDGQPMYGMESQVEEVRVFDGTERLPGDPDMIRYIDKQGQLQ  358
AEO89598.1        PYPISFLLSDLINRRTQRVDGQPMYGMESQVEEVRVFDGTERLPGDPDMIRYIDKQGQLQ  358
AEO89621.1        PYPISFLLSDLINRRTQRVDGQPMYGMESQVEEVRVFDGTERLPGDPDMIRYIDKQGQLQ  358
AEK21505.1        PYPISFLLSDLINRRTQRVDGQPMYGMESQVEEVRVFDGTERLPGDPDMIRYIDKQGQLQ  358
BAG84458.1        PYPISFLLSDLINRRTQRVDGQPMYGMESQVEEVRVFDGTERLPGDPDMIRYIDKQGQLQ  358
BAG84470.1        PYPISFLLSDLINRRTQRVDGQPMYGMESQVEEVRVFDGTERLPGDPDMIRYIDKQGQLQ  358
BAG84482.1        PYPISFLLSDLINRRTQRVDGQPMYGMESQVEEVRVFDGTERLPGDPDMIRYIDKQGQLQ  358
BAG75319.1        PYPISFLLSDLINRRTQRVDGQPMYGMESQVEEVRVFDGTERLPGDPDMIRYIDKQGQLQ  358
BAG75355.1        PYPISFLLSDLINRRTQRVDGQPMYGMESQVEEVRVFDGTERLPGDPDMIRYIDKQGQLQ  358
BAG75391.1        PYPISFLLSDLINRRTQRVDGQPMYGMESQVEEVRVFDGTERLPGDPDMIRYIDKQGQLQ  358
BAG75439.1        PYPISFLLSDLINRRTQRVDGQPMYGMESQVEEVRVFDGTERLPGDPDMIRYIDKQGQLQ  358
BAF93301.1        PYPISFLLSDLINRRTQRVDGQPMYGMESQVEEVRVFDGTERLPGDPDMIRYIDKQGQLQ  358
BAF93331.1        PYPISFLLSDLINRRTQRVDGQPMYGMESQVEEVRVFDGTERLPGDPDMIRYIDKQGQLQ  358
BAF93355.1        PYPISFLLSDLINRRTQRVDGQPMYGMESQVEEVRVFDGTERLPGDPDMIRYIDKQGQLQ  358
BAF76208.1        PYPISFLLSDLINRRTQRVDGQPMYGMESQVEEVRVFDGTERLPGDPDMIRYIDKQGQLQ  358
BAF42877.1        PYPISFLLSDLINRRTQRVDGQPMYGMESQVEEVRVFDGTERLPGDPDMIRYIDKQGQLQ  358
BAF42883.1        PYPISFLLSDLINRRTQRVDGQPMYGMESQVEEVRVFDGTERLPGDPDMIRYIDKQGQLQ  358
BAF42889.1        PYPISFLLSDLINRRTQRVDGQPMYGMESQVEEVRVFDGTERLPGDPDMIRYIDKQGQLQ  358
BAF42961.1        PYPISFLLSDLINRRTQRVDGQPMYGMESQVEEVRVFDGTERLPGDPDMIRYIDKQGQLQ  358
BAF42967.1        PYPISFLLSDLINRRTQRVDGQPMYGMESQVEEVRVFDGTERLPGDPDMIRYIDKQGQLQ  358
BAF42973.1        PYPISFLLSDLINRRTQRVDGQPMYGMESQVEEVRVFDGTERLPGDPDMIRYIDKQGQLQ  358
BAF43003.1        PYPISFLLSDLINRRTQRVDGQPMYGMESQVEEVRVFDGTERLPGDPDMIRYIDKQGQLQ  358
BAF43027.1        PYPISFLLSDLINRRTQRVDGQPMYGMESQVEEVRVFDGTERLPGDPDMIRYIDKQGQLQ  358
BAF43033.1        PYPISFLLSDLINRRTQRVDGQPMYGMESQVEEVRVFDGTERLPGDPDMIRYIDKQGQLQ  358
ABI94659.1        PYPISFLLSDLINRRTQRVDGQPMYGMESQVEEVRVFDGTERLPGDPDMIRYIDKQGQLQ  358
ABI94683.1        PYPISFLLSDLINRRTQRVDGQPMYGMESQVEEVRVFDGTERLPGDPDMIRYIDKQGQLQ  358
ABI94719.1        PYPISFLLSDLINRRTQRVDGQPMYGMESQVEEVRVFDGTERLPGDPDMIRYIDKQGQLQ  358
BAE96053.1        PYPISFLLSDLINRRTQRVDGQPMYGMESQVEEVRVFDGTERLPGDPDMIRYIDKQGQLQ  358
BAE96059.1        PYPISFLLSDLINRRTQRVDGQPMYGMESQVEEVRVFDGTERLPGDPDMIRYIDKQGQLQ  358
ABC18005.1        PYPISFLLSDLINRRTQRVDGQPMYGMESQVEEVRVFDGTERLPGDPDMIRYIDKQGQLQ  358
AAT47377.1        PYPISFLLSDLINRRTQRVDGQPMYGMESQVEEVRVFDGTERLPGDPDMIRYIDKQGQLQ  358
AAT47383.1        PYPISFLLSDLINRRTQRVDGQPMYGMESQVEEVRVFDGTERLPGDPDMIRYIDKQGQLQ  358
AAT47405.1        PYPISFLLSDLINRRTQRVDGQPMYGMESQVEEVRVFDGTERLPGDPDMIRYIDKQGQLQ  358
CAA40239.1        PYPISFLLSDLINRRTQRVDGQPMYGMESQVEEVRVFDGTERLPGDPDMIRYIDKQGQLQ  358
QTJ14940.1        PYPISFLLSDLINRRTQRVDGQPMYGMESQVEEVRVFDGTERLPGDPDMIRYIDKQGQLQ  358
ALF06317.1        PYPISFLLSDLINRRTQRVDGQPMYGMESQVEEVRVFDGTERLPGDPDMIRYIDKQGQLQ  358
```

ALF06307.1	PYPISFLLSDLINRRTQRVDGQPMYGMESQVEEVRVFDGTERLPGDPDMIRYIDKQGQLQ	358
ALF06297.1	PYPISFLLSDLINRRTQRVDGQPMYGMESQVEEVRVFDGTERLPGDPDMIRYIDKQGQLQ	358
ALF06350.1	PYPISFLLSDLINRRTQRVDGQPMYGMESQVEEVRVFDGTEXLPGDPDMIRYIDKQGQLQ	358
AAT47431.1	PYPISFLLSDLINRRTQRVDGQPMYGMESQVEEVRVFDGTERLPGDPDMIRYIDKQGQLQ	358
QTJ15006.1	PYPISFLLSDLINRRTQRVDGQPMYGMESQVEEVRVFDGTERLPGDPDMIRYIDKQGQLQ	358
AYO46667.1	PYPISFLLSDLINRRTQRVDGQPMYGMESQVEEVRVFDGTERLPGDPDMIRYIDKQGQLQ	358
WLD47956.1	PYPISFLLSDLINRRTQRVDGQPMYGMESQVEEVRVFDGTERLPGDPDMIRYIDKQGQLQ	358
QTJ15150.1	PYPISFLLSDLINRRTQRVDGQPMYGMESQVEEVRVFDGTERLPGDPDMIRYIDKQGQLQ	358
UTM62389.1	PYPISFLLSDLINRRTQRVDGQPMYGMESQVEEVRVFDGTERLPGDPDMIRYIDKQGQLQ	358
ABI94701.1	PYPISFLLSDLINRRTQRVDGQPMYGMESQVEEVRVFDGTERLPGDPDMIRYIDKQGQLQ	358
BAF02945.1	PYPISFLLSDLINRRTQRVDGQPMYGMESQVEEVRVFDGTERLPGDPDMIRYIDKQGQLQ	358
CAA40243.1	PYPISFLLSDLINRRTQRVDGQPMYGMESQVEEVRVFDGTERLPGDPDMIRYIDKQGQLQ	358
ABI94725.1	PYPISFLLSDLINRRTQRVDGQPMYGMESQVEEVRVFDGTERLPGDPDMIRYIDKQGQLQ	358
AFQ31660.1	PYPISFLLSDLINRRTQRVDGQPLYGMESQVEEVRVFDGTERLPGDPDMIRYIDKQGQLQ	358
ALF06353.1	PYPISFLLSDLINRRTQRVDGQPMYGMESQVEEVRVFDGTERLPGDPDMIRYIDKQGQLQ	358
ALF06312.1	PYPISFLLSDLINRRTQRVDGQPMYGMESQVEEVRVFDGTERLPGDPDMIRYIDKQGQLQ	358
QEJ80721.1	PYPISFLLSDLINRRTQRVDGQPMYGMESQVEEVRVFDGTERLPGDPDMIRYIDKQGQLQ	358
AYO46614.1	PYPISFLLSDLINRRTQRVDGQPMYGMESQVEEVRVFDGTERLPGDPDMIRYIDKQGQLQ	358
AYO46619.1	PYPISFLLSDLINRRTQRVDGQPMYGMESQVEEVRVFDGTERLPGDPDMIRYIDKQGQLQ	358
AYO46625.1	PYPISFLLSDLINRRTQRVDGQPMYGMESQVEEVRVFDGTERLPGDPDMIRYIDKQGQLQ	358
AYO46626.1	PYPISFLLSDLINRRTQRVDGQPMYGMESQVEEVRVFDGTERLPGDPDMIRYIDKQGQLQ	358
AYO46628.1	PYPISFLLSDLINRRTQRVDGQPMYGMESQVEEVRVFDGTERLPGDPDMIRYIDKQGQLQ	358
AYO46633.1	PYPISFLLSDLINRRTQRVDGQPMYGMESQVEEVRVFDGTERLPGDPDMIRYIDKQGQLQ	358
AYO46636.1	PYPISFLLSDLINRRTQRVDGQPMYGMESQVEEVRVFDGTERLPGDPDMIRYIDKQGQLQ	358
AYO46637.1	PYPISFLLSDLINRRTQRVDGQPMYGMESQVEEVRVFDGTERLPGDPDMIRYIDKQGQLQ	358
AYO46640.1	PYPISFLLSDLINRRTQRVDGQPMYGMESQVEEVRVFDGTERLPGDPDMIRYIDKQGQLQ	358
AYO46648.1	PYPISFLLSDLINRRTQRVDGQPMYGMESQVEEVRVFDGTERLPGDPDMIRYIDKQGQLQ	358
AYO46650.1	PYPISFLLSDLINRRTQRVDGQPMYGMESQVEEVRVFDGTERLPGDPDMIRYIDKQGQLQ	358
AYO46653.1	PYPISFLLSDLINRRTQRVDGQPMYGMESQVEEVRVFDGTERLPGDPDMIRYIDKQGQLQ	358
AYO46654.1	PYPISFLLSDLINRRTQRVDGQPMYGMESQVEEVRVFDGTERLPGDPDMIRYIDKQGQLQ	358
AYO46671.1	PYPISFLLSDLINRRTQRVDGQPMYGMESQVEEVRVFDGTERLPGDPDMIRYIDKQGQLQ	358
AYO46673.1	PYPISFLLSDLINRRTQRVDGQPMYGMESQVEEVRVFDGTERLPGDPDMIRYIDKQGQLQ	358
AYO46674.1	PYPISFLLSDLINRRTQRVDGQPMYGMESQVEEVRVFDGTERLPGDPDMIRYIDKQGQLQ	358
AYO46675.1	PYPISFLLSDLINRRTQRVDGQPMYGMESQVEEVRVFDGTERLPGDPDMIRYIDKQGQLQ	358
AYO46680.1	PYPISFLLSDLINRRTQRVDGQPMYGMESQVEEVRVFDGTERLPGDPDMIRYIDKQGQLQ	358
AYO46681.1	PYPISFLLSDLINRRTQRVDGQPMYGMESQVEEVRVFDGTERLPGDPDMIRYIDKQGQLQ	358
AYO46684.1	PYPISFLLSDLINRRTQRVDGQPMYGMESQVEEVRVFDGTERLPGDPDMIRYIDKQGQLQ	358
AYO46687.1	PYPISFLLSDLINRRTQRVDGQPMYGMESQVEEVRVFDGTERLPGDPDMIRYIDKQGQLQ	358
AEO89574.1	PYPISFLLSDLINRRTQRVDGQPMYGMESQVEEVRVFDGTERLPGDPDMIRYIDKQGQLQ	358
BAF42979.1	PYPISFLLSDLINRRTQRVDGQPMYGMESQVEEVRVFDGTERLPGDPDMIRYIDKQGQLQ	358
BAF43009.1	PYPISFLLSDLINRRTQRVDGQPMYGMESQVEEVRVFDGTERLPGDPDMIRYIDKQGQLQ	358
AYO46678.1	PYPISFLLSDLINRRTQRVDGQPMYGMESQVEEVRVFDGTERLPGDPDMIRYIDKQGQLQ	358
UTM62479.1	PYPISFLLSDLINRRTQRVDGQPMYGMESQVEEVRVFDGTERLPGDPDMIRYIDKQGQLQ	358
QTJ14964.1	PYPISFLLSDLINRRTQRVDGQPMYGMESQVEEVRVFDGTERLPGDPDMIRYIDKQGQLQ	358
QTJ15036.1	PYPISFLLSDLINRRTQRVDGQPMYGMESQVEEVRVFDGTERLPGDPDMIRYIDKQGQLQ	358
QTJ15066.1	PYPISFLLSDLINRRTQRVDGQPMYGMESQVEEVRVFDGTERLPGDPDMIRYIDKQGQLQ	358
QTJ15078.1	PYPISFLLSDLINRRTQRVDGQPMYGMESQVEEVRVFDGTERLPGDPDMIRYIDKQGQLQ	358
QTJ15096.1	PYPISFLLSDLINRRTQRVDGQPMYGMESQVEEVRVFDGTERLPGDPDMIRYIDKQGQLQ	358
AQM40276.1	PYPISFLLSDLINRRTQRVDGQPMYGMESQVEEVRVFDGTERLPGDPDMIRYIDKQGQLQ	358
ABI94689.1	PYPISFLLSDLINRRTQRVDGQPMYGMESQVEEVRVFDGTERLPGDPDMIRYIDKQGQLQ	358
ABI94671.1	PYPISFLLSDLINRRTQRVDGQPMYGMESQVEEVRVFDGTERLPGDPDMIRYIDKQGQLQ	358
AYO46688.1	PYPISFLLSDLINRRTQRVDGQPMYGMESQVEEVRVFDGTERLPGDPDMIRYIDKQGQLQ	358
QTJ15060.1	PYPISFLLSDLINRRTQRVDGQPMYGMESQVEEVRVFDGTERLPGDPDMIRYIDKQGQLQ	358
AFQ31642.1	PYPISFLLSDLINRRTQRVDGQPMYGMESQVEEVRVFDGTERLPGDPDMIRYIDKQGQLQ	358
AYO46616.1	PYPISFLLSDLINRRTQRVDGQPMYGMESQVEEVRVFDGTERLPGDPDMIRYIDKQGQLQ	358
CAA79594.1	PYPISFLLSDLINRRTQRVDGQPMYGMESQVEEVRVFDGTEQLPGDPDMIRYIDRQGQLQ	358
WLD47818.1	PYPISFLLSDLINRRTQRVDGQPMYGMESQVEEVRVFDGTERLPGDPDMIRYIDKQGQLQ	358
UTM62155.1	PYPISFLLSDLINRRTQRVDGQPMYGMESQVEEVRVFDGTERLPGDPDMIRYIDKQGQLQ	358
AFA41907.1	PYPISFLLSDLINRRTQRVDGQPMYGMESQVEEVRVFDGTERLPGDPDMIRYIDKQGQLQ	358
WLD47944.1	PYPISFLLSDLINRRTQRVDGQPMYGMESQVEEVRVFDGTERLPGDPDMIRYIDKQGQLQ	358
AYO46668.1	PYPISFLLSDLINRRTQRVDGQPMYGMESQVEEVRVFDGTERLPGDPDMIRYIDKQGQLQ	358
AFQ31651.1	PYPISFLLSDLINRRTQRVDGQPMYGMESQVEEVRVFDGTERLPGDPDMIRYIDKQGQLQ	358
QTJ14958.1	PYPISFLLSDLINRRTQRVDGQPMYGMESQVEEVRVFDGTERLPGDPDMIRYIDKQGQLQ	358
AYO46618.1	PYPISFLLSDLINRRTQRVDGQPMYGMESQVEEVRVFDGTERLPGDPDMIRYIDKQGQLQ	358

QTJ15018.1	PYPISFLLSDLINRRTQRVDGQPMYGMESQVEEVRVFDGIERLPGDPDMIRYIDKQGQLQ	358
AYO46609.1	PYPISFLLSDLINRRTQRVDGQPMYGMESQVEEVRVFDGTERLPGDPDMIRYIDKQGQLE	358
ALF06356.1	PYPISFLLSDLINRRTQRVDGQPMYGMESQVEEVRVFDGTERLPGDPDMIRYIDKQGQLQ	358
AFQ31646.1	PYPISFLLSDLINRRTQRVDGQPMYGMESQVEEVRVFDGTERLPGDPDMIRYIDKTGQLQ	358
AFQ31659.1	PYPISFLLSDLINRRTQRVDGQPMYGMESQVEEVRVFDGTERLPGDPDMIRYIDKQGQLQ	358
BAF02957.1	PYPISFLLSDLINRRTQRVDGQPMYGMESQVEEVRVFDGTERLPGDPDMIRYIDKQGQLQ	358
AAT47365.1	PYPISFLLSDLINRRTQRVDGQPMYGMESQVEEVRVFDGTERLPGDPDMIRYIDRQGQLQ	358
AAT47389.1	PYPISFLLSDLITRRTQRVDGQPMYGMESQVEEVRVFDGTERLPGDPDMIRYIDKQGQLQ	358
AAT47395.1	PYPISFLLSDLINRRTQRVDGQPMYGMESQVEEVRVFDGTERLPGDPDMIRYIDKQGQLQ	358
AAT47401.1	PYPISFLLSDLINRRTQRVDGQPMYGMESQVEEVRVFDGTERLPGDPDMIRYIDKQGQLQ	358
CAA24307.1	PYLISFLLSDLINRRTQRVDGQPMYGMESQVEEVRVFDGTERLPGDPDMIRYIDKQGQLQ	358
AAT47425.1	PYPISFLLSDLINRRTQRVDGQPMYGMESQVEEVRVFDGTERLPGDPDMIRYIDKQGQLQ	358
AFA41880.1	PYPISFLLSDLINRRTQRVDGQPMYGMESQVEEVRVFDGTEKSSRDPDMIRYIDKQGQLQ	358
WLD47968.1	PYPISFLLSDLINRRTQRVDGQPMYGMESQVEEVRVFDGTEKLPGDPDMIRYIDKQGQLQ	358
AFA41920.1	PYPISFLLSDLINRRTQRVDGQPMYGMESQVEEVRVFDGTEKLPGDPDMIRYIDKQGQLQ	358
CBX88302.1	PYPISFLLSDLINRRTQRVDGQPMYGMESQVEEVRVFDGTEKLPGDPDMIRYIDKQGQLQ	358
AUF70036.1	PYPISFLLSDLINRRTQRVDGQPMYGMESQVEEVRVFDGTEKLPGDPDMIRYIDKQGQLQ	358
ALF06323.1	PYPISFLLSDLINRRTQRVDGQPMYGMESQVEEVRVFDGTERLPGDPDMIRYIDKQGQLQ	358
WLD47890.1	PYPISFLLSDLINRRTQRVDGQPMYGMESQVEEVRVFDGTEKLPGDPDMIRYIDKQGQLQ	358
BAF03097.1	PYPISFLLSDLINRRTQRVDGQPMYGMESQVEEVRVFDGTERLPGDPDMIRYIDKQGQLQ	358
ABI94695.1	PYPISFLLSDLINRRTQRVDGQPMYGMESQVEEVRVFDGTERLPGDPDMIRYIDKQGQLQ	358
ABI94623.1	PYPISFLLSDLINRRTQRVDGQPMYGMESQVEEVRVFDGTERLPGDPDMIRYIDKQGQLQ	358
UTM62533.1	PYPISFLLSDLINRRTQRVDGQPMYGMESQVEEVRVFDGTERLPGDPDMIRYIDKQGQLQ	358
QOS45058.1	PYPISFLLSDLINRRTQRVDGQPMYGMESQVEEVRVFDGTERLPGDPDMIRYIDKQGQLQ	358
AHU87362.1	PYPISFLLSDLINRRTQRVDGQPMYGMESQVEEVRVFDGTERLPGDPDMIRYIDKQGQLQ	358
AHU87363.1	PYPISFLLSDLINRRTQRVDGQPMYGMESQVEEVRVFDGTERLPGDPDMIRYIDKQGQLQ	358
AFQ31658.1	PYPISFLLSDLINRRTQRVDGQPMYGMESQVEEVRVFDGTERLPGDPDMIRYIDKQGQLQ	358
AFA41876.1	PYPISFLLSDLINRRTQRVDGQPMYGMESQVEEVRVFDGTERLPGDPDMIRYIDKQGQLQ	358
AFA41906.1	PYPISFLLSDLINRRTQRVDGQPMYGMESQVEEVRVFDGTERLPGDPDMIRYIDKQGQLQ	358
AEO89604.1	PYPISFLLSDLINRRTQRVDGQPMYGMESQVEEVRVFDGTERLPGDPDMIRYIDKQGQLQ	358
CBX88350.1	PYPISFLLSDLINRRTQRVDGQPMYGMESQVEEVRVFDGTERLPGDPDMIRYIDKQGQLQ	358
BAG84446.1	PYPISFLLSDLINRRTQRVDGQPMYGMESQVEEVRVFDGTERLPGDPDMIRYIDKQGQLQ	358
BAG84464.1	PYPISFLLSDLINRRTQRVDGQPMYGMESQVEEVRVFDGTERLPGDPDMIRYIDKQGQLQ	358
BAF93295.1	PYPISFLLSDLINRRTQRVDGQPMYGMESQVEEVRVFDGTERLPGDPDMIRYIDKQGQLQ	358
BAF93373.1	PYPISFLLSDLINRRTQRVDGQPMYGMESQVEEVRVFDGTERLPGDPDMIRYIDKQGQLQ	358
BAF93379.1	PYPISFLLSDLINRRTQRVDGQPMYGMESQVEEVRVFDGTERLPGDPDMIRYIDKQGQLQ	358
BAF42913.1	PYPISFLLSDLINRRTQRVDGQPMYGMESQVEEVRVFDGTERLPGDPDMIRYIDKQGQLQ	358
BAF42931.1	PYPISFLLSDLINRRTQRVDGQPMYGMESQVEEVRVFDGTERLPGDPDMIRYIDKQGQLQ	358
BAF43015.1	PYPISFLLSDLINRRTQRVDGQPMYGMESQVEEVRVFDGTERLPGDPDMIRYIDKQGQLQ	358
ABI94629.1	PYPISFLLSDLINRRTQRVDGQPMYGMESQVEEVRVFDGTERLPGDPDMIRYIDKQGQLQ	358
ABI94653.1	PYPISFLLSDLINRRTQRVDGQPMYGMESQVEEVRVFDGTERLPGDPDMIRYIDKQGQLQ	358
ABI94707.1	PYPISFLLSDLINRRTQRVDGQPMYGMESQVEEVRVFDGTERLPGDPDMIRYIDKQGQLQ	358
BAF03085.1	PYPISFLLSDLINRRTQRVDGQPMYGMESQVEEVRVFDGTERLPGDPDMIRYIDKQGQLQ	358
BAF03091.1	PYPISFLLSDLINRRTQRVDGQPMYGMESQVEEVRVFDGTERLPGDPDMIRYIDKQGQLQ	358
BAF03103.1	PYPISFLLSDLINRRTQRVDGQPMYGMESQVEEVRVFDGTERLPGDPDMIRYIDKQGQLQ	358
BAF03109.1	PYPISFLLSDLINRRTQRVDGQPMYGMESQVEEVRVFDGTERLPGDPDMIRYIDKQGQLQ	358
BAF93325.1	PYPISFLLSDLINRRTQRVDGQPMYGMESQVEEVRVFDGTERLPGDPDMIRYIDKQGQLQ	358
QTJ14934.1	PYPISFLLSDLINRRTQRVDGQPMYGMESQVEEVRVFDGTERLPGDPDMIRYIDKQGQLQ	358
QTJ14994.1	PYPISFLLSDLINRRTQRVDGQPMYGMESQVEEVRVFDGTERLPGDPDMIRYIDKQGQLQ	358
AFA41886.1	PYPISFLLSDLINRRTQRVDGQPMYGMESQVEEVRVFDGTERLPGDPDMIRYIDKQGQLQ	358
AFQ31650.1	PYPISFLLSDLINRRTQRVDGQPMYGMESQVEEVRVFDGTERLPGDPDMIRYIDKQGQLQ	358
AAT47347.1	PYPISFLLSDLINRGTQRVDGQPMYGMESQVEEVRVFDGTERLPGDPDMIRYIDKQGQLQ	358
ABI94617.1	PYPISFLLSDLINRRTQRVDGQPMYGMESQVEEVRVFDGTERLPGDPDMIRYIDKQGQLQ	358
BAF93319.1	PYPISFLLSDLINRRTQRVDGQPMYGMESQVEEVRVFDGTERLPGDPDMIRYIDKQGQLQ	358
CBX88314.1	PYPISFLLSDLINRRTQRVDGQPMYGMESQVEEVRVFDGTERLPGDPDMIRYIDKQGQLQ	358
ALF06320.1	PYPISFSLSDLINRRTQRVDGQPMYGMESQVEEVRVFDGTERLPGDPDMIRYIDKQGQLQ	358
WLD47830.1	PYPISFLLSDLINRRTQRVDGQPMYGMESQVEEVRVFDGTERLPGDPDMIRYIDKQGQLQ	358
WLD47866.1	PYPISFLLSDLINRRTQRVDGQPMYGMESQVEEVRVFDGTERLPGDPDMIRYIDKQGQLQ	358
UTM61939.1	PYPISFLLSDLINRRTQRVDGQPMYGMESQVEEVRVFDGTERLPGDPDMIRYIDKQGQLQ	358
UTM62023.1	PYPISFLLSDLINRRTQRVDGQPMYGMESQVEEVRVFDGTERLPGDPDMIRYIDKQGQLQ	358
UTM62029.1	PYPISFLLSDLINRRTQRVDGQPMYGMESQVEEVRVFDGTERLPGDPDMIRYIDKQGQLQ	358
UTM62053.1	PYPISFLLSDLINRRTQRVDGQPMYGMESQVEEVRVFDGTERLPGDPDMIRYIDKQGQLQ	358
UTM62059.1	PYPISFLLSDLINRRTQRVDGQPMYGMESQVEEVRVFDGTERLPGDPDMIRYIDKQGQLQ	358
UTM62065.1	PYPISFLLSDLINRRTQRVDGQPMYGMESQVEEVRVFDGTERLPGDPDMIRYIDKQGQLQ	358

UTM62317.1	PYPISFLLSDLINRRTQRVDGQPMYGMESQVEEVRVFDGTERLPGDPDMIRYIDKQGQLQ	358
UTM62323.1	PYPISFLLSDLINRRTQRVDGQPMYGMESQVEEVRVFDGTERLPGDPDMIRYIDKQGQLQ	358
UTM62353.1	PYPISFLLSDLINRRTQRVDGQPMYGMESQVEEVRVFDGTERLPGDPDMIRYIDKQGQLQ	358
UTM62473.1	PYPISFLLSDLINRRTQRVDGQPMYGMESQVEEVRVFDGTERLPGDPDMIRYIDKQGQLQ	358
UTM62491.1	PYPISFLLSDLINRRTQRVDGQPMYGMESQVEEVRVFDGTERLPGDPDMIRYIDKQGQLQ	358
UTM62497.1	PYPISFLLSDLINRRTQRVDGQPMYGMESQVEEVRVFDGTERLPGDPDMIRYIDKQGQLQ	358
UTM62503.1	PYPISFLLSDLINRRTQRVDGQPMYGMESQVEEVRVFDGTERLPGDPDMIRYIDKQGQLQ	358
QTJ14988.1	PYPISFLLSDLINRRTQRVDGQPMYGMESQVEEVRVFDGTERLPGDPDMIRYIDKQGQLQ	358
QTJ15024.1	PYPISFLLSDLINRRTQRVDGQPMYGMESQVEEVRVFDGTERLPGDPDMIRYIDKQGQLQ	358
QTJ15030.1	PYPISFLLSDLINRRTQRVDGQPMYGMESQVEEVRVFDGTERLPGDPDMIRYIDKQGQLQ	358
QTJ15102.1	PYPISFLLSDLINRRTQRVDGQPMYGMESQVEEVRVFDGTERLPGDPDMIRYIDKQGQLQ	358
ALF06300.1	PYPISFLLSDLINRRTQRVDGQPMYGMESQVEEVRVFDGTERLPGDPDMIRYIDKQGQLQ	358
AHU87342.1	PYPISFLLSDLINRRTQRVDGQPMYGMESQVEEVRVFDGTERLPGDPDMIRYIDKQGQLQ	358
AHU87343.1	PYPISFLLSDLINRRTQRVDGQPMYGMESQVEEVRVFDGTERLPGDPDMIRYIDKQGQLQ	358
AHU87346.1	PYPISFLLSDLINRRTQRVDGQPMYGMESQVEEVRVFDGTERLPGDPDMIRYIDKQGQLQ	358
AHU87347.1	PYPISFLLSDLINRRTQRVDGQPMYGMESQVEEVRVFDGTERLPGDPDMIRYIDKQGQLQ	358
AHU87349.1	PYPISFLLSDLINRRTQRVDGQPMYGMESQVEEVRVFDGTERLPGDPDMIRYIDKQGQLQ	358
AHU87356.1	PYPISFLLSDLINRRTQRVDGQPMYGMESQVEEVRVFDGTERLPGDPDMIRYIDKQGQLQ	358
AHU87357.1	PYPISFLLSDLINRRTQRVDGQPMYGMESQVEEVRVFDGTERLPGDPDMIRYIDKQGQLQ	358
AHU87358.1	PYPISFLLSDLINRRTQRVDGQPMYGMESQVEEVRVFDGTERLPGDPDMIRYIDKQGQLQ	358
AHU87359.1	PYPISFLLSDLINRRTQRVDGQPMYGMESQVEEVRVFDGTERLPGDPDMIRYIDKQGQLQ	358
AFQ31643.1	PYPISFLLSDLINRRTQRVDGQPMYGMESQVEEVRVFDGTERLPGDPDMIRYIDKQGQLQ	358
AFQ31656.1	PYPISFLLSDLINRRTQRVDGQPMYGMESQVEEVRVFDGTERLPGDPDMIRYIDKQGQLQ	358
AFA41879.1	PYPISFLLSDLINRRTQRVDGQPMYGMESQVEEVRVFDGTERLPGDPDMIRYIDKQGQLQ	358
AFA41897.1	PYPISFLLSDLINRRTQRVDGQPMYGMESQVEEVRVFDGTERLPGDPDMIRYIDKQGQLQ	358
AFA41900.1	PYPISFLLSDLINRRTQRVDGQPMYGMESQVEEVRVFDGTERLPGDPDMIRYIDKQGQLQ	358
AEO89580.1	PYPISFLLSDLINRRTQRVDGQPMYGMESQVEEVRVFDGTERLPGDPDMIRYIDKQGQLQ	358
AEO89592.1	PYPISFLLSDLINRRTQRVDGQPMYGMESQVEEVRVFDGTERLPGDPDMIRYIDKQGQLQ	358
BAF93277.1	PYPISFLLSDLINRRTQRVDGQPMYGMESQVEEVRVFDGTERLPGDPDMIRYIDKQGQLQ	358
BAF93289.1	PYPISFLLSDLINRRTQRVDGQPMYGMESQVEEVRVFDGTERLPGDPDMIRYIDKQGQLQ	358
BAF93307.1	PYPISFLLSDLINRRTQRVDGQPMYGMESQVEEVRVFDGTERLPGDPDMIRYIDKQGQLQ	358
BAF93337.1	PYPISFLLSDLINRRTQRVDGQPMYGMESQVEEVRVFDGTERLPGDPDMIRYIDKQGQLQ	358
BAF42895.1	PYPISFLLSDLINRRTQRVDGQPMYGMESQVEEVRVFDGTERLPGDPDMIRYIDKQGQLQ	358
BAF42919.1	PYPISFLLSDLINRRTQRVDGQPMYGMESQVEEVRVFDGTERLPGDPDMIRYIDKQGQLQ	358
BAF42943.1	PYPISFLLSDLINRRTQRVDGQPMYGMESQVEEVRVFDGTERLPGDPDMIRYIDKQGQLQ	358
BAF42949.1	PYPISFLLSDLINRRTQRVDGQPMYGMESQVEEVRVFDGTERLPGDPDMIRYIDKQGQLQ	358
ABI94665.1	PYPISFLLSDLINRRTQRVDGQPMYGMESQVEEVRVFDGTERLPGDPDMIRYIDKQGQLQ	358
ABI94677.1	PYPISFLLSDLINRRTQRVDGQPMYGMESQVEEVRVFDGTERLPGDPDMIRYIDKQGQLQ	358
BAF02939.1	PYPISFLLSDLINRRTQRVDGQPMYGMESQVEEVRVFDGTERLPGDPDMIRYIDKQGQLQ	358
AAT47353.1	PYPISFLLSDLINRRTQRVDGQPMYGMESQVEEVRVFDGTERLPGDPDMIRYIDKQGQLQ	358
AAT47359.1	PYPISFLLSDLINRRTQRVDGQPMYGMESQVEEVRVFDGTERLPGDPDMIRYIDKQGQLQ	358
CAA40247.1	PYPISFLLSDLINRRTQRVDGQPMYGMESQVEEVRVFDGTERLPGDPDMIRYIDKQGQLQ	358
QTJ14982.1	PYPISFLLSDLINRRTQRVDGQPMYGMESQVEEVRVFDGTERLPGDPDMIRYIDKQGQLQ	358
QTJ15126.1	PYPISFLLSDLINRRTQRVDGQPMYGMESQVEEVRVFDGTERLPGDPDMIRYIDKQGQLQ	358
ABI94635.1	PYPISFLLSDLINRRTQRVDGQPMYGMESQVEEVRVFDGTEKLPGDPDMIRYIDKQGQLQ	358
ALF06335.1	PYPISFLLSDLINRRTQRVDGQPMYGMESQVEEVRVFDGTERLPGDPDMIRYIDKQGQLQ	358
AHU87348.1	PYPISFLLSDLINRRTQRVDGQPMYGMESQVEEVRVFDGTERLPGDPDMIRYIDKQGQLQ	358
AFQ31649.1	PYPISFLLSDLINRRTQRVDGQPMYGMESQVEEVRVFDGTERLPGDPDMIRYIDKQGQLQ	358
AFQ31654.1	PYPISFLLSDLINRRTQRVDGQPMYGMESQVEEVRVFDGTERLPGDPDMIRYIDKQGQLQ	358
BAF42907.1	PYPISFLLSDLINRRTQRVDGQPMYGMESQVEEVRVFDGTERLPGDPDMIRYIDKQGQLQ	358
WLD47824.1	PYPISFLLSDLINRRTQRVDGQPMYGMESQVEEVRVFDGTEKLPGDPDMIRYIDKQGQLQ	358
WLD47848.1	PYPISFLLSDLINRRTQRVDGQPMYGMESQVEEVRVFDGTEKLPGDPDMIRYIDKQGQLQ	358
WLD47854.1	PYPISFLLSDLINRRTQRVDGQPMYGMESQVEEVRVFDGTEKLPGDPDMIRYIDKQGQLQ	358
WLD47860.1	PYPISFLLSDLINRRTQRVDGQPMYGMESQVEEVRVFDGTEKLPGDPDMIRYIDKQGQLQ	358
WLD47878.1	PYPISFLLSDLINRRTQRVDGQPMYGMESQVEEVRVFDGTEKLPGDPDMIRYIDKQGQLQ	358
WLD47908.1	PYPISFLLSDLINRRTQRVDGQPMYGMESQVEEVRVFDGTEKLPGDPDMIRYIDKQGQLQ	358
WLD47914.1	PYPISFLLSDLINRRTQRVDGQPMYGMESQVEEVRVFDGTEKLPGDPDMIRYIDKQGQLQ	358
WLD47920.1	PYPISFLLSDLINRRTQRVDGQPMYGMESQVEEVRVFDGTEKLPGDPDMIRYIDKQGQLQ	358
WLD47926.1	PYPISFLLSDLINRRTQRVDGQPMYGMESQVEEVRVFDGTEKLPGDPDMIRYIDKQGQLQ	358
WLD47932.1	PYPISFLLSDLINRRTQRVDGQPMYGMESQVEEVRVFDGTEKLPGDPDMIRYIDKQGQLQ	358
WLD47950.1	PYPISFLLSDLINRRTQRVDGQPMYGMESQVEEVRVFDGTEKLPGDPDMIRYIDKQGQLQ	358
WLD47962.1	PYPISFLLSDLINRRTQRVDGQPMYGMESQVEEVRVFDGTEKLPGDPDMIRYIDKQGQLQ	358
WLD47974.1	PYPISFLLSDLINRRTQRVDGQPMYGMESQVEEVRVFDGTEKLPGDPDMIRYIDKQGQLQ	358
WLD47998.1	PYPISFLLSDLINRRTQRVDGQPMYGMESQVEEVRVFDGTEKLPGDPDMIRYIDKQGQLQ	358

```
WLD48004.1        PYPISFLLSDLINRRTQRVDGQPMYGMESQVEEVRVFDGTEKLPGDPDMIRYIDKQGQLQ  358
WLD48016.1        PYPISFLLSDLINRRTQRVDGQPMYGMESQVEEVRVFDGTEKLPGDPDMIRYIDKQGQLQ  358
WLD48022.1        PYPISFLLSDLINRRTQRVDGQPMYGMESQVEEVRVFDGTEKLPGDPDMIRYIDKQGQLQ  358
WLD48034.1        PYPISFLLSDLINRRTQRVDGQPMYGMESQVEEVRVFDGTEKLPGDPDMIRYIDKQGQLQ  358
UTM61909.1        PYPISFLLSDLINRRTQRVDGQPMYGMESQVEEVRVFDGTEKLPGDPDMIRYIDKQGQLQ  358
UTM61915.1        PYPISFLLSDLINRRTQRVDGQPMYGMESQVEEVRVFDGTEKLPGDPDMIRYIDKQGQLQ  358
UTM61921.1        PYPISFLLSDLINRRTQRVDGQPMYGMESQVEEVRVFDGTEKLPGDPDMIRYIDKQGQLQ  358
UTM61927.1        PYPISFLLSDLINRRTQRVDGQPMYGMESQVEEVRVFDGTEKLPGDPDMIRYIDKQGQLQ  358
UTM61933.1        PYPISFLLSDLINRRTQRVDGQPMYGMESQVEEVRVFDGTEKLPGDPDMIRYIDKQGQLQ  358
UTM61963.1        PYPISFLLSDLINRRTQRVDGQPMYGMESQVEEVRVFDGTEKLPGDPDMIRYIDKQGQLQ  358
UTM61969.1        PYPISFLLSDLINRRTQRVDGQPMYGMESQVEEVRVFDGTEKLPGDPDMIRYIDKQGQLQ  358
UTM61975.1        PYPISFLLSDLINRRTQRVDGQPMYGMESQVEEVRVFDGTEKLPGDPDMIRYIDKQGQLQ  358
UTM61981.1        PYPISFLLSDLINRRTQRVDGQPMYGMESQVEEVRVFDGTEKLPGDPDMIRYIDKQGQLQ  358
UTM62005.1        PYPISFLLSDLINRRTQRVDGQPMYGMESQVEEVRVFDGTEKLPGDPDMIRYIDKQGQLQ  358
UTM62017.1        PYPISFLLSDLINRRTQRVDGQPMYGMESQVEEVRVFDGTEKLPGDPDMIRYIDKQGQLQ  358
UTM62035.1        PYPISFLLSDLINRRTQRVDGQPMYGMESQVEEVRVFDGTEKLPGDPDMIRYIDKQGQLQ  358
UTM62041.1        PYPISFLLSDLINRRTQRVDGQPMYGMESQVEEVRVFDGTEKLPGDPDMIRYIDKQGQLQ  358
UTM62047.1        PYPISFLLSDLINRRTQRVDGQPMYGMESQVEEVRVFDGTEKLPGDPDMIRYIDKQGQLQ  358
UTM62083.1        PYPISFLLSDLINRRTQRVDGQPMYGMESQVEEVRVFDGTEKLPGDPDMIRYIDKQGQLQ  358
UTM62089.1        PYPISFLLSDLINRRTQRVDGQPMYGMESQVEEVRVFDGTEKLPGDPDMIRYIDKQGQLQ  358
UTM62095.1        PYPISFLLSDLINRRTQRVDGQPMYGMESQVEEVRVFDGTEKLPGDPDMIRYIDKQGQLQ  358
UTM62149.1        PYPISFLLSDLINRRTQRVDGQPMYGMESQVEEVRVFDGTEKLPGDPDMIRYIDKQGQLQ  358
UTM62161.1        PYPISFLLSDLINRRTQRVDGQPMYGMESQVEEVRVFDGTEKLPGDPDMIRYIDKQGQLQ  358
UTM62167.1        PYPISFLLSDLINRRTQRVDGQPMYGMESQVEEVRVFDGTEKLPGDPDMIRYIDKQGQLQ  358
UTM62173.1        PYPISFLLSDLINRRTQRVDGQPMYGMESQVEEVRVFDGTEKLPGDPDMIRYIDKQGQLQ  358
UTM62179.1        PYPISFLLSDLINRRTQRVDGQPMYGMESQVEEVRVFDGTEKLPGDPDMIRYIDKQGQLQ  358
UTM62185.1        PYPISFLLSDLINRRTQRVDGQPMYGMESQVEEVRVFDGTEKLPGDPDMIRYIDKQGQLQ  358
UTM62191.1        PYPISFLLSDLINRRTQRVDGQPMYGMESQVEEVRVFDGTEKLPGDPDMIRYIDKQGQLQ  358
UTM62197.1        PYPISFLLSDLINRRTQRVDGQPMYGMESQVEEVRVFDGTEKLPGDPDMIRYIDKQGQLQ  358
UTM62203.1        PYPISFLLSDLINRRTQRVDGQPMYGMESQVEEVRVFDGTEKLPGDPDMIRYIDKQGQLQ  358
UTM62209.1        PYPISFLLSDLINRRTQRVDGQPMYGMESQVEEVRVFDGTEKLPGDPDMIRYIDKQGQLQ  358
UTM62227.1        PYPISFLLSDLINRRTQRVDGQPMYGMESQVEEVRVFDGTEKLPGDPDMIRYIDKQGQLQ  358
UTM62239.1        PYPISFLLSDLINRRTQRVDGQPMYGMESQVEEVRVFDGTEKLPGDPDMIRYIDKQGQLQ  358
UTM62245.1        PYPISFLLSDLINRRTQRVDGQPMYGMESQVEEVRVFDGTEKLPGDPDMIRYIDKQGQLQ  358
UTM62251.1        PYPISFLLSDLINRRTQRVDGQPMYGMESQVEEVRVFDGTEKLPGDPDMIRYIDKQGQLQ  358
UTM62257.1        PYPISFLLSDLINRRTQRVDGQPMYGMESQVEEVRVFDGTEKLPGDPDMIRYIDKQGQLQ  358
UTM62263.1        PYPISFLLSDLINRRTQRVDGQPMYGMESQVEEVRVFDGTEKLPGDPDMIRYIDKQGQLQ  358
UTM62269.1        PYPISFLLSDLINRRTQRVDGQPMYGMESQVEEVRVFDGTEKLPGDPDMIRYIDKQGQLQ  358
UTM62287.1        PYPISFLLSDLINRRTQRVDGQPMYGMESQVEEVRVFDGTEKLPGDPDMIRYIDKQGQLQ  358
UTM62293.1        PYPISFLLSDLINRRTQRVDGQPMYGMESQVEEVRVFDGTEKLPGDPDMIRYIDKQGQLQ  358
UTM62299.1        PYPISFLLSDLINRRTQRVDGQPMYGMESQVEEVRVFDGTEKLPGDPDMIRYIDKQGQLQ  358
UTM62305.1        PYPISFLLSDLINRRTQRVDGQPMYGMESQVEEVRVFDGTEKLPGDPDMIRYIDKQGQLQ  358
UTM62311.1        PYPISFLLSDLINRRTQRVDGQPMYGMESQVEEVRVFDGTEKLPGDPDMIRYIDKQGQLQ  358
UTM62359.1        PYPISFLLSDLINRRTQRVDGQPMYGMESQVEEVRVFDGTEKLPGDPDMIRYIDKQGQLQ  358
UTM62365.1        PYPISFLLSDLINRRTQRVDGQPMYGMESQVEEVRVFDGTEKLPGDPDMIRYIDKQGQLQ  358
UTM62371.1        PYPISFLLSDLINRRTQRVDGQPMYGMESQVEEVRVFDGTEKLPGDPDMIRYIDKQGQLQ  358
UTM62377.1        PYPISFLLSDLINRRTQRVDGQPMYGMESQVEEVRVFDGTEKLPGDPDMIRYIDKQGQLQ  358
UTM62455.1        PYPISFLLSDLINRRTQRVDGQPMYGMESQVEEVRVFDGTEKLPGDPDMIRYIDKQGQLQ  358
UTM62461.1        PYPISFLLSDLINRRTQRVDGQPMYGMESQVEEVRVFDGTEKLPGDPDMIRYIDKQGQLQ  358
UTM62485.1        PYPISFLLSDLINRRTQRVDGQPMYGMESQVEEVRVFDGTEKLPGDPDMIRYIDKQGQLQ  358
UTM62509.1        PYPISFLLSDLINRRTQRVDGQPMYGMESQVEEVRVFDGTEKLPGDPDMIRYIDKQGQLQ  358
UTM62515.1        PYPISFLLSDLINRRTQRVDGQPMYGMESQVEEVRVFDGTEKLPGDPDMIRYIDKQGQLQ  358
UTM62521.1        PYPISFLLSDLINRRTQRVDGQPMYGMESQVEEVRVFDGTEKLPGDPDMIRYIDKQGQLQ  358
UTM62527.1        PYPISFLLSDLINRRTQRVDGQPMYGMESQVEEVRVFDGTEKLPGDPDMIRYIDKQGQLQ  358
UTM62539.1        PYPISFLLSDLINRRTQRVDGQPMYGMESQVEEVRVFDGTEKLPGDPDMIRYIDKQGQLQ  358
UTM62557.1        PYPISFLLSDLINRRTQRVDGQPMYGMESQVEEVRVFDGTEKLPGDPDMIRYIDKQGQLQ  358
UTM62563.1        PYPISFLLSDLINRRTQRVDGQPMYGMESQVEEVRVFDGTEKLPGDPDMIRYIDKQGQLQ  358
UTM62569.1        PYPISFLLSDLINRRTQRVDGQPMYGMESQVEEVRVFDGTEKLPGDPDMIRYIDKQGQLQ  358
UTM62575.1        PYPISFLLSDLINRRTQRVDGQPMYGMESQVEEVRVFDGTEKLPGDPDMIRYIDKQGQLQ  358
UTM62581.1        PYPISFLLSDLINRRTQRVDGQPMYGMESQVEEVRVFDGTEKLPGDPDMIRYIDKQGQLQ  358
UTM62605.1        PYPISFLLSDLINRRTQRVDGQPMYGMESQVEEVRVFDGTEKLPGDPDMIRYIDKQGQLQ  358
UTM62617.1        PYPISFLLSDLINRRTQRVDGQPMYGMESQVEEVRVFDGTEKLPGDPDMIRYIDKQGQLQ  358
UTM62623.1        PYPISFLLSDLINRRTQRVDGQPMYGMESQVEEVRVFDGTEKLPGDPDMIRYIDKQGQLQ  358
UTM62629.1        PYPISFLLSDLINRRTQRVDGQPMYGMESQVEEVRVFDGTEKLPGDPDMIRYIDKQGQLQ  358
```

UTM62635.1	PYPISFLLSDLINRRTQRVDGQPMYGMESQVEEVRVFDGTEKLPGDPDMIRYIDKQGQLQ	358
QTJ15048.1	PYPISFLLSDLINRRTQRVDGQPMYGMESQVEEVRVFDGTEKLPGDPDMIRYIDKQGQLQ	358
QTJ15144.1	PYPISFLLSDLINRRTQRVDGQPMYGMESQVEEVRVFDGTEKLPGDPDMIRYIDKQGQLQ	358
AHU87360.1	PYPISFLLSDLINRRTQRVDGQPMYGMESQVEEVRVFDGTEKLPGDPDMIRYIDKQGQLQ	358
AHU87361.1	PYPISFLLSDLINRRTQRVDGQPMYGMESQVEEVRVFDGTEKLPGDPDMIRYIDKQGQLQ	358
AHU87366.1	PYPISFLLSDLINRRTQRVDGQPMYGMESQVEEVRVFDGTEKLPGDPDMIRYIDKQGQLQ	358
AHU87367.1	PYPISFLLSDLINRRTQRVDGQPMYGMESQVEEVRVFDGTEKLPGDPDMIRYIDKQGQLQ	358
AFQ31652.1	PYPISFLLSDLINRRTQRVDGQPMYGMESQVEEVRVFDGTEKLPGDPDMIRYIDKQGQLQ	358
AFA41888.1	PYPISFLLSDLINRRTQRVDGQPMYGMESQVEEVRVFDGTEKLPGDPDMIRYIDKQGQLQ	358
AFA41893.1	PYPISFLLSDLINRRTQRVDGQPMYGMESQVEEVRVFDGTEKLPGDPDMIRYIDKQGQLQ	358
AFA41894.1	PYPISFLLSDLINRRTQRVDGQPMYGMESQVEEVRVFDGTEKLPGDPDMIRYIDKQGQLQ	358
AFA41899.1	PYPISFLLSDLINRRTQRVDGQPMYGMESQVEEVRVFDGTEKLPGDPDMIRYIDKQGQLQ	358
AFA41904.1	PYPISFLLSDLINRRTQRVDGQPMYGMESQVEEVRVFDGTEKLPGDPDMIRYIDKQGQLQ	358
AFA41905.1	PYPISFLLSDLINRRTQRVDGQPMYGMESQVEEVRVFDGTEKLPGDPDMIRYIDKQGQLQ	358
AFA41908.1	PYPISFLLSDLINRRTQRVDGQPMYGMESQVEEVRVFDGTEKLPGDPDMIRYIDKQGQLQ	358
AFA41911.1	PYPISFLLSDLINRRTQRVDGQPMYGMESQVEEVRVFDGTEKLPGDPDMIRYIDKQGQLQ	358
AFA41912.1	PYPISFLLSDLINRRTQRVDGQPMYGMESQVEEVRVFDGTEKLPGDPDMIRYIDKQGQLQ	358
AFA41913.1	PYPISFLLSDLINRRTQRVDGQPMYGMESQVEEVRVFDGTEKLPGDPDMIRYIDKQGQLQ	358
AFA41915.1	PYPISFLLSDLINRRTQRVDGQPMYGMESQVEEVRVFDGTEKLPGDPDMIRYIDKQGQLQ	358
AFA41916.1	PYPISFLLSDLINRRTQRVDGQPMYGMESQVEEVRVFDGTEKLPGDPDMIRYIDKQGQLQ	358
AFA41919.1	PYPISFLLSDLINRRTQRVDGQPMYGMESQVEEVRVFDGTEKLPGDPDMIRYIDKQGQLQ	358
AFA41921.1	PYPISFLLSDLINRRTQRVDGQPMYGMESQVEEVRVFDGTEKLPGDPDMIRYIDKQGQLQ	358
AFA41922.1	PYPISFLLSDLINRRTQRVDGQPMYGMESQVEEVRVFDGTEKLPGDPDMIRYIDKQGQLQ	358
AFA41923.1	PYPISFLLSDLINRRTQRVDGQPMYGMESQVEEVRVFDGTEKLPGDPDMIRYIDKQGQLQ	358
AFA41924.1	PYPISFLLSDLINRRTQRVDGQPMYGMESQVEEVRVFDGTEKLPGDPDMIRYIDKQGQLQ	358
AFA41925.1	PYPISFLLSDLINRRTQRVDGQPMYGMESQVEEVRVFDGTEKLPGDPDMIRYIDKQGQLQ	358
CBX88272.1	PYPISFLLSDLINRRTQRVDGQPMYGMESQVEEVRVFDGTEKLPGDPDMIRYIDKQGQLQ	358
CBX88278.1	PYPISFLLSDLINRRTQRVDGQPMYGMESQVEEVRVFDGTEKLPGDPDMIRYIDKQGQLQ	358
CBX88284.1	PYPISFLLSDLINRRTQRVDGQPMYGMESQVEEVRVFDGTEKLPGDPDMIRYIDKQGQLQ	358
CBX88290.1	PYPISFLLSDLINRRTQRVDGQPMYGMESQVEEVRVFDGTEKLPGDPDMIRYIDKQGQLQ	358
CBX88296.1	PYPISFLLSDLINRRTQRVDGQPMYGMESQVEEVRVFDGTEKLPGDPDMIRYIDKQGQLQ	358
CBX88308.1	PYPISFLLSDLINRRTQRVDGQPMYGMESQVEEVRVFDGTEKLPGDPDMIRYIDKQGQLQ	358
CBX88332.1	PYPISFLLSDLINRRTQRVDGQPMYGMESQVEEVRVFDGTEKLPGDPDMIRYIDKQGQLQ	358
CBX88356.1	PYPISFLLSDLINRRTQRVDGQPMYGMESQVEEVRVFDGTEKLPGDPDMIRYIDKQGQLQ	358
CBX88362.1	PYPISFLLSDLINRRTQRVDGQPMYGMESQVEEVRVFDGTEKLPGDPDMIRYIDKQGQLQ	358
BAF93313.1	PYPISFLLSDLINRRTQRVDGQPMYGMESQVEEVRVFDGTEKLPGDPDMIRYIDKQGQLQ	358
BAF42955.1	PYPISFLLSDLINRRTQRVDGQPMYGMESQVEEVRVFDGTEKLPGDPDMIRYIDKQGQLQ	358
BAF43021.1	PYPISFLLSDLINRRTQRVDGQPMYGMESQVEEVRVFDGTEKLPGDPDMIRYIDKQGQLQ	358
ABI94611.1	PYPISFLLSDLINRRTQRVDGQPMYGMESQVEEVRVFDGTEKLPGDPDMIRYIDKQGQLQ	358
ABI94641.1	PYPISFLLSDLINRRTQRVDGQPMYGMESQVEEVRVFDGTEKLPGDPDMIRYIDKQGQLQ	358
ABI94647.1	PYPISFLLSDLINRRTQRVDGQPMYGMESQVEEVRVFDGTEKLPGDPDMIRYIDKQGQLQ	358
WLD47896.1	PYPISFLLSDLINRRTQRVDGQPMYGMESQVEEVRVFDGTEKLPGDPDMIRYIDKQGQLQ	358
WLD48028.1	PYPISFLLSDLINRRTQRVDGQPMYGMESQVEEVRVFDGTEKLPGDPDMIRYIDKQGQLQ	358
BAG84440.1	PYPISFLLSDLINRRTQRVDGQPMYGMESQVEEVRVFDGTEKLPGDPDMIRYIDKQGQLQ	358
BAF93283.1	PYPISFLLSDLINRRTQRVDGQPMYGMESQVEEVRVFDGTEKLPGDPDMIRYIDKQGQLQ	358
BAF93361.1	PYPISFLLSDLINRRTQRVDGQPMYGMESQVEEVRVFDGTEKLPGDPDMIRYIDKQGQLQ	358
WLD48040.1	PYPISFLLSDLINRRTQRVDGQPMYGMESQVEEVRVFDGTEKLPGDPDMIRYIDKQGQLQ	358
UTM62611.1	PYPISFLLSDLINRRTQRVDGQPMYGMESQVEEVRVFDGTEKLPGDPDMIRYIDKQGQLQ	358
AFQ31645.1	PYPISFLLSDLINRRTQRVDGQPMYGMESQVEEVRVFDGTEKLPGDPDMIRYIDKQGQLQ	358
AFA41909.1	PYPISFLLSDLINRRTQRVDGQPMYGMESQVEEVRVFDGTEKLPGDPDMIRYIDKQGQLQ	358
ABI94713.1	PYPISFLLSDLINRRTQRVDGQPMYGMESQVEEVRVFDGTEKLPGDPDMIRYIDKQGQLQ	358
ABD04662.1	PYPISFLLSDLINRRTQRVDGQPMYGMESQVEEVRVFDGTEKLPGDPDMIRYIDKQGQLQ	358
QTJ15042.1	PYPISFLLSDLINRRTQRVDGQPMYGMESQVEEVRVFDGTETLPGDPDMIRYIDKQGQLQ	358
AGX89705.1	PYPISFLLSDLINRRTQKVDGQPMYGMESQVEEVRVFDGTEQLPGDPDMIRYIDRQGQLQ	358
AGX89710.1	PYPISFLLSDLINRRTQKVDGQPMYGMESQVEEVRVFDGTEQLPGDPDMIRYIDRQGQLQ	358
AGX89717.1	PYPISFLLSDLINRRTQKVDGQPMYGMESQVEEVRVFDGTEQLPGDPDMIRYIDRQGQLQ	358
AFQ31655.1	PYPISFLLSDLINRRTQKVDGQPMYGMESQVEEVRVFDGTEQLPGDPDMIRYIDRQGQLQ	358
AAA46882.1	PYPISFLLSDLINRRTQKVDGQPMYGMESQVEEVRVFDGTEQLPGDPDMIRYIDRQGQLQ	358
P14996.1	PYPISFLLSDLINRRTQKVDGQPMYGMESQVEEVRVFDGTEQLPGDPDMIRYIDRQGQLQ	358
AEO89615.1	PYPISFLLSDLINRRTQKVDGQPMYGMESQVEEVRVFDGTEQLPGDPDMIRYIDRQGQLQ	358
BCV68929.1	PYPISFLLSDLINRRTQKVDGQPMYGMESQVEEVRVFDGTEQLPGDPDMIRYIDRQGQLQ	358
QTJ14952.1	PYPISFLLSDLINRRTQKVDGQPMYGMESQVEEVRVFDGTEQLPGDPDMIRYIDRQGQLQ	358
BAG75157.1	PYPISFLLSDLINRRTQKVDGQPMYGMESQVEEVRVFDGTEQLPGDPDMIRYIDRQGQLQ	358
BAG75211.1	PYPISFLLSDLINRRTQKVDGQPMYGMESQVEEVRVFDGTEQLPGDPDMIRYIDRQGQLQ	358

BAF03017.1	PYPISFLLSDLINRRTQKVDGQPMYGMESQVEEVRVFDGTEQLPGDPDMIRYIDRQGQLQ	358
BAF42925.1	PYPISFLLSDLINRRTQKVDGQPMYGMESQVEEVRVFDGTEQLPGDPDMIRYIDRQGQLQ	358
WLD47836.1	PYPISFLLSDLINRRTQKVDGQPMYGMESQVEEVRVFDGTEQLPGDPDMIRYIDRQGQLQ	358
UEL36354.1	PYPISFLLSDLINRRTQKVDGQPMYGMESQVEEVRVFDGTEQLPGDPDMIRYIDRQGQLQ	358
BAF42901.1	PYPISFLLSDLINRRTQKVDGQPMYGMESQVEEVRVFDGTEQLPGDPDMIRYIDRQGQLQ	358
ABP87973.1	PYPISFLLSDLINRRTQKVDGQPMYGMESQVEEVRVFDGTEQLPGDPDMIRYIDRQGQLQ	358
AFA41878.1	PYPISFLLSDLINRRTQKVDGQPMYGMESQVEEVRVFDGTEQLPGDPDMIRYIDRQGQLQ	358
QHI34655.1	PYPISFLLSDLINRRTQKVDGQPMYGMESQVEEVRVFDGTEQLPGDPDMIRYIDRQGQLQ	358
UTM61945.1	PYPISFLLSDLINRRTQKVDGQPMYGMESQVEEVRVFDGTEQLPGDPDMIRYIDRQGQLQ	358
UTM61987.1	PYPISFLLSDLINRRTQKVDGQPMYGMESQVEEVRVFDGTEQLPGDPDMIRYIDRQGQLQ	358
UTM62233.1	PYPISFLLSDLINRRTQKVDGQPMYGMESQVEEVRVFDGTEQLPGDPDMIRYIDRQGQLQ	358
UTM62341.1	PYPISFLLSDLINRRTQKVDGQPMYGMESQVEEVRVFDGTEQLPGDPDMIRYIDRQGQLQ	358
UTM62347.1	PYPISFLLSDLINRRTQKVDGQPMYGMESQVEEVRVFDGTEQLPGDPDMIRYIDRQGQLQ	358
AHU87354.1	PYPISFLLSDLINRRTQKVDGQPMYGMESQVEEVRVFDGTEQLPGDPDMIRYIDRQGQLQ	358
AHU87355.1	PYPISFLLSDLINRRTQKVDGQPMYGMESQVEEVRVFDGTEQLPGDPDMIRYIDRQGQLQ	358
AHU87364.1	PYPISFLLSDLINRRTQKVDGQPMYGMESQVEEVRVFDGTEQLPGDPDMIRYIDRQGQLQ	358
AHU87365.1	PYPISFLLSDLINRRTQKVDGQPMYGMESQVEEVRVFDGTEQLPGDPDMIRYIDRQGQLQ	358
AFQ31638.1	PYPISFLLSDLINRRTQKVDGQPMYGMESQVEEVRVFDGTEQLPGDPDMIRYIDRQGQLQ	358
AFA41874.1	PYPISFLLSDLINRRTQKVDGQPMYGMESQVEEVRVFDGTEQLPGDPDMIRYIDRQGQLQ	358
AFA41875.1	PYPISFLLSDLINRRTQKVDGQPMYGMESQVEEVRVFDGTEQLPGDPDMIRYIDRQGQLQ	358
AFA41896.1	PYPISFLLSDLINRRTQKVDGQPMYGMESQVEEVRVFDGTEQLPGDPDMIRYIDRQGQLQ	358
BAF93367.1	PYPISFLLSDLINRRTQKVDGQPMYGMESQVEEVRVFDGTEQLPGDPDMIRYIDRQGQLQ	358
CAA79596.1	PYPISFLLSDLINRRTQKVDGQPMYGMESQVEEVRVFDGTEQLPGDPDMIRYIDRQGQLQ	358
AFA41883.1	PYPISFFISDLINRRTQRVDGQPMYGMESQVEEVRVFDGTEQLPGMPDLIRYIDRQGQLQ	358
WLD47812.1	PYPISFLLSDLINRRTQRVDGQPMYGMESQVEEVRVFDGTEQLPGDPDMIRYIDRQGQLQ	358
BAG75277.1	PYPISFLLSDLINRRTQRVDGQPMYGMESQVEEVRVFDGTEQLPGDPDMIRYIDRQGQLQ	358
BAF75138.1	PYPISFLLSDLINRRTQRVDGQPMYGMESQVEEVRVFDGTEQLPGDPDMIRYIDRQGQLQ	358
BAF75102.1	PYPISFLLSDLINRRTQRVDGQPMYGMESQVEEVRVFDGTEQLPGDPDMIRYIDRQGQLQ	358
AYO46643.1	PYPISFLLSDLINRRTQRVDGQPMYGMESQVEEVRVFDGTEQLPGDPDMIRYIDRQGQLQ	358
QUW04844.1	PYPISFLLSDLINRRTQRVDGQPMYGMESQVEEVRVFDGTEQLPGDPDMIRYIDRQGQLQ	358
AYO46683.1	PYPISFLLSDLINRRTQRVDGQPMYGMESQVEEVRVFDGTEQLPGDPDMIRYIDRQGQLQ	358
AYO46645.1	PYPISFLLSDLINRRTQRVDGQPMYGMESQVEEVRVFDGTEQLPGDPDMIRYIDRQGQLQ	358
BAF75180.1	PYPISFLLSDLINRRTQRVDGQPMYGMESQVEEVRVFDGTEQLPGDPDMIRYIDRQGQLQ	358
BAF75204.1	PYPISFLLSDLINRRTQRVDGQPMYGMESQVEEVRVFDGTEQLPGDPDMIRYIDRQGQLQ	358
AYO46631.1	PYPISFLLSDLINRRTQRVDGQPMYGMESQVEEVRVFDGTEQLPGDPDMIRYIDRQGQLQ	358
AYO46608.1	PYPISFLLSDLINRRTQRVDGQPMYGMESQVEEVRVFDGTEQLPGDPDMIRYIDRQGQLQ	358
AYO46611.1	PYPISFLLSDLINRRTQRVDGQPMYGMESQVEEVRVFDGTEQLPGDPDMIRYIDRQGQLQ	358
AYO46615.1	PYPISFLLSDLINRRTQRVDGQPMYGMESQVEEVRVFDGTEQLPGDPDMIRYIDRQGQLQ	358
AYO46639.1	PYPISFLLSDLINRRTQRVDGQPMYGMESQVEEVRVFDGTEQLPGDPDMIRYIDRQGQLQ	358
AYO46649.1	PYPISFLLSDLINRRTQRVDGQPMYGMESQVEEVRVFDGTEQLPGDPDMIRYIDRQGQLQ	358
AYO46655.1	PYPISFLLSDLINRRTQRVDGQPMYGMESQVEEVRVFDGTEQLPGDPDMIRYIDRQGQLQ	358
AYO46664.1	PYPISFLLSDLINRRTQRVDGQPMYGMESQVEEVRVFDGTEQLPGDPDMIRYIDRQGQLQ	358
AYO46669.1	PYPISFLLSDLINRRTQRVDGQPMYGMESQVEEVRVFDGTEQLPGDPDMIRYIDRQGQLQ	358
AYO46685.1	PYPISFLLSDLINRRTQRVDGQPMYGMESQVEEVRVFDGTEQLPGDPDMIRYIDRQGQLQ	358
AYO46686.1	PYPISFLLSDLINRRTQRVDGQPMYGMESQVEEVRVFDGTEQLPGDPDMIRYIDRQGQLQ	358
AYO46690.1	PYPISFLLSDLINRRTQRVDGQPMYGMESQVEEVRVFDGTEQLPGDPDMIRYIDRQGQLQ	358
AYO46627.1	PYPISFLLSDLINRRTQRVDGQPMYGMESQVEEVRVFDGTEQLPGDPDMIRYIDRQGQLQ	358
AYO46630.1	PYPISFLLSDLINRRTQRVDGQPMYGMESQVEEVRVFDGTEQLPGDPDMIRYIDRQGQLQ	358
AYO46656.1	PYPISFLLSDLINRRTQRVDGQPMYGMESQVEEVRVFDGTEQLPGDPDMIRYIDRQGQLQ	358
BAG75217.1	PYPISFLLSDLINRRTQRVDGQPMYGMESQVEEVRVFDGTEKLPGDPDMIRYIDRQGQLQ	358
BAG75229.1	PYPISFLLSDLINRRTQRVDGQPMYGMESQVEEVRVFDGTEKLPGDPDMIRYIDRQGQLQ	358
BAG75235.1	PYPISFLLSDLINRRTQRVDGQPMYGMESQVEEVRVFDGTEKLPGDPDMIRYIDRQGQLQ	358
BAG75259.1	PYPISFLLSDLINRRTQRVDGQPMYGMESQVEEVRVFDGTEKLPGDPDMIRYIDRQGQLQ	358
BAF75150.1	PYPISFLLSDLINRRTQRVDGQPMYGMESQVEEVRVFDGTEKLPGDPDMIRYIDRQGQLQ	358
BAF75156.1	PYPISFLLSDLINRRTQRVDGQPMYGMESQVEEVRVFDGTEKLPGDPDMIRYIDRQGQLQ	358
BAF75174.1	PYPISFLLSDLINRRTQRVDGQPMYGMESQVEEVRVFDGTEKLPGDPDMIRYIDRQGQLQ	358
BAE96083.1	PYPISFLLSDLINRRTQRVDGQPMYGMESQVEEVRVFDGTEKLPGDPDMIRYIDRQGQLQ	358
BAE53654.1	PYPISFLLSDLINRRTQRVDGQPMYGMESQVEEVRVFDGTEKLPGDPDMIRYIDRQGQLQ	358
BAF03035.1	PYPISFLLSDLINRRTQRVDGQPMYGMESQVEEVRVFDGTEKLPGDPDMIRYIDRQGQLQ	358
QHI34656.1	PYPISFLLSDLINRRTQRVDGQPMYGMESQVEEVRVFDGTEQLPGDPDMIRYIDRQGQLQ	358
ARE72396.1	PYPISFLLSDLINRRTQRVDGQPMYGMESQVEEVRVFDCTEQLPGDPDMIRYIDRQGQLQ	358
AFA41885.1	PYPISFLLSDLINRRTQRVDGQPMYGMESQVEEVRVFDGTEQLPGDPDMIRYIDRQGQLQ	358
BAG75271.1	PYPISFLLSDLINRRTQRVDGQPMYGMESQVEEVRVFDGTEQLPGDPDMIRYIDRQGQLQ	358
BAF75090.1	PYPISFLLSDLINRRTQRVDGQPMYGMESQVEEVRVFDGTEQLPGDPDMIRYIDRQGQLQ	358

BAF75306.1	PYPISFLLSDLINRRTQRVDGQPMYGMESQVEEVRVFDGTEQLPGDPDMIRYIDRQGQLQ	358
BAF75342.1	PYPISFLLSDLINRRTQRVDGQPMYGMESQVEEVRVFDGTEQLPGDPDMIRYIDRQGQLQ	358
BAF03029.1	PYPISFLLSDLINRRTQRVDGQPMYGMESQVEEVRVFDGTEQLPGDPDMIRYIDRQGQLQ	358
BAG75205.1	PYPISFLLSDLINRRTQRVDGQPMYGMESQVEEVRVFDGTEQLPGDPDMIRYIDRQGQLQ	358
BAG75223.1	PYPISFLLSDLINRRTQRVDGQPMYGMESQVEEVRVFDGTEQLPGDPDMIRYIDRQGQLQ	358
BAF75144.1	PYPISFLLSDLINRRTQRVDGQPMYGMESQVEEVRVFDGTEQLPGDPDMIRYIDRQGQLQ	358
BAF75162.1	PYPISFLLSDLINRRTQRVDGQPMYGMESQVEEVRVFDGTEQLPGDPDMIRYIDRQGQLQ	358
BAF75168.1	PYPISFLLSDLINRRTQRVDGQPMYGMESQVEEVRVFDGTEQLPGDPDMIRYIDRQGQLQ	358
BAF75240.1	PYPISFLLSDLINRRTQRVDGQPMYGMESQVEEVRVFDGTEQLPGDPDMIRYIDRQGQLQ	358
BAF03023.1	PYPISFLLSDLINRRTQRVDGQPMYGMESQVEEVRVFDGTEQLPGDPDMIRYIDRQGQLQ	358
BAE96077.1	PYPISFLLSDLINRRTQRVDGQPMYGMESQVEEVRVFDGTEQLPGDPDMIRYIDRQGQLQ	358
BAG84476.1	PYPISFLLSDLINRRTQRVDGQPMYGMESQVEEVRVFDGTEQLPGDPDMIRYIDRQGQLQ	358
AYO46661.1	PYPISFLLSDLINRRTQRVDGQPMYGMESQVEEVRVFDGTEQLPGDPDMIRYIDRQGQLQ	358
AYO46634.1	PYPISFLLSDLINRRTQRVDGQPMYGMESQVEEVRVFDGTEQLPGDPDMIRYIDRQGQLQ	358
AYO46657.1	PYPISFLLSDLINRRTQRVDGQPMYGMESQVEEVRVFDGTEQLPGDPDMIRYIDRQGQLQ	358
BAG75379.1	PYPISFLLSDLINRRTQRVDGQPMYGMESQVEEVRVFDGTEQLPGDPDMIRYIDRQGQLQ	358
BAG75409.1	PYPISFLLSDLINRRTQRVDGQPMYGMESQVEEVRVFDGTEQLPGDPDMIRYIDRQGQLQ	358
BAG75415.1	PYPISFLLSDLINRRTQRVDGQPMYGMESQVEEVRVFDGTEQLPGDPDMIRYIDRQGQLQ	358
BAF75096.1	PYPISFLLSDLINRRTQRVDGQPMYGMESQVEEVRVFDGTEQLPGDPDMIRYIDRQGQLQ	358
BAF75192.1	PYPISFLLSDLINRRTQRVDGQPMYGMESQVEEVRVFDGTEQLPGDPDMIRYIDRQGQLQ	358
BAF75198.1	PYPISFLLSDLINRRTQRVDGQPMYGMESQVEEVRVFDGTEQLPGDPDMIRYIDRQGQLQ	358
BAF75210.1	PYPISFLLSDLINRRTQRVDGQPMYGMESQVEEVRVFDGTEQLPGDPDMIRYIDRQGQLQ	358
BAF75234.1	PYPISFLLSDLINRRTQRVDGQPMYGMESQVEEVRVFDGTEQLPGDPDMIRYIDRQGQLQ	358
BAF75264.1	PYPISFLLSDLINRRTQRVDGQPMYGMESQVEEVRVFDGTEQLPGDPDMIRYIDRQGQLQ	358
BAF75270.1	PYPISFLLSDLINRRTQRVDGQPMYGMESQVEEVRVFDGTEQLPGDPDMIRYIDRQGQLQ	358
BAF75282.1	PYPISFLLSDLINRRTQRVDGQPMYGMESQVEEVRVFDGTEQLPGDPDMIRYIDRQGQLQ	358
BAF75300.1	PYPISFLLSDLINRRTQRVDGQPMYGMESQVEEVRVFDGTEQLPGDPDMIRYIDRQGQLQ	358
BAF75312.1	PYPISFLLSDLINRRTQRVDGQPMYGMESQVEEVRVFDGTEQLPGDPDMIRYIDRQGQLQ	358
BAF75318.1	PYPISFLLSDLINRRTQRVDGQPMYGMESQVEEVRVFDGTEQLPGDPDMIRYIDRQGQLQ	358
BAF75324.1	PYPISFLLSDLINRRTQRVDGQPMYGMESQVEEVRVFDGTEQLPGDPDMIRYIDRQGQLQ	358
BAF75336.1	PYPISFLLSDLINRRTQRVDGQPMYGMESQVEEVRVFDGTEQLPGDPDMIRYIDRQGQLQ	358
AFA41889.1	PYPISFLLSDLINRRTQRVDGQPMYGMESQVEEVRVFDGTEQLPGDPDMIRYIDRQGQLQ	358
UEL36357.1	PYPISFLLSDLINRRTQRVDGQPMYGMESQVEEVRVFDGTEQLPGDPDMIRYIDRQGQLQ	358
BCV68920.1	PYPISFLLSDLINRRTQRVDGQPMYGMESQVEEVRVFDGTEKLPGDPDMIRYIDRQGQLQ	358
WLD47992.1	PYPISFLLSDLINRRTQRVDGQPMYGMESQVEEVRVFDGTEQLPGDPDMIRYIDRQGQLQ	358
UTM62413.1	PYPISFLLSDLINRRTQRVDGQPMYGMESQVEEVRVFDGTEQLPGDPDMIRYIDRQGQLQ	358
AHU87344.1	PYPISFLLSDLINRRTQRVDGQPMYGMESQVEEVRVFDGTEQLPGDPDMIRYIDRQGQLQ	358
AHU87345.1	PYPISFLLSDLINRRTQRVDGQPMYGMESQVEEVRVFDGTEQLPGDPDMIRYIDRQGQLQ	358
QTJ14946.1	PYPISFLLSDLINRRTQRVDGQPMYGMESQVEEVRVFDGTEQLPGDPDMIRYIDRQGQLQ	358
AYO46666.1	PYPISFLLSDLINRRTQRVDGQPMYGMESQVEEVRVFDGTEQLPGDPDMIRYIDRQGQLQ	358
WLD47788.1	PYPISFLLSDLINRRTQRVDGQPMYGMESQVEEVRVFDGTEQLPGDPDMIRYIDRQGQLQ	358
WLD47794.1	PYPISFLLSDLINRRTQRVDGQPMYGMESQVEEVRVFDGTEQLPGDPDMIRYIDRQGQLQ	358
WLD47800.1	PYPISFLLSDLINRRTQRVDGQPMYGMESQVEEVRVFDGTEQLPGDPDMIRYIDRQGQLQ	358
WLD47806.1	PYPISFLLSDLINRRTQRVDGQPMYGMESQVEEVRVFDGTEQLPGDPDMIRYIDRQGQLQ	358
WLD47872.1	PYPISFLLSDLINRRTQRVDGQPMYGMESQVEEVRVFDGTEQLPGDPDMIRYIDRQGQLQ	358
WLD47986.1	PYPISFLLSDLINRRTQRVDGQPMYGMESQVEEVRVFDGTEQLPGDPDMIRYIDRQGQLQ	358
WLD48010.1	PYPISFLLSDLINRRTQRVDGQPMYGMESQVEEVRVFDGTEQLPGDPDMIRYIDRQGQLQ	358
UTM61951.1	PYPISFLLSDLINRRTQRVDGQPMYGMESQVEEVRVFDGTEQLPGDPDMIRYIDRQGQLQ	358
UTM61957.1	PYPISFLLSDLINRRTQRVDGQPMYGMESQVEEVRVFDGTEQLPGDPDMIRYIDRQGQLQ	358
UTM61993.1	PYPISFLLSDLINRRTQRVDGQPMYGMESQVEEVRVFDGTEQLPGDPDMIRYIDRQGQLQ	358
UTM61999.1	PYPISFLLSDLINRRTQRVDGQPMYGMESQVEEVRVFDGTEQLPGDPDMIRYIDRQGQLQ	358
UTM62011.1	PYPISFLLSDLINRRTQRVDGQPMYGMESQVEEVRVFDGTEQLPGDPDMIRYIDRQGQLQ	358
UTM62071.1	PYPISFLLSDLINRRTQRVDGQPMYGMESQVEEVRVFDGTEQLPGDPDMIRYIDRQGQLQ	358
UTM62077.1	PYPISFLLSDLINRRTQRVDGQPMYGMESQVEEVRVFDGTEQLPGDPDMIRYIDRQGQLQ	358
UTM62119.1	PYPISFLLSDLINRRTQRVDGQPMYGMESQVEEVRVFDGTEQLPGDPDMIRYIDRQGQLQ	358
UTM62125.1	PYPISFLLSDLINRRTQRVDGQPMYGMESQVEEVRVFDGTEQLPGDPDMIRYIDRQGQLQ	358
UTM62131.1	PYPISFLLSDLINRRTQRVDGQPMYGMESQVEEVRVFDGTEQLPGDPDMIRYIDRQGQLQ	358
UTM62137.1	PYPISFLLSDLINRRTQRVDGQPMYGMESQVEEVRVFDGTEQLPGDPDMIRYIDRQGQLQ	358
UTM62143.1	PYPISFLLSDLINRRTQRVDGQPMYGMESQVEEVRVFDGTEQLPGDPDMIRYIDRQGQLQ	358
UTM62401.1	PYPISFLLSDLINRRTQRVDGQPMYGMESQVEEVRVFDGTEQLPGDPDMIRYIDRQGQLQ	358
UTM62407.1	PYPISFLLSDLINRRTQRVDGQPMYGMESQVEEVRVFDGTEQLPGDPDMIRYIDRQGQLQ	358
UTM62419.1	PYPISFLLSDLINRRTQRVDGQPMYGMESQVEEVRVFDGTEQLPGDPDMIRYIDRQGQLQ	358
UTM62425.1	PYPISFLLSDLINRRTQRVDGQPMYGMESQVEEVRVFDGTEQLPGDPDMIRYIDRQGQLQ	358
UTM62431.1	PYPISFLLSDLINRRTQRVDGQPMYGMESQVEEVRVFDGTEQLPGDPDMIRYIDRQGQLQ	358

```
UTM62437.1        PYPISFLLSDLINRRTQRVDGQPMYGMESQVEEVRVFDGTEQLPGDPDMIRYIDRQGQLQ  358
UTM62641.1        PYPISFLLSDLINRRTQRVDGQPMYGMESQVEEVRVFDGTEQLPGDPDMIRYIDRQGQLQ  358
UTM62647.1        PYPISFLLSDLINRRTQRVDGQPMYGMESQVEEVRVFDGTEQLPGDPDMIRYIDRQGQLQ  358
ALF06326.1        PYPISFLLSDLINRRTQRVDGQPMYGMESQVEEVRVFDGTEQLPGDPDMIRYIDRQGQLQ  358
ALF06329.1        PYPISFLLSDLINRRTQRVDGQPMYGMESQVEEVRVFDGTEQLPGDPDMIRYIDRQGQLQ  358
AHU87338.1        PYPISFLLSDLINRRTQRVDGQPMYGMESQVEEVRVFDGTEQLPGDPDMIRYIDRQGQLQ  358
AHU87339.1        PYPISFLLSDLINRRTQRVDGQPMYGMESQVEEVRVFDGTEQLPGDPDMIRYIDRQGQLQ  358
AHU87350.1        PYPISFLLSDLINRRTQRVDGQPMYGMESQVEEVRVFDGTEQLPGDPDMIRYIDRQGQLQ  358
AHU87351.1        PYPISFLLSDLINRRTQRVDGQPMYGMESQVEEVRVFDGTEQLPGDPDMIRYIDRQGQLQ  358
AHU87352.1        PYPISFLLSDLINRRTQRVDGQPMYGMESQVEEVRVFDGTEQLPGDPDMIRYIDRQGQLQ  358
AHU87353.1        PYPISFLLSDLINRRTQRVDGQPMYGMESQVEEVRVFDGTEQLPGDPDMIRYIDRQGQLQ  358
AFQ31653.1        PYPISFLLSDLINRRTQRVDGQPMYGMESQVEEVRVFDGTEQLPGDPDMIRYIDRQGQLQ  358
AFA41884.1        PYPISFLLSDLINRRTQRVDGQPMYGMESQVEEVRVFDGTEQLPGDPDMIRYIDRQGQLQ  358
CBX88320.1        PYPISFLLSDLINRRTQRVDGQPMYGMESQVEEVRVFDGTEQLPGDPDMIRYIDRQGQLQ  358
CBX88326.1        PYPISFLLSDLINRRTQRVDGQPMYGMESQVEEVRVFDGTEQLPGDPDMIRYIDRQGQLQ  358
CBX88338.1        PYPISFLLSDLINRRTQRVDGQPMYGMESQVEEVRVFDGTEQLPGDPDMIRYIDRQGQLQ  358
CBX88344.1        PYPISFLLSDLINRRTQRVDGQPMYGMESQVEEVRVFDGTEQLPGDPDMIRYIDRQGQLQ  358
BAG84452.1        PYPISFLLSDLINRRTQRVDGQPMYGMESQVEEVRVFDGTEQLPGDPDMIRYIDRQGQLQ  358
BAG75385.1        PYPISFLLSDLINRRTQRVDGQPMYGMESQVEEVRVFDGTEQLPGDPDMIRYIDRQGQLQ  358
BAG75445.1        PYPISFLLSDLINRRTQRVDGQPMYGMESQVEEVRVFDGTEQLPGDPDMIRYIDRQGQLQ  358
BAF93343.1        PYPISFLLSDLINRRTQRVDGQPMYGMESQVEEVRVFDGTEQLPGDPDMIRYIDRQGQLQ  358
BAF75066.1        PYPISFLLSDLINRRTQRVDGQPMYGMESQVEEVRVFDGTEQLPGDPDMIRYIDRQGQLQ  358
BAF75072.1        PYPISFLLSDLINRRTQRVDGQPMYGMESQVEEVRVFDGTEQLPGDPDMIRYIDRQGQLQ  358
BAF75078.1        PYPISFLLSDLINRRTQRVDGQPMYGMESQVEEVRVFDGTEQLPGDPDMIRYIDRQGQLQ  358
BAF75084.1        PYPISFLLSDLINRRTQRVDGQPMYGMESQVEEVRVFDGTEQLPGDPDMIRYIDRQGQLQ  358
BAF75108.1        PYPISFLLSDLINRRTQRVDGQPMYGMESQVEEVRVFDGTEQLPGDPDMIRYIDRQGQLQ  358
BAF75132.1        PYPISFLLSDLINRRTQRVDGQPMYGMESQVEEVRVFDGTEQLPGDPDMIRYIDRQGQLQ  358
BAF75216.1        PYPISFLLSDLINRRTQRVDGQPMYGMESQVEEVRVFDGTEQLPGDPDMIRYIDRQGQLQ  358
BAF75222.1        PYPISFLLSDLINRRTQRVDGQPMYGMESQVEEVRVFDGTEQLPGDPDMIRYIDRQGQLQ  358
BAF75228.1        PYPISFLLSDLINRRTQRVDGQPMYGMESQVEEVRVFDGTEQLPGDPDMIRYIDRQGQLQ  358
BAF75246.1        PYPISFLLSDLINRRTQRVDGQPMYGMESQVEEVRVFDGTEQLPGDPDMIRYIDRQGQLQ  358
BAF75252.1        PYPISFLLSDLINRRTQRVDGQPMYGMESQVEEVRVFDGTEQLPGDPDMIRYIDRQGQLQ  358
BAF75258.1        PYPISFLLSDLINRRTQRVDGQPMYGMESQVEEVRVFDGTEQLPGDPDMIRYIDRQGQLQ  358
BAF75276.1        PYPISFLLSDLINRRTQRVDGQPMYGMESQVEEVRVFDGTEQLPGDPDMIRYIDRQGQLQ  358
BAF75330.1        PYPISFLLSDLINRRTQRVDGQPMYGMESQVEEVRVFDGTEQLPGDPDMIRYIDRQGQLQ  358
BAF03115.1        PYPISFLLSDLINRRTQRVDGQPMYGMESQVEEVRVFDGTEQLPGDPDMIRYIDRQGQLQ  358
BAF03120.1        PYPISFLLSDLINRRTQRVDGQPMYGMESQVEEVRVFDGTEQLPGDPDMIRYIDRQGQLQ  358
WLD47842.1        PYPISFLLSDLINRRTQRVDGQPMYGMESQVEEVRVFDGTEQLPGDPDMIRYIDRQGQLQ  358
WLD47884.1        PYPISFLLSDLINRRTQRVDGQPMYGMESQVEEVRVFDGTEQLPGDPDMIRYIDRQGQLQ  358
UTM62101.1        PYPISFLLSDLINRRTQRVDGQPMYGMESQVEEVRVFDGTEQLPGDPDMIRYIDRQGQLQ  358
UTM62107.1        PYPISFLLSDLINRRTQRVDGQPMYGMESQVEEVRVFDGTEQLPGDPDMIRYIDRQGQLQ  358
UTM62113.1        PYPISFLLSDLINRRTQRVDGQPMYGMESQVEEVRVFDGTEQLPGDPDMIRYIDRQGQLQ  358
AFQ31647.1        PYPISFLLSDLINRRTQRVDGQPMYGMESQVEEVRVFDGTEQLPGDPDMIRYIDRQGQLQ  358
AFA41892.1        PYPISFLLSDLINRRTQRVDGQPMYGMESQVEEVRVFDGTEQLPGDPDMIRYIDRQGQLQ  358
BAG75403.1        PYPISFLLSDLINRRTQRVDGQPMYGMESQVEEVRVFDGTEQLPGDPDMIRYIDRQGQLQ  358
BAF75114.1        PYPISFLLSDLINRRTQRVDGQPMYGMESQVEEVRVFDGTEQLPGDPDMIRYIDRQGQLQ  358
BAF75120.1        PYPISFLLSDLINRRTQRVDGQPMYGMESQVEEVRVFDGTEQLPGDPDMIRYIDRQGQLQ  358
BAF75126.1        PYPISFLLSDLINRRTQRVDGQPMYGMESQVEEVRVFDGTEQLPGDPDMIRYIDRQGQLQ  358
BAF75186.1        PYPISFLLSDLINRRTQRVDGQPMYGMESQVEEVRVFDGTEQLPGDPDMIRYIDRQGQLQ  358
BAF75288.1        PYPISFLLSDLINRRTQRVDGQPMYGMESQVEEVRVFDGTEQLPGDPDMIRYIDRQGQLQ  358
BAF75294.1        PYPISFLLSDLINRRTQRVDGQPMYGMESQVEEVRVFDGTEQLPGDPDMIRYIDRQGQLQ  358
BAF75348.1        PYPISFLLSDLINRRTQRVDGQPMYGMESQVEEVRVFDGTEQLPGDPDMIRYIDRQGQLQ  358
AFA41881.1        PYPISFLLSDLINRRTQRVDGQPMYGMESQVEEVRVFDGTEQLPGDPDMIRYIDRQGQLQ  358
                  ** **  :****.* ** *****:******* ******  *     **:*****: ***:

ALF06344.1        XKMX  362
QTJ15012.1        TKML  362
QTJ15120.1        TKML  362
QHI34657.1        TKMV  362
ARE72390.1        TKML  362
QTJ14970.1        TKMV  362
QTJ14928.1        TKML  364
AAT47419.1        TKML  362
AAT47413.1        TKML  362
```

AFQ31661.1	TKML	362
YP_717939.1	TKML	362
AFK08545.1	TKML	362
P03088.2	TKML	362
CAA24299.1	TKML	362
QTJ14922.1	TKML	362
AFA41877.1	TKML	362
AAT47371.1	TKML	362
AYO46658.1	TKIL	362
AYO46610.1	TKML	362
AYO46689.1	TKML	362
AYO46693.1	TKML	362
BAG75283.1	TKML	362
BAG75313.1	TKML	362
BAG75373.1	TKML	362
BCV68914.1	TKML	362
BCV68917.1	TKML	362
BCV68923.1	TKML	362
AYO46620.1	TKML	362
AYO46646.1	TKML	362
AYO46663.1	TKML	362
AYO46694.1	TKML	362
BAZ99906.1	TKML	362
BAZ99912.1	TKML	362
BAI43576.1	TKML	362
BAI43594.1	TKML	362
BAI43606.1	TKML	362
BAI43624.1	TKML	362
BAH23450.1	TKML	362
BAH23456.1	TKML	362
BAH23462.1	TKML	362
BAH23468.1	TKML	362
BAH23474.1	TKML	362
BAH23480.1	TKML	362
BAH23486.1	TKML	362
BAH23492.1	TKML	362
BAH23498.1	TKML	362
BAH23504.1	TKML	362
BAH23510.1	TKML	362
BAH23516.1	TKML	362
BAH23522.1	TKML	362
BAH23528.1	TKML	362
BAH23534.1	TKML	362
BAH23540.1	TKML	362
BAH23546.1	TKML	362
BAH23552.1	TKML	362
BAH23558.1	TKML	362
BAG84488.1	TKML	362
BAG84494.1	TKML	362
BAG84500.1	TKML	362
BAG84506.1	TKML	362
BAG84512.1	TKML	362
BAG84518.1	TKML	362
BAG84524.1	TKML	362
BAG75163.1	TKML	362
BAG75169.1	TKML	362
BAG75175.1	TKML	362
BAG75181.1	TKML	362
BAG75187.1	TKML	362
BAG75193.1	TKML	362
BAG75199.1	TKML	362
BAG75241.1	TKML	362
BAG75247.1	TKML	362
BAG75253.1	TKML	362

BAG75265.1	TKML	362
BAG75289.1	TKML	362
BAG75295.1	TKML	362
BAG75307.1	TKML	362
BAG75325.1	TKML	362
BAG75331.1	TKML	362
BAG75337.1	TKML	362
BAG75343.1	TKML	362
BAG75397.1	TKML	362
BAG75421.1	TKML	362
BAG75427.1	TKML	362
BAG75433.1	TKML	362
BAF93349.1	TKML	362
BAF76166.1	TKML	362
BAF76172.1	TKML	362
BAF76178.1	TKML	362
BAF76184.1	TKML	362
BAF76190.1	TKML	362
BAF76202.1	TKML	362
BAF42985.1	TKML	362
BAF42991.1	TKML	362
BAF02951.1	TKML	362
BAF02963.1	TKML	362
BAF02969.1	TKML	362
BAF02981.1	TKML	362
BAF02987.1	TKML	362
BAF02993.1	TKML	362
BAF02999.1	TKML	362
BAF03005.1	TKML	362
BAF03011.1	TKML	362
BAE96065.1	TKML	362
BAE53666.1	TKML	362
BAE46623.1	TKML	362
CAA40235.1	TKML	362
BAI43570.1	TKML	362
BAI43582.1	TKML	362
BAI43612.1	TKML	362
BAI43618.1	TKML	362
BAI43630.1	TKML	362
BAG75301.1	TKML	362
BAG75349.1	TKML	362
BAG75367.1	TKML	362
BAF76196.1	TKML	362
BAF02975.1	TKML	362
BAI43600.1	TKML	362
BAE96071.1	TKML	362
BAE53660.1	TKML	362
BCV68926.1	TKML	362
AYO46647.1	TKML	362
BAR80134.1	TKML	362
BAR80140.1	TKML	362
BAI43588.1	TKML	362
BAG75361.1	TKML	362
BAE53642.1	TKML	362
BAE53648.1	TKML	362
AFA41903.1	TKML	362
AFA41918.1	TKML	362
BAF42937.1	TKML	362
BAF42997.1	TKML	362
WLD47902.1	TKML	362
WLD47938.1	TKML	362
WLD47980.1	TKML	362
UTM61891.1	TKML	362
UTM61897.1	TKML	362

UTM61903.1	TKML	362
UTM62215.1	TKML	362
UTM62221.1	TKML	362
UTM62275.1	TKML	362
UTM62281.1	TKML	362
UTM62329.1	TKML	362
UTM62335.1	TKML	362
UTM62383.1	TKML	362
UTM62395.1	TKML	362
UTM62443.1	TKML	362
UTM62449.1	TKML	362
UTM62467.1	TKML	362
UTM62545.1	TKML	362
UTM62551.1	TKML	362
UTM62587.1	TKML	362
UTM62593.1	TKML	362
UTM62599.1	TKML	362
QUW04850.1	TKML	362
QTJ14976.1	TKML	362
QTJ15000.1	TKML	362
QTJ15054.1	TKML	362
QTJ15072.1	TKML	362
QTJ15084.1	TKML	362
QTJ15090.1	TKML	362
QTJ15108.1	TKML	362
QTJ15114.1	TKML	362
QTJ15132.1	TKML	362
QTJ15138.1	TKML	362
AYO46612.1	TKML	362
AYO46613.1	TKML	362
AYO46617.1	TKML	362
AYO46621.1	TKML	362
AYO46622.1	TKML	362
AYO46623.1	TKML	362
AYO46624.1	TKML	362
AYO46629.1	TKML	362
AYO46632.1	TKML	362
AYO46635.1	TKML	362
AYO46638.1	TKML	362
AYO46641.1	TKML	362
AYO46642.1	TKML	362
AYO46644.1	TKML	362
AYO46651.1	TKML	362
AYO46652.1	TKML	362
AYO46659.1	TKML	362
AYO46660.1	TKML	362
AYO46662.1	TKML	362
AYO46665.1	TKML	362
AYO46670.1	TKML	362
AYO46672.1	TKML	362
AYO46676.1	TKML	362
AYO46677.1	TKML	362
AYO46679.1	TKML	362
AYO46682.1	TKML	362
AYO46691.1	TKML	362
AYO46692.1	TKML	362
AUF70040.1	TKML	362
SOH95722.1	TKML	362
SOI09711.1	TKML	362
APG38208.1	TKML	362
ALF06332.1	TKML	362
ALF06341.1	TKML	362
BAR80122.1	TKML	362
BAR80128.1	TKML	362

AJG42164.1	TKML	362
AHU87340.1	TKML	362
AHU87341.1	TKML	362
AHU87368.1	TKML	362
AHU87369.1	TKML	362
AFQ31636.1	TKML	362
AFQ31637.1	TKML	362
AFQ31639.1	TKML	362
AFQ31640.1	TKML	362
AFQ31641.1	TKML	362
AFQ31644.1	TKML	362
AFQ31648.1	TKML	362
AFQ31657.1	TKML	362
AFA41882.1	TKML	362
AFA41887.1	TKML	362
AFA41890.1	TKML	362
AFA41891.1	TKML	362
AFA41895.1	TKML	362
AFA41898.1	TKML	362
AFA41901.1	TKML	362
AFA41902.1	TKML	362
AFA41910.1	TKML	362
AFA41914.1	TKML	362
AFA41917.1	TKML	362
AEO89562.1	TKML	362
AEO89568.1	TKML	362
AEO89586.1	TKML	362
AEO89598.1	TKML	362
AEO89621.1	TKML	362
AEK21505.1	TKML	362
BAG84458.1	TKML	362
BAG84470.1	TKML	362
BAG84482.1	TKML	362
BAG75319.1	TKML	362
BAG75355.1	TKML	362
BAG75391.1	TKML	362
BAG75439.1	TKML	362
BAF93301.1	TKML	362
BAF93331.1	TKML	362
BAF93355.1	TKML	362
BAF76208.1	TKML	362
BAF42877.1	TKML	362
BAF42883.1	TKML	362
BAF42889.1	TKML	362
BAF42961.1	TKML	362
BAF42967.1	TKML	362
BAF42973.1	TKML	362
BAF43003.1	TKML	362
BAF43027.1	TKML	362
BAF43033.1	TKML	362
ABI94659.1	TKML	362
ABI94683.1	TKML	362
ABI94719.1	TKML	362
BAE96053.1	TKML	362
BAE96059.1	TKML	362
ABC18005.1	TKML	362
AAT47377.1	TKML	362
AAT47383.1	TKML	362
AAT47405.1	TKML	362
CAA40239.1	TKML	362
QTJ14940.1	TKML	362
ALF06317.1	TKML	362
ALF06307.1	TKML	362
ALF06297.1	TKMX	362

ALF06350.1	TKML	362
AAT47431.1	TKML	362
QTJ15006.1	TKML	362
AYO46667.1	TKML	362
WLD47956.1	TKML	362
QTJ15150.1	TKML	362
UTM62389.1	TKML	362
ABI94701.1	TKML	362
BAF02945.1	TKML	362
CAA40243.1	TKML	362
ABI94725.1	TKML	362
AFQ31660.1	TKML	362
ALF06353.1	TKML	362
ALF06312.1	TKML	362
QEJ80721.1	TKML	362
AYO46614.1	TKML	362
AYO46619.1	TKML	362
AYO46625.1	TKML	362
AYO46626.1	TKML	362
AYO46628.1	TKML	362
AYO46633.1	TKML	362
AYO46636.1	TKML	362
AYO46637.1	TKML	362
AYO46640.1	TKML	362
AYO46648.1	TKML	362
AYO46650.1	TKML	362
AYO46653.1	TKML	362
AYO46654.1	TKML	362
AYO46671.1	TKML	362
AYO46673.1	TKML	362
AYO46674.1	TKML	362
AYO46675.1	TKML	362
AYO46680.1	TKML	362
AYO46681.1	TKML	362
AYO46684.1	TKML	362
AYO46687.1	TKML	362
AEO89574.1	TKML	362
BAF42979.1	TKML	362
BAF43009.1	TKML	362
AYO46678.1	NKML	362
UTM62479.1	TKML	362
QTJ14964.1	TKML	362
QTJ15036.1	TKML	362
QTJ15066.1	TKML	362
QTJ15078.1	TKML	362
QTJ15096.1	TKML	362
AQM40276.1	TKML	362
ABI94689.1	TKML	362
ABI94671.1	TKML	362
AYO46688.1	TKML	362
QTJ15060.1	TKML	362
AFQ31642.1	TKML	362
AYO46616.1	TKML	362
CAA79594.1	TKMV	362
WLD47818.1	TKML	362
UTM62155.1	TKML	362
AFA41907.1	TKML	362
WLD47944.1	TKML	362
AYO46668.1	TKML	362
AFQ31651.1	TKML	362
QTJ14958.1	TKML	362
AYO46618.1	TKML	362
QTJ15018.1	TKML	362
AYO46609.1	TKML	362

ALF06356.1	TKML	362
AFQ31646.1	TKML	362
AFQ31659.1	TKML	362
BAF02957.1	TKML	362
AAT47365.1	TKML	362
AAT47389.1	TKML	362
AAT47395.1	TKML	362
AAT47401.1	TKML	362
CAA24307.1	TKML	362
AAT47425.1	TKML	362
AFA41880.1	TKML	362
WLD47968.1	TKML	362
AFA41920.1	TKML	362
CBX88302.1	TKML	362
AUF70036.1	TKML	362
ALF06323.1	TKML	362
WLD47890.1	TKML	362
BAF03097.1	TKML	362
ABI94695.1	TKML	362
ABI94623.1	TKML	362
UTM62533.1	TKML	362
QOS45058.1	TKML	362
AHU87362.1	TKML	362
AHU87363.1	TKML	362
AFQ31658.1	TKML	362
AFA41876.1	TKML	362
AFA41906.1	TKML	362
AEO89604.1	TKML	362
CBX88350.1	TKML	362
BAG84446.1	TKML	362
BAG84464.1	TKML	362
BAF93295.1	TKML	362
BAF93373.1	TKML	362
BAF93379.1	TKML	362
BAF42913.1	TKML	362
BAF42931.1	TKML	362
BAF43015.1	TKML	362
ABI94629.1	TKML	362
ABI94653.1	TKML	362
ABI94707.1	TKML	362
BAF03085.1	TKML	362
BAF03091.1	TKML	362
BAF03103.1	TKML	362
BAF03109.1	TKML	362
BAF93325.1	TKMV	362
QTJ14934.1	TKML	362
QTJ14994.1	TKML	362
AFA41886.1	TKML	362
AFQ31650.1	TKML	362
AAT47347.1	TKMV	362
ABI94617.1	TKMV	362
BAF93319.1	TKMV	362
CBX88314.1	TKMV	362
ALF06320.1	TKMV	362
WLD47830.1	TKMV	362
WLD47866.1	TKMV	362
UTM61939.1	TKMV	362
UTM62023.1	TKMV	362
UTM62029.1	TKMV	362
UTM62053.1	TKMV	362
UTM62059.1	TKMV	362
UTM62065.1	TKMV	362
UTM62317.1	TKMV	362
UTM62323.1	TKMV	362

UTM62353.1	TKMV	362
UTM62473.1	TKMV	362
UTM62491.1	TKMV	362
UTM62497.1	TKMV	362
UTM62503.1	TKMV	362
QTJ14988.1	TKMV	362
QTJ15024.1	TKMV	362
QTJ15030.1	TKMV	362
QTJ15102.1	TKMV	362
ALF06300.1	TKMV	362
AHU87342.1	TKMV	362
AHU87343.1	TKMV	362
AHU87346.1	TKMV	362
AHU87347.1	TKMV	362
AHU87349.1	TKMV	362
AHU87356.1	TKMV	362
AHU87357.1	TKMV	362
AHU87358.1	TKMV	362
AHU87359.1	TKMV	362
AFQ31643.1	TKMV	362
AFQ31656.1	TKMV	362
AFA41879.1	TKMV	362
AFA41897.1	TKMV	362
AFA41900.1	TKMV	362
AEO89580.1	TKMV	362
AEO89592.1	TKMV	362
BAF93277.1	TKMV	362
BAF93289.1	TKMV	362
BAF93307.1	TKMV	362
BAF93337.1	TKMV	362
BAF42895.1	TKMV	362
BAF42919.1	TKMV	362
BAF42943.1	TKMV	362
BAF42949.1	TKMV	362
ABI94665.1	TKMV	362
ABI94677.1	TKMV	362
BAF02939.1	TKMV	362
AAT47353.1	TKMV	362
AAT47359.1	TKMV	362
CAA40247.1	TKMV	362
QTJ14982.1	TKMV	362
QTJ15126.1	TKMV	362
ABI94635.1	TKML	362
ALF06335.1	TKML	362
AHU87348.1	TKML	362
AFQ31649.1	TKML	362
AFQ31654.1	TKML	362
BAF42907.1	TKML	362
WLD47824.1	TKML	362
WLD47848.1	TKML	362
WLD47854.1	TKML	362
WLD47860.1	TKML	362
WLD47878.1	TKML	362
WLD47908.1	TKML	362
WLD47914.1	TKML	362
WLD47920.1	TKML	362
WLD47926.1	TKML	362
WLD47932.1	TKML	362
WLD47950.1	TKML	362
WLD47962.1	TKML	362
WLD47974.1	TKML	362
WLD47998.1	TKML	362
WLD48004.1	TKML	362
WLD48016.1	TKML	362

WLD48022.1	TKML	362
WLD48034.1	TKML	362
UTM61909.1	TKML	362
UTM61915.1	TKML	362
UTM61921.1	TKML	362
UTM61927.1	TKML	362
UTM61933.1	TKML	362
UTM61963.1	TKML	362
UTM61969.1	TKML	362
UTM61975.1	TKML	362
UTM61981.1	TKML	362
UTM62005.1	TKML	362
UTM62017.1	TKML	362
UTM62035.1	TKML	362
UTM62041.1	TKML	362
UTM62047.1	TKML	362
UTM62083.1	TKML	362
UTM62089.1	TKML	362
UTM62095.1	TKML	362
UTM62149.1	TKML	362
UTM62161.1	TKML	362
UTM62167.1	TKML	362
UTM62173.1	TKML	362
UTM62179.1	TKML	362
UTM62185.1	TKML	362
UTM62191.1	TKML	362
UTM62197.1	TKML	362
UTM62203.1	TKML	362
UTM62209.1	TKML	362
UTM62227.1	TKML	362
UTM62239.1	TKML	362
UTM62245.1	TKML	362
UTM62251.1	TKML	362
UTM62257.1	TKML	362
UTM62263.1	TKML	362
UTM62269.1	TKML	362
UTM62287.1	TKML	362
UTM62293.1	TKML	362
UTM62299.1	TKML	362
UTM62305.1	TKML	362
UTM62311.1	TKML	362
UTM62359.1	TKML	362
UTM62365.1	TKML	362
UTM62371.1	TKML	362
UTM62377.1	TKML	362
UTM62455.1	TKML	362
UTM62461.1	TKML	362
UTM62485.1	TKML	362
UTM62509.1	TKML	362
UTM62515.1	TKML	362
UTM62521.1	TKML	362
UTM62527.1	TKML	362
UTM62539.1	TKML	362
UTM62557.1	TKML	362
UTM62563.1	TKML	362
UTM62569.1	TKML	362
UTM62575.1	TKML	362
UTM62581.1	TKML	362
UTM62605.1	TKML	362
UTM62617.1	TKML	362
UTM62623.1	TKML	362
UTM62629.1	TKML	362
UTM62635.1	TKML	362
QTJ15048.1	TKML	362

QTJ15144.1	TKML	362
AHU87360.1	TKML	362
AHU87361.1	TKML	362
AHU87366.1	TKML	362
AHU87367.1	TKML	362
AFQ31652.1	TKML	362
AFA41888.1	TKML	362
AFA41893.1	TKML	362
AFA41894.1	TKML	362
AFA41899.1	TKML	362
AFA41904.1	TKML	362
AFA41905.1	TKML	362
AFA41908.1	TKML	362
AFA41911.1	TKML	362
AFA41912.1	TKML	362
AFA41913.1	TKML	362
AFA41915.1	TKML	362
AFA41916.1	TKML	362
AFA41919.1	TKML	362
AFA41921.1	TKML	362
AFA41922.1	TKML	362
AFA41923.1	TKML	362
AFA41924.1	TKML	362
AFA41925.1	TKML	362
CBX88272.1	TKML	362
CBX88278.1	TKML	362
CBX88284.1	TKML	362
CBX88290.1	TKML	362
CBX88296.1	TKML	362
CBX88308.1	TKML	362
CBX88332.1	TKML	362
CBX88356.1	TKML	362
CBX88362.1	TKML	362
BAF93313.1	TKML	362
BAF42955.1	TKML	362
BAF43021.1	TKML	362
ABI94611.1	TKML	362
ABI94641.1	TKML	362
ABI94647.1	TKML	362
WLD47896.1	TKML	362
WLD48028.1	TKML	362
BAG84440.1	TKML	362
BAF93283.1	TKML	362
BAF93361.1	TKML	362
WLD48040.1	TKML	362
UTM62611.1	TKML	362
AFQ31645.1	TKML	362
AFA41909.1	TKML	362
ABI94713.1	TKML	362
ABD04662.1	TKML	362
QTJ15042.1	TKML	362
AGX89705.1	TKMV	362
AGX89710.1	TKMV	362
AGX89717.1	TKMV	362
AFQ31655.1	TKMV	362
AAA46882.1	TKMV	362
P14996.1	TKMV	362
AEO89615.1	TKMV	362
BCV68929.1	TKML	362
QTJ14952.1	TKMV	362
BAG75157.1	TKMV	362
BAG75211.1	TKMV	362
BAF03017.1	TKMV	362
BAF42925.1	TKMV	362

WLD47836.1	TKMV	362
UEL36354.1	TKMV	362
BAF42901.1	TKMV	362
ABP87973.1	TKMV	362
AFA41878.1	TKMV	362
QHI34655.1	TKMV	362
UTM61945.1	TKMV	362
UTM61987.1	TKMV	362
UTM62233.1	TKMV	362
UTM62341.1	TKMV	362
UTM62347.1	TKMV	362
AHU87354.1	TKMV	362
AHU87355.1	TKMV	362
AHU87364.1	TKMV	362
AHU87365.1	TKMV	362
AFQ31638.1	TKMV	362
AFA41874.1	TKMV	362
AFA41875.1	TKMV	362
AFA41896.1	TKMV	362
BAF93367.1	TKMV	362
CAA79596.1	TKMV	362
AFA41883.1	TKMV	362
WLD47812.1	TKMV	362
BAG75277.1	TKMV	362
BAF75138.1	TKMV	362
BAF75102.1	TKMV	362
AYO46643.1	TKMV	362
QUW04844.1	TKMV	362
AYO46683.1	TKMV	362
AYO46645.1	TKMV	362
BAF75180.1	TKMV	362
BAF75204.1	TKMV	362
AYO46631.1	TKMV	362
AYO46608.1	TKMV	362
AYO46611.1	TKMV	362
AYO46615.1	TKMV	362
AYO46639.1	TKMV	362
AYO46649.1	TKMV	362
AYO46655.1	TKMV	362
AYO46664.1	TKMV	362
AYO46669.1	TKMV	362
AYO46685.1	TKMV	362
AYO46686.1	TKMV	362
AYO46690.1	TKMV	362
AYO46627.1	TKMV	362
AYO46630.1	TKMV	362
AYO46656.1	TKMV	362
BAG75217.1	TKMV	362
BAG75229.1	TKMV	362
BAG75235.1	TKMV	362
BAG75259.1	TKMV	362
BAF75150.1	TKMV	362
BAF75156.1	TKMV	362
BAF75174.1	TKMV	362
BAE96083.1	TKMV	362
BAE53654.1	TKMV	362
BAF03035.1	TKMV	362
QHI34656.1	TKMV	362
ARE72396.1	TKMV	362
AFA41885.1	TKMV	362
BAG75271.1	TKMV	362
BAF75090.1	TKMV	362
BAF75306.1	TKMV	362
BAF75342.1	TKMV	362

BAF03029.1	TKMV	362
BAG75205.1	TKMV	362
BAG75223.1	TKMV	362
BAF75144.1	TKMV	362
BAF75162.1	TKMV	362
BAF75168.1	TKMV	362
BAF75240.1	TKMV	362
BAF03023.1	TKMV	362
BAE96077.1	TKMV	362
BAG84476.1	TKMV	362
AYO46661.1	TKMV	362
AYO46634.1	TKMV	362
AYO46657.1	TKMV	362
BAG75379.1	TKMV	362
BAG75409.1	TKMV	362
BAG75415.1	TKMV	362
BAF75096.1	TKMV	362
BAF75192.1	TKMV	362
BAF75198.1	TKMV	362
BAF75210.1	TKMV	362
BAF75234.1	TKMV	362
BAF75264.1	TKMV	362
BAF75270.1	TKMV	362
BAF75282.1	TKMV	362
BAF75300.1	TKMV	362
BAF75312.1	TKMV	362
BAF75318.1	TKMV	362
BAF75324.1	TKMV	362
BAF75336.1	TKMV	362
AFA41889.1	TKMV	362
UEL36357.1	TKMV	362
BCV68920.1	TKML	362
WLD47992.1	TKMV	362
UTM62413.1	TKMV	362
AHU87344.1	TKML	362
AHU87345.1	TKML	362
QTJ14946.1	TKMV	362
AYO46666.1	TKMV	362
WLD47788.1	TKMV	362
WLD47794.1	TKMV	362
WLD47800.1	TKMV	362
WLD47806.1	TKMV	362
WLD47872.1	TKMV	362
WLD47986.1	TKMV	362
WLD48010.1	TKMV	362
UTM61951.1	TKMV	362
UTM61957.1	TKMV	362
UTM61993.1	TKMV	362
UTM61999.1	TKMV	362
UTM62011.1	TKMV	362
UTM62071.1	TKMV	362
UTM62077.1	TKMV	362
UTM62119.1	TKMV	362
UTM62125.1	TKMV	362
UTM62131.1	TKMV	362
UTM62137.1	TKMV	362
UTM62143.1	TKMV	362
UTM62401.1	TKMV	362
UTM62407.1	TKMV	362
UTM62419.1	TKMV	362
UTM62425.1	TKMV	362
UTM62431.1	TKMV	362
UTM62437.1	TKMV	362
UTM62641.1	TKMV	362

```
UTM62647.1        TKMV  362
ALF06326.1        TKMV  362
ALF06329.1        TKMV  362
AHU87338.1        TKMV  362
AHU87339.1        TKMV  362
AHU87350.1        TKMV  362
AHU87351.1        TKMV  362
AHU87352.1        TKMV  362
AHU87353.1        TKMV  362
AFQ31653.1        TKMV  362
AFA41884.1        TKMV  362
CBX88320.1        TKMV  362
CBX88326.1        TKMV  362
CBX88338.1        TKMV  362
CBX88344.1        TKMV  362
BAG84452.1        TKMV  362
BAG75385.1        TKMV  362
BAG75445.1        TKMV  362
BAF93343.1        TKMV  362
BAF75066.1        TKMV  362
BAF75072.1        TKMV  362
BAF75078.1        TKMV  362
BAF75084.1        TKMV  362
BAF75108.1        TKMV  362
BAF75132.1        TKMV  362
BAF75216.1        TKMV  362
BAF75222.1        TKMV  362
BAF75228.1        TKMV  362
BAF75246.1        TKMV  362
BAF75252.1        TKMV  362
BAF75258.1        TKMV  362
BAF75276.1        TKMV  362
BAF75330.1        TKMV  362
BAF03115.1        TKMV  362
BAF03120.1        TKMV  362
WLD47842.1        TKMV  362
WLD47884.1        TKMV  362
UTM62101.1        TKMV  362
UTM62107.1        TKMV  362
UTM62113.1        TKMV  362
AFQ31647.1        TKMV  362
AFA41892.1        TKMV  362
BAG75403.1        TKMV  362
BAF75114.1        TKMV  362
BAF75120.1        TKMV  362
BAF75126.1        TKMV  362
BAF75186.1        TKMV  362
BAF75288.1        TKMV  362
BAF75294.1        TKMV  362
BAF75348.1        TKMV  362
AFA41881.1        TKMV  362
                   *:
```
