# Supplementary material for: The Conserved YPX3L Motif in the BK Polyomavirus VP1 Protein Is Important for Viral Particle Assembly but Not for Its Secretion into Extracellular Vesicles
Source: Viruses. 2024 Jul 13;16(7):1124. doi: 10.3390/v16071124 (PMC11281352; doi:10.3390/v16071124)
Supplement: Supplementary file 1 [file viruses-16-01124-s001.zip › HPyV 02 alignment.pdf]

CLUSTAL O(1.2.4) multiple sequence alignment

```
AEC14619.1      MAPTKRKGERKDPVQVPKLLIRGGVEVLEVKTGVDSITEVECFLTPEMGDPDEHLRGFSK  60
AEC15199.1      MAPTKRKGERKDPVQVPKLLIRGGVEVLEVKTGVDSITEVECFLTPEMGDPDEHLRGFSK  60
AEC15209.1      MVPTKRKGERKDPVQVPKLLIRGGVEVLEVKTGVDSITEVECFLTPEMGDPDEHLRGFSK  60
UPN63098.1      MAPTKRKGERKDPVQVPKLLIRGGVEVLEVKTGVDSITEVECFLTPEMGDPDEHLRGFSN  60
UPN63097.1      MAPTKRKGERKDPVQVPKLLIRGGVEVLEVKTGVDSITEVERFLTPEMGDPDEHLRGFSN  60
UPN63094.1      MAPTKRKGERKDTVQVPKLLIRGGVEVLEVKTGVDPITEVECFLTPEMGDPDEHLRGFSN  60
UPN63096.1      MAPTKRKGERKDPVQVPKLLIRGGVEVLEVKTGVDSITEVECFLTPEMGDPDEHLRGFSN  60
QWT77146.1      MAPTKRKGERKDPVQVPKLLIRGGVEVLEVKTGVDSITEVECFLTPEMGDPDEHLRGFSN  60
UPN63095.1      MAPIKRKGERKDPVQVPKLLIRGGVEVLEVKTGVDSITEVECFLTPEMGDPDEHLRGFSN  60
BAA01966.1      MAPTKRKGERKDPVQVPKLLIRGGVEVLEVKTGVDSITEVECFLTPEMGDPDEHLRGFSK  60
AEC15099.1      MAPTKRKGERKDPVQVPKLLIRGGVEVLEVKTGVDSITEVECFLTPEMGDPDEHLRGFSK  60
UPN63099.1      MAPTKRKGERKDPVQVPKLLIRGGVEVLEVKTGVDSITEVECFLTPEMGDPDEHLRGFSK  60
UPN63086.1      MAPTKRKGERKDPVQVPKLLIRGGVEVLEVKTGVDSITEVECFLTPEMGDPDEHLRGFSK  60
UPN63092.1      MAPTKRKGERKDPVQVPKLLIRGGVEVLEVKTGVDSITEVECFLTPEMGDPDEHLRGFSK  60
AEC15783.1      MAPTKRKGERKDPVQVPKLLIRGGVEVLEVKTGVDSITEVECFLTPEMGDPDEHFRGFSK  60
AEC15735.1      MAPTKRKGERKDPVQVPKLLIRGGVEVLEVKTGVDSITEVECFLTPEMGDPDEHFRGFSK  60
AEC15615.1      MAPTKRKGERKDPVQVPKLLIRGGVEVLEVKTGVDSITEVECFLTPEMGDPDEHFRGFSK  60
AEC15549.1      MAPTKRKGERKDHVQVPKLLIRGGVEVLEVKTGVDSITEVECFLTPEMGDPDEHFRGFSK  60
AEC15081.1      MVPTKRKGERKDPVQVPKLLIRGGVEVLEVKTGVDSITEVECFLTPEMGDPDEHFRGFSK  60
AEC15080.1      MAPTKRKGERKDPVQVPKLLIRGGVEVLEVKTGVDSITEVECFLTPEMGDPDEHFRGFSK  60
AEC15083.1      MAPTKRKGERKDPVQVPKLLIRGGVEVLEVKTGVDSITEVECFLTPEMGDPDEHFRGFSK  60
QWT77109.1      MAPTKRKGERKDPVQVPKLLIRGGVEVLEVKTGVDSITEVECFLTPEMGDPDEHFRGFSK  60
AEC15069.1      MAPTKRKGERKDPVQVPKLLIRGGVEVLEVKTGVDSITEVECFLTPEMGDPDEHFRGFSK  60
AEC15070.1      MAPTKRKGERKDPVQVPKLLIRGGVEVLEVKTGVDSITEVECFLTPEMGDPDEHFRGFSK  60
AEC15071.1      MAPTKRKGERKDPVQVPKLLIRGGVEVLEVKTGVDSITEVECFLTPEMGDPDEHFRGFSK  60
AEC15072.1      MAPTKRKGERKDPVQVPKLLIRGGVEVLEVKTGVDSITEVECFLTPEMGDPDEHFRGFSK  60
AEC15073.1      MAPTKRKGERKDPVQVPKLLIRGGVEVLEVKTGVDSITEVECFLTPEMGDPDEHFRGFSK  60
AEC15074.1      MAPTKRKGERKDPVQVPKLLIRGGVEVLEVKTGVDSITEVECFLTPEMGDPDEHFRGFSK  60
AEC15076.1      MAPTKRKGERKDPVQVPKLLIRGGVEVLEVKTGVDSITEVECFLTPEMGDPDEHFRGFSK  60
AEC15077.1      MAPTKRKGERKDPVQVPKLLIRGGVEVLEVKTGVDSITEVECFLTPEMGDPDEHFRGFSK  60
AEC15078.1      MAPTKRKGERKDPVQVPKLLIRGGVEVLEVKTGVDSITEVECFLTPEMGDPDEHFRGFSK  60
AEC15079.1      MAPTKRKGERKDPVQVPKLLIRGGVEVLEVKTGVDSITEVECFLTPEMGDPDEHFRGFSK  60
AEC15082.1      MAPTKRKGERKDPVQVPKLLIRGGVEVLEVKTGVDSITEVECFLTPEMGDPDEHFRGFSK  60
AEC15084.1      MAPTKRKGERKDPVQVPKLLIRGGVEVLEVKTGVDSITEVECFLTPEMGDPDEHFRGFSK  60
AEC15085.1      MAPTKRKGERKDPVQVPKLLIRGGVEVLEVKTGVDSITEVECFLTPEMGDPDEHFRGFSK  60
AEC15086.1      MAPTKRKGERKDPVQVPKLLIRGGVEVLEVKTGVDSITEVECFLTPEMGDPDEHFRGFSK  60
AEC15087.1      MAPTKRKGERKDPVQVPKLLIRGGVEVLEVKTGVDSITEVECFLTPEMGDPDEHFRGFSK  60
AEC15089.1      MAPTKRKGERKDPVQVPKLLIRGGVEVLEVKTGVDSITEVECFLTPEMGDPDEHFRGFSK  60
AEC15555.1      MAPTKRKGERKDPVQVPKLLIRGGVEVLEVKTGVDSITEVECFLTPEMGDPDEHFRGFSK  60
AEC15561.1      MAPTKRKGERKDPVQVPKLLIRGGVEVLEVKTGVDSITEVECFLTPEMGDPDEHFRGFSK  60
AEC15567.1      MAPTKRKGERKDPVQVPKLLIRGGVEVLEVKTGVDSITEVECFLTPEMGDPDEHFRGFSK  60
AEC15573.1      MAPTKRKGERKDPVQVPKLLIRGGVEVLEVKTGVDSITEVECFLTPEMGDPDEHFRGFSK  60
AEC15579.1      MAPTKRKGERKDPVQVPKLLIRGGVEVLEVKTGVDSITEVECFLTPEMGDPDEHFRGFSK  60
AEC15585.1      MAPTKRKGERKDPVQVPKLLIRGGVEVLEVKTGVDSITEVECFLTPEMGDPDEHFRGFSK  60
AEC15591.1      MAPTKRKGERKDPVQVPKLLIRGGVEVLEVKTGVDSITEVECFLTPEMGDPDEHFRGFSK  60
AEC15597.1      MAPTKRKGERKDPVQVPKLLIRGGVEVLEVKTGVDSITEVECFLTPEMGDPDEHFRGFSK  60
AEC15603.1      MAPTKRKGERKDPVQVPKLLIRGGVEVLEVKTGVDSITEVECFLTPEMGDPDEHFRGFSK  60
AEC15609.1      MAPTKRKGERKDPVQVPKLLIRGGVEVLEVKTGVDSITEVECFLTPEMGDPDEHFRGFSK  60
AEC15621.1      MAPTKRKGERKDPVQVPKLLIRGGVEVLEVKTGVDSITEVECFLTPEMGDPDEHFRGFSK  60
AEC15627.1      MAPTKRKGERKDPVQVPKLLIRGGVEVLEVKTGVDSITEVECFLTPEMGDPDEHFRGFSK  60
AEC15633.1      MAPTKRKGERKDPVQVPKLLIRGGVEVLEVKTGVDSITEVECFLTPEMGDPDEHFRGFSK  60
AEC15639.1      MAPTKRKGERKDPVQVPKLLIRGGVEVLEVKTGVDSITEVECFLTPEMGDPDEHFRGFSK  60
AEC15645.1      MAPTKRKGERKDPVQVPKLLIRGGVEVLEVKTGVDSITEVECFLTPEMGDPDEHFRGFSK  60
AEC15651.1      MAPTKRKGERKDPVQVPKLLIRGGVEVLEVKTGVDSITEVECFLTPEMGDPDEHFRGFSK  60
AEC15657.1      MAPTKRKGERKDPVQVPKLLIRGGVEVLEVKTGVDSITEVECFLTPEMGDPDEHFRGFSK  60
AEC15663.1      MAPTKRKGERKDPVQVPKLLIRGGVEVLEVKTGVDSITEVECFLTPEMGDPDEHFRGFSK  60
AEC15669.1      MAPTKRKGERKDPVQVPKLLIRGGVEVLEVKTGVDSITEVECFLTPEMGDPDEHFRGFSK  60
AEC15675.1      MAPTKRKGERKDPVQVPKLLIRGGVEVLEVKTGVDSITEVECFLTPEMGDPDEHFRGFSK  60
AEC15681.1      MAPTKRKGERKDPVQVPKLLIRGGVEVLEVKTGVDSITEVECFLTPEMGDPDEHFRGFSK  60
AEC15687.1      MAPTKRKGERKDPVQVPKLLIRGGVEVLEVKTGVDSITEVECFLTPEMGDPDEHFRGFSK  60
AEC15693.1      MAPTKRKGERKDPVQVPKLLIRGGVEVLEVKTGVDSITEVECFLTPEMGDPDEHFRGFSK  60
```

```
AEC15699.1        MAPTKRKGERKDPVQVPKLLIRGGVEVLEVKTGVDSITEVECFLTPEMGDPDEHFRGFSK  60
AEC15705.1        MAPTKRKGERKDPVQVPKLLIRGGVEVLEVKTGVDSITEVECFLTPEMGDPDEHFRGFSK  60
AEC15711.1        MAPTKRKGERKDPVQVPKLLIRGGVEVLEVKTGVDSITEVECFLTPEMGDPDEHFRGFSK  60
AEC15717.1        MAPTKRKGERKDPVQVPKLLIRGGVEVLEVKTGVDSITEVECFLTPEMGDPDEHFRGFSK  60
AEC15723.1        MAPTKRKGERKDPVQVPKLLIRGGVEVLEVKTGVDSITEVECFLTPEMGDPDEHFRGFSK  60
AEC15729.1        MAPTKRKGERKDPVQVPKLLIRGGVEVLEVKTGVDSITEVECFLTPEMGDPDEHFRGFSK  60
AEC15741.1        MAPTKRKGERKDPVQVPKLLIRGGVEVLEVKTGVDSITEVECFLTPEMGDPDEHFRGFSK  60
AEC15747.1        MAPTKRKGERKDPVQVPKLLIRGGVEVLEVKTGVDSITEVECFLTPEMGDPDEHFRGFSK  60
AEC15753.1        MAPTKRKGERKDPVQVPKLLIRGGVEVLEVKTGVDSITEVECFLTPEMGDPDEHFRGFSK  60
AEC15759.1        MAPTKRKGERKDPVQVPKLLIRGGVEVLEVKTGVDSITEVECFLTPEMGDPDEHFRGFSK  60
AEC15765.1        MAPTKRKGERKDPVQVPKLLIRGGVEVLEVKTGVDSITEVECFLTPEMGDPDEHFRGFSK  60
AEC15771.1        MAPTKRKGERKDPVQVPKLLIRGGVEVLEVKTGVDSITEVECFLTPEMGDPDEHFRGFSK  60
AEC15777.1        MAPTKRKGERKDPVQVPKLLIRGGVEVLEVKTGVDSITEVECFLTPEMGDPDEHFRGFSK  60
AEC15789.1        MAPTKRKGERKDPVQVPKLLIRGGVEVLEVKTGVDSITEVECFLTPEMGDPDEHFRGFSK  60
AEC15795.1        MAPTKRKGERKDPVQVPKLLIRGGVEVLEVKTGVDSITEVECFLTPEMGDPDEHFRGFSK  60
AEC15801.1        MAPTKRKGERKDPVQVPKLLIRGGVEVLEVKTGVDSITEVECFLTPEMGDPDEHFRGFSK  60
AEC15807.1        MAPTKRKGERKDPVQVPKLLIRGGVEVLEVKTGVDSITEVECFLTPEMGDPDEHFRGFSK  60
AEC15813.1        MAPTKRKGERKDPVQVPKLLIRGGVEVLEVKTGVDSITEVECFLTPEMGDPDEHFRGFSK  60
AEC15075.1        MAPTKRKGERKDPVQVPKLLIRGGVEVLEVKTGVDSITEVECFLTPEMGDPDEHFRGFSK  60
AEC15088.1        MAPTKRKGERKDPVQVPKLLIRGGVEVLEVKTGVDSITEVECFLTPEMGDPDEHFRGFSK  60
AEC14559.1        MAPTKRKGERKDPVQVPKLLIRGGVEVLEVKTGVDSITEVECFLTPEMGDPDEHFRGFSK  60
AEC15023.1        MAPTKRKGERKDPVQVPKLLIRGGVEVLEVKTGVDSITEVECFLTPEMGDPDEHLRGFSK  60
AEC14518.1        MAPTKRKGERKDPVQVPKLLIRGGVEVLEVKTGVDSITEVECFLTPEMGDPDEHFRGFSK  60
AEC14589.1        MAPTKRKGERKDPVQVPKLLIRGGVEVLEVKTGVDSITEVECFLTPEMGDPDEHFRGFSK  60
AEC14625.1        MAPTKRKGERKDPVQVPKLLIRGGVEVLEVKTGVDSITEVECFLTPEMGDPDEHFRGFSK  60
AEC14631.1        MAPTKRKGERKDPVQVPKLLIRGGVEVLEVKTGVDSITEVECFLTPEMGDPDEHFRGFSK  60
AEC14643.1        MAPTKRKGERKDPVQVPKLLIRGGVEVLEVKTGVDSITEVECFLTPEMGDPDEHFRGFSK  60
AEC14966.1        MAPTKRKGERKDPVQVPKLLIRGGVEVLEVKTGVDSITEVECFLTPEMGDPDEHFRGFSK  60
AEC14979.1        MAPTKRKGERKDPVQVPKLLIRGGVEVLEVKTGVDSITEVECFLTPEMGDPDEHFRGFSK  60
AEC14990.1        MAPTKRKGERKDPVQVPKLLIRGGVEVLEVKTGVDSITEVECFLTPEMGDPDEHFRGFSK  60
AEC14999.1        MAPTKRKGERKDPVQVPKLLIRGGVEVLEVKTGVDSITEVECFLTPEMGDPDEHFRGFSK  60
AEC15020.1        MAPTKRKGERKDPVQVPKLLIRGGVEVLEVKTGVDSITEVECFLTPEMGDPDEHFRGFSK  60
AEC14595.1        MAPTKRKGERKDPVQVPKLLIRGGVEVLEVKTGVDSITEVECFLTPEMGDPDEHFRGFSK  60
AEC14703.1        MAPTKRKGERKDPVQVPKLLIRGGVEVLEVKTGVDSITEVECFLTPEMGDPDEHFRGFSK  60
AEC15011.1        MAPTKRKGERKDPVQVPKLLIRGGVEVLEVKTGVDSITEVECFLTPEMGDPDEHFRGFSK  60
AEC15019.1        MAPTKRKGERKDPVQVPKLLIRGGVEVLEVKTGVDSITEVECFLTPEMGDPDEHFRGFSK  60
AEC15014.1        MAPTKRKGERKDPVQVPKLLIRGGVEVLEVKTGVDSITEVECFLTPEMGDPDEHFRGFSK  60
NP_043511.1       MAPTKRKGERKDPVQVPKLLIRGGVEVLEVKTGVDSITEVECFLTPEMGDPDEHLRGFSK  60
UPN63087.1        MAPTKRKGERKDPVQVPKLLIRGGVEVLEVKTGVDSITEVECFLTPEMGDPDEHLRGFSK  60
ASV51760.1        MAPTKRKGERKDPVQVPKLLIRGGVEVLEVKTGVDSITEVECFLTPEMGDPDEHLRGFSK  60
BAV69379.1        MAPTKRKGERKDPVQVPKLLIRGGVEVLEVKTGVDSITEVECFLTPEMGDPDEHLRGFSK  60
AEC15090.1        MAPTKRKGERKDPVQVPKLLIRGGVEVLEVKTGVDSITEVECFLTPEMGDPDEHLRGFSK  60
AEC15091.1        MAPTKRKGERKDPVQVPKLLIRGGVEVLEVKTGVDSITEVECFLTPEMGDPDEHLRGFSK  60
AEC15092.1        MAPTKRKGERKDPVQVPKLLIRGGVEVLEVKTGVDSITEVECFLTPEMGDPDEHLRGFSK  60
AEC15093.1        MAPTKRKGERKDPVQVPKLLIRGGVEVLEVKTGVDSITEVECFLTPEMGDPDEHLRGFSK  60
AEC15094.1        MAPTKRKGERKDPVQVPKLLIRGGVEVLEVKTGVDSITEVECFLTPEMGDPDEHLRGFSK  60
AEC15095.1        MAPTKRKGERKDPVQVPKLLIRGGVEVLEVKTGVDSITEVECFLTPEMGDPDEHLRGFSK  60
AEC15096.1        MAPTKRKGERKDPVQVPKLLIRGGVEVLEVKTGVDSITEVECFLTPEMGDPDEHLRGFSK  60
AEC15097.1        MAPTKRKGERKDPVQVPKLLIRGGVEVLEVKTGVDSITEVECFLTPEMGDPDEHLRGFSK  60
AEC15098.1        MAPTKRKGERKDPVQVPKLLIRGGVEVLEVKTGVDSITEVECFLTPEMGDPDEHLRGFSK  60
AEC15100.1        MAPTKRKGERKDPVQVPKLLIRGGVEVLEVKTGVDSITEVECFLTPEMGDPDEHLRGFSK  60
AEC15101.1        MAPTKRKGERKDPVQVPKLLIRGGVEVLEVKTGVDSITEVECFLTPEMGDPDEHLRGFSK  60
AEC15102.1        MAPTKRKGERKDPVQVPKLLIRGGVEVLEVKTGVDSITEVECFLTPEMGDPDEHLRGFSK  60
AEC15103.1        MAPTKRKGERKDPVQVPKLLIRGGVEVLEVKTGVDSITEVECFLTPEMGDPDEHLRGFSK  60
AEC15104.1        MAPTKRKGERKDPVQVPKLLIRGGVEVLEVKTGVDSITEVECFLTPEMGDPDEHLRGFSK  60
AEC15105.1        MAPTKRKGERKDPVQVPKLLIRGGVEVLEVKTGVDSITEVECFLTPEMGDPDEHLRGFSK  60
AEC15106.1        MAPTKRKGERKDPVQVPKLLIRGGVEVLEVKTGVDSITEVECFLTPEMGDPDEHLRGFSK  60
AEC15107.1        MAPTKRKGERKDPVQVPKLLIRGGVEVLEVKTGVDSITEVECFLTPEMGDPDEHLRGFSK  60
AEC15108.1        MAPTKRKGERKDPVQVPKLLIRGGVEVLEVKTGVDSITEVECFLTPEMGDPDEHLRGFSK  60
AEC15109.1        MAPTKRKGERKDPVQVPKLLIRGGVEVLEVKTGVDSITEVECFLTPEMGDPDEHLRGFSK  60
AEC15110.1        MAPTKRKGERKDPVQVPKLLIRGGVEVLEVKTGVDSITEVECFLTPEMGDPDEHLRGFSK  60
AEC15111.1        MAPTKRKGERKDPVQVPKLLIRGGVEVLEVKTGVDSITEVECFLTPEMGDPDEHLRGFSK  60
AEC15963.1        MAPTKRKGERKDPVQVPKLLIRGGVEVLEVKTGVDSITEVECFLTPEMGDPDEHLRGFSK  60
AEC15969.1        MAPTKRKGERKDPVQVPKLLIRGGVEVLEVKTGVDSITEVECFLTPEMGDPDEHLRGFSK  60
```

AEC15975.1	MAPTKRKGERKDPVQVPKLLIRGGVEVLEVKTGVDSITEVECFLTPEMGDPDEHLRGFSK	60
AEC15981.1	MAPTKRKGERKDPVQVPKLLIRGGVEVLEVKTGVDSITEVECFLTPEMGDPDEHLRGFSK	60
AEC15987.1	MAPTKRKGERKDPVQVPKLLIRGGVEVLEVKTGVDSITEVECFLTPEMGDPDEHLRGFSK	60
AEC15993.1	MAPTKRKGERKDPVQVPKLLIRGGVEVLEVKTGVDSITEVECFLTPEMGDPDEHLRGFSK	60
CAD69038.1	MAPTKRKGERKDPVQVPKLLIRGGVEVLEVKTGVDSITEVECFLTPEMGDPDEHLRGFSK	60
AAK70240.1	MAPTKRKGERKDPVQVPKLLIRGGVEVLEVKTGVDSITEVECFLTPEMGDPDEHLRGFSK	60
BAB11710.1	MAPTKRKGERKDPVQVPKLLIRGGVEVLEVKTGVDSITEVECFLTPEMGDPDEHLRGFSK	60
BAA01958.1	MAPTKRKGERKDPVQVPKLLIRGGVEVLEVKTGVDSITEVECFLTPEMGDPDEHLRGFSK	60
AAA82101.1	MAPTKRKGERKDPVQVPKLLIRGGVEVLEVKTGVDSITEVECFLTPEMGDPDEHLRGFSK	60
P03089.1	MAPTKRKGERKDPVQVPKLLIRGGVEVLEVKTGVDSITEVECFLTPEMGDPDEHLRGFSK	60
UPN63102.1	MAPTKRKGERKDPVQVPKLLIRGGVEVLEVKTGVDSITEVECFLTPEMGDPDEHLRGFSK	60
AEC15025.1	MAPTKRKGERKDPVQVPKLLIRGGVEVLEVKTGVDSITEVECFLTPEMGDPDEHLRGFSK	60
AEC15026.1	MAPTKRKGERKDPVQVPKLLIRGGVEVLEVKTGVDSITEVECFLTPEMGDPDEHLRGFSK	60
AEC15027.1	MAPTKRKGERKDPVQVPKLLIRGGVEVLEVKTGVDSITEVECFLTPEMGDPDEHLRGFSK	60
AEC15028.1	MAPTKRKGERKDPVQVPKLLIRGGVEVLEVKTGVDSITEVECFLTPEMGDPDEHLRGFSK	60
AEC15029.1	MAPTKRKGERKDPVQVPKLLIRGGVEVLEVKTGVDSITEVECFLTPEMGDPDEHLRGFSK	60
AEC15196.1	MAPTKRKGERKDPVQVPKLLIRGGVEVLEVKTGVDSITEVECFLTPEMGDPDEHLRGFSK	60
AEC15197.1	MAPTKRKGERKDPVQVPKLLIRGGVEVLEVKTGVDSITEVECFLTPEMGDPDEHLRGFSK	60
AEC15198.1	MAPTKRKGERKDPVQVPKLLIRGGVEVLEVKTGVDSITEVECFLTPEMGDPDEHLRGFSK	60
AEC15200.1	MAPTKRKGERKDPVQVPKLLIRGGVEVLEVKTGVDSITEVECFLTPEMGDPDEHLRGFSK	60
AEC15201.1	MAPTKRKGERKDPVQVPKLLIRGGVEVLEVKTGVDSITEVECFLTPEMGDPDEHLRGFSK	60
AEC15202.1	MAPTKRKGERKDPVQVPKLLIRGGVEVLEVKTGVDSITEVECFLTPEMGDPDEHLRGFSK	60
AEC15203.1	MAPTKRKGERKDPVQVPKLLIRGGVEVLEVKTGVDSITEVECFLTPEMGDPDEHLRGFSK	60
AEC15204.1	MAPTKRKGERKDPVQVPKLLIRGGVEVLEVKTGVDSITEVECFLTPEMGDPDEHLRGFSK	60
AEC15205.1	MAPTKRKGERKDPVQVPKLLIRGGVEVLEVKTGVDSITEVECFLTPEMGDPDEHLRGFSK	60
AEC15206.1	MAPTKRKGERKDPVQVPKLLIRGGVEVLEVKTGVDSITEVECFLTPEMGDPDEHLRGFSK	60
AEC15207.1	MAPTKRKGERKDPVQVPKLLIRGGVEVLEVKTGVDSITEVECFLTPEMGDPDEHLRGFSK	60
AEC15208.1	MAPTKRKGERKDPVQVPKLLIRGGVEVLEVKTGVDSITEVECFLTPEMGDPDEHLRGFSK	60
AEC15210.1	MAPTKRKGERKDPVQVPKLLIRGGVEVLEVKTGVDSITEVECFLTPEMGDPDEHLRGFSK	60
AEC15211.1	MAPTKRKGERKDPVQVPKLLIRGGVEVLEVKTGVDSITEVECFLTPEMGDPDEHLRGFSK	60
AEC15212.1	MAPTKRKGERKDPVQVPKLLIRGGVEVLEVKTGVDSITEVECFLTPEMGDPDEHLRGFSK	60
AEC15213.1	MAPTKRKGERKDPVQVPKLLIRGGVEVLEVKTGVDSITEVECFLTPEMGDPDEHLRGFSK	60
AEC15214.1	MAPTKRKGERKDPVQVPKLLIRGGVEVLEVKTGVDSITEVECFLTPEMGDPDEHLRGFSK	60
AEC15215.1	MAPTKRKGERKDPVQVPKLLIRGGVEVLEVKTGVDSITEVECFLTPEMGDPDEHLRGFSK	60
AEC15216.1	MAPTKRKGERKDPVQVPKLLIRGGVEVLEVKTGVDSITEVECFLTPEMGDPDEHLRGFSK	60
AEC15217.1	MAPTKRKGERKDPVQVPKLLIRGGVEVLEVKTGVDSITEVECFLTPEMGDPDEHLRGFSK	60
AEC15218.1	MAPTKRKGERKDPVQVPKLLIRGGVEVLEVKTGVDSITEVECFLTPEMGDPDEHLRGFSK	60
AEC15327.1	MAPTKRKGERKDPVQVPKLLIRGGVEVLEVKTGVDSITEVECFLTPEMGDPDEHLRGFSK	60
AEC15333.1	MAPTKRKGERKDPVQVPKLLIRGGVEVLEVKTGVDSITEVECFLTPEMGDPDEHLRGFSK	60
AEC15339.1	MAPTKRKGERKDPVQVPKLLIRGGVEVLEVKTGVDSITEVECFLTPEMGDPDEHLRGFSK	60
AEC15345.1	MAPTKRKGERKDPVQVPKLLIRGGVEVLEVKTGVDSITEVECFLTPEMGDPDEHLRGFSK	60
AEC15351.1	MAPTKRKGERKDPVQVPKLLIRGGVEVLEVKTGVDSITEVECFLTPEMGDPDEHLRGFSK	60
AEC15357.1	MAPTKRKGERKDPVQVPKLLIRGGVEVLEVKTGVDSITEVECFLTPEMGDPDEHLRGFSK	60
AEC15363.1	MAPTKRKGERKDPVQVPKLLIRGGVEVLEVKTGVDSITEVECFLTPEMGDPDEHLRGFSK	60
AEC15369.1	MAPTKRKGERKDPVQVPKLLIRGGVEVLEVKTGVDSITEVECFLTPEMGDPDEHLRGFSK	60
AEC15375.1	MAPTKRKGERKDPVQVPKLLIRGGVEVLEVKTGVDSITEVECFLTPEMGDPDEHLRGFSK	60
AEC15381.1	MAPTKRKGERKDPVQVPKLLIRGGVEVLEVKTGVDSITEVECFLTPEMGDPDEHLRGFSK	60
AEC15387.1	MAPTKRKGERKDPVQVPKLLIRGGVEVLEVKTGVDSITEVECFLTPEMGDPDEHLRGFSK	60
AEC15393.1	MAPTKRKGERKDPVQVPKLLIRGGVEVLEVKTGVDSITEVECFLTPEMGDPDEHLRGFSK	60
AEC15399.1	MAPTKRKGERKDPVQVPKLLIRGGVEVLEVKTGVDSITEVECFLTPEMGDPDEHLRGFSK	60
AEC15405.1	MAPTKRKGERKDPVQVPKLLIRGGVEVLEVKTGVDSITEVECFLTPEMGDPDEHLRGFSK	60
AEC15411.1	MAPTKRKGERKDPVQVPKLLIRGGVEVLEVKTGVDSITEVECFLTPEMGDPDEHLRGFSK	60
AEC15417.1	MAPTKRKGERKDPVQVPKLLIRGGVEVLEVKTGVDSITEVECFLTPEMGDPDEHLRGFSK	60
AEC15423.1	MAPTKRKGERKDPVQVPKLLIRGGVEVLEVKTGVDSITEVECFLTPEMGDPDEHLRGFSK	60
AEC15429.1	MAPTKRKGERKDPVQVPKLLIRGGVEVLEVKTGVDSITEVECFLTPEMGDPDEHLRGFSK	60
AEC15435.1	MAPTKRKGERKDPVQVPKLLIRGGVEVLEVKTGVDSITEVECFLTPEMGDPDEHLRGFSK	60
AEC15441.1	MAPTKRKGERKDPVQVPKLLIRGGVEVLEVKTGVDSITEVECFLTPEMGDPDEHLRGFSK	60
BAA01967.1	MAPTKRKGERKDPVQVPKLLIRGGVEVLEVKTGVDSITEVECFLTPEMGDPDEHLRGFSK	60
ASV51844.1	MAPTKRKGERKDPVQVPKLLIRGGVEVLEVKTGVDSITEVECFLTPEMGDPDEHLRGFSK	60
ASV51850.1	MAPTKRKGERKDPVQVPKLLIRGGVEVLEVKTGVDSITEVECFLTPEMGDPDEHLRGFSK	60
CCW72489.1	MAPTKRKGERKDPVQVPKLLIRGGVEVLEVKTGVDSITEVECFLTPEMGDPNEHLRGFSK	60
AEC15170.1	MAPTKRKGERKDPVQVPKLLIRGGVEVLEVKTGVDSITEVECFLTPEMGDPDEHLRGFSK	60
AEC15171.1	MAPTKRKGERKDPVQVPKLLIRGGVEVLEVKTGVDSITEVECFLTPEMGDPDEHLRGFSK	60
AEC15299.1	MAPTKRKGERKDPVQVPKLLIRGGVEVLEVKTGVDSITEVECFLTPEMGDPDEHLRGFSK	60

BAB93050.1 MAPTKRKGERKDPVQVPKLLIRGGVEVLEVKTGVDSITEVECFLTPEMGDPDEHLRGFSK 60
UPN63100.1 MAPTKRKGERKDPVQVPKLLIRGGVEVLEVKTGVDSITEVECFLTPEMGDPDEHLRGFSK 60
CCW72482.1 MAPTKRKGERKDPVQVPKLLIRGGVEVLEVKTGVDSITEVECFLTPEMGDPDEHLRGFSK 60
AEC15165.1 MAPTKRKGERKDPVQVPKLLIRGGVEVLEVKTGVDSITEVECFLTPEMGDPDEHLRGFSK 60
AEC15166.1 MAPTKRKGERKDPVQVPKLLIRGGVEVLEVKTGVDSITEVECFLTPEMGDPDEHLRGFSK 60
AEC15266.1 MAPTKRKGERKDPVQVPKLLIRGGVEVLEVKTGVDSITEVECFLTPEMGDPDEHLRGFSK 60
AEC15267.1 MAPTKRKGERKDPVQVPKLLIRGGVEVLEVKTGVDSITEVECFLTPEMGDPDEHLRGFSK 60
AEC15268.1 MAPTKRKGERKDPVQVPKLLIRGGVEVLEVKTGVDSITEVECFLTPEMGDPDEHLRGFSK 60
AEC15269.1 MAPTKRKGERKDPVQVPKLLIRGGVEVLEVKTGVDSITEVECFLTPEMGDPDEHLRGFSK 60
AEC15270.1 MAPTKRKGERKDPVQVPKLLIRGGVEVLEVKTGVDSITEVECFLTPEMGDPDEHLRGFSK 60
AEC15271.1 MAPTKRKGERKDPVQVPKLLIRGGVEVLEVKTGVDSITEVECFLTPEMGDPDEHLRGFSK 60
AEC15272.1 MAPTKRKGERKDPVQVPKLLIRGGVEVLEVKTGVDSITEVECFLTPEMGDPDEHLRGFSK 60
AEC15273.1 MAPTKRKGERKDPVQVPKLLIRGGVEVLEVKTGVDSITEVECFLTPEMGDPDEHLRGFSK 60
AEC15274.1 MAPTKRKGERKDPVQVPKLLIRGGVEVLEVKTGVDSITEVECFLTPEMGDPDEHLRGFSK 60
AEC15275.1 MAPTKRKGERKDPVQVPKLLIRGGVEVLEVKTGVDSITEVECFLTPEMGDPDEHLRGFSK 60
AEC15276.1 MAPTKRKGERKDPVQVPKLLIRGGVEVLEVKTGVDSITEVECFLTPEMGDPDEHLRGFSK 60
AEC15277.1 MAPTKRKGERKDPVQVPKLLIRGGVEVLEVKTGVDSITEVECFLTPEMGDPDEHLRGFSK 60
AEC15278.1 MAPTKRKGERKDPVQVPKLLIRGGVEVLEVKTGVDSITEVECFLTPEMGDPDEHLRGFSK 60
AEC15279.1 MAPTKRKGERKDPVQVPKLLIRGGVEVLEVKTGVDSITEVECFLTPEMGDPDEHLRGFSK 60
AEC15280.1 MAPTKRKGERKDPVQVPKLLIRGGVEVLEVKTGVDSITEVECFLTPEMGDPDEHLRGFSK 60
AEC15281.1 MAPTKRKGERKDPVQVPKLLIRGGVEVLEVKTGVDSITEVECFLTPEMGDPDEHLRGFSK 60
AEC15282.1 MAPTKRKGERKDPVQVPKLLIRGGVEVLEVKTGVDSITEVECFLTPEMGDPDEHLRGFSK 60
AEC15283.1 MAPTKRKGERKDPVQVPKLLIRGGVEVLEVKTGVDSITEVECFLTPEMGDPDEHLRGFSK 60
AEC15284.1 MAPTKRKGERKDPVQVPKLLIRGGVEVLEVKTGVDSITEVECFLTPEMGDPDEHLRGFSK 60
AEC15285.1 MAPTKRKGERKDPVQVPKLLIRGGVEVLEVKTGVDSITEVECFLTPEMGDPDEHLRGFSK 60
AEC15286.1 MAPTKRKGERKDPVQVPKLLIRGGVEVLEVKTGVDSITEVECFLTPEMGDPDEHLRGFSK 60
AEC15287.1 MAPTKRKGERKDPVQVPKLLIRGGVEVLEVKTGVDSITEVECFLTPEMGDPDEHLRGFSK 60
AEC15288.1 MAPTKRKGERKDPVQVPKLLIRGGVEVLEVKTGVDSITEVECFLTPEMGDPDEHLRGFSK 60
AEC15289.1 MAPTKRKGERKDPVQVPKLLIRGGVEVLEVKTGVDSITEVECFLTPEMGDPDEHLRGFSK 60
AEC15290.1 MAPTKRKGERKDPVQVPKLLIRGGVEVLEVKTGVDSITEVECFLTPEMGDPDEHLRGFSK 60
AEC15291.1 MAPTKRKGERKDPVQVPKLLIRGGVEVLEVKTGVDSITEVECFLTPEMGDPDEHLRGFSK 60
AEC15292.1 MAPTKRKGERKDPVQVPKLLIRGGVEVLEVKTGVDSITEVECFLTPEMGDPDEHLRGFSK 60
AEC15293.1 MAPTKRKGERKDPVQVPKLLIRGGVEVLEVKTGVDSITEVECFLTPEMGDPDEHLRGFSK 60
AEC15294.1 MAPTKRKGERKDPVQVPKLLIRGGVEVLEVKTGVDSITEVECFLTPEMGDPDEHLRGFSK 60
AEC15295.1 MAPTKRKGERKDPVQVPKLLIRGGVEVLEVKTGVDSITEVECFLTPEMGDPDEHLRGFSK 60
AEC15296.1 MAPTKRKGERKDPVQVPKLLIRGGVEVLEVKTGVDSITEVECFLTPEMGDPDEHLRGFSK 60
AEC15297.1 MAPTKRKGERKDPVQVPKLLIRGGVEVLEVKTGVDSITEVECFLTPEMGDPDEHLRGFSK 60
AEC15298.1 MAPTKRKGERKDPVQVPKLLIRGGVEVLEVKTGVDSITEVECFLTPEMGDPDEHLRGFSK 60
AEC15300.1 MAPTKRKGERKDPVQVPKLLIRGGVEVLEVKTGVDSITEVECFLTPEMGDPDEHLRGFSK 60
AEC15301.1 MAPTKRKGERKDPVQVPKLLIRGGVEVLEVKTGVDSITEVECFLTPEMGDPDEHLRGFSK 60
AEC15302.1 MAPTKRKGERKDPVQVPKLLIRGGVEVLEVKTGVDSITEVECFLTPEMGDPDEHLRGFSK 60
AEC15303.1 MAPTKRKGERKDPVQVPKLLIRGGVEVLEVKTGVDSITEVECFLTPEMGDPDEHLRGFSK 60
AEC15304.1 MAPTKRKGERKDPVQVPKLLIRGGVEVLEVKTGVDSITEVECFLTPEMGDPDEHLRGFSK 60
AEC15305.1 MAPTKRKGERKDPVQVPKLLIRGGVEVLEVKTGVDSITEVECFLTPEMGDPDEHLRGFSK 60
AEC15306.1 MAPTKRKGERKDPVQVPKLLIRGGVEVLEVKTGVDSITEVECFLTPEMGDPDEHLRGFSK 60
AEC15307.1 MAPTKRKGERKDPVQVPKLLIRGGVEVLEVKTGVDSITEVECFLTPEMGDPDEHLRGFSK 60
AEC15308.1 MAPTKRKGERKDPVQVPKLLIRGGVEVLEVKTGVDSITEVECFLTPEMGDPDEHLRGFSK 60
AEC15309.1 MAPTKRKGERKDPVQVPKLLIRGGVEVLEVKTGVDSITEVECFLTPEMGDPDEHLRGFSK 60
AEC15310.1 MAPTKRKGERKDPVQVPKLLIRGGVEVLEVKTGVDSITEVECFLTPEMGDPDEHLRGFSK 60
AEC15311.1 MAPTKRKGERKDPVQVPKLLIRGGVEVLEVKTGVDSITEVECFLTPEMGDPDEHLRGFSK 60
AEC15861.1 MAPTKRKGERKDPVQVPKLLIRGGVEVLEVKTGVDSITEVECFLTPEMGDPDEHLRGFSK 60
AEC15867.1 MAPTKRKGERKDPVQVPKLLIRGGVEVLEVKTGVDSITEVECFLTPEMGDPDEHLRGFSK 60
AEC15873.1 MAPTKRKGERKDPVQVPKLLIRGGVEVLEVKTGVDSITEVECFLTPEMGDPDEHLRGFSK 60
AEC15879.1 MAPTKRKGERKDPVQVPKLLIRGGVEVLEVKTGVDSITEVECFLTPEMGDPDEHLRGFSK 60
AEC15885.1 MAPTKRKGERKDPVQVPKLLIRGGVEVLEVKTGVDSITEVECFLTPEMGDPDEHLRGFSK 60
AEC15891.1 MAPTKRKGERKDPVQVPKLLIRGGVEVLEVKTGVDSITEVECFLTPEMGDPDEHLRGFSK 60
AEC15897.1 MAPTKRKGERKDPVQVPKLLIRGGVEVLEVKTGVDSITEVECFLTPEMGDPDEHLRGFSK 60
AEC15903.1 MAPTKRKGERKDPVQVPKLLIRGGVEVLEVKTGVDSITEVECFLTPEMGDPDEHLRGFSK 60
AEC15909.1 MAPTKRKGERKDPVQVPKLLIRGGVEVLEVKTGVDSITEVECFLTPEMGDPDEHLRGFSK 60
AEC15915.1 MAPTKRKGERKDPVQVPKLLIRGGVEVLEVKTGVDSITEVECFLTPEMGDPDEHLRGFSK 60
AEC15921.1 MAPTKRKGERKDPVQVPKLLIRGGVEVLEVKTGVDSITEVECFLTPEMGDPDEHLRGFSK 60
AEC15927.1 MAPTKRKGERKDPVQVPKLLIRGGVEVLEVKTGVDSITEVECFLTPEMGDPDEHLRGFSK 60
AEC15933.1 MAPTKRKGERKDPVQVPKLLIRGGVEVLEVKTGVDSITEVECFLTPEMGDPDEHLRGFSK 60
AEC15939.1 MAPTKRKGERKDPVQVPKLLIRGGVEVLEVKTGVDSITEVECFLTPEMGDPDEHLRGFSK 60

AEC15945.1	MAPTKRKGERKDPVQVPKLLIRGGVEVLEVKTGVDSITEVECFLTPEMGDPDEHLRGFSK	60
AEC15951.1	MAPTKRKGERKDPVQVPKLLIRGGVEVLEVKTGVDSITEVECFLTPEMGDPDEHLRGFSK	60
AEC15957.1	MAPTKRKGERKDPVQVPKLLIRGGVEVLEVKTGVDSITEVECFLTPEMGDPDEHLRGFSK	60
BAD21259.1	MAPTKRKGERKDPVQVPKLLIRGGVEVLEVKTGVDSITEVECFLTPEMGDPDEHLRGFSK	60
BAD21265.1	MAPTKRKGERKDPVQVPKLLIRGGVEVLEVKTGVDSITEVECFLTPEMGDPDEHLRGFSK	60
AAC59325.1	MAPTKRKGERKDPVQVPKLLIRGGVEVLEVKTGVDSITEVECFLTPEMGDPDEHLRGFSK	60
AEC14524.1	MAPTKRKGERKDPVQVPKLLIRGGVEVLEVKTGVDSITEVECFLTPEMGDPDEHFRGFSK	60
AEC14541.1	MAPTKRKGERKDPVQVPKLLIRGGVEVLEVKTGVDSITEVECFLTPEMGDPDEHFRGFSK	60
AEC14565.1	MAPTKRKGERKDPVQVPKLLIRGGVEVLEVKTGVDSITEVECFLTPEMGDPDEHFRGFSK	60
AEC14571.1	MAPTKRKGERKDPVQVPKLLIRGGVEVLEVKTGVDSITEVECFLTPEMGDPDEHFRGFSK	60
AEC14583.1	MAPTKRKGERKDPVQVPKLLIRGGVEVLEVKTGVDSITEVECFLTPEMGDPDEHFRGFSK	60
AEC14601.1	MAPTKRKGERKDPVQVPKLLIRGGVEVLEVKTGVDSITEVECFLTPEMGDPDEHFRGFSK	60
AEC14607.1	MAPTKRKGERKDPVQVPKLLIRGGVEVLEVKTGVDSITEVECFLTPEMGDPDEHFRGFSK	60
AEC14637.1	MAPTKRKGERKDPVQVPKLLIRGGVEVLEVKTGVDSITEVECFLTPEMGDPDEHFRGFSK	60
AEC14649.1	MAPTKRKGERKDPVQVPKLLIRGGVEVLEVKTGVDSITEVECFLTPEMGDPDEHFRGFSK	60
AEC14655.1	MAPTKRKGERKDPVQVPKLLIRGGVEVLEVKTGVDSITEVECFLTPEMGDPDEHFRGFSK	60
AEC14661.1	MAPTKRKGERKDPVQVPKLLIRGGVEVLEVKTGVDSITEVECFLTPEMGDPDEHFRGFSK	60
AEC14685.1	MAPTKRKGERKDPVQVPKLLIRGGVEVLEVKTGVDSITEVECFLTPEMGDPDEHFRGFSK	60
AEC14697.1	MAPTKRKGERKDPVQVPKLLIRGGVEVLEVKTGVDSITEVECFLTPEMGDPDEHFRGFSK	60
AEC14709.1	MAPTKRKGERKDPVQVPKLLIRGGVEVLEVKTGVDSITEVECFLTPEMGDPDEHFRGFSK	60
AEC14715.1	MAPTKRKGERKDPVQVPKLLIRGGVEVLEVKTGVDSITEVECFLTPEMGDPDEHFRGFSK	60
AEC14963.1	MAPTKRKGERKDPVQVPKLLIRGGVEVLEVKTGVDSITEVECFLTPEMGDPDEHFRGFSK	60
AEC14968.1	MAPTKRKGERKDPVQVPKLLIRGGVEVLEVKTGVDSITEVECFLTPEMGDPDEHFRGFSK	60
AEC14971.1	MAPTKRKGERKDPVQVPKLLIRGGVEVLEVKTGVDSITEVECFLTPEMGDPDEHFRGFSK	60
AEC14972.1	MAPTKRKGERKDPVQVPKLLIRGGVEVLEVKTGVDSITEVECFLTPEMGDPDEHFRGFSK	60
AEC14975.1	MAPTKRKGERKDPVQVPKLLIRGGVEVLEVKTGVDSITEVECFLTPEMGDPDEHFRGFSK	60
AEC14976.1	MAPTKRKGERKDPVQVPKLLIRGGVEVLEVKTGVDSITEVECFLTPEMGDPDEHFRGFSK	60
AEC14977.1	MAPTKRKGERKDPVQVPKLLIRGGVEVLEVKTGVDSITEVECFLTPEMGDPDEHFRGFSK	60
AEC14978.1	MAPTKRKGERKDPVQVPKLLIRGGVEVLEVKTGVDSITEVECFLTPEMGDPDEHFRGFSK	60
AEC14980.1	MAPTKRKGERKDPVQVPKLLIRGGVEVLEVKTGVDSITEVECFLTPEMGDPDEHFRGFSK	60
AEC14981.1	MAPTKRKGERKDPVQVPKLLIRGGVEVLEVKTGVDSITEVECFLTPEMGDPDEHFRGFSK	60
AEC14982.1	MAPTKRKGERKDPVQVPKLLIRGGVEVLEVKTGVDSITEVECFLTPEMGDPDEHFRGFSK	60
AEC14983.1	MAPTKRKGERKDPVQVPKLLIRGGVEVLEVKTGVDSITEVECFLTPEMGDPDEHFRGFSK	60
AEC14985.1	MAPTKRKGERKDPVQVPKLLIRGGVEVLEVKTGVDSITEVECFLTPEMGDPDEHFRGFSK	60
AEC14986.1	MAPTKRKGERKDPVQVPKLLIRGGVEVLEVKTGVDSITEVECFLTPEMGDPDEHFRGFSK	60
AEC14988.1	MAPTKRKGERKDPVQVPKLLIRGGVEVLEVKTGVDSITEVECFLTPEMGDPDEHFRGFSK	60
AEC14989.1	MAPTKRKGERKDPVQVPKLLIRGGVEVLEVKTGVDSITEVECFLTPEMGDPDEHFRGFSK	60
AEC14991.1	MAPTKRKGERKDPVQVPKLLIRGGVEVLEVKTGVDSITEVECFLTPEMGDPDEHFRGFSK	60
AEC14992.1	MAPTKRKGERKDPVQVPKLLIRGGVEVLEVKTGVDSITEVECFLTPEMGDPDEHFRGFSK	60
AEC14996.1	MAPTKRKGERKDPVQVPKLLIRGGVEVLEVKTGVDSITEVECFLTPEMGDPDEHFRGFSK	60
AEC14997.1	MAPTKRKGERKDPVQVPKLLIRGGVEVLEVKTGVDSITEVECFLTPEMGDPDEHFRGFSK	60
AEC14998.1	MAPTKRKGERKDPVQVPKLLIRGGVEVLEVKTGVDSITEVECFLTPEMGDPDEHFRGFSK	60
AEC15002.1	MAPTKRKGERKDPVQVPKLLIRGGVEVLEVKTGVDSITEVECFLTPEMGDPDEHFRGFSK	60
AEC15003.1	MAPTKRKGERKDPVQVPKLLIRGGVEVLEVKTGVDSITEVECFLTPEMGDPDEHFRGFSK	60
AEC15004.1	MAPTKRKGERKDPVQVPKLLIRGGVEVLEVKTGVDSITEVECFLTPEMGDPDEHFRGFSK	60
AEC15005.1	MAPTKRKGERKDPVQVPKLLIRGGVEVLEVKTGVDSITEVECFLTPEMGDPDEHFRGFSK	60
AEC15006.1	MAPTKRKGERKDPVQVPKLLIRGGVEVLEVKTGVDSITEVECFLTPEMGDPDEHFRGFSK	60
AEC15009.1	MAPTKRKGERKDPVQVPKLLIRGGVEVLEVKTGVDSITEVECFLTPEMGDPDEHFRGFSK	60
AEC15012.1	MAPTKRKGERKDPVQVPKLLIRGGVEVLEVKTGVDSITEVECFLTPEMGDPDEHFRGFSK	60
AEC15013.1	MAPTKRKGERKDPVQVPKLLIRGGVEVLEVKTGVDSITEVECFLTPEMGDPDEHFRGFSK	60
AEC15015.1	MAPTKRKGERKDPVQVPKLLIRGGVEVLEVKTGVDSITEVECFLTPEMGDPDEHFRGFSK	60
AEC15017.1	MAPTKRKGERKDPVQVPKLLIRGGVEVLEVKTGVDSITEVECFLTPEMGDPDEHFRGFSK	60
AEC15018.1	MAPTKRKGERKDPVQVPKLLIRGGVEVLEVKTGVDSITEVECFLTPEMGDPDEHFRGFSK	60
AEC15021.1	MAPTKRKGERKDPVQVPKLLIRGGVEVLEVKTGVDSITEVECFLTPEMGDPDEHFRGFSK	60
AEC15022.1	MAPTKRKGERKDPVQVPKLLIRGGVEVLEVKTGVDSITEVECFLTPEMGDPDEHFRGFSK	60
AEC14962.1	MAPTKRKGERKDPVQVPKLLIRGGVEVLEVKTGVDSITEVECFLTPEMGDPDEHXRGFSK	60
AEC14993.1	MAPTKRKGERKDPVQVPKLLIRGGVEVLEVKTGVDSITEVECFLTPEMGDPDEHXRGFSK	60
AEC15007.1	MAPTKRKGERKDPVQVPKLLIRGGVEVLEVKTGVDSITEVECFLTPEMGDPDEHXRGFSK	60
CCW72517.1	MAPTKRKAERKDPVQVPKLLIRGGVEVLEVKTGVDSITEVECFLTPEMGDPDEHLRGFSK	60
BAD21247.2	MAPTKRKAERKDPVQVPKLLIRGGVEVLEVKTGVDSITEVECFLTPEMGDPDEHLRGFSK	60
AAL37695.1	MAPTKRKGERKDPVQVPKLLIRGGVEVLEVKTGVDSITEVECFLTPEMGDPDEHLRGFSK	60
AEC15242.1	MAPTKRKGERKDPVQVPKLLIRGGVEVLEVKTGVDSITEVECFLTPEMGDPDEHLRGFSK	60
AEC15243.1	MAPTKRKGERKDPVQVPKLLIRGGVEVLEVKTGVDSITEVECFLTPEMGDPDEHLRGFSK	60
AEC15244.1	MAPTKRKGERKDPVQVPKLLIRGGVEVLEVKTGVDSITEVECFLTPEMGDPDEHLRGFSK	60

```
AEC15245.1      MAPTKRKGERKDPVQVPKLLIRGGVEVLEVKTGVDSITEVECFLTPEMGDPDEHLRGFSK  60
AEC15246.1      MAPTKRKGERKDPVQVPKLLIRGGVEVLEVKTGVDSITEVECFLTPEMGDPDEHLRGFSK  60
AEC15247.1      MAPTKRKGERKDPVQVPKLLIRGGVEVLEVKTGVDSITEVECFLTPEMGDPDEHLRGFSK  60
AEC15248.1      MAPTKRKGERKDPVQVPKLLIRGGVEVLEVKTGVDSITEVECFLTPEMGDPDEHLRGFSK  60
AEC15249.1      MAPTKRKGERKDPVQVPKLLIRGGVEVLEVKTGVDSITEVECFLTPEMGDPDEHLRGFSK  60
AEC15250.1      MAPTKRKGERKDPVQVPKLLIRGGVEVLEVKTGVDSITEVECFLTPEMGDPDEHLRGFSK  60
AEC15251.1      MAPTKRKGERKDPVQVPKLLIRGGVEVLEVKTGVDSITEVECFLTPEMGDPDEHLRGFSK  60
AEC15252.1      MAPTKRKGERKDPVQVPKLLIRGGVEVLEVKTGVDSITEVECFLTPEMGDPDEHLRGFSK  60
AEC15253.1      MAPTKRKGERKDPVQVPKLLIRGGVEVLEVKTGVDSITEVECFLTPEMGDPDEHLRGFSK  60
AEC15254.1      MAPTKRKGERKDPVQVPKLLIRGGVEVLEVKTGVDSITEVECFLTPEMGDPDEHLRGFSK  60
AEC15255.1      MAPTKRKGERKDPVQVPKLLIRGGVEVLEVKTGVDSITEVECFLTPEMGDPDEHLRGFSK  60
AEC15256.1      MAPTKRKGERKDPVQVPKLLIRGGVEVLEVKTGVDSITEVECFLTPEMGDPDEHLRGFSK  60
AEC15257.1      MAPTKRKGERKDPVQVPKLLIRGGVEVLEVKTGVDSITEVECFLTPEMGDPDEHLRGFSK  60
AEC15258.1      MAPTKRKGERKDPVQVPKLLIRGGVEVLEVKTGVDSITEVECFLTPEMGDPDEHLRGFSK  60
AEC15259.1      MAPTKRKGERKDPVQVPKLLIRGGVEVLEVKTGVDSITEVECFLTPEMGDPDEHLRGFSK  60
AEC15260.1      MAPTKRKGERKDPVQVPKLLIRGGVEVLEVKTGVDSITEVECFLTPEMGDPDEHLRGFSK  60
AEC15261.1      MAPTKRKGERKDPVQVPKLLIRGGVEVLEVKTGVDSITEVECFLTPEMGDPDEHLRGFSK  60
AEC15262.1      MAPTKRKGERKDPVQVPKLLIRGGVEVLEVKTGVDSITEVECFLTPEMGDPDEHLRGFSK  60
AEC15263.1      MAPTKRKGERKDPVQVPKLLIRGGVEVLEVKTGVDSITEVECFLTPEMGDPDEHLRGFSK  60
AEC15264.1      MAPTKRKGERKDPVQVPKLLIRGGVEVLEVKTGVDSITEVECFLTPEMGDPDEHLRGFSK  60
AEC15265.1      MAPTKRKGERKDPVQVPKLLIRGGVEVLEVKTGVDSITEVECFLTPEMGDPDEHLRGFSK  60
AEC15000.1      MAPTKRKGERKDPVQVPKLLIRGGVEVLEVKTGVDSITEVECFLTPEMGDPDEHLRGFSK  60
AEC14721.1      MAPTKRKGERKDPVQVPKLLIRGGVEVLEVKTGVDSITEVECFLTPEMGDPDEHLRGFSK  60
AEC14965.1      MAPTKRKGERKDPVQVPKLLIRGGVEVLEVKTGVDSITEVECFLTPEMGDPDEHLRGFSK  60
AEC14967.1      MAPTKRKGERKDPVQVPKLLIRGGVEVLEVKTGVDSITEVECFLTPEMGDPDEHLRGFSK  60
AEC14969.1      MAPTKRKGERKDPVQVPKLLIRGGVEVLEVKTGVDSITEVECFLTPEMGDPDEHLRGFSK  60
AEC14973.1      MAPTKRKGERKDPVQVPKLLIRGGVEVLEVKTGVDSITEVECFLTPEMGDPDEHLRGFSK  60
AEC14974.1      MAPTKRKGERKDPVQVPKLLIRGGVEVLEVKTGVDSITEVECFLTPEMGDPDEHLRGFSK  60
AEC14994.1      MAPTKRKGERKDPVQVPKLLIRGGVEVLEVKTGVDSITEVECFLTPEMGDPDEHLRGFSK  60
AEC15001.1      MAPTKRKGERKDPVQVPKLLIRGGVEVLEVKTGVDSITEVECFLTPEMGDPDEHLRGFSK  60
AEC15024.1      MAPTKRKGERKDPVQVPKLLIRGGVEVLEVKTGVDSITEVECFLTPEMGDPDEHLRGFSK  60
AEC14667.1      MAPTKRKGERKDPVQVPKLLIRGGVEVLEVKTGVDSITEVECFLTPEMGDPDEHLRGFSK  60
UPN63091.1      MAPTKRKGERKDPVQVPKLLIRGGVEVLEVKTGVDSITEVECFLTPEMGDPDEHLRGFSK  60
AEC14535.1      MAPTKRKGERKDPVQVPKLLIRGGVEVLEVKTGVDSITEVECFLTPEMGDPDEHLRGFSK  60
AEC14547.1      MAPTKRKGERKDPVQVPKLLIRGGVEVLEVKTGVDSITEVECFLTPEMGDPDEHLRGFSK  60
AEC14553.1      MAPTKRKGERKDPVQVPKLLIRGGVEVLEVKTGVDSITEVECFLTPEMGDPDEHLRGFSK  60
AEC14577.1      MAPTKRKGERKDPVQVPKLLIRGGVEVLEVKTGVDSITEVECFLTPEMGDPDEHLRGFSK  60
AEC14613.1      MAPTKRKGERKDPVQVPKLLIRGGVEVLEVKTGVDSITEVECFLTPEMGDPDEHLRGFSK  60
AEC14673.1      MAPTKRKGERKDPVQVPKLLIRGGVEVLEVKTGVDSITEVECFLTPEMGDPDEHLRGFSK  60
AEC14679.1      MAPTKRKGERKDPVQVPKLLIRGGVEVLEVKTGVDSITEVECFLTPEMGDPDEHLRGFSK  60
AEC14727.1      MAPTKRKGERKDPVQVPKLLIRGGVEVLEVKTGVDSITEVECFLTPEMGDPDEHLRGFSK  60
AEC14733.1      MAPTKRKGERKDPVQVPKLLIRGGVEVLEVKTGVDSITEVECFLTPEMGDPDEHLRGFSK  60
AEC14964.1      MAPTKRKGERKDPVQVPKLLIRGGVEVLEVKTGVDSITEVECFLTPEMGDPDEHLRGFSK  60
AEC14995.1      MAPTKRKGERKDPVQVPKLLIRGGVEVLEVKTGVDSITEVECFLTPEMGDPDEHLRGFSK  60
AEC15008.1      MAPTKRKGERKDPVQVPKLLIRGGVEVLEVKTGVDSITEVECFLTPEMGDPDEHLRGFSK  60
AEC14970.1      MAPTKRKGERKDPVQVPKLLIRGGVEVLEVKTGVDSITEVECFLTPEMGDPDEHLRGFSK  60
QTJ15162.1      MAPTKRKGERKDPVQVPKLLIRGGVEVLEVKTGVDSITEVECFLTPEMGDPDEHLRGFSK  60
UYL83831.1      MAPTKRKAERKDPVQVPKLLIRGGVEVLEVKTGVDSITEVECFLTPEMGDPDEHLRGFSK  60
ALF06183.1      MAPTKRKGERKDPVQVPKLLIRGGVEVLEVKTGVDSITEVECFLTPEMGDPBEHLRGFSK  60
QCH41111.1      MAPTKRKGERKDPVQVPKLLIRGGVEVLEVKTGVDSITEVECFLTPEMGDPDEHLRGFSK  60
QCH41112.1      MAPTKRKGERKDPVQVPKLLIRGGVEVLEVKTGVDSITEVECFLTPEMGDPDEHLRGFSK  60
AEC14749.1      MAPTKRKGERKDPVQVPKLLIRGGVEVLEVKTGVDSITEVECFLTPEMGDPDEHLRGFSK  60
ALF06202.1      MAPTKRKGERKDPVQVPKLLIRGGVEVLEVKTGVDSITEVECFLTPEMGDPNEHLRGFSK  60
ALF06186.1      MAPTKRKGERKDPVQVPKLLIRGGVEVLEVKTGVDSITEVECFLTPEMGDPNEHLRGFSK  60
ALF06208.1      MAPTKRKGERKDPVQVPKLLIRGGVEVLEVKTGVDSITEVECFLTPEMGDPNEHLRGFSK  60
ALF06197.1      MAPTKRKGERKDPVQVPKLLIRGGVEVLEVKTGVDSITEVECFLTPEMGDPNEHLRGFSK  60
AEC15037.1      MAPTKRKGERKDPVQVPKLLIRGGVEVLEVKTGVDSITEVECFLTPEMGDPDEHFRGFSK  60
UPN63109.1      MAPTKRKGERKDPVQVPKLLIRGGVEVLEVKTGVDSITEVECFLTPEMGDPDEHFRGFSK  60
UPN63107.1      MAPTKRKGERKDPVQVPKLLIRGGVEVLEVKTGVDSITEVECFLTPEMGDPDEHLRGFSK  60
UPN63108.1      MAPTKRKGERKDPVQVPKLLIRGGVEVLEVKTGVDSITEVECFLTPEMGDPDEHLRGFSK  60
QBQ04202.1      MAPTKRKGERKDPVQVPKLLIRGGVEVLEVKTGVDSITEVECFLTPEMGDPDEHLRGFSK  60
QBM11923.1      MAPTKRKGERKDPVQVPKLLIRGGVEVLEVKTGVDSITEVECFLTPEMGDPDEHLRGFSK  60
AAK70306.1      MAPTKRKGERKHPVQVPKLLIRGGVEVLEVKTGVDSIAEVECFLTPEMGDPDEHLRGFSK  60
AAC59337.1      MAPTKRKGERKDPVQVPKLLIRGGVEVLEVKTGVDSITEVECFLTPEMGDPDEHLRGFSK  60
```

```
AAL37677.1        MAPTKRKGERKDPVQVPKLLIRGGVEVLEVKTGVDSITEVECFLTPEMGDPDEHLRGFSK  60
AAK70276.1        MAPTKRKGERKDPVQVPKLLIRGGVEVLEVKTGVDSITEVECFLTPEMGDPDEHLRGFSK  60
BAC22792.1        MAPTKRKGERKDPVQVPKLLIRGGVEVLEVKTGVDSITEVECFLTPEMGDPDEHLRGFSK  60
BAC22798.1        MAPTKRKGERKDPVQVPKLLIRGGVEVLEVKTGVDSITEVECFLTPEMGDPDEHLRGFSK  60
BAC22804.1        MAPTKRKGERKDPVQVPKLLIRGGVEVLEVKTGVDSITEVECFLTPEMGDPDEHLRGFSK  60
BAC22810.1        MAPTKRKGERKDPVQVPKLLIRGGVEVLEVKTGVDSITEVECFLTPEMGDPDEHLRGFSK  60
BAC22858.1        MAPTKRKGERKDPVQVPKLLIRGGVEVLEVKTGVDSITEVECFLTPEMGDPDEHLRGFSK  60
BAC15701.1        MAPTKRKGERKDPVQVPKLLIRGGVEVLEVKTGVDSITEVECFLTPEMGDPDEHLRGFSK  60
BAB68863.1        MAPTKRKGERKDPVQVPKLLIRGGVEVLEVKTGVDSITEVECFLTPEMGDPDEHLRGFSK  60
BAC66424.1        MAPTKRKGERKDPVQVPKLLIRGGVEVLEVKTGVDSITEVECFLTPEMGDPDEHLRGFSK  60
BAD21235.2        MAPTKRKGERKDPVQVPKLLIRGGVEVLEVKTGVDSITEVECFLTPEMGDPDEHLRGFSK  60
QTJ15210.1        MAPTKRKAERKDPVQVPKLLIRGGVEVLEVKTGVDSITEVECFLTPEMGDPDEHLRGFSK  60
ASV51748.1        MAPTKRKAERKDPVQVPKLLIRGGVEVLEVKTGVDSITEVECFLTPEMGDPDEHLRGFSK  60
ASV51754.1        MAPTKRKAERKDPVQVPKLLIRGGVEVLEVKTGVDSITEVECFLTPEMGDPDEHLRGFSK  60
ASV51778.1        MAPTKRKAERKDPVQVPKLLIRGGVEVLEVKTGVDSITEVECFLTPEMGDPDEHLRGFSK  60
ASV51814.1        MAPTKRKAERKDPVQVPKLLIRGGVEVLEVKTGVDSITEVECFLTPEMGDPDEHLRGFSK  60
CCW72488.1        MAPTKRKAERKDPVQVPKLLIRGGVEVLEVKTGVDSITEVECFLTPEMGDPDEHLRGFSK  60
AEC15219.1        MAPTKRKAERKDPVQVPKLLIRGGVEVLEVKTGVDSITEVECFLTPEMGDPDEHLRGFSK  60
BAD21241.1        MAPTKRKAERKDPVQVPKLLIRGGVEVLEVKTGVDSITEVECFLTPEMGDPDEHLRGFSK  60
BAD21253.1        MAPTKRKAERKDPVQVPKLLIRGGVEVLEVKTGVDSITEVECFLTPEMGDPDEHLRGFSK  60
BAB93086.1        MAPTKRKAERKDPVQVPKLLIRGGVEVLEVKTGVDSITEVECFLTPEMGDPDEHLRGFSK  60
BAB68953.1        MAPTKRKAERKDPVQVPKLLIRGGVEVLEVKTGVDSITEVECFLTPEMGDPDEHLRGFSK  60
BAD98966.1        MAPTKRKGERKDPVQVPKLLIRGGVEVLEVKTGVDSITEVECFLTPEMGDPDEHLRGFSK  60
BAD98972.1        MAPTKRKGERKDPVQVPKLLIRGGVEVLEVKTGVDSITEVECFLTPEMGDPDEHLRGFSK  60
BAD91887.1        MAPTKRKGERKDPVQVPKLLIRGGVEVLEVKTGVDSITEVECFLTPEMGDPDEHLRGFSK  60
BAD27118.1        MAPTKRKGERKDPVQVPKLLIRGGVEVLEVKTGVDSITEVECFLTPEMGDPDEHLRGFSK  60
BAD06227.1        MAPTKRKGERKDPVQVPKLLIRGGVEVLEVKTGVDSITEVECFLTPEMGDPDEHLRGFSK  60
BAC66412.1        MAPTKRKGERKDPVQVPKLLIRGGVEVLEVKTGVDSITEVECFLTPEMGDPDEHLRGFSK  60
BAC66430.1        MAPTKRKGERKDPVQVPKLLIRGGVEVLEVKTGVDSITEVECFLTPEMGDPDEHLRGFSK  60
BAC22816.1        MAPTKRKGERKDPVQVPKLLIRGGVEVLEVKTGVDSITEVECFLTPEMGDPDEHLRGFSK  60
BAB93026.1        MAPTKRKGERKDPVQVPKLLIRGGVEVLEVKTGVDSITEVECFLTPEMGDPDEHLRGFSK  60
BAB93032.1        MAPTKRKGERKDPVQVPKLLIRGGVEVLEVKTGVDSITEVECFLTPEMGDPDEHLRGFSK  60
BAB93074.1        MAPTKRKGERKDPVQVPKLLIRGGVEVLEVKTGVDSITEVECFLTPEMGDPDEHLRGFSK  60
BAB93080.1        MAPTKRKGERKDPVQVPKLLIRGGVEVLEVKTGVDSITEVECFLTPEMGDPDEHLRGFSK  60
BAB68917.1        MAPTKRKGERKDPVQVPKLLIRGGVEVLEVKTGVDSITEVECFLTPEMGDPDEHLRGFSK  60
BAB68923.1        MAPTKRKGERKDPVQVPKLLIRGGVEVLEVKTGVDSITEVECFLTPEMGDPDEHLRGFSK  60
BAB68929.1        MAPTKRKGERKDPVQVPKLLIRGGVEVLEVKTGVDSITEVECFLTPEMGDPDEHLRGFSK  60
BAB68935.1        MAPTKRKGERKDPVQVPKLLIRGGVEVLEVKTGVDSITEVECFLTPEMGDPDEHLRGFSK  60
BAB68941.1        MAPTKRKGERKDPVQVPKLLIRGGVEVLEVKTGVDSITEVECFLTPEMGDPDEHLRGFSK  60
ALF06214.1        MAPTKRKGERKDPVQVPKLLIRGGVEVLEVKTGVDSITEVECFLTPEMGDPDEHLRGFSK  60
AAL37707.1        MAPTKRKGERKDPVQVPKLLIRGGVEVLEVKTGVDSITEVECFLTPEMGDPNEHLRGFSK  60
BBG22688.1        MAPTKRKGERKDPVQVPKLLIRGGVEVLEVKTGVDSITEVECFLTPEMGDPDEHLRGFSK  60
BAB11734.1        MAPTKRKGERKDPVQVPKLLIRGGVEVLEVKTGVDSITEVECFLTPEMGDPDEHLRGFSK  60
BAA05638.1        MAPTKRKGERKDPVQVPKLLIRGGVEVLEVKTGVDSITEVECFLTPEMGDPDEHLRGFSK  60
BAV69361.1        MAPTKRKGERKDPVQVPKLLIRGGVEVLEVKTGVDSITEVECFLTPEMGDPDEHLRGFSK  60
BAV69349.1        MAPTKRKGERKDPVQVPKLLIRGGVEVLEVKTGVDSITEVECFLTPEMGDPDEHLRGFSK  60
BAV69367.1        MAPTKRKGERKDPVQVPKLLIRGGVEVLEVKTGVDSITEVECFLTPEMGDPDEHLRGFSK  60
BAE00135.1        MAPTKRKGERKDPVQVPKLLIRGGVEVLEVKTGVDSITEVECFLTPEMGDPDEHLRGFSK  60
BAE00147.1        MAPTKRKGERKDPVQVPKLLIRGGVEVLEVKTGVDSITEVECFLTPEMGDPDEHLRGFSK  60
BAD11896.1        MAPTKRKGERKDPVQVPKLLIRGGVEVLEVKTGVDSITEVECFLTPEMGDPDEHLRGFSK  60
BAD06126.1        MAPTKRKGERKDPVQVPKLLIRGGVEVLEVKTGVDSITEVECFLTPEMGDPDEHLRGFSK  60
BAC81904.1        MAPTKRKGERKDPVQVPKLLIRGGVEVLEVKTGVDSITEVECFLTPEMGDPDEHLRGFSK  60
BAC81910.1        MAPTKRKGERKDPVQVPKLLIRGGVEVLEVKTGVDSITEVECFLTPEMGDPDEHLRGFSK  60
BAC81916.1        MAPTKRKGERKDPVQVPKLLIRGGVEVLEVKTGVDSITEVECFLTPEMGDPDEHLRGFSK  60
BAC81922.1        MAPTKRKGERKDPVQVPKLLIRGGVEVLEVKTGVDSITEVECFLTPEMGDPDEHLRGFSK  60
BAC81946.1        MAPTKRKGERKDPVQVPKLLIRGGVEVLEVKTGVDSITEVECFLTPEMGDPDEHLRGFSK  60
BAC81952.1        MAPTKRKGERKDPVQVPKLLIRGGVEVLEVKTGVDSITEVECFLTPEMGDPDEHLRGFSK  60
BAC81958.1        MAPTKRKGERKDPVQVPKLLIRGGVEVLEVKTGVDSITEVECFLTPEMGDPDEHLRGFSK  60
AAM89297.1        MAPTKRKGERKDPVQVPKLLIRGGVEVLEVKTGVDSITEVECFLTPEMGDPDEHLRGFSK  60
AAM89303.1        MAPTKRKGERKDPVQVPKLLIRGGVEVLEVKTGVDSITEVECFLTPEMGDPDEHLRGFSK  60
AAM89309.1        MAPTKRKGERKDPVQVPKLLIRGGVEVLEVKTGVDSITEVECFLTPEMGDPDEHLRGFSK  60
BAC66388.1        MAPTKRKGERKDPVQVPKLLIRGGVEVLEVKTGVDSITEVECFLTPEMGDPDEHLRGFSK  60
BAC66400.1        MAPTKRKGERKDPVQVPKLLIRGGVEVLEVKTGVDSITEVECFLTPEMGDPDEHLRGFSK  60
BAC66406.1        MAPTKRKGERKDPVQVPKLLIRGGVEVLEVKTGVDSITEVECFLTPEMGDPDEHLRGFSK  60
```

BAC22756.1	MAPTKRKGERKDPVQVPKLLIRGGVEVLEVKTGVDSITEVECFLTPEMGDPDEHLRGFSK	60
BAC22822.1	MAPTKRKGERKDPVQVPKLLIRGGVEVLEVKTGVDSITEVECFLTPEMGDPDEHLRGFSK	60
BAC22894.1	MAPTKRKGERKDPVQVPKLLIRGGVEVLEVKTGVDSITEVECFLTPEMGDPDEHLRGFSK	60
BAC22900.1	MAPTKRKGERKDPVQVPKLLIRGGVEVLEVKTGVDSITEVECFLTPEMGDPDEHLRGFSK	60
BAC15635.1	MAPTKRKGERKDPVQVPKLLIRGGVEVLEVKTGVDSITEVECFLTPEMGDPDEHLRGFSK	60
BAC15653.1	MAPTKRKGERKDPVQVPKLLIRGGVEVLEVKTGVDSITEVECFLTPEMGDPDEHLRGFSK	60
BAC15665.1	MAPTKRKGERKDPVQVPKLLIRGGVEVLEVKTGVDSITEVECFLTPEMGDPDEHLRGFSK	60
BAC03065.1	MAPTKRKGERKDPVQVPKLLIRGGVEVLEVKTGVDSITEVECFLTPEMGDPDEHLRGFSK	60
AAL37689.1	MAPTKRKGERKDPVQVPKLLIRGGVEVLEVKTGVDSITEVECFLTPEMGDPDEHLRGFSK	60
AAL37701.1	MAPTKRKGERKDPVQVPKLLIRGGVEVLEVKTGVDSITEVECFLTPEMGDPDEHLRGFSK	60
BAB68803.1	MAPTKRKGERKDPVQVPKLLIRGGVEVLEVKTGVDSITEVECFLTPEMGDPDEHLRGFSK	60
BAB68965.1	MAPTKRKGERKDPVQVPKLLIRGGVEVLEVKTGVDSITEVECFLTPEMGDPDEHLRGFSK	60
BAB68971.1	MAPTKRKGERKDPVQVPKLLIRGGVEVLEVKTGVDSITEVECFLTPEMGDPDEHLRGFSK	60
AAK97964.1	MAPTKRKGERKDPVQVPKLLIRGGVEVLEVKTGVDSITEVECFLTPEMGDPDEHLRGFSK	60
AAK97970.1	MAPTKRKGERKDPVQVPKLLIRGGVEVLEVKTGVDSITEVECFLTPEMGDPDEHLRGFSK	60
AAC40841.1	MAPTKRKGERKDPVQVPKLLIRGGVEVLEVKTGVDSITEVECFLTPEMGDPDEHLRGFSK	60
BAA05636.1	MAPTKRKGERKDPVQVPKLLIRGGVEVLEVKTGVDSITEVECFLTPEMGDPDEHLRGFSK	60
BAA01970.1	MAPTKRKGERKDPVQVPKLLIRGGVEVLEVKTGVDSITEVECFLTPEMGDPDEHLRGFSK	60
BAD06144.1	MAPTKRKGERKDPVQVPKLLIRGGVEVLEVKTGVDSITEVECFLTPEMGDPDEHLRGFSK	60
BAC81852.1	MAPTKRKGERKDPVQVPKLLIRGGVEVLEVKTGVDSITEVECFLTPEMGDPDEHLRGFSK	60
BAC81858.1	MAPTKRKGERKDPVQVPKLLIRGGVEVLEVKTGVDSITEVECFLTPEMGDPDEHLRGFSK	60
BAC22864.1	MAPTKRKGERKDPVQVPKLLIRGGVEVLEVKTGVDSITEVECFLTPEMGDPDEHLRGFSK	60
AAM89315.1	MAPTKRKGERKDPVQVPKLLIRGGVEVLEVKTGVDSITEVECFLTPEMGDPDEHLRGFSK	60
BAC66382.1	MAPTKRKGERKDPVQVPKLLIRGGVEVLEVKTGVDSITEVECFLTPEMGDPDEHLRGFSK	60
BAC66394.1	MAPTKRKGERKDPVQVPKLLIRGGVEVLEVKTGVDSITEVECFLTPEMGDPDEHLRGFSK	60
BAC66418.1	MAPTKRKGERKDPVQVPKLLIRGGVEVLEVKTGVDSITEVECFLTPEMGDPDEHLRGFSK	60
BAC22828.1	MAPTKRKGERKDPVQVPKLLIRGGVEVLEVKTGVDSITEVECFLTPEMGDPDEHLRGFSK	60
BAC22846.1	MAPTKRKGERKDPVQVPKLLIRGGVEVLEVKTGVDSITEVECFLTPEMGDPDEHLRGFSK	60
AAM69590.1	MAPTKRKGERKDPVQVPKLLIRGGVEVLEVKTGVDSITEVECFLTPEMGDPDEHLRGFSK	60
BAB93014.1	MAPTKRKGERKDPVQVPKLLIRGGVEVLEVKTGVDSITEVECFLTPEMGDPDEHLRGFSK	60
BAB93020.1	MAPTKRKGERKDPVQVPKLLIRGGVEVLEVKTGVDSITEVECFLTPEMGDPDEHLRGFSK	60
BAB93092.1	MAPTKRKGERKDPVQVPKLLIRGGVEVLEVKTGVDSITEVECFLTPEMGDPDEHLRGFSK	60
BAB93098.1	MAPTKRKGERKDPVQVPKLLIRGGVEVLEVKTGVDSITEVECFLTPEMGDPDEHLRGFSK	60
AAL37665.1	MAPTKRKGERKDPVQVPKLLIRGGVEVLEVKTGVDSITEVECFLTPEMGDPDEHLRGFSK	60
BAB68773.1	MAPTKRKGERKDPVQVPKLLIRGGVEVLEVKTGVDSITEVECFLTPEMGDPDEHLRGFSK	60
BAB68779.1	MAPTKRKGERKDPVQVPKLLIRGGVEVLEVKTGVDSITEVECFLTPEMGDPDEHLRGFSK	60
AAK28460.1	MAPTKRKGERKDPVQVPKLLIRGGVEVLEVKTGVDSITEVECFLTPEMGDPDEHLRGFSK	60
AAK28466.1	MAPTKRKGERKDPVQVPKLLIRGGVEVLEVKTGVDSITEVECFLTPEMGDPDEHLRGFSK	60
BAB11716.1	MAPTKRKGERKDPVQVPKLLIRGGVEVLEVKTGVDSITEVECFLTPEMGDPDEHLRGFSK	60
BAB11722.1	MAPTKRKGERKDPVQVPKLLIRGGVEVLEVKTGVDSITEVECFLTPEMGDPDEHLRGFSK	60
AAK97976.1	MAPTKRKGERKDPVQVPKLLIRGGVEVLEVKTGVDSITEVECFLTPEMGDPDEHLRGFSK	60
BAC15641.1	MAPTKRKGERKDPVQVPKLLIRGGVEVLEVKSGVDSITEVECFLTPEMGDPDEHLRGFSK	60
BAC15647.1	MAPTKRKGERKDPVQVPKLLIRGGVEVLEVKSGVDSITEVECFLTPEMGDPDEHLRGFSK	60
AAL37647.1	MAPTKRKGERKDPVQVPKLLIRGGVEVLEVKTGVDSITEVECFLTPEMGDPDEHLRGFSK	60
BAB68797.1	MAPTKRKGERKDPVQVPKLLIRGGVEVLEVKTGVDSITEVECFLTPEMGDPDEHLRGFSK	60
AAG37198.1	MAPTKRKGERKDPVQVPKLLIRGGVEVLEVKTGVDSITEVECFLTPEMGDPDEHLRGFSK	60
ALF06241.1	MAPTKRKGERKDPVQVPKLLIRGGVEVLEVKTGVDSITEVECFLTPEMGDPDEHLRGFSK	60
AFE56117.1	MAPTKRKGERKDPVQVPKLLIRGGVEVLEVKTGVDSITEVECFLTPEMGDPDEHLRGFSK	60
BAF95600.1	MAPTKRKGERKDPVQVPKLLIRGGVEVLEVKTGVDSITEVECFLTPEMGDPDEHLRGFSK	60
BAD11836.1	MAPTKRKGERKDPVQVPKLLIRGGVEVLEVKTGVDSITEVECFLTPEMGDPDEHLRGFSK	60
BAD11842.1	MAPTKRKGERKDPVQVPKLLIRGGVEVLEVKTGVDSITEVECFLTPEMGDPDEHLRGFSK	60
BAD11848.1	MAPTKRKGERKDPVQVPKLLIRGGVEVLEVKTGVDSITEVECFLTPEMGDPDEHLRGFSK	60
AAM89327.1	MAPTKRKGERKDPVQVPKLLIRGGVEVLEVKTGVDSITEVECFLTPEMGDPDEHLRGFSK	60
AAM89339.1	MAPTKRKGERKDPVQVPKLLIRGGVEVLEVKTGVDSITEVECFLTPEMGDPDEHLRGFSK	60
BAC15707.1	MAPTKRKGERKDPVQVPKLLIRGGVEVLEVKTGVDSITEVECFLTPEMGDPDEHLRGFSK	60
BAB68833.1	MAPTKRKGERKDPVQVPKLLIRGGVEVLEVKTGVDSITEVECFLTPEMGDPDEHLRGFSK	60
BAB68839.1	MAPTKRKGERKDPVQVPKLLIRGGVEVLEVKTGVDSITEVECFLTPEMGDPDEHLRGFSK	60
AYM55604.1	MAPTKRKGERKDPVQVPKLLIRGGVEVLEVKTGVDSITEVECFLTPEMGDPDEHLRGFSK	60
BAV69355.1	MAPTKRKGERKDPVQVPKLLIRGGVEVLEVKTGVDSITEVECFLTPEMGDPDEHLRGFSK	60
ALF06220.1	MAPTKRKGERKDPVQVPKLLIRGGVEVLEVKTGVDSITEVECFLTPEMGDPDEHLRGFSK	60
ALF06247.1	MAPTKRKGERKDPVQVPKLLIRGGVEVLEVKTGVDSITEVECFLTPEMGDPDEHLRGFSK	60
ALF06250.1	MAPTKRKGERKDPVQVPKLLIRGGVEVLEVKTGVDSITEVECFLTPEMGDPDEHLRGFSK	60
ALF06253.1	MAPTKRKGERKDPVQVPKLLIRGGVEVLEVKTGVDSITEVECFLTPEMGDPDEHLRGFSK	60
ALF06259.1	MAPTKRKGERKDPVQVPKLLIRGGVEVLEVKTGVDSITEVECFLTPEMGDPDEHLRGFSK	60

```
ALF06265.1      MAPTKRKGERKDPVQVPKLLIRGGVEVLEVKTGVDSITEVECFLTPEMGDPDEHLRGFSK  60
CCW72502.1      MAPTKRKGERKDPVQVPKLLIRGGVEVLEVKTGVDSITEVECFLTPEMGDPDEHLRGFSK  60
BAF40757.1      MAPTKRKGERKDPVQVPKLLIRGGVEVLEVKTGVDSITEVECFLTPEMGDPDEHLRGFSK  60
BAF40763.1      MAPTKRKGERKDPVQVPKLLIRGGVEVLEVKTGVDSITEVECFLTPEMGDPDEHLRGFSK  60
BAF40769.1      MAPTKRKGERKDPVQVPKLLIRGGVEVLEVKTGVDSITEVECFLTPEMGDPDEHLRGFSK  60
BAF40775.1      MAPTKRKGERKDPVQVPKLLIRGGVEVLEVKTGVDSITEVECFLTPEMGDPDEHLRGFSK  60
BAE94732.1      MAPTKRKGERKDPVQVPKLLIRGGVEVLEVKTGVDSITEVECFLTPEMGDPDEHLRGFSK  60
BAE94738.1      MAPTKRKGERKDPVQVPKLLIRGGVEVLEVKTGVDSITEVECFLTPEMGDPDEHLRGFSK  60
BAE00129.1      MAPTKRKGERKDPVQVPKLLIRGGVEVLEVKTGVDSITEVECFLTPEMGDPDEHLRGFSK  60
BAE00141.1      MAPTKRKGERKDPVQVPKLLIRGGVEVLEVKTGVDSITEVECFLTPEMGDPDEHLRGFSK  60
BAD11824.1      MAPTKRKGERKDPVQVPKLLIRGGVEVLEVKTGVDSITEVECFLTPEMGDPDEHLRGFSK  60
BAD11830.1      MAPTKRKGERKDPVQVPKLLIRGGVEVLEVKTGVDSITEVECFLTPEMGDPDEHLRGFSK  60
BAD11854.1      MAPTKRKGERKDPVQVPKLLIRGGVEVLEVKTGVDSITEVECFLTPEMGDPDEHLRGFSK  60
BAD11860.1      MAPTKRKGERKDPVQVPKLLIRGGVEVLEVKTGVDSITEVECFLTPEMGDPDEHLRGFSK  60
BAD11866.1      MAPTKRKGERKDPVQVPKLLIRGGVEVLEVKTGVDSITEVECFLTPEMGDPDEHLRGFSK  60
BAD11872.1      MAPTKRKGERKDPVQVPKLLIRGGVEVLEVKTGVDSITEVECFLTPEMGDPDEHLRGFSK  60
BAD11878.1      MAPTKRKGERKDPVQVPKLLIRGGVEVLEVKTGVDSITEVECFLTPEMGDPDEHLRGFSK  60
BAD11884.1      MAPTKRKGERKDPVQVPKLLIRGGVEVLEVKTGVDSITEVECFLTPEMGDPDEHLRGFSK  60
BAD11890.1      MAPTKRKGERKDPVQVPKLLIRGGVEVLEVKTGVDSITEVECFLTPEMGDPDEHLRGFSK  60
BAD11902.1      MAPTKRKGERKDPVQVPKLLIRGGVEVLEVKTGVDSITEVECFLTPEMGDPDEHLRGFSK  60
BAD06233.1      MAPTKRKGERKDPVQVPKLLIRGGVEVLEVKTGVDSITEVECFLTPEMGDPDEHLRGFSK  60
BAD06066.1      MAPTKRKGERKDPVQVPKLLIRGGVEVLEVKTGVDSITEVECFLTPEMGDPDEHLRGFSK  60
BAD06078.1      MAPTKRKGERKDPVQVPKLLIRGGVEVLEVKTGVDSITEVECFLTPEMGDPDEHLRGFSK  60
BAD06114.1      MAPTKRKGERKDPVQVPKLLIRGGVEVLEVKTGVDSITEVECFLTPEMGDPDEHLRGFSK  60
BAD06132.1      MAPTKRKGERKDPVQVPKLLIRGGVEVLEVKTGVDSITEVECFLTPEMGDPDEHLRGFSK  60
BAD06138.1      MAPTKRKGERKDPVQVPKLLIRGGVEVLEVKTGVDSITEVECFLTPEMGDPDEHLRGFSK  60
BAD06162.1      MAPTKRKGERKDPVQVPKLLIRGGVEVLEVKTGVDSITEVECFLTPEMGDPDEHLRGFSK  60
BAD06168.1      MAPTKRKGERKDPVQVPKLLIRGGVEVLEVKTGVDSITEVECFLTPEMGDPDEHLRGFSK  60
BAC82365.1      MAPTKRKGERKDPVQVPKLLIRGGVEVLEVKTGVDSITEVECFLTPEMGDPDEHLRGFSK  60
BAC81846.1      MAPTKRKGERKDPVQVPKLLIRGGVEVLEVKTGVDSITEVECFLTPEMGDPDEHLRGFSK  60
BAC81864.1      MAPTKRKGERKDPVQVPKLLIRGGVEVLEVKTGVDSITEVECFLTPEMGDPDEHLRGFSK  60
BAC81870.1      MAPTKRKGERKDPVQVPKLLIRGGVEVLEVKTGVDSITEVECFLTPEMGDPDEHLRGFSK  60
BAC81874.1      MAPTKRKGERKDPVQVPKLLIRGGVEVLEVKTGVDSITEVECFLTPEMGDPDEHLRGFSK  60
BAC81880.1      MAPTKRKGERKDPVQVPKLLIRGGVEVLEVKTGVDSITEVECFLTPEMGDPDEHLRGFSK  60
BAC81886.1      MAPTKRKGERKDPVQVPKLLIRGGVEVLEVKTGVDSITEVECFLTPEMGDPDEHLRGFSK  60
BAC81892.1      MAPTKRKGERKDPVQVPKLLIRGGVEVLEVKTGVDSITEVECFLTPEMGDPDEHLRGFSK  60
BAC81898.1      MAPTKRKGERKDPVQVPKLLIRGGVEVLEVKTGVDSITEVECFLTPEMGDPDEHLRGFSK  60
BAC81928.1      MAPTKRKGERKDPVQVPKLLIRGGVEVLEVKTGVDSITEVECFLTPEMGDPDEHLRGFSK  60
BAC81934.1      MAPTKRKGERKDPVQVPKLLIRGGVEVLEVKTGVDSITEVECFLTPEMGDPDEHLRGFSK  60
BAC81940.1      MAPTKRKGERKDPVQVPKLLIRGGVEVLEVKTGVDSITEVECFLTPEMGDPDEHLRGFSK  60
BAC81964.1      MAPTKRKGERKDPVQVPKLLIRGGVEVLEVKTGVDSITEVECFLTPEMGDPDEHLRGFSK  60
AAM89321.1      MAPTKRKGERKDPVQVPKLLIRGGVEVLEVKTGVDSITEVECFLTPEMGDPDEHLRGFSK  60
AAM89333.1      MAPTKRKGERKDPVQVPKLLIRGGVEVLEVKTGVDSITEVECFLTPEMGDPDEHLRGFSK  60
AAM89345.1      MAPTKRKGERKDPVQVPKLLIRGGVEVLEVKTGVDSITEVECFLTPEMGDPDEHLRGFSK  60
BAC66376.1      MAPTKRKGERKDPVQVPKLLIRGGVEVLEVKTGVDSITEVECFLTPEMGDPDEHLRGFSK  60
BAC22834.1      MAPTKRKGERKDPVQVPKLLIRGGVEVLEVKTGVDSITEVECFLTPEMGDPDEHLRGFSK  60
BAC22840.1      MAPTKRKGERKDPVQVPKLLIRGGVEVLEVKTGVDSITEVECFLTPEMGDPDEHLRGFSK  60
BAC22852.1      MAPTKRKGERKDPVQVPKLLIRGGVEVLEVKTGVDSITEVECFLTPEMGDPDEHLRGFSK  60
BAC22870.1      MAPTKRKGERKDPVQVPKLLIRGGVEVLEVKTGVDSITEVECFLTPEMGDPDEHLRGFSK  60
BAC22876.1      MAPTKRKGERKDPVQVPKLLIRGGVEVLEVKTGVDSITEVECFLTPEMGDPDEHLRGFSK  60
BAC22882.1      MAPTKRKGERKDPVQVPKLLIRGGVEVLEVKTGVDSITEVECFLTPEMGDPDEHLRGFSK  60
BAC22888.1      MAPTKRKGERKDPVQVPKLLIRGGVEVLEVKTGVDSITEVECFLTPEMGDPDEHLRGFSK  60
BAC15629.1      MAPTKRKGERKDPVQVPKLLIRGGVEVLEVKTGVDSITEVECFLTPEMGDPDEHLRGFSK  60
BAC15659.1      MAPTKRKGERKDPVQVPKLLIRGGVEVLEVKTGVDSITEVECFLTPEMGDPDEHLRGFSK  60
BAC15671.1      MAPTKRKGERKDPVQVPKLLIRGGVEVLEVKTGVDSITEVECFLTPEMGDPDEHLRGFSK  60
BAC15677.1      MAPTKRKGERKDPVQVPKLLIRGGVEVLEVKTGVDSITEVECFLTPEMGDPDEHLRGFSK  60
BAC15689.1      MAPTKRKGERKDPVQVPKLLIRGGVEVLEVKTGVDSITEVECFLTPEMGDPDEHLRGFSK  60
BAC15713.1      MAPTKRKGERKDPVQVPKLLIRGGVEVLEVKTGVDSITEVECFLTPEMGDPDEHLRGFSK  60
BAC15731.1      MAPTKRKGERKDPVQVPKLLIRGGVEVLEVKTGVDSITEVECFLTPEMGDPDEHLRGFSK  60
BAC15737.1      MAPTKRKGERKDPVQVPKLLIRGGVEVLEVKTGVDSITEVECFLTPEMGDPDEHLRGFSK  60
BAC15743.1      MAPTKRKGERKDPVQVPKLLIRGGVEVLEVKTGVDSITEVECFLTPEMGDPDEHLRGFSK  60
AAL37641.1      MAPTKRKGERKDPVQVPKLLIRGGVEVLEVKTGVDSITEVECFLTPEMGDPDEHLRGFSK  60
AAL37653.1      MAPTKRKGERKDPVQVPKLLIRGGVEVLEVKTGVDSITEVECFLTPEMGDPDEHLRGFSK  60
AAL37659.1      MAPTKRKGERKDPVQVPKLLIRGGVEVLEVKTGVDSITEVECFLTPEMGDPDEHLRGFSK  60
```

```
AAL37671.1        MAPTKRKGERKDPVQVPKLLIRGGVEVLEVKTGVDSITEVECFLTPEMGDPDEHLRGFSK  60
AAL37683.1        MAPTKRKGERKDPVQVPKLLIRGGVEVLEVKTGVDSITEVECFLTPEMGDPDEHLRGFSK  60
BAB68791.1        MAPTKRKGERKDPVQVPKLLIRGGVEVLEVKTGVDSITEVECFLTPEMGDPDEHLRGFSK  60
BAB68821.1        MAPTKRKGERKDPVQVPKLLIRGGVEVLEVKTGVDSITEVECFLTPEMGDPDEHLRGFSK  60
BAB68845.1        MAPTKRKGERKDPVQVPKLLIRGGVEVLEVKTGVDSITEVECFLTPEMGDPDEHLRGFSK  60
BAB68851.1        MAPTKRKGERKDPVQVPKLLIRGGVEVLEVKTGVDSITEVECFLTPEMGDPDEHLRGFSK  60
BAB68857.1        MAPTKRKGERKDPVQVPKLLIRGGVEVLEVKTGVDSITEVECFLTPEMGDPDEHLRGFSK  60
BAB68869.1        MAPTKRKGERKDPVQVPKLLIRGGVEVLEVKTGVDSITEVECFLTPEMGDPDEHLRGFSK  60
BAB68875.1        MAPTKRKGERKDPVQVPKLLIRGGVEVLEVKTGVDSITEVECFLTPEMGDPDEHLRGFSK  60
AAK97910.1        MAPTKRKGERKDPVQVPKLLIRGGVEVLEVKTGVDSITEVECFLTPEMGDPDEHLRGFSK  60
AAK97916.1        MAPTKRKGERKDPVQVPKLLIRGGVEVLEVKTGVDSITEVECFLTPEMGDPDEHLRGFSK  60
AAK97922.1        MAPTKRKGERKDPVQVPKLLIRGGVEVLEVKTGVDSITEVECFLTPEMGDPDEHLRGFSK  60
AAK97928.1        MAPTKRKGERKDPVQVPKLLIRGGVEVLEVKTGVDSITEVECFLTPEMGDPDEHLRGFSK  60
AAK97934.1        MAPTKRKGERKDPVQVPKLLIRGGVEVLEVKTGVDSITEVECFLTPEMGDPDEHLRGFSK  60
AAK97940.1        MAPTKRKGERKDPVQVPKLLIRGGVEVLEVKTGVDSITEVECFLTPEMGDPDEHLRGFSK  60
AAK97946.1        MAPTKRKGERKDPVQVPKLLIRGGVEVLEVKTGVDSITEVECFLTPEMGDPDEHLRGFSK  60
AAK97952.1        MAPTKRKGERKDPVQVPKLLIRGGVEVLEVKTGVDSITEVECFLTPEMGDPDEHLRGFSK  60
AAK97958.1        MAPTKRKGERKDPVQVPKLLIRGGVEVLEVKTGVDSITEVECFLTPEMGDPDEHLRGFSK  60
AAK97982.1        MAPTKRKGERKDPVQVPKLLIRGGVEVLEVKTGVDSITEVECFLTPEMGDPDEHLRGFSK  60
AAK97988.1        MAPTKRKGERKDPVQVPKLLIRGGVEVLEVKTGVDSITEVECFLTPEMGDPDEHLRGFSK  60
AAK97994.1        MAPTKRKGERKDPVQVPKLLIRGGVEVLEVKTGVDSITEVECFLTPEMGDPDEHLRGFSK  60
BAB11698.1        MAPTKRKGERKDPVQVPKLLIRGGVEVLEVKTGVDSITEVECFLTPEMGDPDEHLRGFSK  60
BAB11704.1        MAPTKRKGERKDPVQVPKLLIRGGVEVLEVKTGVDSITEVECFLTPEMGDPDEHLRGFSK  60
AAC59478.1        MAPTKRKGERKDPVQVPKLLIRGGVEVLEVKTGVDSITEVECFLTPEMGDPDEHLRGFSK  60
AAC59484.1        MAPTKRKGERKDPVQVPKLLIRGGVEVLEVKTGVDSITEVECFLTPEMGDPDEHLRGFSK  60
AAC59490.1        MAPTKRKGERKDPVQVPKLLIRGGVEVLEVKTGVDSITEVECFLTPEMGDPDEHLRGFSK  60
BAA01963.1        MAPTKRKGERKDPVQVPKLLIRGGVEVLEVKTGVDSITEVECFLTPEMGDPDEHLRGFSK  60
BAA01964.1        MAPTKRKGERKDPVQVPKLLIRGGVEVLEVKTGVDSITEVECFLTPEMGDPDEHLRGFSK  60
BAB93068.1        MAPTKRKGERKDPVQVPKLLIRGGVEVLEVKTGVDSITEVECFLTPEMGDPNEHLRGFSK  60
ASV51856.1        MAPTKRKAERKDPVQVPKLLIRGGVEVLEVKTGVDSITEVECFLTPEMGDPNEHLRGFSK  60
ANH21097.1        MAPTKRKAERKDPVQVPKLLIRGGVEVLEVKTGVDSITEVECFLTPEMGDPDEHLRGFSK  60
AEC15167.1        MAPTKRKAERKDPVQVPKLLIRGGVEVLEVKTGVDSITEVECFLTPEMGDPDEHLRGFSK  60
AEC15168.1        MAPTKRKAERKDPVQVPKLLIRGGVEVLEVKTGVDSITEVECFLTPEMGDPDEHLRGFSK  60
AEC15169.1        MAPTKRKAERKDPVQVPKLLIRGGVEVLEVKTGVDSITEVECFLTPEMGDPDEHLRGFSK  60
QTJ15168.1        MAPTKRKGERKDPVQVPKLLIRGGVEVLEVKTGVDSITEVECFLTPEMGDPDEHLRGFSK  60
AEC15173.1        MAPTKRKGERKDPVQVPKLLIRGGVEVLEVKTGVDSITEVECFLTPEMGDPDEHLRGFSK  60
AEC15174.1        MAPTKRKGERKDPVQVPKLLIRGGVEVLEVKTGVDSITEVECFLTPEMGDPDEHLRGFSK  60
AEC15175.1        MAPTKRKGERKDPVQVPKLLIRGGVEVLEVKTGVDSITEVECFLTPEMGDPDEHLRGFSK  60
AEC15176.1        MAPTKRKGERKDPVQVPKLLIRGGVEVLEVKTGVDSITEVECFLTPEMGDPDEHLRGFSK  60
AEC15177.1        MAPTKRKGERKDPVQVPKLLIRGGVEVLEVKTGVDSITEVECFLTPEMGDPDEHLRGFSK  60
AEC15178.1        MAPTKRKGERKDPVQVPKLLIRGGVEVLEVKTGVDSITEVECFLTPEMGDPDEHLRGFSK  60
AEC15179.1        MAPTKRKGERKDPVQVPKLLIRGGVEVLEVKTGVDSITEVECFLTPEMGDPDEHLRGFSK  60
AEC15180.1        MAPTKRKGERKDPVQVPKLLIRGGVEVLEVKTGVDSITEVECFLTPEMGDPDEHLRGFSK  60
AEC15181.1        MAPTKRKGERKDPVQVPKLLIRGGVEVLEVKTGVDSITEVECFLTPEMGDPDEHLRGFSK  60
AEC15182.1        MAPTKRKGERKDPVQVPKLLIRGGVEVLEVKTGVDSITEVECFLTPEMGDPDEHLRGFSK  60
AEC15183.1        MAPTKRKGERKDPVQVPKLLIRGGVEVLEVKTGVDSITEVECFLTPEMGDPDEHLRGFSK  60
AEC15184.1        MAPTKRKGERKDPVQVPKLLIRGGVEVLEVKTGVDSITEVECFLTPEMGDPDEHLRGFSK  60
AEC15185.1        MAPTKRKGERKDPVQVPKLLIRGGVEVLEVKTGVDSITEVECFLTPEMGDPDEHLRGFSK  60
AEC15187.1        MAPTKRKGERKDPVQVPKLLIRGGVEVLEVKTGVDSITEVECFLTPEMGDPDEHLRGFSK  60
AEC15188.1        MAPTKRKGERKDPVQVPKLLIRGGVEVLEVKTGVDSITEVECFLTPEMGDPDEHLRGFSK  60
AEC15189.1        MAPTKRKGERKDPVQVPKLLIRGGVEVLEVKTGVDSITEVECFLTPEMGDPDEHLRGFSK  60
AEC15190.1        MAPTKRKGERKDPVQVPKLLIRGGVEVLEVKTGVDSITEVECFLTPEMGDPDEHLRGFSK  60
AEC15191.1        MAPTKRKGERKDPVQVPKLLIRGGVEVLEVKTGVDSITEVECFLTPEMGDPDEHLRGFSK  60
AEC15193.1        MAPTKRKGERKDPVQVPKLLIRGGVEVLEVKTGVDSITEVECFLTPEMGDPDEHLRGFSK  60
AEC14871.1        MAPTKRKGERKDPVQVPKLLIRGGVEVLEVKTGVDSIIEVECFLTPEMGDPDEHLRGFSK  60
AEC15222.1        MAPTKRKGERKDPVQVPKLLIRGGVEVLEVKTGVDSITEVECFLTPEMGDPDEHLRGFSK  60
AEC15130.1        MAPTKRKGERRDPVQVPKLLIRGGVEVLEVKTGVDSITEVECFLTPEMGDPDEHLRGFSK  60
AEC15144.1        MAPTKRKGERKDPVQVPKLLIRGGVEVLEVKTGVDSITEVECFLTPEMGDPDEHLRGFSK  60
AEC15154.1        MAPTKRKGERKDPVQVPKLLIRGGVEVLEVKTGVDSITEVECFLTPEMGDPDEHLRGFSK  60
AEC15157.1        MAPTKRKGERKDPVQVPKLLIRGGVEVLEVKTGVDSITEVECFLTPEMGDPDEHLRGFSK  60
AEC15160.1        MAPTKRKGERKDPVQVPKLLIRGGVEVLEVKTGVDSITEVECFLTPEMGDPDEHLRGFSK  60
AEC15231.1        MAPTKRKGERKDPVQVPKLLIRGGVEVLEVKTGVDSITEVECFLTPEMGDPDEHLRGFSK  60
ALF06191.1        MAPTKRKGERKDPVQVPKLLIRGGVEVLEVKAGVDSITEVECFLTPEMGDPDEHLRGFSK  60
WLD48112.1        MAPTKRKGERKDPVQVPKLLIRGGVEVLEVKTGVDSITEVECFLTPEMGDPDEHLRGFSK  60
```

QWT77140.1	MAPTKRKGERKDPVQVPKLLIRGGVEVLEVKTGVDSITEVECFLTPEMGDPDEHLRGFSK	60
QTJ15174.1	MAPTKRKGERKDPVQVPKLLIRGGVEVLEVKTGVDSITEVECFLTPEMGDPDEHLRGFSK	60
QTJ15186.1	MAPTKRKGERKDPVQVPKLLIRGGVEVLEVKTGVDSITEVECFLTPEMGDPDEHLRGFSK	60
QTJ15192.1	MAPTKRKGERKDPVQVPKLLIRGGVEVLEVKTGVDSITEVECFLTPEMGDPDEHLRGFSK	60
QTJ15198.1	MAPTKRKGERKDPVQVPKLLIRGGVEVLEVKTGVDSITEVECFLTPEMGDPDEHLRGFSK	60
ASV51772.1	MAPTKRKGERKDPVQVPKLLIRGGVEVLEVKTGVDSITEVECFLTPEMGDPDEHLRGFSK	60
CCW72478.1	MAPTKRKGERKDPVQVPKLLIRGGVEVLEVKTGVDSITEVECFLTPEMGDPDEHLRGFSK	60
CCW72481.1	MAPTKRKGERKDPVQVPKLLIRGGVEVLEVKTGVDSITEVECFLTPEMGDPDEHLRGFSK	60
CCW72487.1	MAPTKRKGERKDPVQVPKLLIRGGVEVLEVKTGVDSITEVECFLTPEMGDPDEHLRGFSK	60
CCW72492.1	MAPTKRKGERKDPVQVPKLLIRGGVEVLEVKTGVDSITEVECFLTPEMGDPDEHLRGFSK	60
CCW72496.1	MAPTKRKGERKDPVQVPKLLIRGGVEVLEVKTGVDSITEVECFLTPEMGDPDEHLRGFSK	60
CCW72499.1	MAPTKRKGERKDPVQVPKLLIRGGVEVLEVKTGVDSITEVECFLTPEMGDPDEHLRGFSK	60
CCW72504.1	MAPTKRKGERKDPVQVPKLLIRGGVEVLEVKTGVDSITEVECFLTPEMGDPDEHLRGFSK	60
CCW72508.1	MAPTKRKGERKDPVQVPKLLIRGGVEVLEVKTGVDSITEVECFLTPEMGDPDEHLRGFSK	60
CCW72510.1	MAPTKRKGERKDPVQVPKLLIRGGVEVLEVKTGVDSITEVECFLTPEMGDPDEHLRGFSK	60
CCW72519.1	MAPTKRKGERKDPVQVPKLLIRGGVEVLEVKTGVDSITEVECFLTPEMGDPDEHLRGFSK	60
CCW72521.1	MAPTKRKGERKDPVQVPKLLIRGGVEVLEVKTGVDSITEVECFLTPEMGDPDEHLRGFSK	60
AEC14864.1	MAPTKRKGERKDPVQVPKLLIRGGVEVLEVKTGVDSITEVECFLTPEMGDPDEHLRGFSK	60
AEC14865.1	MAPTKRKGERKDPVQVPKLLIRGGVEVLEVKTGVDSITEVECFLTPEMGDPDEHLRGFSK	60
AEC14866.1	MAPTKRKGERKDPVQVPKLLIRGGVEVLEVKTGVDSITEVECFLTPEMGDPDEHLRGFSK	60
AEC14867.1	MAPTKRKGERKDPVQVPKLLIRGGVEVLEVKTGVDSITEVECFLTPEMGDPDEHLRGFSK	60
AEC14868.1	MAPTKRKGERKDPVQVPKLLIRGGVEVLEVKTGVDSITEVECFLTPEMGDPDEHLRGFSK	60
AEC14869.1	MAPTKRKGERKDPVQVPKLLIRGGVEVLEVKTGVDSITEVECFLTPEMGDPDEHLRGFSK	60
AEC14870.1	MAPTKRKGERKDPVQVPKLLIRGGVEVLEVKTGVDSITEVECFLTPEMGDPDEHLRGFSK	60
AEC14872.1	MAPTKRKGERKDPVQVPKLLIRGGVEVLEVKTGVDSITEVECFLTPEMGDPDEHLRGFSK	60
AEC14873.1	MAPTKRKGERKDPVQVPKLLIRGGVEVLEVKTGVDSITEVECFLTPEMGDPDEHLRGFSK	60
AEC14874.1	MAPTKRKGERKDPVQVPKLLIRGGVEVLEVKTGVDSITEVECFLTPEMGDPDEHLRGFSK	60
AEC14875.1	MAPTKRKGERKDPVQVPKLLIRGGVEVLEVKTGVDSITEVECFLTPEMGDPDEHLRGFSK	60
AEC14876.1	MAPTKRKGERKDPVQVPKLLIRGGVEVLEVKTGVDSITEVECFLTPEMGDPDEHLRGFSK	60
AEC14877.1	MAPTKRKGERKDPVQVPKLLIRGGVEVLEVKTGVDSITEVECFLTPEMGDPDEHLRGFSK	60
AEC14878.1	MAPTKRKGERKDPVQVPKLLIRGGVEVLEVKTGVDSITEVECFLTPEMGDPDEHLRGFSK	60
AEC14879.1	MAPTKRKGERKDPVQVPKLLIRGGVEVLEVKTGVDSITEVECFLTPEMGDPDEHLRGFSK	60
AEC14880.1	MAPTKRKGERKDPVQVPKLLIRGGVEVLEVKTGVDSITEVECFLTPEMGDPDEHLRGFSK	60
AEC14881.1	MAPTKRKGERKDPVQVPKLLIRGGVEVLEVKTGVDSITEVECFLTPEMGDPDEHLRGFSK	60
AEC14882.1	MAPTKRKGERKDPVQVPKLLIRGGVEVLEVKTGVDSITEVECFLTPEMGDPDEHLRGFSK	60
AEC14883.1	MAPTKRKGERKDPVQVPKLLIRGGVEVLEVKTGVDSITEVECFLTPEMGDPDEHLRGFSK	60
AEC14884.1	MAPTKRKGERKDPVQVPKLLIRGGVEVLEVKTGVDSITEVECFLTPEMGDPDEHLRGFSK	60
AEC14885.1	MAPTKRKGERKDPVQVPKLLIRGGVEVLEVKTGVDSITEVECFLTPEMGDPDEHLRGFSK	60
AEC14886.1	MAPTKRKGERKDPVQVPKLLIRGGVEVLEVKTGVDSITEVECFLTPEMGDPDEHLRGFSK	60
AEC14887.1	MAPTKRKGERKDPVQVPKLLIRGGVEVLEVKTGVDSITEVECFLTPEMGDPDEHLRGFSK	60
AEC14888.1	MAPTKRKGERKDPVQVPKLLIRGGVEVLEVKTGVDSITEVECFLTPEMGDPDEHLRGFSK	60
AEC14889.1	MAPTKRKGERKDPVQVPKLLIRGGVEVLEVKTGVDSITEVECFLTPEMGDPDEHLRGFSK	60
AEC14890.1	MAPTKRKGERKDPVQVPKLLIRGGVEVLEVKTGVDSITEVECFLTPEMGDPDEHLRGFSK	60
AEC14891.1	MAPTKRKGERKDPVQVPKLLIRGGVEVLEVKTGVDSITEVECFLTPEMGDPDEHLRGFSK	60
AEC14892.1	MAPTKRKGERKDPVQVPKLLIRGGVEVLEVKTGVDSITEVECFLTPEMGDPDEHLRGFSK	60
AEC14894.1	MAPTKRKGERKDPVQVPKLLIRGGVEVLEVKTGVDSITEVECFLTPEMGDPDEHLRGFSK	60
AEC14895.1	MAPTKRKGERKDPVQVPKLLIRGGVEVLEVKTGVDSITEVECFLTPEMGDPDEHLRGFSK	60
AEC14896.1	MAPTKRKGERKDPVQVPKLLIRGGVEVLEVKTGVDSITEVECFLTPEMGDPDEHLRGFSK	60
AEC14897.1	MAPTKRKGERKDPVQVPKLLIRGGVEVLEVKTGVDSITEVECFLTPEMGDPDEHLRGFSK	60
AEC14898.1	MAPTKRKGERKDPVQVPKLLIRGGVEVLEVKTGVDSITEVECFLTPEMGDPDEHLRGFSK	60
AEC14899.1	MAPTKRKGERKDPVQVPKLLIRGGVEVLEVKTGVDSITEVECFLTPEMGDPDEHLRGFSK	60
AEC14900.1	MAPTKRKGERKDPVQVPKLLIRGGVEVLEVKTGVDSITEVECFLTPEMGDPDEHLRGFSK	60
AEC14901.1	MAPTKRKGERKDPVQVPKLLIRGGVEVLEVKTGVDSITEVECFLTPEMGDPDEHLRGFSK	60
AEC14902.1	MAPTKRKGERKDPVQVPKLLIRGGVEVLEVKTGVDSITEVECFLTPEMGDPDEHLRGFSK	60
AEC14903.1	MAPTKRKGERKDPVQVPKLLIRGGVEVLEVKTGVDSITEVECFLTPEMGDPDEHLRGFSK	60
AEC14904.1	MAPTKRKGERKDPVQVPKLLIRGGVEVLEVKTGVDSITEVECFLTPEMGDPDEHLRGFSK	60
AEC14905.1	MAPTKRKGERKDPVQVPKLLIRGGVEVLEVKTGVDSITEVECFLTPEMGDPDEHLRGFSK	60
AEC14907.1	MAPTKRKGERKDPVQVPKLLIRGGVEVLEVKTGVDSITEVECFLTPEMGDPDEHLRGFSK	60
AEC14908.1	MAPTKRKGERKDPVQVPKLLIRGGVEVLEVKTGVDSITEVECFLTPEMGDPDEHLRGFSK	60
AEC14909.1	MAPTKRKGERKDPVQVPKLLIRGGVEVLEVKTGVDSITEVECFLTPEMGDPDEHLRGFSK	60
AEC14910.1	MAPTKRKGERKDPVQVPKLLIRGGVEVLEVKTGVDSITEVECFLTPEMGDPDEHLRGFSK	60
AEC14911.1	MAPTKRKGERKDPVQVPKLLIRGGVEVLEVKTGVDSITEVECFLTPEMGDPDEHLRGFSK	60
AEC15164.1	MAPTKRKGERKDPVQVPKLLIRGGVEVLEVKTGVDSITEVECFLTPEMGDPDEHLRGFSK	60
BAF95594.1	MAPTKRKGERKDPVQVPKLLIRGGVEVLEVKTGVDSITEVECFLTPEMGDPDEHLRGFSK	60

```
BAF95606.1      MAPTKRKGERKDPVQVPKLLIRGGVEVLEVKTGVDSITEVECFLTPEMGDPDEHLRGFSK  60
BAF40745.1      MAPTKRKGERKDPVQVPKLLIRGGVEVLEVKTGVDSITEVECFLTPEMGDPDEHLRGFSK  60
BAF40751.1      MAPTKRKGERKDPVQVPKLLIRGGVEVLEVKTGVDSITEVECFLTPEMGDPDEHLRGFSK  60
BAF40781.1      MAPTKRKGERKDPVQVPKLLIRGGVEVLEVKTGVDSITEVECFLTPEMGDPDEHLRGFSK  60
BAF40787.1      MAPTKRKGERKDPVQVPKLLIRGGVEVLEVKTGVDSITEVECFLTPEMGDPDEHLRGFSK  60
BAF40793.1      MAPTKRKGERKDPVQVPKLLIRGGVEVLEVKTGVDSITEVECFLTPEMGDPDEHLRGFSK  60
BAF40799.1      MAPTKRKGERKDPVQVPKLLIRGGVEVLEVKTGVDSITEVECFLTPEMGDPDEHLRGFSK  60
BAF40811.1      MAPTKRKGERKDPVQVPKLLIRGGVEVLEVKTGVDSITEVECFLTPEMGDPDEHLRGFSK  60
BAF40817.1      MAPTKRKGERKDPVQVPKLLIRGGVEVLEVKTGVDSITEVECFLTPEMGDPDEHLRGFSK  60
BAF40823.1      MAPTKRKGERKDPVQVPKLLIRGGVEVLEVKTGVDSITEVECFLTPEMGDPDEHLRGFSK  60
BAF40829.1      MAPTKRKGERKDPVQVPKLLIRGGVEVLEVKTGVDSITEVECFLTPEMGDPDEHLRGFSK  60
BAF40835.1      MAPTKRKGERKDPVQVPKLLIRGGVEVLEVKTGVDSITEVECFLTPEMGDPDEHLRGFSK  60
BAF40841.1      MAPTKRKGERKDPVQVPKLLIRGGVEVLEVKTGVDSITEVECFLTPEMGDPDEHLRGFSK  60
BAF40847.1      MAPTKRKGERKDPVQVPKLLIRGGVEVLEVKTGVDSITEVECFLTPEMGDPDEHLRGFSK  60
BAC81834.1      MAPTKRKGERKDPVQVPKLLIRGGVEVLEVKTGVDSITEVECFLTPEMGDPDEHLRGFSK  60
BAC81840.1      MAPTKRKGERKDPVQVPKLLIRGGVEVLEVKTGVDSITEVECFLTPEMGDPDEHLRGFSK  60
AAN85461.1      MAPTKRKGERKDPVQVPKLLIRGGVEVLEVKTGVDSITEVECFLTPEMGDPDEHLRGFSK  60
AAN85467.1      MAPTKRKGERKDPVQVPKLLIRGGVEVLEVKTGVDSITEVECFLTPEMGDPDEHLRGFSK  60
AAN85473.1      MAPTKRKGERKDPVQVPKLLIRGGVEVLEVKTGVDSITEVECFLTPEMGDPDEHLRGFSK  60
BAC22762.1      MAPTKRKGERKDPVQVPKLLIRGGVEVLEVKTGVDSITEVECFLTPEMGDPDEHLRGFSK  60
BAC22768.1      MAPTKRKGERKDPVQVPKLLIRGGVEVLEVKTGVDSITEVECFLTPEMGDPDEHLRGFSK  60
BAC22774.1      MAPTKRKGERKDPVQVPKLLIRGGVEVLEVKTGVDSITEVECFLTPEMGDPDEHLRGFSK  60
BAC22780.1      MAPTKRKGERKDPVQVPKLLIRGGVEVLEVKTGVDSITEVECFLTPEMGDPDEHLRGFSK  60
BAC22786.1      MAPTKRKGERKDPVQVPKLLIRGGVEVLEVKTGVDSITEVECFLTPEMGDPDEHLRGFSK  60
BAB68809.1      MAPTKRKGERKDPVQVPKLLIRGGVEVLEVKTGVDSITEVECFLTPEMGDPDEHLRGFSK  60
BAB68815.1      MAPTKRKGERKDPVQVPKLLIRGGVEVLEVKTGVDSITEVECFLTPEMGDPDEHLRGFSK  60
BAB68827.1      MAPTKRKGERKDPVQVPKLLIRGGVEVLEVKTGVDSITEVECFLTPEMGDPDEHLRGFSK  60
AAC59496.1      MAPTKRKGERKDPVQVPKLLIRGGVEVLEVKTGVDSITEVECFLTPEMGDPDEHLRGFSK  60
AAC59502.1      MAPTKRKGERKDPVQVPKLLIRGGVEVLEVKTGVDSITEVECFLTPEMGDPDEHLRGFSK  60
AAC59508.1      MAPTKRKGERKDPVQVPKLLIRGGVEVLEVKTGVDSITEVECFLTPEMGDPDEHLRGFSK  60
AAC59514.1      MAPTKRKGERKDPVQVPKLLIRGGVEVLEVKTGVDSITEVECFLTPEMGDPDEHLRGFSK  60
AAC59520.1      MAPTKRKGERKDPVQVPKLLIRGGVEVLEVKTGVDSITEVECFLTPEMGDPDEHLRGFSK  60
AAB62680.1      MAPTKRKGERKDPVQVPKLLIRGGVEVLEVKTGVDSITEVECFLTPEMGDPDEHLRGFSK  60
BAA01961.1      MAPTKRKGERKDPVQVPKLLIRGGVEVLEVKTGVDSITEVECFLTPEMGDPDEHLRGFSK  60
WLD48082.1      MAPTKRKGERKDPVQVPKLLIRGGVEVLEVKTGVDSITEVECFLTPEMGDPDEHLRGFSK  60
WLD48088.1      MAPTKRKGERKDPVQVPKLLIRGGVEVLEVKTGVDSITEVECFLTPEMGDPDEHLRGFSK  60
CCW72516.1      MAPTKRKGERKDPVQVPKLLIRGGVEVLEVKTGVDSITEVECFLTPEMGDPDEHLRGFSK  60
CCW72523.1      MAPTKRKGERKDPVQVPKLLIRGGVEVLEVKTGVDSITEVECFLTPEMGDPDEHLRGFSK  60
AEC14783.1      MAPTKRKGERKDPVQVPKLLIRGGVEVLEVKTGVDSITEVECFLTPEMGDPDEHFRGFSK  60
AEC14784.1      MAPTKRKGERKDPVQVPKLLIRGGVEVLEVKTGVDSITEVECFLTPEMGDPDEHFRGFSK  60
AEC14785.1      MAPTKRKGERKDPVQVPKLLIRGGVEVLEVKTGVDSITEVECFLTPEMGDPDEHFRGFSK  60
AEC14786.1      MAPTKRKGERKDPVQVPKLLIRGGVEVLEVKTGVDSITEVECFLTPEMGDPDEHFRGFSK  60
AEC14787.1      MAPTKRKGERKDPVQVPKLLIRGGVEVLEVKTGVDSITEVECFLTPEMGDPDEHFRGFSK  60
AAB62687.1      MAPTKRKGERKDPVQVPKLLIRGGVEVLEVKTGVDSITEVECFLTPEMGDPDEHFRGFSK  60
AEC14893.1      MAPTKRKGERKDPVQVPKLLIRGGVEVLEVKTGVDSITEVECFLTPEMGDPDEHLRGFSK  60
AEC14906.1      MAPTIRKGERKDPVQVPKLLIRGGVEVLEVKTGVDSITEVECFLTPEMGDPDEHLRGFSK  60
AEC15118.1      MAPTKRKGERKDPVQVPKLLIRGGVEVLEVKTGVDSITEVECFLTPEMGDPDEHLRGFSK  60
AEC15120.1      MAPTKRKGERKDPVQVPKLLIRGGVEVLEVKTGVDSITEVECFLTPEMGDPDEHLRGFSK  60
AEC15121.1      MAPTKRKGERKDPVQVPKLLIRGGVEVLEVKTGVDSITEVECFLTPEMGDPDEHLRGFSK  60
AEC15122.1      MAPTKRKGERKDPVQVPKLLIRGGVEVLEVKTGVDSITEVECFLTPEMGDPDEHLRGFSK  60
AEC15124.1      MAPTKRKGERKDPVQVPKLLIRGGVEVLEVKTGVDSITEVECFLTPEMGDPDEHLRGFSK  60
AEC15125.1      MAPTKRKGERKDPVQVPKLLIRGGVEVLEVKTGVDSITEVECFLTPEMGDPDEHLRGFSK  60
AEC15127.1      MAPTKRKGERKDPVQVPKLLIRGGVEVLEVKTGVDSITEVECFLTPEMGDPDEHLRGFSK  60
AEC15129.1      MAPTKRKGERKDPVQVPKLLIRGGVEVLEVKTGVDSITEVECFLTPEMGDPDEHLRGFSK  60
AEC15131.1      MAPTKRKGERKDPVQVPKLLIRGGVEVLEVKTGVDSITEVECFLTPEMGDPDEHLRGFSK  60
AEC15133.1      MAPTKRKGERKDPVQVPKLLIRGGVEVLEVKTGVDSITEVECFLTPEMGDPDEHLRGFSK  60
AEC15135.1      MAPTKRKGERKDPVQVPKLLIRGGVEVLEVKTGVDSITEVECFLTPEMGDPDEHLRGFSK  60
AEC15136.1      MAPTKRKGERKDPVQVPKLLIRGGVEVLEVKTGVDSITEVECFLTPEMGDPDEHLRGFSK  60
AEC15137.1      MAPTKRKGERKDPVQVPKLLIRGGVEVLEVKTGVDSITEVECFLTPEMGDPDEHLRGFSK  60
AEC15138.1      MAPTKRKGERKDPVQVPKLLIRGGVEVLEVKTGVDSITEVECFLTPEMGDPDEHLRGFSK  60
AEC15139.1      MAPTKRKGERKDPVQVPKLLIRGGVEVLEVKTGVDSITEVECFLTPEMGDPDEHLRGFSK  60
AEC15141.1      MAPTKRKGERKDPVQVPKLLIRGGVEVLEVKTGVDSITEVECFLTPEMGDPDEHLRGFSK  60
AEC15142.1      MAPTKRKGERKDPVQVPKLLIRGGVEVLEVKTGVDSITEVECFLTPEMGDPDEHLRGFSK  60
AEC15143.1      MAPTKRKGERKDPVQVPKLLIRGGVEVLEVKTGVDSITEVECFLTPEMGDPDEHLRGFSK  60
```

AEC15145.1	MAPTKRKGERKDPVQVPKLLIRGGVEVLEVKTGVDSITEVECFLTPEMGDPDEHLRGFSK	60
AEC15146.1	MAPTKRKGERKDPVQVPKLLIRGGVEVLEVKTGVDSITEVECFLTPEMGDPDEHLRGFSK	60
AEC15147.1	MAPTKRKGERKDPVQVPKLLIRGGVEVLEVKTGVDSITEVECFLTPEMGDPDEHLRGFSK	60
AEC15148.1	MAPTKRKGERKDPVQVPKLLIRGGVEVLEVKTGVDSITEVECFLTPEMGDPDEHLRGFSK	60
AEC15149.1	MAPTKRKGERKDPVQVPKLLIRGGVEVLEVKTGVDSITEVECFLTPEMGDPDEHLRGFSK	60
AEC15150.1	MAPTKRKGERKDPVQVPKLLIRGGVEVLEVKTGVDSITEVECFLTPEMGDPDEHLRGFSK	60
AEC15151.1	MAPTKRKGERKDPVQVPKLLIRGGVEVLEVKTGVDSITEVECFLTPEMGDPDEHLRGFSK	60
AEC15152.1	MAPTKRKGERKDPVQVPKLLIRGGVEVLEVKTGVDSITEVECFLTPEMGDPDEHLRGFSK	60
AEC15153.1	MAPTKRKGERKDPVQVPKLLIRGGVEVLEVKTGVDSITEVECFLTPEMGDPDEHLRGFSK	60
AEC15155.1	MAPTKRKGERKDPVQVPKLLIRGGVEVLEVKTGVDSITEVECFLTPEMGDPDEHLRGFSK	60
AEC15156.1	MAPTKRKGERKDPVQVPKLLIRGGVEVLEVKTGVDSITEVECFLTPEMGDPDEHLRGFSK	60
AEC15158.1	MAPTKRKGERKDPVQVPKLLIRGGVEVLEVKTGVDSITEVECFLTPEMGDPDEHLRGFSK	60
AEC15159.1	MAPTKRKGERKDPVQVPKLLIRGGVEVLEVKTGVDSITEVECFLTPEMGDPDEHLRGFSK	60
AEC15161.1	MAPTKRKGERKDPVQVPKLLIRGGVEVLEVKTGVDSITEVECFLTPEMGDPDEHLRGFSK	60
AEC15162.1	MAPTKRKGERKDPVQVPKLLIRGGVEVLEVKTGVDSITEVECFLTPEMGDPDEHLRGFSK	60
AEC15163.1	MAPTKRKGERKDPVQVPKLLIRGGVEVLEVKTGVDSITEVECFLTPEMGDPDEHLRGFSK	60
AEC15226.1	MAPTKRKGERKDPVQVPKLLIRGGVEVLEVKTGVDSITEVECFLTPEMGDPDEHLRGFSK	60
AEC15227.1	MAPTKRKGERKDPVQVPKLLIRGGVEVLEVKTGVDSITEVECFLTPEMGDPDEHLRGFSK	60
AEC15228.1	MAPTKRKGERKDPVQVPKLLIRGGVEVLEVKTGVDSITEVECFLTPEMGDPDEHLRGFSK	60
AEC15229.1	MAPTKRKGERKDPVQVPKLLIRGGVEVLEVKTGVDSITEVECFLTPEMGDPDEHLRGFSK	60
AEC15230.1	MAPTKRKGERKDPVQVPKLLIRGGVEVLEVKTGVDSITEVECFLTPEMGDPDEHLRGFSK	60
AEC15236.1	MAPTKRKGERKDPVQVPKLLIRGGVEVLEVKTGVDSITEVECFLTPEMGDPDEHLRGFSK	60
AEC15237.1	MAPTKRKGERKDPVQVPKLLIRGGVEVLEVKTGVDSITEVECFLTPEMGDPDEHLRGFSK	60
AEC15238.1	MAPTKRKGERKDPVQVPKLLIRGGVEVLEVKTGVDSITEVECFLTPEMGDPDEHLRGFSK	60
AEC15239.1	MAPTKRKGERKDPVQVPKLLIRGGVEVLEVKTGVDSITEVECFLTPEMGDPDEHLRGFSK	60
AEC15240.1	MAPTKRKGERKDPVQVPKLLIRGGVEVLEVKTGVDSITEVECFLTPEMGDPDEHLRGFSK	60
AEC15447.1	MAPTKRKGERKDPVQVPKLLIRGGVEVLEVKTGVDSITEVECFLTPEMGDPDEHLRGFSK	60
AEC15453.1	MAPTKRKGERKDPVQVPKLLIRGGVEVLEVKTGVDSITEVECFLTPEMGDPDEHLRGFSK	60
AEC15459.1	MAPTKRKGERKDPVQVPKLLIRGGVEVLEVKTGVDSITEVECFLTPEMGDPDEHLRGFSK	60
AEC15465.1	MAPTKRKGERKDPVQVPKLLIRGGVEVLEVKTGVDSITEVECFLTPEMGDPDEHLRGFSK	60
AEC15471.1	MAPTKRKGERKDPVQVPKLLIRGGVEVLEVKTGVDSITEVECFLTPEMGDPDEHLRGFSK	60
AEC15477.1	MAPTKRKGERKDPVQVPKLLIRGGVEVLEVKTGVDSITEVECFLTPEMGDPDEHLRGFSK	60
AEC15483.1	MAPTKRKGERKDPVQVPKLLIRGGVEVLEVKTGVDSITEVECFLTPEMGDPDEHLRGFSK	60
AEC15489.1	MAPTKRKGERKDPVQVPKLLIRGGVEVLEVKTGVDSITEVECFLTPEMGDPDEHLRGFSK	60
AEC15543.1	MAPTKRKGERKDPVQVPKLLIRGGVEVLEVKTGVDSITEVECFLTPEMGDPDEHLRGFSK	60
AEC15119.1	MAPTKRKGERKDPVQVPKLLIRGGVEVLEVKTGVDSITEVECFLTPEMGDPDEHLRGFSK	60
AEC15123.1	MAPTKRKGERKDPVQVPKLLIRGGVEVLEVKTGVDSITEVECFLTPEMGDPDEHLRGFSK	60
AEC15126.1	MAPTKRKGERKDPVQVPKLLIRGGVEVLEVKTGVDSITEVECFLTPEMGDPDEHLRGFSK	60
AEC15132.1	MAPTKRKGERKDPVQVPKLLIRGGVEVLEVKTGVDSITEVECFLTPEMGDPDEHLRGFSK	60
AEC15134.1	MAPTKRKGERKDPVQVPKLLIRGGVEVLEVKTGVDSITEVECFLTPEMGDPDEHLRGFSK	60
AEC15140.1	MAPTKRKGERKDPVQVPKLLIRGGVEVLEVKTGVDSITEVECFLTPEMGDPDEHLRGFSK	60
AEC15221.1	MAPTKRKGERKDPVQVPKLLIRGGVEVLEVKTGVDSITEVECFLTPEMGDPDEHLRGFSK	60
AEC15224.1	MAPTKRKGERKDPVQVPKLLIRGGVEVLEVKTGVDSITEVECFLTPEMGDPDEHLRGFSK	60
AEC15225.1	MAPTKRKGERKDPVQVPKLLIRGGVEVLEVKTGVDSITEVECFLTPEMGDPDEHLRGFSK	60
AEC15233.1	MAPTKRKGERKDPVQVPKLLIRGGVEVLEVKTGVDSITEVECFLTPEMGDPDEHLRGFSK	60
AEC15234.1	MAPTKRKGERKDPVQVPKLLIRGGVEVLEVKTGVDSITEVECFLTPEMGDPDEHLRGFSK	60
AEC15235.1	MAPTKRKGERKDPVQVPKLLIRGGVEVLEVKTGVDSITEVECFLTPEMGDPDEHLRGFSK	60
AEC15241.1	MAPTKRKGERKDPVQVPKLLIRGGVEVLEVKTGVDSITEVECFLTPEMGDPDEHLRGFSK	60
AEC15220.1	MAPTKRKGERKDPVQVPKLLIRGGVEVLEVKTGVDSITEVECFLTPEMGDPDEHLRGFSK	60
AEC15223.1	MAPTKRKGERKDPVQVPKLLIRGGVEVLEVKTGVDSITEVECFLTPEMGDPDEHLRGFSK	60
AEC15232.1	MAPTKRKGERKDPVQVPKLLIRGGVEVLEVKTGVDSITEVECFLTPEMGDPDEHLRGFSK	60
AEC15128.1	MAPTKRKGERKDPVQVPKLLIRGGVEVLEVKTGVDSITEVECFLTPEMGDSDEHLRGFSK	60
AEC15495.1	MAPTKRKGERKDPVQVPKLLIRGGVEVLEVKTGVDSITEVECFLTPEMGDSDEHLRGFSK	60
AEC15501.1	MAPTKRKGERKDPVQVPKLLIRGGVEVLEVKTGVDSITEVECFLTPEMGDSDEHLRGFSK	60
AEC15507.1	MAPTKRKGERKDPVQVPKLLIRGGVEVLEVKTGVDSITEVECFLTPEMGDSDEHLRGFSK	60
AEC15513.1	MAPTKRKGERKDPVQVPKLLIRGGVEVLEVKTGVDSITEVECFLTPEMGDSDEHLRGFSK	60
AEC15519.1	MAPTKRKGERKDPVQVPKLLIRGGVEVLEVKTGVDSITEVECFLTPEMGDSDEHLRGFSK	60
AEC15525.1	MAPTKRKGERKDPVQVPKLLIRGGVEVLEVKTGVDSITEVECFLTPEMGDSDEHLRGFSK	60
AEC15531.1	MAPTKRKGERKDPVQVPKLLIRGGVEVLEVKTGVDSITEVECFLTPEMGDSDEHLRGFSK	60
AEC15537.1	MAPTKRKGERKDPVQVPKLLIRGGVEVLEVKTGVDSITEVECFLTPEMGDSDEHLRGFSK	60
BAF40805.1	MAPTKRKGERKDPVQVPKLLIRGGVEVLEVKTGVDSITEVECFLTPEMGDPDEHLRGFSR	60
AAT09843.1	MAPTKRKGERKDPVQVPKLLIRGGVEVLEVKTGVDSITEVECFLTPEMGDPDEHLRGFSK	60
BAA01965.1	MAPTKRKGERKDPVQVPKLLIRGGVEVLEVKTGVDSITEVECFLTPEMGDPDEHLRGFSK	60
AIW00320.1	MAPTKRKGERKDPVQVPKLLIRGGVEVLEVKTGVDSITEVECFLTPEMGDPDEHLRGFSK	60

ALF06194.1	MAPTKRKGERKDPVQVPKLLIRGGVEVLEVKTGVDSITEVECFLTPEMGDPNEHLRGFSK	60
ALF06211.1	MAPTKRKGERKDPVQVPKLLIRGGVEVLEVKTGVDSITEVECFLTPEMGDPDEHLRGFSK	60
AEC14821.1	MAPTKRKGERKDPVQVPKLLIRGGVEVLEVKTGVDSITEVECFLTPEMGDPDEHLRGFSK	60
AEC14860.1	MAPTKRKGERKDPVQVPKLLIRGGVEVLEVKTGVDSITEVECFLTPEMGDPDEHLRGFSK	60
AEC14861.1	MAPTKRKGERKDPVQVPKLXIRGGVEVLEVKTGVDSITEVECFLTPEMGDPDEHLRGFSK	60
AEC14811.1	MAPTKRKGERKDPVQVPKLLIRGGVEVLEVKTGVDSITEVECFLTPEMGDPDEHLRGFSK	60
AEC14806.1	MTPTKRKGERKDPVQVPKLLIRGGVEVLEVKTGVDSITEVECFLTPEMGDPDEHLRGFSK	60
AEC14795.1	MAPTKRKGERKDPVQVPKLLIRGGVEVLEVKTGVDSITEVECFLTPEMGDPDGHLRGFSK	60
AEC14765.1	MAPTKRKGERKDPVQVPKLLIRGGVEVLEVKTGVDSITEVECFLTPEMGDPDEHLRGFSK	60
AEC14774.1	MAPTKRKGEREDPVQVPKLLIRGGVEVLEVKTGVDSITEVECFLTPEMGDPDEHLRGFSK	60
AEC14761.1	MAPTKRKGERKDPVQVPKLLIRGGVEVLEVKTGVDSITEVECFLTPEMGDPDEHLRGFSK	60
AEC14753.1	MAPTKREGERKDPVQVPKLLIRGGVEVLEVKTGVDSITEVECFLTPEMGDPDEHLRGFSK	60
AEC14767.1	MAPTKRKGERKDPVQVPKLLIRGGVEVLEVKTGVDSITEVECFLTPEMGDPDEHLRGFSK	60
AEC14780.1	MAPTKRKGERKDPVQVPKLLIRGGVEVLEVKTGVDSITEVECFLTPEMGDPDEHLRGFSK	60
AEC14760.1	MAPTKGKGERKDPVQVPKLLIRGGVEVLEVKTGVDSITEVECFLTPEMGDPDEHLRGFSK	60
AEC14762.1	MAPTKGKGERKDPVQVPKLLIRGGVEVLEVKTGVDSITEVECFLTPEMGDPDEHLRGFSK	60
AEC14742.1	MAPTKRKGERKDPVQVPKLLIRGGVEVLEVKTGVDSITEVECFLTPEMGDPDEHLRGFSK	60
AEC14763.1	MAPTKRKGERKDPVQVPKLLIRGGVEVLEVKTGVDSITEVECFLTPEMGDPDEHLRGFSK	60
AEC14810.1	MAPTKRKGERKDPVQVPKLLIRGGVEVLEVKTGVGSITEVECFLTPEMGDPDEHLRGFSK	60
AEC14803.1	MAPTKRKGERKDPVQVPKLLIRGGVEVLEVKTGVDSITEVECFLTPEMGDPDEHLRGFSK	60
AEC14802.1	MAPTKRKGERKDPVQVPKLLIRGGVEVLEVKTGVDSITEVECFLTPEMGDPDEHLRGFSK	60
AEC14812.1	MAPTKRKGERKDPVQVPKLLIRGGVEVLEVKTGVDSITEVECFLTPEMGDPDEHLRGFSK	60
AEC14804.1	MAPTKRKGERKDPVQVPKLLIRGGVEVLEVKTGVDSITEVECFLTPEMGDPDEHLRGFSK	60
AEC14797.1	MAPTKRKGERKDPVQVPKLLIRGGVEVLEVKTGVDSITEVECFLTPEMGDPDEHLRGFSK	60
AEC14796.1	MAPTKRKGERKDPVQVPKLLIRGGVEVLEVKTGVDSITEVECFLTPEMGDPDEHLRGFSK	60
AEC14793.1	MAPTKRKGERKDPVQVPKLLIRGGVEVLEVKTGVDSITEVECFLTPEMGDPDEHLRGFSK	60
AEC14791.1	MAPTKRKGERKDPVQVPKLLIRGGVEVLEVKTGVDSITEVECFLTPEMGDPDEHLRGFSK	60
AEC14794.1	MAPTKRKGERKDPVQVPKLLIRGGVEVLEVKTGVDSITEVECFLTPEMGDPDEHLRGFSK	60
AEC14789.1	MAPTKRKGERKDPVQVPKLLIRGGVEVLEVKTGVDSITEVECFLTPEMGDPDEHLRGFSK	60
AEC14743.1	MAPTKRKGERKDPVQVPKLLIRGGVEVLEVKTGVDSITEVECFLTPEMGDPDEHLRGFSK	60
AEC14745.1	MAPTKRKGERKDPVQVPKLLIRGGVEVLEVKTGVDSITEVECFLTPEMGDPDEHLRGFSK	60
AEC14750.1	MAPTKRKGERKDPVQVPKLLIRGGVEVLEVKTGVDSITEVECFLTPEMGDPDEHLRGFSK	60
AEC14754.1	MAPTKRKGERKDPVQVPKLLIRGGVEVLEVKTGVDSITEVECFLTPEMGDPDEHLRGFSK	60
AEC14751.1	MAPTKRKGERKDPVQVPKLLIRGGVEVLEVKTGVDSITEVECFLTPEMGDPDEHLRGFSK	60
AEC14776.1	MAPTKRKGERKDPVQVPKLLIRGGVEVLEVKTGVDSITEVECFLTPEMGDPDEHLRGFSK	60
AEC14755.1	MAPTKRKGERKDPVQVPKLLIRGGVEVLEVKTGVDSITEVECFLTPEMGDPNEHLRGFSK	60
AEC14759.1	MAPTKRKGERKDPVQVPKLLIRGGVEVLEVKTGVDSITEVECFLTPEMGDPDEHLRGFSK	60
AEC14766.1	MAPTKRKGERKDPVQVPKLLIRGGVEVLEVKTGVDSITEVECFLTPEMGDPDEHLRGFSK	60
AEC14781.1	MAPTKRKGERKDPVQVPKLLIRGGVEVLEVKTGVDSITEVECFLTPEMGDPDEHLRGFSK	60
AEC15186.1	MAPTKRKGERKDPVQVPKLLIRGGVEVLEVKTGVDSITEVECFLTPEMGDPDEHLRGFSK	60
AEC15192.1	MAPTKRKGERKDPVQVPKLLIRGGVEVLEVKTGVDSITEVECFLTPEMGDPDEHLRGFSK	60
AEC15194.1	MAPTKRKGERKDPVQVPKLLIRGGVEVLEVKTGVDSITEVECFLTPEMGDPDEHLRGFSK	60
AEC15195.1	MAPTKRKGERKDPVQVPKLLIRGGVEVLEVKTGVDSITEVECFLTPEMGDPDEHLRGFSK	60
QWT77104.1	MAPTKRKGERKDPVQVPKLLIRGGVEVLEVKTGVDSITEVECFLTPEMGDPDEHLRGFSK	60
UPN63088.1	MAPTKRKGERKDPVQVPKLLIRGGVEVLEVKTGVDSITEVECFLTPEMGDPDEHLRGFSK	60
UPN63104.1	MAPTKRKGERKDPVQVPKLLIRGGVEVLEVKTGVDSITEVECFLTPEMGDPDEHFRGFSK	60
UPN63112.1	MAPTKRKGERKDPVQVPKLLIRGGVEVLEVKTGVDSITEVECFLTPEMGDPDEHFRGFSK	60
UPN63113.1	MAPTKRKGERKDPVQVPKLLIRGGVEVLEVKTGVDSITEVERFLTPEMGDPDEHFRGFSK	60
UPN63114.1	MAPTKRKGERKDPVQVPKLLIRGGVEVLEVKTGVDSITEVECFLTPEMGDPDEHFRGFSK	60
UPN63115.1	MAPTKRKGERKDPVQVPKLLIRGGVEVLEVKTGVDSITEVECFLTPEMGDPDEHFRGFSK	60
AEC15172.1	MAPTKRKGERKDPVQVPKLLIRGGVEVLEVKTGVDSITEVECFLTPEMGDPDEHLRGFSK	60
BAE00117.1	MAPTKRKGERKDPVQVPKLLIRGGVEVLEVKTGVDSITEVECFLTPEMGDPDEHLRGFSK	60
BAE00123.1	MAPTKRKGERKDPVQVPKLLIRGGVEVLEVKTGVDSITEVECFLTPEMGDPDEHLRGFSK	60
AAC59331.1	MAPTKRKGERKDPVQVPKLLIRGGVEVLEVKTGVDSITEVECFLTPEMGDPDEHLRGFSK	60
WLD48106.1	MAPTKRKGERKDPVQVPKLLIRGGVEVLEVKTGVDSITEVECFLTPEMGDPDEHLRGFSK	60
UQK62661.1	MAPTKRKGERKDPVQVPKLLIRGGVEVLEVKTGVDSITEVECFLTPEMGDPDEHLRGFSK	60
QTJ15156.1	MAPTKRKGERKDPVQVPKLLIRGGVEVLEVKTGVDSITEVECFLTPEMGDPDEHLRGFSK	60
QNC68213.1	MAPTKRKGERKDPVQVPKLLIRGGVEVLEVKTGVDSITEVECFLTPEMGDPNEHLRGFSK	60
ASV51766.1	MAPTKRKGERKDPVQVPKLLIRGGVEVLEVKTGVDSITEVECFLTPEMGDPDEHLRGFSK	60
ASV51796.1	MAPTKRKGERKDPVQVPKLLIRGGVEVLEVKTGVDSITEVECFLTPEMGDPDEHLRGFSK	60
ASV51802.1	MAPTKRKGERKDPVQVPKLLIRGGVEVLEVKTGVDSITEVECFLTPEMGDPDEHLRGFSK	60
ASV51808.1	MAPTKRKGERKDPVQVPKLLIRGGVEVLEVKTGVDSITEVECFLTPEMGDPDEHLRGFSK	60
ASV51820.1	MAPTKRKGERKDPVQVPKLLIRGGVEVLEVKTGVDSITEVECFLTPEMGDPDEHLRGFSK	60
ASV51826.1	MAPTKRKGERKDPVQVPKLLIRGGVEVLEVKTGVDSITEVECFLTPEMGDPDEHLRGFSK	60

```
ASV51832.1    MAPTKRKGERKDPVQVPKLLIRGGVEVLEVKTGVDSITEVECFLTPEMGDPDEHLRGFSK  60
ASV51838.1    MAPTKRKGERKDPVQVPKLLIRGGVEVLEVKTGVDSITEVECFLTPEMGDPDEHLRGFSK  60
ASV51862.1    MAPTKRKGERKDPVQVPKLLIRGGVEVLEVKTGVDSITEVECFLTPEMGDPDEHLRGFSK  60
ASV51868.1    MAPTKRKGERKDPVQVPKLLIRGGVEVLEVKTGVDSITEVECFLTPEMGDPDEHLRGFSK  60
ASV51874.1    MAPTKRKGERKDPVQVPKLLIRGGVEVLEVKTGVDSITEVECFLTPEMGDPDEHLRGFSK  60
ASV51880.1    MAPTKRKGERKDPVQVPKLLIRGGVEVLEVKTGVDSITEVECFLTPEMGDPDEHLRGFSK  60
ASV51892.1    MAPTKRKGERKDPVQVPKLLIRGGVEVLEVKTGVDSITEVECFLTPEMGDPDEHLRGFSK  60
ANH21098.1    MAPTKRKGERKDPVQVPKLLIRGGVEVLEVKTGVDSITEVECFLTPEMGDPDEHLRGFSK  60
AID67150.1    MAPTKRKGERKDPVQVPKLLIRGGVEVLEVKTGVDSITEVECFLTPEMGDPDEHLRGFSK  60
AID67156.1    MAPTKRKGERKDPVQVPKLLIRGGVEVLEVKTGVDSITEVECFLTPEMGDPDEHLRGFSK  60
AID67162.1    MAPTKRKGERKDPVQVPKLLIRGGVEVLEVKTGVDSITEVECFLTPEMGDPDEHLRGFSK  60
AID67168.1    MAPTKRKGERKDPVQVPKLLIRGGVEVLEVKTGVDSITEVECFLTPEMGDPDEHLRGFSK  60
CCW72479.1    MAPTKRKGERKDPVQVPKLLIRGGVEVLEVKTGVDSITEVECFLTPEMGDPDEHLRGFSK  60
CCW72486.1    MAPTKRKGERKDPVQVPKLLIRGGVEVLEVKTGVDSITEVECFLTPEMGDPDEHLRGFSK  60
CCW72494.1    MAPTKRKGERKDPVQVPKLLIRGGVEVLEVKTGVDSITEVECFLTPEMGDPDEHLRGFSK  60
CCW72514.1    MAPTKRKGERKDPVQVPKLLIRGGVEVLEVKTGVDSITEVECFLTPEMGDPDEHLRGFSK  60
CCW72518.1    MAPTKRKGERKDPVQVPKLLIRGGVEVLEVKTGVDSITEVECFLTPEMGDPDEHLRGFSK  60
CCW72522.1    MAPTKRKGERKDPVQVPKLLIRGGVEVLEVKTGVDSITEVECFLTPEMGDPDEHLRGFSK  60
AFH57194.1    MAPTKRKGERKDPVQVPKLLIRGGVEVLEVKTGVDSITEVECFLTPEMGDPDEHLRGFSK  60
AEC15047.1    MAPTKRKGERKDPVQVPKLLIRGGVEVLEVKTGVDSITEVECFLTPEMGDPDEHLRGFSK  60
AEC15048.1    MAPTKRKGERKDPVQVPKLLIRGGVEVLEVKTGVDSITEVECFLTPEMGDPDEHLRGFSK  60
AEC15049.1    MAPTKRKGERKDPVQVPKLLIRGGVEVLEVKTGVDSITEVECFLTPEMGDPDEHLRGFSK  60
AEC15050.1    MAPTKRKGERKDPVQVPKLLIRGGVEVLEVKTGVDSITEVECFLTPEMGDPDEHLRGFSK  60
AEC15051.1    MAPTKRKGERKDPVQVPKLLIRGGVEVLEVKTGVDSITEVECFLTPEMGDPDEHLRGFSK  60
AEC15052.1    MAPTKRKGERKDPVQVPKLLIRGGVEVLEVKTGVDSITEVECFLTPEMGDPDEHLRGFSK  60
AEC15053.1    MAPTKRKGERKDPVQVPKLLIRGGVEVLEVKTGVDSITEVECFLTPEMGDPDEHLRGFSK  60
AEC15054.1    MAPTKRKGERKDPVQVPKLLIRGGVEVLEVKTGVDSITEVECFLTPEMGDPDEHLRGFSK  60
AEC15055.1    MAPTKRKGERKDPVQVPKLLIRGGVEVLEVKTGVDSITEVECFLTPEMGDPDEHLRGFSK  60
AEC15056.1    MAPTKRKGERKDPVQVPKLLIRGGVEVLEVKTGVDSITEVECFLTPEMGDPDEHLRGFSK  60
AEC15057.1    MAPTKRKGERKDPVQVPKLLIRGGVEVLEVKTGVDSITEVECFLTPEMGDPDEHLRGFSK  60
AEC15058.1    MAPTKRKGERKDPVQVPKLLIRGGVEVLEVKTGVDSITEVECFLTPEMGDPDEHLRGFSK  60
AEC15059.1    MAPTKRKGERKDPVQVPKLLIRGGVEVLEVKTGVDSITEVECFLTPEMGDPDEHLRGFSK  60
AEC15060.1    MAPTKRKGERKDPVQVPKLLIRGGVEVLEVKTGVDSITEVECFLTPEMGDPDEHLRGFSK  60
AEC15061.1    MAPTKRKGERKDPVQVPKLLIRGGVEVLEVKTGVDSITEVECFLTPEMGDPDEHLRGFSK  60
AEC15062.1    MAPTKRKGERKDPVQVPKLLIRGGVEVLEVKTGVDSITEVECFLTPEMGDPDEHLRGFSK  60
AEC15063.1    MAPTKRKGERKDPVQVPKLLIRGGVEVLEVKTGVDSITEVECFLTPEMGDPDEHLRGFSK  60
AEC15064.1    MAPTKRKGERKDPVQVPKLLIRGGVEVLEVKTGVDSITEVECFLTPEMGDPDEHLRGFSK  60
AEC15065.1    MAPTKRKGERKDPVQVPKLLIRGGVEVLEVKTGVDSITEVECFLTPEMGDPDEHLRGFSK  60
AEC15066.1    MAPTKRKGERKDPVQVPKLLIRGGVEVLEVKTGVDSITEVECFLTPEMGDPDEHLRGFSK  60
AEC15067.1    MAPTKRKGERKDPVQVPKLLIRGGVEVLEVKTGVDSITEVECFLTPEMGDPDEHLRGFSK  60
AEC15068.1    MAPTKRKGERKDPVQVPKLLIRGGVEVLEVKTGVDSITEVECFLTPEMGDPDEHLRGFSK  60
AEC15315.1    MAPTKRKGERKDPVQVPKLLIRGGVEVLEVKTGVDSITEVECFLTPEMGDPDEHLRGFSK  60
AEC15321.1    MAPTKRKGERKDPVQVPKLLIRGGVEVLEVKTGVDSITEVECFLTPEMGDPDEHLRGFSK  60
BAD21271.1    MAPTKRKGERKDPVQVPKLLIRGGVEVLEVKTGVDSITEVECFLTPEMGDPDEHLRGFSK  60
BAD21283.2    MAPTKRKGERKDPVQVPKLLIRGGVEVLEVKTGVDSITEVECFLTPEMGDPDEHLRGFSK  60
BAC15683.1    MAPTKRKGERKDPVQVPKLLIRGGVEVLEVKTGVDSITEVECFLTPEMGDPDEHLRGFSK  60
BAC15719.1    MAPTKRKGERKDPVQVPKLLIRGGVEVLEVKTGVDSITEVECFLTPEMGDPDEHLRGFSK  60
BAB93038.1    MAPTKRKGERKDPVQVPKLLIRGGVEVLEVKTGVDSITEVECFLTPEMGDPDEHLRGFSK  60
BAB93056.1    MAPTKRKGERKDPVQVPKLLIRGGVEVLEVKTGVDSITEVECFLTPEMGDPDEHLRGFSK  60
BAB93062.1    MAPTKRKGERKDPVQVPKLLIRGGVEVLEVKTGVDSITEVECFLTPEMGDPDEHLRGFSK  60
BAB68911.1    MAPTKRKGERKDPVQVPKLLIRGGVEVLEVKTGVDSITEVECFLTPEMGDPDEHLRGFSK  60
BAB68947.1    MAPTKRKGERKDPVQVPKLLIRGGVEVLEVKTGVDSITEVECFLTPEMGDPDEHLRGFSK  60
AAK70234.1    MAPTKRKGERKDPVQVPKLLIRGGVEVLEVKTGVDSITEVECFLTPEMGDPDEHLRGFSK  60
AAK70246.1    MAPTKRKGERKDPVQVPKLLIRGGVEVLEVKTGVDSITEVECFLTPEMGDPDEHLRGFSK  60
BAA01959.1    MAPTKRKGERKDPVQVPKLLIRGGVEVLEVKTGVDSITEVECFLTPEMGDPDEHLRGFSK  60
BAA01960.1    MAPTKRKGERKDPVQVPKLLIRGGVEVLEVKTGVDSITEVECFLTPEMGDPDEHLRGFSK  60
QWT77128.1    MAPTKRKGERKDPVQVPKLLIRGGVEVLEVKTGVDSITEVECFLTPEMGDPDEHFRGFSK  60
UPN63089.1    MAPTKRKGERKDPVQVPKLLIRGGVEVLEVKTGVDSITEVECFLTPEMGDPDEHFRGFSK  60
UPN63093.1    MAPTKRKGERKDPVQVPKLLIRGGVEVLEVKTGVDSITEVECFLTPEMGDPDEHFRGFSK  60
UPN63103.1    MAPTKRKGERKDPVQVPKLLIRGGVEVLEVKTGVDSITEVECFLTPEMGDPDEHFRGFSK  60
UPN63105.1    MAPTKRKGERKDPVQVPKLLIRGGVEVLEVKTGVDSITEVECFLTPEMGDPDEHFRGFSK  60
UPN63106.1    MAPTKRKGERKDPVQVPKLLIRGGVEVLEVKTGVDSITEVECFLTPEMGDPDEHFRGFSK  60
UPN63110.1    MAPTKRKGERKDPVQVPKLLIRGGVEVLEVKTGVDSITEVECFLTPEMGDPDEHFRGFSK  60
UPN63111.1    MAPTKRKGERKDPVQVPKLLIRGGVEVLEVKTGVDSITEVECFLTPEMGDPDEHFRGFSK  60
```

AEC15030.1	MAPTKRKGERKDPVQVPKLLIRGGVEVLEVKTGVDSITEVECFLTPEMGDPDEHFRGFSK	60
AEC15031.1	MAPTKRKGERKDPVQVPKLLIRGGVEVLEVKTGVDSITEVECFLTPEMGDPDEHFRGFSK	60
AEC15032.1	MAPTKRKGERKDPVQVPKLLIRGGVEVLEVKTGVDSITEVECFLTPEMGDPDEHFRGFSK	60
AEC15033.1	MAPTKRKGERKDPVQVPKLLIRGGVEVLEVKTGVDSITEVECFLTPEMGDPDEHFRGFSK	60
AEC15034.1	MAPTKRKGERKDPVQVPKLLIRGGVEVLEVKTGVDSITEVECFLTPEMGDPDEHFRGFSK	60
AEC15035.1	MAPTKRKGERKDPVQVPKLLIRGGVEVLEVKTGVDSITEVECFLTPEMGDPDEHFRGFSK	60
AEC15036.1	MAPTKRKGERKDPVQVPKLLIRGGVEVLEVKTGVDSITEVECFLTPEMGDPDEHFRGFSK	60
AEC15038.1	MAPTKRKGERKDPVQVPKLLIRGGVEVLEVKTGVDSITEVECFLTPEMGDPDEHFRGFSK	60
AEC15039.1	MAPTKRKGERKDPVQVPKLLIRGGVEVLEVKTGVDSITEVECFLTPEMGDPDEHFRGFSK	60
AEC15040.1	MAPTKRKGERKDPVQVPKLLIRGGVEVLEVKTGVDSITEVECFLTPEMGDPDEHFRGFSK	60
AEC15041.1	MAPTKRKGERKDPVQVPKLLIRGGVEVLEVKTGVDSITEVECFLTPEMGDPDEHFRGFSK	60
AEC15042.1	MAPTKRKGERKDPVQVPKLLIRGGVEVLEVKTGVDSITEVECFLTPEMGDPDEHFRGFSK	60
AEC15043.1	MAPTKRKGERKDPVQVPKLLIRGGVEVLEVKTGVDSITEVECFLTPEMGDPDEHFRGFSK	60
AEC15044.1	MAPTKRKGERKDPVQVPKLLIRGGVEVLEVKTGVDSITEVECFLTPEMGDPDEHFRGFSK	60
AEC15045.1	MAPTKRKGERKDPVQVPKLLIRGGVEVLEVKTGVDSITEVECFLTPEMGDPDEHFRGFSK	60
AEC15046.1	MAPTKRKGERKDPVQVPKLLIRGGVEVLEVKTGVDSITEVECFLTPEMGDPDEHFRGFSK	60
UPN63083.1	MAPTKRKGERKDPVQVPKLLIRGGVEVLEVKTGVDSITEVECFLTPEMGDPDEHHRGFSK	60
BAE00111.1	MAPTKRKGERKDPVQVPKLLIRGGVEVLEVKTGVDSITEVECFLTPEMGDPDEHLRGFSK	60
BAA01968.1	MAPTKRKGERKDPVQVPKLLIRGGVEVLEVKTGVDSITEVECFLTPEMGDPDEHLRGFSK	60
CCW72491.1	MAPTKRKGERKDPVQVPKLLIRGGVEVLEVKTGVDSITEVECFLTPEMGDPDEHLRGFSK	60
AEC14788.1	MAPTKRKGERKDPVQVPKLLIRGGVEVLEVKTGVDSITEVECFLTPEMGDPDEHLRGFSK	60
AEC14790.1	MAPTKRKGERKDPVQVPKLLIRGGVEVLEVKTGVDSITEVECFLTPEMGDPDEHLRGFSK	60
AEC14792.1	MAPTKRKGERKDPVQVPKLLIRGGVEVLEVKTGVDSITEVECFLTPEMGDPDEHLRGFSK	60
AEC14798.1	MAPTKRKGERKDPVQVPKLLIRGGVEVLEVKTGVDSITEVECFLTPEMGDPDEHLRGFSK	60
AEC14799.1	MAPTKRKGERKDPVQVPKLLIRGGVEVLEVKTGVDSITEVECFLTPEMGDPDEHLRGFSK	60
AEC14800.1	MAPTKRKGERKDPVQVPKLLIRGGVEVLEVKTGVDSITEVECFLTPEMGDPDEHLRGFSK	60
AEC14801.1	MAPTKRKGERKDPVQVPKLLIRGGVEVLEVKTGVDSITEVECFLTPEMGDPDEHLRGFSK	60
AEC14805.1	MAPTKRKGERKDPVQVPKLLIRGGVEVLEVKTGVDSITEVECFLTPEMGDPDEHLRGFSK	60
AEC14807.1	MAPTKRKGERKDPVQVPKLLIRGGVEVLEVKTGVDSITEVECFLTPEMGDPDEHLRGFSK	60
AEC14808.1	MAPTKRKGERKDPVQVPKLLIRGGVEVLEVKTGVDSITEVECFLTPEMGDPDEHLRGFSK	60
AEC14809.1	MAPTKRKGERKDPVQVPKLLIRGGVEVLEVKTGVDSITEVECFLTPEMGDPDEHLRGFSK	60
AEC14813.1	MAPTKRKGERKDPVQVPKLLIRGGVEVLEVKTGVDSITEVECFLTPEMGDPDEHLRGFSK	60
AEC14912.1	MAPTKRKGERKDPVQVPKLLIRGGVEVLEVKTGVDSITEVECFLTPEMGDPDEHLRGFSK	60
AEC14913.1	MAPTKRKGERKDPVQVPKLLIRGGVEVLEVKTGVDSITEVECFLTPEMGDPDEHLRGFSK	60
AEC14914.1	MAPTKRKGERKDPVQVPKLLIRGGVEVLEVKTGVDSITEVECFLTPEMGDPDEHLRGFSK	60
AEC14915.1	MAPTKRKGERKDPVQVPKLLIRGGVEVLEVKTGVDSITEVECFLTPEMGDPDEHLRGFSK	60
AEC14916.1	MAPTKRKGERKDPVQVPKLLIRGGVEVLEVKTGVDSITEVECFLTPEMGDPDEHLRGFSK	60
AEC14917.1	MAPTKRKGERKDPVQVPKLLIRGGVEVLEVKTGVDSITEVECFLTPEMGDPDEHLRGFSK	60
AEC14918.1	MAPTKRKGERKDPVQVPKLLIRGGVEVLEVKTGVDSITEVECFLTPEMGDPDEHLRGFSK	60
AEC14919.1	MAPTKRKGERKDPVQVPKLLIRGGVEVLEVKTGVDSITEVECFLTPEMGDPDEHLRGFSK	60
AEC14920.1	MAPTKRKGERKDPVQVPKLLIRGGVEVLEVKTGVDSITEVECFLTPEMGDPDEHLRGFSK	60
AEC14921.1	MAPTKRKGERKDPVQVPKLLIRGGVEVLEVKTGVDSITEVECFLTPEMGDPDEHLRGFSK	60
AEC14922.1	MAPTKRKGERKDPVQVPKLLIRGGVEVLEVKTGVDSITEVECFLTPEMGDPDEHLRGFSK	60
AEC14923.1	MAPTKRKGERKDPVQVPKLLIRGGVEVLEVKTGVDSITEVECFLTPEMGDPDEHLRGFSK	60
AEC14924.1	MAPTKRKGERKDPVQVPKLLIRGGVEVLEVKTGVDSITEVECFLTPEMGDPDEHLRGFSK	60
AEC14925.1	MAPTKRKGERKDPVQVPKLLIRGGVEVLEVKTGVDSITEVECFLTPEMGDPDEHLRGFSK	60
AEC14926.1	MAPTKRKGERKDPVQVPKLLIRGGVEVLEVKTGVDSITEVECFLTPEMGDPDEHLRGFSK	60
AEC14927.1	MAPTKRKGERKDPVQVPKLLIRGGVEVLEVKTGVDSITEVECFLTPEMGDPDEHLRGFSK	60
AEC14928.1	MAPTKRKGERKDPVQVPKLLIRGGVEVLEVKTGVDSITEVECFLTPEMGDPDEHLRGFSK	60
AEC14929.1	MAPTKRKGERKDPVQVPKLLIRGGVEVLEVKTGVDSITEVECFLTPEMGDPDEHLRGFSK	60
AEC14930.1	MAPTKRKGERKDPVQVPKLLIRGGVEVLEVKTGVDSITEVECFLTPEMGDPDEHLRGFSK	60
AEC14931.1	MAPTKRKGERKDPVQVPKLLIRGGVEVLEVKTGVDSITEVECFLTPEMGDPDEHLRGFSK	60
AEC14932.1	MAPTKRKGERKDPVQVPKLLIRGGVEVLEVKTGVDSITEVECFLTPEMGDPDEHLRGFSK	60
AEC14933.1	MAPTKRKGERKDPVQVPKLLIRGGVEVLEVKTGVDSITEVECFLTPEMGDPDEHLRGFSK	60
AEC14934.1	MAPTKRKGERKDPVQVPKLLIRGGVEVLEVKTGVDSITEVECFLTPEMGDPDEHLRGFSK	60
AEC14935.1	MAPTKRKGERKDPVQVPKLLIRGGVEVLEVKTGVDSITEVECFLTPEMGDPDEHLRGFSK	60
AEC14936.1	MAPTKRKGERKDPVQVPKLLIRGGVEVLEVKTGVDSITEVECFLTPEMGDPDEHLRGFSK	60
AEC14937.1	MAPTKRKGERKDPVQVPKLLIRGGVEVLEVKTGVDSITEVECFLTPEMGDPDEHLRGFSK	60
AEC14938.1	MAPTKRKGERKDPVQVPKLLIRGGVEVLEVKTGVDSITEVECFLTPEMGDPDEHLRGFSK	60
AEC14939.1	MAPTKRKGERKDPVQVPKLLIRGGVEVLEVKTGVDSITEVECFLTPEMGDPDEHLRGFSK	60
AEC14940.1	MAPTKRKGERKDPVQVPKLLIRGGVEVLEVKTGVDSITEVECFLTPEMGDPDEHLRGFSK	60
AEC14941.1	MAPTKRKGERKDPVQVPKLLIRGGVEVLEVKTGVDSITEVECFLTPEMGDPDEHLRGFSK	60
AEC14942.1	MAPTKRKGERKDPVQVPKLLIRGGVEVLEVKTGVDSITEVECFLTPEMGDPDEHLRGFSK	60
AEC14943.1	MAPTKRKGERKDPVQVPKLLIRGGVEVLEVKTGVDSITEVECFLTPEMGDPDEHLRGFSK	60

```
AEC14944.1        MAPTKRKGERKDPVQVPKLLIRGGVEVLEVKTGVDSITEVECFLTPEMGDPDEHLRGFSK  60
AEC14945.1        MAPTKRKGERKDPVQVPKLLIRGGVEVLEVKTGVDSITEVECFLTPEMGDPDEHLRGFSK  60
AEC14946.1        MAPTKRKGERKDPVQVPKLLIRGGVEVLEVKTGVDSITEVECFLTPEMGDPDEHLRGFSK  60
AEC14947.1        MAPTKRKGERKDPVQVPKLLIRGGVEVLEVKTGVDSITEVECFLTPEMGDPDEHLRGFSK  60
AEC14948.1        MAPTKRKGERKDPVQVPKLLIRGGVEVLEVKTGVDSITEVECFLTPEMGDPDEHLRGFSK  60
AEC14949.1        MAPTKRKGERKDPVQVPKLLIRGGVEVLEVKTGVDSITEVECFLTPEMGDPDEHLRGFSK  60
AEC14950.1        MAPTKRKGERKDPVQVPKLLIRGGVEVLEVKTGVDSITEVECFLTPEMGDPDEHLRGFSK  60
AEC14951.1        MAPTKRKGERKDPVQVPKLLIRGGVEVLEVKTGVDSITEVECFLTPEMGDPDEHLRGFSK  60
AEC14952.1        MAPTKRKGERKDPVQVPKLLIRGGVEVLEVKTGVDSITEVECFLTPEMGDPDEHLRGFSK  60
AEC14953.1        MAPTKRKGERKDPVQVPKLLIRGGVEVLEVKTGVDSITEVECFLTPEMGDPDEHLRGFSK  60
AEC14954.1        MAPTKRKGERKDPVQVPKLLIRGGVEVLEVKTGVDSITEVECFLTPEMGDPDEHLRGFSK  60
AEC14955.1        MAPTKRKGERKDPVQVPKLLIRGGVEVLEVKTGVDSITEVECFLTPEMGDPDEHLRGFSK  60
AEC14956.1        MAPTKRKGERKDPVQVPKLLIRGGVEVLEVKTGVDSITEVECFLTPEMGDPDEHLRGFSK  60
AEC14957.1        MAPTKRKGERKDPVQVPKLLIRGGVEVLEVKTGVDSITEVECFLTPEMGDPDEHLRGFSK  60
AEC14958.1        MAPTKRKGERKDPVQVPKLLIRGGVEVLEVKTGVDSITEVECFLTPEMGDPDEHLRGFSK  60
AEC14959.1        MAPTKRKGERKDPVQVPKLLIRGGVEVLEVKTGVDSITEVECFLTPEMGDPDEHLRGFSK  60
AEC14960.1        MAPTKRKGERKDPVQVPKLLIRGGVEVLEVKTGVDSITEVECFLTPEMGDPDEHLRGFSK  60
AEC14961.1        MAPTKRKGERKDPVQVPKLLIRGGVEVLEVKTGVDSITEVECFLTPEMGDPDEHLRGFSK  60
QWT77122.1        MAPTKRKGERKDPVQVPKLLIRGGVEVLEVKTGVDSITEVECFLTPEMGDPDEHLRGFSK  60
UPN63084.1        MAPTKRKGERKDPVQVPKLLIRGGVEVLEVKTGVDSITEVECFLTPEMGDPDEHLRGFSK  60
UPN63101.1        MAPTKRKGERKDPVQVPKLLIRGGVEVLEVKTGVDSITEVECFLTPEMGDPDEHLRGFSK  60
ASV51886.1        MAPTKRKGERKDPVQVPKLLIRGGVEVLEVKTGVDSITEVECFLTPEMGDPNEHLRGFSK  60
CCW72484.1        MAPTKRKGERKDPVQVPKLLIRGGVEVLEVKTGVDSITEVECFLTPEMGDPNEHLRGFSK  60
ANH21099.1        MAPTKRKGERKDPVQVPKLLIRGGVEVLEVKTGVDSITEVECFLTPEMGDPDEHLRGFSK  60
AEC15112.1        MAPTKRKGERKDPVQVPKLLIRGGVEVLEVKTGVDSITEVECFLTPEMGDPDEHLRGFSK  60
AEC15113.1        MAPTKRKGERKDPVQVPKLLIRGGVEVLEVKTGVDSITEVECFLTPEMGDPDEHLRGFSK  60
AEC15114.1        MAPTKRKGERKDPVQVPKLLIRGGVEVLEVKTGVDSITEVECFLTPEMGDPDEHLRGFSK  60
AEC15115.1        MAPTKRKGERKDPVQVPKLLIRGGVEVLEVKTGVDSITEVECFLTPEMGDPDEHLRGFSK  60
AEC15116.1        MAPTKRKGERKDPVQVPKLLIRGGVEVLEVKTGVDSITEVECFLTPEMGDPDEHLRGFSK  60
AEC15117.1        MAPTKRKGERKDPVQVPKLLIRGGVEVLEVKTGVDSITEVECFLTPEMGDPDEHLRGFSK  60
AEC15819.1        MAPTKRKGERKDPVQVPKLLIRGGVEVLEVKTGVDSITEVECFLTPEMGDPDEHLRGFSK  60
AEC15825.1        MAPTKRKGERKDPVQVPKLLIRGGVEVLEVKTGVDSITEVECFLTPEMGDPDEHLRGFSK  60
AEC15831.1        MAPTKRKGERKDPVQVPKLLIRGGVEVLEVKTGVDSITEVECFLTPEMGDPDEHLRGFSK  60
AEC15837.1        MAPTKRKGERKDPVQVPKLLIRGGVEVLEVKTGVDSITEVECFLTPEMGDPDEHLRGFSK  60
AEC15843.1        MAPTKRKGERKDPVQVPKLLIRGGVEVLEVKTGVDSITEVECFLTPEMGDPDEHLRGFSK  60
AEC15849.1        MAPTKRKGERKDPVQVPKLLIRGGVEVLEVKTGVDSITEVECFLTPEMGDPDEHLRGFSK  60
AEC15855.1        MAPTKRKGERKDPVQVPKLLIRGGVEVLEVKTGVDSITEVECFLTPEMGDPDEHLRGFSK  60
CCW72511.1        MAPTKRKGERKDPVQVPKLLIRGGVEVLEVKTGVDSITEVECFLTPEMGDPDEHLRGFSK  60
CCW72525.1        MAPTKRKGERKDPVQVPKLLIRGGVEVLEVKTGVDSITEVECFLTPEMGDPDEHLRGFSK  60
AEC14744.1        MAPTKRKGERKDPVQVPKLLIRGGVEVLEVKTGVDSITEVECFLTPEMGDPDEHLRGFSK  60
AEC14746.1        MAPTKRKGERKDPVQVPKLLIRGGVEVLEVKTGVDSITEVECFLTPEMGDPDEHLRGFSK  60
AEC14747.1        MAPTKRKGERKDPVQVPKLLIRGGVEVLEVKTGVDSITEVECFLTPEMGDPDEHLRGFSK  60
AEC14748.1        MAPTKRKGERKDPVQVPKLLIRGGVEVLEVKTGVDSITEVECFLTPEMGDPDEHLRGFSK  60
AEC14752.1        MAPTKRKGERKDPVQVPKLLIRGGVEVLEVKTGVDSITEVECFLTPEMGDPDEHLRGFSK  60
AEC14756.1        MAPTKRKGERKDPVQVPKLLIRGGVEVLEVKTGVDSITEVECFLTPEMGDPDEHLRGFSK  60
AEC14757.1        MAPTKRKGERKDPVQVPKLLIRGGVEVLEVKTGVDSITEVECFLTPEMGDPDEHLRGFSK  60
AEC14758.1        MAPTKRKGERKDPVQVPKLLIRGGVEVLEVKTGVDSITEVECFLTPEMGDPDEHLRGFSK  60
AEC14764.1        MAPTKRKGERKDPVQVPKLLIRGGVEVLEVKTGVDSITEVECFLTPEMGDPDEHLRGFSK  60
AEC14768.1        MAPTKRKGERKDPVQVPKLLIRGGVEVLEVKTGVDSITEVECFLTPEMGDPDEHLRGFSK  60
AEC14769.1        MAPTKRKGERKDPVQVPKLLIRGGVEVLEVKTGVDSITEVECFLTPEMGDPDEHLRGFSK  60
AEC14770.1        MAPTKRKGERKDPVQVPKLLIRGGVEVLEVKTGVDSITEVECFLTPEMGDPDEHLRGFSK  60
AEC14771.1        MAPTKRKGERKDPVQVPKLLIRGGVEVLEVKTGVDSITEVECFLTPEMGDPDEHLRGFSK  60
AEC14772.1        MAPTKRKGERKDPVQVPKLLIRGGVEVLEVKTGVDSITEVECFLTPEMGDPDEHLRGFSK  60
AEC14773.1        MAPTKRKGERKDPVQVPKLLIRGGVEVLEVKTGVDSITEVECFLTPEMGDPDEHLRGFSK  60
AEC14775.1        MAPTKRKGERKDPVQVPKLLIRGGVEVLEVKTGVDSITEVECFLTPEMGDPDEHLRGFSK  60
AEC14777.1        MAPTKRKGERKDPVQVPKLLIRGGVEVLEVKTGVDSITEVECFLTPEMGDPDEHLRGFSK  60
AEC14778.1        MAPTKRKGERKDPVQVPKLLIRGGVEVLEVKTGVDSITEVECFLTPEMGDPDEHLRGFSK  60
AEC14779.1        MAPTKRKGERKDPVQVPKLLIRGGVEVLEVKTGVDSITEVECFLTPEMGDPDEHLRGFSK  60
AEC14782.1        MAPTKRKGERKDPVQVPKLLIRGGVEVLEVKTGVDSITEVECFLTPEMGDPDEHLRGFSK  60
AEC14814.1        MAPTKRKGERKDPVQVPKLLIRGGVEVLEVKTGVDSITEVECFLTPEMGDPDEHLRGFSK  60
AEC14815.1        MAPTKRKGERKDPVQVPKLLIRGGVEVLEVKTGVDSITEVECFLTPEMGDPDEHLRGFSK  60
AEC14816.1        MAPTKRKGERKDPVQVPKLLIRGGVEVLEVKTGVDSITEVECFLTPEMGDPDEHLRGFSK  60
AEC14817.1        MAPTKRKGERKDPVQVPKLLIRGGVEVLEVKTGVDSITEVECFLTPEMGDPDEHLRGFSK  60
AEC14818.1        MAPTKRKGERKDPVQVPKLLIRGGVEVLEVKTGVDSITEVECFLTPEMGDPDEHLRGFSK  60
```

AEC14819.1	MAPTKRKGERKDPVQVPKLLIRGGVEVLEVKTGVDSITEVECFLTPEMGDPDEHLRGFSK	60
AEC14820.1	MAPTKRKGERKDPVQVPKLLIRGGVEVLEVKTGVDSITEVECFLTPEMGDPDEHLRGFSK	60
AEC14822.1	MAPTKRKGERKDPVQVPKLLIRGGVEVLEVKTGVDSITEVECFLTPEMGDPDEHLRGFSK	60
AEC14823.1	MAPTKRKGERKDPVQVPKLLIRGGVEVLEVKTGVDSITEVECFLTPEMGDPDEHLRGFSK	60
AEC14824.1	MAPTKRKGERKDPVQVPKLLIRGGVEVLEVKTGVDSITEVECFLTPEMGDPDEHLRGFSK	60
AEC14825.1	MAPTKRKGERKDPVQVPKLLIRGGVEVLEVKTGVDSITEVECFLTPEMGDPDEHLRGFSK	60
AEC14826.1	MAPTKRKGERKDPVQVPKLLIRGGVEVLEVKTGVDSITEVECFLTPEMGDPDEHLRGFSK	60
AEC14827.1	MAPTKRKGERKDPVQVPKLLIRGGVEVLEVKTGVDSITEVECFLTPEMGDPDEHLRGFSK	60
AEC14828.1	MAPTKRKGERKDPVQVPKLLIRGGVEVLEVKTGVDSITEVECFLTPEMGDPDEHLRGFSK	60
AEC14829.1	MAPTKRKGERKDPVQVPKLLIRGGVEVLEVKTGVDSITEVECFLTPEMGDPDEHLRGFSK	60
AEC14830.1	MAPTKRKGERKDPVQVPKLLIRGGVEVLEVKTGVDSITEVECFLTPEMGDPDEHLRGFSK	60
AEC14831.1	MAPTKRKGERKDPVQVPKLLIRGGVEVLEVKTGVDSITEVECFLTPEMGDPDEHLRGFSK	60
AEC14832.1	MAPTKRKGERKDPVQVPKLLIRGGVEVLEVKTGVDSITEVECFLTPEMGDPDEHLRGFSK	60
AEC14833.1	MAPTKRKGERKDPVQVPKLLIRGGVEVLEVKTGVDSITEVECFLTPEMGDPDEHLRGFSK	60
AEC14834.1	MAPTKRKGERKDPVQVPKLLIRGGVEVLEVKTGVDSITEVECFLTPEMGDPDEHLRGFSK	60
AEC14835.1	MAPTKRKGERKDPVQVPKLLIRGGVEVLEVKTGVDSITEVECFLTPEMGDPDEHLRGFSK	60
AEC14836.1	MAPTKRKGERKDPVQVPKLLIRGGVEVLEVKTGVDSITEVECFLTPEMGDPDEHLRGFSK	60
AEC14837.1	MAPTKRKGERKDPVQVPKLLIRGGVEVLEVKTGVDSITEVECFLTPEMGDPDEHLRGFSK	60
AEC14838.1	MAPTKRKGERKDPVQVPKLLIRGGVEVLEVKTGVDSITEVECFLTPEMGDPDEHLRGFSK	60
AEC14839.1	MAPTKRKGERKDPVQVPKLLIRGGVEVLEVKTGVDSITEVECFLTPEMGDPDEHLRGFSK	60
AEC14840.1	MAPTKRKGERKDPVQVPKLLIRGGVEVLEVKTGVDSITEVECFLTPEMGDPDEHLRGFSK	60
AEC14841.1	MAPTKRKGERKDPVQVPKLLIRGGVEVLEVKTGVDSITEVECFLTPEMGDPDEHLRGFSK	60
AEC14842.1	MAPTKRKGERKDPVQVPKLLIRGGVEVLEVKTGVDSITEVECFLTPEMGDPDEHLRGFSK	60
AEC14843.1	MAPTKRKGERKDPVQVPKLLIRGGVEVLEVKTGVDSITEVECFLTPEMGDPDEHLRGFSK	60
AEC14844.1	MAPTKRKGERKDPVQVPKLLIRGGVEVLEVKTGVDSITEVECFLTPEMGDPDEHLRGFSK	60
AEC14845.1	MAPTKRKGERKDPVQVPKLLIRGGVEVLEVKTGVDSITEVECFLTPEMGDPDEHLRGFSK	60
AEC14846.1	MAPTKRKGERKDPVQVPKLLIRGGVEVLEVKTGVDSITEVECFLTPEMGDPDEHLRGFSK	60
AEC14847.1	MAPTKRKGERKDPVQVPKLLIRGGVEVLEVKTGVDSITEVECFLTPEMGDPDEHLRGFSK	60
AEC14848.1	MAPTKRKGERKDPVQVPKLLIRGGVEVLEVKTGVDSITEVECFLTPEMGDPDEHLRGFSK	60
AEC14849.1	MAPTKRKGERKDPVQVPKLLIRGGVEVLEVKTGVDSITEVECFLTPEMGDPDEHLRGFSK	60
AEC14850.1	MAPTKRKGERKDPVQVPKLLIRGGVEVLEVKTGVDSITEVECFLTPEMGDPDEHLRGFSK	60
AEC14851.1	MAPTKRKGERKDPVQVPKLLIRGGVEVLEVKTGVDSITEVECFLTPEMGDPDEHLRGFSK	60
AEC14852.1	MAPTKRKGERKDPVQVPKLLIRGGVEVLEVKTGVDSITEVECFLTPEMGDPDEHLRGFSK	60
AEC14853.1	MAPTKRKGERKDPVQVPKLLIRGGVEVLEVKTGVDSITEVECFLTPEMGDPDEHLRGFSK	60
AEC14854.1	MAPTKRKGERKDPVQVPKLLIRGGVEVLEVKTGVDSITEVECFLTPEMGDPDEHLRGFSK	60
AEC14855.1	MAPTKRKGERKDPVQVPKLLIRGGVEVLEVKTGVDSITEVECFLTPEMGDPDEHLRGFSK	60
AEC14856.1	MAPTKRKGERKDPVQVPKLLIRGGVEVLEVKTGVDSITEVECFLTPEMGDPDEHLRGFSK	60
AEC14857.1	MAPTKRKGERKDPVQVPKLLIRGGVEVLEVKTGVDSITEVECFLTPEMGDPDEHLRGFSK	60
AEC14858.1	MAPTKRKGERKDPVQVPKLLIRGGVEVLEVKTGVDSITEVECFLTPEMGDPDEHLRGFSK	60
AEC14859.1	MAPTKRKGERKDPVQVPKLLIRGGVEVLEVKTGVDSITEVECFLTPEMGDPDEHLRGFSK	60
AEC14862.1	MAPTKRKGERKDPVQVPKLLIRGGVEVLEVKTGVDSITEVECFLTPEMGDPDEHLRGFSK	60
AEC14863.1	MAPTKRKGERKDPVQVPKLLIRGGVEVLEVKTGVDSITEVECFLTPEMGDPDEHLRGFSK	60
BAD21289.1	MAPTKRKGERKDPVQVPKLLIRGGVEVLEVKTGVDSITEVECFLTPEMGDPDEHLRGFSK	60
BAD21295.1	MAPTKRKGERKDPVQVPKLLIRGGVEVLEVKTGVDSITEVECFLTPEMGDPDEHLRGFSK	60
BAB93044.1	MAPTKRKGERKDPVQVPKLLIRGGVEVLEVKSGVDSITEVECFLTPEMGDPDEHLRGFSK	60
BAB93104.1	MAPTKRKGERKDPVQVPKLLIRGGVEVLEVKTGVDSITEVECFLTPEMGDPDEHLRGFSK	60
AAK70252.1	MAPTKRKGERKDPVQVPKLLIRGGVEVLEVKTGVDSITEVECFLTPEMGDPDEHLRGFSK	60
BAB68959.1	MAPTKRKGERKDPVQVPKLLIRGGVEVLEVKTGVDSITEVECFLTPEMGDPDEHLRGFSK	60
BAD21229.1	MAPTKRKAERKDPVQVPKLLIRGGVEVLEVKTGVDSITEVECFLTPEMGDPDEHLRGFSK	60
QBX89123.1	MAPTKRKGERKDPVQVPKLLIRGGVEVLEVKTGVDSITEVECFLTPEMGDPDEHLRGFSK	60
BAE02908.1	MAPTKRKGERKDPVQVPKLLIRGGVEVLEVKTGVDSITEVECFLTPEMGDPDEHLRGFSK	60
AAR89259.1	MAPTKRKGERKDPVQVPKLLIRGGVEVLEVKTGVDSITEVECFLTPEMGDPDEHLRGFSK	60
AAR12957.1	MAPTKRKGERKDPVQVPKLLIRGGVEVLEVKTGVDSITEVECFLTPEMGDPDEHLRGFSK	60
AAR13077.1	MAPTKRKGERKDPVQVPKLLIRGGVEVLEVKTGVDSITEVECFLTPEMGDPDEHLRGFSK	60
BCU41036.1	MAPTKRKGERKDPVQVPKLLIRGGVEVLEVKTGVDSITEVECFLTPEMGDPDEHLRGFSK	60
BAE94726.1	MAPTKRKGERKDPVQVPKLLIRGGVEVLEVKTGVDSITEVECFLTPEMGDPDEHLRGFSK	60
AAB94036.1	MAPTKRKGERKDPVQVPKLLIRGGVEVLEVKTGVDSITEVECFLTPEMGDPDEHFRGFSK	60
AAK28478.1	MAPTKRKGERKDPVQVPKLLIRGGVEVLEVKTGVDSITEVECFLTPEMGDPDEHLRGFSK	60
BAB93008.1	MAPTKRKGERKDPVQVPKLLIRGGVEVLEVKTGVDSITEVECFLTPEMGDPDEHLRGFSK	60
AAM69579.1	MAPTKRKGERKHPVQVPKLLIRGGVEVLEVKTGVDSITEVECFLTPEMGDPDEHLRGFSK	60
AAM69584.1	MAPTKRKGERKHPVQVPKLLIRGGVEVLEVKTGVDSITEVECFLTPEMGDPDEHLRGFSK	60
AAM69596.1	MAPTKRKGERKHPVQVPKLLIRGGVEVLEVKTGVDSITEVECFLTPEMGDPDEHLRGFSK	60
AAM69602.1	MAPTKRKGERKHPVQVPKLLIRGGVEVLEVKTGVDSITEVECFLTPEMGDPDEHLRGFSK	60
AAM69608.1	MAPTKRKGERKHPVQVPKLLIRGGVEVLEVKTGVDSITEVECFLTPEMGDPDEHLRGFSK	60

AAK70312.1	MAPTKRKGERKHPVQVPKLLIRGGVEVLEVKTGVDSITEVECFLTPEMGDPDEHLRGFSK	60
WLD48064.1	MAPTKRKGERKDPVQVPKLLIRGGVEVLEVKTGVDSITEVECFLTPEMGDPDEHLRGFSK	60
WLD48100.1	MAPTKRKGERKDPVQVPKLLIRGGVEVLEVKTGVDSITEVECFLTPEMGDPDEHLRGFSK	60
UQK62655.1	MAPTKRKGERKDPVQVPKLLIRGGVEVLEVKTGVDSITEVECFLTPEMGDPDEHLRGFSK	60
QTJ15180.1	MAPTKRKGERKDPVQVPKLLIRGGVEVLEVKTGVDSITEVECFLTPEMGDPDEHLRGFSK	60
QTJ15204.1	MAPTKRKGERKDPVQVPKLLIRGGVEVLEVKTGVDSITEVECFLTPEMGDPDEHLRGFSK	60
ASV51784.1	MAPTKRKGERKDPVQVPKLLIRGGVEVLEVKTGVDSITEVECFLTPEMGDPDEHLRGFSK	60
ASV51790.1	MAPTKRKGERKDPVQVPKLLIRGGVEVLEVKTGVDSITEVECFLTPEMGDPDEHLRGFSK	60
ASV51898.1	MAPTKRKGERKDPVQVPKLLIRGGVEVLEVKTGVDSITEVECFLTPEMGDPDEHLRGFSK	60
ASV51904.1	MAPTKRKGERKDPVQVPKLLIRGGVEVLEVKTGVDSITEVECFLTPEMGDPDEHLRGFSK	60
ASV51910.1	MAPTKRKGERKDPVQVPKLLIRGGVEVLEVKTGVDSITEVECFLTPEMGDPDEHLRGFSK	60
ASV51916.1	MAPTKRKGERKDPVQVPKLLIRGGVEVLEVKTGVDSITEVECFLTPEMGDPDEHLRGFSK	60
CCW72490.1	MAPTKRKGERKDPVQVPKLLIRGGVEVLEVKTGVDSITEVECFLTPEMGDPDEHLRGFSK	60
CCW72495.1	MAPTKRKGERKDPVQVPKLLIRGGVEVLEVKTGVDSITEVECFLTPEMGDPDEHLRGFSK	60
CCW72497.1	MAPTKRKGERKDPVQVPKLLIRGGVEVLEVKTGVDSITEVECFLTPEMGDPDEHLRGFSK	60
CCW72503.1	MAPTKRKGERKDPVQVPKLLIRGGVEVLEVKTGVDSITEVECFLTPEMGDPDEHLRGFSK	60
CCW72505.1	MAPTKRKGERKDPVQVPKLLIRGGVEVLEVKTGVDSITEVECFLTPEMGDPDEHLRGFSK	60
CCW72507.1	MAPTKRKGERKDPVQVPKLLIRGGVEVLEVKTGVDSITEVECFLTPEMGDPDEHLRGFSK	60
CCW72512.1	MAPTKRKGERKDPVQVPKLLIRGGVEVLEVKTGVDSITEVECFLTPEMGDPDEHLRGFSK	60
CCW72515.1	MAPTKRKGERKDPVQVPKLLIRGGVEVLEVKTGVDSITEVECFLTPEMGDPDEHLRGFSK	60
BAB68881.1	MAPTKRKGERKDPVQVPKLLIRGGVEVLEVKTGVDSITEVECFLTPEMGDPDEHLRGFSK	60
BAB68887.1	MAPTKRKGERKDPVQVPKLLIRGGVEVLEVKTGVDSITEVECFLTPEMGDPDEHLRGFSK	60
AAK70294.1	MAPTKRKGERKDPVQVPKLLIRGGVEVLEVKTGVDSITEVECFLTPEMGDPDEHLRGFSK	60
WLD48094.1	MAPTKRKGERKDPVQVPKLLIRGGVEVLEVKTGVDSITEVECFLTPEMGDPDEHLRGFSK	60
CCW72509.1	MAPTKRKGERKDPVQVPKLLIRGGVEVLEVKTGVDSITEVECFLTPEMGDPDEHLRGFSK	60
BAB68893.1	MAPTKRKGERKDPVQVPKLLIRGGVEVLEVKTGVDSITEVECFLTPEMGDPDEHLRGFSK	60
QTJ15216.1	MAPTKRKGERKDPVQVPKLLIRGGVEVLEVKTGVDSITEVECFLTPEMGDPDEHLRGFSK	60
CCW72493.1	MAPTKRKGERKDPVQVPKLLIRGGVEVLEVKTGVDSITEVECFLTPEMGDPDEHLRGFSK	60
CCW72500.1	MAPTKRKGERKDPVQVPKLLIRGGVEVLEVKTGVDSITEVECFLTPEMGDPDEHLRGFSK	60
CCW72506.1	MAPTKRKGERKDPVQVPKLLIRGGVEVLEVKTGVDSITEVECFLTPEMGDPDEHLRGFSK	60
CCW72520.1	MAPTKRKGERKDPVQVPKLLIRGGVEVLEVKTGVDSITEVECFLTPEMGDPDEHLRGFSK	60
BAB68899.1	MAPTKRKGERKDPVQVPKLLIRGGVEVLEVKTGVDSITEVECFLTPEMGDPDEHLRGFSK	60
BAB68905.1	MAPTKRKGERKDPVQVPKLLIRGGVEVLEVKTGVDSITEVECFLTPEMGDPDEHLRGFSK	60
AAK70300.1	MAPTKRKGERKDPVQVPKLLIRGGVEVLEVKTGVDSITEVECFLTPEMGDPDEHLRGFSK	60
AYM55579.1	MAPTKRKGERKDPVQVPKLLIRGGVEVLEVKTGVDSITEVECFLTPEMGDPDEHLRGFSK	60
AYM55580.1	MAPTKRKGERKDPVQVPKLLIRGGVEVLEVKTGVDSITEVECFLTPEMGDPDEHLRGFSK	60
AYM55582.1	MAPTKRKGERKDPVQVPKLLIRGGVEVLEVKTGVDSITEVECFLTPEMGDPDEHLRGFSK	60
AYM55583.1	MAPTNRKGERKDPVQVPKLLIRGGVEVLEVKTGVDSITEVECFLTPEMGDPDEHLRGFSK	60
AYM55584.1	MAPTKRKGERKDPVQVPKLLIRGGVEVLEVKTGVDSITEVECFLTPEMGDPDEHLRGFSK	60
AYM55590.1	MAPTKRKGERKDPVQVPKLLIRGGVEVLEVKTGVDSITEVECFLTPEMGDPDEHLRGFSK	60
AYM55596.1	MAPTKRKGERKDPVQVPKLLIRGGVEVLEVKTGVDSITEVECFLTPEMGDPDEHLRGFSK	60
BAE45366.1	MAPTKRKGERKDPVQVPKLLIRGGVEVLEVKTGVDSITEVECFLTPEMGDPDEHLRGFSK	60
AAG30857.1	MAPTKRKGERKDPVQVPKLLIRGGVEVLEVKTGVDSITEVECFLTPEMGDPDEHLRGFSK	60
BAH36774.1	MAPTKRKGERKDPVQVPKLLIRGGVEVLEVKTGVDSITEVECFLTPEMGDPDEHLRGFSK	60
BAH36780.1	MAPTKRKGERKDPVQVPKLLIRGGVEVLEVKTGVDSITEVECFLTPEMGDPDEHLRGFSK	60
BAH36822.1	MAPTKRKGERKDPVQVPKLLIRGGVEVLEVKTGVDSITEVECFLTPEMGDPDEHLRGFSK	60
BAH36834.1	MAPTKRKGERKDPVQVPKLLIRGGVEVLEVKTGVDSITEVECFLTPEMGDPDEHLRGFSK	60
BAH36840.1	MAPTKRKGERKDPVQVPKLLIRGGVEVLEVKTGVDSITEVECFLTPEMGDPDEHLRGFSK	60
BAH36846.1	MAPTKRKGERKDPVQVPKLLIRGGVEVLEVKTGVDSITEVECFLTPEMGDPDEHLRGFSK	60
BAH36816.1	MAPTKRKGERKDPVQVPKLLIRGGVEVLEVKTGVDSITEVECFLTPEMGDPDEHLRGFSK	60
BAE45444.1	MAPTKRKGERKDPVQVPKLLIRGGVEVLEVKTGVDSITEVECFLTPEMGDPDEHLRGFSK	60
AAT09819.1	MAPTKRKGERKDPVQVPKLLIRGGVEVLEVKTGVDSITEVECFLTPEMGDPDEHLRGFSK	60
BAC03029.1	MAPTKRKGERKDPVQVPKLLIRGGVEVLEVKTGVDSITEVECFLTPEMGDPDEHLRGFSK	60
BAC03095.1	MAPTKRKGERKDPVQVPKLLIRGGVEVLEVKTGVDSITEVECFLTPEMGDPDEHLRGFSK	60
AAM69560.1	MAPTKRKGERKDPVQVPKLLIRGGVEVLEVKTGVDSITEVECFLTPEMGDPDEHLRGFSK	60
AAM69566.1	MAPTKRKGERKDPAQVPKLLIRGGVEVLEVKTGVDSITEVECFLTPEMGDPDEHLRGFSK	60
AAM69572.1	MAPTKRKGERKDPVQVPKLLIRGGVEVLEVKTGVDSITEVECFLTPEMGDPDEHLRGFSK	60
AAB84311.1	MAPTKRKGERKDPVQVPKLLIRGGVEVLEVKTGVDSITEVECFLTPEMGDPDEHLRGFSK	60
BAD06150.1	MAPTKRKGERKDPVQVPKLLIRGGVEVLEVKTGVDSVTEVECFLTPEMGDPDEHLRGFSK	60
AYM55581.1	MAPTKRKGERKDPVQVPKLLIRGGVEVLEVKTGVDSITEVECFLTPEMGDPDEHLRGFSK	60
AYM55588.1	MAPTKRKGERKDPVQVPKLLIRGGVEVLEVKTGVDSITEVECFLTPEMGDPDEHLRGFSK	60
AYM55589.1	MAPTKRKGERKDPVQVPKLLIRGGVEVLEVKTGVDSITEVECFLTPEMGDPDEHLRGFSK	60
AYM55591.1	MAPTKRKGERKDPVQVPKLLIRGGVEVLEVKTGVDSITEVECFLTPEMGDPDEHLRGFSK	60
AYM55592.1	MAPTKRKGERKDPVQVPKLLIRGGVEVLEVKTGVDSITEVECFLTPEMGDPDEHLRGFSK	60

AYM55593.1	MAPTKRKGERKDPVQVPKLLIRGGVEVLEVKTGVDSITEVECFLTPEMGDPDEHLRGFSK	60
AYM55595.1	MAPTKRKGERKDPVQVPKLLIRGGVEVLEVKTGVDSITEVECFLTPEMGDPDEHLRGFSK	60
AYM55597.1	MAPTKRKGERKDPVQVPKLLIRGGVEVLEVKTGVDSITEVECFLTPEMGDPDEHLRGFSK	60
AYM55598.1	MAPTKRKGERKDPVQVPKLLIRGGVEVLEVKTGVDSITEVECFLTPEMGDPDEHLRGFSK	60
AYM55599.1	MAPTKRKGERKDPVQVPKLLIRGGVEVLEVKTGVDSITEVECFLTPEMGDPDEHLRGFSK	60
AYM55600.1	MAPTKRKGERKDPVQVPKLLIRGGVEVLEVKTGVDSITEVECFLTPEMGDPDEHLRGFSK	60
AYM55601.1	MAPTKRKGERKDPVQVPKLLIRGGVEVLEVKTGVDSITEVECFLTPEMGDPDEHLRGFSK	60
AYM55602.1	MAPTKRKGERKDPVQVPKLLIRGGVEVLEVKTGVDSITEVECFLTPEMGDPDEHLRGFSK	60
AYM55603.1	MAPTKRKGERKDPVQVPKLLIRGGVEVLEVKTGVDSITEVECFLTPEMGDPDEHLRGFSK	60
CCW72483.1	MAPTKRKGERKDPVQVPKLLIRGGVEVLEVKTGVDSITEVECFLTPEMGDPDEHLRGFSK	60
CCW72485.1	MAPTKRKGERKDPVQVPKLLIRGGVEVLEVKTGVDSITEVECFLTPEMGDPDEHLRGFSK	60
AFR56669.1	MAPTKRKGERKDPVQVPKLLIRGGVEVLEVKTGVDSITEVECFLTPEMGDPDEHLRGFSK	60
BAH36768.1	MAPTKRKGERKDPVQVPKLLIRGGVEVLEVKTGVDSITEVECFLTPEMGDPDEHLRGFSK	60
BAH36786.1	MAPTKRKGERKDPVQVPKLLIRGGVEVLEVKTGVDSITEVECFLTPEMGDPDEHLRGFSK	60
BAH36792.1	MAPTKRKGERKDPVQVPKLLIRGGVEVLEVKTGVDSITEVECFLTPEMGDPDEHLRGFSK	60
BAH36798.1	MAPTKRKGERKDPVQVPKLLIRGGVEVLEVKTGVDSITEVECFLTPEMGDPDEHLRGFSK	60
BAH36804.1	MAPTKRKGERKDPVQVPKLLIRGGVEVLEVKTGVDSITEVECFLTPEMGDPDEHLRGFSK	60
BAH36810.1	MAPTKRKGERKDPVQVPKLLIRGGVEVLEVKTGVDSITEVECFLTPEMGDPDEHLRGFSK	60
BAH36828.1	MAPTKRKGERKDPVQVPKLLIRGGVEVLEVKTGVDSITEVECFLTPEMGDPDEHLRGFSK	60
BAH36852.1	MAPTKRKGERKDPVQVPKLLIRGGVEVLEVKTGVDSITEVECFLTPEMGDPDEHLRGFSK	60
BAH36858.1	MAPTKRKGERKDPVQVPKLLIRGGVEVLEVKTGVDSITEVECFLTPEMGDPDEHLRGFSK	60
BAE75814.1	MAPTKRKGERKDPVQVPKLLIRGGVEVLEVKTGVDSITEVECFLTPEMGDPDEHLRGFSK	60
BAE75820.1	MAPTKRKGERKDPVQVPKLLIRGGVEVLEVKTGVDSITEVECFLTPEMGDPDEHLRGFSK	60
BAE75826.1	MAPTKRKGERKDPVQVPKLLIRGGVEVLEVKTGVDSITEVECFLTPEMGDPDEHLRGFSK	60
BAE75832.1	MAPTKRKGERKDPVQVPKLLIRGGVEVLEVKTGVDSITEVECFLTPEMGDPDEHLRGFSK	60
BAE75838.1	MAPTKRKGERKDPVQVPKLLIRGGVEVLEVKTGVDSITEVECFLTPEMGDPDEHLRGFSK	60
BAE45360.1	MAPTKRKGERKDPVQVPKLLIRGGVEVLEVKTGVDSITEVECFLTPEMGDPDEHLRGFSK	60
BAE45372.1	MAPTKRKGERKDPVQVPKLLIRGGVEVLEVKTGVDSITEVECFLTPEMGDPDEHLRGFSK	60
BAE45378.1	MAPTKRKGERKDPVQVPKLLIRGGVEVLEVKTGVDSITEVECFLTPEMGDPDEHLRGFSK	60
BAE45384.1	MAPTKRKGERKDPVQVPKLLIRGGVEVLEVKTGVDSITEVECFLTPEMGDPDEHLRGFSK	60
BAE45390.1	MAPTKRKGERKDPVQVPKLLIRGGVEVLEVKTGVDSITEVECFLTPEMGDPDEHLRGFSK	60
BAE45396.1	MAPTKRKGERKDPVQVPKLLIRGGVEVLEVKTGVDSITEVECFLTPEMGDPDEHLRGFSK	60
BAE45402.1	MAPTKRKGERKDPVQVPKLLIRGGVEVLEVKTGVDSITEVECFLTPEMGDPDEHLRGFSK	60
BAE45408.1	MAPTKRKGERKDPVQVPKLLIRGGVEVLEVKTGVDSITEVECFLTPEMGDPDEHLRGFSK	60
BAE45414.1	MAPTKRKGERKDPVQVPKLLIRGGVEVLEVKTGVDSITEVECFLTPEMGDPDEHLRGFSK	60
BAE45420.1	MAPTKRKGERKDPVQVPKLLIRGGVEVLEVKTGVDSITEVECFLTPEMGDPDEHLRGFSK	60
BAE45426.1	MAPTKRKGERKDPVQVPKLLIRGGVEVLEVKTGVDSITEVECFLTPEMGDPDEHLRGFSK	60
BAE45432.1	MAPTKRKGERKDPVQVPKLLIRGGVEVLEVKTGVDSITEVECFLTPEMGDPDEHLRGFSK	60
BAE45438.1	MAPTKRKGERKDPVQVPKLLIRGGVEVLEVKTGVDSITEVECFLTPEMGDPDEHLRGFSK	60
BAD06018.1	MAPTKRKGERKDPVQVPKLLIRGGVEVLEVKTGVDSITEVECFLTPEMGDPDEHLRGFSK	60
BAD06024.1	MAPTKRKGERKDPVQVPKLLIRGGVEVLEVKTGVDSITEVECFLTPEMGDPDEHLRGFSK	60
BAD06030.1	MAPTKRKGERKDPVQVPKLLIRGGVEVLEVKTGVDSITEVECFLTPEMGDPDEHLRGFSK	60
BAD06042.1	MAPTKRKGERKDPVQVPKLLIRGGVEVLEVKTGVDSITEVECFLTPEMGDPDEHLRGFSK	60
BAD06048.1	MAPTKRKGERKDPVQVPKLLIRGGVEVLEVKTGVDSITEVECFLTPEMGDPDEHLRGFSK	60
BAD06054.1	MAPTKRKGERKDPVQVPKLLIRGGVEVLEVKTGVDSITEVECFLTPEMGDPDEHLRGFSK	60
BAD06084.1	MAPTKRKGERKDPVQVPKLLIRGGVEVLEVKTGVDSITEVECFLTPEMGDPDEHLRGFSK	60
BAD06090.1	MAPTKRKGERKDPVQVPKLLIRGGVEVLEVKTGVDSITEVECFLTPEMGDPDEHLRGFSK	60
BAD06096.1	MAPTKRKGERKDPVQVPKLLIRGGVEVLEVKTGVDSITEVECFLTPEMGDPDEHLRGFSK	60
BAD06102.1	MAPTKRKGERKDPVQVPKLLIRGGVEVLEVKTGVDSITEVECFLTPEMGDPDEHLRGFSK	60
BAD06108.1	MAPTKRKGERKDPVQVPKLLIRGGVEVLEVKTGVDSITEVECFLTPEMGDPDEHLRGFSK	60
BAD06120.1	MAPTKRKGERKDPVQVPKLLIRGGVEVLEVKTGVDSITEVECFLTPEMGDPDEHLRGFSK	60
BAC15695.1	MAPTKRKGERKDPVQVPKLLIRGGVEVLEVKTGVDSITEVECFLTPEMGDPDEHLRGFSK	60
BAC15725.1	MAPTKRKGERKDPVQVPKLLIRGGVEVLEVKTGVDSITEVECFLTPEMGDPDEHLRGFSK	60
BAC02951.1	MAPTKRKGERKDPVQVPKLLIRGGVEVLEVKTGVDSITEVECFLTPEMGDPDEHLRGFSK	60
BAC02957.1	MAPTKRKGERKDPVQVPKLLIRGGVEVLEVKTGVDSITEVECFLTPEMGDPDEHLRGFSK	60
BAC02963.1	MAPTKRKGERKDPVQVPKLLIRGGVEVLEVKTGVDSITEVECFLTPEMGDPDEHLRGFSK	60
BAC02969.1	MAPTKRKGERKDPVQVPKLLIRGGVEVLEVKTGVDSITEVECFLTPEMGDPDEHLRGFSK	60
BAC02975.1	MAPTKRKGERKDPVQVPKLLIRGGVEVLEVKTGVDSITEVECFLTPEMGDPDEHLRGFSK	60
BAC02981.1	MAPTKRKGERKDPVQVPKLLIRGGVEVLEVKTGVDSITEVECFLTPEMGDPDEHLRGFSK	60
BAC02987.1	MAPTKRKGERKDPVQVPKLLIRGGVEVLEVKTGVDSITEVECFLTPEMGDPDEHLRGFSK	60
BAC02993.1	MAPTKRKGERKDPVQVPKLLIRGGVEVLEVKTGVDSITEVECFLTPEMGDPDEHLRGFSK	60
BAC02999.1	MAPTKRKGERKDPVQVPKLLIRGGVEVLEVKTGVDSITEVECFLTPEMGDPDEHLRGFSK	60
BAC03005.1	MAPTKRKGERKDPVQVPKLLIRGGVEVLEVKTGVDSITEVECFLTPEMGDPDEHLRGFSK	60
BAC03011.1	MAPTKRKGERKDPVQVPKLLIRGGVEVLEVKTGVDSITEVECFLTPEMGDPDEHLRGFSK	60

BAC03017.1	MAPTKRKGERKDPVQVPKLLIRGGVEVLEVKTGVDSITEVECFLTPEMGDPDEHLRGFSK	60
BAC03023.1	MAPTKRKGERKDPVQVPKLLIRGGVEVLEVKTGVDSITEVECFLTPEMGDPDEHLRGFSK	60
BAC03035.1	MAPTKRKGERKDPVQVPKLLIRGGVEVLEVKTGVDSITEVECFLTPEMGDPDEHLRGFSK	60
BAC03041.1	MAPTKRKGERKDPVQVPKLLIRGGVEVLEVKTGVDSITEVECFLTPEMGDPDEHLRGFSK	60
BAC03047.1	MAPTKRKGERKDPVQVPKLLIRGGVEVLEVKTGVDSITEVECFLTPEMGDPDEHLRGFSK	60
BAC03053.1	MAPTKRKGERKDPVQVPKLLIRGGVEVLEVKTGVDSITEVECFLTPEMGDPDEHLRGFSK	60
BAC03059.1	MAPTKRKGERKDPVQVPKLLIRGGVEVLEVKTGVDSITEVECFLTPEMGDPDEHLRGFSK	60
BAC03071.1	MAPTKRKGERKDPVQVPKLLIRGGVEVLEVKTGVDSITEVECFLTPEMGDPDEHLRGFSK	60
BAC03077.1	MAPTKRKGERKDPVQVPKLLIRGGVEVLEVKTGVDSITEVECFLTPEMGDPDEHLRGFSK	60
BAC03083.1	MAPTKRKGERKDPVQVPKLLIRGGVEVLEVKTGVDSITEVECFLTPEMGDPDEHLRGFSK	60
BAC03089.1	MAPTKRKGERKDPVQVPKLLIRGGVEVLEVKTGVDSITEVECFLTPEMGDPDEHLRGFSK	60
AAM69542.1	MAPTKRKGERKDPVQVPKLLIRGGVEVLEVKTGVDSITEVECFLTPEMGDPDEHLRGFSK	60
AAM69548.1	MAPTKRKGERKDPVQVPKLLIRGGVEVLEVKTGVDSITEVECFLTPEMGDPDEHLRGFSK	60
AAM69554.1	MAPTKRKGERKDPVQVPKLLIRGGVEVLEVKTGVDSITEVECFLTPEMGDPDEHLRGFSK	60
AAM69619.1	MAPTKRKGERKDPVQVPKLLIRGGVEVLEVKTGVDSITEVECFLTPEMGDPDEHLRGFSK	60
BAB68977.1	MAPTKRKGERKDPVQVPKLLIRGGVEVLEVKTGVDSITEVECFLTPEMGDPDEHLRGFSK	60
BAB68983.1	MAPTKRKGERKDPVQVPKLLIRGGVEVLEVKTGVDSITEVECFLTPEMGDPDEHLRGFSK	60
BAB68989.1	MAPTKRKGERKDPVQVPKLLIRGGVEVLEVKTGVDSITEVECFLTPEMGDPDEHLRGFSK	60
BAB68995.1	MAPTKRKGERKDPVQVPKLLIRGGVEVLEVKTGVDSITEVECFLTPEMGDPDEHLRGFSK	60
AAK98000.1	MAPTKRKGERKDPVQVPKLLIRGGVEVLEVKTGVDSITEVECFLTPEMGDPDEHLRGFSK	60
AAK98006.1	MAPTKRKGERKDPVQVPKLLIRGGVEVLEVKTGVDSITEVECFLTPEMGDPDEHLRGFSK	60
AAK98010.1	MAPTKRKGERKDPVQVPKLLIRGGVEVLEVKTGVDSITEVECFLTPEMGDPDEHLRGFSK	60
AAK98018.1	MAPTKRKGERKDPVQVPKLLIRGGVEVLEVKTGVDSITEVECFLTPEMGDPDEHLRGFSK	60
AAK98024.1	MAPTKRKGERKDPVQVPKLLIRGGVEVLEVKTGVDSITEVECFLTPEMGDPDEHLRGFSK	60
AAK98030.1	MAPTKRKGERKDPVQVPKLLIRGGVEVLEVKTGVDSITEVECFLTPEMGDPDEHLRGFSK	60
AAK98036.1	MAPTKRKGERKDPVQVPKLLIRGGVEVLEVKTGVDSITEVECFLTPEMGDPDEHLRGFSK	60
AAK70258.1	MAPTKRKGERKDPVQVPKLLIRGGVEVLEVKTGVDSITEVECFLTPEMGDPDEHLRGFSK	60
AAK70264.1	MAPTKRKGERKDPVQVPKLLIRGGVEVLEVKTGVDSITEVECFLTPEMGDPDEHLRGFSK	60
AAG53896.1	MAPTKRKGERKDPVQVPKLLIRGGVEVLEVKTGVDSITEVECFLTPEMGDPDEHLRGFSK	60
BAA01962.1	MAPTKRKGERKDPVQVPKLLIRGGVEVLEVKTGVDSITEVECFLTPEMGDPDEHLRGFSK	60
ALF06256.1	MAPTKRKGERKDPVQVPKLLIRGGVEVLEVKTGVDSITEVECFLTPEMGDPBEHLRGFSK	60
BAD06060.1	MAPTKRKGERKDPVQVPKLLIRGGVEVLEVKTGVDSVTEVECFLTPEMGDPDEHLRGFSK	60
BAD06072.1	MAPTKRKGERKDPVQVPKLLIRGGVEVLEVKTGVDSVTEVECFLTPEMGDPDEHLRGFSK	60
BAD06156.1	MAPTKRKGERKDPVQVPKLLIRGGVEVLEVKTGVDSVTEVECFLTPEMGDPDEHLRGFSK	60
BAD06174.1	MAPTKRKGERKDPVQVPKLLIRGGVEVLEVKTGVDSVTEVECFLTPEMGDPDEHLRGFSK	60
AAN85449.1	MAPTKRKGERKDPVQVPKLLIRGGVEVLEVKTGVDSVTEVECFLTPEMGDPDEHLRGFSK	60
AAN85455.1	MAPTKRKGERKDPVQVPKLLIRGGVEVLEVKTGVDSVTEVECFLTPEMGDPDEHLRGFSK	60
AAK98042.1	MAPTKRKGERKDPVQVPKLLIRGGVEVLEVKTGVDSVTEVECFLTPEMGDPDEHLRGFSK	60
BAD06036.1	MAPTKRKGERKDPVQVPKLLIRGGVEVLEVKTGVDSITEVECFLTPEMGDPDEHLRGFSK	60
BCP65063.1	MAPTKRKGERKDPVQVPKLLIRGGVEVLEVKTGVDSITEVECFLTPEMGDPDEHLRGFSK	60
BCP56477.1	MAPTKRKGERKDPVQVPKLLIRGGVEVLEVKTGVDSITEVECFLTPEMGDPDEHLRGFSK	60
BAV69373.1	MAPTKRKGERKDPVQVPKLLIRGGVEVLEVKTGVDSITEVECFLTPEMGDPDEHLRGFSK	60
BAE00153.1	MAPTKRKGERKDPVQVPKLLIRGGVEVLEVKTGVDSITEVECFLTPEMGDPDEHLRGFSK	60
BAC22906.1	MAPTKRKGERKDPVQVPKLLIRGGVEVLEVKTGVDSITEVECFLTPEMGDPDEHLRGFSK	60
BAA05639.1	MAPTKRKGERKDPVQVPKLLIRGGVEVLEVKTGVDSITEVECFLTPEMGDPDEHLRGFSK	60
BAB11728.1	MAPTKRKGERKDPVQVPKLLIRGGVEVLEVKTGVDSITEVECFLTPEMGDPDEHLRGFSK	60
BAA05637.1	MAPTKRKGERKDPVQVPKLLIRGGVEVLEVKTGVDSITEVECFLTPEMGDPDEHLRGFSK	60
BAA01969.1	MAPTKRKGERKDPVQVPKLLIRGGVEVLEVKTGVDSITEVECFLTPEMGDPDEHLRGFSK	60
BAE00159.1	MAPTKRKGERKDPVQVPKLLIRGGVEVLEVKTGVDSITEVECFLTPEMGDPDEHLRGFSM	60
BAE00165.1	MAPTKRKGERKDPVQVPKLLIRGGVEVLEVKTGVDSITEVECFLTPEMGDPDEHLRGFSK	60
BAE00171.1	MAPTKRKGERKDPVQVPKLLIRGGVEVLEVKTGVDSITEVECFLTPEMGDPDEHLRGFSK	60
ALF06268.1	MAPTKRKGERKDPVQVPKLLIRGGVEVLEVKTGVDSITEVECFLTPEMGDPDEHLRGFSK	60
AYM55586.1	MAPTKRKGERKDPVQVPKLLIRGGVEVLEVKTGVDSITEVECFLTPEMGDPDEHLRGFSK	60
AYM55585.1	MAPTKRKGERKDPVQVPKLLIRGGVEVLEVKTGVDSITEVECFLTPEMGDPDEHLRGFSK	60
AYM55587.1	MAPTKRKGERKDPVQVPKLLIRGGVEVLEVKTGVDSITEVECFLTPEMGDPDEHLRGFSK	60
AYM55594.1	MAPTKRKGERKDPVQVPKLLIRGGVEVLEVKTGVDSITEVECFLTPEMGDPDEHLRGFSK	60
QBQ65470.1	MAPTKRKGERKDPVQVPKLLIRGGVEVLEVKTGVDSITEVECFLTPEMGDPDEHLRGFSK	60
CCW72501.1	MAPTKRKGERKDPVQVPKLLIRGGVEVLEVKTGVDSITEVECFLTPEMGDPDEHLRGFSK	60
CCW72524.1	MAPTKRKGERKDPVQVPKLLIRGGVEVLEVKTGVDSITEVECFLTPEMGDPDEHLRGFSK	60
QWT77116.1	MAPTKRKGERKDPVQVPKLLIRGGVEVLEVKTGVDSITEVECFLTPEMGDPDEHLRGFSK	60
WLD48046.1	MAPTKRKGERKDPVQVPKLLIRGGVEVLEVKTGVDSITEVECFLTPEMGDPDEHLRGFSK	60
WLD48052.1	MAPTKRKGERKDPVQVPKLLIRGGVEVLEVKTGVDSITEVECFLTPEMGDPDEHLRGFSK	60
AAK70288.1	MAPTKRKGERKDPVQVPKLLIRGGVEVLEVKTGVDSITEVECFLTPEMGDPDEHLRGFSK	60
AAK70270.1	MAPTKRKGERKDPVQVPKLLIRGGVEVLEVKTGVDSITEVECFLTPEMGDPDEHLRGFSK	60

BAD21277.2 MAPTKRKGERKDPVQVPKLLIRGGVEVLEVKTGVDSITEVECFLTPEMGDPDEHLRGFSK 60
CCW72498.1 MAPTKRKGERKDPVQVPKLLIRGGVEVLEVKTGVDSITEVECFLTPEMGDPDEHLRGFSK 60
UPN63085.1 MAPTKRKGERKDPVQVPKLLIRGGVEVLEVKTGVDSITEVECFLTPEMGDPDEHLRGFSK 60
UPN63090.1 MAPTKRKGERKDPVQVPKLLIRGGVEVLEVKTGVDSITEVECFLTPEMGDPDEHLRGFSK 60
QWT77134.1 MAPTKRKGERKDPVQVPKLLIRGGVEVLEVKTGVDSITEVECFLTPEMGDPDEHLRGFSK 60
WLD48070.1 MAPTKRKGERKDPVQVPKLLIRGGVEVLEVKTGVDSITEVECFLTPEMGDPDEHLRGFSK 60
AAK70282.1 MAPTKGKGERKDPVQVPKLLIRGGVEVLEVKTGVDSITEVECFLTPEMGDPNEHLRGFSK 60
WLD48058.1 MAPTKRKGERKDPVQVPKLLIRGGVEVLEVKTGVDSITEVECFLTPEMGDPNEHLRGFSK 60
WLD48076.1 MAPTKRKGERKDPVQVPKLLIRGGVEVLEVKTGVDSITEVECFLTPEMGDPNEHLRGFSK 60
UQK62667.1 MAPTKRKGERKDPVQVPKLLIRGGVEVLEVKTGVDSITEVECFLTPEMGDPNEHLRGFSK 60
CCW72480.1 MAPTKRKGERKDPVQVPKLLIRGGVEVLEVKTGVDSITEVECFLTPEMGDPNEHLRGFSK 60
CCW72513.1 MAPTKRKGERKDPVQVPKLLIRGGVEVLEVKTGVDSITEVECFLTPEMGDPNEHLRGFSK 60
AAM69614.1 MAPTKRKGERKHPVQVPKLLIRGGVEVLEVKTGVDSITEVECFLTPEMGDPDEHLRGFSK 60
AAK70318.1 MAPTKRKGERKHPVQVPKLLIRGGVEVLEVKTGVDSITEVECFLTPEMGDPDEHLRGFSK 60
AAK70324.1 MAPTKRKGERKHPVQVPKLLIRGGVEVLEVKTGVDSITEVECFLTPEMGDPDEHLRGFSK 60
AAT09837.1 MAPTKRKGERKDPVQVPKLLIRGGVEVLEVKTGVDSITEVECFLTPEMGDPDEHLRGFSN 60
AAT09831.1 MAPTKRKGERKDPVQVPKLLIRGGVEVLEVKTGVDSITEVECFLTPEMGDPDEHFRGFSK 60
AAT09825.1 MAPTKRKGERKDPVQVPKLLIRGGVEVLEVKTGVDSITEVECFLTPEMGDPDEHLRGFSK 60
AAR89271.1 MAPTKRKGERKDPVQVPKLLIRGGVEVLEVKTGVDSITEVECFLTPEMGDPDEHLRGFSK 60
AAR89211.1 MAPTKRKGERKDPVQVPKLLIRGGVEVLEVKTGVDSITEVECFLTPEMGDPDEHLRGFSK 60
BAE02956.1 MAPTKRKGERKDPVQVPKLLIRGGVEVLEVKTGVDSITEVECFLTPEMGDPNEHLRGFSK 60
ACF75876.1 MAPTKRKGERKDPVQVPKLLIRGGVEVLEVKTGVDSITEVECFLTPEMGDPDEHLRGFSK 60
QBM11924.1 MAPTKRKGERKDPVQVPKLLIRGGVEVLEVKTGVDSITEVECFLTPEMGDPDEHLRGFSK 60
QBM11858.1 MAPTKRKGERKDPVQVPKLLIRGGVEVLEVKTGVDSITEVECFLTPEMGDPDEHLRGFSK 60
QBQ04203.1 MAPTKRKGERKDPVQVPKLLIRGGVEVLEVKTGVDSITEVECFLTPEMGDPDEHLRGFSK 60
QBQ65473.1 MAPTKRKGERKDPVQVPKLLIRGGVEVLEVKTGVDSITEVECFLTPEMGDPDEHLRGFSK 60
QBQ65472.1 MAPTKRKGERKDPVQVPKLLIRGGVEVLEVKTGVDSITEVECFLTPEMGDPDEHLRGFSK 60
QBM11920.1 MAPTKRKGERKDPVQVPKLLIRGGVEVLEVKTGVDSITEVECFLTPEMGDPDEHLRGFSK 60
QAA95693.1 MAPTKRKGERKDPVQVPKLLIRGGVEVLEVKTGVDSITEVECFLTPEMGDPDEHLRGFSK 60
QBX89122.1 MAPTKRKGERKDPVQVPKLLIRGGVEVLEVKTGVDSITEVECFLTPEMGDPDEHLRGFSK 60
QID57946.1 MAPTKRKGERKDPVQVPKLLIRGGVEVLEVKTGVDSITEVECFLTPEMGDPDEHLRGFSK 60
QID57952.1 MAPTKRKGERKDPVQVPKLLIRGGVEVLEVKTGVDSITEVECFLTPEMGDPDEHLRGFSK 60
QID57958.1 MAPTKRKGERKDPVQVPKLLIRGGVEVLEVKTGVDSITEVECFLTPEMGDPDEHLRGFSK 60
QID57964.1 MAPTKRKGERKDPVQVPKLLIRGGVEVLEVKTGVDSITEVECFLTPEMGDPDEHLRGFSK 60
QID57970.1 MAPTKRKGERKDPVQVPKLLIRGGVEVLEVKTGVDSITEVECFLTPEMGDPDEHLRGFSK 60
QID57976.1 MAPTKRKGERKDPVQVPKLLIRGGVEVLEVKTGVDSITEVECFLTPEMGDPDEHLRGFSK 60
QCH41110.1 MAPTKRKGERKDPVQVPKLLIRGGVEVLEVKTGVDSITEVECFLTPEMGDPDEHLRGFSK 60
QBX89120.1 MAPTKRKGERKDPVQVPKLLIRGGVEVLEVKTGVDSITEVECFLTPEMGDPDEHLRGFSK 60
QBX89121.1 MAPTKRKGERKDPVQVPKLLIRGGVEVLEVKTGVDSITEVECFLTPEMGDPDEHLRGFSK 60
QBX89124.1 MAPTKRKGERKDPVQVPKLLIRGGVEVLEVKTGVDSITEVECFLTPEMGDPDEHLRGFSK 60
QBQ65471.1 MAPTKRKGERKDPVQVPKLLIRGGVEVLEVKTGVDSITEVECFLTPEMGDPDEHLRGFSK 60
QBQ65474.1 MAPTKRKGERKDPVQVPKLLIRGGVEVLEVKTGVDSITEVECFLTPEMGDPDEHLRGFSK 60
QBQ65476.1 MAPTKRKGERKDPVQVPKLLIRGGVEVLEVKTGVDSITEVECFLTPEMGDPDEHLRGFSK 60
QBQ04201.1 MAPTKRKGERKDPVQVPKLLIRGGVEVLEVKTGVDSITEVECFLTPEMGDPDEHLRGFSK 60
QBM11921.1 MAPTKRKGERKDPVQVPKLLIRGGVEVLEVKTGVDSITEVECFLTPEMGDPDEHLRGFSK 60
QBM11922.1 MAPTKRKGERKDPVQVPKLLIRGGVEVLEVKTGVDSITEVECFLTPEMGDPDEHLRGFSK 60
CDJ79845.1 MAPTKRKGERKDPVQVPKLLIRGGVEVLEVKTGVDSITEVECFLTPEMGDPDEHLRGFSK 60
BAE02932.1 MAPTKRKGERKDPVQVPKLLIRGGVEVLEVKTGVDSITEVECFLTPEMGDPDEHLRGFSK 60
BAE02938.1 MAPTKRKGERKDPVQVPKLLIRGGVEVLEVKTGVDSITEVECFLTPEMGDPDEHLRGFSK 60
BAE02950.1 MAPTKRKGERKDPVQVPKLLIRGGVEVLEVKTGVDSITEVECFLTPEMGDPDEHLRGFSK 60
BAE03004.1 MAPTKRKGERKDPVQVPKLLIRGGVEVLEVKTGVDSITEVECFLTPEMGDPDEHLRGFSK 60
BAE03118.1 MAPTKRKGERKDPVQVPKLLIRGGVEVLEVKTGVDSITEVECFLTPEMGDPDEHLRGFSK 60
BAE03124.1 MAPTKRKGERKDPVQVPKLLIRGGVEVLEVKTGVDSITEVECFLTPEMGDPDEHLRGFSK 60
BAE03130.1 MAPTKRKGERKDPVQVPKLLIRGGVEVLEVKTGVDSITEVECFLTPEMGDPDEHLRGFSK 60
BAE03136.1 MAPTKRKGERKDPVQVPKLLIRGGVEVLEVKTGVDSITEVECFLTPEMGDPDEHLRGFSK 60
BAE03142.1 MAPTKRKGERKDPVQVPKLLIRGGVEVLEVKTGVDSITEVECFLTPEMGDPDEHLRGFSK 60
BAE03148.1 MAPTKRKGERKDPVQVPKLLIRGGVEVLEVKTGVDSITEVECFLTPEMGDPDEHLRGFSK 60
BAE03154.1 MAPTKRKGERKDPVQVPKLLIRGGVEVLEVKTGVDSITEVECFLTPEMGDPDEHLRGFSK 60
QBQ04200.1 MAPTKRKGERKDPVQVPKLLIRGGVEVLEVKTGVDSITEVECFLTPEMGDPDEHLRGFSK 60
UYL83839.1 MAPTKRKGERKDPVQVPKLLIRGGVEVLEVKTGVDSITEVECFLTPEMGDPDEHLRGFSK 60
ALF06262.1 MAPTKRKGERKDPVQVPKLLIRGGVEVLEVKTGVDSITEVECFLTPEMGDPDEHLRGFSK 60
ABH10586.1 MAPTKRKGERKDPVQVPKLLIRGGVEVLEVKTGVDSITEVECFLTPEMGDPDEHLRGFSK 60
BAE02890.1 MAPTKRKGERKDPVQVPKLLIRGGVEVLEVKTGVDSITEVECFLTPEMGDPDEHLRGFSK 60
BAE02896.1 MAPTKRKGERKDPVQVPKLLIRGGVEVLEVKTGVDSITEVECFLTPEMGDPDEHLRGFSK 60

```
BAE02902.1      MAPTKRKGERKDPVQVPKLLIRGGVEVLEVKTGVDSITEVECFLTPEMGDPDEHLRGFSK  60
BAE02914.1      MAPTKRKGERKDPVQVPKLLIRGGVEVLEVKTGVDSITEVECFLTPEMGDPDEHLRGFSK  60
BAE02920.1      MAPTKRKGERKDPVQVPKLLIRGGVEVLEVKTGVDSITEVECFLTPEMGDPDEHLRGFSK  60
BAE02944.1      MAPTKRKGERKDPVQVPKLLIRGGVEVLEVKTGVDSITEVECFLTPEMGDPDEHLRGFSK  60
BAE02962.1      MAPTKRKGERKDPVQVPKLLIRGGVEVLEVKTGVDSITEVECFLTPEMGDPDEHLRGFSK  60
BAE02968.1      MAPTKRKGERKDPVQVPKLLIRGGVEVLEVKTGVDSITEVECFLTPEMGDPDEHLRGFSK  60
BAE02974.1      MAPTKRKGERKDPVQVPKLLIRGGVEVLEVKTGVDSITEVECFLTPEMGDPDEHLRGFSK  60
BAE02980.1      MAPTKRKGERKDPVQVPKLLIRGGVEVLEVKTGVDSITEVECFLTPEMGDPDEHLRGFSK  60
BAE02986.1      MAPTKRKGERKDPVQVPKLLIRGGVEVLEVKTGVDSITEVECFLTPEMGDPDEHLRGFSK  60
BAE02992.1      MAPTKRKGERKDPVQVPKLLIRGGVEVLEVKTGVDSITEVECFLTPEMGDPDEHLRGFSK  60
BAE02998.1      MAPTKRKGERKDPVQVPKLLIRGGVEVLEVKTGVDSITEVECFLTPEMGDPDEHLRGFSK  60
BAE03010.1      MAPTKRKGERKDPVQVPKLLIRGGVEVLEVKTGVDSITEVECFLTPEMGDPDEHLRGFSK  60
BAE03016.1      MAPTKRKGERKDPVQVPKLLIRGGVEVLEVKTGVDSITEVECFLTPEMGDPDEHLRGFSK  60
BAE03022.1      MAPTKRKGERKDPVQVPKLLIRGGVEVLEVKTGVDSITEVECFLTPEMGDPDEHLRGFSK  60
BAE03028.1      MAPTKRKGERKDPVQVPKLLIRGGVEVLEVKTGVDSITEVECFLTPEMGDPDEHLRGFSK  60
BAE03034.1      MAPTKRKGERKDPVQVPKLLIRGGVEVLEVKTGVDSITEVECFLTPEMGDPDEHLRGFSK  60
BAE03040.1      MAPTKRKGERKDPVQVPKLLIRGGVEVLEVKTGVDSITEVECFLTPEMGDPDEHLRGFSK  60
BAE03046.1      MAPTKRKGERKDPVQVPKLLIRGGVEVLEVKTGVDSITEVECFLTPEMGDPDEHLRGFSK  60
BAE03052.1      MAPTKRKGERKDPVQVPKLLIRGGVEVLEVKTGVDSITEVECFLTPEMGDPDEHLRGFSK  60
BAE03058.1      MAPTKRKGERKDPVQVPKLLIRGGVEVLEVKTGVDSITEVECFLTPEMGDPDEHLRGFSK  60
BAE03064.1      MAPTKRKGERKDPVQVPKLLIRGGVEVLEVKTGVDSITEVECFLTPEMGDPDEHLRGFSK  60
BAE03070.1      MAPTKRKGERKDPVQVPKLLIRGGVEVLEVKTGVDSITEVECFLTPEMGDPDEHLRGFSK  60
BAE03076.1      MAPTKRKGERKDPVQVPKLLIRGGVEVLEVKTGVDSITEVECFLTPEMGDPDEHLRGFSK  60
BAE03082.1      MAPTKRKGERKDPVQVPKLLIRGGVEVLEVKTGVDSITEVECFLTPEMGDPDEHLRGFSK  60
BAE03088.1      MAPTKRKGERKDPVQVPKLLIRGGVEVLEVKTGVDSITEVECFLTPEMGDPDEHLRGFSK  60
BAE03094.1      MAPTKRKGERKDPVQVPKLLIRGGVEVLEVKTGVDSITEVECFLTPEMGDPDEHLRGFSK  60
BAE03100.1      MAPTKRKGERKDPVQVPKLLIRGGVEVLEVKTGVDSITEVECFLTPEMGDPDEHLRGFSK  60
BAE03106.1      MAPTKRKGERKDPVQVPKLLIRGGVEVLEVKTGVDSITEVECFLTPEMGDPDEHLRGFSK  60
BAE03112.1      MAPTKRKGERKDPVQVPKLLIRGGVEVLEVKTGVDSITEVECFLTPEMGDPDEHLRGFSK  60
BAE03160.1      MAPTKRKGERKDPVQVPKLLIRGGVEVLEVKTGVDSITEVECFLTPEMGDPDEHLRGFSK  60
BAE03166.1      MAPTKRKGERKDPVQVPKLLIRGGVEVLEVKTGVDSITEVECFLTPEMGDPDEHLRGFSK  60
AAQ88264.1      MAPTKRKGERKDPVQVPKLLIRGGVEVLEVKTGVDSITEVECFLTPEMGDPDEHLRGFSK  60
AAQ88270.1      MAPTKRKGERKDPVQVPKLLIRGGVEVLEVKTGVDSITEVECFLTPEMGDPDEHLRGFSK  60
AAR89187.1      MAPTKRKGERKDPVQVPKLLIRGGVEVLEVKTGVDSITEVECFLTPEMGDPDEHLRGFSK  60
AAR89193.1      MAPTKRKGERKDPVQVPKLLIRGGVEVLEVKTGVDSITEVECFLTPEMGDPDEHLRGFSK  60
AAR89199.1      MAPTKRKGERKDPVQVPKLLIRGGVEVLEVKTGVDSITEVECFLTPEMGDPDEHLRGFSK  60
AAR89205.1      MAPTKRKGERKDPVQVPKLLIRGGVEVLEVKTGVDSITEVECFLTPEMGDPDEHLRGFSK  60
AAR89217.1      MAPTKRKGERKDPVQVPKLLIRGGVEVLEVKTGVDSITEVECFLTPEMGDPDEHLRGFSK  60
AAR89223.1      MAPTKRKGERKDPVQVPKLLIRGGVEVLEVKTGVDSITEVECFLTPEMGDPDEHLRGFSK  60
AAR89229.1      MAPTKRKGERKDPVQVPKLLIRGGVEVLEVKTGVDSITEVECFLTPEMGDPDEHLRGFSK  60
AAR89235.1      MAPTKRKGERKDPVQVPKLLIRGGVEVLEVKTGVDSITEVECFLTPEMGDPDEHLRGFSK  60
AAR89241.1      MAPTKRKGERKDPVQVPKLLIRGGVEVLEVKTGVDSITEVECFLTPEMGDPDEHLRGFSK  60
AAR89247.1      MAPTKRKGERKDPVQVPKLLIRGGVEVLEVKTGVDSITEVECFLTPEMGDPDEHLRGFSK  60
AAR89253.1      MAPTKRKGERKDPVQVPKLLIRGGVEVLEVKTGVDSITEVECFLTPEMGDPDEHLRGFSK  60
AAR32743.1      MAPTKRKGERKDPVQVPKLLIRGGVEVLEVKTGVDSITEVECFLTPEMGDPDEHLRGFSK  60
AAR89265.1      MAPTKRKGERKDPVQVPKLLIRGGVEVLEVKTGVDSITEVECFLTPEMGDPDEHLRGFSK  60
AAR89277.1      MAPTKRKGERKDPVQVPKLLIRGGVEVLEVKTGVDSITEVECFLTPEMGDPDEHLRGFSK  60
AAR89283.1      MAPTKRKGERKDPVQVPKLLIRGGVEVLEVKTGVDSITEVECFLTPEMGDPDEHLRGFSK  60
AAR02463.1      MAPTKRKGERKDPVQVPKLLIRGGVEVLEVKTGVDSITEVECFLTPEMGDPDEHLRGFSK  60
AAR06661.1      MAPTKRKGERKDPVQVPKLLIRGGVEVLEVKTGVDSITEVECFLTPEMGDPDEHLRGFSK  60
AAR13659.1      MAPTKRKGERKDPVQVPKLLIRGGVEVLEVKTGVDSITEVECFLTPEMGDPDEHLRGFSK  60
AAR02457.1      MAPTKRKGERKDPVQVPKLLIRGGVEVLEVKTGVDSITEVECFLTPEMGDPDEHLRGFSK  60
BAB68785.1      MAPTKRKGERKDPVQVPKLLIRGGVEVLEVKTGVDSITEVECFLTPEMGDPDEHLRGFSK  60
AAK28472.1      MAPTKRKGERKDPVQVPKLLIRGGVEVLEVKTGVDSITEVECFLTPEMGDPDEHLRGFSK  60
AAG34667.1      MAPTKRKGERKDPVQVPKLLIRGGVEVLEVKTGVDSITEVECFLTPEMGDPDEHLRGFSK  60
AAG34673.1      MAPTKRKGERKDPVQVPKLLIRGGVEVLEVKTGVDSITEVECFLTPEMGDPDEHLRGFSK  60
AAB41705.1      MAPTKRKGERKDPVQVPKLLIRGGVEVLEVKTGVDSITEVECFLTPEMGDPDEHLRGFSK  60
AAB41711.1      MAPTKRKGERKDPVQVPKLLIRGGVEVLEVKTGVDSITEVECFLTPEMGDPDEHLRGFSK  60
AAB41717.1      MAPTKRKGERKDPVQVPKLLIRGGVEVLEVKTGVDSITEVECFLTPEMGDPDEHLRGFSK  60
BAE02926.1      MAPTKRKGERKDPVQVPKLLIRGGVEVLEVKTGVDSITEVECFLTPEMGDPDEHLRGFSK  60
                *.*   :.**.. .***** ***********:**. : *** ********   * ****

AEC14619.1      SISISDTFESDSPNKDMLPCYSVARIPLPNLNEDLTCGNILMWEAVTLKTEVIGVTSLMN  120
AEC15199.1      SISISDTFESDSPNKDMLPCYSVARIPLPNLNEDLTCGNILMWEAVTLKTEVIGVTSLMN  120
```

```
AEC15209.1        SISISDTFESDSPNKDMLPCYSVARIPLPNLNEDLTCGNILMWEAVTLKTEVIGVTSLMN  120
UPN63098.1        SISISDTFESDSPNRDMLPCYSVARIPLPNLNEDLTCGNILMWEAVTLKTEVIGVTSLMN  120
UPN63097.1        SISISDTFESDSPNRDVLPCYSVARIPLPNLNEDLTCGNILMWEAVTLKTEVIGVTSLMN  120
UPN63094.1        SISISDTFESDSPNRDMLPCYSVARIPLPNLNEDLTCGNILMWEAVTLKTEVIGVTSLMN  120
UPN63096.1        SISISDTFESDSPNRDMLPCYSVARIPLPNLNEDLTCGNILMWEAVTLKTEVIGVTSLMN  120
QWT77146.1        SISISDTFESDSPNRDMLPCYSVARIPLPNLNEDLTCGNILMWEAVTLKTEVIGVTSLMN  120
UPN63095.1        SISISDTFESDSPNRDMLPCYSVARIPLPNLNEDLTCGNILMWEAVTLKTEVIGVTSLMN  120
BAA01966.1        SISISHTFESDSPNRDMLPCYSVARIPLPNLNEDLTCGNILMWEAVTLKTEVIGVTSLMN  120
AEC15099.1        SISISDTFESDSPNRDMLPCYSVARIPLPNLNEDLTCGNILMWEAVTLKTEVIGVTSLMN  120
UPN63099.1        SISISDTFESDSPNRDMLPCYSVARIPLPNLNEDLTCGNILMWEAVTLKTEVIGVTSLMN  120
UPN63086.1        SISISDTFESDSPNRDMLPCYSVARIPLPNLNEDLTCGNILMWEAVTLKTEVIGVTSLMN  120
UPN63092.1        SISISDTFESDSPNRDMLPCYSVARIPLPNLNEDLTCGNILMWEAVTLKTEVIGVTSLMN  120
AEC15783.1        SISISDTFESDSPNRDMLPCYSVARIPLPNLNEDLTCGNILMWEAVTLKTEVIGVTSLMN  120
AEC15735.1        SISISDTFESDSPNRDMLPCYSVARIPLPNLNEDLTCGNILMWEAVTLKTEVIGVTSLMN  120
AEC15615.1        SISISDTFESDSPNRDMLPCYSVARIPLPNLNEDLTCGNILMWEAVTLKTEVIGVTSLMN  120
AEC15549.1        SISISDTFESDSPNRDMLPCYSVARIPLPNLNEDLTCGNILMWEAVTLKTEVIGVTSLMN  120
AEC15081.1        SISISDTFESDSPNRDMLPCYSVARIPLPNLNEDLTCGNILMWEAVTLKTEVIGVTSLMN  120
AEC15080.1        SISISDTFESDSPNRDMLPCYSVARIPLPNLNEDLTCGNILMWEAVTLKTEVIGVTSLMN  120
AEC15083.1        SISISDTFESDSPNRDMLPCYSVARIPLPNLNEDLTCGNILMWEAVTLKTEVIGVTSLMN  120
QWT77109.1        SISISDTFESDSPNRDMLPCYSVARIPLPNLNEDLTCGNILMWEAVTLKTEVIGVTSLMN  120
AEC15069.1        SISISDTFESDSPNRDMLPCYSVARIPLPNLNEDLTCGNILMWEAVTLKTEVIGVTSLMN  120
AEC15070.1        SISISDTFESDSPNRDMLPCYSVARIPLPNLNEDLTCGNILMWEAVTLKTEVIGVTSLMN  120
AEC15071.1        SISISDTFESDSPNRDMLPCYSVARIPLPNLNEDLTCGNILMWEAVTLKTEVIGVTSLMN  120
AEC15072.1        SISISDTFESDSPNRDMLPCYSVARIPLPNLNEDLTCGNILMWEAVTLKTEVIGVTSLMN  120
AEC15073.1        SISISDTFESDSPNRDMLPCYSVARIPLPNLNEDLTCGNILMWEAVTLKTEVIGVTSLMN  120
AEC15074.1        SISISDTFESDSPNRDMLPCYSVARIPLPNLNEDLTCGNILMWEAVTLKTEVIGVTSLMN  120
AEC15076.1        SISISDTFESDSPNRDMLPCYSVARIPLPNLNEDLTCGNILMWEAVTLKTEVIGVTSLMN  120
AEC15077.1        SISISDTFESDSPNRDMLPCYSVARIPLPNLNEDLTCGNILMWEAVTLKTEVIGVTSLMN  120
AEC15078.1        SISISDTFESDSPNRDMLPCYSVARIPLPNLNEDLTCGNILMWEAVTLKTEVIGVTSLMN  120
AEC15079.1        SISISDTFESDSPNRDMLPCYSVARIPLPNLNEDLTCGNILMWEAVTLKTEVIGVTSLMN  120
AEC15082.1        SISISDTFESDSPNRDMLPCYSVARIPLPNLNEDLTCGNILMWEAVTLKTEVIGVTSLMN  120
AEC15084.1        SISISDTFESDSPNRDMLPCYSVARIPLPNLNEDLTCGNILMWEAVTLKTEVIGVTSLMN  120
AEC15085.1        SISISDTFESDSPNRDMLPCYSVARIPLPNLNEDLTCGNILMWEAVTLKTEVIGVTSLMN  120
AEC15086.1        SISISDTFESDSPNRDMLPCYSVARIPLPNLNEDLTCGNILMWEAVTLKTEVIGVTSLMN  120
AEC15087.1        SISISDTFESDSPNRDMLPCYSVARIPLPNLNEDLTCGNILMWEAVTLKTEVIGVTSLMN  120
AEC15089.1        SISISDTFESDSPNRDMLPCYSVARIPLPNLNEDLTCGNILMWEAVTLKTEVIGVTSLMN  120
AEC15555.1        SISISDTFESDSPNRDMLPCYSVARIPLPNLNEDLTCGNILMWEAVTLKTEVIGVTSLMN  120
AEC15561.1        SISISDTFESDSPNRDMLPCYSVARIPLPNLNEDLTCGNILMWEAVTLKTEVIGVTSLMN  120
AEC15567.1        SISISDTFESDSPNRDMLPCYSVARIPLPNLNEDLTCGNILMWEAVTLKTEVIGVTSLMN  120
AEC15573.1        SISISDTFESDSPNRDMLPCYSVARIPLPNLNEDLTCGNILMWEAVTLKTEVIGVTSLMN  120
AEC15579.1        SISISDTFESDSPNRDMLPCYSVARIPLPNLNEDLTCGNILMWEAVTLKTEVIGVTSLMN  120
AEC15585.1        SISISDTFESDSPNRDMLPCYSVARIPLPNLNEDLTCGNILMWEAVTLKTEVIGVTSLMN  120
AEC15591.1        SISISDTFESDSPNRDMLPCYSVARIPLPNLNEDLTCGNILMWEAVTLKTEVIGVTSLMN  120
AEC15597.1        SISISDTFESDSPNRDMLPCYSVARIPLPNLNEDLTCGNILMWEAVTLKTEVIGVTSLMN  120
AEC15603.1        SISISDTFESDSPNRDMLPCYSVARIPLPNLNEDLTCGNILMWEAVTLKTEVIGVTSLMN  120
AEC15609.1        SISISDTFESDSPNRDMLPCYSVARIPLPNLNEDLTCGNILMWEAVTLKTEVIGVTSLMN  120
AEC15621.1        SISISDTFESDSPNRDMLPCYSVARIPLPNLNEDLTCGNILMWEAVTLKTEVIGVTSLMN  120
AEC15627.1        SISISDTFESDSPNRDMLPCYSVARIPLPNLNEDLTCGNILMWEAVTLKTEVIGVTSLMN  120
AEC15633.1        SISISDTFESDSPNRDMLPCYSVARIPLPNLNEDLTCGNILMWEAVTLKTEVIGVTSLMN  120
AEC15639.1        SISISDTFESDSPNRDMLPCYSVARIPLPNLNEDLTCGNILMWEAVTLKTEVIGVTSLMN  120
AEC15645.1        SISISDTFESDSPNRDMLPCYSVARIPLPNLNEDLTCGNILMWEAVTLKTEVIGVTSLMN  120
AEC15651.1        SISISDTFESDSPNRDMLPCYSVARIPLPNLNEDLTCGNILMWEAVTLKTEVIGVTSLMN  120
AEC15657.1        SISISDTFESDSPNRDMLPCYSVARIPLPNLNEDLTCGNILMWEAVTLKTEVIGVTSLMN  120
AEC15663.1        SISISDTFESDSPNRDMLPCYSVARIPLPNLNEDLTCGNILMWEAVTLKTEVIGVTSLMN  120
AEC15669.1        SISISDTFESDSPNRDMLPCYSVARIPLPNLNEDLTCGNILMWEAVTLKTEVIGVTSLMN  120
AEC15675.1        SISISDTFESDSPNRDMLPCYSVARIPLPNLNEDLTCGNILMWEAVTLKTEVIGVTSLMN  120
AEC15681.1        SISISDTFESDSPNRDMLPCYSVARIPLPNLNEDLTCGNILMWEAVTLKTEVIGVTSLMN  120
AEC15687.1        SISISDTFESDSPNRDMLPCYSVARIPLPNLNEDLTCGNILMWEAVTLKTEVIGVTSLMN  120
AEC15693.1        SISISDTFESDSPNRDMLPCYSVARIPLPNLNEDLTCGNILMWEAVTLKTEVIGVTSLMN  120
AEC15699.1        SISISDTFESDSPNRDMLPCYSVARIPLPNLNEDLTCGNILMWEAVTLKTEVIGVTSLMN  120
AEC15705.1        SISISDTFESDSPNRDMLPCYSVARIPLPNLNEDLTCGNILMWEAVTLKTEVIGVTSLMN  120
AEC15711.1        SISISDTFESDSPNRDMLPCYSVARIPLPNLNEDLTCGNILMWEAVTLKTEVIGVTSLMN  120
AEC15717.1        SISISDTFESDSPNRDMLPCYSVARIPLPNLNEDLTCGNILMWEAVTLKTEVIGVTSLMN  120
AEC15723.1        SISISDTFESDSPNRDMLPCYSVARIPLPNLNEDLTCGNILMWEAVTLKTEVIGVTSLMN  120
```

AEC15729.1 SISISDTFESDSPNRDMLPCYSVARIPLPNLNEDLTCGNILMWEAVTLKTEVIGVTSLMN 120
AEC15741.1 SISISDTFESDSPNRDMLPCYSVARIPLPNLNEDLTCGNILMWEAVTLKTEVIGVTSLMN 120
AEC15747.1 SISISDTFESDSPNRDMLPCYSVARIPLPNLNEDLTCGNILMWEAVTLKTEVIGVTSLMN 120
AEC15753.1 SISISDTFESDSPNRDMLPCYSVARIPLPNLNEDLTCGNILMWEAVTLKTEVIGVTSLMN 120
AEC15759.1 SISISDTFESDSPNRDMLPCYSVARIPLPNLNEDLTCGNILMWEAVTLKTEVIGVTSLMN 120
AEC15765.1 SISISDTFESDSPNRDMLPCYSVARIPLPNLNEDLTCGNILMWEAVTLKTEVIGVTSLMN 120
AEC15771.1 SISISDTFESDSPNRDMLPCYSVARIPLPNLNEDLTCGNILMWEAVTLKTEVIGVTSLMN 120
AEC15777.1 SISISDTFESDSPNRDMLPCYSVARIPLPNLNEDLTCGNILMWEAVTLKTEVIGVTSLMN 120
AEC15789.1 SISISDTFESDSPNRDMLPCYSVARIPLPNLNEDLTCGNILMWEAVTLKTEVIGVTSLMN 120
AEC15795.1 SISISDTFESDSPNRDMLPCYSVARIPLPNLNEDLTCGNILMWEAVTLKTEVIGVTSLMN 120
AEC15801.1 SISISDTFESDSPNRDMLPCYSVARIPLPNLNEDLTCGNILMWEAVTLKTEVIGVTSLMN 120
AEC15807.1 SISISDTFESDSPNRDMLPCYSVARIPLPNLNEDLTCGNILMWEAVTLKTEVIGVTSLMN 120
AEC15813.1 SISISDTFESDSPNRDMLPCYSVARIPLPNLNEDLTCGNILMWEAVTLKTEVIGVTSLMN 120
AEC15075.1 SISISDTFESDSPNRDMLPCYSVARIPLPNLNEDLTCGNILMWEAVTLKTEVIGVTSLMN 120
AEC15088.1 SISISDTFESDSPNRDMLPCYSVARIPLPNLNEDLTCGNILMWEAVTLKTEVIGVTSLMN 120
AEC14559.1 SISISDTFESDSPNKDMLPCYSVARIPLPNLNEDLTCGNILMWEAVTLKTEVIGVTSLMN 120
AEC15023.1 SISISDTFESDSPNKDMLPCYSVARIPLPNLNEDLTCGNILMWEAVTLKTEVIGVTSLMN 120
AEC14518.1 SISISDTFESDSPNKDMLPCYSVARIPLPNLNEDLTCGNILMWEAVTLKTEVIGVTSLMN 120
AEC14589.1 SISISDTFESDSPNKDMLPCYSVARIPLPNLNEDLTCGNILMWEAVTLKTEVIGVTSLMN 120
AEC14625.1 SISISDTFESDSPNKDMLPCYSVARIPLPNLNEDLTCGNILMWEAVTLKTEVIGVTSLMN 120
AEC14631.1 SISISDTFESDSPNKDMLPCYSVARIPLPNLNEDLTCGNILMWEAVTLKTEVIGVTSLMN 120
AEC14643.1 SISISDTFESDSPNKDMLPCYSVARIPLPNLNEDLTCGNILMWEAVTLKTEVIGVTSLMN 120
AEC14966.1 SISISDTFESDSPNKDMLPCYSVARIPLPNLNEDLTCGNILMWEAVTLKTEVIGVTSLMN 120
AEC14979.1 SISISDTFESDSPNKDMLPCYSVARIPLPNLNEDLTCGNILMWEAVTLKTEVIGVTSLMN 120
AEC14990.1 SISISDTFESDSPNKDMLPCYSVARIPLPNLNEDLTCGNILMWEAVTLKTEVIGVTSLMN 120
AEC14999.1 SISISDTFESDSPNKDMLPCYSVARIPLPNLNEDLTCGNILMWEAVTLKTEVIGVTSLMN 120
AEC15020.1 SISISDTFESDSPNKDMLPCYSVARIPLPNLNEDLTCGNILMWEAVTLKTEVIGVTSLMN 120
AEC14595.1 SISISDTFESDSPNKDMLPCYSVARIPLPNLNEDLTCGNILMWEAVTLKTEVIGVTSLMN 120
AEC14703.1 SISISDTFESDSPNKDMLPCYSVARIPLPNLNEDLTCGNILMWEAVTLKTEVIGVTSLMN 120
AEC15011.1 SISISDTFESDSPNKDMLPCYSVARIPLPNLNEDLTCGNILMWEAVTLKTEVIGVTSLMN 120
AEC15019.1 SISISDTFESDSPNKDMLPCYSVARIPLPNLNEDLTCGNILMWEAVTLKTEVIGVTSLMN 120
AEC15014.1 SISISDTFESDSPNKDMLPCYSVARIPLPNLNEDLTCGNILMWEAVTLKTEVIGVTSLMN 120
NP_043511.1 SISISDTFESDSPNRDMLPCYSVARIPLPNLNEDLTCGNILMWEAVTLKTEVIGVTSLMN 120
UPN63087.1 SISISDTFESDSPNRDMLPCYSVARIPLPNLNEDLTCGNILMWEAVTLKTEVIGVTSLMN 120
ASV51760.1 SISISDTFESDSPNRDMLPCYSVARIPLPNLNEDLTCGNILMWEAVTLKTEVIGVTSLMN 120
BAV69379.1 SISISDTFESDSPNRDMLPCYSVARIPLPNLNEDLTCGNILMWEAVTLKTEVIGVTSLMN 120
AEC15090.1 SISISDTFESDSPNRDMLPCYSVARIPLPNLNEDLTCGNILMWEAVTLKTEVIGVTSLMN 120
AEC15091.1 SISISDTFESDSPNRDMLPCYSVARIPLPNLNEDLTCGNILMWEAVTLKTEVIGVTSLMN 120
AEC15092.1 SISISDTFESDSPNRDMLPCYSVARIPLPNLNEDLTCGNILMWEAVTLKTEVIGVTSLMN 120
AEC15093.1 SISISDTFESDSPNRDMLPCYSVARIPLPNLNEDLTCGNILMWEAVTLKTEVIGVTSLMN 120
AEC15094.1 SISISDTFESDSPNRDMLPCYSVARIPLPNLNEDLTCGNILMWEAVTLKTEVIGVTSLMN 120
AEC15095.1 SISISDTFESDSPNRDMLPCYSVARIPLPNLNEDLTCGNILMWEAVTLKTEVIGVTSLMN 120
AEC15096.1 SISISDTFESDSPNRDMLPCYSVARIPLPNLNEDLTCGNILMWEAVTLKTEVIGVTSLMN 120
AEC15097.1 SISISDTFESDSPNRDMLPCYSVARIPLPNLNEDLTCGNILMWEAVTLKTEVIGVTSLMN 120
AEC15098.1 SISISDTFESDSPNRDMLPCYSVARIPLPNLNEDLTCGNILMWEAVTLKTEVIGVTSLMN 120
AEC15100.1 SISISDTFESDSPNRDMLPCYSVARIPLPNLNEDLTCGNILMWEAVTLKTEVIGVTSLMN 120
AEC15101.1 SISISDTFESDSPNRDMLPCYSVARIPLPNLNEDLTCGNILMWEAVTLKTEVIGVTSLMN 120
AEC15102.1 SISISDTFESDSPNRDMLPCYSVARIPLPNLNEDLTCGNILMWEAVTLKTEVIGVTSLMN 120
AEC15103.1 SISISDTFESDSPNRDMLPCYSVARIPLPNLNEDLTCGNILMWEAVTLKTEVIGVTSLMN 120
AEC15104.1 SISISDTFESDSPNRDMLPCYSVARIPLPNLNEDLTCGNILMWEAVTLKTEVIGVTSLMN 120
AEC15105.1 SISISDTFESDSPNRDMLPCYSVARIPLPNLNEDLTCGNILMWEAVTLKTEVIGVTSLMN 120
AEC15106.1 SISISDTFESDSPNRDMLPCYSVARIPLPNLNEDLTCGNILMWEAVTLKTEVIGVTSLMN 120
AEC15107.1 SISISDTFESDSPNRDMLPCYSVARIPLPNLNEDLTCGNILMWEAVTLKTEVIGVTSLMN 120
AEC15108.1 SISISDTFESDSPNRDMLPCYSVARIPLPNLNEDLTCGNILMWEAVTLKTEVIGVTSLMN 120
AEC15109.1 SISISDTFESDSPNRDMLPCYSVARIPLPNLNEDLTCGNILMWEAVTLKTEVIGVTSLMN 120
AEC15110.1 SISISDTFESDSPNRDMLPCYSVARIPLPNLNEDLTCGNILMWEAVTLKTEVIGVTSLMN 120
AEC15111.1 SISISDTFESDSPNRDMLPCYSVARIPLPNLNEDLTCGNILMWEAVTLKTEVIGVTSLMN 120
AEC15963.1 SISISDTFESDSPNRDMLPCYSVARIPLPNLNEDLTCGNILMWEAVTLKTEVIGVTSLMN 120
AEC15969.1 SISISDTFESDSPNRDMLPCYSVARIPLPNLNEDLTCGNILMWEAVTLKTEVIGVTSLMN 120
AEC15975.1 SISISDTFESDSPNRDMLPCYSVARIPLPNLNEDLTCGNILMWEAVTLKTEVIGVTSLMN 120
AEC15981.1 SISISDTFESDSPNRDMLPCYSVARIPLPNLNEDLTCGNILMWEAVTLKTEVIGVTSLMN 120
AEC15987.1 SISISDTFESDSPNRDMLPCYSVARIPLPNLNEDLTCGNILMWEAVTLKTEVIGVTSLMN 120
AEC15993.1 SISISDTFESDSPNRDMLPCYSVARIPLPNLNEDLTCGNILMWEAVTLKTEVIGVTSLMN 120
CAD69038.1 SISISDTFESDSPNRDMLPCYSVARIPLPNLNEDLTCGNILMWEAVTLKTEVIGVTSLMN 120

AAK70240.1	SISISDTFESDSPNRDMLPCYSVARIPLPNLNEDLTCGNILMWEAVTLKTEVIGVTSLMN	120
BAB11710.1	SISISDTFESDSPNRDMLPCYSVARIPLPNLNEDLTCGNILMWEAVTLKTEVIGVTSLMN	120
BAA01958.1	SISISDTFESDSPNRDMLPCYSVARIPLPNLNEDLTCGNILMWEAVTLKTEVIGVTSLMN	120
AAA82101.1	SISISDTFESDSPNRDMLPCYSVARIPLPNLNEDLTCGNILMWEAVTLKTEVIGVTSLMN	120
P03089.1	SISISDTFESDSPNRDMLPCYSVARIPLPNLNEDLTCGNILMWEAVTLKTEVIGVTSLMN	120
UPN63102.1	SISISDTFESDSPNKDMLPCYSVARIPLPNLNEDLTCGNILMWEAVTLKTEVIGVTSLMN	120
AEC15025.1	SISISDTFESDSPNKDMLPCYSVARIPLPNLNEDLTCGNILMWEAVTLKTEVIGVTSLMN	120
AEC15026.1	SISISDTFESDSPNKDMLPCYSVARIPLPNLNEDLTCGNILMWEAVTLKTEVIGVTSLMN	120
AEC15027.1	SISISDTFESDSPNKDMLPCYSVARIPLPNLNEDLTCGNILMWEAVTLKTEVIGVTSLMN	120
AEC15028.1	SISISDTFESDSPNKDMLPCYSVARIPLPNLNEDLTCGNILMWEAVTLKTEVIGVTSLMN	120
AEC15029.1	SISISDTFESDSPNKDMLPCYSVARIPLPNLNEDLTCGNILMWEAVTLKTEVIGVTSLMN	120
AEC15196.1	SISISDTFESDSPNKDMLPCYSVARIPLPNLNEDLTCGNILMWEAVTLKTEVIGVTSLMN	120
AEC15197.1	SISISDTFESDSPNKDMLPCYSVARIPLPNLNEDLTCGNILMWEAVTLKTEVIGVTSLMN	120
AEC15198.1	SISISDTFESDSPNKDMLPCYSVARIPLPNLNEDLTCGNILMWEAVTLKTEVIGVTSLMN	120
AEC15200.1	SISISDTFESDSPNKDMLPCYSVARIPLPNLNEDLTCGNILMWEAVTLKTEVIGVTSLMN	120
AEC15201.1	SISISDTFESDSPNKDMLPCYSVARIPLPNLNEDLTCGNILMWEAVTLKTEVIGVTSLMN	120
AEC15202.1	SISISDTFESDSPNKDMLPCYSVARIPLPNLNEDLTCGNILMWEAVTLKTEVIGVTSLMN	120
AEC15203.1	SISISDTFESDSPNKDMLPCYSVARIPLPNLNEDLTCGNILMWEAVTLKTEVIGVTSLMN	120
AEC15204.1	SISISDTFESDSPNKDMLPCYSVARIPLPNLNEDLTCGNILMWEAVTLKTEVIGVTSLMN	120
AEC15205.1	SISISDTFESDSPNKDMLPCYSVARIPLPNLNEDLTCGNILMWEAVTLKTEVIGVTSLMN	120
AEC15206.1	SISISDTFESDSPNKDMLPCYSVARIPLPNLNEDLTCGNILMWEAVTLKTEVIGVTSLMN	120
AEC15207.1	SISISDTFESDSPNKDMLPCYSVARIPLPNLNEDLTCGNILMWEAVTLKTEVIGVTSLMN	120
AEC15208.1	SISISDTFESDSPNKDMLPCYSVARIPLPNLNEDLTCGNILMWEAVTLKTEVIGVTSLMN	120
AEC15210.1	SISISDTFESDSPNKDMLPCYSVARIPLPNLNEDLTCGNILMWEAVTLKTEVIGVTSLMN	120
AEC15211.1	SISISDTFESDSPNKDMLPCYSVARIPLPNLNEDLTCGNILMWEAVTLKTEVIGVTSLMN	120
AEC15212.1	SISISDTFESDSPNKDMLPCYSVARIPLPNLNEDLTCGNILMWEAVTLKTEVIGVTSLMN	120
AEC15213.1	SISISDTFESDSPNKDMLPCYSVARIPLPNLNEDLTCGNILMWEAVTLKTEVIGVTSLMN	120
AEC15214.1	SISISDTFESDSPNKDMLPCYSVARIPLPNLNEDLTCGNILMWEAVTLKTEVIGVTSLMN	120
AEC15215.1	SISISDTFESDSPNKDMLPCYSVARIPLPNLNEDLTCGNILMWEAVTLKTEVIGVTSLMN	120
AEC15216.1	SISISDTFESDSPNKDMLPCYSVARIPLPNLNEDLTCGNILMWEAVTLKTEVIGVTSLMN	120
AEC15217.1	SISISDTFESDSPNKDMLPCYSVARIPLPNLNEDLTCGNILMWEAVTLKTEVIGVTSLMN	120
AEC15218.1	SISISDTFESDSPNKDMLPCYSVARIPLPNLNEDLTCGNILMWEAVTLKTEVIGVTSLMN	120
AEC15327.1	SISISDTFESDSPNKDMLPCYSVARIPLPNLNEDLTCGNILMWEAVTLKTEVIGVTSLMN	120
AEC15333.1	SISISDTFESDSPNKDMLPCYSVARIPLPNLNEDLTCGNILMWEAVTLKTEVIGVTSLMN	120
AEC15339.1	SISISDTFESDSPNKDMLPCYSVARIPLPNLNEDLTCGNILMWEAVTLKTEVIGVTSLMN	120
AEC15345.1	SISISDTFESDSPNKDMLPCYSVARIPLPNLNEDLTCGNILMWEAVTLKTEVIGVTSLMN	120
AEC15351.1	SISISDTFESDSPNKDMLPCYSVARIPLPNLNEDLTCGNILMWEAVTLKTEVIGVTSLMN	120
AEC15357.1	SISISDTFESDSPNKDMLPCYSVARIPLPNLNEDLTCGNILMWEAVTLKTEVIGVTSLMN	120
AEC15363.1	SISISDTFESDSPNKDMLPCYSVARIPLPNLNEDLTCGNILMWEAVTLKTEVIGVTSLMN	120
AEC15369.1	SISISDTFESDSPNKDMLPCYSVARIPLPNLNEDLTCGNILMWEAVTLKTEVIGVTSLMN	120
AEC15375.1	SISISDTFESDSPNKDMLPCYSVARIPLPNLNEDLTCGNILMWEAVTLKTEVIGVTSLMN	120
AEC15381.1	SISISDTFESDSPNKDMLPCYSVARIPLPNLNEDLTCGNILMWEAVTLKTEVIGVTSLMN	120
AEC15387.1	SISISDTFESDSPNKDMLPCYSVARIPLPNLNEDLTCGNILMWEAVTLKTEVIGVTSLMN	120
AEC15393.1	SISISDTFESDSPNKDMLPCYSVARIPLPNLNEDLTCGNILMWEAVTLKTEVIGVTSLMN	120
AEC15399.1	SISISDTFESDSPNKDMLPCYSVARIPLPNLNEDLTCGNILMWEAVTLKTEVIGVTSLMN	120
AEC15405.1	SISISDTFESDSPNKDMLPCYSVARIPLPNLNEDLTCGNILMWEAVTLKTEVIGVTSLMN	120
AEC15411.1	SISISDTFESDSPNKDMLPCYSVARIPLPNLNEDLTCGNILMWEAVTLKTEVIGVTSLMN	120
AEC15417.1	SISISDTFESDSPNKDMLPCYSVARIPLPNLNEDLTCGNILMWEAVTLKTEVIGVTSLMN	120
AEC15423.1	SISISDTFESDSPNKDMLPCYSVARIPLPNLNEDLTCGNILMWEAVTLKTEVIGVTSLMN	120
AEC15429.1	SISISDTFESDSPNKDMLPCYSVARIPLPNLNEDLTCGNILMWEAVTLKTEVIGVTSLMN	120
AEC15435.1	SISISDTFESDSPNKDMLPCYSVARIPLPNLNEDLTCGNILMWEAVTLKTEVIGVTSLMN	120
AEC15441.1	SISISDTFESDSPNKDMLPCYSVARIPLPNLNEDLTCGNILMWEAVTLKTEVIGVTSLMN	120
BAA01967.1	SISISDTFESDSPNKDMLPCYSVARIPLPNLNEDLTCGNILMWEAVTLKTEVIGVTSLMN	120
ASV51844.1	SISISDTFESDSPNKDMLPCYSVARIPLPNLNEDLTCGNILMWEAVTLKTEVIGVTSLMN	120
ASV51850.1	SISISDTFESDSPNKDMLPCYSVARIPLPNLNEDLTCGNILMWEAVTLKTEVIGVTSLMN	120
CCW72489.1	SISISDTFESDSPNKDMLPCYSVARIPLPNLNEDLTCGNILMWEAVTLKTEVIGVTSLMN	120
AEC15170.1	SISISDTFESDSPNKDMLPCYSVARIPLPNLNEDLTCGNILMWEAVTLKTEVIGVTSLMN	120
AEC15171.1	SISISDTFESDSPNKDMLPCYSVARIPLPNLNEDLTCGNILMWEAVTLKTEVIGVTSLMN	120
AEC15299.1	SISISDTFESDSPNKDMLPCYSVARIPLPNLNEDLTCGNILMWEAVTLKTEVIGVTSLMN	120
BAB93050.1	SISISDTFDSDSPNKDMLPCYSVARIPLPNLNEDLTCGNILMWEAVTLKTEVIGVTSLMN	120
UPN63100.1	SISISDTFESDSPNKDMLPCYSVARIPLPNLNEDLTCGNILMWEAVTLKTEVIGVTSLMN	120
CCW72482.1	SISISDTFESDSPNKDMLPCYSVARIPLPNLNEDLTCGNILMWEAVTLKTEVIGVTSLMN	120
AEC15165.1	SISISDTFESDSPNKDMLPCYSVARIPLPNLNEDLTCGNILMWEAVTLKTEVIGVTSLMN	120
AEC15166.1	SISISDTFESDSPNKDMLPCYSVARIPLPNLNEDLTCGNILMWEAVTLKTEVIGVTSLMN	120

```
AEC15266.1      SISISDTFESDSPNKDMLPCYSVARIPLPNLNEDLTCGNILMWEAVTLKTEVIGVTSLMN  120
AEC15267.1      SISISDTFESDSPNKDMLPCYSVARIPLPNLNEDLTCGNILMWEAVTLKTEVIGVTSLMN  120
AEC15268.1      SISISDTFESDSPNKDMLPCYSVARIPLPNLNEDLTCGNILMWEAVTLKTEVIGVTSLMN  120
AEC15269.1      SISISDTFESDSPNKDMLPCYSVARIPLPNLNEDLTCGNILMWEAVTLKTEVIGVTSLMN  120
AEC15270.1      SISISDTFESDSPNKDMLPCYSVARIPLPNLNEDLTCGNILMWEAVTLKTEVIGVTSLMN  120
AEC15271.1      SISISDTFESDSPNKDMLPCYSVARIPLPNLNEDLTCGNILMWEAVTLKTEVIGVTSLMN  120
AEC15272.1      SISISDTFESDSPNKDMLPCYSVARIPLPNLNEDLTCGNILMWEAVTLKTEVIGVTSLMN  120
AEC15273.1      SISISDTFESDSPNKDMLPCYSVARIPLPNLNEDLTCGNILMWEAVTLKTEVIGVTSLMN  120
AEC15274.1      SISISDTFESDSPNKDMLPCYSVARIPLPNLNEDLTCGNILMWEAVTLKTEVIGVTSLMN  120
AEC15275.1      SISISDTFESDSPNKDMLPCYSVARIPLPNLNEDLTCGNILMWEAVTLKTEVIGVTSLMN  120
AEC15276.1      SISISDTFESDSPNKDMLPCYSVARIPLPNLNEDLTCGNILMWEAVTLKTEVIGVTSLMN  120
AEC15277.1      SISISDTFESDSPNKDMLPCYSVARIPLPNLNEDLTCGNILMWEAVTLKTEVIGVTSLMN  120
AEC15278.1      SISISDTFESDSPNKDMLPCYSVARIPLPNLNEDLTCGNILMWEAVTLKTEVIGVTSLMN  120
AEC15279.1      SISISDTFESDSPNKDMLPCYSVARIPLPNLNEDLTCGNILMWEAVTLKTEVIGVTSLMN  120
AEC15280.1      SISISDTFESDSPNKDMLPCYSVARIPLPNLNEDLTCGNILMWEAVTLKTEVIGVTSLMN  120
AEC15281.1      SISISDTFESDSPNKDMLPCYSVARIPLPNLNEDLTCGNILMWEAVTLKTEVIGVTSLMN  120
AEC15282.1      SISISDTFESDSPNKDMLPCYSVARIPLPNLNEDLTCGNILMWEAVTLKTEVIGVTSLMN  120
AEC15283.1      SISISDTFESDSPNKDMLPCYSVARIPLPNLNEDLTCGNILMWEAVTLKTEVIGVTSLMN  120
AEC15284.1      SISISDTFESDSPNKDMLPCYSVARIPLPNLNEDLTCGNILMWEAVTLKTEVIGVTSLMN  120
AEC15285.1      SISISDTFESDSPNKDMLPCYSVARIPLPNLNEDLTCGNILMWEAVTLKTEVIGVTSLMN  120
AEC15286.1      SISISDTFESDSPNKDMLPCYSVARIPLPNLNEDLTCGNILMWEAVTLKTEVIGVTSLMN  120
AEC15287.1      SISISDTFESDSPNKDMLPCYSVARIPLPNLNEDLTCGNILMWEAVTLKTEVIGVTSLMN  120
AEC15288.1      SISISDTFESDSPNKDMLPCYSVARIPLPNLNEDLTCGNILMWEAVTLKTEVIGVTSLMN  120
AEC15289.1      SISISDTFESDSPNKDMLPCYSVARIPLPNLNEDLTCGNILMWEAVTLKTEVIGVTSLMN  120
AEC15290.1      SISISDTFESDSPNKDMLPCYSVARIPLPNLNEDLTCGNILMWEAVTLKTEVIGVTSLMN  120
AEC15291.1      SISISDTFESDSPNKDMLPCYSVARIPLPNLNEDLTCGNILMWEAVTLKTEVIGVTSLMN  120
AEC15292.1      SISISDTFESDSPNKDMLPCYSVARIPLPNLNEDLTCGNILMWEAVTLKTEVIGVTSLMN  120
AEC15293.1      SISISDTFESDSPNKDMLPCYSVARIPLPNLNEDLTCGNILMWEAVTLKTEVIGVTSLMN  120
AEC15294.1      SISISDTFESDSPNKDMLPCYSVARIPLPNLNEDLTCGNILMWEAVTLKTEVIGVTSLMN  120
AEC15295.1      SISISDTFESDSPNKDMLPCYSVARIPLPNLNEDLTCGNILMWEAVTLKTEVIGVTSLMN  120
AEC15296.1      SISISDTFESDSPNKDMLPCYSVARIPLPNLNEDLTCGNILMWEAVTLKTEVIGVTSLMN  120
AEC15297.1      SISISDTFESDSPNKDMLPCYSVARIPLPNLNEDLTCGNILMWEAVTLKTEVIGVTSLMN  120
AEC15298.1      SISISDTFESDSPNKDMLPCYSVARIPLPNLNEDLTCGNILMWEAVTLKTEVIGVTSLMN  120
AEC15300.1      SISISDTFESDSPNKDMLPCYSVARIPLPNLNEDLTCGNILMWEAVTLKTEVIGVTSLMN  120
AEC15301.1      SISISDTFESDSPNKDMLPCYSVARIPLPNLNEDLTCGNILMWEAVTLKTEVIGVTSLMN  120
AEC15302.1      SISISDTFESDSPNKDMLPCYSVARIPLPNLNEDLTCGNILMWEAVTLKTEVIGVTSLMN  120
AEC15303.1      SISISDTFESDSPNKDMLPCYSVARIPLPNLNEDLTCGNILMWEAVTLKTEVIGVTSLMN  120
AEC15304.1      SISISDTFESDSPNKDMLPCYSVARIPLPNLNEDLTCGNILMWEAVTLKTEVIGVTSLMN  120
AEC15305.1      SISISDTFESDSPNKDMLPCYSVARIPLPNLNEDLTCGNILMWEAVTLKTEVIGVTSLMN  120
AEC15306.1      SISISDTFESDSPNKDMLPCYSVARIPLPNLNEDLTCGNILMWEAVTLKTEVIGVTSLMN  120
AEC15307.1      SISISDTFESDSPNKDMLPCYSVARIPLPNLNEDLTCGNILMWEAVTLKTEVIGVTSLMN  120
AEC15308.1      SISISDTFESDSPNKDMLPCYSVARIPLPNLNEDLTCGNILMWEAVTLKTEVIGVTSLMN  120
AEC15309.1      SISISDTFESDSPNKDMLPCYSVARIPLPNLNEDLTCGNILMWEAVTLKTEVIGVTSLMN  120
AEC15310.1      SISISDTFESDSPNKDMLPCYSVARIPLPNLNEDLTCGNILMWEAVTLKTEVIGVTSLMN  120
AEC15311.1      SISISDTFESDSPNKDMLPCYSVARIPLPNLNEDLTCGNILMWEAVTLKTEVIGVTSLMN  120
AEC15861.1      SISISDTFESDSPNKDMLPCYSVARIPLPNLNEDLTCGNILMWEAVTLKTEVIGVTSLMN  120
AEC15867.1      SISISDTFESDSPNKDMLPCYSVARIPLPNLNEDLTCGNILMWEAVTLKTEVIGVTSLMN  120
AEC15873.1      SISISDTFESDSPNKDMLPCYSVARIPLPNLNEDLTCGNILMWEAVTLKTEVIGVTSLMN  120
AEC15879.1      SISISDTFESDSPNKDMLPCYSVARIPLPNLNEDLTCGNILMWEAVTLKTEVIGVTSLMN  120
AEC15885.1      SISISDTFESDSPNKDMLPCYSVARIPLPNLNEDLTCGNILMWEAVTLKTEVIGVTSLMN  120
AEC15891.1      SISISDTFESDSPNKDMLPCYSVARIPLPNLNEDLTCGNILMWEAVTLKTEVIGVTSLMN  120
AEC15897.1      SISISDTFESDSPNKDMLPCYSVARIPLPNLNEDLTCGNILMWEAVTLKTEVIGVTSLMN  120
AEC15903.1      SISISDTFESDSPNKDMLPCYSVARIPLPNLNEDLTCGNILMWEAVTLKTEVIGVTSLMN  120
AEC15909.1      SISISDTFESDSPNKDMLPCYSVARIPLPNLNEDLTCGNILMWEAVTLKTEVIGVTSLMN  120
AEC15915.1      SISISDTFESDSPNKDMLPCYSVARIPLPNLNEDLTCGNILMWEAVTLKTEVIGVTSLMN  120
AEC15921.1      SISISDTFESDSPNKDMLPCYSVARIPLPNLNEDLTCGNILMWEAVTLKTEVIGVTSLMN  120
AEC15927.1      SISISDTFESDSPNKDMLPCYSVARIPLPNLNEDLTCGNILMWEAVTLKTEVIGVTSLMN  120
AEC15933.1      SISISDTFESDSPNKDMLPCYSVARIPLPNLNEDLTCGNILMWEAVTLKTEVIGVTSLMN  120
AEC15939.1      SISISDTFESDSPNKDMLPCYSVARIPLPNLNEDLTCGNILMWEAVTLKTEVIGVTSLMN  120
AEC15945.1      SISISDTFESDSPNKDMLPCYSVARIPLPNLNEDLTCGNILMWEAVTLKTEVIGVTSLMN  120
AEC15951.1      SISISDTFESDSPNKDMLPCYSVARIPLPNLNEDLTCGNILMWEAVTLKTEVIGVTSLMN  120
AEC15957.1      SISISDTFESDSPNKDMLPCYSVARIPLPNLNEDLTCGNILMWEAVTLKTEVIGVTSLMN  120
BAD21259.1      SISISDTFESDSPNKDMLPCYSVARIPLPNLNEDLTCGNILMWEAVTLKTEVIGVTSLMN  120
BAD21265.1      SISISDTFESDSPNKDMLPCYSVARIPLPNLNEDLTCGNILMWEAVTLKTEVIGVTSLMN  120
```

AAC59325.1	SISISDTFESDSPNKDMLPCYSVARIPLPNLNEDLTCGNILMWEAVTLKTEVIGVTSLMN	120
AEC14524.1	SISISDTFESDSPNKDMLPCYSVARIPLPNLNEDLTCGNILMWEAVTLKTEVIGVTSLMN	120
AEC14541.1	SISISDTFESDSPNKDMLPCYSVARIPLPNLNEDLTCGNILMWEAVTLKTEVIGVTSLMN	120
AEC14565.1	SISISDTFESDSPNKDMLPCYSVARIPLPNLNEDLTCGNILMWEAVTLKTEVIGVTSLMN	120
AEC14571.1	SISISDTFESDSPNKDMLPCYSVARIPLPNLNEDLTCGNILMWEAVTLKTEVIGVTSLMN	120
AEC14583.1	SISISDTFESDSPNKDMLPCYSVARIPLPNLNEDLTCGNILMWEAVTLKTEVIGVTSLMN	120
AEC14601.1	SISISDTFESDSPNKDMLPCYSVARIPLPNLNEDLTCGNILMWEAVTLKTEVIGVTSLMN	120
AEC14607.1	SISISDTFESDSPNKDMLPCYSVARIPLPNLNEDLTCGNILMWEAVTLKTEVIGVTSLMN	120
AEC14637.1	SISISDTFESDSPNKDMLPCYSVARIPLPNLNEDLTCGNILMWEAVTLKTEVIGVTSLMN	120
AEC14649.1	SISISDTFESDSPNKDMLPCYSVARIPLPNLNEDLTCGNILMWEAVTLKTEVIGVTSLMN	120
AEC14655.1	SISISDTFESDSPNKDMLPCYSVARIPLPNLNEDLTCGNILMWEAVTLKTEVIGVTSLMN	120
AEC14661.1	SISISDTFESDSPNKDMLPCYSVARIPLPNLNEDLTCGNILMWEAVTLKTEVIGVTSLMN	120
AEC14685.1	SISISDTFESDSPNKDMLPCYSVARIPLPNLNEDLTCGNILMWEAVTLKTEVIGVTSLMN	120
AEC14697.1	SISISDTFESDSPNKDMLPCYSVARIPLPNLNEDLTCGNILMWEAVTLKTEVIGVTSLMN	120
AEC14709.1	SISISDTFESDSPNKDMLPCYSVARIPLPNLNEDLTCGNILMWEAVTLKTEVIGVTSLMN	120
AEC14715.1	SISISDTFESDSPNKDMLPCYSVARIPLPNLNEDLTCGNILMWEAVTLKTEVIGVTSLMN	120
AEC14963.1	SISISDTFESDSPNKDMLPCYSVARIPLPNLNEDLTCGNILMWEAVTLKTEVIGVTSLMN	120
AEC14968.1	SISISDTFESDSPNKDMLPCYSVARIPLPNLNEDLTCGNILMWEAVTLKTEVIGVTSLMN	120
AEC14971.1	SISISDTFESDSPNKDMLPCYSVARIPLPNLNEDLTCGNILMWEAVTLKTEVIGVTSLMN	120
AEC14972.1	SISISDTFESDSPNKDMLPCYSVARIPLPNLNEDLTCGNILMWEAVTLKTEVIGVTSLMN	120
AEC14975.1	SISISDTFESDSPNKDMLPCYSVARIPLPNLNEDLTCGNILMWEAVTLKTEVIGVTSLMN	120
AEC14976.1	SISISDTFESDSPNKDMLPCYSVARIPLPNLNEDLTCGNILMWEAVTLKTEVIGVTSLMN	120
AEC14977.1	SISISDTFESDSPNKDMLPCYSVARIPLPNLNEDLTCGNILMWEAVTLKTEVIGVTSLMN	120
AEC14978.1	SISISDTFESDSPNKDMLPCYSVARIPLPNLNEDLTCGNILMWEAVTLKTEVIGVTSLMN	120
AEC14980.1	SISISDTFESDSPNKDMLPCYSVARIPLPNLNEDLTCGNILMWEAVTLKTEVIGVTSLMN	120
AEC14981.1	SISISDTFESDSPNKDMLPCYSVARIPLPNLNEDLTCGNILMWEAVTLKTEVIGVTSLMN	120
AEC14982.1	SISISDTFESDSPNKDMLPCYSVARIPLPNLNEDLTCGNILMWEAVTLKTEVIGVTSLMN	120
AEC14983.1	SISISDTFESDSPNKDMLPCYSVARIPLPNLNEDLTCGNILMWEAVTLKTEVIGVTSLMN	120
AEC14985.1	SISISDTFESDSPNKDMLPCYSVARIPLPNLNEDLTCGNILMWEAVTLKTEVIGVTSLMN	120
AEC14986.1	SISISDTFESDSPNKDMLPCYSVARIPLPNLNEDLTCGNILMWEAVTLKTEVIGVTSLMN	120
AEC14988.1	SISISDTFESDSPNKDMLPCYSVARIPLPNLNEDLTCGNILMWEAVTLKTEVIGVTSLMN	120
AEC14989.1	SISISDTFESDSPNKDMLPCYSVARIPLPNLNEDLTCGNILMWEAVTLKTEVIGVTSLMN	120
AEC14991.1	SISISDTFESDSPNKDMLPCYSVARIPLPNLNEDLTCGNILMWEAVTLKTEVIGVTSLMN	120
AEC14992.1	SISISDTFESDSPNKDMLPCYSVARIPLPNLNEDLTCGNILMWEAVTLKTEVIGVTSLMN	120
AEC14996.1	SISISDTFESDSPNKDMLPCYSVARIPLPNLNEDLTCGNILMWEAVTLKTEVIGVTSLMN	120
AEC14997.1	SISISDTFESDSPNKDMLPCYSVARIPLPNLNEDLTCGNILMWEAVTLKTEVIGVTSLMN	120
AEC14998.1	SISISDTFESDSPNKDMLPCYSVARIPLPNLNEDLTCGNILMWEAVTLKTEVIGVTSLMN	120
AEC15002.1	SISISDTFESDSPNKDMLPCYSVARIPLPNLNEDLTCGNILMWEAVTLKTEVIGVTSLMN	120
AEC15003.1	SISISDTFESDSPNKDMLPCYSVARIPLPNLNEDLTCGNILMWEAVTLKTEVIGVTSLMN	120
AEC15004.1	SISISDTFESDSPNKDMLPCYSVARIPLPNLNEDLTCGNILMWEAVTLKTEVIGVTSLMN	120
AEC15005.1	SISISDTFESDSPNKDMLPCYSVARIPLPNLNEDLTCGNILMWEAVTLKTEVIGVTSLMN	120
AEC15006.1	SISISDTFESDSPNKDMLPCYSVARIPLPNLNEDLTCGNILMWEAVTLKTEVIGVTSLMN	120
AEC15009.1	SISISDTFESDSPNKDMLPCYSVARIPLPNLNEDLTCGNILMWEAVTLKTEVIGVTSLMN	120
AEC15012.1	SISISDTFESDSPNKDMLPCYSVARIPLPNLNEDLTCGNILMWEAVTLKTEVIGVTSLMN	120
AEC15013.1	SISISDTFESDSPNKDMLPCYSVARIPLPNLNEDLTCGNILMWEAVTLKTEVIGVTSLMN	120
AEC15015.1	SISISDTFESDSPNKDMLPCYSVARIPLPNLNEDLTCGNILMWEAVTLKTEVIGVTSLMN	120
AEC15017.1	SISISDTFESDSPNKDMLPCYSVARIPLPNLNEDLTCGNILMWEAVTLKTEVIGVTSLMN	120
AEC15018.1	SISISDTFESDSPNKDMLPCYSVARIPLPNLNEDLTCGNILMWEAVTLKTEVIGVTSLMN	120
AEC15021.1	SISISDTFESDSPNKDMLPCYSVARIPLPNLNEDLTCGNILMWEAVTLKTEVIGVTSLMN	120
AEC15022.1	SISISDTFESDSPNKDMLPCYSVARIPLPNLNEDLTCGNILMWEAVTLKTEVIGVTSLMN	120
AEC14962.1	SISISDTFESDSPNKDMLPCYSVARIPLPNLNEDLTCGNILMWEAVTLKTEVIGVTSLMN	120
AEC14993.1	SISISDTFESDSPNKDMLPCYSVARIPLPNLNEDLTCGNILMWEAVTLKTEVIGVTSLMN	120
AEC15007.1	SISISDTFESDSPNKDMLPCYSVARIPLPNLNEDLTCGNILMWEAVTLKTEVIGVTSLMN	120
CCW72517.1	SISISDTFESDSPNKDMLPCYSVARIPLPNLNEDLTCGNILMWEAVTLKTEVIGVTSLMN	120
BAD21247.2	SISISDTFESDSPNKDMLPCYSVARIPLPNLNEDLTCGNILMWEAVTLKTEVIGVTSLMN	120
AAL37695.1	SISISDTFESDSPYKDMLPCYSVARIPLPNLNEDLTCGNILMWEAVTLKTEVIGVTALMN	120
AEC15242.1	SISISDTFESDSPNKDMLPCYSVARIPLPNLNEDLTCGNILMWEAVTLKTEVIGVTSLMN	120
AEC15243.1	SISISDTFESDSPNKDMLPCYSVARIPLPNLNEDLTCGNILMWEAVTLKTEVIGVTSLMN	120
AEC15244.1	SISISDTFESDSPNKDMLPCYSVARIPLPNLNEDLTCGNILMWEAVTLKTEVIGVTSLMN	120
AEC15245.1	SISISDTFESDSPNKDMLPCYSVARIPLPNLNEDLTCGNILMWEAVTLKTEVIGVTSLMN	120
AEC15246.1	SISISDTFESDSPNKDMLPCYSVARIPLPNLNEDLTCGNILMWEAVTLKTEVIGVTSLMN	120
AEC15247.1	SISISDTFESDSPNKDMLPCYSVARIPLPNLNEDLTCGNILMWEAVTLKTEVIGVTSLMN	120
AEC15248.1	SISISDTFESDSPNKDMLPCYSVARIPLPNLNEDLTCGNILMWEAVTLKTEVIGVTSLMN	120
AEC15249.1	SISISDTFESDSPNKDMLPCYSVARIPLPNLNEDLTCGNILMWEAVTLKTEVIGVTSLMN	120

```
AEC15250.1        SISISDTFESDSPNKDMLPCYSVARIPLPNLNEDLTCGNILMWEAVTLKTEVIGVTSLMN  120
AEC15251.1        SISISDTFESDSPNKDMLPCYSVARIPLPNLNEDLTCGNILMWEAVTLKTEVIGVTSLMN  120
AEC15252.1        SISISDTFESDSPNKDMLPCYSVARIPLPNLNEDLTCGNILMWEAVTLKTEVIGVTSLMN  120
AEC15253.1        SISISDTFESDSPNKDMLPCYSVARIPLPNLNEDLTCGNILMWEAVTLKTEVIGVTSLMN  120
AEC15254.1        SISISDTFESDSPNKDMLPCYSVARIPLPNLNEDLTCGNILMWEAVTLKTEVIGVTSLMN  120
AEC15255.1        SISISDTFESDSPNKDMLPCYSVARIPLPNLNEDLTCGNILMWEAVTLKTEVIGVTSLMN  120
AEC15256.1        SISISDTFESDSPNKDMLPCYSVARIPLPNLNEDLTCGNILMWEAVTLKTEVIGVTSLMN  120
AEC15257.1        SISISDTFESDSPNKDMLPCYSVARIPLPNLNEDLTCGNILMWEAVTLKTEVIGVTSLMN  120
AEC15258.1        SISISDTFESDSPNKDMLPCYSVARIPLPNLNEDLTCGNILMWEAVTLKTEVIGVTSLMN  120
AEC15259.1        SISISDTFESDSPNKDMLPCYSVARIPLPNLNEDLTCGNILMWEAVTLKTEVIGVTSLMN  120
AEC15260.1        SISISDTFESDSPNKDMLPCYSVARIPLPNLNEDLTCGNILMWEAVTLKTEVIGVTSLMN  120
AEC15261.1        SISISDTFESDSPNKDMLPCYSVARIPLPNLNEDLTCGNILMWEAVTLKTEVIGVTSLMN  120
AEC15262.1        SISISDTFESDSPNKDMLPCYSVARIPLPNLNEDLTCGNILMWEAVTLKTEVIGVTSLMN  120
AEC15263.1        SISISDTFESDSPNKDMLPCYSVARIPLPNLNEDLTCGNILMWEAVTLKTEVIGVTSLMN  120
AEC15264.1        SISISDTFESDSPNKDMLPCYSVARIPLPNLNEDLTCGNILMWEAVTLKTEVIGVTSLMN  120
AEC15265.1        SISISDTFESDSPNKDMLPCYSVARIPLPNLNEDLTCGNILMWEAVTLKTEVIGVTSLMN  120
AEC15000.1        SISISDTFESDSPNKDMLPCYSVARIPLPNLNEDLTCGNILMWEAVTLKTEVIGVTSLMN  120
AEC14721.1        SISISDTFESDSPNKDMLPCYSVARIPLPNLNEDLTCGNILMWEAVTLKTEVIGVTSLMN  120
AEC14965.1        SISISDTFESDSPNKDMLPCYSVARIPLPNLNEDLTCGNILMWEAVTLKTEVIGVTSLMN  120
AEC14967.1        SISISDTFESDSPNKDMLPCYSVARIPLPNLNEDLTCGNILMWEAVTLKTEVIGVTSLMN  120
AEC14969.1        SISISDTFESDSPNKDMLPCYSVARIPLPNLNEDLTCGNILMWEAVTLKTEVIGVTSLMN  120
AEC14973.1        SISISDTFESDSPNKDMLPCYSVARIPLPNLNEDLTCGNILMWEAVTLKTEVIGVTSLMN  120
AEC14974.1        SISISDTFESDSPNKDMLPCYSVARIPLPNLNEDLTCGNILMWEAVTLKTEVIGVTSLMN  120
AEC14994.1        SISISDTFESDSPNKDMLPCYSVARIPLPNLNEDLTCGNILMWEAVTLKTEVIGVTSLMN  120
AEC15001.1        SISISDTFESDSPNKDMLPCYSVARIPLPNLNEDLTCGNILMWEAVTLKTEVIGVTSLMN  120
AEC15024.1        SISISDTFESDSPNKDMLPCYSVARIPLPNLNEDLTCGNILMWEAVTLKTEVIGVTSLMN  120
AEC14667.1        SISISDTFESDSPNKDMLPCYSVARIPLPNLNEDLTCGNILMWEAVTLKTEVIGVTSLMN  120
UPN63091.1        SISISDTFESDSPNKDMLPCYSVARIPLPNLNEDLTCGNILMWEAVTLKTEVIGVTSLMN  120
AEC14535.1        SISISDTFESDSPNKDMLPCYSVARIPLPNLNEDLTCGNILMWEAVTLKTEVIGVTSLMN  120
AEC14547.1        SISISDTFESDSPNKDMLPCYSVARIPLPNLNEDLTCGNILMWEAVTLKTEVIGVTSLMN  120
AEC14553.1        SISISDTFESDSPNKDMLPCYSVARIPLPNLNEDLTCGNILMWEAVTLKTEVIGVTSLMN  120
AEC14577.1        SISISDTFESDSPNKDMLPCYSVARIPLPNLNEDLTCGNILMWEAVTLKTEVIGVTSLMN  120
AEC14613.1        SISISDTFESDSPNKDMLPCYSVARIPLPNLNEDLTCGNILMWEAVTLKTEVIGVTSLMN  120
AEC14673.1        SISISDTFESDSPNKDMLPCYSVARIPLPNLNEDLTCGNILMWEAVTLKTEVIGVTSLMN  120
AEC14679.1        SISISDTFESDSPNKDMLPCYSVARIPLPNLNEDLTCGNILMWEAVTLKTEVIGVTSLMN  120
AEC14727.1        SISISDTFESDSPNKDMLPCYSVARIPLPNLNEDLTCGNILMWEAVTLKTEVIGVTSLMN  120
AEC14733.1        SISISDTFESDSPNKDMLPCYSVARIPLPNLNEDLTCGNILMWEAVTLKTEVIGVTSLMN  120
AEC14964.1        SISISDTFESDSPNKDMLPCYSVARIPLPNLNEDLTCGNILMWEAVTLKTEVIGVTSLMN  120
AEC14995.1        SISISDTFESDSPNKDMLPCYSVARIPLPNLNEDLTCGNILMWEAVTLKTEVIGVTSLMN  120
AEC15008.1        SISISDTFESDSPNKDMLPCYSVARIPLPNLNEDLTCGNILMWEAVTLKTEVIGVTSLMN  120
AEC14970.1        SISISDTFESDSPNKDMLPCYSVARIPLPNLNEDLTCGNILMWEAVTLKTEVIGVTSLMN  120
QTJ15162.1        SISXSXTFESDSPNKDMLPCYSVARIPLPNLNEDLTCGNILMWEAVTLKTEVLGVTTLMN  120
UYL83831.1        SISISDTFESDSPNKDMLPCYSVARIPLPNLNEDLTCGNILMWEAVTLKTEVIGVTSLMN  120
ALF06183.1        SISISDTFESDSPXKDMLPCYSVARIPLPNLNEDLTCGNILMWEAVTLKTEVIGVTXLMN  120
QCH41111.1        SISISDTFDSDSPSKDMLPCYSVARIPLPNLNEDLTCGNILMWEAVTLKTEVIGVTTLMN  120
QCH41112.1        SISISDTFDSDSPSKDMLPCYSVARIPLPNLNEDLTCGNILMWEAVTLKTEVIGVTTLMN  120
AEC14749.1        SISISHTFESDSPSKDMLPRFSVARIPLPNLNEDLTCGNILMWEAVTLKTEVIGVTSLMN  120
ALF06202.1        SISISDTFESDSPSKDMLPCYSVARIPLPNLNEDLTCGNILMWEAVTLKTEVIGVTSLMN  120
ALF06186.1        SISISDTFESDSPSKDMLPCYSVARIPLPNLNEDLTCGNILMWEAVTLKTEVIGVTSLMN  120
ALF06208.1        SISISDTFESDSPSKDMLPCYSVARIPLPNLNEDLTCGNILMWEAVTLKIEVIGVTSLMN  120
ALF06197.1        SISISDTFESDSPSKDMLPCYSVARIPLPNLNEDLTRGNILMWEAVTLKTEVIGVTSLMN  120
AEC15037.1        SISISDTFESDSPSKDMLPCYSVARIPLPNLNEDLTCGNILMWEAVTLKTEVIGVTSLMN  120
UPN63109.1        SISISDTFESDSPSKDMLPCYSVARIPLPNLNEDLTCGNILMWEAVTLKTEVIGVTSLMN  120
UPN63107.1        SISISDTFESDSPSKDMLPCYSVARIPLPNLNEDLTCGNILMWEAVTLKTEVIGVTSLMN  120
UPN63108.1        SISISDTFESDSPSKDMLPCYSVARIPLPNLNEDLTCGNILMWEAVTLKTEVIGVTSLMN  120
QBQ04202.1        SISISDTFESDSPNKDMLPCYSVARIPLPNLNEDLTCGNILMWEAVTLKTEVIGVTTLMN  120
QBM11923.1        SISISDTFESDSPNKEMLPCYSVARIPLPNLNEDLTCGNILMWEAVTLKTEVIGVTTLMN  120
AAK70306.1        SISISDTFESDSPNKDMLPCYSVARIPLPNLNEDLTCGNILMWEAVTLKTEVIGVTTLMN  120
AAC59337.1        SISISDTFESDSPNKDMLPCYSVARVPLPNLNEDLTCGNILMWEAVTLKTEVIGVTTLMN  120
AAL37677.1        SISISDTFESDSPNKDMLPCYSVARIPLPNLNEDLTCGNILMWEAVTLKTEVIGVTTLMN  120
AAK70276.1        SISISDTFESDSPNKDMLPCYSVARIPLPNLNEDLTCGNILMWEAVTLKTEVIGVTTLMN  120
BAC22792.1        SISISDTFESDSPNKDMLPCYSVARIPLPNLNEDLTCGNILMWEAVTLKTEVIGVTALMN  120
BAC22798.1        SISISDTFESDSPNKDMLPCYSVARIPLPNLNEDLTCGNILMWEAVTLKTEVIGVTALMN  120
BAC22804.1        SISISDTFESDSPNKDMLPCYSVARIPLPNLNEDLTCGNILMWEAVTLKTEVIGVTALMN  120
```

```
BAC22810.1        SISISDTFESDSPNKDMLPCYSVARIPLPNLNEDLTCGNILMWEAVTLKTEVIGVTALMN  120
BAC22858.1        SISISDTFESDSPNKDMLPCYSVARIPLPNLNEDLTCGNILMWEAVTLKTEVIGVTALMN  120
BAC15701.1        SISISDTFESDSPNKDMLPCYSVARIPLPNLNEDLTCGNILMWEAVTLKTEVIGVTALMN  120
BAB68863.1        SISISDTFESDSPNKDMLPCYSVARIPLPNLNEDLTCGNILMWEAVTLKTEVIGVTALMN  120
BAC66424.1        SISISDTFESDSPNKDMLPCYSVARIPLPNLNEDLTCGNILMWEAVTLKTEVIGVTSLMN  120
BAD21235.2        SISISDTFESDSPNKDMLPCYSVARIPLPNLNEDLTCGNILMWEAVTLKTEVIGVTSLMN  120
QTJ15210.1        SISISDTFESDSPNKDMLPCYSVARIPLPNLNEDLTCGNILMWEAVTLKTEVIGVTSLMN  120
ASV51748.1        SISISDTFESDSPNKDMLPCYSVARIPLPNLNEDLTCGNILMWEAVTLKTEVIGVTSLMN  120
ASV51754.1        SISISDTFESDSPNKDMLPCYSVARIPLPNLNEDLTCGNILMWEAVTLKTEVIGVTSLMN  120
ASV51778.1        SISISDTFESDSPNKDMLPCYSVARIPLPNLNEDLTCGNILMWEAVTLKTEVIGVTSLMN  120
ASV51814.1        SISISDTFESDSPNKDMLPCYSVARIPLPNLNEDLTCGNILMWEAVTLKTEVIGVTSLMN  120
CCW72488.1        SISISDTFESDSPNKDMLPCYSVARIPLPNLNEDLTCGNILMWEAVTLKTEVIGVTSLMN  120
AEC15219.1        SISISDTFESDSPNKDMLPCYSVARIPLPNLNEDLTCGNILMWEAVTLKTEVIGVTSLMN  120
BAD21241.1        SISISDTFESDSPNKDMLPCYSVARIPLPNLNEDLTCGNILMWEAVTLKTEVIGVTSLMN  120
BAD21253.1        SISISDTFESDSPNKDMLPCYSVARIPLPNLNEDLTCGNILMWEAVTLKTEVIGVTSLMN  120
BAB93086.1        SISISDTFESDSPNKDMLPCYSVARIPLPNLNEDLTCGNILMWEAVTLKTEVIGVTSLMN  120
BAB68953.1        SISISDTFESDSPNKDMLPCYSVARIPLPNLNEDLTCGNILMWEAVTLKTEVIGVTSLMN  120
BAD98966.1        SISISDTFESDSPNKDMLPCYSVARIPLPNLNEDLTCGNILMWEAVTLKTEVIGVTSLMN  120
BAD98972.1        SISISDTFESDSPNKDMLPCYSVARIPLPNLNEDLTCGNILMWEAVTLKTEVIGVTSLMN  120
BAD91887.1        SISISDTFESDSPNKDMLPCYSVARIPLPNLNEDLTCGNILMWEAVTLKTEVIGVTSLMN  120
BAD27118.1        SISISDTFESDSPNKDMLPCYSVARIPLPNLNEDLTCGNILMWEAVTLKTEVIGVTSLMN  120
BAD06227.1        SISISDTFESDSPNKDMLPCYSVARIPLPNLNEDLTCGNILMWEAVTLKTEVIGVTSLMN  120
BAC66412.1        SISISDTFESDSPNKDMLPCYSVARIPLPNLNEDLTCGNILMWEAVTLKTEVIGVTSLMN  120
BAC66430.1        SISISDTFESDSPNKDMLPCYSVARIPLPNLNEDLTCGNILMWEAVTLKTEVIGVTSLMN  120
BAC22816.1        SISISDTFESDSPNKDMLPCYSVARIPLPNLNEDLTCGNILMWEAVTLKTEVIGVTSLMN  120
BAB93026.1        SISISDTFESDSPNKDMLPCYSVARIPLPNLNEDLTCGNILMWEAVTLKTEVIGVTSLMN  120
BAB93032.1        SISISDTFESDSPNKDMLPCYSVARIPLPNLNEDLTCGNILMWEAVTLKTEVIGVTSLMN  120
BAB93074.1        SISISDTFESDSPNKDMLPCYSVARIPLPNLNEDLTCGNILMWEAVTLKTEVIGVTSLMN  120
BAB93080.1        SISISDTFESDSPNKDMLPCYSVARIPLPNLNEDLTCGNILMWEAVTLKTEVIGVTSLMN  120
BAB68917.1        SISISDTFESDSPNKDMLPCYSVARIPLPNLNEDLTCGNILMWEAVTLKTEVIGVTSLMN  120
BAB68923.1        SISISDTFESDSPNKDMLPCYSVARIPLPNLNEDLTCGNILMWEAVTLKTEVIGVTSLMN  120
BAB68929.1        SISISDTFESDSPNKDMLPCYSVARIPLPNLNEDLTCGNILMWEAVTLKTEVIGVTSLMN  120
BAB68935.1        SISISDTFESDSPNKDMLPCYSVARIPLPNLNEDLTCGNILMWEAVTLKTEVIGVTSLMN  120
BAB68941.1        SISISDTFESDSPNKDMLPCYSVARIPLPNLNEDLTCGNILMWEAVTLKTEVIGVTSLMN  120
ALF06214.1        SISISDTFESDSPXKDMLPCYSVARIPLPNLNEDLTCGNILMWEAVTLKTEVIGVTXLMN  120
AAL37707.1        SISISDTFESDSPNKDMLPCYSVARIPLPNLNEDLTCGNILMWEAVTLKTEVIGVTALMN  120
BBG22688.1        SISISDTFESDSPNKDMLPCYSVARIPLPNLNEDLTCGNILMWEAVTLKTEVIGVTTLMN  120
BAB11734.1        SISISDTFESDSPNKDMLPCYSVARIPLPNLNEDLTCGNILMWEAVTLKTEVIGVTTLMN  120
BAA05638.1        SISISDTFESDSPNKDMLPCYSVARIPLPNLNEDLTCGNILMWEAVTLKTEVIGVTTLMN  120
BAV69361.1        SISISDTFESDSPNKDMLPCYSVARIPLPNLNEDLTCGNILMWEAVTLKTEVIGVTTLMN  120
BAV69349.1        SISISXTFESDSPNKDMLPCYSVARIPLPNLNEDLTCGNILMWEAVTLKTEVIGVTTLMN  120
BAV69367.1        SISISHTFESDSPNKDMLPCYSVARIPLPNLNEDLTCGNILMWEAVTLKTEVIGVTTLMN  120
BAE00135.1        LISISDTFESDSPNKDMLPCYSVARIPLPNLNEDLTCGNILMWEAVTLKTEVIGVTTLMN  120
BAE00147.1        SISISDTFESDSPNKDMLPCYSVARIPLPNLNEDLTCGNILMWEAVTLKTEVLGVTTLMN  120
BAD11896.1        SISISDTFESDSPNKDMLPCYSVARIPLPNLNEDLTCGNILMWEAVTLKTEVLGVTTLMN  120
BAD06126.1        SISISDTFESDSPNKDMLPCYSVARIPLPNLNEDLTCGNILMWEAVTLKTEVLGVTTLMN  120
BAC81904.1        SISISDTFESDSPNKDMLPCYSVARIPLPNLNEDLTCGNILMWEAVTLKTEVLGVTTLMN  120
BAC81910.1        SISISDTFESDSPNKDMLPCYSVARIPLPNLNEDLTCGNILMWEAVTLKTEVLGVTTLMN  120
BAC81916.1        SISISDTFESDSPNKDMLPCYSVARIPLPNLNEDLTCGNILMWEAVTLKTEVLGVTTLMN  120
BAC81922.1        SISISDTFESDSPNKDMLPCYSVARIPLPNLNEDLTCGNILMWEAVTLKTEVLGVTTLMN  120
BAC81946.1        SISISDTFESDSPNKDMLPCYSVARIPLPNLNEDLTCGNILMWEAVTLKTEVLGVTTLMN  120
BAC81952.1        SISISDTFESDSPNKDMLPCYSVARIPLPNLNEDLTCGNILMWEAVTLKTEVLGVTTLMN  120
BAC81958.1        SISISDTFESDSPNKDMLPCYSVARIPLPNLNEDLTCGNILMWEAVTLKTEVLGVTTLMN  120
AAM89297.1        SISISDTFESDSPNKDMLPCYSVARIPLPNLNEDLTCGNILMWEAVTLKTEVLGVTTLMN  120
AAM89303.1        SISISDTFESDSPNKDMLPCYSVARIPLPNLNEDLTCGNILMWEAVTLKTEVLGVTTLMN  120
AAM89309.1        SISISDTFESDSPNKDMLPCYSVARIPLPNLNEDLTCGNILMWEAVTLKTEVLGVTTLMN  120
BAC66388.1        SISISDTFESDSPNKDMLPCYSVARIPLPNLNEDLTCGNILMWEAVTLKTEVLGVTTLMN  120
BAC66400.1        SISISDTFESDSPNKDMLPCYSVARIPLPNLNEDLTCGNILMWEAVTLKTEVLGVTTLMN  120
BAC66406.1        SISISDTFESDSPNKDMLPCYSVARIPLPNLNEDLTCGNILMWEAVTLKTEVLGVTTLMN  120
BAC22756.1        SISISDTFESDSPNKDMLPCYSVARIPLPNLNEDLTCGNILMWEAVTLKTEVLGVTTLMN  120
BAC22822.1        SISISDTFESDSPNKDMLPCYSVARIPLPNLNEDLTCGNILMWEAVTLKTEVLGVTTLMN  120
BAC22894.1        SISISDTFESDSPNKDMLPCYSVARIPLPNLNEDLTCGNILMWEAVTLKTEVLGVTTLMN  120
BAC22900.1        SISISDTFESDSPNKDMLPCYSVARIPLPNLNEDLTCGNILMWEAVTLKTEVLGVTTLMN  120
BAC15635.1        SISISDTFESDSPNKDMLPCYSVARIPLPNLNEDLTCGNILMWEAVTLKTEVLGVTTLMN  120
```

BAC15653.1	SISISDTFESDSPNKDMLPCYSVARIPLPNLNEDLTCGNILMWEAVTLKTEVLGVTTLMN	120
BAC15665.1	SISISDTFESDSPNKDMLPCYSVARIPLPNLNEDLTCGNILMWEAVTLKTEVLGVTTLMN	120
BAC03065.1	SISISDTFESDSPNKDMLPCYSVARIPLPNLNEDLTCGNILMWEAVTLKTEVLGVTTLMN	120
AAL37689.1	SISISDTFESDSPNKDMLPCYSVARIPLPNLNEDLTCGNILMWEAVTLKTEVLGVTTLMN	120
AAL37701.1	SISISDTFESDSPNKDMLPCYSVARIPLPNLNEDLTCGNILMWEAVTLKTEVLGVTTLMN	120
BAB68803.1	SISISDTFESDSPNKDMLPCYSVARIPLPNLNEDLTCGNILMWEAVTLKTEVLGVTTLMN	120
BAB68965.1	SISISDTFESDSPNKDMLPCYSVARIPLPNLNEDLTCGNILMWEAVTLKTEVLGVTTLMN	120
BAB68971.1	SISISDTFESDSPNKDMLPCYSVARIPLPNLNEDLTCGNILMWEAVTLKTEVLGVTTLMN	120
AAK97964.1	SISISDTFESDSPNKDMLPCYSVARIPLPNLNEDLTCGNILMWEAVTLKTEVLGVTTLMN	120
AAK97970.1	SISISDTFESDSPNKDMLPCYSVARIPLPNLNEDLTCGNILMWEAVTLKTEVLGVTTLMN	120
AAC40841.1	SISISDTFESDSPNKDMLPCYSVARIPLPNLNEDLTCGNILMWEAVTLKTEVLGVTTLMN	120
BAA05636.1	SISISDTFESDSPNKDMLPCYSVARIPLPNLNEDLTCGNILMWEAVTLKTEVLGVTTLMN	120
BAA01970.1	SISISDTFESDSPNKDMLPCYSVARIPLPNLNEDLTCGNILMWEAVTLKTEVLGVTTLMN	120
BAD06144.1	SISISDTFESDSPNKDMLPCYSVARIPLPNLNEDLTCGNILMWEAVTLKTEVIGVTTLMN	120
BAC81852.1	SISISDTFEGDSPNKDMLPCYSVARIPLPNLNEDLTCGNILMWEAVTLKTEVIGVTTLMN	120
BAC81858.1	SISISDTFEGDSPNKDMLPCYSVARIPLPNLNEDLTCGNILMWEAVTLKTEVIGVTTLMN	120
BAC22864.1	SISISDTFEGDSPNKDMLPCYSVARIPLPNLNEDLTCGNILMWEAVTLKTEVIGVTTLMN	120
AAM89315.1	SISISDTFESDSPNKDMLPCYSVARIPLPNLNEDLTCGNILMWEAVTLKTEVIGVTTLMN	120
BAC66382.1	SISISDTFESDSPNKDMLPCYSVARIPLPNLNEDLTCGNILMWEAVTLKTEVIGVTTLMN	120
BAC66394.1	SISISDTFESDSPNKDMLPCYSVARIPLPNLNEDLTCGNILMWEAVTLKTEVIGVTTLMN	120
BAC66418.1	SISISDTFESDSPNKDMLPCYSVARIPLPNLNEDLTCGNILMWEAVTLKTEVIGVTTLMN	120
BAC22828.1	SISISDTFESDSPNKDMLPCYSVARIPLPNLNEDLTCGNILMWEAVTLKTEVIGVTTLMN	120
BAC22846.1	SISISDTFESDSPNKDMLPCYSVARIPLPNLNEDLTCGNILMWEAVTLKTEVIGVTTLMN	120
AAM69590.1	SISISDTFESDSPNKDMLPCYSVARIPLPNLNEDLTCGNILMWEAVTLKTEVIGVTTLMN	120
BAB93014.1	SISISDTFESDSPNKDMLPCYSVARIPLPNLNEDLTCGNILMWEAVTLKTEVIGVTTLMN	120
BAB93020.1	SISISDTFESDSPNKDMLPCYSVARIPLPNLNEDLTCGNILMWEAVTLKTEVIGVTTLMN	120
BAB93092.1	SISISDTFESDSPNKDMLPCYSVARIPLPNLNEDLTCGNILMWEAVTLKTEVIGVTTLMN	120
BAB93098.1	SISISDTFESDSPNKDMLPCYSVARIPLPNLNEDLTCGNILMWEAVTLKTEVIGVTTLMN	120
AAL37665.1	SISISDTFESDSPNKDMLPCYSVARIPLPNLNEDLTCGNILMWEAVTLKTEVIGVTTLMN	120
BAB68773.1	SISISDTFESDSPNKDMLPCYSVARIPLPNLNEDLTCGNILMWEAVTLKTEVIGVTTLMN	120
BAB68779.1	SISISDTFESDSPNKDMLPCYSVARIPLPNLNEDLTCGNILMWEAVTLKTEVIGVTTLMN	120
AAK28460.1	SISISDTFESDSPNKDMLPCYSVARIPLPNLNEDLTCGNILMWEAVTLKTEVIGVTTLMN	120
AAK28466.1	SISISDTFESDSPNKDMLPCYSVARIPLPNLNEDLTCGNILMWEAVTLKTEVIGVTTLMN	120
BAB11716.1	SISISDTFESDSPNKDMLPCYSVARIPLPNLNEDLTCGNILMWEAVTLKTEVIGVTTLMN	120
BAB11722.1	SISISDTFESDSPNKDMLPCYSVARIPLPNLNEDLTCGNILMWEAVTLKTEVIGVTTLMN	120
AAK97976.1	SISISDTFESDSPNKDMLPCYSVARIPLPNLNEDLTCGNILMWEAVTLKTEVIGVTTLMN	120
BAC15641.1	SISISDTFESDSPNKDMLPCYSVARIPLPNLNEDLTCGNILMWEAVTLKTEVIGVTTLMN	120
BAC15647.1	SISISDTFESDSPNKDMLPCYSVARIPLPNLNEDLTCGNILMWEAVTLKTEVIGVTTLMN	120
AAL37647.1	SISTSDTFESDSPNKDMLPCYSVARIPLPNLNEDLTCGNILMWEAVTLKTEVIGVTTLMN	120
BAB68797.1	SISISDTFESDSPNKDMLPCYSVARIPLPNLNEDLTCGNILMWEAVTLKTEVIGVTTLMN	120
AAG37198.1	SISISDTFESDSPNKDMLPCYSVARIPLPNLNEDLTCGNILMWEAVTLKTEVIGVTTLMN	120
ALF06241.1	SISISDTFASDSPNKDMLPCYSVARIPLPNLNEDLTCGNILMWEAVTLKTEVIGVTTLMN	120
AFE56117.1	SISISDTFDSDSPNKDMLPCYSVARIPLPNLNEDLTCGNILMWEAVTLKTEVIGVTTLMN	120
BAF95600.1	SISISDTFDSDSPNKDMLPCYSVARIPLPNLNEDLTCGNILMWEAVTLKTEVIGVTTLMN	120
BAD11836.1	SISISDTFDSDSPNKDMLPCYSVARIPLPNLNEDLTCGNILMWEAVTLKTEVIGVTTLMN	120
BAD11842.1	SISISDTFDSDSPNKDMLPCYSVARIPLPNLNEDLTCGNILMWEAVTLKTEVIGVTTLMN	120
BAD11848.1	SISISDTFDSDSPNKDMLPCYSVARIPLPNLNEDLTCGNILMWEAVTLKTEVIGVTTLMN	120
AAM89327.1	SISISDTFDSDSPNKDMLPCYSVARIPLPNLNEDLTCGNILMWEAVTLKTEVIGVTTLMN	120
AAM89339.1	SISISDTFDSDSPNKDMLPCYSVARIPLPNLNEDLTCGNILMWEAVTLKTEVIGVTTLMN	120
BAC15707.1	SISISDTFDSDSPNKDMLPCYSVARIPLPNLNEDLTCGNILMWEAVTLKTEVIGVTTLMN	120
BAB68833.1	SISISDTFDSDSPNKDMLPCYSVARIPLPNLNEDLTCGNILMWEAVTLKTEVIGVTTLMN	120
BAB68839.1	SISISDTFDSDSPNKDMLPCYSVARIPLPNLNEDLTCGNILMWEAVTLKTEVIGVTTLMN	120
AYM55604.1	SISISDTFESDSPNKDMLPCYSVARIPLPNLNEDLTCGNILMWEAVTLKTEVIGVTTLMN	120
BAV69355.1	SISISDTFESDSPNKDMLPCYSVARIPLPNLNEDLTCGNILMWEAVTLKTEVIGVTTLMN	120
ALF06220.1	SISISDTFESDSPNKDMLPCYSVARIPLPNLNEDLTCGNILMWEAVTLKTEVIGVTTLMN	120
ALF06247.1	SISISDTFESDSPNKDMLPCYSVARIPLPNLNEDLTCGNILMWEAVTLKTEVIGVTTLMN	120
ALF06250.1	SISISDTFESDSPNKDMLPCYSVARIPLPNLNEDLTCGNILMWEAVTLKTEVIGVTTLMN	120
ALF06253.1	SISISDTFESDSPNKDMLPCYSVARIPLPNLNEDLTCGNILMWEAVTLKTEVIGVTTLMN	120
ALF06259.1	SISISDTFESDSPNKDMLPCYSVARIPLPNLNEDLTCGNILMWEAVTLKTEVIGVTTLMN	120
ALF06265.1	SISISDTFESDSPNKDMLPCYSVARIPLPNLNEDLTCGNILMWEAVTLKTEVIGVTTLMN	120
CCW72502.1	SISISDTFESDSPNKDMLPCYSVARIPLPNLNEDLTCGNILMWEAVTLKTEVIGVTTLMN	120
BAF40757.1	SISISDTFESDSPNKDMLPCYSVARIPLPNLNEDLTCGNILMWEAVTLKTEVIGVTTLMN	120
BAF40763.1	SISISDTFESDSPNKDMLPCYSVARIPLPNLNEDLTCGNILMWEAVTLKTEVIGVTTLMN	120
BAF40769.1	SISISDTFESDSPNKDMLPCYSVARIPLPNLNEDLTCGNILMWEAVTLKTEVIGVTTLMN	120

```
BAF40775.1	SISISDTFESDSPNKDMLPCYSVARIPLPNLNEDLTCGNILMWEAVTLKTEVIGVTTLMN	120
BAE94732.1	SISISDTFESDSPNKDMLPCYSVARIPLPNLNEDLTCGNILMWEAVTLKTEVIGVTTLMN	120
BAE94738.1	SISISDTFESDSPNKDMLPCYSVARIPLPNLNEDLTCGNILMWEAVTLKTEVIGVTTLMN	120
BAE00129.1	SISISDTFESDSPNKDMLPCYSVARIPLPNLNEDLTCGNILMWEAVTLKTEVIGVTTLMN	120
BAE00141.1	SISISDTFESDSPNKDMLPCYSVARIPLPNLNEDLTCGNILMWEAVTLKTEVIGVTTLMN	120
BAD11824.1	SISISDTFESDSPNKDMLPCYSVARIPLPNLNEDLTCGNILMWEAVTLKTEVIGVTTLMN	120
BAD11830.1	SISISDTFESDSPNKDMLPCYSVARIPLPNLNEDLTCGNILMWEAVTLKTEVIGVTTLMN	120
BAD11854.1	SISISDTFESDSPNKDMLPCYSVARIPLPNLNEDLTCGNILMWEAVTLKTEVIGVTTLMN	120
BAD11860.1	SISISDTFESDSPNKDMLPCYSVARIPLPNLNEDLTCGNILMWEAVTLKTEVIGVTTLMN	120
BAD11866.1	SISISDTFESDSPNKDMLPCYSVARIPLPNLNEDLTCGNILMWEAVTLKTEVIGVTTLMN	120
BAD11872.1	SISISDTFESDSPNKDMLPCYSVARIPLPNLNEDLTCGNILMWEAVTLKTEVIGVTTLMN	120
BAD11878.1	SISISDTFESDSPNKDMLPCYSVARIPLPNLNEDLTCGNILMWEAVTLKTEVIGVTTLMN	120
BAD11884.1	SISISDTFESDSPNKDMLPCYSVARIPLPNLNEDLTCGNILMWEAVTLKTEVIGVTTLMN	120
BAD11890.1	SISISDTFESDSPNKDMLPCYSVARIPLPNLNEDLTCGNILMWEAVTLKTEVIGVTTLMN	120
BAD11902.1	SISISDTFESDSPNKDMLPCYSVARIPLPNLNEDLTCGNILMWEAVTLKTEVIGVTTLMN	120
BAD06233.1	SISISDTFESDSPNKDMLPCYSVARIPLPNLNEDLTCGNILMWEAVTLKTEVIGVTTLMN	120
BAD06066.1	SISISDTFESDSPNKDMLPCYSVARIPLPNLNEDLTCGNILMWEAVTLKTEVIGVTTLMN	120
BAD06078.1	SISISDTFESDSPNKDMLPCYSVARIPLPNLNEDLTCGNILMWEAVTLKTEVIGVTTLMN	120
BAD06114.1	SISISDTFESDSPNKDMLPCYSVARIPLPNLNEDLTCGNILMWEAVTLKTEVIGVTTLMN	120
BAD06132.1	SISISDTFESDSPNKDMLPCYSVARIPLPNLNEDLTCGNILMWEAVTLKTEVIGVTTLMN	120
BAD06138.1	SISISDTFESDSPNKDMLPCYSVARIPLPNLNEDLTCGNILMWEAVTLKTEVIGVTTLMN	120
BAD06162.1	SISISDTFESDSPNKDMLPCYSVARIPLPNLNEDLTCGNILMWEAVTLKTEVIGVTTLMN	120
BAD06168.1	SISISDTFESDSPNKDMLPCYSVARIPLPNLNEDLTCGNILMWEAVTLKTEVIGVTTLMN	120
BAC82365.1	SISISDTFESDSPNKDMLPCYSVARIPLPNLNEDLTCGNILMWEAVTLKTEVIGVTTLMN	120
BAC81846.1	SISISDTFESDSPNKDMLPCYSVARIPLPNLNEDLTCGNILMWEAVTLKTEVIGVTTLMN	120
BAC81864.1	SISISDTFESDSPNKDMLPCYSVARIPLPNLNEDLTCGNILMWEAVTLKTEVIGVTTLMN	120
BAC81870.1	SISISDTFESDSPNKDMLPCYSVARIPLPNLNEDLTCGNILMWEAVTLKTEVIGVTTLMN	120
BAC81874.1	SISISDTFESDSPNKDMLPCYSVARIPLPNLNEDLTCGNILMWEAVTLKTEVIGVTTLMN	120
BAC81880.1	SISISDTFESDSPNKDMLPCYSVARIPLPNLNEDLTCGNILMWEAVTLKTEVIGVTTLMN	120
BAC81886.1	SISISDTFESDSPNKDMLPCYSVARIPLPNLNEDLTCGNILMWEAVTLKTEVIGVTTLMN	120
BAC81892.1	SISISDTFESDSPNKDMLPCYSVARIPLPNLNEDLTCGNILMWEAVTLKTEVIGVTTLMN	120
BAC81898.1	SISISDTFESDSPNKDMLPCYSVARIPLPNLNEDLTCGNILMWEAVTLKTEVIGVTTLMN	120
BAC81928.1	SISISDTFESDSPNKDMLPCYSVARIPLPNLNEDLTCGNILMWEAVTLKTEVIGVTTLMN	120
BAC81934.1	SISISDTFESDSPNKDMLPCYSVARIPLPNLNEDLTCGNILMWEAVTLKTEVIGVTTLMN	120
BAC81940.1	SISISDTFESDSPNKDMLPCYSVARIPLPNLNEDLTCGNILMWEAVTLKTEVIGVTTLMN	120
BAC81964.1	SISISDTFESDSPNKDMLPCYSVARIPLPNLNEDLTCGNILMWEAVTLKTEVIGVTTLMN	120
AAM89321.1	SISISDTFESDSPNKDMLPCYSVARIPLPNLNEDLTCGNILMWEAVTLKTEVIGVTTLMN	120
AAM89333.1	SISISDTFESDSPNKDMLPCYSVARIPLPNLNEDLTCGNILMWEAVTLKTEVIGVTTLMN	120
AAM89345.1	SISISDTFESDSPNKDMLPCYSVARIPLPNLNEDLTCGNILMWEAVTLKTEVIGVTTLMN	120
BAC66376.1	SISISDTFESDSPNKDMLPCYSVARIPLPNLNEDLTCGNILMWEAVTLKTEVIGVTTLMN	120
BAC22834.1	SISISDTFESDSPNKDMLPCYSVARIPLPNLNEDLTCGNILMWEAVTLKTEVIGVTTLMN	120
BAC22840.1	SISISDTFESDSPNKDMLPCYSVARIPLPNLNEDLTCGNILMWEAVTLKTEVIGVTTLMN	120
BAC22852.1	SISISDTFESDSPNKDMLPCYSVARIPLPNLNEDLTCGNILMWEAVTLKTEVIGVTTLMN	120
BAC22870.1	SISISDTFESDSPNKDMLPCYSVARIPLPNLNEDLTCGNILMWEAVTLKTEVIGVTTLMN	120
BAC22876.1	SISISDTFESDSPNKDMLPCYSVARIPLPNLNEDLTCGNILMWEAVTLKTEVIGVTTLMN	120
BAC22882.1	SISISDTFESDSPNKDMLPCYSVARIPLPNLNEDLTCGNILMWEAVTLKTEVIGVTTLMN	120
BAC22888.1	SISISDTFESDSPNKDMLPCYSVARIPLPNLNEDLTCGNILMWEAVTLKTEVIGVTTLMN	120
BAC15629.1	SISISDTFESDSPNKDMLPCYSVARIPLPNLNEDLTCGNILMWEAVTLKTEVIGVTTLMN	120
BAC15659.1	SISISDTFESDSPNKDMLPCYSVARIPLPNLNEDLTCGNILMWEAVTLKTEVIGVTTLMN	120
BAC15671.1	SISISDTFESDSPNKDMLPCYSVARIPLPNLNEDLTCGNILMWEAVTLKTEVIGVTTLMN	120
BAC15677.1	SISISDTFESDSPNKDMLPCYSVARIPLPNLNEDLTCGNILMWEAVTLKTEVIGVTTLMN	120
BAC15689.1	SISISDTFESDSPNKDMLPCYSVARIPLPNLNEDLTCGNILMWEAVTLKTEVIGVTTLMN	120
BAC15713.1	SISISDTFESDSPNKDMLPCYSVARIPLPNLNEDLTCGNILMWEAVTLKTEVIGVTTLMN	120
BAC15731.1	SISISDTFESDSPNKDMLPCYSVARIPLPNLNEDLTCGNILMWEAVTLKTEVIGVTTLMN	120
BAC15737.1	SISISDTFESDSPNKDMLPCYSVARIPLPNLNEDLTCGNILMWEAVTLKTEVIGVTTLMN	120
BAC15743.1	SISISDTFESDSPNKDMLPCYSVARIPLPNLNEDLTCGNILMWEAVTLKTEVIGVTTLMN	120
AAL37641.1	SISISDTFESDSPNKDMLPCYSVARIPLPNLNEDLTCGNILMWEAVTLKTEVIGVTTLMN	120
AAL37653.1	SISISDTFESDSPNKDMLPCYSVARIPLPNLNEDLTCGNILMWEAVTLKTEVIGVTTLMN	120
AAL37659.1	SISISDTFESDSPNKDMLPCYSVARIPLPNLNEDLTCGNILMWEAVTLKTEVIGVTTLMN	120
AAL37671.1	SISISDTFESDSPNKDMLPCYSVARIPLPNLNEDLTCGNILMWEAVTLKTEVIGVTTLMN	120
AAL37683.1	SISISDTFESDSPNKDMLPCYSVARIPLPNLNEDLTCGNILMWEAVTLKTEVIGVTTLMN	120
BAB68791.1	SISISDTFESDSPNKDMLPCYSVARIPLPNLNEDLTCGNILMWEAVTLKTEVIGVTTLMN	120
BAB68821.1	SISISDTFESDSPNKDMLPCYSVARIPLPNLNEDLTCGNILMWEAVTLKTEVIGVTTLMN	120
BAB68845.1	SISISDTFESDSPNKDMLPCYSVARIPLPNLNEDLTCGNILMWEAVTLKTEVIGVTTLMN	120
```

BAB68851.1	SISISDTFESDSPNKDMLPCYSVARIPLPNLNEDLTCGNILMWEAVTLKTEVIGVTTLMN	120
BAB68857.1	SISISDTFESDSPNKDMLPCYSVARIPLPNLNEDLTCGNILMWEAVTLKTEVIGVTTLMN	120
BAB68869.1	SISISDTFESDSPNKDMLPCYSVARIPLPNLNEDLTCGNILMWEAVTLKTEVIGVTTLMN	120
BAB68875.1	SISISDTFESDSPNKDMLPCYSVARIPLPNLNEDLTCGNILMWEAVTLKTEVIGVTTLMN	120
AAK97910.1	SISISDTFESDSPNKDMLPCYSVARIPLPNLNEDLTCGNILMWEAVTLKTEVIGVTTLMN	120
AAK97916.1	SISISDTFESDSPNKDMLPCYSVARIPLPNLNEDLTCGNILMWEAVTLKTEVIGVTTLMN	120
AAK97922.1	SISISDTFESDSPNKDMLPCYSVARIPLPNLNEDLTCGNILMWEAVTLKTEVIGVTTLMN	120
AAK97928.1	SISISDTFESDSPNKDMLPCYSVARIPLPNLNEDLTCGNILMWEAVTLKTEVIGVTTLMN	120
AAK97934.1	SISISDTFESDSPNKDMLPCYSVARIPLPNLNEDLTCGNILMWEAVTLKTEVIGVTTLMN	120
AAK97940.1	SISISDTFESDSPNKDMLPCYSVARIPLPNLNEDLTCGNILMWEAVTLKTEVIGVTTLMN	120
AAK97946.1	SISISDTFESDSPNKDMLPCYSVARIPLPNLNEDLTCGNILMWEAVTLKTEVIGVTTLMN	120
AAK97952.1	SISISDTFESDSPNKDMLPCYSVARIPLPNLNEDLTCGNILMWEAVTLKTEVIGVTTLMN	120
AAK97958.1	SISISDTFESDSPNKDMLPCYSVARIPLPNLNEDLTCGNILMWEAVTLKTEVIGVTTLMN	120
AAK97982.1	SISISDTFESDSPNKDMLPCYSVARIPLPNLNEDLTCGNILMWEAVTLKTEVIGVTTLMN	120
AAK97988.1	SISISDTFESDSPNKDMLPCYSVARIPLPNLNEDLTCGNILMWEAVTLKTEVIGVTTLMN	120
AAK97994.1	SISISDTFESDSPNKDMLPCYSVARIPLPNLNEDLTCGNILMWEAVTLKTEVIGVTTLMN	120
BAB11698.1	SISISDTFESDSPNKDMLPCYSVARIPLPNLNEDLTCGNILMWEAVTLKTEVIGVTTLMN	120
BAB11704.1	SISISDTFESDSPNKDMLPCYSVARIPLPNLNEDLTCGNILMWEAVTLKTEVIGVTTLMN	120
AAC59478.1	SISISDTFESDSPNKDMLPCYSVARIPLPNLNEDLTCGNILMWEAVTLKTEVIGVTTLMN	120
AAC59484.1	SISISDTFESDSPNKDMLPCYSVARIPLPNLNEDLTCGNILMWEAVTLKTEVIGVTTLMN	120
AAC59490.1	SISISDTFESDSPNKDMLPCYSVARIPLPNLNEDLTCGNILMWEAVTLKTEVIGVTTLMN	120
BAA01963.1	SISISDTFESDSPNKDMLPCYSVARIPLPNLNEDLTCGNILMWEAVTLKTEVIGVTTLMN	120
BAA01964.1	SISISDTFESDSPNKDMLPCYSVARIPLPNLNEDLTCGNILMWEAVTLKTEVIGVTTLMN	120
BAB93068.1	SISISDTFESDSPDKDMLPCYSVARIPLPNLNEDLTCGNILMWEAVTLKTEVIGVTSLMN	120
ASV51856.1	SISISDTFESDSPNKDMLPCYSVARIPLPNLNEDLTCGNILMWEAVTLKTEVIGVTSLMN	120
ANH21097.1	SISISDTFESDSPNKDMLPCYSVARIPLPNLNEDLTCGNILMWEAVTLKTEVIGVTSLMN	120
AEC15167.1	SISISDTFESDSPNKDMLPCYSVARIPLPNLNEDLTCGNILMWEAVTLKTEVIGVTSLMN	120
AEC15168.1	SISISDTFESDSPNKDMLPCYSVARIPLPNLNEDLTCGNILMWEAVTLKTEVIGVTSLMN	120
AEC15169.1	SISISDTFESDSPNKDMLPCYSVARIPLPNLNEDLTCGNILMWEAVTLKTEVIGVTSLMN	120
QTJ15168.1	SISISDTFDSDSPSKDMLPCYSVARIPLPNLNEDLTCGNILMWEAVTLKTEVIGVTTLMN	120
AEC15173.1	SISISDTFESDSPSKDMLPCYSVARIPLPNLNEDLTCGNILMWEAVTLKTEVIGVTTLMN	120
AEC15174.1	SISISDTFESDSPSKDMLPCYSVARIPLPNLNEDLTCGNILMWEAVTLKTEVIGVTTLMN	120
AEC15175.1	SISISDTFESDSPSKDMLPCYSVARIPLPNLNEDLTCGNILMWEAVTLKTEVIGVTTLMN	120
AEC15176.1	SISISDTFESDSPSKDMLPCYSVARIPLPNLNEDLTCGNILMWEAVTLKTEVIGVTTLMN	120
AEC15177.1	SISISDTFESDSPSKDMLPCYSVARIPLPNLNEDLTCGNILMWEAVTLKTEVIGVTTLMN	120
AEC15178.1	SISISDTFESDSPSKDMLPCYSVARIPLPNLNEDLTCGNILMWEAVTLKTEVIGVTTLMN	120
AEC15179.1	SISISDTFESDSPSKDMLPCYSVARIPLPNLNEDLTCGNILMWEAVTLKTEVIGVTTLMN	120
AEC15180.1	SISISDTFESDSPSKDMLPCYSVARIPLPNLNEDLTCGNILMWEAVTLKTEVIGVTTLMN	120
AEC15181.1	SISISDTFESDSPSKDMLPCYSVARIPLPNLNEDLTCGNILMWEAVTLKTEVIGVTTLMN	120
AEC15182.1	SISISDTFESDSPSKDMLPCYSVARIPLPNLNEDLTCGNILMWEAVTLKTEVIGVTTLMN	120
AEC15183.1	SISISDTFESDSPSKDMLPCYSVARIPLPNLNEDLTCGNILMWEAVTLKTEVIGVTTLMN	120
AEC15184.1	SISISDTFESDSPSKDMLPCYSVARIPLPNLNEDLTCGNILMWEAVTLKTEVIGVTTLMN	120
AEC15185.1	SISISDTFESDSPSKDMLPCYSVARIPLPNLNEDLTCGNILMWEAVTLKTEVIGVTTLMN	120
AEC15187.1	SISISDTFESDSPSKDMLPCYSVARIPLPNLNEDLTCGNILMWEAVTLKTEVIGVTTLMN	120
AEC15188.1	SISISDTFESDSPSKDMLPCYSVARIPLPNLNEDLTCGNILMWEAVTLKTEVIGVTTLMN	120
AEC15189.1	SISISDTFESDSPSKDMLPCYSVARIPLPNLNEDLTCGNILMWEAVTLKTEVIGVTTLMN	120
AEC15190.1	SISISDTFESDSPSKDMLPCYSVARIPLPNLNEDLTCGNILMWEAVTLKTEVIGVTTLMN	120
AEC15191.1	SISISDTFESDSPSKDMLPCYSVARIPLPNLNEDLTCGNILMWEAVTLKTEVIGVTTLMN	120
AEC15193.1	SISISDTFESDSPSKDMLPCYSVARIPLPNLNEDLTCGNILMWEAVTLKTEVIGVTTLMN	120
AEC14871.1	SISISDTFESDSPNKDMLPCYSVARIPLPNLNEDLTCGNILMWEAVTLKTEVIGVTTLMN	120
AEC15222.1	PISISDTFESDSPNKDMLPCYSVARIPLPNLNEDLTCGNILMWEAVTLKTEVIGVTTLMN	120
AEC15130.1	PISISDTFESDSPNKDMLPCYSVARIPLPNLNEDLTCGNILMWEAVTLKTEVIGVTTLMN	120
AEC15144.1	PISISDTFESDSPNKDMLPCYSVARIPLPNLNEDLTCGNILMWEAVTLKTEVIGVTTLMN	120
AEC15154.1	PISISDTFESDSPNKDMLPCYSVARIPLPNLNEDLTCGNILMWEAVTLKTEVIGVTTLMN	120
AEC15157.1	PISISDTFESDSPNKDMLPCYSVARIPLPNLNEDLTCGNILMWEAVTLKTEVIGVTTLMN	120
AEC15160.1	PISISDTFESDSPNKDMLPCYSVARIPLPNLNEDLTCGNILMWEAVTLKTEVIGVTTLMN	120
AEC15231.1	PISISDTFESDSPNKDMLPCYSVARIPLPNLNEDLTCGNILMWEAVTLKTEVIGVTTLMN	120
ALF06191.1	SISISDTFESDSPNKDMLPCYSVARIPLPNLNEDLTCGNILMWEAVTLKTEVIGVTSLMN	120
WLD48112.1	SISISDTFESDSPNKDMLPCYSVARIPLPNLNEDLTCGNILMWEAVTLKTEVIGVTTLMN	120
QWT77140.1	SISISDTFESDSPNKDMLPCYSVARIPLPNLNEDLTCGNILMWEAVTLKTEVIGVTTLMN	120
QTJ15174.1	SISISDTFESDSPNKDMLPCYSVARIPLPNLNEDLTCGNILMWEAVTLKTEVIGVTTLMN	120
QTJ15186.1	SISISDTFESDSPNKDMLPCYSVARIPLPNLNEDLTCGNILMWEAVTLKTEVIGVTTLMN	120
QTJ15192.1	SISISDTFESDSPNKDMLPCYSVARIPLPNLNEDLTCGNILMWEAVTLKTEVIGVTTLMN	120
QTJ15198.1	SISISDTFESDSPNKDMLPCYSVARIPLPNLNEDLTCGNILMWEAVTLKTEVIGVTTLMN	120

```
ASV51772.1        SISISDTFESDSPNKDMLPCYSVARIPLPNLNEDLTCGNILMWEAVTLKTEVIGVTTLMN  120
CCW72478.1        SISISDTFESDSPNKDMLPCYSVARIPLPNLNEDLTCGNILMWEAVTLKTEVIGVTTLMN  120
CCW72481.1        SISISDTFESDSPNKDMLPCYSVARIPLPNLNEDLTCGNILMWEAVTLKTEVIGVTTLMN  120
CCW72487.1        SISISDTFESDSPNKDMLPCYSVARIPLPNLNEDLTCGNILMWEAVTLKTEVIGVTTLMN  120
CCW72492.1        SISISDTFESDSPNKDMLPCYSVARIPLPNLNEDLTCGNILMWEAVTLKTEVIGVTTLMN  120
CCW72496.1        SISISDTFESDSPNKDMLPCYSVARIPLPNLNEDLTCGNILMWEAVTLKTEVIGVTTLMN  120
CCW72499.1        SISISDTFESDSPNKDMLPCYSVARIPLPNLNEDLTCGNILMWEAVTLKTEVIGVTTLMN  120
CCW72504.1        SISISDTFESDSPNKDMLPCYSVARIPLPNLNEDLTCGNILMWEAVTLKTEVIGVTTLMN  120
CCW72508.1        SISISDTFESDSPNKDMLPCYSVARIPLPNLNEDLTCGNILMWEAVTLKTEVIGVTTLMN  120
CCW72510.1        SISISDTFESDSPNKDMLPCYSVARIPLPNLNEDLTCGNILMWEAVTLKTEVIGVTTLMN  120
CCW72519.1        SISISDTFESDSPNKDMLPCYSVARIPLPNLNEDLTCGNILMWEAVTLKTEVIGVTTLMN  120
CCW72521.1        SISISDTFESDSPNKDMLPCYSVARIPLPNLNEDLTCGNILMWEAVTLKTEVIGVTTLMN  120
AEC14864.1        SISISDTFESDSPNKDMLPCYSVARIPLPNLNEDLTCGNILMWEAVTLKTEVIGVTTLMN  120
AEC14865.1        SISISDTFESDSPNKDMLPCYSVARIPLPNLNEDLTCGNILMWEAVTLKTEVIGVTTLMN  120
AEC14866.1        SISISDTFESDSPNKDMLPCYSVARIPLPNLNEDLTCGNILMWEAVTLKTEVIGVTTLMN  120
AEC14867.1        SISISDTFESDSPNKDMLPCYSVARIPLPNLNEDLTCGNILMWEAVTLKTEVIGVTTLMN  120
AEC14868.1        SISISDTFESDSPNKDMLPCYSVARIPLPNLNEDLTCGNILMWEAVTLKTEVIGVTTLMN  120
AEC14869.1        SISISDTFESDSPNKDMLPCYSVARIPLPNLNEDLTCGNILMWEAVTLKTEVIGVTTLMN  120
AEC14870.1        SISISDTFESDSPNKDMLPCYSVARIPLPNLNEDLTCGNILMWEAVTLKTEVIGVTTLMN  120
AEC14872.1        SISISDTFESDSPNKDMLPCYSVARIPLPNLNEDLTCGNILMWEAVTLKTEVIGVTTLMN  120
AEC14873.1        SISISDTFESDSPNKDMLPCYSVARIPLPNLNEDLTCGNILMWEAVTLKTEVIGVTTLMN  120
AEC14874.1        SISISDTFESDSPNKDMLPCYSVARIPLPNLNEDLTCGNILMWEAVTLKTEVIGVTTLMN  120
AEC14875.1        SISISDTFESDSPNKDMLPCYSVARIPLPNLNEDLTCGNILMWEAVTLKTEVIGVTTLMN  120
AEC14876.1        SISISDTFESDSPNKDMLPCYSVARIPLPNLNEDLTCGNILMWEAVTLKTEVIGVTTLMN  120
AEC14877.1        SISISDTFESDSPNKDMLPCYSVARIPLPNLNEDLTCGNILMWEAVTLKTEVIGVTTLMN  120
AEC14878.1        SISISDTFESDSPNKDMLPCYSVARIPLPNLNEDLTCGNILMWEAVTLKTEVIGVTTLMN  120
AEC14879.1        SISISDTFESDSPNKDMLPCYSVARIPLPNLNEDLTCGNILMWEAVTLKTEVIGVTTLMN  120
AEC14880.1        SISISDTFESDSPNKDMLPCYSVARIPLPNLNEDLTCGNILMWEAVTLKTEVIGVTTLMN  120
AEC14881.1        SISISDTFESDSPNKDMLPCYSVARIPLPNLNEDLTCGNILMWEAVTLKTEVIGVTTLMN  120
AEC14882.1        SISISDTFESDSPNKDMLPCYSVARIPLPNLNEDLTCGNILMWEAVTLKTEVIGVTTLMN  120
AEC14883.1        SISISDTFESDSPNKDMLPCYSVARIPLPNLNEDLTCGNILMWEAVTLKTEVIGVTTLMN  120
AEC14884.1        SISISDTFESDSPNKDMLPCYSVARIPLPNLNEDLTCGNILMWEAVTLKTEVIGVTTLMN  120
AEC14885.1        SISISDTFESDSPNKDMLPCYSVARIPLPNLNEDLTCGNILMWEAVTLKTEVIGVTTLMN  120
AEC14886.1        SISISDTFESDSPNKDMLPCYSVARIPLPNLNEDLTCGNILMWEAVTLKTEVIGVTTLMN  120
AEC14887.1        SISISDTFESDSPNKDMLPCYSVARIPLPNLNEDLTCGNILMWEAVTLKTEVIGVTTLMN  120
AEC14888.1        SISISDTFESDSPNKDMLPCYSVARIPLPNLNEDLTCGNILMWEAVTLKTEVIGVTTLMN  120
AEC14889.1        SISISDTFESDSPNKDMLPCYSVARIPLPNLNEDLTCGNILMWEAVTLKTEVIGVTTLMN  120
AEC14890.1        SISISDTFESDSPNKDMLPCYSVARIPLPNLNEDLTCGNILMWEAVTLKTEVIGVTTLMN  120
AEC14891.1        SISISDTFESDSPNKDMLPCYSVARIPLPNLNEDLTCGNILMWEAVTLKTEVIGVTTLMN  120
AEC14892.1        SISISDTFESDSPNKDMLPCYSVARIPLPNLNEDLTCGNILMWEAVTLKTEVIGVTTLMN  120
AEC14894.1        SISISDTFESDSPNKDMLPCYSVARIPLPNLNEDLTCGNILMWEAVTLKTEVIGVTTLMN  120
AEC14895.1        SISISDTFESDSPNKDMLPCYSVARIPLPNLNEDLTCGNILMWEAVTLKTEVIGVTTLMN  120
AEC14896.1        SISISDTFESDSPNKDMLPCYSVARIPLPNLNEDLTCGNILMWEAVTLKTEVIGVTTLMN  120
AEC14897.1        SISISDTFESDSPNKDMLPCYSVARIPLPNLNEDLTCGNILMWEAVTLKTEVIGVTTLMN  120
AEC14898.1        SISISDTFESDSPNKDMLPCYSVARIPLPNLNEDLTCGNILMWEAVTLKTEVIGVTTLMN  120
AEC14899.1        SISISDTFESDSPNKDMLPCYSVARIPLPNLNEDLTCGNILMWEAVTLKTEVIGVTTLMN  120
AEC14900.1        SISISDTFESDSPNKDMLPCYSVARIPLPNLNEDLTCGNILMWEAVTLKTEVIGVTTLMN  120
AEC14901.1        SISISDTFESDSPNKDMLPCYSVARIPLPNLNEDLTCGNILMWEAVTLKTEVIGVTTLMN  120
AEC14902.1        SISISDTFESDSPNKDMLPCYSVARIPLPNLNEDLTCGNILMWEAVTLKTEVIGVTTLMN  120
AEC14903.1        SISISDTFESDSPNKDMLPCYSVARIPLPNLNEDLTCGNILMWEAVTLKTEVIGVTTLMN  120
AEC14904.1        SISISDTFESDSPNKDMLPCYSVARIPLPNLNEDLTCGNILMWEAVTLKTEVIGVTTLMN  120
AEC14905.1        SISISDTFESDSPNKDMLPCYSVARIPLPNLNEDLTCGNILMWEAVTLKTEVIGVTTLMN  120
AEC14907.1        SISISDTFESDSPNKDMLPCYSVARIPLPNLNEDLTCGNILMWEAVTLKTEVIGVTTLMN  120
AEC14908.1        SISISDTFESDSPNKDMLPCYSVARIPLPNLNEDLTCGNILMWEAVTLKTEVIGVTTLMN  120
AEC14909.1        SISISDTFESDSPNKDMLPCYSVARIPLPNLNEDLTCGNILMWEAVTLKTEVIGVTTLMN  120
AEC14910.1        SISISDTFESDSPNKDMLPCYSVARIPLPNLNEDLTCGNILMWEAVTLKTEVIGVTTLMN  120
AEC14911.1        SISISDTFESDSPNKDMLPCYSVARIPLPNLNEDLTCGNILMWEAVTLKTEVIGVTTLMN  120
AEC15164.1        SISISDTFESDSPNKDMLPCYSVARIPLPNLNEDLTCGNILMWEAVTLKTEVIGVTTLMN  120
BAF95594.1        SISISDTFESDSPNKDMLPCYSVARIPLPNLNEDLTCGNILMWEAVTLKTEVIGVTTLMN  120
BAF95606.1        SISISDTFESDSPNKDMLPCYSVARIPLPNLNEDLTCGNILMWEAVTLKTEVIGVTTLMN  120
BAF40745.1        SISISDTFESDSPNKDMLPCYSVARIPLPNLNEDLTCGNILMWEAVTLKTEVIGVTTLMN  120
BAF40751.1        SISISDTFESDSPNKDMLPCYSVARIPLPNLNEDLTCGNILMWEAVTLKTEVIGVTTLMN  120
BAF40781.1        SISISDTFESDSPNKDMLPCYSVARIPLPNLNEDLTCGNILMWEAVTLKTEVIGVTTLMN  120
BAF40787.1        SISISDTFESDSPNKDMLPCYSVARIPLPNLNEDLTCGNILMWEAVTLKTEVIGVTTLMN  120
```

```
BAF40793.1        SISISDTFESDSPNKDMLPCYSVARIPLPNLNEDLTCGNILMWEAVTLKTEVIGVTTLMN  120
BAF40799.1        SISISDTFESDSPNKDMLPCYSVARIPLPNLNEDLTCGNILMWEAVTLKTEVIGVTTLMN  120
BAF40811.1        SISISDTFESDSPNKDMLPCYSVARIPLPNLNEDLTCGNILMWEAVTLKTEVIGVTTLMN  120
BAF40817.1        SISISDTFESDSPNKDMLPCYSVARIPLPNLNEDLTCGNILMWEAVTLKTEVIGVTTLMN  120
BAF40823.1        SISISDTFESDSPNKDMLPCYSVARIPLPNLNEDLTCGNILMWEAVTLKTEVIGVTTLMN  120
BAF40829.1        SISISDTFESDSPNKDMLPCYSVARIPLPNLNEDLTCGNILMWEAVTLKTEVIGVTTLMN  120
BAF40835.1        SISISDTFESDSPNKDMLPCYSVARIPLPNLNEDLTCGNILMWEAVTLKTEVIGVTTLMN  120
BAF40841.1        SISISDTFESDSPNKDMLPCYSVARIPLPNLNEDLTCGNILMWEAVTLKTEVIGVTTLMN  120
BAF40847.1        SISISDTFESDSPNKDMLPCYSVARIPLPNLNEDLTCGNILMWEAVTLKTEVIGVTTLMN  120
BAC81834.1        SISISDTFESDSPNKDMLPCYSVARIPLPNLNEDLTCGNILMWEAVTLKTEVIGVTTLMN  120
BAC81840.1        SISISDTFESDSPNKDMLPCYSVARIPLPNLNEDLTCGNILMWEAVTLKTEVIGVTTLMN  120
AAN85461.1        SISISDTFESDSPNKDMLPCYSVARIPLPNLNEDLTCGNILMWEAVTLKTEVIGVTTLMN  120
AAN85467.1        SISISDTFESDSPNKDMLPCYSVARIPLPNLNEDLTCGNILMWEAVTLKTEVIGVTTLMN  120
AAN85473.1        SISISDTFESDSPNKDMLPCYSVARIPLPNLNEDLTCGNILMWEAVTLKTEVIGVTTLMN  120
BAC22762.1        SISISDTFESDSPNKDMLPCYSVARIPLPNLNEDLTCGNILMWEAVTLKTEVIGVTTLMN  120
BAC22768.1        SISISDTFESDSPNKDMLPCYSVARIPLPNLNEDLTCGNILMWEAVTLKTEVIGVTTLMN  120
BAC22774.1        SISISDTFESDSPNKDMLPCYSVARIPLPNLNEDLTCGNILMWEAVTLKTEVIGVTTLMN  120
BAC22780.1        SISISDTFESDSPNKDMLPCYSVARIPLPNLNEDLTCGNILMWEAVTLKTEVIGVTTLMN  120
BAC22786.1        SISISDTFESDSPNKDMLPCYSVARIPLPNLNEDLTCGNILMWEAVTLKTEVIGVTTLMN  120
BAB68809.1        SISISDTFESDSPNKDMLPCYSVARIPLPNLNEDLTCGNILMWEAVTLKTEVIGVTTLMN  120
BAB68815.1        SISISDTFESDSPNKDMLPCYSVARIPLPNLNEDLTCGNILMWEAVTLKTEVIGVTTLMN  120
BAB68827.1        SISISDTFESDSPNKDMLPCYSVARIPLPNLNEDLTCGNILMWEAVTLKTEVIGVTTLMN  120
AAC59496.1        SISISDTFESDSPNKDMLPCYSVARIPLPNLNEDLTCGNILMWEAVTLKTEVIGVTTLMN  120
AAC59502.1        SISISDTFESDSPNKDMLPCYSVARIPLPNLNEDLTCGNILMWEAVTLKTEVIGVTTLMN  120
AAC59508.1        SISISDTFESDSPNKDMLPCYSVARIPLPNLNEDLTCGNILMWEAVTLKTEVIGVTTLMN  120
AAC59514.1        SISISDTFESDSPNKDMLPCYSVARIPLPNLNEDLTCGNILMWEAVTLKTEVIGVTTLMN  120
AAC59520.1        SISISDTFESDSPNKDMLPCYSVARIPLPNLNEDLTCGNILMWEAVTLKTEVIGVTTLMN  120
AAB62680.1        SISISDTFESDSPNKDMLPCYSVARIPLPNLNEDLTCGNILMWEAVTLKTEVIGVTTLMN  120
BAA01961.1        SISISDTFESDSPNKDMLPCYSVARIPLPNLNEDLTCGNILMWEAVTLKTEVIGVTTLMN  120
WLD48082.1        SISISATFESDSPNKDMLPCYSVARIPLPNLNEDLTCGNILMWEAVTLKTEVIGVTTLMN  120
WLD48088.1        SISISATFESDSPNKDMLPCYSVARIPLPNLNEDLTCGNILMWEAVTLKTEVIGVTTLMN  120
CCW72516.1        SISISDTFEGDSPNKDMLPCYSVARIPLPNLNEDLTCGNILMWEAVTLKTEVIGVTTLMN  120
CCW72523.1        SISISDTFESDSPNKDMLPCYSVARIPLPNLNEDLTCGNILMWEAVTLKTEVIGVTTLMN  120
AEC14783.1        SISISDTFESDSPNKDMLPCYSVARIPLPNLNEDLTCGNILMWEAVTLKTEVIGVTTLMN  120
AEC14784.1        SISISDTFESDSPNKDMLPCYSVARIPLPNLNEDLTCGNILMWEAVTLKTEVIGVTTLMN  120
AEC14785.1        SISISDTFESDSPNKDMLPCYSVARIPLPNLNEDLTCGNILMWEAVTLKTEVIGVTTLMN  120
AEC14786.1        SISISDTFESDSPNKDMLPCYSVARIPLPNLNEDLTCGNILMWEAVTLKTEVIGVTTLMN  120
AEC14787.1        SISISDTFESDSPNKDMLPCYSVARIPLPNLNEDLTCGNILMWEAVTLKTEVIGVTTLMN  120
AAB62687.1        SISISDTFESDSPNKDMLPCYSVARIPLPNLNEDLTCGNILMWEAVTLKTEVIGVTTLMN  120
AEC14893.1        SISISDTFESDSPNKDMLPCYSVARIPLPNLNEDLTCGNILMWEAVTLKTEVIGVTTLMN  120
AEC14906.1        SISISDTFESDSPNKDMLPCYSVARIPLPNLNEDLTCGNILMWEAVTLKTEVIGVTTLMN  120
AEC15118.1        PISISDTFESDSPNKDMLPCYSVARIPLPNLNEDLTCGNILMWEAVTLKTEVIGVTTLMN  120
AEC15120.1        PISISDTFESDSPNKDMLPCYSVARIPLPNLNEDLTCGNILMWEAVTLKTEVIGVTTLMN  120
AEC15121.1        PISISDTFESDSPNKDMLPCYSVARIPLPNLNEDLTCGNILMWEAVTLKTEVIGVTTLMN  120
AEC15122.1        PISISDTFESDSPNKDMLPCYSVARIPLPNLNEDLTCGNILMWEAVTLKTEVIGVTTLMN  120
AEC15124.1        PISISDTFESDSPNKDMLPCYSVARIPLPNLNEDLTCGNILMWEAVTLKTEVIGVTTLMN  120
AEC15125.1        PISISDTFESDSPNKDMLPCYSVARIPLPNLNEDLTCGNILMWEAVTLKTEVIGVTTLMN  120
AEC15127.1        PISISDTFESDSPNKDMLPCYSVARIPLPNLNEDLTCGNILMWEAVTLKTEVIGVTTLMN  120
AEC15129.1        PISISDTFESDSPNKDMLPCYSVARIPLPNLNEDLTCGNILMWEAVTLKTEVIGVTTLMN  120
AEC15131.1        PISISDTFESDSPNKDMLPCYSVARIPLPNLNEDLTCGNILMWEAVTLKTEVIGVTTLMN  120
AEC15133.1        PISISDTFESDSPNKDMLPCYSVARIPLPNLNEDLTCGNILMWEAVTLKTEVIGVTTLMN  120
AEC15135.1        PISISDTFESDSPNKDMLPCYSVARIPLPNLNEDLTCGNILMWEAVTLKTEVIGVTTLMN  120
AEC15136.1        PISISDTFESDSPNKDMLPCYSVARIPLPNLNEDLTCGNILMWEAVTLKTEVIGVTTLMN  120
AEC15137.1        PISISDTFESDSPNKDMLPCYSVARIPLPNLNEDLTCGNILMWEAVTLKTEVIGVTTLMN  120
AEC15138.1        PISISDTFESDSPNKDMLPCYSVARIPLPNLNEDLTCGNILMWEAVTLKTEVIGVTTLMN  120
AEC15139.1        PISISDTFESDSPNKDMLPCYSVARIPLPNLNEDLTCGNILMWEAVTLKTEVIGVTTLMN  120
AEC15141.1        PISISDTFESDSPNKDMLPCYSVARIPLPNLNEDLTCGNILMWEAVTLKTEVIGVTTLMN  120
AEC15142.1        PISISDTFESDSPNKDMLPCYSVARIPLPNLNEDLTCGNILMWEAVTLKTEVIGVTTLMN  120
AEC15143.1        PISISDTFESDSPNKDMLPCYSVARIPLPNLNEDLTCGNILMWEAVTLKTEVIGVTTLMN  120
AEC15145.1        PISISDTFESDSPNKDMLPCYSVARIPLPNLNEDLTCGNILMWEAVTLKTEVIGVTTLMN  120
AEC15146.1        PISISDTFESDSPNKDMLPCYSVARIPLPNLNEDLTCGNILMWEAVTLKTEVIGVTTLMN  120
AEC15147.1        PISISDTFESDSPNKDMLPCYSVARIPLPNLNEDLTCGNILMWEAVTLKTEVIGVTTLMN  120
AEC15148.1        PISISDTFESDSPNKDMLPCYSVARIPLPNLNEDLTCGNILMWEAVTLKTEVIGVTTLMN  120
AEC15149.1        PISISDTFESDSPNKDMLPCYSVARIPLPNLNEDLTCGNILMWEAVTLKTEVIGVTTLMN  120
```

AEC15150.1	PISISDTFESDSPNKDMLPCYSVARIPLPNLNEDLTCGNILMWEAVTLKTEVIGVTTLMN	120
AEC15151.1	PISISDTFESDSPNKDMLPCYSVARIPLPNLNEDLTCGNILMWEAVTLKTEVIGVTTLMN	120
AEC15152.1	PISISDTFESDSPNKDMLPCYSVARIPLPNLNEDLTCGNILMWEAVTLKTEVIGVTTLMN	120
AEC15153.1	PISISDTFESDSPNKDMLPCYSVARIPLPNLNEDLTCGNILMWEAVTLKTEVIGVTTLMN	120
AEC15155.1	PISISDTFESDSPNKDMLPCYSVARIPLPNLNEDLTCGNILMWEAVTLKTEVIGVTTLMN	120
AEC15156.1	PISISDTFESDSPNKDMLPCYSVARIPLPNLNEDLTCGNILMWEAVTLKTEVIGVTTLMN	120
AEC15158.1	PISISDTFESDSPNKDMLPCYSVARIPLPNLNEDLTCGNILMWEAVTLKTEVIGVTTLMN	120
AEC15159.1	PISISDTFESDSPNKDMLPCYSVARIPLPNLNEDLTCGNILMWEAVTLKTEVIGVTTLMN	120
AEC15161.1	PISISDTFESDSPNKDMLPCYSVARIPLPNLNEDLTCGNILMWEAVTLKTEVIGVTTLMN	120
AEC15162.1	PISISDTFESDSPNKDMLPCYSVARIPLPNLNEDLTCGNILMWEAVTLKTEVIGVTTLMN	120
AEC15163.1	PISISDTFESDSPNKDMLPCYSVARIPLPNLNEDLTCGNILMWEAVTLKTEVIGVTTLMN	120
AEC15226.1	PISISDTFESDSPNKDMLPCYSVARIPLPNLNEDLTCGNILMWEAVTLKTEVIGVTTLMN	120
AEC15227.1	PISISDTFESDSPNKDMLPCYSVARIPLPNLNEDLTCGNILMWEAVTLKTEVIGVTTLMN	120
AEC15228.1	PISISDTFESDSPNKDMLPCYSVARIPLPNLNEDLTCGNILMWEAVTLKTEVIGVTTLMN	120
AEC15229.1	PISISDTFESDSPNKDMLPCYSVARIPLPNLNEDLTCGNILMWEAVTLKTEVIGVTTLMN	120
AEC15230.1	PISISDTFESDSPNKDMLPCYSVARIPLPNLNEDLTCGNILMWEAVTLKTEVIGVTTLMN	120
AEC15236.1	PISISDTFESDSPNKDMLPCYSVARIPLPNLNEDLTCGNILMWEAVTLKTEVIGVTTLMN	120
AEC15237.1	PISISDTFESDSPNKDMLPCYSVARIPLPNLNEDLTCGNILMWEAVTLKTEVIGVTTLMN	120
AEC15238.1	PISISDTFESDSPNKDMLPCYSVARIPLPNLNEDLTCGNILMWEAVTLKTEVIGVTTLMN	120
AEC15239.1	PISISDTFESDSPNKDMLPCYSVARIPLPNLNEDLTCGNILMWEAVTLKTEVIGVTTLMN	120
AEC15240.1	PISISDTFESDSPNKDMLPCYSVARIPLPNLNEDLTCGNILMWEAVTLKTEVIGVTTLMN	120
AEC15447.1	PISISDTFESDSPNKDMLPCYSVARIPLPNLNEDLTCGNILMWEAVTLKTEVIGVTTLMN	120
AEC15453.1	PISISDTFESDSPNKDMLPCYSVARIPLPNLNEDLTCGNILMWEAVTLKTEVIGVTTLMN	120
AEC15459.1	PISISDTFESDSPNKDMLPCYSVARIPLPNLNEDLTCGNILMWEAVTLKTEVIGVTTLMN	120
AEC15465.1	PISISDTFESDSPNKDMLPCYSVARIPLPNLNEDLTCGNILMWEAVTLKTEVIGVTTLMN	120
AEC15471.1	PISISDTFESDSPNKDMLPCYSVARIPLPNLNEDLTCGNILMWEAVTLKTEVIGVTTLMN	120
AEC15477.1	PISISDTFESDSPNKDMLPCYSVARIPLPNLNEDLTCGNILMWEAVTLKTEVIGVTTLMN	120
AEC15483.1	PISISDTFESDSPNKDMLPCYSVARIPLPNLNEDLTCGNILMWEAVTLKTEVIGVTTLMN	120
AEC15489.1	PISISDTFESDSPNKDMLPCYSVARIPLPNLNEDLTCGNILMWEAVTLKTEVIGVTTLMN	120
AEC15543.1	PISISDTFESDSPNKDMLPCYSVARIPLPNLNEDLTCGNILMWEAVTLKTEVIGVTTLMN	120
AEC15119.1	TISISDTFESDSPNKDMLPCYSVARIPLPNLNEDLTCGNILMWEAVTLKTEVIGVTTLMN	120
AEC15123.1	TISISDTFESDSPNKDMLPCYSVARIPLPNLNEDLTCGNILMWEAVTLKTEVIGVTTLMN	120
AEC15126.1	TISISDTFESDSPNKDMLPCYSVARIPLPNLNEDLTCGNILMWEAVTLKTEVIGVTTLMN	120
AEC15132.1	TISISDTFESDSPNKDMLPCYSVARIPLPNLNEDLTCGNILMWEAVTLKTEVIGVTTLMN	120
AEC15134.1	TISISDTFESDSPNKDMLPCYSVARIPLPNLNEDLTCGNILMWEAVTLKTEVIGVTTLMN	120
AEC15140.1	TISISDTFESDSPNKDMLPCYSVARIPLPNLNEDLTCGNILMWEAVTLKTEVIGVTTLMN	120
AEC15221.1	TISISDTFESDSPNKDMLPCYSVARIPLPNLNEDLTCGNILMWEAVTLKTEVIGVTTLMN	120
AEC15224.1	TISISDTFESDSPNKDMLPCYSVARIPLPNLNEDLTCGNILMWEAVTLKTEVIGVTTLMN	120
AEC15225.1	TISISDTFESDSPNKDMLPCYSVARIPLPNLNEDLTCGNILMWEAVTLKTEVIGVTTLMN	120
AEC15233.1	TISISDTFESDSPNKDMLPCYSVARIPLPNLNEDLTCGNILMWEAVTLKTEVIGVTTLMN	120
AEC15234.1	TISISDTFESDSPNKDMLPCYSVARIPLPNLNEDLTCGNILMWEAVTLKTEVIGVTTLMN	120
AEC15235.1	TISISDTFESDSPNKDMLPCYSVARIPLPNLNEDLTCGNILMWEAVTLKTEVIGVTTLMN	120
AEC15241.1	TISISDTFESDSPNKDMLPCYSVARIPLPNLNEDLTCGNILMWEAVTLKTEVIGVTTLMN	120
AEC15220.1	XISISDTFESDSPNKDMLPCYSVARIPLPNLNEDLTCGNILMWEAVTLKTEVIGVTTLMN	120
AEC15223.1	XISISDTFESDSPNKDMLPCYSVARIPLPNLNEDLTCGNILMWEAVTLKTEVIGVTTLMN	120
AEC15232.1	XISISDTFESDSPNKDMLPCYSVARIPLPNLNEDLTCGNILMWEAVTLKTEVIGVTTLMN	120
AEC15128.1	SISISDTFESDSPNKDMLPCYSVARIPLPNLNEDLTCGNILMWEAVTLKTEVIGVTTLMN	120
AEC15495.1	SISISDTFESDSPNKDMLPCYSVARIPLPNLNEDLTCGNILMWEAVTLKTEVIGVTTLMN	120
AEC15501.1	SISISDTFESDSPNKDMLPCYSVARIPLPNLNEDLTCGNILMWEAVTLKTEVIGVTTLMN	120
AEC15507.1	SISISDTFESDSPNKDMLPCYSVARIPLPNLNEDLTCGNILMWEAVTLKTEVIGVTTLMN	120
AEC15513.1	SISISDTFESDSPNKDMLPCYSVARIPLPNLNEDLTCGNILMWEAVTLKTEVIGVTTLMN	120
AEC15519.1	SISISDTFESDSPNKDMLPCYSVARIPLPNLNEDLTCGNILMWEAVTLKTEVIGVTTLMN	120
AEC15525.1	SISISDTFESDSPNKDMLPCYSVARIPLPNLNEDLTCGNILMWEAVTLKTEVIGVTTLMN	120
AEC15531.1	SISISDTFESDSPNKDMLPCYSVARIPLPNLNEDLTCGNILMWEAVTLKTEVIGVTTLMN	120
AEC15537.1	SISISDTFESDSPNKDMLPCYSVARIPLPNLNEDLTCGNILMWEAVTLKTEVIGVTTLMN	120
BAF40805.1	SISISDTFESDSPNKDMLPCYSVARIPLPNLNEDLTCGNILMWEAVTLKTEVIGVTTLMN	120
AAT09843.1	SISISDTFESDSPNKDMLPCYSVARIPLPNLNEDLTCGNILMWEAVTLKTEVIGVTTLMN	120
BAA01965.1	SISISDTFESDSPNKDMLPCYSVARIPLPNLNEDLTCGNILMWEAVTLKTEVIGVTTLMN	120
AIW00320.1	SISISDTFESDSPSKDMLPCYSVARIPLPNLNEDLTCGNILMWEAVTLKTEVIGVTSLMN	120
ALF06194.1	SISISDTFESDSPSKDMLPCYSVARIPLPNLNEDLTCGNILMWEAVTLKTEVIGVTSLMN	120
ALF06211.1	SISISDTFESDSPSKDMLPCYSVARIPLPNLNEDLTRGNILMWEAVTLKTEVIGVTSLMN	120
AEC14821.1	SISISGTFESDSPSKDMLPCYSVARIPLPNLNEDLTCGNILMWEAVTLKTEVIGVTSLMN	120
AEC14860.1	SISISGTFESDSPSKDMLPCYSVARIPLPNLNEDLTCGNILMWEAVTLKTEVIGVTSLMN	120
AEC14861.1	SISISGTFESDSPSKDMLPCYSVARIPLPNLNEDLTCGNILMWEAVTLKTEVIGVTSLMN	120

AEC14811.1	SISISDTFQSDSPSKDMLPCYSVARIPLPNLNEDLTCGNILMWEAVALKTEVIGVTSLMN	120
AEC14806.1	SISISDTFQSDSPSKDMLPCYSVARIPLPNLNEDLTCGNILMWEAVTLKTEVIGVTSLMN	120
AEC14795.1	SISISDTFQSDSPSKDMLPCYSVARIPLPNLNEDLTCGNILMWEAVTLKTEVIGVTSLMN	120
AEC14765.1	SISISHTFEGDSPSKDMLPCYSVARIPLPNLNEDLTCGNILMWEAVTLKTEVIGVTSLMN	120
AEC14774.1	SISISHTFESDSPSKDMLPCYSVARIPLPNLNEDLTCGNILMWEAVTLKTEVIGVTSLMN	120
AEC14761.1	SISISHTFESDSPSKDMLPCYSVARIPLPNLNEDLTCGNILMWEAVTLKTEVIGVTSLMN	120
AEC14753.1	SISISHTFESDSPSKDMLPCYSVARIPLPNLNEDLTCGNILMWEAVTLKTEVIGVTSLMN	120
AEC14767.1	SISISHTFESDSPSKDMLPCYSVARIPLPNLNEDLTCGNILMWEAVTLKTEVIGVTSLMN	120
AEC14780.1	SISISHTFESDSPSKDMLPCYSVARIPLPNLNEDLTCGNILMWEAVTLKTEVIGVTSLMN	120
AEC14760.1	SISISHTFESDSPSKDMLPCYSVARIPLPNLNEDLTCGNILMWEAVTLKTEVIGVTSLMN	120
AEC14762.1	SISISHTFESDSPSKDMLPCYSVARIPLPNLNEDLTCGNILMWEAVTLKTEVIGVTSLMN	120
AEC14742.1	SISISHTFESDSPSKDMLPCYSVARIPLPNLNEDLTCGNILMWEAVTLKTEVIGVTSLMN	120
AEC14763.1	SISISHTFESDSPSKDMLPCYSVARIPLPNLNEDLTCGNILMWEAVTLKTEVIGVTSLMN	120
AEC14810.1	SISISDTFQSDSPSKDMLPCYSVARIPLPNLNEDLTCGNILMWEAVTLKTEVIGVTSLMN	120
AEC14803.1	SISISDTFQSDSPSKDMLPCYSVARIPLPNLNEDLTCGNILMWEAVTLKTEVIGVTSLMN	120
AEC14802.1	SISISDTFQSDSPSKDVLPCYSVARIPLPNLNEDLTCGNIPMWEAVTLKTEVIGVTSLMN	120
AEC14812.1	SISISDTFQSDSPSKDVLPCYSVARIPLPNLNEDLTCGNIPMWEAVTLKTEVIGVTSLMN	120
AEC14804.1	SISISDTFQSDSPSKDMLPCYSVARIPLPNLNEDLTCGNIPMWEAVTLKTEVIGVTSLMN	120
AEC14797.1	SISISDTFQSDSPSKDMLPCYSVARIPLPNLNEDLTCGNILMWEAVTLKTEVIGVTSLMN	120
AEC14796.1	SISISDTFQSDSPSKDMLPCYSVARIPLPNLNEDLTCGNILMWEAVTLKTEVIGVTSLMN	120
AEC14793.1	SISISDTFQSDPPSKDMLPCYSVARIPLPNLNEDLTCGNILMWEAVTLKTEVIGVTSLMN	120
AEC14791.1	SISISDTFQSDSPSKDTLPCYSVARIPLPNLNEDLTCGNILMWEAVTLKTEVIGVTSLMN	120
AEC14794.1	SISISDTFQSDSPSKDTLPCYSVARIPLPNLNEDLTCGNILMWEAVTLKTEVIGVTSLMN	120
AEC14789.1	SISISDTFQSDSPSKDMLPCYSVARIPLPNLNEDLTCGNILMWEAVTLKTEVIGVTSLMN	120
AEC14743.1	SISISHTFESDSPSKDMLPCYSVARIPLPNLNEDLTCGNILMWEAVTLKTEVIGVTSLMN	120
AEC14745.1	SISISHTFESDSPSKDMLPCYSVARIPLPNLNEDLTCGNILMWEAVTLKTEVIGVTSLMN	120
AEC14750.1	SISISHTFESDSPSKDMLPCYSVARIPLPNLNEDLTCGNILMWEAVTLKTEVIGVTSLMN	120
AEC14754.1	SISISHTFESDSPSKDMLPCYSVARIPLPNLNEDLTCGNILMWEAVTLKTEVIGVTSLMN	120
AEC14751.1	SISISHTFESDSPSKDMLPCYSVARIPLPNLNEDLTCGNILMWEAVTLKTEVIGVTSLMN	120
AEC14776.1	SISISHTFESDSPSKDMLPCYSVARIPLPNLNEDLTCGNILMWEAVTLKTEVIGVTSLMN	120
AEC14755.1	SISISHTFESDSPSKDMLPCYSVARIPLPNLNEDLTCGNILMWEAVTLKTEVIGVTSLMN	120
AEC14759.1	SISISHTFESDSPSKDMLPCYSVARIPLPNLNEDLTCGNILMWEAVTLKTEVIGVTSLMN	120
AEC14766.1	SISISHTFESDSPSKDMLPCYSVARIPLPNLNEDLTCGNILMWEAVTLKTEVIGVTSLMN	120
AEC14781.1	SISISHTFESDSPSKDMLPCYSVARIPLPNLNEDLTCGNILMWEAVTLKTEVIGVTSLMN	120
AEC15186.1	SISISDTFESDSPSKDMLPCYSVARIPLPNLNEDLTCGNILMWEAVTLKTEVIGVTTLMN	120
AEC15192.1	SISISDTFESDSPSKDMLPCYSVARIPLPNLNEDLTCGNILMWEAVTLKTEVIGVTTLMN	120
AEC15194.1	PISISDTFESDSPSKDMLPCYSVARIPLPNLNEDLTCGNILMWEAVTLKTEVIGVTTLMN	120
AEC15195.1	SISISDTFESDSPSKDMLPCYSVARIPLPNLNEDLTCGNILMWEAVTLKTEVIGVTTLMN	120
QWT77104.1	SISISDTFESDSPSKDMLPCYSVARIPLPNLNEDLTCGNILMWEAVTLKTEVIGVTSLMN	120
UPN63088.1	SISISDTFESDSPSKEMLPCYSVARIPLPNLNEDLTCGNILMWEAVTLKTEVIGVTSLMN	120
UPN63104.1	SISISDTFESDSPSKDMLPCYSVARIPLPNLNEDLTCGNILMWEAVTLKTEVIGVTSLMN	120
UPN63112.1	SISISDTFESDSPSKDMLPCYSVARIPLPNLNEDLTCGNILMWEAVTLKTEVIGVTSLMN	120
UPN63113.1	SISISDTFESDSPSKDMLPCYSVARIPLPNLNEDLTCGNILMWEAVTLKTEVIGVTSLMN	120
UPN63114.1	SISISDTFESDSPSKDMLPCYSVARIPLPNLNEDLTCGNILMWEAVTLKTEVIGVTSLMN	120
UPN63115.1	SISISDTFESDSPSKDMLPCYSVARIPLPNLNEDLTCGNILMWEAVTLKTEVIGVTSLMN	120
AEC15172.1	SISISDTFESDSPSKDMLPCYSVARIPLPNLNEDLTCGNILMWEAVTLKTEVIGVTSLMN	120
BAE00117.1	SISISDTFESDSPSKDMLPCYSVARIPLPNLNEDLTCGNILMWEAVTLKTEVIGVTSLMN	120
BAE00123.1	SISISDTFESDSPSKDMLPCYSVARIPLPNLNEDLTCGNILMWEAVTLKTEVIGVTSLMN	120
AAC59331.1	SISVSDTFESDSPSKDMLPCYSVARIPLPNLNEDLTCGNILMWEAVTLKTEVIGVTSLMN	120
WLD48106.1	SISISDTFESDSPSKDMLPCYSVARIPLPNLNEDLTCGNILMWEAVTLKTEVIGVTSLMN	120
UQK62661.1	SISISDTFESDSPSKDMLPCYSVARIPLPNLNEDLTCGNILMWEAVTLKTEVIGVTSLMN	120
QTJ15156.1	SISISDTFESDSPSKDMLPCYSVARIPLPNLNEDLTCGNILMWEAVTLKTEVIGVTSLMN	120
QNC68213.1	SISISDTFESDSPSKDMLPCYSVARIPLPNLNEDLTCGNILMWEAVTLKTEVIGVTSLMN	120
ASV51766.1	SISISDTFESDSPSKDMLPCYSVARIPLPNLNEDLTCGNILMWEAVTLKTEVIGVTSLMN	120
ASV51796.1	SISISDTFESDSPSKDMLPCYSVARIPLPNLNEDLTCGNILMWEAVTLKTEVIGVTSLMN	120
ASV51802.1	SISISDTFESDSPSKDMLPCYSVARIPLPNLNEDLTCGNILMWEAVTLKTEVIGVTSLMN	120
ASV51808.1	SISISDTFESDSPSKDMLPCYSVARIPLPNLNEDLTCGNILMWEAVTLKTEVIGVTSLMN	120
ASV51820.1	SISISDTFESDSPSKDMLPCYSVARIPLPNLNEDLTCGNILMWEAVTLKTEVIGVTSLMN	120
ASV51826.1	SISISDTFESDSPSKDMLPCYSVARIPLPNLNEDLTCGNILMWEAVTLKTEVIGVTSLMN	120
ASV51832.1	SISISDTFESDSPSKDMLPCYSVARIPLPNLNEDLTCGNILMWEAVTLKTEVIGVTSLMN	120
ASV51838.1	SISISDTFESDSPSKDMLPCYSVARIPLPNLNEDLTCGNILMWEAVTLKTEVIGVTSLMN	120
ASV51862.1	SISISDTFESDSPSKDMLPCYSVARIPLPNLNEDLTCGNILMWEAVTLKTEVIGVTSLMN	120
ASV51868.1	SISISDTFESDSPSKDMLPCYSVARIPLPNLNEDLTCGNILMWEAVTLKTEVIGVTSLMN	120
ASV51874.1	SISISDTFESDSPSKDMLPCYSVARIPLPNLNEDLTCGNILMWEAVTLKTEVIGVTSLMN	120

ASV51880.1	SISISDTFESDSPSKDMLPCYSVARIPLPNLNEDLTCGNILMWEAVTLKTEVIGVTSLMN	120
ASV51892.1	SISISDTFESDSPSKDMLPCYSVARIPLPNLNEDLTCGNILMWEAVTLKTEVIGVTSLMN	120
ANH21098.1	SISISDTFESDSPSKDMLPCYSVARIPLPNLNEDLTCGNILMWEAVTLKTEVIGVTSLMN	120
AID67150.1	SISISDTFESDSPSKDMLPCYSVARIPLPNLNEDLTCGNILMWEAVTLKTEVIGVTSLMN	120
AID67156.1	SISISDTFESDSPSKDMLPCYSVARIPLPNLNEDLTCGNILMWEAVTLKTEVIGVTSLMN	120
AID67162.1	SISISDTFESDSPSKDMLPCYSVARIPLPNLNEDLTCGNILMWEAVTLKTEVIGVTSLMN	120
AID67168.1	SISISDTFESDSPSKDMLPCYSVARIPLPNLNEDLTCGNILMWEAVTLKTEVIGVTSLMN	120
CCW72479.1	SISISDTFESDSPSKDMLPCYSVARIPLPNLNEDLTCGNILMWEAVTLKTEVIGVTSLMN	120
CCW72486.1	SISISDTFESDSPSKDMLPCYSVARIPLPNLNEDLTCGNILMWEAVTLKTEVIGVTSLMN	120
CCW72494.1	SISISDTFESDSPSKDMLPCYSVARIPLPNLNEDLTCGNILMWEAVTLKTEVIGVTSLMN	120
CCW72514.1	SISISDTFESDSPSKDMLPCYSVARIPLPNLNEDLTCGNILMWEAVTLKTEVIGVTSLMN	120
CCW72518.1	SISISDTFESDSPSKDMLPCYSVARIPLPNLNEDLTCGNILMWEAVTLKTEVIGVTSLMN	120
CCW72522.1	SISISDTFESDSPSKDMLPCYSVARIPLPNLNEDLTCGNILMWEAVTLKTEVIGVTSLMN	120
AFH57194.1	SISISDTFESDSPSKDMLPCYSVARIPLPNLNEDLTCGNILMWEAVTLKTEVIGVTSLMN	120
AEC15047.1	SISISDTFESDSPSKDMLPCYSVARIPLPNLNEDLTCGNILMWEAVTLKTEVIGVTSLMN	120
AEC15048.1	SISISDTFESDSPSKDMLPCYSVARIPLPNLNEDLTCGNILMWEAVTLKTEVIGVTSLMN	120
AEC15049.1	SISISDTFESDSPSKDMLPCYSVARIPLPNLNEDLTCGNILMWEAVTLKTEVIGVTSLMN	120
AEC15050.1	SISISDTFESDSPSKDMLPCYSVARIPLPNLNEDLTCGNILMWEAVTLKTEVIGVTSLMN	120
AEC15051.1	SISISDTFESDSPSKDMLPCYSVARIPLPNLNEDLTCGNILMWEAVTLKTEVIGVTSLMN	120
AEC15052.1	SISISDTFESDSPSKDMLPCYSVARIPLPNLNEDLTCGNILMWEAVTLKTEVIGVTSLMN	120
AEC15053.1	SISISDTFESDSPSKDMLPCYSVARIPLPNLNEDLTCGNILMWEAVTLKTEVIGVTSLMN	120
AEC15054.1	SISISDTFESDSPSKDMLPCYSVARIPLPNLNEDLTCGNILMWEAVTLKTEVIGVTSLMN	120
AEC15055.1	SISISDTFESDSPSKDMLPCYSVARIPLPNLNEDLTCGNILMWEAVTLKTEVIGVTSLMN	120
AEC15056.1	SISISDTFESDSPSKDMLPCYSVARIPLPNLNEDLTCGNILMWEAVTLKTEVIGVTSLMN	120
AEC15057.1	SISISDTFESDSPSKDMLPCYSVARIPLPNLNEDLTCGNILMWEAVTLKTEVIGVTSLMN	120
AEC15058.1	SISISDTFESDSPSKDMLPCYSVARIPLPNLNEDLTCGNILMWEAVTLKTEVIGVTSLMN	120
AEC15059.1	SISISDTFESDSPSKDMLPCYSVARIPLPNLNEDLTCGNILMWEAVTLKTEVIGVTSLMN	120
AEC15060.1	SISISDTFESDSPSKDMLPCYSVARIPLPNLNEDLTCGNILMWEAVTLKTEVIGVTSLMN	120
AEC15061.1	SISISDTFESDSPSKDMLPCYSVARIPLPNLNEDLTCGNILMWEAVTLKTEVIGVTSLMN	120
AEC15062.1	SISISDTFESDSPSKDMLPCYSVARIPLPNLNEDLTCGNILMWEAVTLKTEVIGVTSLMN	120
AEC15063.1	SISISDTFESDSPSKDMLPCYSVARIPLPNLNEDLTCGNILMWEAVTLKTEVIGVTSLMN	120
AEC15064.1	SISISDTFESDSPSKDMLPCYSVARIPLPNLNEDLTCGNILMWEAVTLKTEVIGVTSLMN	120
AEC15065.1	SISISDTFESDSPSKDMLPCYSVARIPLPNLNEDLTCGNILMWEAVTLKTEVIGVTSLMN	120
AEC15066.1	SISISDTFESDSPSKDMLPCYSVARIPLPNLNEDLTCGNILMWEAVTLKTEVIGVTSLMN	120
AEC15067.1	SISISDTFESDSPSKDMLPCYSVARIPLPNLNEDLTCGNILMWEAVTLKTEVIGVTSLMN	120
AEC15068.1	SISISDTFESDSPSKDMLPCYSVARIPLPNLNEDLTCGNILMWEAVTLKTEVIGVTSLMN	120
AEC15315.1	SISISDTFESDSPSKDMLPCYSVARIPLPNLNEDLTCGNILMWEAVTLKTEVIGVTSLMN	120
AEC15321.1	SISISDTFESDSPSKDMLPCYSVARIPLPNLNEDLTCGNILMWEAVTLKTEVIGVTSLMN	120
BAD21271.1	SISISDTFESDSPSKDMLPCYSVARIPLPNLNEDLTCGNILMWEAVTLKTEVIGVTSLMN	120
BAD21283.2	SISISDTFESDSPSKDMLPCYSVARIPLPNLNEDLTCGNILMWEAVTLKTEVIGVTSLMN	120
BAC15683.1	SISISDTFESDSPSKDMLPCYSVARIPLPNLNEDLTCGNILMWEAVTLKTEVIGVTSLMN	120
BAC15719.1	SISISDTFESDSPSKDMLPCYSVARIPLPNLNEDLTCGNILMWEAVTLKTEVIGVTSLMN	120
BAB93038.1	SISISDTFESDSPSKDMLPCYSVARIPLPNLNEDLTCGNILMWEAVTLKTEVIGVTSLMN	120
BAB93056.1	SISISDTFESDSPSKDMLPCYSVARIPLPNLNEDLTCGNILMWEAVTLKTEVIGVTSLMN	120
BAB93062.1	SISISDTFESDSPSKDMLPCYSVARIPLPNLNEDLTCGNILMWEAVTLKTEVIGVTSLMN	120
BAB68911.1	SISISDTFESDSPSKDMLPCYSVARIPLPNLNEDLTCGNILMWEAVTLKTEVIGVTSLMN	120
BAB68947.1	SISISDTFESDSPSKDMLPCYSVARIPLPNLNEDLTCGNILMWEAVTLKTEVIGVTSLMN	120
AAK70234.1	SISISDTFESDSPSKDMLPCYSVARIPLPNLNEDLTCGNILMWEAVTLKTEVIGVTSLMN	120
AAK70246.1	SISISDTFESDSPSKDMLPCYSVARIPLPNLNEDLTCGNILMWEAVTLKTEVIGVTSLMN	120
BAA01959.1	SISISDTFESDSPSKDMLPCYSVARIPLPNLNEDLTCGNILMWEAVTLKTEVIGVTSLMN	120
BAA01960.1	SISISDTFESDSPSKDMLPCYSVARIPLPNLNEDLTCGNILMWEAVTLKTEVIGVTSLMN	120
QWT77128.1	SISISDTFESDSPSKDMLPCYSVARIPLPNLNEDLTCGNILMWEAVTLKTEVIGVTSLMN	120
UPN63089.1	SISISDTFESDSPSKDMLPCYSVARIPLPNLNEDLTCGNILMWEAVTLKTEVIGVTSLMN	120
UPN63093.1	SISISDTFESDSPSKDMLPCYSVARIPLPNLNEDLTCGNILMWEAVTLKTEVIGVTSLMN	120
UPN63103.1	SISISDTFESDSPSKDMLPCYSVARIPLPNLNEDLTCGNILMWEAVTLKTEVIGVTSLMN	120
UPN63105.1	SISISDTFESDSPSKDMLPCYSVARIPLPNLNEDLTCGNILMWEAVTLKTEVIGVTSLMN	120
UPN63106.1	SISISDTFESDSPSKDMLPCYSVARIPLPNLNEDLTCGNILMWEAVTLKTEVIGVTSLMN	120
UPN63110.1	SISISDTFESDSPSKDMLPCYSVARIPLPNLNEDLTCGNILMWEAVTLKTEVIGVTSLMN	120
UPN63111.1	SISISDTFESDSPSKDMLPCYSVARIPLPNLNEDLTCGNILMWEAVTLKTEVIGVTSLMN	120
AEC15030.1	SISISDTFESDSPSKDMLPCYSVARIPLPNLNEDLTCGNILMWEAVTLKTEVIGVTSLMN	120
AEC15031.1	SISISDTFESDSPSKDMLPCYSVARIPLPNLNEDLTCGNILMWEAVTLKTEVIGVTSLMN	120
AEC15032.1	SISISDTFESDSPSKDMLPCYSVARIPLPNLNEDLTCGNILMWEAVTLKTEVIGVTSLMN	120
AEC15033.1	SISISDTFESDSPSKDMLPCYSVARIPLPNLNEDLTCGNILMWEAVTLKTEVIGVTSLMN	120
AEC15034.1	SISISDTFESDSPSKDMLPCYSVARIPLPNLNEDLTCGNILMWEAVTLKTEVIGVTSLMN	120

AEC15035.1	SISISDTFESDSPSKDMLPCYSVARIPLPNLNEDLTCGNILMWEAVTLKTEVIGVTSLMN	120
AEC15036.1	SISISDTFESDSPSKDMLPCYSVARIPLPNLNEDLTCGNILMWEAVTLKTEVIGVTSLMN	120
AEC15038.1	SISISDTFESDSPSKDMLPCYSVARIPLPNLNEDLTCGNILMWEAVTLKTEVIGVTSLMN	120
AEC15039.1	SISISDTFESDSPSKDMLPCYSVARIPLPNLNEDLTCGNILMWEAVTLKTEVIGVTSLMN	120
AEC15040.1	SISISDTFESDSPSKDMLPCYSVARIPLPNLNEDLTCGNILMWEAVTLKTEVIGVTSLMN	120
AEC15041.1	SISISDTFESDSPSKDMLPCYSVARIPLPNLNEDLTCGNILMWEAVTLKTEVIGVTSLMN	120
AEC15042.1	SISISDTFESDSPSKDMLPCYSVARIPLPNLNEDLTCGNILMWEAVTLKTEVIGVTSLMN	120
AEC15043.1	SISISDTFESDSPSKDMLPCYSVARIPLPNLNEDLTCGNILMWEAVTLKTEVIGVTSLMN	120
AEC15044.1	SISISDTFESDSPSKDMLPCYSVARIPLPNLNEDLTCGNILMWEAVTLKTEVIGVTSLMN	120
AEC15045.1	SISISDTFESDSPSKDMLPCYSVARIPLPNLNEDLTCGNILMWEAVTLKTEVIGVTSLMN	120
AEC15046.1	SISISDTFESDSPSKDMLPCYSVARIPLPNLNEDLTCGNILMWEAVTLKTEVIGVTSLMN	120
UPN63083.1	SISISDTFESDSPSKDMLPCYSVARIPLPNLNEDLTCGNILMWEAVTLKTEVIGVTSLMN	120
BAE00111.1	SISISDTFESDSPSKDMLPCYSVARIPLPNLNEDLTCGNILMWEAVTLKTEVIGVTSLMN	120
BAA01968.1	SISISDTFESDSPSKDMLPCYSVARIPLPNLNEDLTCGNILMWEAVTLKTEVIGVTSLMN	120
CCW72491.1	SISISDTFGSDSPSKDMLPCYSVARIPLPNLNEDLTCGNILMWEAVTLKTEVIGVTSLMN	120
AEC14788.1	SISISDTFQSDSPSKDMLPCYSVARIPLPNLNEDLTCGNILMWEAVTLKTEVIGVTSLMN	120
AEC14790.1	SISISDTFQSDSPSKDMLPCYSVARIPLPNLNEDLTCGNILMWEAVTLKTEVIGVTSLMN	120
AEC14792.1	SISISDTFQSDSPSKDMLPCYSVARIPLPNLNEDLTCGNILMWEAVTLKTEVIGVTSLMN	120
AEC14798.1	SISISDTFQSDSPSKDMLPCYSVARIPLPNLNEDLTCGNILMWEAVTLKTEVIGVTSLMN	120
AEC14799.1	SISISDTFQSDSPSKDMLPCYSVARIPLPNLNEDLTCGNILMWEAVTLKTEVIGVTSLMN	120
AEC14800.1	SISISDTFQSDSPSKDMLPCYSVARIPLPNLNEDLTCGNILMWEAVTLKTEVIGVTSLMN	120
AEC14801.1	SISISDTFQSDSPSKDMLPCYSVARIPLPNLNEDLTCGNILMWEAVTLKTEVIGVTSLMN	120
AEC14805.1	SISISDTFQSDSPSKDMLPCYSVARIPLPNLNEDLTCGNILMWEAVTLKTEVIGVTSLMN	120
AEC14807.1	SISISDTFQSDSPSKDMLPCYSVARIPLPNLNEDLTCGNILMWEAVTLKTEVIGVTSLMN	120
AEC14808.1	SISISDTFQSDSPSKDMLPCYSVARIPLPNLNEDLTCGNILMWEAVTLKTEVIGVTSLMN	120
AEC14809.1	SISISDTFQSDSPSKDMLPCYSVARIPLPNLNEDLTCGNILMWEAVTLKTEVIGVTSLMN	120
AEC14813.1	SISISDTFQSDSPSKDMLPCYSVARIPLPNLNEDLTCGNILMWEAVTLKTEVIGVTSLMN	120
AEC14912.1	SISISDTFQSDSPSKDMLPCYSVARIPLPNLNEDLTCGNILMWEAVTLKTEVIGVTSLMN	120
AEC14913.1	SISISDTFQSDSPSKDMLPCYSVARIPLPNLNEDLTCGNILMWEAVTLKTEVIGVTSLMN	120
AEC14914.1	SISISDTFQSDSPSKDMLPCYSVARIPLPNLNEDLTCGNILMWEAVTLKTEVIGVTSLMN	120
AEC14915.1	SISISDTFQSDSPSKDMLPCYSVARIPLPNLNEDLTCGNILMWEAVTLKTEVIGVTSLMN	120
AEC14916.1	SISISDTFQSDSPSKDMLPCYSVARIPLPNLNEDLTCGNILMWEAVTLKTEVIGVTSLMN	120
AEC14917.1	SISISDTFQSDSPSKDMLPCYSVARIPLPNLNEDLTCGNILMWEAVTLKTEVIGVTSLMN	120
AEC14918.1	SISISDTFQSDSPSKDMLPCYSVARIPLPNLNEDLTCGNILMWEAVTLKTEVIGVTSLMN	120
AEC14919.1	SISISDTFQSDSPSKDMLPCYSVARIPLPNLNEDLTCGNILMWEAVTLKTEVIGVTSLMN	120
AEC14920.1	SISISDTFQSDSPSKDMLPCYSVARIPLPNLNEDLTCGNILMWEAVTLKTEVIGVTSLMN	120
AEC14921.1	SISISDTFQSDSPSKDMLPCYSVARIPLPNLNEDLTCGNILMWEAVTLKTEVIGVTSLMN	120
AEC14922.1	SISISDTFQSDSPSKDMLPCYSVARIPLPNLNEDLTCGNILMWEAVTLKTEVIGVTSLMN	120
AEC14923.1	SISISDTFQSDSPSKDMLPCYSVARIPLPNLNEDLTCGNILMWEAVTLKTEVIGVTSLMN	120
AEC14924.1	SISISDTFQSDSPSKDMLPCYSVARIPLPNLNEDLTCGNILMWEAVTLKTEVIGVTSLMN	120
AEC14925.1	SISISDTFQSDSPSKDMLPCYSVARIPLPNLNEDLTCGNILMWEAVTLKTEVIGVTSLMN	120
AEC14926.1	SISISDTFQSDSPSKDMLPCYSVARIPLPNLNEDLTCGNILMWEAVTLKTEVIGVTSLMN	120
AEC14927.1	SISISDTFQSDSPSKDMLPCYSVARIPLPNLNEDLTCGNILMWEAVTLKTEVIGVTSLMN	120
AEC14928.1	SISISDTFQSDSPSKDMLPCYSVARIPLPNLNEDLTCGNILMWEAVTLKTEVIGVTSLMN	120
AEC14929.1	SISISDTFQSDSPSKDMLPCYSVARIPLPNLNEDLTCGNILMWEAVTLKTEVIGVTSLMN	120
AEC14930.1	SISISDTFQSDSPSKDMLPCYSVARIPLPNLNEDLTCGNILMWEAVTLKTEVIGVTSLMN	120
AEC14931.1	SISISDTFQSDSPSKDMLPCYSVARIPLPNLNEDLTCGNILMWEAVTLKTEVIGVTSLMN	120
AEC14932.1	SISISDTFQSDSPSKDMLPCYSVARIPLPNLNEDLTCGNILMWEAVTLKTEVIGVTSLMN	120
AEC14933.1	SISISDTFQSDSPSKDMLPCYSVARIPLPNLNEDLTCGNILMWEAVTLKTEVIGVTSLMN	120
AEC14934.1	SISISDTFQSDSPSKDMLPCYSVARIPLPNLNEDLTCGNILMWEAVTLKTEVIGVTSLMN	120
AEC14935.1	SISISDTFQSDSPSKDMLPCYSVARIPLPNLNEDLTCGNILMWEAVTLKTEVIGVTSLMN	120
AEC14936.1	SISISDTFQSDSPSKDMLPCYSVARIPLPNLNEDLTCGNILMWEAVTLKTEVIGVTSLMN	120
AEC14937.1	SISISDTFQSDSPSKDMLPCYSVARIPLPNLNEDLTCGNILMWEAVTLKTEVIGVTSLMN	120
AEC14938.1	SISISDTFQSDSPSKDMLPCYSVARIPLPNLNEDLTCGNILMWEAVTLKTEVIGVTSLMN	120
AEC14939.1	SISISDTFQSDSPSKDMLPCYSVARIPLPNLNEDLTCGNILMWEAVTLKTEVIGVTSLMN	120
AEC14940.1	SISISDTFQSDSPSKDMLPCYSVARIPLPNLNEDLTCGNILMWEAVTLKTEVIGVTSLMN	120
AEC14941.1	SISISDTFQSDSPSKDMLPCYSVARIPLPNLNEDLTCGNILMWEAVTLKTEVIGVTSLMN	120
AEC14942.1	SISISDTFQSDSPSKDMLPCYSVARIPLPNLNEDLTCGNILMWEAVTLKTEVIGVTSLMN	120
AEC14943.1	SISISDTFQSDSPSKDMLPCYSVARIPLPNLNEDLTCGNILMWEAVTLKTEVIGVTSLMN	120
AEC14944.1	SISISDTFQSDSPSKDMLPCYSVARIPLPNLNEDLTCGNILMWEAVTLKTEVIGVTSLMN	120
AEC14945.1	SISISDTFQSDSPSKDMLPCYSVARIPLPNLNEDLTCGNILMWEAVTLKTEVIGVTSLMN	120
AEC14946.1	SISISDTFQSDSPSKDMLPCYSVARIPLPNLNEDLTCGNILMWEAVTLKTEVIGVTSLMN	120
AEC14947.1	SISISDTFQSDSPSKDMLPCYSVARIPLPNLNEDLTCGNILMWEAVTLKTEVIGVTSLMN	120
AEC14948.1	SISISDTFQSDSPSKDMLPCYSVARIPLPNLNEDLTCGNILMWEAVTLKTEVIGVTSLMN	120

AEC14949.1	SISISDTFQSDSPSKDMLPCYSVARIPLPNLNEDLTCGNILMWEAVTLKTEVIGVTSLMN	120
AEC14950.1	SISISDTFQSDSPSKDMLPCYSVARIPLPNLNEDLTCGNILMWEAVTLKTEVIGVTSLMN	120
AEC14951.1	SISISDTFQSDSPSKDMLPCYSVARIPLPNLNEDLTCGNILMWEAVTLKTEVIGVTSLMN	120
AEC14952.1	SISISDTFQSDSPSKDMLPCYSVARIPLPNLNEDLTCGNILMWEAVTLKTEVIGVTSLMN	120
AEC14953.1	SISISDTFQSDSPSKDMLPCYSVARIPLPNLNEDLTCGNILMWEAVTLKTEVIGVTSLMN	120
AEC14954.1	SISISDTFQSDSPSKDMLPCYSVARIPLPNLNEDLTCGNILMWEAVTLKTEVIGVTSLMN	120
AEC14955.1	SISISDTFQSDSPSKDMLPCYSVARIPLPNLNEDLTCGNILMWEAVTLKTEVIGVTSLMN	120
AEC14956.1	SISISDTFQSDSPSKDMLPCYSVARIPLPNLNEDLTCGNILMWEAVTLKTEVIGVTSLMN	120
AEC14957.1	SISISDTFQSDSPSKDMLPCYSVARIPLPNLNEDLTCGNILMWEAVTLKTEVIGVTSLMN	120
AEC14958.1	SISISDTFQSDSPSKDMLPCYSVARIPLPNLNEDLTCGNILMWEAVTLKTEVIGVTSLMN	120
AEC14959.1	SISISDTFQSDSPSKDMLPCYSVARIPLPNLNEDLTCGNILMWEAVTLKTEVIGVTSLMN	120
AEC14960.1	SISISDTFQSDSPSKDMLPCYSVARIPLPNLNEDLTCGNILMWEAVTLKTEVIGVTSLMN	120
AEC14961.1	SISISDTFQSDSPSKDMLPCYSVARIPLPNLNEDLTCGNILMWEAVTLKTEVIGVTSLMN	120
QWT77122.1	SISISDTFESDSPSKDMLPCYSVARIPLPNLNEDLTCGNILMWEAVTLKTEVIGVTSLMN	120
UPN63084.1	SISISDTFESDSPSKDMLPCYSVARIPLPNLNEDLTCGNILMWEAVTLKTEVIGVTSLMN	120
UPN63101.1	SISISDTFESDSPSKDMLPCYSVARIPLPNLNEDLTCGNILMWEAVTLKTEVIGVTSLMN	120
ASV51886.1	SISISDTFESDSPSKDMLPCYSVARIPLPNLNEDLTCGNILMWEAVTLKTEVIGVTSLMN	120
CCW72484.1	SISISDTFESDSPSKDMLPCYSVARIPLPNLNEDLTCGNILMWEAVTLKTEVIGVTSLMN	120
ANH21099.1	SISISDTFESDSPSKDMLPCYSVARIPLPNLNEDLTCGNILMWEAVTLKTEVIGVTSLMN	120
AEC15112.1	SISISDTFESDSPSKDMLPCYSVARIPLPNLNEDLTCGNILMWEAVTLKTEVIGVTSLMN	120
AEC15113.1	SISISDTFESDSPSKDMLPCYSVARIPLPNLNEDLTCGNILMWEAVTLKTEVIGVTSLMN	120
AEC15114.1	SISISDTFESDSPSKDMLPCYSVARIPLPNLNEDLTCGNILMWEAVTLKTEVIGVTSLMN	120
AEC15115.1	SISISDTFESDSPSKDMLPCYSVARIPLPNLNEDLTCGNILMWEAVTLKTEVIGVTSLMN	120
AEC15116.1	SISISDTFESDSPSKDMLPCYSVARIPLPNLNEDLTCGNILMWEAVTLKTEVIGVTSLMN	120
AEC15117.1	SISISDTFESDSPSKDMLPCYSVARIPLPNLNEDLTCGNILMWEAVTLKTEVIGVTSLMN	120
AEC15819.1	SISISDTFESDSPSKDMLPCYSVARIPLPNLNEDLTCGNILMWEAVTLKTEVIGVTSLMN	120
AEC15825.1	SISISDTFESDSPSKDMLPCYSVARIPLPNLNEDLTCGNILMWEAVTLKTEVIGVTSLMN	120
AEC15831.1	SISISDTFESDSPSKDMLPCYSVARIPLPNLNEDLTCGNILMWEAVTLKTEVIGVTSLMN	120
AEC15837.1	SISISDTFESDSPSKDMLPCYSVARIPLPNLNEDLTCGNILMWEAVTLKTEVIGVTSLMN	120
AEC15843.1	SISISDTFESDSPSKDMLPCYSVARIPLPNLNEDLTCGNILMWEAVTLKTEVIGVTSLMN	120
AEC15849.1	SISISDTFESDSPSKDMLPCYSVARIPLPNLNEDLTCGNILMWEAVTLKTEVIGVTSLMN	120
AEC15855.1	SISISDTFESDSPSKDMLPCYSVARIPLPNLNEDLTCGNILMWEAVTLKTEVIGVTSLMN	120
CCW72511.1	SISISDXFESDSPSKDMLPCYSVARIPLPNLNEDLTCGNILMWEAVTLKTEVIGVTSLMN	120
CCW72525.1	SISISDTFESDSPSKDMLPCYSVARIPLPNLNEDLTCGNILMWEAVTLKTEVIGVTSLMN	120
AEC14744.1	SISISHTFESDSPSKDMLPCYSVARIPLPNLNEDLTCGNILMWEAVTLKTEVIGVTSLMN	120
AEC14746.1	SISISHTFESDSPSKDMLPCYSVARIPLPNLNEDLTCGNILMWEAVTLKTEVIGVTSLMN	120
AEC14747.1	SISISHTFESDSPSKDMLPCYSVARIPLPNLNEDLTCGNILMWEAVTLKTEVIGVTSLMN	120
AEC14748.1	SISISHTFESDSPSKDMLPCYSVARIPLPNLNEDLTCGNILMWEAVTLKTEVIGVTSLMN	120
AEC14752.1	SISISHTFESDSPSKDMLPCYSVARIPLPNLNEDLTCGNILMWEAVTLKTEVIGVTSLMN	120
AEC14756.1	SISISHTFESDSPSKDMLPCYSVARIPLPNLNEDLTCGNILMWEAVTLKTEVIGVTSLMN	120
AEC14757.1	SISISHTFESDSPSKDMLPCYSVARIPLPNLNEDLTCGNILMWEAVTLKTEVIGVTSLMN	120
AEC14758.1	SISISHTFESDSPSKDMLPCYSVARIPLPNLNEDLTCGNILMWEAVTLKTEVIGVTSLMN	120
AEC14764.1	SISISHTFESDSPSKDMLPCYSVARIPLPNLNEDLTCGNILMWEAVTLKTEVIGVTSLMN	120
AEC14768.1	SISISHTFESDSPSKDMLPCYSVARIPLPNLNEDLTCGNILMWEAVTLKTEVIGVTSLMN	120
AEC14769.1	SISISHTFESDSPSKDMLPCYSVARIPLPNLNEDLTCGNILMWEAVTLKTEVIGVTSLMN	120
AEC14770.1	SISISHTFESDSPSKDMLPCYSVARIPLPNLNEDLTCGNILMWEAVTLKTEVIGVTSLMN	120
AEC14771.1	SISISHTFESDSPSKDMLPCYSVARIPLPNLNEDLTCGNILMWEAVTLKTEVIGVTSLMN	120
AEC14772.1	SISISHTFESDSPSKDMLPCYSVARIPLPNLNEDLTCGNILMWEAVTLKTEVIGVTSLMN	120
AEC14773.1	SISISHTFESDSPSKDMLPCYSVARIPLPNLNEDLTCGNILMWEAVTLKTEVIGVTSLMN	120
AEC14775.1	SISISHTFESDSPSKDMLPCYSVARIPLPNLNEDLTCGNILMWEAVTLKTEVIGVTSLMN	120
AEC14777.1	SISISHTFESDSPSKDMLPCYSVARIPLPNLNEDLTCGNILMWEAVTLKTEVIGVTSLMN	120
AEC14778.1	SISISHTFESDSPSKDMLPCYSVARIPLPNLNEDLTCGNILMWEAVTLKTEVIGVTSLMN	120
AEC14779.1	SISISHTFESDSPSKDMLPCYSVARIPLPNLNEDLTCGNILMWEAVTLKTEVIGVTSLMN	120
AEC14782.1	SISISHTFESDSPSKDMLPCYSVARIPLPNLNEDLTCGNILMWEAVTLKTEVIGVTSLMN	120
AEC14814.1	SISISGTFESDSPSKDMLPCYSVARIPLPNLNEDLTCGNILMWEAVTLKTEVIGVTSLMN	120
AEC14815.1	SISISGTFESDSPSKDMLPCYSVARIPLPNLNEDLTCGNILMWEAVTLKTEVIGVTSLMN	120
AEC14816.1	SISISGTFESDSPSKDMLPCYSVARIPLPNLNEDLTCGNILMWEAVTLKTEVIGVTSLMN	120
AEC14817.1	SISISGTFESDSPSKDMLPCYSVARIPLPNLNEDLTCGNILMWEAVTLKTEVIGVTSLMN	120
AEC14818.1	SISISGTFESDSPSKDMLPCYSVARIPLPNLNEDLTCGNILMWEAVTLKTEVIGVTSLMN	120
AEC14819.1	SISISGTFESDSPSKDMLPCYSVARIPLPNLNEDLTCGNILMWEAVTLKTEVIGVTSLMN	120
AEC14820.1	SISISGTFESDSPSKDMLPCYSVARIPLPNLNEDLTCGNILMWEAVTLKTEVIGVTSLMN	120
AEC14822.1	SISISGTFESDSPSKDMLPCYSVARIPLPNLNEDLTCGNILMWEAVTLKTEVIGVTSLMN	120
AEC14823.1	SISISGTFESDSPSKDMLPCYSVARIPLPNLNEDLTCGNILMWEAVTLKTEVIGVTSLMN	120
AEC14824.1	SISISGTFESDSPSKDMLPCYSVARIPLPNLNEDLTCGNILMWEAVTLKTEVIGVTSLMN	120

```
AEC14825.1        SISISGTFESDSPSKDMLPCYSVARIPLPNLNEDLTCGNILMWEAVTLKTEVIGVTSLMN  120
AEC14826.1        SISISGTFESDSPSKDMLPCYSVARIPLPNLNEDLTCGNILMWEAVTLKTEVIGVTSLMN  120
AEC14827.1        SISISGTFESDSPSKDMLPCYSVARIPLPNLNEDLTCGNILMWEAVTLKTEVIGVTSLMN  120
AEC14828.1        SISISGTFESDSPSKDMLPCYSVARIPLPNLNEDLTCGNILMWEAVTLKTEVIGVTSLMN  120
AEC14829.1        SISISGTFESDSPSKDMLPCYSVARIPLPNLNEDLTCGNILMWEAVTLKTEVIGVTSLMN  120
AEC14830.1        SISISGTFESDSPSKDMLPCYSVARIPLPNLNEDLTCGNILMWEAVTLKTEVIGVTSLMN  120
AEC14831.1        SISISGTFESDSPSKDMLPCYSVARIPLPNLNEDLTCGNILMWEAVTLKTEVIGVTSLMN  120
AEC14832.1        SISISGTFESDSPSKDMLPCYSVARIPLPNLNEDLTCGNILMWEAVTLKTEVIGVTSLMN  120
AEC14833.1        SISISGTFESDSPSKDMLPCYSVARIPLPNLNEDLTCGNILMWEAVTLKTEVIGVTSLMN  120
AEC14834.1        SISISGTFESDSPSKDMLPCYSVARIPLPNLNEDLTCGNILMWEAVTLKTEVIGVTSLMN  120
AEC14835.1        SISISGTFESDSPSKDMLPCYSVARIPLPNLNEDLTCGNILMWEAVTLKTEVIGVTSLMN  120
AEC14836.1        SISISGTFESDSPSKDMLPCYSVARIPLPNLNEDLTCGNILMWEAVTLKTEVIGVTSLMN  120
AEC14837.1        SISISGTFESDSPSKDMLPCYSVARIPLPNLNEDLTCGNILMWEAVTLKTEVIGVTSLMN  120
AEC14838.1        SISISGTFESDSPSKDMLPCYSVARIPLPNLNEDLTCGNILMWEAVTLKTEVIGVTSLMN  120
AEC14839.1        SISISGTFESDSPSKDMLPCYSVARIPLPNLNEDLTCGNILMWEAVTLKTEVIGVTSLMN  120
AEC14840.1        SISISGTFESDSPSKDMLPCYSVARIPLPNLNEDLTCGNILMWEAVTLKTEVIGVTSLMN  120
AEC14841.1        SISISGTFESDSPSKDMLPCYSVARIPLPNLNEDLTCGNILMWEAVTLKTEVIGVTSLMN  120
AEC14842.1        SISISGTFESDSPSKDMLPCYSVARIPLPNLNEDLTCGNILMWEAVTLKTEVIGVTSLMN  120
AEC14843.1        SISISGTFESDSPSKDMLPCYSVARIPLPNLNEDLTCGNILMWEAVTLKTEVIGVTSLMN  120
AEC14844.1        SISISGTFESDSPSKDMLPCYSVARIPLPNLNEDLTCGNILMWEAVTLKTEVIGVTSLMN  120
AEC14845.1        SISISGTFESDSPSKDMLPCYSVARIPLPNLNEDLTCGNILMWEAVTLKTEVIGVTSLMN  120
AEC14846.1        SISISGTFESDSPSKDMLPCYSVARIPLPNLNEDLTCGNILMWEAVTLKTEVIGVTSLMN  120
AEC14847.1        SISISGTFESDSPSKDMLPCYSVARIPLPNLNEDLTCGNILMWEAVTLKTEVIGVTSLMN  120
AEC14848.1        SISISGTFESDSPSKDMLPCYSVARIPLPNLNEDLTCGNILMWEAVTLKTEVIGVTSLMN  120
AEC14849.1        SISISGTFESDSPSKDMLPCYSVARIPLPNLNEDLTCGNILMWEAVTLKTEVIGVTSLMN  120
AEC14850.1        SISISGTFESDSPSKDMLPCYSVARIPLPNLNEDLTCGNILMWEAVTLKTEVIGVTSLMN  120
AEC14851.1        SISISGTFESDSPSKDMLPCYSVARIPLPNLNEDLTCGNILMWEAVTLKTEVIGVTSLMN  120
AEC14852.1        SISISGTFESDSPSKDMLPCYSVARIPLPNLNEDLTCGNILMWEAVTLKTEVIGVTSLMN  120
AEC14853.1        SISISGTFESDSPSKDMLPCYSVARIPLPNLNEDLTCGNILMWEAVTLKTEVIGVTSLMN  120
AEC14854.1        SISISGTFESDSPSKDMLPCYSVARIPLPNLNEDLTCGNILMWEAVTLKTEVIGVTSLMN  120
AEC14855.1        SISISGTFESDSPSKDMLPCYSVARIPLPNLNEDLTCGNILMWEAVTLKTEVIGVTSLMN  120
AEC14856.1        SISISGTFESDSPSKDMLPCYSVARIPLPNLNEDLTCGNILMWEAVTLKTEVIGVTSLMN  120
AEC14857.1        SISISGTFESDSPSKDMLPCYSVARIPLPNLNEDLTCGNILMWEAVTLKTEVIGVTSLMN  120
AEC14858.1        SISISGTFESDSPSKDMLPCYSVARIPLPNLNEDLTCGNILMWEAVTLKTEVIGVTSLMN  120
AEC14859.1        SISISGTFESDSPSKDMLPCYSVARIPLPNLNEDLTCGNILMWEAVTLKTEVIGVTSLMN  120
AEC14862.1        SISISGTFESDSPSKDMLPCYSVARIPLPNLNEDLTCGNILMWEAVTLKTEVIGVTSLMN  120
AEC14863.1        SISISGTFESDSPSKDMLPCYSVARIPLPNLNEDLTCGNILMWEAVTLKTEVIGVTSLMN  120
BAD21289.1        SISISDTFESDSPSKDMLPCYSVARIPLPNLNEDLTCGNILMWEAVTLKTEVIGVTSLMN  120
BAD21295.1        SISISDTFESDSPSKDMLPCYSVARIPLPNLNEDLTCGNILMWEAVTLKTEVIGVTSLMN  120
BAB93044.1        SISISDTFESDSPSKDMLPCYSVARIPLPNLNEDLTCGNILMWEAVTLKTEVIGVTSLMN  120
BAB93104.1        SISISDTFESDSPSKDMLPCYSVARIPLPNLNEDLTCGNILMWEAVTLKTEVIGVTSLMN  120
AAK70252.1        SISISDTFESDSPSKDMLPCYSVARIPLPNLNEDLTCGNILMWEAVTLKTEVIGVTSLMN  120
BAB68959.1        SISISDTFESDSPTKDMLPCYSVARIPLPILNEDLTCGNILMWEAVTLKTEVIGVTSLMN  120
BAD21229.1        SISISDTFESDSPNKDMLPCYSVARIPLPNLNEDLTCGNILMWEAVTLKTEVIGVTSLMN  120
QBX89123.1        SISISDTFESDSPNKDMLPCYSVARIPLPNLNEDLTCGNILMWEAVTLKTEVIGVTTLMN  120
BAE02908.1        SISISDTFESDSPNKDMLPCYSVARIPLPNLNEDLTCGNILMWEAVTLKTEVIGVTTLMN  120
AAR89259.1        SISISDTFESDSPNKDMLPCYSVARIPLPNLNEDLTCGNILMWEAVTLKTEVIGVTTLMN  120
AAR12957.1        SISISDTFESDSPNKDMLPCYSVARIPLPNLNEDLTCGNILMWEAVTLKTEVIGVTTLMN  120
AAR13077.1        SISISDTFESDSPNKDMLPCYSVARIPLPNLNEDLTCGNILMWEAVTLKTEVIGVTTLMN  120
BCU41036.1        SISISDTFDSDSPNKDMLPCYSVARIPLPNLNEDLTCGNILMWEAVTLKTEVIGVTTLMN  120
BAE94726.1        SISISDTFDSDSPNKDMLPCYSVARIPLPNLNEDLTCGNILMWEAVTLKTEVIGVTTLMN  120
AAB94036.1        SISISDTFESDSPNKDMLPCYSVARIPLPNLNEDLTCGNILMWEAVTLKTEVIGVTTLMN  120
AAK28478.1        SISISDTFESDSPNKDMLPCYSVARIPLPNLNEDLTCGNILMWEAVTLKTEVIGVTTLMN  120
BAB93008.1        SISISDTFESDSPNKDMLPCYSVARIPLPNLNEDLTCGNILMWEAVTLKTEVIGVTTLMN  120
AAM69579.1        SISISDTFESDSPNKDMLPCYSVARIPLPNLNEDLTCGNILMWEAVTLKTEVIGVTTLMN  120
AAM69584.1        SISISDTFESDSPNKDMLPCYSVARIPLPNLNEDLTCGNILMWEAVTLKTEVIGVTTLMN  120
AAM69596.1        SISISDTFESDSPNKDMLPCYSVARIPLPNLNEDLTCGNILMWEAVTLKTEVIGVTTLMN  120
AAM69602.1        SISISDTFESDSPNKDMLPCYSVARIPLPNLNEDLTCGNILMWEAVTLKTEVIGVTTLMN  120
AAM69608.1        SISISDTFESDSPNKDMLPCYSVARIPLPNLNEDLTCGNILMWEAVTLKTEVIGVTTLMN  120
AAK70312.1        SISISDTFESDSPNKDMLPCYSVARIPLPNLNEDLTCGNILMWEAVTLKTEVIGVTTLMN  120
WLD48064.1        SISISDTFESDSPNKDMLPCYSVARIPLPNLNEDLTCGNILMWEAVTLKTEVIGVTTLMN  120
WLD48100.1        SISISDTFESDSPNKDMLPCYSVARIPLPNLNEDLTCGNILMWEAVTLKTEVIGVTTLMN  120
UQK62655.1        SISISDTFESDSPNKDMLPCYSVARIPLPNLNEDLTCGNILMWEAVTLKTEVIGVTTLMN  120
QTJ15180.1        SISISDTFESDSPNKDMLPCYSVARIPLPNLNEDLTCGNILMWEAVTLKTEVIGVTTLMN  120
```

QTJ15204.1	SISISDTFESDSPNKDMLPCYSVARIPLPNLNEDLTCGNILMWEAVTLKTEVIGVTTLMN	120
ASV51784.1	SISISDTFESDSPNKDMLPCYSVARIPLPNLNEDLTCGNILMWEAVTLKTEVIGVTTLMN	120
ASV51790.1	SISISDTFESDSPNKDMLPCYSVARIPLPNLNEDLTCGNILMWEAVTLKTEVIGVTTLMN	120
ASV51898.1	SISISDTFESDSPNKDMLPCYSVARIPLPNLNEDLTCGNILMWEAVTLKTEVIGVTTLMN	120
ASV51904.1	SISISDTFESDSPNKDMLPCYSVARIPLPNLNEDLTCGNILMWEAVTLKTEVIGVTTLMN	120
ASV51910.1	SISISDTFESDSPNKDMLPCYSVARIPLPNLNEDLTCGNILMWEAVTLKTEVIGVTTLMN	120
ASV51916.1	SISISDTFESDSPNKDMLPCYSVARIPLPNLNEDLTCGNILMWEAVTLKTEVIGVTTLMN	120
CCW72490.1	SISISDTFESDSPNKDMLPCYSVARIPLPNLNEDLTCGNILMWEAVTLKTEVIGVTTLMN	120
CCW72495.1	SISISDTFESDSPNKDMLPCYSVARIPLPNLNEDLTCGNILMWEAVTLKTEVIGVTTLMN	120
CCW72497.1	SISISDTFESDSPNKDMLPCYSVARIPLPNLNEDLTCGNILMWEAVTLKTEVIGVTTLMN	120
CCW72503.1	SISISDTFESDSPNKDMLPCYSVARIPLPNLNEDLTCGNILMWEAVTLKTEVIGVTTLMN	120
CCW72505.1	SISISDTFESDSPNKDMLPCYSVARIPLPNLNEDLTCGNILMWEAVTLKTEVIGVTTLMN	120
CCW72507.1	SISISDTFESDSPNKDMLPCYSVARIPLPNLNEDLTCGNILMWEAVTLKTEVIGVTTLMN	120
CCW72512.1	SISISDTFESDSPNKDMLPCYSVARIPLPNLNEDLTCGNILMWEAVTLKTEVIGVTTLMN	120
CCW72515.1	SISISDTFESDSPNKDMLPCYSVARIPLPNLNEDLTCGNILMWEAVTLKTEVIGVTTLMN	120
BAB68881.1	SISISDTFESDSPNKDMLPCYSVARIPLPNLNEDLTCGNILMWEAVTLKTEVIGVTTLMN	120
BAB68887.1	SISISDTFESDSPNKDMLPCYSVARIPLPNLNEDLTCGNILMWEAVTLKTEVIGVTTLMN	120
AAK70294.1	SISISDTFESDSPNKDMLPCYSVARIPLPNLNEDLTCGNILMWEAVTLKTEVIGVTTLMN	120
WLD48094.1	SISISDTFESDSPNKDMLPCYSVARIPLPNLNEDLTCGNILMWEAVTLKTEVIGVTTLMN	120
CCW72509.1	SISISDTFESDSPNKDMLPCYSVARIPLPNLNEDLTCGNILMWEAVTLKTEVIGVTTLMN	120
BAB68893.1	SISISDTFESDSPNKDMLPCYSVARIPLPNLNEDLTCGNILMWEAVTLKTEVIGVTTLMN	120
QTJ15216.1	SISISDTFESDSPNKDMLPCYSVARIPLPNLNEDLTCGNILMWEAVTLKTEVIGVTTLMN	120
CCW72493.1	SISISDTFESDSPNKDMLPCYSVARIPLPNLNEDLTCGNILMWEAVTLKTEVIGVTTLMN	120
CCW72500.1	SISISDTFESDSPNKDMLPCYSVARIPLPNLNEDLTCGNILMWEAVTLKTEVIGVTTLMN	120
CCW72506.1	SISISDTFESDSPNKDMLPCYSVARIPLPNLNEDLTCGNILMWEAVTLKTEVIGVTTLMN	120
CCW72520.1	SISISDTFESDSPNKDMLPCYSVARIPLPNLNEDLTCGNILMWEAVTLKTEVIGVTTLMN	120
BAB68899.1	SISISDTFESDSPNKDMLPCYSVARIPLPNLNEDLTCGNILMWEAVTLKTEVIGVTTLMN	120
BAB68905.1	SISISDTFESDSPNKDMLPCYSVARIPLPNLNEDLTCGNILMWEAVTLKTEVIGVTTLMN	120
AAK70300.1	SISISDTFESDSPNKDMLPCYSVARIPLPNLNEDLTCGNILMWEAVTLKTEVIGVTTLMN	120
AYM55579.1	SISISDTFESDSPNKDMLPCYSVARIPLPNLNEDLTCGNILMWEAVTLKTEVLGVTTLMN	120
AYM55580.1	SISISDTFESDSPNKDMLPCYSVARIPLPNLNEDLTCGNILMWEAVTLKTEVLGVTTLMN	120
AYM55582.1	SISISDTFESDSPNKDMLPCYSVARIPLPNLNEDLTCGNILMWEAVTLKTEVLGVTTLMN	120
AYM55583.1	SISISDTFESDSPNKDMLPCYSVARIPLPNLNEDLTCGNILMWEAVTLKTEVLGVTTLMN	120
AYM55584.1	SISISDTFESDSPNKDMLPCYSVARIPLPNLNEDLTCGNILMWKAVTLKTEVLGVTTLMN	120
AYM55590.1	SISISDTFESDSPNKDMLPCYSVARIPLPNLNEDLTCGNILMWEAVTLKTEVLGVTTLMN	120
AYM55596.1	SISISDTFESDSPNKDMLPCYSVARIPLPNLNEDLTCGNILMWEAVTLKTEVLGVTTLMN	120
BAE45366.1	SISISDTFESDSPNKDMLPCYSVARIPLPNLNEDLTCGNILMWEAVTLKTEVLGVTTLMN	120
AAG30857.1	SISISDTFESDSPNKDMLPCYSVARIPLPNLNEDLTCGNILMWEAVTLKTEVLGVTTLMN	120
BAH36774.1	SISISDTFESDSPNKDMLPCYSVARIPLPNLNEDLTCGNILMWEAVTLKTEVLGVTTLMN	120
BAH36780.1	SISISDTFESDSPNKDMLPCYSVARIPLPNLNEDLTCGNILMWEAVTLKTEVLGVTTLMN	120
BAH36822.1	SISISDTFESDSPNKDMLPCYSVARIPLPNLNEDLTCGNILMWEAVTLKTEVLGVTTLMN	120
BAH36834.1	SISISDTFESDSPNKDMLPCYSVARIPLPNLNEDLTCGNILMWEAVTLKTEVLGVTTLMN	120
BAH36840.1	SISISDTFESDSPNKDMLPCYSVARIPLPNLNEDLTCGNILMWEAVTLKTEVLGVTTLMN	120
BAH36846.1	SISISDTFESDSPNKDMLPCYSVARIPLPNLNEDLTCGNILMWEAVTLKTEVLGVTTLMN	120
BAH36816.1	SISISDTFESDSPNKDMLPCYSVARIPLPNLNEDLTCGNILMWEAVTLKTEVLGVTTLMN	120
BAE45444.1	SISISDTFESDSPNKDMLPCYSVARIPLPNLNEDLTCGNILMWEAVTLKTEVLGVTTLMN	120
AAT09819.1	SISISDTFESDSPNKDMLPCYSVARIPLPNLNEDLTCGNILMWEAVTLKTEVLGVTTLMN	120
BAC03029.1	SISISDTFESDSPNKDMLPCYSVARIPLPNLNEDLTCGNILMWEAVTLKTEVLGVTTLMN	120
BAC03095.1	SISISDTFESDSPHKDMLPCYSVARIPLPNLNEDLTCGNILMWEAVTLKTEVLGVTTLMN	120
AAM69560.1	SISISDTFESDSPNKDMLPCYSVARIPLPNLNEDLTCGNILMWEAVTLKTEVLGVTTLMN	120
AAM69566.1	SISISDTFESDSPNKDMLPCYSVARIPLPNLNEDLTCGNILMWEAVTLKTEVLGVTTLMN	120
AAM69572.1	SISISDTFESDSPNKDMLPCYSVARIPLPNLNEDLTCGNILMWEAVTLKTEVLGVTTLMN	120
AAB84311.1	SISISDTFESDSPNKDMLPCYSVARIPLPNLNEDLTCGNILMWEAVTLKTEVLGVTTLMN	120
BAD06150.1	SISISDTFESDSPNKDMLPCYSVARIPLPNLNEDLTCGNILMWEAVTLKTEVIGVTTLMN	120
AYM55581.1	SISISDTFESDSPNKDMLPCYSVARIPLPNLNEDLTCGNILMWEAVTLKTEVLGVTTLMN	120
AYM55588.1	SISISDTFESDSPNKDMLPCYSVARIPLPNLNEDLTCGNILMWEAVTLKTEVLGVTTLMN	120
AYM55589.1	SISISDTFESDSPNKDMLPCYSVARIPLPNLNEDLTCGNILMWEAVTLKTEVLGVTTLMN	120
AYM55591.1	SISISDTFESDSPNKDMLPCYSVARIPLPNLNEDLTCGNILMWEAVTLKTEVLGVTTLMN	120
AYM55592.1	SISISDTFESDSPNKDMLPCYSVARIPLPNLNEDLTCGNILMWEAVTLKTEVLGVTTLMN	120
AYM55593.1	SISISDTFESDSPNKDMLPCYSVARIPLPNLNEDLTCGNILMWEAVTLKTEVLGVTTLMN	120
AYM55595.1	SISISDTFESDSPNKDMLPCYSVARIPLPNLNEDLTCGNILMWEAVTLKTEVLGVTTLMN	120
AYM55597.1	SISISDTFESDSPNKDMLPCYSVARIPLPNLNEDLTCGNILMWEAVTLKTEVLGVTTLMN	120
AYM55598.1	SISISDTFESDSPNKDMLPCYSVARIPLPNLNEDLTCGNILMWEAVTLKTEVLGVTTLMN	120
AYM55599.1	SISISDTFESDSPNKDMLPCYSVARIPLPNLNEDLTCGNILMWEAVTLKTEVLGVTTLMN	120

```
AYM55600.1      SISISDTFESDSPNKDMLPCYSVARIPLPNLNEDLTCGNILMWEAVTLKTEVLGVTTLMN 120
AYM55601.1      SISISDTFESDSPNKDMLPCYSVARIPLPNLNEDLTCGNILMWEAVTLKTEVLGVTTLMN 120
AYM55602.1      SISISDTFESDSPNKDMLPCYSVARIPLPNLNEDLTCGNILMWEAVTLKTEVLGVTTLMN 120
AYM55603.1      SISISDTFESDSPNKDMLPCYSVARIPLPNLNEDLTCGNILMWEAVTLKTEVLGVTTLMN 120
CCW72483.1      SISISDTFESDSPNKDMLPCYSVARIPLPNLNEDLTCGNILMWEAVTLKTEVLGVTTLMN 120
CCW72485.1      SISISDTFESDSPNKDMLPCYSVARIPLPNLNEDLTCGNILMWEAVTLKTEVLGVTTLMN 120
AFR56669.1      SISISDTFESDSPNKDMLPCYSVARIPLPNLNEDLTCGNILMWEAVTLKTEVLGVTTLMN 120
BAH36768.1      SISISDTFESDSPNKDMLPCYSVARIPLPNLNEDLTCGNILMWEAVTLKTEVLGVTTLMN 120
BAH36786.1      SISISDTFESDSPNKDMLPCYSVARIPLPNLNEDLTCGNILMWEAVTLKTEVLGVTTLMN 120
BAH36792.1      SISISDTFESDSPNKDMLPCYSVARIPLPNLNEDLTCGNILMWEAVTLKTEVLGVTTLMN 120
BAH36798.1      SISISDTFESDSPNKDMLPCYSVARIPLPNLNEDLTCGNILMWEAVTLKTEVLGVTTLMN 120
BAH36804.1      SISISDTFESDSPNKDMLPCYSVARIPLPNLNEDLTCGNILMWEAVTLKTEVLGVTTLMN 120
BAH36810.1      SISISDTFESDSPNKDMLPCYSVARIPLPNLNEDLTCGNILMWEAVTLKTEVLGVTTLMN 120
BAH36828.1      SISISDTFESDSPNKDMLPCYSVARIPLPNLNEDLTCGNILMWEAVTLKTEVLGVTTLMN 120
BAH36852.1      SISISDTFESDSPNKDMLPCYSVARIPLPNLNEDLTCGNILMWEAVTLKTEVLGVTTLMN 120
BAH36858.1      SISISDTFESDSPNKDMLPCYSVARIPLPNLNEDLTCGNILMWEAVTLKTEVLGVTTLMN 120
BAE75814.1      SISISDTFESDSPNKDMLPCYSVARIPLPNLNEDLTCGNILMWEAVTLKTEVLGVTTLMN 120
BAE75820.1      SISISDTFESDSPNKDMLPCYSVARIPLPNLNEDLTCGNILMWEAVTLKTEVLGVTTLMN 120
BAE75826.1      SISISDTFESDSPNKDMLPCYSVARIPLPNLNEDLTCGNILMWEAVTLKTEVLGVTTLMN 120
BAE75832.1      SISISDTFESDSPNKDMLPCYSVARIPLPNLNEDLTCGNILMWEAVTLKTEVLGVTTLMN 120
BAE75838.1      SISISDTFESDSPNKDMLPCYSVARIPLPNLNEDLTCGNILMWEAVTLKTEVLGVTTLMN 120
BAE45360.1      SISISDTFESDSPNKDMLPCYSVARIPLPNLNEDLTCGNILMWEAVTLKTEVLGVTTLMN 120
BAE45372.1      SISISDTFESDSPNKDMLPCYSVARIPLPNLNEDLTCGNILMWEAVTLKTEVLGVTTLMN 120
BAE45378.1      SISISDTFESDSPNKDMLPCYSVARIPLPNLNEDLTCGNILMWEAVTLKTEVLGVTTLMN 120
BAE45384.1      SISISDTFESDSPNKDMLPCYSVARIPLPNLNEDLTCGNILMWEAVTLKTEVLGVTTLMN 120
BAE45390.1      SISISDTFESDSPNKDMLPCYSVARIPLPNLNEDLTCGNILMWEAVTLKTEVLGVTTLMN 120
BAE45396.1      SISISDTFESDSPNKDMLPCYSVARIPLPNLNEDLTCGNILMWEAVTLKTEVLGVTTLMN 120
BAE45402.1      SISISDTFESDSPNKDMLPCYSVARIPLPNLNEDLTCGNILMWEAVTLKTEVLGVTTLMN 120
BAE45408.1      SISISDTFESDSPNKDMLPCYSVARIPLPNLNEDLTCGNILMWEAVTLKTEVLGVTTLMN 120
BAE45414.1      SISISDTFESDSPNKDMLPCYSVARIPLPNLNEDLTCGNILMWEAVTLKTEVLGVTTLMN 120
BAE45420.1      SISISDTFESDSPNKDMLPCYSVARIPLPNLNEDLTCGNILMWEAVTLKTEVLGVTTLMN 120
BAE45426.1      SISISDTFESDSPNKDMLPCYSVARIPLPNLNEDLTCGNILMWEAVTLKTEVLGVTTLMN 120
BAE45432.1      SISISDTFESDSPNKDMLPCYSVARIPLPNLNEDLTCGNILMWEAVTLKTEVLGVTTLMN 120
BAE45438.1      SISISDTFESDSPNKDMLPCYSVARIPLPNLNEDLTCGNILMWEAVTLKTEVLGVTTLMN 120
BAD06018.1      SISISDTFESDSPNKDMLPCYSVARIPLPNLNEDLTCGNILMWEAVTLKTEVLGVTTLMN 120
BAD06024.1      SISISDTFESDSPNKDMLPCYSVARIPLPNLNEDLTCGNILMWEAVTLKTEVLGVTTLMN 120
BAD06030.1      SISISDTFESDSPNKDMLPCYSVARIPLPNLNEDLTCGNILMWEAVTLKTEVLGVTTLMN 120
BAD06042.1      SISISDTFESDSPNKDMLPCYSVARIPLPNLNEDLTCGNILMWEAVTLKTEVLGVTTLMN 120
BAD06048.1      SISISDTFESDSPNKDMLPCYSVARIPLPNLNEDLTCGNILMWEAVTLKTEVLGVTTLMN 120
BAD06054.1      SISISDTFESDSPNKDMLPCYSVARIPLPNLNEDLTCGNILMWEAVTLKTEVLGVTTLMN 120
BAD06084.1      SISISDTFESDSPNKDMLPCYSVARIPLPNLNEDLTCGNILMWEAVTLKTEVLGVTTLMN 120
BAD06090.1      SISISDTFESDSPNKDMLPCYSVARIPLPNLNEDLTCGNILMWEAVTLKTEVLGVTTLMN 120
BAD06096.1      SISISDTFESDSPNKDMLPCYSVARIPLPNLNEDLTCGNILMWEAVTLKTEVLGVTTLMN 120
BAD06102.1      SISISDTFESDSPNKDMLPCYSVARIPLPNLNEDLTCGNILMWEAVTLKTEVLGVTTLMN 120
BAD06108.1      SISISDTFESDSPNKDMLPCYSVARIPLPNLNEDLTCGNILMWEAVTLKTEVLGVTTLMN 120
BAD06120.1      SISISDTFESDSPNKDMLPCYSVARIPLPNLNEDLTCGNILMWEAVTLKTEVLGVTTLMN 120
BAC15695.1      SISISDTFESDSPNKDMLPCYSVARIPLPNLNEDLTCGNILMWEAVTLKTEVLGVTTLMN 120
BAC15725.1      SISISDTFESDSPNKDMLPCYSVARIPLPNLNEDLTCGNILMWEAVTLKTEVLGVTTLMN 120
BAC02951.1      SISISDTFESDSPNKDMLPCYSVARIPLPNLNEDLTCGNILMWEAVTLKTEVLGVTTLMN 120
BAC02957.1      SISISDTFESDSPNKDMLPCYSVARIPLPNLNEDLTCGNILMWEAVTLKTEVLGVTTLMN 120
BAC02963.1      SISISDTFESDSPNKDMLPCYSVARIPLPNLNEDLTCGNILMWEAVTLKTEVLGVTTLMN 120
BAC02969.1      SISISDTFESDSPNKDMLPCYSVARIPLPNLNEDLTCGNILMWEAVTLKTEVLGVTTLMN 120
BAC02975.1      SISISDTFESDSPNKDMLPCYSVARIPLPNLNEDLTCGNILMWEAVTLKTEVLGVTTLMN 120
BAC02981.1      SISISDTFESDSPNKDMLPCYSVARIPLPNLNEDLTCGNILMWEAVTLKTEVLGVTTLMN 120
BAC02987.1      SISISDTFESDSPNKDMLPCYSVARIPLPNLNEDLTCGNILMWEAVTLKTEVLGVTTLMN 120
BAC02993.1      SISISDTFESDSPNKDMLPCYSVARIPLPNLNEDLTCGNILMWEAVTLKTEVLGVTTLMN 120
BAC02999.1      SISISDTFESDSPNKDMLPCYSVARIPLPNLNEDLTCGNILMWEAVTLKTEVLGVTTLMN 120
BAC03005.1      SISISDTFESDSPNKDMLPCYSVARIPLPNLNEDLTCGNILMWEAVTLKTEVLGVTTLMN 120
BAC03011.1      SISISDTFESDSPNKDMLPCYSVARIPLPNLNEDLTCGNILMWEAVTLKTEVLGVTTLMN 120
BAC03017.1      SISISDTFESDSPNKDMLPCYSVARIPLPNLNEDLTCGNILMWEAVTLKTEVLGVTTLMN 120
BAC03023.1      SISISDTFESDSPNKDMLPCYSVARIPLPNLNEDLTCGNILMWEAVTLKTEVLGVTTLMN 120
BAC03035.1      SISISDTFESDSPNKDMLPCYSVARIPLPNLNEDLTCGNILMWEAVTLKTEVLGVTTLMN 120
BAC03041.1      SISISDTFESDSPNKDMLPCYSVARIPLPNLNEDLTCGNILMWEAVTLKTEVLGVTTLMN 120
BAC03047.1      SISISDTFESDSPNKDMLPCYSVARIPLPNLNEDLTCGNILMWEAVTLKTEVLGVTTLMN 120
```

BAC03053.1	SISISDTFESDSPNKDMLPCYSVARIPLPNLNEDLTCGNILMWEAVTLKTEVLGVTTLMN	120
BAC03059.1	SISISDTFESDSPNKDMLPCYSVARIPLPNLNEDLTCGNILMWEAVTLKTEVLGVTTLMN	120
BAC03071.1	SISISDTFESDSPNKDMLPCYSVARIPLPNLNEDLTCGNILMWEAVTLKTEVLGVTTLMN	120
BAC03077.1	SISISDTFESDSPNKDMLPCYSVARIPLPNLNEDLTCGNILMWEAVTLKTEVLGVTTLMN	120
BAC03083.1	SISISDTFESDSPNKDMLPCYSVARIPLPNLNEDLTCGNILMWEAVTLKTEVLGVTTLMN	120
BAC03089.1	SISISDTFESDSPNKDMLPCYSVARIPLPNLNEDLTCGNILMWEAVTLKTEVLGVTTLMN	120
AAM69542.1	SISISDTFESDSPNKDMLPCYSVARIPLPNLNEDLTCGNILMWEAVTLKTEVLGVTTLMN	120
AAM69548.1	SISISDTFESDSPNKDMLPCYSVARIPLPNLNEDLTCGNILMWEAVTLKTEVLGVTTLMN	120
AAM69554.1	SISISDTFESDSPNKDMLPCYSVARIPLPNLNEDLTCGNILMWEAVTLKTEVLGVTTLMN	120
AAM69619.1	SISISDTFESDSPNKDMLPCYSVARIPLPNLNEDLTCGNILMWEAVTLKTEVLGVTTLMN	120
BAB68977.1	SISISDTFESDSPNKDMLPCYSVARIPLPNLNEDLTCGNILMWEAVTLKTEVLGVTTLMN	120
BAB68983.1	SISISDTFESDSPNKDMLPCYSVARIPLPNLNEDLTCGNILMWEAVTLKTEVLGVTTLMN	120
BAB68989.1	SISISDTFESDSPNKDMLPCYSVARIPLPNLNEDLTCGNILMWEAVTLKTEVLGVTTLMN	120
BAB68995.1	SISISDTFESDSPNKDMLPCYSVARIPLPNLNEDLTCGNILMWEAVTLKTEVLGVTTLMN	120
AAK98000.1	SISISDTFESDSPNKDMLPCYSVARIPLPNLNEDLTCGNILMWEAVTLKTEVLGVTTLMN	120
AAK98006.1	SISISDTFESDSPNKDMLPCYSVARIPLPNLNEDLTCGNILMWEAVTLKTEVLGVTTLMN	120
AAK98010.1	SISISDTFESDSPNKDMLPCYSVARIPLPNLNEDLTCGNILMWEAVTLKTEVLGVTTLMN	120
AAK98018.1	SISISDTFESDSPNKDMLPCYSVARIPLPNLNEDLTCGNILMWEAVTLKTEVLGVTTLMN	120
AAK98024.1	SISISDTFESDSPNKDMLPCYSVARIPLPNLNEDLTCGNILMWEAVTLKTEVLGVTTLMN	120
AAK98030.1	SISISDTFESDSPNKDMLPCYSVARIPLPNLNEDLTCGNILMWEAVTLKTEVLGVTTLMN	120
AAK98036.1	SISISDTFESDSPNKDMLPCYSVARIPLPNLNEDLTCGNILMWEAVTLKTEVLGVTTLMN	120
AAK70258.1	SISISDTFESDSPNKDMLPCYSVARIPLPNLNEDLTCGNILMWEAVTLKTEVLGVTTLMN	120
AAK70264.1	SISISDTFESDSPNKDMLPCYSVARIPLPNLNEDLTCGNILMWEAVTLKTEVLGVTTLMN	120
AAG53896.1	SISISDTFESDSPNKDMLPCYSVARIPLPNLNEDLTCGNILMWEAVTLKTEVLGVTTLMN	120
BAA01962.1	SISISDTFESDSPNKDMLPCYSVARIPLPNLNEDLTCGNILMWEAVTLKTEVLGVTTLMN	120
ALF06256.1	SISISDTFESDSPNKDMLPCYSVARIPLPNLNEDLTCGNILMWEAVTLKTEVJGVTTLMN	120
BAD06060.1	SISISDTFESDSPNKDMLPCYSVARIPLPNLNEDLTCGNILMWEAVTLKTEVIGVTTLMN	120
BAD06072.1	SISISDTFESDSPNKDMLPCYSVARIPLPNLNEDLTCGNILMWEAVTLKTEVIGVTTLMN	120
BAD06156.1	SISISDTFESDSPNKDMLPCYSVARIPLPNLNEDLTCGNILMWEAVTLKTEVIGVTTLMN	120
BAD06174.1	SISISDTFESDSPNKDMLPCYSVARIPLPNLNEDLTCGNILMWEAVTLKTEVIGVTTLMN	120
AAN85449.1	SISISDTFESDSPNKDMLPCYSVARIPLPNLNEDLTCGNILMWEAVTLKTEVIGVTTLMN	120
AAN85455.1	SISISDTFESDSPNKDMLPCYSVARIPLPNLNEDLTCGNILMWEAVTLKTEVIGVTTLMN	120
AAK98042.1	SISISDTFESDSPNKDMLPCYSVARIPLPNLNEDLTCGNILMWEAVTLKTEVIGVTTLMN	120
BAD06036.1	SISISDTFESDSPNKDMLPCYSVARIPLPNLNEDLTCGNILMWEAVTLKTEVLGVTTLMN	120
BCP65063.1	SISISDTFESDSPNKDMLPCYSVARIPLPNLNEDLTCGNILMWEAVTLKTEVLGVTTLMN	120
BCP56477.1	SISISDTFESDSPNKDMLPCYSVARIPLPNLNEDLTCGNILMWEAVTLKTEVLGVTTLMN	120
BAV69373.1	SISISDTFESDSPNKDMLPCYSVARIPLPNLNEDLTCGNILMWEAVTLKTEVLGVTTLMN	120
BAE00153.1	SISISDTFESDSPNKDMLPCYSVARIPLPNLNEDLTCGNILMWEAVTLKTEVLGVTTLMN	120
BAC22906.1	SISISDTFESDSPNKDMLPCYSVARIPLPNLNEDLTCGNILMWEAVTLKTEVLGVTTLMN	120
BAA05639.1	SISISDTFESDSPNKDMLPCYSVARIPLPNLNEDLTCGNILMWEAVTLKTEVLGVTTLMN	120
BAB11728.1	SISISDTFESDSPNKDMLPCYSVARIPLPNLNEDLTCGNILMWEAVTLKTEVLGVTTLMN	120
BAA05637.1	SISISDTFESDSPNKDMLPCYSVARIPLPNLNEDLTCGNILMWEAVTLKTEVLGVTTLMN	120
BAA01969.1	SISISDTFESDSPNKDMLPCYSVARIPLPNLNEDLTCGNILMWEAVTLKTEVLGVTTLMN	120
BAE00159.1	SISISDTFESDSPNKDMLPCYSVARIPLPNLNEDLTCGNILMWEAVTLKTEVLGVTTLMN	120
BAE00165.1	SISISDTFESDSPNKDMLPCYSVARIPLPNLNEDLTCGNILMWEAVTLKTEVLGVTTLMN	120
BAE00171.1	SISISDTFESDSPNKDMLPCYSVARIPLPNLNEDLTCGNILMWEAVTLKTEVLGVTTLMN	120
ALF06268.1	SISISDTFEGDSPNKDMLPCYSVARIPLPNLNEDLTCGNILMWEAVTLKTEVLGVTTLMN	120
AYM55586.1	SISISDTFESDSPNKDMLPCYSVARIPLPNLNEDLTCGNILMWEAVTLKTEVLGVTTLMN	120
AYM55585.1	SISISDTFESDSPNKDMLPCYSVARIPLPNLNEDLTCGNILMWEAVTLKTEVLGVTTLMN	120
AYM55587.1	SISISDTFESDSPNKDMLPCYSVARIPLPNLNEDLTCGNILMWEAVTLKTEVLGVTTLMN	120
AYM55594.1	SISISDTFESDSPNKDMLPCYSVARIPLPNLNEDLTCGNIQMWEAVTLKTEVLGVTTLMN	120
QBQ65470.1	SISISDTFDSDSPNKDMLPCYSVARIPLPNLNEDLTCGNILMWEAVTLKTEVIGVTTLMN	120
CCW72501.1	SISISDTFESDSPNKDMLPCYSVARIPLPNLNEDLTCGNILMWEAVTLKTEVIGVTTLMN	120
CCW72524.1	SISISDTFESDSPNKDMLPCYSVARIPLPNLNEDLTCGNILMWEAVTLKTEVIGVTTLMN	120
QWT77116.1	PIAISDTFESDSPNKDMLPCYSVARIPLPNLNEDLTCGNILMWEAVTLKTEVIGVTTLMN	120
WLD48046.1	SISISDTFESDSPNKEMLPCYSVARIPLPNLNEDLTCGNILMWEAVTLKTEVIGVTTLMN	120
WLD48052.1	SISISDTFESDSPNKEMLPCYSVARIPLPNLNEDLTCGNILMWEAVTLKTEVIGVTTLMN	120
AAK70288.1	SISISETFESDSPNKDMLPCYSVARIPLPNLNEDLTCGNILMWEAVTLKTEVIGVTTLMN	120
AAK70270.1	SISISDTFESDSPNKDMLPCYSVARIPLPNLNEDLTCGNILMWEAVTLKTEVIGVTTLMN	120
BAD21277.2	SISISDTFESDSPNKDMLPCYSVARIPLPNLNEDLTCGNILMWEAVTLKTEVIGVTTLMN	120
CCW72498.1	SISISDTFESDSPNKDMLPCYSVARIPLPNLNEDLTCGNILMWEAVTLKTEVIGVTTLMN	120
UPN63085.1	SISISDTFESDSPNKDMLPCYSVARIPLPNLNEDLTCGNILMWEAVTLKTEVIGVTTLMN	120
UPN63090.1	SISISDTFESDSPNKDMLPCYSVARIPLPNLNEDLTCGNILMWEAVTLKTEVIGVTTLMN	120
QWT77134.1	SISISDTFESDSPNKDMLPCYSVARIPLPNLNEDLTCGNILMWEAVTLKTEVIGVTTLMN	120

WLD48070.1	SISISDTFEGDSPNKDMLPCYSVARIPLPNLNEDLTCGNILMWEAVTLKTEVIGVTTLMN	120
AAK70282.1	SISISDTFESDSPNKDMLPCYSVARIPLPNLNEDLTCGNILMWEAVTLKTEVIGVTTLMN	120
WLD48058.1	SISISDTFESDSPNKDMLPCYSVARIPLPNLNEDLTCGNILMWEAVTLKTEVIGVTTLMN	120
WLD48076.1	SISISDTFESDSPNKDMLPCYSVARIPLPNLNEDLTCGNILMWEAVTLKTEVIGVTTLMN	120
UQK62667.1	SISISDTFESDSPNKDMLPCYSVARIPLPNLNEDLTCGNILMWEAVTLKTEVIGVTTLMN	120
CCW72480.1	SISISDTFESDSPNKDMLPCYSVARIPLPNLNEDLTCGNILMWEAVTLKTEVIGVTTLMN	120
CCW72513.1	SISISDTFESDSPNKDMLPCYSVARIPLPNLNEDLTCGNILMWEAVTLKTEVIGVTTLMN	120
AAM69614.1	SISISDTFESDSPNKDMLPCYSVARIPLPNLNEDLTCGNILMWEAVTLKTEVIGVTTLMS	120
AAK70318.1	SISISDTFESDSPNKDMLPCYSVARIPLPNLNEDLTCGNILMWEAVTLKTEVIGVTTLMN	120
AAK70324.1	SISISDTFESDSPNKDMLPCYSVARIPLPNLNEDLTCGNILMWEAVTLKTEVIGVTTLMN	120
AAT09837.1	SISISDTFESDSPNKDMLPCYSVARIPLPNLNEDLTCGNILMWEAVTLKTEVIGVTTLMN	120
AAT09831.1	SISISDTFESDSPNKDMLPCYSVARIPLPNLNEDLTCGNILMWEAVTLKTEVIGVTTLMN	120
AAT09825.1	SISISDTFESDSPNKDMLPCYSVARIPLPNLNEDLTCGNILMWEAVTLKTEVIGVTTLMN	120
AAR89271.1	SISISDTFESDSPNKDMLPCYSVARIPLPNLNEDLTCGNILMWEAVTLKTEVIGETTLMN	120
AAR89211.1	SISISDTFESDSPNKDMLPCYSVARIPLPNLNEDLTCGNILMWEAVTLKTEVIGVTTLMN	120
BAE02956.1	SISISDTFESDSPNKDMLPCYSVARIPLPNLNEDLTCGNILMWEAVTLKTEVIGVTTLMN	120
ACF75876.1	SISISDTFESDSPNKDMLPCYSVARIPLPNLNEDLTCGNILMWEAVTLKTEVIGVTTLMN	120
QBM11924.1	SISISDTFESDSPNKDMLPCYSVARIPLPNLNEDLTCGNILMWEAVTLKTEVIGVTTLMN	120
QBM11858.1	SISISDTFESDSPNKDMLPCYSVARIPLPNLNEDLTCGNILMWEAVTLKTEVIGVTTLMN	120
QBQ04203.1	SISISDTFESDSPNKDMLPCYSVARIPLPNLNEDLTCGNILMWEAVTLKTEVIGVTTLMN	120
QBQ65473.1	SISISDTFESDSPNKDMLPCYSVARIPLPNLNEDLTCGNILMWEAVTLKTEVIGVTTLMN	120
QBQ65472.1	SISISDTFESDSPNKDMLPCYSVARIPLPNLNEDLTCGNILMWEAVTLKTEVIGVTTLMN	120
QBM11920.1	SISISDTFESDSPNKDMLPCYSVARIPLPNLNEDLTCGNILMWEAVTLKTEVIGVTTLMN	120
QAA95693.1	SISISDTFESDSPNKDMLPCYSVARIPLPNLNEDLTCGNILMWEAVTLKTEVIGVTTLMN	120
QBX89122.1	SISISDTFESDSPNKDMLPCYSVARIPLPNLNEDLTCGNILMWEAVTLKTEVIGVTTLMN	120
QID57946.1	SISISDTFESDSPNKDMLPCYSVARIPLPNLNEDLTCGNILMWEAVTLKTEVIGVTTLMN	120
QID57952.1	SISISDTFESDSPNKDMLPCYSVARIPLPNLNEDLTCGNILMWEAVTLKTEVIGVTTLMN	120
QID57958.1	SISISDTFESDSPNKDMLPCYSVARIPLPNLNEDLTCGNILMWEAVTLKTEVIGVTTLMN	120
QID57964.1	SISISDTFESDSPNKDMLPCYSVARIPLPNLNEDLTCGNILMWEAVTLKTEVIGVTTLMN	120
QID57970.1	SISISDTFESDSPNKDMLPCYSVARIPLPNLNEDLTCGNILMWEAVTLKTEVIGVTTLMN	120
QID57976.1	SISISDTFESDSPNKDMLPCYSVARIPLPNLNEDLTCGNILMWEAVTLKTEVIGVTTLMN	120
QCH41110.1	SISISDTFESDSPNKDMLPCYSVARIPLPNLNEDLTCGNILMWEAVTLKTEVIGVTTLMN	120
QBX89120.1	SISISDTFESDSPNKDMLPCYSVARIPLPNLNEDLTCGNILMWEAVTLKTEVIGVTTLMN	120
QBX89121.1	SISISDTFESDSPNKDMLPCYSVARIPLPNLNEDLTCGNILMWEAVTLKTEVIGVTTLMN	120
QBX89124.1	SISISDTFESDSPNKDMLPCYSVARIPLPNLNEDLTCGNILMWEAVTLKTEVIGVTTLMN	120
QBQ65471.1	SISISDTFESDSPNKDMLPCYSVARIPLPNLNEDLTCGNILMWEAVTLKTEVIGVTTLMN	120
QBQ65474.1	SISISDTFESDSPNKDMLPCYSVARIPLPNLNEDLTCGNILMWEAVTLKTEVIGVTTLMN	120
QBQ65476.1	SISISDTFESDSPNKDMLPCYSVARIPLPNLNEDLTCGNILMWEAVTLKTEVIGVTTLMN	120
QBQ04201.1	SISISDTFESDSPNKDMLPCYSVARIPLPNLNEDLTCGNILMWEAVTLKTEVIGVTTLMN	120
QBM11921.1	SISISDTFESDSPNKDMLPCYSVARIPLPNLNEDLTCGNILMWEAVTLKTEVIGVTTLMN	120
QBM11922.1	SISISDTFESDSPNKDMLPCYSVARIPLPNLNEDLTCGNILMWEAVTLKTEVIGVTTLMN	120
CDJ79845.1	SISISDTFESDSPNKDMLPCYSVARIPLPNLNEDLTCGNILMWEAVTLKTEVIGVTTLMN	120
BAE02932.1	SISISDTFESDSPNKDMLPCYSVARIPLPNLNEDLTCGNILMWEAVTLKTEVIGVTTLMN	120
BAE02938.1	SISISDTFESDSPNKDMLPCYSVARIPLPNLNEDLTCGNILMWEAVTLKTEVIGVTTLMN	120
BAE02950.1	SISISDTFESDSPNKDMLPCYSVARIPLPNLNEDLTCGNILMWEAVTLKTEVIGVTTLMN	120
BAE03004.1	SISISDTFESDSPNKDMLPCYSVARIPLPNLNEDLTCGNILMWEAVTLKTEVIGVTTLMN	120
BAE03118.1	SISISDTFESDSPNKDMLPCYSVARIPLPNLNEDLTCGNILMWEAVTLKTEVIGVTTLMN	120
BAE03124.1	SISISDTFESDSPNKDMLPCYSVARIPLPNLNEDLTCGNILMWEAVTLKTEVIGVTTLMN	120
BAE03130.1	SISISDTFESDSPNKDMLPCYSVARIPLPNLNEDLTCGNILMWEAVTLKTEVIGVTTLMN	120
BAE03136.1	SISISDTFESDSPNKDMLPCYSVARIPLPNLNEDLTCGNILMWEAVTLKTEVIGVTTLMN	120
BAE03142.1	SISISDTFESDSPNKDMLPCYSVARIPLPNLNEDLTCGNILMWEAVTLKTEVIGVTTLMN	120
BAE03148.1	SISISDTFESDSPNKDMLPCYSVARIPLPNLNEDLTCGNILMWEAVTLKTEVIGVTTLMN	120
BAE03154.1	SISISDTFESDSPNKDMLPCYSVARIPLPNLNEDLTCGNILMWEAVTLKTEVIGVTTLMN	120
QBQ04200.1	SISISDTFESDSPNKDMLPCYSVARIPLPNLNEDLTCGNILMWEAVTLKTEVIGVTTLMN	120
UYL83839.1	SISISDTFESDSPNKDMLPCYSVARIPLPNLNEDLTCGNILMWEAVTLKTEVIGVTTLMN	120
ALF06262.1	SISISDTFESDSPNKDMLPCYSVARIPLPNLNEDLTCGNILMWEAVTLKTEVIGVTTLMN	120
ABH10586.1	SISISDTFESDSPNKDMLPCYSVARIPLPNLNEDLTCGNILMWEAVTLKTEVIGVTTLMN	120
BAE02890.1	SISISDTFESDSPNKDMLPCYSVARIPLPNLNEDLTCGNILMWEAVTLKTEVIGVTTLMN	120
BAE02896.1	SISISDTFESDSPNKDMLPCYSVARIPLPNLNEDLTCGNILMWEAVTLKTEVIGVTTLMN	120
BAE02902.1	SISISDTFESDSPNKDMLPCYSVARIPLPNLNEDLTCGNILMWEAVTLKTEVIGVTTLMN	120
BAE02914.1	SISISDTFESDSPNKDMLPCYSVARIPLPNLNEDLTCGNILMWEAVTLKTEVIGVTTLMN	120
BAE02920.1	SISISDTFESDSPNKDMLPCYSVARIPLPNLNEDLTCGNILMWEAVTLKTEVIGVTTLMN	120
BAE02944.1	SISISDTFESDSPNKDMLPCYSVARIPLPNLNEDLTCGNILMWEAVTLKTEVIGVTTLMN	120
BAE02962.1	SISISDTFESDSPNKDMLPCYSVARIPLPNLNEDLTCGNILMWEAVTLKTEVIGVTTLMN	120

```
BAE02968.1          SISISDTFESDSPNKDMLPCYSVARIPLPNLNEDLTCGNILMWEAVTLKTEVIGVTTLMN  120
BAE02974.1          SISISDTFESDSPNKDMLPCYSVARIPLPNLNEDLTCGNILMWEAVTLKTEVIGVTTLMN  120
BAE02980.1          SISISDTFESDSPNKDMLPCYSVARIPLPNLNEDLTCGNILMWEAVTLKTEVIGVTTLMN  120
BAE02986.1          SISISDTFESDSPNKDMLPCYSVARIPLPNLNEDLTCGNILMWEAVTLKTEVIGVTTLMN  120
BAE02992.1          SISISDTFESDSPNKDMLPCYSVARIPLPNLNEDLTCGNILMWEAVTLKTEVIGVTTLMN  120
BAE02998.1          SISISDTFESDSPNKDMLPCYSVARIPLPNLNEDLTCGNILMWEAVTLKTEVIGVTTLMN  120
BAE03010.1          SISISDTFESDSPNKDMLPCYSVARIPLPNLNEDLTCGNILMWEAVTLKTEVIGVTTLMN  120
BAE03016.1          SISISDTFESDSPNKDMLPCYSVARIPLPNLNEDLTCGNILMWEAVTLKTEVIGVTTLMN  120
BAE03022.1          SISISDTFESDSPNKDMLPCYSVARIPLPNLNEDLTCGNILMWEAVTLKTEVIGVTTLMN  120
BAE03028.1          SISISDTFESDSPNKDMLPCYSVARIPLPNLNEDLTCGNILMWEAVTLKTEVIGVTTLMN  120
BAE03034.1          SISISDTFESDSPNKDMLPCYSVARIPLPNLNEDLTCGNILMWEAVTLKTEVIGVTTLMN  120
BAE03040.1          SISISDTFESDSPNKDMLPCYSVARIPLPNLNEDLTCGNILMWEAVTLKTEVIGVTTLMN  120
BAE03046.1          SISISDTFESDSPNKDMLPCYSVARIPLPNLNEDLTCGNILMWEAVTLKTEVIGVTTLMN  120
BAE03052.1          SISISDTFESDSPNKDMLPCYSVARIPLPNLNEDLTCGNILMWEAVTLKTEVIGVTTLMN  120
BAE03058.1          SISISDTFESDSPNKDMLPCYSVARIPLPNLNEDLTCGNILMWEAVTLKTEVIGVTTLMN  120
BAE03064.1          SISISDTFESDSPNKDMLPCYSVARIPLPNLNEDLTCGNILMWEAVTLKTEVIGVTTLMN  120
BAE03070.1          SISISDTFESDSPNKDMLPCYSVARIPLPNLNEDLTCGNILMWEAVTLKTEVIGVTTLMN  120
BAE03076.1          SISISDTFESDSPNKDMLPCYSVARIPLPNLNEDLTCGNILMWEAVTLKTEVIGVTTLMN  120
BAE03082.1          SISISDTFESDSPNKDMLPCYSVARIPLPNLNEDLTCGNILMWEAVTLKTEVIGVTTLMN  120
BAE03088.1          SISISDTFESDSPNKDMLPCYSVARIPLPNLNEDLTCGNILMWEAVTLKTEVIGVTTLMN  120
BAE03094.1          SISISDTFESDSPNKDMLPCYSVARIPLPNLNEDLTCGNILMWEAVTLKTEVIGVTTLMN  120
BAE03100.1          SISISDTFESDSPNKDMLPCYSVARIPLPNLNEDLTCGNILMWEAVTLKTEVIGVTTLMN  120
BAE03106.1          SISISDTFESDSPNKDMLPCYSVARIPLPNLNEDLTCGNILMWEAVTLKTEVIGVTTLMN  120
BAE03112.1          SISISDTFESDSPNKDMLPCYSVARIPLPNLNEDLTCGNILMWEAVTLKTEVIGVTTLMN  120
BAE03160.1          SISISDTFESDSPNKDMLPCYSVARIPLPNLNEDLTCGNILMWEAVTLKTEVIGVTTLMN  120
BAE03166.1          SISISDTFESDSPNKDMLPCYSVARIPLPNLNEDLTCGNILMWEAVTLKTEVIGVTTLMN  120
AAQ88264.1          SISISDTFESDSPNKDMLPCYSVARIPLPNLNEDLTCGNILMWEAVTLKTEVIGVTTLMN  120
AAQ88270.1          SISISDTFESDSPNKDMLPCYSVARIPLPNLNEDLTCGNILMWEAVTLKTEVIGVTTLMN  120
AAR89187.1          SISISDTFESDSPNKDMLPCYSVARIPLPNLNEDLTCGNILMWEAVTLKTEVIGVTTLMN  120
AAR89193.1          SISISDTFESDSPNKDMLPCYSVARIPLPNLNEDLTCGNILMWEAVTLKTEVIGVTTLMN  120
AAR89199.1          SISISDTFESDSPNKDMLPCYSVARIPLPNLNEDLTCGNILMWEAVTLKTEVIGVTTLMN  120
AAR89205.1          SISISDTFESDSPNKDMLPCYSVARIPLPNLNEDLTCGNILMWEAVTLKTEVIGVTTLMN  120
AAR89217.1          SISISDTFESDSPNKDMLPCYSVARIPLPNLNEDLTCGNILMWEAVTLKTEVIGVTTLMN  120
AAR89223.1          SISISDTFESDSPNKDMLPCYSVARIPLPNLNEDLTCGNILMWEAVTLKTEVIGVTTLMN  120
AAR89229.1          SISISDTFESDSPNKDMLPCYSVARIPLPNLNEDLTCGNILMWEAVTLKTEVIGVTTLMN  120
AAR89235.1          SISISDTFESDSPNKDMLPCYSVARIPLPNLNEDLTCGNILMWEAVTLKTEVIGVTTLMN  120
AAR89241.1          SISISDTFESDSPNKDMLPCYSVARIPLPNLNEDLTCGNILMWEAVTLKTEVIGVTTLMN  120
AAR89247.1          SISISDTFESDSPNKDMLPCYSVARIPLPNLNEDLTCGNILMWEAVTLKTEVIGVTTLMN  120
AAR89253.1          SISISDTFESDSPNKDMLPCYSVARIPLPNLNEDLTCGNILMWEAVTLKTEVIGVTTLMN  120
AAR32743.1          SISISDTFESDSPNKDMLPCYSVARIPLPNLNEDLTCGNILMWEAVTLKTEVIGVTTLMN  120
AAR89265.1          SISISDTFESDSPNKDMLPCYSVARIPLPNLNEDLTCGNILMWEAVTLKTEVIGVTTLMN  120
AAR89277.1          SISISDTFESDSPNKDMLPCYSVARIPLPNLNEDLTCGNILMWEAVTLKTEVIGVTTLMN  120
AAR89283.1          SISISDTFESDSPNKDMLPCYSVARIPLPNLNEDLTCGNILMWEAVTLKTEVIGVTTLMN  120
AAR02463.1          SISISDTFESDSPNKDMLPCYSVARIPLPNLNEDLTCGNILMWEAVTLKTEVIGVTTLMN  120
AAR06661.1          SISISDTFESDSPNKDMLPCYSVARIPLPNLNEDLTCGNILMWEAVTLKTEVIGVTTLMN  120
AAR13659.1          SISISDTFESDSPNKDMLPCYSVARIPLPNLNEDLTCGNILMWEAVTLKTEVIGVTTLMN  120
AAR02457.1          SISISDTFESDSPNKDMLPCYSVARIPLPNLNEDLTCGNILMWEAVTLKTEVIGVTTLMN  120
BAB68785.1          SISISDTFESDSPNKDMLPCYSVARIPLPNLNEDLTCGNILMWEAVTLKTEVIGVTTLMN  120
AAK28472.1          SISISDTFESDSPNKDMLPCYSVARIPLPNLNEDLTCGNILMWEAVTLKTEVIGVTTLMN  120
AAG34667.1          SISISDTFESDSPNKDMLPCYSVARIPLPNLNEDLTCGNILMWEAVTLKTEVIGVTTLMN  120
AAG34673.1          SISISDTFESDSPNKDMLPCYSVARIPLPNLNEDLTCGNILMWEAVTLKTEVIGVTTLMN  120
AAB41705.1          SISISDTFESDSPNKDMLPCYSVARIPLPNLNEDLTCGNILMWEAVTLKTEVIGVTTLMN  120
AAB41711.1          SISISDTFESDSPNKDMLPCYSVARIPLPNLNEDLTCGNILMWEAVTLKTEVIGVTTLMN  120
AAB41717.1          SISISDTFESDSPNKDMLPCYSVARIPLPNLNEDLTCGNILMWEAVTLKTEVIGVTTLMN  120
BAE02926.1          SISISDTFESDSPNKDMLPCYSVARIPLPNLNEDLTCGNILMWEAVTLKTEVIGVTTLMN  120
                     *: *  * .* * :: ** :****:*** ****** *** **:**:** ** * * **.

AEC14619.1          VHSNXXXXXXXXAGKPVQGTSFHFFSVGGEALELQGVLFNYRTKYPDGTIFPKNATVQSQ  180
AEC15199.1          VHSNGQATHDNGAGKPVQGTSFHFFSVGGEALELQGVLFNYRTKYPDGTIFPKNATVQSQ  180
AEC15209.1          VHSNGQATHDNGAGKPVQGTSFHFFSVGGEALELQGVLFNYRTKYPDGTIFPKNATVQSQ  180
UPN63098.1          VHSNGQATHDNGAGKPVQSTSFHFFSVGGEALELQGVLFNHRTKYPDGTIFPKDATVQSQ  180
UPN63097.1          VHSNGQATHDNGAGKPVQGTSFHFFSVGGEALELQGVLFNYRTKYPDGAIFPKNATVQSQ  180
UPN63094.1          VHSNGQATHDNGAGKPVQGTSFHFFSVGGEALELQGVLFNYRTKYPDGTIFPKNATVQSQ  180
UPN63096.1          VHSNGRATHDNGAGKPVQGTSFHFFSVGGEALELQGVLFNYRTKYPDGTIFPKNATVQSQ  180
```

QWT77146.1	VHSNGQATHDNGAGKPVQGTSFHFFSVGGEALELQGVLFNYRTKYPDGTIFPKNATVQSQ	180
UPN63095.1	VHSNGQATHDNGAGKPVQGTSFHFFSVGGEALELQGVLFNYRTKYPDGTIFPKNATVQSQ	180
BAA01966.1	VHSNGQATHDNGAGKPVQGTSFHFFSVGGEALELQGVLFNYRTKYPDGTIFPKNATVQSQ	180
AEC15099.1	VHSNGQATHDNGAGKPVQGTSFHFFSVGGEALELQGVLFNYRTKYPDGTIFPKNATVQSQ	180
UPN63099.1	VHSNGQATHDNGAGKPVQGTSFHFFSVGGEALELQGVLFNYRTKYPDGTIFPKNATVQSQ	180
UPN63086.1	VHSNGQATHDNGAGKPVQGTSFHFFSVGGEALELQGVLFNYRTKYPDGTIFPKNATVQSQ	180
UPN63092.1	VHSNGQATHDNGAGKPVQGTSFHFFSVGGEALELQGVLFNYRTKYPDGTIFPKNATVQSQ	180
AEC15783.1	VHSNGQATHDNGAGKPVQGTSFHFFFVGGEALELQGVLFNYRTKYPDGTIFPKNATVQSQ	180
AEC15735.1	VHSNGQATHDNGAGKPVQGTSFHFFSVGGEALELQGVLFNYRTKYPDGTIFPKNATVQSQ	180
AEC15615.1	VHSNGQVTHDNGAGKPVQGTSFHFFSVGGEALELQGVLFNYRTKYPDGTIFPKNATVQSQ	180
AEC15549.1	VHSNGQATHDNGAGKPVQGTSFHFFSVGGEALELQGVLFNYRTKYPDGTIFPKNATVQSQ	180
AEC15081.1	VHSNGQATHDNGAGKPVQGTSFHFFSVGGEALELQGVLFNYRTKYPDGTIFPKNATVQSQ	180
AEC15080.1	VHSNGQATHDNGAGKPVQGTSFHFFSVGGEALELQGVLFNYRTKYPDGTIFPKNATVQSQ	180
AEC15083.1	VHSNGQATHDNGAGKPVQGTSFHFFSVGGEALELQGVLFNYRTKYPDGTIFPKNATVQSQ	180
QWT77109.1	VHSNGQATHDNGAGKPVQGTSFHFFSVGGEALELQGVLFNYRTKYPDGTIFPKNATVQSQ	180
AEC15069.1	VHSNGQATHDNGAGKPVQGTSFHFFSVGGEALELQGVLFNYRTKYPDGTIFPKNATVQSQ	180
AEC15070.1	VHSNGQATHDNGAGKPVQGTSFHFFSVGGEALELQGVLFNYRTKYPDGTIFPKNATVQSQ	180
AEC15071.1	VHSNGQATHDNGAGKPVQGTSFHFFSVGGEALELQGVLFNYRTKYPDGTIFPKNATVQSQ	180
AEC15072.1	VHSNGQATHDNGAGKPVQGTSFHFFSVGGEALELQGVLFNYRTKYPDGTIFPKNATVQSQ	180
AEC15073.1	VHSNGQATHDNGAGKPVQGTSFHFFSVGGEALELQGVLFNYRTKYPDGTIFPKNATVQSQ	180
AEC15074.1	VHSNGQATHDNGAGKPVQGTSFHFFSVGGEALELQGVLFNYRTKYPDGTIFPKNATVQSQ	180
AEC15076.1	VHSNGQATHDNGAGKPVQGTSFHFFSVGGEALELQGVLFNYRTKYPDGTIFPKNATVQSQ	180
AEC15077.1	VHSNGQATHDNGAGKPVQGTSFHFFSVGGEALELQGVLFNYRTKYPDGTIFPKNATVQSQ	180
AEC15078.1	VHSNGQATHDNGAGKPVQGTSFHFFSVGGEALELQGVLFNYRTKYPDGTIFPKNATVQSQ	180
AEC15079.1	VHSNGQATHDNGAGKPVQGTSFHFFSVGGEALELQGVLFNYRTKYPDGTIFPKNATVQSQ	180
AEC15082.1	VHSNGQATHDNGAGKPVQGTSFHFFSVGGEALELQGVLFNYRTKYPDGTIFPKNATVQSQ	180
AEC15084.1	VHSNGQATHDNGAGKPVQGTSFHFFSVGGEALELQGVLFNYRTKYPDGTIFPKNATVQSQ	180
AEC15085.1	VHSNGQATHDNGAGKPVQGTSFHFFSVGGEALELQGVLFNYRTKYPDGTIFPKNATVQSQ	180
AEC15086.1	VHSNGQATHDNGAGKPVQGTSFHFFSVGGEALELQGVLFNYRTKYPDGTIFPKNATVQSQ	180
AEC15087.1	VHSNGQATHDNGAGKPVQGTSFHFFSVGGEALELQGVLFNYRTKYPDGTIFPKNATVQSQ	180
AEC15089.1	VHSNGQATHDNGAGKPVQGTSFHFFSVGGEALELQGVLFNYRTKYPDGTIFPKNATVQSQ	180
AEC15555.1	VHSNGQATHDNGAGKPVQGTSFHFFSVGGEALELQGVLFNYRTKYPDGTIFPKNATVQSQ	180
AEC15561.1	VHSNGQATHDNGAGKPVQGTSFHFFSVGGEALELQGVLFNYRTKYPDGTIFPKNATVQSQ	180
AEC15567.1	VHSNGQATHDNGAGKPVQGTSFHFFSVGGEALELQGVLFNYRTKYPDGTIFPKNATVQSQ	180
AEC15573.1	VHSNGQATHDNGAGKPVQGTSFHFFSVGGEALELQGVLFNYRTKYPDGTIFPKNATVQSQ	180
AEC15579.1	VHSNGQATHDNGAGKPVQGTSFHFFSVGGEALELQGVLFNYRTKYPDGTIFPKNATVQSQ	180
AEC15585.1	VHSNGQATHDNGAGKPVQGTSFHFFSVGGEALELQGVLFNYRTKYPDGTIFPKNATVQSQ	180
AEC15591.1	VHSNGQATHDNGAGKPVQGTSFHFFSVGGEALELQGVLFNYRTKYPDGTIFPKNATVQSQ	180
AEC15597.1	VHSNGQATHDNGAGKPVQGTSFHFFSVGGEALELQGVLFNYRTKYPDGTIFPKNATVQSQ	180
AEC15603.1	VHSNGQATHDNGAGKPVQGTSFHFFSVGGEALELQGVLFNYRTKYPDGTIFPKNATVQSQ	180
AEC15609.1	VHSNGQATHDNGAGKPVQGTSFHFFSVGGEALELQGVLFNYRTKYPDGTIFPKNATVQSQ	180
AEC15621.1	VHSNGQATHDNGAGKPVQGTSFHFFSVGGEALELQGVLFNYRTKYPDGTIFPKNATVQSQ	180
AEC15627.1	VHSNGQATHDNGAGKPVQGTSFHFFSVGGEALELQGVLFNYRTKYPDGTIFPKNATVQSQ	180
AEC15633.1	VHSNGQATHDNGAGKPVQGTSFHFFSVGGEALELQGVLFNYRTKYPDGTIFPKNATVQSQ	180
AEC15639.1	VHSNGQATHDNGAGKPVQGTSFHFFSVGGEALELQGVLFNYRTKYPDGTIFPKNATVQSQ	180
AEC15645.1	VHSNGQATHDNGAGKPVQGTSFHFFSVGGEALELQGVLFNYRTKYPDGTIFPKNATVQSQ	180
AEC15651.1	VHSNGQATHDNGAGKPVQGTSFHFFSVGGEALELQGVLFNYRTKYPDGTIFPKNATVQSQ	180
AEC15657.1	VHSNGQATHDNGAGKPVQGTSFHFFSVGGEALELQGVLFNYRTKYPDGTIFPKNATVQSQ	180
AEC15663.1	VHSNGQATHDNGAGKPVQGTSFHFFSVGGEALELQGVLFNYRTKYPDGTIFPKNATVQSQ	180
AEC15669.1	VHSNGQATHDNGAGKPVQGTSFHFFSVGGEALELQGVLFNYRTKYPDGTIFPKNATVQSQ	180
AEC15675.1	VHSNGQATHDNGAGKPVQGTSFHFFSVGGEALELQGVLFNYRTKYPDGTIFPKNATVQSQ	180
AEC15681.1	VHSNGQATHDNGAGKPVQGTSFHFFSVGGEALELQGVLFNYRTKYPDGTIFPKNATVQSQ	180
AEC15687.1	VHSNGQATHDNGAGKPVQGTSFHFFSVGGEALELQGVLFNYRTKYPDGTIFPKNATVQSQ	180
AEC15693.1	VHSNGQATHDNGAGKPVQGTSFHFFSVGGEALELQGVLFNYRTKYPDGTIFPKNATVQSQ	180
AEC15699.1	VHSNGQATHDNGAGKPVQGTSFHFFSVGGEALELQGVLFNYRTKYPDGTIFPKNATVQSQ	180
AEC15705.1	VHSNGQATHDNGAGKPVQGTSFHFFSVGGEALELQGVLFNYRTKYPDGTIFPKNATVQSQ	180
AEC15711.1	VHSNGQATHDNGAGKPVQGTSFHFFSVGGEALELQGVLFNYRTKYPDGTIFPKNATVQSQ	180
AEC15717.1	VHSNGQATHDNGAGKPVQGTSFHFFSVGGEALELQGVLFNYRTKYPDGTIFPKNATVQSQ	180
AEC15723.1	VHSNGQATHDNGAGKPVQGTSFHFFSVGGEALELQGVLFNYRTKYPDGTIFPKNATVQSQ	180
AEC15729.1	VHSNGQATHDNGAGKPVQGTSFHFFSVGGEALELQGVLFNYRTKYPDGTIFPKNATVQSQ	180
AEC15741.1	VHSNGQATHDNGAGKPVQGTSFHFFSVGGEALELQGVLFNYRTKYPDGTIFPKNATVQSQ	180
AEC15747.1	VHSNGQATHDNGAGKPVQGTSFHFFSVGGEALELQGVLFNYRTKYPDGTIFPKNATVQSQ	180
AEC15753.1	VHSNGQATHDNGAGKPVQGTSFHFFSVGGEALELQGVLFNYRTKYPDGTIFPKNATVQSQ	180
AEC15759.1	VHSNGQATHDNGAGKPVQGTSFHFFSVGGEALELQGVLFNYRTKYPDGTIFPKNATVQSQ	180

```
AEC15765.1       VHSNGQATHDNGAGKPVQGTSFHFFSVGGEALELQGVLFNYRTKYPDGTIFPKNATVQSQ  180
AEC15771.1       VHSNGQATHDNGAGKPVQGTSFHFFSVGGEALELQGVLFNYRTKYPDGTIFPKNATVQSQ  180
AEC15777.1       VHSNGQATHDNGAGKPVQGTSFHFFSVGGEALELQGVLFNYRTKYPDGTIFPKNATVQSQ  180
AEC15789.1       VHSNGQATHDNGAGKPVQGTSFHFFSVGGEALELQGVLFNYRTKYPDGTIFPKNATVQSQ  180
AEC15795.1       VHSNGQATHDNGAGKPVQGTSFHFFSVGGEALELQGVLFNYRTKYPDGTIFPKNATVQSQ  180
AEC15801.1       VHSNGQATHDNGAGKPVQGTSFHFFSVGGEALELQGVLFNYRTKYPDGTIFPKNATVQSQ  180
AEC15807.1       VHSNGQATHDNGAGKPVQGTSFHFFSVGGEALELQGVLFNYRTKYPDGTIFPKNATVQSQ  180
AEC15813.1       VHSNGQATHDNGAGKPVQGTSFHFFSVGGEALELQGVLFNYRTKYPDGTIFPKNATVQSQ  180
AEC15075.1       VHSNGQATHDNGAGKPVQGTSFHFFSVGGEALELQGVLFNYRTKYPDGTIFPKNATVQSQ  180
AEC15088.1       VHSNGQATHDNGAGKPVQGTSFHFFSVGGEALELQGVLFNYRTKYPDGTIFPKNATVQSQ  180
AEC14559.1       VHSNGQATHDNGAGKPVQGTSFHFFSVGGEALELQGGAFNYRTKYPDGTIFPKNATVQSQ  180
AEC15023.1       VHSNGQATHDNGAGKPVQGTSFHFFSVGGEALELQGVLFNYRTKYPDGTIFPKNATVQSQ  180
AEC14518.1       VHSNGQATHDNGAGKPVQGTSFHFFSVGGEALELQGVLFNYRTKYPDGTIFPKNATVQSQ  180
AEC14589.1       VHSNGQATHDNGAGKPVQGTSFHFFSVGGEALELQGVLFNYRTKYPDGTIFPKNATVQSQ  180
AEC14625.1       VHSNGQATHDNGAGKPVQGTSFHFFSVGGEALELQGVLFNYRTKYPDGTIFPKNATVQSQ  180
AEC14631.1       VHSNGQATHDNGAGKPVQGTSFHFFSVGGEALELQGVLFNYRTKYPDGTIFPKNATVQSQ  180
AEC14643.1       VHSNGQATHDNGAGKPVQGTSFHFFSVGGEALELQGVLFNYRTKYPDGTIFPKNATVQSQ  180
AEC14966.1       VHSNGQATHDNGAGKPVQGTSFHFFSVGGEALELQGVLFNYRTKYPDGTIFPKNATVQSQ  180
AEC14979.1       VHSNGQATHDNGAGKPVQGTSFHFFSVGGEALELQGVLFNYRTKYPDGTIFPKNATVQSQ  180
AEC14990.1       VHSNGQATHDNGAGKPVQGTSFHFFSVGGEALELQGVLFNYRTKYPDGTIFPKNATVQSQ  180
AEC14999.1       VHSNGQATHDNGAGKPVQGTSFHFFSVGGEALELQGVLFNYRTKYPDGTIFPKNATVQSQ  180
AEC15020.1       VHSNGQATHDNGAGKPVQGTSFHFFSVGGEALELQGVLFNYRTKYPDGTIFPKNATVQSQ  180
AEC14595.1       VHSNGQATHDNGAGKPVQGTSFHFFSVGGEALELQGVLFNYRTKYPDGTIFPKNATVQSQ  180
AEC14703.1       VHSNGQATHDNGAGKPVQGTSFHFFSVGGEALELQGVLFNYRTKYPDGTIFPKNATVQSQ  180
AEC15011.1       VHSNGQATHDNGAGKPVQGTSFHFFSVGGEALELQGVLFNYRTKYPDGTIFPKNATVQSQ  180
AEC15019.1       VHSNGQATHDNGAGKPVQGTSFHFFSVGGEALELQGVLFNYRTKYPDGTIFPKNATVQSQ  180
AEC15014.1       VHSNGQATHDNGAGKPVQGTSFHFFSVGGEALELQGVLFNYRTKYPDGTIFPKNATVQSQ  180
NP_043511.1      VHSNGQATHDNGAGKPVQGTSFHFFSVGGEALELQGVLFNYRTKYPDGTIFPKNATVQSQ  180
UPN63087.1       VHSNGQATHDNGAGKPVQGTSFHFFSVGGEALELQGVLFNYRTKYPDGTIFPKNATVQSQ  180
ASV51760.1       VHSNGQATHDNGAGKPVQGTSFHFFSVGGEALELQGVLFNYRTKYPDGTIFPKNATVQSQ  180
BAV69379.1       VHSNGQATHDNGAGKPVQGTSFHFFSVGGEALELQGVLFNYRTKYPDGTIFPKNATVQSQ  180
AEC15090.1       VHSNGQATHDNGAGKPVQGTSFHFFSVGGEALELQGVLFNYRTKYPDGTIFPKNATVQSQ  180
AEC15091.1       VHSNGQATHDNGAGKPVQGTSFHFFSVGGEALELQGVLFNYRTKYPDGTIFPKNATVQSQ  180
AEC15092.1       VHSNGQATHDNGAGKPVQGTSFHFFSVGGEALELQGVLFNYRTKYPDGTIFPKNATVQSQ  180
AEC15093.1       VHSNGQATHDNGAGKPVQGTSFHFFSVGGEALELQGVLFNYRTKYPDGTIFPKNATVQSQ  180
AEC15094.1       VHSNGQATHDNGAGKPVQGTSFHFFSVGGEALELQGVLFNYRTKYPDGTIFPKNATVQSQ  180
AEC15095.1       VHSNGQATHDNGAGKPVQGTSFHFFSVGGEALELQGVLFNYRTKYPDGTIFPKNATVQSQ  180
AEC15096.1       VHSNGQATHDNGAGKPVQGTSFHFFSVGGEALELQGVLFNYRTKYPDGTIFPKNATVQSQ  180
AEC15097.1       VHSNGQATHDNGAGKPVQGTSFHFFSVGGEALELQGVLFNYRTKYPDGTIFPKNATVQSQ  180
AEC15098.1       VHSNGQATHDNGAGKPVQGTSFHFFSVGGEALELQGVLFNYRTKYPDGTIFPKNATVQSQ  180
AEC15100.1       VHSNGQATHDNGAGKPVQGTSFHFFSVGGEALELQGVLFNYRTKYPDGTIFPKNATVQSQ  180
AEC15101.1       VHSNGQATHDNGAGKPVQGTSFHFFSVGGEALELQGVLFNYRTKYPDGTIFPKNATVQSQ  180
AEC15102.1       VHSNGQATHDNGAGKPVQGTSFHFFSVGGEALELQGVLFNYRTKYPDGTIFPKNATVQSQ  180
AEC15103.1       VHSNGQATHDNGAGKPVQGTSFHFFSVGGEALELQGVLFNYRTKYPDGTIFPKNATVQSQ  180
AEC15104.1       VHSNGQATHDNGAGKPVQGTSFHFFSVGGEALELQGVLFNYRTKYPDGTIFPKNATVQSQ  180
AEC15105.1       VHSNGQATHDNGAGKPVQGTSFHFFSVGGEALELQGVLFNYRTKYPDGTIFPKNATVQSQ  180
AEC15106.1       VHSNGQATHDNGAGKPVQGTSFHFFSVGGEALELQGVLFNYRTKYPDGTIFPKNATVQSQ  180
AEC15107.1       VHSNGQATHDNGAGKPVQGTSFHFFSVGGEALELQGVLFNYRTKYPDGTIFPKNATVQSQ  180
AEC15108.1       VHSNGQATHDNGAGKPVQGTSFHFFSVGGEALELQGVLFNYRTKYPDGTIFPKNATVQSQ  180
AEC15109.1       VHSNGQATHDNGAGKPVQGTSFHFFSVGGEALELQGVLFNYRTKYPDGTIFPKNATVQSQ  180
AEC15110.1       VHSNGQATHDNGAGKPVQGTSFHFFSVGGEALELQGVLFNYRTKYPDGTIFPKNATVQSQ  180
AEC15111.1       VHSNGQATHDNGAGKPVQGTSFHFFSVGGEALELQGVLFNYRTKYPDGTIFPKNATVQSQ  180
AEC15963.1       VHSNGQATHDNGAGKPVQGTSFHFFSVGGEALELQGVLFNYRTKYPDGTIFPKNATVQSQ  180
AEC15969.1       VHSNGQATHDNGAGKPVQGTSFHFFSVGGEALELQGVLFNYRTKYPDGTIFPKNATVQSQ  180
AEC15975.1       VHSNGQATHDNGAGKPVQGTSFHFFSVGGEALELQGVLFNYRTKYPDGTIFPKNATVQSQ  180
AEC15981.1       VHSNGQATHDNGAGKPVQGTSFHFFSVGGEALELQGVLFNYRTKYPDGTIFPKNATVQSQ  180
AEC15987.1       VHSNGQATHDNGAGKPVQGTSFHFFSVGGEALELQGVLFNYRTKYPDGTIFPKNATVQSQ  180
AEC15993.1       VHSNGQATHDNGAGKPVQGTSFHFFSVGGEALELQGVLFNYRTKYPDGTIFPKNATVQSQ  180
CAD69038.1       VHSNGQATHDNGAGKPVQGTSFHFFSVGGEALELQGVLFNYRTKYPDGTIFPKNATVQSQ  180
AAK70240.1       VHSNGQATHDNGAGKPVQGTSFHFFSVGGEALELQGVLFNYRTKYPDGTIFPKNATVQSQ  180
BAB11710.1       VHSNGQATHDNGAGKPVQGTSFHFFSVGGEALELQGVLFNYRTKYPDGTIFPKNATVQSQ  180
BAA01958.1       VHSNGQATHDNGAGKPVQGTSFHFFSVGGEALELQGVLFNYRTKYPDGTIFPKNATVQSQ  180
AAA82101.1       VHSNGQATHDNGAGKPVQGTSFHFFSVGGEALELQGVLFNYRTKYPDGTIFPKNATVQSQ  180
P03089.1         VHSNGQATHDNGAGKPVQGTSFHFFSVGGEALELQGVLFNYRTKYPDGTIFPKNATVQSQ  180
```

UPN63102.1	VHSNGQATHDNGAGKPVQGTSFHFFSVGGEALELQGVLFNYRTKYPDGTIFPKNATVQSQ	180
AEC15025.1	VHSNGQATHDNGAGKPVQGTSFHFFSVGGEALELQGVLFNYRTKYPDGTIFPKNATVQSQ	180
AEC15026.1	VHSNGQATHDNGAGKPVQGTSFHFFSVGGEALELQGVLFNYRTKYPDGTIFPKNATVQSQ	180
AEC15027.1	VHSNGQATHDNGAGKPVQGTSFHFFSVGGEALELQGVLFNYRTKYPDGTIFPKNATVQSQ	180
AEC15028.1	VHSNGQATHDNGAGKPVQGTSFHFFSVGGEALELQGVLFNYRTKYPDGTIFPKNATVQSQ	180
AEC15029.1	VHSNGQATHDNGAGKPVQGTSFHFFSVGGEALELQGVLFNYRTKYPDGTIFPKNATVQSQ	180
AEC15196.1	VHSNGQATHDNGAGKPVQGTSFHFFSVGGEALELQGVLFNYRTKYPDGTIFPKNATVQSQ	180
AEC15197.1	VHSNGQATHDNGAGKPVQGTSFHFFSVGGEALELQGVLFNYRTKYPDGTIFPKNATVQSQ	180
AEC15198.1	VHSNGQATHDNGAGKPVQGTSFHFFSVGGEALELQGVLFNYRTKYPDGTIFPKNATVQSQ	180
AEC15200.1	VHSNGQATHDNGAGKPVQGTSFHFFSVGGEALELQGVLFNYRTKYPDGTIFPKNATVQSQ	180
AEC15201.1	VHSNGQATHDNGAGKPVQGTSFHFFSVGGEALELQGVLFNYRTKYPDGTIFPKNATVQSQ	180
AEC15202.1	VHSNGQATHDNGAGKPVQGTSFHFFSVGGEALELQGVLFNYRTKYPDGTIFPKNATVQSQ	180
AEC15203.1	VHSNGQATHDNGAGKPVQGTSFHFFSVGGEALELQGVLFNYRTKYPDGTIFPKNATVQSQ	180
AEC15204.1	VHSNGQATHDNGAGKPVQGTSFHFFSVGGEALELQGVLFNYRTKYPDGTIFPKNATVQSQ	180
AEC15205.1	VHSNGQATHDNGAGKPVQGTSFHFFSVGGEALELQGVLFNYRTKYPDGTIFPKNATVQSQ	180
AEC15206.1	VHSNGQATHDNGAGKPVQGTSFHFFSVGGEALELQGVLFNYRTKYPDGTIFPKNATVQSQ	180
AEC15207.1	VHSNGQATHDNGAGKPVQGTSFHFFSVGGEALELQGVLFNYRTKYPDGTIFPKNATVQSQ	180
AEC15208.1	VHSNGQATHDNGAGKPVQGTSFHFFSVGGEALELQGVLFNYRTKYPDGTIFPKNATVQSQ	180
AEC15210.1	VHSNGQATHDNGAGKPVQGTSFHFFSVGGEALELQGVLFNYRTKYPDGTIFPKNATVQSQ	180
AEC15211.1	VHSNGQATHDNGAGKPVQGTSFHFFSVGGEALELQGVLFNYRTKYPDGTIFPKNATVQSQ	180
AEC15212.1	VHSNGQATHDNGAGKPVQGTSFHFFSVGGEALELQGVLFNYRTKYPDGTIFPKNATVQSQ	180
AEC15213.1	VHSNGQATHDNGAGKPVQGTSFHFFSVGGEALELQGVLFNYRTKYPDGTIFPKNATVQSQ	180
AEC15214.1	VHSNGQATHDNGAGKPVQGTSFHFFSVGGEALELQGVLFNYRTKYPDGTIFPKNATVQSQ	180
AEC15215.1	VHSNGQATHDNGAGKPVQGTSFHFFSVGGEALELQGVLFNYRTKYPDGTIFPKNATVQSQ	180
AEC15216.1	VHSNGQATHDNGAGKPVQGTSFHFFSVGGEALELQGVLFNYRTKYPDGTIFPKNATVQSQ	180
AEC15217.1	VHSNGQATHDNGAGKPVQGTSFHFFSVGGEALELQGVLFNYRTKYPDGTIFPKNATVQSQ	180
AEC15218.1	VHSNGQATHDNGAGKPVQGTSFHFFSVGGEALELQGVLFNYRTKYPDGTIFPKNATVQSQ	180
AEC15327.1	VHSNGQATHDNGAGKPVQGTSFHFFSVGGEALELQGVLFNYRTKYPDGTIFPKNATVQSQ	180
AEC15333.1	VHSNGQATHDNGAGKPVQGTSFHFFSVGGEALELQGVLFNYRTKYPDGTIFPKNATVQSQ	180
AEC15339.1	VHSNGQATHDNGAGKPVQGTSFHFFSVGGEALELQGVLFNYRTKYPDGTIFPKNATVQSQ	180
AEC15345.1	VHSNGQATHDNGAGKPVQGTSFHFFSVGGEALELQGVLFNYRTKYPDGTIFPKNATVQSQ	180
AEC15351.1	VHSNGQATHDNGAGKPVQGTSFHFFSVGGEALELQGVLFNYRTKYPDGTIFPKNATVQSQ	180
AEC15357.1	VHSNGQATHDNGAGKPVQGTSFHFFSVGGEALELQGVLFNYRTKYPDGTIFPKNATVQSQ	180
AEC15363.1	VHSNGQATHDNGAGKPVQGTSFHFFSVGGEALELQGVLFNYRTKYPDGTIFPKNATVQSQ	180
AEC15369.1	VHSNGQATHDNGAGKPVQGTSFHFFSVGGEALELQGVLFNYRTKYPDGTIFPKNATVQSQ	180
AEC15375.1	VHSNGQATHDNGAGKPVQGTSFHFFSVGGEALELQGVLFNYRTKYPDGTIFPKNATVQSQ	180
AEC15381.1	VHSNGQATHDNGAGKPVQGTSFHFFSVGGEALELQGVLFNYRTKYPDGTIFPKNATVQSQ	180
AEC15387.1	VHSNGQATHDNGAGKPVQGTSFHFFSVGGEALELQGVLFNYRTKYPDGTIFPKNATVQSQ	180
AEC15393.1	VHSNGQATHDNGAGKPVQGTSFHFFSVGGEALELQGVLFNYRTKYPDGTIFPKNATVQSQ	180
AEC15399.1	VHSNGQATHDNGAGKPVQGTSFHFFSVGGEALELQGVLFNYRTKYPDGTIFPKNATVQSQ	180
AEC15405.1	VHSNGQATHDNGAGKPVQGTSFHFFSVGGEALELQGVLFNYRTKYPDGTIFPKNATVQSQ	180
AEC15411.1	VHSNGQATHDNGAGKPVQGTSFHFFSVGGEALELQGVLFNYRTKYPDGTIFPKNATVQSQ	180
AEC15417.1	VHSNGQATHDNGAGKPVQGTSFHFFSVGGEALELQGVLFNYRTKYPDGTIFPKNATVQSQ	180
AEC15423.1	VHSNGQATHDNGAGKPVQGTSFHFFSVGGEALELQGVLFNYRTKYPDGTIFPKNATVQSQ	180
AEC15429.1	VHSNGQATHDNGAGKPVQGTSFHFFSVGGEALELQGVLFNYRTKYPDGTIFPKNATVQSQ	180
AEC15435.1	VHSNGQATHDNGAGKPVQGTSFHFFSVGGEALELQGVLFNYRTKYPDGTIFPKNATVQSQ	180
AEC15441.1	VHSNGQATHDNGAGKPVQGTSFHFFSVGGEALELQGVLFNYRTKYPDGTIFPKNATVQSQ	180
BAA01967.1	VHSNGQATHDNGAGKPVQGTSFHFFSVGGEALELQGVLFNYRTKYPDGTIFPKNATVQSQ	180
ASV51844.1	VHSNGQASHDNGAGKPVQGTSFHFFSVGGEALELQGVLFNYRTKYPDGTIFPKNATVQSQ	180
ASV51850.1	VHSNGQASHDNGAGKPVQGTSFHFFSVGGEALELQGVLFNYRTKYPDGTIFPKNATVQSQ	180
CCW72489.1	VHSNGQATHDNGAGKPVQGTSFHFFSVGGEALELQGVLFNYRTKYPDGTIFPKNATVQSQ	180
AEC15170.1	VHSNGQATHDNGAGKPVQGTSFHFFSVGGEALELQGVLFNYRTKYPDGTIFPKNATVQSQ	180
AEC15171.1	VHSNGQATHDNGAGKPVQGTSFHFFSVGGEALELQGVLFNYRTKYPDGTIFPKNATVQSQ	180
AEC15299.1	VHSNGQATHDNGAGKPVQGTSFHFFSVGGEALELQGVLFNYRTKYPDGTIFPKNATVQSQ	180
BAB93050.1	VHSNGQATHDNGAGKPVQGTSFHFFSVGGEALELQGVLFNYRTKYPDGTIFPKNATVQSQ	180
UPN63100.1	VHSNGQATHDNGAGKPVQGTSFHFFSVGGEALELQGVLFNYRTKYPDGTIFPKNATVQSQ	180
CCW72482.1	VHSNGQATHDNGAGKPVQGTSFHFFSVGGEALELQGVLFNYRTKYPDGTIFPKNATVQSQ	180
AEC15165.1	VHSNGQATHDNGAGKPVQGTSFHFFSVGGEALELQGVLFNYRTKYPDGTIFPKNATVQSQ	180
AEC15166.1	VHSNGQATHDNGAGKPVQGTSFHFFSVGGEALELQGVLFNYRTKYPDGTIFPKNATVQSQ	180
AEC15266.1	VHSNGQATHDNGAGKPVQGTSFHFFSVGGEALELQGVLFNYRTKYPDGTIFPKNATVQSQ	180
AEC15267.1	VHSNGQATHDNGAGKPVQGTSFHFFSVGGEALELQGVLFNYRTKYPDGTIFPKNATVQSQ	180
AEC15268.1	VHSNGQATHDNGAGKPVQGTSFHFFSVGGEALELQGVLFNYRTKYPDGTIFPKNATVQSQ	180
AEC15269.1	VHSNGQATHDNGAGKPVQGTSFHFFSVGGEALELQGVLFNYRTKYPDGTIFPKNATVQSQ	180
AEC15270.1	VHSNGQATHDNGAGKPVQGTSFHFFSVGGEALELQGVLFNYRTKYPDGTIFPKNATVQSQ	180

```
AEC15271.1        VHSNGQATHDNGAGKPVQGTSFHFFSVGGEALELQGVLFNYRTKYPDGTIFPKNATVQSQ  180
AEC15272.1        VHSNGQATHDNGAGKPVQGTSFHFFSVGGEALELQGVLFNYRTKYPDGTIFPKNATVQSQ  180
AEC15273.1        VHSNGQATHDNGAGKPVQGTSFHFFSVGGEALELQGVLFNYRTKYPDGTIFPKNATVQSQ  180
AEC15274.1        VHSNGQATHDNGAGKPVQGTSFHFFSVGGEALELQGVLFNYRTKYPDGTIFPKNATVQSQ  180
AEC15275.1        VHSNGQATHDNGAGKPVQGTSFHFFSVGGEALELQGVLFNYRTKYPDGTIFPKNATVQSQ  180
AEC15276.1        VHSNGQATHDNGAGKPVQGTSFHFFSVGGEALELQGVLFNYRTKYPDGTIFPKNATVQSQ  180
AEC15277.1        VHSNGQATHDNGAGKPVQGTSFHFFSVGGEALELQGVLFNYRTKYPDGTIFPKNATVQSQ  180
AEC15278.1        VHSNGQATHDNGAGKPVQGTSFHFFSVGGEALELQGVLFNYRTKYPDGTIFPKNATVQSQ  180
AEC15279.1        VHSNGQATHDNGAGKPVQGTSFHFFSVGGEALELQGVLFNYRTKYPDGTIFPKNATVQSQ  180
AEC15280.1        VHSNGQATHDNGAGKPVQGTSFHFFSVGGEALELQGVLFNYRTKYPDGTIFPKNATVQSQ  180
AEC15281.1        VHSNGQATHDNGAGKPVQGTSFHFFSVGGEALELQGVLFNYRTKYPDGTIFPKNATVQSQ  180
AEC15282.1        VHSNGQATHDNGAGKPVQGTSFHFFSVGGEALELQGVLFNYRTKYPDGTIFPKNATVQSQ  180
AEC15283.1        VHSNGQATHDNGAGKPVQGTSFHFFSVGGEALELQGVLFNYRTKYPDGTIFPKNATVQSQ  180
AEC15284.1        VHSNGQATHDNGAGKPVQGTSFHFFSVGGEALELQGVLFNYRTKYPDGTIFPKNATVQSQ  180
AEC15285.1        VHSNGQATHDNGAGKPVQGTSFHFFSVGGEALELQGVLFNYRTKYPDGTIFPKNATVQSQ  180
AEC15286.1        VHSNGQATHDNGAGKPVQGTSFHFFSVGGEALELQGVLFNYRTKYPDGTIFPKNATVQSQ  180
AEC15287.1        VHSNGQATHDNGAGKPVQGTSFHFFSVGGEALELQGVLFNYRTKYPDGTIFPKNATVQSQ  180
AEC15288.1        VHSNGQATHDNGAGKPVQGTSFHFFSVGGEALELQGVLFNYRTKYPDGTIFPKNATVQSQ  180
AEC15289.1        VHSNGQATHDNGAGKPVQGTSFHFFSVGGEALELQGVLFNYRTKYPDGTIFPKNATVQSQ  180
AEC15290.1        VHSNGQATHDNGAGKPVQGTSFHFFSVGGEALELQGVLFNYRTKYPDGTIFPKNATVQSQ  180
AEC15291.1        VHSNGQATHDNGAGKPVQGTSFHFFSVGGEALELQGVLFNYRTKYPDGTIFPKNATVQSQ  180
AEC15292.1        VHSNGQATHDNGAGKPVQGTSFHFFSVGGEALELQGVLFNYRTKYPDGTIFPKNATVQSQ  180
AEC15293.1        VHSNGQATHDNGAGKPVQGTSFHFFSVGGEALELQGVLFNYRTKYPDGTIFPKNATVQSQ  180
AEC15294.1        VHSNGQATHDNGAGKPVQGTSFHFFSVGGEALELQGVLFNYRTKYPDGTIFPKNATVQSQ  180
AEC15295.1        VHSNGQATHDNGAGKPVQGTSFHFFSVGGEALELQGVLFNYRTKYPDGTIFPKNATVQSQ  180
AEC15296.1        VHSNGQATHDNGAGKPVQGTSFHFFSVGGEALELQGVLFNYRTKYPDGTIFPKNATVQSQ  180
AEC15297.1        VHSNGQATHDNGAGKPVQGTSFHFFSVGGEALELQGVLFNYRTKYPDGTIFPKNATVQSQ  180
AEC15298.1        VHSNGQATHDNGAGKPVQGTSFHFFSVGGEALELQGVLFNYRTKYPDGTIFPKNATVQSQ  180
AEC15300.1        VHSNGQATHDNGAGKPVQGTSFHFFSVGGEALELQGVLFNYRTKYPDGTIFPKNATVQSQ  180
AEC15301.1        VHSNGQATHDNGAGKPVQGTSFHFFSVGGEALELQGVLFNYRTKYPDGTIFPKNATVQSQ  180
AEC15302.1        VHSNGQATHDNGAGKPVQGTSFHFFSVGGEALELQGVLFNYRTKYPDGTIFPKNATVQSQ  180
AEC15303.1        VHSNGQATHDNGAGKPVQGTSFHFFSVGGEALELQGVLFNYRTKYPDGTIFPKNATVQSQ  180
AEC15304.1        VHSNGQATHDNGAGKPVQGTSFHFFSVGGEALELQGVLFNYRTKYPDGTIFPKNATVQSQ  180
AEC15305.1        VHSNGQATHDNGAGKPVQGTSFHFFSVGGEALELQGVLFNYRTKYPDGTIFPKNATVQSQ  180
AEC15306.1        VHSNGQATHDNGAGKPVQGTSFHFFSVGGEALELQGVLFNYRTKYPDGTIFPKNATVQSQ  180
AEC15307.1        VHSNGQATHDNGAGKPVQGTSFHFFSVGGEALELQGVLFNYRTKYPDGTIFPKNATVQSQ  180
AEC15308.1        VHSNGQATHDNGAGKPVQGTSFHFFSVGGEALELQGVLFNYRTKYPDGTIFPKNATVQSQ  180
AEC15309.1        VHSNGQATHDNGAGKPVQGTSFHFFSVGGEALELQGVLFNYRTKYPDGTIFPKNATVQSQ  180
AEC15310.1        VHSNGQATHDNGAGKPVQGTSFHFFSVGGEALELQGVLFNYRTKYPDGTIFPKNATVQSQ  180
AEC15311.1        VHSNGQATHDNGAGKPVQGTSFHFFSVGGEALELQGVLFNYRTKYPDGTIFPKNATVQSQ  180
AEC15861.1        VHSNGQATHDNGAGKPVQGTSFHFFSVGGEALELQGVLFNYRTKYPDGTIFPKNATVQSQ  180
AEC15867.1        VHSNGQATHDNGAGKPVQGTSFHFFSVGGEALELQGVLFNYRTKYPDGTIFPKNATVQSQ  180
AEC15873.1        VHSNGQATHDNGAGKPVQGTSFHFFSVGGEALELQGVLFNYRTKYPDGTIFPKNATVQSQ  180
AEC15879.1        VHSNGQATHDNGAGKPVQGTSFHFFSVGGEALELQGVLFNYRTKYPDGTIFPKNATVQSQ  180
AEC15885.1        VHSNGQATHDNGAGKPVQGTSFHFFSVGGEALELQGVLFNYRTKYPDGTIFPKNATVQSQ  180
AEC15891.1        VHSNGQATHDNGAGKPVQGTSFHFFSVGGEALELQGVLFNYRTKYPDGTIFPKNATVQSQ  180
AEC15897.1        VHSNGQATHDNGAGKPVQGTSFHFFSVGGEALELQGVLFNYRTKYPDGTIFPKNATVQSQ  180
AEC15903.1        VHSNGQATHDNGAGKPVQGTSFHFFSVGGEALELQGVLFNYRTKYPDGTIFPKNATVQSQ  180
AEC15909.1        VHSNGQATHDNGAGKPVQGTSFHFFSVGGEALELQGVLFNYRTKYPDGTIFPKNATVQSQ  180
AEC15915.1        VHSNGQATHDNGAGKPVQGTSFHFFSVGGEALELQGVLFNYRTKYPDGTIFPKNATVQSQ  180
AEC15921.1        VHSNGQATHDNGAGKPVQGTSFHFFSVGGEALELQGVLFNYRTKYPDGTIFPKNATVQSQ  180
AEC15927.1        VHSNGQATHDNGAGKPVQGTSFHFFSVGGEALELQGVLFNYRTKYPDGTIFPKNATVQSQ  180
AEC15933.1        VHSNGQATHDNGAGKPVQGTSFHFFSVGGEALELQGVLFNYRTKYPDGTIFPKNATVQSQ  180
AEC15939.1        VHSNGQATHDNGAGKPVQGTSFHFFSVGGEALELQGVLFNYRTKYPDGTIFPKNATVQSQ  180
AEC15945.1        VHSNGQATHDNGAGKPVQGTSFHFFSVGGEALELQGVLFNYRTKYPDGTIFPKNATVQSQ  180
AEC15951.1        VHSNGQATHDNGAGKPVQGTSFHFFSVGGEALELQGVLFNYRTKYPDGTIFPKNATVQSQ  180
AEC15957.1        VHSNGQATHDNGAGKPVQGTSFHFFSVGGEALELQGVLFNYRTKYPDGTIFPKNATVQSQ  180
BAD21259.1        VHSNGQATHDNGAGKPVQGTSFHFFSVGGEALELQGVLFNYRTKYPDGTIFPKNATVQSQ  180
BAD21265.1        VHSNGQATHDNGAGKPVQGTSFHFFSVGGEALELQGVLFNYRTKYPDGTIFPKNATVQSQ  180
AAC59325.1        VHSNGQATHDNGAGKPVQGTSFHFFSVGGEALELQGVLFNYRTKYPDGTIFPKNATVQSQ  180
AEC14524.1        VHSNGQATHDNGAGKPVQGTSFHFFSVGGEALELQGVLFNYRTKYPDGTIFPKNATVQSQ  180
AEC14541.1        VHSNGQATHDNGAGKPVQGTSFHFFSVGGEALELQGVLFNYRTKYPDGTIFPKNATVQSQ  180
AEC14565.1        VHSNGQATHDNGAGKPVQGTSFHFFSVGGEALELQGVLFNYRTKYPDGTIFPKNATVQSQ  180
AEC14571.1        VHSNGQATHDNGAGKPVQGTSFHFFSVGGEALELQGVLFNYRTKYPDGTIFPKNATVQSQ  180
```

AEC14583.1	VHSNGQATHDNGAGKPVQGTSFHFFSVGGEALELQGVLFNYRTKYPDGTIFPKNATVQSQ	180
AEC14601.1	VHSNGQATHDNGAGKPVQGTSFHFFSVGGEALELQGVLFNYRTKYPDGTIFPKNATVQSQ	180
AEC14607.1	VHSNGQATHDNGAGKPVQGTSFHFFSVGGEALELQGVLFNYRTKYPDGTIFPKNATVQSQ	180
AEC14637.1	VHSNGQATHDNGAGKPVQGTSFHFFSVGGEALELQGVLFNYRTKYPDGTIFPKNATVQSQ	180
AEC14649.1	VHSNGQATHDNGAGKPVQGTSFHFFSVGGEALELQGVLFNYRTKYPDGTIFPKNATVQSQ	180
AEC14655.1	VHSNGQATHDNGAGKPVQGTSFHFFSVGGEALELQGVLFNYRTKYPDGTIFPKNATVQSQ	180
AEC14661.1	VHSNGQATHDNGAGKPVQGTSFHFFSVGGEALELQGVLFNYRTKYPDGTIFPKNATVQSQ	180
AEC14685.1	VHSNGQATHDNGAGKPVQGTSFHFFSVGGEALELQGVLFNYRTKYPDGTIFPKNATVQSQ	180
AEC14697.1	VHSNGQATHDNGAGKPVQGTSFHFFSVGGEALELQGVLFNYRTKYPDGTIFPKNATVQSQ	180
AEC14709.1	VHSNGQATHDNGAGKPVQGTSFHFFSVGGEALELQGVLFNYRTKYPDGTIFPKNATVQSQ	180
AEC14715.1	VHSNGQATHDNGAGKPVQGTSFHFFSVGGEALELQGVLFNYRTKYPDGTIFPKNATVQSQ	180
AEC14963.1	VHSNGQATHDNGAGKPVQGTSFHFFSVGGEALELQGVLFNYRTKYPDGTIFPKNATVQSQ	180
AEC14968.1	VHSNGQATHDNGAGKPVQGTSFHFFSVGGEALELQGVLFNYRTKYPDGTIFPKNATVQSQ	180
AEC14971.1	VHSNGQATHDNGAGKPVQGTSFHFFSVGGEALELQGVLFNYRTKYPDGTIFPKNATVQSQ	180
AEC14972.1	VHSNGQATHDNGAGKPVQGTSFHFFSVGGEALELQGVLFNYRTKYPDGTIFPKNATVQSQ	180
AEC14975.1	VHSNGQATHDNGAGKPVQGTSFHFFSVGGEALELQGVLFNYRTKYPDGTIFPKNATVQSQ	180
AEC14976.1	VHSNGQATHDNGAGKPVQGTSFHFFSVGGEALELQGVLFNYRTKYPDGTIFPKNATVQSQ	180
AEC14977.1	VHSNGQATHDNGAGKPVQGTSFHFFSVGGEALELQGVLFNYRTKYPDGTIFPKNATVQSQ	180
AEC14978.1	VHSNGQATHDNGAGKPVQGTSFHFFSVGGEALELQGVLFNYRTKYPDGTIFPKNATVQSQ	180
AEC14980.1	VHSNGQATHDNGAGKPVQGTSFHFFSVGGEALELQGVLFNYRTKYPDGTIFPKNATVQSQ	180
AEC14981.1	VHSNGQATHDNGAGKPVQGTSFHFFSVGGEALELQGVLFNYRTKYPDGTIFPKNATVQSQ	180
AEC14982.1	VHSNGQATHDNGAGKPVQGTSFHFFSVGGEALELQGVLFNYRTKYPDGTIFPKNATVQSQ	180
AEC14983.1	VHSNGQATHDNGAGKPVQGTSFHFFSVGGEALELQGVLFNYRTKYPDGTIFPKNATVQSQ	180
AEC14985.1	VHSNGQATHDNGAGKPVQGTSFHFFSVGGEALELQGVLFNYRTKYPDGTIFPKNATVQSQ	180
AEC14986.1	VHSNGQATHDNGAGKPVQGTSFHFFSVGGEALELQGVLFNYRTKYPDGTIFPKNATVQSQ	180
AEC14988.1	VHSNGQATHDNGAGKPVQGTSFHFFSVGGEALELQGVLFNYRTKYPDGTIFPKNATVQSQ	180
AEC14989.1	VHSNGQATHDNGAGKPVQGTSFHFFSVGGEALELQGVLFNYRTKYPDGTIFPKNATVQSQ	180
AEC14991.1	VHSNGQATHDNGAGKPVQGTSFHFFSVGGEALELQGVLFNYRTKYPDGTIFPKNATVQSQ	180
AEC14992.1	VHSNGQATHDNGAGKPVQGTSFHFFSVGGEALELQGVLFNYRTKYPDGTIFPKNATVQSQ	180
AEC14996.1	VHSNGQATHDNGAGKPVQGTSFHFFSVGGEALELQGVLFNYRTKYPDGTIFPKNATVQSQ	180
AEC14997.1	VHSNGQATHDNGAGKPVQGTSFHFFSVGGEALELQGVLFNYRTKYPDGTIFPKNATVQSQ	180
AEC14998.1	VHSNGQATHDNGAGKPVQGTSFHFFSVGGEALELQGVLFNYRTKYPDGTIFPKNATVQSQ	180
AEC15002.1	VHSNGQATHDNGAGKPVQGTSFHFFSVGGEALELQGVLFNYRTKYPDGTIFPKNATVQSQ	180
AEC15003.1	VHSNGQATHDNGAGKPVQGTSFHFFSVGGEALELQGVLFNYRTKYPDGTIFPKNATVQSQ	180
AEC15004.1	VHSNGQATHDNGAGKPVQGTSFHFFSVGGEALELQGVLFNYRTKYPDGTIFPKNATVQSQ	180
AEC15005.1	VHSNGQATHDNGAGKPVQGTSFHFFSVGGEALELQGVLFNYRTKYPDGTIFPKNATVQSQ	180
AEC15006.1	VHSNGQATHDNGAGKPVQGTSFHFFSVGGEALELQGVLFNYRTKYPDGTIFPKNATVQSQ	180
AEC15009.1	VHSNGQATHDNGAGKPVQGTSFHFFSVGGEALELQGVLFNYRTKYPDGTIFPKNATVQSQ	180
AEC15012.1	VHSNGQATHDNGAGKPVQGTSFHFFSVGGEALELQGVLFNYRTKYPDGTIFPKNATVQSQ	180
AEC15013.1	VHSNGQATHDNGAGKPVQGTSFHFFSVGGEALELQGVLFNYRTKYPDGTIFPKNATVQSQ	180
AEC15015.1	VHSNGQATHDNGAGKPVQGTSFHFFSVGGEALELQGVLFNYRTKYPDGTIFPKNATVQSQ	180
AEC15017.1	VHSNGQATHDNGAGKPVQGTSFHFFSVGGEALELQGVLFNYRTKYPDGTIFPKNATVQSQ	180
AEC15018.1	VHSNGQATHDNGAGKPVQGTSFHFFSVGGEALELQGVLFNYRTKYPDGTIFPKNATVQSQ	180
AEC15021.1	VHSNGQATHDNGAGKPVQGTSFHFFSVGGEALELQGVLFNYRTKYPDGTIFPKNATVQSQ	180
AEC15022.1	VHSNGQATHDNGAGKPVQGTSFHFFSVGGEALELQGVLFNYRTKYPDGTIFPKNATVQSQ	180
AEC14962.1	VHSNGQATHDNGAGKPVQGTSFHFFSVGGEALELQGVLFNYRTKYPDGTIFPKNATVQSQ	180
AEC14993.1	VHSNGQATHDNGAGKPVQGTSFHFFSVGGEALELQGVLFNYRTKYPDGTIFPKNATVQSQ	180
AEC15007.1	VHSNGQATHDNGAGKPVQGTSFHFFSVGGEALELQGVLFNYRTKYPDGTIFPKNATVQSQ	180
CCW72517.1	VHSNGQATHDNGAGKPVQGTSFHFFSVGGEALELQGVVFNYRTKYPDGTIFPKNATVQSQ	180
BAD21247.2	VHSNGQATHDNGAGKPVQGTSFHFFSVGGEALELQGVVFNYRTKYPDGTIFPKNATVQSQ	180
AAL37695.1	VHSNGQATHDNGAGKPVQGTSFHFFSVGGEALELQGVLFNYRTKYPDGTIFPKNATVQSQ	180
AEC15242.1	VHSNGQATHDNGAGKPVQGTSFHFFSVGGEALELQGVLFNYRTKYPDGTIFPKNATVQSQ	180
AEC15243.1	VHSNGQATHDNGAGKPVQGTSFHFFSVGGEALELQGVLFNYRTKYPDGTIFPKNATVQSQ	180
AEC15244.1	VHSNGQATHDNGAGKPVQGTSFHFFSVGGEALELQGVLFNYRTKYPDGTIFPKNATVQSQ	180
AEC15245.1	VHSNGQATHDNGAGKPVQGTSFHFFSVGGEALELQGVLFNYRTKYPDGTIFPKNATVQSQ	180
AEC15246.1	VHSNGQATHDNGAGKPVQGTSFHFFSVGGEALELQGVLFNYRTKYPDGTIFPKNATVQSQ	180
AEC15247.1	VHSNGQATHDNGAGKPVQGTSFHFFSVGGEALELQGVLFNYRTKYPDGTIFPKNATVQSQ	180
AEC15248.1	VHSNGQATHDNGAGKPVQGTSFHFFSVGGEALELQGVLFNYRTKYPDGTIFPKNATVQSQ	180
AEC15249.1	VHSNGQATHDNGAGKPVQGTSFHFFSVGGEALELQGVLFNYRTKYPDGTIFPKNATVQSQ	180
AEC15250.1	VHSNGQATHDNGAGKPVQGTSFHFFSVGGEALELQGVLFNYRTKYPDGTIFPKNATVQSQ	180
AEC15251.1	VHSNGQATHDNGAGKPVQGTSFHFFSVGGEALELQGVLFNYRTKYPDGTIFPKNATVQSQ	180
AEC15252.1	VHSNGQATHDNGAGKPVQGTSFHFFSVGGEALELQGVLFNYRTKYPDGTIFPKNATVQSQ	180
AEC15253.1	VHSNGQATHDNGAGKPVQGTSFHFFSVGGEALELQGVLFNYRTKYPDGTIFPKNATVQSQ	180
AEC15254.1	VHSNGQATHDNGAGKPVQGTSFHFFSVGGEALELQGVLFNYRTKYPDGTIFPKNATVQSQ	180

AEC15255.1	VHSNGQATHDNGAGKPVQGTSFHFFSVGGEALELQGVLFNYRTKYPDGTIFPKNATVQSQ	180
AEC15256.1	VHSNGQATHDNGAGKPVQGTSFHFFSVGGEALELQGVLFNYRTKYPDGTIFPKNATVQSQ	180
AEC15257.1	VHSNGQATHDNGAGKPVQGTSFHFFSVGGEALELQGVLFNYRTKYPDGTIFPKNATVQSQ	180
AEC15258.1	VHSNGQATHDNGAGKPVQGTSFHFFSVGGEALELQGVLFNYRTKYPDGTIFPKNATVQSQ	180
AEC15259.1	VHSNGQATHDNGAGKPVQGTSFHFFSVGGEALELQGVLFNYRTKYPDGTIFPKNATVQSQ	180
AEC15260.1	VHSNGQATHDNGAGKPVQGTSFHFFSVGGEALELQGVLFNYRTKYPDGTIFPKNATVQSQ	180
AEC15261.1	VHSNGQATHDNGAGKPVQGTSFHFFSVGGEALELQGVLFNYRTKYPDGTIFPKNATVQSQ	180
AEC15262.1	VHSNGQATHDNGAGKPVQGTSFHFFSVGGEALELQGVLFNYRTKYPDGTIFPKNATVQSQ	180
AEC15263.1	VHSNGQATHDNGAGKPVQGTSFHFFSVGGEALELQGVLFNYRTKYPDGTIFPKNATVQSQ	180
AEC15264.1	VHSNGQATHDNGAGKPVQGTSFHFFSVGGEALELQGVLFNYRTKYPDGTIFPKNATVQSQ	180
AEC15265.1	VHSNGQATHDNGAGKPVQGTSFHFFSVGGEALELQGVLFNYRTKYPDGTIFPKNATVQSQ	180
AEC15000.1	VHSNGQATHDNGAGKPVQGTSFHFFSVGGEALELQGVLFNYRTKYPDGTIFPKNATVQSQ	180
AEC14721.1	VHSNGQATHDNGAGKPVQGTSFHFFSVGGEALELQGVLFNYRTKYPDGTIFPKNATVQSQ	180
AEC14965.1	VHSNGQATHDNGAGKPVQGTSFHFFSVGGEALELQGVLFNYRTKYPDGTIFPKNATVQSQ	180
AEC14967.1	VHSNGQATHDNGAGKPVQGTSFHFFSVGGEALELQGVLFNYRTKYPDGTIFPKNATVQSQ	180
AEC14969.1	VHSNGQATHDNGAGKPVQGTSFHFFSVGGEALELQGVLFNYRTKYPDGTIFPKNATVQSQ	180
AEC14973.1	VHSNGQATHDNGAGKPVQGTSFHFFSVGGEALELQGVLFNYRTKYPDGTIFPKNATVQSQ	180
AEC14974.1	VHSNGQATHDNGAGKPVQGTSFHFFSVGGEALELQGVLFNYRTKYPDGTIFPKNATVQSQ	180
AEC14994.1	VHSNGQATHDNGAGKPVQGTSFHFFSVGGEALELQGVLFNYRTKYPDGTIFPKNATVQSQ	180
AEC15001.1	VHSNGQATHDNGAGKPVQGTSFHFFSVGGEALELQGVLFNYRTKYPDGTIFPKNATVQSQ	180
AEC15024.1	VHSNGQATHDNGAGKPVQGTSFHFFSVGGEALELQGVLFNYRTKYPDGTIFPKNATVQSQ	180
AEC14667.1	VHSNGQATHDNGAGKPVQGTSFHFFSVGGEALELQGVLFNYRTKYPDGTIFPKNATVQSQ	180
UPN63091.1	VHSNGQATHDNGAGKPVQGTSFHFFSVGGEALELQGVLFNYRTKYPDGTIFPKNATVQSQ	180
AEC14535.1	VHSNGQATHDNGAGKPVQGTSFHFFSVGGEALELQGVLFNYRTKYPDGTIFPKNATVQSQ	180
AEC14547.1	VHSNGQATHDNGAGKPVQGTSFHFFSVGGEALELQGVLFNYRTKYPDGTIFPKNATVQSQ	180
AEC14553.1	VHSNGQATHDNGAGKPVQGTSFHFFSVGGEALELQGVLFNYRTKYPDGTIFPKNATVQSQ	180
AEC14577.1	VHSNGQATHDNGAGKPVQGTSFHFFSVGGEALELQGVLFNYRTKYPDGTIFPKNATVQSQ	180
AEC14613.1	VHSNGQATHDNGAGKPVQGTSFHFFSVGGEALELQGVLFNYRTKYPDGTIFPKNATVQSQ	180
AEC14673.1	VHSNGQATHDNGAGKPVQGTSFHFFSVGGEALELQGVLFNYRTKYPDGTIFPKNATVQSQ	180
AEC14679.1	VHSNGQATHDNGAGKPVQGTSFHFFSVGGEALELQGVLFNYRTKYPDGTIFPKNATVQSQ	180
AEC14727.1	VHSNGQATHDNGAGKPVQGTSFHFFSVGGEALELQGVLFNYRTKYPDGTIFPKNATVQSQ	180
AEC14733.1	VHSNGQATHDNGAGKPVQGTSFHFFSVGGEALELQGVLFNYRTKYPDGTIFPKNATVQSQ	180
AEC14964.1	VHSNGQATHDNGAGKPVQGTSFHFFSVGGEALELQGVLFNYRTKYPDGTIFPKNATVQSQ	180
AEC14995.1	VHSNGQATHDNGAGKPVQGTSFHFFSVGGEALELQGVLFNYRTKYPDGTIFPKNATVQSQ	180
AEC15008.1	VHSNGQATHDNGAGKPVQGTSFHFFSVGGEALELQGVLFNYRTKYPDGTIFPKNATVQSQ	180
AEC14970.1	VHSNGQATHDNGAGKPVQGTSFHFFSVGGEALELQGVLFNYRTKYPDGTIFPKNATVQSQ	180
QTJ15162.1	VHSNGQATHDNGAGKPVQGTSFHFFSVGXXXXXLQGVVFNYRTKYPDGTIFPKNATVQSQ	180
UYL83831.1	VHSNGQATHDNGAGKPVQGTSFHFFSVGGEALELQGVVFNYRTKYPDGTIFPI---VQSQ	177
ALF06183.1	VHSNGQATHDNGAGKPVQGTSFHFFSVGGEALELQGVVFNYRTXYPDXTIFPKNATVQSQ	180
QCH41111.1	VHSNGQAAHDNGAGKPVQGTSFHFFSVGGEALELQGVVFNYRTKYPDGTIFPKNATVQSQ	180
QCH41112.1	VHSNGQAAHDNGAGKPVQGTSFHFFSVGGEALELQGVVFNYRTKYPDGTIFPKNATVQSQ	180
AEC14749.1	VHSNGQAAHDNGAGKPVQGTSFHFFSVGGEALELRGVVFNYRTKYPDGTIFPKNATVQSQ	180
ALF06202.1	VHSNGQAAHDNGAGKPVQGTSFHFFSVGGEALELQGVVFNYRTKYPDETIFPKNATVQSQ	180
ALF06186.1	VHSNGQAAHDNGAGKPVQGTSFHFFSVGGEALELQGVVFNYRTKYPDETIFPKNATVQSQ	180
ALF06208.1	VHSNGQAAHDNGAGKPVQGTSFHFFSVGGEALELQGVVFNYRTKYPDETIFPKNATVQSQ	180
ALF06197.1	VHSNGQAAHDNGAGKPVQGTSFHFFSVGGGALELQGVVFNYRTKYPDETIFPKNATVQSQ	180
AEC15037.1	VHSNGQAAHDNGAGKPVQGTSFHFFSVGGGALELQGVVFNYRTKYPDGTIFPKNATVQSQ	180
UPN63109.1	VHSNGQAAHDNGAGKPVQGTSFHFFSVGGEALELQGVVFNYRTKYPDGTIFPKNATVQSQ	180
UPN63107.1	VHSNGQAAHDNGAGKPVQGTSFHFFSVGGEALELQGVVFNYRTKYPDGTIFPKNATVQSQ	180
UPN63108.1	VHSNGQAAHDNGAGKPVQGTSFHFFSVGGEALELQGVVFNYRTKYPDGTIFPKNATVQSQ	180
QBQ04202.1	VHSNGQATHDNGAAKPVQGTSFHFFSVGGEALELQGVVFNYRTTYPDGTIFPKNATVQSQ	180
QBM11923.1	VHSNGQATHDNGAAKPVQGTSFHFFSVGGEALELQGVVFNYRTTYPDGTIFPKNATVQSQ	180
AAK70306.1	VHSNGQATHDNGAGKPVQGTSFHFFSVGGEALELQGVVFNYRTTYPDGTIFPKNATVQSQ	180
AAC59337.1	VHSNGQATHDNGAAKPVQGTSFHFFSVGGEALELQGVAFNYRTTYPDGTIFPKNATVQSQ	180
AAL37677.1	VHSNGQATHDNGAGKPVQGTSFHFFSVGGEALKLQGVVFNYRTKYPDGPIFPKNATGQSQ	180
AAK70276.1	VHSNGQATHDNGAAKPVQGTSFHFFSVGGEALELQGVVFNYRTTYPDGPIFPKNATVHSQ	180
BAC22792.1	VHSNGQATHDNGAGKPVQGTSFHFFSVGGEALELQGVVFNYRTKYPDGTIFPKNATVQSQ	180
BAC22798.1	VHSNGQATHDNGAGKPVQGTSFHFFSVGGEALELQGVVFNYRTKYPDGTIFPKNATVQSQ	180
BAC22804.1	VHSNGQATHDNGAGKPVQGTSFHFFSVGGEALELQGVVFNYRTKYPDGTIFPKNATVQSQ	180
BAC22810.1	VHSNGQATHDNGAGKPVQGTSFHFFSVGGEALELQGVVFNYRTKYPDGTIFPKNATVQSQ	180
BAC22858.1	VHSNGQATHDNGAGKPVQGTSFHFFSVGGEALELQGVVFNYRTKYPDGTIFPKNATVQSQ	180
BAC15701.1	VHSNGQATHDNGAGKPVQGTSFHFFSVGGEALELQGVVFNYRTKYPDGTIFPKNATVQSQ	180
BAB68863.1	VHSNGQATHDNGAGKPVQGTSFHFFSVGGEALELQGVVFNYRTKYPDGTIFPKNATVQSQ	180
BAC66424.1	VHSNGQATHDKGAGKPVQGTSFHFFSVGGEALELQGVVFNYRTKYPDGTIFPKNATVQSQ	180

```
BAD21235.2        VHSNGQATHDNGAGKPVQGTSFHFFSVGGEALELQGVVFNYRTKYPDGTIFPKNATVQSQ  180
QTJ15210.1        VHSNGQATHDNGAGKPVQGTSFHFFSVGGEALELQGVVFNYRTKYPDGTIFPKNATVQSQ  180
ASV51748.1        VHSNGQATHDNGAGKPVQGTSFHFFSVGGEALELQGVVFNYRTKYPDGTIFPKNATVQSQ  180
ASV51754.1        VHSNGQATHDNGAGKPVQGTSFHFFSVGGEALELQGVVFNYRTKYPDGTIFPKNATVQSQ  180
ASV51778.1        VHSNGQATHDNGAGKPVQGTSFHFFSVGGEALELQGVVFNYRTKYPDGTIFPKNATVQSQ  180
ASV51814.1        VHSNGQATHDNGAGKPVQGTSFHFFSVGGEALELQGVVFNYRTKYPDGTIFPKNATVQSQ  180
CCW72488.1        VHSNGQATHDNGAGKPVQGTSFHFFSVGGEALELQGVVFNYRTKYPDGTIFPKNATVQSQ  180
AEC15219.1        VHSNGQATHDNGAGKPVQGTSFHFFSVGGEALELQGVVFNYRTKYPDGTIFPKNATVQSQ  180
BAD21241.1        VHSNGQATHDNGAGKPVQGTSFHFFSVGGEALELQGVVFNYRTKYPDGTIFPKNATVQSQ  180
BAD21253.1        VHSNGQATHDNGAGKPVQGTSFHFFSVGGEALELQGVVFNYRTKYPDGTIFPKNATVQSQ  180
BAB93086.1        VHSNGQATHDNGAGKPVQGTSFHFFSVGGEALELQGVVFNYRTKYPDGTIFPKNATVQSQ  180
BAB68953.1        VHSNGQATHDNGAGKPVQGTSFHFFSVGGEALELQGVVFNYRTKYPDGTIFPKNATVQSQ  180
BAD98966.1        VHSNGQATHDNGAGKPVQGTSFHFFSVGGEALELQGVVFNYRTKYPDGTIFPKNATVQSQ  180
BAD98972.1        VHSNGQATHDNGAGKPVQGTSFHFFSVGGEALELQGVVFNYRTKYPDGTIFPKNATVQSQ  180
BAD91887.1        VHSNGQATHDNGAGKPVQGTSFHFFSVGGEALELQGVVFNYRTKYPDGTIFPKNATVQSQ  180
BAD27118.1        VHSNGQATHDNGAGKPVQGTSFHFFSVGGEALELQGVVFNYRTKYPDGTIFPKNATVQSQ  180
BAD06227.1        VHSNGQATHDNGAGKPVQGTSFHFFSVGGEALELQGVVFNYRTKYPDGTIFPKNATVQSQ  180
BAC66412.1        VHSNGQATHDNGAGKPVQGTSFHFFSVGGEALELQGVVFNYRTKYPDGTIFPKNATVQSQ  180
BAC66430.1        VHSNGQATHDNGAGKPVQGTSFHFFSVGGEALELQGVVFNYRTKYPDGTIFPKNATVQSQ  180
BAC22816.1        VHSNGQATHDNGAGKPVQGTSFHFFSVGGEALELQGVVFNYRTKYPDGTIFPKNATVQSQ  180
BAB93026.1        VHSNGQATHDNGAGKPVQGTSFHFFSVGGEALELQGVVFNYRTKYPDGTIFPKNATVQSQ  180
BAB93032.1        VHSNGQATHDNGAGKPVQGTSFHFFSVGGEALELQGVVFNYRTKYPDGTIFPKNATVQSQ  180
BAB93074.1        VHSNGQATHDNGAGKPVQGTSFHFFSVGGEALELQGVVFNYRTKYPDGTIFPKNATVQSQ  180
BAB93080.1        VHSNGQATHDNGAGKPVQGTSFHFFSVGGEALELQGVVFNYRTKYPDGTIFPKNATVQSQ  180
BAB68917.1        VHSNGQATHDNGAGKPVQGTSFHFFSVGGEALELQGVVFNYRTKYPDGTIFPKNATVQSQ  180
BAB68923.1        VHSNGQATHDNGAGKPVQGTSFHFFSVGGEALELQGVVFNYRTKYPDGTIFPKNATVQSQ  180
BAB68929.1        VHSNGQATHDNGAGKPVQGTSFHFFSVGGEALELQGVVFNYRTKYPDGTIFPKNATVQSQ  180
BAB68935.1        VHSNGQATHDNGAGKPVQGTSFHFFSVGGEALELQGVVFNYRTKYPDGTIFPKNATVQSQ  180
BAB68941.1        VHSNGQATHDNGAGKPVQGTSFHFFSVGGEALELQGVVFNYRTKYPDGTIFPKNATVQSQ  180
ALF06214.1        VHSNGQATHDNGAGKPVQGTSFHFFSVGGEALELQGVVFNYRTKYPDGTIFPKNATVQSQ  180
AAL37707.1        VHSNGQATHDNGAGKPVQGTSFHFFSVGGEALELQGVVFNYRTKYPDGTIFPKNATVQSQ  180
BBG22688.1        VHSNGQATHDNGAGKPVQGTSFHFFSVGGEALELQGVVFNYRTKYPDGTIFPKNATVQSQ  180
BAB11734.1        VHSNGQATHDNGAGKPVQGTSFHFFSVGGEALELQGVVFNYRTKYPDGTIFPKNATVQSQ  180
BAA05638.1        VHSNGQATHDNGAGKPVQGTSFHFFSVGGEALELQGVVFNYRTKYPDGTIFPKNATVQSQ  180
BAV69361.1        VHSNGQATHDNGAGKPVQGTSFHFFSVGGEALELQGVVFNYRTKYPDGTIFPKNATVQSQ  180
BAV69349.1        VHSNGQATHDNGAGKPVQGTSFHFFSVGGEALELQGVVFNYRTKYPDGTIFPKNATVQSQ  180
BAV69367.1        VHSNGQATHDNGAGKPVQGTSFHFFSVGGEALELQGVVFNYRTKYPDGTIFPKNATVQSQ  180
BAE00135.1        VHSNGQATHDNGAGKPVQGTSFHFFSVGGEALELQGVVFNYRTKYPDGTIFPKNATVQSQ  180
BAE00147.1        VHSNGQATHDNGAGKPVQGTSFHFFSVGGEALELQGVVFNYRTKYPDGTIFPKNATVQSQ  180
BAD11896.1        VHSNGQATHDNGAGKPVQGTSFHFFSVGGEALELQGVVFNYRTKYPDGTIFPKNATVQSQ  180
BAD06126.1        VHSNGQATHDNGAGKPVQGTSFHFFSVGGEALELQGVVFNYRTKYPDGTIFPKNATVQSQ  180
BAC81904.1        VHSNGQATHDNGAGKPVQGTSFHFFSVGGEALELQGVVFNYRTKYPDGTIFPKNATVQSQ  180
BAC81910.1        VHSNGQATHDNGAGKPVQGTSFHFFSVGGEALELQGVVFNYRTKYPDGTIFPKNATVQSQ  180
BAC81916.1        VHSNGQATHDNGAGKPVQGTSFHFFSVGGEALELQGVVFNYRTKYPDGTIFPKNATVQSQ  180
BAC81922.1        VHSNGQATHDNGAGKPVQGTSFHFFSVGGEALELQGVVFNYRTKYPDGTIFPKNATVQSQ  180
BAC81946.1        VHSNGQATHDNGAGKPVQGTSFHFFSVGGEALELQGVVFNYRTKYPDGTIFPKNATVQSQ  180
BAC81952.1        VHSNGQATHDNGAGKPVQGTSFHFFSVGGEALELQGVVFNYRTKYPDGTIFPKNATVQSQ  180
BAC81958.1        VHSNGQATHDNGAGKPVQGTSFHFFSVGGEALELQGVVFNYRTKYPDGTIFPKNATVQSQ  180
AAM89297.1        VHSNGQATHDNGAGKPVQGTSFHFFSVGGEALELQGVVFNYRTKYPDGTIFPKNATVQSQ  180
AAM89303.1        VHSNGQATHDNGAGKPVQGTSFHFFSVGGEALELQGVVFNYRTKYPDGTIFPKNATVQSQ  180
AAM89309.1        VHSNGQATHDNGAGKPVQGTSFHFFSVGGEALELQGVVFNYRTKYPDGTIFPKNATVQSQ  180
BAC66388.1        VHSNGQATHDNGAGKPVQGTSFHFFSVGGEALELQGVVFNYRTKYPDGTIFPKNATVQSQ  180
BAC66400.1        VHSNGQATHDNGAGKPVQGTSFHFFSVGGEALELQGVVFNYRTKYPDGTIFPKNATVQSQ  180
BAC66406.1        VHSNGQATHDNGAGKPVQGTSFHFFSVGGEALELQGVVFNYRTKYPDGTIFPKNATVQSQ  180
BAC22756.1        VHSNGQATHDNGAGKPVQGTSFHFFSVGGEALELQGVVFNYRTKYPDGTIFPKNATVQSQ  180
BAC22822.1        VHSNGQATHDNGAGKPVQGTSFHFFSVGGEALELQGVVFNYRTKYPDGTIFPKNATVQSQ  180
BAC22894.1        VHSNGQATHDNGAGKPVQGTSFHFFSVGGEALELQGVVFNYRTKYPDGTIFPKNATVQSQ  180
BAC22900.1        VHSNGQATHDNGAGKPVQGTSFHFFSVGGEALELQGVVFNYRTKYPDGTIFPKNATVQSQ  180
BAC15635.1        VHSNGQATHDNGAGKPVQGTSFHFFSVGGEALELQGVVFNYRTKYPDGTIFPKNATVQSQ  180
BAC15653.1        VHSNGQATHDNGAGKPVQGTSFHFFSVGGEALELQGVVFNYRTKYPDGTIFPKNATVQSQ  180
BAC15665.1        VHSNGQATHDNGAGKPVQGTSFHFFSVGGEALELQGVVFNYRTKYPDGTIFPKNATVQSQ  180
BAC03065.1        VHSNGQATHDNGAGKPVQGTSFHFFSVGGEALELQGVVFNYRTKYPDGTIFPKNATVQSQ  180
AAL37689.1        VHSNGQATHDNGAGKPVQGTSFHFFSVGGEALELQGVVFNYRTKYPDGTIFPKNATVQSQ  180
AAL37701.1        VHSNGQATHDNGAGKPVQGTSFHFFSVGGEALELQGVVFNYRTKYPDGTIFPKNATVQSQ  180
```

BAB68803.1	VHSNGQATHDNGAGKPVQGTSFHFFSVGGEALELQGVVFNYRTKYPDGTIFPKNATVQSQ	180
BAB68965.1	VHSNGQATHDNGAGKPVQGTSFHFFSVGGEALELQGVVFNYRTKYPDGTIFPKNATVQSQ	180
BAB68971.1	VHSNGQATHDNGAGKPVQGTSFHFFSVGGEALELQGVVFNYRTKYPDGTIFPKNATVQSQ	180
AAK97964.1	VHSNGQATHDNGAGKPVQGTSFHFFSVGGEALELQGVVFNYRTKYPDGTIFPKNATVQSQ	180
AAK97970.1	VHSNGQATHDNGAGKPVQGTSFHFFSVGGEALELQGVVFNYRTKYPDGTIFPKNATVQSQ	180
AAC40841.1	VHSNGQATHDNGAGKPVQGTSFHFFSVGGEALELQGVVFNYRTKYPDGTIFPKNATVQSQ	180
BAA05636.1	VHSNGQATHDNGAGKPVQGTSFHFFSVGGEALELQGVVFNYRTKYPDGTIFPKNATVQSQ	180
BAA01970.1	VHSNGQATHDNGAGKPVQGTSFHFFSVGGEALELQGVVFNYRTKYPDGTIFPKNATVQSQ	180
BAD06144.1	VHSNGQATHDNGAGKPVQGTSFHFFSVGGEALELQGVVFNYRTKYPDGTIFPKNATVQSQ	180
BAC81852.1	VHSNGQATHDNGAGKPVQGTSFHFFSVGGEALELQGVVFNYRTKYPDGTIFPKNATVQSQ	180
BAC81858.1	VHSNGQATHDNGAGKPVQGTSFHFFSVGGEALELQGVVFNYRTKYPDGTIFPKNATVQSQ	180
BAC22864.1	VHSNGQATHDNGAGKPVQGTSFHFFSVGGEALELQGVVFNYRTKYPDGTIFPKNATVQSQ	180
AAM89315.1	VHSNGQATHDNGAGKPVQGTSFHFFSVGGEALELQGVVFNYRTKYPDGTIFPKNATVQSQ	180
BAC66382.1	VHSNGQATHDNGAGKPVQGTSFHFFSVGGEALELQGVVFNYRTTYPDGTIFPKNATVQSQ	180
BAC66394.1	VHSNGQATHDNGAGKPVQGTSFHFFSVGGEALELQGVVFNYRTTYPDGTIFPKNATVQSQ	180
BAC66418.1	VHSNGQATHDNGAGKPVQGTSFHFFSVGGEALELQGVVFNYRTTYPDGTIFPKNATVQSQ	180
BAC22828.1	VHSNGQATHDNGAGKPVQGTSFHFFSVGGEALELQGVVFNYRTTYPDGTIFPKNATVQSQ	180
BAC22846.1	VHSNGQATHDNGAGKPVQGTSFHFFSVGGEALELQGVVFNYRTTYPDGTIFPKNATVQSQ	180
AAM69590.1	VHSNGQATHDNGAGKPVQGTSFHFFSVGGEALELQGVVFNYRTTYPDGTIFPKNATVQSQ	180
BAB93014.1	VHSNGQATHDNGAGKPVQGTSFHFFSVGGEALELQGVVFNYRTTYPDGTIFPKNATVQSQ	180
BAB93020.1	VHSNGQATHDNGAGKPVQGTSFHFFSVGGEALELQGVVFNYRTTYPDGTIFPKNATVQSQ	180
BAB93092.1	VHSNGQATHDNGAGKPVQGTSFHFFSVGGEALELQGVVFNYRTTYPDGTIFPKNATVQSQ	180
BAB93098.1	VHSNGQATHDNGAGKPVQGTSFHFFSVGGEALELQGVVFNYRTTYPDGTIFPKNATVQSQ	180
AAL37665.1	VHSNGQATHDNGAGKPVQGTSFHFFSVGGEALELQGVVFNYRTTYPDGTIFPKNATVQSQ	180
BAB68773.1	VHSNGQATHDNGAGKPVQGTSFHFFSVGGEALELQGVVFNYRTTYPDGTIFPKNATVQSQ	180
BAB68779.1	VHSNGQATHDNGAGKPVQGTSFHFFSVGGEALELQGVVFNYRTTYPDGTIFPKNATVQSQ	180
AAK28460.1	VHSNGQATHDNGAGKPVQGTSFHFFSVGGEALELQGVVFNYRTTYPDGTIFPKNATVQSQ	180
AAK28466.1	VHSNGQATHDNGAGKPVQGTSFHFFSVGGEALELQGVVFNYRTTYPDGTIFPKNATVQSQ	180
BAB11716.1	VHSNGQATHDNGAGKPVQGTSFHFFSVGGEALELQGVVFNYRTTYPDGTIFPKNATVQSQ	180
BAB11722.1	VHSNGQATHDNGAGKPVQGTSFHFFSVGGEALELQGVVFNYRTTYPDGTIFPKNATVQSQ	180
AAK97976.1	VHSNGQATHDNGAGKPVQGTSFHFFSVGGEALELQGVVFNYRTMYPDGTIFPKNATVQSQ	180
BAC15641.1	VHSNGQATHDNGAGKPVQGTSFHFFSVGGEALELQGVVFNYRTKYPDGTIFPKNATVQSQ	180
BAC15647.1	VHSNGQATHDNGAGKPVQGTSFHFFSVGGEALELQGVVFNYRTKYPDGTIFPKNATVQSQ	180
AAL37647.1	VHSNGQATHDNGAGKPVQGTSFHFFSVGGEALELQGVVFNYRTKYPDGTIFPKNATVQSQ	180
BAB68797.1	VHSNGQATHDNGAGKPVQGTSFHFFSVGGEALELQGVVFNYRTKYPDGTIFPKNATVQSQ	180
AAG37198.1	VHSNGQATHDNGAGKPVQGTSFHFFSVGGEALELQGVVFNYRTKYPDGTIFPKNATVQSQ	180
ALF06241.1	VHSNGQATHDNGAGKPVQGTSFHFFSVGGEALELQGVVFNYRTKYPDGTIFPKNATVQSQ	180
AFE56117.1	VHSNGQATHDNGAGKPVQGTSFHFFSVGGEALELQGVVFNYRTKYPDGTIFPKNATVQSQ	180
BAF95600.1	VHSNGQATHDNGAGKPVQGTSFHFFSVGGEALELQGVVFNYRTKYPDGTIFPKNATVQSQ	180
BAD11836.1	VHSNGQATHDNGAGKPVQGTSFHFFSVGGEALELQGVVFNYRTKYPDGTIFPKNATVQSQ	180
BAD11842.1	VHSNGQATHDNGAGKPVQGTSFHFFSVGGEALELQGVVFNYRTKYPDGTIFPKNATVQSQ	180
BAD11848.1	VHSNGQATHDNGAGKPVQGTSFHFFSVGGEALELQGVVFNYRTKYPDGTIFPKNATVQSQ	180
AAM89327.1	VHSNGQATHDNGAGKPVQGTSFHFFSVGGEALELQGVVFNYRTKYPDGTIFPKNATVQSQ	180
AAM89339.1	VHSNGQATHDNGAGKPVQGTSFHFFSVGGEALELQGVVFNYRTKYPDGTIFPKNATVQSQ	180
BAC15707.1	VHSNGQATHDNGAGKPVQGTSFHFFSVGGEALELQGVLFNYRTKYPDGTIFPKNATVQSQ	180
BAB68833.1	VHSNGQATHDNGAGKPVQGTSFHFFSVGGEALELQGVLFNYRTKYPDGTIFPKNATVQSQ	180
BAB68839.1	VHSNGQATHDNGAGKPVQGTSFHFFSVGGEALELQGVLFNYRTKYPDGTIFPKNATVQSQ	180
AYM55604.1	VHSNGQATHDNGAGKPVQGTSFHFFSVGGEALELQGVVFNYRTKYPDGTIFPKNATVQSQ	180
BAV69355.1	VHSNGQATHDNGAGKPVQGTSFHFFSVGGEALELQGVVFNYRTKYPDGTIFPKNATVQSQ	180
ALF06220.1	VHSNGQATHDNGAGKPVQGTSFHFFSVGGEALELQGVVFNYRTKYPDGTIFPKNATVQSQ	180
ALF06247.1	VHSNGQATHDNGAGKPVQGTSFHFFSVGGEALELQGVVFNYRTKYPDGTIFPKNATVQSQ	180
ALF06250.1	VHSNGQATHDNGAGKPVQGTSFHFFSVGGEALELQGVVFNYRTKYPDGTIFPKNATVQSQ	180
ALF06253.1	VHSNGQATHDNGAGKPVQGTSFHFFSVGGEALELQGVVFNYRTKYPDGTIFPKNATVQSQ	180
ALF06259.1	VHSNGQATHDNGAGKPVQGTSFHFFSVGGEALELQGVVFNYRTKYPDGTIFPKNATVQSQ	180
ALF06265.1	VHSNGQATHDNGAGKPVQGTSFHFFSVGGEALELQGVVFNYRTKYPDGTIFPKNATVQSQ	180
CCW72502.1	VHSNGQATHDNGAGKPVQGTSFHFFSVGGEALELQGVVFNYRTKYPDGTIFPKNATVQSQ	180
BAF40757.1	VHSNGQATHDNGAGKPVQGTSFHFFSVGGEALELQGVVFNYRTKYPDGTIFPKNATVQSQ	180
BAF40763.1	VHSNGQATHDNGAGKPVQGTSFHFFSVGGEALELQGVVFNYRTKYPDGTIFPKNATVQSQ	180
BAF40769.1	VHSNGQATHDNGAGKPVQGTSFHFFSVGGEALELQGVVFNYRTKYPDGTIFPKNATVQSQ	180
BAF40775.1	VHSNGQATHDNGAGKPVQGTSFHFFSVGGEALELQGVVFNYRTKYPDGTIFPKNATVQSQ	180
BAE94732.1	VHSNGQATHDNGAGKPVQGTSFHFFSVGGEALELQGVVFNYRTKYPDGTIFPKNATVQSQ	180
BAE94738.1	VHSNGQATHDNGAGKPVQGTSFHFFSVGGEALELQGVVFNYRTKYPDGTIFPKNATVQSQ	180
BAE00129.1	VHSNGQATHDNGAGKPVQGTSFHFFSVGGEALELQGVVFNYRTKYPDGTIFPKNATVQSQ	180
BAE00141.1	VHSNGQATHDNGAGKPVQGTSFHFFSVGGEALELQGVVFNYRTKYPDGTIFPKNATVQSQ	180

BAD11824.1	VHSNGQATHDNGAGKPVQGTSFHFFSVGGEALELQGVVFNYRTKYPDGTIFPKNATVQSQ	180
BAD11830.1	VHSNGQATHDNGAGKPVQGTSFHFFSVGGEALELQGVVFNYRTKYPDGTIFPKNATVQSQ	180
BAD11854.1	VHSNGQATHDNGAGKPVQGTSFHFFSVGGEALELQGVVFNYRTKYPDGTIFPKNATVQSQ	180
BAD11860.1	VHSNGQATHDNGAGKPVQGTSFHFFSVGGEALELQGVVFNYRTKYPDGTIFPKNATVQSQ	180
BAD11866.1	VHSNGQATHDNGAGKPVQGTSFHFFSVGGEALELQGVVFNYRTKYPDGTIFPKNATVQSQ	180
BAD11872.1	VHSNGQATHDNGAGKPVQGTSFHFFSVGGEALELQGVVFNYRTKYPDGTIFPKNATVQSQ	180
BAD11878.1	VHSNGQATHDNGAGKPVQGTSFHFFSVGGEALELQGVVFNYRTKYPDGTIFPKNATVQSQ	180
BAD11884.1	VHSNGQATHDNGAGKPVQGTSFHFFSVGGEALELQGVVFNYRTKYPDGTIFPKNATVQSQ	180
BAD11890.1	VHSNGQATHDNGAGKPVQGTSFHFFSVGGEALELQGVVFNYRTKYPDGTIFPKNATVQSQ	180
BAD11902.1	VHSNGQATHDNGAGKPVQGTSFHFFSVGGEALELQGVVFNYRTKYPDGTIFPKNATVQSQ	180
BAD06233.1	VHSNGQATHDNGAGKPVQGTSFHFFSVGGEALELQGVVFNYRTKYPDGTIFPKNATVQSQ	180
BAD06066.1	VHSNGQATHDNGAGKPVQGTSFHFFSVGGEALELQGVVFNYRTKYPDGTIFPKNATVQSQ	180
BAD06078.1	VHSNGQATHDNGAGKPVQGTSFHFFSVGGEALELQGVVFNYRTKYPDGTIFPKNATVQSQ	180
BAD06114.1	VHSNGQATHDNGAGKPVQGTSFHFFSVGGEALELQGVVFNYRTKYPDGTIFPKNATVQSQ	180
BAD06132.1	VHSNGQATHDNGAGKPVQGTSFHFFSVGGEALELQGVVFNYRTKYPDGTIFPKNATVQSQ	180
BAD06138.1	VHSNGQATHDNGAGKPVQGTSFHFFSVGGEALELQGVVFNYRTKYPDGTIFPKNATVQSQ	180
BAD06162.1	VHSNGQATHDNGAGKPVQGTSFHFFSVGGEALELQGVVFNYRTKYPDGTIFPKNATVQSQ	180
BAD06168.1	VHSNGQATHDNGAGKPVQGTSFHFFSVGGEALELQGVVFNYRTKYPDGTIFPKNATVQSQ	180
BAC82365.1	VHSNGQATHDNGAGKPVQGTSFHFFSVGGEALELQGVVFNYRTKYPDGTIFPKNATVQSQ	180
BAC81846.1	VHSNGQATHDNGAGKPVQGTSFHFFSVGGEALELQGVVFNYRTKYPDGTIFPKNATVQSQ	180
BAC81864.1	VHSNGQATHDNGAGKPVQGTSFHFFSVGGEALELQGVVFNYRTKYPDGTIFPKNATVQSQ	180
BAC81870.1	VHSNGQATHDNGAGKPVQGTSFHFFSVGGEALELQGVVFNYRTKYPDGTIFPKNATVQSQ	180
BAC81874.1	VHSNGQATHDNGAGKPVQGTSFHFFSVGGEALELQGVVFNYRTKYPDGTIFPKNATVQSQ	180
BAC81880.1	VHSNGQATHDNGAGKPVQGTSFHFFSVGGEALELQGVVFNYRTKYPDGTIFPKNATVQSQ	180
BAC81886.1	VHSNGQATHDNGAGKPVQGTSFHFFSVGGEALELQGVVFNYRTKYPDGTIFPKNATVQSQ	180
BAC81892.1	VHSNGQATHDNGAGKPVQGTSFHFFSVGGEALELQGVVFNYRTKYPDGTIFPKNATVQSQ	180
BAC81898.1	VHSNGQATHDNGAGKPVQGTSFHFFSVGGEALELQGVVFNYRTKYPDGTIFPKNATVQSQ	180
BAC81928.1	VHSNGQATHDNGAGKPVQGTSFHFFSVGGEALELQGVVFNYRTKYPDGTIFPKNATVQSQ	180
BAC81934.1	VHSNGQATHDNGAGKPVQGTSFHFFSVGGEALELQGVVFNYRTKYPDGTIFPKNATVQSQ	180
BAC81940.1	VHSNGQATHDNGAGKPVQGTSFHFFSVGGEALELQGVVFNYRTKYPDGTIFPKNATVQSQ	180
BAC81964.1	VHSNGQATHDNGAGKPVQGTSFHFFSVGGEALELQGVVFNYRTKYPDGTIFPKNATVQSQ	180
AAM89321.1	VHSNGQATHDNGAGKPVQGTSFHFFSVGGEALELQGVVFNYRTKYPDGTIFPKNATVQSQ	180
AAM89333.1	VHSNGQATHDNGAGKPVQGTSFHFFSVGGEALELQGVVFNYRTKYPDGTIFPKNATVQSQ	180
AAM89345.1	VHSNGQATHDNGAGKPVQGTSFHFFSVGGEALELQGVVFNYRTKYPDGTIFPKNATVQSQ	180
BAC66376.1	VHSNGQATHDNGAGKPVQGTSFHFFSVGGEALELQGVVFNYRTKYPDGTIFPKNATVQSQ	180
BAC22834.1	VHSNGQATHDNGAGKPVQGTSFHFFSVGGEALELQGVVFNYRTKYPDGTIFPKNATVQSQ	180
BAC22840.1	VHSNGQATHDNGAGKPVQGTSFHFFSVGGEALELQGVVFNYRTKYPDGTIFPKNATVQSQ	180
BAC22852.1	VHSNGQATHDNGAGKPVQGTSFHFFSVGGEALELQGVVFNYRTKYPDGTIFPKNATVQSQ	180
BAC22870.1	VHSNGQATHDNGAGKPVQGTSFHFFSVGGEALELQGVVFNYRTKYPDGTIFPKNATVQSQ	180
BAC22876.1	VHSNGQATHDNGAGKPVQGTSFHFFSVGGEALELQGVVFNYRTKYPDGTIFPKNATVQSQ	180
BAC22882.1	VHSNGQATHDNGAGKPVQGTSFHFFSVGGEALELQGVVFNYRTKYPDGTIFPKNATVQSQ	180
BAC22888.1	VHSNGQATHDNGAGKPVQGTSFHFFSVGGEALELQGVVFNYRTKYPDGTIFPKNATVQSQ	180
BAC15629.1	VHSNGQATHDNGAGKPVQGTSFHFFSVGGEALELQGVVFNYRTKYPDGTIFPKNATVQSQ	180
BAC15659.1	VHSNGQATHDNGAGKPVQGTSFHFFSVGGEALELQGVVFNYRTKYPDGTIFPKNATVQSQ	180
BAC15671.1	VHSNGQATHDNGAGKPVQGTSFHFFSVGGEALELQGVVFNYRTKYPDGTIFPKNATVQSQ	180
BAC15677.1	VHSNGQATHDNGAGKPVQGTSFHFFSVGGEALELQGVVFNYRTKYPDGTIFPKNATVQSQ	180
BAC15689.1	VHSNGQATHDNGAGKPVQGTSFHFFSVGGEALELQGVVFNYRTKYPDGTIFPKNATVQSQ	180
BAC15713.1	VHSNGQATHDNGAGKPVQGTSFHFFSVGGEALELQGVVFNYRTKYPDGTIFPKNATVQSQ	180
BAC15731.1	VHSNGQATHDNGAGKPVQGTSFHFFSVGGEALELQGVVFNYRTKYPDGTIFPKNATVQSQ	180
BAC15737.1	VHSNGQATHDNGAGKPVQGTSFHFFSVGGEALELQGVVFNYRTKYPDGTIFPKNATVQSQ	180
BAC15743.1	VHSNGQATHDNGAGKPVQGTSFHFFSVGGEALELQGVVFNYRTKYPDGTIFPKNATVQSQ	180
AAL37641.1	VHSNGQATHDNGAGKPVQGTSFHFFSVGGEALELQGVVFNYRTKYPDGTIFPKNATVQSQ	180
AAL37653.1	VHSNGQATHDNGAGKPVQGTSFHFFSVGGEALELQGVVFNYRTKYPDGTIFPKNATVQSQ	180
AAL37659.1	VHSNGQATHDNGAGKPVQGTSFHFFSVGGEALELQGVVFNYRTKYPDGTIFPKNATVQSQ	180
AAL37671.1	VHSNGQATHDNGAGKPVQGTSFHFFSVGGEALELQGVVFNYRTKYPDGTIFPKNATVQSQ	180
AAL37683.1	VHSNGQATHDNGAGKPVQGTSFHFFSVGGEALELQGVVFNYRTKYPDGTIFPKNATVQSQ	180
BAB68791.1	VHSNGQATHDNGAGKPVQGTSFHFFSVGGEALELQGVVFNYRTKYPDGTIFPKNATVQSQ	180
BAB68821.1	VHSNGQATHDNGAGKPVQGTSFHFFSVGGEALELQGVVFNYRTKYPDGTIFPKNATVQSQ	180
BAB68845.1	VHSNGQATHDNGAGKPVQGTSFHFFSVGGEALELQGVVFNYRTKYPDGTIFPKNATVQSQ	180
BAB68851.1	VHSNGQATHDNGAGKPVQGTSFHFFSVGGEALELQGVVFNYRTKYPDGTIFPKNATVQSQ	180
BAB68857.1	VHSNGQATHDNGAGKPVQGTSFHFFSVGGEALELQGVVFNYRTKYPDGTIFPKNATVQSQ	180
BAB68869.1	VHSNGQATHDNGAGKPVQGTSFHFFSVGGEALELQGVVFNYRTKYPDGTIFPKNATVQSQ	180
BAB68875.1	VHSNGQATHDNGAGKPVQGTSFHFFSVGGEALELQGVVFNYRTKYPDGTIFPKNATVQSQ	180
AAK97910.1	VHSNGQATHDNGAGKPVQGTSFHFFSVGGEALELQGVVFNYRTKYPDGTIFPKNATVQSQ	180

AAK97916.1	VHSNGQATHDNGAGKPVQGTSFHFFSVGGEALELQGVVFNYRTKYPDGTIFPKNATVQSQ	180
AAK97922.1	VHSNGQATHDNGAGKPVQGTSFHFFSVGGEALELQGVVFNYRTKYPDGTIFPKNATVQSQ	180
AAK97928.1	VHSNGQATHDNGAGKPVQGTSFHFFSVGGEALELQGVVFNYRTKYPDGTIFPKNATVQSQ	180
AAK97934.1	VHSNGQATHDNGAGKPVQGTSFHFFSVGGEALELQGVVFNYRTKYPDGTIFPKNATVQSQ	180
AAK97940.1	VHSNGQATHDNGAGKPVQGTSFHFFSVGGEALELQGVVFNYRTKYPDGTIFPKNATVQSQ	180
AAK97946.1	VHSNGQATHDNGAGKPVQGTSFHFFSVGGEALELQGVVFNYRTKYPDGTIFPKNATVQSQ	180
AAK97952.1	VHSNGQATHDNGAGKPVQGTSFHFFSVGGEALELQGVVFNYRTKYPDGTIFPKNATVQSQ	180
AAK97958.1	VHSNGQATHDNGAGKPVQGTSFHFFSVGGEALELQGVVFNYRTKYPDGTIFPKNATVQSQ	180
AAK97982.1	VHSNGQATHDNGAGKPVQGTSFHFFSVGGEALELQGVVFNYRTKYPDGTIFPKNATVQSQ	180
AAK97988.1	VHSNGQATHDNGAGKPVQGTSFHFFSVGGEALELQGVVFNYRTKYPDGTIFPKNATVQSQ	180
AAK97994.1	VHSNGQATHDNGAGKPVQGTSFHFFSVGGEALELQGVVFNYRTKYPDGTIFPKNATVQSQ	180
BAB11698.1	VHSNGQATHDNGAGKPVQGTSFHFFSVGGEALELQGVVFNYRTKYPDGTIFPKNATVQSQ	180
BAB11704.1	VHSNGQATHDNGAGKPVQGTSFHFFSVGGEALELQGVVFNYRTKYPDGTIFPKNATVQSQ	180
AAC59478.1	VHSNGQATHDNGAGKPVQGTSFHFFSVGGEALELQGVVFNYRTKYPDGTIFPKNATVQSQ	180
AAC59484.1	VHSNGQATHDNGAGKPVQGTSFHFFSVGGEALELQGVVFNYRTKYPDGTIFPKNATVQSQ	180
AAC59490.1	VHSNGQATHDNGAGKPVQGTSFHFFSVGGEALELQGVVFNYRTKYPDGTIFPKNATVQSQ	180
BAA01963.1	VHSNGQATHDNGAGKPVQGTSFHFFSVGGEALELQGVVFNYRTKYPDGTIFPKNATVQSQ	180
BAA01964.1	VHSNGQATHDNGAGKPVQGTSFHFFSVGGEALELQGVVFNYRTKYPDGTIFPKNATVQSQ	180
BAB93068.1	VHSNGQATHDNGAGKPVQGTSFHFFSVGGEALELQGVVFNYRTKYPDGTIFPKNATVQSQ	180
ASV51856.1	VHSNGQATHDNGAGKPVQGTSFHFFSVGGEALELQGVVFNYRTKYPDGTIFPKNATVQSQ	180
ANH21097.1	VHSNGQATHDNGAGKPVQGTSFHFFSVGGEALELQGVVFNYRTKYPDGTIFPKNATVQSQ	180
AEC15167.1	VHSNGQATHDNGAGKPVQGTSFHFFSVGGEALELQGVVFNYRTKYPDGTIFPKNATVQSQ	180
AEC15168.1	VHSNGQATHDNGAGKPVQGTSFHFFSVGGEALELQGVVFNYRTKYPDGTIFPKNATVQSQ	180
AEC15169.1	VHSNGQATHDNGAGKPVQGTSFHFFSVGGEALELQGVVFNYRTKYPDGTIFPKNATVQSQ	180
QTJ15168.1	VHSNGQAAHDNGAGKPVQGTSFHFFSVGGEALELQGVVFNYRTKYPDGTIFPKNATVQSQ	180
AEC15173.1	VHSNGQAAHDNGAGKPVQGTSFHFFSVGGEALELQGVVFNYRTKYPDGTIFPKNATVQSQ	180
AEC15174.1	VHSNGQAAHDNGAGKPVQGTSFHFFSVGGEALELQGVVFNYRTKYPDGTIFPKNATVQSQ	180
AEC15175.1	VHSNGQAAHDNGAGKPVQGTSFHFFSVGGEALELQGVVFNYRTKYPDGTIFPKNATVQSQ	180
AEC15176.1	VHSNGQAAHDNGAGKPVQGTSFHFFSVGGEALELQGVVFNYRTKYPDGTIFPKNATVQSQ	180
AEC15177.1	VHSNGQAAHDNGAGKPVQGTSFHFFSVGGEALELQGVVFNYRTKYPDGTIFPKNATVQSQ	180
AEC15178.1	VHSNGQAAHDNGAGKPVQGTSFHFFSVGGEALELQGVVFNYRTKYPDGTIFPKNATVQSQ	180
AEC15179.1	VHSNGQAAHDNGAGKPVQGTSFHFFSVGGEALELQGVVFNYRTKYPDGTIFPKNATVQSQ	180
AEC15180.1	VHSNGQAAHDNGAGKPVQGTSFHFFSVGGEALELQGVVFNYRTKYPDGTIFPKNATVQSQ	180
AEC15181.1	VHSNGQAAHDNGAGKPVQGTSFHFFSVGGEALELQGVVFNYRTKYPDGTIFPKNATVQSQ	180
AEC15182.1	VHSNGQAAHDNGAGKPVQGTSFHFFSVGGEALELQGVVFNYRTKYPDGTIFPKNATVQSQ	180
AEC15183.1	VHSNGQAAHDNGAGKPVQGTSFHFFSVGGEALELQGVVFNYRTKYPDGTIFPKNATVQSQ	180
AEC15184.1	VHSNGQAAHDNGAGKPVQGTSFHFFSVGGEALELQGVVFNYRTKYPDGTIFPKNATVQSQ	180
AEC15185.1	VHSNGQAAHDNGAGKPVQGTSFHFFSVGGEALELQGVVFNYRTKYPDGTIFPKNATVQSQ	180
AEC15187.1	VHSNGQAAHDNGAGKPVQGTSFHFFSVGGEALELQGVVFNYRTKYPDGTIFPKNATVQSQ	180
AEC15188.1	VHSNGQAAHDNGAGKPVQGTSFHFFSVGGEALELQGVVFNYRTKYPDGTIFPKNATVQSQ	180
AEC15189.1	VHSNGQAAHDNGAGKPVQGTSFHFFSVGGEALELQGVVFNYRTKYPDGTIFPKNATVQSQ	180
AEC15190.1	VHSNGQAAHDNGAGKPVQGTSFHFFSVGGEALELQGVVFNYRTKYPDGTIFPKNATVQSQ	180
AEC15191.1	VHSNGQAAHDNGAGKPVQGTSFHFFSVGGEALELQGVVFNYRTKYPDGTIFPKNATVQSQ	180
AEC15193.1	VHSNGQAAHDNGAGKPVQGTSFHFFSVGGEALELQGVVFNYRTKYPDGTIFPKNATVQSQ	180
AEC14871.1	VHSNGQAAHDNGAGKSVQGTSFHFFSVGGEALELQGVVFNYRTKYPDGTIFPKNATVQSQ	180
AEC15222.1	VHSNGQAAHDNGAGKSVQGTSFHFFSVGGEALELQGVVFNYRTKYPDGTIFPKNATVQSQ	180
AEC15130.1	VHSNGQAAHDNGAGKPVQGTSFHFFSVGGEALELQGVVFNYRTKYPDGTIFPKNATVQSQ	180
AEC15144.1	VHSNGQAAHDNGAGKPVQGTSFHFFSVGGEALELQGVVFNYRTKYPDGTIFPKNATVQSQ	180
AEC15154.1	VHSNGQAAHDNGAGKPVQGTSFHFFSVGGEALELQGVVFNYRTKYPDGTIFPKNATVQSQ	180
AEC15157.1	VHSNGQAAHDNGAGKPVQGTSFHFFSVGGEALELQGVVFNYRTKYPDGTIFPKNATVQSQ	180
AEC15160.1	VHSNGQAAHDNGAGKPVQGTSFHFFSVGGEALELQGVVFNYRTKYPDGTIFPKNATVQSQ	180
AEC15231.1	VHSNGQAAHDNGAGKPVQGTSFHFFSVGGEALELQGVVFNYRTKYPDGTIFPKNATVQSQ	180
ALF06191.1	VHSNGQAAHDNGAGKPVQGTSFHFFSVGGEALELQGVVFNYRTKYPDETIFPKNATVQSQ	180
WLD48112.1	VHSNGQAAHDNGAGKPVQGTSFHFFSVGGEALELQGVVFNYRTKYPDGTIFPKNATVQSQ	180
QWT77140.1	VHSNGQAAHDNGAGKPVQGTSFHFFSVGGEALELQGVVFNYRTKYPDGTIFPKNATVQSQ	180
QTJ15174.1	VHSNGQAAHDNGAGKPVQGTSFHFFSVGGEALELQGVVFNYRTKYPDGTIFPKNATVQSQ	180
QTJ15186.1	VHSNGQAAHDNGAGKPVQGTSFHFFSVGGEALELQGVVFNYRTKYPDGTIFPKNATVQSQ	180
QTJ15192.1	VHSNGQAAHDNGAGKPVQGTSFHFFSVGGEALELQGVVFNYRTKYPDGTIFPKNATVQSQ	180
QTJ15198.1	VHSNGQAAHDNGAGKPVQGTSFHFFSVGGEALELQGVVFNYRTKYPDGTIFPKNATVQSQ	180
ASV51772.1	VHSNGQAAHDNGAGKPVQGTSFHFFSVGGEALELQGVVFNYRTKYPDGTIFPKNATVQSQ	180
CCW72478.1	VHSNGQAAHDNGAGKPVQGTSFHFFSVGGEALELQGVVFNYRTKYPDGTIFPKNATVQSQ	180
CCW72481.1	VHSNGQAAHDNGAGKPVQGTSFHFFSVGGEALELQGVVFNYRTKYPDGTIFPKNATVQSQ	180
CCW72487.1	VHSNGQAAHDNGAGKPVQGTSFHFFSVGGEALELQGVVFNYRTKYPDGTIFPKNATVQSQ	180
CCW72492.1	VHSNGQAAHDNGAGKPVQGTSFHFFSVGGEALELQGVVFNYRTKYPDGTIFPKNATVQSQ	180

CCW72496.1	VHSNGQAAHDNGAGKPVQGTSFHFFSVGGEALELQGVVFNYRTKYPDGTIFPKNATVQSQ	180
CCW72499.1	VHSNGQAAHDNGAGKPVQGTSFHFFSVGGEALELQGVVFNYRTKYPDGTIFPKNATVQSQ	180
CCW72504.1	VHSNGQAAHDNGAGKPVQGTSFHFFSVGGEALELQGVVFNYRTKYPDGTIFPKNATVQSQ	180
CCW72508.1	VHSNGQAAHDNGAGKPVQGTSFHFFSVGGEALELQGVVFNYRTKYPDGTIFPKNATVQSQ	180
CCW72510.1	VHSNGQAAHDNGAGKPVQGTSFHFFSVGGEALELQGVVFNYRTKYPDGTIFPKNATVQSQ	180
CCW72519.1	VHSNGQAAHDNGAGKPVQGTSFHFFSVGGEALELQGVVFNYRTKYPDGTIFPKNATVQSQ	180
CCW72521.1	VHSNGQAAHDNGAGKPVQGTSFHFFSVGGEALELQGVVFNYRTKYPDGTIFPKNATVQSQ	180
AEC14864.1	VHSNGQAAHDNGAGKPVQGTSFHFFSVGGEALELQGVVFNYRTKYPDGTIFPKNATVQSQ	180
AEC14865.1	VHSNGQAAHDNGAGKPVQGTSFHFFSVGGEALELQGVVFNYRTKYPDGTIFPKNATVQSQ	180
AEC14866.1	VHSNGQAAHDNGAGKPVQGTSFHFFSVGGEALELQGVVFNYRTKYPDGTIFPKNATVQSQ	180
AEC14867.1	VHSNGQAAHDNGAGKPVQGTSFHFFSVGGEALELQGVVFNYRTKYPDGTIFPKNATVQSQ	180
AEC14868.1	VHSNGQAAHDNGAGKPVQGTSFHFFSVGGEALELQGVVFNYRTKYPDGTIFPKNATVQSQ	180
AEC14869.1	VHSNGQAAHDNGAGKPVQGTSFHFFSVGGEALELQGVVFNYRTKYPDGTIFPKNATVQSQ	180
AEC14870.1	VHSNGQAAHDNGAGKPVQGTSFHFFSVGGEALELQGVVFNYRTKYPDGTIFPKNATVQSQ	180
AEC14872.1	VHSNGQAAHDNGAGKPVQGTSFHFFSVGGEALELQGVVFNYRTKYPDGTIFPKNATVQSQ	180
AEC14873.1	VHSNGQAAHDNGAGKPVQGTSFHFFSVGGEALELQGVVFNYRTKYPDGTIFPKNATVQSQ	180
AEC14874.1	VHSNGQAAHDNGAGKPVQGTSFHFFSVGGEALELQGVVFNYRTKYPDGTIFPKNATVQSQ	180
AEC14875.1	VHSNGQAAHDNGAGKPVQGTSFHFFSVGGEALELQGVVFNYRTKYPDGTIFPKNATVQSQ	180
AEC14876.1	VHSNGQAAHDNGAGKPVQGTSFHFFSVGGEALELQGVVFNYRTKYPDGTIFPKNATVQSQ	180
AEC14877.1	VHSNGQAAHDNGAGKPVQGTSFHFFSVGGEALELQGVVFNYRTKYPDGTIFPKNATVQSQ	180
AEC14878.1	VHSNGQAAHDNGAGKPVQGTSFHFFSVGGEALELQGVVFNYRTKYPDGTIFPKNATVQSQ	180
AEC14879.1	VHSNGQAAHDNGAGKPVQGTSFHFFSVGGEALELQGVVFNYRTKYPDGTIFPKNATVQSQ	180
AEC14880.1	VHSNGQAAHDNGAGKPVQGTSFHFFSVGGEALELQGVVFNYRTKYPDGTIFPKNATVQSQ	180
AEC14881.1	VHSNGQAAHDNGAGKPVQGTSFHFFSVGGEALELQGVVFNYRTKYPDGTIFPKNATVQSQ	180
AEC14882.1	VHSNGQAAHDNGAGKPVQGTSFHFFSVGGEALELQGVVFNYRTKYPDGTIFPKNATVQSQ	180
AEC14883.1	VHSNGQAAHDNGAGKPVQGTSFHFFSVGGEALELQGVVFNYRTKYPDGTIFPKNATVQSQ	180
AEC14884.1	VHSNGQAAHDNGAGKPVQGTSFHFFSVGGEALELQGVVFNYRTKYPDGTIFPKNATVQSQ	180
AEC14885.1	VHSNGQAAHDNGAGKPVQGTSFHFFSVGGEALELQGVVFNYRTKYPDGTIFPKNATVQSQ	180
AEC14886.1	VHSNGQAAHDNGAGKPVQGTSFHFFSVGGEALELQGVVFNYRTKYPDGTIFPKNATVQSQ	180
AEC14887.1	VHSNGQAAHDNGAGKPVQGTSFHFFSVGGEALELQGVVFNYRTKYPDGTIFPKNATVQSQ	180
AEC14888.1	VHSNGQAAHDNGAGKPVQGTSFHFFSVGGEALELQGVVFNYRTKYPDGTIFPKNATVQSQ	180
AEC14889.1	VHSNGQAAHDNGAGKPVQGTSFHFFSVGGEALELQGVVFNYRTKYPDGTIFPKNATVQSQ	180
AEC14890.1	VHSNGQAAHDNGAGKPVQGTSFHFFSVGGEALELQGVVFNYRTKYPDGTIFPKNATVQSQ	180
AEC14891.1	VHSNGQAAHDNGAGKPVQGTSFHFFSVGGEALELQGVVFNYRTKYPDGTIFPKNATVQSQ	180
AEC14892.1	VHSNGQAAHDNGAGKPVQGTSFHFFSVGGEALELQGVVFNYRTKYPDGTIFPKNATVQSQ	180
AEC14894.1	VHSNGQAAHDNGAGKPVQGTSFHFFSVGGEALELQGVVFNYRTKYPDGTIFPKNATVQSQ	180
AEC14895.1	VHSNGQAAHDNGAGKPVQGTSFHFFSVGGEALELQGVVFNYRTKYPDGTIFPKNATVQSQ	180
AEC14896.1	VHSNGQAAHDNGAGKPVQGTSFHFFSVGGEALELQGVVFNYRTKYPDGTIFPKNATVQSQ	180
AEC14897.1	VHSNGQAAHDNGAGKPVQGTSFHFFSVGGEALELQGVVFNYRTKYPDGTIFPKNATVQSQ	180
AEC14898.1	VHSNGQAAHDNGAGKPVQGTSFHFFSVGGEALELQGVVFNYRTKYPDGTIFPKNATVQSQ	180
AEC14899.1	VHSNGQAAHDNGAGKPVQGTSFHFFSVGGEALELQGVVFNYRTKYPDGTIFPKNATVQSQ	180
AEC14900.1	VHSNGQAAHDNGAGKPVQGTSFHFFSVGGEALELQGVVFNYRTKYPDGTIFPKNATVQSQ	180
AEC14901.1	VHSNGQAAHDNGAGKPVQGTSFHFFSVGGEALELQGVVFNYRTKYPDGTIFPKNATVQSQ	180
AEC14902.1	VHSNGQAAHDNGAGKPVQGTSFHFFSVGGEALELQGVVFNYRTKYPDGTIFPKNATVQSQ	180
AEC14903.1	VHSNGQAAHDNGAGKPVQGTSFHFFSVGGEALELQGVVFNYRTKYPDGTIFPKNATVQSQ	180
AEC14904.1	VHSNGQAAHDNGAGKPVQGTSFHFFSVGGEALELQGVVFNYRTKYPDGTIFPKNATVQSQ	180
AEC14905.1	VHSNGQAAHDNGAGKPVQGTSFHFFSVGGEALELQGVVFNYRTKYPDGTIFPKNATVQSQ	180
AEC14907.1	VHSNGQAAHDNGAGKPVQGTSFHFFSVGGEALELQGVVFNYRTKYPDGTIFPKNATVQSQ	180
AEC14908.1	VHSNGQAAHDNGAGKPVQGTSFHFFSVGGEALELQGVVFNYRTKYPDGTIFPKNATVQSQ	180
AEC14909.1	VHSNGQAAHDNGAGKPVQGTSFHFFSVGGEALELQGVVFNYRTKYPDGTIFPKNATVQSQ	180
AEC14910.1	VHSNGQAAHDNGAGKPVQGTSFHFFSVGGEALELQGVVFNYRTKYPDGTIFPKNATVQSQ	180
AEC14911.1	VHSNGQAAHDNGAGKPVQGTSFHFFSVGGEALELQGVVFNYRTKYPDGTIFPKNATVQSQ	180
AEC15164.1	VHSNGQAAHDNGAGKPVQGTSFHFFSVGGEALELQGVVFNYRTKYPDGTIFPKNATVQSQ	180
BAF95594.1	VHSNGQAAHDNGAGKPVQGTSFHFFSVGGEALELQGVVFNYRTKYPDGTIFPKNATVQSQ	180
BAF95606.1	VHSNGQAAHDNGAGKPVQGTSFHFFSVGGEALELQGVVFNYRTKYPDGTIFPKNATVQSQ	180
BAF40745.1	VHSNGQAAHDNGAGKPVQGTSFHFFSVGGEALELQGVVFNYRTKYPDGTIFPKNATVQSQ	180
BAF40751.1	VHSNGQAAHDNGAGKPVQGTSFHFFSVGGEALELQGVVFNYRTKYPDGTIFPKNATVQSQ	180
BAF40781.1	VHSNGQAAHDNGAGKPVQGTSFHFFSVGGEALELQGVVFNYRTKYPDGTIFPKNATVQSQ	180
BAF40787.1	VHSNGQAAHDNGAGKPVQGTSFHFFSVGGEALELQGVVFNYRTKYPDGTIFPKNATVQSQ	180
BAF40793.1	VHSNGQAAHDNGAGKPVQGTSFHFFSVGGEALELQGVVFNYRTKYPDGTIFPKNATVQSQ	180
BAF40799.1	VHSNGQAAHDNGAGKPVQGTSFHFFSVGGEALELQGVVFNYRTKYPDGTIFPKNATVQSQ	180
BAF40811.1	VHSNGQAAHDNGAGKPVQGTSFHFFSVGGEALELQGVVFNYRTKYPDGTIFPKNATVQSQ	180
BAF40817.1	VHSNGQAAHDNGAGKPVQGTSFHFFSVGGEALELQGVVFNYRTKYPDGTIFPKNATVQSQ	180
BAF40823.1	VHSNGQAAHDNGAGKPVQGTSFHFFSVGGEALELQGVVFNYRTKYPDGTIFPKNATVQSQ	180

```
BAF40829.1      VHSNGQAAHDNGAGKPVQGTSFHFFSVGGEALELQGVVFNYRTKYPDGTIFPKNATVQSQ  180
BAF40835.1      VHSNGQAAHDNGAGKPVQGTSFHFFSVGGEALELQGVVFNYRTKYPDGTIFPKNATVQSQ  180
BAF40841.1      VHSNGQAAHDNGAGKPVQGTSFHFFSVGGEALELQGVVFNYRTKYPDGTIFPKNATVQSQ  180
BAF40847.1      VHSNGQAAHDNGAGKPVQGTSFHFFSVGGEALELQGVVFNYRTKYPDGTIFPKNATVQSQ  180
BAC81834.1      VHSNGQAAHDNGAGKPVQGTSFHFFSVGGEALELQGVVFNYRTKYPDGTIFPKNATVQSQ  180
BAC81840.1      VHSNGQAAHDNGAGKPVQGTSFHFFSVGGEALELQGVVFNYRTKYPDGTIFPKNATVQSQ  180
AAN85461.1      VHSNGQAAHDNGAGKPVQGTSFHFFSVGGEALELQGVVFNYRTKYPDGTIFPKNATVQSQ  180
AAN85467.1      VHSNGQAAHDNGAGKPVQGTSFHFFSVGGEALELQGVVFNYRTKYPDGTIFPKNATVQSQ  180
AAN85473.1      VHSNGQAAHDNGAGKPVQGTSFHFFSVGGEALELQGVVFNYRTKYPDGTIFPKNATVQSQ  180
BAC22762.1      VHSNGQAAHDNGAGKPVQGTSFHFFSVGGEALELQGVVFNYRTKYPDGTIFPKNATVQSQ  180
BAC22768.1      VHSNGQAAHDNGAGKPVQGTSFHFFSVGGEALELQGVVFNYRTKYPDGTIFPKNATVQSQ  180
BAC22774.1      VHSNGQAAHDNGAGKPVQGTSFHFFSVGGEALELQGVVFNYRTKYPDGTIFPKNATVQSQ  180
BAC22780.1      VHSNGQAAHDNGAGKPVQGTSFHFFSVGGEALELQGVVFNYRTKYPDGTIFPKNATVQSQ  180
BAC22786.1      VHSNGQAAHDNGAGKPVQGTSFHFFSVGGEALELQGVVFNYRTKYPDGTIFPKNATVQSQ  180
BAB68809.1      VHSNGQAAHDNGAGKPVQGTSFHFFSVGGEALELQGVVFNYRTKYPDGTIFPKNATVQSQ  180
BAB68815.1      VHSNGQAAHDNGAGKPVQGTSFHFFSVGGEALELQGVVFNYRTKYPDGTIFPKNATVQSQ  180
BAB68827.1      VHSNGQAAHDNGAGKPVQGTSFHFFSVGGEALELQGVVFNYRTKYPDGTIFPKNATVQSQ  180
AAC59496.1      VHSNGQAAHDNGAGKPVQGTSFHFFSVGGEALELQGVVFNYRTKYPDGTIFPKNATVQSQ  180
AAC59502.1      VHSNGQAAHDNGAGKPVQGTSFHFFSVGGEALELQGVVFNYRTKYPDGTIFPKNATVQSQ  180
AAC59508.1      VHSNGQAAHDNGAGKPVQGTSFHFFSVGGEALELQGVVFNYRTKYPDGTIFPKNATVQSQ  180
AAC59514.1      VHSNGQAAHDNGAGKPVQGTSFHFFSVGGEALELQGVVFNYRTKYPDGTIFPKNATVQSQ  180
AAC59520.1      VHSNGQAAHDNGAGKPVQGTSFHFFSVGGEALELQGVVFNYRTKYPDGTIFPKNATVQSQ  180
AAB62680.1      VHSNGQAAHDNGAGKPVQGTSFHFFSVGGEALELQGVVFNYRTKYPDGTIFPKNATVQSQ  180
BAA01961.1      VHSNGQAAHDNGAGKPVQGTSFHFFSVGGEALELQGVVFNYRTKYPDGTIFPKNATVQSQ  180
WLD48082.1      VHSNGQAAHDNGAGKPVQGTSFHFFSVGGEALELQGVVFNYRTKYPDGTIFPKNATVQSQ  180
WLD48088.1      VHSNGQAAHDNGAGKPVQGTSFHFFSVGGEALELQGVVFNYRTKYPDGTIFPKNATVQSQ  180
CCW72516.1      VHSNGQAAHDNGAGKPVQGTSFHFFSVGGEALELQGVVFNYRTKYPDGTIFPKNATVQSQ  180
CCW72523.1      VHSNGQAAHDNGAGKPVQGTSFHFFSVGGEALELQGVVFNYRTKYPDGTIFPKNATVQSQ  180
AEC14783.1      VHSNGQAAHDNGAGKPVQGTSFHFFSVGGEALELQGVVFNYRTKYPDGTIFPKNATVQSQ  180
AEC14784.1      VHSNGQAAHDNGAGKPVQGTSFHFFSVGGEALELQGVVFNYRTKYPDGTIFPKNATVQSQ  180
AEC14785.1      VHSNGQAAHDNGAGKPVQGTSFHFFSVGGEALELQGVVFNYRTKYPDGTIFPKNATVQSQ  180
AEC14786.1      VHSNGQAAHDNGAGKPVQGTSFHFFSVGGEALELQGVVFNYRTKYPDGTIFPKNATVQSQ  180
AEC14787.1      VHSNGQAAHDNGAGKPVQGTSFHFFSVGGEALELQGVVFNYRTKYPDGTIFPKNATVQSQ  180
AAB62687.1      VHSNGQAAHDNGAGKPVQGTSFHFFSVGGEALELQGVVFNYRTKYPDGTIFPKNATVQSQ  180
AEC14893.1      VHSNGQAAHDNGAGKPVQGTSFHFFSVGGEALELQGVVFNYGTKYPDGTIFPKNATVQSQ  180
AEC14906.1      VHSNGQAAHDNGAGKPVQGTSFHFFSVGGEALELQGVVFNYRTKYPDGTIFPKNATVQSQ  180
AEC15118.1      VHSNGQAAHDNGAGKPVQGTSFHFFSVGGEALELQGVVFNYRTKYPDGTIFPKNATVQSQ  180
AEC15120.1      VHSNGQAAHDNGAGKPVQGTSFHFFSVGGEALELQGVVFNYRTKYPDGTIFPKNATVQSQ  180
AEC15121.1      VHSNGQAAHDNGAGKPVQGTSFHFFSVGGEALELQGVVFNYRTKYPDGTIFPKNATVQSQ  180
AEC15122.1      VHSNGQAAHDNGAGKPVQGTSFHFFSVGGEALELQGVVFNYRTKYPDGTIFPKNATVQSQ  180
AEC15124.1      VHSNGQAAHDNGAGKPVQGTSFHFFSVGGEALELQGVVFNYRTKYPDGTIFPKNATVQSQ  180
AEC15125.1      VHSNGQAAHDNGAGKPVQGTSFHFFSVGGEALELQGVVFNYRTKYPDGTIFPKNATVQSQ  180
AEC15127.1      VHSNGQAAHDNGAGKPVQGTSFHFFSVGGEALELQGVVFNYRTKYPDGTIFPKNATVQSQ  180
AEC15129.1      VHSNGQAAHDNGAGKPVQGTSFHFFSVGGEALELQGVVFNYRTKYPDGTIFPKNATVQSQ  180
AEC15131.1      VHSNGQAAHDNGAGKPVQGTSFHFFSVGGEALELQGVVFNYRTKYPDGTIFPKNATVQSQ  180
AEC15133.1      VHSNGQAAHDNGAGKPVQGTSFHFFSVGGEALELQGVVFNYRTKYPDGTIFPKNATVQSQ  180
AEC15135.1      VHSNGQAAHDNGAGKPVQGTSFHFFSVGGEALELQGVVFNYRTKYPDGTIFPKNATVQSQ  180
AEC15136.1      VHSNGQAAHDNGAGKPVQGTSFHFFSVGGEALELQGVVFNYRTKYPDGTIFPKNATVQSQ  180
AEC15137.1      VHSNGQAAHDNGAGKPVQGTSFHFFSVGGEALELQGVVFNYRTKYPDGTIFPKNATVQSQ  180
AEC15138.1      VHSNGQAAHDNGAGKPVQGTSFHFFSVGGEALELQGVVFNYRTKYPDGTIFPKNATVQSQ  180
AEC15139.1      VHSNGQAAHDNGAGKPVQGTSFHFFSVGGEALELQGVVFNYRTKYPDGTIFPKNATVQSQ  180
AEC15141.1      VHSNGQAAHDNGAGKPVQGTSFHFFSVGGEALELQGVVFNYRTKYPDGTIFPKNATVQSQ  180
AEC15142.1      VHSNGQAAHDNGAGKPVQGTSFHFFSVGGEALELQGVVFNYRTKYPDGTIFPKNATVQSQ  180
AEC15143.1      VHSNGQAAHDNGAGKPVQGTSFHFFSVGGEALELQGVVFNYRTKYPDGTIFPKNATVQSQ  180
AEC15145.1      VHSNGQAAHDNGAGKPVQGTSFHFFSVGGEALELQGVVFNYRTKYPDGTIFPKNATVQSQ  180
AEC15146.1      VHSNGQAAHDNGAGKPVQGTSFHFFSVGGEALELQGVVFNYRTKYPDGTIFPKNATVQSQ  180
AEC15147.1      VHSNGQAAHDNGAGKPVQGTSFHFFSVGGEALELQGVVFNYRTKYPDGTIFPKNATVQSQ  180
AEC15148.1      VHSNGQAAHDNGAGKPVQGTSFHFFSVGGEALELQGVVFNYRTKYPDGTIFPKNATVQSQ  180
AEC15149.1      VHSNGQAAHDNGAGKPVQGTSFHFFSVGGEALELQGVVFNYRTKYPDGTIFPKNATVQSQ  180
AEC15150.1      VHSNGQAAHDNGAGKPVQGTSFHFFSVGGEALELQGVVFNYRTKYPDGTIFPKNATVQSQ  180
AEC15151.1      VHSNGQAAHDNGAGKPVQGTSFHFFSVGGEALELQGVVFNYRTKYPDGTIFPKNATVQSQ  180
AEC15152.1      VHSNGQAAHDNGAGKPVQGTSFHFFSVGGEALELQGVVFNYRTKYPDGTIFPKNATVQSQ  180
AEC15153.1      VHSNGQAAHDNGAGKPVQGTSFHFFSVGGEALELQGVVFNYRTKYPDGTIFPKNATVQSQ  180
AEC15155.1      VHSNGQAAHDNGAGKPVQGTSFHFFSVGGEALELQGVVFNYRTKYPDGTIFPKNATVQSQ  180
```

AEC15156.1	VHSNGQAAHDNGAGKPVQGTSFHFFSVGGEALELQGVVFNYRTKYPDGTIFPKNATVQSQ	180
AEC15158.1	VHSNGQAAHDNGAGKPVQGTSFHFFSVGGEALELQGVVFNYRTKYPDGTIFPKNATVQSQ	180
AEC15159.1	VHSNGQAAHDNGAGKPVQGTSFHFFSVGGEALELQGVVFNYRTKYPDGTIFPKNATVQSQ	180
AEC15161.1	VHSNGQAAHDNGAGKPVQGTSFHFFSVGGEALELQGVVFNYRTKYPDGTIFPKNATVQSQ	180
AEC15162.1	VHSNGQAAHDNGAGKPVQGTSFHFFSVGGEALELQGVVFNYRTKYPDGTIFPKNATVQSQ	180
AEC15163.1	VHSNGQAAHDNGAGKPVQGTSFHFFSVGGEALELQGVVFNYRTKYPDGTIFPKNATVQSQ	180
AEC15226.1	VHSNGQAAHDNGAGKPVQGTSFHFFSVGGEALELQGVVFNYRTKYPDGTIFPKNATVQSQ	180
AEC15227.1	VHSNGQAAHDNGAGKPVQGTSFHFFSVGGEALELQGVVFNYRTKYPDGTIFPKNATVQSQ	180
AEC15228.1	VHSNGQAAHDNGAGKPVQGTSFHFFSVGGEALELQGVVFNYRTKYPDGTIFPKNATVQSQ	180
AEC15229.1	VHSNGQAAHDNGAGKPVQGTSFHFFSVGGEALELQGVVFNYRTKYPDGTIFPKNATVQSQ	180
AEC15230.1	VHSNGQAAHDNGAGKPVQGTSFHFFSVGGEALELQGVVFNYRTKYPDGTIFPKNATVQSQ	180
AEC15236.1	VHSNGQAAHDNGAGKPVQGTSFHFFSVGGEALELQGVVFNYRTKYPDGTIFPKNATVQSQ	180
AEC15237.1	VHSNGQAAHDNGAGKPVQGTSFHFFSVGGEALELQGVVFNYRTKYPDGTIFPKNATVQSQ	180
AEC15238.1	VHSNGQAAHDNGAGKPVQGTSFHFFSVGGEALELQGVVFNYRTKYPDGTIFPKNATVQSQ	180
AEC15239.1	VHSNGQAAHDNGAGKPVQGTSFHFFSVGGEALELQGVVFNYRTKYPDGTIFPKNATVQSQ	180
AEC15240.1	VHSNGQAAHDNGAGKPVQGTSFHFFSVGGEALELQGVVFNYRTKYPDGTIFPKNATVQSQ	180
AEC15447.1	VHSNGQAAHDNGAGKPVQGTSFHFFSVGGEALELQGVVFNYRTKYPDGTIFPKNATVQSQ	180
AEC15453.1	VHSNGQAAHDNGAGKPVQGTSFHFFSVGGEALELQGVVFNYRTKYPDGTIFPKNATVQSQ	180
AEC15459.1	VHSNGQAAHDNGAGKPVQGTSFHFFSVGGEALELQGVVFNYRTKYPDGTIFPKNATVQSQ	180
AEC15465.1	VHSNGQAAHDNGAGKPVQGTSFHFFSVGGEALELQGVVFNYRTKYPDGTIFPKNATVQSQ	180
AEC15471.1	VHSNGQAAHDNGAGKPVQGTSFHFFSVGGEALELQGVVFNYRTKYPDGTIFPKNATVQSQ	180
AEC15477.1	VHSNGQAAHDNGAGKPVQGTSFHFFSVGGEALELQGVVFNYRTKYPDGTIFPKNATVQSQ	180
AEC15483.1	VHSNGQAAHDNGAGKPVQGTSFHFFSVGGEALELQGVVFNYRTKYPDGTIFPKNATVQSQ	180
AEC15489.1	VHSNGQAAHDNGAGKPVQGTSFHFFSVGGEALELQGVVFNYRTKYPDGTIFPKNATVQSQ	180
AEC15543.1	VHSNGQAAHDNGAGKPVQGTSFHFFSVGGEALELQGVVFNYRTKYPDGTIFPKNATVQSQ	180
AEC15119.1	VHSNGQAAHDNGAGKPVQGTSFHFFSVGGEALELQGVVFNYRTKYPDGTIFPKNATVQSQ	180
AEC15123.1	VHSNGQAAHDNGAGKPVQGTSFHFFSVGGEALELQGVVFNYRTKYPDGTIFPKNATVQSQ	180
AEC15126.1	VHSNGQAAHDNGAGKPVQGTSFHFFSVGGEALELQGVVFNYRTKYPDGTIFPKNATVQSQ	180
AEC15132.1	VHSNGQAAHDNGAGKPVQGTSFHFFSVGGEALELQGVVFNYRTKYPDGTIFPKNATVQSQ	180
AEC15134.1	VHSNGQAAHDNGAGKPVQGTSFHFFSVGGEALELQGVVFNYRTKYPDGTIFPKNATVQSQ	180
AEC15140.1	VHSNGQAAHDNGAGKPVQGTSFHFFSVGGEALELQGVVFNYRTKYPDGTIFPKNATVQSQ	180
AEC15221.1	VHSNGQAAHDNGAGKPVQGTSFHFFSVGGEALELQGVVFNYRTKYPDGTIFPKNATVQSQ	180
AEC15224.1	VHSNGQAAHDNGAGKPVQGTSFHFFSVGGEALELQGVVFNYRTKYPDGTIFPKNATVQSQ	180
AEC15225.1	VHSNGQAAHDNGAGKPVQGTSFHFFSVGGEALELQGVVFNYRTKYPDGTIFPKNATVQSQ	180
AEC15233.1	VHSNGQAAHDNGAGKPVQGTSFHFFSVGGEALELQGVVFNYRTKYPDGTIFPKNATVQSQ	180
AEC15234.1	VHSNGQAAHDNGAGKPVQGTSFHFFSVGGEALELQGVVFNYRTKYPDGTIFPKNATVQSQ	180
AEC15235.1	VHSNGQAAHDNGAGKPVQGTSFHFFSVGGEALELQGVVFNYRTKYPDGTIFPKNATVQSQ	180
AEC15241.1	VHSNGQAAHDNGAGKPVQGTSFHFFSVGGEALELQGVVFNYRTKYPDGTIFPKNATVQSQ	180
AEC15220.1	VHSNGQAAHDNGAGKPVQGTSFHFFSVGGEALELQGVVFNYRTKYPDGTIFPKNATVQSQ	180
AEC15223.1	VHSNGQAAHDNGAGKPVQGTSFHFFSVGGEALELQGVVFNYRTKYPDGTIFPKNATVQSQ	180
AEC15232.1	VHSNGQAAHDNGAGKPVQGTSFHFFSVGGEALELQGVVFNYRTKYPDGTIFPKNATVQSQ	180
AEC15128.1	VHSNGQAAHDNGAGKPVQGTSFHFFSVGGEALELQGVVFNYRTKYPDGTIFPKNATVQSQ	180
AEC15495.1	VHSNGQAAHDNGAGKPVQGTSFHFFSVGGEALELQGVVFNYRTKYPDGTIFPKNATVQSQ	180
AEC15501.1	VHSNGQAAHDNGAGKPVQGTSFHFFSVGGEALELQGVVFNYRTKYPDGTIFPKNATVQSQ	180
AEC15507.1	VHSNGQAAHDNGAGKPVQGTSFHFFSVGGEALELQGVVFNYRTKYPDGTIFPKNATVQSQ	180
AEC15513.1	VHSNGQAAHDNGAGKPVQGTSFHFFSVGGEALELQGVVFNYRTKYPDGTIFPKNATVQSQ	180
AEC15519.1	VHSNGQAAHDNGAGKPVQGTSFHFFSVGGEALELQGVVFNYRTKYPDGTIFPKNATVQSQ	180
AEC15525.1	VHSNGQAAHDNGAGKPVQGTSFHFFSVGGEALELQGVVFNYRTKYPDGTIFPKNATVQSQ	180
AEC15531.1	VHSNGQAAHDNGAGKPVQGTSFHFFSVGGEALELQGVVFNYRTKYPDGTIFPKNATVQSQ	180
AEC15537.1	VHSNGQAAHDNGAGKPVQGTSFHFFSVGGEALELQGVVFNYRTKYPDGTIFPKNATVQSQ	180
BAF40805.1	VHSNGQAAHDNGAGKPVQGTSFHFFSVGGEALELQGVVFNYRTKYPDGTIFPKNATVQSQ	180
AAT09843.1	VHSNGQAAHDNGAGKPVQGTSFHFFSVGGEALELQGVVFNYRTKYPDGTIFPKNATVQSQ	180
BAA01965.1	VHSNGQAAHDNGAGKPVQGTSFHFFSVGGEALELQGVVFNYRTKYPDGTIFPKNATVQSQ	180
AIW00320.1	VHSNGQAAHDNGAGKPVQGTSFHFFSVGGEALELQGVVFNYRTKYPDGTIFPKNATVQSQ	180
ALF06194.1	VHSNGQAAHDNGAGKPVQGTSFHFFSVGGEALELQGVVFNYRTKYPDETIFPKNATVQSQ	180
ALF06211.1	VHSNGQAAHDNGAGKPVQGTSFHFFSVGGEALELQGVVFNYRTKYPDETIFPKNATVQSQ	180
AEC14821.1	VHSNGQAAHDNGAGKPVQGTSFHFFSVGGEALELQGVVFNYRTKYPDGTIFPKNATVQSQ	180
AEC14860.1	VHSNGQAAHDNGAGKPVQGTSFHFFSVGGEALELQGVVFNYRTKYPDGTIFPKNATVQSQ	180
AEC14861.1	VHSNGQAAHDNGAGKPVQGTSFHFFSVGGEALELQGVVFNYRTKYPDGTIFPKNATVQSQ	180
AEC14811.1	VHSNGQAAHDNGAGKPVQGTSFHFFSVGGEALELQGVVFNYRTKYPDGTIFPKNATVQSQ	180
AEC14806.1	VHSNGQAAHDNGAGKPVQGTSFHFFSVGGEALELQGVVFNYRTKYPDGTIFPKNATVQSQ	180
AEC14795.1	VHSNGQAAHDNGAGKPVQGTSFHFFSVGGEALELQGVVFNYRTKYPDGTIFPKNATVQSQ	180
AEC14765.1	VHSNGQAAHDNGAGKPVQGTSFHFFSVGGEALELQGVVFNYRAKYPDGTIFPKNATVQSQ	180
AEC14774.1	VHSNGQAAHDNGAGKPVQGTSFHFFSVGGEALELQGVVFNYRAKYPDGTIFPKNATVQSQ	180

AEC14761.1	VHSNGQAAHDNGAGKLVQGTSFHFFSVGGEALELQGVVFNYRTTYPDGTIFPKNATVQSQ	180
AEC14753.1	VHSNGQAAHDNGAGKPVQGTSFHFFSVGGEALELQGVVFNYRTKYPDGTIFPKNATVQSQ	180
AEC14767.1	VHSNGQAAHDNGAGKPVQGTSFHFFSVGGEALELQGVVFNYRTKYPDGTIFPKNATAQSQ	180
AEC14780.1	VHSNGQAAHDNGAGKPVRGTSFHFFSVGGEALELQGVVFNYRTKYPDGTIFPKNATAQSQ	180
AEC14760.1	VHSNGQAAHDNGAGKPVQGTSFHFFSVGGEALELQGVVFNYRTKYPDGTIFPKNATVQSQ	180
AEC14762.1	VHSNGQAAHDNGAGKPVQGTSFHFFSVGGEALELQGVVFNYRTKYPDGTIFPKNATVQSQ	180
AEC14742.1	VHSNGQAAHDNGAGKPVQGTSFHFFSVGGEALELQGVVFNYRTRYPDGTIFPKNATVQSQ	180
AEC14763.1	VHSNGQAAHDNGAGKPVQGTSFHFFSVGGEALELQGVVFNYRTKYPDGTIFPKNATVQSQ	180
AEC14810.1	VHSNGQAAHDNGAGKPVQGTSFHFFSVGGEALELQGVVFNYRTKYPDGTIFPKNATVQSQ	180
AEC14803.1	VHSNGQAAHDNGAGKPVQGTSFHFFSVGGEALELQGVVFNYRTKYPDGTIFPKNATVQSQ	180
AEC14802.1	VHSNGQAAHDNGAGKPVQGTSFHFFSVGGEALELQGVVFNYRTKYPDGTIFPKNATVQSQ	180
AEC14812.1	VHSNGQAAHDNGAGKPVQGTSFHFFSVGGEALELQGVVFNYRTKYPDGTIFPKNATVQSQ	180
AEC14804.1	VHSNGQAAHDNGAGKPVQGTSFHFFSVGGEALELQGVVFNYRTKYPDGTIFPKNATVQSQ	180
AEC14797.1	VHSNGQAAHDNGAGKPVQGTSFHFFSVGGEALELQGVVFNYRTKYPDGTIFPKNATVQSQ	180
AEC14796.1	VHSNGQAAHDNGAGKPVQGTSFHFFSVGGEALELQGVVFNYRTKYPDGTIFPKNATVQSQ	180
AEC14793.1	VHSNGQAAHDNGAGKPVQGTSFHFFSVGGEALELQGVVFNYRTKYPDGTIFPKNATVQSQ	180
AEC14791.1	VHSNGQAAHDNGAGKPVQGTSFHFFSVGGEALELQGVVFNYRTKYPDGTIFPKNATVQSQ	180
AEC14794.1	VHSNGQAAHDNGAGKPVQGTSFHFFSVGGEALELQGVVFNYRTKYPDGTIFPKNATVQSQ	180
AEC14789.1	VHSNGQAAHGNGAGKPVQGTSFHFFSVGGEALELQGVVFNYRTKYPDGTIFPKNATVQSQ	180
AEC14743.1	VHSNGQAAHDNGAGKPVQGTSFHFFSVGGEALELQGVVCNYRTKYPDGTIFPKNATVQSQ	180
AEC14745.1	VHSNGQAAHDNGAGKPVQGTSFHFFSVGGEALELQGVVFNYRTKYPDGTIFPKNATVQSQ	180
AEC14750.1	VHSNGQAAHDNGAGKPVQGTSFHFFSVGGEALELQGVVFNYRTKYPDGTIFPKNATVQSQ	180
AEC14754.1	VHSNGQAAHDNGAGKPVQGTSFHFFSVGGEALELQGVVFNYRTKYPDGTIFPKNATVQSQ	180
AEC14751.1	VHSNGQAAHDNGAGKPVQGTSFHFFSVGGEALELQGVVFNYRTKYPDGTIFPKNATVQSQ	180
AEC14776.1	VHSNGQAAHDNGAGKPVQGTSFHFFSVGGEALELQGVVFNYRTKYPDGTIFPKNATVQSQ	180
AEC14755.1	VHSNGQAAHDDGAGKPVQGTSFHFFSVGGEALELQGVVFNYRTKYPDGTIFPKNATVQSQ	180
AEC14759.1	VHSNGQAAHDNGAGKPVQGTSFHFFSVGGEALELQGVVFNYRTKYPDGTIFPKNATVQSQ	180
AEC14766.1	VHSNGQAAHGNGAGKPVQGTSFHFFSVGGEALELQGVVFNYRTKYPDGTIFPKNATVQSQ	180
AEC14781.1	VHSNGQAAHDNGAGKPVQGTSFHFFSVGGEALELQGVVFNYRTKYPDGTIFPKNATVQSQ	180
AEC15186.1	VHSNGQAAHDNGAGKPVQGTSFHFFSVGGEALELQGVVFNYRTKYPDGTIFPKNATVQSQ	180
AEC15192.1	VHSNGQAAHDNGAGKPVQGTSFHFFSVGGEALELQGVVFNYRTKYPDGTIFPKNATVQSQ	180
AEC15194.1	VHSNGQAAHDNGAGKPVQGTSFHFFSVGGEALELQGVVFNYRTKYPDGTIFPKNATVQSQ	180
AEC15195.1	VHSNGQAAHDNGAGKPVQGTSFHFFSVGGEALELQGVVFNYRTKYPDGTIFPKNATVQSQ	180
QWT77104.1	VHSNGQVAHDNGAGKPVQGTSFHFFSVGGEALELQGVVFNYRTKYPDGTIFPKNATVQSQ	180
UPN63088.1	VHSNGQAAHDNGAGKPVQGTSFHFFSVGGEALELQGVVFNYRTKYPDGTIFPKNATVQSQ	180
UPN63104.1	VHSNGQAAHDNGAGKPVQGTSFHFFSVGGEALELQGVVFNYRTTYPDGTIFPKNATVQSQ	180
UPN63112.1	VHSNGQAAHDNGARKPVQGTSFHFFSVGGEALELQGVVFNYRTKYPDGTIFPKNATVQSQ	180
UPN63113.1	VHSNGQAAHDNGAGKPVQGTSFHFFSVGGEALELQGVVFNYRTKYPDGTIFPKNATVQSQ	180
UPN63114.1	VHSNGQAAHDNGAGKPVQGTSFHFFSVGGEALELQGVVFNYRTKYPDGTIFPKNATVQSQ	180
UPN63115.1	VHSNGQAAHDNGAGKPVQGTSFHFFSVGGEALELQGVVFNYRTKYPDGTIFPKNATVQSQ	180
AEC15172.1	VHSNGQAAHDNGAGKPVQGTSFHFFSVGGEALELQGVVFNYRTKYPDGTIFPKNATVQSQ	180
BAE00117.1	VHSNGQAAHDNGAGKPVQGTSFHFFSVGGEALELQGVVFNYRTKYPDGTIFPKNATVQSQ	180
BAE00123.1	VHSNGQAAHDNGAGKPVQGTSFHFFSVGGEALELQGVVFNYRTKYPDGTIFPKNATVQSQ	180
AAC59331.1	VHSNGQAAHDNGAGKPVQGTSFHFFSVGGEALELQGVVFNYRTKYPDGTIFPKNATVQSQ	180
WLD48106.1	VHSNGQAAHDNGAGKPVQGTSFHFFSVGGEALELQGVVFNYRTKYPDGTIFPKNATVQSQ	180
UQK62661.1	VHSNGQAAHDNGAGKPVQGTSFHFFSVGGEALELQGVVFNYRTKYPDGTIFPKNATVQSQ	180
QTJ15156.1	VHSNGQAAHDNGAGKPVQGTSFHFFSVGGEALELQGVVFNYRTKYPDGTIFPKNATVQSQ	180
QNC68213.1	VHSNGQAAHDNGAGKPVQGTSFHFFSVGGEALELQGVVFNYRTKYPDGTIFPKNATVQSQ	180
ASV51766.1	VHSNGQAAHDNGAGKPVQGTSFHFFSVGGEALELQGVVFNYRTKYPDGTIFPKNATVQSQ	180
ASV51796.1	VHSNGQAAHDNGAGKPVQGTSFHFFSVGGEALELQGVVFNYRTKYPDGTIFPKNATVQSQ	180
ASV51802.1	VHSNGQAAHDNGAGKPVQGTSFHFFSVGGEALELQGVVFNYRTKYPDGTIFPKNATVQSQ	180
ASV51808.1	VHSNGQAAHDNGAGKPVQGTSFHFFSVGGEALELQGVVFNYRTKYPDGTIFPKNATVQSQ	180
ASV51820.1	VHSNGQAAHDNGAGKPVQGTSFHFFSVGGEALELQGVVFNYRTKYPDGTIFPKNATVQSQ	180
ASV51826.1	VHSNGQAAHDNGAGKPVQGTSFHFFSVGGEALELQGVVFNYRTKYPDGTIFPKNATVQSQ	180
ASV51832.1	VHSNGQAAHDNGAGKPVQGTSFHFFSVGGEALELQGVVFNYRTKYPDGTIFPKNATVQSQ	180
ASV51838.1	VHSNGQAAHDNGAGKPVQGTSFHFFSVGGEALELQGVVFNYRTKYPDGTIFPKNATVQSQ	180
ASV51862.1	VHSNGQAAHDNGAGKPVQGTSFHFFSVGGEALELQGVVFNYRTKYPDGTIFPKNATVQSQ	180
ASV51868.1	VHSNGQAAHDNGAGKPVQGTSFHFFSVGGEALELQGVVFNYRTTYPDGTIFPKNATVQSQ	180
ASV51874.1	VHSNGQAAHDNGAGKPVQGTSFHFFSVGGEALELQGVVFNYRTKYPDGTIFPKNATVQSQ	180
ASV51880.1	VHSNGQAAHDNGAGKPVQGTSFHFFSVGGEALELQGVVFNYRTKYPDGTIFPKNATVQSQ	180
ASV51892.1	VHSNGQAAHDNGAGKPVQGTSFHFFSVGGEALELQGVVFNYRTKYPDGTIFPKNATVQSQ	180
ANH21098.1	VHSNGQAAHDNGAGKPVQGTSFHFFSVGGEALELQGVVFNYRTKYPDGTIFPKNATVQSQ	180
AID67150.1	VHSNGQAAHDNGAGKPVQGTSFHFFSVGGEALELQGVVFNYRTKYPDGTIFPKNATVQSQ	180
AID67156.1	VHSNGQAAHDNGAGKPVQGTSFHFFSVGGEALELQGVVFNYRTKYPDGTIFPKNATVQSQ	180

AID67162.1	VHSNGQAAHDNGAGKPVQGTSFHFFSVGGEALELQGVVFNYRTKYPDGTIFPKNATVQSQ	180
AID67168.1	VHSNGQAAHDNGAGKPVQGTSFHFFSVGGEALELQGVVFNYRTKYPDGTIFPKNATVQSQ	180
CCW72479.1	VHSNGQAAHDNGAGKPVQGTSFHFFSVGGEALELQGVVFNYRTKYPDGTIFPKNATVQSQ	180
CCW72486.1	VHSNGQAAHDNGAGKPVQGTSFHFFSVGGEALELQGVVFNYRTKYPDGTIFPKNATVQSQ	180
CCW72494.1	VHSNGQAAHDNGAGKPVQGTSFHFFSVGGEALELQGVVFNYRTKYPDGTIFPKNATVQSQ	180
CCW72514.1	VHSNGQAAHDNGAGKPVQGTSFHFFSVGGEALELQGVVFNYRTKYPDGTIFPKNATVQSQ	180
CCW72518.1	VHSNGQAAHDNGAGKPVQGTSFHFFSVGGEALELQGVVFNYRTKYPDGTIFPKNATVQSQ	180
CCW72522.1	VHSNGQAAHDNGAGKPVQGTSFHFFSVGGEALELQGVVFNYRTKYPDGTIFPKNATVQSQ	180
AFH57194.1	VHSNGQAAHDNGAGKPVQGTSFHFFSVGGEALELQGVVFNYRTKYPDGTIFPKNATVQSQ	180
AEC15047.1	VHSNGQAAHDNGAGKPVQGTSFHFFSVGGEALELQGVVFNYRTKYPDGTIFPKNATVQSQ	180
AEC15048.1	VHSNGQAAHDNGAGKPVQGTSFHFFSVGGEALELQGVVFNYRTKYPDGTIFPKNATVQSQ	180
AEC15049.1	VHSNGQAAHDNGAGKPVQGTSFHFFSVGGEALELQGVVFNYRTKYPDGTIFPKNATVQSQ	180
AEC15050.1	VHSNGQAAHDNGAGKPVQGTSFHFFSVGGEALELQGVVFNYRTKYPDGTIFPKNATVQSQ	180
AEC15051.1	VHSNGQAAHDNGAGKPVQGTSFHFFSVGGEALELQGVVFNYRTKYPDGTIFPKNATVQSQ	180
AEC15052.1	VHSNGQAAHDNGAGKPVQGTSFHFFSVGGEALELQGVVFNYRTKYPDGTIFPKNATVQSQ	180
AEC15053.1	VHSNGQAAHDNGAGKPVQGTSFHFFSVGGEALELQGVVFNYRTKYPDGTIFPKNATVQSQ	180
AEC15054.1	VHSNGQAAHDNGAGKPVQGTSFHFFSVGGEALELQGVVFNYRTKYPDGTIFPKNATVQSQ	180
AEC15055.1	VHSNGQAAHDNGAGKPVQGTSFHFFSVGGEALELQGVVFNYRTKYPDGTIFPKNATVQSQ	180
AEC15056.1	VHSNGQAAHDNGAGKPVQGTSFHFFSVGGEALELQGVVFNYRTKYPDGTIFPKNATVQSQ	180
AEC15057.1	VHSNGQAAHDNGAGKPVQGTSFHFFSVGGEALELQGVVFNYRTKYPDGTIFPKNATVQSQ	180
AEC15058.1	VHSNGQAAHDNGAGKPVQGTSFHFFSVGGEALELQGVVFNYRTKYPDGTIFPKNATVQSQ	180
AEC15059.1	VHSNGQAAHDNGAGKPVQGTSFHFFSVGGEALELQGVVFNYRTKYPDGTIFPKNATVQSQ	180
AEC15060.1	VHSNGQAAHDNGAGKPVQGTSFHFFSVGGEALELQGVVFNYRTKYPDGTIFPKNATVQSQ	180
AEC15061.1	VHSNGQAAHDNGAGKPVQGTSFHFFSVGGEALELQGVVFNYRTKYPDGTIFPKNATVQSQ	180
AEC15062.1	VHSNGQAAHDNGAGKPVQGTSFHFFSVGGEALELQGVVFNYRTKYPDGTIFPKNATVQSQ	180
AEC15063.1	VHSNGQAAHDNGAGKPVQGTSFHFFSVGGEALELQGVVFNYRTKYPDGTIFPKNATVQSQ	180
AEC15064.1	VHSNGQAAHDNGAGKPVQGTSFHFFSVGGEALELQGVVFNYRTKYPDGTIFPKNATVQSQ	180
AEC15065.1	VHSNGQAAHDNGAGKPVQGTSFHFFSVGGEALELQGVVFNYRTKYPDGTIFPKNATVQSQ	180
AEC15066.1	VHSNGQAAHDNGAGKPVQGTSFHFFSVGGEALELQGVVFNYRTKYPDGTIFPKNATVQSQ	180
AEC15067.1	VHSNGQAAHDNGAGKPVQGTSFHFFSVGGEALELQGVVFNYRTKYPDGTIFPKNATVQSQ	180
AEC15068.1	VHSNGQAAHDNGAGKPVQGTSFHFFSVGGEALELQGVVFNYRTKYPDGTIFPKNATVQSQ	180
AEC15315.1	VHSNGQAAHDNGAGKPVQGTSFHFFSVGGEALELQGVVFNYRTKYPDGTIFPKNATVQSQ	180
AEC15321.1	VHSNGQAAHDNGAGKPVQGTSFHFFSVGGEALELQGVVFNYRTKYPDGTIFPKNATVQSQ	180
BAD21271.1	VHSNGQAAHDNGAGKPVQGTSFHFFSVGGEALELQGVVFNYRTKYPDGTIFPKNATVQSQ	180
BAD21283.2	VHSNGQAAHDNGAGKPVQGTSFHFFSVGGEALELQGVVFNYRTKYPDGTIFPKNATVQSQ	180
BAC15683.1	VHSNGQAAHDNGAGKPVQGTSFHFFSVGGEALELQGVVFNYRTKYPDGTIFPKNATVQSQ	180
BAC15719.1	VHSNGQAAHDNGAGKPVQGTSFHFFSVGGEALELQGVVFNYRTKYPDGTIFPKNATVQSQ	180
BAB93038.1	VHSNGQAAHDNGAGKPVQGTSFHFFSVGGEALELQGVVFNYRTKYPDGTIFPKNATVQSQ	180
BAB93056.1	VHSNGQAAHDNGAGKPVQGTSFHFFSVGGEALELQGVVFNYRTKYPDGTIFPKNATVQSQ	180
BAB93062.1	VHSNGQAAHDNGAGKPVQGTSFHFFSVGGEALELQGVVFNYRTKYPDGTIFPKNATVQSQ	180
BAB68911.1	VHSNGQAAHDNGAGKPVQGTSFHFFSVGGEALELQGVVFNYRTKYPDGTIFPKNATVQSQ	180
BAB68947.1	VHSNGQAAHDNGAGKPVQGTSFHFFSVGGEALELQGVVFNYRTKYPDGTIFPKNATVQSQ	180
AAK70234.1	VHSNGQAAHDNGAGKPVQGTSFHFFSVGGEALELQGVVFNYRTKYPDGTIFPKNATVQSQ	180
AAK70246.1	VHSNGQAAHDNGAGKPVQGTSFHFFSVGGEALELQGVVFNYRTKYPDGTIFPKNATVQSQ	180
BAA01959.1	VHSNGQAAHDNGAGKPVQGTSFHFFSVGGEALELQGVVFNYRTKYPDGTIFPKNATVQSQ	180
BAA01960.1	VHSNGQAAHDNGAGKPVQGTSFHFFSVGGEALELQGVVFNYRTKYPDGTIFPKNATVQSQ	180
QWT77128.1	VHSNGQAAHDNGAGKPVQGTSFHFFSVGGEALELQGVVFNYRTKYPDGTIFPKNATVQSQ	180
UPN63089.1	VHSNGQAAHDNGAGKPVQGTSFHFFSVGGEALELQGVVFNYRTKYPDGTIFPKNATVQSQ	180
UPN63093.1	VHSNGQAAHDNGAGKPVQGTSFHFFSVGGEALELQGVVFNYRTKYPDGTIFPKNATVQSQ	180
UPN63103.1	VHSNGQAAHDNGAGKPVQGTSFHFFSVGGEALELQGVVFNYRTKYPDGTIFPKNATVQSQ	180
UPN63105.1	VHSNGQAAHDNGAGKPVQGTSFHFFSVGGEALELQGVVFNYRTKYPDGTIFPKNATVQSQ	180
UPN63106.1	VHSNGQAAHDNGAGKPVQGTSFHFFSVGGEALELQGVVFNYRTKYPDGTIFPKNATVQSQ	180
UPN63110.1	VHSNGQAAHDNGAGKPVQGTSFHFFSVGGEALELQGVVFNYRTKYPDGTIFPKNATVQSQ	180
UPN63111.1	VHSNGQAAHDNGAGKPVQGTSFHFFSVGGEALELQGVVFNYRTKYPDGTIFPKNATVQSQ	180
AEC15030.1	VHSNGQAAHDNGAGKPVQGTSFHFFSVGGEALELQGVVFNYRTKYPDGTIFPKNATVQSQ	180
AEC15031.1	VHSNGQAAHDNGAGKPVQGTSFHFFSVGGEALELQGVVFNYRTKYPDGTIFPKNATVQSQ	180
AEC15032.1	VHSNGQAAHDNGAGKPVQGTSFHFFSVGGEALELQGVVFNYRTKYPDGTIFPKNATVQSQ	180
AEC15033.1	VHSNGQAAHDNGAGKPVQGTSFHFFSVGGEALELQGVVFNYRTKYPDGTIFPKNATVQSQ	180
AEC15034.1	VHSNGQAAHDNGAGKPVQGTSFHFFSVGGEALELQGVVFNYRTKYPDGTIFPKNATVQSQ	180
AEC15035.1	VHSNGQAAHDNGAGKPVQGTSFHFFSVGGEALELQGVVFNYRTKYPDGTIFPKNATVQSQ	180
AEC15036.1	VHSNGQAAHDNGAGKPVQGTSFHFFSVGGEALELQGVVFNYRTKYPDGTIFPKNATVQSQ	180
AEC15038.1	VHSNGQAAHDNGAGKPVQGTSFHFFSVGGEALELQGVVFNYRTKYPDGTIFPKNATVQSQ	180
AEC15039.1	VHSNGQAAHDNGAGKPVQGTSFHFFSVGGEALELQGVVFNYRTKYPDGTIFPKNATVQSQ	180
AEC15040.1	VHSNGQAAHDNGAGKPVQGTSFHFFSVGGEALELQGVVFNYRTKYPDGTIFPKNATVQSQ	180

```
AEC15041.1      VHSNGQAAHDNGAGKPVQGTSFHFFSVGGEALELQGVVFNYRTKYPDGTIFPKNATVQSQ  180
AEC15042.1      VHSNGQAAHDNGAGKPVQGTSFHFFSVGGEALELQGVVFNYRTKYPDGTIFPKNATVQSQ  180
AEC15043.1      VHSNGQAAHDNGAGKPVQGTSFHFFSVGGEALELQGVVFNYRTKYPDGTIFPKNATVQSQ  180
AEC15044.1      VHSNGQAAHDNGAGKPVQGTSFHFFSVGGEALELQGVVFNYRTKYPDGTIFPKNATVQSQ  180
AEC15045.1      VHSNGQAAHDNGAGKPVQGTSFHFFSVGGEALELQGVVFNYRTKYPDGTIFPKNATVQSQ  180
AEC15046.1      VHSNGQAAHDNGAGKPVQGTSFHFFSVGGEALELQGVVFNYRTKYPDGTIFPKNATVQSQ  180
UPN63083.1      VHSNGQAAHDNGAGKPVQGTSFHFFSVGGEALELQGVVFNYRTKYPDGTIFPKNATVQSQ  180
BAE00111.1      VHSNGQAAHDNGAGKPVQGTSFHFFSVGGEALELQGVVFNYRTKYPDGTIFPKNATVQSQ  180
BAA01968.1      VHSNGQAAHDNGAGKPVQGTSFHFFSVGGEALELQGVVFNYRTKYPDGTIFPKNATVQSQ  180
CCW72491.1      VHSNGQAAHDNGAGKPVQGTSFHFFSVGGEALELQGVVFNYRTKYPDGTIFPKNATVQSQ  180
AEC14788.1      VHSNGQAAHDNGAGKPVQGTSFHFFSVGGEALELQGVVFNYRTKYPDGTIFPKNATVQSQ  180
AEC14790.1      VHSNGQAAHDNGAGKPVQGTSFHFFSVGGEALELQGVVFNYRTKYPDGTIFPKNATVQSQ  180
AEC14792.1      VHSNGQAAHDNGAGKPVQGTSFHFFSVGGEALELQGVVFNYRTKYPDGTIFPKNATVQSQ  180
AEC14798.1      VHSNGQAAHDNGAGKPVQGTSFHFFSVGGEALELQGVVFNYRTKYPDGTIFPKNATVQSQ  180
AEC14799.1      VHSNGQAAHDNGAGKPVQGTSFHFFSVGGEALELQGVVFNYRTKYPDGTIFPKNATVQSQ  180
AEC14800.1      VHSNGQAAHDNGAGKPVQGTSFHFFSVGGEALELQGVVFNYRTKYPDGTIFPKNATVQSQ  180
AEC14801.1      VHSNGQAAHDNGAGKPVQGTSFHFFSVGGEALELQGVVFNYRTKYPDGTIFPKNATVQSQ  180
AEC14805.1      VHSNGQAAHDNGAGKPVQGTSFHFFSVGGEALELQGVVFNYRTKYPDGTIFPKNATVQSQ  180
AEC14807.1      VHSNGQAAHDNGAGKPVQGTSFHFFSVGGEALELQGVVFNYRTKYPDGTIFPKNATVQSQ  180
AEC14808.1      VHSNGQAAHDNGAGKPVQGTSFHFFSVGGEALELQGVVFNYRTKYPDGTIFPKNATVQSQ  180
AEC14809.1      VHSNGQAAHDNGAGKPVQGTSFHFFSVGGEALELQGVVFNYRTKYPDGTIFPKNATVQSQ  180
AEC14813.1      VHSNGQAAHDNGAGKPVQGTSFHFFSVGGEALELQGVVFNYRTKYPDGTIFPKNATVQSQ  180
AEC14912.1      VHSNGQAAHDNGAGKPVQGTSFHFFSVGGEALELQGVVFNYRTKYPDGTIFPKNATVQSQ  180
AEC14913.1      VHSNGQAAHDNGAGKPVQGTSFHFFSVGGEALELQGVVFNYRTKYPDGTIFPKNATVQSQ  180
AEC14914.1      VHSNGQAAHDNGAGKPVQGTSFHFFSVGGEALELQGVVFNYRTKYPDGTIFPKNATVQSQ  180
AEC14915.1      VHSNGQAAHDNGAGKPVQGTSFHFFSVGGEALELQGVVFNYRTKYPDGTIFPKNATVQSQ  180
AEC14916.1      VHSNGQAAHDNGAGKPVQGTSFHFFSVGGEALELQGVVFNYRTKYPDGTIFPKNATVQSQ  180
AEC14917.1      VHSNGQAAHDNGAGKPVQGTSFHFFSVGGEALELQGVVFNYRTKYPDGTIFPKNATVQSQ  180
AEC14918.1      VHSNGQAAHDNGAGKPVQGTSFHFFSVGGEALELQGVVFNYRTKYPDGTIFPKNATVQSQ  180
AEC14919.1      VHSNGQAAHDNGAGKPVQGTSFHFFSVGGEALELQGVVFNYRTKYPDGTIFPKNATVQSQ  180
AEC14920.1      VHSNGQAAHDNGAGKPVQGTSFHFFSVGGEALELQGVVFNYRTKYPDGTIFPKNATVQSQ  180
AEC14921.1      VHSNGQAAHDNGAGKPVQGTSFHFFSVGGEALELQGVVFNYRTKYPDGTIFPKNATVQSQ  180
AEC14922.1      VHSNGQAAHDNGAGKPVQGTSFHFFSVGGEALELQGVVFNYRTKYPDGTIFPKNATVQSQ  180
AEC14923.1      VHSNGQAAHDNGAGKPVQGTSFHFFSVGGEALELQGVVFNYRTKYPDGTIFPKNATVQSQ  180
AEC14924.1      VHSNGQAAHDNGAGKPVQGTSFHFFSVGGEALELQGVVFNYRTKYPDGTIFPKNATVQSQ  180
AEC14925.1      VHSNGQAAHDNGAGKPVQGTSFHFFSVGGEALELQGVVFNYRTKYPDGTIFPKNATVQSQ  180
AEC14926.1      VHSNGQAAHDNGAGKPVQGTSFHFFSVGGEALELQGVVFNYRTKYPDGTIFPKNATVQSQ  180
AEC14927.1      VHSNGQAAHDNGAGKPVQGTSFHFFSVGGEALELQGVVFNYRTKYPDGTIFPKNATVQSQ  180
AEC14928.1      VHSNGQAAHDNGAGKPVQGTSFHFFSVGGEALELQGVVFNYRTKYPDGTIFPKNATVQSQ  180
AEC14929.1      VHSNGQAAHDNGAGKPVQGTSFHFFSVGGEALELQGVVFNYRTKYPDGTIFPKNATVQSQ  180
AEC14930.1      VHSNGQAAHDNGAGKPVQGTSFHFFSVGGEALELQGVVFNYRTKYPDGTIFPKNATVQSQ  180
AEC14931.1      VHSNGQAAHDNGAGKPVQGTSFHFFSVGGEALELQGVVFNYRTKYPDGTIFPKNATVQSQ  180
AEC14932.1      VHSNGQAAHDNGAGKPVQGTSFHFFSVGGEALELQGVVFNYRTKYPDGTIFPKNATVQSQ  180
AEC14933.1      VHSNGQAAHDNGAGKPVQGTSFHFFSVGGEALELQGVVFNYRTKYPDGTIFPKNATVQSQ  180
AEC14934.1      VHSNGQAAHDNGAGKPVQGTSFHFFSVGGEALELQGVVFNYRTKYPDGTIFPKNATVQSQ  180
AEC14935.1      VHSNGQAAHDNGAGKPVQGTSFHFFSVGGEALELQGVVFNYRTKYPDGTIFPKNATVQSQ  180
AEC14936.1      VHSNGQAAHDNGAGKPVQGTSFHFFSVGGEALELQGVVFNYRTKYPDGTIFPKNATVQSQ  180
AEC14937.1      VHSNGQAAHDNGAGKPVQGTSFHFFSVGGEALELQGVVFNYRTKYPDGTIFPKNATVQSQ  180
AEC14938.1      VHSNGQAAHDNGAGKPVQGTSFHFFSVGGEALELQGVVFNYRTKYPDGTIFPKNATVQSQ  180
AEC14939.1      VHSNGQAAHDNGAGKPVQGTSFHFFSVGGEALELQGVVFNYRTKYPDGTIFPKNATVQSQ  180
AEC14940.1      VHSNGQAAHDNGAGKPVQGTSFHFFSVGGEALELQGVVFNYRTKYPDGTIFPKNATVQSQ  180
AEC14941.1      VHSNGQAAHDNGAGKPVQGTSFHFFSVGGEALELQGVVFNYRTKYPDGTIFPKNATVQSQ  180
AEC14942.1      VHSNGQAAHDNGAGKPVQGTSFHFFSVGGEALELQGVVFNYRTKYPDGTIFPKNATVQSQ  180
AEC14943.1      VHSNGQAAHDNGAGKPVQGTSFHFFSVGGEALELQGVVFNYRTKYPDGTIFPKNATVQSQ  180
AEC14944.1      VHSNGQAAHDNGAGKPVQGTSFHFFSVGGEALELQGVVFNYRTKYPDGTIFPKNATVQSQ  180
AEC14945.1      VHSNGQAAHDNGAGKPVQGTSFHFFSVGGEALELQGVVFNYRTKYPDGTIFPKNATVQSQ  180
AEC14946.1      VHSNGQAAHDNGAGKPVQGTSFHFFSVGGEALELQGVVFNYRTKYPDGTIFPKNATVQSQ  180
AEC14947.1      VHSNGQAAHDNGAGKPVQGTSFHFFSVGGEALELQGVVFNYRTKYPDGTIFPKNATVQSQ  180
AEC14948.1      VHSNGQAAHDNGAGKPVQGTSFHFFSVGGEALELQGVVFNYRTKYPDGTIFPKNATVQSQ  180
AEC14949.1      VHSNGQAAHDNGAGKPVQGTSFHFFSVGGEALELQGVVFNYRTKYPDGTIFPKNATVQSQ  180
AEC14950.1      VHSNGQAAHDNGAGKPVQGTSFHFFSVGGEALELQGVVFNYRTKYPDGTIFPKNATVQSQ  180
AEC14951.1      VHSNGQAAHDNGAGKPVQGTSFHFFSVGGEALELQGVVFNYRTKYPDGTIFPKNATVQSQ  180
AEC14952.1      VHSNGQAAHDNGAGKPVQGTSFHFFSVGGEALELQGVVFNYRTKYPDGTIFPKNATVQSQ  180
AEC14953.1      VHSNGQAAHDNGAGKPVQGTSFHFFSVGGEALELQGVVFNYRTKYPDGTIFPKNATVQSQ  180
```

```
AEC14954.1        VHSNGQAAHDNGAGKPVQGTSFHFFSVGGEALELQGVVFNYRTKYPDGTIFPKNATVQSQ  180
AEC14955.1        VHSNGQAAHDNGAGKPVQGTSFHFFSVGGEALELQGVVFNYRTKYPDGTIFPKNATVQSQ  180
AEC14956.1        VHSNGQAAHDNGAGKPVQGTSFHFFSVGGEALELQGVVFNYRTKYPDGTIFPKNATVQSQ  180
AEC14957.1        VHSNGQAAHDNGAGKPVQGTSFHFFSVGGEALELQGVVFNYRTKYPDGTIFPKNATVQSQ  180
AEC14958.1        VHSNGQAAHDNGAGKPVQGTSFHFFSVGGEALELQGVVFNYRTKYPDGTIFPKNATVQSQ  180
AEC14959.1        VHSNGQAAHDNGAGKPVQGTSFHFFSVGGEALELQGVVFNYRTKYPDGTIFPKNATVQSQ  180
AEC14960.1        VHSNGQAAHDNGAGKPVQGTSFHFFSVGGEALELQGVVFNYRTKYPDGTIFPKNATVQSQ  180
AEC14961.1        VHSNGQAAHDNGAGKPVQGTSFHFFSVGGEALELQGVVFNYRTKYPDGTIFPKNATVQSQ  180
QWT77122.1        VHSNGQAAHDNGAGKPVQGTSFHFFSVGGEALELQGVVFNYRTKYPDGTIFPKNATVQSQ  180
UPN63084.1        VHSNGQAAHDNGAGKPVQGTSFHFFSVGGEALELQGVVFNYRTKYPDGTIFPKNATVQSQ  180
UPN63101.1        VHSNGQAAHDNGAGKPVQGTSFHFFSVGGEALELQGVVFHYRTKYPDGTIFPKNATVQSQ  180
ASV51886.1        VHSNGQAAHDNGAGKPVQGTSFHFFSVGGEALELQGVVFNYRTKYPDGTIFPKNATVQSQ  180
CCW72484.1        VHSNGQAAHDNGAGKPVQGTSFHFFSVGGEALELQGVVFNYRTKYPDGTIFPKNATVQSQ  180
ANH21099.1        VHSNGQAAHDNGAGKPVQGTSFHFFSVGGEALELQGVVFNYRTKYPDGTIFPKNATVQSQ  180
AEC15112.1        VHSNGQAAHDNGAGKPVQGTSFHFFSVGGEALELQGVVFNYRTKYPDGTIFPKNATVQSQ  180
AEC15113.1        VHSNGQAAHDNGAGKPVQGTSFHFFSVGGEALELQGVVFNYRTKYPDGTIFPKNATVQSQ  180
AEC15114.1        VHSNGQAAHDNGAGKPVQGTSFHFFSVGGEALELQGVVFNYRTKYPDGTIFPKNATVQSQ  180
AEC15115.1        VHSNGQAAHDNGAGKPVQGTSFHFFSVGGEALELQGVVFNYRTKYPDGTIFPKNATVQSQ  180
AEC15116.1        VHSNGQAAHDNGAGKPVQGTSFHFFSVGGEALELQGVVFNYRTKYPDGTIFPKNATVQSQ  180
AEC15117.1        VHSNGQAAHDNGAGKPVQGTSFHFFSVGGEALELQGVVFNYRTKYPDGTIFPKNATVQSQ  180
AEC15819.1        VHSNGQAAHDNGAGKPVQGTSFHFFSVGGEALELQGVVFNYRTKYPDGTIFPKNATVQSQ  180
AEC15825.1        VHSNGQAAHDNGAGKPVQGTSFHFFSVGGEALELQGVVFNYRTKYPDGTIFPKNATVQSQ  180
AEC15831.1        VHSNGQAAHDNGAGKPVQGTSFHFFSVGGEALELQGVVFNYRTKYPDGTIFPKNATVQSQ  180
AEC15837.1        VHSNGQAAHDNGAGKPVQGTSFHFFSVGGEALELQGVVFNYRTKYPDGTIFPKNATVQSQ  180
AEC15843.1        VHSNGQAAHDNGAGKPVQGTSFHFFSVGGEALELQGVVFNYRTKYPDGTIFPKNATVQSQ  180
AEC15849.1        VHSNGQAAHDNGAGKPVQGTSFHFFSVGGEALELQGVVFNYRTKYPDGTIFPKNATVQSQ  180
AEC15855.1        VHSNGQAAHDNGAGKPVQGTSFHFFSVGGEALELQGVVFNYRTKYPDGTIFPKNATVQSQ  180
CCW72511.1        VHSNGQAAHDNGAGKPVQGTSFHFFSVGGEALELQGVVFNYRTKYPDGTIFPKNATVQSQ  180
CCW72525.1        VHSNGQAAHDNGAGKPVQGTSFHFFSVGGEALELQGVVFNYRTKYPDGTIFPKNVTVQSQ  180
AEC14744.1        VHSNGQAAHDNGAGKPVQGTSFHFFSVGGEALELQGVVFNYRTKYPDGTIFPKNATVQSQ  180
AEC14746.1        VHSNGQAAHDNGAGKPVQGTSFHFFSVGGEALELQGVVFNYRTKYPDGTIFPKNATVQSQ  180
AEC14747.1        VHSNGQAAHDNGAGKPVQGTSFHFFSVGGEALELQGVVFNYRTKYPDGTIFPKNATVQSQ  180
AEC14748.1        VHSNGQAAHDNGAGKPVQGTSFHFFSVGGEALELQGVVFNYRTKYPDGTIFPKNATVQSQ  180
AEC14752.1        VHSNGQAAHDNGAGKPVQGTSFHFFSVGGEALELQGVVFNYRTKYPDGTIFPKNATVQSQ  180
AEC14756.1        VHSNGQAAHDNGAGKPVQGTSFHFFSVGGEALELQGVVFNYRTKYPDGTIFPKNATVQSQ  180
AEC14757.1        VHSNGQAAHDNGAGKPVQGTSFHFFSVGGEALELQGVVFNYRTKYPDGTIFPKNATVQSQ  180
AEC14758.1        VHSNGQAAHDNGAGKPVQGTSFHFFSVGGEALELQGVVFNYRTKYPDGTIFPKNATVQSQ  180
AEC14764.1        VHSNGQAAHDNGAGKPVQGTSFHFFSVGGEALELQGVVFNYRTKYPDGTIFPKNATVQSQ  180
AEC14768.1        VHSNGQAAHDNGAGKPVQGTSFHFFSVGGEALELQGVVFNYRTKYPDGTIFPKNATVQSQ  180
AEC14769.1        VHSNGQAAHDNGAGKPVQGTSFHFFSVGGEALELQGVVFNYRTKYPDGTIFPKNATVQSQ  180
AEC14770.1        VHSNGQAAHDNGAGKPVQGTSFHFFSVGGEALELQGVVFNYRTKYPDGTIFPKNATVQSQ  180
AEC14771.1        VHSNGQAAHDNGAGKPVQGTSFHFFSVGGEALELQGVVFNYRTKYPDGTIFPKNATVQSQ  180
AEC14772.1        VHSNGQAAHDNGAGKPVQGTSFHFFSVGGEALELQGVVFNYRTKYPDGTIFPKNATVQSQ  180
AEC14773.1        VHSNGQAAHDNGAGKPVQGTSFHFFSVGGEALELQGVVFNYRTKYPDGTIFPKNATVQSQ  180
AEC14775.1        VHSNGQAAHDNGAGKPVQGTSFHFFSVGGEALELQGVVFNYRTKYPDGTIFPKNATVQSQ  180
AEC14777.1        VHSNGQAAHDNGAGKPVQGTSFHFFSVGGEALELQGVVFNYRTKYPDGTIFPKNATVQSQ  180
AEC14778.1        VHSNGQAAHDNGAGKPVQGTSFHFFSVGGEALELQGVVFNYRTKYPDGTIFPKNATVQSQ  180
AEC14779.1        VHSNGQAAHDNGAGKPVQGTSFHFFSVGGEALELQGVVFNYRTKYPDGTIFPKNATVQSQ  180
AEC14782.1        VHSNGQAAHDNGAGKPVQGTSFHFFSVGGEALELQGVVFNYRTKYPDGTIFPKNATVQSQ  180
AEC14814.1        VHSNGQAAHDNGAGKPVQGTSFHFFSVGGEALELQGVVFNYRTKYPDGTIFPKNATVQSQ  180
AEC14815.1        VHSNGQAAHDNGAGKPVQGTSFHFFSVGGEALELQGVVFNYRTKYPDGTIFPKNATVQSQ  180
AEC14816.1        VHSNGQAAHDNGAGKPVQGTSFHFFSVGGEALELQGVVFNYRTKYPDGTIFPKNATVQSQ  180
AEC14817.1        VHSNGQAAHDNGAGKPVQGTSFHFFSVGGEALELQGVVFNYRTKYPDGTIFPKNATVQSQ  180
AEC14818.1        VHSNGQAAHDNGAGKPVQGTSFHFFSVGGEALELQGVVFNYRTKYPDGTIFPKNATVQSQ  180
AEC14819.1        VHSNGQAAHDNGAGKPVQGTSFHFFSVGGEALELQGVVFNYRTKYPDGTIFPKNATVQSQ  180
AEC14820.1        VHSNGQAAHDNGAGKPVQGTSFHFFSVGGEALELQGVVFNYRTKYPDGTIFPKNATVQSQ  180
AEC14822.1        VHSNGQAAHDNGAGKPVQGTSFHFFSVGGEALELQGVVFNYRTKYPDGTIFPKNATVQSQ  180
AEC14823.1        VHSNGQAAHDNGAGKPVQGTSFHFFSVGGEALELQGVVFNYRTKYPDGTIFPKNATVQSQ  180
AEC14824.1        VHSNGQAAHDNGAGKPVQGTSFHFFSVGGEALELQGVVFNYRTKYPDGTIFPKNATVQSQ  180
AEC14825.1        VHSNGQAAHDNGAGKPVQGTSFHFFSVGGEALELQGVVFNYRTKYPDGTIFPKNATVQSQ  180
AEC14826.1        VHSNGQAAHDNGAGKPVQGTSFHFFSVGGEALELQGVVFNYRTKYPDGTIFPKNATVQSQ  180
AEC14827.1        VHSNGQAAHDNGAGKPVQGTSFHFFSVGGEALELQGVVFNYRTKYPDGTIFPKNATVQSQ  180
AEC14828.1        VHSNGQAAHDNGAGKPVQGTSFHFFSVGGEALELQGVVFNYRTKYPDGTIFPKNATVQSQ  180
AEC14829.1        VHSNGQAAHDNGAGKPVQGTSFHFFSVGGEALELQGVVFNYRTKYPDGTIFPKNATVQSQ  180
```

AEC14830.1	VHSNGQAAHDNGAGKPVQGTSFHFFSVGGEALELQGVVFNYRTKYPDGTIFPKNATVQSQ	180
AEC14831.1	VHSNGQAAHDNGAGKPVQGTSFHFFSVGGEALELQGVVFNYRTKYPDGTIFPKNATVQSQ	180
AEC14832.1	VHSNGQAAHDNGAGKPVQGTSFHFFSVGGEALELQGVVFNYRTKYPDGTIFPKNATVQSQ	180
AEC14833.1	VHSNGQAAHDNGAGKPVQGTSFHFFSVGGEALELQGVVFNYRTKYPDGTIFPKNATVQSQ	180
AEC14834.1	VHSNGQAAHDNGAGKPVQGTSFHFFSVGGEALELQGVVFNYRTKYPDGTIFPKNATVQSQ	180
AEC14835.1	VHSNGQAAHDNGAGKPVQGTSFHFFSVGGEALELQGVVFNYRTKYPDGTIFPKNATVQSQ	180
AEC14836.1	VHSNGQAAHDNGAGKPVQGTSFHFFSVGGEALELQGVVFNYRTKYPDGTIFPKNATVQSQ	180
AEC14837.1	VHSNGQAAHDNGAGKPVQGTSFHFFSVGGEALELQGVVFNYRTKYPDGTIFPKNATVQSQ	180
AEC14838.1	VHSNGQAAHDNGAGKPVQGTSFHFFSVGGEALELQGVVFNYRTKYPDGTIFPKNATVQSQ	180
AEC14839.1	VHSNGQAAHDNGAGKPVQGTSFHFFSVGGEALELQGVVFNYRTKYPDGTIFPKNATVQSQ	180
AEC14840.1	VHSNGQAAHDNGAGKPVQGTSFHFFSVGGEALELQGVVFNYRTKYPDGTIFPKNATVQSQ	180
AEC14841.1	VHSNGQAAHDNGAGKPVQGTSFHFFSVGGEALELQGVVFNYRTKYPDGTIFPKNATVQSQ	180
AEC14842.1	VHSNGQAAHDNGAGKPVQGTSFHFFSVGGEALELQGVVFNYRTKYPDGTIFPKNATVQSQ	180
AEC14843.1	VHSNGQAAHDNGAGKPVQGTSFHFFSVGGEALELQGVVFNYRTKYPDGTIFPKNATVQSQ	180
AEC14844.1	VHSNGQAAHDNGAGKPVQGTSFHFFSVGGEALELQGVVFNYRTKYPDGTIFPKNATVQSQ	180
AEC14845.1	VHSNGQAAHDNGAGKPVQGTSFHFFSVGGEALELQGVVFNYRTKYPDGTIFPKNATVQSQ	180
AEC14846.1	VHSNGQAAHDNGAGKPVQGTSFHFFSVGGEALELQGVVFNYRTKYPDGTIFPKNATVQSQ	180
AEC14847.1	VHSNGQAAHDNGAGKPVQGTSFHFFSVGGEALELQGVVFNYRTKYPDGTIFPKNATVQSQ	180
AEC14848.1	VHSNGQAAHDNGAGKPVQGTSFHFFSVGGEALELQGVVFNYRTKYPDGTIFPKNATVQSQ	180
AEC14849.1	VHSNGQAAHDNGAGKPVQGTSFHFFSVGGEALELQGVVFNYRTKYPDGTIFPKNATVQSQ	180
AEC14850.1	VHSNGQAAHDNGAGKPVQGTSFHFFSVGGEALELQGVVFNYRTKYPDGTIFPKNATVQSQ	180
AEC14851.1	VHSNGQAAHDNGAGKPVQGTSFHFFSVGGEALELQGVVFNYRTKYPDGTIFPKNATVQSQ	180
AEC14852.1	VHSNGQAAHDNGAGKPVQGTSFHFFSVGGEALELQGVVFNYRTKYPDGTIFPKNATVQSQ	180
AEC14853.1	VHSNGQAAHDNGAGKPVQGTSFHFFSVGGEALELQGVVFNYRTKYPDGTIFPKNATVQSQ	180
AEC14854.1	VHSNGQAAHDNGAGKPVQGTSFHFFSVGGEALELQGVVFNYRTKYPDGTIFPKNATVQSQ	180
AEC14855.1	VHSNGQAAHDNGAGKPVQGTSFHFFSVGGEALELQGVVFNYRTKYPDGTIFPKNATVQSQ	180
AEC14856.1	VHSNGQAAHDNGAGKPVQGTSFHFFSVGGEALELQGVVFNYRTKYPDGTIFPKNATVQSQ	180
AEC14857.1	VHSNGQAAHDNGAGKPVQGTSFHFFSVGGEALELQGVVFNYRTKYPDGTIFPKNATVQSQ	180
AEC14858.1	VHSNGQAAHDNGAGKPVQGTSFHFFSVGGEALELQGVVFNYRTKYPDGTIFPKNATVQSQ	180
AEC14859.1	VHSNGQAAHDNGAGKPVQGTSFHFFSVGGEALELQGVVFNYRTKYPDGTIFPKNATVQSQ	180
AEC14862.1	VHSNGQAAHDNGAGKPVQGTSFHFFSVGGEALELQGVVFNYRTKYPDGTIFPKNATVQSQ	180
AEC14863.1	VHSNGQAAHDNGAGKPVQGTSFHFFSVGGEALELQGVVFNYRTKYPDGTIFPKNATVQSQ	180
BAD21289.1	VHSNGQAAHDNGAGKPVQGTSFHFFSVGGEALELQGVVFNYRTKYPDGTIFPKNATVQSQ	180
BAD21295.1	VHSNGQAAHDNGAGKPVQGTSFHFFSVGGEALELQGVVFNYRTKYPDGTIFPKNATVQSQ	180
BAB93044.1	VHSNGQAAHDNGAGKPVQGTSFHFFSVGGEALELQGVVFNYRTKYPDGTIFPKNATVQSQ	180
BAB93104.1	VHSNGQAAHDNGAGKPVQGTSFHFFSVGGEALELQGVVFNYRTKYPDGTIFPKNATVQSQ	180
AAK70252.1	VHSNGQAAHDNGAGKPVQGTSFHFFSVGGEALELQGVVFNYRTKYPDGTIFPKNATVQSQ	180
BAB68959.1	VHSNGQAAHDNGAGKPVQGTSFHFFSVGGEALELQGVVFNYRTKYPDGTIFPKNATVQSQ	180
BAD21229.1	VHSNGQATHDNGAGKPVQGTSFHFFSVGGEALKLQGVVFNYRTKYPDGTIFPKNATVQSQ	180
QBX89123.1	VHSNGQATHDNGAGKPVQGTSFHFFSVGGEALELQGVVFNYRTKYPDGTIFPKNATVQSQ	180
BAE02908.1	VHSNGQATHDNGAGKPVQGTSFHFFSVGGEALELQGVVFNYRTTYPDGTIFPKNATVQSQ	180
AAR89259.1	VHSNGQATHDNGAGKPVQGTSFHFFSVGGEALELQGVVFNYRTTYPDGTIFPKNATVQSQ	180
AAR12957.1	VHSNGQATHDNGAGKPVQGTSFHFFSVGGEALELQGVVFNYRTTYPDGTIFPKNATVQSQ	180
AAR13077.1	VHSNGQATHDNGAGKPVQGTSFHFFSVGGEALELQGVVFNYRTTYPDGTIFPKNATVQSQ	180
BCU41036.1	VHSNGQATHDNGAGKPVQGTSFHFFSVGGEALELQGVVFNYRTKYPDGTIFPKNATVQSQ	180
BAE94726.1	VHSNGQATHDNGAGKPVQGTSFHFFSVGGEALELQGVVFNYRTKYPDGTIFPKNATVQSQ	180
AAB94036.1	VHSNGQATHDNGAGKPVQGTSFHFFSVGGEALELQGVVFNYRTTYPDGTIFPKNATVQSQ	180
AAK28478.1	VHSNGQATHDNGAGKPVQGTSFHFFSVGGEALELQGVVFNYRTTYPDGTIFPKNATVQSQ	180
BAB93008.1	VHSNGQATHDNGAGKPVQGTSFHFFSVGGEALELQGVVFNYRTTYPDGTIFPKNATVQSQ	180
AAM69579.1	VHSNGQATHDNGAGKPVQGTSFHFFSVGGEALELQGVVFNYRTTYPDGTIFPKNATVQSQ	180
AAM69584.1	VHSNGQATHDNGAGKPVQGTSFHFFSVGGEALELQGVVFNYRTTYPDGTIFPKNATVQSQ	180
AAM69596.1	VHSNGQATHDNGAGKPVQGTSFHFFSVGGEALELQGVVFNYRTTYPDGTIFPKNATVQSQ	180
AAM69602.1	VHSNGQATHDNGAGKPVQGTSFHFFSVGGEALELQGVVFNYRTTYPDGTIFPKNATVQSQ	180
AAM69608.1	VHSNGQATHDNGAGKPVQGTSFHFFSVGGEALELQGVVFNYRTTYPDGTIFPKNATVQSQ	180
AAK70312.1	VHSNGQATHDNGAGKPVQGTSFHFFSVGGEALELQGVVFNYRTTYPDGTIFPKNATVQSQ	180
WLD48064.1	VHSNGQATHDNGAAKPVQGTSFHFFSVGGEALELQGVVFNYRTTYPDGTIFPKNATVQSQ	180
WLD48100.1	VHSNGQATHDNGAAKPVQGTSFHFFSVGGEALELQGVVFNYRTTYPDGTIFPKNATVQSQ	180
UQK62655.1	VHSNGQATHDNGAAKPVQGTSFHFFSVGGEALELQGVVFNYRTTYPDGTIFPKNATVQSQ	180
QTJ15180.1	VHSNGQATHDNGAAKPVQGTSFHFFSVGGEALELQGVVFNYRTTYPDGTIFPKNATVQSQ	180
QTJ15204.1	VHSNGQATHDNGAAKPVQGTSFHFFSVGGEALELQGVVFNYRTTYPDGTIFPKNATVQSQ	180
ASV51784.1	VHSNGQATHDNGAAKPVQGTSFHFFSVGGEALELQGVVFNYRTTYPDGTIFPKNATVQSQ	180
ASV51790.1	VHSNGQATHDNGAAKPVQGTSFHFFSVGGEALELQGVVFNYRTTYPDGTIFPKNATVQSQ	180
ASV51898.1	VHSNGQATHDNGAAKPVQGTSFHFFSVGGEALELQGVVFNYRTTYPDGTIFPKNATVQSQ	180
ASV51904.1	VHSNGQATHDNGAAKPVQGTSFHFFSVGGEALELQGVVFNYRTTYPDGTIFPKNATVQSQ	180

ASV51910.1	VHSNGQATHDNGAAKPVQGTSFHFFSVGGEALELQGVVFNYRTTYPDGTIFPKNATVQSQ	180
ASV51916.1	VHSNGQATHDNGAAKPVQGTSFHFFSVGGEALELQGVVFNYRTTYPDGTIFPKNATVQSQ	180
CCW72490.1	VHSNGQATHDNGAAKPVQGTSFHFFSVGGEALELQGVVFNYRTTYPDGTIFPKNATVQSQ	180
CCW72495.1	VHSNGQATHDNGAAKPVQGTSFHFFSVGGEALELQGVVFNYRTTYPDGTIFPKNATVQSQ	180
CCW72497.1	VHSNGQATHDNGAAKPVQGTSFHFFSVGGEALELQGVVFNYRTTYPDGTIFPKNATVQSQ	180
CCW72503.1	VHSNGQATHDNGAAKPVQGTSFHFFSVGGEALELQGVVFNYRTTYPDGTIFPKNATVQSQ	180
CCW72505.1	VHSNGQATHDNGAAKPVQGTSFHFFSVGGEALELQGVVFNYRTTYPDGTIFPKNATVQSQ	180
CCW72507.1	VHSNGQATHDNGAAKPVQGTSFHFFSVGGEALELQGVVFNYRTTYPDGTIFPKNATVQSQ	180
CCW72512.1	VHSNGQATHDNGAAKPVQGTSFHFFSVGGEALELQGVVFNYRTTYPDGTIFPKNATVQSQ	180
CCW72515.1	VHSNGQATHDNGAAKPVQGTSFHFFSVGGEALELQGVVFNYRTTYPDGTIFPKNATVQSQ	180
BAB68881.1	VHSNGQATHDNGAAKPVQGTSFHFFSVGGEALELQGVVFNYRTTYPDGTIFPKNATVQSQ	180
BAB68887.1	VHSNGQATHDNGAAKPVQGTSFHFFSVGGEALELQGVVFNYRTTYPDGTIFPKNATVQSQ	180
AAK70294.1	VHSNGQATHDNGAAKPVQGTSFHFFSVGGEALELQGVVFNYRTTYPDGTIFPKNATVQSQ	180
WLD48094.1	VHSNGQASHDNGAAKPVQGTSFHFFSVGGEALELQGVVFNYRTTYPDGTIFPKNATVQSQ	180
CCW72509.1	VHSNGQASHDNGAAKPVQGTSFHFFSVGGEALELQGVVFNYRTTYPDGTIFPKNATVQSQ	180
BAB68893.1	VHSNGQASHDNGAAKPVQGTSFHFFSVGGEALELQGVVFNYRTTYPDGTIFPKNATVQSQ	180
QTJ15216.1	VHSNGQAAHDNGAAKPVQGTSFHFFSVGGEALELQGVVFNYRTTYPDGTIFPKNATVQSQ	180
CCW72493.1	VHSNGQAAHDNGAAKPVQGTSFHFFSVGGEALELQGVVFNYRTTYPDGTIFPKNATVQSQ	180
CCW72500.1	VHSNGQAAHDNGAAKPVQGTSFHFFSVGGEALELQGVVFNYRTTYPDGTIFPKNATVQSQ	180
CCW72506.1	VHSNGQAAHDNGAAKPVQGTSFHFFSVGGEALELQGVVFNYRTTYPDGTIFPKNATVQSQ	180
CCW72520.1	VHSNGQAAHDNGAAKPVQGTSFHFFSVGGEALELQGVVFNYRTTYPDGTIFPKNATVQSQ	180
BAB68899.1	VHSNGQAAHDNGAAKPVQGTSFHFFSVGGEALELQGVVFNYRTTYPDGTIFPKNATVQSQ	180
BAB68905.1	VHSNGQAAHDNGAAKPVQGTSFHFFSVGGEALELQGVVFNYRTTYPDGTIFPKNATVQSQ	180
AAK70300.1	VHSNGQAAHDNGAAKPVQGTSFHFFSVGGEALELQGVVFNYRTTYPDGTIFPKNATVQSQ	180
AYM55579.1	VHSNGQATHDNGAGKPVQGTSFHFFSVGGEALELRGVVFNYRTKYPDGTIFPKNATVQSQ	180
AYM55580.1	VHSNGQATHDNGAGKPVQGTSFHFFSVGGEALELQGVVFNYRTKYPDGTIFPKNATVQSQ	180
AYM55582.1	VHSNGQATHDNGAGKPVQGTSFHFFSVGGEALELQGVVFNYRTKYPDGTIFPKNATVQSQ	180
AYM55583.1	VHSNGQATHDNGAGKPVQGTSFHFFSVGGEALELQGVVFNYRTKYPDGTIFPKNATVQSQ	180
AYM55584.1	VHSNGQATHDNGAGKPVQGTSFHFFSVGGEALELQGVVFNYRTKYPDGTIFPKNATVQSQ	180
AYM55590.1	VHSNGQATHDNGAGKPVQGTSFHFFSVGGEALELQGVVFNYRTKYPDGTIFPKNATVQSQ	180
AYM55596.1	VHSNGQATHDNGAGKPVQGTSFHFFSVGGEALELQGVVFNYRTKYPDGTIFPKNATVQSQ	180
BAE45366.1	VHSNGQATHDNGAGKPVQGTSFHFFSVGGEALELQGVVFNYRTKYPDGTIFPKNATVQSQ	180
AAG30857.1	VHSNGQATHDNGAGKPVQGTSFHFFSVGGEALELQGVVFNYRTKYPDGTIFPKNATVQSQ	180
BAH36774.1	VHSNGQATHDNGAGKPVQGTSFHFFSVGGEALELQGVVFNYRTKYPDGTIFPKNATVQSQ	180
BAH36780.1	VHSNGQATHDNGAGKPVQGTSFHFFSVGGEALELQGVVFNYRTKYPDGTIFPKNATVQSQ	180
BAH36822.1	VHSNGQATHDNGAGKPVQGTSFHFFSVGGEALELQGVVFNYRTKYPDGTIFPKNATVQSQ	180
BAH36834.1	VHSNGQATHDNGAGKPVQGTSFHFFSVGGEALELQGVVFNYRTKYPDGTIFPKNATVQSQ	180
BAH36840.1	VHSNGQATHDNGAGKPVQGTSFHFFSVGGEALELQGVVFNYRTKYPDGTIFPKNATVQSQ	180
BAH36846.1	VHSNGQATHDNGAGKPVQGTSFHFFSVGGEALELQGVVFNYRTKYPDGTIFPKNATVQSQ	180
BAH36816.1	VHSNGQATHDNGAGKPVQGTSFHFFSVGGEALELQGVVFNYRTKYPDGTIFPKNATVQSQ	180
BAE45444.1	VHSNGQATHDNGAGKPVQGTSFHFFSVGGEALELQGVVFNYRTKYPDGTIYPKNATVQSQ	180
AAT09819.1	VHSNGQATHDNGAGKPVQGTSFHFFSVGGEALELQGVVFNYRTKYPDGTIFPKNATVQSQ	180
BAC03029.1	VHSNGQATHDNGAGKPVQGTSFHFFSVGGEALELQGVVFNYRTKYPDGTIFPKNATVQSQ	180
BAC03095.1	VHSNGQATHDNGAGKPVQGTSFHFFSVGGEALELQGVVFNYRTKYPDGTIFPKNATVQSQ	180
AAM69560.1	VHSNGQATHDNGAGKPVQGTSFHFFSVGGEALELQGVVFNYRTKYPDGTIFPKNATVQSQ	180
AAM69566.1	VHSNGQATHDNGAGKPVQGTSFHFFSVGGEALELQGVVFNYRTKYPDGTIFPKNATVQSQ	180
AAM69572.1	VHSNGQATHDNGAGKPVQGTSFHFFSVGGEALELQGVVFNYRTKYPDGTIFPKNATVQSQ	180
AAB84311.1	VHSNGQATHDNGAGKPVQGTSFHFFSVGGEALELQGVVFNYRTKYPDGTIFPKNATVQSQ	180
BAD06150.1	VHSNGQATHDNGAGKPVQGTSFHFFSVGGEALELQGVVFNYRTKYPDGTIFPKNATVQSQ	180
AYM55581.1	VHSNGQATHDNGAGKPVQGTSFHFFSVGGEALELQGVVFNYRTKYPDGTIFPKNATVQSQ	180
AYM55588.1	VHSNGQATHDNGAGKPVQGTSFHFFSVGGEALELQGVVFNYRTKYPDGTIFPKNATVQSQ	180
AYM55589.1	VHSNGQATHDNGAGKPVQGTSFHFFSVGGEALELQGVVFNYRTKYPDGTIFPKNATVQSQ	180
AYM55591.1	VHSNGQATHDNGAGKPVQGTSFHFFSVGGEALELQGVVFNYRTKYPDGTIFPKNATVQSQ	180
AYM55592.1	VHSNGQATHDNGAGKPVQGTSFHFFSVGGEALELQGVVFNYRTKYPDGTIFPKNATVQSQ	180
AYM55593.1	VHSNGQATHDNGAGKPVQGTSFHFFSVGGEALELQGVVFNYRTKYPDGTIFPKNATVQSQ	180
AYM55595.1	VHSNGQATHDNGAGKPVQGTSFHFFSVGGEALELQGVVFNYRTKYPDGTIFPKNATVQSQ	180
AYM55597.1	VHSNGQATHDNGAGKPVQGTSFHFFSVGGEALELQGVVFNYRTKYPDGTIFPKNATVQSQ	180
AYM55598.1	VHSNGQATHDNGAGKPVQGTSFHFFSVGGEALELQGVVFNYRTKYPDGTIFPKNATVQSQ	180
AYM55599.1	VHSNGQATHDNGAGKPVQGTSFHFFSVGGEALELQGVVFNYRTKYPDGTIFPKNATVQSQ	180
AYM55600.1	VHSNGQATHDNGAGKPVQGTSFHFFSVGGEALELQGVVFNYRTKYPDGTIFPKNATVQSQ	180
AYM55601.1	VHSNGQATHDNGAGKPVQGTSFHFFSVGGEALELQGVVFNYRTKYPDGTIFPKNATVQSQ	180
AYM55602.1	VHSNGQATHDNGAGKPVQGTSFHFFSVGGEALELQGVVFNYRTKYPDGTIFPKNATVQSQ	180
AYM55603.1	VHSNGQATHDNGAGKPVQGTSFHFFSVGGEALELQGVVFNYRTKYPDGTIFPKNATVQSQ	180
CCW72483.1	VHSNGQATHDNGAGKPVQGTSFHFFSVGGEALELQGVVFNYRTKYPDGTIFPKNATVQSQ	180

CCW72485.1	VHSNGQATHDNGAGKPVQGTSFHFFSVGGEALELQGVVFNYRTKYPDGTIFPKNATVQSQ	180
AFR56669.1	VHSNGQATHDNGAGKPVQGTSFHFFSVGGEALELQGVVFNYRTKYPDGTIFPKNATVQSQ	180
BAH36768.1	VHSNGQATHDNGAGKPVQGTSFHFFSVGGEALELQGVVFNYRTKYPDGTIFPKNATVQSQ	180
BAH36786.1	VHSNGQATHDNGAGKPVQGTSFHFFSVGGEALELQGVVFNYRTKYPDGTIFPKNATVQSQ	180
BAH36792.1	VHSNGQATHDNGAGKPVQGTSFHFFSVGGEALELQGVVFNYRTKYPDGTIFPKNATVQSQ	180
BAH36798.1	VHSNGQATHDNGAGKPVQGTSFHFFSVGGEALELQGVVFNYRTKYPDGTIFPKNATVQSQ	180
BAH36804.1	VHSNGQATHDNGAGKPVQGTSFHFFSVGGEALELQGVVFNYRTKYPDGTIFPKNATVQSQ	180
BAH36810.1	VHSNGQATHDNGAGKPVQGTSFHFFSVGGEALELQGVVFNYRTKYPDGTIFPKNATVQSQ	180
BAH36828.1	VHSNGQATHDNGAGKPVQGTSFHFFSVGGEALELQGVVFNYRTKYPDGTIFPKNATVQSQ	180
BAH36852.1	VHSNGQATHDNGAGKPVQGTSFHFFSVGGEALELQGVVFNYRTKYPDGTIFPKNATVQSQ	180
BAH36858.1	VHSNGQATHDNGAGKPVQGTSFHFFSVGGEALELQGVVFNYRTKYPDGTIFPKNATVQSQ	180
BAE75814.1	VHSNGQATHDNGAGKPVQGTSFHFFSVGGEALELQGVVFNYRTKYPDGTIFPKNATVQSQ	180
BAE75820.1	VHSNGQATHDNGAGKPVQGTSFHFFSVGGEALELQGVVFNYRTKYPDGTIFPKNATVQSQ	180
BAE75826.1	VHSNGQATHDNGAGKPVQGTSFHFFSVGGEALELQGVVFNYRTKYPDGTIFPKNATVQSQ	180
BAE75832.1	VHSNGQATHDNGAGKPVQGTSFHFFSVGGEALELQGVVFNYRTKYPDGTIFPKNATVQSQ	180
BAE75838.1	VHSNGQATHDNGAGKPVQGTSFHFFSVGGEALELQGVVFNYRTKYPDGTIFPKNATVQSQ	180
BAE45360.1	VHSNGQATHDNGAGKPVQGTSFHFFSVGGEALELQGVVFNYRTKYPDGTIFPKNATVQSQ	180
BAE45372.1	VHSNGQATHDNGAGKPVQGTSFHFFSVGGEALELQGVVFNYRTKYPDGTIFPKNATVQSQ	180
BAE45378.1	VHSNGQATHDNGAGKPVQGTSFHFFSVGGEALELQGVVFNYRTKYPDGTIFPKNATVQSQ	180
BAE45384.1	VHSNGQATHDNGAGKPVQGTSFHFFSVGGEALELQGVVFNYRTKYPDGTIFPKNATVQSQ	180
BAE45390.1	VHSNGQATHDNGAGKPVQGTSFHFFSVGGEALELQGVVFNYRTKYPDGTIFPKNATVQSQ	180
BAE45396.1	VHSNGQATHDNGAGKPVQGTSFHFFSVGGEALELQGVVFNYRTKYPDGTIFPKNATVQSQ	180
BAE45402.1	VHSNGQATHDNGAGKPVQGTSFHFFSVGGEALELQGVVFNYRTKYPDGTIFPKNATVQSQ	180
BAE45408.1	VHSNGQATHDNGAGKPVQGTSFHFFSVGGEALELQGVVFNYRTKYPDGTIFPKNATVQSQ	180
BAE45414.1	VHSNGQATHDNGAGKPVQGTSFHFFSVGGEALELQGVVFNYRTKYPDGTIFPKNATVQSQ	180
BAE45420.1	VHSNGQATHDNGAGKPVQGTSFHFFSVGGEALELQGVVFNYRTKYPDGTIFPKNATVQSQ	180
BAE45426.1	VHSNGQATHDNGAGKPVQGTSFHFFSVGGEALELQGVVFNYRTKYPDGTIFPKNATVQSQ	180
BAE45432.1	VHSNGQATHDNGAGKPVQGTSFHFFSVGGEALELQGVVFNYRTKYPDGTIFPKNATVQSQ	180
BAE45438.1	VHSNGQATHDNGAGKPVQGTSFHFFSVGGEALELQGVVFNYRTKYPDGTIFPKNATVQSQ	180
BAD06018.1	VHSNGQATHDNGAGKPVQGTSFHFFSVGGEALELQGVVFNYRTKYPDGTIFPKNATVQSQ	180
BAD06024.1	VHSNGQATHDNGAGKPVQGTSFHFFSVGGEALELQGVVFNYRTKYPDGTIFPKNATVQSQ	180
BAD06030.1	VHSNGQATHDNGAGKPVQGTSFHFFSVGGEALELQGVVFNYRTKYPDGTIFPKNATVQSQ	180
BAD06042.1	VHSNGQATHDNGAGKPVQGTSFHFFSVGGEALELQGVVFNYRTKYPDGTIFPKNATVQSQ	180
BAD06048.1	VHSNGQATHDNGAGKPVQGTSFHFFSVGGEALELQGVVFNYRTKYPDGTIFPKNATVQSQ	180
BAD06054.1	VHSNGQATHDNGAGKPVQGTSFHFFSVGGEALELQGVVFNYRTKYPDGTIFPKNATVQSQ	180
BAD06084.1	VHSNGQATHDNGAGKPVQGTSFHFFSVGGEALELQGVVFNYRTKYPDGTIFPKNATVQSQ	180
BAD06090.1	VHSNGQATHDNGAGKPVQGTSFHFFSVGGEALELQGVVFNYRTKYPDGTIFPKNATVQSQ	180
BAD06096.1	VHSNGQATHDNGAGKPVQGTSFHFFSVGGEALELQGVVFNYRTKYPDGTIFPKNATVQSQ	180
BAD06102.1	VHSNGQATHDNGAGKPVQGTSFHFFSVGGEALELQGVVFNYRTKYPDGTIFPKNATVQSQ	180
BAD06108.1	VHSNGQATHDNGAGKPVQGTSFHFFSVGGEALELQGVVFNYRTKYPDGTIFPKNATVQSQ	180
BAD06120.1	VHSNGQATHDNGAGKPVQGTSFHFFSVGGEALELQGVVFNYRTKYPDGTIFPKNATVQSQ	180
BAC15695.1	VHSNGQATHDNGAGKPVQGTSFHFFSVGGEALELQGVVFNYRTKYPDGTIFPKNATVQSQ	180
BAC15725.1	VHSNGQATHDNGAGKPVQGTSFHFFSVGGEALELQGVVFNYRTKYPDGTIFPKNATVQSQ	180
BAC02951.1	VHSNGQATHDNGAGKPVQGTSFHFFSVGGEALELQGVVFNYRTKYPDGTIFPKNATVQSQ	180
BAC02957.1	VHSNGQATHDNGAGKPVQGTSFHFFSVGGEALELQGVVFNYRTKYPDGTIFPKNATVQSQ	180
BAC02963.1	VHSNGQATHDNGAGKPVQGTSFHFFSVGGEALELQGVVFNYRTKYPDGTIFPKNATVQSQ	180
BAC02969.1	VHSNGQATHDNGAGKPVQGTSFHFFSVGGEALELQGVVFNYRTKYPDGTIFPKNATVQSQ	180
BAC02975.1	VHSNGQATHDNGAGKPVQGTSFHFFSVGGEALELQGVVFNYRTKYPDGTIFPKNATVQSQ	180
BAC02981.1	VHSNGQATHDNGAGKPVQGTSFHFFSVGGEALELQGVVFNYRTKYPDGTIFPKNATVQSQ	180
BAC02987.1	VHSNGQATHDNGAGKPVQGTSFHFFSVGGEALELQGVVFNYRTKYPDGTIFPKNATVQSQ	180
BAC02993.1	VHSNGQATHDNGAGKPVQGTSFHFFSVGGEALELQGVVFNYRTKYPDGTIFPKNATVQSQ	180
BAC02999.1	VHSNGQATHDNGAGKPVQGTSFHFFSVGGEALELQGVVFNYRTKYPDGTIFPKNATVQSQ	180
BAC03005.1	VHSNGQATHDNGAGKPVQGTSFHFFSVGGEALELQGVVFNYRTKYPDGTIFPKNATVQSQ	180
BAC03011.1	VHSNGQATHDNGAGKPVQGTSFHFFSVGGEALELQGVVFNYRTKYPDGTIFPKNATVQSQ	180
BAC03017.1	VHSNGQATHDNGAGKPVQGTSFHFFSVGGEALELQGVVFNYRTKYPDGTIFPKNATVQSQ	180
BAC03023.1	VHSNGQATHDNGAGKPVQGTSFHFFSVGGEALELQGVVFNYRTKYPDGTIFPKNATVQSQ	180
BAC03035.1	VHSNGQATHDNGAGKPVQGTSFHFFSVGGEALELQGVVFNYRTKYPDGTIFPKNATVQSQ	180
BAC03041.1	VHSNGQATHDNGAGKPVQGTSFHFFSVGGEALELQGVVFNYRTKYPDGTIFPKNATVQSQ	180
BAC03047.1	VHSNGQATHDNGAGKPVQGTSFHFFSVGGEALELQGVVFNYRTKYPDGTIFPKNATVQSQ	180
BAC03053.1	VHSNGQATHDNGAGKPVQGTSFHFFSVGGEALELQGVVFNYRTKYPDGTIFPKNATVQSQ	180
BAC03059.1	VHSNGQATHDNGAGKPVQGTSFHFFSVGGEALELQGVVFNYRTKYPDGTIFPKNATVQSQ	180
BAC03071.1	VHSNGQATHDNGAGKPVQGTSFHFFSVGGEALELQGVVFNYRTKYPDGTIFPKNATVQSQ	180
BAC03077.1	VHSNGQATHDNGAGKPVQGTSFHFFSVGGEALELQGVVFNYRTKYPDGTIFPKNATVQSQ	180
BAC03083.1	VHSNGQATHDNGAGKPVQGTSFHFFSVGGEALELQGVVFNYRTKYPDGTIFPKNATVQSQ	180

```
BAC03089.1        VHSNGQATHDNGAGKPVQGTSFHFFSVGGEALELQGVVFNYRTKYPDGTIFPKNATVQSQ  180
AAM69542.1        VHSNGQATHDNGAGKPVQGTSFHFFSVGGEALELQGVVFNYRTKYPDGTIFPKNATVQSQ  180
AAM69548.1        VHSNGQATHDNGAGKPVQGTSFHFFSVGGEALELQGVVFNYRTKYPDGTIFPKNATVQSQ  180
AAM69554.1        VHSNGQATHDNGAGKPVQGTSFHFFSVGGEALELQGVVFNYRTKYPDGTIFPKNATVQSQ  180
AAM69619.1        VHSNGQATHDNGAGKPVQGTSFHFFSVGGEALELQGVVFNYRTKYPDGTIFPKNATVQSQ  180
BAB68977.1        VHSNGQATHDNGAGKPVQGTSFHFFSVGGEALELQGVVFNYRTKYPDGTIFPKNATVQSQ  180
BAB68983.1        VHSNGQATHDNGAGKPVQGTSFHFFSVGGEALELQGVVFNYRTKYPDGTIFPKNATVQSQ  180
BAB68989.1        VHSNGQATHDNGAGKPVQGTSFHFFSVGGEALELQGVVFNYRTKYPDGTIFPKNATVQSQ  180
BAB68995.1        VHSNGQATHDNGAGKPVQGTSFHFFSVGGEALELQGVVFNYRTKYPDGTIFPKNATVQSQ  180
AAK98000.1        VHSNGQATHDNGAGKPVQGTSFHFFSVGGEALELQGVVFNYRTKYPDGTIFPKNATVQSQ  180
AAK98006.1        VHSNGQATHDNGAGKPVQGTSFHFFSVGGEALELQGVVFNYRTKYPDGTIFPKNATVQSQ  180
AAK98010.1        VHSNGQATHDNGAGKPVQGTSFHFFSVGGEALELQGVVFNYRTKYPDGTIFPKNATVQSQ  180
AAK98018.1        VHSNGQATHDNGAGKPVQGTSFHFFSVGGEALELQGVVFNYRTKYPDGTIFPKNATVQSQ  180
AAK98024.1        VHSNGQATHDNGAGKPVQGTSFHFFSVGGEALELQGVVFNYRTKYPDGTIFPKNATVQSQ  180
AAK98030.1        VHSNGQATHDNGAGKPVQGTSFHFFSVGGEALELQGVVFNYRTKYPDGTIFPKNATVQSQ  180
AAK98036.1        VHSNGQATHDNGAGKPVQGTSFHFFSVGGEALELQGVVFNYRTKYPDGTIFPKNATVQSQ  180
AAK70258.1        VHSNGQATHDNGAGKPVQGTSFHFFSVGGEALELQGVVFNYRTKYPDGTIFPKNATVQSQ  180
AAK70264.1        VHSNGQATHDNGAGKPVQGTSFHFFSVGGEALELQGVVFNYRTKYPDGTIFPKNATVQSQ  180
AAG53896.1        VHSNGQATHDNGAGKPVQGTSFHFFSVGGEALELQGVVFNYRTKYPDGTIFPKNATVQSQ  180
BAA01962.1        VHSNGQATHDNGAGKPVQGTSFHFFSVGGEALELQGVVFNYRTKYPDGTIFPKNATVQSQ  180
ALF06256.1        VHSNGQATHDNGAGKPVQGTSFHFFSVGGEALELQGVVFNYRTKYPDGTIFPKNATVQSQ  180
BAD06060.1        VHSNGQATHDNGAGKPVQGTSFHFFSVGGEALELQGVVFNYRTKYPDGTIFPKNATVQSQ  180
BAD06072.1        VHSNGQATHDNGAGKPVQGTSFHFFSVGGEALELQGVVFNYRTKYPDGTIFPKNATVQSQ  180
BAD06156.1        VHSNGQATHDNGAGKPVQGTSFHFFSVGGEALELQGVVFNYRTKYPDGTIFPKNATVQSQ  180
BAD06174.1        VHSNGQATHDNGAGKPVQGTSFHFFSVGGEALELQGVVFNYRTKYPDGTIFPKNATVQSQ  180
AAN85449.1        VHSNGQATHDNGAGKPVQGTSFHFFSVGGEALELQGVVFNYRTKYPDGTIFPKNATVQSQ  180
AAN85455.1        VHSNGQATHDNGAGKPVQGTSFHFFSVGGEALELQGVVFNYRTKYPDGTIFPKNATVQSQ  180
AAK98042.1        VHSNGQATHDNGAGKPVQGTSFHFFSVGGEALELQGVVFNYRTKYPDGTIFPKNATVQSQ  180
BAD06036.1        VHSNGQATHDNGAGKPVQGTSFHFFSVGGEALELQGVLFNYRTKYPDGTIFPKNATVQSQ  180
BCP65063.1        VHSNGQATHDNGAGKPVQGTSFHFFSVGGEALELQGVVFNYRTKYPDGTIFPKNATVQSQ  180
BCP56477.1        VHSNGQATHDNGAGKPVQGTSFHFFSVGGEALELQGVVFNYRTKYPDGTIFPKNATVQSQ  180
BAV69373.1        VHSNGQATHDNGAGKPVQGTSFHFFSVGGEALELQGVVFNYRTKYPDGTIFPKNATVQSQ  180
BAE00153.1        VHSNGQATHDNGAGKPVQGTSFHFFSVGGEALELQGVVFNYRTKYPDGTIFPKNATVQSQ  180
BAC22906.1        VHSNGQATHDNGAGKPVQGTSFHFFSVGGEALELQGVVFNYRTKYPDGTIFPKNATVQSQ  180
BAA05639.1        VHSNGQATHDNGAGKPVQGTSFHFFSVGGEALELQGVVFNYRTKYPDGTIFPKNATVQSQ  180
BAB11728.1        VHSNGQATHDNGAGKPVQGTSFHFFSVGGEALELQGVVFNYRTKYPDGTIFPKNATVQSQ  180
BAA05637.1        VHSNGQATHDNGAGKPVQGTSFHFFSVGGEALELQGVVFNYRTKYPDGTIFPKNATVQSQ  180
BAA01969.1        VHSNGQATHDNGAGKPVQGTSFHFFSVGGEALELQGVVFNYRTKYPDGTIFPKNATVQSQ  180
BAE00159.1        VHSNGQATHDNGAGKPVQGTSFHFFSVGGEALELQGVVFNYRTKYPDGTIFPKNATVQSQ  180
BAE00165.1        VHCNGQATHDNGAGKPVQGTSFHFFSVGGEALELQGVVFNYRTKYPDGTIFPKNATVQSQ  180
BAE00171.1        VHCNGQATHDNGAGKPVQGTSFHFFSVGGEALELQGVVFNYRTKYPDGTIFPKNATVQSQ  180
ALF06268.1        VHSNGQATHDNGAGKPVQGTSFHFFSVGGEALELQGVVFNYRTKYPDGTIFPKNATVQSQ  180
AYM55586.1        VHSNGQATHDNGAGKPVQGTSFHFFSVGGEALELQGVVFNYRTKYPDGTIFPKNATVQSQ  180
AYM55585.1        VHSNGQATHDNGAGKPVQGTSFLFFSVGGEALELQGVVFIYRTKYPDGAIFPKNATVQSQ  180
AYM55587.1        VHSNGQATHDNGAGKPVQGTSFLFFSVGGEALELQGVVFNYRTKYPDGTIFPKNATVQSQ  180
AYM55594.1        VHSNGQATHDNGAGKPVQGTSFHFFSVGGEALELQGVVFNYRTKYPDGTIFPKNATVQSQ  180
QBQ65470.1        VHSNGQATHDNGAGKPVQGTSFHFFSVGGEALELQGVLFNYRTKYPDGTIFPKNATVQSQ  180
CCW72501.1        VHSNGQAAHDNGAAKPVQGTSFHFFSVGGEALELQGVVFNYRTTYPDGTIFPKNATVQSQ  180
CCW72524.1        VHSNGQATHDNGAAKPVQGTSFHFFSVGGEALELQGVVFNYRTTYPDGTIFPKNATVQSQ  180
QWT77116.1        VHSNGQAAHDNGAAKPVQGTSFHFFSVGGEALELQGVVFNYRTTYPDGTIFPKNATVQSQ  180
WLD48046.1        VHSNGQASHDNGAAKPVQGTSFHFFSVGGEALELQGVVFNYRTTYPDGTIFPKNATVQSQ  180
WLD48052.1        VHSNGQASHDNGAAKPVQGTSFHFFSVGGEALELQGVVFNYRTTYPDGTIFPKNATVQSQ  180
AAK70288.1        VHSNGQASHDNGAAKPVQGTSFHFFSVGGEALELQGVVFNYRTTYPDGTIFPKNATVQSQ  180
AAK70270.1        VHSNGQATHDNGAAKPVQGTSFHFFSVGGEALELQGVVFNYRTTYPDGTIFPKNATVQSQ  180
BAD21277.2        VHSNGQATHDNGAAKPVQGTSFHFFSVGGEALELQGVVFNYRTTYPHGTIFPKNATVQSQ  180
CCW72498.1        VHSNGQAAHDXGAAKPVQGTSFHFFSVGGEALELQGVVFNYRTTYPDGTIFPKNATVQSQ  180
UPN63085.1        VHSNGQAAHDNGAAKPVQGTSFHFFSVGGEALELQGVVFNYRTTYPDGTIFPKNATVQSQ  180
UPN63090.1        VHSNGQAAHDNGAAKPVQGTSFHFFSVGGEALELQGVVFNYRTTYPDGTIFPKNATVQSQ  180
QWT77134.1        VHSNGQAAHDNGAAKPVQGTSFHFFSVGGEALELQGVVFNYRTTYPDGTIFPKNATVQSQ  180
WLD48070.1        VHSNGQAAHDNGAAKPVQGTSFHFFSVGGEALELQGVVFNYRTTYPDGTIFPKNATVQSQ  180
AAK70282.1        VHSNGQATHDNGAAKPVQGTSFHFFSVGGEALELQGVVFNYRTTYPDGTIFPKNATVQSQ  180
WLD48058.1        VHSNGQAAHDNGAAKPVQGTSFHFFSVGGEALELQGVVFNYRTTYPDGTIFPKNATVQSQ  180
WLD48076.1        VHSNGQAAHDNGAAKPVQGTSFHFFSVGGEALELQGVVFNYRTTYPDGTIFPKNATVQSQ  180
UQK62667.1        VHSNGQATHDNGAAKPVQGTSFHFFSVGGEALELQGVVFNYRTTYPDGTIFPKNATVQSQ  180
```

```
CCW72480.1      VHSNGQATHDNGAAKPVQGTSFHFFSVGGEALELQGVVFNYRTTYPDGTIFPKNATVQSQ  180
CCW72513.1      VHSNGQASHDNGAAKPVQGTSFHFFSVGGEALELQGVVFNYRTTYPDGTIFPKNATVQSQ  180
AAM69614.1      VHSNGQATHDNGAGKPVQGTSFHFFSVGGEALELQGVVFNYRTTYPDGTIFPKNATVQSQ  180
AAK70318.1      VHSNGQATHDNGAGKPVQGTSFHFFSVGGEALELQGVVFNYRTTYPDGTIFPKNATVQSQ  180
AAK70324.1      VHSNGQATHDNGAGKPVQGTSFHFFSVGGEALELQGVVFNYRTTYPDGTIFPKNATVQSQ  180
AAT09837.1      VHSNGQATHDNGAAKPVQGTSFHFFSVGGEALELQGVVFNYRTTYPDGTIFPKNATVQSQ  180
AAT09831.1      VHSNGQATHDNGAAKPVQGTSFHFFSVGGEALELQGVVFNYRTTYPDGTIFPKNATVQSQ  180
AAT09825.1      VHSNGQATHDNGAAKPVQGTSFHFFSVGGEALELQGVVFNYRTTYPDGTIFPKNATVQSQ  180
AAR89271.1      VHSNGQATHDNGAAKPVQGTSFHFFSVGGEALELQGVVFNYRTTYPDGTIFPKNATVQSQ  180
AAR89211.1      VHSNGQATHDNGAAKPVQGTSFHFFSVGGEALELQGVVFNYRTTYPDGTIFPKNATVQSQ  180
BAE02956.1      VHSNGQATHDNGAAKPVQGTSFHFFSVGGEALELQGVVFNYRTTYPDGTIFPKNATVQSQ  180
ACF75876.1      VHSNGQATHDNGAAKPVQGTSFHFFSVGGEALELQGVVFNYRTTYPDGTIFPKNATVQSQ  180
QBM11924.1      VHSNGQATHDNGAAKPVQGTSFHFFSVGGEALELQGVVFNYRTTYPDGTIFPKNATVQSQ  180
QBM11858.1      VHSNGQATHDNGAAKPVQGTSFHFFSVGGEALELQGVVFNYRTTYPDGTIFPKNATVQSQ  180
QBQ04203.1      VHSNGQATQDNGAAKPVQGTSFHFFSVGGEALELQGVVFNYRTTYPDGTIFPKNATVQSQ  180
QBQ65473.1      VHSNGQATHDNGAAKPVQGTSFHFFSVGGEALELQGVVFNYRTTYPDGTIFPKNATVQSQ  180
QBQ65472.1      VHSNGQATQDNGAAKPVQGTSFHFFSVGGEALELQGVVFNYRTTYPDGTIFPKNATVQSQ  180
QBM11920.1      VHSNGQATHDNGAAKPVQGTSFHFFSVGGEALELQGVVFNYRTTYPDGTIFPKNATVQSQ  180
QAA95693.1      VHSNGQATHDNGAAKPVQGTSFHFFSVGGEALELQGVVFNYRTTYPDGTIFPKNATVQSQ  180
QBX89122.1      VHSNGQATHDNGAAKPVQGTSFHFFSVGGEALELQGVVFNYRTTYPDGTIFPKNATVQSQ  180
QID57946.1      VHSNGQATHDNGAAKPVQGTSFHFFSVGGEALELQGVVFNYRTTYPDGTIFPKNATVQSQ  180
QID57952.1      VHSNGQATHDNGAAKPVQGTSFHFFSVGGEALELQGVVFNYRTTYPDGTIFPKNATVQSQ  180
QID57958.1      VHSNGQATHDNGAAKPVQGTSFHFFSVGGEALELQGVVFNYRTTYPDGTIFPKNATVQSQ  180
QID57964.1      VHSNGQATHDNGAAKPVQGTSFHFFSVGGEALELQGVVFNYRTTYPDGTIFPKNATVQSQ  180
QID57970.1      VHSNGQATHDNGAAKPVQGTSFHFFSVGGEALELQGVVFNYRTTYPDGTIFPKNATVQSQ  180
QID57976.1      VHSNGQATHDNGAAKPVQGTSFHFFSVGGEALELQGVVFNYRTTYPDGTIFPKNATVQSQ  180
QCH41110.1      VHSNGQATHDNGAAKPVQGTSFHFFSVGGEALELQGVVFNYRTTYPDGTIFPKNATVQSQ  180
QBX89120.1      VHSNGQATHDNGAAKPVQGTSFHFFSVGGEALELQGVVFNYRTTYPDGTIFPKNATVQSQ  180
QBX89121.1      VHSNGQATHDNGAAKPVQGTSFHFFSVGGEALELQGVVFNYRTTYPDGTIFPKNATVQSQ  180
QBX89124.1      VHSNGQATHDNGAAKPVQGTSFHFFSVGGEALELQGVVFNYRTTYPDGTIFPKNATVQSQ  180
QBQ65471.1      VHSNGQATHDNGAAKPVQGTSFHFFSVGGEALELQGVVFNYRTTYPDGTIFPKNATVQSQ  180
QBQ65474.1      VHSNGQATHDNGAAKPVQGTSFHFFSVGGEALELQGVVFNYRTTYPDGTIFPKNATVQSQ  180
QBQ65476.1      VHSNGQATHDNGAAKPVQGTSFHFFSVGGEALELQGVVFNYRTTYPDGTIFPKNATVQSQ  180
QBQ04201.1      VHSNGQATHDNGAAKPVQGTSFHFFSVGGEALELQGVVFNYRTTYPDGTIFPKNATVQSQ  180
QBM11921.1      VHSNGQATHDNGAAKPVQGTSFHFFSVGGEALELQGVVFNYRTTYPDGTIFPKNATVQSQ  180
QBM11922.1      VHSNGQATHDNGAAKPVQGTSFHFFSVGGEALELQGVVFNYRTTYPDGTIFPKNATVQSQ  180
CDJ79845.1      VHSNGQATHDNGAAKPVQGTSFHFFSVGGEALELQGVVFNYRTTYPDGTIFPKNATVQSQ  180
BAE02932.1      VHSNGQATHDNGAAKPVQGTSFHFFSVGGEALELQGVVFNYRTTYPDGTIFPKNATVQSQ  180
BAE02938.1      VHSNGQATHDNGAAKPVQGTSFHFFSVGGEALELQGVVFNYRTTYPDGTIFPKNATVQSQ  180
BAE02950.1      VHSNGQATHDNGAAKPVQGTSFHFFSVGGEALELQGVVFNYRTTYPDGTIFPKNATVQSQ  180
BAE03004.1      VHSNGQATHDNGAAKPVQGTSFHFFSVGGEALELQGVVFNYRTTYPDGTIFPKNATVQSQ  180
BAE03118.1      VHSNGQATHDNGAAKPVQGTSFHFFSVGGEALELQGVVFNYRTTYPDGTIFPKNATVQSQ  180
BAE03124.1      VHSNGQATHDNGAAKPVQGTSFHFFSVGGEALELQGVVFNYRTTYPDGTIFPKNATVQSQ  180
BAE03130.1      VHSNGQATHDNGAAKPVQGTSFHFFSVGGEALELQGVVFNYRTTYPDGTIFPKNATVQSQ  180
BAE03136.1      VHSNGQATHDNGAAKPVQGTSFHFFSVGGEALELQGVVFNYRTTYPDGTIFPKNATVQSQ  180
BAE03142.1      VHSNGQATHDNGAAKPVQGTSFHFFSVGGEALELQGVVFNYRTTYPDGTIFPKNATVQSQ  180
BAE03148.1      VHSNGQATHDNGAAKPVQGTSFHFFSVGGEALELQGVVFNYRTTYPDGTIFPKNATVQSQ  180
BAE03154.1      VHSNGQATHDNGAAKPVQGTSFHFFSVGGEALELQGVVFNYRTTYPDGTIFPKNATVQSQ  180
QBQ04200.1      VHSNGQATHDNGAAKPVQGTSFHFFSVGGEALELQGVVFNYRTTYPDGTIFPKNATVQSQ  180
UYL83839.1      VHSNGQATHDNGAAKPVQGTSFHFFSVGGEALELQGVVFNYRTTYPDGTIFPKNATVQSQ  180
ALF06262.1      VHSNGQATHDNGAAKPVQGTSFHFFSVGGEALELQGVVFNYRTTYPDGTIFPKNATVQSQ  180
ABH10586.1      VHSNGQATHDNGAAKPVQGTSFHFFSVGGEALELQGVVFNYRTTYPDGTIFPKNATVQSQ  180
BAE02890.1      VHSNGQATHDNGAAKPVQGTSFHFFSVGGEALELQGVVFNYRTTYPDGTIFPKNATVQSQ  180
BAE02896.1      VHSNGQATHDNGAAKPVQGTSFHFFSVGGEALELQGVVFNYRTTYPDGTIFPKNATVQSQ  180
BAE02902.1      VHSNGQATHDNGAAKPVQGTSFHFFSVGGEALELQGVVFNYRTTYPDGTIFPKNATVQSQ  180
BAE02914.1      VHSNGQATHDNGAAKPVQGTSFHFFSVGGEALELQGVVFNYRTTYPDGTIFPKNATVQSQ  180
BAE02920.1      VHSNGQATHDNGAAKPVQGTSFHFFSVGGEALELQGVVFNYRTTYPDGTIFPKNATVQSQ  180
BAE02944.1      VHSNGQATHDNGAAKPVQGTSFHFFSVGGEALELQGVVFNYRTTYPDGTIFPKNATVQSQ  180
BAE02962.1      VHSNGQATHDNGAAKPVQGTSFHFFSVGGEALELQGVVFNYRTTYPDGTIFPKNATVQSQ  180
BAE02968.1      VHSNGQATHDNGAAKPVQGTSFHFFSVGGEALELQGVVFNYRTTYPDGTIFPKNATVQSQ  180
BAE02974.1      VHSNGQATHDNGAAKPVQGTSFHFFSVGGEALELQGVVFNYRTTYPDGTIFPKNATVQSQ  180
BAE02980.1      VHSNGQATHDNGAAKPVQGTSFHFFSVGGEALELQGVVFNYRTTYPDGTIFPKNATVQSQ  180
BAE02986.1      VHSNGQATHDNGAAKPVQGTSFHFFSVGGEALELQGVVFNYRTTYPDGTIFPKNATVQSQ  180
BAE02992.1      VHSNGQATHDNGAAKPVQGTSFHFFSVGGEALELQGVVFNYRTTYPDGTIFPKNATVQSQ  180
```

```
BAE02998.1      VHSNGQATHDNGAAKPVQGTSFHFFSVGGEALELQGVVFNYRTTYPDGTIFPKNATVQSQ  180
BAE03010.1      VHSNGQATHDNGAAKPVQGTSFHFFSVGGEALELQGVVFNYRTTYPDGTIFPKNATVQSQ  180
BAE03016.1      VHSNGQATHDNGAAKPVQGTSFHFFSVGGEALELQGVVFNYRTTYPDGTIFPKNATVQSQ  180
BAE03022.1      VHSNGQATHDNGAAKPVQGTSFHFFSVGGEALELQGVVFNYRTTYPDGTIFPKNATVQSQ  180
BAE03028.1      VHSNGQATHDNGAAKPVQGTSFHFFSVGGEALELQGVVFNYRTTYPDGTIFPKNATVQSQ  180
BAE03034.1      VHSNGQATHDNGAAKPVQGTSFHFFSVGGEALELQGVVFNYRTTYPDGTIFPKNATVQSQ  180
BAE03040.1      VHSNGQATHDNGAAKPVQGTSFHFFSVGGEALELQGVVFNYRTTYPDGTIFPKNATVQSQ  180
BAE03046.1      VHSNGQATHDNGAAKPVQGTSFHFFSVGGEALELQGVVFNYRTTYPDGTIFPKNATVQSQ  180
BAE03052.1      VHSNGQATHDNGAAKPVQGTSFHFFSVGGEALELQGVVFNYRTTYPDGTIFPKNATVQSQ  180
BAE03058.1      VHSNGQATHDNGAAKPVQGTSFHFFSVGGEALELQGVVFNYRTTYPDGTIFPKNATVQSQ  180
BAE03064.1      VHSNGQATHDNGAAKPVQGTSFHFFSVGGEALELQGVVFNYRTTYPDGTIFPKNATVQSQ  180
BAE03070.1      VHSNGQATHDNGAAKPVQGTSFHFFSVGGEALELQGVVFNYRTTYPDGTIFPKNATVQSQ  180
BAE03076.1      VHSNGQATHDNGAAKPVQGTSFHFFSVGGEALELQGVVFNYRTTYPDGTIFPKNATVQSQ  180
BAE03082.1      VHSNGQATHDNGAAKPVQGTSFHFFSVGGEALELQGVVFNYRTTYPDGTIFPKNATVQSQ  180
BAE03088.1      VHSNGQATHDNGAAKPVQGTSFHFFSVGGEALELQGVVFNYRTTYPDGTIFPKNATVQSQ  180
BAE03094.1      VHSNGQATHDNGAAKPVQGTSFHFFSVGGEALELQGVVFNYRTTYPDGTIFPKNATVQSQ  180
BAE03100.1      VHSNGQATHDNGAAKPVQGTSFHFFSVGGEALELQGVVFNYRTTYPDGTIFPKNATVQSQ  180
BAE03106.1      VHSNGQATHDNGAAKPVQGTSFHFFSVGGEALELQGVVFNYRTTYPDGTIFPKNATVQSQ  180
BAE03112.1      VHSNGQATHDNGAAKPVQGTSFHFFSVGGEALELQGVVFNYRTTYPDGTIFPKNATVQSQ  180
BAE03160.1      VHSNGQATHDNGAAKPVQGTSFHFFSVGGEALELQGVVFNYRTTYPDGTIFPKNATVQSQ  180
BAE03166.1      VHSNGQATHDNGAAKPVQGTSFHFFSVGGEALELQGVVFNYRTTYPDGTIFPKNATVQSQ  180
AAQ88264.1      VHSNGQATHDNGAAKPVQGTSFHFFSVGGEALELQGVVFNYRTTYPDGTIFPKNATVQSQ  180
AAQ88270.1      VHSNGQATHDNGAAKPVQGTSFHFFSVGGEALELQGVVFNYRTTYPDGTIFPKNATVQSQ  180
AAR89187.1      VHSNGQATHDNGAAKPVQGTSFHFFSVGGEALELQGVVFNYRTTYPDGTIFPKNATVQSQ  180
AAR89193.1      VHSNGQATHDNGAAKPVQGTSFHFFSVGGEALELQGVVFNYRTTYPDGTIFPKNATVQSQ  180
AAR89199.1      VHSNGQATHDNGAAKPVQGTSFHFFSVGGEALELQGVVFNYRTTYPDGTIFPKNATVQSQ  180
AAR89205.1      VHSNGQATHDNGAAKPVQGTSFHFFSVGGEALELQGVVFNYRTTYPDGTIFPKNATVQSQ  180
AAR89217.1      VHSNGQATHDNGAAKPVQGTSFHFFSVGGEALELQGVVFNYRTTYPDGTIFPKNATVQSQ  180
AAR89223.1      VHSNGQATHDNGAAKPVQGTSFHFFSVGGEALELQGVVFNYRTTYPDGTIFPKNATVQSQ  180
AAR89229.1      VHSNGQATHDNGAAKPVQGTSFHFFSVGGEALELQGVVFNYRTTYPDGTIFPKNATVQSQ  180
AAR89235.1      VHSNGQATHDNGAAKPVQGTSFHFFSVGGEALELQGVVFNYRTTYPDGTIFPKNATVQSQ  180
AAR89241.1      VHSNGQATHDNGAAKPVQGTSFHFFSVGGEALELQGVVFNYRTTYPDGTIFPKNATVQSQ  180
AAR89247.1      VHSNGQATHDNGAAKPVQGTSFHFFSVGGEALELQGVVFNYRTTYPDGTIFPKNATVQSQ  180
AAR89253.1      VHSNGQATHDNGAAKPVQGTSFHFFSVGGEALELQGVVFNYRTTYPDGTIFPKNATVQSQ  180
AAR32743.1      VHSNGQATHDNGAAKPVQGTSFHFFSVGGEALELQGVVFNYRTTYPDGTIFPKNATVQSQ  180
AAR89265.1      VHSNGQATHDNGAAKPVQGTSFHFFSVGGEALELQGVVFNYRTTYPDGTIFPKNATVQSQ  180
AAR89277.1      VHSNGQATHDNGAAKPVQGTSFHFFSVGGEALELQGVVFNYRTTYPDGTIFPKNATVQSQ  180
AAR89283.1      VHSNGQATHDNGAAKPVQGTSFHFFSVGGEALELQGVVFNYRTTYPDGTIFPKNATVQSQ  180
AAR02463.1      VHSNGQATHDNGAAKPVQGTSFHFFSVGGEALELQGVVFNYRTTYPDGTIFPKNATVQSQ  180
AAR06661.1      VHSNGQATHDNGAAKPVQGTSFHFFSVGGEALELQGVVFNYRTTYPDGTIFPKNATVQSQ  180
AAR13659.1      VHSNGQATHDNGAAKPVQGTSFHFFSVGGEALELQGVVFNYRTTYPDGTIFPKNATVQSQ  180
AAR02457.1      VHSNGQATHDNGAAKPVQGTSFHFFSVGGEALELQGVVFNYRTTYPDGTIFPKNATVQSQ  180
BAB68785.1      VHSNGQATHDNGAAKPVQGTSFHFFSVGGEALELQGVVFNYRTTYPDGTIFPKNATVQSQ  180
AAK28472.1      VHSNGQATHDNGAAKPVQGTSFHFFSVGGEALELQGVVFNYRTTYPDGTIFPKNATVQSQ  180
AAG34667.1      VHSNGQATHDNGAAKPVQGTSFHFFSVGGEALELQGVVFNYRTTYPDGTIFPKNATVQSQ  180
AAG34673.1      VHSNGQATHDNGAAKPVQGTSFHFFSVGGEALELQGVVFNYRTTYPDGTIFPKNATVQSQ  180
AAB41705.1      VHSNGQATHDNGAAKPVQGTSFHFFSVGGEALELQGVVFNYRTTYPDGTIFPKNATVQSQ  180
AAB41711.1      VHSNGQATHDNGAAKPVQGTSFHFFSVGGEALELQGVVFNYRTTYPDGTIFPKNATVQSQ  180
AAB41717.1      VHSNGQATHDNGAAKPVQGTSFHFFSVGGEALELQGVVFNYRTTYPDGTIFPKNATVQSQ  180
BAE02926.1      VHSNGQATHDNGAAKPVQGTSFHFFSVGGEALELQGVVFNYRTKYPDGTIFPKNATVQSQ  180
                **.*        * * *:.*** ** **     *:*    : : **.  *:*     :**

AEC14619.1      VMNTEHKAYLDKNKAYPVECWVPDPXRXENTRYXXTXTGGENVPPVLHITNTATTVLLDE  240
AEC15199.1      VMNTEHKAYLDKNKAYPVECWVPDPTRNENTRYFGTLTGGENVPPVLHITNTATTVLLDE  240
AEC15209.1      VMNTEHKAYLDKNKAYPVECWVPDPTRNENTRYFGTLTGGENVPPVLHITNTATTVLLDE  240
UPN63098.1      VMNTEHKAYLDKNKAYPVECWVPDPTRNENTRYFGTLTGGENVPPVLHITNTATTVLLDE  240
UPN63097.1      VMNTEHKAYLDKNKAYPVECWVPDPTRNENTRYFGTLTGGENVPPVLHITNTATTVLLDE  240
UPN63094.1      VMNTEHKAYLDKNKAYPVECWVPDPTRNENTRYFGTLTGGENVPPVLHIPNTATTVLLDE  240
UPN63096.1      VMNTEHKAYLDKNKAYPVECWVPDPTRNENTRYFGTLTGGENVPPVLHITNTATTVLLDE  240
QWT77146.1      VMNTEHKAYLDKNKAYPVECWVPDPTRNENTRYFGTLTGGENVPPVLHITNTATTVLLDE  240
UPN63095.1      VMNTEHKAYLDKNKAYPVECWVPDPTRNENTRYFGTLTGGENVPPVLHITNTATTVLLDE  240
BAA01966.1      VMNTEHKAYLDKNKAYPVECWVPDPTRNENTRYFGTLTGGENVPPVLHITNTATTVLLDE  240
AEC15099.1      VMNTEHKAYLDKNKAYPVECWVPDPTRNENTRYFGTLTGGENVPSVLHITNTATTVLLDE  240
UPN63099.1      VMNTEHKAYLDKNKAYPVECWVPDPTRNENTRYFGTLTGGENVPPVLHITNTATTVLLDE  240
```

UPN63086.1	VMNTEHKAYLDKNKAYPVECWVPDPTRNENTRYFGTLTGGENVPPVLHITNTATTVLLDE	240
UPN63092.1	VMNTEHKAYLDKNKAYPVECWVPDPTRNENTRYFGTLTGGENVPPVLHITNTATTVLLDE	240
AEC15783.1	VMNTEHKAYLDKNKAYPVECWVPDPTRNENTRYFGTLTGGENVPPVLHITNTATTVLLDE	240
AEC15735.1	VMNTEHKAYLDKNKAYPVECWVPDPTRNENTRYSGTLTGGENVPPVLHITNTATTVLLDE	240
AEC15615.1	VMNTEHKAYLDKNKAYPVECWVPDPTRNENTRYFGTLTGGENVPPVLHITNTATTVLLDE	240
AEC15549.1	VMNTEHKAYLDKNKAYPVECWVPDPTRNENTRYFGTLTGGENVPPVLHITNTATTVLLDE	240
AEC15081.1	VMNTEHKAYLDKNKAYPVECWVPDPTRNENTRYFGTLTGGENVPPVLHITNTATTVLLDE	240
AEC15080.1	VMNTEHKAYLDKNKAYPVECWVPDPTRNENTRYFGTLTGGENVPPVLHITNTATTVLLDE	240
AEC15083.1	VMNTEHKAYLDKNKAYPVECWVPDPTRNENTRYFGTLTGGENVPPVLHITNTATTVLLDE	240
QWT77109.1	VMNTEHKAYLDKNKAYPVECWVPDPTRNENTRYFGTLTGGENVPPVLHITNTATTVLLDE	240
AEC15069.1	VMNTEHKAYLDKNKAYPVECWVPDPTRNENTRYFGTLTGGENVPPVLHITNTATTVLLDE	240
AEC15070.1	VMNTEHKAYLDKNKAYPVECWVPDPTRNENTRYFGTLTGGENVPPVLHITNTATTVLLDE	240
AEC15071.1	VMNTEHKAYLDKNKAYPVECWVPDPTRNENTRYFGTLTGGENVPPVLHITNTATTVLLDE	240
AEC15072.1	VMNTEHKAYLDKNKAYPVECWVPDPTRNENTRYFGTLTGGENVPPVLHITNTATTVLLDE	240
AEC15073.1	VMNTEHKAYLDKNKAYPVECWVPDPTRNENTRYFGTLTGGENVPPVLHITNTATTVLLDE	240
AEC15074.1	VMNTEHKAYLDKNKAYPVECWVPDPTRNENTRYFGTLTGGENVPPVLHITNTATTVLLDE	240
AEC15076.1	VMNTEHKAYLDKNKAYPVECWVPDPTRNENTRYFGTLTGGENVPPVLHITNTATTVLLDE	240
AEC15077.1	VMNTEHKAYLDKNKAYPVECWVPDPTRNENTRYFGTLTGGENVPPVLHITNTATTVLLDE	240
AEC15078.1	VMNTEHKAYLDKNKAYPVECWVPDPTRNENTRYFGTLTGGENVPPVLHITNTATTVLLDE	240
AEC15079.1	VMNTEHKAYLDKNKAYPVECWVPDPTRNENTRYFGTLTGGENVPPVLHITNTATTVLLDE	240
AEC15082.1	VMNTEHKAYLDKNKAYPVECWVPDPTRNENTRYFGTLTGGENVPPVLHITNTATTVLLDE	240
AEC15084.1	VMNTEHKAYLDKNKAYPVECWVPDPTRNENTRYFGTLTGGENVPPVLHITNTATTVLLDE	240
AEC15085.1	VMNTEHKAYLDKNKAYPVECWVPDPTRNENTRYFGTLTGGENVPPVLHITNTATTVLLDE	240
AEC15086.1	VMNTEHKAYLDKNKAYPVECWVPDPTRNENTRYFGTLTGGENVPPVLHITNTATTVLLDE	240
AEC15087.1	VMNTEHKAYLDKNKAYPVECWVPDPTRNENTRYFGTLTGGENVPPVLHITNTATTVLLDE	240
AEC15089.1	VMNTEHKAYLDKNKAYPVECWVPDPTRNENTRYFGTLTGGENVPPVLHITNTATTVLLDE	240
AEC15555.1	VMNTEHKAYLDKNKAYPVECWVPDPTRNENTRYFGTLTGGENVPPVLHITNTATTVLLDE	240
AEC15561.1	VMNTEHKAYLDKNKAYPVECWVPDPTRNENTRYFGTLTGGENVPPVLHITNTATTVLLDE	240
AEC15567.1	VMNTEHKAYLDKNKAYPVECWVPDPTRNENTRYFGTLTGGENVPPVLHITNTATTVLLDE	240
AEC15573.1	VMNTEHKAYLDKNKAYPVECWVPDPTRNENTRYFGTLTGGENVPPVLHITNTATTVLLDE	240
AEC15579.1	VMNTEHKAYLDKNKAYPVECWVPDPTRNENTRYFGTLTGGENVPPVLHITNTATTVLLDE	240
AEC15585.1	VMNTEHKAYLDKNKAYPVECWVPDPTRNENTRYFGTLTGGENVPPVLHITNTATTVLLDE	240
AEC15591.1	VMNTEHKAYLDKNKAYPVECWVPDPTRNENTRYFGTLTGGENVPPVLHITNTATTVLLDE	240
AEC15597.1	VMNTEHKAYLDKNKAYPVECWVPDPTRNENTRYFGTLTGGENVPPVLHITNTATTVLLDE	240
AEC15603.1	VMNTEHKAYLDKNKAYPVECWVPDPTRNENTRYFGTLTGGENVPPVLHITNTATTVLLDE	240
AEC15609.1	VMNTEHKAYLDKNKAYPVECWVPDPTRNENTRYFGTLTGGENVPPVLHITNTATTVLLDE	240
AEC15621.1	VMNTEHKAYLDKNKAYPVECWVPDPTRNENTRYFGTLTGGENVPPVLHITNTATTVLLDE	240
AEC15627.1	VMNTEHKAYLDKNKAYPVECWVPDPTRNENTRYFGTLTGGENVPPVLHITNTATTVLLDE	240
AEC15633.1	VMNTEHKAYLDKNKAYPVECWVPDPTRNENTRYFGTLTGGENVPPVLHITNTATTVLLDE	240
AEC15639.1	VMNTEHKAYLDKNKAYPVECWVPDPTRNENTRYFGTLTGGENVPPVLHITNTATTVLLDE	240
AEC15645.1	VMNTEHKAYLDKNKAYPVECWVPDPTRNENTRYFGTLTGGENVPPVLHITNTATTVLLDE	240
AEC15651.1	VMNTEHKAYLDKNKAYPVECWVPDPTRNENTRYFGTLTGGENVPPVLHITNTATTVLLDE	240
AEC15657.1	VMNTEHKAYLDKNKAYPVECWVPDPTRNENTRYFGTLTGGENVPPVLHITNTATTVLLDE	240
AEC15663.1	VMNTEHKAYLDKNKAYPVECWVPDPTRNENTRYFGTLTGGENVPPVLHITNTATTVLLDE	240
AEC15669.1	VMNTEHKAYLDKNKAYPVECWVPDPTRNENTRYFGTLTGGENVPPVLHITNTATTVLLDE	240
AEC15675.1	VMNTEHKAYLDKNKAYPVECWVPDPTRNENTRYFGTLTGGENVPPVLHITNTATTVLLDE	240
AEC15681.1	VMNTEHKAYLDKNKAYPVECWVPDPTRNENTRYFGTLTGGENVPPVLHITNTATTVLLDE	240
AEC15687.1	VMNTEHKAYLDKNKAYPVECWVPDPTRNENTRYFGTLTGGENVPPVLHITNTATTVLLDE	240
AEC15693.1	VMNTEHKAYLDKNKAYPVECWVPDPTRNENTRYFGTLTGGENVPPVLHITNTATTVLLDE	240
AEC15699.1	VMNTEHKAYLDKNKAYPVECWVPDPTRNENTRYFGTLTGGENVPPVLHITNTATTVLLDE	240
AEC15705.1	VMNTEHKAYLDKNKAYPVECWVPDPTRNENTRYFGTLTGGENVPPVLHITNTATTVLLDE	240
AEC15711.1	VMNTEHKAYLDKNKAYPVECWVPDPTRNENTRYFGTLTGGENVPPVLHITNTATTVLLDE	240
AEC15717.1	VMNTEHKAYLDKNKAYPVECWVPDPTRNENTRYFGTLTGGENVPPVLHITNTATTVLLDE	240
AEC15723.1	VMNTEHKAYLDKNKAYPVECWVPDPTRNENTRYFGTLTGGENVPPVLHITNTATTVLLDE	240
AEC15729.1	VMNTEHKAYLDKNKAYPVECWVPDPTRNENTRYFGTLTGGENVPPVLHITNTATTVLLDE	240
AEC15741.1	VMNTEHKAYLDKNKAYPVECWVPDPTRNENTRYFGTLTGGENVPPVLHITNTATTVLLDE	240
AEC15747.1	VMNTEHKAYLDKNKAYPVECWVPDPTRNENTRYFGTLTGGENVPPVLHITNTATTVLLDE	240
AEC15753.1	VMNTEHKAYLDKNKAYPVECWVPDPTRNENTRYFGTLTGGENVPPVLHITNTATTVLLDE	240
AEC15759.1	VMNTEHKAYLDKNKAYPVECWVPDPTRNENTRYFGTLTGGENVPPVLHITNTATTVLLDE	240
AEC15765.1	VMNTEHKAYLDKNKAYPVECWVPDPTRNENTRYFGTLTGGENVPPVLHITNTATTVLLDE	240
AEC15771.1	VMNTEHKAYLDKNKAYPVECWVPDPTRNENTRYFGTLTGGENVPPVLHITNTATTVLLDE	240
AEC15777.1	VMNTEHKAYLDKNKAYPVECWVPDPTRNENTRYFGTLTGGENVPPVLHITNTATTVLLDE	240
AEC15789.1	VMNTEHKAYLDKNKAYPVECWVPDPTRNENTRYFGTLTGGENVPPVLHITNTATTVLLDE	240
AEC15795.1	VMNTEHKAYLDKNKAYPVECWVPDPTRNENTRYFGTLTGGENVPPVLHITNTATTVLLDE	240

AEC15801.1	VMNTEHKAYLDKNKAYPVECWVPDPTRNENTRYFGTLTGGENVPPVLHITNTATTVLLDE	240
AEC15807.1	VMNTEHKAYLDKNKAYPVECWVPDPTRNENTRYFGTLTGGENVPPVLHITNTATTVLLDE	240
AEC15813.1	VMNTEHKAYLDKNKAYPVECWVPDPTRNENTRYFGTLTGGENVPPVLHITNTATTVLLDE	240
AEC15075.1	VMNTEHKAYLDKNKAYPVECWVPDPTRNENTRYFGTLTGGENVPPVLHITNTATTVLLDE	240
AEC15088.1	VMNTEHKAYLDKNKAYPVECWVPDPTRNENTRYFGTLTGGENVPPVLHITNTATTVLLDE	240
AEC14559.1	VMNTEHKAYLDKNKAYPVECWVPDPTRNENTRYFGTLTGGENVPPVLHITNTATTVLLDE	240
AEC15023.1	VMNTEHKAYLDKNKAYPVECWVPDPTRNENTRYFGTLTGGENVPPVLHITNTATTVLLDE	240
AEC14518.1	VMNTEHKAYLDKNKAYPVECWVPDPTRNENTRYFGTLTGGENVPPVLHITNTATTVLLDE	240
AEC14589.1	VMNTEHKAYLDKNKAYPVECWVPDPTRNENTRYFGTLTGGENVPPVLHITNTATTVLLDE	240
AEC14625.1	VMNTEHKAYLDKNKAYPVECWVPDPTRNENTRYFGTLTGGENVPPVLHITNTATTVLLDE	240
AEC14631.1	VMNTEHKAYLDKNKAYPVECWVPDPTRNENTRYFGTLTGGENVPPVLHITNTATTVLLDE	240
AEC14643.1	VMNTEHKAYLDKNKAYPVECWVPDPTRNENTRYFGTLTGGENVPPVLHITNTATTVLLDE	240
AEC14966.1	VMNTEHKAYLDKNKAYPVECWVPDPTRNENTRYFGTLTGGENVPPVLHITNTATTVLLDE	240
AEC14979.1	VMNTEHKAYLDKNKAYPVECWVPDPTRNENTRYFGTLTGGENVPPVLHITNTATTVLLDE	240
AEC14990.1	VMNTEHKAYLDKNKAYPVECWVPDPTRNENTRYFGTLTGGENVPPVLHITNTATTVLLDE	240
AEC14999.1	VMNTEHKAYLDKNKAYPVECWVPDPTRNENTRYFGTLTGGENVPPVLHITNTATTVLLDE	240
AEC15020.1	VMNTEHKAYLDKNKAYPVECWVPDPTRNENTRYFGTLTGGENVPPVLHITNTATTVLLDE	240
AEC14595.1	VMNTEHKAYLDKNKAYPVECWVPDPTRNENTRYFGTLTGGENVPPVLHITNTATTVLLDE	240
AEC14703.1	VMNTEHKAYLDKNKAYPVECWVPDPTRNENTRYFGTLTGGENVPPVLHITNTATTVLLDE	240
AEC15011.1	VMNTEHKAYLDKNKAYPVECWVPDPTRNENXRYFGTLTGGENVPPVLHITNTATTVLLDE	240
AEC15019.1	VMNTEHKAYLDKNKAYPVECWVPDPTRNENXRYFGTLTGGENVPPVLHITNTATTVLLDE	240
AEC15014.1	VMNTEHKAYLDKNKAYPVECWVPDPTRNENTRYFGTLTGGENVPPVLHITNTATTVLLDE	240
NP_043511.1	VMNTEHKAYLDKNKAYPVECWVPDPTRNENTRYFGTLTGGENVPPVLHITNTATTVLLDE	240
UPN63087.1	VMNTEHKAYLDKNKAYPVECWVPDPTRNENTRYFGTLTGGENVPPVLHITNTATTVLLDE	240
ASV51760.1	VMNTEHKAYLDKNKAYPVECWVPDPTRNENTRYFGTLTGGENVPPVLHITNTATTVLLDE	240
BAV69379.1	VMNTEHKAYLDKNKAYPVECWVPDPTRNENTRYFGTLTGGENVPPVLHITNTATTVLLDE	240
AEC15090.1	VMNTEHKAYLDKNKAYPVECWVPDPTRNENTRYFGTLTGGENVPPVLHITNTATTVLLDE	240
AEC15091.1	VMNTEHKAYLDKNKAYPVECWVPDPTRNENTRYFGTLTGGENVPPVLHITNTATTVLLDE	240
AEC15092.1	VMNTEHKAYLDKNKAYPVECWVPDPTRNENTRYFGTLTGGENVPPVLHITNTATTVLLDE	240
AEC15093.1	VMNTEHKAYLDKNKAYPVECWVPDPTRNENTRYFGTLTGGENVPPVLHITNTATTVLLDE	240
AEC15094.1	VMNTEHKAYLDKNKAYPVECWVPDPTRNENTRYFGTLTGGENVPPVLHITNTATTVLLDE	240
AEC15095.1	VMNTEHKAYLDKNKAYPVECWVPDPTRNENTRYFGTLTGGENVPPVLHITNTATTVLLDE	240
AEC15096.1	VMNTEHKAYLDKNKAYPVECWVPDPTRNENTRYFGTLTGGENVPPVLHITNTATTVLLDE	240
AEC15097.1	VMNTEHKAYLDKNKAYPVECWVPDPTRNENTRYFGTLTGGENVPPVLHITNTATTVLLDE	240
AEC15098.1	VMNTEHKAYLDKNKAYPVECWVPDPTRNENTRYFGTLTGGENVPPVLHITNTATTVLLDE	240
AEC15100.1	VMNTEHKAYLDKNKAYPVECWVPDPTRNENTRYFGTLTGGENVPPVLHITNTATTVLLDE	240
AEC15101.1	VMNTEHKAYLDKNKAYPVECWVPDPTRNENTRYFGTLTGGENVPPVLHITNTATTVLLDE	240
AEC15102.1	VMNTEHKAYLDKNKAYPVECWVPDPTRNENTRYFGTLTGGENVPPVLHITNTATTVLLDE	240
AEC15103.1	VMNTEHKAYLDKNKAYPVECWVPDPTRNENTRYFGTLTGGENVPPVLHITNTATTVLLDE	240
AEC15104.1	VMNTEHKAYLDKNKAYPVECWVPDPTRNENTRYFGTLTGGENVPPVLHITNTATTVLLDE	240
AEC15105.1	VMNTEHKAYLDKNKAYPVECWVPDPTRNENTRYFGTLTGGENVPPVLHITNTATTVLLDE	240
AEC15106.1	VMNTEHKAYLDKNKAYPVECWVPDPTRNENTRYFGTLTGGENVPPVLHITNTATTVLLDE	240
AEC15107.1	VMNTEHKAYLDKNKAYPVECWVPDPTRNENTRYFGTLTGGENVPPVLHITNTATTVLLDE	240
AEC15108.1	VMNTEHKAYLDKNKAYPVECWVPDPTRNENTRYFGTLTGGENVPPVLHITNTATTVLLDE	240
AEC15109.1	VMNTEHKAYLDKNKAYPVECWVPDPTRNENTRYFGTLTGGENVPPVLHITNTATTVLLDE	240
AEC15110.1	VMNTEHKAYLDKNKAYPVECWVPDPTRNENTRYFGTLTGGENVPPVLHITNTATTVLLDE	240
AEC15111.1	VMNTEHKAYLDKNKAYPVECWVPDPTRNENTRYFGTLTGGENVPPVLHITNTATTVLLDE	240
AEC15963.1	VMNTEHKAYLDKNKAYPVECWVPDPTRNENTRYFGTLTGGENVPPVLHITNTATTVLLDE	240
AEC15969.1	VMNTEHKAYLDKNKAYPVECWVPDPTRNENTRYFGTLTGGENVPPVLHITNTATTVLLDE	240
AEC15975.1	VMNTEHKAYLDKNKAYPVECWVPDPTRNENTRYFGTLTGGENVPPVLHITNTATTVLLDE	240
AEC15981.1	VMNTEHKAYLDKNKAYPVECWVPDPTRNENTRYFGTLTGGENVPPVLHITNTATTVLLDE	240
AEC15987.1	VMNTEHKAYLDKNKAYPVECWVPDPTRNENTRYFGTLTGGENVPPVLHITNTATTVLLDE	240
AEC15993.1	VMNTEHKAYLDKNKAYPVECWVPDPTRNENTRYFGTLTGGENVPPVLHITNTATTVLLDE	240
CAD69038.1	VMNTEHKAYLDKNKAYPVECWVPDPTRNENTRYFGTLTGGENVPPVLHITNTATTVLLDE	240
AAK70240.1	VMNTEHKAYLDKNKAYPVECWVPDPTRNENTRYFGTLTGGENVPPVLHITNTATTVLLDE	240
BAB11710.1	VMNTEHKAYLDKNKAYPVECWVPDPTRNENTRYFGTLTGGENVPPVLHITNTATTVLLDE	240
BAA01958.1	VMNTEHKAYLDKNKAYPVECWVPDPTRNENTRYFGTLTGGENVPPVLHITNTATTVLLDE	240
AAA82101.1	VMNTEHKAYLDKNKAYPVECWVPDPTRNENTRYFGTLTGGENVPPVLHITNTATTVLLDE	240
P03089.1	VMNTEHKAYLDKNKAYPVECWVPDPTRNENTRYFGTLTGGENVPPVLHITNTATTVLLDE	240
UPN63102.1	VMNTEHKAYLDKNKAYPVECWVPDPTRNENTRYFGTLTGGENVPPVLHITNTATTVLLDE	240
AEC15025.1	VMNTEHKAYLDKNKAYPVECWVPDPTRNENTRYFGTLTGGENVPPVLHITNTATTVLLDE	240
AEC15026.1	VMNTEHKAYLDKNKAYPVECWVPDPTRNENTRYFGTLTGGENVPPVLHITNTATTVLLDE	240
AEC15027.1	VMNTEHKAYLDKNKAYPVECWVPDPTRNENTRYFGTLTGGENVPPVLHITNTATTVLLDE	240
AEC15028.1	VMNTEHKAYLDKNKAYPVECWVPDPTRNENTRYFGTLTGGENVPPVLHITNTATTVLLDE	240

AEC15029.1	VMNTEHKAYLDKNKAYPVECWVPDPTRNENTRYFGTLTGGENVPPVLHITNTATTVLLDE	240
AEC15196.1	VMNTEHKAYLDKNKAYPVECWVPDPTRNENTRYFGTLTGGENVPPVLHITNTATTVLLDE	240
AEC15197.1	VMNTEHKAYLDKNKAYPVECWVPDPTRNENTRYFGTLTGGENVPPVLHITNTATTVLLDE	240
AEC15198.1	VMNTEHKAYLDKNKAYPVECWVPDPTRNENTRYFGTLTGGENVPPVLHITNTATTVLLDE	240
AEC15200.1	VMNTEHKAYLDKNKAYPVECWVPDPTRNENTRYFGTLTGGENVPPVLHITNTATTVLLDE	240
AEC15201.1	VMNTEHKAYLDKNKAYPVECWVPDPTRNENTRYFGTLTGGENVPPVLHITNTATTVLLDE	240
AEC15202.1	VMNTEHKAYLDKNKAYPVECWVPDPTRNENTRYFGTLTGGENVPPVLHITNTATTVLLDE	240
AEC15203.1	VMNTEHKAYLDKNKAYPVECWVPDPTRNENTRYFGTLTGGENVPPVLHITNTATTVLLDE	240
AEC15204.1	VMNTEHKAYLDKNKAYPVECWVPDPTRNENTRYFGTLTGGENVPPVLHITNTATTVLLDE	240
AEC15205.1	VMNTEHKAYLDKNKAYPVECWVPDPTRNENTRYFGTLTGGENVPPVLHITNTATTVLLDE	240
AEC15206.1	VMNTEHKAYLDKNKAYPVECWVPDPTRNENTRYFGTLTGGENVPPVLHITNTATTVLLDE	240
AEC15207.1	VMNTEHKAYLDKNKAYPVECWVPDPTRNENTRYFGTLTGGENVPPVLHITNTATTVLLDE	240
AEC15208.1	VMNTEHKAYLDKNKAYPVECWVPDPTRNENTRYFGTLTGGENVPPVLHITNTATTVLLDE	240
AEC15210.1	VMNTEHKAYLDKNKAYPVECWVPDPTRNENTRYFGTLTGGENVPPVLHITNTATTVLLDE	240
AEC15211.1	VMNTEHKAYLDKNKAYPVECWVPDPTRNENTRYFGTLTGGENVPPVLHITNTATTVLLDE	240
AEC15212.1	VMNTEHKAYLDKNKAYPVECWVPDPTRNENTRYFGTLTGGENVPPVLHITNTATTVLLDE	240
AEC15213.1	VMNTEHKAYLDKNKAYPVECWVPDPTRNENTRYFGTLTGGENVPPVLHITNTATTVLLDE	240
AEC15214.1	VMNTEHKAYLDKNKAYPVECWVPDPTRNENTRYFGTLTGGENVPPVLHITNTATTVLLDE	240
AEC15215.1	VMNTEHKAYLDKNKAYPVECWVPDPTRNENTRYFGTLTGGENVPPVLHITNTATTVLLDE	240
AEC15216.1	VMNTEHKAYLDKNKAYPVECWVPDPTRNENTRYFGTLTGGENVPPVLHITNTATTVLLDE	240
AEC15217.1	VMNTEHKAYLDKNKAYPVECWVPDPTRNENTRYFGTLTGGENVPPVLHITNTATTVLLDE	240
AEC15218.1	VMNTEHKAYLDKNKAYPVECWVPDPTRNENTRYFGTLTGGENVPPVLHITNTATTVLLDE	240
AEC15327.1	VMNTEHKAYLDKNKAYPVECWVPDPTRNENTRYFGTLTGGENVPPVLHITNTATTVLLDE	240
AEC15333.1	VMNTEHKAYLDKNKAYPVECWVPDPTRNENTRYFGTLTGGENVPPVLHITNTATTVLLDE	240
AEC15339.1	VMNTEHKAYLDKNKAYPVECWVPDPTRNENTRYFGTLTGGENVPPVLHITNTATTVLLDE	240
AEC15345.1	VMNTEHKAYLDKNKAYPVECWVPDPTRNENTRYFGTLTGGENVPPVLHITNTATTVLLDE	240
AEC15351.1	VMNTEHKAYLDKNKAYPVECWVPDPTRNENTRYFGTLTGGENVPPVLHITNTATTVLLDE	240
AEC15357.1	VMNTEHKAYLDKNKAYPVECWVPDPTRNENTRYFGTLTGGENVPPVLHITNTATTVLLDE	240
AEC15363.1	VMNTEHKAYLDKNKAYPVECWVPDPTRNENTRYFGTLTGGENVPPVLHITNTATTVLLDE	240
AEC15369.1	VMNTEHKAYLDKNKAYPVECWVPDPTRNENTRYFGTLTGGENVPPVLHITNTATTVLLDE	240
AEC15375.1	VMNTEHKAYLDKNKAYPVECWVPDPTRNENTRYFGTLTGGENVPPVLHITNTATTVLLDE	240
AEC15381.1	VMNTEHKAYLDKNKAYPVECWVPDPTRNENTRYFGTLTGGENVPPVLHITNTATTVLLDE	240
AEC15387.1	VMNTEHKAYLDKNKAYPVECWVPDPTRNENTRYFGTLTGGENVPPVLHITNTATTVLLDE	240
AEC15393.1	VMNTEHKAYLDKNKAYPVECWVPDPTRNENTRYFGTLTGGENVPPVLHITNTATTVLLDE	240
AEC15399.1	VMNTEHKAYLDKNKAYPVECWVPDPTRNENTRYFGTLTGGENVPPVLHITNTATTVLLDE	240
AEC15405.1	VMNTEHKAYLDKNKAYPVECWVPDPTRNENTRYFGTLTGGENVPPVLHITNTATTVLLDE	240
AEC15411.1	VMNTEHKAYLDKNKAYPVECWVPDPTRNENTRYFGTLTGGENVPPVLHITNTATTVLLDE	240
AEC15417.1	VMNTEHKAYLDKNKAYPVECWVPDPTRNENTRYFGTLTGGENVPPVLHITNTATTVLLDE	240
AEC15423.1	VMNTEHKAYLDKNKAYPVECWVPDPTRNENTRYFGTLTGGENVPPVLHITNTATTVLLDE	240
AEC15429.1	VMNTEHKAYLDKNKAYPVECWVPDPTRNENTRYFGTLTGGENVPPVLHITNTATTVLLDE	240
AEC15435.1	VMNTEHKAYLDKNKAYPVECWVPDPTRNENTRYFGTLTGGENVPPVLHITNTATTVLLDE	240
AEC15441.1	VMNTEHKAYLDKNKAYPVECWVPDPTRNENTRYFGTLTGGENVPPVLHITNTATTVLLDE	240
BAA01967.1	VMNTEHKAYLDKNKAYPVECWVPDPTRNENTRYFGTLTGGENVPPVLHITNTATTVLLDE	240
ASV51844.1	VMNTEHKAYLDKNKAYPVECWVPDPTRNENTRYFGTLTGGENVPPVLHITNTATTVLLDE	240
ASV51850.1	VMNTEHKAYLDKNKAYPVECWVPDPTRNENTRYFGTLTGGENVPPVLHITNTATTVLLDE	240
CCW72489.1	VMNTEHKAYLDKNKAYPVECWVPDPTRNENTRYFGTLTGGENVPPVLHITNTATTVLLDE	240
AEC15170.1	VMNTEHKAYLDKNKAYPVECWVPDPTRNENTRYFGTLTGGENVPPVLHITNTATTVLLDE	240
AEC15171.1	VMNTEHKAYLDKNKAYPVECWVPDPTRNENTRYFGTLTGGENVPPVLHITNTATTVLLDE	240
AEC15299.1	VMNTEHKAYLDKNKAYPVECWVPDPTRNENTRYFGTLTGGENVPPVLHITNTATTVLLDE	240
BAB93050.1	VMNTEHKAYLDKNKAYPVECWVPDPTRNENTRYFGTLTGGENVPPVLHITNTATTVLLDE	240
UPN63100.1	VMNTEHKAYLDKNKAYPVECWVPDPTRNENTRYFGTLTGGENVPPVLHITNTATTVLLDE	240
CCW72482.1	VMNTEHKAYLDKNKAYPVECWVPDPTRNENTRYFGTLTGGENVPPVLHITNTATTVLLDE	240
AEC15165.1	VMNTEHKAYLDKNKAYPVECWVPDPTRNENTRYFGTLTGGENVPPVLHITNTATTVLLDE	240
AEC15166.1	VMNTEHKAYLDKNKAYPVECWVPDPTRNENTRYFGTLTGGENVPPVLHITNTATTVLLDE	240
AEC15266.1	VMNTEHKAYLDKNKAYPVECWVPDPTRNENTRYFGTLTGGENVPPVLHITNTATTVLLDE	240
AEC15267.1	VMNTEHKAYLDKNKAYPVECWVPDPTRNENTRYFGTLTGGENVPPVLHITNTATTVLLDE	240
AEC15268.1	VMNTEHKAYLDKNKAYPVECWVPDPTRNENTRYFGTLTGGENVPPVLHITNTATTVLLDE	240
AEC15269.1	VMNTEHKAYLDKNKAYPVECWVPDPTRNENTRYFGTLTGGENVPPVLHITNTATTVLLDE	240
AEC15270.1	VMNTEHKAYLDKNKAYPVECWVPDPTRNENTRYFGTLTGGENVPPVLHITNTATTVLLDE	240
AEC15271.1	VMNTEHKAYLDKNKAYPVECWVPDPTRNENTRYFGTLTGGENVPPVLHITNTATTVLLDE	240
AEC15272.1	VMNTEHKAYLDKNKAYPVECWVPDPTRNENTRYFGTLTGGENVPPVLHITNTATTVLLDE	240
AEC15273.1	VMNTEHKAYLDKNKAYPVECWVPDPTRNENTRYFGTLTGGENVPPVLHITNTATTVLLDE	240
AEC15274.1	VMNTEHKAYLDKNKAYPVECWVPDPTRNENTRYFGTLTGGENVPPVLHITNTATTVLLDE	240
AEC15275.1	VMNTEHKAYLDKNKAYPVECWVPDPTRNENTRYFGTLTGGENVPPVLHITNTATTVLLDE	240

AEC15276.1	VMNTEHKAYLDKNKAYPVECWVPDPTRNENTRYFGTLTGGENVPPVLHITNTATTVLLDE	240
AEC15277.1	VMNTEHKAYLDKNKAYPVECWVPDPTRNENTRYFGTLTGGENVPPVLHITNTATTVLLDE	240
AEC15278.1	VMNTEHKAYLDKNKAYPVECWVPDPTRNENTRYFGTLTGGENVPPVLHITNTATTVLLDE	240
AEC15279.1	VMNTEHKAYLDKNKAYPVECWVPDPTRNENTRYFGTLTGGENVPPVLHITNTATTVLLDE	240
AEC15280.1	VMNTEHKAYLDKNKAYPVECWVPDPTRNENTRYFGTLTGGENVPPVLHITNTATTVLLDE	240
AEC15281.1	VMNTEHKAYLDKNKAYPVECWVPDPTRNENTRYFGTLTGGENVPPVLHITNTATTVLLDE	240
AEC15282.1	VMNTEHKAYLDKNKAYPVECWVPDPTRNENTRYFGTLTGGENVPPVLHITNTATTVLLDE	240
AEC15283.1	VMNTEHKAYLDKNKAYPVECWVPDPTRNENTRYFGTLTGGENVPPVLHITNTATTVLLDE	240
AEC15284.1	VMNTEHKAYLDKNKAYPVECWVPDPTRNENTRYFGTLTGGENVPPVLHITNTATTVLLDE	240
AEC15285.1	VMNTEHKAYLDKNKAYPVECWVPDPTRNENTRYFGTLTGGENVPPVLHITNTATTVLLDE	240
AEC15286.1	VMNTEHKAYLDKNKAYPVECWVPDPTRNENTRYFGTLTGGENVPPVLHITNTATTVLLDE	240
AEC15287.1	VMNTEHKAYLDKNKAYPVECWVPDPTRNENTRYFGTLTGGENVPPVLHITNTATTVLLDE	240
AEC15288.1	VMNTEHKAYLDKNKAYPVECWVPDPTRNENTRYFGTLTGGENVPPVLHITNTATTVLLDE	240
AEC15289.1	VMNTEHKAYLDKNKAYPVECWVPDPTRNENTRYFGTLTGGENVPPVLHITNTATTVLLDE	240
AEC15290.1	VMNTEHKAYLDKNKAYPVECWVPDPTRNENTRYFGTLTGGENVPPVLHITNTATTVLLDE	240
AEC15291.1	VMNTEHKAYLDKNKAYPVECWVPDPTRNENTRYFGTLTGGENVPPVLHITNTATTVLLDE	240
AEC15292.1	VMNTEHKAYLDKNKAYPVECWVPDPTRNENTRYFGTLTGGENVPPVLHITNTATTVLLDE	240
AEC15293.1	VMNTEHKAYLDKNKAYPVECWVPDPTRNENTRYFGTLTGGENVPPVLHITNTATTVLLDE	240
AEC15294.1	VMNTEHKAYLDKNKAYPVECWVPDPTRNENTRYFGTLTGGENVPPVLHITNTATTVLLDE	240
AEC15295.1	VMNTEHKAYLDKNKAYPVECWVPDPTRNENTRYFGTLTGGENVPPVLHITNTATTVLLDE	240
AEC15296.1	VMNTEHKAYLDKNKAYPVECWVPDPTRNENTRYFGTLTGGENVPPVLHITNTATTVLLDE	240
AEC15297.1	VMNTEHKAYLDKNKAYPVECWVPDPTRNENTRYFGTLTGGENVPPVLHITNTATTVLLDE	240
AEC15298.1	VMNTEHKAYLDKNKAYPVECWVPDPTRNENTRYFGTLTGGENVPPVLHITNTATTVLLDE	240
AEC15300.1	VMNTEHKAYLDKNKAYPVECWVPDPTRNENTRYFGTLTGGENVPPVLHITNTATTVLLDE	240
AEC15301.1	VMNTEHKAYLDKNKAYPVECWVPDPTRNENTRYFGTLTGGENVPPVLHITNTATTVLLDE	240
AEC15302.1	VMNTEHKAYLDKNKAYPVECWVPDPTRNENTRYFGTLTGGENVPPVLHITNTATTVLLDE	240
AEC15303.1	VMNTEHKAYLDKNKAYPVECWVPDPTRNENTRYFGTLTGGENVPPVLHITNTATTVLLDE	240
AEC15304.1	VMNTEHKAYLDKNKAYPVECWVPDPTRNENTRYFGTLTGGENVPPVLHITNTATTVLLDE	240
AEC15305.1	VMNTEHKAYLDKNKAYPVECWVPDPTRNENTRYFGTLTGGENVPPVLHITNTATTVLLDE	240
AEC15306.1	VMNTEHKAYLDKNKAYPVECWVPDPTRNENTRYFGTLTGGENVPPVLHITNTATTVLLDE	240
AEC15307.1	VMNTEHKAYLDKNKAYPVECWVPDPTRNENTRYFGTLTGGENVPPVLHITNTATTVLLDE	240
AEC15308.1	VMNTEHKAYLDKNKAYPVECWVPDPTRNENTRYFGTLTGGENVPPVLHITNTATTVLLDE	240
AEC15309.1	VMNTEHKAYLDKNKAYPVECWVPDPTRNENTRYFGTLTGGENVPPVLHITNTATTVLLDE	240
AEC15310.1	VMNTEHKAYLDKNKAYPVECWVPDPTRNENTRYFGTLTGGENVPPVLHITNTATTVLLDE	240
AEC15311.1	VMNTEHKAYLDKNKAYPVECWVPDPTRNENTRYFGTLTGGENVPPVLHITNTATTVLLDE	240
AEC15861.1	VMNTEHKAYLDKNKAYPVECWVPDPTRNENTRYFGTLTGGENVPPVLHITNTATTVLLDE	240
AEC15867.1	VMNTEHKAYLDKNKAYPVECWVPDPTRNENTRYFGTLTGGENVPPVLHITNTATTVLLDE	240
AEC15873.1	VMNTEHKAYLDKNKAYPVECWVPDPTRNENTRYFGTLTGGENVPPVLHITNTATTVLLDE	240
AEC15879.1	VMNTEHKAYLDKNKAYPVECWVPDPTRNENTRYFGTLTGGENVPPVLHITNTATTVLLDE	240
AEC15885.1	VMNTEHKAYLDKNKAYPVECWVPDPTRNENTRYFGTLTGGENVPPVLHITNTATTVLLDE	240
AEC15891.1	VMNTEHKAYLDKNKAYPVECWVPDPTRNENTRYFGTLTGGENVPPVLHITNTATTVLLDE	240
AEC15897.1	VMNTEHKAYLDKNKAYPVECWVPDPTRNENTRYFGTLTGGENVPPVLHITNTATTVLLDE	240
AEC15903.1	VMNTEHKAYLDKNKAYPVECWVPDPTRNENTRYFGTLTGGENVPPVLHITNTATTVLLDE	240
AEC15909.1	VMNTEHKAYLDKNKAYPVECWVPDPTRNENTRYFGTLTGGENVPPVLHITNTATTVLLDE	240
AEC15915.1	VMNTEHKAYLDKNKAYPVECWVPDPTRNENTRYFGTLTGGENVPPVLHITNTATTVLLDE	240
AEC15921.1	VMNTEHKAYLDKNKAYPVECWVPDPTRNENTRYFGTLTGGENVPPVLHITNTATTVLLDE	240
AEC15927.1	VMNTEHKAYLDKNKAYPVECWVPDPTRNENTRYFGTLTGGENVPPVLHITNTATTVLLDE	240
AEC15933.1	VMNTEHKAYLDKNKAYPVECWVPDPTRNENTRYFGTLTGGENVPPVLHITNTATTVLLDE	240
AEC15939.1	VMNTEHKAYLDKNKAYPVECWVPDPTRNENTRYFGTLTGGENVPPVLHITNTATTVLLDE	240
AEC15945.1	VMNTEHKAYLDKNKAYPVECWVPDPTRNENTRYFGTLTGGENVPPVLHITNTATTVLLDE	240
AEC15951.1	VMNTEHKAYLDKNKAYPVECWVPDPTRNENTRYFGTLTGGENVPPVLHITNTATTVLLDE	240
AEC15957.1	VMNTEHKAYLDKNKAYPVECWVPDPTRNENTRYFGTLTGGENVPPVLHITNTATTVLLDE	240
BAD21259.1	VMNTEHKAYLDKNKAYPVECWVPDPTRNENTRYFGTLTGGENVPPVLHITNTATTVLLDE	240
BAD21265.1	VMNTEHKAYLDKNKAYPVECWVPDPTRNENTRYFGTLTGGENVPPVLHITNTATTVLLDE	240
AAC59325.1	VMNTEHKAYLDKNKAYPVECWVPDPTRNENTRYFGTLTGGENVPPVLHITNTATTVLLDE	240
AEC14524.1	VMNTEHKAYLDKNKAYPVECWVPDPTRNENTRYFGTLTGGENVPPVLHITNTATTVLLDE	240
AEC14541.1	VMNTEHKAYLDKNKAYPVECWVPDPTRNENTRYFGTLTGGENVPPVLHITNTATTVLLDE	240
AEC14565.1	VMNTEHKAYLDKNKAYPVECWVPDPTRNENTRYFGTLTGGENVPPVLHITNTATTVLLDE	240
AEC14571.1	VMNTEHKAYLDKNKAYPVECWVPDPTRNENTRYFGTLTGGENVPPVLHITNTATTVLLDE	240
AEC14583.1	VMNTEHKAYLDKNKAYPVECWVPDPTRNENTRYFGTLTGGENVPPVLHITNTATTVLLDE	240
AEC14601.1	VMNTEHKAYLDKNKAYPVECWVPDPTRNENTRYFGTLTGGENVPPVLHITNTATTVLLDE	240
AEC14607.1	VMNTEHKAYLDKNKAYPVECWVPDPTRNENTRYFGTLTGGENVPPVLHITNTATTVLLDE	240
AEC14637.1	VMNTEHKAYLDKNKAYPVECWVPDPTRNENTRYFGTLTGGENVPPVLHITNTATTVLLDE	240
AEC14649.1	VMNTEHKAYLDKNKAYPVECWVPDPTRNENTRYFGTLTGGENVPPVLHITNTATTVLLDE	240

AEC14655.1	VMNTEHKAYLDKNKAYPVECWVPDPTRNENTRYFGTLTGGENVPPVLHITNTATTVLLDE	240
AEC14661.1	VMNTEHKAYLDKNKAYPVECWVPDPTRNENTRYFGTLTGGENVPPVLHITNTATTVLLDE	240
AEC14685.1	VMNTEHKAYLDKNKAYPVECWVPDPTRNENTRYFGTLTGGENVPPVLHITNTATTVLLDE	240
AEC14697.1	VMNTEHKAYLDKNKAYPVECWVPDPTRNENTRYFGTLTGGENVPPVLHITNTATTVLLDE	240
AEC14709.1	VMNTEHKAYLDKNKAYPVECWVPDPTRNENTRYFGTLTGGENVPPVLHITNTATTVLLDE	240
AEC14715.1	VMNTEHKAYLDKNKAYPVECWVPDPTRNENTRYFGTLTGGENVPPVLHITNTATTVLLDE	240
AEC14963.1	VMNTEHKAYLDKNKAYPVECWVPDPTRNENTRYFGTLTGGENVPPVLHITNTATTVLLDE	240
AEC14968.1	VMNTEHKAYLDKNKAYPVECWVPDPTRNENTRYFGTLTGGENVPPVLHITNTATTVLLDE	240
AEC14971.1	VMNTEHKAYLDKNKAYPVECWVPDPTRNENTRYFGTLTGGENVPPVLHITNTATTVLLDE	240
AEC14972.1	VMNTEHKAYLDKNKAYPVECWVPDPTRNENTRYFGTLTGGENVPPVLHITNTATTVLLDE	240
AEC14975.1	VMNTEHKAYLDKNKAYPVECWVPDPTRNENTRYFGTLTGGENVPPVLHITNTATTVLLDE	240
AEC14976.1	VMNTEHKAYLDKNKAYPVECWVPDPTRNENTRYFGTLTGGENVPPVLHITNTATTVLLDE	240
AEC14977.1	VMNTEHKAYLDKNKAYPVECWVPDPTRNENTRYFGTLTGGENVPPVLHITNTATTVLLDE	240
AEC14978.1	VMNTEHKAYLDKNKAYPVECWVPDPTRNENTRYFGTLTGGENVPPVLHITNTATTVLLDE	240
AEC14980.1	VMNTEHKAYLDKNKAYPVECWVPDPTRNENTRYFGTLTGGENVPPVLHITNTATTVLLDE	240
AEC14981.1	VMNTEHKAYLDKNKAYPVECWVPDPTRNENTRYFGTLTGGENVPPVLHITNTATTVLLDE	240
AEC14982.1	VMNTEHKAYLDKNKAYPVECWVPDPTRNENTRYFGTLTGGENVPPVLHITNTATTVLLDE	240
AEC14983.1	VMNTEHKAYLDKNKAYPVECWVPDPTRNENTRYFGTLTGGENVPPVLHITNTATTVLLDE	240
AEC14985.1	VMNTEHKAYLDKNKAYPVECWVPDPTRNENTRYFGTLTGGENVPPVLHITNTATTVLLDE	240
AEC14986.1	VMNTEHKAYLDKNKAYPVECWVPDPTRNENTRYFGTLTGGENVPPVLHITNTATTVLLDE	240
AEC14988.1	VMNTEHKAYLDKNKAYPVECWVPDPTRNENTRYFGTLTGGENVPPVLHITNTATTVLLDE	240
AEC14989.1	VMNTEHKAYLDKNKAYPVECWVPDPTRNENTRYFGTLTGGENVPPVLHITNTATTVLLDE	240
AEC14991.1	VMNTEHKAYLDKNKAYPVECWVPDPTRNENTRYFGTLTGGENVPPVLHITNTATTVLLDE	240
AEC14992.1	VMNTEHKAYLDKNKAYPVECWVPDPTRNENTRYFGTLTGGENVPPVLHITNTATTVLLDE	240
AEC14996.1	VMNTEHKAYLDKNKAYPVECWVPDPTRNENTRYFGTLTGGENVPPVLHITNTATTVLLDE	240
AEC14997.1	VMNTEHKAYLDKNKAYPVECWVPDPTRNENTRYFGTLTGGENVPPVLHITNTATTVLLDE	240
AEC14998.1	VMNTEHKAYLDKNKAYPVECWVPDPTRNENTRYFGTLTGGENVPPVLHITNTATTVLLDE	240
AEC15002.1	VMNTEHKAYLDKNKAYPVECWVPDPTRNENTRYFGTLTGGENVPPVLHITNTATTVLLDE	240
AEC15003.1	VMNTEHKAYLDKNKAYPVECWVPDPTRNENTRYFGTLTGGENVPPVLHITNTATTVLLDE	240
AEC15004.1	VMNTEHKAYLDKNKAYPVECWVPDPTRNENTRYFGTLTGGENVPPVLHITNTATTVLLDE	240
AEC15005.1	VMNTEHKAYLDKNKAYPVECWVPDPTRNENTRYFGTLTGGENVPPVLHITNTATTVLLDE	240
AEC15006.1	VMNTEHKAYLDKNKAYPVECWVPDPTRNENTRYFGTLTGGENVPPVLHITNTATTVLLDE	240
AEC15009.1	VMNTEHKAYLDKNKAYPVECWVPDPTRNENTRYFGTLTGGENVPPVLHITNTATTVLLDE	240
AEC15012.1	VMNTEHKAYLDKNKAYPVECWVPDPTRNENTRYFGTLTGGENVPPVLHITNTATTVLLDE	240
AEC15013.1	VMNTEHKAYLDKNKAYPVECWVPDPTRNENTRYFGTLTGGENVPPVLHITNTATTVLLDE	240
AEC15015.1	VMNTEHKAYLDKNKAYPVECWVPDPTRNENTRYFGTLTGGENVPPVLHITNTATTVLLDE	240
AEC15017.1	VMNTEHKAYLDKNKAYPVECWVPDPTRNENTRYFGTLTGGENVPPVLHITNTATTVLLDE	240
AEC15018.1	VMNTEHKAYLDKNKAYPVECWVPDPTRNENTRYFGTLTGGENVPPVLHITNTATTVLLDE	240
AEC15021.1	VMNTEHKAYLDKNKAYPVECWVPDPTRNENTRYFGTLTGGENVPPVLHITNTATTVLLDE	240
AEC15022.1	VMNTEHKAYLDKNKAYPVECWVPDPTRNENTRYFGTLTGGENVPPVLHITNTATTVLLDE	240
AEC14962.1	VMNTEHKAYLDKNKAYPVECWVPDPTRNENTRYFGTLTGGENVPPVLHITNTATTVLLDE	240
AEC14993.1	VMNTEHKAYLDKNKAYPVECWVPDPTRNENTRYFGTLTGGENVPPVLHITNTATTVLLDE	240
AEC15007.1	VMNTEHKAYLDKNKAYPVECWVPDPTRNENTRYFGTLTGGENVPPVLHITNTATTVLLDE	240
CCW72517.1	VMNTEHKAYLDKNKAYPVECWVPDPTRNENTRYFGTLTGGENVPPVLHITNTATTVLLDE	240
BAD21247.2	VMNTEHKAYLDKNKAYPVECWVPDPTRNENTRYFGTLTGGENVPPVLHITNTATTVLLDE	240
AAL37695.1	VMNTEHKAYLDKNKAYPVECWVPDPTRNENTRYFGTLTGGENVPPVLHITNTATTVLLDE	240
AEC15242.1	VMNTEHKAYLDKNKAYPVECWVPDPTRNENTRYFGTLTGGENVPPVLHITNTATTVLLDE	240
AEC15243.1	VMNTEHKAYLDKNKAYPVECWVPDPTRNENTRYFGTLTGGENVPPVLHITNTATTVLLDE	240
AEC15244.1	VMNTEHKAYLDKNKAYPVECWVPDPTRNENTRYFGTLTGGENVPPVLHITNTATTVLLDE	240
AEC15245.1	VMNTEHKAYLDKNKAYPVECWVPDPTRNENTRYFGTLTGGENVPPVLHITNTATTVLLDE	240
AEC15246.1	VMNTEHKAYLDKNKAYPVECWVPDPTRNENTRYFGTLTGGENVPPVLHITNTATTVLLDE	240
AEC15247.1	VMNTEHKAYLDKNKAYPVECWVPDPTRNENTRYFGTLTGGENVPPVLHITNTATTVLLDE	240
AEC15248.1	VMNTEHKAYLDKNKAYPVECWVPDPTRNENTRYFGTLTGGENVPPVLHITNTATTVLLDE	240
AEC15249.1	VMNTEHKAYLDKNKAYPVECWVPDPTRNENTRYFGTLTGGENVPPVLHITNTATTVLLDE	240
AEC15250.1	VMNTEHKAYLDKNKAYPVECWVPDPTRNENTRYFGTLTGGENVPPVLHITNTATTVLLDE	240
AEC15251.1	VMNTEHKAYLDKNKAYPVECWVPDPTRNENTRYFGTLTGGENVPPVLHITNTATTVLLDE	240
AEC15252.1	VMNTEHKAYLDKNKAYPVECWVPDPTRNENTRYFGTLTGGENVPPVLHITNTATTVLLDE	240
AEC15253.1	VMNTEHKAYLDKNKAYPVECWVPDPTRNENTRYFGTLTGGENVPPVLHITNTATTVLLDE	240
AEC15254.1	VMNTEHKAYLDKNKAYPVECWVPDPTRNENTRYFGTLTGGENVPPVLHITNTATTVLLDE	240
AEC15255.1	VMNTEHKAYLDKNKAYPVECWVPDPTRNENTRYFGTLTGGENVPPVLHITNTATTVLLDE	240
AEC15256.1	VMNTEHKAYLDKNKAYPVECWVPDPTRNENTRYFGTLTGGENVPPVLHITNTATTVLLDE	240
AEC15257.1	VMNTEHKAYLDKNKAYPVECWVPDPTRNENTRYFGTLTGGENVPPVLHITNTATTVLLDE	240
AEC15258.1	VMNTEHKAYLDKNKAYPVECWVPDPTRNENTRYFGTLTGGENVPPVLHITNTATTVLLDE	240
AEC15259.1	VMNTEHKAYLDKNKAYPVECWVPDPTRNENTRYFGTLTGGENVPPVLHITNTATTVLLDE	240

AEC15260.1	VMNTEHKAYLDKNKAYPVECWVPDPTRNENTRYFGTLTGGENVPPVLHITNTATTVLLDE	240
AEC15261.1	VMNTEHKAYLDKNKAYPVECWVPDPTRNENTRYFGTLTGGENVPPVLHITNTATTVLLDE	240
AEC15262.1	VMNTEHKAYLDKNKAYPVECWVPDPTRNENTRYFGTLTGGENVPPVLHITNTATTVLLDE	240
AEC15263.1	VMNTEHKAYLDKNKAYPVECWVPDPTRNENTRYFGTLTGGENVPPVLHITNTATTVLLDE	240
AEC15264.1	VMNTEHKAYLDKNKAYPVECWVPDPTRNENTRYFGTLTGGENVPPVLHITNTATTVLLDE	240
AEC15265.1	VMNTEHKAYLDKNKAYPVECWVPDPTRNENTRYFGTLTGGENVPPVLHITNTATTVLLDE	240
AEC15000.1	VMNTEHKAYLDKNKAYPVECWVPDPTRNENTRYFGTLTGGENVPPVLHITNTATTVLLDE	240
AEC14721.1	VMNTEHKAYLDKNKAYPVECWVPDPTRNENTRYFGTLTGGENVPPVLHITNTATTVLLDE	240
AEC14965.1	VMNTEHKAYLDKNKAYPVECWVPDPTRNENTRYFGTLTGGENVPPVLHITNTATTVLLDE	240
AEC14967.1	VMNTEHKAYLDKNKAYPVECWVPDPTRNENTRYFGTLTGGENVPPVLHITNTATTVLLDE	240
AEC14969.1	VMNTEHKAYLDKNKAYPVECWVPDPTRNENTRYFGTLTGGENVPPVLHITNTATTVLLDE	240
AEC14973.1	VMNTEHKAYLDKNKAYPVECWVPDPTRNENTRYFGTLTGGENVPPVLHITNTATTVLLDE	240
AEC14974.1	VMNTEHKAYLDKNKAYPVECWVPDPTRNENTRYFGTLTGGENVPPVLHITNTATTVLLDE	240
AEC14994.1	VMNTEHKAYLDKNKAYPVECWVPDPTRNENTRYFGTLTGGENVPPVLHITNTATTVLLDE	240
AEC15001.1	VMNTEHKAYLDKNKAYPVECWVPDPTRNENTRYFGTLTGGENVPPVLHITNTATTVLLDE	240
AEC15024.1	VMNTEHKAYLDKNKAYPVECWVPDPTRNENTRYFGTLTGGENVPPVLHITNTATTVLLDE	240
AEC14667.1	VMNTEHKAYLDKNKAYPVECWVPDPTRNENTRYFGTLTGGENVPPVLHITNTATTVLLDE	240
UPN63091.1	VMNTEHKAYLDKNKAYPVECWVPDPTRNENTRYFGTLTGGENVPPVLHITNTATTVLLDE	240
AEC14535.1	VMNTEHKAYLDKNKAYPVECWVPDPTRNENTRYFGTLTGGENVPPVLHITNTATTVLLDE	240
AEC14547.1	VMNTEHKAYLDKNKAYPVECWVPDPTRNENTRYFGTLTGGENVPPVLHITNTATTVLLDE	240
AEC14553.1	VMNTEHKAYLDKNKAYPVECWVPDPTRNENTRYFGTLTGGENVPPVLHITNTATTVLLDE	240
AEC14577.1	VMNTEHKAYLDKNKAYPVECWVPDPTRNENTRYFGTLTGGENVPPVLHITNTATTVLLDE	240
AEC14613.1	VMNTEHKAYLDKNKAYPVECWVPDPTRNENTRYFGTLTGGENVPPVLHITNTATTVLLDE	240
AEC14673.1	VMNTEHKAYLDKNKAYPVECWVPDPTRNENTRYFGTLTGGENVPPVLHITNTATTVLLDE	240
AEC14679.1	VMNTEHKAYLDKNKAYPVECWVPDPTRNENTRYFGTLTGGENVPPVLHITNTATTVLLDE	240
AEC14727.1	VMNTEHKAYLDKNKAYPVECWVPDPTRNENTRYFGTLTGGENVPPVLHITNTATTVLLDE	240
AEC14733.1	VMNTEHKAYLDKNKAYPVECWVPDPTRNENTRYFGTLTGGENVPPVLHITNTATTVLLDE	240
AEC14964.1	VMNTEHKAYLDKNKAYPVECWVPDPTRNENTRYFGTLTGGENVPPVLHITNTATTVLLDE	240
AEC14995.1	VMNTEHKAYLDKNKAYPVECWVPDPTRNENTRYFGTLTGGENVPPVLHITNTATTVLLDE	240
AEC15008.1	VMNTEHKAYLDKNKAYPVECWVPDPTRNENTRYFGTLTGGENVPPVLHITNTATTVLLDE	240
AEC14970.1	VMNTEHKAYLDKNKAYPVECWVPDPTRNENTRYFGTLTGGENVPPVLHITNTATTVLLDE	240
QTJ15162.1	VMNTEHKAYLDKNKAYPVECWVPDPTRNENTRYFGTLTGGENVPPVLHITNTATTVLLDE	240
UYL83831.1	VMNTEHKAYLDKNKAYPVECWVPDPTRNENTRYFGTLTGGENVPPVLHITNTATTVLLDE	237
ALF06183.1	VMNTEHKAYLDKNKAYPVECWVPDPTRNENTRYFGTLTGGENVPPVLHITNTATTVLLDE	240
QCH41111.1	VMNTEHKAYLDKNKAYPVECWVPDPTRNENTRYFGTLTGGENVPPVLHITNTATTVLLDE	240
QCH41112.1	VMNTEHKAYLDKNKAYPVECWVPDPTRNENTRYFGTLTGGENVPPVLHITNTATTVLLDE	240
AEC14749.1	VMNTEHKAYLDKNKAYPVECWVPDPTRNENTRYFGTLTGGENVPPVLHITNTATTVLLDE	240
ALF06202.1	VMNTEHKAYLDKNKAYPVECWVPDPTRNENTRYFGTLTGGGNVPPVLHITNTAATVLLDE	240
ALF06186.1	VMNTEHKAYLDKNKAYPVECWVPDPTGNENTRYFGTLTGGENVPPVLHITNTATTVLLDE	240
ALF06208.1	VMNTEHKAYLDKNKAYPVECWVPDPTRNENTRYFGTLTGGENVPPVLHITNTATTVLLDE	240
ALF06197.1	VMNTEHKAYLDKNKAYPVECWVPDPTRNENTRYFGTLTGGENAPPVLHITNTATTVLLDE	240
AEC15037.1	VMNTEHKAYLDKNKAYPVECWVPNPTRNENTRYFGTLTGGENVPPVLHITNTATTVLLDE	240
UPN63109.1	VMNTEHKAYLDKNKAYPVECWVPDPTRNENTRYFGTLTGGENVPPVLHITNTATTVLLDE	240
UPN63107.1	VMNTEHKAYLDKNKAYPVECWVPDPTRNENTRYFGTLTGGENVPPVLHITNTATTVLLDE	240
UPN63108.1	VMNTEHKAYLDKNKAYPVECWVPDPTRNENTRYFGTLTGGENVPPVLHITNTATTVLLDE	240
QBQ04202.1	VMNTEHKAYLDKNKAYPVECWVPDPTRNENTRYFGTLTGGENVPPVLHITNTATTVLLDE	240
QBM11923.1	VMNTEHKAYLDKNKAYPVECWVPDPTRNENTRYFGTLTGGENVPPVLHITNTATTVLLDE	240
AAK70306.1	VMNTEHKAYLDKNKAYPVECWVPDPTRNENTRYFGTLTGGENVPPVLHITNTATTVLLDE	240
AAC59337.1	VMNTEHKAYLDKNKVYPVECWVPDPTRNENTRYFGTLTGGENVPPVLHITNTATTVLLDE	240
AAL37677.1	VMNPEPKGYLDKNKAYPVECWVPDPTRNENTRYFGTLTGGENVPPVLHITNTATTVLLDE	240
AAK70276.1	VMTTEHKGSLDKTKAYPVECWVPDPTRNENTRYFGTLTGGENVPPVLHITNTATTVLLDE	240
BAC22792.1	VMNTEHKAYLDKNKAYPVECWVPDPTRNENTRYFGTLTGGENVPPVLHITNTATTVLLDE	240
BAC22798.1	VMNTEHKAYLDKNKAYPVECWVPDPTRNENTRYFGTLTGGENVPPVLHITNTATTVLLDE	240
BAC22804.1	VMNTEHKAYLDKNKAYPVECWVPDPTRNENTRYFGTLTGGENVPPVLHITNTATTVLLDE	240
BAC22810.1	VMNTEHKAYLDKNKAYPVECWVPDPTRNENTRYFGTLTGGENVPPVLHITNTATTVLLDE	240
BAC22858.1	VMNTEHKAYLDKNKAYPVECWVPDPTRNENTRYFGTLTGGENVPPVLHITNTATTVLLDE	240
BAC15701.1	VMNTEHKAYLDKNKAYPVECWVPDPTRNENTRYFGTLTGGENVPPVLHITNTATTVLLDE	240
BAB68863.1	VMNTEHKAYLDKNKAYPVECWVPDPTRNENTRYFGTLTGGENVPPVLHITNTATTVLLDE	240
BAC66424.1	VMNTEHKAYLDKNKAYPVECWVPDPTRNENTRYFGTLTGGENVPPVLHITNTATTVLLDE	240
BAD21235.2	VMNTDHKAYLDKNKAYPVECWVPDPTRNENTRYFGTLTGGENVPPVLHITNTATTVLLDE	240
QTJ15210.1	VMNTEHKAYLDKNKAYPVECWVPDPTRNENTRYFGTLTGGENVPPVLHITNTATTVLLDE	240
ASV51748.1	VMNTEHKAYLDKNKAYPVECWVPDPTRNENTRYFGTLTGGENVPPVLHITNTATTVLLDE	240
ASV51754.1	VMNTEHKAYLDKNKAYPVECWVPDPTRNENTRYFGTLTGGENVPPVLHITNTATTVLLDE	240
ASV51778.1	VMNTEHKAYLDKNKAYPVECWVPDPTRNENTRYFGTLTGGENVPPVLHITNTATTVLLDE	240

ASV51814.1	VMNTEHKAYLDKNKAYPVECWVPDPTRNENTRYFGTLTGGENVPPVLHITNTATTVLLDE	240
CCW72488.1	VMNTEHKAYLDKNKAYPVECWVPDPTRNENTRYFGTLTGGENVPPVLHITNTATTVLLDE	240
AEC15219.1	VMNTEHKAYLDKNKAYPVECWVPDPTRNENTRYFGTLTGGENVPPVLHITNTATTVLLDE	240
BAD21241.1	VMNTEHKAYLDKNKAYPVECWVPDPTRNENTRYFGTLTGGENVPPVLHITNTATTVLLDE	240
BAD21253.1	VMNTEHKAYLDKNKAYPVECWVPDPTRNENTRYFGTLTGGENVPPVLHITNTATTVLLDE	240
BAB93086.1	VMNTEHKAYLDKNKAYPVECWVPDPTRNENTRYFGTLTGGENVPPVLHITNTATTVLLDE	240
BAB68953.1	VMNTEHKAYLDKNKAYPVECWVPDPTRNENTRYFGTLTGGENVPPVLHITNTATTVLLDE	240
BAD98966.1	VMNTEHKAYLDKNKAYPVECWVPDPTRNENTRYFGTLTGGENVPPVLHITNTATTVLLDE	240
BAD98972.1	VMNTEHKAYLDKNKAYPVECWVPDPTRNENTRYFGTLTGGENVPPVLHITNTATTVLLDE	240
BAD91887.1	VMNTEHKAYLDKNKAYPVECWVPDPTRNENTRYFGTLTGGENVPPVLHITNTATTVLLDE	240
BAD27118.1	VMNTEHKAYLDKNKAYPVECWVPDPTRNENTRYFGTLTGGENVPPVLHITNTATTVLLDE	240
BAD06227.1	VMNTEHKAYLDKNKAYPVECWVPDPTRNENTRYFGTLTGGENVPPVLHITNTATTVLLDE	240
BAC66412.1	VMNTEHKAYLDKNKAYPVECWVPDPTRNENTRYFGTLTGGENVPPVLHITNTATTVLLDE	240
BAC66430.1	VMNTEHKAYLDKNKAYPVECWVPDPTRNENTRYFGTLTGGENVPPVLHITNTATTVLLDE	240
BAC22816.1	VMNTEHKAYLDKNKAYPVECWVPDPTRNENTRYFGTLTGGENVPPVLHITNTATTVLLDE	240
BAB93026.1	VMNTEHKAYLDKNKAYPVECWVPDPTRNENTRYFGTLTGGENVPPVLHITNTATTVLLDE	240
BAB93032.1	VMNTEHKAYLDKNKAYPVECWVPDPTRNENTRYFGTLTGGENVPPVLHITNTATTVLLDE	240
BAB93074.1	VMNTEHKAYLDKNKAYPVECWVPDPTRNENTRYFGTLTGGENVPPVLHITNTATTVLLDE	240
BAB93080.1	VMNTEHKAYLDKNKAYPVECWVPDPTRNENTRYFGTLTGGENVPPVLHITNTATTVLLDE	240
BAB68917.1	VMNTEHKAYLDKNKAYPVECWVPDPTRNENTRYFGTLTGGENVPPVLHITNTATTVLLDE	240
BAB68923.1	VMNTEHKAYLDKNKAYPVECWVPDPTRNENTRYFGTLTGGENVPPVLHITNTATTVLLDE	240
BAB68929.1	VMNTEHKAYLDKNKAYPVECWVPDPTRNENTRYFGTLTGGENVPPVLHITNTATTVLLDE	240
BAB68935.1	VMNTEHKAYLDKNKAYPVECWVPDPTRNENTRYFGTLTGGENVPPVLHITNTATTVLLDE	240
BAB68941.1	VMNTEHKAYLDKNKAYPVECWVPDPTRNENTRYFGTLTGGENVPPVLHITNTATTVLLDE	240
ALF06214.1	VMNTEHKAYLDKNKAYPVECWVPDPTRNENTRYFGTLTGGENVPPVLHITNTATTVLLDE	240
AAL37707.1	VMNTEHKAYLDKNKAYPVECWVPDPTRNENTRYFGTLTGGENVPPVLHITNTATTVLLDE	240
BBG22688.1	VMNTEHKAYLDKNKAYPVECWVPDPTRNENTRYFGTLTGGENVPPVLHITNTATTVLLDE	240
BAB11734.1	VMNTEHKAYLDKNKAYPVECWVPDPTRNENTRYFGTLTGGENVPPVLHITNTATTVLLDE	240
BAA05638.1	VMNTEHKAYLDKNKAYPVECWVPDPTRNENTRYFGTLTGGENVPPVLHITNTATTVLLDE	240
BAV69361.1	VMNTEHKAYLDKNKAYPVECWVPDPTRNENTRYFGTLTGGENVPPVLHITNTATTVLLDE	240
BAV69349.1	VMNTEHKAYLDKNKAYPVECWVPDPTRNENTRYFGTLTGGENVPPVLHITNTATTVLLDE	240
BAV69367.1	VMNTEHKAYLDKNKAYPVECWVPDPTRNENTRYFGTLTGGENVPPVLHITNTATTVLLDE	240
BAE00135.1	VMNTEHKAYLDKNKAYPVECWVPDPTRNENTRYFGTLTGGENVPPVLHITNTATTVLLDE	240
BAE00147.1	VMNTEHKAYLDKNKAYPVECWVPDPTRNENTRYFGTLTGGENVPPVLHITNTATTVLLDE	240
BAD11896.1	VMNTEHKAYLDKNKAYPVECWVPDPTRNENTRYFGTLTGGENVPPVLHITNTATTVLLDE	240
BAD06126.1	VMNTEHKAYLDKNKAYPVECWVPDPTRNENTRYFGTLTGGENVPPVLHITNTATTVLLDE	240
BAC81904.1	VMNTEHKAYLDKNKAYPVECWVPDPTRNENTRYFGTLTGGENVPPVLHITNTATTVLLDE	240
BAC81910.1	VMNTEHKAYLDKNKAYPVECWVPDPTRNENTRYFGTLTGGENVPPVLHITNTATTVLLDE	240
BAC81916.1	VMNTEHKAYLDKNKAYPVECWVPDPTRNENTRYFGTLTGGENVPPVLHITNTATTVLLDE	240
BAC81922.1	VMNTEHKAYLDKNKAYPVECWVPDPTRNENTRYFGTLTGGENVPPVLHITNTATTVLLDE	240
BAC81946.1	VMNTEHKAYLDKNKAYPVECWVPDPTRNENTRYFGTLTGGENVPPVLHITNTATTVLLDE	240
BAC81952.1	VMNTEHKAYLDKNKAYPVECWVPDPTRNENTRYFGTLTGGENVPPVLHITNTATTVLLDE	240
BAC81958.1	VMNTEHKAYLDKNKAYPVECWVPDPTRNENTRYFGTLTGGENVPPVLHITNTATTVLLDE	240
AAM89297.1	VMNTEHKAYLDKNKAYPVECWVPDPTRNENTRYFGTLTGGENVPPVLHITNTATTVLLDE	240
AAM89303.1	VMNTEHKAYLDKNKAYPVECWVPDPTRNENTRYFGTLTGGENVPPVLHITNTATTVLLDE	240
AAM89309.1	VMNTEHKAYLDKNKAYPVECWVPDPTRNENTRYFGTLTGGENVPPVLHITNTATTVLLDE	240
BAC66388.1	VMNTEHKAYLDKNKAYPVECWVPDPTRNENTRYFGTLTGGENVPPVLHITNTATTVLLDE	240
BAC66400.1	VMNTEHKAYLDKNKAYPVECWVPDPTRNENTRYFGTLTGGENVPPVLHITNTATTVLLDE	240
BAC66406.1	VMNTEHKAYLDKNKAYPVECWVPDPTRNENTRYFGTLTGGENVPPVLHITNTATTVLLDE	240
BAC22756.1	VMNTEHKAYLDKNKAYPVECWVPDPTRNENTRYFGTLTGGENVPPVLHITNTATTVLLDE	240
BAC22822.1	VMNTEHKAYLDKNKAYPVECWVPDPTRNENTRYFGTLTGGENVPPVLHITNTATTVLLDE	240
BAC22894.1	VMNTEHKAYLDKNKAYPVECWVPDPTRNENTRYFGTLTGGENVPPVLHITNTATTVLLDE	240
BAC22900.1	VMNTEHKAYLDKNKAYPVECWVPDPTRNENTRYFGTLTGGENVPPVLHITNTATTVLLDE	240
BAC15635.1	VMNTEHKAYLDKNKAYPVECWVPDPTRNENTRYFGTLTGGENVPPVLHITNTATTVLLDE	240
BAC15653.1	VMNTEHKAYLDKNKAYPVECWVPDPTRNENTRYFGTLTGGENVPPVLHITNTATTVLLDE	240
BAC15665.1	VMNTEHKAYLDKNKAYPVECWVPDPTRNENTRYFGTLTGGENVPPVLHITNTATTVLLDE	240
BAC03065.1	VMNTEHKAYLDKNKAYPVECWVPDPTRNENTRYFGTLTGGENVPPVLHITNTATTVLLDE	240
AAL37689.1	VMNTEHKAYLDKNKAYPVECWVPDPTRNENTRYFGTLTGGENVPPVLHITNTATTVLLDE	240
AAL37701.1	VMNTEHKAYLDKNKAYPVECWVPDPTRNENTRYFGTLTGGENVPPVLHITNTATTVLLDE	240
BAB68803.1	VMNTEHKAYLDKNKAYPVECWVPDPTRNENTRYFGTLTGGENVPPVLHITNTATTVLLDE	240
BAB68965.1	VMNTEHKAYLDKNKAYPVECWVPDPTRNENTRYFGTLTGGENVPPVLHITNTATTVLLDE	240
BAB68971.1	VMNTEHKAYLDKNKAYPVECWVPDPTRNENTRYFGTLTGGENVPPVLHITNTATTVLLDE	240
AAK97964.1	VMNTEHKAYLDKNKAYPVECWVPDPTRNENTRYFGTLTGGENVPPVLHITNTATTVLLDE	240
AAK97970.1	VMNTEHKAYLDKNKAYPVECWVPDPTRNENTRYFGTLTGGENVPPVLHITNTATTVLLDE	240

AAC40841.1	VMNTEHKAYLDKNKAYPVECWVPDPTRNENTRYFGTLTGGENVPPVLHITNTATTVLLDE	240
BAA05636.1	VMNTEHKAYLDKNKAYPVECWVPDPTRNENTRYFGTLTGGENVPPVLHITNTATTVLLDE	240
BAA01970.1	VMNTEHKAYLDKNKAYPVECWVPDPTRNENTRYFGTLTGGENVPPVLHITNTATTVLLDE	240
BAD06144.1	VMNTEHKAYLDKNKAYPVECWVPDPTRNENTRYFGTLTGGENVPPVLHITNTATTVLLDE	240
BAC81852.1	VMNTEHKAYLDKNKAYPVECWVPDPTRNENTRYFGTLTGGENVPPVLHITNTATTVLLDE	240
BAC81858.1	VMNTEHKAYLDKNKAYPVECWVPDPTRNENTRYFGTLTGGENVPPVLHITNTATTVLLDE	240
BAC22864.1	VMNTEHKAYLDKNKAYPVECWVPDPTRNENTRYFGTLTGGENVPPVLHITNTATTVLLDE	240
AAM89315.1	VMNTEHKAYLDKNKAYPVECWVPDPTRNENTRYFGTLTGGENVPPVLHITNTATTVLLDE	240
BAC66382.1	VMNTEHKAYLDKNKAYPVECWVPDPTRNENTRYFGTLTGGENVPPVLHITNTATTVLLDE	240
BAC66394.1	VMNTEHKAYLDKNKAYPVECWVPDPTRNENTRYFGTLTGGENVPPVLHITNTATTVLLDE	240
BAC66418.1	VMNTEHKAYLDKNKAYPVECWVPDPTRNENTRYFGTLTGGENVPPVLHITNTATTVLLDE	240
BAC22828.1	VMNTEHKAYLDKNKAYPVECWVPDPTRNENTRYFGTLTGGENVPPVLHITNTATTVLLDE	240
BAC22846.1	VMNTEHKAYLDKNKAYPVECWVPDPTRNENTRYFGTLTGGENVPPVLHITNTATTVLLDE	240
AAM69590.1	VMNTEHKAYLDKNKAYPVECWVPDPTRNENTRYFGTLTGGENVPPVLHITNTATTVLLDE	240
BAB93014.1	VMNTEHKAYLDKNKAYPVECWVPDPTRNENTRYFGTLTGGENVPPVLHITNTATTVLLDE	240
BAB93020.1	VMNTEHKAYLDKNKAYPVECWVPDPTRNENTRYFGTLTGGENVPPVLHITNTATTVLLDE	240
BAB93092.1	VMNTEHKAYLDKNKAYPVECWVPDPTRNENTRYFGTLTGGENVPPVLHITNTATTVLLDE	240
BAB93098.1	VMNTEHKAYLDKNKAYPVECWVPDPTRNENTRYFGTLTGGENVPPVLHITNTATTVLLDE	240
AAL37665.1	VMNTEHKAYLDKNKAYPVECWVPDPTRNENTRYFGTLTGGENVPPVLHITNTATTVLLDE	240
BAB68773.1	VMNTEHKAYLDKNKAYPVECWVPDPTRNENTRYFGTLTGGENVPPVLHITNTATTVLLDE	240
BAB68779.1	VMNTEHKAYLDKNKAYPVECWVPDPTRNENTRYFGTLTGGENVPPVLHITNTATTVLLDE	240
AAK28460.1	VMNTEHKAYLDKNKAYPVECWVPDPTRNENTRYFGTLTGGENVPPVLHITNTATTVLLDE	240
AAK28466.1	VMNTEHKAYLDKNKAYPVECWVPDPTRNENTRYFGTLTGGENVPPVLHITNTATTVLLDE	240
BAB11716.1	VMNTEHKAYLDKNKAYPVECWVPDPTRNENTRYFGTLTGGENVPPVLHITNTATTVLLDE	240
BAB11722.1	VMNTEHKAYLDKNKAYPVECWVPDPTRNENTRYFGTLTGGENVPPVLHITNTATTVLLDE	240
AAK97976.1	VMNTEHKAYLDKNKAYPVECWVPDPTRNENTRYFGTLTGGENVPPVLHITNTATTVLLDE	240
BAC15641.1	VMNTEHKAYLDKNKAYPVECWVPDPTRNENTRYFGTLTGGENVPPVLHITNTATTVLLDE	240
BAC15647.1	VMNTEHKAYLDKNKAYPVECWVPDPTRNENTRYFGTLTGGENVPPVLHITNTATTVLLDE	240
AAL37647.1	VMNTEHKAYLDKNKAYPVECWVPDPTRNENTRYFGTLTGGENVPPVLHITNTATTVLLDE	240
BAB68797.1	VMNTEHKAYLDRNKAYPVECWVPDPTRNENTRYFGTLTGGENVPPVLHITNTATTVLLDE	240
AAG37198.1	VMNTEHKAYLDKNKAYPVECWVPDPTRNENTRYFGTLTGGENVPPVLHITNTATTVLLDE	240
ALF06241.1	VMNTEHKAYLDKNKAYPVECWVPDPTRNENTRYFGTLTGGENVPPVLHITNTATTVLLDE	240
AFE56117.1	VMNTEHKAYLDKNKAYPVECWVPDPTRNENTRYFGTLTGGENVPPVLHITNTATTVLLDE	240
BAF95600.1	VMNTEHKAYLDKNKAYPVECWVPDPTRNENTRYFGTLTGGENVPPVLHITNTATTVLLDE	240
BAD11836.1	VMNTEHKAYLDKNKAYPVECWVPDPTRNENTRYFGTLTGGENVPPVLHITNTATTVLLDE	240
BAD11842.1	VMNTEHKAYLDKNKAYPVECWVPDPTRNENTRYFGTLTGGENVPPVLHITNTATTVLLDE	240
BAD11848.1	VMNTEHKAYLDKNKAYPVECWVPDPTRNENTRYFGTLTGGENVPPVLHITNTATTVLLDE	240
AAM89327.1	VMNTEHKAYLDKNKAYPVECWVPDPTRNENTRYFGTLTGGENVPPVLHITNTATTVLLDE	240
AAM89339.1	VMNTEHKAYLDKNKAYPVECWVPDPTRNENTRYFGTLTGGENVPPVLHITNTATTVLLDE	240
BAC15707.1	VMNTEHKAYLDKNKAYPVECWVPDPTRNENTRYFGTLTGGENVPPVLHITNTATTVLLDE	240
BAB68833.1	VMNTEHKAYLDKNKAYPVECWVPDPTRNENTRYFGTLTGGENVPPVLHITNTATTVLLDE	240
BAB68839.1	VMNTEHKAYLDKNKAYPVECWVPDPTRNENTRYFGTLTGGENVPPVLHITNTATTVLLDE	240
AYM55604.1	VMNTEHKAYLDKNKAYPVECWVPDPTRNENTRYFGTLTGGENVPPVLHITNTATTVLLDE	240
BAV69355.1	VMNTEHKAYLDKNKAYPVECWVPDPTRNENTRYFGTLTGGENVPPVLHITNTATTVLLDE	240
ALF06220.1	VMNTEHKAYLDKNKAYPVECWVPDPTRNENTRYFGTLTGGENVPPVLHITNTATTVLLDE	240
ALF06247.1	VMNTEHKAYLDKNKAYPVECWVPDPTRNENTRYFGTLTGGENVPPVLHITNTATTVLLDE	240
ALF06250.1	VMNTEHKAYLDKNKAYPVECWVPDPTRNENTRYFGTLTGGENVPPVLHITNTATTVLLDE	240
ALF06253.1	VMNTEHKAYLDKNKAYPVECWVPDPTRNENTRYFGTLTGGENVPPVLHITNTATTVLLDE	240
ALF06259.1	VMNTEHKAYLDKNKAYPVECWVPDPTRNENTRYFGTLTGGENVPPVLHITNTATTVLLDE	240
ALF06265.1	VMNTEHKAYLDKNKAYPVECWVPDPTRNENTRYFGTLTGGENVPPVLHITNTATTVLLDE	240
CCW72502.1	VMNTEHKAYLDKNKAYPVECWVPDPTRNENTRYFGTLTGGENVPPVLHITNTATTVLLDE	240
BAF40757.1	VMNTEHKAYLDKNKAYPVECWVPDPTRNENTRYFGTLTGGENVPPVLHITNTATTVLLDE	240
BAF40763.1	VMNTEHKAYLDKNKAYPVECWVPDPTRNENTRYFGTLTGGENVPPVLHITNTATTVLLDE	240
BAF40769.1	VMNTEHKAYLDKNKAYPVECWVPDPTRNENTRYFGTLTGGENVPPVLHITNTATTVLLDE	240
BAF40775.1	VMNTEHKAYLDKNKAYPVECWVPDPTRNENTRYFGTLTGGENVPPVLHITNTATTVLLDE	240
BAE94732.1	VMNTEHKAYLDKNKAYPVECWVPDPTRNENTRYFGTLTGGENVPPVLHITNTATTVLLDE	240
BAE94738.1	VMNTEHKAYLDKNKAYPVECWVPDPTRNENTRYFGTLTGGENVPPVLHITNTATTVLLDE	240
BAE00129.1	VMNTEHKAYLDKNKAYPVECWVPDPTRNENTRYFGTLTGGENVPPVLHITNTATTVLLDE	240
BAE00141.1	VMNTEHKAYLDKNKAYPVECWVPDPTRNENTRYFGTLTGGENVPPVLHITNTATTVLLDE	240
BAD11824.1	VMNTEHKAYLDKNKAYPVECWVPDPTRNENTRYFGTLTGGENVPPVLHITNTATTVLLDE	240
BAD11830.1	VMNTEHKAYLDKNKAYPVECWVPDPTRNENTRYFGTLTGGENVPPVLHITNTATTVLLDE	240
BAD11854.1	VMNTEHKAYLDKNKAYPVECWVPDPTRNENTRYFGTLTGGENVPPVLHITNTATTVLLDE	240
BAD11860.1	VMNTEHKAYLDKNKAYPVECWVPDPTRNENTRYFGTLTGGENVPPVLHITNTATTVLLDE	240
BAD11866.1	VMNTEHKAYLDKNKAYPVECWVPDPTRNENTRYFGTLTGGENVPPVLHITNTATTVLLDE	240

```
BAD11872.1      VMNTEHKAYLDKNKAYPVECWVPDPTRNENTRYFGTLTGGENVPPVLHITNTATTVLLDE  240
BAD11878.1      VMNTEHKAYLDKNKAYPVECWVPDPTRNENTRYFGTLTGGENVPPVLHITNTATTVLLDE  240
BAD11884.1      VMNTEHKAYLDKNKAYPVECWVPDPTRNENTRYFGTLTGGENVPPVLHITNTATTVLLDE  240
BAD11890.1      VMNTEHKAYLDKNKAYPVECWVPDPTRNENTRYFGTLTGGENVPPVLHITNTATTVLLDE  240
BAD11902.1      VMNTEHKAYLDKNKAYPVECWVPDPTRNENTRYFGTLTGGENVPPVLHITNTATTVLLDE  240
BAD06233.1      VMNTEHKAYLDKNKAYPVECWVPDPTRNENTRYFGTLTGGENVPPVLHITNTATTVLLDE  240
BAD06066.1      VMNTEHKAYLDKNKAYPVECWVPDPTRNENTRYFGTLTGGENVPPVLHITNTATTVLLDE  240
BAD06078.1      VMNTEHKAYLDKNKAYPVECWVPDPTRNENTRYFGTLTGGENVPPVLHITNTATTVLLDE  240
BAD06114.1      VMNTEHKAYLDKNKAYPVECWVPDPTRNENTRYFGTLTGGENVPPVLHITNTATTVLLDE  240
BAD06132.1      VMNTEHKAYLDKNKAYPVECWVPDPTRNENTRYFGTLTGGENVPPVLHITNTATTVLLDE  240
BAD06138.1      VMNTEHKAYLDKNKAYPVECWVPDPTRNENTRYFGTLTGGENVPPVLHITNTATTVLLDE  240
BAD06162.1      VMNTEHKAYLDKNKAYPVECWVPDPTRNENTRYFGTLTGGENVPPVLHITNTATTVLLDE  240
BAD06168.1      VMNTEHKAYLDKNKAYPVECWVPDPTRNENTRYFGTLTGGENVPPVLHITNTATTVLLDE  240
BAC82365.1      VMNTEHKAYLDKNKAYPVECWVPDPTRNENTRYFGTLTGGENVPPVLHITNTATTVLLDE  240
BAC81846.1      VMNTEHKAYLDKNKAYPVECWVPDPTRNENTRYFGTLTGGENVPPVLHITNTATTVLLDE  240
BAC81864.1      VMNTEHKAYLDKNKAYPVECWVPDPTRNENTRYFGTLTGGENVPPVLHITNTATTVLLDE  240
BAC81870.1      VMNTEHKAYLDKNKAYPVECWVPDPTRNENTRYFGTLTGGENVPPVLHITNTATTVLLDE  240
BAC81874.1      VMNTEHKAYLDKNKAYPVECWVPDPTRNENTRYFGTLTGGENVPPVLHITNTATTVLLDE  240
BAC81880.1      VMNTEHKAYLDKNKAYPVECWVPDPTRNENTRYFGTLTGGENVPPVLHITNTATTVLLDE  240
BAC81886.1      VMNTEHKAYLDKNKAYPVECWVPDPTRNENTRYFGTLTGGENVPPVLHITNTATTVLLDE  240
BAC81892.1      VMNTEHKAYLDKNKAYPVECWVPDPTRNENTRYFGTLTGGENVPPVLHITNTATTVLLDE  240
BAC81898.1      VMNTEHKAYLDKNKAYPVECWVPDPTRNENTRYFGTLTGGENVPPVLHITNTATTVLLDE  240
BAC81928.1      VMNTEHKAYLDKNKAYPVECWVPDPTRNENTRYFGTLTGGENVPPVLHITNTATTVLLDE  240
BAC81934.1      VMNTEHKAYLDKNKAYPVECWVPDPTRNENTRYFGTLTGGENVPPVLHITNTATTVLLDE  240
BAC81940.1      VMNTEHKAYLDKNKAYPVECWVPDPTRNENTRYFGTLTGGENVPPVLHITNTATTVLLDE  240
BAC81964.1      VMNTEHKAYLDKNKAYPVECWVPDPTRNENTRYFGTLTGGENVPPVLHITNTATTVLLDE  240
AAM89321.1      VMNTEHKAYLDKNKAYPVECWVPDPTRNENTRYFGTLTGGENVPPVLHITNTATTVLLDE  240
AAM89333.1      VMNTEHKAYLDKNKAYPVECWVPDPTRNENTRYFGTLTGGENVPPVLHITNTATTVLLDE  240
AAM89345.1      VMNTEHKAYLDKNKAYPVECWVPDPTRNENTRYFGTLTGGENVPPVLHITNTATTVLLDE  240
BAC66376.1      VMNTEHKAYLDKNKAYPVECWVPDPTRNENTRYFGTLTGGENVPPVLHITNTATTVLLDE  240
BAC22834.1      VMNTEHKAYLDKNKAYPVECWVPDPTRNENTRYFGTLTGGENVPPVLHITNTATTVLLDE  240
BAC22840.1      VMNTEHKAYLDKNKAYPVECWVPDPTRNENTRYFGTLTGGENVPPVLHITNTATTVLLDE  240
BAC22852.1      VMNTEHKAYLDKNKAYPVECWVPDPTRNENTRYFGTLTGGENVPPVLHITNTATTVLLDE  240
BAC22870.1      VMNTEHKAYLDKNKAYPVECWVPDPTRNENTRYFGTLTGGENVPPVLHITNTATTVLLDE  240
BAC22876.1      VMNTEHKAYLDKNKAYPVECWVPDPTRNENTRYFGTLTGGENVPPVLHITNTATTVLLDE  240
BAC22882.1      VMNTEHKAYLDKNKAYPVECWVPDPTRNENTRYFGTLTGGENVPPVLHITNTATTVLLDE  240
BAC22888.1      VMNTEHKAYLDKNKAYPVECWVPDPTRNENTRYFGTLTGGENVPPVLHITNTATTVLLDE  240
BAC15629.1      VMNTEHKAYLDKNKAYPVECWVPDPTRNENTRYFGTLTGGENVPPVLHITNTATTVLLDE  240
BAC15659.1      VMNTEHKAYLDKNKAYPVECWVPDPTRNENTRYFGTLTGGENVPPVLHITNTATTVLLDE  240
BAC15671.1      VMNTEHKAYLDKNKAYPVECWVPDPTRNENTRYFGTLTGGENVPPVLHITNTATTVLLDE  240
BAC15677.1      VMNTEHKAYLDKNKAYPVECWVPDPTRNENTRYFGTLTGGENVPPVLHITNTATTVLLDE  240
BAC15689.1      VMNTEHKAYLDKNKAYPVECWVPDPTRNENTRYFGTLTGGENVPPVLHITNTATTVLLDE  240
BAC15713.1      VMNTEHKAYLDKNKAYPVECWVPDPTRNENTRYFGTLTGGENVPPVLHITNTATTVLLDE  240
BAC15731.1      VMNTEHKAYLDKNKAYPVECWVPDPTRNENTRYFGTLTGGENVPPVLHITNTATTVLLDE  240
BAC15737.1      VMNTEHKAYLDKNKAYPVECWVPDPTRNENTRYFGTLTGGENVPPVLHITNTATTVLLDE  240
BAC15743.1      VMNTEHKAYLDKNKAYPVECWVPDPTRNENTRYFGTLTGGENVPPVLHITNTATTVLLDE  240
AAL37641.1      VMNTEHKAYLDKNKAYPVECWVPDPTRNENTRYFGTLTGGENVPPVLHITNTATTVLLDE  240
AAL37653.1      VMNTEHKAYLDKNKAYPVECWVPDPTRNENTRYFGTLTGGENVPPVLHITNTATTVLLDE  240
AAL37659.1      VMNTEHKAYLDKNKAYPVECWVPDPTRNENTRYFGTLTGGENVPPVLHITNTATTVLLDE  240
AAL37671.1      VMNTEHKAYLDKNKAYPVECWVPDPTRNENTRYFGTLTGGENVPPVLHITNTATTVLLDE  240
AAL37683.1      VMNTEHKAYLDKNKAYPVECWVPDPTRNENTRYFGTLTGGENVPPVLHITNTATTVLLDE  240
BAB68791.1      VMNTEHKAYLDKNKAYPVECWVPDPTRNENTRYFGTLTGGENVPPVLHITNTATTVLLDE  240
BAB68821.1      VMNTEHKAYLDKNKAYPVECWVPDPTRNENTRYFGTLTGGENVPPVLHITNTATTVLLDE  240
BAB68845.1      VMNTEHKAYLDKNKAYPVECWVPDPTRNENTRYFGTLTGGENVPPVLHITNTATTVLLDE  240
BAB68851.1      VMNTEHKAYLDKNKAYPVECWVPDPTRNENTRYFGTLTGGENVPPVLHITNTATTVLLDE  240
BAB68857.1      VMNTEHKAYLDKNKAYPVECWVPDPTRNENTRYFGTLTGGENVPPVLHITNTATTVLLDE  240
BAB68869.1      VMNTEHKAYLDKNKAYPVECWVPDPTRNENTRYFGTLTGGENVPPVLHITNTATTVLLDE  240
BAB68875.1      VMNTEHKAYLDKNKAYPVECWVPDPTRNENTRYFGTLTGGENVPPVLHITNTATTVLLDE  240
AAK97910.1      VMNTEHKAYLDKNKAYPVECWVPDPTRNENTRYFGTLTGGENVPPVLHITNTATTVLLDE  240
AAK97916.1      VMNTEHKAYLDKNKAYPVECWVPDPTRNENTRYFGTLTGGENVPPVLHITNTATTVLLDE  240
AAK97922.1      VMNTEHKAYLDKNKAYPVECWVPDPTRNENTRYFGTLTGGENVPPVLHITNTATTVLLDE  240
AAK97928.1      VMNTEHKAYLDKNKAYPVECWVPDPTRNENTRYFGTLTGGENVPPVLHITNTATTVLLDE  240
AAK97934.1      VMNTEHKAYLDKNKAYPVECWVPDPTRNENTRYFGTLTGGENVPPVLHITNTATTVLLDE  240
AAK97940.1      VMNTEHKAYLDKNKAYPVECWVPDPTRNENTRYFGTLTGGENVPPVLHITNTATTVLLDE  240
```

AAK97946.1	VMNTEHKAYLDKNKAYPVECWVPDPTRNENTRYFGTLTGGENVPPVLHITNTATTVLLDE	240
AAK97952.1	VMNTEHKAYLDKNKAYPVECWVPDPTRNENTRYFGTLTGGENVPPVLHITNTATTVLLDE	240
AAK97958.1	VMNTEHKAYLDKNKAYPVECWVPDPTRNENTRYFGTLTGGENVPPVLHITNTATTVLLDE	240
AAK97982.1	VMNTEHKAYLDKNKAYPVECWVPDPTRNENTRYFGTLTGGENVPPVLHITNTATTVLLDE	240
AAK97988.1	VMNTEHKAYLDKNKAYPVECWVPDPTRNENTRYFGTLTGGENVPPVLHITNTATTVLLDE	240
AAK97994.1	VMNTEHKAYLDKNKAYPVECWVPDPTRNENTRYFGTLTGGENVPPVLHITNTATTVLLDE	240
BAB11698.1	VMNTEHKAYLDKNKAYPVECWVPDPTRNENTRYFGTLTGGENVPPVLHITNTATTVLLDE	240
BAB11704.1	VMNTEHKAYLDKNKAYPVECWVPDPTRNENTRYFGTLTGGENVPPVLHITNTATTVLLDE	240
AAC59478.1	VMNTEHKAYLDKNKAYPVECWVPDPTRNENTRYFGTLTGGENVPPVLHITNTATTVLLDE	240
AAC59484.1	VMNTEHKAYLDKNKAYPVECWVPDPTRNENTRYFGTLTGGENVPPVLHITNTATTVLLDE	240
AAC59490.1	VMNTEHKAYLDKNKAYPVECWVPDPTRNENTRYFGTLTGGENVPPVLHITNTATTVLLDE	240
BAA01963.1	VMNTEHKAYLDKNKAYPVECWVPDPTRNENTRYFGTLTGGENVPPVLHITNTATTVLLDE	240
BAA01964.1	VMNTEHKAYLDKNKAYPVECWVPDPTRNENTRYFGTLTGGENVPPVLHITNTATTVLLDE	240
BAB93068.1	VMNTEHKAYLDKNKAYPVECWVPDPTRNENTRYFGTLTGGENVPPVLHITNTATTVLLDE	240
ASV51856.1	VMNTEHKAYLDKNKAYPVECWVPDPTRNENTRYFGTLTGGENVPPVLHITNTATTVLLDE	240
ANH21097.1	VMNTEHKAYLDKNKAYPVECWVPDPTRNENTRYFGTLTGGENVPPVLHITNTATTVLLDE	240
AEC15167.1	VMNTEHKAYLDKNKAYPVECWVPDPTRNENTRYFGTLTGGENVPPVLHITNTATTVLLDE	240
AEC15168.1	VMNTEHKAYLDKNKAYPVECWVPDPTRNENTRYFGTLTGGENVPPVLHITNTATTVLLDE	240
AEC15169.1	VMNTEHKAYLDKNKAYPVECWVPDPTRNENTRYFGTLTGGENVPPVLHITNTATTVLLDE	240
QTJ15168.1	VMNTEHKAYLDKNKAYPVECWVPDPTRNENTRYFGTLTGGENVPPVLHITNTATTVLLDE	240
AEC15173.1	VMNTEHKAYLDKNKAYPVECWVPDPTRNENTRYFGTLTGGENVPPVLHITNTATTVLLDE	240
AEC15174.1	VMNTEHKAYLDKNKAYPVECWVPDPTRNENTRYFGTLTGGENVPPVLHITNTATTVLLDE	240
AEC15175.1	VMNTEHKAYLDKNKAYPVECWVPDPTRNENTRYFGTLTGGENVPPVLHITNTATTVLLDE	240
AEC15176.1	VMNTEHKAYLDKNKAYPVECWVPDPTRNENTRYFGTLTGGENVPPVLHITNTATTVLLDE	240
AEC15177.1	VMNTEHKAYLDKNKAYPVECWVPDPTRNENTRYFGTLTGGENVPPVLHITNTATTVLLDE	240
AEC15178.1	VMNTEHKAYLDKNKAYPVECWVPDPTRNENTRYFGTLTGGENVPPVLHITNTATTVLLDE	240
AEC15179.1	VMNTEHKAYLDKNKAYPVECWVPDPTRNENTRYFGTLTGGENVPPVLHITNTATTVLLDE	240
AEC15180.1	VMNTEHKAYLDKNKAYPVECWVPDPTRNENTRYFGTLTGGENVPPVLHITNTATTVLLDE	240
AEC15181.1	VMNTEHKAYLDKNKAYPVECWVPDPTRNENTRYFGTLTGGENVPPVLHITNTATTVLLDE	240
AEC15182.1	VMNTEHKAYLDKNKAYPVECWVPDPTRNENTRYFGTLTGGENVPPVLHITNTATTVLLDE	240
AEC15183.1	VMNTEHKAYLDKNKAYPVECWVPDPTRNENTRYFGTLTGGENVPPVLHITNTATTVLLDE	240
AEC15184.1	VMNTEHKAYLDKNKAYPVECWVPDPTRNENTRYFGTLTGGENVPPVLHITNTATTVLLDE	240
AEC15185.1	VMNTEHKAYLDKNKAYPVECWVPDPTRNENTRYFGTLTGGENVPPVLHITNTATTVLLDE	240
AEC15187.1	VMNTEHKAYLDKNKAYPVECWVPDPTRNENTRYFGTLTGGENVPPVLHITNTATTVLLDE	240
AEC15188.1	VMNTEHKAYLDKNKAYPVECWVPDPTRNENTRYFGTLTGGENVPPVLHITNTATTVLLDE	240
AEC15189.1	VMNTEHKAYLDKNKAYPVECWVPDPTRNENTRYFGTLTGGENVPPVLHITNTATTVLLDE	240
AEC15190.1	VMNTEHKAYLDKNKAYPVECWVPDPTRNENTRYFGTLTGGENVPPVLHITNTATTVLLDE	240
AEC15191.1	VMNTEHKAYLDKNKAYPVECWVPDPTRNENTRYFGTLTGGENVPPVLHITNTATTVLLDE	240
AEC15193.1	VMNTEHKAYLDKNKAYPVECWVPDPTRNENTRYFGTLTGGENVPPVLHITNTATTVLLDE	240
AEC14871.1	VMNTEHKAYLDKNKAYPVECWVPDPTRNENTRYFGTLTGGENVPPVLHITNTATTVLLDE	240
AEC15222.1	VMNTEHKAYLDKNKAYPVECWVPDPTRNENTRYFGTLTGGENVPPVLHITNTATTVLLDE	240
AEC15130.1	VMNTEHKAYLDKNKAYPVECWVPDPTRNENTRYFGTLTGGENVPPVLHITNTATTVLLDE	240
AEC15144.1	VMNTEHKAYLDKNKAYPVECWVPDPTRNENTRYFGTLTGGENVPPVLHITNTATTVLLDE	240
AEC15154.1	VMNTEHKAYLDKNKAYPVECWVPDPTRNENTRYFGTLTGGENVPPVLHITNTATTVLLDE	240
AEC15157.1	VMNTEHKAYLDKNKAYPVECWVPDPTRNENTRYFGTLTGGENVPPVLHITNTATTVLLDE	240
AEC15160.1	VMNTEHKAYLDKNKAYPVECWVPDPTRNENTRYFGTLTGGENVPPVLHITNTATTVLLDE	240
AEC15231.1	VMNTEHKAYLDKNKAYPVECWVPDPTRNENTRYFGILTGGENVPPVLHITNTATTVLLDE	240
ALF06191.1	VMNTEHKAYLDKNKAYPVECWVPDPTRNENTRYFGTLTGGENVPPVLHITNTATTVLLDE	240
WLD48112.1	VMNTEHKAYLDKNKAYPVECWVPDPTRNENTRYFGTLTGGENVPPVLHITNTATTVLLDE	240
QWT77140.1	VMNTEHKAYLDKNKAYPVECWVPDPTRNENTRYFGTLTGGENVPPVLHITNTATTVLLDE	240
QTJ15174.1	VMNTEHKAYLDKNKAYPVECWVPDPTRNENTRYFGTLTGGENVPPVLHITNTATTVLLDE	240
QTJ15186.1	VMNTEHKAYLDKNKAYPVECWVPDPTRNENTRYFGTLTGGENVPPVLHITNTATTVLLDE	240
QTJ15192.1	VMNTEHKAYLDKNKAYPVECWVPDPTRNENTRYFGTLTGGENVPPVLHITNTATTVLLDE	240
QTJ15198.1	VMNTEHKAYLDKNKAYPVECWVPDPTRNENTRYFGTLTGGENVPPVLHITNTATTVLLDE	240
ASV51772.1	VMNTEHKAYLDKNKAYPVECWVPDPTRNENTRYFGTLTGGENVPPVLHITNTATTVLLDE	240
CCW72478.1	VMNTEHKAYLDKNKAYPVECWVPDPTRNENTRYFGTLTGGENVPPVLHITNTATTVLLDE	240
CCW72481.1	VMNTEHKAYLDKNKAYPVECWVPDPTRNENTRYFGTLTGGENVPPVLHITNTATTVLLDE	240
CCW72487.1	VMNTEHKAYLDKNKAYPVECWVPDPTRNENTRYFGTLTGGENVPPVLHITNTATTVLLDE	240
CCW72492.1	VMNTEHKAYLDKNKAYPVECWVPDPTRNENTRYFGTLTGGENVPPVLHITNTATTVLLDE	240
CCW72496.1	VMNTEHKAYLDKNKAYPVECWVPDPTRNENTRYFGTLTGGENVPPVLHITNTATTVLLDE	240
CCW72499.1	VMNTEHKAYLDKNKAYPVECWVPDPTRNENTRYFGTLTGGENVPPVLHITNTATTVLLDE	240
CCW72504.1	VMNTEHKAYLDKNKAYPVECWVPDPTRNENTRYFGTLTGGENVPPVLHITNTATTVLLDE	240
CCW72508.1	VMNTEHKAYLDKNKAYPVECWVPDPTRNENTRYFGTLTGGENVPPVLHITNTATTVLLDE	240
CCW72510.1	VMNTEHKAYLDKNKAYPVECWVPDPTRNENTRYFGTLTGGENVPPVLHITNTATTVLLDE	240

CCW72519.1	VMNTEHKAYLDKNKAYPVECWVPDPTRNENTRYFGTLTGGENVPPVLHITNTATTVLLDE	240
CCW72521.1	VMNTEHKAYLDKNKAYPVECWVPDPTRNENTRYFGTLTGGENVPPVLHITNTATTVLLDE	240
AEC14864.1	VMNTEHKAYLDKNKAYPVECWVPDPTRNENTRYFGTLTGGENVPPVLHITNTATTVLLDE	240
AEC14865.1	VMNTEHKAYLDKNKAYPVECWVPDPTRNENTRYFGTLTGGENVPPVLHITNTATTVLLDE	240
AEC14866.1	VMNTEHKAYLDKNKAYPVECWVPDPTRNENTRYFGTLTGGENVPPVLHITNTATTVLLDE	240
AEC14867.1	VMNTEHKAYLDKNKAYPVECWVPDPTRNENTRYFGTLTGGENVPPVLHITNTATTVLLDE	240
AEC14868.1	VMNTEHKAYLDKNKAYPVECWVPDPTRNENTRYFGTLTGGENVPPVLHITNTATTVLLDE	240
AEC14869.1	VMNTEHKAYLDKNKAYPVECWVPDPTRNENTRYFGTLTGGENVPPVLHITNTATTVLLDE	240
AEC14870.1	VMNTEHKAYLDKNKAYPVECWVPDPTRNENTRYFGTLTGGENVPPVLHITNTATTVLLDE	240
AEC14872.1	VMNTEHKAYLDKNKAYPVECWVPDPTRNENTRYFGTLTGGENVPPVLHITNTATTVLLDE	240
AEC14873.1	VMNTEHKAYLDKNKAYPVECWVPDPTRNENTRYFGTLTGGENVPPVLHITNTATTVLLDE	240
AEC14874.1	VMNTEHKAYLDKNKAYPVECWVPDPTRNENTRYFGTLTGGENVPPVLHITNTATTVLLDE	240
AEC14875.1	VMNTEHKAYLDKNKAYPVECWVPDPTRNENTRYFGTLTGGENVPPVLHITNTATTVLLDE	240
AEC14876.1	VMNTEHKAYLDKNKAYPVECWVPDPTRNENTRYFGTLTGGENVPPVLHITNTATTVLLDE	240
AEC14877.1	VMNTEHKAYLDKNKAYPVECWVPDPTRNENTRYFGTLTGGENVPPVLHITNTATTVLLDE	240
AEC14878.1	VMNTEHKAYLDKNKAYPVECWVPDPTRNENTRYFGTLTGGENVPPVLHITNTATTVLLDE	240
AEC14879.1	VMNTEHKAYLDKNKAYPVECWVPDPTRNENTRYFGTLTGGENVPPVLHITNTATTVLLDE	240
AEC14880.1	VMNTEHKAYLDKNKAYPVECWVPDPTRNENTRYFGTLTGGENVPPVLHITNTATTVLLDE	240
AEC14881.1	VMNTEHKAYLDKNKAYPVECWVPDPTRNENTRYFGTLTGGENVPPVLHITNTATTVLLDE	240
AEC14882.1	VMNTEHKAYLDKNKAYPVECWVPDPTRNENTRYFGTLTGGENVPPVLHITNTATTVLLDE	240
AEC14883.1	VMNTEHKAYLDKNKAYPVECWVPDPTRNENTRYFGTLTGGENVPPVLHITNTATTVLLDE	240
AEC14884.1	VMNTEHKAYLDKNKAYPVECWVPDPTRNENTRYFGTLTGGENVPPVLHITNTATTVLLDE	240
AEC14885.1	VMNTEHKAYLDKNKAYPVECWVPDPTRNENTRYFGTLTGGENVPPVLHITNTATTVLLDE	240
AEC14886.1	VMNTEHKAYLDKNKAYPVECWVPDPTRNENTRYFGTLTGGENVPPVLHITNTATTVLLDE	240
AEC14887.1	VMNTEHKAYLDKNKAYPVECWVPDPTRNENTRYFGTLTGGENVPPVLHITNTATTVLLDE	240
AEC14888.1	VMNTEHKAYLDKNKAYPVECWVPDPTRNENTRYFGTLTGGENVPPVLHITNTATTVLLDE	240
AEC14889.1	VMNTEHKAYLDKNKAYPVECWVPDPTRNENTRYFGTLTGGENVPPVLHITNTATTVLLDE	240
AEC14890.1	VMNTEHKAYLDKNKAYPVECWVPDPTRNENTRYFGTLTGGENVPPVLHITNTATTVLLDE	240
AEC14891.1	VMNTEHKAYLDKNKAYPVECWVPDPTRNENTRYFGTLTGGENVPPVLHITNTATTVLLDE	240
AEC14892.1	VMNTEHKAYLDKNKAYPVECWVPDPTRNENTRYFGTLTGGENVPPVLHITNTATTVLLDE	240
AEC14894.1	VMNTEHKAYLDKNKAYPVECWVPDPTRNENTRYFGTLTGGENVPPVLHITNTATTVLLDE	240
AEC14895.1	VMNTEHKAYLDKNKAYPVECWVPDPTRNENTRYFGTLTGGENVPPVLHITNTATTVLLDE	240
AEC14896.1	VMNTEHKAYLDKNKAYPVECWVPDPTRNENTRYFGTLTGGENVPPVLHITNTATTVLLDE	240
AEC14897.1	VMNTEHKAYLDKNKAYPVECWVPDPTRNENTRYFGTLTGGENVPPVLHITNTATTVLLDE	240
AEC14898.1	VMNTEHKAYLDKNKAYPVECWVPDPTRNENTRYFGTLTGGENVPPVLHITNTATTVLLDE	240
AEC14899.1	VMNTEHKAYLDKNKAYPVECWVPDPTRNENTRYFGTLTGGENVPPVLHITNTATTVLLDE	240
AEC14900.1	VMNTEHKAYLDKNKAYPVECWVPDPTRNENTRYFGTLTGGENVPPVLHITNTATTVLLDE	240
AEC14901.1	VMNTEHKAYLDKNKAYPVECWVPDPTRNENTRYFGTLTGGENVPPVLHITNTATTVLLDE	240
AEC14902.1	VMNTEHKAYLDKNKAYPVECWVPDPTRNENTRYFGTLTGGENVPPVLHITNTATTVLLDE	240
AEC14903.1	VMNTEHKAYLDKNKAYPVECWVPDPTRNENTRYFGTLTGGENVPPVLHITNTATTVLLDE	240
AEC14904.1	VMNTEHKAYLDKNKAYPVECWVPDPTRNENTRYFGTLTGGENVPPVLHITNTATTVLLDE	240
AEC14905.1	VMNTEHKAYLDKNKAYPVECWVPDPTRNENTRYFGTLTGGENVPPVLHITNTATTVLLDE	240
AEC14907.1	VMNTEHKAYLDKNKAYPVECWVPDPTRNENTRYFGTLTGGENVPPVLHITNTATTVLLDE	240
AEC14908.1	VMNTEHKAYLDKNKAYPVECWVPDPTRNENTRYFGTLTGGENVPPVLHITNTATTVLLDE	240
AEC14909.1	VMNTEHKAYLDKNKAYPVECWVPDPTRNENTRYFGTLTGGENVPPVLHITNTATTVLLDE	240
AEC14910.1	VMNTEHKAYLDKNKAYPVECWVPDPTRNENTRYFGTLTGGENVPPVLHITNTATTVLLDE	240
AEC14911.1	VMNTEHKAYLDKNKAYPVECWVPDPTRNENTRYFGTLTGGENVPPVLHITNTATTVLLDE	240
AEC15164.1	VMNTEHKAYLDKNKAYPVECWVPDPTRNENTRYFGTLTGGENVPPVLHITNTATTVLLDE	240
BAF95594.1	VMNTEHKAYLDKNKAYPVECWVPDPTRNENTRYFGTLTGGENVPPVLHITNTATTVLLDE	240
BAF95606.1	VMNTEHKAYLDKNKAYPVECWVPDPTRNENTRYFGTLTGGENVPPVLHITNTATTVLLDE	240
BAF40745.1	VMNTEHKAYLDKNKAYPVECWVPDPTRNENTRYFGTLTGGENVPPVLHITNTATTVLLDE	240
BAF40751.1	VMNTEHKAYLDKNKAYPVECWVPDPTRNENTRYFGTLTGGENVPPVLHITNTATTVLLDE	240
BAF40781.1	VMNTEHKAYLDKNKAYPVECWVPDPTRNENTRYFGTLTGGENVPPVLHITNTATTVLLDE	240
BAF40787.1	VMNTEHKAYLDKNKAYPVECWVPDPTRNENTRYFGTLTGGENVPPVLHITNTATTVLLDE	240
BAF40793.1	VMNTEHKAYLDKNKAYPVECWVPDPTRNENTRYFGTLTGGENVPPVLHITNTATTVLLDE	240
BAF40799.1	VMNTEHKAYLDKNKAYPVECWVPDPTRNENTRYFGTLTGGENVPPVLHITNTATTVLLDE	240
BAF40811.1	VMNTEHKAYLDKNKAYPVECWVPDPTRNENTRYFGTLTGGENVPPVLHITNTATTVLLDE	240
BAF40817.1	VMNTEHKAYLDKNKAYPVECWVPDPTRNENTRYFGTLTGGENVPPVLHITNTATTVLLDE	240
BAF40823.1	VMNTEHKAYLDKNKAYPVECWVPDPTRNENTRYFGTLTGGENVPPVLHITNTATTVLLDE	240
BAF40829.1	VMNTEHKAYLDKNKAYPVECWVPDPTRNENTRYFGTLTGGENVPPVLHITNTATTVLLDE	240
BAF40835.1	VMNTEHKAYLDKNKAYPVECWVPDPTRNENTRYFGTLTGGENVPPVLHITNTATTVLLDE	240
BAF40841.1	VMNTEHKAYLDKNKAYPVECWVPDPTRNENTRYFGTLTGGENVPPVLHITNTATTVLLDE	240
BAF40847.1	VMNTEHKAYLDKNKAYPVECWVPDPTRNENTRYFGTLTGGENVPPVLHITNTATTVLLDE	240
BAC81834.1	VMNTEHKAYLDKNKAYPVECWVPDPTRNENTRYFGTLTGGENVPPVLHITNTATTVLLDE	240

```
BAC81840.1      VMNTEHKAYLDKNKAYPVECWVPDPTRNENTRYFGTLTGGENVPPVLHITNTATTVLLDE  240
AAN85461.1      VMNTEHKAYLDKNKAYPVECWVPDPTRNENTRYFGTLTGGENVPPVLHITNTATTVLLDE  240
AAN85467.1      VMNTEHKAYLDKNKAYPVECWVPDPTRNENTRYFGTLTGGENVPPVLHITNTATTVLLDE  240
AAN85473.1      VMNTEHKAYLDKNKAYPVECWVPDPTRNENTRYFGTLTGGENVPPVLHITNTATTVLLDE  240
BAC22762.1      VMNTEHKAYLDKNKAYPVECWVPDPTRNENTRYFGTLTGGENVPPVLHITNTATTVLLDE  240
BAC22768.1      VMNTEHKAYLDKNKAYPVECWVPDPTRNENTRYFGTLTGGENVPPVLHITNTATTVLLDE  240
BAC22774.1      VMNTEHKAYLDKNKAYPVECWVPDPTRNENTRYFGTLTGGENVPPVLHITNTATTVLLDE  240
BAC22780.1      VMNTEHKAYLDKNKAYPVECWVPDPTRNENTRYFGTLTGGENVPPVLHITNTATTVLLDE  240
BAC22786.1      VMNTEHKAYLDKNKAYPVECWVPDPTRNENTRYFGTLTGGENVPPVLHITNTATTVLLDE  240
BAB68809.1      VMNTEHKAYLDKNKAYPVECWVPDPTRNENTRYFGTLTGGENVPPVLHITNTATTVLLDE  240
BAB68815.1      VMNTEHKAYLDKNKAYPVECWVPDPTRNENTRYFGTLTGGENVPPVLHITNTATTVLLDE  240
BAB68827.1      VMNTEHKAYLDKNKAYPVECWVPDPTRNENTRYFGTLTGGENVPPVLHITNTATTVLLDE  240
AAC59496.1      VMNTEHKAYLDKNKAYPVECWVPDPTRNENTRYFGTLTGGENVPPVLHITNTATTVLLDE  240
AAC59502.1      VMNTEHKAYLDKNKAYPVECWVPDPTRNENTRYFGTLTGGENVPPVLHITNTATTVLLDE  240
AAC59508.1      VMNTEHKAYLDKNKAYPVECWVPDPTRNENTRYFGTLTGGENVPPVLHITNTATTVLLDE  240
AAC59514.1      VMNTEHKAYLDKNKAYPVECWVPDPTRNENTRYFGTLTGGENVPPVLHITNTATTVLLDE  240
AAC59520.1      VMNTEHKAYLDKNKAYPVECWVPDPTRNENTRYFGTLTGGENVPPVLHITNTATTVLLDE  240
AAB62680.1      VMNTEHKAYLDKNKAYPVECWVPDPTRNENTRYFGTLTGGENVPPVLHITNTATTVLLDE  240
BAA01961.1      VMNTEHKAYLDKNKAYPVECWVPDPTRNENTRYFGTLTGGENVPPVLHITNTATTVLLDE  240
WLD48082.1      VMNTEHKAYLDKNKAYPVECWVPDPTRNENTRYFGTLTGGENVPPVLHITNTATTVLLDE  240
WLD48088.1      VMNTEHKAYLDKNKAYPVECWVPDPTRNENTRYFGTLTGGENVPPVLHITNTATTVLLDE  240
CCW72516.1      VMNTEHKAYLDKNKAYPVECWVPDPTRNENTRYFGTLTGGENVPPVLHITNTATTVLLDE  240
CCW72523.1      VMNTEHKAYLDKNKAYPVECWVPDPTRNENTRYFGTLTGGENVPPVLHITNTATTVLLDE  240
AEC14783.1      VMNTEHKAYLDKNKAYPVECWVPDPTRNENTRYFGTLTGGENVPPVLHITNTATTVLLDE  240
AEC14784.1      VMNTEHKAYLDKNKAYPVECWVPDPTRNENTRYFGTLTGGENVPPVLHITNTATTVLLDE  240
AEC14785.1      VMNTEHKAYLDKNKAYPVECWVPDPTRNENTRYFGTLTGGENVPPVLHITNTATTVLLDE  240
AEC14786.1      VMNTEHKAYLDKNKAYPVECWVPDPTRNENTRYFGTLTGGENVPPVLHITNTATTVLLDE  240
AEC14787.1      VMNTEHKAYLDKNKAYPVECWVPDPTRNENTRYFGTLTGGENVPPVLHITNTATTVLLDE  240
AAB62687.1      VMNTEHKAYLDKNKAYPVECWVPDPTRNENTRYFGTLTGGENVPPVLHITNTATTVLLDE  240
AEC14893.1      VMNTEHKAYLDKNKAYPVECWVPDPTRNENTRYFGTLTGGENVPPVLHITNTATTVLLDE  240
AEC14906.1      VMNTEHKAYLDKNKAYPVECWVPDPTRNENTRYFGTLTGGENVPPVLHITNTATTVLLDE  240
AEC15118.1      VMNTEHKAYLDKNKAYPVECWVPDPTRNENTRYFGTLTGGENVPPVLHITNTATTVLLDE  240
AEC15120.1      VMNTEHKAYLDKNKAYPVECWVPDPTRNENTRYFGTLTGGENVPPVLHITNTATTVLLDE  240
AEC15121.1      VMNTEHKAYLDKNKAYPVECWVPDPTRNENTRYFGTLTGGENVPPVLHITNTATTVLLDE  240
AEC15122.1      VMNTEHKAYLDKNKAYPVECWVPDPTRNENTRYFGTLTGGENVPPVLHITNTATTVLLDE  240
AEC15124.1      VMNTEHKAYLDKNKAYPVECWVPDPTRNENTRYFGTLTGGENVPPVLHITNTATTVLLDE  240
AEC15125.1      VMNTEHKAYLDKNKAYPVECWVPDPTRNENTRYFGTLTGGENVPPVLHITNTATTVLLDE  240
AEC15127.1      VMNTEHKAYLDKNKAYPVECWVPDPTRNENTRYFGTLTGGENVPPVLHITNTATTVLLDE  240
AEC15129.1      VMNTEHKAYLDKNKAYPVECWVPDPTRNENTRYFGTLTGGENVPPVLHITNTATTVLLDE  240
AEC15131.1      VMNTEHKAYLDKNKAYPVECWVPDPTRNENTRYFGTLTGGENVPPVLHITNTATTVLLDE  240
AEC15133.1      VMNTEHKAYLDKNKAYPVECWVPDPTRNENTRYFGTLTGGENVPPVLHITNTATTVLLDE  240
AEC15135.1      VMNTEHKAYLDKNKAYPVECWVPDPTRNENTRYFGTLTGGENVPPVLHITNTATTVLLDE  240
AEC15136.1      VMNTEHKAYLDKNKAYPVECWVPDPTRNENTRYFGTLTGGENVPPVLHITNTATTVLLDE  240
AEC15137.1      VMNTEHKAYLDKNKAYPVECWVPDPTRNENTRYFGTLTGGENVPPVLHITNTATTVLLDE  240
AEC15138.1      VMNTEHKAYLDKNKAYPVECWVPDPTRNENTRYFGTLTGGENVPPVLHITNTATTVLLDE  240
AEC15139.1      VMNTEHKAYLDKNKAYPVECWVPDPTRNENTRYFGTLTGGENVPPVLHITNTATTVLLDE  240
AEC15141.1      VMNTEHKAYLDKNKAYPVECWVPDPTRNENTRYFGTLTGGENVPPVLHITNTATTVLLDE  240
AEC15142.1      VMNTEHKAYLDKNKAYPVECWVPDPTRNENTRYFGTLTGGENVPPVLHITNTATTVLLDE  240
AEC15143.1      VMNTEHKAYLDKNKAYPVECWVPDPTRNENTRYFGTLTGGENVPPVLHITNTATTVLLDE  240
AEC15145.1      VMNTEHKAYLDKNKAYPVECWVPDPTRNENTRYFGTLTGGENVPPVLHITNTATTVLLDE  240
AEC15146.1      VMNTEHKAYLDKNKAYPVECWVPDPTRNENTRYFGTLTGGENVPPVLHITNTATTVLLDE  240
AEC15147.1      VMNTEHKAYLDKNKAYPVECWVPDPTRNENTRYFGTLTGGENVPPVLHITNTATTVLLDE  240
AEC15148.1      VMNTEHKAYLDKNKAYPVECWVPDPTRNENTRYFGTLTGGENVPPVLHITNTATTVLLDE  240
AEC15149.1      VMNTEHKAYLDKNKAYPVECWVPDPTRNENTRYFGTLTGGENVPPVLHITNTATTVLLDE  240
AEC15150.1      VMNTEHKAYLDKNKAYPVECWVPDPTRNENTRYFGTLTGGENVPPVLHITNTATTVLLDE  240
AEC15151.1      VMNTEHKAYLDKNKAYPVECWVPDPTRNENTRYFGTLTGGENVPPVLHITNTATTVLLDE  240
AEC15152.1      VMNTEHKAYLDKNKAYPVECWVPDPTRNENTRYFGTLTGGENVPPVLHITNTATTVLLDE  240
AEC15153.1      VMNTEHKAYLDKNKAYPVECWVPDPTRNENTRYFGTLTGGENVPPVLHITNTATTVLLDE  240
AEC15155.1      VMNTEHKAYLDKNKAYPVECWVPDPTRNENTRYFGTLTGGENVPPVLHITNTATTVLLDE  240
AEC15156.1      VMNTEHKAYLDKNKAYPVECWVPDPTRNENTRYFGTLTGGENVPPVLHITNTATTVLLDE  240
AEC15158.1      VMNTEHKAYLDKNKAYPVECWVPDPTRNENTRYFGTLTGGENVPPVLHITNTATTVLLDE  240
AEC15159.1      VMNTEHKAYLDKNKAYPVECWVPDPTRNENTRYFGTLTGGENVPPVLHITNTATTVLLDE  240
AEC15161.1      VMNTEHKAYLDKNKAYPVECWVPDPTRNENTRYFGTLTGGENVPPVLHITNTATTVLLDE  240
AEC15162.1      VMNTEHKAYLDKNKAYPVECWVPDPTRNENTRYFGTLTGGENVPPVLHITNTATTVLLDE  240
```

AEC15163.1	VMNTEHKAYLDKNKAYPVECWVPDPTRNENTRYFGTLTGGENVPPVLHITNTATTVLLDE	240
AEC15226.1	VMNTEHKAYLDKNKAYPVECWVPDPTRNENTRYFGTLTGGENVPPVLHITNTATTVLLDE	240
AEC15227.1	VMNTEHKAYLDKNKAYPVECWVPDPTRNENTRYFGTLTGGENVPPVLHITNTATTVLLDE	240
AEC15228.1	VMNTEHKAYLDKNKAYPVECWVPDPTRNENTRYFGTLTGGENVPPVLHITNTATTVLLDE	240
AEC15229.1	VMNTEHKAYLDKNKAYPVECWVPDPTRNENTRYFGTLTGGENVPPVLHITNTATTVLLDE	240
AEC15230.1	VMNTEHKAYLDKNKAYPVECWVPDPTRNENTRYFGTLTGGENVPPVLHITNTATTVLLDE	240
AEC15236.1	VMNTEHKAYLDKNKAYPVECWVPDPTRNENTRYFGTLTGGENVPPVLHITNTATTVLLDE	240
AEC15237.1	VMNTEHKAYLDKNKAYPVECWVPDPTRNENTRYFGTLTGGENVPPVLHITNTATTVLLDE	240
AEC15238.1	VMNTEHKAYLDKNKAYPVECWVPDPTRNENTRYFGTLTGGENVPPVLHITNTATTVLLDE	240
AEC15239.1	VMNTEHKAYLDKNKAYPVECWVPDPTRNENTRYFGTLTGGENVPPVLHITNTATTVLLDE	240
AEC15240.1	VMNTEHKAYLDKNKAYPVECWVPDPTRNENTRYFGTLTGGENVPPVLHITNTATTVLLDE	240
AEC15447.1	VMNTEHKAYLDKNKAYPVECWVPDPTRNENTRYFGTLTGGENVPPVLHITNTATTVLLDE	240
AEC15453.1	VMNTEHKAYLDKNKAYPVECWVPDPTRNENTRYFGTLTGGENVPPVLHITNTATTVLLDE	240
AEC15459.1	VMNTEHKAYLDKNKAYPVECWVPDPTRNENTRYFGTLTGGENVPPVLHITNTATTVLLDE	240
AEC15465.1	VMNTEHKAYLDKNKAYPVECWVPDPTRNENTRYFGTLTGGENVPPVLHITNTATTVLLDE	240
AEC15471.1	VMNTEHKAYLDKNKAYPVECWVPDPTRNENTRYFGTLTGGENVPPVLHITNTATTVLLDE	240
AEC15477.1	VMNTEHKAYLDKNKAYPVECWVPDPTRNENTRYFGTLTGGENVPPVLHITNTATTVLLDE	240
AEC15483.1	VMNTEHKAYLDKNKAYPVECWVPDPTRNENTRYFGTLTGGENVPPVLHITNTATTVLLDE	240
AEC15489.1	VMNTEHKAYLDKNKAYPVECWVPDPTRNENTRYFGTLTGGENVPPVLHITNTATTVLLDE	240
AEC15543.1	VMNTEHKAYLDKNKAYPVECWVPDPTRNENTRYFGTLTGGENVPPVLHITNTATTVLLDE	240
AEC15119.1	VMNTEHKAYLDKNKAYPVECWVPDPTRNENTRYFGTLTGGENVPPVLHITNTATTVLLDE	240
AEC15123.1	VMNTEHKAYLDKNKAYPVECWVPDPTRNENTRYFGTLTGGENVPPVLHITNTATTVLLDE	240
AEC15126.1	VMNTEHKAYLDKNKAYPVECWVPDPTRNENTRYFGTLTGGENVPPVLHITNTATTVLLDE	240
AEC15132.1	VMNTEHKAYLDKNKAYPVECWVPDPTRNENTRYFGTLTGGENVPPVLHITNTATTVLLDE	240
AEC15134.1	VMNTEHKAYLDKNKAYPVECWVPDPTRNENTRYFGTLTGGENVPPVLHITNTATTVLLDE	240
AEC15140.1	VMNTEHKAYLDKNKAYPVECWVPDPTRNENTRYFGTLTGGENVPPVLHITNTATTVLLDE	240
AEC15221.1	VMNTEHKAYLDKNKAYPVECWVPDPTRNENTRYFGTLTGGENVPPVLHITNTATTVLLDE	240
AEC15224.1	VMNTEHKAYLDKNKAYPVECWVPDPTRNENTRYFGTLTGGENVPPVLHITNTATTVLLDE	240
AEC15225.1	VMNTEHKAYLDKNKAYPVECWVPDPTRNENTRYFGTLTGGENVPPVLHITNTATTVLLDE	240
AEC15233.1	VMNTEHKAYLDKNKAYPVECWVPDPTRNENTRYFGTLTGGENVPPVLHITNTATTVLLDE	240
AEC15234.1	VMNTEHKAYLDKNKAYPVECWVPDPTRNENTRYFGTLTGGENVPPVLHITNTATTVLLDE	240
AEC15235.1	VMNTEHKAYLDKNKAYPVECWVPDPTRNENTRYFGTLTGGENVPPVLHITNTATTVLLDE	240
AEC15241.1	VMNTEHKAYLDKNKAYPVECWVPDPTRNENTRYFGTLTGGENVPPVLHITNTATTVLLDE	240
AEC15220.1	VMNTEHKAYLDKNKAYPVECWVPDPTRNENTRYFGTLTGGENVPPVLHITNTATTVLLDE	240
AEC15223.1	VMNTEHKAYLDKNKAYPVECWVPDPTRNENTRYFGTLTGGENVPPVLHITNTATTVLLDE	240
AEC15232.1	VMNTEHKAYLDKNKAYPVECWVPDPTRNENTRYFGTLTGGENVPPVLHITNTATTVLLDE	240
AEC15128.1	VMNTEHKAYLDKNKAYPVECWVPDPTRNENTRYFGTLTGGENVPPVLHITNTATTVLLDE	240
AEC15495.1	VMNTEHKAYLDKNKAYPVECWVPDPTRNENTRYFGTLTGGENVPPVLHITNTATTVLLDE	240
AEC15501.1	VMNTEHKAYLDKNKAYPVECWVPDPTRNENTRYFGTLTGGENVPPVLHITNTATTVLLDE	240
AEC15507.1	VMNTEHKAYLDKNKAYPVECWVPDPTRNENTRYFGTLTGGENVPPVLHITNTATTVLLDE	240
AEC15513.1	VMNTEHKAYLDKNKAYPVECWVPDPTRNENTRYFGTLTGGENVPPVLHITNTATTVLLDE	240
AEC15519.1	VMNTEHKAYLDKNKAYPVECWVPDPTRNENTRYFGTLTGGENVPPVLHITNTATTVLLDE	240
AEC15525.1	VMNTEHKAYLDKNKAYPVECWVPDPTRNENTRYFGTLTGGENVPPVLHITNTATTVLLDE	240
AEC15531.1	VMNTEHKAYLDKNKAYPVECWVPDPTRNENTRYFGTLTGGENVPPVLHITNTATTVLLDE	240
AEC15537.1	VMNTEHKAYLDKNKAYPVECWVPDPTRNENTRYFGTLTGGENVPPVLHITNTATTVLLDE	240
BAF40805.1	VMNTEHKAYLDKNKAYPVECWVPDPTRNENTRYFGTLTGGENVPPVLHITNTATTVLLDE	240
AAT09843.1	VMNTEHKAYLDKNKAYPVECWVPDPTRNENTRYFGTLTGGENVPPVLHITNTATTVLLDE	240
BAA01965.1	VMNTEHKAYLDKNKAYPVECWVPDPTRNENTRYFGTLTGGENVPPVLHITNTATTVLLDE	240
AIW00320.1	VMNTEHKAYLDKNKAYPVECWVPDPTRNENTRYFGTLTGGENVPPVLHITNTATTVLLDE	240
ALF06194.1	VMNTEHKAYLDKNKAYPVECWVPDPTRNENTRYFGTLTGGENVPPVLHITNTATTVLLDE	240
ALF06211.1	VMNTEHKAYLDKNKAYPVECWVPDPTRNENTRYFGTLTGGENVPPVLHITNTATTVLLDE	240
AEC14821.1	VMNTEHKAYLDKNKAYPVECWVPDPTRNENTRYFGTLTGGENVPPVLHITNTATTVLLDE	240
AEC14860.1	VMNTEHKAYLDKNKAYPVECWVPDPTRNENTXYFGTLTGGENVPPVLHITNTATTVLLDE	240
AEC14861.1	VMNTEHKAYLDKNKAYPVECWVPDPTRNENTRYFGTLTGGENVPPVLHITNTATTVLLDE	240
AEC14811.1	VMNTEHKAYLDKNKAYPVECWVPDPTRNENTRYFGTLTGGENVPPVLHITNTATTVLLDE	240
AEC14806.1	VMNTEHKAYLDKNKAYPVECWVPDPTRNENTRYFGTLAGGENVPPVLHITNTATTVLLDE	240
AEC14795.1	VMNTEHKAYLDKNKAYPVECWVPDPTRNENTRYFGTLTGGENVPPVLHITNTATTVLLDE	240
AEC14765.1	VMNTEHKAYLDKNKAYPVECWVPDPTRNENTRYFGTLTGGENVPPVLHITNTATTVLLDE	240
AEC14774.1	VMNTEHKAYLDKNKAYPVECWVPDPTRNENTRYFGTLTGGENVPPVLHITNTATTVLLDE	240
AEC14761.1	VMNTEHKAYLDKNKAYPVECWVPDPTRNENTRYFGTLTGGENVPPVLHITNTATTVLLDE	240
AEC14753.1	VMNTEHKAYLDKNKAYPVECWVPDPTRNENTRYFGTLTGGENVPPVLHITNTATTVLLDE	240
AEC14767.1	VMNTEHKAYLDKNKAYPVECWVPDPTRNENTRYFGTLTGGENVPPVLHITNTATTVLLDE	240
AEC14780.1	VMNTEHKAYLDKNKAYPVECWVPDPTRNENTRYFGTLTGGENVPPVLHITNTATTVLLDE	240
AEC14760.1	VMNTEHKAYLDKNKAYPVECWVPDPTRNENTRYFGTLTGGENVPPVLHITNTATTALLDE	240

AEC14762.1	VMNTEHKAYLDKNKAYPVECWVPDPTRNENTRYFGTLTGGENVPPVLHITNTATTVLLDE	240
AEC14742.1	VMNTEHKAYLDKNKAYPVECWVPDPTRNENTRYFGTLTGGENVPPVLHITNTATTVLLDE	240
AEC14763.1	VMNTEHKAYLDKNKAYPVECWVPDPTRNENTRYFGTLTGGENVPPVLHITNTATTVLLDE	240
AEC14810.1	VMNTEHKAYLDKNKAYPVECWVPDPTRNENTRYFGTLTGGENVPPVLHITNTATTVLLDE	240
AEC14803.1	VMNTEHKAYLDKNKAYPVECWVPDPTRNENTRYFGTLTGGENVPPVLHITNTATTVLLDE	240
AEC14802.1	VMNTEHKAYLDKNKAYPVECWVPDPTRNENTRYFGTLTGGENVPPVLHITNTATTVLLDE	240
AEC14812.1	VMNTEHKAYLDKNKAYPVECWVPDPTRNENTRYFGTLTGGENVPPVLHITNTATTVLLDE	240
AEC14804.1	VMNTEHKAYLDKNKAYPVECWVPDPTRNENTRYFGTLTGGENVPPVLHITNTATTVLLDE	240
AEC14797.1	VMNTEHKAYLDKNKAYPVECWVPDPTRNENTRYFGTLTGGENVPPVLHITNTATTVLLDE	240
AEC14796.1	VMNTEHKAYLDKNKAYPVECWVPDPTRNENTRYFGTLTGGENVPPVLHITNTAATVLLDE	240
AEC14793.1	VMNTEHKAYLDKNKAYPVECWVPDPTRNENTRYFGTLTGGENVPPVLHITNTATTVLLDE	240
AEC14791.1	VMNTEHKAYLDKNKAYPVECWVPDPTRNENTRYFGTLTGGENVPPVLHITNTATTVLLDE	240
AEC14794.1	VMNTEHKAYLDKNKAYPVECWVPDPTRNENTRYFGTLTGGENVPPVLHITNTATTVLLDE	240
AEC14789.1	VMNTEHKAYLDKNKAYPVECWVPDPTRNENTRYFGTLTGGENVPPVLHITNTATTVLLDE	240
AEC14743.1	VMNTEHKAYLDKNKAYPVECWVPDPTRNENTRYFGTLTGGENVPPVLHITNTATTVLLDE	240
AEC14745.1	VMNTEHKAYLDKNKAYPVECWVPDPTRNENTRYFGTLTGGENVPPVLHITNTATTVLLDE	240
AEC14750.1	VMNTEHKAYLDKNKAYPVECWVPDPTRNENTRYFGTLTGGENVPPVLHITNTATTVLLDE	240
AEC14754.1	VMNTEHKAYLDKNKAYPVECWVPDPTRNENTRYFGTLTGGENVPPVLHITNTATTVLLDE	240
AEC14751.1	VVNTEHKAYLDKNKAYPVECWVPDPTRNENTRYFGTLTGGENVPPVLHITNTATTVLLDE	240
AEC14776.1	VVNTEHKAYLDKNKAYPVECWVPDPTRNENTRYFGTLTGGENVPPVLHITNTATTVLLDE	240
AEC14755.1	VMNTEHKAYLDKNKAYPVECWVPDPTRNENTRYFGTLTGGENVPPVLHITNTATTVLLDE	240
AEC14759.1	VMNTEHKAYPDKNKAYPVECWVPDPTRNENTRYFGTLTGGENVPPVLHITNTATTVLLDE	240
AEC14766.1	VMNTEHKAYLDKNKAYPVECWVPDPTRNENTRYFGTLTGGENVPPVLHITNTATTVLLDE	240
AEC14781.1	VMNTEHKAYLDKNKAYPVECWVPDPTRNENTRYFGTLTGGENVPPVLHITNTATTVLLDE	240
AEC15186.1	VMNTEHKAYLDKNKAYPVECWVPDPTRNENTRYFGTLTGGENVPPVLHITNTATTVLLDE	240
AEC15192.1	VMNTEHKAYLDKNKAYPVECWVPDPTRNENTRYFGTLTGGENVPPVLHITNTATTVLLDE	240
AEC15194.1	VMNTEHKAYLDKNKAYPVECWVPDPTRNENTRYFGTLTGGENVPPVLHITNTATTVLLDE	240
AEC15195.1	VMNTEHKAYLDKNKAYPVECWVPDPTRNENTRYFGTLTGGENVPPVLHITNTATTVLLDD	240
QWT77104.1	VMNTEHKAYLDKNKAYPVECWVPDPTRNENTRYFGTLTGGENVPPVLHITNTATTVLLDE	240
UPN63088.1	VMNTEHKAYLDKNKAYPVECWVPDPTRNENTRYFGTLTGGENVPPVLHITNTATTVLLDE	240
UPN63104.1	VMNTEHRAYLDKNKAYPVECWVPDPTRNENTRYFGTLTGGENVPPVLHITNTATTVLLDE	240
UPN63112.1	VMNTEHKAYLDKNKAYPVECWVPDPTRNENTRYFGTLTGGENVPPVLHITNTATTVLLDE	240
UPN63113.1	VMNTEHKAYLDKNKAYPVECWVPDPTRNENTRYFGTLTGGENVPPVLHITNTATTVLLDE	240
UPN63114.1	VMNTEHKAYLDKNKAYPVECWVPDPTRNENTRYFGTLTGGENVPPVLHITNTATTVLLDE	240
UPN63115.1	VMNTEHKAYLDKNKAYPVECWVPDPTRNENTRYFGTLTGGENVPPVLHITNTATTVLLDE	240
AEC15172.1	VMNTEHKAYLDKNKAYPVECWVPDPTRNENTRYFGTLTGGENVPPVLHITNTATTVLLDE	240
BAE00117.1	VMNTEHKAYLDKNKAYPVECWVPDPTRNENTRYFGTLTGGENVPPVLHITNTATTVLLDE	240
BAE00123.1	VMNTEHKAYLDKNKAYPVECWVPDPTRNENTRYFGTLTGGENVPPVLHITNTATTVLLDE	240
AAC59331.1	VMNTEHKAYLDKNKAYPVECWVPDPTRNENTRYFGTLTGGESVPPVLHITNTATTVLLDE	240
WLD48106.1	VMNTEHKAYLDKNKAYPVECWVPDPTRNENTRYFGTLTGGENVPPVLHITNTATTVLLDE	240
UQK62661.1	VMNTEHKAYLDKNKAYPVECWVPDPTRNENTRYFGTLTGGENVPPVLHITNTATTVLLDE	240
QTJ15156.1	VMNTEHKAYLDKNKAYPVECWVPDPTRNENTRYFGTLTGGENVPPVLHITNTATTVLLDE	240
QNC68213.1	VMNTEHKAYLDKNKAYPVECWVPDPTRNENTRYFGTLTGGENVPPVLHITNTATTVLLDE	240
ASV51766.1	VMNTEHKAYLDKNKAYPVECWVPDPTRNENTRYFGTLTGGENVPPVLHITNTATTVLLDE	240
ASV51796.1	VMNTEHKAYLDKNKAYPVECWVPDPTRNENTRYFGTLTGGENVPPVLHITNTATTVLLDE	240
ASV51802.1	VMNTEHKAYLDKNKAYPVECWVPDPTRNENTRYFGTLTGGENVPPVLHITNTATTVLLDE	240
ASV51808.1	VMNTEHKAYLDKNKAYPVECWVPDPTRNENTRYFGTLTGGENVPPVLHITNTATTVLLDE	240
ASV51820.1	VMNTEHKAYLDKNKAYPVECWVPDPTRNENTRYFGTLTGGENVPPVLHITNTATTVLLDE	240
ASV51826.1	VMNTEHKAYLDKNKAYPVECWVPDPTRNENTRYFGTLTGGENVPPVLHITNTATTVLLDE	240
ASV51832.1	VMNTEHKAYLDKNKAYPVECWVPDPTRNENTRYFGTLTGGENVPPVLHITNTATTVLLDE	240
ASV51838.1	VMNTEHKAYLDKNKAYPVECWVPDPTRNENTRYFGTLTGGENVPPVLHITNTATTVLLDE	240
ASV51862.1	VMNTEHKAYLDKNKAYPVECWVPDPTRNENTRYFGTLTGGENVPPVLHITNTATTVLLDE	240
ASV51868.1	VMNTEHKAYLDKNKAYPVECWVPDPTRNENTRYFGTLTGGENVPPVLHITNTATTVLLDE	240
ASV51874.1	VMNTEHKAYLDKNKAYPVECWVPDPTRNENTRYFGTLTGGENVPPVLHITNTATTVLLDE	240
ASV51880.1	VMNTEHKAYLDKNKAYPVECWVPDPTRNENTRYFGTLTGGENVPPVLHITNTATTVLLDE	240
ASV51892.1	VMNTEHKAYLDKNKAYPVECWVPDPTRNENTRYFGTLTGGENVPPVLHITNTATTVLLDE	240
ANH21098.1	VMNTEHKAYLDKNKAYPVECWVPDPTRNENTRYFGTLTGGENVPPVLHITNTATTVLLDE	240
AID67150.1	VMNTEHKAYLDKNKAYPVECWVPDPTRNENTRYFGTLTGGENVPPVLHITNTATTVLLDE	240
AID67156.1	VMNTEHKAYLDKNKAYPVECWVPDPTRNENTRYFGTLTGGENVPPVLHITNTATTVLLDE	240
AID67162.1	VMNTEHKAYLDKNKAYPVECWVPDPTRNENTRYFGTLTGGENVPPVLHITNTATTVLLDE	240
AID67168.1	VMNTEHKAYLDKNKAYPVECWVPDPTRNENTRYFGTLTGGENVPPVLHITNTATTVLLDE	240
CCW72479.1	VMNTEHKAYLDKNKAYPVECWVPDPTRNENTRYFGTLTGGENVPPVLHITNTATTVLLDE	240
CCW72486.1	VMNTEHKAYLDKNKAYPVECWVPDPTRNENTRYFGTLTGGENVPPVLHITNTATTVLLDE	240
CCW72494.1	VMNTEHKAYLDKNKAYPVECWVPDPTRNENTRYFGTLTGGENVPPVLHITNTATTVLLDE	240

CCW72514.1	VMNTEHKAYLDKNKAYPVECWVPDPTRNENTRYFGTLTGGENVPPVLHITNTATTVLLDE	240
CCW72518.1	VMNTEHKAYLDKNKAYPVECWVPDPTRNENTRYFGTLTGGENVPPVLHITNTATTVLLDE	240
CCW72522.1	VMNTEHKAYLDKNKAYPVECWVPDPTRNENTRYFGTLTGGENVPPVLHITNTATTVLLDE	240
AFH57194.1	VMNTEHKAYLDKNKAYPVECWVPDPTRNENTRYFGTLTGGENVPPVLHITNTATTVLLDE	240
AEC15047.1	VMNTEHKAYLDKNKAYPVECWVPDPTRNENTRYFGTLTGGENVPPVLHITNTATTVLLDE	240
AEC15048.1	VMNTEHKAYLDKNKAYPVECWVPDPTRNENTRYFGTLTGGENVPPVLHITNTATTVLLDE	240
AEC15049.1	VMNTEHKAYLDKNKAYPVECWVPDPTRNENTRYFGTLTGGENVPPVLHITNTATTVLLDE	240
AEC15050.1	VMNTEHKAYLDKNKAYPVECWVPDPTRNENTRYFGTLTGGENVPPVLHITNTATTVLLDE	240
AEC15051.1	VMNTEHKAYLDKNKAYPVECWVPDPTRNENTRYFGTLTGGENVPPVLHITNTATTVLLDE	240
AEC15052.1	VMNTEHKAYLDKNKAYPVECWVPDPTRNENTRYFGTLTGGENVPPVLHITNTATTVLLDE	240
AEC15053.1	VMNTEHKAYLDKNKAYPVECWVPDPTRNENTRYFGTLTGGENVPPVLHITNTATTVLLDE	240
AEC15054.1	VMNTEHKAYLDKNKAYPVECWVPDPTRNENTRYFGTLTGGENVPPVLHITNTATTVLLDE	240
AEC15055.1	VMNTEHKAYLDKNKAYPVECWVPDPTRNENTRYFGTLTGGENVPPVLHITNTATTVLLDE	240
AEC15056.1	VMNTEHKAYLDKNKAYPVECWVPDPTRNENTRYFGTLTGGENVPPVLHITNTATTVLLDE	240
AEC15057.1	VMNTEHKAYLDKNKAYPVECWVPDPTRNENTRYFGTLTGGENVPPVLHITNTATTVLLDE	240
AEC15058.1	VMNTEHKAYLDKNKAYPVECWVPDPTRNENTRYFGTLTGGENVPPVLHITNTATTVLLDE	240
AEC15059.1	VMNTEHKAYLDKNKAYPVECWVPDPTRNENTRYFGTLTGGENVPPVLHITNTATTVLLDE	240
AEC15060.1	VMNTEHKAYLDKNKAYPVECWVPDPTRNENTRYFGTLTGGENVPPVLHITNTATTVLLDE	240
AEC15061.1	VMNTEHKAYLDKNKAYPVECWVPDPTRNENTRYFGTLTGGENVPPVLHITNTATTVLLDE	240
AEC15062.1	VMNTEHKAYLDKNKAYPVECWVPDPTRNENTRYFGTLTGGENVPPVLHITNTATTVLLDE	240
AEC15063.1	VMNTEHKAYLDKNKAYPVECWVPDPTRNENTRYFGTLTGGENVPPVLHITNTATTVLLDE	240
AEC15064.1	VMNTEHKAYLDKNKAYPVECWVPDPTRNENTRYFGTLTGGENVPPVLHITNTATTVLLDE	240
AEC15065.1	VMNTEHKAYLDKNKAYPVECWVPDPTRNENTRYFGTLTGGENVPPVLHITNTATTVLLDE	240
AEC15066.1	VMNTEHKAYLDKNKAYPVECWVPDPTRNENTRYFGTLTGGENVPPVLHITNTATTVLLDE	240
AEC15067.1	VMNTEHKAYLDKNKAYPVECWVPDPTRNENTRYFGTLTGGENVPPVLHITNTATTVLLDE	240
AEC15068.1	VMNTEHKAYLDKNKAYPVECWVPDPTRNENTRYFGTLTGGENVPPVLHITNTATTVLLDE	240
AEC15315.1	VMNTEHKAYLDKNKAYPVECWVPDPTRNENTRYFGTLTGGENVPPVLHITNTATTVLLDE	240
AEC15321.1	VMNTEHKAYLDKNKAYPVECWVPDPTRNENTRYFGTLTGGENVPPVLHITNTATTVLLDE	240
BAD21271.1	VMNTEHKAYLDKNKAYPVECWVPDPTRNENTRYFGTLTGGENVPPVLHITNTATTVLLDE	240
BAD21283.2	VMNTEHKAYLDKNKAYPVECWVPDPTRNENTRYFGTLTGGENVPPVLHITNTATTVLLDE	240
BAC15683.1	VMNTEHKAYLDKNKAYPVECWVPDPTRNENTRYFGTLTGGENVPPVLHITNTATTVLLDE	240
BAC15719.1	VMNTEHKAYLDKNKAYPVECWVPDPTRNENTRYFGTLTGGENVPPVLHITNTATTVLLDE	240
BAB93038.1	VMNTEHKAYLDKNKAYPVECWVPDPTRNENTRYFGTLTGGENVPPVLHITNTATTVLLDE	240
BAB93056.1	VMNTEHKAYLDKNKAYPVECWVPDPTRNENTRYFGTLTGGENVPPVLHITNTATTVLLDE	240
BAB93062.1	VMNTEHKAYLDKNKAYPVECWVPDPTRNENTRYFGTLTGGENVPPVLHITNTATTVLLDE	240
BAB68911.1	VMNTEHKAYLDKNKAYPVECWVPDPTRNENTRYFGTLTGGENVPPVLHITNTATTVLLDE	240
BAB68947.1	VMNTEHKAYLDKNKAYPVECWVPDPTRNENTRYFGTLTGGENVPPVLHITNTATTVLLDE	240
AAK70234.1	VMNTEHKAYLDKNKAYPVECWVPDPTRNENTRYFGTLTGGENVPPVLHITNTATTVLLDE	240
AAK70246.1	VMNTEHKAYLDKNKAYPVECWVPDPTRNENTRYFGTLTGGENVPPVLHITNTATTVLLDE	240
BAA01959.1	VMNTEHKAYLDKNKAYPVECWVPDPTRNENTRYFGTLTGGENVPPVLHITNTATTVLLDE	240
BAA01960.1	VMNTEHKAYLDKNKAYPVECWVPDPTRNENTRYFGTLTGGENVPPVLHITNTATTVLLDE	240
QWT77128.1	VMNTEHKAYLDKNKAYPVECWVPDPTRNENTRYFGTLTGGENVPPVLHITNTATTVLLDE	240
UPN63089.1	VMNTEHKAYLDKNKAYPVECWVPDPTRNENTRYFGTLTGGENVPPVLHITNTATTVLLDE	240
UPN63093.1	VMNTEHKAYLDKNKAYPVECWVPDPTRNENTRYFGTLTGGENVPPVLHITNTATTVLLDE	240
UPN63103.1	VMNTEHKAYLDKNKAYPVECWVPDPTRNENTRYFGTLTGGENVPPVLHITNTATTVLLDE	240
UPN63105.1	VMNTEHKAYLDKNKAYPVECWVPDPTRNENTRYFGTLTGGENVPPVLHITNTATTVLLDE	240
UPN63106.1	VMNTEHKAYLDKNKAYPVECWVPDPTRNENTRYFGTLTGGENVPPVLHITNTATTVLLDE	240
UPN63110.1	VMNTEHKAYLDKNKAYPVECWVPDPTRNENTRYFGTLTGGENVPPVLHITNTATTVLLDE	240
UPN63111.1	VMNTEHKAYLDKNKAYPVECWVPDPTRNENTRYFGTLTGGENVPPVLHITNTATTVLLDE	240
AEC15030.1	VMNTEHKAYLDKNKAYPVECWVPDPTRNENTRYFGTLTGGENVPPVLHITNTATTVLLDE	240
AEC15031.1	VMNTEHKAYLDKNKAYPVECWVPDPTRNENTRYFGTLTGGENVPPVLHITNTATTVLLDE	240
AEC15032.1	VMNTEHKAYLDKNKAYPVECWVPDPTRNENTRYFGTLTGGENVPPVLHITNTATTVLLDE	240
AEC15033.1	VMNTEHKAYLDKNKAYPVECWVPDPTRNENTRYFGTLTGGENVPPVLHITNTATTVLLDE	240
AEC15034.1	VMNTEHKAYLDKNKAYPVECWVPDPTRNENTRYFGTLTGGENVPPVLHITNTATTVLLDE	240
AEC15035.1	VMNTEHKAYLDKNKAYPVECWVPDPTRNENTRYFGTLTGGENVPPVLHITNTATTVLLDE	240
AEC15036.1	VMNTEHKAYLDKNKAYPVECWVPDPTRNENTRYFGTLTGGENVPPVLHITNTATTVLLDE	240
AEC15038.1	VMNTEHKAYLDKNKAYPVECWVPDPTRNENTRYFGTLTGGENVPPVLHITNTATTVLLDE	240
AEC15039.1	VMNTEHKAYLDKNKAYPVECWVPDPTRNENTRYFGTLTGGENVPPVLHITNTATTVLLDE	240
AEC15040.1	VMNTEHKAYLDKNKAYPVECWVPDPTRNENTRYFGTLTGGENVPPVLHITNTATTVLLDE	240
AEC15041.1	VMNTEHKAYLDKNKAYPVECWVPDPTRNENTRYFGTLTGGENVPPVLHITNTATTVLLDE	240
AEC15042.1	VMNTEHKAYLDKNKAYPVECWVPDPTRNENTRYFGTLTGGENVPPVLHITNTATTVLLDE	240
AEC15043.1	VMNTEHKAYLDKNKAYPVECWVPDPTRNENTRYFGTLTGGENVPPVLHITNTATTVLLDE	240
AEC15044.1	VMNTEHKAYLDKNKAYPVECWVPDPTRNENTRYFGTLTGGENVPPVLHITNTATTVLLDE	240
AEC15045.1	VMNTEHKAYLDKNKAYPVECWVPDPTRNENTRYFGTLTGGENVPPVLHITNTATTVLLDE	240

AEC15046.1	VMNTEHKAYLDKNKAYPVECWVPDPTRNENTRYFGTLTGGENVPPVLHITNTATTVLLDE	240
UPN63083.1	VMNTEHKAYLDKNKAYPVECWVPDPTRNENTRYFGTLTGGENVPPVLHITNTATTVLLDE	240
BAE00111.1	VMNTEHKAYLDKNKAYPVECWVPDPTRNENTRYFGTLTGGENVPPVLHITNTATTVLLDE	240
BAA01968.1	VMNTEHKAYLDKNKAYPVECWVPDPTRNENTRYFGTLTGGENVPPVLHITNTATTVLLDE	240
CCW72491.1	VMNTEHKAYLDKNKAYPVECWVPDPTRNENTRYFGTLTGGENVPPVLHITNTATTVLLDE	240
AEC14788.1	VMNTEHKAYLDKNKAYPVECWVPDPTRNENTRYFGTLTGGENVPPVLHITNTATTVLLDE	240
AEC14790.1	VMNTEHKAYLDKNKAYPVECWVPDPTRNENTRYFGTLTGGENVPPVLHITNTATTVLLDE	240
AEC14792.1	VMNTEHKAYLDKNKAYPVECWVPDPTRNENTRYFGTLTGGENVPPVLHITNTATTVLLDE	240
AEC14798.1	VMNTEHKAYLDKNKAYPVECWVPDPTRNENTRYFGTLTGGENVPPVLHITNTATTVLLDE	240
AEC14799.1	VMNTEHKAYLDKNKAYPVECWVPDPTRNENTRYFGTLTGGENVPPVLHITNTATTVLLDE	240
AEC14800.1	VMNTEHKAYLDKNKAYPVECWVPDPTRNENTRYFGTLTGGENVPPVLHITNTATTVLLDE	240
AEC14801.1	VMNTEHKAYLDKNKAYPVECWVPDPTRNENTRYFGTLTGGENVPPVLHITNTATTVLLDE	240
AEC14805.1	VMNTEHKAYLDKNKAYPVECWVPDPTRNENTRYFGTLTGGENVPPVLHITNTATTVLLDE	240
AEC14807.1	VMNTEHKAYLDKNKAYPVECWVPDPTRNENTRYFGTLTGGENVPPVLHITNTATTVLLDE	240
AEC14808.1	VMNTEHKAYLDKNKAYPVECWVPDPTRNENTRYFGTLTGGENVPPVLHITNTATTVLLDE	240
AEC14809.1	VMNTEHKAYLDKNKAYPVECWVPDPTRNENTRYFGTLTGGENVPPVLHITNTATTVLLDE	240
AEC14813.1	VMNTEHKAYLDKNKAYPVECWVPDPTRNENTRYFGTLTGGENVPPVLHITNTATTVLLDE	240
AEC14912.1	VMNTEHKAYLDKNKAYPVECWVPDPTRNENTRYFGTLTGGENVPPVLHITNTATTVLLDE	240
AEC14913.1	VMNTEHKAYLDKNKAYPVECWVPDPTRNENTRYFGTLTGGENVPPVLHITNTATTVLLDE	240
AEC14914.1	VMNTEHKAYLDKNKAYPVECWVPDPTRNENTRYFGTLTGGENVPPVLHITNTATTVLLDE	240
AEC14915.1	VMNTEHKAYLDKNKAYPVECWVPDPTRNENTRYFGTLTGGENVPPVLHITNTATTVLLDE	240
AEC14916.1	VMNTEHKAYLDKNKAYPVECWVPDPTRNENTRYFGTLTGGENVPPVLHITNTATTVLLDE	240
AEC14917.1	VMNTEHKAYLDKNKAYPVECWVPDPTRNENTRYFGTLTGGENVPPVLHITNTATTVLLDE	240
AEC14918.1	VMNTEHKAYLDKNKAYPVECWVPDPTRNENTRYFGTLTGGENVPPVLHITNTATTVLLDE	240
AEC14919.1	VMNTEHKAYLDKNKAYPVECWVPDPTRNENTRYFGTLTGGENVPPVLHITNTATTVLLDE	240
AEC14920.1	VMNTEHKAYLDKNKAYPVECWVPDPTRNENTRYFGTLTGGENVPPVLHITNTATTVLLDE	240
AEC14921.1	VMNTEHKAYLDKNKAYPVECWVPDPTRNENTRYFGTLTGGENVPPVLHITNTATTVLLDE	240
AEC14922.1	VMNTEHKAYLDKNKAYPVECWVPDPTRNENTRYFGTLTGGENVPPVLHITNTATTVLLDE	240
AEC14923.1	VMNTEHKAYLDKNKAYPVECWVPDPTRNENTRYFGTLTGGENVPPVLHITNTATTVLLDE	240
AEC14924.1	VMNTEHKAYLDKNKAYPVECWVPDPTRNENTRYFGTLTGGENVPPVLHITNTATTVLLDE	240
AEC14925.1	VMNTEHKAYLDKNKAYPVECWVPDPTRNENTRYFGTLTGGENVPPVLHITNTATTVLLDE	240
AEC14926.1	VMNTEHKAYLDKNKAYPVECWVPDPTRNENTRYFGTLTGGENVPPVLHITNTATTVLLDE	240
AEC14927.1	VMNTEHKAYLDKNKAYPVECWVPDPTRNENTRYFGTLTGGENVPPVLHITNTATTVLLDE	240
AEC14928.1	VMNTEHKAYLDKNKAYPVECWVPDPTRNENTRYFGTLTGGENVPPVLHITNTATTVLLDE	240
AEC14929.1	VMNTEHKAYLDKNKAYPVECWVPDPTRNENTRYFGTLTGGENVPPVLHITNTATTVLLDE	240
AEC14930.1	VMNTEHKAYLDKNKAYPVECWVPDPTRNENTRYFGTLTGGENVPPVLHITNTATTVLLDE	240
AEC14931.1	VMNTEHKAYLDKNKAYPVECWVPDPTRNENTRYFGTLTGGENVPPVLHITNTATTVLLDE	240
AEC14932.1	VMNTEHKAYLDKNKAYPVECWVPDPTRNENTRYFGTLTGGENVPPVLHITNTATTVLLDE	240
AEC14933.1	VMNTEHKAYLDKNKAYPVECWVPDPTRNENTRYFGTLTGGENVPPVLHITNTATTVLLDE	240
AEC14934.1	VMNTEHKAYLDKNKAYPVECWVPDPTRNENTRYFGTLTGGENVPPVLHITNTATTVLLDE	240
AEC14935.1	VMNTEHKAYLDKNKAYPVECWVPDPTRNENTRYFGTLTGGENVPPVLHITNTATTVLLDE	240
AEC14936.1	VMNTEHKAYLDKNKAYPVECWVPDPTRNENTRYFGTLTGGENVPPVLHITNTATTVLLDE	240
AEC14937.1	VMNTEHKAYLDKNKAYPVECWVPDPTRNENTRYFGTLTGGENVPPVLHITNTATTVLLDE	240
AEC14938.1	VMNTEHKAYLDKNKAYPVECWVPDPTRNENTRYFGTLTGGENVPPVLHITNTATTVLLDE	240
AEC14939.1	VMNTEHKAYLDKNKAYPVECWVPDPTRNENTRYFGTLTGGENVPPVLHITNTATTVLLDE	240
AEC14940.1	VMNTEHKAYLDKNKAYPVECWVPDPTRNENTRYFGTLTGGENVPPVLHITNTATTVLLDE	240
AEC14941.1	VMNTEHKAYLDKNKAYPVECWVPDPTRNENTRYFGTLTGGENVPPVLHITNTATTVLLDE	240
AEC14942.1	VMNTEHKAYLDKNKAYPVECWVPDPTRNENTRYFGTLTGGENVPPVLHITNTATTVLLDE	240
AEC14943.1	VMNTEHKAYLDKNKAYPVECWVPDPTRNENTRYFGTLTGGENVPPVLHITNTATTVLLDE	240
AEC14944.1	VMNTEHKAYLDKNKAYPVECWVPDPTRNENTRYFGTLTGGENVPPVLHITNTATTVLLDE	240
AEC14945.1	VMNTEHKAYLDKNKAYPVECWVPDPTRNENTRYFGTLTGGENVPPVLHITNTATTVLLDE	240
AEC14946.1	VMNTEHKAYLDKNKAYPVECWVPDPTRNENTRYFGTLTGGENVPPVLHITNTATTVLLDE	240
AEC14947.1	VMNTEHKAYLDKNKAYPVECWVPDPTRNENTRYFGTLTGGENVPPVLHITNTATTVLLDE	240
AEC14948.1	VMNTEHKAYLDKNKAYPVECWVPDPTRNENTRYFGTLTGGENVPPVLHITNTATTVLLDE	240
AEC14949.1	VMNTEHKAYLDKNKAYPVECWVPDPTRNENTRYFGTLTGGENVPPVLHITNTATTVLLDE	240
AEC14950.1	VMNTEHKAYLDKNKAYPVECWVPDPTRNENTRYFGTLTGGENVPPVLHITNTATTVLLDE	240
AEC14951.1	VMNTEHKAYLDKNKAYPVECWVPDPTRNENTRYFGTLTGGENVPPVLHITNTATTVLLDE	240
AEC14952.1	VMNTEHKAYLDKNKAYPVECWVPDPTRNENTRYFGTLTGGENVPPVLHITNTATTVLLDE	240
AEC14953.1	VMNTEHKAYLDKNKAYPVECWVPDPTRNENTRYFGTLTGGENVPPVLHITNTATTVLLDE	240
AEC14954.1	VMNTEHKAYLDKNKAYPVECWVPDPTRNENTRYFGTLTGGENVPPVLHITNTATTVLLDE	240
AEC14955.1	VMNTEHKAYLDKNKAYPVECWVPDPTRNENTRYFGTLTGGENVPPVLHITNTATTVLLDE	240
AEC14956.1	VMNTEHKAYLDKNKAYPVECWVPDPTRNENTRYFGTLTGGENVPPVLHITNTATTVLLDE	240
AEC14957.1	VMNTEHKAYLDKNKAYPVECWVPDPTRNENTRYFGTLTGGENVPPVLHITNTATTVLLDE	240
AEC14958.1	VMNTEHKAYLDKNKAYPVECWVPDPTRNENTRYFGTLTGGENVPPVLHITNTATTVLLDE	240

AEC14959.1	VMNTEHKAYLDKNKAYPVECWVPDPTRNENTRYFGTLTGGENVPPVLHITNTATTVLLDE	240
AEC14960.1	VMNTEHKAYLDKNKAYPVECWVPDPTRNENTRYFGTLTGGENVPPVLHITNTATTVLLDE	240
AEC14961.1	VMNTEHKAYLDKNKAYPVECWVPDPTRNENTRYFGTLTGGENVPPVLHITNTATTVLLDE	240
QWT77122.1	VMNTEHKAYLDKNKAYPVECWVPDPTRNENTRYFGTLTGGENVPPVLHITNTATTVLLDE	240
UPN63084.1	VMNTEHKAYLDKNKAYPVECWVPDPTRNENTRYFGTLTGGENVPPVLHITNTATTVLLDE	240
UPN63101.1	VMNTEHKAYLDKNKAYPVECWVPDPTRNENTRYFGTLTGGENVPPVLHITNTATTVLLDE	240
ASV51886.1	VMNTEHKAYLDKNKAYPVECWVPDPTRNENTRYFGTLTGGENVPPVLHITNTATTVLLDE	240
CCW72484.1	VMNTEHKAYLDKNKAYPVECWVPDPTRNENTRYFGTLTGGENVPPVLHITNTATTVLLDE	240
ANH21099.1	VMNTEHKAYLDKNKAYPVECWVPDPTRNENTRYFGTLTGGENVPPVLHITNTATTVLLDE	240
AEC15112.1	VMNTEHKAYLDKNKAYPVECWVPDPTRNENTRYFGTLTGGENVPPVLHITNTATTVLLDE	240
AEC15113.1	VMNTEHKAYLDKNKAYPVECWVPDPTRNENTRYFGTLTGGENVPPVLHITNTATTVLLDE	240
AEC15114.1	VMNTEHKAYLDKNKAYPVECWVPDPTRNENTRYFGTLTGGENVPPVLHITNTATTVLLDE	240
AEC15115.1	VMNTEHKAYLDKNKAYPVECWVPDPTRNENTRYFGTLTGGENVPPVLHITNTATTVLLDE	240
AEC15116.1	VMNTEHKAYLDKNKAYPVECWVPDPTRNENTRYFGTLTGGENVPPVLHITNTATTVLLDE	240
AEC15117.1	VMNTEHKAYLDKNKAYPVECWVPDPTRNENTRYFGTLTGGENVPPVLHITNTATTVLLDE	240
AEC15819.1	VMNTEHKAYLDKNKAYPVECWVPDPTRNENTRYFGTLTGGENVPPVLHITNTATTVLLDE	240
AEC15825.1	VMNTEHKAYLDKNKAYPVECWVPDPTRNENTRYFGTLTGGENVPPVLHITNTATTVLLDE	240
AEC15831.1	VMNTEHKAYLDKNKAYPVECWVPDPTRNENTRYFGTLTGGENVPPVLHITNTATTVLLDE	240
AEC15837.1	VMNTEHKAYLDKNKAYPVECWVPDPTRNENTRYFGTLTGGENVPPVLHITNTATTVLLDE	240
AEC15843.1	VMNTEHKAYLDKNKAYPVECWVPDPTRNENTRYFGTLTGGENVPPVLHITNTATTVLLDE	240
AEC15849.1	VMNTEHKAYLDKNKAYPVECWVPDPTRNENTRYFGTLTGGENVPPVLHITNTATTVLLDE	240
AEC15855.1	VMNTEHKAYLDKNKAYPVECWVPDPTRNENTRYFGTLTGGENVPPVLHITNTATTVLLDE	240
CCW72511.1	VMNTEHKAYLDKNKAYPVECWVPDPTRNENTRYFGTLTGGENVPPVLHITNTATTVLLDE	240
CCW72525.1	VMNTEHKAYLDKNKAYPVECWVPDPTRNENTRYFGTLTGGENVPPVLHITNTATTVLLDE	240
AEC14744.1	VMNTEHKAYLDKNKAYPVECWVPDPTRNENTRYFGTLTGGENVPPVLHITNTATTVLLDE	240
AEC14746.1	VMNTEHKAYLDKNKAYPVECWVPDPTRNENTRYFGTLTGGENVPPVLHITNTATTVLLDE	240
AEC14747.1	VMNTEHKAYLDKNKAYPVECWVPDPTRNENTRYFGTLTGGENVPPVLHITNTATTVLLDE	240
AEC14748.1	VMNTEHKAYLDKNKAYPVECWVPDPTRNENTRYFGTLTGGENVPPVLHITNTATTVLLDE	240
AEC14752.1	VMNTEHKAYLDKNKAYPVECWVPDPTRNENTRYFGTLTGGENVPPVLHITNTATTVLLDE	240
AEC14756.1	VMNTEHKAYLDKNKAYPVECWVPDPTRNENTRYFGTLTGGENVPPVLHITNTATTVLLDE	240
AEC14757.1	VMNTEHKAYLDKNKAYPVECWVPDPTRNENTRYFGTLTGGENVPPVLHITNTATTVLLDE	240
AEC14758.1	VMNTEHKAYLDKNKAYPVECWVPDPTRNENTRYFGTLTGGENVPPVLHITNTATTVLLDE	240
AEC14764.1	VMNTEHKAYLDKNKAYPVECWVPDPTRNENTRYFGTLTGGENVPPVLHITNTATTVLLDE	240
AEC14768.1	VMNTEHKAYLDKNKAYPVECWVPDPTRNENTRYFGTLTGGENVPPVLHITNTATTVLLDE	240
AEC14769.1	VMNTEHKAYLDKNKAYPVECWVPDPTRNENTRYFGTLTGGENVPPVLHITNTATTVLLDE	240
AEC14770.1	VMNTEHKAYLDKNKAYPVECWVPDPTRNENTRYFGTLTGGENVPPVLHITNTATTVLLDE	240
AEC14771.1	VMNTEHKAYLDKNKAYPVECWVPDPTRNENTRYFGTLTGGENVPPVLHITNTATTVLLDE	240
AEC14772.1	VMNTEHKAYLDKNKAYPVECWVPDPTRNENTRYFGTLTGGENVPPVLHITNTATTVLLDE	240
AEC14773.1	VMNTEHKAYLDKNKAYPVECWVPDPTRNENTRYFGTLTGGENVPPVLHITNTATTVLLDE	240
AEC14775.1	VMNTEHKAYLDKNKAYPVECWVPDPTRNENTRYFGTLTGGENVPPVLHITNTATTVLLDE	240
AEC14777.1	VMNTEHKAYLDKNKAYPVECWVPDPTRNENTRYFGTLTGGENVPPVLHITNTATTVLLDE	240
AEC14778.1	VMNTEHKAYLDKNKAYPVECWVPDPTRNENTRYFGTLTGGENVPPVLHITNTATTVLLDE	240
AEC14779.1	VMNTEHKAYLDKNKAYPVECWVPDPTRNENTRYFGTLTGGENVPPVLHITNTATTVLLDE	240
AEC14782.1	VMNTEHKAYLDKNKAYPVECWVPDPTRNENTRYFGTLTGGENVPPVLHITNTATTVLLDE	240
AEC14814.1	VMNTEHKAYLDKNKAYPVECWVPDPTRNENTRYFGTLTGGENVPPVLHITNTATTVLLDE	240
AEC14815.1	VMNTEHKAYLDKNKAYPVECWVPDPTRNENTRYFGTLTGGENVPPVLHITNTATTVLLDE	240
AEC14816.1	VMNTEHKAYLDKNKAYPVECWVPDPTRNENTRYFGTLTGGENVPPVLHITNTATTVLLDE	240
AEC14817.1	VMNTEHKAYLDKNKAYPVECWVPDPTRNENTRYFGTLTGGENVPPVLHITNTATTVLLDE	240
AEC14818.1	VMNTEHKAYLDKNKAYPVECWVPDPTRNENTRYFGTLTGGENVPPVLHITNTATTVLLDE	240
AEC14819.1	VMNTEHKAYLDKNKAYPVECWVPDPTRNENTRYFGTLTGGENVPPVLHITNTATTVLLDE	240
AEC14820.1	VMNTEHKAYLDKNKAYPVECWVPDPTRNENTRYFGTLTGGENVPPVLHITNTATTVLLDE	240
AEC14822.1	VMNTEHKAYLDKNKAYPVECWVPDPTRNENTRYFGTLTGGENVPPVLHITNTATTVLLDE	240
AEC14823.1	VMNTEHKAYLDKNKAYPVECWVPDPTRNENTRYFGTLTGGENVPPVLHITNTATTVLLDE	240
AEC14824.1	VMNTEHKAYLDKNKAYPVECWVPDPTRNENTRYFGTLTGGENVPPVLHITNTATTVLLDE	240
AEC14825.1	VMNTEHKAYLDKNKAYPVECWVPDPTRNENTRYFGTLTGGENVPPVLHITNTATTVLLDE	240
AEC14826.1	VMNTEHKAYLDKNKAYPVECWVPDPTRNENTRYFGTLTGGENVPPVLHITNTATTVLLDE	240
AEC14827.1	VMNTEHKAYLDKNKAYPVECWVPDPTRNENTRYFGTLTGGENVPPVLHITNTATTVLLDE	240
AEC14828.1	VMNTEHKAYLDKNKAYPVECWVPDPTRNENTRYFGTLTGGENVPPVLHITNTATTVLLDE	240
AEC14829.1	VMNTEHKAYLDKNKAYPVECWVPDPTRNENTRYFGTLTGGENVPPVLHITNTATTVLLDE	240
AEC14830.1	VMNTEHKAYLDKNKAYPVECWVPDPTRNENTRYFGTLTGGENVPPVLHITNTATTVLLDE	240
AEC14831.1	VMNTEHKAYLDKNKAYPVECWVPDPTRNENTRYFGTLTGGENVPPVLHITNTATTVLLDE	240
AEC14832.1	VMNTEHKAYLDKNKAYPVECWVPDPTRNENTRYFGTLTGGENVPPVLHITNTATTVLLDE	240
AEC14833.1	VMNTEHKAYLDKNKAYPVECWVPDPTRNENTRYFGTLTGGENVPPVLHITNTATTVLLDE	240
AEC14834.1	VMNTEHKAYLDKNKAYPVECWVPDPTRNENTRYFGTLTGGENVPPVLHITNTATTVLLDE	240

```
AEC14835.1        VMNTEHKAYLDKNKAYPVECWVPDPTRNENTRYFGTLTGGENVPPVLHITNTATTVLLDE  240
AEC14836.1        VMNTEHKAYLDKNKAYPVECWVPDPTRNENTRYFGTLTGGENVPPVLHITNTATTVLLDE  240
AEC14837.1        VMNTEHKAYLDKNKAYPVECWVPDPTRNENTRYFGTLTGGENVPPVLHITNTATTVLLDE  240
AEC14838.1        VMNTEHKAYLDKNKAYPVECWVPDPTRNENTRYFGTLTGGENVPPVLHITNTATTVLLDE  240
AEC14839.1        VMNTEHKAYLDKNKAYPVECWVPDPTRNENTRYFGTLTGGENVPPVLHITNTATTVLLDE  240
AEC14840.1        VMNTEHKAYLDKNKAYPVECWVPDPTRNENTRYFGTLTGGENVPPVLHITNTATTVLLDE  240
AEC14841.1        VMNTEHKAYLDKNKAYPVECWVPDPTRNENTRYFGTLTGGENVPPVLHITNTATTVLLDE  240
AEC14842.1        VMNTEHKAYLDKNKAYPVECWVPDPTRNENTRYFGTLTGGENVPPVLHITNTATTVLLDE  240
AEC14843.1        VMNTEHKAYLDKNKAYPVECWVPDPTRNENTRYFGTLTGGENVPPVLHITNTATTVLLDE  240
AEC14844.1        VMNTEHKAYLDKNKAYPVECWVPDPTRNENTRYFGTLTGGENVPPVLHITNTATTVLLDE  240
AEC14845.1        VMNTEHKAYLDKNKAYPVECWVPDPTRNENTRYFGTLTGGENVPPVLHITNTATTVLLDE  240
AEC14846.1        VMNTEHKAYLDKNKAYPVECWVPDPTRNENTRYFGTLTGGENVPPVLHITNTATTVLLDE  240
AEC14847.1        VMNTEHKAYLDKNKAYPVECWVPDPTRNENTRYFGTLTGGENVPPVLHITNTATTVLLDE  240
AEC14848.1        VMNTEHKAYLDKNKAYPVECWVPDPTRNENTRYFGTLTGGENVPPVLHITNTATTVLLDE  240
AEC14849.1        VMNTEHKAYLDKNKAYPVECWVPDPTRNENTRYFGTLTGGENVPPVLHITNTATTVLLDE  240
AEC14850.1        VMNTEHKAYLDKNKAYPVECWVPDPTRNENTRYFGTLTGGENVPPVLHITNTATTVLLDE  240
AEC14851.1        VMNTEHKAYLDKNKAYPVECWVPDPTRNENTRYFGTLTGGENVPPVLHITNTATTVLLDE  240
AEC14852.1        VMNTEHKAYLDKNKAYPVECWVPDPTRNENTRYFGTLTGGENVPPVLHITNTATTVLLDE  240
AEC14853.1        VMNTEHKAYLDKNKAYPVECWVPDPTRNENTRYFGTLTGGENVPPVLHITNTATTVLLDE  240
AEC14854.1        VMNTEHKAYLDKNKAYPVECWVPDPTRNENTRYFGTLTGGENVPPVLHITNTATTVLLDE  240
AEC14855.1        VMNTEHKAYLDKNKAYPVECWVPDPTRNENTRYFGTLTGGENVPPVLHITNTATTVLLDE  240
AEC14856.1        VMNTEHKAYLDKNKAYPVECWVPDPTRNENTRYFGTLTGGENVPPVLHITNTATTVLLDE  240
AEC14857.1        VMNTEHKAYLDKNKAYPVECWVPDPTRNENTRYFGTLTGGENVPPVLHITNTATTVLLDE  240
AEC14858.1        VMNTEHKAYLDKNKAYPVECWVPDPTRNENTRYFGTLTGGENVPPVLHITNTATTVLLDE  240
AEC14859.1        VMNTEHKAYLDKNKAYPVECWVPDPTRNENTRYFGTLTGGENVPPVLHITNTATTVLLDE  240
AEC14862.1        VMNTEHKAYLDKNKAYPVECWVPDPTRNENTRYFGTLTGGENVPPVLHITNTATTVLLDE  240
AEC14863.1        VMNTEHKAYLDKNKAYPVECWVPDPTRNENTRYFGTLTGGENVPPVLHITNTATTVLLDE  240
BAD21289.1        VMNTEHKAYLDENKAYPVECWVPDPTRNENTRYFGTLTGGENVPPVLHITNTATTVLLDE  240
BAD21295.1        VMNTEHKAYLDENKAYPVECWVPDPTRNENTRYFGTLTGGENVPPVLHITNTATTVLLDE  240
BAB93044.1        VMNTEHKAYLDKNKAYPVECWVPDPTRNENTRYFGTLTGGENVPPVLHITNTATTVLLDE  240
BAB93104.1        VMNTEHKAYLDKNKAYPVECWGPDPTRNENTRYFGTLTGGENVPPVLHITNTATTVLLDE  240
AAK70252.1        VMNTEHKAYLDKNKAYPVECWVPDPTRNENTRYFGTLTGGENVPPVLHITNTATTVLLDE  240
BAB68959.1        VMNTEHKAYLDKNKAYPVECWVPDPTRNENTRYFGTLTGGENVPPVLHITNTATTVLLDE  240
BAD21229.1        VMNTEHKAYLDKNKAYPVECWVPDPTRNENTRYFGTLTGGENVPPVLHITNTATTVLLDE  240
QBX89123.1        VMNTEHKAYLDKNKAYPVECWVPDPTRNENTRYFGTLTGGENVPPVLHITNTATTVLLDE  240
BAE02908.1        VMNTEHKAYLDKNKAYPVECWVPDPTRNENTRYFGTLTGGENVPPVLHITNTATTVLLDE  240
AAR89259.1        VMNTEHKAYLDKNKAYPVECWVPDPTRNENTRYFGTLTGGENVPPVLHITNTATTVLLDE  240
AAR12957.1        VMNTEHKAYLDKNKAYPVECWVPDPTRNENTRYFGTLTGGENVPPVLHITNTATTVLLDE  240
AAR13077.1        VMNTEHKAYLDKNKAYPVECWVPDPTRNENTRYFGTLTGGENVPPVLHITNTATTVLLDE  240
BCU41036.1        VMNTEHKAYLDKNKAYPVECWVPDPTRNENTRYFGTLTGGENVPPVLHITNTATTVLLDE  240
BAE94726.1        VMNTEHKAYLDKNKAYPVECWVPDPTRNENTRYFGTLTGGENVPPVLHITNTATTVLLDE  240
AAB94036.1        VMNTEHKAYLDKNKAYPVECWVPDPTRNENTRYFGTLTGGENVPPVLHITNTATTVLLDE  240
AAK28478.1        VMNTEHKAYLDKNKAYPVECWVPDPTRNENTRYFGTLTGGENVPPVLHITNTATTVLLDE  240
BAB93008.1        VMNTEHKAYLDKNKAYPVECWVPDPTRNENTRYFGTLTGGENVPPVLHITNTATTVLLDE  240
AAM69579.1        VMNTEHKAYLDKNKAYPVECWVPDPTRNENTRYFGTLTGGENVPPVLHITNTATTVLLDE  240
AAM69584.1        VMNTEHKAYLDKNKAYPVECWVPDPTRNENTRYFGTLTGGENVPPVLHITNTATTVLLDE  240
AAM69596.1        VMNTEHKAYLDKNKAYPVECWVPDPTRNENTRYFGTLTGGENVPPVLHITNTATTVLLDE  240
AAM69602.1        VMNTEHKAYLDKNKAYPVECWVPDPTRNENTRYFGTLTGGENVPPVLHITNTATTVLLDE  240
AAM69608.1        VMNTEHKAYLDKNKAYPVECWVPDPTRNENTRYFGTLTGGENVPPVLHITNTATTVLLDE  240
AAK70312.1        VMNTEHKAYLDKNKAYPVECWVPDPTRNENTRYFGTLTGGENVPPVLHITNTATTVLLDE  240
WLD48064.1        VMNTEHKAYLDKNKAYPVECWVPDPTRNENTRYFGTLTGGENVPPVLHITNTATTVLLDE  240
WLD48100.1        VMNTEHKAYLDKNKAYPVECWVPDPTRNENTRYFGTLTGGENVPPVLHITNTATTVLLDE  240
UQK62655.1        VMNTEHKAYLDKNKAYPVECWVPDPTRNENTRYFGTLTGGENVPPVLHITNTATTVLLDE  240
QTJ15180.1        VMNTEHKAYLDKNKAYPVECWVPDPTRNENTRYFGTLTGGENVPPVLHITNTATTVLLDE  240
QTJ15204.1        VMNTEHKAYLDKNKAYPVECWVPDPTRNENTRYFGTLTGGENVPPVLHITNTATTVLLDE  240
ASV51784.1        VMNTEHKAYLDKNKAYPVECWVPDPTRNENTRYFGTLTGGENVPPVLHITNTATTVLLDE  240
ASV51790.1        VMNTEHKAYLDKNKAYPVECWVPDPTRNENTRYFGTLTGGENVPPVLHITNTATTVLLDE  240
ASV51898.1        VMNTEHKAYLDKNKAYPVECWVPDPTRNENTRYFGTLTGGENVPPVLHITNTATTVLLDE  240
ASV51904.1        VMNTEHKAYLDKNKAYPVECWVPDPTRNENTRYFGTLTGGENVPPVLHITNTATTVLLDE  240
ASV51910.1        VMNTEHKAYLDKNKAYPVECWVPDPTRNENTRYFGTLTGGENVPPVLHITNTATTVLLDE  240
ASV51916.1        VMNTEHKAYLDKNKAYPVECWVPDPTRNENTRYFGTLTGGENVPPVLHITNTATTVLLDE  240
CCW72490.1        VMNTEHKAYLDKNKAYPVECWVPDPTRNENTRYFGTLTGGENVPPVLHITNTATTVLLDE  240
CCW72495.1        VMNTEHKAYLDKNKAYPVECWVPDPTRNENTRYFGTLTGGENVPPVLHITNTATTVLLDE  240
CCW72497.1        VMNTEHKAYLDKNKAYPVECWVPDPTRNENTRYFGTLTGGENVPPVLHITNTATTVLLDE  240
```

CCW72503.1	VMNTEHKAYLDKNKAYPVECWVPDPTRNENTRYFGTLTGGENVPPVLHITNTATTVLLDE	240
CCW72505.1	VMNTEHKAYLDKNKAYPVECWVPDPTRNENTRYFGTLTGGENVPPVLHITNTATTVLLDE	240
CCW72507.1	VMNTEHKAYLDKNKAYPVECWVPDPTRNENTRYFGTLTGGENVPPVLHITNTATTVLLDE	240
CCW72512.1	VMNTEHKAYLDKNKAYPVECWVPDPTRNENTRYFGTLTGGENVPPVLHITNTATTVLLDE	240
CCW72515.1	VMNTEHKAYLDKNKAYPVECWVPDPTRNENTRYFGTLTGGENVPPVLHITNTATTVLLDE	240
BAB68881.1	VMNTEHKAYLDKNKAYPVECWVPDPTRNENTRYFGTLTGGENVPPVLHITNTATTVLLDE	240
BAB68887.1	VMNTEHKAYLDKNKAYPVECWVPDPTRNENTRYFGTLTGGENVPPVLHITNTATTVLLDE	240
AAK70294.1	VMNTEHKAYLDKNKAYPVECWVPDPTRNENTRYFGTLTGGENVPPVLHITNTATTVLLDE	240
WLD48094.1	VMNTEHKAYLDKNKAYPVECWVPDPTRNENTRYFGTLTGGENVPPVLHITNTATTVLLDE	240
CCW72509.1	VMNTEHKAYLDKNKAYPVECWVPDPTRNENTRYFGTLTGGENVPPVLHITNTATTVLLDE	240
BAB68893.1	VMNTEHKAYLDKNKAYPVECWVPDPTRNENTRYFGTLTGGENVPPVLHITNTATTVLLDE	240
QTJ15216.1	VMNTEHKAYLDKNKAYPVECWVPDPTRNENTRYFGTLTGGENVPPVLHITNTATTVLLDE	240
CCW72493.1	VMNTEHKAYLDKNKAYPVECWVPDPTRNENTRYFGTLTGGENVPPVLHITNTATTVLLDE	240
CCW72500.1	VMNTEHKAYLDKNKAYPVECWVPDPTRNENTRYFGTLTGGENVPPVLHITNTATTVLLDE	240
CCW72506.1	VMNTEHKAYLDKNKAYPVECWVPDPTRNENTRYFGTLTGGENVPPVLHITNTATTVLLDE	240
CCW72520.1	VMNTEHKAYLDKNKAYPVECWVPDPTRNENTRYFGTLTGGENVPPVLHITNTATTVLLDE	240
BAB68899.1	VMNTEHKAYLDKNKAYPVECWVPDPTRNENTRYFGTLTGGENVPPVLHITNTATTVLLDE	240
BAB68905.1	VMNTEHKAYLDKNKAYPVECWVPDPTRNENTRYFGTLTGGENVPPVLHITNTATTVLLDE	240
AAK70300.1	VMNTEHKAYLDKNKAYPVECWVPDPTRNENTRYFGTLTGGENVPPVLHITNTATTVLLDE	240
AYM55579.1	VMNTEHKAYLDKNKAYPVECWVPDPTRNENTRYFGTLTGGENVPPVLHITNTATTVLLDE	240
AYM55580.1	VMNTEHKAYLDKNKAYPVECWVPDPTRNENTRYFGTLTGGENVPPVLHITNTATTVLLDE	240
AYM55582.1	VMNTEHKAYLDKNKAYPVECWVPDPTRNENTRYFGTLTGGENVPPVLHITNTATTVLLDE	240
AYM55583.1	VMNTEHKAYLDKNKAYPVECWVPDPTRNENTRYFGTLTGGENVPPVLHITNTATTVLLDE	240
AYM55584.1	VMNTEHKAYLDKNKAYPVECWVPDPTRNENTRYFGTLTGGENVPPVLHITNTATTVLLDE	240
AYM55590.1	VMNTEHKAYLDKNKAYPVECWVPDPTRNENTRYFGTLTGGENVPPVLHITNTATTVLLDE	240
AYM55596.1	VMNTEHKAYLDKNKAYPVECWVPDPTRNENTRYFGTLTGGENVPPVLHITNTATTVLLDE	240
BAE45366.1	VMNTEHKAYLDKNKAYPVECWVPDPTRNENTRYFGTLTGGENVPPVLHITNTATTVLLDE	240
AAG30857.1	VMNTEHKAYLDKNKAYPVECWVPDPTRNENTRYFGTLTGGENVPPVLHITNTATTVLLDE	240
BAH36774.1	VMNTEHKAYLDKNKAYPVECWVPDPTRNENPRYFGTLTGGENVPPVLHITNTATTVLLDE	240
BAH36780.1	VMNTEHKAYLDKNKAYPVECWVPDPTRNENPRYFGTLTGGENVPPVLHITNTATTVLLDE	240
BAH36822.1	VMNTEHKAYLDKNKAYPVECWVPDPTRNENPRYFGTLTGGENVPPVLHITNTATTVLLDE	240
BAH36834.1	VMNTEHKAYLDKNKAYPVECWVPDPTRNENPRYFGTLTGGENVPPVLHITNTATTVLLDE	240
BAH36840.1	VMNTEHKAYLDKNKAYPVECWVPDPTRNENPRYFGTLTGGENVPPVLHITNTATTVLLDE	240
BAH36846.1	VMNTEHKAYLDKNKAYPVECWVPDPTRNENPRYFGTLTGGENVPPVLHITNTATTVLLDE	240
BAH36816.1	VMNTEHKAYLDKNKAYPVECWVPDPTRNENTRYFGTLKGGENVPPVLHITNTATTVLLDE	240
BAE45444.1	VMNTEHKAYLDKNKAYPVECWVPDPTRNENTRYFGTLTGGENVPPVLHITNTATTVLLDE	240
AAT09819.1	VMNTEHKAYLDKNKAYPVECWVPDPTRNENTRYFGTLTGGENVPPVLHITNTATTVLLDE	240
BAC03029.1	VMNTEHKAYLDKNKAYPVECWVPDPTRNENTRYFGTLTGGENVPPVLHITNTATTVLLDE	240
BAC03095.1	VMNTEHKAYLDKNKAYPVECWVPDPTRNENTRYFGTLTGGENVPPVLHITNTATTVLLDE	240
AAM69560.1	VMNTEHKAYLDKNKAYPVECWVPDPTRNENTRYFGTLTGGENVPPVLHITNTATTVLLDE	240
AAM69566.1	VMNTEHKAYLDKNKAYPVECWVPDPTRNENTRYFGTLTGGENVPPVLHITNTATTVLLDE	240
AAM69572.1	VMNTEHKAYLDKNKAYPVECWVPDPTRNENTRYFGTLTGGENVPPVLHITNTATTVLLDE	240
AAB84311.1	VMNTEHKAYLDKNKAYPVECWVPDPTRNENTRYFGTLTGGENVPPVLHITNTATTVLLDE	240
BAD06150.1	VMNTEHKAYLDKNKAYPVECWVPDPTRNENTRYFGTLTGGENVPPVLHITNTATTVLLDE	240
AYM55581.1	VMNTEHKAYLDKNKAYPVECWVPDPTRNENTRYFGTLTGGENVPPVLHITNTATTVLLDE	240
AYM55588.1	VMNTEHKAYLDKNKAYPVECWVPDPTRNENTRYFGTLTGGENVPPVLHITNTATTVLLDE	240
AYM55589.1	VMNTEHKAYLDKNKAYPVECWVPDPTRNENTRYFGTLTGGENVPPVLHITNTATTVLLDE	240
AYM55591.1	VMNTEHKAYLDKNKAYPVECWVPDPTRNENTRYFGTLTGGENVPPVLHITNTATTVLLDE	240
AYM55592.1	VMNTEHKAYLDKNKAYPVECWVPDPTRNENTRYFGTLTGGENVPPVLHITNTATTVLLDE	240
AYM55593.1	VMNTEHKAYLDKNKAYPVECWVPDPTRNENTRYFGTLTGGENVPPVLHITNTATTVLLDE	240
AYM55595.1	VMNTEHKAYLDKNKAYPVECWVPDPTRNENTRYFGTLTGGENVPPVLHITNTATTVLLDE	240
AYM55597.1	VMNTEHKAYLDKNKAYPVECWVPDPTRNENTRYFGTLTGGENVPPVLHITNTATTVLLDE	240
AYM55598.1	VMNTEHKAYLDKNKAYPVECWVPDPTRNENTRYFGTLTGGENVPPVLHITNTATTVLLDE	240
AYM55599.1	VMNTEHKAYLDKNKAYPVECWVPDPTRNENTRYFGTLTGGENVPPVLHITNTATTVLLDE	240
AYM55600.1	VMNTEHKAYLDKNKAYPVECWVPDPTRNENTRYFGTLTGGENVPPVLHITNTATTVLLDE	240
AYM55601.1	VMNTEHKAYLDKNKAYPVECWVPDPTRNENTRYFGTLTGGENVPPVLHITNTATTVLLDE	240
AYM55602.1	VMNTEHKAYLDKNKAYPVECWVPDPTRNENTRYFGTLTGGENVPPVLHITNTATTVLLDE	240
AYM55603.1	VMNTEHKAYLDKNKAYPVECWVPDPTRNENTRYFGTLTGGENVPPVLHITNTATTVLLDE	240
CCW72483.1	VMNTEHKAYLDKNKAYPVECWVPDPTRNENTRYFGTLTGGENVPPVLHITNTATTVLLDE	240
CCW72485.1	VMNTEHKAYLDKNKAYPVECWVPDPTRNENTRYFGTLTGGENVPPVLHITNTATTVLLDE	240
AFR56669.1	VMNTEHKAYLDKNKAYPVECWVPDPTRNENTRYFGTLTGGENVPPVLHITNTATTVLLDE	240
BAH36768.1	VMNTEHKAYLDKNKAYPVECWVPDPTRNENTRYFGTLTGGENVPPVLHITNTATTVLLDE	240
BAH36786.1	VMNTEHKAYLDKNKAYPVECWVPDPTRNENTRYFGTLTGGENVPPVLHITNTATTVLLDE	240
BAH36792.1	VMNTEHKAYLDKNKAYPVECWVPDPTRNENTRYFGTLTGGENVPPVLHITNTATTVLLDE	240

BAH36798.1	VMNTEHKAYLDKNKAYPVECWVPDPTRNENTRYFGTLTGGENVPPVLHITNTATTVLLDE	240
BAH36804.1	VMNTEHKAYLDKNKAYPVECWVPDPTRNENTRYFGTLTGGENVPPVLHITNTATTVLLDE	240
BAH36810.1	VMNTEHKAYLDKNKAYPVECWVPDPTRNENTRYFGTLTGGENVPPVLHITNTATTVLLDE	240
BAH36828.1	VMNTEHKAYLDKNKAYPVECWVPDPTRNENTRYFGTLTGGENVPPVLHITNTATTVLLDE	240
BAH36852.1	VMNTEHKAYLDKNKAYPVECWVPDPTRNENTRYFGTLTGGENVPPVLHITNTATTVLLDE	240
BAH36858.1	VMNTEHKAYLDKNKAYPVECWVPDPTRNENTRYFGTLTGGENVPPVLHITNTATTVLLDE	240
BAE75814.1	VMNTEHKAYLDKNKAYPVECWVPDPTRNENTRYFGTLTGGENVPPVLHITNTATTVLLDE	240
BAE75820.1	VMNTEHKAYLDKNKAYPVECWVPDPTRNENTRYFGTLTGGENVPPVLHITNTATTVLLDE	240
BAE75826.1	VMNTEHKAYLDKNKAYPVECWVPDPTRNENTRYFGTLTGGENVPPVLHITNTATTVLLDE	240
BAE75832.1	VMNTEHKAYLDKNKAYPVECWVPDPTRNENTRYFGTLTGGENVPPVLHITNTATTVLLDE	240
BAE75838.1	VMNTEHKAYLDKNKAYPVECWVPDPTRNENTRYFGTLTGGENVPPVLHITNTATTVLLDE	240
BAE45360.1	VMNTEHKAYLDKNKAYPVECWVPDPTRNENTRYFGTLTGGENVPPVLHITNTATTVLLDE	240
BAE45372.1	VMNTEHKAYLDKNKAYPVECWVPDPTRNENTRYFGTLTGGENVPPVLHITNTATTVLLDE	240
BAE45378.1	VMNTEHKAYLDKNKAYPVECWVPDPTRNENTRYFGTLTGGENVPPVLHITNTATTVLLDE	240
BAE45384.1	VMNTEHKAYLDKNKAYPVECWVPDPTRNENTRYFGTLTGGENVPPVLHITNTATTVLLDE	240
BAE45390.1	VMNTEHKAYLDKNKAYPVECWVPDPTRNENTRYFGTLTGGENVPPVLHITNTATTVLLDE	240
BAE45396.1	VMNTEHKAYLDKNKAYPVECWVPDPTRNENTRYFGTLTGGENVPPVLHITNTATTVLLDE	240
BAE45402.1	VMNTEHKAYLDKNKAYPVECWVPDPTRNENTRYFGTLTGGENVPPVLHITNTATTVLLDE	240
BAE45408.1	VMNTEHKAYLDKNKAYPVECWVPDPTRNENTRYFGTLTGGENVPPVLHITNTATTVLLDE	240
BAE45414.1	VMNTEHKAYLDKNKAYPVECWVPDPTRNENTRYFGTLTGGENVPPVLHITNTATTVLLDE	240
BAE45420.1	VMNTEHKAYLDKNKAYPVECWVPDPTRNENTRYFGTLTGGENVPPVLHITNTATTVLLDE	240
BAE45426.1	VMNTEHKAYLDKNKAYPVECWVPDPTRNENTRYFGTLTGGENVPPVLHITNTATTVLLDE	240
BAE45432.1	VMNTEHKAYLDKNKAYPVECWVPDPTRNENTRYFGTLTGGENVPPVLHITNTATTVLLDE	240
BAE45438.1	VMNTEHKAYLDKNKAYPVECWVPDPTRNENTRYFGTLTGGENVPPVLHITNTATTVLLDE	240
BAD06018.1	VMNTEHKAYLDKNKAYPVECWVPDPTRNENTRYFGTLTGGENVPPVLHITNTATTVLLDE	240
BAD06024.1	VMNTEHKAYLDKNKAYPVECWVPDPTRNENTRYFGTLTGGENVPPVLHITNTATTVLLDE	240
BAD06030.1	VMNTEHKAYLDKNKAYPVECWVPDPTRNENTRYFGTLTGGENVPPVLHITNTATTVLLDE	240
BAD06042.1	VMNTEHKAYLDKNKAYPVECWVPDPTRNENTRYFGTLTGGENVPPVLHITNTATTVLLDE	240
BAD06048.1	VMNTEHKAYLDKNKAYPVECWVPDPTRNENTRYFGTLTGGENVPPVLHITNTATTVLLDE	240
BAD06054.1	VMNTEHKAYLDKNKAYPVECWVPDPTRNENTRYFGTLTGGENVPPVLHITNTATTVLLDE	240
BAD06084.1	VMNTEHKAYLDKNKAYPVECWVPDPTRNENTRYFGTLTGGENVPPVLHITNTATTVLLDE	240
BAD06090.1	VMNTEHKAYLDKNKAYPVECWVPDPTRNENTRYFGTLTGGENVPPVLHITNTATTVLLDE	240
BAD06096.1	VMNTEHKAYLDKNKAYPVECWVPDPTRNENTRYFGTLTGGENVPPVLHITNTATTVLLDE	240
BAD06102.1	VMNTEHKAYLDKNKAYPVECWVPDPTRNENTRYFGTLTGGENVPPVLHITNTATTVLLDE	240
BAD06108.1	VMNTEHKAYLDKNKAYPVECWVPDPTRNENTRYFGTLTGGENVPPVLHITNTATTVLLDE	240
BAD06120.1	VMNTEHKAYLDKNKAYPVECWVPDPTRNENTRYFGTLTGGENVPPVLHITNTATTVLLDE	240
BAC15695.1	VMNTEHKAYLDKNKAYPVECWVPDPTRNENTRYFGTLTGGENVPPVLHITNTATTVLLDE	240
BAC15725.1	VMNTEHKAYLDKNKAYPVECWVPDPTRNENTRYFGTLTGGENVPPVLHITNTATTVLLDE	240
BAC02951.1	VMNTEHKAYLDKNKAYPVECWVPDPTRNENTRYFGTLTGGENVPPVLHITNTATTVLLDE	240
BAC02957.1	VMNTEHKAYLDKNKAYPVECWVPDPTRNENTRYFGTLTGGENVPPVLHITNTATTVLLDE	240
BAC02963.1	VMNTEHKAYLDKNKAYPVECWVPDPTRNENTRYFGTLTGGENVPPVLHITNTATTVLLDE	240
BAC02969.1	VMNTEHKAYLDKNKAYPVECWVPDPTRNENTRYFGTLTGGENVPPVLHITNTATTVLLDE	240
BAC02975.1	VMNTEHKAYLDKNKAYPVECWVPDPTRNENTRYFGTLTGGENVPPVLHITNTATTVLLDE	240
BAC02981.1	VMNTEHKAYLDKNKAYPVECWVPDPTRNENTRYFGTLTGGENVPPVLHITNTATTVLLDE	240
BAC02987.1	VMNTEHKAYLDKNKAYPVECWVPDPTRNENTRYFGTLTGGENVPPVLHITNTATTVLLDE	240
BAC02993.1	VMNTEHKAYLDKNKAYPVECWVPDPTRNENTRYFGTLTGGENVPPVLHITNTATTVLLDE	240
BAC02999.1	VMNTEHKAYLDKNKAYPVECWVPDPTRNENTRYFGTLTGGENVPPVLHITNTATTVLLDE	240
BAC03005.1	VMNTEHKAYLDKNKAYPVECWVPDPTRNENTRYFGTLTGGENVPPVLHITNTATTVLLDE	240
BAC03011.1	VMNTEHKAYLDKNKAYPVECWVPDPTRNENTRYFGTLTGGENVPPVLHITNTATTVLLDE	240
BAC03017.1	VMNTEHKAYLDKNKAYPVECWVPDPTRNENTRYFGTLTGGENVPPVLHITNTATTVLLDE	240
BAC03023.1	VMNTEHKAYLDKNKAYPVECWVPDPTRNENTRYFGTLTGGENVPPVLHITNTATTVLLDE	240
BAC03035.1	VMNTEHKAYLDKNKAYPVECWVPDPTRNENTRYFGTLTGGENVPPVLHITNTATTVLLDE	240
BAC03041.1	VMNTEHKAYLDKNKAYPVECWVPDPTRNENTRYFGTLTGGENVPPVLHITNTATTVLLDE	240
BAC03047.1	VMNTEHKAYLDKNKAYPVECWVPDPTRNENTRYFGTLTGGENVPPVLHITNTATTVLLDE	240
BAC03053.1	VMNTEHKAYLDKNKAYPVECWVPDPTRNENTRYFGTLTGGENVPPVLHITNTATTVLLDE	240
BAC03059.1	VMNTEHKAYLDKNKAYPVECWVPDPTRNENTRYFGTLTGGENVPPVLHITNTATTVLLDE	240
BAC03071.1	VMNTEHKAYLDKNKAYPVECWVPDPTRNENTRYFGTLTGGENVPPVLHITNTATTVLLDE	240
BAC03077.1	VMNTEHKAYLDKNKAYPVECWVPDPTRNENTRYFGTLTGGENVPPVLHITNTATTVLLDE	240
BAC03083.1	VMNTEHKAYLDKNKAYPVECWVPDPTRNENTRYFGTLTGGENVPPVLHITNTATTVLLDE	240
BAC03089.1	VMNTEHKAYLDKNKAYPVECWVPDPTRNENTRYFGTLTGGENVPPVLHITNTATTVLLDE	240
AAM69542.1	VMNTEHKAYLDKNKAYPVECWVPDPTRNENTRYFGTLTGGENVPPVLHITNTATTVLLDE	240
AAM69548.1	VMNTEHKAYLDKNKAYPVECWVPDPTRNENTRYFGTLTGGENVPPVLHITNTATTVLLDE	240
AAM69554.1	VMNTEHKAYLDKNKAYPVECWVPDPTRNENTRYFGTLTGGENVPPVLHITNTATTVLLDE	240
AAM69619.1	VMNTEHKAYLDKNKAYPVECWVPDPTRNENTRYFGTLTGGENVPPVLHITNTATTVLLDE	240

BAB68977.1	VMNTEHKAYLDKNKAYPVECWVPDPTRNENTRYFGTLTGGENVPPVLHITNTATTVLLDE	240
BAB68983.1	VMNTEHKAYLDKNKAYPVECWVPDPTRNENTRYFGTLTGGENVPPVLHITNTATTVLLDE	240
BAB68989.1	VMNTEHKAYLDKNKAYPVECWVPDPTRNENTRYFGTLTGGENVPPVLHITNTATTVLLDE	240
BAB68995.1	VMNTEHKAYLDKNKAYPVECWVPDPTRNENTRYFGTLTGGENVPPVLHITNTATTVLLDE	240
AAK98000.1	VMNTEHKAYLDKNKAYPVECWVPDPTRNENTRYFGTLTGGENVPPVLHITNTATTVLLDE	240
AAK98006.1	VMNTEHKAYLDKNKAYPVECWVPDPTRNENTRYFGTLTGGENVPPVLHITNTATTVLLDE	240
AAK98010.1	VMNTEHKAYLDKNKAYPVECWVPDPTRNENTRYFGTLTGGENVPPVLHITNTATTVLLDE	240
AAK98018.1	VMNTEHKAYLDKNKAYPVECWVPDPTRNENTRYFGTLTGGENVPPVLHITNTATTVLLDE	240
AAK98024.1	VMNTEHKAYLDKNKAYPVECWVPDPTRNENTRYFGTLTGGENVPPVLHITNTATTVLLDE	240
AAK98030.1	VMNTEHKAYLDKNKAYPVECWVPDPTRNENTRYFGTLTGGENVPPVLHITNTATTVLLDE	240
AAK98036.1	VMNTEHKAYLDKNKAYPVECWVPDPTRNENTRYFGTLTGGENVPPVLHITNTATTVLLDE	240
AAK70258.1	VMNTEHKAYLDKNKAYPVECWVPDPTRNENTRYFGTLTGGENVPPVLHITNTATTVLLDE	240
AAK70264.1	VMNTEHKAYLDKNKAYPVECWVPDPTRNENTRYFGTLTGGENVPPVLHITNTATTVLLDE	240
AAG53896.1	VMNTEHKAYLDKNKAYPVECWVPDPTRNENTRYFGTLTGGENVPPVLHITNTATTVLLDE	240
BAA01962.1	VMNTEHKAYLDKNKAYPVECWVPDPTRNENTRYFGTLTGGENVPPVLHITNTATTVLLDE	240
ALF06256.1	VMNTEHKAYLDKNKAYPVECWVPDPTRNENTRYFGTLTGGENVPPVLHITNTATTVLLDE	240
BAD06060.1	VMNTEHKAYLDKNKAYPVECWVPDPTRNENTRYFGTLTGGENVPPVLHITNTATTVLLDE	240
BAD06072.1	VMNTEHKAYLDKNKAYPVECWVPDPTRNENTRYFGTLTGGENVPPVLHITNTATTVLLDE	240
BAD06156.1	VMNTEHKAYLDKNKAYPVECWVPDPTRNENTRYFGTLTGGENVPPVLHITNTATTVLLDE	240
BAD06174.1	VMNTEHKAYLDKNKAYPVECWVPDPTRNENTRYFGTLTGGENVPPVLHITNTATTVLLDE	240
AAN85449.1	VMNTEHKAYLDKNKAYPVECWVPDPTRNENTRYFGTLTGGENVPPVLHITNTATTVLLDE	240
AAN85455.1	VMNTEHKAYLDKNKAYPVECWVPDPTRNENTRYFGTLTGGENVPPVLHITNTATTVLLDE	240
AAK98042.1	VMNTEHKAYLDKNKAYPVECWVPDPTRNENTRYFGTLTGGENVPPVLHITNTATTVLLDE	240
BAD06036.1	VMNTEHKAYLDKNKAYPVECWVPDPTRNENTRYFGTLTGGENVPPVLHITNTATTVLLDE	240
BCP65063.1	VMNTEHKAYLDKNKAYPVECWVPDPTRNENTRYFGTLTGGENVPPVLHITNTATTVLLDE	240
BCP56477.1	VMNTEHKAYLDKNKAYPVECWVPDPTRNENTRYFGTLTGGENVPPVLHITNTATTVLLDE	240
BAV69373.1	VMNTEHKAYLDKNKAYPVECWVPDPTRNENTRYFGTLTGGENVPPVLHITNTATTVLLDE	240
BAE00153.1	VMNTEHKAYLDKNKAYPVECWVPDPTRNENTRYFGTLTGGENVPPVLHITNTATTVLLDE	240
BAC22906.1	VMNTEHKAYLDKNKAYPVECWVPDPTRNENTRYFGTLTGGENVPPVLHITNTATTVLLDE	240
BAA05639.1	VMNTEHKAYLDKNKAYPVECWVPDPTRNENTRYFGTLTGGENVPPVLHITNTATTVLLDE	240
BAB11728.1	VMNTEHKAYLDKNKAYPVECWVPDPTRNENTRYFGTLTGGENVPPVLHITNTATTVLLDE	240
BAA05637.1	VMNTEHKAYLDKNKAYPVECWVPDPTRNENTRYFGTLTGGENVPPVLHITNTATTVLLDE	240
BAA01969.1	VMNTEHKAYLDKNKAYPVECWVPDPTRNENTRYFGTLTGGENVPPVLHITNTATTVLLDE	240
BAE00159.1	VMNTEHKAYLDKNKAYPVECWVPDPTRNENTRYFGTLTGGENVPPVLHITNTATTVLLDE	240
BAE00165.1	VMNTEHKAYLDKNKAYPVECWVPDPTRNENTRYFGTLTGGENVPPVLHITNTATTVLLDE	240
BAE00171.1	VMNTEHKAYLDKNKAYPVECWVPDPTRNENTRYFGTLTGGENVPPVLHITNTATTVLLDE	240
ALF06268.1	VMNTEHKAYLDKNKAYPVECWVPDPTRNENTRYFGTLTGGENVPPVLHITNTATTVLLDE	240
AYM55586.1	VMNTEHKAYLDKNKAYPVECWVPDPTRNENTRYFGTLTGGENVPPVLHITNTATTVLLDE	240
AYM55585.1	VMNTEHKAYLDKNKAYPVECWVPDPTRNENTRYFGTLTGGENVPPVLHITNTATTVLLDE	240
AYM55587.1	VMNTEHKAYLDKNKAYPVECWVPDPTRNENTRYFGTLTGGENVPPVLHITNTATTVLLDE	240
AYM55594.1	VMNTEHKAYLDKNKAYPVECWVPDPTRNENTRYFGTLTGGENVPPVLHITNTATTVLLDE	240
QBQ65470.1	VMNTEHKAYLDKNKAYPVECWVPDPTRNENTRYFGTLTGGENVPPVLHITNTATTVLLDE	240
CCW72501.1	VMNTEHKAYLDKNKAYPVECWVPDPTRNENTRYFGTLTGGENVPPVLHITNTATTVLLDE	240
CCW72524.1	VMNTEHKAYLDKNKAYPVECWVPDPTRNENTRYFGTLTGGENVPPVLHITNTATTVLLDE	240
QWT77116.1	VMNTEHKAYLDKNKAYPVECWVPDPTRNENTRYFGTLTGGENVPPVLHITNTATTVLLDE	240
WLD48046.1	VMNTEHKAYLDKNKAYPVECWVPDPTRNENTRYFGTLTGGENVPPVLHITNTATTVLLDE	240
WLD48052.1	VMNTEHKAYLDKNKAYPVECWVPDPTRNENTRYFGTLTGGENVPPVLHITNTATTVLLDE	240
AAK70288.1	VMNTEHKAYLDKNKAYPVECWVPDPTRNENTRYFGTLTGGENVPPVLHITNTATTVLLDE	240
AAK70270.1	VMNTEHKAYLYKNKAYPVECWVPDPTRNENTRYFGTLTGGENVPPVLHITNTATTVLLDE	240
BAD21277.2	VMNTEHKAYLDKNKAYPVECWVPDPTRNENTRYFGTLTGGENVPPVLHITNTATTVLLDE	240
CCW72498.1	VMNTEHKAYLDKNKAYPVECWVPDPTRNENTRYFGTLTGGENVPPVLHITNTATTVLLDE	240
UPN63085.1	VMNTEHKAYLDKNKAYPVECWVPDPTRNENTRYFGTLTGGENVPPVLHITNTATTVLLDE	240
UPN63090.1	VMNTEHKAYLDKNKAYPVECWVPDPTRNENTRYFGTLTGGENVPPVLHITNTATTVLLDE	240
QWT77134.1	VMNTEHKAYLDKNKAYPVECWVPDPTRNENTRYFGTLTGGENVPPVLHITNTATTVLLDE	240
WLD48070.1	VMNTEHKAYLDKNKAYPVECWVPDPTRNENTRYFGTLTGGENVPPVLHITNTATTVLLDE	240
AAK70282.1	VMNTEHKAYLDKNKAYPVECWVPDPTRNENTRYFGTLTGGENVPPVLHITNTATTVLLDE	240
WLD48058.1	VMNTEHKAYLDKNKAYPVECWVPDPTRNENTRYFGTLTGGENVPPVLHITNTATTVLLDE	240
WLD48076.1	VMNTEHKAYLDKNKAYPVECWVPDPTRNENTRYFGTLTGGENVPPVLHITNTATTVLLDE	240
UQK62667.1	VMNTEHKAYLDKNKAYPVECWVPDPTRNENTRYFGTLTGGENVPPVLHITNTATTVLLDE	240
CCW72480.1	VMNTEHKAYLDKNKAYPVECWVPDPTRNENTRYFGTLTGGENVPPVLHITNTATTVLLDE	240
CCW72513.1	VMNTEHKAYLDKNKAYPVECWVPDPTRNENTRYFGTLTGGENVPPVLHITNTATTVLLDE	240
AAM69614.1	VMNTEHKAYLDKNKAYPVECWVPDPTRNENTRYFGTLTGGENVPPVLHITNTATTALLDE	240
AAK70318.1	VMNTEHKAYLDKNKAYPVECWVPDPTRNENTRYFGTLTGGENVPPVLHITNTATTVLLDE	240
AAK70324.1	VMNTEHKAYLDKNKAYPVECWVPDPTGNENTGYFGTLTGGENVPPVLHITNTATTVLLDE	240

```
AAT09837.1      VMNTEHKAYLDKNKAYPVECWVPDPTRNENTRYFGTLTGGENVPPVLHITNTATTVLLDE  240
AAT09831.1      VMNTEHKAYLDKNKAYPVECWVPDPTRNENTRYFGTLTGGENVPPVLHITNTATTVLLDE  240
AAT09825.1      VMNTEHKAYLDKNKAYPVECWVPDPTRNENTRYFGTLTGGENVPPVLHITNTATTVLLDE  240
AAR89271.1      VMNTEHKAYLDKNKAYPVECWVPDPTRNENTRYFGTLTGGENVPPVLHITNTATTVLLDE  240
AAR89211.1      VMNTEHKAYLDKNKAYPVECWVPDPTRNENTRYFGTLTGGENVPPVLHITNTATTVLLDE  240
BAE02956.1      VMNTEHKAYLDKNKAYPVECWVPDPTRNENTRYFGTLTGGENVPPVLHITNTATTVLLDE  240
ACF75876.1      VMNTEHKAYLDKNKAYPVECWVPDPTRNENTRYFGTLTGGENVPPVLHITNTATTVLLDE  240
QBM11924.1      VMNTEHKAYLDKNKAYPVECWVPDPTRNENTRYFGTLTGGENVPPVLHITNTATTVLLDE  240
QBM11858.1      VMNTEHKAYLDKNKAYPVECWVPDPTRNENTRYFGTLTGGENVPPVLHITNTATTVLLDE  240
QBQ04203.1      VMNTEHKAYLDKNKAYPVECWVPDPTRNENTRYFGTLTGGENVPPVLHITNTATTVLLDE  240
QBQ65473.1      VMNTEHKAYLDKNKAYPVECWVPDPTRNENTRYFGTLTGGENVPPVLHITNTATTVLLDE  240
QBQ65472.1      VMNTEHKAYLDKNKAYPVECWVPDPTRNENTRYFGTLTGGENVPPVLHITNTATTVLLDE  240
QBM11920.1      VMNTEHKAYLDKNKAYPVECWVPDPTRNENTRYFGTLTGGENVPPVLHITNTATTVLLDE  240
QAA95693.1      VMNTEHKAYLDKNKAYPVECWVPDPTRNENTRYFGTLTGGENVPPVLHITNTATTVLLDE  240
QBX89122.1      VMNTEHKAYLDKNKAYPVECWVPDPTRNENTRYFGTLTGGENVPPVLHITNTATTVLLDE  240
QID57946.1      VMNTEHKAYLDKNKAYPVECWVPDPTRNENTRYFGTLTGGENVPPVLHITNTATTVLLDE  240
QID57952.1      VMNTEHKAYLDKNKAYPVECWVPDPTRNENTRYFGTLTGGENVPPVLHITNTATTVLLDE  240
QID57958.1      VMNTEHKAYLDKNKAYPVECWVPDPTRNENTRYFGTLTGGENVPPVLHITNTATTVLLDE  240
QID57964.1      VMNTEHKAYLDKNKAYPVECWVPDPTRNENTRYFGTLTGGENVPPVLHITNTATTVLLDE  240
QID57970.1      VMNTEHKAYLDKNKAYPVECWVPDPTRNENTRYFGTLTGGENVPPVLHITNTATTVLLDE  240
QID57976.1      VMNTEHKAYLDKNKAYPVECWVPDPTRNENTRYFGTLTGGENVPPVLHITNTATTVLLDE  240
QCH41110.1      VMNTEHKAYLDKNKAYPVECWVPDPTRNENTRYFGTLTGGENVPPVLHITNTATTVLLDE  240
QBX89120.1      VMNTEHKAYLDKNKAYPVECWVPDPTRNENTRYFGTLTGGENVPPVLHITNTATTVLLDE  240
QBX89121.1      VMNTEHKAYLDKNKAYPVECWVPDPTRNENTRYFGTLTGGENVPPVLHITNTATTVLLDE  240
QBX89124.1      VMNTEHKAYLDKNKAYPVECWVPDPTRNENTRYFGTLTGGENVPPVLHITNTATTVLLDE  240
QBQ65471.1      VMNTEHKAYLDKNKAYPVECWVPDPTRNENTRYFGTLTGGENVPPVLHITNTATTVLLDE  240
QBQ65474.1      VMNTEHKAYLDKNKAYPVECWVPDPTRNENTRYFGTLTGGENVPPVLHITNTATTVLLDE  240
QBQ65476.1      VMNTEHKAYLDKNKAYPVECWVPDPTRNENTRYFGTLTGGENVPPVLHITNTATTVLLDE  240
QBQ04201.1      VMNTEHKAYLDKNKAYPVECWVPDPTRNENTRYFGTLTGGENVPPVLHITNTATTVLLDE  240
QBM11921.1      VMNTEHKAYLDKNKAYPVECWVPDPTRNENTRYFGTLTGGENVPPVLHITNTATTVLLDE  240
QBM11922.1      VMNTEHKAYLDKNKAYPVECWVPDPTRNENTRYFGTLTGGENVPPVLHITNTATTVLLDE  240
CDJ79845.1      VMNTEHKAYLDKNKAYPVECWVPDPTRNENTRYFGTLTGGENVPPVLHITNTATTVLLDE  240
BAE02932.1      VMNTEHKAYLDKNKAYPVECWVPDPTRNENTRYFGTLTGGENVPPVLHITNTATTVLLDE  240
BAE02938.1      VMNTEHKAYLDKNKAYPVECWVPDPTRNENTRYFGTLTGGENVPPVLHITNTATTVLLDE  240
BAE02950.1      VMNTEHKAYLDKNKAYPVECWVPDPTRNENTRYFGTLTGGENVPPVLHITNTATTVLLDE  240
BAE03004.1      VMNTEHKAYLDKNKAYPVECWVPDPTRNENTRYFGTLTGGENVPPVLHITNTATTVLLDE  240
BAE03118.1      VMNTEHKAYLDKNKAYPVECWVPDPTRNENTRYFGTLTGGENVPPVLHITNTATTVLLDE  240
BAE03124.1      VMNTEHKAYLDKNKAYPVECWVPDPTRNENTRYFGTLTGGENVPPVLHITNTATTVLLDE  240
BAE03130.1      VMNTEHKAYLDKNKAYPVECWVPDPTRNENTRYFGTLTGGENVPPVLHITNTATTVLLDE  240
BAE03136.1      VMNTEHKAYLDKNKAYPVECWVPDPTRNENTRYFGTLTGGENVPPVLHITNTATTVLLDE  240
BAE03142.1      VMNTEHKAYLDKNKAYPVECWVPDPTRNENTRYFGTLTGGENVPPVLHITNTATTVLLDE  240
BAE03148.1      VMNTEHKAYLDKNKAYPVECWVPDPTRNENTRYFGTLTGGENVPPVLHITNTATTVLLDE  240
BAE03154.1      VMNTEHKAYLDKNKAYPVECWVPDPTRNENTRYFGTLTGGENVPPVLHITNTATTVLLDE  240
QBQ04200.1      VMNTEHKAYLDKNKAYPVECWVPDPTRNENTRYFGTLTGGENVPPVLHITNTATTVLLDE  240
UYL83839.1      VMNTEHKAYLDKNKAYPVECWVPDPTRNENTRYFGTLTGGENVPPVLHITNTATTVLLDE  240
ALF06262.1      VMNTEHKAYLDKNKAYPVECWVPDPTRNENTRYFGTLTGGENVPPVLHITNTATTVLLDE  240
ABH10586.1      VMNTEHKAYLDKNKAYPVECWVPDPTRNENTRYFGTLTGGENVPPVLHITNTATTVLLDE  240
BAE02890.1      VMNTEHKAYLDKNKAYPVECWVPDPTRNENTRYFGTLTGGENVPPVLHITNTATTVLLDE  240
BAE02896.1      VMNTEHKAYLDKNKAYPVECWVPDPTRNENTRYFGTLTGGENVPPVLHITNTATTVLLDE  240
BAE02902.1      VMNTEHKAYLDKNKAYPVECWVPDPTRNENTRYFGTLTGGENVPPVLHITNTATTVLLDE  240
BAE02914.1      VMNTEHKAYLDKNKAYPVECWVPDPTRNENTRYFGTLTGGENVPPVLHITNTATTVLLDE  240
BAE02920.1      VMNTEHKAYLDKNKAYPVECWVPDPTRNENTRYFGTLTGGENVPPVLHITNTATTVLLDE  240
BAE02944.1      VMNTEHKAYLDKNKAYPVECWVPDPTRNENTRYFGTLTGGENVPPVLHITNTATTVLLDE  240
BAE02962.1      VMNTEHKAYLDKNKAYPVECWVPDPTRNENTRYFGTLTGGENVPPVLHITNTATTVLLDE  240
BAE02968.1      VMNTEHKAYLDKNKAYPVECWVPDPTRNENTRYFGTLTGGENVPPVLHITNTATTVLLDE  240
BAE02974.1      VMNTEHKAYLDKNKAYPVECWVPDPTRNENTRYFGTLTGGENVPPVLHITNTATTVLLDE  240
BAE02980.1      VMNTEHKAYLDKNKAYPVECWVPDPTRNENTRYFGTLTGGENVPPVLHITNTATTVLLDE  240
BAE02986.1      VMNTEHKAYLDKNKAYPVECWVPDPTRNENTRYFGTLTGGENVPPVLHITNTATTVLLDE  240
BAE02992.1      VMNTEHKAYLDKNKAYPVECWVPDPTRNENTRYFGTLTGGENVPPVLHITNTATTVLLDE  240
BAE02998.1      VMNTEHKAYLDKNKAYPVECWVPDPTRNENTRYFGTLTGGENVPPVLHITNTATTVLLDE  240
BAE03010.1      VMNTEHKAYLDKNKAYPVECWVPDPTRNENTRYFGTLTGGENVPPVLHITNTATTVLLDE  240
BAE03016.1      VMNTEHKAYLDKNKAYPVECWVPDPTRNENTRYFGTLTGGENVPPVLHITNTATTVLLDE  240
BAE03022.1      VMNTEHKAYLDKNKAYPVECWVPDPTRNENTRYFGTLTGGENVPPVLHITNTATTVLLDE  240
BAE03028.1      VMNTEHKAYLDKNKAYPVECWVPDPTRNENTRYFGTLTGGENVPPVLHITNTATTVLLDE  240
```

```
BAE03034.1      VMNTEHKAYLDKNKAYPVECWVPDPTRNENTRYFGTLTGGENVPPVLHITNTATTVLLDE 240
BAE03040.1      VMNTEHKAYLDKNKAYPVECWVPDPTRNENTRYFGTLTGGENVPPVLHITNTATTVLLDE 240
BAE03046.1      VMNTEHKAYLDKNKAYPVECWVPDPTRNENTRYFGTLTGGENVPPVLHITNTATTVLLDE 240
BAE03052.1      VMNTEHKAYLDKNKAYPVECWVPDPTRNENTRYFGTLTGGENVPPVLHITNTATTVLLDE 240
BAE03058.1      VMNTEHKAYLDKNKAYPVECWVPDPTRNENTRYFGTLTGGENVPPVLHITNTATTVLLDE 240
BAE03064.1      VMNTEHKAYLDKNKAYPVECWVPDPTRNENTRYFGTLTGGENVPPVLHITNTATTVLLDE 240
BAE03070.1      VMNTEHKAYLDKNKAYPVECWVPDPTRNENTRYFGTLTGGENVPPVLHITNTATTVLLDE 240
BAE03076.1      VMNTEHKAYLDKNKAYPVECWVPDPTRNENTRYFGTLTGGENVPPVLHITNTATTVLLDE 240
BAE03082.1      VMNTEHKAYLDKNKAYPVECWVPDPTRNENTRYFGTLTGGENVPPVLHITNTATTVLLDE 240
BAE03088.1      VMNTEHKAYLDKNKAYPVECWVPDPTRNENTRYFGTLTGGENVPPVLHITNTATTVLLDE 240
BAE03094.1      VMNTEHKAYLDKNKAYPVECWVPDPTRNENTRYFGTLTGGENVPPVLHITNTATTVLLDE 240
BAE03100.1      VMNTEHKAYLDKNKAYPVECWVPDPTRNENTRYFGTLTGGENVPPVLHITNTATTVLLDE 240
BAE03106.1      VMNTEHKAYLDKNKAYPVECWVPDPTRNENTRYFGTLTGGENVPPVLHITNTATTVLLDE 240
BAE03112.1      VMNTEHKAYLDKNKAYPVECWVPDPTRNENTRYFGTLTGGENVPPVLHITNTATTVLLDE 240
BAE03160.1      VMNTEHKAYLDKNKAYPVECWVPDPTRNENTRYFGTLTGGENVPPVLHITNTATTVLLDE 240
BAE03166.1      VMNTEHKAYLDKNKAYPVECWVPDPTRNENTRYFGTLTGGENVPPVLHITNTATTVLLDE 240
AAQ88264.1      VMNTEHKAYLDKNKAYPVECWVPDPTRNENTRYFGTLTGGENVPPVLHITNTATTVLLDE 240
AAQ88270.1      VMNTEHKAYLDKNKAYPVECWVPDPTRNENTRYFGTLTGGENVPPVLHITNTATTVLLDE 240
AAR89187.1      VMNTEHKAYLDKNKAYPVECWVPDPTRNENTRYFGTLTGGENVPPVLHITNTATTVLLDE 240
AAR89193.1      VMNTEHKAYLDKNKAYPVECWVPDPTRNENTRYFGTLTGGENVPPVLHITNTATTVLLDE 240
AAR89199.1      VMNTEHKAYLDKNKAYPVECWVPDPTRNENTRYFGTLTGGENVPPVLHITNTATTVLLDE 240
AAR89205.1      VMNTEHKAYLDKNKAYPVECWVPDPTRNENTRYFGTLTGGENVPPVLHITNTATTVLLDE 240
AAR89217.1      VMNTEHKAYLDKNKAYPVECWVPDPTRNENTRYFGTLTGGENVPPVLHITNTATTVLLDE 240
AAR89223.1      VMNTEHKAYLDKNKAYPVECWVPDPTRNENTRYFGTLTGGENVPPVLHITNTATTVLLDE 240
AAR89229.1      VMNTEHKAYLDKNKAYPVECWVPDPTRNENTRYFGTLTGGENVPPVLHITNTATTVLLDE 240
AAR89235.1      VMNTEHKAYLDKNKAYPVECWVPDPTRNENTRYFGTLTGGENVPPVLHITNTATTVLLDE 240
AAR89241.1      VMNTEHKAYLDKNKAYPVECWVPDPTRNENTRYFGTLTGGENVPPVLHITNTATTVLLDE 240
AAR89247.1      VMNTEHKAYLDKNKAYPVECWVPDPTRNENTRYFGTLTGGENVPPVLHITNTATTVLLDE 240
AAR89253.1      VMNTEHKAYLDKNKAYPVECWVPDPTRNENTRYFGTLTGGENVPPVLHITNTATTVLLDE 240
AAR32743.1      VMNTEHKAYLDKNKAYPVECWVPDPTRNENTRYFGTLTGGENVPPVLHITNTATTVLLDE 240
AAR89265.1      VMNTEHKAYLDKNKAYPVECWVPDPTRNENTRYFGTLTGGENVPPVLHITNTATTVLLDE 240
AAR89277.1      VMNTEHKAYLDKNKAYPVECWVPDPTRNENTRYFGTLTGGENVPPVLHITNTATTVLLDE 240
AAR89283.1      VMNTEHKAYLDKNKAYPVECWVPDPTRNENTRYFGTLTGGENVPPVLHITNTATTVLLDE 240
AAR02463.1      VMNTEHKAYLDKNKAYPVECWVPDPTRNENTRYFGTLTGGENVPPVLHITNTATTVLLDE 240
AAR06661.1      VMNTEHKAYLDKNKAYPVECWVPDPTRNENTRYFGTLTGGENVPPVLHITNTATTVLLDE 240
AAR13659.1      VMNTEHKAYLDKNKAYPVECWVPDPTRNENTRYFGTLTGGENVPPVLHITNTATTVLLDE 240
AAR02457.1      VMNTEHKAYLDKNKAYPVECWVPDPTRNENTRYFGTLTGGENVPPVLHITNTATTVLLDE 240
BAB68785.1      VMNTEHKAYLDKNKAYPVECWVPDPTRNENTRYFGTLTGGENVPPVLHITNTATTVLLDE 240
AAK28472.1      VMNTEHKAYLDKNKAYPVECWVPDPTRNENTRYFGTLTGGENVPPVLHITNTATTVLLDE 240
AAG34667.1      VMNTEHKAYLDKNKAYPVECWVPDPTRNENTRYFGTLTGGENVPPVLHITNTATTVLLDE 240
AAG34673.1      VMNTEHKAYLDKNKAYPVECWVPDPTRNENTRYFGTLTGGENVPPVLHITNTATTVLLDE 240
AAB41705.1      VMNTEHKAYLDKNKAYPVECWVPDPTRNENTRYFGTLTGGENVPPVLHITNTATTVLLDE 240
AAB41711.1      VMNTEHKAYLDKNKAYPVECWVPDPTRNENTRYFGTLTGGENVPPVLHITNTATTVLLDE 240
AAB41717.1      VMNTEHKAYLDKNKAYPVECWVPDPTRNENTRYFGTLTGGENVPPVLHITNTATTVLLDE 240
BAE02926.1      VMNTEHKAYLDKNKAYPVECWVPDPTRNENTRYFGTLTGGENVPPVLHITNTATTVLLDE 240
                *:. : :.   ..*.****** *:*   **  *     ** ..* **** ***:*.***:

AEC14619.1      FGVGPLCKGDNLYLSAVDVCGMFTNRSGCQQWRGLSRYFKVQLRKRRVKNPYPISFLLTD 300
AEC15199.1      FGVGPLCRGDNLYLSAVDVCGMFTNRLGSQQWRGLSRYFKVQLRKRRVKNPYPISFLLTD 300
AEC15209.1      FGVGPLCKGDNLYLSAVDVCGMFTNRLGSQQWRGLSRYFKVQLRKRRVKNPYPISFLLTD 300
UPN63098.1      FGVGPLCKGDNLYLSAVDVCGMFTNRSGSQQWRGLSRYFKVQLRKRRVKNPYPISFLLTD 300
UPN63097.1      FGVGPLCKGDNLYLSAVDVCGMFTNRSGSQQWRGLSRYFKVQLRKRRVKNPYPISFLLTD 300
UPN63094.1      FGVGPLCKGDNLYLSAVDVCGMFTNRSGSQQWRGLSRYFKVQLRKRRVKNPYPISFLLTD 300
UPN63096.1      FGVGPLCKGDNLYLSAVDVCGMFTNRSGSQQWRGLSRYFKVQLRKRRVKNPYPISFLLTD 300
QWT77146.1      FGVGPLCKGDNLYLSAVDVCGMFTNRSGSQQWRGLSRYFKVQLRKRRVKNPYPISFLLTD 300
UPN63095.1      FGVGPLCKGDNLYLSAVDVCGMFTNRSGSQQWRGLSRYFKVQLRKRRVKNPYPISFLLTD 300
BAA01966.1      FGVGPLCKGDNLYLSAVDVCGMFTNRSGSQQWRGLSRYFKVQLRKRRVKNPYPISFLLTD 300
AEC15099.1      FGVGPLCKGDNLYLSAVDVCGMFTNRSGSQQWRGLSRYFKVQLRKRRVKNPYPISFLLTD 300
UPN63099.1      FGVGPLCKGDNLYLSAVDVCGMFTHRSGSQQWRGLSRYFKVQLRKRRVKNPYPISFLLTD 300
UPN63086.1      FGVGPLCKGDNLYLSAVDVCGMFTNRSGFQQWRGLSRYFKVQLRKRRVKNPYPISFLLTD 300
UPN63092.1      FGVGPLCKGDNLYLSAVDVCGMFTNRSGFQQWRGLSRYFKVQLRKRRVKNPYPISFLLTD 300
AEC15783.1      FGVGPLCKGDNLYLSAVDVCGMFTNRSGSQQWRGLSRYFKVQLRKRRVKNPYPISFLLTD 300
AEC15735.1      FGVGPLCKGDNLYLSAVDVCGMFTNRSGSQQWRGLSRYFKVQLRKRRVKNPYPISFLLTD 300
AEC15615.1      FGVGPLCKGDNLYLSAVDVCGMFTNRSGSQQWRGLSRYFKVQLRKRRVKNPYPISFLLTD 300
```

```
AEC15549.1        FGVGPLCKGDNLYLSAVDVCGMFTNRSGSQQWRGLSRYFKVQLRKRRVKNPYPISFLLTD  300
AEC15081.1        FGVGPLCKGDNLYLSAVDVCGMFTNRSGSQQWRGLSRYFKVQLRKRRVKNPYPISFLLTD  300
AEC15080.1        FGVGPLXKGDNLYLSAVDVCGMFTNRSGSQQWRGLSRYFKVQLRKRRVKNPYPISFLLTD  300
AEC15083.1        FGVGPLXKGDNLYLSAVDVCGMFTNRSGSQQWRGLSRYFKVQLRKRRVKNPYPISFLLTD  300
QWT77109.1        FGVGPLCKGDNLYLSAVDVCGMFTNRSGSQQWRGLSRYFKVQLRKRRVKNPYPISFLLTD  300
AEC15069.1        FGVGPLCKGDNLYLSAVDVCGMFTNRSGSQQWRGLSRYFKVQLRKRRVKNPYPISFLLTD  300
AEC15070.1        FGVGPLCKGDNLYLSAVDVCGMFTNRSGSQQWRGLSRYFKVQLRKRRVKNPYPISFLLTD  300
AEC15071.1        FGVGPLCKGDNLYLSAVDVCGMFTNRSGSQQWRGLSRYFKVQLRKRRVKNPYPISFLLTD  300
AEC15072.1        FGVGPLCKGDNLYLSAVDVCGMFTNRSGSQQWRGLSRYFKVQLRKRRVKNPYPISFLLTD  300
AEC15073.1        FGVGPLCKGDNLYLSAVDVCGMFTNRSGSQQWRGLSRYFKVQLRKRRVKNPYPISFLLTD  300
AEC15074.1        FGVGPLCKGDNLYLSAVDVCGMFTNRSGSQQWRGLSRYFKVQLRKRRVKNPYPISFLLTD  300
AEC15076.1        FGVGPLCKGDNLYLSAVDVCGMFTNRSGSQQWRGLSRYFKVQLRKRRVKNPYPISFLLTD  300
AEC15077.1        FGVGPLCKGDNLYLSAVDVCGMFTNRSGSQQWRGLSRYFKVQLRKRRVKNPYPISFLLTD  300
AEC15078.1        FGVGPLCKGDNLYLSAVDVCGMFTNRSGSQQWRGLSRYFKVQLRKRRVKNPYPISFLLTD  300
AEC15079.1        FGVGPLCKGDNLYLSAVDVCGMFTNRSGSQQWRGLSRYFKVQLRKRRVKNPYPISFLLTD  300
AEC15082.1        FGVGPLCKGDNLYLSAVDVCGMFTNRSGSQQWRGLSRYFKVQLRKRRVKNPYPISFLLTD  300
AEC15084.1        FGVGPLCKGDNLYLSAVDVCGMFTNRSGSQQWRGLSRYFKVQLRKRRVKNPYPISFLLTD  300
AEC15085.1        FGVGPLCKGDNLYLSAVDVCGMFTNRSGSQQWRGLSRYFKVQLRKRRVKNPYPISFLLTD  300
AEC15086.1        FGVGPLCKGDNLYLSAVDVCGMFTNRSGSQQWRGLSRYFKVQLRKRRVKNPYPISFLLTD  300
AEC15087.1        FGVGPLCKGDNLYLSAVDVCGMFTNRSGSQQWRGLSRYFKVQLRKRRVKNPYPISFLLTD  300
AEC15089.1        FGVGPLCKGDNLYLSAVDVCGMFTNRSGSQQWRGLSRYFKVQLRKRRVKNPYPISFLLTD  300
AEC15555.1        FGVGPLCKGDNLYLSAVDVCGMFTNRSGSQQWRGLSRYFKVQLRKRRVKNPYPISFLLTD  300
AEC15561.1        FGVGPLCKGDNLYLSAVDVCGMFTNRSGSQQWRGLSRYFKVQLRKRRVKNPYPISFLLTD  300
AEC15567.1        FGVGPLCKGDNLYLSAVDVCGMFTNRSGSQQWRGLSRYFKVQLRKRRVKNPYPISFLLTD  300
AEC15573.1        FGVGPLCKGDNLYLSAVDVCGMFTNRSGSQQWRGLSRYFKVQLRKRRVKNPYPISFLLTD  300
AEC15579.1        FGVGPLCKGDNLYLSAVDVCGMFTNRSGSQQWRGLSRYFKVQLRKRRVKNPYPISFLLTD  300
AEC15585.1        FGVGPLCKGDNLYLSAVDVCGMFTNRSGSQQWRGLSRYFKVQLRKRRVKNPYPISFLLTD  300
AEC15591.1        FGVGPLCKGDNLYLSAVDVCGMFTNRSGSQQWRGLSRYFKVQLRKRRVKNPYPISFLLTD  300
AEC15597.1        FGVGPLCKGDNLYLSAVDVCGMFTNRSGSQQWRGLSRYFKVQLRKRRVKNPYPISFLLTD  300
AEC15603.1        FGVGPLCKGDNLYLSAVDVCGMFTNRSGSQQWRGLSRYFKVQLRKRRVKNPYPISFLLTD  300
AEC15609.1        FGVGPLCKGDNLYLSAVDVCGMFTNRSGSQQWRGLSRYFKVQLRKRRVKNPYPISFLLTD  300
AEC15621.1        FGVGPLCKGDNLYLSAVDVCGMFTNRSGSQQWRGLSRYFKVQLRKRRVKNPYPISFLLTD  300
AEC15627.1        FGVGPLCKGDNLYLSAVDVCGMFTNRSGSQQWRGLSRYFKVQLRKRRVKNPYPISFLLTD  300
AEC15633.1        FGVGPLCKGDNLYLSAVDVCGMFTNRSGSQQWRGLSRYFKVQLRKRRVKNPYPISFLLTD  300
AEC15639.1        FGVGPLCKGDNLYLSAVDVCGMFTNRSGSQQWRGLSRYFKVQLRKRRVKNPYPISFLLTD  300
AEC15645.1        FGVGPLCKGDNLYLSAVDVCGMFTNRSGSQQWRGLSRYFKVQLRKRRVKNPYPISFLLTD  300
AEC15651.1        FGVGPLCKGDNLYLSAVDVCGMFTNRSGSQQWRGLSRYFKVQLRKRRVKNPYPISFLLTD  300
AEC15657.1        FGVGPLCKGDNLYLSAVDVCGMFTNRSGSQQWRGLSRYFKVQLRKRRVKNPYPISFLLTD  300
AEC15663.1        FGVGPLCKGDNLYLSAVDVCGMFTNRSGSQQWRGLSRYFKVQLRKRRVKNPYPISFLLTD  300
AEC15669.1        FGVGPLCKGDNLYLSAVDVCGMFTNRSGSQQWRGLSRYFKVQLRKRRVKNPYPISFLLTD  300
AEC15675.1        FGVGPLCKGDNLYLSAVDVCGMFTNRSGSQQWRGLSRYFKVQLRKRRVKNPYPISFLLTD  300
AEC15681.1        FGVGPLCKGDNLYLSAVDVCGMFTNRSGSQQWRGLSRYFKVQLRKRRVKNPYPISFLLTD  300
AEC15687.1        FGVGPLCKGDNLYLSAVDVCGMFTNRSGSQQWRGLSRYFKVQLRKRRVKNPYPISFLLTD  300
AEC15693.1        FGVGPLCKGDNLYLSAVDVCGMFTNRSGSQQWRGLSRYFKVQLRKRRVKNPYPISFLLTD  300
AEC15699.1        FGVGPLCKGDNLYLSAVDVCGMFTNRSGSQQWRGLSRYFKVQLRKRRVKNPYPISFLLTD  300
AEC15705.1        FGVGPLCKGDNLYLSAVDVCGMFTNRSGSQQWRGLSRYFKVQLRKRRVKNPYPISFLLTD  300
AEC15711.1        FGVGPLCKGDNLYLSAVDVCGMFTNRSGSQQWRGLSRYFKVQLRKRRVKNPYPISFLLTD  300
AEC15717.1        FGVGPLCKGDNLYLSAVDVCGMFTNRSGSQQWRGLSRYFKVQLRKRRVKNPYPISFLLTD  300
AEC15723.1        FGVGPLCKGDNLYLSAVDVCGMFTNRSGSQQWRGLSRYFKVQLRKRRVKNPYPISFLLTD  300
AEC15729.1        FGVGPLCKGDNLYLSAVDVCGMFTNRSGSQQWRGLSRYFKVQLRKRRVKNPYPISFLLTD  300
AEC15741.1        FGVGPLCKGDNLYLSAVDVCGMFTNRSGSQQWRGLSRYFKVQLRKRRVKNPYPISFLLTD  300
AEC15747.1        FGVGPLCKGDNLYLSAVDVCGMFTNRSGSQQWRGLSRYFKVQLRKRRVKNPYPISFLLTD  300
AEC15753.1        FGVGPLCKGDNLYLSAVDVCGMFTNRSGSQQWRGLSRYFKVQLRKRRVKNPYPISFLLTD  300
AEC15759.1        FGVGPLCKGDNLYLSAVDVCGMFTNRSGSQQWRGLSRYFKVQLRKRRVKNPYPISFLLTD  300
AEC15765.1        FGVGPLCKGDNLYLSAVDVCGMFTNRSGSQQWRGLSRYFKVQLRKRRVKNPYPISFLLTD  300
AEC15771.1        FGVGPLCKGDNLYLSAVDVCGMFTNRSGSQQWRGLSRYFKVQLRKRRVKNPYPISFLLTD  300
AEC15777.1        FGVGPLCKGDNLYLSAVDVCGMFTNRSGSQQWRGLSRYFKVQLRKRRVKNPYPISFLLTD  300
AEC15789.1        FGVGPLCKGDNLYLSAVDVCGMFTNRSGSQQWRGLSRYFKVQLRKRRVKNPYPISFLLTD  300
AEC15795.1        FGVGPLCKGDNLYLSAVDVCGMFTNRSGSQQWRGLSRYFKVQLRKRRVKNPYPISFLLTD  300
AEC15801.1        FGVGPLCKGDNLYLSAVDVCGMFTNRSGSQQWRGLSRYFKVQLRKRRVKNPYPISFLLTD  300
AEC15807.1        FGVGPLCKGDNLYLSAVDVCGMFTNRSGSQQWRGLSRYFKVQLRKRRVKNPYPISFLLTD  300
AEC15813.1        FGVGPLCKGDNLYLSAVDVCGMFTNRSGSQQWRGLSRYFKVQLRKRRVKNPYPISFLLTD  300
AEC15075.1        FGVGPLCKGDNLYLSAVDVCGMFTNRSGSQQWRGLSRYFKVQLRKRRAKNPYPISFLLTD  300
AEC15088.1        FGVGPLCKGDNLYLSAVDVCGMFTNRSGSQQWRGLSRYFKVQLRKRRAKNPYPISFLLTD  300
```

AEC14559.1	FGVGPLCKGDNLYLSAVDVCGMFTNRSGSQQWRGLSRYFKVQLRKRRVKNPYPISFLLTD	300
AEC15023.1	FGVGPLCKGDNLYLSAVDVCGMFTSRSGSQQWRGLSRYFKVQLRKRRVKNPYPISFLLTD	300
AEC14518.1	FGVGPLCKGDNLYLSAVDVCGMFTSRSGSQQWRGLSRYFKVQLRKRRVKNPYPISFLLTD	300
AEC14589.1	FGVGPLCKGDNLYLSAVDVCGMFTSRSGSQQWRGLSRYFKVQLRKRRVKNPYPISFLLTD	300
AEC14625.1	FGVGPLCKGDNLYLSAVDVCGMFTSRSGSQQWRGLSRYFKVQLRKRRVKNPYPISFLLTD	300
AEC14631.1	FGVGPLCKGDNLYLSAVDVCGMFTSRSGSQQWRGLSRYFKVQLRKRRVKNPYPISFLLTD	300
AEC14643.1	FGVGPLCKGDNLYLSAVDVCGMFTSRSGSQQWRGLSRYFKVQLRKRRVKNPYPISFLLTD	300
AEC14966.1	FGVGPLCKGDNLYLSAVDVCGMFTSRSGSQQWRGLSRYFKVQLRKRRVKNPYPISFLLTD	300
AEC14979.1	FGVGPLCKGDNLYLSAVDVCGMFTSRSGSQQWRGLSRYFKVQLRKRRVKNPYPISFLLTD	300
AEC14990.1	FGVGPLCKGDNLYLSAVDVCGMFTSRSGSQQWRGLSRYFKVQLRKRRVKNPYPISFLLTD	300
AEC14999.1	FGVGPLCKGDNLYLSAVDVCGMFTSRSGSQQWRGLSRYFKVQLRKRRVKNPYPISFLLTD	300
AEC15020.1	FGVGPLCKGDNLYLSAVDVCGMFTSRSGSQQWRGLSRYFKVQLRKRRVKNPYPISFLLTD	300
AEC14595.1	FGVGPLCKGDNLYLSAVDVCGMFTXRSGSQQWRGLSRYFKVQLRKRRVKNPYPISFLLTD	300
AEC14703.1	FGVGPLCKGDNLYLSAVDVCGMFTHRSGSQQWRGLSRYFKVQLRKRRVKNPYPISFLLTD	300
AEC15011.1	FGVGPLCKGDNLYLSAVDVCGMFTNRSGSQQWRGLSRYFKVQLRKRRVKNPYPISFLLTD	300
AEC15019.1	FGVGPLCKGDNLYLSAVDVCGMFTNRSGSQQWRGLSRYFKVQLRKRRVKNPYPISFLLTD	300
AEC15014.1	FGVGPLCKGDNLYLSAVDVCGMFTNRFGSQQWRGLSRYFKVQLRKRRVKNPYPISFLLTD	300
NP_043511.1	FGVGPLCKGDNLYLSAVDVCGMFTNRSGSQQWRGLSRYFKVQLRKRRVKNPYPISFLLTD	300
UPN63087.1	FGVGPLCKGDNLYLSAVDVCGMFTNRSGSQQWRGLSRYFKVQLRKRRVKNPYPISFLLTD	300
ASV51760.1	FGVGPLCKGDNLYLSAVDVCGMFTNRSGSQQWRGLSRYFKVQLRKRRVKNPYPISFLLTD	300
BAV69379.1	FGVGPLCKGDNLYLSAVDVCGMFTNRSGSQQWRGLSRYFKVQLRKRRVKNPYPISFLLTD	300
AEC15090.1	FGVGPLCKGDNLYLSAVDVCGMFTNRSGSQQWRGLSRYFKVQLRKRRVKNPYPISFLLTD	300
AEC15091.1	FGVGPLCKGDNLYLSAVDVCGMFTNRSGSQQWRGLSRYFKVQLRKRRVKNPYPISFLLTD	300
AEC15092.1	FGVGPLCKGDNLYLSAVDVCGMFTNRSGSQQWRGLSRYFKVQLRKRRVKNPYPISFLLTD	300
AEC15093.1	FGVGPLCKGDNLYLSAVDVCGMFTNRSGSQQWRGLSRYFKVQLRKRRVKNPYPISFLLTD	300
AEC15094.1	FGVGPLCKGDNLYLSAVDVCGMFTNRSGSQQWRGLSRYFKVQLRKRRVKNPYPISFLLTD	300
AEC15095.1	FGVGPLCKGDNLYLSAVDVCGMFTNRSGSQQWRGLSRYFKVQLRKRRVKNPYPISFLLTD	300
AEC15096.1	FGVGPLCKGDNLYLSAVDVCGMFTNRSGSQQWRGLSRYFKVQLRKRRVKNPYPISFLLTD	300
AEC15097.1	FGVGPLCKGDNLYLSAVDVCGMFTNRSGSQQWRGLSRYFKVQLRKRRVKNPYPISFLLTD	300
AEC15098.1	FGVGPLCKGDNLYLSAVDVCGMFTNRSGSQQWRGLSRYFKVQLRKRRVKNPYPISFLLTD	300
AEC15100.1	FGVGPLCKGDNLYLSAVDVCGMFTNRSGSQQWRGLSRYFKVQLRKRRVKNPYPISFLLTD	300
AEC15101.1	FGVGPLCKGDNLYLSAVDVCGMFTNRSGSQQWRGLSRYFKVQLRKRRVKNPYPISFLLTD	300
AEC15102.1	FGVGPLCKGDNLYLSAVDVCGMFTNRSGSQQWRGLSRYFKVQLRKRRVKNPYPISFLLTD	300
AEC15103.1	FGVGPLCKGDNLYLSAVDVCGMFTNRSGSQQWRGLSRYFKVQLRKRRVKNPYPISFLLTD	300
AEC15104.1	FGVGPLCKGDNLYLSAVDVCGMFTNRSGSQQWRGLSRYFKVQLRKRRVKNPYPISFLLTD	300
AEC15105.1	FGVGPLCKGDNLYLSAVDVCGMFTNRSGSQQWRGLSRYFKVQLRKRRVKNPYPISFLLTD	300
AEC15106.1	FGVGPLCKGDNLYLSAVDVCGMFTNRSGSQQWRGLSRYFKVQLRKRRVKNPYPISFLLTD	300
AEC15107.1	FGVGPLCKGDNLYLSAVDVCGMFTNRSGSQQWRGLSRYFKVQLRKRRVKNPYPISFLLTD	300
AEC15108.1	FGVGPLCKGDNLYLSAVDVCGMFTNRSGSQQWRGLSRYFKVQLRKRRVKNPYPISFLLTD	300
AEC15109.1	FGVGPLCKGDNLYLSAVDVCGMFTNRSGSQQWRGLSRYFKVQLRKRRVKNPYPISFLLTD	300
AEC15110.1	FGVGPLCKGDNLYLSAVDVCGMFTNRSGSQQWRGLSRYFKVQLRKRRVKNPYPISFLLTD	300
AEC15111.1	FGVGPLCKGDNLYLSAVDVCGMFTNRSGSQQWRGLSRYFKVQLRKRRVKNPYPISFLLTD	300
AEC15963.1	FGVGPLCKGDNLYLSAVDVCGMFTNRSGSQQWRGLSRYFKVQLRKRRVKNPYPISFLLTD	300
AEC15969.1	FGVGPLCKGDNLYLSAVDVCGMFTNRSGSQQWRGLSRYFKVQLRKRRVKNPYPISFLLTD	300
AEC15975.1	FGVGPLCKGDNLYLSAVDVCGMFTNRSGSQQWRGLSRYFKVQLRKRRVKNPYPISFLLTD	300
AEC15981.1	FGVGPLCKGDNLYLSAVDVCGMFTNRSGSQQWRGLSRYFKVQLRKRRVKNPYPISFLLTD	300
AEC15987.1	FGVGPLCKGDNLYLSAVDVCGMFTNRSGSQQWRGLSRYFKVQLRKRRVKNPYPISFLLTD	300
AEC15993.1	FGVGPLCKGDNLYLSAVDVCGMFTNRSGSQQWRGLSRYFKVQLRKRRVKNPYPISFLLTD	300
CAD69038.1	FGVGPLCKGDNLYLSAVDVCGMFTNRSGSQQWRGLSRYFKVQLRKRRVKNPYPISFLLTD	300
AAK70240.1	FGVGPLCKGDNLYLSAVDVCGMFTNRSGSQQWRGLSRYFKVQLRKRRVKNPYPISFLLTD	300
BAB11710.1	FGVGPLCKGDNLYLSAVDVCGMFTNRSGSQQWRGLSRYFKVQLRKRRVKNPYPISFLLTD	300
BAA01958.1	FGVGPLCKGDNLYLSAVDVCGMFTNRSGSQQWRGLSRYFKVQLRKRRVKNPYPISFLLTD	300
AAA82101.1	FGVGPLCKGDNLYLSAVDVCGMFTNRSGSQQWRGLSRYFKVQLRKRRVKNPYPISFLLTD	300
P03089.1	FGVGPLCKGDNLYLSAVDVCGMFTNRSGSQQWRGLSRYFKVQLRKRRVKNPYPISFLLTD	300
UPN63102.1	FGVGPLCKGDNLYLSAVDVCGMFTNRLGSQQWRGLSRYFKVQLRKRRVKNPYPISFLLTD	300
AEC15025.1	FGVGPLCKGDNLYLSAVDVCGMFTNRLGSQQWRGLSRYFKVQLRKRRVKNPYPISFLLTD	300
AEC15026.1	FGVGPLCKGDNLYLSAVDVCGMFTNRLGSQQWRGLSRYFKVQLRKRRVKNPYPISFLLTD	300
AEC15027.1	FGVGPLCKGDNLYLSAVDVCGMFTNRLGSQQWRGLSRYFKVQLRKRRVKNPYPISFLLTD	300
AEC15028.1	FGVGPLCKGDNLYLSAVDVCGMFTNRLGSQQWRGLSRYFKVQLRKRRVKNPYPISFLLTD	300
AEC15029.1	FGVGPLCKGDNLYLSAVDVCGMFTNRLGSQQWRGLSRYFKVQLRKRRVKNPYPISFLLTD	300
AEC15196.1	FGVGPLCKGDNLYLSAVDVCGMFTNRLGSQQWRGLSRYFKVQLRKRRVKNPYPISFLLTD	300
AEC15197.1	FGVGPLCKGDNLYLSAVDVCGMFTNRLGSQQWRGLSRYFKVQLRKRRVKNPYPISFLLTD	300
AEC15198.1	FGVGPLCKGDNLYLSAVDVCGMFTNRLGSQQWRGLSRYFKVQLRKRRVKNPYPISFLLTD	300
AEC15200.1	FGVGPLCKGDNLYLSAVDVCGMFTNRLGSQQWRGLSRYFKVQLRKRRVKNPYPISFLLTD	300

AEC15201.1	FGVGPLCKGDNLYLSAVDVCGMFTNRLGSQQWRGLSRYFKVQLRKRRVKNPYPISFLLTD	300
AEC15202.1	FGVGPLCKGDNLYLSAVDVCGMFTNRLGSQQWRGLSRYFKVQLRKRRVKNPYPISFLLTD	300
AEC15203.1	FGVGPLCKGDNLYLSAVDVCGMFTNRLGSQQWRGLSRYFKVQLRKRRVKNPYPISFLLTD	300
AEC15204.1	FGVGPLCKGDNLYLSAVDVCGMFTNRLGSQQWRGLSRYFKVQLRKRRVKNPYPISFLLTD	300
AEC15205.1	FGVGPLCKGDNLYLSAVDVCGMFTNRLGSQQWRGLSRYFKVQLRKRRVKNPYPISFLLTD	300
AEC15206.1	FGVGPLCKGDNLYLSAVDVCGMFTNRLGSQQWRGLSRYFKVQLRKRRVKNPYPISFLLTD	300
AEC15207.1	FGVGPLCKGDNLYLSAVDVCGMFTNRLGSQQWRGLSRYFKVQLRKRRVKNPYPISFLLTD	300
AEC15208.1	FGVGPLCKGDNLYLSAVDVCGMFTNRLGSQQWRGLSRYFKVQLRKRRVKNPYPISFLLTD	300
AEC15210.1	FGVGPLCKGDNLYLSAVDVCGMFTNRLGSQQWRGLSRYFKVQLRKRRVKNPYPISFLLTD	300
AEC15211.1	FGVGPLCKGDNLYLSAVDVCGMFTNRLGSQQWRGLSRYFKVQLRKRRVKNPYPISFLLTD	300
AEC15212.1	FGVGPLCKGDNLYLSAVDVCGMFTNRLGSQQWRGLSRYFKVQLRKRRVKNPYPISFLLTD	300
AEC15213.1	FGVGPLCKGDNLYLSAVDVCGMFTNRLGSQQWRGLSRYFKVQLRKRRVKNPYPISFLLTD	300
AEC15214.1	FGVGPLCKGDNLYLSAVDVCGMFTNRLGSQQWRGLSRYFKVQLRKRRVKNPYPISFLLTD	300
AEC15215.1	FGVGPLCKGDNLYLSAVDVCGMFTNRLGSQQWRGLSRYFKVQLRKRRVKNPYPISFLLTD	300
AEC15216.1	FGVGPLCKGDNLYLSAVDVCGMFTNRLGSQQWRGLSRYFKVQLRKRRVKNPYPISFLLTD	300
AEC15217.1	FGVGPLCKGDNLYLSAVDVCGMFTNRLGSQQWRGLSRYFKVQLRKRRVKNPYPISFLLTD	300
AEC15218.1	FGVGPLCKGDNLYLSAVDVCGMFTNRLGSQQWRGLSRYFKVQLRKRRVKNPYPISFLLTD	300
AEC15327.1	FGVGPLCKGDNLYLSAVDVCGMFTNRLGSQQWRGLSRYFKVQLRKRRVKNPYPISFLLTD	300
AEC15333.1	FGVGPLCKGDNLYLSAVDVCGMFTNRLGSQQWRGLSRYFKVQLRKRRVKNPYPISFLLTD	300
AEC15339.1	FGVGPLCKGDNLYLSAVDVCGMFTNRLGSQQWRGLSRYFKVQLRKRRVKNPYPISFLLTD	300
AEC15345.1	FGVGPLCKGDNLYLSAVDVCGMFTNRLGSQQWRGLSRYFKVQLRKRRVKNPYPISFLLTD	300
AEC15351.1	FGVGPLCKGDNLYLSAVDVCGMFTNRLGSQQWRGLSRYFKVQLRKRRVKNPYPISFLLTD	300
AEC15357.1	FGVGPLCKGDNLYLSAVDVCGMFTNRLGSQQWRGLSRYFKVQLRKRRVKNPYPISFLLTD	300
AEC15363.1	FGVGPLCKGDNLYLSAVDVCGMFTNRLGSQQWRGLSRYFKVQLRKRRVKNPYPISFLLTD	300
AEC15369.1	FGVGPLCKGDNLYLSAVDVCGMFTNRLGSQQWRGLSRYFKVQLRKRRVKNPYPISFLLTD	300
AEC15375.1	FGVGPLCKGDNLYLSAVDVCGMFTNRLGSQQWRGLSRYFKVQLRKRRVKNPYPISFLLTD	300
AEC15381.1	FGVGPLCKGDNLYLSAVDVCGMFTNRLGSQQWRGLSRYFKVQLRKRRVKNPYPISFLLTD	300
AEC15387.1	FGVGPLCKGDNLYLSAVDVCGMFTNRLGSQQWRGLSRYFKVQLRKRRVKNPYPISFLLTD	300
AEC15393.1	FGVGPLCKGDNLYLSAVDVCGMFTNRLGSQQWRGLSRYFKVQLRKRRVKNPYPISFLLTD	300
AEC15399.1	FGVGPLCKGDNLYLSAVDVCGMFTNRLGSQQWRGLSRYFKVQLRKRRVKNPYPISFLLTD	300
AEC15405.1	FGVGPLCKGDNLYLSAVDVCGMFTNRLGSQQWRGLSRYFKVQLRKRRVKNPYPISFLLTD	300
AEC15411.1	FGVGPLCKGDNLYLSAVDVCGMFTNRLGSQQWRGLSRYFKVQLRKRRVKNPYPISFLLTD	300
AEC15417.1	FGVGPLCKGDNLYLSAVDVCGMFTNRLGSQQWRGLSRYFKVQLRKRRVKNPYPISFLLTD	300
AEC15423.1	FGVGPLCKGDNLYLSAVDVCGMFTNRLGSQQWRGLSRYFKVQLRKRRVKNPYPISFLLTD	300
AEC15429.1	FGVGPLCKGDNLYLSAVDVCGMFTNRLGSQQWRGLSRYFKVQLRKRRVKNPYPISFLLTD	300
AEC15435.1	FGVGPLCKGDNLYLSAVDVCGMFTNRLGSQQWRGLSRYFKVQLRKRRVKNPYPISFLLTD	300
AEC15441.1	FGVGPLCKGDNLYLSAVDVCGMFTNRLGSQQWRGLSRYFKVQLRKRRVKNPYPISFLLTD	300
BAA01967.1	FGVGPLCKGDNLYLSAVDVCGMFTNRLGSQQWRGLSRYFKVQLRKRRVKNPYPISFLLTD	300
ASV51844.1	FGVGPLCKGDNLYLSAVDVCGMFTNRSGSQQWRGLSRYFKVQLRKRRVKNPYPISFLLTD	300
ASV51850.1	FGVGPLCKGDNLYLSAVDVCGMFTNRSGSQQWRGLSRYFKVQLRKRRVKNPYPISFLLTD	300
CCW72489.1	FGVGPLCKGDNLYLSAVDVCGMFTNRSGSQQWRGLSRYFKVQLRKRRVKNPYPISFLLTD	300
AEC15170.1	FGVGPLCKGDNLYLSAVDVCGMFTNRSGFQQWRGLSRYFKVQLRKRRVKNPYPISFLLTD	300
AEC15171.1	FGVGPLCKGDNLYLSAVDVCGMFTNRSGFQQWRGLSRYFKVQLRKRRVKNPYPISFLLTD	300
AEC15299.1	FGVGPLCKGDNLYLSAVDVCGMFTNRSGSQQWRGLSRYFKVQLRKRRVKNPYPISFLLTD	300
BAB93050.1	FGVGPLCKGDNLYLSAVDVCGMFTNRSGSQQWRGLSRYFKVQLRKRRVKNPYPISFLLTD	300
UPN63100.1	FGVGPLCKGDNLYLSAVDVCGMFTNRSGSQQWRGLSRYFKVQLRKRRVKNPYPISFLLTD	300
CCW72482.1	FGVGPLCKGDNLYLSAVDVCGMFTNRSGSQQWRGLSRYFKVQLRKRRVKNPYPISFLLTD	300
AEC15165.1	FGVGPLCKGDNLYLSAVDVCGMFTNRSGSQQWRGLSRYFKVQLRKRRVKNPYPISFLLTD	300
AEC15166.1	FGVGPLCKGDNLYLSAVDVCGMFTNRSGSQQWRGLSRYFKVQLRKRRVKNPYPISFLLTD	300
AEC15266.1	FGVGPLCKGDNLYLSAVDVCGMFTNRSGSQQWRGLSRYFKVQLRKRRVKNPYPISFLLTD	300
AEC15267.1	FGVGPLCKGDNLYLSAVDVCGMFTNRSGSQQWRGLSRYFKVQLRKRRVKNPYPISFLLTD	300
AEC15268.1	FGVGPLCKGDNLYLSAVDVCGMFTNRSGSQQWRGLSRYFKVQLRKRRVKNPYPISFLLTD	300
AEC15269.1	FGVGPLCKGDNLYLSAVDVCGMFTNRSGSQQWRGLSRYFKVQLRKRRVKNPYPISFLLTD	300
AEC15270.1	FGVGPLCKGDNLYLSAVDVCGMFTNRSGSQQWRGLSRYFKVQLRKRRVKNPYPISFLLTD	300
AEC15271.1	FGVGPLCKGDNLYLSAVDVCGMFTNRSGSQQWRGLSRYFKVQLRKRRVKNPYPISFLLTD	300
AEC15272.1	FGVGPLCKGDNLYLSAVDVCGMFTNRSGSQQWRGLSRYFKVQLRKRRVKNPYPISFLLTD	300
AEC15273.1	FGVGPLCKGDNLYLSAVDVCGMFTNRSGSQQWRGLSRYFKVQLRKRRVKNPYPISFLLTD	300
AEC15274.1	FGVGPLCKGDNLYLSAVDVCGMFTNRSGSQQWRGLSRYFKVQLRKRRVKNPYPISFLLTD	300
AEC15275.1	FGVGPLCKGDNLYLSAVDVCGMFTNRSGSQQWRGLSRYFKVQLRKRRVKNPYPISFLLTD	300
AEC15276.1	FGVGPLCKGDNLYLSAVDVCGMFTNRSGSQQWRGLSRYFKVQLRKRRVKNPYPISFLLTD	300
AEC15277.1	FGVGPLCKGDNLYLSAVDVCGMFTNRSGSQQWRGLSRYFKVQLRKRRVKNPYPISFLLTD	300
AEC15278.1	FGVGPLCKGDNLYLSAVDVCGMFTNRSGSQQWRGLSRYFKVQLRKRRVKNPYPISFLLTD	300
AEC15279.1	FGVGPLCKGDNLYLSAVDVCGMFTNRSGSQQWRGLSRYFKVQLRKRRVKNPYPISFLLTD	300
AEC15280.1	FGVGPLCKGDNLYLSAVDVCGMFTNRSGSQQWRGLSRYFKVQLRKRRVKNPYPISFLLTD	300

AEC15281.1	FGVGPLCKGDNLYLSAVDVCGMFTNRSGSQQWRGLSRYFKVQLRKRRVKNPYPISFLLTD	300
AEC15282.1	FGVGPLCKGDNLYLSAVDVCGMFTNRSGSQQWRGLSRYFKVQLRKRRVKNPYPISFLLTD	300
AEC15283.1	FGVGPLCKGDNLYLSAVDVCGMFTNRSGSQQWRGLSRYFKVQLRKRRVKNPYPISFLLTD	300
AEC15284.1	FGVGPLCKGDNLYLSAVDVCGMFTNRSGSQQWRGLSRYFKVQLRKRRVKNPYPISFLLTD	300
AEC15285.1	FGVGPLCKGDNLYLSAVDVCGMFTNRSGSQQWRGLSRYFKVQLRKRRVKNPYPISFLLTD	300
AEC15286.1	FGVGPLCKGDNLYLSAVDVCGMFTNRSGSQQWRGLSRYFKVQLRKRRVKNPYPISFLLTD	300
AEC15287.1	FGVGPLCKGDNLYLSAVDVCGMFTNRSGSQQWRGLSRYFKVQLRKRRVKNPYPISFLLTD	300
AEC15288.1	FGVGPLCKGDNLYLSAVDVCGMFTNRSGSQQWRGLSRYFKVQLRKRRVKNPYPISFLLTD	300
AEC15289.1	FGVGPLCKGDNLYLSAVDVCGMFTNRSGSQQWRGLSRYFKVQLRKRRVKNPYPISFLLTD	300
AEC15290.1	FGVGPLCKGDNLYLSAVDVCGMFTNRSGSQQWRGLSRYFKVQLRKRRVKNPYPISFLLTD	300
AEC15291.1	FGVGPLCKGDNLYLSAVDVCGMFTNRSGSQQWRGLSRYFKVQLRKRRVKNPYPISFLLTD	300
AEC15292.1	FGVGPLCKGDNLYLSAVDVCGMFTNRSGSQQWRGLSRYFKVQLRKRRVKNPYPISFLLTD	300
AEC15293.1	FGVGPLCKGDNLYLSAVDVCGMFTNRSGSQQWRGLSRYFKVQLRKRRVKNPYPISFLLTD	300
AEC15294.1	FGVGPLCKGDNLYLSAVDVCGMFTNRSGSQQWRGLSRYFKVQLRKRRVKNPYPISFLLTD	300
AEC15295.1	FGVGPLCKGDNLYLSAVDVCGMFTNRSGSQQWRGLSRYFKVQLRKRRVKNPYPISFLLTD	300
AEC15296.1	FGVGPLCKGDNLYLSAVDVCGMFTNRSGSQQWRGLSRYFKVQLRKRRVKNPYPISFLLTD	300
AEC15297.1	FGVGPLCKGDNLYLSAVDVCGMFTNRSGSQQWRGLSRYFKVQLRKRRVKNPYPISFLLTD	300
AEC15298.1	FGVGPLCKGDNLYLSAVDVCGMFTNRSGSQQWRGLSRYFKVQLRKRRVKNPYPISFLLTD	300
AEC15300.1	FGVGPLCKGDNLYLSAVDVCGMFTNRSGSQQWRGLSRYFKVQLRKRRVKNPYPISFLLTD	300
AEC15301.1	FGVGPLCKGDNLYLSAVDVCGMFTNRSGSQQWRGLSRYFKVQLRKRRVKNPYPISFLLTD	300
AEC15302.1	FGVGPLCKGDNLYLSAVDVCGMFTNRSGSQQWRGLSRYFKVQLRKRRVKNPYPISFLLTD	300
AEC15303.1	FGVGPLCKGDNLYLSAVDVCGMFTNRSGSQQWRGLSRYFKVQLRKRRVKNPYPISFLLTD	300
AEC15304.1	FGVGPLCKGDNLYLSAVDVCGMFTNRSGSQQWRGLSRYFKVQLRKRRVKNPYPISFLLTD	300
AEC15305.1	FGVGPLCKGDNLYLSAVDVCGMFTNRSGSQQWRGLSRYFKVQLRKRRVKNPYPISFLLTD	300
AEC15306.1	FGVGPLCKGDNLYLSAVDVCGMFTNRSGSQQWRGLSRYFKVQLRKRRVKNPYPISFLLTD	300
AEC15307.1	FGVGPLCKGDNLYLSAVDVCGMFTNRSGSQQWRGLSRYFKVQLRKRRVKNPYPISFLLTD	300
AEC15308.1	FGVGPLCKGDNLYLSAVDVCGMFTNRSGSQQWRGLSRYFKVQLRKRRVKNPYPISFLLTD	300
AEC15309.1	FGVGPLCKGDNLYLSAVDVCGMFTNRSGSQQWRGLSRYFKVQLRKRRVKNPYPISFLLTD	300
AEC15310.1	FGVGPLCKGDNLYLSAVDVCGMFTNRSGSQQWRGLSRYFKVQLRKRRVKNPYPISFLLTD	300
AEC15311.1	FGVGPLCKGDNLYLSAVDVCGMFTNRSGSQQWRGLSRYFKVQLRKRRVKNPYPISFLLTD	300
AEC15861.1	FGVGPLCKGDNLYLSAVDVCGMFTNRSGSQQWRGLSRYFKVQLRKRRVKNPYPISFLLTD	300
AEC15867.1	FGVGPLCKGDNLYLSAVDVCGMFTNRSGSQQWRGLSRYFKVQLRKRRVKNPYPISFLLTD	300
AEC15873.1	FGVGPLCKGDNLYLSAVDVCGMFTNRSGSQQWRGLSRYFKVQLRKRRVKNPYPISFLLTD	300
AEC15879.1	FGVGPLCKGDNLYLSAVDVCGMFTNRSGSQQWRGLSRYFKVQLRKRRVKNPYPISFLLTD	300
AEC15885.1	FGVGPLCKGDNLYLSAVDVCGMFTNRSGSQQWRGLSRYFKVQLRKRRVKNPYPISFLLTD	300
AEC15891.1	FGVGPLCKGDNLYLSAVDVCGMFTNRSGSQQWRGLSRYFKVQLRKRRVKNPYPISFLLTD	300
AEC15897.1	FGVGPLCKGDNLYLSAVDVCGMFTNRSGSQQWRGLSRYFKVQLRKRRVKNPYPISFLLTD	300
AEC15903.1	FGVGPLCKGDNLYLSAVDVCGMFTNRSGSQQWRGLSRYFKVQLRKRRVKNPYPISFLLTD	300
AEC15909.1	FGVGPLCKGDNLYLSAVDVCGMFTNRSGSQQWRGLSRYFKVQLRKRRVKNPYPISFLLTD	300
AEC15915.1	FGVGPLCKGDNLYLSAVDVCGMFTNRSGSQQWRGLSRYFKVQLRKRRVKNPYPISFLLTD	300
AEC15921.1	FGVGPLCKGDNLYLSAVDVCGMFTNRSGSQQWRGLSRYFKVQLRKRRVKNPYPISFLLTD	300
AEC15927.1	FGVGPLCKGDNLYLSAVDVCGMFTNRSGSQQWRGLSRYFKVQLRKRRVKNPYPISFLLTD	300
AEC15933.1	FGVGPLCKGDNLYLSAVDVCGMFTNRSGSQQWRGLSRYFKVQLRKRRVKNPYPISFLLTD	300
AEC15939.1	FGVGPLCKGDNLYLSAVDVCGMFTNRSGSQQWRGLSRYFKVQLRKRRVKNPYPISFLLTD	300
AEC15945.1	FGVGPLCKGDNLYLSAVDVCGMFTNRSGSQQWRGLSRYFKVQLRKRRVKNPYPISFLLTD	300
AEC15951.1	FGVGPLCKGDNLYLSAVDVCGMFTNRSGSQQWRGLSRYFKVQLRKRRVKNPYPISFLLTD	300
AEC15957.1	FGVGPLCKGDNLYLSAVDVCGMFTNRSGSQQWRGLSRYFKVQLRKRRVKNPYPISFLLTD	300
BAD21259.1	FGVGPLCKGDNLYLSAVDVCGMFTNRSGSQQWRGLSRYFKVQLRKRRVKNPYPISFLLTD	300
BAD21265.1	FGVGPLCKGDNLYLSAVDVCGMFTNRSGSQQWRGLSRYFKVQLRKRRVKNPYPISFLLTD	300
AAC59325.1	FGVGPLCKGDNLYLSAVDVCGMFTNRSGSQQWRGLSRYFKVQLRKRRVKNPYPISFLLTD	300
AEC14524.1	FGVGPLCKGDNLYLSAVDVCGMFTNRSGSQQWRGLSRYFKVQLRKRRVKNPYPISFLLTD	300
AEC14541.1	FGVGPLCKGDNLYLSAVDVCGMFTNRSGSQQWRGLSRYFKVQLRKRRVKNPYPISFLLTD	300
AEC14565.1	FGVGPLCKGDNLYLSAVDVCGMFTNRSGSQQWRGLSRYFKVQLRKRRVKNPYPISFLLTD	300
AEC14571.1	FGVGPLCKGDNLYLSAVDVCGMFTNRSGSQQWRGLSRYFKVQLRKRRVKNPYPISFLLTD	300
AEC14583.1	FGVGPLCKGDNLYLSAVDVCGMFTNRSGSQQWRGLSRYFKVQLRKRRVKNPYPISFLLTD	300
AEC14601.1	FGVGPLCKGDNLYLSAVDVCGMFTNRSGSQQWRGLSRYFKVQLRKRRVKNPYPISFLLTD	300
AEC14607.1	FGVGPLCKGDNLYLSAVDVCGMFTNRSGSQQWRGLSRYFKVQLRKRRVKNPYPISFLLTD	300
AEC14637.1	FGVGPLCKGDNLYLSAVDVCGMFTNRSGSQQWRGLSRYFKVQLRKRRVKNPYPISFLLTD	300
AEC14649.1	FGVGPLCKGDNLYLSAVDVCGMFTNRSGSQQWRGLSRYFKVQLRKRRVKNPYPISFLLTD	300
AEC14655.1	FGVGPLCKGDNLYLSAVDVCGMFTNRSGSQQWRGLSRYFKVQLRKRRVKNPYPISFLLTD	300
AEC14661.1	FGVGPLCKGDNLYLSAVDVCGMFTNRSGSQQWRGLSRYFKVQLRKRRVKNPYPISFLLTD	300
AEC14685.1	FGVGPLCKGDNLYLSAVDVCGMFTNRSGSQQWRGLSRYFKVQLRKRRVKNPYPISFLLTD	300
AEC14697.1	FGVGPLCKGDNLYLSAVDVCGMFTNRSGSQQWRGLSRYFKVQLRKRRVKNPYPISFLLTD	300
AEC14709.1	FGVGPLCKGDNLYLSAVDVCGMFTNRSGSQQWRGLSRYFKVQLRKRRVKNPYPISFLLTD	300

AEC14715.1	FGVGPLCKGDNLYLSAVDVCGMFTNRSGSQQWRGLSRYFKVQLRKRRVKNPYPISFLLTD	300
AEC14963.1	FGVGPLCKGDNLYLSAVDVCGMFTNRSGSQQWRGLSRYFKVQLRKRRVKNPYPISFLLTD	300
AEC14968.1	FGVGPLCKGDNLYLSAVDVCGMFTNRSGSQQWRGLSRYFKVQLRKRRVKNPYPISFLLTD	300
AEC14971.1	FGVGPLCKGDNLYLSAVDVCGMFTNRSGSQQWRGLSRYFKVQLRKRRVKNPYPISFLLTD	300
AEC14972.1	FGVGPLCKGDNLYLSAVDVCGMFTNRSGSQQWRGLSRYFKVQLRKRRVKNPYPISFLLTD	300
AEC14975.1	FGVGPLCKGDNLYLSAVDVCGMFTNRSGSQQWRGLSRYFKVQLRKRRVKNPYPISFLLTD	300
AEC14976.1	FGVGPLCKGDNLYLSAVDVCGMFTNRSGSQQWRGLSRYFKVQLRKRRVKNPYPISFLLTD	300
AEC14977.1	FGVGPLCKGDNLYLSAVDVCGMFTNRSGSQQWRGLSRYFKVQLRKRRVKNPYPISFLLTD	300
AEC14978.1	FGVGPLCKGDNLYLSAVDVCGMFTNRSGSQQWRGLSRYFKVQLRKRRVKNPYPISFLLTD	300
AEC14980.1	FGVGPLCKGDNLYLSAVDVCGMFTNRSGSQQWRGLSRYFKVQLRKRRVKNPYPISFLLTD	300
AEC14981.1	FGVGPLCKGDNLYLSAVDVCGMFTNRSGSQQWRGLSRYFKVQLRKRRVKNPYPISFLLTD	300
AEC14982.1	FGVGPLCKGDNLYLSAVDVCGMFTNRSGSQQWRGLSRYFKVQLRKRRVKNPYPISFLLTD	300
AEC14983.1	FGVGPLCKGDNLYLSAVDVCGMFTNRSGSQQWRGLSRYFKVQLRKRRVKNPYPISFLLTD	300
AEC14985.1	FGVGPLCKGDNLYLSAVDVCGMFTNRSGSQQWRGLSRYFKVQLRKRRVKNPYPISFLLTD	300
AEC14986.1	FGVGPLCKGDNLYLSAVDVCGMFTNRSGSQQWRGLSRYFKVQLRKRRVKNPYPISFLLTD	300
AEC14988.1	FGVGPLCKGDNLYLSAVDVCGMFTNRSGSQQWRGLSRYFKVQLRKRRVKNPYPISFLLTD	300
AEC14989.1	FGVGPLCKGDNLYLSAVDVCGMFTNRSGSQQWRGLSRYFKVQLRKRRVKNPYPISFLLTD	300
AEC14991.1	FGVGPLCKGDNLYLSAVDVCGMFTNRSGSQQWRGLSRYFKVQLRKRRVKNPYPISFLLTD	300
AEC14992.1	FGVGPLCKGDNLYLSAVDVCGMFTNRSGSQQWRGLSRYFKVQLRKRRVKNPYPISFLLTD	300
AEC14996.1	FGVGPLCKGDNLYLSAVDVCGMFTNRSGSQQWRGLSRYFKVQLRKRRVKNPYPISFLLTD	300
AEC14997.1	FGVGPLCKGDNLYLSAVDVCGMFTNRSGSQQWRGLSRYFKVQLRKRRVKNPYPISFLLTD	300
AEC14998.1	FGVGPLCKGDNLYLSAVDVCGMFTNRSGSQQWRGLSRYFKVQLRKRRVKNPYPISFLLTD	300
AEC15002.1	FGVGPLCKGDNLYLSAVDVCGMFTNRSGSQQWRGLSRYFKVQLRKRRVKNPYPISFLLTD	300
AEC15003.1	FGVGPLCKGDNLYLSAVDVCGMFTNRSGSQQWRGLSRYFKVQLRKRRVKNPYPISFLLTD	300
AEC15004.1	FGVGPLCKGDNLYLSAVDVCGMFTNRSGSQQWRGLSRYFKVQLRKRRVKNPYPISFLLTD	300
AEC15005.1	FGVGPLCKGDNLYLSAVDVCGMFTNRSGSQQWRGLSRYFKVQLRKRRVKNPYPISFLLTD	300
AEC15006.1	FGVGPLCKGDNLYLSAVDVCGMFTNRSGSQQWRGLSRYFKVQLRKRRVKNPYPISFLLTD	300
AEC15009.1	FGVGPLCKGDNLYLSAVDVCGMFTNRSGSQQWRGLSRYFKVQLRKRRVKNPYPISFLLTD	300
AEC15012.1	FGVGPLCKGDNLYLSAVDVCGMFTNRSGSQQWRGLSRYFKVQLRKRRVKNPYPISFLLTD	300
AEC15013.1	FGVGPLCKGDNLYLSAVDVCGMFTNRSGSQQWRGLSRYFKVQLRKRRVKNPYPISFLLTD	300
AEC15015.1	FGVGPLCKGDNLYLSAVDVCGMFTNRSGSQQWRGLSRYFKVQLRKRRVKNPYPISFLLTD	300
AEC15017.1	FGVGPLCKGDNLYLSAVDVCGMFTNRSGSQQWRGLSRYFKVQLRKRRVKNPYPISFLLTD	300
AEC15018.1	FGVGPLCKGDNLYLSAVDVCGMFTNRSGSQQWRGLSRYFKVQLRKRRVKNPYPISFLLTD	300
AEC15021.1	FGVGPLCKGDNLYLSAVDVCGMFTNRSGSQQWRGLSRYFKVQLRKRRVKNPYPISFLLTD	300
AEC15022.1	FGVGPLCKGDNLYLSAVDVCGMFTNRSGSQQWRGLSRYFKVQLRKRRVKNPYPISFLLTD	300
AEC14962.1	FGVGPLCKGDNLYLSAVDVCGMFTNRSGSQQWRGLSRYFKVQLRKRRVKNPYPISFLLTD	300
AEC14993.1	FGVGPLCKGDNLYLSAVDVCGMFTNRSGSQQWRGLSRYFKVQLRKRRVKNPYPISFLLTD	300
AEC15007.1	FGVGPLCKGDNLYLSAVDVCGMFTNRSGSQQWRGLSRYFKVQLRKRRVKNPYPISFLLTD	300
CCW72517.1	FGVGPLCKGDNLYLSAVDVCGMFTNRSGSQQWRGLSRYFKVQLRKRRVKNPYPISFLLTD	300
BAD21247.2	FGVGPLCKGDNLYLSAVDVCGMFTNRSGSQQWRGLSRYFKVQLRKRRVKNPYPISFLLTD	300
AAL37695.1	FGVGPLCKGDNLYLSAVDVCGMFTNRSGSQQWRGLSRYFKVQLRKRRVKNPYPISFLLTD	300
AEC15242.1	FGVGPLCKGDNLYLSAVDVCGMFTNRFGSQQWRGLSRYFKVQLRKRRVKNPYPISFLLTD	300
AEC15243.1	FGVGPLCKGDNLYLSAVDVCGMFTNRFGSQQWRGLSRYFKVQLRKRRVKNPYPISFLLTD	300
AEC15244.1	FGVGPLCKGDNLYLSAVDVCGMFTNRFGSQQWRGLSRYFKVQLRKRRVKNPYPISFLLTD	300
AEC15245.1	FGVGPLCKGDNLYLSAVDVCGMFTNRFGSQQWRGLSRYFKVQLRKRRVKNPYPISFLLTD	300
AEC15246.1	FGVGPLCKGDNLYLSAVDVCGMFTNRFGSQQWRGLSRYFKVQLRKRRVKNPYPISFLLTD	300
AEC15247.1	FGVGPLCKGDNLYLSAVDVCGMFTNRFGSQQWRGLSRYFKVQLRKRRVKNPYPISFLLTD	300
AEC15248.1	FGVGPLCKGDNLYLSAVDVCGMFTNRFGSQQWRGLSRYFKVQLRKRRVKNPYPISFLLTD	300
AEC15249.1	FGVGPLCKGDNLYLSAVDVCGMFTNRFGSQQWRGLSRYFKVQLRKRRVKNPYPISFLLTD	300
AEC15250.1	FGVGPLCKGDNLYLSAVDVCGMFTNRFGSQQWRGLSRYFKVQLRKRRVKNPYPISFLLTD	300
AEC15251.1	FGVGPLCKGDNLYLSAVDVCGMFTNRFGSQQWRGLSRYFKVQLRKRRVKNPYPISFLLTD	300
AEC15252.1	FGVGPLCKGDNLYLSAVDVCGMFTNRFGSQQWRGLSRYFKVQLRKRRVKNPYPISFLLTD	300
AEC15253.1	FGVGPLCKGDNLYLSAVDVCGMFTNRFGSQQWRGLSRYFKVQLRKRRVKNPYPISFLLTD	300
AEC15254.1	FGVGPLCKGDNLYLSAVDVCGMFTNRFGSQQWRGLSRYFKVQLRKRRVKNPYPISFLLTD	300
AEC15255.1	FGVGPLCKGDNLYLSAVDVCGMFTNRFGSQQWRGLSRYFKVQLRKRRVKNPYPISFLLTD	300
AEC15256.1	FGVGPLCKGDNLYLSAVDVCGMFTNRFGSQQWRGLSRYFKVQLRKRRVKNPYPISFLLTD	300
AEC15257.1	FGVGPLCKGDNLYLSAVDVCGMFTNRFGSQQWRGLSRYFKVQLRKRRVKNPYPISFLLTD	300
AEC15258.1	FGVGPLCKGDNLYLSAVDVCGMFTNRFGSQQWRGLSRYFKVQLRKRRVKNPYPISFLLTD	300
AEC15259.1	FGVGPLCKGDNLYLSAVDVCGMFTNRFGSQQWRGLSRYFKVQLRKRRVKNPYPISFLLTD	300
AEC15260.1	FGVGPLCKGDNLYLSAVDVCGMFTNRFGSQQWRGLSRYFKVQLRKRRVKNPYPISFLLTD	300
AEC15261.1	FGVGPLCKGDNLYLSAVDVCGMFTNRFGSQQWRGLSRYFKVQLRKRRVKNPYPISFLLTD	300
AEC15262.1	FGVGPLCKGDNLYLSAVDVCGMFTNRFGSQQWRGLSRYFKVQLRKRRVKNPYPISFLLTD	300
AEC15263.1	FGVGPLCKGDNLYLSAVDVCGMFTNRFGSQQWRGLSRYFKVQLRKRRVKNPYPISFLLTD	300
AEC15264.1	FGVGPLCKGDNLYLSAVDVCGMFTNRFGSQQWRGLSRYFKVQLRKRRVKNPYPISFLLTD	300

AEC15265.1	FGVGPLCKGDNLYLSAVDVCGMFTNRFGSQQWRGLSRYFKVQLRKRRVKNPYPISFLLTD	300
AEC15000.1	FGVGPLCKGDNLYLSAVDVCGMFTHRSGSQQWRGLSRYFKVQLRKRRVKNPYPISFLLTD	300
AEC14721.1	FGVGPLCKGDNLYLSAVDVCGMFTSRSGSQQWRGLSRYFKVQLRKRRVKNPYPISFLLTD	300
AEC14965.1	FGVGPLCKGDNLYLSAVDVCGMFTSRSGSQQWRGLSRYFKVQLRKRRVKNPYPISFLLTD	300
AEC14967.1	FGVGPLCKGDNLYLSAVDVCGMFTSRSGSQQWRGLSRYFKVQLRKRRVKNPYPISFLLTD	300
AEC14969.1	FGVGPLCKGDNLYLSAVDVCGMFTSRSGSQQWRGLSRYFKVQLRKRRVKNPYPISFLLTD	300
AEC14973.1	FGVGPLCKGDNLYLSAVDVCGMFTSRSGSQQWRGLSRYFKVQLRKRRVKNPYPISFLLTD	300
AEC14974.1	FGVGPLCKGDNLYLSAVDVCGMFTSRSGSQQWRGLSRYFKVQLRKRRVKNPYPISFLLTD	300
AEC14994.1	FGVGPLCKGDNLYLSAVDVCGMFTSRSGSQQWRGLSRYFKVQLRKRRVKNPYPISFLLTD	300
AEC15001.1	FGVGPLCKGDNLYLSAVDVCGMFTSRSGSQQWRGLSRYFKVQLRKRRVKNPYPISFLLTD	300
AEC15024.1	FGVGPLCKGDNLYLSAVDVCGMFTSRSGSQQWRGLSRYFKVQLRKRRVKNPYPISFLLTD	300
AEC14667.1	FGVGPLCKGDNLYLSAVDVCGMFTXRSGSQQWRGLSRYFKVQLRKRRVKNPYPISFLLTD	300
UPN63091.1	FGVGPLCKGDNLYLSAVDVCGMFTNRSGFQQWRGLSRYFKVQLRKRRVKNPYPISFLLTD	300
AEC14535.1	FGVGPLCKGDNLYLSAVDVCGMFTNRSGYQQWRGLSRYFKVQLRKRRVKNPYPISFLLTD	300
AEC14547.1	FGVGPLCKGDNLYLSAVDVCGMFTNRSGSQQWRGLSRYFKVQLRKRRVKNPYPISFLLTD	300
AEC14553.1	FGVGPLCKGDNLYLSAVDVCGMFTNRSGSQQWRGLSRYFKVQLRKRRVKNPYPISFLLTD	300
AEC14577.1	FGVGPLCKGDNLYLSAVDVCGMFTNRSGSQQWRGLSRYFKVQLRKRRVKNPYPISFLLTD	300
AEC14613.1	FGVGPLCKGDNLYLSAVDVCGMFTNRSGSQQWRGLSRYFKVQLRKRRVKNPYPISFLLTD	300
AEC14673.1	FGVGPLCKGDNLYLSAVDVCGMFTNRSGSQQWRGLSRYFKVQLRKRRVKNPYPISFLLTD	300
AEC14679.1	FGVGPLCKGDNLYLSAVDVCGMFTNRSGSQQWRGLSRYFKVQLRKRRVKNPYPISFLLTD	300
AEC14727.1	FGVGPLCKGDNLYLSAVDVCGMFTNRSGSQQWRGLSRYFKVQLRKRRVKNPYPISFLLTD	300
AEC14733.1	FGVGPLCKGDNLYLSAVDVCGMFTNRSGSQQWRGLSRYFKVQLRKRRVKNPYPISFLLTD	300
AEC14964.1	FGVGPLCKGDNLYLSAVDVCGMFTNRSGSQQWRGLSRYFKVQLRKRRVKNPYPISFLLTD	300
AEC14995.1	FGVGPLCKGDNLYLSAVDVCGMFTNRSGSQQWRGLSRYFKVQLRKRRVKNPYPISFLLTD	300
AEC15008.1	FGVGPLCKGDNLYLSAVDVCGMFTNRSGSQQWRGLSRYFKVQLRKRRVKNPYPISFLLTD	300
AEC14970.1	FGVGPLCKGDNLYLSAVDVCGMFTNRSGCQQWRGLSRYFKVQLRKRRVKNPYPISFLLTD	300
QTJ15162.1	FGVGPLCKGDNLYLSAVDVCGMFTNRSGSQQWRGLSRYFKVQLRKRRVKNPYPISFLLTD	300
UYL83831.1	FGVGPLCKGDNLYLSAVDVCGMFTNRSGSQQWRGLSRYFKVQLRKRRVKNPYPISFLLTD	297
ALF06183.1	FGVGPLCKGDNLYLSAVDVCGMFTNRSGSQQWRGLSRYFKVQLRKRRVKNPYPISFLLTD	300
QCH41111.1	FGVGPLCKGDNLYLSAVDVCGMFTNRSGSQQWRGLSRYFKVQLRKRRVKNPYPISFLLTD	300
QCH41112.1	FGVGPLCKGDNLYLSAVDVCGMFTNRSGSQQWRGLSRYFKVQLRKRRGKNPYPISFLLTD	300
AEC14749.1	FGVGPLCKGDNLYLSAVDVCGMFTNRSGSQQWRGLSRYFKVQLRKRRVKNPYPISFLLTD	300
ALF06202.1	FGVGPLCKGDNLYLSAVDVCSMFTNRSGSQQWRGLSRYFKVQLRKRRVKNPYPISFLLTD	300
ALF06186.1	FGVGPLCKGDNLYLSAVDVCGMFTNRSGSQQWRGLSRYFRVQLRKRRVKNPYPISFLLTD	300
ALF06208.1	FGVGPLCKGDNLYLSAVDVCGMFTNRSGSRQWRGLSRYFKVQLRKRRVKNPYPISFLLTD	300
ALF06197.1	FGVGPLCKGDNLYLSAVDVCGMFTNRSGSQQWRGLSRYFKVQLRKRRVKNPYPISFLLTD	300
AEC15037.1	FGVGPLCKGDNLYLSAVDVCGMFTNKSGSQQWRGLSRYFKVQLRKRRVKNPYPISFLLTD	300
UPN63109.1	FGVGPLCKGDNLYLSAVDVCGMFTNRSGSQQWRGLSRYFKVQLRKRRVKNPYPISFLLTD	300
UPN63107.1	FGVGPLCKGDNLYLSAVDVCGMFTNRSGYQQWRGLSRYFKVQLRKRRVKNPYPISFLLTD	300
UPN63108.1	FGVGPLCKGDNLYLSAVDVCGMFTNRSGPQQWRGLSRYFKVQLRKRRVKNPYPISFLLTD	300
QBQ04202.1	FGVGPLCKGDNLYLSAVDVCGMFTNRSGSQQWRGLSRYFKVQLRKRRVKNPYPISFLLTD	300
QBM11923.1	FGVGPLCKGDNLYLSAVDVCGMFTNRSGSQQWRGLSRYFKVQLRKRRVKNPYPISFLLTD	300
AAK70306.1	FGAGPLCKGDNLYLSAVDVCGMFTNRSGSQQWRGLSRYFKVQLRKRRVKNPYPISFLLTD	300
AAC59337.1	FGVGPLCKGDNLYLSAVDVCGMFTNRSGSQQWRGLSRYFKVQLRKRRVKNPYPISFLLTD	300
AAL37677.1	FGVGPLCKGDNLYLSAVDVCGMFTNRSGSQQWRGLSRYFKVQLRKRRVKNPYPISFLLTD	300
AAK70276.1	FGVGPLCKGDNLYLSAVDVCGMFTNRSGSQQWRGLSRYFKVQLRKRRVKNPYPISFLLTD	300
BAC22792.1	FGVGPLCKGDNLYLSAVDVCGMFTNRSGSQQWRGLSRYFKVQLRKRRVKNPYPISFLLTD	300
BAC22798.1	FGVGPLCKGDNLYLSAVDVCGMFTNRSGSQQWRGLSRYFKVQLRKRRVKNPYPISFLLTD	300
BAC22804.1	FGVGPLCKGDNLYLSAVDVCGMFTNRSGSQQWRGLSRYFKVQLRKRRVKNPYPISFLLTD	300
BAC22810.1	FGVGPLCKGDNLYLSAVDVCGMFTNRSGSQQWRGLSRYFKVQLRKRRVKNPYPISFLLTD	300
BAC22858.1	FGVGPLCKGDNLYLSAVDVCGMFTNRSGSQQWRGLSRYFKVQLRKRRVKNPYPISFLLTD	300
BAC15701.1	FGVGPLCKGDNLYLSAVDVCGMFTNRSGSQQWRGLSRYFKVQLRKRRVKNPYPISFLLTD	300
BAB68863.1	FGVGPLCKGDNLYLSAVDVCGMFTNRSGSQQWRGLSRYFKVQLRKRRVKNPYPISFLLTD	300
BAC66424.1	FGVGPLCKGDNLYLSAVDVCGMFTNRSGSQQWRGLSRYFKVQLRKRRVKNPYPISFLLTD	300
BAD21235.2	FGVGPLCKGDNLYLSAVDVCGMFTNRSGSQQWRGLSRYFKVQLRKRRVKNPYPISFLLTD	300
QTJ15210.1	FGVGPLCKGDNLYLSAVDVCGMFTNRSGSQQWRGLSRYFKVQLRKRRVKNPYPISFLLTD	300
ASV51748.1	FGVGPLCKGDNLYLSAVDVCGMFTNRSGSQQWRGLSRYFKVQLRKRRVKNPYPISFLLTD	300
ASV51754.1	FGVGPLCKGDNLYLSAVDVCGMFTNRSGSQQWRGLSRYFKVQLRKRRVKNPYPISFLLTD	300
ASV51778.1	FGVGPLCKGDNLYLSAVDVCGMFTNRSGSQQWRGLSRYFKVQLRKRRVKNPYPISFLLTD	300
ASV51814.1	FGVGPLCKGDNLYLSAVDVCGMFTNRSGSQQWRGLSRYFKVQLRKRRVKNPYPISFLLTD	300
CCW72488.1	FGVGPLCKGDNLYLSAVDVCGMFTNRSGSQQWRGLSRYFKVQLRKRRVKNPYPISFLLTD	300
AEC15219.1	FGVGPLCKGDNLYLSAVDVCGMFTNRSGSQQWRGLSRYFKVQLRKRRVKNPYPISFLLTD	300
BAD21241.1	FGVGPLCKGDNLYLSAVDVCGMFTNRSGSQQWRGLSRYFKVQLRKRRVKNPYPISFLLTD	300
BAD21253.1	FGVGPLCKGDNLYLSAVDVCGMFTNRSGSQQWRGLSRYFKVQLRKRRVKNPYPISFLLTD	300

BAB93086.1	FGVGPLCKGDNLYLSAVDVCGMFTNRSGSQQWRGLSRYFKVQLRKRRVKNPYPISFLLTD	300
BAB68953.1	FGVGPLCKGDNLYLSAVDVCGMFTNRSGSQQWRGLSRYFKVQLRKRRVKNPYPISFLLTD	300
BAD98966.1	FGVGPLCKGDNLYLSAVDVCGMFTNRSGSQQWRGLSRYFKVQLRKRRVKNPYPISFLLTD	300
BAD98972.1	FGVGPLCKGDNLYLSAVDVCGMFTNRSGSQQWRGLSRYFKVQLRKRRVKNPYPISFLLTD	300
BAD91887.1	FGVGPLCKGDNLYLSAVDVCGMFTNRSGSQQWRGLSRYFKVQLRKRRVKNPYPISFLLTD	300
BAD27118.1	FGVGPLCKGDNLYLSAVDVCGMFTNRSGSQQWRGLSRYFKVQLRKRRVKNPYPISFLLTD	300
BAD06227.1	FGVGPLCKGDNLYLSAVDVCGMFTNRSGSQQWRGLSRYFKVQLRKRRVKNPYPISFLLTD	300
BAC66412.1	FGVGPLCKGDNLYLSAVDVCGMFTNRSGSQQWRGLSRYFKVQLRKRRVKNPYPISFLLTD	300
BAC66430.1	FGVGPLCKGDNLYLSAVDVCGMFTNRSGSQQWRGLSRYFKVQLRKRRVKNPYPISFLLTD	300
BAC22816.1	FGVGPLCKGDNLYLSAVDVCGMFTNRSGSQQWRGLSRYFKVQLRKRRVKNPYPISFLLTD	300
BAB93026.1	FGVGPLCKGDNLYLSAVDVCGMFTNRSGSQQWRGLSRYFKVQLRKRRVKNPYPISFLLTD	300
BAB93032.1	FGVGPLCKGDNLYLSAVDVCGMFTNRSGSQQWRGLSRYFKVQLRKRRVKNPYPISFLLTD	300
BAB93074.1	FGVGPLCKGDNLYLSAVDVCGMFTNRSGSQQWRGLSRYFKVQLRKRRVKNPYPISFLLTD	300
BAB93080.1	FGVGPLCKGDNLYLSAVDVCGMFTNRSGSQQWRGLSRYFKVQLRKRRVKNPYPISFLLTD	300
BAB68917.1	FGVGPLCKGDNLYLSAVDVCGMFTNRSGSQQWRGLSRYFKVQLRKRRVKNPYPISFLLTD	300
BAB68923.1	FGVGPLCKGDNLYLSAVDVCGMFTNRSGSQQWRGLSRYFKVQLRKRRVKNPYPISFLLTD	300
BAB68929.1	FGVGPLCKGDNLYLSAVDVCGMFTNRSGSQQWRGLSRYFKVQLRKRRVKNPYPISFLLTD	300
BAB68935.1	FGVGPLCKGDNLYLSAVDVCGMFTNRSGSQQWRGLSRYFKVQLRKRRVKNPYPISFLLTD	300
BAB68941.1	FGVGPLCKGDNLYLSAVDVCGMFTNRSGSQQWRGLSRYFKVQLRKRRVKNPYPISFLLTD	300
ALF06214.1	FGVGPLCKGDNLYLSAVDVCGMFTNRSGSQQWRGLSRYFKVQLRKRRVKNPYPISFLLTD	300
AAL37707.1	FGVGPLCKGDNLYLSAVDVCGMFTNRSGSQQWRGLSRYFKVQLRKRRVKNPYPISFLLTD	300
BBG22688.1	FGVGPLCKGDNLYLSAVDVCGMFTNRSGYQQWRGLSRYFKVQLRKRRVKNPYPISFLLTD	300
BAB11734.1	FGVGPLCKGDNLYLSAVDVCGMFTNRSGYQQWRGLSRYFKVQLRKRRVKNPYPISFLLTD	300
BAA05638.1	FGVGPLCKGDNLYLSAVDVCGMFTNRSGYQQWRGLSRYFKVQLRKRRVKNPYPISFLLTD	300
BAV69361.1	FGVGPLCKGDNLYLSAVDVCGMFTNRSGCQQWRGLSRYFKVQLRKRRVKNPYPISFLLTD	300
BAV69349.1	FGVGPLCKGDNLYLSAVDVCGMFTNRSGSQQWRGLSRYFKVQLRKRRVKNPYPISFLLTD	300
BAV69367.1	FGVGPLCKGDNLYLSAVDVCGMFTNRSGSQQWRGLSRYFKVQLRKRRVKNPYPISFLLTD	300
BAE00135.1	FGVGPLCKGDNLYLSAVDVCGMFTNRSGSQQWRGLSRYFKVQLRKRRVKNPYPISFLLTD	300
BAE00147.1	FGVGPLCKGDNLYLSAVDVCGMFTNRSGSQQWRGLSRYFKVQLRKRRVKNPYPISFLLTD	300
BAD11896.1	FGVGPLCKGDNLYLSAVDVCGMFTNRSGSQQWRGLSRYFKVQLRKRRVKNPYPISFLLTD	300
BAD06126.1	FGVGPLCKGDNLYLSAVDVCGMFTNRSGSQQWRGLSRYFKVQLRKRRVKNPYPISFLLTD	300
BAC81904.1	FGVGPLCKGDNLYLSAVDVCGMFTNRSGSQQWRGLSRYFKVQLRKRRVKNPYPISFLLTD	300
BAC81910.1	FGVGPLCKGDNLYLSAVDVCGMFTNRSGSQQWRGLSRYFKVQLRKRRVKNPYPISFLLTD	300
BAC81916.1	FGVGPLCKGDNLYLSAVDVCGMFTNRSGSQQWRGLSRYFKVQLRKRRVKNPYPISFLLTD	300
BAC81922.1	FGVGPLCKGDNLYLSAVDVCGMFTNRSGSQQWRGLSRYFKVQLRKRRVKNPYPISFLLTD	300
BAC81946.1	FGVGPLCKGDNLYLSAVDVCGMFTNRSGSQQWRGLSRYFKVQLRKRRVKNPYPISFLLTD	300
BAC81952.1	FGVGPLCKGDNLYLSAVDVCGMFTNRSGSQQWRGLSRYFKVQLRKRRVKNPYPISFLLTD	300
BAC81958.1	FGVGPLCKGDNLYLSAVDVCGMFTNRSGSQQWRGLSRYFKVQLRKRRVKNPYPISFLLTD	300
AAM89297.1	FGVGPLCKGDNLYLSAVDVCGMFTNRSGSQQWRGLSRYFKVQLRKRRVKNPYPISFLLTD	300
AAM89303.1	FGVGPLCKGDNLYLSAVDVCGMFTNRSGSQQWRGLSRYFKVQLRKRRVKNPYPISFLLTD	300
AAM89309.1	FGVGPLCKGDNLYLSAVDVCGMFTNRSGSQQWRGLSRYFKVQLRKRRVKNPYPISFLLTD	300
BAC66388.1	FGVGPLCKGDNLYLSAVDVCGMFTNRSGSQQWRGLSRYFKVQLRKRRVKNPYPISFLLTD	300
BAC66400.1	FGVGPLCKGDNLYLSAVDVCGMFTNRSGSQQWRGLSRYFKVQLRKRRVKNPYPISFLLTD	300
BAC66406.1	FGVGPLCKGDNLYLSAVDVCGMFTNRSGSQQWRGLSRYFKVQLRKRRVKNPYPISFLLTD	300
BAC22756.1	FGVGPLCKGDNLYLSAVDVCGMFTNRSGSQQWRGLSRYFKVQLRKRRVKNPYPISFLLTD	300
BAC22822.1	FGVGPLCKGDNLYLSAVDVCGMFTNRSGSQQWRGLSRYFKVQLRKRRVKNPYPISFLLTD	300
BAC22894.1	FGVGPLCKGDNLYLSAVDVCGMFTNRSGSQQWRGLSRYFKVQLRKRRVKNPYPISFLLTD	300
BAC22900.1	FGVGPLCKGDNLYLSAVDVCGMFTNRSGSQQWRGLSRYFKVQLRKRRVKNPYPISFLLTD	300
BAC15635.1	FGVGPLCKGDNLYLSAVDVCGMFTNRSGSQQWRGLSRYFKVQLRKRRVKNPYPISFLLTD	300
BAC15653.1	FGVGPLCKGDNLYLSAVDVCGMFTNRSGSQQWRGLSRYFKVQLRKRRVKNPYPISFLLTD	300
BAC15665.1	FGVGPLCKGDNLYLSAVDVCGMFTNRSGSQQWRGLSRYFKVQLRKRRVKNPYPISFLLTD	300
BAC03065.1	FGVGPLCKGDNLYLSAVDVCGMFTNRSGSQQWRGLSRYFKVQLRKRRVKNPYPISFLLTD	300
AAL37689.1	FGVGPLCKGDNLYLSAVDVCGMFTNRSGSQQWRGLSRYFKVQLRKRRVKNPYPISFLLTD	300
AAL37701.1	FGVGPLCKGDNLYLSAVDVCGMFTNRSGSQQWRGLSRYFKVQLRKRRVKNPYPISFLLTD	300
BAB68803.1	FGVGPLCKGDNLYLSAVDVCGMFTNRSGSQQWRGLSRYFKVQLRKRRVKNPYPISFLLTD	300
BAB68965.1	FGVGPLCKGDNLYLSAVDVCGMFTNRSGSQQWRGLSRYFKVQLRKRRVKNPYPISFLLTD	300
BAB68971.1	FGVGPLCKGDNLYLSAVDVCGMFTNRSGSQQWRGLSRYFKVQLRKRRVKNPYPISFLLTD	300
AAK97964.1	FGVGPLCKGDNLYLSAVDVCGMFTNRSGSQQWRGLSRYFKVQLRKRRVKNPYPISFLLTD	300
AAK97970.1	FGVGPLCKGDNLYLSAVDVCGMFTNRSGSQQWRGLSRYFKVQLRKRRVKNPYPISFLLTD	300
AAC40841.1	FGVGPLCKGDNLYLSAVDVCGMFTNRSGSQQWRGLSRYFKVQLRKRRVKNPYPISFLLTD	300
BAA05636.1	FGVGPLCKGDNLYLSAVDVCGMFTNRSGSQQWRGLSRYFKVQLRKRRVKNPYPISFLLTD	300
BAA01970.1	FGVGPLCKGDNLYLSAVDVCGMFTNRSGSQQWRGLSRYFKVQLRKRRVKNPYPISFLLTD	300
BAD06144.1	FGVGPLCKGDNLYLSAVDVCGMFTNRSGSQQWRGLSRYFKVQLRKRRVKNPYPISFLLTD	300
BAC81852.1	FGVGPLCKGDNLYLSAVDVCGMFTNRSGSQQWRGLSRYFKVQLRKRRVKNPYPISFLLTD	300

BAC81858.1	FGVGPLCKGDNLYLSAVDVCGMFTNRSGSQQWRGLSRYFKVQLRKRRVKNPYPISFLLTD	300
BAC22864.1	FGVGPLCKGDNLYLSAVDVCGMFTNRSGSQQWRGLSRYFKVQLRKRRVKNPYPISFLLTD	300
AAM89315.1	FGVGPLCKGDNLYLSAVYVCGMFTNRSGSQQWRGLSRYFKVQLRKRRVKNPYPISFLLTD	300
BAC66382.1	FGVGPLCKGDNLYLSAVDVCGMFTNRSGSQQWRGLSRYFKVQLRKRRVKNPYPISFLLTD	300
BAC66394.1	FGVGPLCKGDNLYLSAVDVCGMFTNRSGSQQWRGLSRYFKVQLRKRRVKNPYPISFLLTD	300
BAC66418.1	FGVGPLCKGDNLYLSAVDVCGMFTNRSGSQQWRGLSRYFKVQLRKRRVKNPYPISFLLTD	300
BAC22828.1	FGVGPLCKGDNLYLSAVDVCGMFTNRSGSQQWRGLSRYFKVQLRKRRVKNPYPISFLLTD	300
BAC22846.1	FGVGPLCKGDNLYLSAVDVCGMFTNRSGSQQWRGLSRYFKVQLRKRRVKNPYPISFLLTD	300
AAM69590.1	FGVGPLCKGDNLYLSAVDVCGMFTNRSGSQQWRGLSRYFKVQLRKRRVKNPYPISFLLTD	300
BAB93014.1	FGVGPLCKGDNLYLSAVDVCGMFTNRSGSQQWRGLSRYFKVQLRKRRVKNPYPISFLLTD	300
BAB93020.1	FGVGPLCKGDNLYLSAVDVCGMFTNRSGSQQWRGLSRYFKVQLRKRRVKNPYPISFLLTD	300
BAB93092.1	FGVGPLCKGDNLYLSAVDVCGMFTNRSGSQQWRGLSRYFKVQLRKRRVKNPYPISFLLTD	300
BAB93098.1	FGVGPLCKGDNLYLSAVDVCGMFTNRSGSQQWRGLSRYFKVQLRKRRVKNPYPISFLLTD	300
AAL37665.1	FGVGPLCKGDNLYLSAVDVCGMFTNRSGSQQWRGLSRYFKVQLRKRRVKNPYPISFLLTD	300
BAB68773.1	FGVGPLCKGDNLYLSAVDVCGMFTNRSGSQQWRGLSRYFKVQLRKRRVKNPYPISFLLTD	300
BAB68779.1	FGVGPLCKGDNLYLSAVDVCGMFTNRSGSQQWRGLSRYFKVQLRKRRVKNPYPISFLLTD	300
AAK28460.1	FGVGPLCKGDNLYLSAVDVCGMFTNRSGSQQWRGLSRYFKVQLRKRRVKNPYPISFLLTD	300
AAK28466.1	FGVGPLCKGDNLYLSAVDVCGMFTNRSGSQQWRGLSRYFKVQLRKRRVKNPYPISFLLTD	300
BAB11716.1	FGVGPLCKGDNLYLSAVDVCGMFTNRSGSQQWRGLSRYFKVQLRKRRVKNPYPISFLLTD	300
BAB11722.1	FGVGPLCKGDNLYLSAVDVCGMFTNRSGSQQWRGLSRYFKVQLRKRRVKNPYPISFLLTD	300
AAK97976.1	FGVGPLCKGDNLYLSAVDVCGMFTNRSGSQQWRGLSRYFKVQLRKRRVKNPYPISFLLTD	300
BAC15641.1	FGVGPLCKGDNLYLSAVDVCGMFTNRSGSQQWRGLSRYFKVQLRKRRVKNPYPISFLLTD	300
BAC15647.1	FGVGPLCKGDNLYLSAVDVCGMFTNRSGSQQWRGLSRYFKVQLRKRRVKNPYPISFLLTD	300
AAL37647.1	FGVGPLCKGDNLYLSAVDVCGMFTNRSGSQQWRGLSRYFKVQLRKRRVKNPYPISFLLTD	300
BAB68797.1	FGVGPLCKGDNLYLSAVDVCGMFTNRSGSQQWRGLSRYFKVQLRKRRVKNPYPISFLLTD	300
AAG37198.1	FGVGPLCKGDNLYLSAVDVCGMFTNRSGSQQWRGLSRYFKVQLRKRRVKNPYPISFLLTD	300
ALF06241.1	FGVGPLCKGDNLYLSAVDVCGMFTNRSGSQQWRGLSRYFKVQLRKRRVKNPYPISFLLTD	300
AFE56117.1	FGVGPLCKGDNLYLSAVDVCGMFTNRSGSQQWRGLSRYFKVQLRKRRVKNPYPISFLLTD	300
BAF95600.1	FGVGPLCKGDNLYLSAVDVCGMFTNRSGSQQWRGLSRYFKVQLRKRRVKNPYPISFLLTD	300
BAD11836.1	FGVGPLCKGDNLYLSAVDVCGMFTNRSGSQQWRGLSRYFKVQLRKRRVKNPYPISFLLTD	300
BAD11842.1	FGVGPLCKGDNLYLSAVDVCGMFTNRSGSQQWRGLSRYFKVQLRKRRVKNPYPISFLLTD	300
BAD11848.1	FGVGPLCKGDNLYLSAVDVCGMFTNRSGSQQWRGLSRYFKVQLRKRRVKNPYPISFLLTD	300
AAM89327.1	FGVGPLCKGDNLYLSAVDVCGMFTNRSGSQQWRGLSRYFKVQLRKRRVKNPYPISFLLTD	300
AAM89339.1	FGVGPLCKGDNLYLSAVDVCGMFTNRSGSQQWRGLSRYFKVQLRKRRVKNPYPISFLLTD	300
BAC15707.1	FGVGPLCKGDNLYLSAVDVCGMFTNRSGSQQWRGLSRYFKVQLRKRRVKNPYPISFLLTD	300
BAB68833.1	FGVGPLCKGDNLYLSAVDVCGMFTNRSGSQQWRGLSRYFKVQLRKRRVKNPYPISFLLTD	300
BAB68839.1	FGVGPLCKGDNLYLSAVDVCGMFTNRSGSQQWRGLSRYFKVQLRKRRVKNPYPISFLLTD	300
AYM55604.1	FGVGPLCKGDNLYLSAVDVCGMFTNRSGSQQWRGLSRYFKVQLRKRRVKNPYPISFLLTD	300
BAV69355.1	FGVGPLCKGDNLYLSAVDVCGMFTNRSGSQQWRGLSRYFKVQLRKRRVKNPYPISFLLTD	300
ALF06220.1	FGVGPLCKGDNLYLSAVDVCGMFTNRSGSQQWRGLSRYFKVQLRKRRVKNPYPISFLLTD	300
ALF06247.1	FGVGPLCKGDNLYLSAVDVCGMFTNRSGSQQWRGLSRYFKVQLRKRRVKNPYPISFLLTD	300
ALF06250.1	FGVGPLCKGDNLYLSAVDVCGMFTNRSGSQQWRGLSRYFKVQLRKRRVKNPYPISFLLTD	300
ALF06253.1	FGVGPLCKGDNLYLSAVDVCGMFTNRSGSQQWRGLSRYFKVQLRKRRVKNPYPISFLLTD	300
ALF06259.1	FGVGPLCKGDNLYLSAVDVCGMFTNRSGSQQWRGLSRYFKVQLRKRRVKNPYPISFLLTD	300
ALF06265.1	FGVGPLCKGDNLYLSAVDVCGMFTNRSGSQQWRGLSRYFKVQLRKRRVKNPYPISFLLTD	300
CCW72502.1	FGVGPLCKGDNLYLSAVDVCGMFTNRSGSQQWRGLSRYFKVQLRKRRVKNPYPISFLLTD	300
BAF40757.1	FGVGPLCKGDNLYLSAVDVCGMFTNRSGSQQWRGLSRYFKVQLRKRRVKNPYPISFLLTD	300
BAF40763.1	FGVGPLCKGDNLYLSAVDVCGMFTNRSGSQQWRGLSRYFKVQLRKRRVKNPYPISFLLTD	300
BAF40769.1	FGVGPLCKGDNLYLSAVDVCGMFTNRSGSQQWRGLSRYFKVQLRKRRVKNPYPISFLLTD	300
BAF40775.1	FGVGPLCKGDNLYLSAVDVCGMFTNRSGSQQWRGLSRYFKVQLRKRRVKNPYPISFLLTD	300
BAE94732.1	FGVGPLCKGDNLYLSAVDVCGMFTNRSGSQQWRGLSRYFKVQLRKRRVKNPYPISFLLTD	300
BAE94738.1	FGVGPLCKGDNLYLSAVDVCGMFTNRSGSQQWRGLSRYFKVQLRKRRVKNPYPISFLLTD	300
BAE00129.1	FGVGPLCKGDNLYLSAVDVCGMFTNRSGSQQWRGLSRYFKVQLRKRRVKNPYPISFLLTD	300
BAE00141.1	FGVGPLCKGDNLYLSAVDVCGMFTNRSGSQQWRGLSRYFKVQLRKRRVKNPYPISFLLTD	300
BAD11824.1	FGVGPLCKGDNLYLSAVDVCGMFTNRSGSQQWRGLSRYFKVQLRKRRVKNPYPISFLLTD	300
BAD11830.1	FGVGPLCKGDNLYLSAVDVCGMFTNRSGSQQWRGLSRYFKVQLRKRRVKNPYPISFLLTD	300
BAD11854.1	FGVGPLCKGDNLYLSAVDVCGMFTNRSGSQQWRGLSRYFKVQLRKRRVKNPYPISFLLTD	300
BAD11860.1	FGVGPLCKGDNLYLSAVDVCGMFTNRSGSQQWRGLSRYFKVQLRKRRVKNPYPISFLLTD	300
BAD11866.1	FGVGPLCKGDNLYLSAVDVCGMFTNRSGSQQWRGLSRYFKVQLRKRRVKNPYPISFLLTD	300
BAD11872.1	FGVGPLCKGDNLYLSAVDVCGMFTNRSGSQQWRGLSRYFKVQLRKRRVKNPYPISFLLTD	300
BAD11878.1	FGVGPLCKGDNLYLSAVDVCGMFTNRSGSQQWRGLSRYFKVQLRKRRVKNPYPISFLLTD	300
BAD11884.1	FGVGPLCKGDNLYLSAVDVCGMFTNRSGSQQWRGLSRYFKVQLRKRRVKNPYPISFLLTD	300
BAD11890.1	FGVGPLCKGDNLYLSAVDVCGMFTNRSGSQQWRGLSRYFKVQLRKRRVKNPYPISFLLTD	300
BAD11902.1	FGVGPLCKGDNLYLSAVDVCGMFTNRSGSQQWRGLSRYFKVQLRKRRVKNPYPISFLLTD	300

BAD06233.1	FGVGPLCKGDNLYLSAVDVCGMFTNRSGSQQWRGLSRYFKVQLRKRRVKNPYPISFLLTD	300
BAD06066.1	FGVGPLCKGDNLYLSAVDVCGMFTNRSGSQQWRGLSRYFKVQLRKRRVKNPYPISFLLTD	300
BAD06078.1	FGVGPLCKGDNLYLSAVDVCGMFTNRSGSQQWRGLSRYFKVQLRKRRVKNPYPISFLLTD	300
BAD06114.1	FGVGPLCKGDNLYLSAVDVCGMFTNRSGSQQWRGLSRYFKVQLRKRRVKNPYPISFLLTD	300
BAD06132.1	FGVGPLCKGDNLYLSAVDVCGMFTNRSGSQQWRGLSRYFKVQLRKRRVKNPYPISFLLTD	300
BAD06138.1	FGVGPLCKGDNLYLSAVDVCGMFTNRSGSQQWRGLSRYFKVQLRKRRVKNPYPISFLLTD	300
BAD06162.1	FGVGPLCKGDNLYLSAVDVCGMFTNRSGSQQWRGLSRYFKVQLRKRRVKNPYPISFLLTD	300
BAD06168.1	FGVGPLCKGDNLYLSAVDVCGMFTNRSGSQQWRGLSRYFKVQLRKRRVKNPYPISFLLTD	300
BAC82365.1	FGVGPLCKGDNLYLSAVDVCGMFTNRSGSQQWRGLSRYFKVQLRKRRVKNPYPISFLLTD	300
BAC81846.1	FGVGPLCKGDNLYLSAVDVCGMFTNRSGSQQWRGLSRYFKVQLRKRRVKNPYPISFLLTD	300
BAC81864.1	FGVGPLCKGDNLYLSAVDVCGMFTNRSGSQQWRGLSRYFKVQLRKRRVKNPYPISFLLTD	300
BAC81870.1	FGVGPLCKGDNLYLSAVDVCGMFTNRSGSQQWRGLSRYFKVQLRKRRVKNPYPISFLLTD	300
BAC81874.1	FGVGPLCKGDNLYLSAVDVCGMFTNRSGSQQWRGLSRYFKVQLRKRRVKNPYPISFLLTD	300
BAC81880.1	FGVGPLCKGDNLYLSAVDVCGMFTNRSGSQQWRGLSRYFKVQLRKRRVKNPYPISFLLTD	300
BAC81886.1	FGVGPLCKGDNLYLSAVDVCGMFTNRSGSQQWRGLSRYFKVQLRKRRVKNPYPISFLLTD	300
BAC81892.1	FGVGPLCKGDNLYLSAVDVCGMFTNRSGSQQWRGLSRYFKVQLRKRRVKNPYPISFLLTD	300
BAC81898.1	FGVGPLCKGDNLYLSAVDVCGMFTNRSGSQQWRGLSRYFKVQLRKRRVKNPYPISFLLTD	300
BAC81928.1	FGVGPLCKGDNLYLSAVDVCGMFTNRSGSQQWRGLSRYFKVQLRKRRVKNPYPISFLLTD	300
BAC81934.1	FGVGPLCKGDNLYLSAVDVCGMFTNRSGSQQWRGLSRYFKVQLRKRRVKNPYPISFLLTD	300
BAC81940.1	FGVGPLCKGDNLYLSAVDVCGMFTNRSGSQQWRGLSRYFKVQLRKRRVKNPYPISFLLTD	300
BAC81964.1	FGVGPLCKGDNLYLSAVDVCGMFTNRSGSQQWRGLSRYFKVQLRKRRVKNPYPISFLLTD	300
AAM89321.1	FGVGPLCKGDNLYLSAVDVCGMFTNRSGSQQWRGLSRYFKVQLRKRRVKNPYPISFLLTD	300
AAM89333.1	FGVGPLCKGDNLYLSAVDVCGMFTNRSGSQQWRGLSRYFKVQLRKRRVKNPYPISFLLTD	300
AAM89345.1	FGVGPLCKGDNLYLSAVDVCGMFTNRSGSQQWRGLSRYFKVQLRKRRVKNPYPISFLLTD	300
BAC66376.1	FGVGPLCKGDNLYLSAVDVCGMFTNRSGSQQWRGLSRYFKVQLRKRRVKNPYPISFLLTD	300
BAC22834.1	FGVGPLCKGDNLYLSAVDVCGMFTNRSGSQQWRGLSRYFKVQLRKRRVKNPYPISFLLTD	300
BAC22840.1	FGVGPLCKGDNLYLSAVDVCGMFTNRSGSQQWRGLSRYFKVQLRKRRVKNPYPISFLLTD	300
BAC22852.1	FGVGPLCKGDNLYLSAVDVCGMFTNRSGSQQWRGLSRYFKVQLRKRRVKNPYPISFLLTD	300
BAC22870.1	FGVGPLCKGDNLYLSAVDVCGMFTNRSGSQQWRGLSRYFKVQLRKRRVKNPYPISFLLTD	300
BAC22876.1	FGVGPLCKGDNLYLSAVDVCGMFTNRSGSQQWRGLSRYFKVQLRKRRVKNPYPISFLLTD	300
BAC22882.1	FGVGPLCKGDNLYLSAVDVCGMFTNRSGSQQWRGLSRYFKVQLRKRRVKNPYPISFLLTD	300
BAC22888.1	FGVGPLCKGDNLYLSAVDVCGMFTNRSGSQQWRGLSRYFKVQLRKRRVKNPYPISFLLTD	300
BAC15629.1	FGVGPLCKGDNLYLSAVDVCGMFTNRSGSQQWRGLSRYFKVQLRKRRVKNPYPISFLLTD	300
BAC15659.1	FGVGPLCKGDNLYLSAVDVCGMFTNRSGSQQWRGLSRYFKVQLRKRRVKNPYPISFLLTD	300
BAC15671.1	FGVGPLCKGDNLYLSAVDVCGMFTNRSGSQQWRGLSRYFKVQLRKRRVKNPYPISFLLTD	300
BAC15677.1	FGVGPLCKGDNLYLSAVDVCGMFTNRSGSQQWRGLSRYFKVQLRKRRVKNPYPISFLLTD	300
BAC15689.1	FGVGPLCKGDNLYLSAVDVCGMFTNRSGSQQWRGLSRYFKVQLRKRRVKNPYPISFLLTD	300
BAC15713.1	FGVGPLCKGDNLYLSAVDVCGMFTNRSGSQQWRGLSRYFKVQLRKRRVKNPYPISFLLTD	300
BAC15731.1	FGVGPLCKGDNLYLSAVDVCGMFTNRSGSQQWRGLSRYFKVQLRKRRVKNPYPISFLLTD	300
BAC15737.1	FGVGPLCKGDNLYLSAVDVCGMFTNRSGSQQWRGLSRYFKVQLRKRRVKNPYPISFLLTD	300
BAC15743.1	FGVGPLCKGDNLYLSAVDVCGMFTNRSGSQQWRGLSRYFKVQLRKRRVKNPYPISFLLTD	300
AAL37641.1	FGVGPLCKGDNLYLSAVDVCGMFTNRSGSQQWRGLSRYFKVQLRKRRVKNPYPISFLLTD	300
AAL37653.1	FGVGPLCKGDNLYLSAVDVCGMFTNRSGSQQWRGLSRYFKVQLRKRRVKNPYPISFLLTD	300
AAL37659.1	FGVGPLCKGDNLYLSAVDVCGMFTNRSGSQQWRGLSRYFKVQLRKRRVKNPYPISFLLTD	300
AAL37671.1	FGVGPLCKGDNLYLSAVDVCGMFTNRSGSQQWRGLSRYFKVQLRKRRVKNPYPISFLLTD	300
AAL37683.1	FGVGPLCKGDNLYLSAVDVCGMFTNRSGSQQWRGLSRYFKVQLRKRRVKNPYPISFLLTD	300
BAB68791.1	FGVGPLCKGDNLYLSAVDVCGMFTNRSGSQQWRGLSRYFKVQLRKRRVKNPYPISFLLTD	300
BAB68821.1	FGVGPLCKGDNLYLSAVDVCGMFTNRSGSQQWRGLSRYFKVQLRKRRVKNPYPISFLLTD	300
BAB68845.1	FGVGPLCKGDNLYLSAVDVCGMFTNRSGSQQWRGLSRYFKVQLRKRRVKNPYPISFLLTD	300
BAB68851.1	FGVGPLCKGDNLYLSAVDVCGMFTNRSGSQQWRGLSRYFKVQLRKRRVKNPYPISFLLTD	300
BAB68857.1	FGVGPLCKGDNLYLSAVDVCGMFTNRSGSQQWRGLSRYFKVQLRKRRVKNPYPISFLLTD	300
BAB68869.1	FGVGPLCKGDNLYLSAVDVCGMFTNRSGSQQWRGLSRYFKVQLRKRRVKNPYPISFLLTD	300
BAB68875.1	FGVGPLCKGDNLYLSAVDVCGMFTNRSGSQQWRGLSRYFKVQLRKRRVKNPYPISFLLTD	300
AAK97910.1	FGVGPLCKGDNLYLSAVDVCGMFTNRSGSQQWRGLSRYFKVQLRKRRVKNPYPISFLLTD	300
AAK97916.1	FGVGPLCKGDNLYLSAVDVCGMFTNRSGSQQWRGLSRYFKVQLRKRRVKNPYPISFLLTD	300
AAK97922.1	FGVGPLCKGDNLYLSAVDVCGMFTNRSGSQQWRGLSRYFKVQLRKRRVKNPYPISFLLTD	300
AAK97928.1	FGVGPLCKGDNLYLSAVDVCGMFTNRSGSQQWRGLSRYFKVQLRKRRVKNPYPISFLLTD	300
AAK97934.1	FGVGPLCKGDNLYLSAVDVCGMFTNRSGSQQWRGLSRYFKVQLRKRRVKNPYPISFLLTD	300
AAK97940.1	FGVGPLCKGDNLYLSAVDVCGMFTNRSGSQQWRGLSRYFKVQLRKRRVKNPYPISFLLTD	300
AAK97946.1	FGVGPLCKGDNLYLSAVDVCGMFTNRSGSQQWRGLSRYFKVQLRKRRVKNPYPISFLLTD	300
AAK97952.1	FGVGPLCKGDNLYLSAVDVCGMFTNRSGSQQWRGLSRYFKVQLRKRRVKNPYPISFLLTD	300
AAK97958.1	FGVGPLCKGDNLYLSAVDVCGMFTNRSGSQQWRGLSRYFKVQLRKRRVKNPYPISFLLTD	300
AAK97982.1	FGVGPLCKGDNLYLSAVDVCGMFTNRSGSQQWRGLSRYFKVQLRKRRVKNPYPISFLLTD	300
AAK97988.1	FGVGPLCKGDNLYLSAVDVCGMFTNRSGSQQWRGLSRYFKVQLRKRRVKNPYPISFLLTD	300

AAK97994.1	FGVGPLCKGDNLYLSAVDVCGMFTNRSGSQQWRGLSRYFKVQLRKRRVKNPYPISFLLTD	300
BAB11698.1	FGVGPLCKGDNLYLSAVDVCGMFTNRSGSQQWRGLSRYFKVQLRKRRVKNPYPISFLLTD	300
BAB11704.1	FGVGPLCKGDNLYLSAVDVCGMFTNRSGSQQWRGLSRYFKVQLRKRRVKNPYPISFLLTD	300
AAC59478.1	FGVGPLCKGDNLYLSAVDVCGMFTNRSGSQQWRGLSRYFKVQLRKRRVKNPYPISFLLTD	300
AAC59484.1	FGVGPLCKGDNLYLSAVDVCGMFTNRSGSQQWRGLSRYFKVQLRKRRVKNPYPISFLLTD	300
AAC59490.1	FGVGPLCKGDNLYLSAVDVCGMFTNRSGSQQWRGLSRYFKVQLRKRRVKNPYPISFLLTD	300
BAA01963.1	FGVGPLCKGDNLYLSAVDVCGMFTNRSGSQQWRGLSRYFKVQLRKRRVKNPYPISFLLTD	300
BAA01964.1	FGVGPLCKGDNLYLSAVDVCGMFTNRSGSQQWRGLSRYFKVQLRKRRVKNPYPISFLLTD	300
BAB93068.1	FGVGPLCKGDNLYLSAVDVCGMFTNRSGSQQWRGLSRYFKVQLRKRRVKNPYPISFLLTD	300
ASV51856.1	FGVGPLCKGDNLYLSAVDVCGMFTNRSGSQQWRGLSRYFKVQLRKRRVKNPYPISFLLTD	300
ANH21097.1	FGVGPLCKGDNLYLSAVDVCGMFTNRSGFQQWRGLSRYFKVQLRKRRVKNPYPISFLLTD	300
AEC15167.1	FGVGPLCKGDNLYLSAVDVCGMFTNRSGFQQWRGLSRYFKVQLRKRRVKNPYPISFLLTD	300
AEC15168.1	FGVGPLCKGDNLYLSAVDVCGMFTNRSGFQQWRGLSRYFKVQLRKRRVKNPYPISFLLTD	300
AEC15169.1	FGVGPLCKGDNLYLSAVDVCGMFTNRSGFQQWRGLSRYFKVQLRKRRVKNPYPISFLLTD	300
QTJ15168.1	FGVGPLCKGDNLYLSAVDVCGMFTNRSGSQQWRGLSRYFKVQLRKRRVKNPYPISFLLTD	300
AEC15173.1	FGVGPLCKGDNLYLSAVDVCGMFTNRSGSQKWRGLSRYFKVQLRKRRVKNPYPISFLLTD	300
AEC15174.1	FGVGPLCKGDNLYLSAVDVCGMFTNRSGSQKWRGLSRYFKVQLRKRRVKNPYPISFLLTD	300
AEC15175.1	FGVGPLCKGDNLYLSAVDVCGMFTNRSGSQKWRGLSRYFKVQLRKRRVKNPYPISFLLTD	300
AEC15176.1	FGVGPLCKGDNLYLSAVDVCGMFTNRSGSQKWRGLSRYFKVQLRKRRVKNPYPISFLLTD	300
AEC15177.1	FGVGPLCKGDNLYLSAVDVCGMFTNRSGSQKWRGLSRYFKVQLRKRRVKNPYPISFLLTD	300
AEC15178.1	FGVGPLCKGDNLYLSAVDVCGMFTNRSGSQKWRGLSRYFKVQLRKRRVKNPYPISFLLTD	300
AEC15179.1	FGVGPLCKGDNLYLSAVDVCGMFTNRSGSQKWRGLSRYFKVQLRKRRVKNPYPISFLLTD	300
AEC15180.1	FGVGPLCKGDNLYLSAVDVCGMFTNRSGSQKWRGLSRYFKVQLRKRRVKNPYPISFLLTD	300
AEC15181.1	FGVGPLCKGDNLYLSAVDVCGMFTNRSGSQKWRGLSRYFKVQLRKRRVKNPYPISFLLTD	300
AEC15182.1	FGVGPLCKGDNLYLSAVDVCGMFTNRSGSQKWRGLSRYFKVQLRKRRVKNPYPISFLLTD	300
AEC15183.1	FGVGPLCKGDNLYLSAVDVCGMFTNRSGSQKWRGLSRYFKVQLRKRRVKNPYPISFLLTD	300
AEC15184.1	FGVGPLCKGDNLYLSAVDVCGMFTNRSGSQKWRGLSRYFKVQLRKRRVKNPYPISFLLTD	300
AEC15185.1	FGVGPLCKGDNLYLSAVDVCGMFTNRSGSQKWRGLSRYFKVQLRKRRVKNPYPISFLLTD	300
AEC15187.1	FGVGPLCKGDNLYLSAVDVCGMFTNRSGSQKWRGLSRYFKVQLRKRRVKNPYPISFLLTD	300
AEC15188.1	FGVGPLCKGDNLYLSAVDVCGMFTNRSGSQKWRGLSRYFKVQLRKRRVKNPYPISFLLTD	300
AEC15189.1	FGVGPLCKGDNLYLSAVDVCGMFTNRSGSQKWRGLSRYFKVQLRKRRVKNPYPISFLLTD	300
AEC15190.1	FGVGPLCKGDNLYLSAVDVCGMFTNRSGSQKWRGLSRYFKVQLRKRRVKNPYPISFLLTD	300
AEC15191.1	FGVGPLCKGDNLYLSAVDVCGMFTNRSGSQKWRGLSRYFKVQLRKRRVKNPYPISFLLTD	300
AEC15193.1	FGVGPLCKGDNLYLSAVDVCGMFTNRSGSQKWRGLSRYFKVQLRKRRVKNPYPISFLLTD	300
AEC14871.1	FGVGPLCKGDNLYLSAVDVCGMFTNRSGSQQWRGLSRYFKVQLRKRRVKNPYPISFLLTD	300
AEC15222.1	FGVGPLCKGDNLYLSAVDVCGMFTNRSGSQQWRGLSRYFKVQLRKRRVKNPYPISFLLTD	300
AEC15130.1	FGVGPLCKGDNLYLSAVDVCGMFTNRSGSQQWRGLSRYFKVQLRKRRVKNPYPISFLLTD	300
AEC15144.1	FGVGPLCKGDNLYLSAVDVCGMFTNRSGSQQWRGLSRYFKVQLRKGRVKNPYPISFLLTD	300
AEC15154.1	FGVGPLCKGDNLYLSAVNVCGMFTNRSGSQQWRGLSRYFKVQLRKRRVKNPYPISFLLTD	300
AEC15157.1	FGVGPLCKGDNLYLSAVDVCGMFTNRSGSQQWRGLSRYFKVQLRKRRVKNPYPISFLLTD	300
AEC15160.1	FGVGPLCKGDNLYLSAVDVCGMFTNRSGSQQWRGLSRYFKVQLRKRRVKNPYPISFLLTD	300
AEC15231.1	FGVGPLCKGDNLYLSAVDVCGMFTNRSGSQQWRGLSRYFKVQLRKRRVKNPYPISFLLTD	300
ALF06191.1	FGVGPLCKGDNLYLSAVDVCGMFTNRSGSQQWRGLSRYFKVQLRKRRVKNPYPISFLLTD	300
WLD48112.1	FGVGPLCKGDNLYLSAVDVCGMFTNRSGSQQWRGLSRYFKVQLRKRRVKNPYPISFLLTD	300
QWT77140.1	FGVGPLCKGDNLYLSAVDVCGMFTNRSGSQQWRGLSRYFKVQLRKRRVKNPYPISFLLTD	300
QTJ15174.1	FGVGPLCKGDNLYLSAVDVCGMFTNRSGSQQWRGLSRYFKVQLRKRRVKNPYPISFLLTD	300
QTJ15186.1	FGVGPLCKGDNLYLSAVDVCGMFTNRSGSQQWRGLSRYFKVQLRKRRVKNPYPISFLLTD	300
QTJ15192.1	FGVGPLCKGDNLYLSAVDVCGMFTNRSGSQQWRGLSRYFKVQLRKRRVKNPYPISFLLTD	300
QTJ15198.1	FGVGPLCKGDNLYLSAVDVCGMFTNRSGSQQWRGLSRYFKVQLRKRRVKNPYPISFLLTD	300
ASV51772.1	FGVGPLCKGDNLYLSAVDVCGMFTNRSGSQQWRGLSRYFKVQLRKRRVKNPYPISFLLTD	300
CCW72478.1	FGVGPLCKGDNLYLSAVDVCGMFTNRSGSQQWRGLSRYFKVQLRKRRVKNPYPISFLLTD	300
CCW72481.1	FGVGPLCKGDNLYLSAVDVCGMFTNRSGSQQWRGLSRYFKVQLRKRRVKNPYPISFLLTD	300
CCW72487.1	FGVGPLCKGDNLYLSAVDVCGMFTNRSGSQQWRGLSRYFKVQLRKRRVKNPYPISFLLTD	300
CCW72492.1	FGVGPLCKGDNLYLSAVDVCGMFTNRSGSQQWRGLSRYFKVQLRKRRVKNPYPISFLLTD	300
CCW72496.1	FGVGPLCKGDNLYLSAVDVCGMFTNRSGSQQWRGLSRYFKVQLRKRRVKNPYPISFLLTD	300
CCW72499.1	FGVGPLCKGDNLYLSAVDVCGMFTNRSGSQQWRGLSRYFKVQLRKRRVKNPYPISFLLTD	300
CCW72504.1	FGVGPLCKGDNLYLSAVDVCGMFTNRSGSQQWRGLSRYFKVQLRKRRVKNPYPISFLLTD	300
CCW72508.1	FGVGPLCKGDNLYLSAVDVCGMFTNRSGSQQWRGLSRYFKVQLRKRRVKNPYPISFLLTD	300
CCW72510.1	FGVGPLCKGDNLYLSAVDVCGMFTNRSGSQQWRGLSRYFKVQLRKRRVKNPYPISFLLTD	300
CCW72519.1	FGVGPLCKGDNLYLSAVDVCGMFTNRSGSQQWRGLSRYFKVQLRKRRVKNPYPISFLLTD	300
CCW72521.1	FGVGPLCKGDNLYLSAVDVCGMFTNRSGSQQWRGLSRYFKVQLRKRRVKNPYPISFLLTD	300
AEC14864.1	FGVGPLCKGDNLYLSAVDVCGMFTNRSGSQQWRGLSRYFKVQLRKRRVKNPYPISFLLTD	300
AEC14865.1	FGVGPLCKGDNLYLSAVDVCGMFTNRSGSQQWRGLSRYFKVQLRKRRVKNPYPISFLLTD	300
AEC14866.1	FGVGPLCKGDNLYLSAVDVCGMFTNRSGSQQWRGLSRYFKVQLRKRRVKNPYPISFLLTD	300

AEC14867.1	FGVGPLCKGDNLYLSAVDVCGMFTNRSGSQQWRGLSRYFKVQLRKRRVKNPYPISFLLTD	300
AEC14868.1	FGVGPLCKGDNLYLSAVDVCGMFTNRSGSQQWRGLSRYFKVQLRKRRVKNPYPISFLLTD	300
AEC14869.1	FGVGPLCKGDNLYLSAVDVCGMFTNRSGSQQWRGLSRYFKVQLRKRRVKNPYPISFLLTD	300
AEC14870.1	FGVGPLCKGDNLYLSAVDVCGMFTNRSGSQQWRGLSRYFKVQLRKRRVKNPYPISFLLTD	300
AEC14872.1	FGVGPLCKGDNLYLSAVDVCGMFTNRSGSQQWRGLSRYFKVQLRKRRVKNPYPISFLLTD	300
AEC14873.1	FGVGPLCKGDNLYLSAVDVCGMFTNRSGSQQWRGLSRYFKVQLRKRRVKNPYPISFLLTD	300
AEC14874.1	FGVGPLCKGDNLYLSAVDVCGMFTNRSGSQQWRGLSRYFKVQLRKRRVKNPYPISFLLTD	300
AEC14875.1	FGVGPLCKGDNLYLSAVDVCGMFTNRSGSQQWRGLSRYFKVQLRKRRVKNPYPISFLLTD	300
AEC14876.1	FGVGPLCKGDNLYLSAVDVCGMFTNRSGSQQWRGLSRYFKVQLRKRRVKNPYPISFLLTD	300
AEC14877.1	FGVGPLCKGDNLYLSAVDVCGMFTNRSGSQQWRGLSRYFKVQLRKRRVKNPYPISFLLTD	300
AEC14878.1	FGVGPLCKGDNLYLSAVDVCGMFTNRSGSQQWRGLSRYFKVQLRKRRVKNPYPISFLLTD	300
AEC14879.1	FGVGPLCKGDNLYLSAVDVCGMFTNRSGSQQWRGLSRYFKVQLRKRRVKNPYPISFLLTD	300
AEC14880.1	FGVGPLCKGDNLYLSAVDVCGMFTNRSGSQQWRGLSRYFKVQLRKRRVKNPYPISFLLTD	300
AEC14881.1	FGVGPLCKGDNLYLSAVDVCGMFTNRSGSQQWRGLSRYFKVQLRKRRVKNPYPISFLLTD	300
AEC14882.1	FGVGPLCKGDNLYLSAVDVCGMFTNRSGSQQWRGLSRYFKVQLRKRRVKNPYPISFLLTD	300
AEC14883.1	FGVGPLCKGDNLYLSAVDVCGMFTNRSGSQQWRGLSRYFKVQLRKRRVKNPYPISFLLTD	300
AEC14884.1	FGVGPLCKGDNLYLSAVDVCGMFTNRSGSQQWRGLSRYFKVQLRKRRVKNPYPISFLLTD	300
AEC14885.1	FGVGPLCKGDNLYLSAVDVCGMFTNRSGSQQWRGLSRYFKVQLRKRRVKNPYPISFLLTD	300
AEC14886.1	FGVGPLCKGDNLYLSAVDVCGMFTNRSGSQQWRGLSRYFKVQLRKRRVKNPYPISFLLTD	300
AEC14887.1	FGVGPLCKGDNLYLSAVDVCGMFTNRSGSQQWRGLSRYFKVQLRKRRVKNPYPISFLLTD	300
AEC14888.1	FGVGPLCKGDNLYLSAVDVCGMFTNRSGSQQWRGLSRYFKVQLRKRRVKNPYPISFLLTD	300
AEC14889.1	FGVGPLCKGDNLYLSAVDVCGMFTNRSGSQQWRGLSRYFKVQLRKRRVKNPYPISFLLTD	300
AEC14890.1	FGVGPLCKGDNLYLSAVDVCGMFTNRSGSQQWRGLSRYFKVQLRKRRVKNPYPISFLLTD	300
AEC14891.1	FGVGPLCKGDNLYLSAVDVCGMFTNRSGSQQWRGLSRYFKVQLRKRRVKNPYPISFLLTD	300
AEC14892.1	FGVGPLCKGDNLYLSAVDVCGMFTNRSGSQQWRGLSRYFKVQLRKRRVKNPYPISFLLTD	300
AEC14894.1	FGVGPLCKGDNLYLSAVDVCGMFTNRSGSQQWRGLSRYFKVQLRKRRVKNPYPISFLLTD	300
AEC14895.1	FGVGPLCKGDNLYLSAVDVCGMFTNRSGSQQWRGLSRYFKVQLRKRRVKNPYPISFLLTD	300
AEC14896.1	FGVGPLCKGDNLYLSAVDVCGMFTNRSGSQQWRGLSRYFKVQLRKRRVKNPYPISFLLTD	300
AEC14897.1	FGVGPLCKGDNLYLSAVDVCGMFTNRSGSQQWRGLSRYFKVQLRKRRVKNPYPISFLLTD	300
AEC14898.1	FGVGPLCKGDNLYLSAVDVCGMFTNRSGSQQWRGLSRYFKVQLRKRRVKNPYPISFLLTD	300
AEC14899.1	FGVGPLCKGDNLYLSAVDVCGMFTNRSGSQQWRGLSRYFKVQLRKRRVKNPYPISFLLTD	300
AEC14900.1	FGVGPLCKGDNLYLSAVDVCGMFTNRSGSQQWRGLSRYFKVQLRKRRVKNPYPISFLLTD	300
AEC14901.1	FGVGPLCKGDNLYLSAVDVCGMFTNRSGSQQWRGLSRYFKVQLRKRRVKNPYPISFLLTD	300
AEC14902.1	FGVGPLCKGDNLYLSAVDVCGMFTNRSGSQQWRGLSRYFKVQLRKRRVKNPYPISFLLTD	300
AEC14903.1	FGVGPLCKGDNLYLSAVDVCGMFTNRSGSQQWRGLSRYFKVQLRKRRVKNPYPISFLLTD	300
AEC14904.1	FGVGPLCKGDNLYLSAVDVCGMFTNRSGSQQWRGLSRYFKVQLRKRRVKNPYPISFLLTD	300
AEC14905.1	FGVGPLCKGDNLYLSAVDVCGMFTNRSGSQQWRGLSRYFKVQLRKRRVKNPYPISFLLTD	300
AEC14907.1	FGVGPLCKGDNLYLSAVDVCGMFTNRSGSQQWRGLSRYFKVQLRKRRVKNPYPISFLLTD	300
AEC14908.1	FGVGPLCKGDNLYLSAVDVCGMFTNRSGSQQWRGLSRYFKVQLRKRRVKNPYPISFLLTD	300
AEC14909.1	FGVGPLCKGDNLYLSAVDVCGMFTNRSGSQQWRGLSRYFKVQLRKRRVKNPYPISFLLTD	300
AEC14910.1	FGVGPLCKGDNLYLSAVDVCGMFTNRSGSQQWRGLSRYFKVQLRKRRVKNPYPISFLLTD	300
AEC14911.1	FGVGPLCKGDNLYLSAVDVCGMFTNRSGSQQWRGLSRYFKVQLRKRRVKNPYPISFLLTD	300
AEC15164.1	FGVGPLCKGDNLYLSAVDVCGMFTNRSGSQQWRGLSRYFKVQLRKRRVKNPYPISFLLTD	300
BAF95594.1	FGVGPLCKGDNLYLSAVDVCGMFTNRSGSQQWRGLSRYFKVQLRKRRVKNPYPISFLLTD	300
BAF95606.1	FGVGPLCKGDNLYLSAVDVCGMFTNRSGSQQWRGLSRYFKVQLRKRRVKNPYPISFLLTD	300
BAF40745.1	FGVGPLCKGDNLYLSAVDVCGMFTNRSGSQQWRGLSRYFKVQLRKRRVKNPYPISFLLTD	300
BAF40751.1	FGVGPLCKGDNLYLSAVDVCGMFTNRSGSQQWRGLSRYFKVQLRKRRVKNPYPISFLLTD	300
BAF40781.1	FGVGPLCKGDNLYLSAVDVCGMFTNRSGSQQWRGLSRYFKVQLRKRRVKNPYPISFLLTD	300
BAF40787.1	FGVGPLCKGDNLYLSAVDVCGMFTNRSGSQQWRGLSRYFKVQLRKRRVKNPYPISFLLTD	300
BAF40793.1	FGVGPLCKGDNLYLSAVDVCGMFTNRSGSQQWRGLSRYFKVQLRKRRVKNPYPISFLLTD	300
BAF40799.1	FGVGPLCKGDNLYLSAVDVCGMFTNRSGSQQWRGLSRYFKVQLRKRRVKNPYPISFLLTD	300
BAF40811.1	FGVGPLCKGDNLYLSAVDVCGMFTNRSGSQQWRGLSRYFKVQLRKRRVKNPYPISFLLTD	300
BAF40817.1	FGVGPLCKGDNLYLSAVDVCGMFTNRSGSQQWRGLSRYFKVQLRKRRVKNPYPISFLLTD	300
BAF40823.1	FGVGPLCKGDNLYLSAVDVCGMFTNRSGSQQWRGLSRYFKVQLRKRRVKNPYPISFLLTD	300
BAF40829.1	FGVGPLCKGDNLYLSAVDVCGMFTNRSGSQQWRGLSRYFKVQLRKRRVKNPYPISFLLTD	300
BAF40835.1	FGVGPLCKGDNLYLSAVDVCGMFTNRSGSQQWRGLSRYFKVQLRKRRVKNPYPISFLLTD	300
BAF40841.1	FGVGPLCKGDNLYLSAVDVCGMFTNRSGSQQWRGLSRYFKVQLRKRRVKNPYPISFLLTD	300
BAF40847.1	FGVGPLCKGDNLYLSAVDVCGMFTNRSGSQQWRGLSRYFKVQLRKRRVKNPYPISFLLTD	300
BAC81834.1	FGVGPLCKGDNLYLSAVDVCGMFTNRSGSQQWRGLSRYFKVQLRKRRVKNPYPISFLLTD	300
BAC81840.1	FGVGPLCKGDNLYLSAVDVCGMFTNRSGSQQWRGLSRYFKVQLRKRRVKNPYPISFLLTD	300
AAN85461.1	FGVGPLCKGDNLYLSAVDVCGMFTNRSGSQQWRGLSRYFKVQLRKRRVKNPYPISFLLTD	300
AAN85467.1	FGVGPLCKGDNLYLSAVDVCGMFTNRSGSQQWRGLSRYFKVQLRKRRVKNPYPISFLLTD	300
AAN85473.1	FGVGPLCKGDNLYLSAVDVCGMFTNRSGSQQWRGLSRYFKVQLRKRRVKNPYPISFLLTD	300
BAC22762.1	FGVGPLCKGDNLYLSAVDVCGMFTNRSGSQQWRGLSRYFKVQLRKRRVKNPYPISFLLTD	300

```
BAC22768.1      FGVGPLCKGDNLYLSAVDVCGMFTNRSGSQQWRGLSRYFKVQLRKRRVKNPYPISFLLTD  300
BAC22774.1      FGVGPLCKGDNLYLSAVDVCGMFTNRSGSQQWRGLSRYFKVQLRKRRVKNPYPISFLLTD  300
BAC22780.1      FGVGPLCKGDNLYLSAVDVCGMFTNRSGSQQWRGLSRYFKVQLRKRRVKNPYPISFLLTD  300
BAC22786.1      FGVGPLCKGDNLYLSAVDVCGMFTNRSGSQQWRGLSRYFKVQLRKRRVKNPYPISFLLTD  300
BAB68809.1      FGVGPLCKGDNLYLSAVDVCGMFTNRSGSQQWRGLSRYFKVQLRKRRVKNPYPISFLLTD  300
BAB68815.1      FGVGPLCKGDNLYLSAVDVCGMFTNRSGSQQWRGLSRYFKVQLRKRRVKNPYPISFLLTD  300
BAB68827.1      FGVGPLCKGDNLYLSAVDVCGMFTNRSGSQQWRGLSRYFKVQLRKRRVKNPYPISFLLTD  300
AAC59496.1      FGVGPLCKGDNLYLSAVDVCGMFTNRSGSQQWRGLSRYFKVQLRKRRVKNPYPISFLLTD  300
AAC59502.1      FGVGPLCKGDNLYLSAVDVCGMFTNRSGSQQWRGLSRYFKVQLRKRRVKNPYPISFLLTD  300
AAC59508.1      FGVGPLCKGDNLYLSAVDVCGMFTNRSGSQQWRGLSRYFKVQLRKRRVKNPYPISFLLTD  300
AAC59514.1      FGVGPLCKGDNLYLSAVDVCGMFTNRSGSQQWRGLSRYFKVQLRKRRVKNPYPISFLLTD  300
AAC59520.1      FGVGPLCKGDNLYLSAVDVCGMFTNRSGSQQWRGLSRYFKVQLRKRRVKNPYPISFLLTD  300
AAB62680.1      FGVGPLCKGDNLYLSAVDVCGMFTNRSGSQQWRGLSRYFKVQLRKRRVKNPYPISFLLTD  300
BAA01961.1      FGVGPLCKGDNLYLSAVDVCGMFTNRSGSQQWRGLSRYFKVQLRKRRVKNPYPISFLLTD  300
WLD48082.1      FGVGPLCKGDNLYLSAVDVCGMFTNRSGSQQWRGLSRYFKVQLRKRRVKNPYPISFLLTD  300
WLD48088.1      FGVGPLCKGDNLYLSAVDVCGMFTNRSGSQQWRGLSRYFKVQLRKRRVKNPYPISFLLTD  300
CCW72516.1      FGVGPLCKGDNLYLSAVDVCGMFTNRSGSQQWRGLSRYFKVQLRKRRVKNPYPISFLLTD  300
CCW72523.1      FGVGPLCKGDNLYLSAVDVCGMFTNRSGSQQWRGLSRYFKVQLRKRRVKNPYPISFLLTD  300
AEC14783.1      FGVGPLCKGDNLYLSAVDVCGMFTNRSGSQQWRGLSRYFKVQLRKRRVKNPYPISFLLTD  300
AEC14784.1      FGVGPLCKGDNLYLSAVDVCGMFTNRSGSQQWRGLSRYFKVQLRKRRVKNPYPISFLLTD  300
AEC14785.1      FGVGPLCKGDNLYLSAVDVCGMFTNRSGSQQWRGLSRYFKVQLRKRRVKNPYPISFLLTD  300
AEC14786.1      FGVGPLCKGDNLYLSAVDVCGMFTNRSGSQQWRGLSRYFKVQLRKRRVKNPYPISFLLTD  300
AEC14787.1      FGVGPLCKGDNLYLSAVDVCGMFTNRSGSQQWRGLSRYFKVQLRKRRVKNPYPISFLLTD  300
AAB62687.1      FGVGPLCKGDNLYLSAVDVCGMFTNRSGSQQWRGLSRYFKVQLRKRRVKNPYPISFLLTD  300
AEC14893.1      FGVGPLCKGDNLYLSAVDVCGMFTNRSGSQQWRGLSRYFKVQLRKRRVKNPYPISFLLTD  300
AEC14906.1      FGVGPLCKGDNLYLSAVDVCGMFTNRSGSQQWRGLSRYFKVQLRKRRVKNPYPISFLLTD  300
AEC15118.1      FGVGPLCKGDNLYLSAVDVCGMFTNRSGSQQWRGLSRYFKVQLRKRRVKNPYPISFLLTD  300
AEC15120.1      FGVGPLCKGDNLYLSAVDVCGMFTNRSGSQQWRGLSRYFKVQLRKRRVKNPYPISFLLTD  300
AEC15121.1      FGVGPLCKGDNLYLSAVDVCGMFTNRSGSQQWRGLSRYFKVQLRKRRVKNPYPISFLLTD  300
AEC15122.1      FGVGPLCKGDNLYLSAVDVCGMFTNRSGSQQWRGLSRYFKVQLRKRRVKNPYPISFLLTD  300
AEC15124.1      FGVGPLCKGDNLYLSAVDVCGMFTNRSGSQQWRGLSRYFKVQLRKRRVKNPYPISFLLTD  300
AEC15125.1      FGVGPLCKGDNLYLSAVDVCGMFTNRSGSQQWRGLSRYFKVQLRKRRVKNPYPISFLLTD  300
AEC15127.1      FGVGPLCKGDNLYLSAVDVCGMFTNRSGSQQWRGLSRYFKVQLRKRRVKNPYPISFLLTD  300
AEC15129.1      FGVGPLCKGDNLYLSAVDVCGMFTNRSGSQQWRGLSRYFKVQLRKRRVKNPYPISFLLTD  300
AEC15131.1      FGVGPLCKGDNLYLSAVDVCGMFTNRSGSQQWRGLSRYFKVQLRKRRVKNPYPISFLLTD  300
AEC15133.1      FGVGPLCKGDNLYLSAVDVCGMFTNRSGSQQWRGLSRYFKVQLRKRRVKNPYPISFLLTD  300
AEC15135.1      FGVGPLCKGDNLYLSAVDVCGMFTNRSGSQQWRGLSRYFKVQLRKRRVKNPYPISFLLTD  300
AEC15136.1      FGVGPLCKGDNLYLSAVDVCGMFTNRSGSQQWRGLSRYFKVQLRKRRVKNPYPISFLLTD  300
AEC15137.1      FGVGPLCKGDNLYLSAVDVCGMFTNRSGSQQWRGLSRYFKVQLRKRRVKNPYPISFLLTD  300
AEC15138.1      FGVGPLCKGDNLYLSAVDVCGMFTNRSGSQQWRGLSRYFKVQLRKRRVKNPYPISFLLTD  300
AEC15139.1      FGVGPLCKGDNLYLSAVDVCGMFTNRSGSQQWRGLSRYFKVQLRKRRVKNPYPISFLLTD  300
AEC15141.1      FGVGPLCKGDNLYLSAVDVCGMFTNRSGSQQWRGLSRYFKVQLRKRRVKNPYPISFLLTD  300
AEC15142.1      FGVGPLCKGDNLYLSAVDVCGMFTNRSGSQQWRGLSRYFKVQLRKRRVKNPYPISFLLTD  300
AEC15143.1      FGVGPLCKGDNLYLSAVDVCGMFTNRSGSQQWRGLSRYFKVQLRKRRVKNPYPISFLLTD  300
AEC15145.1      FGVGPLCKGDNLYLSAVDVCGMFTNRSGSQQWRGLSRYFKVQLRKRRVKNPYPISFLLTD  300
AEC15146.1      FGVGPLCKGDNLYLSAVDVCGMFTNRSGSQQWRGLSRYFKVQLRKRRVKNPYPISFLLTD  300
AEC15147.1      FGVGPLCKGDNLYLSAVDVCGMFTNRSGSQQWRGLSRYFKVQLRKRRVKNPYPISFLLTD  300
AEC15148.1      FGVGPLCKGDNLYLSAVDVCGMFTNRSGSQQWRGLSRYFKVQLRKRRVKNPYPISFLLTD  300
AEC15149.1      FGVGPLCKGDNLYLSAVDVCGMFTNRSGSQQWRGLSRYFKVQLRKRRVKNPYPISFLLTD  300
AEC15150.1      FGVGPLCKGDNLYLSAVDVCGMFTNRSGSQQWRGLSRYFKVQLRKRRVKNPYPISFLLTD  300
AEC15151.1      FGVGPLCKGDNLYLSAVDVCGMFTNRSGSQQWRGLSRYFKVQLRKRRVKNPYPISFLLTD  300
AEC15152.1      FGVGPLCKGDNLYLSAVDVCGMFTNRSGSQQWRGLSRYFKVQLRKRRVKNPYPISFLLTD  300
AEC15153.1      FGVGPLCKGDNLYLSAVDVCGMFTNRSGSQQWRGLSRYFKVQLRKRRVKNPYPISFLLTD  300
AEC15155.1      FGVGPLCKGDNLYLSAVDVCGMFTNRSGSQQWRGLSRYFKVQLRKRRVKNPYPISFLLTD  300
AEC15156.1      FGVGPLCKGDNLYLSAVDVCGMFTNRSGSQQWRGLSRYFKVQLRKRRVKNPYPISFLLTD  300
AEC15158.1      FGVGPLCKGDNLYLSAVDVCGMFTNRSGSQQWRGLSRYFKVQLRKRRVKNPYPISFLLTD  300
AEC15159.1      FGVGPLCKGDNLYLSAVDVCGMFTNRSGSQQWRGLSRYFKVQLRKRRVKNPYPISFLLTD  300
AEC15161.1      FGVGPLCKGDNLYLSAVDVCGMFTNRSGSQQWRGLSRYFKVQLRKRRVKNPYPISFLLTD  300
AEC15162.1      FGVGPLCKGDNLYLSAVDVCGMFTNRSGSQQWRGLSRYFKVQLRKRRVKNPYPISFLLTD  300
AEC15163.1      FGVGPLCKGDNLYLSAVDVCGMFTNRSGSQQWRGLSRYFKVQLRKRRVKNPYPISFLLTD  300
AEC15226.1      FGVGPLCKGDNLYLSAVDVCGMFTNRSGSQQWRGLSRYFKVQLRKRRVKNPYPISFLLTD  300
AEC15227.1      FGVGPLCKGDNLYLSAVDVCGMFTNRSGSQQWRGLSRYFKVQLRKRRVKNPYPISFLLTD  300
AEC15228.1      FGVGPLCKGDNLYLSAVDVCGMFTNRSGSQQWRGLSRYFKVQLRKRRVKNPYPISFLLTD  300
AEC15229.1      FGVGPLCKGDNLYLSAVDVCGMFTNRSGSQQWRGLSRYFKVQLRKRRVKNPYPISFLLTD  300
```

AEC15230.1	FGVGPLCKGDNLYLSAVDVCGMFTNRSGSQQWRGLSRYFKVQLRKRRVKNPYPISFLLTD	300
AEC15236.1	FGVGPLCKGDNLYLSAVDVCGMFTNRSGSQQWRGLSRYFKVQLRKRRVKNPYPISFLLTD	300
AEC15237.1	FGVGPLCKGDNLYLSAVDVCGMFTNRSGSQQWRGLSRYFKVQLRKRRVKNPYPISFLLTD	300
AEC15238.1	FGVGPLCKGDNLYLSAVDVCGMFTNRSGSQQWRGLSRYFKVQLRKRRVKNPYPISFLLTD	300
AEC15239.1	FGVGPLCKGDNLYLSAVDVCGMFTNRSGSQQWRGLSRYFKVQLRKRRVKNPYPISFLLTD	300
AEC15240.1	FGVGPLCKGDNLYLSAVDVCGMFTNRSGSQQWRGLSRYFKVQLRKRRVKNPYPISFLLTD	300
AEC15447.1	FGVGPLCKGDNLYLSAVDVCGMFTNRSGSQQWRGLSRYFKVQLRKRRVKNPYPISFLLTD	300
AEC15453.1	FGVGPLCKGDNLYLSAVDVCGMFTNRSGSQQWRGLSRYFKVQLRKRRVKNPYPISFLLTD	300
AEC15459.1	FGVGPLCKGDNLYLSAVDVCGMFTNRSGSQQWRGLSRYFKVQLRKRRVKNPYPISFLLTD	300
AEC15465.1	FGVGPLCKGDNLYLSAVDVCGMFTNRSGSQQWRGLSRYFKVQLRKRRVKNPYPISFLLTD	300
AEC15471.1	FGVGPLCKGDNLYLSAVDVCGMFTNRSGSQQWRGLSRYFKVQLRKRRVKNPYPISFLLTD	300
AEC15477.1	FGVGPLCKGDNLYLSAVDVCGMFTNRSGSQQWRGLSRYFKVQLRKRRVKNPYPISFLLTD	300
AEC15483.1	FGVGPLCKGDNLYLSAVDVCGMFTNRSGSQQWRGLSRYFKVQLRKRRVKNPYPISFLLTD	300
AEC15489.1	FGVGPLCKGDNLYLSAVDVCGMFTNRSGSQQWRGLSRYFKVQLRKRRVKNPYPISFLLTD	300
AEC15543.1	FGVGPLCKGDNLYLSAVDVCGMFTNRSGSQQWRGLSRYFKVQLRKRRVKNPYPISFLLTD	300
AEC15119.1	FGVGPLCKGDNLYLSAVDVCGMFTNRSGSQQWRGLSRYFKVQLRKRRVKNPYPISFLLTD	300
AEC15123.1	FGVGPLCKGDNLYLSAVDVCGMFTNRSGSQQWRGLSRYFKVQLRKRRVKNPYPISFLLTD	300
AEC15126.1	FGVGPLCKGDNLYLSAVDVCGMFTNRSGSQQWRGLSRYFKVQLRKRRVKNPYPISFLLTD	300
AEC15132.1	FGVGPLCKGDNLYLSAVDVCGMFTNRSGSQQWRGLSRYFKVQLRKRRVKNPYPISFLLTD	300
AEC15134.1	FGVGPLCKGDNLYLSAVDVCGMFTNRSGSQQWRGLSRYFKVQLRKRRVKNPYPISFLLTD	300
AEC15140.1	FGVGPLCKGDNLYLSAVDVCGMFTNRSGSQQWRGLSRYFKVQLRKRRVKNPYPISFLLTD	300
AEC15221.1	FGVGPLCKGDNLYLSAVDVCGMFTNRSGSQQWRGLSRYFKVQLRKRRVKNPYPISFLLTD	300
AEC15224.1	FGVGPLCKGDNLYLSAVDVCGMFTNRSGSQQWRGLSRYFKVQLRKRRVKNPYPISFLLTD	300
AEC15225.1	FGVGPLCKGDNLYLSAVDVCGMFTNRSGSQQWRGLSRYFKVQLRKRRVKNPYPISFLLTD	300
AEC15233.1	FGVGPLCKGDNLYLSAVDVCGMFTNRSGSQQWRGLSRYFKVQLRKRRVKNPYPISFLLTD	300
AEC15234.1	FGVGPLCKGDNLYLSAVDVCGMFTNRSGSQQWRGLSRYFKVQLRKRRVKNPYPISFLLTD	300
AEC15235.1	FGVGPLCKGDNLYLSAVDVCGMFTNRSGSQQWRGLSRYFKVQLRKRRVKNPYPISFLLTD	300
AEC15241.1	FGVGPLCKGDNLYLSAVDVCGMFTNRSGSQQWRGLSRYFKVQLRKRRVKNPYPISFLLTD	300
AEC15220.1	FGVGPLCKGDNLYLSAVDVCGMFTNRSGSQQWRGLSRYFKVQLRKRRVKNPYPISFLLTD	300
AEC15223.1	FGVGPLCKGDNLYLSAVDVCGMFTNRSGSQQWRGLSRYFKVQLRKRRVKNPYPISFLLTD	300
AEC15232.1	FGVGPLCKGDNLYLSAVDVCGMFTNRSGSQQWRGLSRYFKVQLRKRRVKNPYPISFLLTD	300
AEC15128.1	FGVGPLCKGDNLYLSAVDVCGMFTNRSGSQQWRGLSRYFKVQLRKRRVKNPYPISFLLTD	300
AEC15495.1	FGVGPLCKGDNLYLSAVDVCGMFTNRSGSQQWRGLSRYFKVQLRKRRVKNPYPISFLLTD	300
AEC15501.1	FGVGPLCKGDNLYLSAVDVCGMFTNRSGSQQWRGLSRYFKVQLRKRRVKNPYPISFLLTD	300
AEC15507.1	FGVGPLCKGDNLYLSAVDVCGMFTNRSGSQQWRGLSRYFKVQLRKRRVKNPYPISFLLTD	300
AEC15513.1	FGVGPLCKGDNLYLSAVDVCGMFTNRSGSQQWRGLSRYFKVQLRKRRVKNPYPISFLLTD	300
AEC15519.1	FGVGPLCKGDNLYLSAVDVCGMFTNRSGSQQWRGLSRYFKVQLRKRRVKNPYPISFLLTD	300
AEC15525.1	FGVGPLCKGDNLYLSAVDVCGMFTNRSGSQQWRGLSRYFKVQLRKRRVKNPYPISFLLTD	300
AEC15531.1	FGVGPLCKGDNLYLSAVDVCGMFTNRSGSQQWRGLSRYFKVQLRKRRVKNPYPISFLLTD	300
AEC15537.1	FGVGPLCKGDNLYLSAVDVCGMFTNRSGSQQWRGLSRYFKVQLRKRRVKNPYPISFLLTD	300
BAF40805.1	FGVGPLCKGDNLYLSAVDVCGMFTNRSGSQQWRGLSRYFKVQLRKRRVKNPYPISFLLTD	300
AAT09843.1	FGVGPLCKGDNLYLSAVDVCGMFTNRSGCQQWRGLSRYFKVQLRKRRVKNPYPISFLLTD	300
BAA01965.1	FGVGPLCKGDNLYLSAVDVCGMFTNRSGFQQWRGLSRYFKVQLRKRRVKNPYPISFLLTD	300
AIW00320.1	FGVGPLCKGDNLYLSAVDVCGMFTNRSGSQQWRGLSRYFKVQLRKRRVKNPYPISFLLTD	300
ALF06194.1	FGVGPLCKGDNLYLSAVDVCGMFTNRSGSQQWRGLSRYFKVQLRRRRVKNPYPISFLLTD	300
ALF06211.1	FGVGPLCKGDNLYLSAVDVCGMFTNRSGSQQWRGLSRYFKVQLRKRRVKNPYPISFLLTD	300
AEC14821.1	FGVGPLCKGDNLYLSAVDVCDMFTNRSGSQQWRGLSRYFKVQLRKRRVKNPYPISFLLTD	300
AEC14860.1	FGVGPLCKGDNLYLSAVDVCGMFTNRSGSQQWRGLSRYFKVQLRKRRVKNPYPISFLLTD	300
AEC14861.1	FGVGPLCKGDNLYLSAVDVCGMFTNRSGSQQWRGLSRYFKVQLRKRRVKNPYPISFLLTD	300
AEC14811.1	FGVGPLCKGDNLYLSAVDVCGMFTNRSGSQQWRGLSRYFKVQLRKRRVKNPYPISFFLTD	300
AEC14806.1	FGVGPLCKGDNLYLSAVDVCGMFTNRSGSQQWRGLSRYFKVQLRKRRVKNPYPISFLLTD	300
AEC14795.1	FGVGPLCKGDNLYLSAVDVRGMFTNRSGSQQWRGLSRYFKVQLRKRRVKNPYPISFLLTD	300
AEC14765.1	FGVGPLCKGDNLYLSAVDVCGMFTNRSGSQQWRGLSRYFKVQLRKRRVKNPYPISFLLTD	300
AEC14774.1	FGVGPLCKGDNLYLSAVDVCGMFTNRSGSQQWRGLSRYFKVQLRKRRVKNPYPISFLLTD	300
AEC14761.1	FGVGPLCKGDNLYLSAVDVCGMFTNRSGSQQWRGLSRYFKVQLRKRRVKNPYPISFLLTD	300
AEC14753.1	FGVGPLCKGDNLYLSAVDVCGMFTNRSCSQQWRGLSRYFKVQLRKRRVKNPYPISFLLTD	300
AEC14767.1	FGVGPLCKGDNLYLSAVDVCGMFTNRSGSQQWRGLSRYFKVQLRKRRVKNPYPISFLLTD	300
AEC14780.1	FGVGPLCKGDNLYLSAVDVCGMFTNRSGSQQWRGLSRYFKVQLRKRRVKNPYPISFLLTD	300
AEC14760.1	FGVGPLCKGDNLYLSAVDVCGMFTNRSGSQQWRGLSRYFKVQLRKRRVKNPYPISFLLTD	300
AEC14762.1	FGVGPLCKGDNLYLSAVDVCGMFTNRSGSQQWRGLSRYFKVQLRKRRVKNPYPISFLLTD	300
AEC14742.1	FGVGPLCKGDNLYLSAVDVCGMFTNRSGSQQWRGLSRYFKVQLRKRRVKNPYPISFLLTD	300
AEC14763.1	FGVGPLCKGDNLYLSAVDVCGMFTNRSGSQQWRGLSRYFKVQLRKRRVKNPYPISFLLTD	300
AEC14810.1	FGVGPLCKGDNLYLSAVDVCGMFTNRSGSQQWRGLSRYFKVQLRKRRVKNPYPISFLLTD	300
AEC14803.1	FGVGPLCKGDNLYLSAVDVCGMFTNRSGSQQWRGLSRYFKVQLRKRRVKNPYPISFLLTD	300

AEC14802.1	FGVGPLCKGDNLYLSAVDVCGMFTNRSGSQQWRGLSRYFKVQLRKRRVKNPYPISFLLTD	300
AEC14812.1	FGVGPLCKGDNLYLSAVDVCGMFTNRSGSQQWRGLSRYFKVQLRKRRVKNPYPISFLLTD	300
AEC14804.1	FGVGPLCKGDNLYLSAVDVCGMFTNRSGSQQWRGLSRYFKVQLRKRRVKNPYPISFLLTD	300
AEC14797.1	FGVGPLCKGDNLYLSAVDVCGMFTNRSGSQQWRGLSRYFKVQLRKRRVKNPYPISFLLTD	300
AEC14796.1	FGVGPLCKGDNLYLSAVDVCGMFTNRSGSQQWRGLSRYFKVQLRKRRVKNPYPISFLLTD	300
AEC14793.1	FGVGPLCKGDNLYLSAVDVCGMFTNRSGSQQWRGLSRYFKVQLRKRRVKNPYPISFLLTD	300
AEC14791.1	FGVGPLCKGDNLYLSAVDVCGMFTNRSGSQQWRGLSRYFKVQLRKRRVKNPYPISFLLTD	300
AEC14794.1	FGVGPLCKGDNLYLSAVDVCGMFTNRSGSQQWRGLSRYFKVQLRKRRVKNPYPISFLLTD	300
AEC14789.1	FGVGPLCKGDNLYLSAVDVCGMFTNRSGSQQWRGLSRYFKVQLRKRRVKNPYPISFLLTD	300
AEC14743.1	FGVGPLCKGDNLYLSAVDVCGMFTNRSGSQQWRGLSRYFKVQLRKRRVKNPYPISFLLTD	300
AEC14745.1	FGVGPLCKGDNLYLSAVDVCGMFTNRSGSQQWRGLSRYFKVQLRKRRIKNPYPISFLLTD	300
AEC14750.1	FGVGPLCKGDNLYLSAVDVCGMFTNRSGSQQWRGLSRYFKVQLRKRRVKNPYPISFLLTD	300
AEC14754.1	FGVGPLCKGDNLYLSAVDVCGMFTNRSGSQQWRGLSRYFKVQLRKRRVKNPYPISFLLTD	300
AEC14751.1	FGVGPLCKGDNLYLSAVDVCGMFTNRSGSQQWRGLSRYFKVQLRKRRVKNPYPISFLLTD	300
AEC14776.1	FGVGPLCKGDNLYLSAVDVCGMFTNRSGSQQWRGLSRYFKVQLRKRRVKNPYPISFLLTD	300
AEC14755.1	FGVGPLCKGDNLYLSAVDVCGMFTNRSGSQQWRGLSRYFKVQLRKRRVKNPYPISFLLTD	300
AEC14759.1	FGVGPLCKGDNLYLSAVDVCGMFTNRSGSQQWRGLSRYFKVQLRKRRVKNPYPISFLLTD	300
AEC14766.1	FGVGPLCKGDNLYLSAVDVCGMFTNRSGSQQWRGLSRYFKVQLRKRRVKNPYPISFLLTD	300
AEC14781.1	FGVGPLCKGDNLYLSAVDVCGMFTNRSGSQQWRGLSRYFKVQLRKRRVKNPYPISFLLAD	300
AEC15186.1	FGVGPLCKGDNVYLSAVDVCGMFTNRSGSQKWRGLSRYFKVQLRKRRVKNPYPISFLLTD	300
AEC15192.1	FGVGPLCKGDNLYLSAVDVCGMFTNRSGSQKWRGLSRYFKVQLRKRRVKNPYPISFLLTD	300
AEC15194.1	FGVGPLCKGDNLYLSAVDVCGMFTNRSGSQKWRGLSRYFKVQLRKRRVKNPYPISFLLTD	300
AEC15195.1	FGVGPLCKGDNLYLSAVDVCGMFTNRSGSQKWRGLSRYFKVQLRKRRVKNPYPISFLLTD	300
QWT77104.1	FGVGPLCKGDNLYLSAVDVCGMFTDRSGSQQWRGLSRYFKVQLRKRRVKNPYPISFLLTD	300
UPN63088.1	FGVGPLCKGDNLYLSAVDVCGMFTDRSGSQQWRGLSRYFKVQLRKRRVKNPYPISFLLTD	300
UPN63104.1	FGVGPLCKGDNLYLSAVDVCGMFTNRSGSQQWRGLSRYFKVQLRKRRVKNPYPISFLLTD	300
UPN63112.1	FGVGPLCKGDNLYLSAVDVCGMFTNRSGSQQWRGLSRYFKVQLRKRRVKNPYPISFLLTD	300
UPN63113.1	FGVGPLCKGDNLYLSAVDVCGMFTNRSGSQQWRGLSRYFKVQLRKRRVKNPYPISFLLTD	300
UPN63114.1	FGVGPLCKGDNLYLSAVDVCGMFTNRSGSQQWRGLSRYFKVQLRKRRVKNPYPISFLLTD	300
UPN63115.1	FGVGPLCKGDNLYLSAVDVCGMFTNRSGSQQWRGLSRYFKVQLRKRRVKNPYPISFLLTD	300
AEC15172.1	FGVGPLCKGDNLYLSAVDVCGMFTNRXGSQXWRGLSRYFKVQLRKRRVKNPYPISFLLTD	300
BAE00117.1	FGVGPLCKGDNLYLSAVDVCGMFTNRSGFQQWRGLSRYFKVQLRKRRVKNPYPISFLLTD	300
BAE00123.1	FGVGPLCKGDNLYLSAVDVCGMFTNRSGFQQWRGLSRYFKVQLRKRRVKNPYPISFLLTD	300
AAC59331.1	FGVGPLCKGDNLYLSAVDVCGMFTNRSGSQQWRGLSRYFKVQLRKRRVKNPYPISFLLTD	300
WLD48106.1	FGVGPLCKGDNLYLSAVDVCGMFTNRSGSQQWRGLSRYFKVQLRKRRVKNPYPISFLLTD	300
UQK62661.1	FGVGPLCKGDNLYLSAVDVCGMFTNRSGSQQWRGLSRYFKVQLRKRRVKNPYPISFLLTD	300
QTJ15156.1	FGVGPLCKGDNLYLSAVDVCGMFTNRSGSQQWRGLSRYFKVQLRKRRVKNPYPISFLLTD	300
QNC68213.1	FGVGPLCKGDNLYLSAVDVCGMFTNRSGSQQWRGLSRYFKVQLRKRRVKNPYPISFLLTD	300
ASV51766.1	FGVGPLCKGDNLYLSAVDVCGMFTNRSGSQQWRGLSRYFKVQLRKRRVKNPYPISFLLTD	300
ASV51796.1	FGVGPLCKGDNLYLSAVDVCGMFTNRSGSQQWRGLSRYFKVQLRKRRVKNPYPISFLLTD	300
ASV51802.1	FGVGPLCKGDNLYLSAVDVCGMFTNRSGSQQWRGLSRYFKVQLRKRRVKNPYPISFLLTD	300
ASV51808.1	FGVGPLCKGDNLYLSAVDVCGMFTNRSGSQQWRGLSRYFKVQLRKRRVKNPYPISFLLTD	300
ASV51820.1	FGVGPLCKGDNLYLSAVDVCGMFTNRSGSQQWRGLSRYFKVQLRKRRVKNPYPISFLLTD	300
ASV51826.1	FGVGPLCKGDNLYLSAVDVCGMFTNRSGSQQWRGLSRYFKVQLRKRRVKNPYPISFLLTD	300
ASV51832.1	FGVGPLCKGDNLYLSAVDVCGMFTNRSGSQQWRGLSRYFKVQLRKRRVKNPYPISFLLTD	300
ASV51838.1	FGVGPLCKGDNLYLSAVDVCGMFTNRSGSQQWRGLSRYFKVQLRKRRVKNPYPISFLLTD	300
ASV51862.1	FGVGPLCKGDNLYLSAVDVCGMFTNRSGSQQWRGLSRYFKVQLRKRRVKNPYPISFLLTD	300
ASV51868.1	FGVGPLCKGDNLYLSAVDVCGMFTNRSGSQQWRGLSRYFKVQLRKRRVKNPYPISFLLTD	300
ASV51874.1	FGVGPLCKGDNLYLSAVDVCGMFTNRSGSQQWRGLSRYFKVQLRKRRVKNPYPISFLLTD	300
ASV51880.1	FGVGPLCKGDNLYLSAVDVCGMFTNRSGSQQWRGLSRYFKVQLRKRRVKNPYPISFLLTD	300
ASV51892.1	FGVGPLCKGDNLYLSAVDVCGMFTNRSGSQQWRGLSRYFKVQLRKRRVKNPYPISFLLTD	300
ANH21098.1	FGVGPLCKGDNLYLSAVDVCGMFTNRSGSQQWRGLSRYFKVQLRKRRVKNPYPISFLLTD	300
AID67150.1	FGVGPLCKGDNLYLSAVDVCGMFTNRSGSQQWRGLSRYFKVQLRKRRVKNPYPISFLLTD	300
AID67156.1	FGVGPLCKGDNLYLSAVDVCGMFTNRSGSQQWRGLSRYFKVQLRKRRVKNPYPISFLLTD	300
AID67162.1	FGVGPLCKGDNLYLSAVDVCGMFTNRSGSQQWRGLSRYFKVQLRKRRVKNPYPISFLLTD	300
AID67168.1	FGVGPLCKGDNLYLSAVDVCGMFTNRSGSQQWRGLSRYFKVQLRKRRVKNPYPISFLLTD	300
CCW72479.1	FGVGPLCKGDNLYLSAVDVCGMFTNRSGSQQWRGLSRYFKVQLRKRRVKNPYPISFLLTD	300
CCW72486.1	FGVGPLCKGDNLYLSAVDVCGMFTNRSGSQQWRGLSRYFKVQLRKRRVKNPYPISFLLTD	300
CCW72494.1	FGVGPLCKGDNLYLSAVDVCGMFTNRSGSQQWRGLSRYFKVQLRKRRVKNPYPISFLLTD	300
CCW72514.1	FGVGPLCKGDNLYLSAVDVCGMFTNRSGSQQWRGLSRYFKVQLRKRRVKNPYPISFLLTD	300
CCW72518.1	FGVGPLCKGDNLYLSAVDVCGMFTNRSGSQQWRGLSRYFKVQLRKRRVKNPYPISFLLTD	300
CCW72522.1	FGVGPLCKGDNLYLSAVDVCGMFTNRSGSQQWRGLSRYFKVQLRKRRVKNPYPISFLLTD	300
AFH57194.1	FGVGPLCKGDNLYLSAVDVCGMFTNRSGSQQWRGLSRYFKVQLRKRRVKNPYPISFLLTD	300
AEC15047.1	FGVGPLCKGDNLYLSAVDVCGMFTNRSGSQQWRGLSRYFKVQLRKRRVKNPYPISFLLTD	300

```
AEC15048.1      FGVGPLCKGDNLYLSAVDVCGMFTNRSGSQQWRGLSRYFKVQLRKRRVKNPYPISFLLTD 300
AEC15049.1      FGVGPLCKGDNLYLSAVDVCGMFTNRSGSQQWRGLSRYFKVQLRKRRVKNPYPISFLLTD 300
AEC15050.1      FGVGPLCKGDNLYLSAVDVCGMFTNRSGSQQWRGLSRYFKVQLRKRRVKNPYPISFLLTD 300
AEC15051.1      FGVGPLCKGDNLYLSAVDVCGMFTNRSGSQQWRGLSRYFKVQLRKRRVKNPYPISFLLTD 300
AEC15052.1      FGVGPLCKGDNLYLSAVDVCGMFTNRSGSQQWRGLSRYFKVQLRKRRVKNPYPISFLLTD 300
AEC15053.1      FGVGPLCKGDNLYLSAVDVCGMFTNRSGSQQWRGLSRYFKVQLRKRRVKNPYPISFLLTD 300
AEC15054.1      FGVGPLCKGDNLYLSAVDVCGMFTNRSGSQQWRGLSRYFKVQLRKRRVKNPYPISFLLTD 300
AEC15055.1      FGVGPLCKGDNLYLSAVDVCGMFTNRSGSQQWRGLSRYFKVQLRKRRVKNPYPISFLLTD 300
AEC15056.1      FGVGPLCKGDNLYLSAVDVCGMFTNRSGSQQWRGLSRYFKVQLRKRRVKNPYPISFLLTD 300
AEC15057.1      FGVGPLCKGDNLYLSAVDVCGMFTNRSGSQQWRGLSRYFKVQLRKRRVKNPYPISFLLTD 300
AEC15058.1      FGVGPLCKGDNLYLSAVDVCGMFTNRSGSQQWRGLSRYFKVQLRKRRVKNPYPISFLLTD 300
AEC15059.1      FGVGPLCKGDNLYLSAVDVCGMFTNRSGSQQWRGLSRYFKVQLRKRRVKNPYPISFLLTD 300
AEC15060.1      FGVGPLCKGDNLYLSAVDVCGMFTNRSGSQQWRGLSRYFKVQLRKRRVKNPYPISFLLTD 300
AEC15061.1      FGVGPLCKGDNLYLSAVDVCGMFTNRSGSQQWRGLSRYFKVQLRKRRVKNPYPISFLLTD 300
AEC15062.1      FGVGPLCKGDNLYLSAVDVCGMFTNRSGSQQWRGLSRYFKVQLRKRRVKNPYPISFLLTD 300
AEC15063.1      FGVGPLCKGDNLYLSAVDVCGMFTNRSGSQQWRGLSRYFKVQLRKRRVKNPYPISFLLTD 300
AEC15064.1      FGVGPLCKGDNLYLSAVDVCGMFTNRSGSQQWRGLSRYFKVQLRKRRVKNPYPISFLLTD 300
AEC15065.1      FGVGPLCKGDNLYLSAVDVCGMFTNRSGSQQWRGLSRYFKVQLRKRRVKNPYPISFLLTD 300
AEC15066.1      FGVGPLCKGDNLYLSAVDVCGMFTNRSGSQQWRGLSRYFKVQLRKRRVKNPYPISFLLTD 300
AEC15067.1      FGVGPLCKGDNLYLSAVDVCGMFTNRSGSQQWRGLSRYFKVQLRKRRVKNPYPISFLLTD 300
AEC15068.1      FGVGPLCKGDNLYLSAVDVCGMFTNRSGSQQWRGLSRYFKVQLRKRRVKNPYPISFLLTD 300
AEC15315.1      FGVGPLCKGDNLYLSAVDVCGMFTNRSGSQQWRGLSRYFKVQLRKRRVKNPYPISFLLTD 300
AEC15321.1      FGVGPLCKGDNLYLSAVDVCGMFTNRSGSQQWRGLSRYFKVQLRKRRVKNPYPISFLLTD 300
BAD21271.1      FGVGPLCKGDNLYLSAVDVCGMFTNRSGSQQWRGLSRYFKVQLRKRRVKNPYPISFLLTD 300
BAD21283.2      FGVGPLCKGDNLYLSAVDVCGMFTNRSGSQQWRGLSRYFKVQLRKRRVKNPYPISFLLTD 300
BAC15683.1      FGVGPLCKGDNLYLSAVDVCGMFTNRSGSQQWRGLSRYFKVQLRKRRVKNPYPISFLLTD 300
BAC15719.1      FGVGPLCKGDNLYLSAVDVCGMFTNRSGSQQWRGLSRYFKVQLRKRRVKNPYPISFLLTD 300
BAB93038.1      FGVGPLCKGDNLYLSAVDVCGMFTNRSGSQQWRGLSRYFKVQLRKRRVKNPYPISFLLTD 300
BAB93056.1      FGVGPLCKGDNLYLSAVDVCGMFTNRSGSQQWRGLSRYFKVQLRKRRVKNPYPISFLLTD 300
BAB93062.1      FGVGPLCKGDNLYLSAVDVCGMFTNRSGSQQWRGLSRYFKVQLRKRRVKNPYPISFLLTD 300
BAB68911.1      FGVGPLCKGDNLYLSAVDVCGMFTNRSGSQQWRGLSRYFKVQLRKRRVKNPYPISFLLTD 300
BAB68947.1      FGVGPLCKGDNLYLSAVDVCGMFTNRSGSQQWRGLSRYFKVQLRKRRVKNPYPISFLLTD 300
AAK70234.1      FGVGPLCKGDNLYLSAVDVCGMFTNRSGSQQWRGLSRYFKVQLRKRRVKNPYPISFLLTD 300
AAK70246.1      FGVGPLCKGDNLYLSAVDVCGMFTNRSGSQQWRGLSRYFKVQLRKRRVKNPYPISFLLTD 300
BAA01959.1      FGVGPLCKGDNLYLSAVDVCGMFTNRSGSQQWRGLSRYFKVQLRKRRVKNPYPISFLLTD 300
BAA01960.1      FGVGPLCKGDNLYLSAVDVCGMFTNRSGSQQWRGLSRYFKVQLRKRRVKNPYPISFLLTD 300
QWT77128.1      FGVGPLCKGDNLYLSAVDVCGMFTNRSGSQQWRGLSRYFKVQLRKRRVKNPYPISFLLTD 300
UPN63089.1      FGVGPLCKGDNLYLSAVDVCGMFTNRSGSQQWRGLSRYFKVQLRKRRVKNPYPISFLLTD 300
UPN63093.1      FGVGPLCKGDNLYLSAVDVCGMFTNRSGSQQWRGLSRYFKVQLRKRRVKNPYPISFLLTD 300
UPN63103.1      FGVGPLCKGDNLYLSAVDVCGMFTNRSGSQQWRGLSRYFKVQLRKRRVKNPYPISFLLTD 300
UPN63105.1      FGVGPLCKGDNLYLSAVDVCGMFTNRSGSQQWRGLSRYFKVQLRKRRVKNPYPISFLLTD 300
UPN63106.1      FGVGPLCKGDNLYLSAVDVCGMFTNRSGSQQWRGLSRYFKVQLRKRRVKNPYPISFLLTD 300
UPN63110.1      FGVGPLCKGDNLYLSAVDVCGMFTNRSGSQQWRGLSRYFKVQLRKRRVKNPYPISFLLTD 300
UPN63111.1      FGVGPLCKGDNLYLSAVDVCGMFTNRSGSQQWRGLSRYFKVQLRKRRVKNPYPISFLLTD 300
AEC15030.1      FGVGPLCKGDNLYLSAVDVCGMFTNRSGSQQWRGLSRYFKVQLRKRRVKNPYPISFLLTD 300
AEC15031.1      FGVGPLCKGDNLYLSAVDVCGMFTNRSGSQQWRGLSRYFKVQLRKRRVKNPYPISFLLTD 300
AEC15032.1      FGVGPLCKGDNLYLSAVDVCGMFTNRSGSQQWRGLSRYFKVQLRKRRVKNPYPISFLLTD 300
AEC15033.1      FGVGPLCKGDNLYLSAVDVCGMFTNRSGSQQWRGLSRYFKVQLRKRRVKNPYPISFLLTD 300
AEC15034.1      FGVGPLCKGDNLYLSAVDVCGMFTNRSGSQQWRGLSRYFKVQLRKRRVKNPYPISFLLTD 300
AEC15035.1      FGVGPLCKGDNLYLSAVDVCGMFTNRSGSQQWRGLSRYFKVQLRKRRVKNPYPISFLLTD 300
AEC15036.1      FGVGPLCKGDNLYLSAVDVCGMFTNRSGSQQWRGLSRYFKVQLRKRRVKNPYPISFLLTD 300
AEC15038.1      FGVGPLCKGDNLYLSAVDVCGMFTNRSGSQQWRGLSRYFKVQLRKRRVKNPYPISFLLTD 300
AEC15039.1      FGVGPLCKGDNLYLSAVDVCGMFTNRSGSQQWRGLSRYFKVQLRKRRVKNPYPISFLLTD 300
AEC15040.1      FGVGPLCKGDNLYLSAVDVCGMFTNRSGSQQWRGLSRYFKVQLRKRRVKNPYPISFLLTD 300
AEC15041.1      FGVGPLCKGDNLYLSAVDVCGMFTNRSGSQQWRGLSRYFKVQLRKRRVKNPYPISFLLTD 300
AEC15042.1      FGVGPLCKGDNLYLSAVDVCGMFTNRSGSQQWRGLSRYFKVQLRKRRVKNPYPISFLLTD 300
AEC15043.1      FGVGPLCKGDNLYLSAVDVCGMFTNRSGSQQWRGLSRYFKVQLRKRRVKNPYPISFLLTD 300
AEC15044.1      FGVGPLCKGDNLYLSAVDVCGMFTNRSGSQQWRGLSRYFKVQLRKRRVKNPYPISFLLTD 300
AEC15045.1      FGVGPLCKGDNLYLSAVDVCGMFTNRSGSQQWRGLSRYFKVQLRKRRVKNPYPISFLLTD 300
AEC15046.1      FGVGPLCKGDNLYLSAVDVCGMFTNRSGSQQWRGLSRYFKVQLRKRRVKNPYPISFLLTD 300
UPN63083.1      FGVGPLCKGDNLYLSAVDVCGMFTNRSGSQQWRGLSRYFKVQLRKRRVKNPYPISFLLTD 300
BAE00111.1      FGVGPLCKGDNLYLSAVDVCGMFTNRSGSQQWRGLSRYFKVQLRKRRVKNPYPISFLLTD 300
BAA01968.1      FGVGPLCKGDNLYLSAVDVCGMFTNRSGSQQWRGLSRYFKVQLRKRRVKNPYPISFLLTD 300
CCW72491.1      FGVGPLCKGDNLYLSAVDVCGMFTNRSGSQQWRGLSRYFKVQLRKRRVKNPYPISFLLTD 300
```

AEC14788.1	FGVGPLCKGDNLYLSAVDVCGMFTNRSGSQQWRGLSRYFKVQLRKRRVKNPYPISFLLTD	300
AEC14790.1	FGVGPLCKGDNLYLSAVDVCGMFTNRSGSQQWRGLSRYFKVQLRKRRVKNPYPISFLLTD	300
AEC14792.1	FGVGPLCKGDNLYLSAVDVCGMFTNRSGSQQWRGLSRYFKVQLRKRRVKNPYPISFLLTD	300
AEC14798.1	FGVGPLCKGDNLYLSAVDVCGMFTNRSGSQQWRGLSRYFKVQLRKRRVKNPYPISFLLTD	300
AEC14799.1	FGVGPLCKGDNLYLSAVDVCGMFTNRSGSQQWRGLSRYFKVQLRKRRVKNPYPISFLLTD	300
AEC14800.1	FGVGPLCKGDNLYLSAVDVCGMFTNRSGSQQWRGLSRYFKVQLRKRRVKNPYPISFLLTD	300
AEC14801.1	FGVGPLCKGDNLYLSAVDVCGMFTNRSGSQQWRGLSRYFKVQLRKRRVKNPYPISFLLTD	300
AEC14805.1	FGVGPLCKGDNLYLSAVDVCGMFTNRSGSQQWRGLSRYFKVQLRKRRVKNPYPISFLLTD	300
AEC14807.1	FGVGPLCKGDNLYLSAVDVCGMFTNRSGSQQWRGLSRYFKVQLRKRRVKNPYPISFLLTD	300
AEC14808.1	FGVGPLCKGDNLYLSAVDVCGMFTNRSGSQQWRGLSRYFKVQLRKRRVKNPYPISFLLTD	300
AEC14809.1	FGVGPLCKGDNLYLSAVDVCGMFTNRSGSQQWRGLSRYFKVQLRKRRVKNPYPISFLLTD	300
AEC14813.1	FGVGPLCKGDNLYLSAVDVCGMFTNRSGSQQWRGLSRYFKVQLRKRRVKNPYPISFLLTD	300
AEC14912.1	FGVGPLCKGDNLYLSAVDVCGMFTNRSGSQQWRGLSRYFKVQLRKRRVKNPYPISFLLTD	300
AEC14913.1	FGVGPLCKGDNLYLSAVDVCGMFTNRSGSQQWRGLSRYFKVQLRKRRVKNPYPISFLLTD	300
AEC14914.1	FGVGPLCKGDNLYLSAVDVCGMFTNRSGSQQWRGLSRYFKVQLRKRRVKNPYPISFLLTD	300
AEC14915.1	FGVGPLCKGDNLYLSAVDVCGMFTNRSGSQQWRGLSRYFKVQLRKRRVKNPYPISFLLTD	300
AEC14916.1	FGVGPLCKGDNLYLSAVDVCGMFTNRSGSQQWRGLSRYFKVQLRKRRVKNPYPISFLLTD	300
AEC14917.1	FGVGPLCKGDNLYLSAVDVCGMFTNRSGSQQWRGLSRYFKVQLRKRRVKNPYPISFLLTD	300
AEC14918.1	FGVGPLCKGDNLYLSAVDVCGMFTNRSGSQQWRGLSRYFKVQLRKRRVKNPYPISFLLTD	300
AEC14919.1	FGVGPLCKGDNLYLSAVDVCGMFTNRSGSQQWRGLSRYFKVQLRKRRVKNPYPISFLLTD	300
AEC14920.1	FGVGPLCKGDNLYLSAVDVCGMFTNRSGSQQWRGLSRYFKVQLRKRRVKNPYPISFLLTD	300
AEC14921.1	FGVGPLCKGDNLYLSAVDVCGMFTNRSGSQQWRGLSRYFKVQLRKRRVKNPYPISFLLTD	300
AEC14922.1	FGVGPLCKGDNLYLSAVDVCGMFTNRSGSQQWRGLSRYFKVQLRKRRVKNPYPISFLLTD	300
AEC14923.1	FGVGPLCKGDNLYLSAVDVCGMFTNRSGSQQWRGLSRYFKVQLRKRRVKNPYPISFLLTD	300
AEC14924.1	FGVGPLCKGDNLYLSAVDVCGMFTNRSGSQQWRGLSRYFKVQLRKRRVKNPYPISFLLTD	300
AEC14925.1	FGVGPLCKGDNLYLSAVDVCGMFTNRSGSQQWRGLSRYFKVQLRKRRVKNPYPISFLLTD	300
AEC14926.1	FGVGPLCKGDNLYLSAVDVCGMFTNRSGSQQWRGLSRYFKVQLRKRRVKNPYPISFLLTD	300
AEC14927.1	FGVGPLCKGDNLYLSAVDVCGMFTNRSGSQQWRGLSRYFKVQLRKRRVKNPYPISFLLTD	300
AEC14928.1	FGVGPLCKGDNLYLSAVDVCGMFTNRSGSQQWRGLSRYFKVQLRKRRVKNPYPISFLLTD	300
AEC14929.1	FGVGPLCKGDNLYLSAVDVCGMFTNRSGSQQWRGLSRYFKVQLRKRRVKNPYPISFLLTD	300
AEC14930.1	FGVGPLCKGDNLYLSAVDVCGMFTNRSGSQQWRGLSRYFKVQLRKRRVKNPYPISFLLTD	300
AEC14931.1	FGVGPLCKGDNLYLSAVDVCGMFTNRSGSQQWRGLSRYFKVQLRKRRVKNPYPISFLLTD	300
AEC14932.1	FGVGPLCKGDNLYLSAVDVCGMFTNRSGSQQWRGLSRYFKVQLRKRRVKNPYPISFLLTD	300
AEC14933.1	FGVGPLCKGDNLYLSAVDVCGMFTNRSGSQQWRGLSRYFKVQLRKRRVKNPYPISFLLTD	300
AEC14934.1	FGVGPLCKGDNLYLSAVDVCGMFTNRSGSQQWRGLSRYFKVQLRKRRVKNPYPISFLLTD	300
AEC14935.1	FGVGPLCKGDNLYLSAVDVCGMFTNRSGSQQWRGLSRYFKVQLRKRRVKNPYPISFLLTD	300
AEC14936.1	FGVGPLCKGDNLYLSAVDVCGMFTNRSGSQQWRGLSRYFKVQLRKRRVKNPYPISFLLTD	300
AEC14937.1	FGVGPLCKGDNLYLSAVDVCGMFTNRSGSQQWRGLSRYFKVQLRKRRVKNPYPISFLLTD	300
AEC14938.1	FGVGPLCKGDNLYLSAVDVCGMFTNRSGSQQWRGLSRYFKVQLRKRRVKNPYPISFLLTD	300
AEC14939.1	FGVGPLCKGDNLYLSAVDVCGMFTNRSGSQQWRGLSRYFKVQLRKRRVKNPYPISFLLTD	300
AEC14940.1	FGVGPLCKGDNLYLSAVDVCGMFTNRSGSQQWRGLSRYFKVQLRKRRVKNPYPISFLLTD	300
AEC14941.1	FGVGPLCKGDNLYLSAVDVCGMFTNRSGSQQWRGLSRYFKVQLRKRRVKNPYPISFLLTD	300
AEC14942.1	FGVGPLCKGDNLYLSAVDVCGMFTNRSGSQQWRGLSRYFKVQLRKRRVKNPYPISFLLTD	300
AEC14943.1	FGVGPLCKGDNLYLSAVDVCGMFTNRSGSQQWRGLSRYFKVQLRKRRVKNPYPISFLLTD	300
AEC14944.1	FGVGPLCKGDNLYLSAVDVCGMFTNRSGSQQWRGLSRYFKVQLRKRRVKNPYPISFLLTD	300
AEC14945.1	FGVGPLCKGDNLYLSAVDVCGMFTNRSGSQQWRGLSRYFKVQLRKRRVKNPYPISFLLTD	300
AEC14946.1	FGVGPLCKGDNLYLSAVDVCGMFTNRSGSQQWRGLSRYFKVQLRKRRVKNPYPISFLLTD	300
AEC14947.1	FGVGPLCKGDNLYLSAVDVCGMFTNRSGSQQWRGLSRYFKVQLRKRRVKNPYPISFLLTD	300
AEC14948.1	FGVGPLCKGDNLYLSAVDVCGMFTNRSGSQQWRGLSRYFKVQLRKRRVKNPYPISFLLTD	300
AEC14949.1	FGVGPLCKGDNLYLSAVDVCGMFTNRSGSQQWRGLSRYFKVQLRKRRVKNPYPISFLLTD	300
AEC14950.1	FGVGPLCKGDNLYLSAVDVCGMFTNRSGSQQWRGLSRYFKVQLRKRRVKNPYPISFLLTD	300
AEC14951.1	FGVGPLCKGDNLYLSAVDVCGMFTNRSGSQQWRGLSRYFKVQLRKRRVKNPYPISFLLTD	300
AEC14952.1	FGVGPLCKGDNLYLSAVDVCGMFTNRSGSQQWRGLSRYFKVQLRKRRVKNPYPISFLLTD	300
AEC14953.1	FGVGPLCKGDNLYLSAVDVCGMFTNRSGSQQWRGLSRYFKVQLRKRRVKNPYPISFLLTD	300
AEC14954.1	FGVGPLCKGDNLYLSAVDVCGMFTNRSGSQQWRGLSRYFKVQLRKRRVKNPYPISFLLTD	300
AEC14955.1	FGVGPLCKGDNLYLSAVDVCGMFTNRSGSQQWRGLSRYFKVQLRKRRVKNPYPISFLLTD	300
AEC14956.1	FGVGPLCKGDNLYLSAVDVCGMFTNRSGSQQWRGLSRYFKVQLRKRRVKNPYPISFLLTD	300
AEC14957.1	FGVGPLCKGDNLYLSAVDVCGMFTNRSGSQQWRGLSRYFKVQLRKRRVKNPYPISFLLTD	300
AEC14958.1	FGVGPLCKGDNLYLSAVDVCGMFTNRSGSQQWRGLSRYFKVQLRKRRVKNPYPISFLLTD	300
AEC14959.1	FGVGPLCKGDNLYLSAVDVCGMFTNRSGSQQWRGLSRYFKVQLRKRRVKNPYPISFLLTD	300
AEC14960.1	FGVGPLCKGDNLYLSAVDVCGMFTNRSGSQQWRGLSRYFKVQLRKRRVKNPYPISFLLTD	300
AEC14961.1	FGVGPLCKGDNLYLSAVDVCGMFTNRSGSQQWRGLSRYFKVQLRKRRVKNPYPISFLLTD	300
QWT77122.1	FGVGPLCKGDNLYLSAVDVCGMFTYRSGSQQWRGLSRYFKVQLRKRRVKNPYPISFLLTD	300
UPN63084.1	FGVGPLCKGDNLYLSAVDVCGMFTNRSGSQKWRGLSRYFKVQLRKRRVKNPYPISFLLTD	300

UPN63101.1	FGVGPLCKGDNLYLSAVDVCGMFTNRSGSQQWRGLSRYFKVQLRKRRVKNPYPISFLLTD	300
ASV51886.1	FGVGPLCKGDNLYLSAVDVCGMFTNRSGSQQWRGLSRYFKVQLRKRRVKNPYPISFLLTD	300
CCW72484.1	FGVGPLCKGDNLYLSAVDVCGMFTNRSGSQQWRGLSRYFKVQLRKRRVKNPYPISFLLTD	300
ANH21099.1	FGVGPLCKGDNLYLSAVDVCGMFTNRSGFQQWRGLSRYFKVQLRKRRVKNPYPISFLLTD	300
AEC15112.1	FGVGPLCKGDNLYLSAVDVCGMFTNRSGFQQWRGLSRYFKVQLRKRRVKNPYPISFLLTD	300
AEC15113.1	FGVGPLCKGDNLYLSAVDVCGMFTNRSGFQQWRGLSRYFKVQLRKRRVKNPYPISFLLTD	300
AEC15114.1	FGVGPLCKGDNLYLSAVDVCGMFTNRSGFQQWRGLSRYFKVQLRKRRVKNPYPISFLLTD	300
AEC15115.1	FGVGPLCKGDNLYLSAVDVCGMFTNRSGFQQWRGLSRYFKVQLRKRRVKNPYPISFLLTD	300
AEC15116.1	FGVGPLCKGDNLYLSAVDVCGMFTNRSGFQQWRGLSRYFKVQLRKRRVKNPYPISFLLTD	300
AEC15117.1	FGVGPLCKGDNLYLSAVDVCGMFTNRSGFQQWRGLSRYFKVQLRKRRVKNPYPISFLLTD	300
AEC15819.1	FGVGPLCKGDNLYLSAVDVCGMFTNRSGFQQWRGLSRYFKVQLRKRRVKNPYPISFLLTD	300
AEC15825.1	FGVGPLCKGDNLYLSAVDVCGMFTNRSGFQQWRGLSRYFKVQLRKRRVKNPYPISFLLTD	300
AEC15831.1	FGVGPLCKGDNLYLSAVDVCGMFTNRSGFQQWRGLSRYFKVQLRKRRVKNPYPISFLLTD	300
AEC15837.1	FGVGPLCKGDNLYLSAVDVCGMFTNRSGFQQWRGLSRYFKVQLRKRRVKNPYPISFLLTD	300
AEC15843.1	FGVGPLCKGDNLYLSAVDVCGMFTNRSGFQQWRGLSRYFKVQLRKRRVKNPYPISFLLTD	300
AEC15849.1	FGVGPLCKGDNLYLSAVDVCGMFTNRSGFQQWRGLSRYFKVQLRKRRVKNPYPISFLLTD	300
AEC15855.1	FGVGPLCKGDNLYLSAVDVCGMFTNRSGFQQWRGLSRYFKVQLRKRRVKNPYPISFLLTD	300
CCW72511.1	FGVGPLCKGDNLYLSAVDVCGMFTNRSGSQQWRGLSRYFKVQLRKRRVKNPYPISFLLTD	300
CCW72525.1	FGVGPLCKGDNLYLSAVDVCGMFTNRSGSQQWRGLSRYFKVQLRKRRVKNPYPISFLLTD	300
AEC14744.1	FGVGPLCKGDNLYLSAVDVCGMFTNRSGSQQWRGLSRYFKVQLRKRRVKNPYPISFLLTD	300
AEC14746.1	FGVGPLCKGDNLYLSAVDVCGMFTNRSGSQQWRGLSRYFKVQLRKRRVKNPYPISFLLTD	300
AEC14747.1	FGVGPLCKGDNLYLSAVDVCGMFTNRSGSQQWRGLSRYFKVQLRKRRVKNPYPISFLLTD	300
AEC14748.1	FGVGPLCKGDNLYLSAVDVCGMFTNRSGSQQWRGLSRYFKVQLRKRRVKNPYPISFLLTD	300
AEC14752.1	FGVGPLCKGDNLYLSAVDVCGMFTNRSGSQQWRGLSRYFKVQLRKRRVKNPYPISFLLTD	300
AEC14756.1	FGVGPLCKGDNLYLSAVDVCGMFTNRSGSQQWRGLSRYFKVQLRKRRVKNPYPISFLLTD	300
AEC14757.1	FGVGPLCKGDNLYLSAVDVCGMFTNRSGSQQWRGLSRYFKVQLRKRRVKNPYPISFLLTD	300
AEC14758.1	FGVGPLCKGDNLYLSAVDVCGMFTNRSGSQQWRGLSRYFKVQLRKRRVKNPYPISFLLTD	300
AEC14764.1	FGVGPLCKGDNLYLSAVDVCGMFTNRSGSQQWRGLSRYFKVQLRKRRVKNPYPISFLLTD	300
AEC14768.1	FGVGPLCKGDNLYLSAVDVCGMFTNRSGSQQWRGLSRYFKVQLRKRRVKNPYPISFLLTD	300
AEC14769.1	FGVGPLCKGDNLYLSAVDVCGMFTNRSGSQQWRGLSRYFKVQLRKRRVKNPYPISFLLTD	300
AEC14770.1	FGVGPLCKGDNLYLSAVDVCGMFTNRSGSQQWRGLSRYFKVQLRKRRVKNPYPISFLLTD	300
AEC14771.1	FGVGPLCKGDNLYLSAVDVCGMFTNRSGSQQWRGLSRYFKVQLRKRRVKNPYPISFLLTD	300
AEC14772.1	FGVGPLCKGDNLYLSAVDVCGMFTNRSGSQQWRGLSRYFKVQLRKRRVKNPYPISFLLTD	300
AEC14773.1	FGVGPLCKGDNLYLSAVDVCGMFTNRSGSQQWRGLSRYFKVQLRKRRVKNPYPISFLLTD	300
AEC14775.1	FGVGPLCKGDNLYLSAVDVCGMFTNRSGSQQWRGLSRYFKVQLRKRRVKNPYPISFLLTD	300
AEC14777.1	FGVGPLCKGDNLYLSAVDVCGMFTNRSGSQQWRGLSRYFKVQLRKRRVKNPYPISFLLTD	300
AEC14778.1	FGVGPLCKGDNLYLSAVDVCGMFTNRSGSQQWRGLSRYFKVQLRKRRVKNPYPISFLLTD	300
AEC14779.1	FGVGPLCKGDNLYLSAVDVCGMFTNRSGSQQWRGLSRYFKVQLRKRRVKNPYPISFLLTD	300
AEC14782.1	FGVGPLCKGDNLYLSAVDVCGMFTNRSGSQQWRGLSRYFKVQLRKRRVKNPYPISFLLTD	300
AEC14814.1	FGVGPLCKGDNLYLSAVDVCGMFTNRSGSQQWRGLSRYFKVQLRKRRVKNPYPISFLLTD	300
AEC14815.1	FGVGPLCKGDNLYLSAVDVCGMFTNRSGSQQWRGLSRYFKVQLRKRRVKNPYPISFLLTD	300
AEC14816.1	FGVGPLCKGDNLYLSAVDVCGMFTNRSGSQQWRGLSRYFKVQLRKRRVKNPYPISFLLTD	300
AEC14817.1	FGVGPLCKGDNLYLSAVDVCGMFTNRSGSQQWRGLSRYFKVQLRKRRVKNPYPISFLLTD	300
AEC14818.1	FGVGPLCKGDNLYLSAVDVCGMFTNRSGSQQWRGLSRYFKVQLRKRRVKNPYPISFLLTD	300
AEC14819.1	FGVGPLCKGDNLYLSAVDVCGMFTNRSGSQQWRGLSRYFKVQLRKRRVKNPYPISFLLTD	300
AEC14820.1	FGVGPLCKGDNLYLSAVDVCGMFTNRSGSQQWRGLSRYFKVQLRKRRVKNPYPISFLLTD	300
AEC14822.1	FGVGPLCKGDNLYLSAVDVCGMFTNRSGSQQWRGLSRYFKVQLRKRRVKNPYPISFLLTD	300
AEC14823.1	FGVGPLCKGDNLYLSAVDVCGMFTNRSGSQQWRGLSRYFKVQLRKRRVKNPYPISFLLTD	300
AEC14824.1	FGVGPLCKGDNLYLSAVDVCGMFTNRSGSQQWRGLSRYFKVQLRKRRVKNPYPISFLLTD	300
AEC14825.1	FGVGPLCKGDNLYLSAVDVCGMFTNRSGSQQWRGLSRYFKVQLRKRRVKNPYPISFLLTD	300
AEC14826.1	FGVGPLCKGDNLYLSAVDVCGMFTNRSGSQQWRGLSRYFKVQLRKRRVKNPYPISFLLTD	300
AEC14827.1	FGVGPLCKGDNLYLSAVDVCGMFTNRSGSQQWRGLSRYFKVQLRKRRVKNPYPISFLLTD	300
AEC14828.1	FGVGPLCKGDNLYLSAVDVCGMFTNRSGSQQWRGLSRYFKVQLRKRRVKNPYPISFLLTD	300
AEC14829.1	FGVGPLCKGDNLYLSAVDVCGMFTNRSGSQQWRGLSRYFKVQLRKRRVKNPYPISFLLTD	300
AEC14830.1	FGVGPLCKGDNLYLSAVDVCGMFTNRSGSQQWRGLSRYFKVQLRKRRVKNPYPISFLLTD	300
AEC14831.1	FGVGPLCKGDNLYLSAVDVCGMFTNRSGSQQWRGLSRYFKVQLRKRRVKNPYPISFLLTD	300
AEC14832.1	FGVGPLCKGDNLYLSAVDVCGMFTNRSGSQQWRGLSRYFKVQLRKRRVKNPYPISFLLTD	300
AEC14833.1	FGVGPLCKGDNLYLSAVDVCGMFTNRSGSQQWRGLSRYFKVQLRKRRVKNPYPISFLLTD	300
AEC14834.1	FGVGPLCKGDNLYLSAVDVCGMFTNRSGSQQWRGLSRYFKVQLRKRRVKNPYPISFLLTD	300
AEC14835.1	FGVGPLCKGDNLYLSAVDVCGMFTNRSGSQQWRGLSRYFKVQLRKRRVKNPYPISFLLTD	300
AEC14836.1	FGVGPLCKGDNLYLSAVDVCGMFTNRSGSQQWRGLSRYFKVQLRKRRVKNPYPISFLLTD	300
AEC14837.1	FGVGPLCKGDNLYLSAVDVCGMFTNRSGSQQWRGLSRYFKVQLRKRRVKNPYPISFLLTD	300
AEC14838.1	FGVGPLCKGDNLYLSAVDVCGMFTNRSGSQQWRGLSRYFKVQLRKRRVKNPYPISFLLTD	300
AEC14839.1	FGVGPLCKGDNLYLSAVDVCGMFTNRSGSQQWRGLSRYFKVQLRKRRVKNPYPISFLLTD	300

```
AEC14840.1        FGVGPLCKGDNLYLSAVDVCGMFTNRSGSQQWRGLSRYFKVQLRKRRVKNPYPISFLLTD  300
AEC14841.1        FGVGPLCKGDNLYLSAVDVCGMFTNRSGSQQWRGLSRYFKVQLRKRRVKNPYPISFLLTD  300
AEC14842.1        FGVGPLCKGDNLYLSAVDVCGMFTNRSGSQQWRGLSRYFKVQLRKRRVKNPYPISFLLTD  300
AEC14843.1        FGVGPLCKGDNLYLSAVDVCGMFTNRSGSQQWRGLSRYFKVQLRKRRVKNPYPISFLLTD  300
AEC14844.1        FGVGPLCKGDNLYLSAVDVCGMFTNRSGSQQWRGLSRYFKVQLRKRRVKNPYPISFLLTD  300
AEC14845.1        FGVGPLCKGDNLYLSAVDVCGMFTNRSGSQQWRGLSRYFKVQLRKRRVKNPYPISFLLTD  300
AEC14846.1        FGVGPLCKGDNLYLSAVDVCGMFTNRSGSQQWRGLSRYFKVQLRKRRVKNPYPISFLLTD  300
AEC14847.1        FGVGPLCKGDNLYLSAVDVCGMFTNRSGSQQWRGLSRYFKVQLRKRRVKNPYPISFLLTD  300
AEC14848.1        FGVGPLCKGDNLYLSAVDVCGMFTNRSGSQQWRGLSRYFKVQLRKRRVKNPYPISFLLTD  300
AEC14849.1        FGVGPLCKGDNLYLSAVDVCGMFTNRSGSQQWRGLSRYFKVQLRKRRVKNPYPISFLLTD  300
AEC14850.1        FGVGPLCKGDNLYLSAVDVCGMFTNRSGSQQWRGLSRYFKVQLRKRRVKNPYPISFLLTD  300
AEC14851.1        FGVGPLCKGDNLYLSAVDVCGMFTNRSGSQQWRGLSRYFKVQLRKRRVKNPYPISFLLTD  300
AEC14852.1        FGVGPLCKGDNLYLSAVDVCGMFTNRSGSQQWRGLSRYFKVQLRKRRVKNPYPISFLLTD  300
AEC14853.1        FGVGPLCKGDNLYLSAVDVCGMFTNRSGSQQWRGLSRYFKVQLRKRRVKNPYPISFLLTD  300
AEC14854.1        FGVGPLCKGDNLYLSAVDVCGMFTNRSGSQQWRGLSRYFKVQLRKRRVKNPYPISFLLTD  300
AEC14855.1        FGVGPLCKGDNLYLSAVDVCGMFTNRSGSQQWRGLSRYFKVQLRKRRVKNPYPISFLLTD  300
AEC14856.1        FGVGPLCKGDNLYLSAVDVCGMFTNRSGSQQWRGLSRYFKVQLRKRRVKNPYPISFLLTD  300
AEC14857.1        FGVGPLCKGDNLYLSAVDVCGMFTNRSGSQQWRGLSRYFKVQLRKRRVKNPYPISFLLTD  300
AEC14858.1        FGVGPLCKGDNLYLSAVDVCGMFTNRSGSQQWRGLSRYFKVQLRKRRVKNPYPISFLLTD  300
AEC14859.1        FGVGPLCKGDNLYLSAVDVCGMFTNRSGSQQWRGLSRYFKVQLRKRRVKNPYPISFLLTD  300
AEC14862.1        FGVGPLCKGDNLYLSAVDVCGMFTNRSGSQQWRGLSRYFKVQLRKRRVKNPYPISFLLTD  300
AEC14863.1        FGVGPLCKGDNLYLSAVDVCGMFTNRSGSQQWRGLSRYFKVQLRKRRVKNPYPISFLLTD  300
BAD21289.1        FGVGPLCKGDNLYLSAVDVCGMFTNRSGSQQWRGLSRYFKVQLRKRRVKNPYPISFLLTD  300
BAD21295.1        FGVGPLCKGDNLYLSAVDVCGMFTNRSGSQQWRGLSRYFKVQLRKRRVKNPYPISFLLTD  300
BAB93044.1        FGVGPLCKGDNLYLSAVDVCGMFTNRSGSQQWRGLSRYFKVQLRKRRVKNPYPISFLLTD  300
BAB93104.1        FGVGPLCKGDNLYLSAVDVCGMFTNRSGSQQWRGLSRYFKVQLRKRRVKNPYPISFLLTD  300
AAK70252.1        FGVGPLCKGDNLYLSAADVCGMFTNRSGSQQWRGLSRYFKVQLRKRRVKNPYPISFLLTD  300
BAB68959.1        FGVGPLCKGDNLYLSAVDVCGMFTNRSGSQQWRGLSRYFKVQLRKRRVKNPYPISFLLTD  300
BAD21229.1        FGVGPLCKGDNLYLSAVDVCGMFTNRSGTQQWRGLSRYFKVQLRKRRVKNPYPISFLLTD  300
QBX89123.1        FGVGPLCKGDNLYLSAVDVCGMFTNRSGSQQWRGLSRYFKVQLRKRRVKNPYPISFLLTD  300
BAE02908.1        FGVGPLCKGDNLYLSAVDVCGMFTNRSGSQQWRGLSRYFKVQLRKRRVKNPYPISFLLTD  300
AAR89259.1        FGVGPLCKGDNLYLSAVDVCGMFTNRSGSQQWRGLSRYFKVQLRKRRVKNPYPISFLLTD  300
AAR12957.1        FGVGPLCKGDNLYLSAVDVCGMFTNRSGSQQWRGLSRYFKVQLRKRRVKNPYPISFLLTD  300
AAR13077.1        FGVGPLCKGDNLYLSAVDVCGMFTNRSGSQQWRGLSRYFKVQLRKRRVKNPYPISFLLTD  300
BCU41036.1        FGVGPLCKGDNLYLSAVDVCGMFTKRSGSQQWRGLSRYFKVQLRKRRVKNPYPISFLLTD  300
BAE94726.1        FGVGPLCKGDNLYLSAVDVCGMFTNRSGFQQWRGLSRYFKVQLRKRRVKNPYPISFLLTD  300
AAB94036.1        FGVGPLCKGDNLYLSAVDVCGMFTNRSGSQQWRGLSRYFKVQLRKRRVKNPYPISFLLTD  300
AAK28478.1        FGVGPLCKGDNLYLSAVDVCGMFTNRSGAQQWRGLSRYFKVQLRKRRVKNPYPISFLLTD  300
BAB93008.1        FGVGPLCKGDNLYLSAVDVCGMFTNRSGSQQWRGLSRYFKVQLRKRRVKNPYPISFLLTD  300
AAM69579.1        FGVGPLCKGDNLYLSAVDVCGMFTNRSGSQQWRGLSRYFKVQLRKRRVKNPYPISFLLTD  300
AAM69584.1        FGVGPLCKGDNLYLSAVDVCGMFTNRSGSQQWRGLSRYFKVQLRKRRVKNPYPISFLLTD  300
AAM69596.1        FGVGPLCKGDNLYLSAVDVCGMFTNRSGSQQWRGLSRYFKVQLRKRRVKNPYPISFLLTD  300
AAM69602.1        FGVGPLCKGDNLYLSAVDVCGMFTNRSGSQQWRGLSRYFKVQLRKRRVKNPYPISFLLTD  300
AAM69608.1        FGVGPLCKGDNLYLSAVDVCGMFTNRSGSQQWRGLSRYFKVQLRKRRVKNPYPISFLLTD  300
AAK70312.1        FGVGPLCKGDNLYLSAVDVCGMFTNRSGSQQWRGLSRYFKVQLRKRRVKNPYPISFLLTD  300
WLD48064.1        FGVGPLCKGDNLYLSAVDVCGMFTNRSGSQQWRGLSRYFKVQLRKRRVKNPYPISFLLTD  300
WLD48100.1        FGVGPLCKGDNLYLSAVDVCGMFTNRSGSQQWRGLSRYFKVQLRKRRVKNPYPISFLLTD  300
UQK62655.1        FGVGPLCKGDNLYLSAVDVCGMFTNRSGSQQWRGLSRYFKVQLRKRRVKNPYPISFLLTD  300
QTJ15180.1        FGVGPLCKGDNLYLSAVDVCGMFTNRSGSQQWRGLSRYFKVQLRKRRVKNPYPISFLLTD  300
QTJ15204.1        FGVGPLCKGDNLYLSAVDVCGMFTNRSGSQQWRGLSRYFKVQLRKRRVKNPYPISFLLTD  300
ASV51784.1        FGVGPLCKGDNLYLSAVDVCGMFTNRSGSQQWRGLSRYFKVQLRKRRVKNPYPISFLLTD  300
ASV51790.1        FGVGPLCKGDNLYLSAVDVCGMFTNRSGSQQWRGLSRYFKVQLRKRRVKNPYPISFLLTD  300
ASV51898.1        FGVGPLCKGDNLYLSAVDVCGMFTNRSGSQQWRGLSRYFKVQLRKRRVKNPYPISFLLTD  300
ASV51904.1        FGVGPLCKGDNLYLSAVDVCGMFTNRSGSQQWRGLSRYFKVQLRKRRVKNPYPISFLLTD  300
ASV51910.1        FGVGPLCKGDNLYLSAVDVCGMFTNRSGSQQWRGLSRYFKVQLRKRRVKNPYPISFLLTD  300
ASV51916.1        FGVGPLCKGDNLYLSAVDVCGMFTNRSGSQQWRGLSRYFKVQLRKRRVKNPYPISFLLTD  300
CCW72490.1        FGVGPLCKGDNLYLSAVDVCGMFTNRSGSQQWRGLSRYFKVQLRKRRVKNPYPISFLLTD  300
CCW72495.1        FGVGPLCKGDNLYLSAVDVCGMFTNRSGSQQWRGLSRYFKVQLRKRRVKNPYPISFLLTD  300
CCW72497.1        FGVGPLCKGDNLYLSAVDVCGMFTNRSGSQQWRGLSRYFKVQLRKRRVKNPYPISFLLTD  300
CCW72503.1        FGVGPLCKGDNLYLSAVDVCGMFTNRSGSQQWRGLSRYFKVQLRKRRVKNPYPISFLLTD  300
CCW72505.1        FGVGPLCKGDNLYLSAVDVCGMFTNRSGSQQWRGLSRYFKVQLRKRRVKNPYPISFLLTD  300
CCW72507.1        FGVGPLCKGDNLYLSAVDVCGMFTNRSGSQQWRGLSRYFKVQLRKRRVKNPYPISFLLTD  300
CCW72512.1        FGVGPLCKGDNLYLSAVDVCGMFTNRSGSQQWRGLSRYFKVQLRKRRVKNPYPISFLLTD  300
CCW72515.1        FGVGPLCKGDNLYLSAVDVCGMFTNRSGSQQWRGLSRYFKVQLRKRRVKNPYPISFLLTD  300
```

BAB68881.1	FGVGPLCKGDNLYLSAVDVCGMFTNRSGSQQWRGLSRYFKVQLRKRRVKNPYPISFLLTD	300
BAB68887.1	FGVGPLCKGDNLYLSAVDVCGMFTNRSGSQQWRGLSRYFKVQLRKRRVKNPYPISFLLTD	300
AAK70294.1	FGVGPLCKGDNLYLSAVDVCGMFTNRSGSQQWRGLSRYFKVQLRKRRVKNPYPISFLLTD	300
WLD48094.1	FGVGPLCKGDNLYLSAVDVCGMFTNRSGSQQWRGLSRYFKVQLRKRRVKNPYPISFLLTD	300
CCW72509.1	FGVGPLCKGDNLYLSAVDVCGMFTNRSGSQQWRGLSRYFKVQLRKRRVKNPYPISFLLTD	300
BAB68893.1	FGVGPLCKGDNLYLSAVDVCGMFTNRSGSQQWRGLSRYFKVQLRKRRVKNPYPISFLLTD	300
QTJ15216.1	FGVGPLCKGDNLYLSAVDVCGMFTNRSGSQQWRGLSRYFKVQLRKRRVKNPYPISFLLTD	300
CCW72493.1	FGVGPLCKGDNLYLSAVDVCGMFTNRSGSQQWRGLSRYFKVQLRKRRVKNPYPISFLLTD	300
CCW72500.1	FGVGPLCKGDNLYLSAVDVCGMFTNRSGSQQWRGLSRYFKVQLRKRRVKNPYPISFLLTD	300
CCW72506.1	FGVGPLCKGDNLYLSAVDVCGMFTNRSGSQQWRGLSRYFKVQLRKRRVKNPYPISFLLTD	300
CCW72520.1	FGVGPLCKGDNLYLSAVDVCGMFTNRSGSQQWRGLSRYFKVQLRKRRVKNPYPISFLLTD	300
BAB68899.1	FGVGPLCKGDNLYLSAVDVCGMFTNRSGSQQWRGLSRYFKVQLRKRRVKNPYPISFLLTD	300
BAB68905.1	FGVGPLCKGDNLYLSAVDVCGMFTNRSGSQQWRGLSRYFKVQLRKRRVKNPYPISFLLTD	300
AAK70300.1	FGVGPLCKGDNLYLSAVDVCGMFTNRSGSQQWRGLSRYFKVQLRKRRVKNPYPISFLLTD	300
AYM55579.1	FGVGPLCKGDNLYLSAVDVCGMFTNRSGSQQWRGLSRYFKVQLRKRRVKNPYPISFLLTD	300
AYM55580.1	FGVGPLCKGDNLYLSAVDVCGMFTNRSGSQQWRGLSRYFKVQLRKRRVKNPYPISFLLTD	300
AYM55582.1	FGVGPLCKGDNLYLSAVDVCGMFTNRSGSQQWRGLSRYFKVQLRKRRVKNPYPISFLLTD	300
AYM55583.1	FGVGPLCKGDNLYLSAVDVCGMFTNRSGSQQWRGLSRYFKVQLRKRRVKNPYPISFLLTD	300
AYM55584.1	FGVGPLCKGDNLYLSAVDVCGMFTNRSGSQQWRGLSRYFKVQLRKRRVKNPYPISFLLTD	300
AYM55590.1	FGVGPLCKGDNLYLSAVDVCGMFTNRSGSQQWRGLSRYFKVQLRKRRVKNPYPISFLLTD	300
AYM55596.1	FGVGPLCKGDNLYLSAVDVCGMFTNRSGSQQWRGLSRYFKVQLRKRRVKNPYPISFLLTD	300
BAE45366.1	FGVGPLCKGDNLYLSAVDVCGMFTNRSGSQQWRGLSRYFKVQLRKRRVKNPYPISFLLTD	300
AAG30857.1	FGVGPLCKGDNLYLSAVDVCGMFTNRSGSQQWRGLSRYFKVQLRKRRVKNPYPISFLLTD	300
BAH36774.1	FGVGPLCKGDNLYLSAVDVCGMFTNRSGSQQWRGLSRYFKVQLRKRRVKNPYPISFLLTD	300
BAH36780.1	FGVGPLCKGDNLYLSAVDVCGMFTNRSGSQQWRGLSRYFKVQLRKRRVKNPYPISFLLTD	300
BAH36822.1	FGVGPLCKGDNLYLSAVDVCGMFTNRSGSQQWRGLSRYFKVQLRKRRVKNPYPISFLLTD	300
BAH36834.1	FGVGPLCKGDNLYLSAVDVCGMFTNRSGSQQWRGLSRYFKVQLRKRRVKNPYPISFLLTD	300
BAH36840.1	FGVGPLCKGDNLYLSAVDVCGMFTNRSGSQQWRGLSRYFKVQLRKRRVKNPYPISFLLTD	300
BAH36846.1	FGVGPLCKGDNLYLSAVDVCGMFTNRSGSQQWRGLSRYFKVQLRKRRVKNPYPISFLLTD	300
BAH36816.1	FGVGPLCKGDNLYLSAVDVCGMFTNRSGSQQWRGLSRYFKVQLRKRRVKNPYPISFLLTD	300
BAE45444.1	FGVGPLCKGDNLYLSAVDVCGMFTNRSGSQQWRGLSRYFKVQLRKRRVKNPYPISFLLTD	300
AAT09819.1	FGVGPLCKGDNLYLSAVDVCGMFTTRSGSQQWRGLSRYFKVQLRKRRVKNPYPISFLLTD	300
BAC03029.1	YGVGPLCKGDNLYLSAVDVCGMFTNRSGSQQWRGLSRYFKVQLRKRRVKNPYPISFLLTD	300
BAC03095.1	FGVGPLCKGDNLYLSAVDVCGMFTNRSGSQQWRGLSRYFKVQLRKRRVKNPYPISFLLTD	300
AAM69560.1	FGVGPLCKGDNLYLSAVDVCGTFTNRSGSQQWRGLSRYFKVQLRKRRVKNPYPISFLLTD	300
AAM69566.1	FGVGPLCKGDNLYLSAVDVCGMFTNRSGSQQWRGLSRYFKVQLRKRRVKNPYPISFLLTD	300
AAM69572.1	FGVGPLCKGDNLYLSAVDVCGMFTNRSGSQQWRGLSRYFKVQLRKRRVKNPYPISFLLTD	300
AAB84311.1	FGVGPLCKGDNLYLSAVDVCGMFTNRSGSQQWRGLSRYFKVQLRKRRVKNPYPISFLLTD	300
BAD06150.1	FGVGPLCKGDNLYLSAVDVCGMFTNRSGSQQWRGLSRYFKVQLRKRKVKNPYPISFLLTD	300
AYM55581.1	FGVGPLCKGDNLYLSAVDVCGMFTNRSGSQQWRGLSRYFKVQLRKRRVKNPYPISFLLTD	300
AYM55588.1	FGVGPLCKGDNLYLSAVDVCGMFTNRSGSQQWRGLSRYFKVQLRKRRVKNPYPISFLLTD	300
AYM55589.1	FGVGPLCKGDNLYLSAVDVCGMFTNRSGSQQWRGLSRYFKVQLRKRRVKNPYPISFLLTD	300
AYM55591.1	FGVGPLCKGDNLYLSAVDVCGMFTNRSGSQQWRGLSRYFKVQLRKRRVKNPYPISFLLTD	300
AYM55592.1	FGVGPLCKGDNLYLSAVDVCGMFTNRSGSQQWRGLSRYFKVQLRKRRVKNPYPISFLLTD	300
AYM55593.1	FGVGPLCKGDNLYLSAVDVCGMFTNRSGSQQWRGLSRYFKVQLRKRRVKNPYPISFLLTD	300
AYM55595.1	FGVGPLCKGDNLYLSAVDVCGMFTNRSGSQQWRGLSRYFKVQLRKRRVKNPYPISFLLTD	300
AYM55597.1	FGVGPLCKGDNLYLSAVDVCGMFTNRSGSQQWRGLSRYFKVQLRKRRVKNPYPISFLLTD	300
AYM55598.1	FGVGPLCKGDNLYLSAVDVCGMFTNRSGSQQWRGLSRYFKVQLRKRRVKNPYPISFLLTD	300
AYM55599.1	FGVGPLCKGDNLYLSAVDVCGMFTNRSGSQQWRGLSRYFKVQLRKRRVKNPYPISFLLTD	300
AYM55600.1	FGVGPLCKGDNLYLSAVDVCGMFTNRSGSQQWRGLSRYFKVQLRKRRVKNPYPISFLLTD	300
AYM55601.1	FGVGPLCKGDNLYLSAVDVCGMFTNRSGSQQWRGLSRYFKVQLRKRRVKNPYPISFLLTD	300
AYM55602.1	FGVGPLCKGDNLYLSAVDVCGMFTNRSGSQQWRGLSRYFKVQLRKRRVKNPYPISFLLTD	300
AYM55603.1	FGVGPLCKGDNLYLSAVDVCGMFTNRSGSQQWRGLSRYFKVQLRKRRVKNPYPISFLLTD	300
CCW72483.1	FGVGPLCKGDNLYLSAVDVCGMFTNRSGSQQWRGLSRYFKVQLRKRRVKNPYPISFLLTD	300
CCW72485.1	FGVGPLCKGDNLYLSAVDVCGMFTNRSGSQQWRGLSRYFKVQLRKRRVKNPYPISFLLTD	300
AFR56669.1	FGVGPLCKGDNLYLSAVDVCGMFTNRSGSQQWRGLSRYFKVQLRKRRVKNPYPISFLLTD	300
BAH36768.1	FGVGPLCKGDNLYLSAVDVCGMFTNRSGSQQWRGLSRYFKVQLRKRRVKNPYPISFLLTD	300
BAH36786.1	FGVGPLCKGDNLYLSAVDVCGMFTNRSGSQQWRGLSRYFKVQLRKRRVKNPYPISFLLTD	300
BAH36792.1	FGVGPLCKGDNLYLSAVDVCGMFTNRSGSQQWRGLSRYFKVQLRKRRVKNPYPISFLLTD	300
BAH36798.1	FGVGPLCKGDNLYLSAVDVCGMFTNRSGSQQWRGLSRYFKVQLRKRRVKNPYPISFLLTD	300
BAH36804.1	FGVGPLCKGDNLYLSAVDVCGMFTNRSGSQQWRGLSRYFKVQLRKRRVKNPYPISFLLTD	300
BAH36810.1	FGVGPLCKGDNLYLSAVDVCGMFTNRSGSQQWRGLSRYFKVQLRKRRVKNPYPISFLLTD	300
BAH36828.1	FGVGPLCKGDNLYLSAVDVCGMFTNRSGSQQWRGLSRYFKVQLRKRRVKNPYPISFLLTD	300
BAH36852.1	FGVGPLCKGDNLYLSAVDVCGMFTNRSGSQQWRGLSRYFKVQLRKRRVKNPYPISFLLTD	300

BAH36858.1	FGVGPLCKGDNLYLSAVDVCGMFTNRSGSQQWRGLSRYFKVQLRKRRVKNPYPISFLLTD	300
BAE75814.1	FGVGPLCKGDNLYLSAVDVCGMFTNRSGSQQWRGLSRYFKVQLRKRRVKNPYPISFLLTD	300
BAE75820.1	FGVGPLCKGDNLYLSAVDVCGMFTNRSGSQQWRGLSRYFKVQLRKRRVKNPYPISFLLTD	300
BAE75826.1	FGVGPLCKGDNLYLSAVDVCGMFTNRSGSQQWRGLSRYFKVQLRKRRVKNPYPISFLLTD	300
BAE75832.1	FGVGPLCKGDNLYLSAVDVCGMFTNRSGSQQWRGLSRYFKVQLRKRRVKNPYPISFLLTD	300
BAE75838.1	FGVGPLCKGDNLYLSAVDVCGMFTNRSGSQQWRGLSRYFKVQLRKRRVKNPYPISFLLTD	300
BAE45360.1	FGVGPLCKGDNLYLSAVDVCGMFTNRSGSQQWRGLSRYFKVQLRKRRVKNPYPISFLLTD	300
BAE45372.1	FGVGPLCKGDNLYLSAVDVCGMFTNRSGSQQWRGLSRYFKVQLRKRRVKNPYPISFLLTD	300
BAE45378.1	FGVGPLCKGDNLYLSAVDVCGMFTNRSGSQQWRGLSRYFKVQLRKRRVKNPYPISFLLTD	300
BAE45384.1	FGVGPLCKGDNLYLSAVDVCGMFTNRSGSQQWRGLSRYFKVQLRKRRVKNPYPISFLLTD	300
BAE45390.1	FGVGPLCKGDNLYLSAVDVCGMFTNRSGSQQWRGLSRYFKVQLRKRRVKNPYPISFLLTD	300
BAE45396.1	FGVGPLCKGDNLYLSAVDVCGMFTNRSGSQQWRGLSRYFKVQLRKRRVKNPYPISFLLTD	300
BAE45402.1	FGVGPLCKGDNLYLSAVDVCGMFTNRSGSQQWRGLSRYFKVQLRKRRVKNPYPISFLLTD	300
BAE45408.1	FGVGPLCKGDNLYLSAVDVCGMFTNRSGSQQWRGLSRYFKVQLRKRRVKNPYPISFLLTD	300
BAE45414.1	FGVGPLCKGDNLYLSAVDVCGMFTNRSGSQQWRGLSRYFKVQLRKRRVKNPYPISFLLTD	300
BAE45420.1	FGVGPLCKGDNLYLSAVDVCGMFTNRSGSQQWRGLSRYFKVQLRKRRVKNPYPISFLLTD	300
BAE45426.1	FGVGPLCKGDNLYLSAVDVCGMFTNRSGSQQWRGLSRYFKVQLRKRRVKNPYPISFLLTD	300
BAE45432.1	FGVGPLCKGDNLYLSAVDVCGMFTNRSGSQQWRGLSRYFKVQLRKRRVKNPYPISFLLTD	300
BAE45438.1	FGVGPLCKGDNLYLSAVDVCGMFTNRSGSQQWRGLSRYFKVQLRKRRVKNPYPISFLLTD	300
BAD06018.1	FGVGPLCKGDNLYLSAVDVCGMFTNRSGSQQWRGLSRYFKVQLRKRRVKNPYPISFLLTD	300
BAD06024.1	FGVGPLCKGDNLYLSAVDVCGMFTNRSGSQQWRGLSRYFKVQLRKRRVKNPYPISFLLTD	300
BAD06030.1	FGVGPLCKGDNLYLSAVDVCGMFTNRSGSQQWRGLSRYFKVQLRKRRVKNPYPISFLLTD	300
BAD06042.1	FGVGPLCKGDNLYLSAVDVCGMFTNRSGSQQWRGLSRYFKVQLRKRRVKNPYPISFLLTD	300
BAD06048.1	FGVGPLCKGDNLYLSAVDVCGMFTNRSGSQQWRGLSRYFKVQLRKRRVKNPYPISFLLTD	300
BAD06054.1	FGVGPLCKGDNLYLSAVDVCGMFTNRSGSQQWRGLSRYFKVQLRKRRVKNPYPISFLLTD	300
BAD06084.1	FGVGPLCKGDNLYLSAVDVCGMFTNRSGSQQWRGLSRYFKVQLRKRRVKNPYPISFLLTD	300
BAD06090.1	FGVGPLCKGDNLYLSAVDVCGMFTNRSGSQQWRGLSRYFKVQLRKRRVKNPYPISFLLTD	300
BAD06096.1	FGVGPLCKGDNLYLSAVDVCGMFTNRSGSQQWRGLSRYFKVQLRKRRVKNPYPISFLLTD	300
BAD06102.1	FGVGPLCKGDNLYLSAVDVCGMFTNRSGSQQWRGLSRYFKVQLRKRRVKNPYPISFLLTD	300
BAD06108.1	FGVGPLCKGDNLYLSAVDVCGMFTNRSGSQQWRGLSRYFKVQLRKRRVKNPYPISFLLTD	300
BAD06120.1	FGVGPLCKGDNLYLSAVDVCGMFTNRSGSQQWRGLSRYFKVQLRKRRVKNPYPISFLLTD	300
BAC15695.1	FGVGPLCKGDNLYLSAVDVCGMFTNRSGSQQWRGLSRYFKVQLRKRRVKNPYPISFLLTD	300
BAC15725.1	FGVGPLCKGDNLYLSAVDVCGMFTNRSGSQQWRGLSRYFKVQLRKRRVKNPYPISFLLTD	300
BAC02951.1	FGVGPLCKGDNLYLSAVDVCGMFTNRSGSQQWRGLSRYFKVQLRKRRVKNPYPISFLLTD	300
BAC02957.1	FGVGPLCKGDNLYLSAVDVCGMFTNRSGSQQWRGLSRYFKVQLRKRRVKNPYPISFLLTD	300
BAC02963.1	FGVGPLCKGDNLYLSAVDVCGMFTNRSGSQQWRGLSRYFKVQLRKRRVKNPYPISFLLTD	300
BAC02969.1	FGVGPLCKGDNLYLSAVDVCGMFTNRSGSQQWRGLSRYFKVQLRKRRVKNPYPISFLLTD	300
BAC02975.1	FGVGPLCKGDNLYLSAVDVCGMFTNRSGSQQWRGLSRYFKVQLRKRRVKNPYPISFLLTD	300
BAC02981.1	FGVGPLCKGDNLYLSAVDVCGMFTNRSGSQQWRGLSRYFKVQLRKRRVKNPYPISFLLTD	300
BAC02987.1	FGVGPLCKGDNLYLSAVDVCGMFTNRSGSQQWRGLSRYFKVQLRKRRVKNPYPISFLLTD	300
BAC02993.1	FGVGPLCKGDNLYLSAVDVCGMFTNRSGSQQWRGLSRYFKVQLRKRRVKNPYPISFLLTD	300
BAC02999.1	FGVGPLCKGDNLYLSAVDVCGMFTNRSGSQQWRGLSRYFKVQLRKRRVKNPYPISFLLTD	300
BAC03005.1	FGVGPLCKGDNLYLSAVDVCGMFTNRSGSQQWRGLSRYFKVQLRKRRVKNPYPISFLLTD	300
BAC03011.1	FGVGPLCKGDNLYLSAVDVCGMFTNRSGSQQWRGLSRYFKVQLRKRRVKNPYPISFLLTD	300
BAC03017.1	FGVGPLCKGDNLYLSAVDVCGMFTNRSGSQQWRGLSRYFKVQLRKRRVKNPYPISFLLTD	300
BAC03023.1	FGVGPLCKGDNLYLSAVDVCGMFTNRSGSQQWRGLSRYFKVQLRKRRVKNPYPISFLLTD	300
BAC03035.1	FGVGPLCKGDNLYLSAVDVCGMFTNRSGSQQWRGLSRYFKVQLRKRRVKNPYPISFLLTD	300
BAC03041.1	FGVGPLCKGDNLYLSAVDVCGMFTNRSGSQQWRGLSRYFKVQLRKRRVKNPYPISFLLTD	300
BAC03047.1	FGVGPLCKGDNLYLSAVDVCGMFTNRSGSQQWRGLSRYFKVQLRKRRVKNPYPISFLLTD	300
BAC03053.1	FGVGPLCKGDNLYLSAVDVCGMFTNRSGSQQWRGLSRYFKVQLRKRRVKNPYPISFLLTD	300
BAC03059.1	FGVGPLCKGDNLYLSAVDVCGMFTNRSGSQQWRGLSRYFKVQLRKRRVKNPYPISFLLTD	300
BAC03071.1	FGVGPLCKGDNLYLSAVDVCGMFTNRSGSQQWRGLSRYFKVQLRKRRVKNPYPISFLLTD	300
BAC03077.1	FGVGPLCKGDNLYLSAVDVCGMFTNRSGSQQWRGLSRYFKVQLRKRRVKNPYPISFLLTD	300
BAC03083.1	FGVGPLCKGDNLYLSAVDVCGMFTNRSGSQQWRGLSRYFKVQLRKRRVKNPYPISFLLTD	300
BAC03089.1	FGVGPLCKGDNLYLSAVDVCGMFTNRSGSQQWRGLSRYFKVQLRKRRVKNPYPISFLLTD	300
AAM69542.1	FGVGPLCKGDNLYLSAVDVCGMFTNRSGSQQWRGLSRYFKVQLRKRRVKNPYPISFLLTD	300
AAM69548.1	FGVGPLCKGDNLYLSAVDVCGMFTNRSGSQQWRGLSRYFKVQLRKRRVKNPYPISFLLTD	300
AAM69554.1	FGVGPLCKGDNLYLSAVDVCGMFTNRSGSQQWRGLSRYFKVQLRKRRVKNPYPISFLLTD	300
AAM69619.1	FGVGPLCKGDNLYLSAVDVCGMFTNRSGSQQWRGLSRYFKVQLRKRRVKNPYPISFLLTD	300
BAB68977.1	FGVGPLCKGDNLYLSAVDVCGMFTNRSGSQQWRGLSRYFKVQLRKRRVKNPYPISFLLTD	300
BAB68983.1	FGVGPLCKGDNLYLSAVDVCGMFTNRSGSQQWRGLSRYFKVQLRKRRVKNPYPISFLLTD	300
BAB68989.1	FGVGPLCKGDNLYLSAVDVCGMFTNRSGSQQWRGLSRYFKVQLRKRRVKNPYPISFLLTD	300
BAB68995.1	FGVGPLCKGDNLYLSAVDVCGMFTNRSGSQQWRGLSRYFKVQLRKRRVKNPYPISFLLTD	300
AAK98000.1	FGVGPLCKGDNLYLSAVDVCGMFTNRSGSQQWRGLSRYFKVQLRKRRVKNPYPISFLLTD	300

AAK98006.1	FGVGPLCKGDNLYLSAVDVCGMFTNRSGSQQWRGLSRYFKVQLRKRRVKNPYPISFLLTD	300
AAK98010.1	FGVGPLCKGDNLYLSAVDVCGMFTNRSGSQQWRGLSRYFKVQLRKRRVKNPYPISFLLTD	300
AAK98018.1	FGVGPLCKGDNLYLSAVDVCGMFTNRSGSQQWRGLSRYFKVQLRKRRVKNPYPISFLLTD	300
AAK98024.1	FGVGPLCKGDNLYLSAVDVCGMFTNRSGSQQWRGLSRYFKVQLRKRRVKNPYPISFLLTD	300
AAK98030.1	FGVGPLCKGDNLYLSAVDVCGMFTNRSGSQQWRGLSRYFKVQLRKRRVKNPYPISFLLTD	300
AAK98036.1	FGVGPLCKGDNLYLSAVDVCGMFTNRSGSQQWRGLSRYFKVQLRKRRVKNPYPISFLLTD	300
AAK70258.1	FGVGPLCKGDNLYLSAVDVCGMFTNRSGSQQWRGLSRYFKVQLRKRRVKNPYPISFLLTD	300
AAK70264.1	FGVGPLCKGDNLYLSAVDVCGMFTNRSGSQQWRGLSRYFKVQLRKRRVKNPYPISFLLTD	300
AAG53896.1	FGVGPLCKGDNLYLSAVDVCGMFTNRSGSQQWRGLSRYFKVQLRKRRVKNPYPISFLLTD	300
BAA01962.1	FGVGPLCKGDNLYLSAVDVCGMFTNRSGSQQWRGLSRYFKVQLRKRRVKNPYPISFLLTD	300
ALF06256.1	FGVGPLCKGDNLYLSAVDVCGMFTNRSGSQQWRGLSRYFKVQLRKRRVKNPYPISFLLTD	300
BAD06060.1	FGVGPLCKGDNLYLSAVDVCGMFTNRSGSQQWRGLSRYFKVQLRKRRVKNPYPISFLLTD	300
BAD06072.1	FGVGPLCKGDNLYLSAVDVCGMFTNRSGSQQWRGLSRYFKVQLRKRRVKNPYPISFLLTD	300
BAD06156.1	FGVGPLCKGDNLYLSAVDVCGMFTNRSGSQQWRGLSRYFKVQLRKRRVKNPYPISFLLTD	300
BAD06174.1	FGVGPLCKGDNLYLSAVDVCGMFTNRSGSQQWRGLSRYFKVQLRKRRVKNPYPISFLLTD	300
AAN85449.1	FGVGPLCKGDNLYLSAVDVCGMFTNRSGSQQWRGLSRYFKVQLRKRRVKNPYPISFLLTD	300
AAN85455.1	FGVGPLCKGDNLYLSAVDVCGMFTNRSGSQQWRGLSRYFKVQLRKRRVKNPYPISFLLTD	300
AAK98042.1	FGVGPLCKGDNLYLSAVDVCGMFTNRSGSQQWRGLSRYFKVQLRKRRVKNPYPISFLLTD	300
BAD06036.1	FGVGPLCKGDNLYLSAVDVCGMFTNRSGSQQWRGLSRYFKVQLRKRRVKNPYPISFLLTD	300
BCP65063.1	FGVGPLCKGDNLYLSAVDVCGMFTDRSGSQQWRGLSRYFKVQLRKRRVKNPYPISFLLTD	300
BCP56477.1	FGVGPLCKGDNLYLSAVDVCGMFTDRSGSQQWRGLSRYFKVQLRKRRVKNPYPISFLLTD	300
BAV69373.1	FGVGPLCKGDNLYLSAVDVCGMFTDRSGSQQWRGLSRYFKVQLRKRRVKNPYPISFLLTD	300
BAE00153.1	FGVGPLCKGDNLYLSAVDVCGMFTNRSGYQQWRGLSRYFKVQLRKRRVKNPYPISFLLTD	300
BAC22906.1	FGVGPLCKGDNLYLSAVDVCGMFTNRSGTQQWRGLSRYFKVQLRKRRVKNPYPISFLLTD	300
BAA05639.1	FGVGPLCKGDNLYLSAVDVCGMFTNRSGTQQWRGLSRYFKVQLRKRRVKNPYPISFLLTD	300
BAB11728.1	FGVGPLCKGDNLYLSAVDVCGMFTNRSGFQQWRGLSRYFKVQLRKRRVKNPYPISFLLTD	300
BAA05637.1	FGVGPLCKGDNLYLSAVDVCGMFTNRSGFQQWRGLSRYFKVQLRKRRVKNPYPISFLLTD	300
BAA01969.1	FGVGPLCKGDNLYLSAVDVCGMFTNRSGFQQWRGLSRYFKVQLRKRRVKNPYPISFLLTD	300
BAE00159.1	FGVGPLCKGDNLYLSAVDVCGMFTNRSGSQQWRGLSRYFKVQLRKRRVKNPYPISFLLTD	300
BAE00165.1	FGVGPLCKGDNLYLSAVDVCGMFTNRSGSQQWRGLSRYFKVQLRKRRVKNPYPISFLLTD	300
BAE00171.1	FGVGPLCKGDNLYLSAVDVCGMFTNRSGSQQWRGLSRYFKVQLRKRRVKNPYPISFLLTD	300
ALF06268.1	FGVGPLCKGDNLYLSAVDVCGMFTNRSGSQQWRGLSRYFKVQLRKRRVKNPYPISFLLTD	300
AYM55586.1	FGVGPLCKGDNLYLSAVDVCGMFTNRSGSQQWRGLSRYFKVQLRKRRVKNPYPISFLLTD	300
AYM55585.1	FGVGPLCKGDNLYLSAVDVCGMFTNRSGSQQWRGLSRYFKVQLRKRRVKNPYPISFLLTD	300
AYM55587.1	FGVGPLCKGDNLYLSAVDVCGMFTNRSGSQQWRGLSRYFKVQLRKRRVKNPYPISFLLTD	300
AYM55594.1	FGVGPLCKGDNLYLSAVDVCGMFTNRSGSQQWRGLSRYFKVQLRKRRVKNPYPISFLLTD	300
QBQ65470.1	FGVGPLCKGDNLYLSAVDVCGMFTNRSGSQQWRGLSRYFKVQLRKRRVKNPYPISFLLTD	300
CCW72501.1	FGVGPLCKGDNLYLSAVDVCGMFTNRSGSQQWRGLSRYFKVQLRKRRVKNPYPISFLLTD	300
CCW72524.1	FGVGPLCKGDNLYLSAVDVCGMFTNRSGSQQWRGLSRYFKVQLRKRRVKNPYPISFLLTD	300
QWT77116.1	FGVGPLCKGDNLYLSAVDVCGMFTNRSGSQQWRGLSRYFKVQLRKRRVKNPYPISFLLTD	300
WLD48046.1	FGVGPLCKGDNLYLSAVDVCGMYTNRSGSQQWRGLSRYFKVQLRKRRVKNPYPISFLLTD	300
WLD48052.1	FGVGPLCKGDNLYLSAVDVCGMYTNRSGSQQWRGLSRYFKVQLRKRRVKNPYPISFLLTD	300
AAK70288.1	FGVGPLCKGDNLYLSAVDVCGMFTNRSGSQQWRGLSRYFKVQLRKRRVKNPYPISFLLTD	300
AAK70270.1	FGVGPLCKGDNLYLSAVDVCGMFTNRSGSQQWRGLSRYFKVQLRKRRVKNPYPISFLLTD	300
BAD21277.2	FGVGPLCKGDNLYLSAVDVCGMFTNRSGSQQWRGLSRYFKVQLRKRRVKNPYPISFLLTD	300
CCW72498.1	FGVGPLCKGDNLYLSAVDVCGMFTNRSGSQQWRGLSRYFKVQLRKRRVKNPYPISFLLTD	300
UPN63085.1	FGVGPLCKGDNLYLSAVDVCGMFTNRSGYQQWRGLSRYFKVQLRKRRVKNPYPISFLLTD	300
UPN63090.1	FGVGPLCKGDNLYLSAVDVCGMFTNRSGYQQWRGLSRYFKVQLRKRRVKNPYPISFLLTD	300
QWT77134.1	FGVGPLCKGDNLYLSAVDVCGMFTHRSGSQQWRGLSRYFKVQLRKRRVKNPYPISFLLTD	300
WLD48070.1	FGVGPLCKGDNLYLSAVDVCGMFTNRSGSQQWRGLSRYFKVQLRKRRVKNPYPISFLLTD	300
AAK70282.1	FGVGPLCKGDNLYLSAVDVCGMFTNRSGSQQWRGLSRYFKVQLRKRRVKNPYPISFLLTD	300
WLD48058.1	FGVGPLCKGDNLYLSAVDVCGMFTNRSGSQQWRGLSRYFKVQLRKRRVKNPYPISFLLTD	300
WLD48076.1	FGVGPLCKGDNLYLSAVDVCGMFTNRSGSQQWRGLSRYFKVQLRKRRVKNPYPISFLLTD	300
UQK62667.1	FGVGPLCKGDNLYLSAVDVCGMFTNRSGSQQWRGLSRYFKVQLRKRRVKNPYPISFLLTD	300
CCW72480.1	FGVGPLCKGDNLYLSAVDVCGMFTNRSGSQQWRGLSRYFKVQLRKRRVKNPYPISFLLTD	300
CCW72513.1	FGVGPLCKGDNLYLSAVDVCGMFTNRSGSQQWRGLSRYFKVQLRKRRVKNPYPISFLLTD	300
AAM69614.1	FGVGPLCKGDNLYLSAVDVCGMFTNRSGSQQWRGLSRYFKVQLRKRRVKNPYPISFLLTD	300
AAK70318.1	FGVGPLCKGDNLYLSAADVCGMFTNRSGSQQWRGLSRYFRVQLRKRRVKNPYPISFLLTD	300
AAK70324.1	FGVGPLCKGDNLYLSAVDVCGMFTNRSGSQQWRGLSRYFKVQLRKRRVKNPYPISFLLTD	300
AAT09837.1	FGVGPLCKGDNLYLSAVDVCGMFTNRSGSQQWRGLSRYFKVQLRKRRVKNPYPISFLLTD	300
AAT09831.1	FGVGPLCKGDNLYLSAVDVCGMFTNRSGSQQWRGLSRYFKVQLRKRRVKNPYPISFLLTD	300
AAT09825.1	FGVGPLCKGDNLYLSAVDVCGMFTNRFGSQQWRGLSRYFKVQLRKRRVKNPYPISFLLTD	300
AAR89271.1	FGVGPLCKGDNLYLSAVDVCGMFTNRSGSQQWRGLSRYFKVQLRKRRVKNPYPISFLLTD	300
AAR89211.1	FGVGPLCKGDNLYLSAVDVCGMFTNRSGSQQWRGLSKYFKVQLRKRRVKNPYPISFLLTD	300

BAE02956.1	FGVGPLCKGDNLYLSAVDVCGMFTNRSGSQQWRGLSRYFKVQLRKRRVKNPYPISFLLTD	300
ACF75876.1	FGVGPLCKGDNLYLSAVDVCGMFTNRSGYQQWRGLSRYFKVQLRKRRVKNPYPISFLLTD	300
QBM11924.1	FGVGPLCKGDNLYLSAVDVCGMFTNRSGSQQWRGLSRYFKVQLRKRRVKNPYPISFLLTD	300
QBM11858.1	FGVGPLCKGDNLYLSAVDVCGMFTNRSGSQQWRGLSRYFKVQLRKRRVKNPYPISFLLTD	300
QBQ04203.1	FGVGPLCKGDNLYLSAVDVCGMFTNRSGSQQWRGLSRYFKVQLRKRRVKNPYPISFLLTD	300
QBQ65473.1	FGVGPLCKGDNLYLSAVDVCGMFTNRSGSQQWRGLSRYFKVQLRKRRVKNPYPISFLLTD	300
QBQ65472.1	FGVGPLCKGDNLYLSAVDVCGMFTNRSGSQQWRGLSRYFKVQLRKRRVKNPYPISFLLTD	300
QBM11920.1	FGVGPLCKGDNLYLSAVDVCGMFTNRSGSQQWRGLSRYFKVQLRKRRVKNPYPISFLLTD	300
QAA95693.1	FGVGPLCKGDNLYLSAVDVCGMFTNRSGSQQWRGLSRYFKVQLRKRRVKNPYPISFLLTD	300
QBX89122.1	FGVGPLCKGDNLYLSAVDVCGMFTNRSGSQQWRGLSRYFKVQLRKRRVKNPYPISFLLTD	300
QID57946.1	FGVGPLCKGDNLYLSAVDVCGMFTNRSGSQQWRGLSRYFKVQLRKRRVKNPYPISFLLTD	300
QID57952.1	FGVGPLCKGDNLYLSAVDVCGMFTNRSGSQQWRGLSRYFKVQLRKRRVKNPYPISFLLTD	300
QID57958.1	FGVGPLCKGDNLYLSAVDVCGMFTNRSGSQQWRGLSRYFKVQLRKRRVKNPYPISFLLTD	300
QID57964.1	FGVGPLCKGDNLYLSAVDVCGMFTNRSGSQQWRGLSRYFKVQLRKRRVKNPYPISFLLTD	300
QID57970.1	FGVGPLCKGDNLYLSAVDVCGMFTNRSGSQQWRGLSRYFKVQLRKRRVKNPYPISFLLTD	300
QID57976.1	FGVGPLCKGDNLYLSAVDVCGMFTNRSGSQQWRGLSRYFKVQLRKRRVKNPYPISFLLTD	300
QCH41110.1	FGVGPLCKGDNLYLSAVDVCGMFTNRSGSQQWRGLSRYFKVQLRKRRVKNPYPISFLLTD	300
QBX89120.1	FGVGPLCKGDNLYLSAVDVCGMFTNRSGSQQWRGLSRYFKVQLRKRRVKNPYPISFLLTD	300
QBX89121.1	FGVGPLCKGDNLYLSAVDVCGMFTNRSGSQQWRGLSRYFKVQLRKRRVKNPYPISFLLTD	300
QBX89124.1	FGVGPLCKGDNLYLSAVDVCGMFTNRSGSQQWRGLSRYFKVQLRKRRVKNPYPISFLLTD	300
QBQ65471.1	FGVGPLCKGDNLYLSAVDVCGMFTNRSGSQQWRGLSRYFKVQLRKRRVKNPYPISFLLTD	300
QBQ65474.1	FGVGPLCKGDNLYLSAVDVCGMFTNRSGSQQWRGLSRYFKVQLRKRRVKNPYPISFLLTD	300
QBQ65476.1	FGVGPLCKGDNLYLSAVDVCGMFTNRSGSQQWRGLSRYFKVQLRKRRVKNPYPISFLLTD	300
QBQ04201.1	FGVGPLCKGDNLYLSAVDVCGMFTNRSGSQQWRGLSRYFKVQLRKRRVKNPYPISFLLTD	300
QBM11921.1	FGVGPLCKGDNLYLSAVDVCGMFTNRSGSQQWRGLSRYFKVQLRKRRVKNPYPISFLLTD	300
QBM11922.1	FGVGPLCKGDNLYLSAVDVCGMFTNRSGSQQWRGLSRYFKVQLRKRRVKNPYPISFLLTD	300
CDJ79845.1	FGVGPLCKGDNLYLSAVDVCGMFTNRSGSQQWRGLSRYFKVQLRKRRVKNPYPISFLLTD	300
BAE02932.1	FGVGPLCKGDNLYLSAVDVCGMFTNRSGSQQWRGLSRYFKVQLRKRRVKNPYPISFLLTD	300
BAE02938.1	FGVGPLCKGDNLYLSAVDVCGMFTNRSGSQQWRGLSRYFKVQLRKRRVKNPYPISFLLTD	300
BAE02950.1	FGVGPLCKGDNLYLSAVDVCGMFTNRSGSQQWRGLSRYFKVQLRKRRVKNPYPISFLLTD	300
BAE03004.1	FGVGPLCKGDNLYLSAVDVCGMFTNRSGSQQWRGLSRYFKVQLRKRRVKNPYPISFLLTD	300
BAE03118.1	FGVGPLCKGDNLYLSAVDVCGMFTNRSGSQQWRGLSRYFKVQLRKRRVKNPYPISFLLTD	300
BAE03124.1	FGVGPLCKGDNLYLSAVDVCGMFTNRSGSQQWRGLSRYFKVQLRKRRVKNPYPISFLLTD	300
BAE03130.1	FGVGPLCKGDNLYLSAVDVCGMFTNRSGSQQWRGLSRYFKVQLRKRRVKNPYPISFLLTD	300
BAE03136.1	FGVGPLCKGDNLYLSAVDVCGMFTNRSGSQQWRGLSRYFKVQLRKRRVKNPYPISFLLTD	300
BAE03142.1	FGVGPLCKGDNLYLSAVDVCGMFTNRSGSQQWRGLSRYFKVQLRKRRVKNPYPISFLLTD	300
BAE03148.1	FGVGPLCKGDNLYLSAVDVCGMFTNRSGSQQWRGLSRYFKVQLRKRRVKNPYPISFLLTD	300
BAE03154.1	FGVGPLCKGDNLYLSAVDVCGMFTNRSGSQQWRGLSRYFKVQLRKRRVKNPYPISFLLTD	300
QBQ04200.1	FGVGPLCKGDNLYLSAVDVCGMFTNRSGSQQWRGLSRYFKVQLRKRRVKNPYPISFLLTD	300
UYL83839.1	FGVGPLCKGDNLYLSAVDVCGMFTNRSGSQQWRGLSRYFKVQLRKRRVKNPYPISFLLTD	300
ALF06262.1	FGVGPLCKGDNLYLSAVDVCGMFTNRSGSQQWRGLSRYFKVQLRKRRVKNPYPISFLLTD	300
ABH10586.1	FGVGPLCKGDNLYLSAVDVCGMFTNRSGSQQWRGLSRYFKVQLRKRRVKNPYPISFLLTD	300
BAE02890.1	FGVGPLCKGDNLYLSAVDVCGMFTNRSGSQQWRGLSRYFKVQLRKRRVKNPYPISFLLTD	300
BAE02896.1	FGVGPLCKGDNLYLSAVDVCGMFTNRSGSQQWRGLSRYFKVQLRKRRVKNPYPISFLLTD	300
BAE02902.1	FGVGPLCKGDNLYLSAVDVCGMFTNRSGSQQWRGLSRYFKVQLRKRRVKNPYPISFLLTD	300
BAE02914.1	FGVGPLCKGDNLYLSAVDVCGMFTNRSGSQQWRGLSRYFKVQLRKRRVKNPYPISFLLTD	300
BAE02920.1	FGVGPLCKGDNLYLSAVDVCGMFTNRSGSQQWRGLSRYFKVQLRKRRVKNPYPISFLLTD	300
BAE02944.1	FGVGPLCKGDNLYLSAVDVCGMFTNRSGSQQWRGLSRYFKVQLRKRRVKNPYPISFLLTD	300
BAE02962.1	FGVGPLCKGDNLYLSAVDVCGMFTNRSGSQQWRGLSRYFKVQLRKRRVKNPYPISFLLTD	300
BAE02968.1	FGVGPLCKGDNLYLSAVDVCGMFTNRSGSQQWRGLSRYFKVQLRKRRVKNPYPISFLLTD	300
BAE02974.1	FGVGPLCKGDNLYLSAVDVCGMFTNRSGSQQWRGLSRYFKVQLRKRRVKNPYPISFLLTD	300
BAE02980.1	FGVGPLCKGDNLYLSAVDVCGMFTNRSGSQQWRGLSRYFKVQLRKRRVKNPYPISFLLTD	300
BAE02986.1	FGVGPLCKGDNLYLSAVDVCGMFTNRSGSQQWRGLSRYFKVQLRKRRVKNPYPISFLLTD	300
BAE02992.1	FGVGPLCKGDNLYLSAVDVCGMFTNRSGSQQWRGLSRYFKVQLRKRRVKNPYPISFLLTD	300
BAE02998.1	FGVGPLCKGDNLYLSAVDVCGMFTNRSGSQQWRGLSRYFKVQLRKRRVKNPYPISFLLTD	300
BAE03010.1	FGVGPLCKGDNLYLSAVDVCGMFTNRSGSQQWRGLSRYFKVQLRKRRVKNPYPISFLLTD	300
BAE03016.1	FGVGPLCKGDNLYLSAVDVCGMFTNRSGSQQWRGLSRYFKVQLRKRRVKNPYPISFLLTD	300
BAE03022.1	FGVGPLCKGDNLYLSAVDVCGMFTNRSGSQQWRGLSRYFKVQLRKRRVKNPYPISFLLTD	300
BAE03028.1	FGVGPLCKGDNLYLSAVDVCGMFTNRSGSQQWRGLSRYFKVQLRKRRVKNPYPISFLLTD	300
BAE03034.1	FGVGPLCKGDNLYLSAVDVCGMFTNRSGSQQWRGLSRYFKVQLRKRRVKNPYPISFLLTD	300
BAE03040.1	FGVGPLCKGDNLYLSAVDVCGMFTNRSGSQQWRGLSRYFKVQLRKRRVKNPYPISFLLTD	300
BAE03046.1	FGVGPLCKGDNLYLSAVDVCGMFTNRSGSQQWRGLSRYFKVQLRKRRVKNPYPISFLLTD	300
BAE03052.1	FGVGPLCKGDNLYLSAVDVCGMFTNRSGSQQWRGLSRYFKVQLRKRRVKNPYPISFLLTD	300
BAE03058.1	FGVGPLCKGDNLYLSAVDVCGMFTNRSGSQQWRGLSRYFKVQLRKRRVKNPYPISFLLTD	300

```
BAE03064.1        FGVGPLCKGDNLYLSAVDVCGMFTNRSGSQQWRGLSRYFKVQLRKRRVKNPYPISFLLTD  300
BAE03070.1        FGVGPLCKGDNLYLSAVDVCGMFTNRSGSQQWRGLSRYFKVQLRKRRVKNPYPISFLLTD  300
BAE03076.1        FGVGPLCKGDNLYLSAVDVCGMFTNRSGSQQWRGLSRYFKVQLRKRRVKNPYPISFLLTD  300
BAE03082.1        FGVGPLCKGDNLYLSAVDVCGMFTNRSGSQQWRGLSRYFKVQLRKRRVKNPYPISFLLTD  300
BAE03088.1        FGVGPLCKGDNLYLSAVDVCGMFTNRSGSQQWRGLSRYFKVQLRKRRVKNPYPISFLLTD  300
BAE03094.1        FGVGPLCKGDNLYLSAVDVCGMFTNRSGSQQWRGLSRYFKVQLRKRRVKNPYPISFLLTD  300
BAE03100.1        FGVGPLCKGDNLYLSAVDVCGMFTNRSGSQQWRGLSRYFKVQLRKRRVKNPYPISFLLTD  300
BAE03106.1        FGVGPLCKGDNLYLSAVDVCGMFTNRSGSQQWRGLSRYFKVQLRKRRVKNPYPISFLLTD  300
BAE03112.1        FGVGPLCKGDNLYLSAVDVCGMFTNRSGSQQWRGLSRYFKVQLRKRRVKNPYPISFLLTD  300
BAE03160.1        FGVGPLCKGDNLYLSAVDVCGMFTNRSGSQQWRGLSRYFKVQLRKRRVKNPYPISFLLTD  300
BAE03166.1        FGVGPLCKGDNLYLSAVDVCGMFTNRSGSQQWRGLSRYFKVQLRKRRVKNPYPISFLLTD  300
AAQ88264.1        FGVGPLCKGDNLYLSAVDVCGMFTNRSGSQQWRGLSRYFKVQLRKRRVKNPYPISFLLTD  300
AAQ88270.1        FGVGPLCKGDNLYLSAVDVCGMFTNRSGSQQWRGLSRYFKVQLRKRRVKNPYPISFLLTD  300
AAR89187.1        FGVGPLCKGDNLYLSAVDVCGMFTNRSGSQQWRGLSRYFKVQLRKRRVKNPYPISFLLTD  300
AAR89193.1        FGVGPLCKGDNLYLSAVDVCGMFTNRSGSQQWRGLSRYFKVQLRKRRVKNPYPISFLLTD  300
AAR89199.1        FGVGPLCKGDNLYLSAVDVCGMFTNRSGSQQWRGLSRYFKVQLRKRRVKNPYPISFLLTD  300
AAR89205.1        FGVGPLCKGDNLYLSAVDVCGMFTNRSGSQQWRGLSRYFKVQLRKRRVKNPYPISFLLTD  300
AAR89217.1        FGVGPLCKGDNLYLSAVDVCGMFTNRSGSQQWRGLSRYFKVQLRKRRVKNPYPISFLLTD  300
AAR89223.1        FGVGPLCKGDNLYLSAVDVCGMFTNRSGSQQWRGLSRYFKVQLRKRRVKNPYPISFLLTD  300
AAR89229.1        FGVGPLCKGDNLYLSAVDVCGMFTNRSGSQQWRGLSRYFKVQLRKRRVKNPYPISFLLTD  300
AAR89235.1        FGVGPLCKGDNLYLSAVDVCGMFTNRSGSQQWRGLSRYFKVQLRKRRVKNPYPISFLLTD  300
AAR89241.1        FGVGPLCKGDNLYLSAVDVCGMFTNRSGSQQWRGLSRYFKVQLRKRRVKNPYPISFLLTD  300
AAR89247.1        FGVGPLCKGDNLYLSAVDVCGMFTNRSGSQQWRGLSRYFKVQLRKRRVKNPYPISFLLTD  300
AAR89253.1        FGVGPLCKGDNLYLSAVDVCGMFTNRSGSQQWRGLSRYFKVQLRKRRVKNPYPISFLLTD  300
AAR32743.1        FGVGPLCKGDNLYLSAVDVCGMFTNRSGSQQWRGLSRYFKVQLRKRRVKNPYPISFLLTD  300
AAR89265.1        FGVGPLCKGDNLYLSAVDVCGMFTNRSGSQQWRGLSRYFKVQLRKRRVKNPYPISFLLTD  300
AAR89277.1        FGVGPLCKGDNLYLSAVDVCGMFTNRSGSQQWRGLSRYFKVQLRKRRVKNPYPISFLLTD  300
AAR89283.1        FGVGPLCKGDNLYLSAVDVCGMFTNRSGSQQWRGLSRYFKVQLRKRRVKNPYPISFLLTD  300
AAR02463.1        FGVGPLCKGDNLYLSAVDVCGMFTNRSGSQQWRGLSRYFKVQLRKRRVKNPYPISFLLTD  300
AAR06661.1        FGVGPLCKGDNLYLSAVDVCGMFTNRSGSQQWRGLSRYFKVQLRKRRVKNPYPISFLLTD  300
AAR13659.1        FGVGPLCKGDNLYLSAVDVCGMFTNRSGSQQWRGLSRYFKVQLRKRRVKNPYPISFLLTD  300
AAR02457.1        FGVGPLCKGDNLYLSAVDVCGMFTNRSGSQQWRGLSRYFKVQLRKRRVKNPYPISFLLTD  300
BAB68785.1        FGVGPLCKGDNLYLSAVDVCGMFTNRSGSQQWRGLSRYFKVQLRKRRVKNPYPISFLLTD  300
AAK28472.1        FGVGPLCKGDNLYLSAVDVCGMFTNRSGSQQWRGLSRYFKVQLRKRRVKNPYPISFLLTD  300
AAG34667.1        FGVGPLCKGDNLYLSAVDVCGMFTNRSGSQQWRGLSRYFKVQLRKRRVKNPYPISFLLTD  300
AAG34673.1        FGVGPLCKGDNLYLSAVDVCGMFTNRSGSQQWRGLSRYFKVQLRKRRVKNPYPISFLLTD  300
AAB41705.1        FGVGPLCKGDNLYLSAVDVCGMFTNRSGSQQWRGLSRYFKVQLRKRRVKNPYPISFLLTD  300
AAB41711.1        FGVGPLCKGDNLYLSAVDVCGMFTNRSGSQQWRGLSRYFKVQLRKRRVKNPYPISFLLTD  300
AAB41717.1        FGVGPLCKGDNLYLSAVDVCGMFTNRSGSQQWRGLSRYFKVQLRKRRVKNPYPISFLLTD  300
BAE02926.1        FGVGPLCKGDNLYLSAVDVCGMFTNRSGSQQWRGLSRYFKVQLRKRRVKNPYPISFLLTD  300
                  :*.*** :***:****. * . :* :   : *****:**:****: : ********:*:*

AEC14619.1        LINRRTPRVDGQPMYGMDAQVEEVRVFEGTEELPGDPDMMRYVDRYGQLQTKML  354
AEC15199.1        LINRRTPRVDGQPMYGMDAQVEEVRVFEGTEELPGDPDMMRYVDKYGQLQTKML  354
AEC15209.1        LINRRTPRVDGQPMYGMDAQVEEVRVFEGTEELPGDPDMMRYVDKYGQLQTKML  354
UPN63098.1        LINRRTPRVDGQPMYGMDAQVEEVRVFEGTEELPGDPDMMRYVDKYGQLQTKML  354
UPN63097.1        LINRRTPRVDGQPMYGMDAQVEEVRVFEGTEELPGDPDMMRYVDKYGQLQTKML  354
UPN63094.1        LINRRTPRVDGQPMYGMDAQVEEVRVFEGTEELPGDPDMMRYVDKYGQLQTKML  354
UPN63096.1        LINRRTPRVDGQPMYGMDAQVEEVRVFEGTEELPGDPDMMRYVDKYGQLQTKML  354
QWT77146.1        LINRRTPRVDGQPMYGMDAQVEEVRVFEGTEELPGDPDMMRYVDKYGQLQTKML  354
UPN63095.1        LINRRTPRVDGQPMYGMDAQVEEVRVFEGTEELPGDPDMMRYVDKYGQLQTKML  354
BAA01966.1        LINRRTPRVDGQPMYGMDAQVEEVRVFEGTEELPGDPDMMRYVDKYGQLQTKML  354
AEC15099.1        LINRRTPRVDGQPMYGMDAQVEEVRVFEGTEELPGDPDMMRYVDKYGQLQTKML  354
UPN63099.1        LINRRTPRVDGQPMYGMDAQVEEVRVFEGTEELPGDPDMMRYVDKYGQLQTKML  354
UPN63086.1        LINRRTPRVDGQPMYGMDAQVEEVRVFEGTEELPGDPDMMRYVDKYGQLQTKML  354
UPN63092.1        LINRRTPRVDGQPMYGMDAQVEEVRVFEGTEELPGDPDMMRYVDKYGQLQTKML  354
AEC15783.1        LINRRTPRVDGQPMYGMDAQVEEVRVFEGTEELPGDPDMMRYVDKYGQLQTKML  354
AEC15735.1        LINRRTPRVDGQPMYGMDAQVEEVRVFEGTEELPGDPDMMRYVDKYGQLQTKML  354
AEC15615.1        LINRRTPRVDGQPMYGMDAQVEEVRVFEGTEELPGDPDMMRYVDKYGQLQTKML  354
AEC15549.1        LINRRTPRVDGQPMYGMDAQVEEVRVFEGTEELPGDPDMMRYVDKYGQLQTKML  354
AEC15081.1        LINRRTPRVDGQPMYGMDAQVEEVRVFEGTEELPGDPDMMRYVDKYGQLQTKML  354
AEC15080.1        LINRRTPRVDGQPMYGMDAQVEEVRVFEGTEELPGDPDMMRYVDKYGQLQTKML  354
AEC15083.1        LINRRTPRVDGQPMYGMDAQVEEVRVFEGTEELPGDPDMMRYVDKYGQLQTKML  354
QWT77109.1        LINRRTPRVDGQPMYGMDAQVEEVRVFEGTEELPGDPDMMRYVDKYGQLQTKML  354
```

AEC15069.1	LINRRTPRVDGQPMYGMDAQVEEVRVFEGTEELPGDPDMMRYVDKYGQLQTKML	354
AEC15070.1	LINRRTPRVDGQPMYGMDAQVEEVRVFEGTEELPGDPDMMRYVDKYGQLQTKML	354
AEC15071.1	LINRRTPRVDGQPMYGMDAQVEEVRVFEGTEELPGDPDMMRYVDKYGQLQTKML	354
AEC15072.1	LINRRTPRVDGQPMYGMDAQVEEVRVFEGTEELPGDPDMMRYVDKYGQLQTKML	354
AEC15073.1	LINRRTPRVDGQPMYGMDAQVEEVRVFEGTEELPGDPDMMRYVDKYGQLQTKML	354
AEC15074.1	LINRRTPRVDGQPMYGMDAQVEEVRVFEGTEELPGDPDMMRYVDKYGQLQTKML	354
AEC15076.1	LINRRTPRVDGQPMYGMDAQVEEVRVFEGTEELPGDPDMMRYVDKYGQLQTKML	354
AEC15077.1	LINRRTPRVDGQPMYGMDAQVEEVRVFEGTEELPGDPDMMRYVDKYGQLQTKML	354
AEC15078.1	LINRRTPRVDGQPMYGMDAQVEEVRVFEGTEELPGDPDMMRYVDKYGQLQTKML	354
AEC15079.1	LINRRTPRVDGQPMYGMDAQVEEVRVFEGTEELPGDPDMMRYVDKYGQLQTKML	354
AEC15082.1	LINRRTPRVDGQPMYGMDAQVEEVRVFEGTEELPGDPDMMRYVDKYGQLQTKML	354
AEC15084.1	LINRRTPRVDGQPMYGMDAQVEEVRVFEGTEELPGDPDMMRYVDKYGQLQTKML	354
AEC15085.1	LINRRTPRVDGQPMYGMDAQVEEVRVFEGTEELPGDPDMMRYVDKYGQLQTKML	354
AEC15086.1	LINRRTPRVDGQPMYGMDAQVEEVRVFEGTEELPGDPDMMRYVDKYGQLQTKML	354
AEC15087.1	LINRRTPRVDGQPMYGMDAQVEEVRVFEGTEELPGDPDMMRYVDKYGQLQTKML	354
AEC15089.1	LINRRTPRVDGQPMYGMDAQVEEVRVFEGTEELPGDPDMMRYVDKYGQLQTKML	354
AEC15555.1	LINRRTPRVDGQPMYGMDAQVEEVRVFEGTEELPGDPDMMRYVDKYGQLQTKML	354
AEC15561.1	LINRRTPRVDGQPMYGMDAQVEEVRVFEGTEELPGDPDMMRYVDKYGQLQTKML	354
AEC15567.1	LINRRTPRVDGQPMYGMDAQVEEVRVFEGTEELPGDPDMMRYVDKYGQLQTKML	354
AEC15573.1	LINRRTPRVDGQPMYGMDAQVEEVRVFEGTEELPGDPDMMRYVDKYGQLQTKML	354
AEC15579.1	LINRRTPRVDGQPMYGMDAQVEEVRVFEGTEELPGDPDMMRYVDKYGQLQTKML	354
AEC15585.1	LINRRTPRVDGQPMYGMDAQVEEVRVFEGTEELPGDPDMMRYVDKYGQLQTKML	354
AEC15591.1	LINRRTPRVDGQPMYGMDAQVEEVRVFEGTEELPGDPDMMRYVDKYGQLQTKML	354
AEC15597.1	LINRRTPRVDGQPMYGMDAQVEEVRVFEGTEELPGDPDMMRYVDKYGQLQTKML	354
AEC15603.1	LINRRTPRVDGQPMYGMDAQVEEVRVFEGTEELPGDPDMMRYVDKYGQLQTKML	354
AEC15609.1	LINRRTPRVDGQPMYGMDAQVEEVRVFEGTEELPGDPDMMRYVDKYGQLQTKML	354
AEC15621.1	LINRRTPRVDGQPMYGMDAQVEEVRVFEGTEELPGDPDMMRYVDKYGQLQTKML	354
AEC15627.1	LINRRTPRVDGQPMYGMDAQVEEVRVFEGTEELPGDPDMMRYVDKYGQLQTKML	354
AEC15633.1	LINRRTPRVDGQPMYGMDAQVEEVRVFEGTEELPGDPDMMRYVDKYGQLQTKML	354
AEC15639.1	LINRRTPRVDGQPMYGMDAQVEEVRVFEGTEELPGDPDMMRYVDKYGQLQTKML	354
AEC15645.1	LINRRTPRVDGQPMYGMDAQVEEVRVFEGTEELPGDPDMMRYVDKYGQLQTKML	354
AEC15651.1	LINRRTPRVDGQPMYGMDAQVEEVRVFEGTEELPGDPDMMRYVDKYGQLQTKML	354
AEC15657.1	LINRRTPRVDGQPMYGMDAQVEEVRVFEGTEELPGDPDMMRYVDKYGQLQTKML	354
AEC15663.1	LINRRTPRVDGQPMYGMDAQVEEVRVFEGTEELPGDPDMMRYVDKYGQLQTKML	354
AEC15669.1	LINRRTPRVDGQPMYGMDAQVEEVRVFEGTEELPGDPDMMRYVDKYGQLQTKML	354
AEC15675.1	LINRRTPRVDGQPMYGMDAQVEEVRVFEGTEELPGDPDMMRYVDKYGQLQTKML	354
AEC15681.1	LINRRTPRVDGQPMYGMDAQVEEVRVFEGTEELPGDPDMMRYVDKYGQLQTKML	354
AEC15687.1	LINRRTPRVDGQPMYGMDAQVEEVRVFEGTEELPGDPDMMRYVDKYGQLQTKML	354
AEC15693.1	LINRRTPRVDGQPMYGMDAQVEEVRVFEGTEELPGDPDMMRYVDKYGQLQTKML	354
AEC15699.1	LINRRTPRVDGQPMYGMDAQVEEVRVFEGTEELPGDPDMMRYVDKYGQLQTKML	354
AEC15705.1	LINRRTPRVDGQPMYGMDAQVEEVRVFEGTEELPGDPDMMRYVDKYGQLQTKML	354
AEC15711.1	LINRRTPRVDGQPMYGMDAQVEEVRVFEGTEELPGDPDMMRYVDKYGQLQTKML	354
AEC15717.1	LINRRTPRVDGQPMYGMDAQVEEVRVFEGTEELPGDPDMMRYVDKYGQLQTKML	354
AEC15723.1	LINRRTPRVDGQPMYGMDAQVEEVRVFEGTEELPGDPDMMRYVDKYGQLQTKML	354
AEC15729.1	LINRRTPRVDGQPMYGMDAQVEEVRVFEGTEELPGDPDMMRYVDKYGQLQTKML	354
AEC15741.1	LINRRTPRVDGQPMYGMDAQVEEVRVFEGTEELPGDPDMMRYVDKYGQLQTKML	354
AEC15747.1	LINRRTPRVDGQPMYGMDAQVEEVRVFEGTEELPGDPDMMRYVDKYGQLQTKML	354
AEC15753.1	LINRRTPRVDGQPMYGMDAQVEEVRVFEGTEELPGDPDMMRYVDKYGQLQTKML	354
AEC15759.1	LINRRTPRVDGQPMYGMDAQVEEVRVFEGTEELPGDPDMMRYVDKYGQLQTKML	354
AEC15765.1	LINRRTPRVDGQPMYGMDAQVEEVRVFEGTEELPGDPDMMRYVDKYGQLQTKML	354
AEC15771.1	LINRRTPRVDGQPMYGMDAQVEEVRVFEGTEELPGDPDMMRYVDKYGQLQTKML	354
AEC15777.1	LINRRTPRVDGQPMYGMDAQVEEVRVFEGTEELPGDPDMMRYVDKYGQLQTKML	354
AEC15789.1	LINRRTPRVDGQPMYGMDAQVEEVRVFEGTEELPGDPDMMRYVDKYGQLQTKML	354
AEC15795.1	LINRRTPRVDGQPMYGMDAQVEEVRVFEGTEELPGDPDMMRYVDKYGQLQTKML	354
AEC15801.1	LINRRTPRVDGQPMYGMDAQVEEVRVFEGTEELPGDPDMMRYVDKYGQLQTKML	354
AEC15807.1	LINRRTPRVDGQPMYGMDAQVEEVRVFEGTEELPGDPDMMRYVDKYGQLQTKML	354
AEC15813.1	LINRRTPRVDGQPMYGMDAQVEEVRVFEGTEELPGDPDMMRYVDKYGQLQTKML	354
AEC15075.1	LINRRTPRVDGQPMYGMDAQVEEVRVFEGTEELPGDPDMMRYVDKYGQLQTKML	354
AEC15088.1	LINRRTPRVDGQPMYGMDAQVEEVRVFEGTEELPGDPDMMRYVDKYGQLQTKML	354
AEC14559.1	LINRRTPRVDGQPMYGMDAQVEEVRVFEGTEELPGDPDMMRYVDRYGQLQTKML	354
AEC15023.1	LINRRTPRVDGQPMYGMDAQVEEVKVFEGTEELPGDPDMMRYVDRYGQLQTKML	354
AEC14518.1	LINRRTPRVDGQPMYGMDAQVEEVRVFEGTEELPGDPDMMRYVDRYGQLQTKML	354
AEC14589.1	LINRRTPRVDGQPMYGMDAQVEEVRVFEGTEELPGDPDMMRYVDRYGQLQTKML	354
AEC14625.1	LINRRTPRVDGQPMYGMDAQVEEVRVFEGTEELPGDPDMMRYVDRYGQLQTKML	354

AEC14631.1	LINRRTPRVDGQPMYGMDAQVEEVRVFEGTEELPGDPDMMRYVDRYGQLQTKML	354
AEC14643.1	LINRRTPRVDGQPMYGMDAQVEEVRVFEGTEELPGDPDMMRYVDRYGQLQTKML	354
AEC14966.1	LINRRTPRVDGQPMYGMDAQVEEVRVFEGTEELPGDPDMMRYVDRYGQLQTKML	354
AEC14979.1	LINRRTPRVDGQPMYGMDAQVEEVRVFEGTEELPGDPDMMRYVDRYGQLQTKML	354
AEC14990.1	LINRRTPRVDGQPMYGMDAQVEEVRVFEGTEELPGDPDMMRYVDRYGQLQTKML	354
AEC14999.1	LINRRTPRVDGQPMYGMDAQVEEVRVFEGTEELPGDPDMMRYVDRYGQLQTKML	354
AEC15020.1	LINRRTPRVDGQPMYGMDAQVEEVRVFEGTEELPGDPDMMRYVDRYGQLQTKML	354
AEC14595.1	LINRRTPRVDGQPMYGMDAQVEEVRVFEGTEELPGDPDMMRYVDRYGQLQTKML	354
AEC14703.1	LINRRTPRVDGQPMYGMDAQVEEVRVFEGTEELPGDPDMMRYVDRYGQLQTKML	354
AEC15011.1	LINRRTPRVDGQPMYGMDAQVEEVRVFEGTEELPGDPDMMRYVDRYGQLQTKML	354
AEC15019.1	LINRRTPRVDGQPMYGMDAQVEEVRVFEGTEELPGDPDMMRYVDRYGQLQTKML	354
AEC15014.1	LINRRTPRVDGQPMYGMDAQVEEVRVFEGTEELPGDPDMMRYVDRYGQLQTKML	354
NP_043511.1	LINRRTPRVDGQPMYGMDAQVEEVRVFEGTEELPGDPDMMRYVDKYGQLQTKML	354
UPN63087.1	LINRRTPRVDGQPMYGMDAQVEEVRVFEGTEELPGDPDMMRYVDKYGQLQTKML	354
ASV51760.1	LINRRTPRVDGQPMYGMDAQVEEVRVFEGTEELPGDPDMMRYVDKYGQLQTKML	354
BAV69379.1	LINRRTPRVDGQPMYGMDAQVEEVRVFEGTEELPGDPDMMRYVDKYGQLQTKML	354
AEC15090.1	LINRRTPRVDGQPMYGMDAQVEEVRVFEGTEELPGDPDMMRYVDKYGQLQTKML	354
AEC15091.1	LINRRTPRVDGQPMYGMDAQVEEVRVFEGTEELPGDPDMMRYVDKYGQLQTKML	354
AEC15092.1	LINRRTPRVDGQPMYGMDAQVEEVRVFEGTEELPGDPDMMRYVDKYGQLQTKML	354
AEC15093.1	LINRRTPRVDGQPMYGMDAQVEEVRVFEGTEELPGDPDMMRYVDKYGQLQTKML	354
AEC15094.1	LINRRTPRVDGQPMYGMDAQVEEVRVFEGTEELPGDPDMMRYVDKYGQLQTKML	354
AEC15095.1	LINRRTPRVDGQPMYGMDAQVEEVRVFEGTEELPGDPDMMRYVDKYGQLQTKML	354
AEC15096.1	LINRRTPRVDGQPMYGMDAQVEEVRVFEGTEELPGDPDMMRYVDKYGQLQTKML	354
AEC15097.1	LINRRTPRVDGQPMYGMDAQVEEVRVFEGTEELPGDPDMMRYVDKYGQLQTKML	354
AEC15098.1	LINRRTPRVDGQPMYGMDAQVEEVRVFEGTEELPGDPDMMRYVDKYGQLQTKML	354
AEC15100.1	LINRRTPRVDGQPMYGMDAQVEEVRVFEGTEELPGDPDMMRYVDKYGQLQTKML	354
AEC15101.1	LINRRTPRVDGQPMYGMDAQVEEVRVFEGTEELPGDPDMMRYVDKYGQLQTKML	354
AEC15102.1	LINRRTPRVDGQPMYGMDAQVEEVRVFEGTEELPGDPDMMRYVDKYGQLQTKML	354
AEC15103.1	LINRRTPRVDGQPMYGMDAQVEEVRVFEGTEELPGDPDMMRYVDKYGQLQTKML	354
AEC15104.1	LINRRTPRVDGQPMYGMDAQVEEVRVFEGTEELPGDPDMMRYVDKYGQLQTKML	354
AEC15105.1	LINRRTPRVDGQPMYGMDAQVEEVRVFEGTEELPGDPDMMRYVDKYGQLQTKML	354
AEC15106.1	LINRRTPRVDGQPMYGMDAQVEEVRVFEGTEELPGDPDMMRYVDKYGQLQTKML	354
AEC15107.1	LINRRTPRVDGQPMYGMDAQVEEVRVFEGTEELPGDPDMMRYVDKYGQLQTKML	354
AEC15108.1	LINRRTPRVDGQPMYGMDAQVEEVRVFEGTEELPGDPDMMRYVDKYGQLQTKML	354
AEC15109.1	LINRRTPRVDGQPMYGMDAQVEEVRVFEGTEELPGDPDMMRYVDKYGQLQTKML	354
AEC15110.1	LINRRTPRVDGQPMYGMDAQVEEVRVFEGTEELPGDPDMMRYVDKYGQLQTKML	354
AEC15111.1	LINRRTPRVDGQPMYGMDAQVEEVRVFEGTEELPGDPDMMRYVDKYGQLQTKML	354
AEC15963.1	LINRRTPRVDGQPMYGMDAQVEEVRVFEGTEELPGDPDMMRYVDKYGQLQTKML	354
AEC15969.1	LINRRTPRVDGQPMYGMDAQVEEVRVFEGTEELPGDPDMMRYVDKYGQLQTKML	354
AEC15975.1	LINRRTPRVDGQPMYGMDAQVEEVRVFEGTEELPGDPDMMRYVDKYGQLQTKML	354
AEC15981.1	LINRRTPRVDGQPMYGMDAQVEEVRVFEGTEELPGDPDMMRYVDKYGQLQTKML	354
AEC15987.1	LINRRTPRVDGQPMYGMDAQVEEVRVFEGTEELPGDPDMMRYVDKYGQLQTKML	354
AEC15993.1	LINRRTPRVDGQPMYGMDAQVEEVRVFEGTEELPGDPDMMRYVDKYGQLQTKML	354
CAD69038.1	LINRRTPRVDGQPMYGMDAQVEEVRVFEGTEELPGDPDMMRYVDKYGQLQTKML	354
AAK70240.1	LINRRTPRVDGQPMYGMDAQVEEVRVFEGTEELPGDPDMMRYVDKYGQLQTKML	354
BAB11710.1	LINRRTPRVDGQPMYGMDAQVEEVRVFEGTEELPGDPDMMRYVDKYGQLQTKML	354
BAA01958.1	LINRRTPRVDGQPMYGMDAQVEEVRVFEGTEELPGDPDMMRYVDKYGQLQTKML	354
AAA82101.1	LINRRTPRVDGQPMYGMDAQVEEVRVFEGTEELPGDPDMMRYVDKYGQLQTKML	354
P03089.1	LINRRTPRVDGQPMYGMDAQVEEVRVFEGTEELPGDPDMMRYVDKYGQLQTKML	354
UPN63102.1	LINRRTPRVDGQPMYGMDAQVEEVRVFEGTEELPGDPDMMRYVDKYGQLQTKML	354
AEC15025.1	LINRRTPRVDGQPMYGMDAQVEEVRVFEGTEELPGDPDMMRYVDKYGQLQTKML	354
AEC15026.1	LINRRTPRVDGQPMYGMDAQVEEVRVFEGTEELPGDPDMMRYVDKYGQLQTKML	354
AEC15027.1	LINRRTPRVDGQPMYGMDAQVEEVRVFEGTEELPGDPDMMRYVDKYGQLQTKML	354
AEC15028.1	LINRRTPRVDGQPMYGMDAQVEEVRVFEGTEELPGDPDMMRYVDKYGQLQTKML	354
AEC15029.1	LINRRTPRVDGQPMYGMDAQVEEVRVFEGTEELPGDPDMMRYVDKYGQLQTKML	354
AEC15196.1	LINRRTPRVDGQPMYGMDAQVEEVRVFEGTEELPGDPDMMRYVDKYGQLQTKML	354
AEC15197.1	LINRRTPRVDGQPMYGMDAQVEEVRVFEGTEELPGDPDMMRYVDKYGQLQTKML	354
AEC15198.1	LINRRTPRVDGQPMYGMDAQVEEVRVFEGTEELPGDPDMMRYVDKYGQLQTKML	354
AEC15200.1	LINRRTPRVDGQPMYGMDAQVEEVRVFEGTEELPGDPDMMRYVDKYGQLQTKML	354
AEC15201.1	LINRRTPRVDGQPMYGMDAQVEEVRVFEGTEELPGDPDMMRYVDKYGQLQTKML	354
AEC15202.1	LINRRTPRVDGQPMYGMDAQVEEVRVFEGTEELPGDPDMMRYVDKYGQLQTKML	354
AEC15203.1	LINRRTPRVDGQPMYGMDAQVEEVRVFEGTEELPGDPDMMRYVDKYGQLQTKML	354
AEC15204.1	LINRRTPRVDGQPMYGMDAQVEEVRVFEGTEELPGDPDMMRYVDKYGQLQTKML	354
AEC15205.1	LINRRTPRVDGQPMYGMDAQVEEVRVFEGTEELPGDPDMMRYVDKYGQLQTKML	354

AEC15206.1	LINRRTPRVDGQPMYGMDAQVEEVRVFEGTEELPGDPDMMRYVDKYGQLQTKML	354
AEC15207.1	LINRRTPRVDGQPMYGMDAQVEEVRVFEGTEELPGDPDMMRYVDKYGQLQTKML	354
AEC15208.1	LINRRTPRVDGQPMYGMDAQVEEVRVFEGTEELPGDPDMMRYVDKYGQLQTKML	354
AEC15210.1	LINRRTPRVDGQPMYGMDAQVEEVRVFEGTEELPGDPDMMRYVDKYGQLQTKML	354
AEC15211.1	LINRRTPRVDGQPMYGMDAQVEEVRVFEGTEELPGDPDMMRYVDKYGQLQTKML	354
AEC15212.1	LINRRTPRVDGQPMYGMDAQVEEVRVFEGTEELPGDPDMMRYVDKYGQLQTKML	354
AEC15213.1	LINRRTPRVDGQPMYGMDAQVEEVRVFEGTEELPGDPDMMRYVDKYGQLQTKML	354
AEC15214.1	LINRRTPRVDGQPMYGMDAQVEEVRVFEGTEELPGDPDMMRYVDKYGQLQTKML	354
AEC15215.1	LINRRTPRVDGQPMYGMDAQVEEVRVFEGTEELPGDPDMMRYVDKYGQLQTKML	354
AEC15216.1	LINRRTPRVDGQPMYGMDAQVEEVRVFEGTEELPGDPDMMRYVDKYGQLQTKML	354
AEC15217.1	LINRRTPRVDGQPMYGMDAQVEEVRVFEGTEELPGDPDMMRYVDKYGQLQTKML	354
AEC15218.1	LINRRTPRVDGQPMYGMDAQVEEVRVFEGTEELPGDPDMMRYVDKYGQLQTKML	354
AEC15327.1	LINRRTPRVDGQPMYGMDAQVEEVRVFEGTEELPGDPDMMRYVDKYGQLQTKML	354
AEC15333.1	LINRRTPRVDGQPMYGMDAQVEEVRVFEGTEELPGDPDMMRYVDKYGQLQTKML	354
AEC15339.1	LINRRTPRVDGQPMYGMDAQVEEVRVFEGTEELPGDPDMMRYVDKYGQLQTKML	354
AEC15345.1	LINRRTPRVDGQPMYGMDAQVEEVRVFEGTEELPGDPDMMRYVDKYGQLQTKML	354
AEC15351.1	LINRRTPRVDGQPMYGMDAQVEEVRVFEGTEELPGDPDMMRYVDKYGQLQTKML	354
AEC15357.1	LINRRTPRVDGQPMYGMDAQVEEVRVFEGTEELPGDPDMMRYVDKYGQLQTKML	354
AEC15363.1	LINRRTPRVDGQPMYGMDAQVEEVRVFEGTEELPGDPDMMRYVDKYGQLQTKML	354
AEC15369.1	LINRRTPRVDGQPMYGMDAQVEEVRVFEGTEELPGDPDMMRYVDKYGQLQTKML	354
AEC15375.1	LINRRTPRVDGQPMYGMDAQVEEVRVFEGTEELPGDPDMMRYVDKYGQLQTKML	354
AEC15381.1	LINRRTPRVDGQPMYGMDAQVEEVRVFEGTEELPGDPDMMRYVDKYGQLQTKML	354
AEC15387.1	LINRRTPRVDGQPMYGMDAQVEEVRVFEGTEELPGDPDMMRYVDKYGQLQTKML	354
AEC15393.1	LINRRTPRVDGQPMYGMDAQVEEVRVFEGTEELPGDPDMMRYVDKYGQLQTKML	354
AEC15399.1	LINRRTPRVDGQPMYGMDAQVEEVRVFEGTEELPGDPDMMRYVDKYGQLQTKML	354
AEC15405.1	LINRRTPRVDGQPMYGMDAQVEEVRVFEGTEELPGDPDMMRYVDKYGQLQTKML	354
AEC15411.1	LINRRTPRVDGQPMYGMDAQVEEVRVFEGTEELPGDPDMMRYVDKYGQLQTKML	354
AEC15417.1	LINRRTPRVDGQPMYGMDAQVEEVRVFEGTEELPGDPDMMRYVDKYGQLQTKML	354
AEC15423.1	LINRRTPRVDGQPMYGMDAQVEEVRVFEGTEELPGDPDMMRYVDKYGQLQTKML	354
AEC15429.1	LINRRTPRVDGQPMYGMDAQVEEVRVFEGTEELPGDPDMMRYVDKYGQLQTKML	354
AEC15435.1	LINRRTPRVDGQPMYGMDAQVEEVRVFEGTEELPGDPDMMRYVDKYGQLQTKML	354
AEC15441.1	LINRRTPRVDGQPMYGMDAQVEEVRVFEGTEELPGDPDMMRYVDKYGQLQTKML	354
BAA01967.1	LINRRTPRVDGQPMYGMDAQVEEVRVFEGTEELPGDPDMMRYVDKYGQLQTKML	354
ASV51844.1	LINRRTPRVDGQPMYGMDAQVEEVRVFEGTEELPGDPDMMRYVDKYGQLQTKML	354
ASV51850.1	LINRRTPRVDGQPMYGMDAQVEEVRVFEGTEELPGDPDMMRYVDKYGQLQTKML	354
CCW72489.1	LINRRTPRVDGQPMYGMDAQVEEVRVFEGTEELPGDPDMMRYVDKYGQLQTKML	354
AEC15170.1	LINRRTPRVDGQPMYGMDAQVEEVRVFEGTEELPGDPDMMRYVDKYGQLQTKML	354
AEC15171.1	LINRRTPRVDGQPMYGMDAQVEEVRVFEGTEELPGDPDMMRYVDKYGQLQTKML	354
AEC15299.1	LINRRTPRVDGQPMYGMDTQVEEVRVFEGTEELPGDPDMMRYVDKYGQLQTKML	354
BAB93050.1	LINRRTPRVDGQPMYGMDAQVEEVRVFEGTEELPGDPDMMRYVDKYGQLQTKML	354
UPN63100.1	LINRRTPRVDGQPMYGMDAQVEEVRVFEGTEELPGDPDMMRYVDKYGQLQTKML	354
CCW72482.1	LINRRTPRVDGQPMYGMDAQVEEVRVFEGTEELPGDPDMMRYVDKYGQLQTKML	354
AEC15165.1	LINRRTPRVDGQPMYGMDAQVEEVRVFEGTEELPGDPDMMRYVDKYGQLQTKML	354
AEC15166.1	LINRRTPRVDGQPMYGMDAQVEEVRVFEGTEELPGDPDMMRYVDKYGQLQTKML	354
AEC15266.1	LINRRTPRVDGQPMYGMDAQVEEVRVFEGTEELPGDPDMMRYVDKYGQLQTKML	354
AEC15267.1	LINRRTPRVDGQPMYGMDAQVEEVRVFEGTEELPGDPDMMRYVDKYGQLQTKML	354
AEC15268.1	LINRRTPRVDGQPMYGMDAQVEEVRVFEGTEELPGDPDMMRYVDKYGQLQTKML	354
AEC15269.1	LINRRTPRVDGQPMYGMDAQVEEVRVFEGTEELPGDPDMMRYVDKYGQLQTKML	354
AEC15270.1	LINRRTPRVDGQPMYGMDAQVEEVRVFEGTEELPGDPDMMRYVDKYGQLQTKML	354
AEC15271.1	LINRRTPRVDGQPMYGMDAQVEEVRVFEGTEELPGDPDMMRYVDKYGQLQTKML	354
AEC15272.1	LINRRTPRVDGQPMYGMDAQVEEVRVFEGTEELPGDPDMMRYVDKYGQLQTKML	354
AEC15273.1	LINRRTPRVDGQPMYGMDAQVEEVRVFEGTEELPGDPDMMRYVDKYGQLQTKML	354
AEC15274.1	LINRRTPRVDGQPMYGMDAQVEEVRVFEGTEELPGDPDMMRYVDKYGQLQTKML	354
AEC15275.1	LINRRTPRVDGQPMYGMDAQVEEVRVFEGTEELPGDPDMMRYVDKYGQLQTKML	354
AEC15276.1	LINRRTPRVDGQPMYGMDAQVEEVRVFEGTEELPGDPDMMRYVDKYGQLQTKML	354
AEC15277.1	LINRRTPRVDGQPMYGMDAQVEEVRVFEGTEELPGDPDMMRYVDKYGQLQTKML	354
AEC15278.1	LINRRTPRVDGQPMYGMDAQVEEVRVFEGTEELPGDPDMMRYVDKYGQLQTKML	354
AEC15279.1	LINRRTPRVDGQPMYGMDAQVEEVRVFEGTEELPGDPDMMRYVDKYGQLQTKML	354
AEC15280.1	LINRRTPRVDGQPMYGMDAQVEEVRVFEGTEELPGDPDMMRYVDKYGQLQTKML	354
AEC15281.1	LINRRTPRVDGQPMYGMDAQVEEVRVFEGTEELPGDPDMMRYVDKYGQLQTKML	354
AEC15282.1	LINRRTPRVDGQPMYGMDAQVEEVRVFEGTEELPGDPDMMRYVDKYGQLQTKML	354
AEC15283.1	LINRRTPRVDGQPMYGMDAQVEEVRVFEGTEELPGDPDMMRYVDKYGQLQTKML	354
AEC15284.1	LINRRTPRVDGQPMYGMDAQVEEVRVFEGTEELPGDPDMMRYVDKYGQLQTKML	354
AEC15285.1	LINRRTPRVDGQPMYGMDAQVEEVRVFEGTEELPGDPDMMRYVDKYGQLQTKML	354

AEC15286.1	LINRRTPRVDGQPMYGMDAQVEEVRVFEGTEELPGDPDMMRYVDKYGQLQTKML	354
AEC15287.1	LINRRTPRVDGQPMYGMDAQVEEVRVFEGTEELPGDPDMMRYVDKYGQLQTKML	354
AEC15288.1	LINRRTPRVDGQPMYGMDAQVEEVRVFEGTEELPGDPDMMRYVDKYGQLQTKML	354
AEC15289.1	LINRRTPRVDGQPMYGMDAQVEEVRVFEGTEELPGDPDMMRYVDKYGQLQTKML	354
AEC15290.1	LINRRTPRVDGQPMYGMDAQVEEVRVFEGTEELPGDPDMMRYVDKYGQLQTKML	354
AEC15291.1	LINRRTPRVDGQPMYGMDAQVEEVRVFEGTEELPGDPDMMRYVDKYGQLQTKML	354
AEC15292.1	LINRRTPRVDGQPMYGMDAQVEEVRVFEGTEELPGDPDMMRYVDKYGQLQTKML	354
AEC15293.1	LINRRTPRVDGQPMYGMDAQVEEVRVFEGTEELPGDPDMMRYVDKYGQLQTKML	354
AEC15294.1	LINRRTPRVDGQPMYGMDAQVEEVRVFEGTEELPGDPDMMRYVDKYGQLQTKML	354
AEC15295.1	LINRRTPRVDGQPMYGMDAQVEEVRVFEGTEELPGDPDMMRYVDKYGQLQTKML	354
AEC15296.1	LINRRTPRVDGQPMYGMDAQVEEVRVFEGTEELPGDPDMMRYVDKYGQLQTKML	354
AEC15297.1	LINRRTPRVDGQPMYGMDAQVEEVRVFEGTEELPGDPDMMRYVDKYGQLQTKML	354
AEC15298.1	LINRRTPRVDGQPMYGMDAQVEEVRVFEGTEELPGDPDMMRYVDKYGQLQTKML	354
AEC15300.1	LINRRTPRVDGQPMYGMDAQVEEVRVFEGTEELPGDPDMMRYVDKYGQLQTKML	354
AEC15301.1	LINRRTPRVDGQPMYGMDAQVEEVRVFEGTEELPGDPDMMRYVDKYGQLQTKML	354
AEC15302.1	LINRRTPRVDGQPMYGMDAQVEEVRVFEGTEELPGDPDMMRYVDKYGQLQTKML	354
AEC15303.1	LINRRTPRVDGQPMYGMDAQVEEVRVFEGTEELPGDPDMMRYVDKYGQLQTKML	354
AEC15304.1	LINRRTPRVDGQPMYGMDAQVEEVRVFEGTEELPGDPDMMRYVDKYGQLQTKML	354
AEC15305.1	LINRRTPRVDGQPMYGMDAQVEEVRVFEGTEELPGDPDMMRYVDKYGQLQTKML	354
AEC15306.1	LINRRTPRVDGQPMYGMDAQVEEVRVFEGTEELPGDPDMMRYVDKYGQLQTKML	354
AEC15307.1	LINRRTPRVDGQPMYGMDAQVEEVRVFEGTEELPGDPDMMRYVDKYGQLQTKML	354
AEC15308.1	LINRRTPRVDGQPMYGMDAQVEEVRVFEGTEELPGDPDMMRYVDKYGQLQTKML	354
AEC15309.1	LINRRTPRVDGQPMYGMDAQVEEVRVFEGTEELPGDPDMMRYVDKYGQLQTKML	354
AEC15310.1	LINRRTPRVDGQPMYGMDAQVEEVRVFEGTEELPGDPDMMRYVDKYGQLQTKML	354
AEC15311.1	LINRRTPRVDGQPMYGMDAQVEEVRVFEGTEELPGDPDMMRYVDKYGQLQTKML	354
AEC15861.1	LINRRTPRVDGQPMYGMDAQVEEVRVFEGTEELPGDPDMMRYVDKYGQLQTKML	354
AEC15867.1	LINRRTPRVDGQPMYGMDAQVEEVRVFEGTEELPGDPDMMRYVDKYGQLQTKML	354
AEC15873.1	LINRRTPRVDGQPMYGMDAQVEEVRVFEGTEELPGDPDMMRYVDKYGQLQTKML	354
AEC15879.1	LINRRTPRVDGQPMYGMDAQVEEVRVFEGTEELPGDPDMMRYVDKYGQLQTKML	354
AEC15885.1	LINRRTPRVDGQPMYGMDAQVEEVRVFEGTEELPGDPDMMRYVDKYGQLQTKML	354
AEC15891.1	LINRRTPRVDGQPMYGMDAQVEEVRVFEGTEELPGDPDMMRYVDKYGQLQTKML	354
AEC15897.1	LINRRTPRVDGQPMYGMDAQVEEVRVFEGTEELPGDPDMMRYVDKYGQLQTKML	354
AEC15903.1	LINRRTPRVDGQPMYGMDAQVEEVRVFEGTEELPGDPDMMRYVDKYGQLQTKML	354
AEC15909.1	LINRRTPRVDGQPMYGMDAQVEEVRVFEGTEELPGDPDMMRYVDKYGQLQTKML	354
AEC15915.1	LINRRTPRVDGQPMYGMDAQVEEVRVFEGTEELPGDPDMMRYVDKYGQLQTKML	354
AEC15921.1	LINRRTPRVDGQPMYGMDAQVEEVRVFEGTEELPGDPDMMRYVDKYGQLQTKML	354
AEC15927.1	LINRRTPRVDGQPMYGMDAQVEEVRVFEGTEELPGDPDMMRYVDKYGQLQTKML	354
AEC15933.1	LINRRTPRVDGQPMYGMDAQVEEVRVFEGTEELPGDPDMMRYVDKYGQLQTKML	354
AEC15939.1	LINRRTPRVDGQPMYGMDAQVEEVRVFEGTEELPGDPDMMRYVDKYGQLQTKML	354
AEC15945.1	LINRRTPRVDGQPMYGMDAQVEEVRVFEGTEELPGDPDMMRYVDKYGQLQTKML	354
AEC15951.1	LINRRTPRVDGQPMYGMDAQVEEVRVFEGTEELPGDPDMMRYVDKYGQLQTKML	354
AEC15957.1	LINRRTPRVDGQPMYGMDAQVEEVRVFEGTEELPGDPDMMRYVDKYGQLQTKML	354
BAD21259.1	LINRRTPRVDGQPMYGMDAQVEEVRVFEGTEELPGDPDMMRYVDKYGQLQTKML	354
BAD21265.1	LINRRTPRVDGQPMYGMDAQVEEVRVFEGTEELPGDPDMMRYVDKYGQLQTKML	354
AAC59325.1	LINRRTPRVDGQPMYGMDAQVEEVRVFEGTEELPGDPDMMRYVDKYGQLQTKML	354
AEC14524.1	LINRRTPRVDGQPMYGMDAQVEEVRVFEGTEELPGDPDMMRYVDRYGQLQTKML	354
AEC14541.1	LINRRTPRVDGQPMYGMDAQVEEVRVFEGTEELPGDPDMMRYVDRYGQLQTKML	354
AEC14565.1	LINRRTPRVDGQPMYGMDAQVEEVRVFEGTEELPGDPDMMRYVDRYGQLQTKML	354
AEC14571.1	LINRRTPRVDGQPMYGMDAQVEEVRVFEGTEELPGDPDMMRYVDRYGQLQTKML	354
AEC14583.1	LINRRTPRVDGQPMYGMDAQVEEVRVFEGTEELPGDPDMMRYVDRYGQLQTKML	354
AEC14601.1	LINRRTPRVDGQPMYGMDAQVEEVRVFEGTEELPGDPDMMRYVDRYGQLQTKML	354
AEC14607.1	LINRRTPRVDGQPMYGMDAQVEEVRVFEGTEELPGDPDMMRYVDRYGQLQTKML	354
AEC14637.1	LINRRTPRVDGQPMYGMDAQVEEVRVFEGTEELPGDPDMMRYVDRYGQLQTKML	354
AEC14649.1	LINRRTPRVDGQPMYGMDAQVEEVRVFEGTEELPGDPDMMRYVDRYGQLQTKML	354
AEC14655.1	LINRRTPRVDGQPMYGMDAQVEEVRVFEGTEELPGDPDMMRYVDRYGQLQTKML	354
AEC14661.1	LINRRTPRVDGQPMYGMDAQVEEVRVFEGTEELPGDPDMMRYVDRYGQLQTKML	354
AEC14685.1	LINRRTPRVDGQPMYGMDAQVEEVRVFEGTEELPGDPDMMRYVDRYGQLQTKML	354
AEC14697.1	LINRRTPRVDGQPMYGMDAQVEEVRVFEGTEELPGDPDMMRYVDRYGQLQTKML	354
AEC14709.1	LINRRTPRVDGQPMYGMDAQVEEVRVFEGTEELPGDPDMMRYVDRYGQLQTKML	354
AEC14715.1	LINRRTPRVDGQPMYGMDAQVEEVRVFEGTEELPGDPDMMRYVDRYGQLQTKML	354
AEC14963.1	LINRRTPRVDGQPMYGMDAQVEEVRVFEGTEELPGDPDMMRYVDRYGQLQTKML	354
AEC14968.1	LINRRTPRVDGQPMYGMDAQVEEVRVFEGTEELPGDPDMMRYVDRYGQLQTKML	354
AEC14971.1	LINRRTPRVDGQPMYGMDAQVEEVRVFEGTEELPGDPDMMRYVDRYGQLQTKML	354
AEC14972.1	LINRRTPRVDGQPMYGMDAQVEEVRVFEGTEELPGDPDMMRYVDRYGQLQTKML	354

AEC14975.1	LINRRTPRVDGQPMYGMDAQVEEVRVFEGTEELPGDPDMMRYVDRYGQLQTKML	354
AEC14976.1	LINRRTPRVDGQPMYGMDAQVEEVRVFEGTEELPGDPDMMRYVDRYGQLQTKML	354
AEC14977.1	LINRRTPRVDGQPMYGMDAQVEEVRVFEGTEELPGDPDMMRYVDRYGQLQTKML	354
AEC14978.1	LINRRTPRVDGQPMYGMDAQVEEVRVFEGTEELPGDPDMMRYVDRYGQLQTKML	354
AEC14980.1	LINRRTPRVDGQPMYGMDAQVEEVRVFEGTEELPGDPDMMRYVDRYGQLQTKML	354
AEC14981.1	LINRRTPRVDGQPMYGMDAQVEEVRVFEGTEELPGDPDMMRYVDRYGQLQTKML	354
AEC14982.1	LINRRTPRVDGQPMYGMDAQVEEVRVFEGTEELPGDPDMMRYVDRYGQLQTKML	354
AEC14983.1	LINRRTPRVDGQPMYGMDAQVEEVRVFEGTEELPGDPDMMRYVDRYGQLQTKML	354
AEC14985.1	LINRRTPRVDGQPMYGMDAQVEEVRVFEGTEELPGDPDMMRYVDRYGQLQTKML	354
AEC14986.1	LINRRTPRVDGQPMYGMDAQVEEVRVFEGTEELPGDPDMMRYVDRYGQLQTKML	354
AEC14988.1	LINRRTPRVDGQPMYGMDAQVEEVRVFEGTEELPGDPDMMRYVDRYGQLQTKML	354
AEC14989.1	LINRRTPRVDGQPMYGMDAQVEEVRVFEGTEELPGDPDMMRYVDRYGQLQTKML	354
AEC14991.1	LINRRTPRVDGQPMYGMDAQVEEVRVFEGTEELPGDPDMMRYVDRYGQLQTKML	354
AEC14992.1	LINRRTPRVDGQPMYGMDAQVEEVRVFEGTEELPGDPDMMRYVDRYGQLQTKML	354
AEC14996.1	LINRRTPRVDGQPMYGMDAQVEEVRVFEGTEELPGDPDMMRYVDRYGQLQTKML	354
AEC14997.1	LINRRTPRVDGQPMYGMDAQVEEVRVFEGTEELPGDPDMMRYVDRYGQLQTKML	354
AEC14998.1	LINRRTPRVDGQPMYGMDAQVEEVRVFEGTEELPGDPDMMRYVDRYGQLQTKML	354
AEC15002.1	LINRRTPRVDGQPMYGMDAQVEEVRVFEGTEELPGDPDMMRYVDRYGQLQTKML	354
AEC15003.1	LINRRTPRVDGQPMYGMDAQVEEVRVFEGTEELPGDPDMMRYVDRYGQLQTKML	354
AEC15004.1	LINRRTPRVDGQPMYGMDAQVEEVRVFEGTEELPGDPDMMRYVDRYGQLQTKML	354
AEC15005.1	LINRRTPRVDGQPMYGMDAQVEEVRVFEGTEELPGDPDMMRYVDRYGQLQTKML	354
AEC15006.1	LINRRTPRVDGQPMYGMDAQVEEVRVFEGTEELPGDPDMMRYVDRYGQLQTKML	354
AEC15009.1	LINRRTPRVDGQPMYGMDAQVEEVRVFEGTEELPGDPDMMRYVDRYGQLQTKML	354
AEC15012.1	LINRRTPRVDGQPMYGMDAQVEEVRVFEGTEELPGDPDMMRYVDRYGQLQTKML	354
AEC15013.1	LINRRTPRVDGQPMYGMDAQVEEVRVFEGTEELPGDPDMMRYVDRYGQLQTKML	354
AEC15015.1	LINRRTPRVDGQPMYGMDAQVEEVRVFEGTEELPGDPDMMRYVDRYGQLQTKML	354
AEC15017.1	LINRRTPRVDGQPMYGMDAQVEEVRVFEGTEELPGDPDMMRYVDRYGQLQTKML	354
AEC15018.1	LINRRTPRVDGQPMYGMDAQVEEVRVFEGTEELPGDPDMMRYVDRYGQLQTKML	354
AEC15021.1	LINRRTPRVDGQPMYGMDAQVEEVRVFEGTEELPGDPDMMRYVDRYGQLQTKML	354
AEC15022.1	LINRRTPRVDGQPMYGMDAQVEEVRVFEGTEELPGDPDMMRYVDRYGQLQTKML	354
AEC14962.1	LINRRTPRVDGQPMYGMDAQVEEVRVFEGTEELPGDPDMMRYVDRYGQLQTKML	354
AEC14993.1	LINRRTPRVDGQPMYGMDAQVEEVRVFEGTEELPGDPDMMRYVDRYGQLQTKML	354
AEC15007.1	LINRRTPRVDGQPMYGMDAQVEEVRVFEGTEELPGDPDMMRYVDRYGQLQTKML	354
CCW72517.1	LINRRTPRVDGQPMYGMDAQVEEVRVFEGTEELPGDPDMMRYVDKYGQLQTKML	354
BAD21247.2	LINRRTPRVDGQPMYGMDAQVEEVRVFEGTEELPGDPDMMRYVDKYGQLQTKML	354
AAL37695.1	LINRRTPRVDGQPMYGMDAQVEEVRVFEGTEELPGDPDMMRYVDRYGQLQTKML	354
AEC15242.1	LINRRTPRVDGQPMYGMDAQVEEVRVFEGTEELPGDPDMMRYVDRYGQLQTKML	354
AEC15243.1	LINRRTPRVDGQPMYGMDAQVEEVRVFEGTEELPGDPDMMRYVDRYGQLQTKML	354
AEC15244.1	LINRRTPRVDGQPMYGMDAQVEEVRVFEGTEELPGDPDMMRYVDRYGQLQTKML	354
AEC15245.1	LINRRTPRVDGQPMYGMDAQVEEVRVFEGTEELPGDPDMMRYVDRYGQLQTKML	354
AEC15246.1	LINRRTPRVDGQPMYGMDAQVEEVRVFEGTEELPGDPDMMRYVDRYGQLQTKML	354
AEC15247.1	LINRRTPRVDGQPMYGMDAQVEEVRVFEGTEELPGDPDMMRYVDRYGQLQTKML	354
AEC15248.1	LINRRTPRVDGQPMYGMDAQVEEVRVFEGTEELPGDPDMMRYVDRYGQLQTKML	354
AEC15249.1	LINRRTPRVDGQPMYGMDAQVEEVRVFEGTEELPGDPDMMRYVDRYGQLQTKML	354
AEC15250.1	LINRRTPRVDGQPMYGMDAQVEEVRVFEGTEELPGDPDMMRYVDRYGQLQTKML	354
AEC15251.1	LINRRTPRVDGQPMYGMDAQVEEVRVFEGTEELPGDPDMMRYVDRYGQLQTKML	354
AEC15252.1	LINRRTPRVDGQPMYGMDAQVEEVRVFEGTEELPGDPDMMRYVDRYGQLQTKML	354
AEC15253.1	LINRRTPRVDGQPMYGMDAQVEEVRVFEGTEELPGDPDMMRYVDRYGQLQTKML	354
AEC15254.1	LINRRTPRVDGQPMYGMDAQVEEVRVFEGTEELPGDPDMMRYVDRYGQLQTKML	354
AEC15255.1	LINRRTPRVDGQPMYGMDAQVEEVRVFEGTEELPGDPDMMRYVDRYGQLQTKML	354
AEC15256.1	LINRRTPRVDGQPMYGMDAQVEEVRVFEGTEELPGDPDMMRYVDRYGQLQTKML	354
AEC15257.1	LINRRTPRVDGQPMYGMDAQVEEVRVFEGTEELPGDPDMMRYVDRYGQLQTKML	354
AEC15258.1	LINRRTPRVDGQPMYGMDAQVEEVRVFEGTEELPGDPDMMRYVDRYGQLQTKML	354
AEC15259.1	LINRRTPRVDGQPMYGMDAQVEEVRVFEGTEELPGDPDMMRYVDRYGQLQTKML	354
AEC15260.1	LINRRTPRVDGQPMYGMDAQVEEVRVFEGTEELPGDPDMMRYVDRYGQLQTKML	354
AEC15261.1	LINRRTPRVDGQPMYGMDAQVEEVRVFEGTEELPGDPDMMRYVDRYGQLQTKML	354
AEC15262.1	LINRRTPRVDGQPMYGMDAQVEEVRVFEGTEELPGDPDMMRYVDRYGQLQTKML	354
AEC15263.1	LINRRTPRVDGQPMYGMDAQVEEVRVFEGTEELPGDPDMMRYVDRYGQLQTKML	354
AEC15264.1	LINRRTPRVDGQPMYGMDAQVEEVRVFEGTEELPGDPDMMRYVDRYGQLQTKML	354
AEC15265.1	LINRRTPRVDGQPMYGMDAQVEEVRVFEGTEELPGDPDMMRYVDRYGQLQTKML	354
AEC15000.1	LINRRTPRVDGQPMYGMDAQVEEVRVFEGTEELPGDPDMMRYVDRYGQLQTKML	354
AEC14721.1	LINRRTPRVDGQPMYGMDAQVEEVRVFEGTEELPGDPDMMRYVDRYGQLQTKML	354
AEC14965.1	LINRRTPRVDGQPMYGMDAQVEEVRVFEGTEELPGDPDMMRYVDRYGQLQTKML	354
AEC14967.1	LINRRTPRVDGQPMYGMDAQVEEVRVFEGTEELPGDPDMMRYVDRYGQLQTKML	354

AEC14969.1	LINRRTPRVDGQPMYGMDAQVEEVRVFEGTEELPGDPDMMRYVDRYGQLQTKML	354
AEC14973.1	LINRRTPRVDGQPMYGMDAQVEEVRVFEGTEELPGDPDMMRYVDRYGQLQTKML	354
AEC14974.1	LINRRTPRVDGQPMYGMDAQVEEVRVFEGTEELPGDPDMMRYVDRYGQLQTKML	354
AEC14994.1	LINRRTPRVDGQPMYGMDAQVEEVRVFEGTEELPGDPDMMRYVDRYGQLQTKML	354
AEC15001.1	LINRRTPRVDGQPMYGMDAQVEEVRVFEGTEELPGDPDMMRYVDRYGQLQTKML	354
AEC15024.1	LINRRTPRVDGQPMYGMDAQVEEVRVFEGTEELPGDPDMMRYVDRYGQLQTKML	354
AEC14667.1	LINRRTPRVDGQPMYGMDAQVEEVRVFEGTEELPGDPDMMRYVDRYGQLQTKML	354
UPN63091.1	LINRRTPRVDGQPMYGMDAQVEEVRVFEGTEELPGDPDMMRYVDRYGQLQTKML	354
AEC14535.1	LINRRTPRVDGQPMYGMDAQVEEVRVFEGTEELPGDPDMMRYVDRYGQLQTKML	354
AEC14547.1	LINRRTPRVDGQPMYGMDAQVEEVRVFEGTEELPGDPDMMRYVDRYGQLQTKML	354
AEC14553.1	LINRRTPRVDGQPMYGMDAQVEEVRVFEGTEELPGDPDMMRYVDRYGQLQTKML	354
AEC14577.1	LINRRTPRVDGQPMYGMDAQVEEVRVFEGTEELPGDPDMMRYVDRYGQLQTKML	354
AEC14613.1	LINRRTPRVDGQPMYGMDAQVEEVRVFEGTEELPGDPDMMRYVDRYGQLQTKML	354
AEC14673.1	LINRRTPRVDGQPMYGMDAQVEEVRVFEGTEELPGDPDMMRYVDRYGQLQTKML	354
AEC14679.1	LINRRTPRVDGQPMYGMDAQVEEVRVFEGTEELPGDPDMMRYVDRYGQLQTKML	354
AEC14727.1	LINRRTPRVDGQPMYGMDAQVEEVRVFEGTEELPGDPDMMRYVDRYGQLQTKML	354
AEC14733.1	LINRRTPRVDGQPMYGMDAQVEEVRVFEGTEELPGDPDMMRYVDRYGQLQTKML	354
AEC14964.1	LINRRTPRVDGQPMYGMDAQVEEVRVFEGTEELPGDPDMMRYVDRYGQLQTKML	354
AEC14995.1	LINRRTPRVDGQPMYGMDAQVEEVRVFEGTEELPGDPDMMRYVDRYGQLQTKML	354
AEC15008.1	LINRRTPRVDGQPMYGMDAQVEEVRVFEGTEELPGDPDMMRYVDRYGQLQTKML	354
AEC14970.1	LINRRTPRVDGQPMYGMDAQVEEVRVFEGTEELPGDPDMMRYVDRYGQLQTKML	354
QTJ15162.1	LINRRTPRVDGQPMYGMDAQIEEVRVFEGTEELPGDPDMMRYVDKYGQLQTKML	354
UYL83831.1	LINRRTPRVDGQPMYGMDAQVEEVRVFEGTEELPGDPDMMRYVDRYGQLQTKML	351
ALF06183.1	LINRRTPRVDGQPMYGMDAQXEEVRVFEGTEZLPGDPDMMRYVDXYGQLQTKM-	353
QCH41111.1	LINRRTPRVDGQPMYGMDAQVEEVKVFEGTEELPGDPDLMRYVDKYGQLQTKML	354
QCH41112.1	LINRRTPRVDGQPMYGMDAQVEEVKVFEGTEELPGDPDLMRYVDKYGQLQTKML	354
AEC14749.1	SINRRTPRVDGQPMYGMDAQVEEVRVFEGTEELPGDPDMMRYVDRYGQLQTKML	354
ALF06202.1	LINRRTPRVDGQPMYGMDAQVEEVRVFEGTEELPGDPDMMRYVDRYGQLQTK--	352
ALF06186.1	LINRRTPRVDGQPMYGMDAQVEEVRVFEGTEELPGDPDMMRYVDRYGQLQTKML	354
ALF06208.1	LINRRTPRVDGQPMYGMDAQVEEVRVFEGTEELPGDPDMMRYVDRYGQLQTK--	352
ALF06197.1	LINRRTPRVDGQPMYGMDAQVEEVRVFEGTEELPGDPDMMRYVDRYGQLQTK--	352
AEC15037.1	LINRRTPRVDGQPMYGMDAQVEEVRVFEGTEELPGDPDMMRYVDRYGQLQTKML	354
UPN63109.1	LINRRTPRVDGQPMYGMDAQVEEVRVFEGTEELPGDPDMMRYVDRYAHLQSKML	354
UPN63107.1	LINRRTPRVDGQPMYGMDAQVGEVRVFEGTEELPGDPDMMRYVDRSGHLQSKML	354
UPN63108.1	LINRRTPRVDGQPMYGMDAQVEEVRVFEGTEELPGDPDMMRYVDRSGHMGSKML	354
QBQ04202.1	LINRRTPRVDGQPMYGMDAQIEEVRVFEGTEQLPGDPDLMRYVDKYGHWQTKML	354
QBM11923.1	LINRRTPRVDGQPMYGMDAQVKKVRVFEGTEELPGDPELMRYVDKYGQLQTKML	354
AAK70306.1	LINRRTPGVDGQPMYGMDAQVEEVRVFEGTEELPGDPDMMRYVDRYGQLQTKML	354
AAC59337.1	LINRRTPRVDGQPMYGMDAQVEEVRVFEGTEELPEDPDMMRYVDRYGQLQTKML	354
AAL37677.1	LINRRTPRVDGQPMYGMDAQVEEVRVFEGTEELPGDPDMMRYVDRYGQLQTKML	354
AAK70276.1	LINRRTPRVDGQPMYGMDAQVEEVRVFEGTEELPGDPDMMRYVDRYGQLQTKML	354
BAC22792.1	LINRRTPRVDGQPMYGMDAQVEEVRVFEGTEELPGDPDMMRYVDRYGQLQTKML	354
BAC22798.1	LINRRTPRVDGQPMYGMDAQVEEVRVFEGTEELPGDPDMMRYVDRYGQLQTKML	354
BAC22804.1	LINRRTPRVDGQPMYGMDAQVEEVRVFEGTEELPGDPDMMRYVDRYGQLQTKML	354
BAC22810.1	LINRRTPRVDGQPMYGMDAQVEEVRVFEGTEELPGDPDMMRYVDRYGQLQTKML	354
BAC22858.1	LINRRTPRVDGQPMYGMDAQVEEVRVFEGTEELPGDPDMMRYVDRYGQLQTKML	354
BAC15701.1	LINRRTPRVDGQPMYGMDAQVEEVRVFEGTEELPGDPDMMRYVDRYGQLQTKML	354
BAB68863.1	LINRRTPRVDGQPMYGMDAQVEEVRVFEGTEELPGDPDMMRYVDRYGQLQTKML	354
BAC66424.1	LINRRTPRVDGQPMYGMDAQVEEVRVFEGTEELPGDPDMMRYVDRYGQLQTKML	354
BAD21235.2	LINRRTPRVDGQPMYGMDAQVEEVRVFEGTEELPGDPDMMRYVDRYGQLQTKML	354
QTJ15210.1	LINRRTPRVDGQPMYGMDAQVEEVRVFEGTEELPGDPDMMRYVDRYGQLQTKML	354
ASV51748.1	LINRRTPRVDGQPMYGMDAQVEEVRVFEGTEELPGDPDMMRYVDRYGQLQTKML	354
ASV51754.1	LINRRTPRVDGQPMYGMDAQVEEVRVFEGTEELPGDPDMMRYVDRYGQLQTKML	354
ASV51778.1	LINRRTPRVDGQPMYGMDAQVEEVRVFEGTEELPGDPDMMRYVDRYGQLQTKML	354
ASV51814.1	LINRRTPRVDGQPMYGMDAQVEEVRVFEGTEELPGDPDMMRYVDRYGQLQTKML	354
CCW72488.1	LINRRTPRVDGQPMYGMDAQVEEVRVFEGTEELPGDPDMMRYVDRYGQLQTKML	354
AEC15219.1	LINRRTPRVDGQPMYGMDAQVEEVRVFEGTEELPGDPDMMRYVDRYGQLQTKML	354
BAD21241.1	LINRRTPRVDGQPMYGMDAQVEEVRVFEGTEELPGDPDMMRYVDRYGQLQTKML	354
BAD21253.1	LINRRTPRVDGQPMYGMDAQVEEVRVFEGTEELPGDPDMMRYVDRYGQLQTKML	354
BAB93086.1	LINRRTPRVDGQPMYGMDAQVEEVRVFEGTEELPGDPDMMRYVDRYGQLQTKML	354
BAB68953.1	LINRRTPRVDGQPMYGMDAQVEEVRVFEGTEELPGDPDMMRYVDRYGQLQTKML	354
BAD98966.1	LINRRTPRVDGQPMYGMDAQVEEVRVFEGTEELPGDPDMMRYVDRYGQLQTKML	354
BAD98972.1	LINRRTPRVDGQPMYGMDAQVEEVRVFEGTEELPGDPDMMRYVDRYGQLQTKML	354
BAD91887.1	LINRRTPRVDGQPMYGMDAQVEEVRVFEGTEELPGDPDMMRYVDRYGQLQTKML	354

BAD27118.1	LINRRTPRVDGQPMYGMDAQVEEVRVFEGTEELPGDPDMMRYVDRYGQLQTKML	354
BAD06227.1	LINRRTPRVDGQPMYGMDAQVEEVRVFEGTEELPGDPDMMRYVDRYGQLQTKML	354
BAC66412.1	LINRRTPRVDGQPMYGMDAQVEEVRVFEGTEELPGDPDMMRYVDRYGQLQTKML	354
BAC66430.1	LINRRTPRVDGQPMYGMDAQVEEVRVFEGTEELPGDPDMMRYVDRYGQLQTKML	354
BAC22816.1	LINRRTPRVDGQPMYGMDAQVEEVRVFEGTEELPGDPDMMRYVDRYGQLQTKML	354
BAB93026.1	LINRRTPRVDGQPMYGMDAQVEEVRVFEGTEELPGDPDMMRYVDRYGQLQTKML	354
BAB93032.1	LINRRTPRVDGQPMYGMDAQVEEVRVFEGTEELPGDPDMMRYVDRYGQLQTKML	354
BAB93074.1	LINRRTPRVDGQPMYGMDAQVEEVRVFEGTEELPGDPDMMRYVDRYGQLQTKML	354
BAB93080.1	LINRRTPRVDGQPMYGMDAQVEEVRVFEGTEELPGDPDMMRYVDRYGQLQTKML	354
BAB68917.1	LINRRTPRVDGQPMYGMDAQVEEVRVFEGTEELPGDPDMMRYVDRYGQLQTKML	354
BAB68923.1	LINRRTPRVDGQPMYGMDAQVEEVRVFEGTEELPGDPDMMRYVDRYGQLQTKML	354
BAB68929.1	LINRRTPRVDGQPMYGMDAQVEEVRVFEGTEELPGDPDMMRYVDRYGQLQTKML	354
BAB68935.1	LINRRTPRVDGQPMYGMDAQVEEVRVFEGTEELPGDPDMMRYVDRYGQLQTKML	354
BAB68941.1	LINRRTPRVDGQPMYGMDAQVEEVRVFEGTEELPGDPDMMRYVDRYGQLQTKML	354
ALF06214.1	LINRRTPRVDGQPMYGMDAQVEEVRVFEGTEELPGDPDMMRYVDRYGQLQT---	351
AAL37707.1	LINRRTPRVDGQPMYGMDAQVEEVRVFEGTEELPGDPDMMRYVDRYGQLQTKML	354
BBG22688.1	LINRRTPRVDGQPMYGMDAQVEEVRVFEGTEELPGDPDMMRYVDRYGQLQTKML	354
BAB11734.1	LINRRTPRVDGQPMYGMDAQVEEVRVFEGTEELPGDPDMMRYVDRYGQLQTKML	354
BAA05638.1	LINRRTPRVDGQPMYGMDAQVEEVRVFEGTEELPGDPDMMRYVDRYGQLQTKML	354
BAV69361.1	LINRRTPRVDGQPMYGMDAQVEEVRVFEGTEELPGDPDMMRYVDRYGQLQTKML	354
BAV69349.1	LINRRTPRVDGQPMYGMDAQVEEVRVFEGTEELPGDPDMMRYVDRYGQLQTKML	354
BAV69367.1	LINRRTPRVDGQPMYGMDAQVEEVRVFEGTEELPGDPDMMRYVDRYGQLQTKML	354
BAE00135.1	LINRRTPRVDGQPMYGMDAQVEEVRVFEGTEELPGDPDMMRYVDRYGQLQTKML	354
BAE00147.1	LINRRTPRVDGQPMYGMDAQVEEVRVFEGTEELPGDPDMMRYVDRYGQLQTKML	354
BAD11896.1	LINRRTPRVDGQPMYGMDAQVEEVRVFEGTEELPGDPDMMRYVDRYGQLQTKML	354
BAD06126.1	LINRRTPRVDGQPMYGMDAQVEEVRVFEGTEELPGDPDMMRYVDRYGQLQTKML	354
BAC81904.1	LINRRTPRVDGQPMYGMDAQVEEVRVFEGTEELPGDPDMMRYVDRYGQLQTKML	354
BAC81910.1	LINRRTPRVDGQPMYGMDAQVEEVRVFEGTEELPGDPDMMRYVDRYGQLQTKML	354
BAC81916.1	LINRRTPRVDGQPMYGMDAQVEEVRVFEGTEELPGDPDMMRYVDRYGQLQTKML	354
BAC81922.1	LINRRTPRVDGQPMYGMDAQVEEVRVFEGTEELPGDPDMMRYVDRYGQLQTKML	354
BAC81946.1	LINRRTPRVDGQPMYGMDAQVEEVRVFEGTEELPGDPDMMRYVDRYGQLQTKML	354
BAC81952.1	LINRRTPRVDGQPMYGMDAQVEEVRVFEGTEELPGDPDMMRYVDRYGQLQTKML	354
BAC81958.1	LINRRTPRVDGQPMYGMDAQVEEVRVFEGTEELPGDPDMMRYVDRYGQLQTKML	354
AAM89297.1	LINRRTPRVDGQPMYGMDAQVEEVRVFEGTEELPGDPDMMRYVDRYGQLQTKML	354
AAM89303.1	LINRRTPRVDGQPMYGMDAQVEEVRVFEGTEELPGDPDMMRYVDRYGQLQTKML	354
AAM89309.1	LINRRTPRVDGQPMYGMDAQVEEVRVFEGTEELPGDPDMMRYVDRYGQLQTKML	354
BAC66388.1	LINRRTPRVDGQPMYGMDAQVEEVRVFEGTEELPGDPDMMRYVDRYGQLQTKML	354
BAC66400.1	LINRRTPRVDGQPMYGMDAQVEEVRVFEGTEELPGDPDMMRYVDRYGQLQTKML	354
BAC66406.1	LINRRTPRVDGQPMYGMDAQVEEVRVFEGTEELPGDPDMMRYVDRYGQLQTKML	354
BAC22756.1	LINRRTPRVDGQPMYGMDAQVEEVRVFEGTEELPGDPDMMRYVDRYGQLQTKML	354
BAC22822.1	LINRRTPRVDGQPMYGMDAQVEEVRVFEGTEELPGDPDMMRYVDRYGQLQTKML	354
BAC22894.1	LINRRTPRVDGQPMYGMDAQVEEVRVFEGTEELPGDPDMMRYVDRYGQLQTKML	354
BAC22900.1	LINRRTPRVDGQPMYGMDAQVEEVRVFEGTEELPGDPDMMRYVDRYGQLQTKML	354
BAC15635.1	LINRRTPRVDGQPMYGMDAQVEEVRVFEGTEELPGDPDMMRYVDRYGQLQTKML	354
BAC15653.1	LINRRTPRVDGQPMYGMDAQVEEVRVFEGTEELPGDPDMMRYVDRYGQLQTKML	354
BAC15665.1	LINRRTPRVDGQPMYGMDAQVEEVRVFEGTEELPGDPDMMRYVDRYGQLQTKML	354
BAC03065.1	LINRRTPRVDGQPMYGMDAQVEEVRVFEGTEELPGDPDMMRYVDRYGQLQTKML	354
AAL37689.1	LINRRTPRVDGQPMYGMDAQVEEVRVFEGTEELPGDPDMMRYVDRYGQLQTKML	354
AAL37701.1	LINRRTPRVDGQPMYGMDAQVEEVRVFEGTEELPGDPDMMRYVDRYGQLQTKML	354
BAB68803.1	LINRRTPRVDGQPMYGMDAQVEEVRVFEGTEELPGDPDMMRYVDRYGQLQTKML	354
BAB68965.1	LINRRTPRVDGQPMYGMDAQVEEVRVFEGTEELPGDPDMMRYVDRYGQLQTKML	354
BAB68971.1	LINRRTPRVDGQPMYGMDAQVEEVRVFEGTEELPGDPDMMRYVDRYGQLQTKML	354
AAK97964.1	LINRRTPRVDGQPMYGMDAQVEEVRVFEGTEELPGDPDMMRYVDRYGQLQTKML	354
AAK97970.1	LINRRTPRVDGQPMYGMDAQVEEVRVFEGTEELPGDPDMMRYVDRYGQLQTKML	354
AAC40841.1	LINRRTPRVDGQPMYGMDAQVEEVRVFEGTEELPGDPDMMRYVDRYGQLQTKML	354
BAA05636.1	LINRRTPRVDGQPMYGMDAQVEEVRVFEGTEELPGDPDMMRYVDRYGQLQTKML	354
BAA01970.1	LINRRTPRVDGQPMYGMDAQVEEVRVFEGTEELPGDPDMMRYVDRYGQLQTKML	354
BAD06144.1	LINRRTPRVNGQPMYGMDAQVEEVRVFEGTEELPGDPDMMRYVDRYGQLQTKML	354
BAC81852.1	LINRRTPRVDGQPMYGMDAQVEEVRVFEGTEELPGDPDMMRYVDRYGQLQTKML	354
BAC81858.1	LINRRTPRVDGQPMYGMDAQVEEVRVFEGTEELPGDPDMMRYVDRYGQLQTKML	354
BAC22864.1	LINRRTPRVDGQPMYGMDAQVEEVRVFEGTEELPGDPDMMRYVDRYGQLQTKML	354
AAM89315.1	LINRRTPRVDGQPMYGMDAQVEEVRVFEGTEELPGDPDMMRYVDRYGQLQTKML	354
BAC66382.1	LINRRTPRVDGQPMYGMDAQVEEVRVFEGTEELPGDPDMMRYVDRYGQLQTKML	354
BAC66394.1	LINRRTPRVDGQPMYGMDAQVEEVRVFEGTEELPGDPDMMRYVDRYGQLQTKML	354

BAC66418.1	LINRRTPRVDGQPMYGMDAQVEEVRVFEGTEELPGDPDMMRYVDRYGQLQTKML	354
BAC22828.1	LINRRTPRVDGQPMYGMDAQVEEVRVFEGTEELPGDPDMMRYVDRYGQLQTKML	354
BAC22846.1	LINRRTPRVDGQPMYGMDAQVEEVRVFEGTEELPGDPDMMRYVDRYGQLQTKML	354
AAM69590.1	LINRRTPRVDGQPMYGMDAQVEEVRVFEGTEELPGDPDMMRYVDRYGQLQTKML	354
BAB93014.1	LINRRTPRVDGQPMYGMDAQVEEVRVFEGTEELPGDPDMMRYVDRYGQLQTKML	354
BAB93020.1	LINRRTPRVDGQPMYGMDAQVEEVRVFEGTEELPGDPDMMRYVDRYGQLQTKML	354
BAB93092.1	LINRRTPRVDGQPMYGMDAQVEEVRVFEGTEELPGDPDMMRYVDRYGQLQTKML	354
BAB93098.1	LINRRTPRVDGQPMYGMDAQVEEVRVFEGTEELPGDPDMMRYVDRYGQLQTKML	354
AAL37665.1	LINRRTPRVDGQPMYGMDAQVEEVRVFEGTEELPGDPDMMRYVDRYGQLQTKML	354
BAB68773.1	LINRRTPRVDGQPMYGMDAQVEEVRVFEGTEELPGDPDMMRYVDRYGQLQTKML	354
BAB68779.1	LINRRTPRVDGQPMYGMDAQVEEVRVFEGTEELPGDPDMMRYVDRYGQLQTKML	354
AAK28460.1	LINRRTPRVDGQPMYGMDAQVEEVRVFEGTEELPGDPDMMRYVDRYGQLQTKML	354
AAK28466.1	LINRRTPRVDGQPMYGMDAQVEEVRVFEGTEELPGDPDMMRYVDRYGQLQTKML	354
BAB11716.1	LINRRTPRVDGQPMYGMDAQVEEVRVFEGTEELPGDPDMMRYVDRYGQLQTKML	354
BAB11722.1	LINRRTPRVDGQPMYGMDAQVEEVRVFEGTEELPGDPDMMRYVDRYGQLQTKML	354
AAK97976.1	LINRRTPRVDGQPMYGMDAQVEEVRVFEGTEELPGDPDMMRYVDRYGQLQTKML	354
BAC15641.1	LINRRTPRVDGQPMYGMDAQVEEVRVFEGTEELPGDPDMMRYVDRYGQLQTKML	354
BAC15647.1	LINRRTPRVDGQPMYGMDAQVEEVRVFEGTEELPGDPDMMRYVDRYGQLQTKML	354
AAL37647.1	LINRRTPRVDGQPMYGMDAQVEEVRVFEGTEELPGDPDMMRYVDRYGQLQTKML	354
BAB68797.1	LINRRTPRVDGQPMYGMDAQVEEVRVFEGTEELPGDPDMMRYVDRYGQLQTKML	354
AAG37198.1	LINRRTPRVDGQPMYGMDAHVEEVRVFEGTEELPGDPDMMRYVDRYGQLQTKML	354
ALF06241.1	LINRRTPRVDGQPMYGMDAQVEEVRVFEGTEELPGDPDMMRYVDRYGQLQTK--	352
AFE56117.1	LINRRTPRVDGQPMYGMDAQVEEVRVFEGTEELPGDPDMMRYVDRYGQLQTKML	354
BAF95600.1	LINRRTPRVDGQPMYGMDAQVEEVRVFEGTEELPGDPDMMRYVDRYGQLQTKML	354
BAD11836.1	LINRRTPRVDGQPMYGMDAQVEEVRVFEGTEELPGDPDMMRYVDRYGQLQTKML	354
BAD11842.1	LINRRTPRVDGQPMYGMDAQVEEVRVFEGTEELPGDPDMMRYVDRYGQLQTKML	354
BAD11848.1	LINRRTPRVDGQPMYGMDAQVEEVRVFEGTEELPGDPDMMRYVDRYGQLQTKML	354
AAM89327.1	LINRRTPRVDGQPMYGMDAQVEEVRVFEGTEELPGDPDMMRYVDRYGQLQTKML	354
AAM89339.1	LINRRTPRVDGQPMYGMDAQVEEVRVFEGTEELPGDPDMMRYVDRYGQLQTKML	354
BAC15707.1	LINRRTPRVDGQPMYGMDAQVEEVRVFEGTEELPGDPDMMRYVDRYGQLQTKML	354
BAB68833.1	LINRRTPRVDGQPMYGMDAQVEEVRVFEGTEELPGDPDMMRYVDRYGQLQTKML	354
BAB68839.1	LINRRTPRVDGQPMYGMDAQVEEVRVFEGTEELPGDPDMMRYVDRYGQLQTKML	354
AYM55604.1	LINRRTPRVDGQPMYGMDAQVEEVRVFEGTEELPGDPDMMRYVDRYGQLQTKML	354
BAV69355.1	LINRRTPRVDGQPMYGMDAQVEEVRVFEGTEELPGDPDMMRYVDRYGQLQTKML	354
ALF06220.1	LINRRTPRVDGQPMYGMDAQVEEVRVFEGTEELPGDPDMMRYVDRYGQLQTKM-	353
ALF06247.1	LINRRTPRVDGQPMYGMDAQVEEVRVFEGTEELPGDPDMMRYVDRYGQLQTK--	352
ALF06250.1	LINRRTPRVDGQPMYGMDAQVEEVRVFEGTEELPGDPDMMRYVDRYGQLQTKML	354
ALF06253.1	LINRRTPRVDGQPMYGMDAQVEEVRVFEGTEELPGDPDMMRYVDRYGQLQTK--	352
ALF06259.1	LINRRTPRVDGQPMYGMDAQVEEVRVFEGTEELPGDPDMMRYVDRYGQLQTKM-	353
ALF06265.1	LINRRTPRVDGQPMYGMDAQVEEVRVFEGTEELPGDPDMMRYVDRYGQLQTKM-	353
CCW72502.1	LINRRTPRVDGQPMYGMDAQVEEVRVFEGTEELPGDPDMMRYVDRYGQLQTKML	354
BAF40757.1	LINRRTPRVDGQPMYGMDAQVEEVRVFEGTEELPGDPDMMRYVDRYGQLQTKML	354
BAF40763.1	LINRRTPRVDGQPMYGMDAQVEEVRVFEGTEELPGDPDMMRYVDRYGQLQTKML	354
BAF40769.1	LINRRTPRVDGQPMYGMDAQVEEVRVFEGTEELPGDPDMMRYVDRYGQLQTKML	354
BAF40775.1	LINRRTPRVDGQPMYGMDAQVEEVRVFEGTEELPGDPDMMRYVDRYGQLQTKML	354
BAE94732.1	LINRRTPRVDGQPMYGMDAQVEEVRVFEGTEELPGDPDMMRYVDRYGQLQTKML	354
BAE94738.1	LINRRTPRVDGQPMYGMDAQVEEVRVFEGTEELPGDPDMMRYVDRYGQLQTKML	354
BAE00129.1	LINRRTPRVDGQPMYGMDAQVEEVRVFEGTEELPGDPDMMRYVDRYGQLQTKML	354
BAE00141.1	LINRRTPRVDGQPMYGMDAQVEEVRVFEGTEELPGDPDMMRYVDRYGQLQTKML	354
BAD11824.1	LINRRTPRVDGQPMYGMDAQVEEVRVFEGTEELPGDPDMMRYVDRYGQLQTKML	354
BAD11830.1	LINRRTPRVDGQPMYGMDAQVEEVRVFEGTEELPGDPDMMRYVDRYGQLQTKML	354
BAD11854.1	LINRRTPRVDGQPMYGMDAQVEEVRVFEGTEELPGDPDMMRYVDRYGQLQTKML	354
BAD11860.1	LINRRTPRVDGQPMYGMDAQVEEVRVFEGTEELPGDPDMMRYVDRYGQLQTKML	354
BAD11866.1	LINRRTPRVDGQPMYGMDAQVEEVRVFEGTEELPGDPDMMRYVDRYGQLQTKML	354
BAD11872.1	LINRRTPRVDGQPMYGMDAQVEEVRVFEGTEELPGDPDMMRYVDRYGQLQTKML	354
BAD11878.1	LINRRTPRVDGQPMYGMDAQVEEVRVFEGTEELPGDPDMMRYVDRYGQLQTKML	354
BAD11884.1	LINRRTPRVDGQPMYGMDAQVEEVRVFEGTEELPGDPDMMRYVDRYGQLQTKML	354
BAD11890.1	LINRRTPRVDGQPMYGMDAQVEEVRVFEGTEELPGDPDMMRYVDRYGQLQTKML	354
BAD11902.1	LINRRTPRVDGQPMYGMDAQVEEVRVFEGTEELPGDPDMMRYVDRYGQLQTKML	354
BAD06233.1	LINRRTPRVDGQPMYGMDAQVEEVRVFEGTEELPGDPDMMRYVDRYGQLQTKML	354
BAD06066.1	LINRRTPRVDGQPMYGMDAQVEEVRVFEGTEELPGDPDMMRYVDRYGQLQTKML	354
BAD06078.1	LINRRTPRVDGQPMYGMDAQVEEVRVFEGTEELPGDPDMMRYVDRYGQLQTKML	354
BAD06114.1	LINRRTPRVDGQPMYGMDAQVEEVRVFEGTEELPGDPDMMRYVDRYGQLQTKML	354
BAD06132.1	LINRRTPRVDGQPMYGMDAQVEEVRVFEGTEELPGDPDMMRYVDRYGQLQTKML	354

BAD06138.1	LINRRTPRVDGQPMYGMDAQVEEVRVFEGTEELPGDPDMMRYVDRYGQLQTKML	354
BAD06162.1	LINRRTPRVDGQPMYGMDAQVEEVRVFEGTEELPGDPDMMRYVDRYGQLQTKML	354
BAD06168.1	LINRRTPRVDGQPMYGMDAQVEEVRVFEGTEELPGDPDMMRYVDRYGQLQTKML	354
BAC82365.1	LINRRTPRVDGQPMYGMDAQVEEVRVFEGTEELPGDPDMMRYVDRYGQLQTKML	354
BAC81846.1	LINRRTPRVDGQPMYGMDAQVEEVRVFEGTEELPGDPDMMRYVDRYGQLQTKML	354
BAC81864.1	LINRRTPRVDGQPMYGMDAQVEEVRVFEGTEELPGDPDMMRYVDRYGQLQTKML	354
BAC81870.1	LINRRTPRVDGQPMYGMDAQVEEVRVFEGTEELPGDPDMMRYVDRYGQLQTKML	354
BAC81874.1	LINRRTPRVDGQPMYGMDAQVEEVRVFEGTEELPGDPDMMRYVDRYGQLQTKML	354
BAC81880.1	LINRRTPRVDGQPMYGMDAQVEEVRVFEGTEELPGDPDMMRYVDRYGQLQTKML	354
BAC81886.1	LINRRTPRVDGQPMYGMDAQVEEVRVFEGTEELPGDPDMMRYVDRYGQLQTKML	354
BAC81892.1	LINRRTPRVDGQPMYGMDAQVEEVRVFEGTEELPGDPDMMRYVDRYGQLQTKML	354
BAC81898.1	LINRRTPRVDGQPMYGMDAQVEEVRVFEGTEELPGDPDMMRYVDRYGQLQTKML	354
BAC81928.1	LINRRTPRVDGQPMYGMDAQVEEVRVFEGTEELPGDPDMMRYVDRYGQLQTKML	354
BAC81934.1	LINRRTPRVDGQPMYGMDAQVEEVRVFEGTEELPGDPDMMRYVDRYGQLQTKML	354
BAC81940.1	LINRRTPRVDGQPMYGMDAQVEEVRVFEGTEELPGDPDMMRYVDRYGQLQTKML	354
BAC81964.1	LINRRTPRVDGQPMYGMDAQVEEVRVFEGTEELPGDPDMMRYVDRYGQLQTKML	354
AAM89321.1	LINRRTPRVDGQPMYGMDAQVEEVRVFEGTEELPGDPDMMRYVDRYGQLQTKML	354
AAM89333.1	LINRRTPRVDGQPMYGMDAQVEEVRVFEGTEELPGDPDMMRYVDRYGQLQTKML	354
AAM89345.1	LINRRTPRVDGQPMYGMDAQVEEVRVFEGTEELPGDPDMMRYVDRYGQLQTKML	354
BAC66376.1	LINRRTPRVDGQPMYGMDAQVEEVRVFEGTEELPGDPDMMRYVDRYGQLQTKML	354
BAC22834.1	LINRRTPRVDGQPMYGMDAQVEEVRVFEGTEELPGDPDMMRYVDRYGQLQTKML	354
BAC22840.1	LINRRTPRVDGQPMYGMDAQVEEVRVFEGTEELPGDPDMMRYVDRYGQLQTKML	354
BAC22852.1	LINRRTPRVDGQPMYGMDAQVEEVRVFEGTEELPGDPDMMRYVDRYGQLQTKML	354
BAC22870.1	LINRRTPRVDGQPMYGMDAQVEEVRVFEGTEELPGDPDMMRYVDRYGQLQTKML	354
BAC22876.1	LINRRTPRVDGQPMYGMDAQVEEVRVFEGTEELPGDPDMMRYVDRYGQLQTKML	354
BAC22882.1	LINRRTPRVDGQPMYGMDAQVEEVRVFEGTEELPGDPDMMRYVDRYGQLQTKML	354
BAC22888.1	LINRRTPRVDGQPMYGMDAQVEEVRVFEGTEELPGDPDMMRYVDRYGQLQTKML	354
BAC15629.1	LINRRTPRVDGQPMYGMDAQVEEVRVFEGTEELPGDPDMMRYVDRYGQLQTKML	354
BAC15659.1	LINRRTPRVDGQPMYGMDAQVEEVRVFEGTEELPGDPDMMRYVDRYGQLQTKML	354
BAC15671.1	LINRRTPRVDGQPMYGMDAQVEEVRVFEGTEELPGDPDMMRYVDRYGQLQTKML	354
BAC15677.1	LINRRTPRVDGQPMYGMDAQVEEVRVFEGTEELPGDPDMMRYVDRYGQLQTKML	354
BAC15689.1	LINRRTPRVDGQPMYGMDAQVEEVRVFEGTEELPGDPDMMRYVDRYGQLQTKML	354
BAC15713.1	LINRRTPRVDGQPMYGMDAQVEEVRVFEGTEELPGDPDMMRYVDRYGQLQTKML	354
BAC15731.1	LINRRTPRVDGQPMYGMDAQVEEVRVFEGTEELPGDPDMMRYVDRYGQLQTKML	354
BAC15737.1	LINRRTPRVDGQPMYGMDAQVEEVRVFEGTEELPGDPDMMRYVDRYGQLQTKML	354
BAC15743.1	LINRRTPRVDGQPMYGMDAQVEEVRVFEGTEELPGDPDMMRYVDRYGQLQTKML	354
AAL37641.1	LINRRTPRVDGQPMYGMDAQVEEVRVFEGTEELPGDPDMMRYVDRYGQLQTKML	354
AAL37653.1	LINRRTPRVDGQPMYGMDAQVEEVRVFEGTEELPGDPDMMRYVDRYGQLQTKML	354
AAL37659.1	LINRRTPRVDGQPMYGMDAQVEEVRVFEGTEELPGDPDMMRYVDRYGQLQTKML	354
AAL37671.1	LINRRTPRVDGQPMYGMDAQVEEVRVFEGTEELPGDPDMMRYVDRYGQLQTKML	354
AAL37683.1	LINRRTPRVDGQPMYGMDAQVEEVRVFEGTEELPGDPDMMRYVDRYGQLQTKML	354
BAB68791.1	LINRRTPRVDGQPMYGMDAQVEEVRVFEGTEELPGDPDMMRYVDRYGQLQTKML	354
BAB68821.1	LINRRTPRVDGQPMYGMDAQVEEVRVFEGTEELPGDPDMMRYVDRYGQLQTKML	354
BAB68845.1	LINRRTPRVDGQPMYGMDAQVEEVRVFEGTEELPGDPDMMRYVDRYGQLQTKML	354
BAB68851.1	LINRRTPRVDGQPMYGMDAQVEEVRVFEGTEELPGDPDMMRYVDRYGQLQTKML	354
BAB68857.1	LINRRTPRVDGQPMYGMDAQVEEVRVFEGTEELPGDPDMMRYVDRYGQLQTKML	354
BAB68869.1	LINRRTPRVDGQPMYGMDAQVEEVRVFEGTEELPGDPDMMRYVDRYGQLQTKML	354
BAB68875.1	LINRRTPRVDGQPMYGMDAQVEEVRVFEGTEELPGDPDMMRYVDRYGQLQTKML	354
AAK97910.1	LINRRTPRVDGQPMYGMDAQVEEVRVFEGTEELPGDPDMMRYVDRYGQLQTKML	354
AAK97916.1	LINRRTPRVDGQPMYGMDAQVEEVRVFEGTEELPGDPDMMRYVDRYGQLQTKML	354
AAK97922.1	LINRRTPRVDGQPMYGMDAQVEEVRVFEGTEELPGDPDMMRYVDRYGQLQTKML	354
AAK97928.1	LINRRTPRVDGQPMYGMDAQVEEVRVFEGTEELPGDPDMMRYVDRYGQLQTKML	354
AAK97934.1	LINRRTPRVDGQPMYGMDAQVEEVRVFEGTEELPGDPDMMRYVDRYGQLQTKML	354
AAK97940.1	LINRRTPRVDGQPMYGMDAQVEEVRVFEGTEELPGDPDMMRYVDRYGQLQTKML	354
AAK97946.1	LINRRTPRVDGQPMYGMDAQVEEVRVFEGTEELPGDPDMMRYVDRYGQLQTKML	354
AAK97952.1	LINRRTPRVDGQPMYGMDAQVEEVRVFEGTEELPGDPDMMRYVDRYGQLQTKML	354
AAK97958.1	LINRRTPRVDGQPMYGMDAQVEEVRVFEGTEELPGDPDMMRYVDRYGQLQTKML	354
AAK97982.1	LINRRTPRVDGQPMYGMDAQVEEVRVFEGTEELPGDPDMMRYVDRYGQLQTKML	354
AAK97988.1	LINRRTPRVDGQPMYGMDAQVEEVRVFEGTEELPGDPDMMRYVDRYGQLQTKML	354
AAK97994.1	LINRRTPRVDGQPMYGMDAQVEEVRVFEGTEELPGDPDMMRYVDRYGQLQTKML	354
BAB11698.1	LINRRTPRVDGQPMYGMDAQVEEVRVFEGTEELPGDPDMMRYVDRYGQLQTKML	354
BAB11704.1	LINRRTPRVDGQPMYGMDAQVEEVRVFEGTEELPGDPDMMRYVDRYGQLQTKML	354
AAC59478.1	LINRRTPRVDGQPMYGMDAQVEEVRVFEGTEELPGDPDMMRYVDRYGQLQTKML	354
AAC59484.1	LINRRTPRVDGQPMYGMDAQVEEVRVFEGTEELPGDPDMMRYVDRYGQLQTKML	354

AAC59490.1	LINRRTPRVDGQPMYGMDAQVEEVRVFEGTEELPGDPDMMRYVDRYGQLQTKML	354
BAA01963.1	LINRRTPRVDGQPMYGMDAQVEEVRVFEGTEELPGDPDMMRYVDRYGQLQTKML	354
BAA01964.1	LINRRTPRVDGQPMYGMDAQVEEVRVFEGTEELPGDPDMMRYVDRYGQLQTKML	354
BAB93068.1	LINRRTPRVDGQPMYGMDAQVEEVRVFEGTEELPGDPDMMRYVDRYGQLQTKML	354
ASV51856.1	LINRRTPRVDGQPMYGMDAQVEEVRVFEGTEELPGDPDMMRYVDRYGQLQTKML	354
ANH21097.1	LINRRTPRVDGQPMYGMDAQVEEVRVFEGTEELPGDPDMMRYVDRYGQLQTKML	354
AEC15167.1	LINRRTPRVDGQPMYGMDAQVEEVRVFEGTEELPGDPDMMRYVDRYGQLQTKML	354
AEC15168.1	LINRRTPRVDGQPMYGMDAQVEEVRVFEGTEELPGDPDMMRYVDRYGQLQTKML	354
AEC15169.1	LINRRTPRVDGQPMYGMDAQVEEVRVFEGTEELPGDPDMMRYVDRYGQLQTKML	354
QTJ15168.1	LINRRTPRVDGQPMYGMDAQVEEVRVFEGTEELPGDPDMMRYVDRYGQLQTKML	354
AEC15173.1	LINRRTPRVDGQPMYGMDAQVEEVRVFEGTEELPGDPDMMRYVDRYGQLQTKML	354
AEC15174.1	LINRRTPRVDGQPMYGMDAQVEEVRVFEGTEELPGDPDMMRYVDRYGQLQTKML	354
AEC15175.1	LINRRTPRVDGQPMYGMDAQVEEVRVFEGTEELPGDPDMMRYVDRYGQLQTKML	354
AEC15176.1	LINRRTPRVDGQPMYGMDAQVEEVRVFEGTEELPGDPDMMRYVDRYGQLQTKML	354
AEC15177.1	LINRRTPRVDGQPMYGMDAQVEEVRVFEGTEELPGDPDMMRYVDRYGQLQTKML	354
AEC15178.1	LINRRTPRVDGQPMYGMDAQVEEVRVFEGTEELPGDPDMMRYVDRYGQLQTKML	354
AEC15179.1	LINRRTPRVDGQPMYGMDAQVEEVRVFEGTEELPGDPDMMRYVDRYGQLQTKML	354
AEC15180.1	LINRRTPRVDGQPMYGMDAQVEEVRVFEGTEELPGDPDMMRYVDRYGQLQTKML	354
AEC15181.1	LINRRTPRVDGQPMYGMDAQVEEVRVFEGTEELPGDPDMMRYVDRYGQLQTKML	354
AEC15182.1	LINRRTPRVDGQPMYGMDAQVEEVRVFEGTEELPGDPDMMRYVDRYGQLQTKML	354
AEC15183.1	LINRRTPRVDGQPMYGMDAQVEEVRVFEGTEELPGDPDMMRYVDRYGQLQTKML	354
AEC15184.1	LINRRTPRVDGQPMYGMDAQVEEVRVFEGTEELPGDPDMMRYVDRYGQLQTKML	354
AEC15185.1	LINRRTPRVDGQPMYGMDAQVEEVRVFEGTEELPGDPDMMRYVDRYGQLQTKML	354
AEC15187.1	LINRRTPRVDGQPMYGMDAQVEEVRVFEGTEELPGDPDMMRYVDRYGQLQTKML	354
AEC15188.1	LINRRTPRVDGQPMYGMDAQVEEVRVFEGTEELPGDPDMMRYVDRYGQLQTKML	354
AEC15189.1	LINRRTPRVDGQPMYGMDAQVEEVRVFEGTEELPGDPDMMRYVDRYGQLQTKML	354
AEC15190.1	LINRRTPRVDGQPMYGMDAQVEEVRVFEGTEELPGDPDMMRYVDRYGQLQTKML	354
AEC15191.1	LINRRTPRVDGQPMYGMDAQVEEVRVFEGTEELPGDPDMMRYVDRYGQLQTKML	354
AEC15193.1	LINRRTPRVDGQPMYGMDAQVEEVRVFEGTEELPGDPDMMRYVDRYGQLQTKML	354
AEC14871.1	LINRRTPRVDGQPMYGMDAQVEEVRVFEGTEELPGDPDMMRYVDRYGQLQTKML	354
AEC15222.1	LINRRTPRVDGQPMYGMDAQVEEVRVFEGTEELPGDPDMMRYVDRYGQLQTKML	354
AEC15130.1	LINRRTPRVDGQPMYGMDAQVEEVRVFEGTEELPGDPDMMRYVDRYGQLQTKML	354
AEC15144.1	LINRRTPRVDGQPMYGMDAQVEEVRVFEGTEELPGDPDMMRYVDRYGQLQTKML	354
AEC15154.1	LINRRTPRVDGQPMYGMDAQVEEVRVFEGTEELPGDPDMMRYVDRYGQLQTKML	354
AEC15157.1	LINRRTPRVDGQPMYGMDAQVEEVRVFEDTEELPGDPDMMRYVDRYGQLQTKML	354
AEC15160.1	LINRRTPRIDGQPMYGMDAQVEEVRVFEGTEELPGDPDMMRYVDRYGQLQTKML	354
AEC15231.1	LINRRTPRVDGQPMYGMDAQVEEVRVFEGTEELPGDPDMMRYVDRYGQLQTKML	354
ALF06191.1	LINRRTPRVDGQPMYGMDAQVEEVRVFEGTEELPGDPDMMRYVDRYGQLQTKML	354
WLD48112.1	LINRRTPRVDGQPMYGMDAQVEEVRVFEGTEELPGDPDMMRYVDRYGQLQTKML	354
QWT77140.1	LINRRTPRVDGQPMYGMDAQVEEVRVFEGTEELPGDPDMMRYVDRYGQLQTKML	354
QTJ15174.1	LINRRTPRVDGQPMYGMDAQVEEVRVFEGTEELPGDPDMMRYVDRYGQLQTKML	354
QTJ15186.1	LINRRTPRVDGQPMYGMDAQVEEVRVFEGTEELPGDPDMMRYVDRYGQLQTKML	354
QTJ15192.1	LINRRTPRVDGQPMYGMDAQVEEVRVFEGTEELPGDPDMMRYVDRYGQLQTKML	354
QTJ15198.1	LINRRTPRVDGQPMYGMDAQVEEVRVFEGTEELPGDPDMMRYVDRYGQLQTKML	354
ASV51772.1	LINRRTPRVDGQPMYGMDAQVEEVRVFEGTEELPGDPDMMRYVDRYGQLQTKML	354
CCW72478.1	LINRRTPRVDGQPMYGMDAQVEEVRVFEGTEELPGDPDMMRYVDRYGQLQTKML	354
CCW72481.1	LINRRTPRVDGQPMYGMDAQVEEVRVFEGTEELPGDPDMMRYVDRYGQLQTKML	354
CCW72487.1	LINRRTPRVDGQPMYGMDAQVEEVRVFEGTEELPGDPDMMRYVDRYGQLQTKML	354
CCW72492.1	LINRRTPRVDGQPMYGMDAQVEEVRVFEGTEELPGDPDMMRYVDRYGQLQTKML	354
CCW72496.1	LINRRTPRVDGQPMYGMDAQVEEVRVFEGTEELPGDPDMMRYVDRYGQLQTKML	354
CCW72499.1	LINRRTPRVDGQPMYGMDAQVEEVRVFEGTEELPGDPDMMRYVDRYGQLQTKML	354
CCW72504.1	LINRRTPRVDGQPMYGMDAQVEEVRVFEGTEELPGDPDMMRYVDRYGQLQTKML	354
CCW72508.1	LINRRTPRVDGQPMYGMDAQVEEVRVFEGTEELPGDPDMMRYVDRYGQLQTKML	354
CCW72510.1	LINRRTPRVDGQPMYGMDAQVEEVRVFEGTEELPGDPDMMRYVDRYGQLQTKML	354
CCW72519.1	LINRRTPRVDGQPMYGMDAQVEEVRVFEGTEELPGDPDMMRYVDRYGQLQTKML	354
CCW72521.1	LINRRTPRVDGQPMYGMDAQVEEVRVFEGTEELPGDPDMMRYVDRYGQLQTKML	354
AEC14864.1	LINRRTPRVDGQPMYGMDAQVEEVRVFEGTEELPGDPDMMRYVDRYGQLQTKML	354
AEC14865.1	LINRRTPRVDGQPMYGMDAQVEEVRVFEGTEELPGDPDMMRYVDRYGQLQTKML	354
AEC14866.1	LINRRTPRVDGQPMYGMDAQVEEVRVFEGTEELPGDPDMMRYVDRYGQLQTKML	354
AEC14867.1	LINRRTPRVDGQPMYGMDAQVEEVRVFEGTEELPGDPDMMRYVDRYGQLQTKML	354
AEC14868.1	LINRRTPRVDGQPMYGMDAQVEEVRVFEGTEELPGDPDMMRYVDRYGQLQTKML	354
AEC14869.1	LINRRTPRVDGQPMYGMDAQVEEVRVFEGTEELPGDPDMMRYVDRYGQLQTKML	354
AEC14870.1	LINRRTPRVDGQPMYGMDAQVEEVRVFEGTEELPGDPDMMRYVDRYGQLQTKML	354
AEC14872.1	LINRRTPRVDGQPMYGMDAQVEEVRVFEGTEELPGDPDMMRYVDRYGQLQTKML	354

AEC14873.1	LINRRTPRVDGQPMYGMDAQVEEVRVFEGTEELPGDPDMMRYVDRYGQLQTKML	354
AEC14874.1	LINRRTPRVDGQPMYGMDAQVEEVRVFEGTEELPGDPDMMRYVDRYGQLQTKML	354
AEC14875.1	LINRRTPRVDGQPMYGMDAQVEEVRVFEGTEELPGDPDMMRYVDRYGQLQTKML	354
AEC14876.1	LINRRTPRVDGQPMYGMDAQVEEVRVFEGTEELPGDPDMMRYVDRYGQLQTKML	354
AEC14877.1	LINRRTPRVDGQPMYGMDAQVEEVRVFEGTEELPGDPDMMRYVDRYGQLQTKML	354
AEC14878.1	LINRRTPRVDGQPMYGMDAQVEEVRVFEGTEELPGDPDMMRYVDRYGQLQTKML	354
AEC14879.1	LINRRTPRVDGQPMYGMDAQVEEVRVFEGTEELPGDPDMMRYVDRYGQLQTKML	354
AEC14880.1	LINRRTPRVDGQPMYGMDAQVEEVRVFEGTEELPGDPDMMRYVDRYGQLQTKML	354
AEC14881.1	LINRRTPRVDGQPMYGMDAQVEEVRVFEGTEELPGDPDMMRYVDRYGQLQTKML	354
AEC14882.1	LINRRTPRVDGQPMYGMDAQVEEVRVFEGTEELPGDPDMMRYVDRYGQLQTKML	354
AEC14883.1	LINRRTPRVDGQPMYGMDAQVEEVRVFEGTEELPGDPDMMRYVDRYGQLQTKML	354
AEC14884.1	LINRRTPRVDGQPMYGMDAQVEEVRVFEGTEELPGDPDMMRYVDRYGQLQTKML	354
AEC14885.1	LINRRTPRVDGQPMYGMDAQVEEVRVFEGTEELPGDPDMMRYVDRYGQLQTKML	354
AEC14886.1	LINRRTPRVDGQPMYGMDAQVEEVRVFEGTEELPGDPDMMRYVDRYGQLQTKML	354
AEC14887.1	LINRRTPRVDGQPMYGMDAQVEEVRVFEGTEELPGDPDMMRYVDRYGQLQTKML	354
AEC14888.1	LINRRTPRVDGQPMYGMDAQVEEVRVFEGTEELPGDPDMMRYVDRYGQLQTKML	354
AEC14889.1	LINRRTPRVDGQPMYGMDAQVEEVRVFEGTEELPGDPDMMRYVDRYGQLQTKML	354
AEC14890.1	LINRRTPRVDGQPMYGMDAQVEEVRVFEGTEELPGDPDMMRYVDRYGQLQTKML	354
AEC14891.1	LINRRTPRVDGQPMYGMDAQVEEVRVFEGTEELPGDPDMMRYVDRYGQLQTKML	354
AEC14892.1	LINRRTPRVDGQPMYGMDAQVEEVRVFEGTEELPGDPDMMRYVDRYGQLQTKML	354
AEC14894.1	LINRRTPRVDGQPMYGMDAQVEEVRVFEGTEELPGDPDMMRYVDRYGQLQTKML	354
AEC14895.1	LINRRTPRVDGQPMYGMDAQVEEVRVFEGTEELPGDPDMMRYVDRYGQLQTKML	354
AEC14896.1	LINRRTPRVDGQPMYGMDAQVEEVRVFEGTEELPGDPDMMRYVDRYGQLQTKML	354
AEC14897.1	LINRRTPRVDGQPMYGMDAQVEEVRVFEGTEELPGDPDMMRYVDRYGQLQTKML	354
AEC14898.1	LINRRTPRVDGQPMYGMDAQVEEVRVFEGTEELPGDPDMMRYVDRYGQLQTKML	354
AEC14899.1	LINRRTPRVDGQPMYGMDAQVEEVRVFEGTEELPGDPDMMRYVDRYGQLQTKML	354
AEC14900.1	LINRRTPRVDGQPMYGMDAQVEEVRVFEGTEELPGDPDMMRYVDRYGQLQTKML	354
AEC14901.1	LINRRTPRVDGQPMYGMDAQVEEVRVFEGTEELPGDPDMMRYVDRYGQLQTKML	354
AEC14902.1	LINRRTPRVDGQPMYGMDAQVEEVRVFEGTEELPGDPDMMRYVDRYGQLQTKML	354
AEC14903.1	LINRRTPRVDGQPMYGMDAQVEEVRVFEGTEELPGDPDMMRYVDRYGQLQTKML	354
AEC14904.1	LINRRTPRVDGQPMYGMDAQVEEVRVFEGTEELPGDPDMMRYVDRYGQLQTKML	354
AEC14905.1	LINRRTPRVDGQPMYGMDAQVEEVRVFEGTEELPGDPDMMRYVDRYGQLQTKML	354
AEC14907.1	LINRRTPRVDGQPMYGMDAQVEEVRVFEGTEELPGDPDMMRYVDRYGQLQTKML	354
AEC14908.1	LINRRTPRVDGQPMYGMDAQVEEVRVFEGTEELPGDPDMMRYVDRYGQLQTKML	354
AEC14909.1	LINRRTPRVDGQPMYGMDAQVEEVRVFEGTEELPGDPDMMRYVDRYGQLQTKML	354
AEC14910.1	LINRRTPRVDGQPMYGMDAQVEEVRVFEGTEELPGDPDMMRYVDRYGQLQTKML	354
AEC14911.1	LINRRTPRVDGQPMYGMDAQVEEVRVFEGTEELPGDPDMMRYVDRYGQLQTKML	354
AEC15164.1	LINRRTPRVDGQPMYGMDAQVEEVRVFEGTEELPGDPDMMRYVDRYGQLQTKML	354
BAF95594.1	LINRRTPRVDGQPMYGMDAQVEEVRVFEGTEELPGDPDMMRYVDRYGQLQTKML	354
BAF95606.1	LINRRTPRVDGQPMYGMDAQVEEVRVFEGTEELPGDPDMMRYVDRYGQLQTKML	354
BAF40745.1	LINRRTPRVDGQPMYGMDAQVEEVRVFEGTEELPGDPDMMRYVDRYGQLQTKML	354
BAF40751.1	LINRRTPRVDGQPMYGMDAQVEEVRVFEGTEELPGDPDMMRYVDRYGQLQTKML	354
BAF40781.1	LINRRTPRVDGQPMYGMDAQVEEVRVFEGTEELPGDPDMMRYVDRYGQLQTKML	354
BAF40787.1	LINRRTPRVDGQPMYGMDAQVEEVRVFEGTEELPGDPDMMRYVDRYGQLQTKML	354
BAF40793.1	LINRRTPRVDGQPMYGMDAQVEEVRVFEGTEELPGDPDMMRYVDRYGQLQTKML	354
BAF40799.1	LINRRTPRVDGQPMYGMDAQVEEVRVFEGTEELPGDPDMMRYVDRYGQLQTKML	354
BAF40811.1	LINRRTPRVDGQPMYGMDAQVEEVRVFEGTEELPGDPDMMRYVDRYGQLQTKML	354
BAF40817.1	LINRRTPRVDGQPMYGMDAQVEEVRVFEGTEELPGDPDMMRYVDRYGQLQTKML	354
BAF40823.1	LINRRTPRVDGQPMYGMDAQVEEVRVFEGTEELPGDPDMMRYVDRYGQLQTKML	354
BAF40829.1	LINRRTPRVDGQPMYGMDAQVEEVRVFEGTEELPGDPDMMRYVDRYGQLQTKML	354
BAF40835.1	LINRRTPRVDGQPMYGMDAQVEEVRVFEGTEELPGDPDMMRYVDRYGQLQTKML	354
BAF40841.1	LINRRTPRVDGQPMYGMDAQVEEVRVFEGTEELPGDPDMMRYVDRYGQLQTKML	354
BAF40847.1	LINRRTPRVDGQPMYGMDAQVEEVRVFEGTEELPGDPDMMRYVDRYGQLQTKML	354
BAC81834.1	LINRRTPRVDGQPMYGMDAQVEEVRVFEGTEELPGDPDMMRYVDRYGQLQTKML	354
BAC81840.1	LINRRTPRVDGQPMYGMDAQVEEVRVFEGTEELPGDPDMMRYVDRYGQLQTKML	354
AAN85461.1	LINRRTPRVDGQPMYGMDAQVEEVRVFEGTEELPGDPDMMRYVDRYGQLQTKML	354
AAN85467.1	LINRRTPRVDGQPMYGMDAQVEEVRVFEGTEELPGDPDMMRYVDRYGQLQTKML	354
AAN85473.1	LINRRTPRVDGQPMYGMDAQVEEVRVFEGTEELPGDPDMMRYVDRYGQLQTKML	354
BAC22762.1	LINRRTPRVDGQPMYGMDAQVEEVRVFEGTEELPGDPDMMRYVDRYGQLQTKML	354
BAC22768.1	LINRRTPRVDGQPMYGMDAQVEEVRVFEGTEELPGDPDMMRYVDRYGQLQTKML	354
BAC22774.1	LINRRTPRVDGQPMYGMDAQVEEVRVFEGTEELPGDPDMMRYVDRYGQLQTKML	354
BAC22780.1	LINRRTPRVDGQPMYGMDAQVEEVRVFEGTEELPGDPDMMRYVDRYGQLQTKML	354
BAC22786.1	LINRRTPRVDGQPMYGMDAQVEEVRVFEGTEELPGDPDMMRYVDRYGQLQTKML	354
BAB68809.1	LINRRTPRVDGQPMYGMDAQVEEVRVFEGTEELPGDPDMMRYVDRYGQLQTKML	354

BAB68815.1	LINRRTPRVDGQPMYGMDAQVEEVRVFEGTEELPGDPDMMRYVDRYGQLQTKML	354
BAB68827.1	LINRRTPRVDGQPMYGMDAQVEEVRVFEGTEELPGDPDMMRYVDRYGQLQTKML	354
AAC59496.1	LINRRTPRVDGQPMYGMDAQVEEVRVFEGTEELPGDPDMMRYVDRYGQLQTKML	354
AAC59502.1	LINRRTPRVDGQPMYGMDAQVEEVRVFEGTEELPGDPDMMRYVDRYGQLQTKML	354
AAC59508.1	LINRRTPRVDGQPMYGMDAQVEEVRVFEGTEELPGDPDMMRYVDRYGQLQTKML	354
AAC59514.1	LINRRTPRVDGQPMYGMDAQVEEVRVFEGTEELPGDPDMMRYVDRYGQLQTKML	354
AAC59520.1	LINRRTPRVDGQPMYGMDAQVEEVRVFEGTEELPGDPDMMRYVDRYGQLQTKML	354
AAB62680.1	LINRRTPRVDGQPMYGMDAQVEEVRVFEGTEELPGDPDMMRYVDRYGQLQTKML	354
BAA01961.1	LINRRTPRVDGQPMYGMDAQVEEVRVFEGTEELPGDPDMMRYVDRYGQLQTKML	354
WLD48082.1	LINRRTPRVDGQPMYGMDAQVEEVRVFEGTEELPGDPDMMRYVDRYGQLQTKML	354
WLD48088.1	LINRRTPRVDGQPMYGMDAQVEEVRVFEGTEELPGDPDMMRYVDRYGQLQTKML	354
CCW72516.1	LINRRTPRVDGQPMYGMDAQVEEVRVFEGTEELPGDPDMMRYVDRYGQLQTKML	354
CCW72523.1	LINRRTPRVDGQPMYGMDAQVEEVRVFEGTEELPGDPDMMRYXDRYGQLQTKML	354
AEC14783.1	LINRRTPRVDGQPMYGMDAQVEEVRVFEGTEELPGDPDMMRYVDRYGQLQTKML	354
AEC14784.1	LINRRTPRVDGQPMYGMDAQVEEVRVFEGTEELPGDPDMMRYVDRYGQLQTKML	354
AEC14785.1	LINRRTPRVDGQPMYGMDAQVEEVRVFEGTEELPGDPDMMRYVDRYGQLQTKML	354
AEC14786.1	LINRRTPRVDGQPMYGMDAQVEEVRVFEGTEELPGDPDMMRYVDRYGQLQTKML	354
AEC14787.1	LINRRTPRVDGQPMYGMDAQVEEVRVFEGTEELPGDPDMMRYVDRYGQLQTKML	354
AAB62687.1	LINRRTPRVDGQPMYGMDAQVEEVRVFEGTEELPGDPDMMRYVDRYGQLQTKML	354
AEC14893.1	LINRRTPRVDGQPMYGMDAQVEEVRVFEGTEELPGDPDMMRYVDRYGQLQTKML	354
AEC14906.1	LINRRTPRVDGQPMYGMDAQVEEVRVFEGTEELPGDPDMMRYVDRYGQLQTKML	354
AEC15118.1	LINRRTPRVDGQPMYGMDAQVEEVRVFEGTEELPGDPDMMRYVDRYGQLQTKML	354
AEC15120.1	LINRRTPRVDGQPMYGMDAQVEEVRVFEGTEELPGDPDMMRYVDRYGQLQTKML	354
AEC15121.1	LINRRTPRVDGQPMYGMDAQVEEVRVFEGTEELPGDPDMMRYVDRYGQLQTKML	354
AEC15122.1	LINRRTPRVDGQPMYGMDAQVEEVRVFEGTEELPGDPDMMRYVDRYGQLQTKML	354
AEC15124.1	LINRRTPRVDGQPMYGMDAQVEEVRVFEGTEELPGDPDMMRYVDRYGQLQTKML	354
AEC15125.1	LINRRTPRVDGQPMYGMDAQVEEVRVFEGTEELPGDPDMMRYVDRYGQLQTKML	354
AEC15127.1	LINRRTPRVDGQPMYGMDAQVEEVRVFEGTEELPGDPDMMRYVDRYGQLQTKML	354
AEC15129.1	LINRRTPRVDGQPMYGMDAQVEEVRVFEGTEELPGDPDMMRYVDRYGQLQTKML	354
AEC15131.1	LINRRTPRVDGQPMYGMDAQVEEVRVFEGTEELPGDPDMMRYVDRYGQLQTKML	354
AEC15133.1	LINRRTPRVDGQPMYGMDAQVEEVRVFEGTEELPGDPDMMRYVDRYGQLQTKML	354
AEC15135.1	LINRRTPRVDGQPMYGMDAQVEEVRVFEGTEELPGDPDMMRYVDRYGQLQTKML	354
AEC15136.1	LINRRTPRVDGQPMYGMDAQVEEVRVFEGTEELPGDPDMMRYVDRYGQLQTKML	354
AEC15137.1	LINRRTPRVDGQPMYGMDAQVEEVRVFEGTEELPGDPDMMRYVDRYGQLQTKML	354
AEC15138.1	LINRRTPRVDGQPMYGMDAQVEEVRVFEGTEELPGDPDMMRYVDRYGQLQTKML	354
AEC15139.1	LINRRTPRVDGQPMYGMDAQVEEVRVFEGTEELPGDPDMMRYVDRYGQLQTKML	354
AEC15141.1	LINRRTPRVDGQPMYGMDAQVEEVRVFEGTEELPGDPDMMRYVDRYGQLQTKML	354
AEC15142.1	LINRRTPRVDGQPMYGMDAQVEEVRVFEGTEELPGDPDMMRYVDRYGQLQTKML	354
AEC15143.1	LINRRTPRVDGQPMYGMDAQVEEVRVFEGTEELPGDPDMMRYVDRYGQLQTKML	354
AEC15145.1	LINRRTPRVDGQPMYGMDAQVEEVRVFEGTEELPGDPDMMRYVDRYGQLQTKML	354
AEC15146.1	LINRRTPRVDGQPMYGMDAQVEEVRVFEGTEELPGDPDMMRYVDRYGQLQTKML	354
AEC15147.1	LINRRTPRVDGQPMYGMDAQVEEVRVFEGTEELPGDPDMMRYVDRYGQLQTKML	354
AEC15148.1	LINRRTPRVDGQPMYGMDAQVEEVRVFEGTEELPGDPDMMRYVDRYGQLQTKML	354
AEC15149.1	LINRRTPRVDGQPMYGMDAQVEEVRVFEGTEELPGDPDMMRYVDRYGQLQTKML	354
AEC15150.1	LINRRTPRVDGQPMYGMDAQVEEVRVFEGTEELPGDPDMMRYVDRYGQLQTKML	354
AEC15151.1	LINRRTPRVDGQPMYGMDAQVEEVRVFEGTEELPGDPDMMRYVDRYGQLQTKML	354
AEC15152.1	LINRRTPRVDGQPMYGMDAQVEEVRVFEGTEELPGDPDMMRYVDRYGQLQTKML	354
AEC15153.1	LINRRTPRVDGQPMYGMDAQVEEVRVFEGTEELPGDPDMMRYVDRYGQLQTKML	354
AEC15155.1	LINRRTPRVDGQPMYGMDAQVEEVRVFEGTEELPGDPDMMRYVDRYGQLQTKML	354
AEC15156.1	LINRRTPRVDGQPMYGMDAQVEEVRVFEGTEELPGDPDMMRYVDRYGQLQTKML	354
AEC15158.1	LINRRTPRVDGQPMYGMDAQVEEVRVFEGTEELPGDPDMMRYVDRYGQLQTKML	354
AEC15159.1	LINRRTPRVDGQPMYGMDAQVEEVRVFEGTEELPGDPDMMRYVDRYGQLQTKML	354
AEC15161.1	LINRRTPRVDGQPMYGMDAQVEEVRVFEGTEELPGDPDMMRYVDRYGQLQTKML	354
AEC15162.1	LINRRTPRVDGQPMYGMDAQVEEVRVFEGTEELPGDPDMMRYVDRYGQLQTKML	354
AEC15163.1	LINRRTPRVDGQPMYGMDAQVEEVRVFEGTEELPGDPDMMRYVDRYGQLQTKML	354
AEC15226.1	LINRRTPRVDGQPMYGMDAQVEEVRVFEGTEELPGDPDMMRYVDRYGQLQTKML	354
AEC15227.1	LINRRTPRVDGQPMYGMDAQVEEVRVFEGTEELPGDPDMMRYVDRYGQLQTKML	354
AEC15228.1	LINRRTPRVDGQPMYGMDAQVEEVRVFEGTEELPGDPDMMRYVDRYGQLQTKML	354
AEC15229.1	LINRRTPRVDGQPMYGMDAQVEEVRVFEGTEELPGDPDMMRYVDRYGQLQTKML	354
AEC15230.1	LINRRTPRVDGQPMYGMDAQVEEVRVFEGTEELPGDPDMMRYVDRYGQLQTKML	354
AEC15236.1	LINRRTPRVDGQPMYGMDAQVEEVRVFEGTEELPGDPDMMRYVDRYGQLQTKML	354
AEC15237.1	LINRRTPRVDGQPMYGMDAQVEEVRVFEGTEELPGDPDMMRYVDRYGQLQTKML	354
AEC15238.1	LINRRTPRVDGQPMYGMDAQVEEVRVFEGTEELPGDPDMMRYVDRYGQLQTKML	354
AEC15239.1	LINRRTPRVDGQPMYGMDAQVEEVRVFEGTEELPGDPDMMRYVDRYGQLQTKML	354

AEC15240.1	LINRRTPRVDGQPMYGMDAQVEEVRVFEGTEELPGDPDMMRYVDRYGQLQTKML	354
AEC15447.1	LINRRTPRVDGQPMYGMDAQVEEVRVFEGTEELPGDPDMMRYVDRYGQLQTKML	354
AEC15453.1	LINRRTPRVDGQPMYGMDAQVEEVRVFEGTEELPGDPDMMRYVDRYGQLQTKML	354
AEC15459.1	LINRRTPRVDGQPMYGMDAQVEEVRVFEGTEELPGDPDMMRYVDRYGQLQTKML	354
AEC15465.1	LINRRTPRVDGQPMYGMDAQVEEVRVFEGTEELPGDPDMMRYVDRYGQLQTKML	354
AEC15471.1	LINRRTPRVDGQPMYGMDAQVEEVRVFEGTEELPGDPDMMRYVDRYGQLQTKML	354
AEC15477.1	LINRRTPRVDGQPMYGMDAQVEEVRVFEGTEELPGDPDMMRYVDRYGQLQTKML	354
AEC15483.1	LINRRTPRVDGQPMYGMDAQVEEVRVFEGTEELPGDPDMMRYVDRYGQLQTKML	354
AEC15489.1	LINRRTPRVDGQPMYGMDAQVEEVRVFEGTEELPGDPDMMRYVDRYGQLQTKML	354
AEC15543.1	LINRRTPRVDGQPMYGMDAQVEEVRVFEGTEELPGDPDMMRYVDRYGQLQTKML	354
AEC15119.1	LINRRTPRVDGQPMYGMDAQVEEVRVFEGTEELPGDPDMMRYVDRYGQLQTKML	354
AEC15123.1	LINRRTPRVDGQPMYGMDAQVEEVRVFEGTEELPGDPDMMRYVDRYGQLQTKML	354
AEC15126.1	LINRRTPRVDGQPMYGMDAQVEEVRVFEGTEELPGDPDMMRYVDRYGQLQTKML	354
AEC15132.1	LINRRTPRVDGQPMYGMDAQVEEVRVFEGTEELPGDPDMMRYVDRYGQLQTKML	354
AEC15134.1	LINRRTPRVDGQPMYGMDAQVEEVRVFEGTEELPGDPDMMRYVDRYGQLQTKML	354
AEC15140.1	LINRRTPRVDGQPMYGMDAQVEEVRVFEGTEELPGDPDMMRYVDRYGQLQTKML	354
AEC15221.1	LINRRTPRVDGQPMYGMDAQVEEVRVFEGTEELPGDPDMMRYVDRYGQLQTKML	354
AEC15224.1	LINRRTPRVDGQPMYGMDAQVEEVRVFEGTEELPGDPDMMRYVDRYGQLQTKML	354
AEC15225.1	LINRRTPRVDGQPMYGMDAQVEEVRVFEGTEELPGDPDMMRYVDRYGQLQTKML	354
AEC15233.1	LINRRTPRVDGQPMYGMDAQVEEVRVFEGTEELPGDPDMMRYVDRYGQLQTKML	354
AEC15234.1	LINRRTPRVDGQPMYGMDAQVEEVRVFEGTEELPGDPDMMRYVDRYGQLQTKML	354
AEC15235.1	LINRRTPRVDGQPMYGMDAQVEEVRVFEGTEELPGDPDMMRYVDRYGQLQTKML	354
AEC15241.1	LINRRTPRVDGQPMYGMDAQVEEVRVFEGTEELPGDPDMMRYVDRYGQLQTKML	354
AEC15220.1	LINRRTPRVDGQPMYGMDAQVEEVRVFEGTEELPGDPDMMRYVDRYGQLQTKML	354
AEC15223.1	LINRRTPRVDGQPMYGMDAQVEEVRVFEGTEELPGDPDMMRYVDRYGQLQTKML	354
AEC15232.1	LINRRTPRVDGQPMYGMDAQVEEVRVFEGTEELPGDPDMMRYVDRYGQLQTKML	354
AEC15128.1	LINRRTPRVDGQPMYGMDAQVEEVRVFEGTEELPGDPDMMRYVDRYGQLQTKML	354
AEC15495.1	LINRRTPRVDGQPMYGMDAQVEEVRVFEGTEELPGDPDMMRYVDRYGQLQTKML	354
AEC15501.1	LINRRTPRVDGQPMYGMDAQVEEVRVFEGTEELPGDPDMMRYVDRYGQLQTKML	354
AEC15507.1	LINRRTPRVDGQPMYGMDAQVEEVRVFEGTEELPGDPDMMRYVDRYGQLQTKML	354
AEC15513.1	LINRRTPRVDGQPMYGMDAQVEEVRVFEGTEELPGDPDMMRYVDRYGQLQTKML	354
AEC15519.1	LINRRTPRVDGQPMYGMDAQVEEVRVFEGTEELPGDPDMMRYVDRYGQLQTKML	354
AEC15525.1	LINRRTPRVDGQPMYGMDAQVEEVRVFEGTEELPGDPDMMRYVDRYGQLQTKML	354
AEC15531.1	LINRRTPRVDGQPMYGMDAQVEEVRVFEGTEELPGDPDMMRYVDRYGQLQTKML	354
AEC15537.1	LINRRTPRVDGQPMYGMDAQVEEVRVFEGTEELPGDPDMMRYVDRYGQLQTKML	354
BAF40805.1	LINRRTPRVDGQPMYGMDAQVEEVRVFEGTEELPGDPDMMRYVDRYGQLQTKML	354
AAT09843.1	LINRRTPRVDGQPMYGMDAQVEEVRVFEGTEELPGDPDMMRYVDRYGQLQTKML	354
BAA01965.1	LINRRTPRVDGQPMYGMDAQVEEVRVFEGTEELPGDPDMMRYVDRYGQLQTKML	354
AIW00320.1	LINRRTPRVDGQPMYGMDAQVEEVRVFEGTEELPGDPDMMRYVDRYGQLKML--	352
ALF06194.1	LINRRTPRVDGQPMYGMDAQVEEVRVFEGTEELPGDPDMMRYVDRYGQLQTKML	354
ALF06211.1	LINRRTPRVDGQPMYGMDAQVEEVRVFEGTEELPGDPDMMRYVDRYGQLQTKM-	353
AEC14821.1	LINRRTPRVDGQPMYGMDAQVEEVRVFEGTEELPGDPDMMRYVDRYGQLQTKML	354
AEC14860.1	LINRRTPRVDGQPMYGMDAQVEEVRVFEGTEELPGDPDMMRYVDRYGQLQTKML	354
AEC14861.1	LINRRTPRVDGQPMYGMDAQVEEVRVFEGTEELPGDPDMMRYVDRYGQLQTKML	354
AEC14811.1	LINRRTPRVDGQPMYGMDAQVEEVRVFEGTEELPGDPDMMRYVDRYGQLQTKML	354
AEC14806.1	LINRRTPRVDGQPMYGMDAQVEEVRVFEGTEELPGDPDMMRYVDRYGQLQTKML	354
AEC14795.1	LINRRTPRVDGQPMYGMDAQVEEVRVFEGTEELPGDPDMMRYVDRYGQLQTKML	354
AEC14765.1	LINRRTPRVDGQPMYGMDAQVEEVRVFEGTEELPGDPDMMRYVDRYGQLQTKML	354
AEC14774.1	LINRRTPRVDGQPMYGMDAQVEEVRVFEGTEELPGDPDMMRYVDRYGQLQTKML	354
AEC14761.1	LINRRTPRVDGQPMYGMDAQVEEVRVFEGTEELPGDPDMMRYVDRYGQLQTKML	354
AEC14753.1	LINRRTPRVDGQPMYGMDAQVEEVRVFEGTEELPGDPDMMRYVDRYGQLQTKML	354
AEC14767.1	LINRRTPRVDGQPMYGMDAQVEEVRVFEGTEELPGDPDMMRYVDRYGQLQTKML	354
AEC14780.1	LINRRTPRVDGQPMYGMDAQVEEVRVFEGTEELPGDPDMMRYVDRYGQLQTKML	354
AEC14760.1	LINRRTPRVDGQPMYGMDAQVEEVRVFEGTEELPGDPDMMRYVDRYGQLQTKML	354
AEC14762.1	LINRRTPRVDGQPMYGMDAQVEEVRVFEGTEELPGDPDMMRYVDRYGQLQTKML	354
AEC14742.1	LINRRTPRVDGQPMHGMDAQVEEVRVFEGTEELPGDPDMMRYVDRYGQLQTKML	354
AEC14763.1	LINRRTPRVDGQPMHGMDAQVEEVRVFEGTEELPGDPDMMRYVDRYGQLQTKML	354
AEC14810.1	LINRRTPRVDGQPMYGMDAQVEEVRVFEGTEELPGDPDMMRYVDRYGQLQTKML	354
AEC14803.1	LINGRTPRVDGQPMYGMDAQVEEVRVFEGTEELPGDPDMMRYVDRYGQLQTKML	354
AEC14802.1	LINRRTPRVDGQPMYGMDAQVEEVRVFEGTEELPGDPDMMRYVDRYGQLQTKML	354
AEC14812.1	LINRRTPRVDGQPMYGMDAQVEEVRVFEGTEELPGDPDMMRYVDRYGQLQTKML	354
AEC14804.1	LINRRTPRVDGQPMYGMDAQVEEVRVFEGTEELPGDPDMMRYVDRYGQLQTKML	354
AEC14797.1	LINRRTPRVDGQPMYGMDAQVEEVRVFEGTEELPGDPDVMRYVDRYGQLQTKML	354
AEC14796.1	LINRRTPRVDGQPMYGMDAQVEEVRVFEGTEELPGDPDMMRYVDRYGQLQTKML	354

AEC14793.1 LINRRTPRVDGQPMYGMDAQVEEVRVFEGTEELPGDPDMMRYVDRYGQLQTKML 354
AEC14791.1 LINRRTPRVDGQPMYGMDAQVEEVRVFEGTEELPGDPDMMRYVDRYGQLQTKML 354
AEC14794.1 LINRRTPRVDGQPMYGMDAQVEEVRVFEGTEELPGDPDMMRYVDRYGQLQTKML 354
AEC14789.1 LINRRTPRVDGQPMYGMDAQVEEVRVFEGTEELPGDPDMMRYVDRYGQLQTKML 354
AEC14743.1 LINRRTPRVDGQPMYGMDAQVEEVRVFEGTEELPGDPDMMRYVDRYGQLQTKML 354
AEC14745.1 LINRRTPRVDGQPMYGMDAQVEEVRVFEGTEELPGDPDMMRYVDRYGQLQTKML 354
AEC14750.1 LINRRTPRVDGQPMYGMGAQVEEVRVFEGTEELPGDPDMMRYVDRYGQLQTKML 354
AEC14754.1 LINRRTPRVDGQPMYGMGAQVEEVRVFEGTEELPGDPDMMRYVDRYGQLQTKML 354
AEC14751.1 LINRRTPRVDGQPMYGMDAQVEEVRVFEGTEELPGDPDMMRYVDRYGQLQTKML 354
AEC14776.1 LINRRTPRVDGQPMYGMDAQVEEVRVFEGTEELPGDPDMMRYVDRYGQLQTKML 354
AEC14755.1 LINRRTPRVDGQPMYGMDAQVEEVRVFEGTEELPGDPDMMRYVDRYGQLQTKML 354
AEC14759.1 LINRRTPRVDGQPMYGMDAQVEEVRVFEGTEELPGDPDMMRYVDRYGQLQTKML 354
AEC14766.1 LINRRTPRVDGQPMYGMDAQVEEVRVFEGTEELPGDPDMMRYVDRYGQLQTKML 354
AEC14781.1 LINRRTPRVDGQPMYGMDAQVEEVRVFEGTEELPGDPDMMRYVDRYGQLQTKML 354
AEC15186.1 LINRRTPRVDGQPMYGMDAQVEEVRVFEGTEELPGDPDMMRYVDRYGQLQTKML 354
AEC15192.1 LINRRTPRVDGQPMYDMDAQVEEVRVFEGTEELPGDPDMMRYVDRYGQLQTKML 354
AEC15194.1 LINRRTPRVDGQPMYGMDAQVEEVRVFEGTEELPGDPDMMRYVDRYGQLQTKML 354
AEC15195.1 LINRRTPRVDGQPMYGMDAQVEEVRVFEGTEELPGDPDMMRYVDRYGQLQTKML 354
QWT77104.1 LINRRTPRVDGQPMYGMDAQVEEVRVFEGTEELPGDPDMMRYVDRYGQLQTKML 354
UPN63088.1 LINRRTPRVDGQPMYGMDAQVEEVRVFEGTEELPGDPDMMRYVDRYGQLQTKML 354
UPN63104.1 LINRRTPRVDGQPMYGMDAQVEEVRVFEGTEELPGDPDMMRYVDRYGQLQTKML 354
UPN63112.1 LINRRTPRVDGQPMYGMDAQVEEVRVFEGTEELPGDPDMMRYVDRYGQLQTKML 354
UPN63113.1 LINRRTPRVDGQPMYGMDAQVEEVRVFEGTEELPGDPDMMRYVDRYGQLQTKML 354
UPN63114.1 LINRRTPRVDGQPMYGMDAQVGEVRVFEGTEELPGDPDMMRYVDRYGQLQTKML 354
UPN63115.1 LVNRRTPRVDGQPMYGMDAQVEEVRVFEGTEELPGDPDMMRYVDRYGQLQTKML 354
AEC15172.1 LINRRTPRVDGQPMYGMDAQVEEVRVFEGTEELPGDPDMMRYVDRYGQLQTKML 354
BAE00117.1 LINRRTPRVDGQPMYGMDAQVEEVRVFEGTEELPGDPDMMRYVDKYGQLQTKML 354
BAE00123.1 LINRRTPRVDGQPMYGMDAQVEEVRVFEGTEELPGDPDMMRYVDKYGQLQTKML 354
AAC59331.1 LINRRTPRVDGQPMYGMDAQVEEVRVFEGTEELPGDPDMMRYVDRYGQLQTKML 354
WLD48106.1 LINRRTPRVDGQPMYGMDAQVEEVRVFEGTEELPGDPDMMRYVDRYGQLQTKML 354
UQK62661.1 LINRRTPRVDGQPMYGMDAQVEEVRVFEGTEELPGDPDMMRYVDRYGQLQTKML 354
QTJ15156.1 LINRRTPRVDGQPMYGMDAQVEEVRVFEGTEELPGDPDMMRYVDRYGQLQTKML 354
QNC68213.1 LINRRTPRVDGQPMYGMDAQVEEVRVFEGTEELPGDPDMMRYVDRYGQLQTKML 354
ASV51766.1 LINRRTPRVDGQPMYGMDAQVEEVRVFEGTEELPGDPDMMRYVDRYGQLQTKML 354
ASV51796.1 LINRRTPRVDGQPMYGMDAQVEEVRVFEGTEELPGDPDMMRYVDRYGQLQTKML 354
ASV51802.1 LINRRTPRVDGQPMYGMDAQVEEVRVFEGTEELPGDPDMMRYVDRYGQLQTKML 354
ASV51808.1 LINRRTPRVDGQPMYGMDAQVEEVRVFEGTEELPGDPDMMRYVDRYGQLQTKML 354
ASV51820.1 LINRRTPRVDGQPMYGMDAQVEEVRVFEGTEELPGDPDMMRYVDRYGQLQTKML 354
ASV51826.1 LINRRTPRVDGQPMYGMDAQVEEVRVFEGTEELPGDPDMMRYVDRYGQLQTKML 354
ASV51832.1 LINRRTPRVDGQPMYGMDAQVEEVRVFEGTEELPGDPDMMRYVDRYGQLQTKML 354
ASV51838.1 LINRRTPRVDGQPMYGMDAQVEEVRVFEGTEELPGDPDMMRYVDRYGQLQTKML 354
ASV51862.1 LINRRTPRVDGQPMYGMDAQVEEVRVFEGTEELPGDPDMMRYVDRYGQLQTKML 354
ASV51868.1 LINRRTPRVDGQPMYGMDAQVEEVRVFEGTEELPGDPDMMRYVDRYGQLQTKML 354
ASV51874.1 LINRRTPRVDGQPMYGMDAQVEEVRVFEGTEELPGDPDMMRYVDRYGQLQTKML 354
ASV51880.1 LINRRTPRVDGQPMYGMDAQVEEVRVFEGTEELPGDPDMMRYVDRYGQLQTKML 354
ASV51892.1 LINRRTPRVDGQPMYGMDAQVEEVRVFEGTEELPGDPDMMRYVDRYGQLQTKML 354
ANH21098.1 LINRRTPRVDGQPMYGMDAQVEEVRVFEGTEELPGDPDMMRYVDRYGQLQTKML 354
AID67150.1 LINRRTPRVDGQPMYGMDAQVEEVRVFEGTEELPGDPDMMRYVDRYGQLQTKML 354
AID67156.1 LINRRTPRVDGQPMYGMDAQVEEVRVFEGTEELPGDPDMMRYVDRYGQLQTKML 354
AID67162.1 LINRRTPRVDGQPMYGMDAQVEEVRVFEGTEELPGDPDMMRYVDRYGQLQTKML 354
AID67168.1 LINRRTPRVDGQPMYGMDAQVEEVRVFEGTEELPGDPDMMRYVDRYGQLQTKML 354
CCW72479.1 LINRRTPRVDGQPMYGMDAQVEEVRVFEGTEELPGDPDMMRYVDRYGQLQTKML 354
CCW72486.1 LINRRTPRVDGQPMYGMDAQVEEVRVFEGTEELPGDPDMMRYVDRYGQLQTKML 354
CCW72494.1 LINRRTPRVDGQPMYGMDAQVEEVRVFEGTEELPGDPDMMRYVDRYGQLQTKML 354
CCW72514.1 LINRRTPRVDGQPMYGMDAQVEEVRVFEGTEELPGDPDMMRYVDRYGQLQTKML 354
CCW72518.1 LINRRTPRVDGQPMYGMDAQVEEVRVFEGTEELPGDPDMMRYVDRYGQLQTKML 354
CCW72522.1 LINRRTPRVDGQPMYGMDAQVEEVRVFEGTEELPGDPDMMRYVDRYGQLQTKML 354
AFH57194.1 LINRRTPRVDGQPMYGMDAQVEEVRVFEGTEELPGDPDMMRYVDRYGQLQTKML 354
AEC15047.1 LINRRTPRVDGQPMYGMDAQVEEVRVFEGTEELPGDPDMMRYVDRYGQLQTKML 354
AEC15048.1 LINRRTPRVDGQPMYGMDAQVEEVRVFEGTEELPGDPDMMRYVDRYGQLQTKML 354
AEC15049.1 LINRRTPRVDGQPMYGMDAQVEEVRVFEGTEELPGDPDMMRYVDRYGQLQTKML 354
AEC15050.1 LINRRTPRVDGQPMYGMDAQVEEVRVFEGTEELPGDPDMMRYVDRYGQLQTKML 354
AEC15051.1 LINRRTPRVDGQPMYGMDAQVEEVRVFEGTEELPGDPDMMRYVDRYGQLQTKML 354
AEC15052.1 LINRRTPRVDGQPMYGMDAQVEEVRVFEGTEELPGDPDMMRYVDRYGQLQTKML 354

AEC15053.1 LINRRTPRVDGQPMYGMDAQVEEVRVFEGTEELPGDPDMMRYVDRYGQLQTKML 354
AEC15054.1 LINRRTPRVDGQPMYGMDAQVEEVRVFEGTEELPGDPDMMRYVDRYGQLQTKML 354
AEC15055.1 LINRRTPRVDGQPMYGMDAQVEEVRVFEGTEELPGDPDMMRYVDRYGQLQTKML 354
AEC15056.1 LINRRTPRVDGQPMYGMDAQVEEVRVFEGTEELPGDPDMMRYVDRYGQLQTKML 354
AEC15057.1 LINRRTPRVDGQPMYGMDAQVEEVRVFEGTEELPGDPDMMRYVDRYGQLQTKML 354
AEC15058.1 LINRRTPRVDGQPMYGMDAQVEEVRVFEGTEELPGDPDMMRYVDRYGQLQTKML 354
AEC15059.1 LINRRTPRVDGQPMYGMDAQVEEVRVFEGTEELPGDPDMMRYVDRYGQLQTKML 354
AEC15060.1 LINRRTPRVDGQPMYGMDAQVEEVRVFEGTEELPGDPDMMRYVDRYGQLQTKML 354
AEC15061.1 LINRRTPRVDGQPMYGMDAQVEEVRVFEGTEELPGDPDMMRYVDRYGQLQTKML 354
AEC15062.1 LINRRTPRVDGQPMYGMDAQVEEVRVFEGTEELPGDPDMMRYVDRYGQLQTKML 354
AEC15063.1 LINRRTPRVDGQPMYGMDAQVEEVRVFEGTEELPGDPDMMRYVDRYGQLQTKML 354
AEC15064.1 LINRRTPRVDGQPMYGMDAQVEEVRVFEGTEELPGDPDMMRYVDRYGQLQTKML 354
AEC15065.1 LINRRTPRVDGQPMYGMDAQVEEVRVFEGTEELPGDPDMMRYVDRYGQLQTKML 354
AEC15066.1 LINRRTPRVDGQPMYGMDAQVEEVRVFEGTEELPGDPDMMRYVDRYGQLQTKML 354
AEC15067.1 LINRRTPRVDGQPMYGMDAQVEEVRVFEGTEELPGDPDMMRYVDRYGQLQTKML 354
AEC15068.1 LINRRTPRVDGQPMYGMDAQVEEVRVFEGTEELPGDPDMMRYVDRYGQLQTKML 354
AEC15315.1 LINRRTPRVDGQPMYGMDAQVEEVRVFEGTEELPGDPDMMRYVDRYGQLQTKML 354
AEC15321.1 LINRRTPRVDGQPMYGMDAQVEEVRVFEGTEELPGDPDMMRYVDRYGQLQTKML 354
BAD21271.1 LINRRTPRVDGQPMYGMDAQVEEVRVFEGTEELPGDPDMMRYVDRYGQLQTKML 354
BAD21283.2 LINRRTPRVDGQPMYGMDAQVEEVRVFEGTEELPGDPDMMRYVDRYGQLQTKML 354
BAC15683.1 LINRRTPRVDGQPMYGMDAQVEEVRVFEGTEELPGDPDMMRYVDRYGQLQTKML 354
BAC15719.1 LINRRTPRVDGQPMYGMDAQVEEVRVFEGTEELPGDPDMMRYVDRYGQLQTKML 354
BAB93038.1 LINRRTPRVDGQPMYGMDAQVEEVRVFEGTEELPGDPDMMRYVDRYGQLQTKML 354
BAB93056.1 LINRRTPRVDGQPMYGMDAQVEEVRVFEGTEELPGDPDMMRYVDRYGQLQTKML 354
BAB93062.1 LINRRTPRVDGQPMYGMDAQVEEVRVFEGTEELPGDPDMMRYVDRYGQLQTKML 354
BAB68911.1 LINRRTPRVDGQPMYGMDAQVEEVRVFEGTEELPGDPDMMRYVDRYGQLQTKML 354
BAB68947.1 LINRRTPRVDGQPMYGMDAQVEEVRVFEGTEELPGDPDMMRYVDRYGQLQTKML 354
AAK70234.1 LINRRTPRVDGQPMYGMDAQVEEVRVFEGTEELPGDPDMMRYVDRYGQLQTKML 354
AAK70246.1 LINRRTPRVDGQPMYGMDAQVEEVRVFEGTEELPGDPDMMRYVDRYGQLQTKML 354
BAA01959.1 LINRRTPRVDGQPMYGMDAQVEEVRVFEGTEELPGDPDMMRYVDRYGQLQTKML 354
BAA01960.1 LINRRTPRVDGQPMYGMDAQVEEVRVFEGTEELPGDPDMMRYVDRYGQLQTKML 354
QWT77128.1 LINRRTPRVDGQPMYGMDAQVEEVRVFEGTEELPGDPDMMRYVDRYGQLQTKML 354
UPN63089.1 LINRRTPRVDGQPMYGMDAQVEEVRVFEGTEELPGDPDMMRYVDRYGQLQTKML 354
UPN63093.1 LINRRTPRVDGQPMYGMDAQVEEVRVFEGTEELPGDPDMMRYVDRYGQLQTKML 354
UPN63103.1 LINRRTPRVDGQPMYGMDAQVEEVRVFEGTEELPGDPDMMRYVDRYGQLQTKML 354
UPN63105.1 LINRRTPRVDGQPMYGMDAQVEEVRVFEGTEELPGDPDMMRYVDRYGQLQTKML 354
UPN63106.1 LINRRTPRVDGQPMYGMDAQVEEVRVFEGTEELPGDPDMMRYVDRYGQLQTKML 354
UPN63110.1 LINRRTPRVDGQPMYGMDAQVEEVRVFEGTEELPGDPDMMRYVDRYGQLQTKML 354
UPN63111.1 LINRRTPRVDGQPMYGMDAQVEEVRVFEGTEELPGDPDMMRYVDRYGQLQTKML 354
AEC15030.1 LINRRTPRVDGQPMYGMDAQVEEVRVFEGTEELPGDPDMMRYVDRYGQLQTKML 354
AEC15031.1 LINRRTPRVDGQPMYGMDAQVEEVRVFEGTEELPGDPDMMRYVDRYGQLQTKML 354
AEC15032.1 LINRRTPRVDGQPMYGMDAQVEEVRVFEGTEELPGDPDMMRYVDRYGQLQTKML 354
AEC15033.1 LINRRTPRVDGQPMYGMDAQVEEVRVFEGTEELPGDPDMMRYVDRYGQLQTKML 354
AEC15034.1 LINRRTPRVDGQPMYGMDAQVEEVRVFEGTEELPGDPDMMRYVDRYGQLQTKML 354
AEC15035.1 LINRRTPRVDGQPMYGMDAQVEEVRVFEGTEELPGDPDMMRYVDRYGQLQTKML 354
AEC15036.1 LINRRTPRVDGQPMYGMDAQVEEVRVFEGTEELPGDPDMMRYVDRYGQLQTKML 354
AEC15038.1 LINRRTPRVDGQPMYGMDAQVEEVRVFEGTEELPGDPDMMRYVDRYGQLQTKML 354
AEC15039.1 LINRRTPRVDGQPMYGMDAQVEEVRVFEGTEELPGDPDMMRYVDRYGQLQTKML 354
AEC15040.1 LINRRTPRVDGQPMYGMDAQVEEVRVFEGTEELPGDPDMMRYVDRYGQLQTKML 354
AEC15041.1 LINRRTPRVDGQPMYGMDAQVEEVRVFEGTEELPGDPDMMRYVDRYGQLQTKML 354
AEC15042.1 LINRRTPRVDGQPMYGMDAQVEEVRVFEGTEELPGDPDMMRYVDRYGQLQTKML 354
AEC15043.1 LINRRTPRVDGQPMYGMDAQVEEVRVFEGTEELPGDPDMMRYVDRYGQLQTKML 354
AEC15044.1 LINRRTPRVDGQPMYGMDAQVEEVRVFEGTEELPGDPDMMRYVDRYGQLQTKML 354
AEC15045.1 LINRRTPRVDGQPMYGMDAQVEEVRVFEGTEELPGDPDMMRYVDRYGQLQTKML 354
AEC15046.1 LINRRTPRVDGQPMYGMDAQVEEVRVFEGTEELPGDPDMMRYVDRYGQLQTKML 354
UPN63083.1 LINRRTPRVDGQPMYGMDAQVEEVRVFEGTEELPGDPDMMRYVDRYGQLQTKML 354
BAE00111.1 LINRRTPRVDGQPMYGMDAQVEEVRVFEGTEELPGDPDMMRYVDKYGQLQTKML 354
BAA01968.1 LINRRTPRVDGQPMYGMDAQVEEVRVFEGTEELPGDPDMMRYVDKYGQLQTKML 354
CCW72491.1 LINRRTPRVDGQPMYGMDAQVEEVRVFEGTEELPGDPDMMRYVDRYGQLQTKML 354
AEC14788.1 LINRRTPRVDGQPMYGMDAQVEEVRVFEGTEELPGDPDMMRYVDRYGQLQTKML 354
AEC14790.1 LINRRTPRVDGQPMYGMDAQVEEVRVFEGTEELPGDPDMMRYVDRYGQLQTKML 354
AEC14792.1 LINRRTPRVDGQPMYGMDAQVEEVRVFEGTEELPGDPDMMRYVDRYGQLQTKML 354
AEC14798.1 LINRRTPRVDGQPMYGMDAQVEEVRVFEGTEELPGDPDMMRYVDRYGQLQTKML 354
AEC14799.1 LINRRTPRVDGQPMYGMDAQVEEVRVFEGTEELPGDPDMMRYVDRYGQLQTKML 354

AEC14800.1	LINRRTPRVDGQPMYGMDAQVEEVRVFEGTEELPGDPDMMRYVDRYGQLQTKML	354
AEC14801.1	LINRRTPRVDGQPMYGMDAQVEEVRVFEGTEELPGDPDMMRYVDRYGQLQTKML	354
AEC14805.1	LINRRTPRVDGQPMYGMDAQVEEVRVFEGTEELPGDPDMMRYVDRYGQLQTKML	354
AEC14807.1	LINRRTPRVDGQPMYGMDAQVEEVRVFEGTEELPGDPDMMRYVDRYGQLQTKML	354
AEC14808.1	LINRRTPRVDGQPMYGMDAQVEEVRVFEGTEELPGDPDMMRYVDRYGQLQTKML	354
AEC14809.1	LINRRTPRVDGQPMYGMDAQVEEVRVFEGTEELPGDPDMMRYVDRYGQLQTKML	354
AEC14813.1	LINRRTPRVDGQPMYGMDAQVEEVRVFEGTEELPGDPDMMRYVDRYGQLQTKML	354
AEC14912.1	LINRRTPRVDGQPMYGMDAQVEEVRVFEGTEELPGDPDMMRYVDRYGQLQTKML	354
AEC14913.1	LINRRTPRVDGQPMYGMDAQVEEVRVFEGTEELPGDPDMMRYVDRYGQLQTKML	354
AEC14914.1	LINRRTPRVDGQPMYGMDAQVEEVRVFEGTEELPGDPDMMRYVDRYGQLQTKML	354
AEC14915.1	LINRRTPRVDGQPMYGMDAQVEEVRVFEGTEELPGDPDMMRYVDRYGQLQTKML	354
AEC14916.1	LINRRTPRVDGQPMYGMDAQVEEVRVFEGTEELPGDPDMMRYVDRYGQLQTKML	354
AEC14917.1	LINRRTPRVDGQPMYGMDAQVEEVRVFEGTEELPGDPDMMRYVDRYGQLQTKML	354
AEC14918.1	LINRRTPRVDGQPMYGMDAQVEEVRVFEGTEELPGDPDMMRYVDRYGQLQTKML	354
AEC14919.1	LINRRTPRVDGQPMYGMDAQVEEVRVFEGTEELPGDPDMMRYVDRYGQLQTKML	354
AEC14920.1	LINRRTPRVDGQPMYGMDAQVEEVRVFEGTEELPGDPDMMRYVDRYGQLQTKML	354
AEC14921.1	LINRRTPRVDGQPMYGMDAQVEEVRVFEGTEELPGDPDMMRYVDRYGQLQTKML	354
AEC14922.1	LINRRTPRVDGQPMYGMDAQVEEVRVFEGTEELPGDPDMMRYVDRYGQLQTKML	354
AEC14923.1	LINRRTPRVDGQPMYGMDAQVEEVRVFEGTEELPGDPDMMRYVDRYGQLQTKML	354
AEC14924.1	LINRRTPRVDGQPMYGMDAQVEEVRVFEGTEELPGDPDMMRYVDRYGQLQTKML	354
AEC14925.1	LINRRTPRVDGQPMYGMDAQVEEVRVFEGTEELPGDPDMMRYVDRYGQLQTKML	354
AEC14926.1	LINRRTPRVDGQPMYGMDAQVEEVRVFEGTEELPGDPDMMRYVDRYGQLQTKML	354
AEC14927.1	LINRRTPRVDGQPMYGMDAQVEEVRVFEGTEELPGDPDMMRYVDRYGQLQTKML	354
AEC14928.1	LINRRTPRVDGQPMYGMDAQVEEVRVFEGTEELPGDPDMMRYVDRYGQLQTKML	354
AEC14929.1	LINRRTPRVDGQPMYGMDAQVEEVRVFEGTEELPGDPDMMRYVDRYGQLQTKML	354
AEC14930.1	LINRRTPRVDGQPMYGMDAQVEEVRVFEGTEELPGDPDMMRYVDRYGQLQTKML	354
AEC14931.1	LINRRTPRVDGQPMYGMDAQVEEVRVFEGTEELPGDPDMMRYVDRYGQLQTKML	354
AEC14932.1	LINRRTPRVDGQPMYGMDAQVEEVRVFEGTEELPGDPDMMRYVDRYGQLQTKML	354
AEC14933.1	LINRRTPRVDGQPMYGMDAQVEEVRVFEGTEELPGDPDMMRYVDRYGQLQTKML	354
AEC14934.1	LINRRTPRVDGQPMYGMDAQVEEVRVFEGTEELPGDPDMMRYVDRYGQLQTKML	354
AEC14935.1	LINRRTPRVDGQPMYGMDAQVEEVRVFEGTEELPGDPDMMRYVDRYGQLQTKML	354
AEC14936.1	LINRRTPRVDGQPMYGMDAQVEEVRVFEGTEELPGDPDMMRYVDRYGQLQTKML	354
AEC14937.1	LINRRTPRVDGQPMYGMDAQVEEVRVFEGTEELPGDPDMMRYVDRYGQLQTKML	354
AEC14938.1	LINRRTPRVDGQPMYGMDAQVEEVRVFEGTEELPGDPDMMRYVDRYGQLQTKML	354
AEC14939.1	LINRRTPRVDGQPMYGMDAQVEEVRVFEGTEELPGDPDMMRYVDRYGQLQTKML	354
AEC14940.1	LINRRTPRVDGQPMYGMDAQVEEVRVFEGTEELPGDPDMMRYVDRYGQLQTKML	354
AEC14941.1	LINRRTPRVDGQPMYGMDAQVEEVRVFEGTEELPGDPDMMRYVDRYGQLQTKML	354
AEC14942.1	LINRRTPRVDGQPMYGMDAQVEEVRVFEGTEELPGDPDMMRYVDRYGQLQTKML	354
AEC14943.1	LINRRTPRVDGQPMYGMDAQVEEVRVFEGTEELPGDPDMMRYVDRYGQLQTKML	354
AEC14944.1	LINRRTPRVDGQPMYGMDAQVEEVRVFEGTEELPGDPDMMRYVDRYGQLQTKML	354
AEC14945.1	LINRRTPRVDGQPMYGMDAQVEEVRVFEGTEELPGDPDMMRYVDRYGQLQTKML	354
AEC14946.1	LINRRTPRVDGQPMYGMDAQVEEVRVFEGTEELPGDPDMMRYVDRYGQLQTKML	354
AEC14947.1	LINRRTPRVDGQPMYGMDAQVEEVRVFEGTEELPGDPDMMRYVDRYGQLQTKML	354
AEC14948.1	LINRRTPRVDGQPMYGMDAQVEEVRVFEGTEELPGDPDMMRYVDRYGQLQTKML	354
AEC14949.1	LINRRTPRVDGQPMYGMDAQVEEVRVFEGTEELPGDPDMMRYVDRYGQLQTKML	354
AEC14950.1	LINRRTPRVDGQPMYGMDAQVEEVRVFEGTEELPGDPDMMRYVDRYGQLQTKML	354
AEC14951.1	LINRRTPRVDGQPMYGMDAQVEEVRVFEGTEELPGDPDMMRYVDRYGQLQTKML	354
AEC14952.1	LINRRTPRVDGQPMYGMDAQVEEVRVFEGTEELPGDPDMMRYVDRYGQLQTKML	354
AEC14953.1	LINRRTPRVDGQPMYGMDAQVEEVRVFEGTEELPGDPDMMRYVDRYGQLQTKML	354
AEC14954.1	LINRRTPRVDGQPMYGMDAQVEEVRVFEGTEELPGDPDMMRYVDRYGQLQTKML	354
AEC14955.1	LINRRTPRVDGQPMYGMDAQVEEVRVFEGTEELPGDPDMMRYVDRYGQLQTKML	354
AEC14956.1	LINRRTPRVDGQPMYGMDAQVEEVRVFEGTEELPGDPDMMRYVDRYGQLQTKML	354
AEC14957.1	LINRRTPRVDGQPMYGMDAQVEEVRVFEGTEELPGDPDMMRYVDRYGQLQTKML	354
AEC14958.1	LINRRTPRVDGQPMYGMDAQVEEVRVFEGTEELPGDPDMMRYVDRYGQLQTKML	354
AEC14959.1	LINRRTPRVDGQPMYGMDAQVEEVRVFEGTEELPGDPDMMRYVDRYGQLQTKML	354
AEC14960.1	LINRRTPRVDGQPMYGMDAQVEEVRVFEGTEELPGDPDMMRYVDRYGQLQTKML	354
AEC14961.1	LINRRTPRVDGQPMYGMDAQVEEVRVFEGTEELPGDPDMMRYVDRYGQLQTKML	354
QWT77122.1	LINRRTPRVDGQPMYGMDAQVEEVRVFEGTEELPGDPDMMRYVDRYGQLQTKML	354
UPN63084.1	LINRRTPRVDGQPMYGMDAQVEEVRVFEGTEELPGDPDMMRYVDRYGQLQTKML	354
UPN63101.1	LINRRTPRVDGQPMYGMDAQVEEVRVFEGTEELPGDPDMMRYVDRYGQLQTKML	354
ASV51886.1	LINRRTPRVDGQPMYGMDAQVEEVRVFEGTEELPGDPDMMRYVDRYGQLQTKML	354
CCW72484.1	LINRRTPRVDGQPMYGMDAQVEEVRVFEGTEELPGDPDMMRYVDRYGQLQTKML	354
ANH21099.1	LINRRTPRVDGQPMYGMDAQVEEVRVFEGTEELPGDPDMMRYVDRYGQLQTKML	354
AEC15112.1	LINRRTPRVDGQPMYGMDAQVEEVRVFEGTEELPGDPDMMRYVDRYGQLQTKML	354

AEC15113.1	LINRRTPRVDGQPMYGMDAQVEEVRVFEGTEELPGDPDMMRYVDRYGQLQTKML	354
AEC15114.1	LINRRTPRVDGQPMYGMDAQVEEVRVFEGTEELPGDPDMMRYVDRYGQLQTKML	354
AEC15115.1	LINRRTPRVDGQPMYGMDAQVEEVRVFEGTEELPGDPDMMRYVDRYGQLQTKML	354
AEC15116.1	LINRRTPRVDGQPMYGMDAQVEEVRVFEGTEELPGDPDMMRYVDRYGQLQTKML	354
AEC15117.1	LINRRTPRVDGQPMYGMDAQVEEVRVFEGTEELPGDPDMMRYVDRYGQLQTKML	354
AEC15819.1	LINRRTPRVDGQPMYGMDAQVEEVRVFEGTEELPGDPDMMRYVDRYGQLQTKML	354
AEC15825.1	LINRRTPRVDGQPMYGMDAQVEEVRVFEGTEELPGDPDMMRYVDRYGQLQTKML	354
AEC15831.1	LINRRTPRVDGQPMYGMDAQVEEVRVFEGTEELPGDPDMMRYVDRYGQLQTKML	354
AEC15837.1	LINRRTPRVDGQPMYGMDAQVEEVRVFEGTEELPGDPDMMRYVDRYGQLQTKML	354
AEC15843.1	LINRRTPRVDGQPMYGMDAQVEEVRVFEGTEELPGDPDMMRYVDRYGQLQTKML	354
AEC15849.1	LINRRTPRVDGQPMYGMDAQVEEVRVFEGTEELPGDPDMMRYVDRYGQLQTKML	354
AEC15855.1	LINRRTPRVDGQPMYGMDAQVEEVRVFEGTEELPGDPDMMRYVDRYGQLQTKML	354
CCW72511.1	LINRRTPRVDGQPMYGMDAQVEEVRVFEGTEELPGDPDMMRYVDRYGQLQTKML	354
CCW72525.1	LINRRTPRVDGQPMYGMDAQVEEVRVFEGTEELPGDPDMMRYVDRYGQLQTKML	354
AEC14744.1	LINRRTPRVDGQPMYGMDAQVEEVRVFEGTEELPGDPDMMRYVDRYGQLQTKML	354
AEC14746.1	LINRRTPRVDGQPMYGMDAQVEEVRVFEGTEELPGDPDMMRYVDRYGQLQTKML	354
AEC14747.1	LINRRTPRVDGQPMYGMDAQVEEVRVFEGTEELPGDPDMMRYVDRYGQLQTKML	354
AEC14748.1	LINRRTPRVDGQPMYGMDAQVEEVRVFEGTEELPGDPDMMRYVDRYGQLQTKML	354
AEC14752.1	LINRRTPRVDGQPMYGMDAQVEEVRVFEGTEELPGDPDMMRYVDRYGQLQTKML	354
AEC14756.1	LINRRTPRVDGQPMYGMDAQVEEVRVFEGTEELPGDPDMMRYVDRYGQLQTKML	354
AEC14757.1	LINRRTPRVDGQPMYGMDAQVEEVRVFEGTEELPGDPDMMRYVDRYGQLQTKML	354
AEC14758.1	LINRRTPRVDGQPMYGMDAQVEEVRVFEGTEELPGDPDMMRYVDRYGQLQTKML	354
AEC14764.1	LINRRTPRVDGQPMYGMDAQVEEVRVFEGTEELPGDPDMMRYVDRYGQLQTKML	354
AEC14768.1	LINRRTPRVDGQPMYGMDAQVEEVRVFEGTEELPGDPDMMRYVDRYGQLQTKML	354
AEC14769.1	LINRRTPRVDGQPMYGMDAQVEEVRVFEGTEELPGDPDMMRYVDRYGQLQTKML	354
AEC14770.1	LINRRTPRVDGQPMYGMDAQVEEVRVFEGTEELPGDPDMMRYVDRYGQLQTKML	354
AEC14771.1	LINRRTPRVDGQPMYGMDAQVEEVRVFEGTEELPGDPDMMRYVDRYGQLQTKML	354
AEC14772.1	LINRRTPRVDGQPMYGMDAQVEEVRVFEGTEELPGDPDMMRYVDRYGQLQTKML	354
AEC14773.1	LINRRTPRVDGQPMYGMDAQVEEVRVFEGTEELPGDPDMMRYVDRYGQLQTKML	354
AEC14775.1	LINRRTPRVDGQPMYGMDAQVEEVRVFEGTEELPGDPDMMRYVDRYGQLQTKML	354
AEC14777.1	LINRRTPRVDGQPMYGMDAQVEEVRVFEGTEELPGDPDMMRYVDRYGQLQTKML	354
AEC14778.1	LINRRTPRVDGQPMYGMDAQVEEVRVFEGTEELPGDPDMMRYVDRYGQLQTKML	354
AEC14779.1	LINRRTPRVDGQPMYGMDAQVEEVRVFEGTEELPGDPDMMRYVDRYGQLQTKML	354
AEC14782.1	LINRRTPRVDGQPMYGMDAQVEEVRVFEGTEELPGDPDMMRYVDRYGQLQTKML	354
AEC14814.1	LINRRTPRVDGQPMYGMDAQVEEVRVFEGTEELPGDPDMMRYVDRYGQLQTKML	354
AEC14815.1	LINRRTPRVDGQPMYGMDAQVEEVRVFEGTEELPGDPDMMRYVDRYGQLQTKML	354
AEC14816.1	LINRRTPRVDGQPMYGMDAQVEEVRVFEGTEELPGDPDMMRYVDRYGQLQTKML	354
AEC14817.1	LINRRTPRVDGQPMYGMDAQVEEVRVFEGTEELPGDPDMMRYVDRYGQLQTKML	354
AEC14818.1	LINRRTPRVDGQPMYGMDAQVEEVRVFEGTEELPGDPDMMRYVDRYGQLQTKML	354
AEC14819.1	LINRRTPRVDGQPMYGMDAQVEEVRVFEGTEELPGDPDMMRYVDRYGQLQTKML	354
AEC14820.1	LINRRTPRVDGQPMYGMDAQVEEVRVFEGTEELPGDPDMMRYVDRYGQLQTKML	354
AEC14822.1	LINRRTPRVDGQPMYGMDAQVEEVRVFEGTEELPGDPDMMRYVDRYGQLQTKML	354
AEC14823.1	LINRRTPRVDGQPMYGMDAQVEEVRVFEGTEELPGDPDMMRYVDRYGQLQTKML	354
AEC14824.1	LINRRTPRVDGQPMYGMDAQVEEVRVFEGTEELPGDPDMMRYVDRYGQLQTKML	354
AEC14825.1	LINRRTPRVDGQPMYGMDAQVEEVRVFEGTEELPGDPDMMRYVDRYGQLQTKML	354
AEC14826.1	LINRRTPRVDGQPMYGMDAQVEEVRVFEGTEELPGDPDMMRYVDRYGQLQTKML	354
AEC14827.1	LINRRTPRVDGQPMYGMDAQVEEVRVFEGTEELPGDPDMMRYVDRYGQLQTKML	354
AEC14828.1	LINRRTPRVDGQPMYGMDAQVEEVRVFEGTEELPGDPDMMRYVDRYGQLQTKML	354
AEC14829.1	LINRRTPRVDGQPMYGMDAQVEEVRVFEGTEELPGDPDMMRYVDRYGQLQTKML	354
AEC14830.1	LINRRTPRVDGQPMYGMDAQVEEVRVFEGTEELPGDPDMMRYVDRYGQLQTKML	354
AEC14831.1	LINRRTPRVDGQPMYGMDAQVEEVRVFEGTEELPGDPDMMRYVDRYGQLQTKML	354
AEC14832.1	LINRRTPRVDGQPMYGMDAQVEEVRVFEGTEELPGDPDMMRYVDRYGQLQTKML	354
AEC14833.1	LINRRTPRVDGQPMYGMDAQVEEVRVFEGTEELPGDPDMMRYVDRYGQLQTKML	354
AEC14834.1	LINRRTPRVDGQPMYGMDAQVEEVRVFEGTEELPGDPDMMRYVDRYGQLQTKML	354
AEC14835.1	LINRRTPRVDGQPMYGMDAQVEEVRVFEGTEELPGDPDMMRYVDRYGQLQTKML	354
AEC14836.1	LINRRTPRVDGQPMYGMDAQVEEVRVFEGTEELPGDPDMMRYVDRYGQLQTKML	354
AEC14837.1	LINRRTPRVDGQPMYGMDAQVEEVRVFEGTEELPGDPDMMRYVDRYGQLQTKML	354
AEC14838.1	LINRRTPRVDGQPMYGMDAQVEEVRVFEGTEELPGDPDMMRYVDRYGQLQTKML	354
AEC14839.1	LINRRTPRVDGQPMYGMDAQVEEVRVFEGTEELPGDPDMMRYVDRYGQLQTKML	354
AEC14840.1	LINRRTPRVDGQPMYGMDAQVEEVRVFEGTEELPGDPDMMRYVDRYGQLQTKML	354
AEC14841.1	LINRRTPRVDGQPMYGMDAQVEEVRVFEGTEELPGDPDMMRYVDRYGQLQTKML	354
AEC14842.1	LINRRTPRVDGQPMYGMDAQVEEVRVFEGTEELPGDPDMMRYVDRYGQLQTKML	354
AEC14843.1	LINRRTPRVDGQPMYGMDAQVEEVRVFEGTEELPGDPDMMRYVDRYGQLQTKML	354
AEC14844.1	LINRRTPRVDGQPMYGMDAQVEEVRVFEGTEELPGDPDMMRYVDRYGQLQTKML	354

AEC14845.1	LINRRTPRVDGQPMYGMDAQVEEVRVFEGTEELPGDPDMMRYVDRYGQLQTKML	354
AEC14846.1	LINRRTPRVDGQPMYGMDAQVEEVRVFEGTEELPGDPDMMRYVDRYGQLQTKML	354
AEC14847.1	LINRRTPRVDGQPMYGMDAQVEEVRVFEGTEELPGDPDMMRYVDRYGQLQTKML	354
AEC14848.1	LINRRTPRVDGQPMYGMDAQVEEVRVFEGTEELPGDPDMMRYVDRYGQLQTKML	354
AEC14849.1	LINRRTPRVDGQPMYGMDAQVEEVRVFEGTEELPGDPDMMRYVDRYGQLQTKML	354
AEC14850.1	LINRRTPRVDGQPMYGMDAQVEEVRVFEGTEELPGDPDMMRYVDRYGQLQTKML	354
AEC14851.1	LINRRTPRVDGQPMYGMDAQVEEVRVFEGTEELPGDPDMMRYVDRYGQLQTKML	354
AEC14852.1	LINRRTPRVDGQPMYGMDAQVEEVRVFEGTEELPGDPDMMRYVDRYGQLQTKML	354
AEC14853.1	LINRRTPRVDGQPMYGMDAQVEEVRVFEGTEELPGDPDMMRYVDRYGQLQTKML	354
AEC14854.1	LINRRTPRVDGQPMYGMDAQVEEVRVFEGTEELPGDPDMMRYVDRYGQLQTKML	354
AEC14855.1	LINRRTPRVDGQPMYGMDAQVEEVRVFEGTEELPGDPDMMRYVDRYGQLQTKML	354
AEC14856.1	LINRRTPRVDGQPMYGMDAQVEEVRVFEGTEELPGDPDMMRYVDRYGQLQTKML	354
AEC14857.1	LINRRTPRVDGQPMYGMDAQVEEVRVFEGTEELPGDPDMMRYVDRYGQLQTKML	354
AEC14858.1	LINRRTPRVDGQPMYGMDAQVEEVRVFEGTEELPGDPDMMRYVDRYGQLQTKML	354
AEC14859.1	LINRRTPRVDGQPMYGMDAQVEEVRVFEGTEELPGDPDMMRYVDRYGQLQTKML	354
AEC14862.1	LINRRTPRVDGQPMYGMDAQVEEVRVFEGTEELPGDPDMMRYVDRYGQLQTKML	354
AEC14863.1	LINRRTPRVDGQPMYGMDAQVEEVRVFEGTEELPGDPDMMRYVDRYGQLQTKML	354
BAD21289.1	LINRRTPRVDGQPMYGMDAQVEEVRVFEGTEELPGDPDMMRYVDRYGQLQTKML	354
BAD21295.1	LINRRTPRVDGQPMYGMDAQVEEVRVFEGTEELPGDPDMMRYVDRYGQLQTKML	354
BAB93044.1	LINRRTPRVDGQPMYGMDAQVEEVRVFEGTEELPGDPDMMRYVDRYGQLQTKML	354
BAB93104.1	LINRRTPRVDGQPMYGMDAQVEEVRVFEGTEELPGDPDMMRYVDRYGQLQTKML	354
AAK70252.1	LINRRTPRVDGQPMYGMDAQVEEVRVFEGTEELPGDPDMMRYVDRYGQLQTKML	354
BAB68959.1	LINRRTPRVDGQPMYGMDAQVEEVRVFEGTEELPGDPDMMRYVDRYGQLQTKML	354
BAD21229.1	LINRRTPRVDGQPMYGMDAQVEEVRVFEGTEELPGDPDMMRYVDRYGQLQTKML	354
QBX89123.1	LINRRTPRVDGQPMYGMDAQIEEVRVFEGTEQLPGDPDLMRYVDRYGQLQTKML	354
BAE02908.1	LINRRTPRVDGQPMYGMDAQIEEVRVFEGTEQLPGDPDMMRYVDRYGQLQTKML	354
AAR89259.1	LINRRTPRVDGQPMYGMDAQIEEVRVFEGTEQLPGDPDMMRYVDRYGQLQTKML	354
AAR12957.1	LINRRTPRVDGQPMYGMDAQIEEVRVFEGTEQLPGDPDMMRYVDRYGQLQTKML	354
AAR13077.1	LINRRTPRVDGQPMYGMDAQIEEVRVFEGTEQLPGDPDMMRYVDRYGQLQTKML	354
BCU41036.1	LINRRTPRVDGQPMYGMDAQVEEVRVFEGTEELPGDPDMMRYVDRYGQLQTKML	354
BAE94726.1	LINRRTPRVDGQPMYGMDAQVEEVRVFEGTEELPGDPDMMRYVDRYGQLQTKML	354
AAB94036.1	LINRRTPRVDGQPMYGMDAQVEEVRVFEGTEELPGDPDMMRYVDRYGQLQTKML	354
AAK28478.1	LINRRTPRVDGQPMYGMDAQVEEVRVFEGTEELPGDPDMMRYVDRYGQLQTKML	354
BAB93008.1	LINRRTPRVDGQPMYGMDAQVEEVRVFEGTEELPGDPDMMRYVDKYGQLQTKML	354
AAM69579.1	LINRRTPRVDGQPMYGMDAQVEEVRVFEGTEELPGDPDMMRYVDRYGQLQTKML	354
AAM69584.1	LINRRTPRVDGQPMYGMDAQVEEVRVFEGTEELPGDPDMMRYVDRYGQLQTKML	354
AAM69596.1	LINRRTPRVDGQPMYGMDAQVEEVRVFEGTEELPGDPDMMRYVDRYGQLQTKML	354
AAM69602.1	LINRRTPRVDGQPMYGMDAQVEEVRVFEGTEELPGDPDMMRYVDRYGQLQTKML	354
AAM69608.1	LINRRTPRVDGQPMYGMDAQVEEVRVFEGTEELPGDPDMMRYVDRYGQLQTKML	354
AAK70312.1	LINRRTPRVDGQPMYGMDAQVEEVRVFEGTEELPGDPDMMRYVDRYGQLQTKML	354
WLD48064.1	LINRRTPRVDGQPMYGMDAQVEEVRVFEGTEELPGDPDMMRYVDRYGQLQTKML	354
WLD48100.1	LINRRTPRVDGQPMYGMDAQVEEVRVFEGTEELPGDPDMMRYVDRYGQLQTKML	354
UQK62655.1	LINRRTPRVDGQPMYGMDAQVEEVRVFEGTEELPGDPDMMRYVDRYGQLQTKML	354
QTJ15180.1	LINRRTPRVDGQPMYGMDAQVEEVRVFEGTEELPGDPDMMRYVDRYGQLQTKML	354
QTJ15204.1	LINRRTPRVDGQPMYGMDAQVEEVRVFEGTEELPGDPDMMRYVDRYGQLQTKML	354
ASV51784.1	LINRRTPRVDGQPMYGMDAQVEEVRVFEGTEELPGDPDMMRYVDRYGQLQTKML	354
ASV51790.1	LINRRTPRVDGQPMYGMDAQVEEVRVFEGTEELPGDPDMMRYVDRYGQLQTKML	354
ASV51898.1	LINRRTPRVDGQPMYGMDAQVEEVRVFEGTEELPGDPDMMRYVDRYGQLQTKML	354
ASV51904.1	LINRRTPRVDGQPMYGMDAQVEEVRVFEGTEELPGDPDMMRYVDRYGQLQTKML	354
ASV51910.1	LINRRTPRVDGQPMYGMDAQVEEVRVFEGTEELPGDPDMMRYVDRYGQLQTKML	354
ASV51916.1	LINRRTPRVDGQPMYGMDAQVEEVRVFEGTEELPGDPDMMRYVDRYGQLQTKML	354
CCW72490.1	LINRRTPRVDGQPMYGMDAQVEEVRVFEGTEELPGDPDMMRYVDRYGQLQTKML	354
CCW72495.1	LINRRTPRVDGQPMYGMDAQVEEVRVFEGTEELPGDPDMMRYVDRYGQLQTKML	354
CCW72497.1	LINRRTPRVDGQPMYGMDAQVEEVRVFEGTEELPGDPDMMRYVDRYGQLQTKML	354
CCW72503.1	LINRRTPRVDGQPMYGMDAQVEEVRVFEGTEELPGDPDMMRYVDRYGQLQTKML	354
CCW72505.1	LINRRTPRVDGQPMYGMDAQVEEVRVFEGTEELPGDPDMMRYVDRYGQLQTKML	354
CCW72507.1	LINRRTPRVDGQPMYGMDAQVEEVRVFEGTEELPGDPDMMRYVDRYGQLQTKML	354
CCW72512.1	LINRRTPRVDGQPMYGMDAQVEEVRVFEGTEELPGDPDMMRYVDRYGQLQTKML	354
CCW72515.1	LINRRTPRVDGQPMYGMDAQVEEVRVFEGTEELPGDPDMMRYVDRYGQLQTKML	354
BAB68881.1	LINRRTPRVDGQPMYGMDAQVEEVRVFEGTEELPGDPDMMRYVDRYGQLQTKML	354
BAB68887.1	LINRRTPRVDGQPMYGMDAQVEEVRVFEGTEELPGDPDMMRYVDRYGQLQTKML	354
AAK70294.1	LINRRTPRVDGQPMYGMDAQVEEVRVFEGTEELPGDPDMMRYVDRYGQLQTKML	354
WLD48094.1	LINRRTPRVDGQPMYGMDAQVEEVRVFEGTEELPGDPDMMRYVDRYGQLQTKML	354
CCW72509.1	LINRRTPRVDGQPMYGMDAQVEEVRVFEGTEELPGDPDMMRYVDRYGQLQTKML	354

BAB68893.1	LINRRTPRVDGQPMYGMDAQVEEVRVFEGTEELPGDPDMMRYVDRYGQLQTKML	354
QTJ15216.1	LINRRTPRVDGQPMYGMDAQVEEVRVFEGTEELPGDPDMMRYVDRYGQLQTKML	354
CCW72493.1	LINRRTPRVDGQPMYGMDAQVEEVRVFEGTEELPGDPDMMRYVDRYGQLQTKML	354
CCW72500.1	LINRRTPRVDGQPMYGMDAQVEEVRVFEGTEELPGDPDMMRYVDRYGQLQTKML	354
CCW72506.1	LINRRTPRVDGQPMYGMDAQVEEVRVFEGTEELPGDPDMMRYVDRYGQLQTKML	354
CCW72520.1	LINRRTPRVDGQPMYGMDAQVEEVRVFEGTEELPGDPDMMRYVDRYGQLQTKML	354
BAB68899.1	LINRRTPRVDGQPMYGMDAQVEEVRVFEGTEELPGDPDMMRYVDRYGQLQTKML	354
BAB68905.1	LINRRTPRVDGQPMYGMDAQVEEVRVFEGTEELPGDPDMMRYVDRYGQLQTKML	354
AAK70300.1	LINRRTPRVDGQPMYGMDAQVEEVRVFEGTEELPGDPDMMRYVDRYGQLQTKML	354
AYM55579.1	LINRRTPRVDGQPMYGMDAQIEEVRVFEGTEELPGDPDMMRYVDRYGQLQTKML	354
AYM55580.1	LINRRTPRVDGQPMYGMDAQIEEVRVFEGTEELPGDPDMRRYVDRYGQLQTKML	354
AYM55582.1	LINRRTPRVDGQPMYGVDAQIEEVRVFEGTEELPGDPDMMRYVDRYGQLQTKML	354
AYM55583.1	LINRRTPRVDGQPMYGMDAQIEEVRVFEGTEELPGDPDMMRYVDRYGQLQTKML	354
AYM55584.1	LINRRTPRVDGQPMYGMDAQIEEVRVFEGTEELPGDPDMMRYVDRYGQLQTKML	354
AYM55590.1	LINRRTPRVDGQPMYGMDAQIEEVRVFEGTEELPGDPDMMRYVDRYRQLQTKML	354
AYM55596.1	LINRRTPRVDGQPMYGMDAQIEEVRVFEGTEELPGDPDMMRYVDKYGQLQTKML	354
BAE45366.1	LINRRTPRVDGQPMYGMDAQIEEVRVFEGTEELPGDPDMMRYVDKYGQLQTKML	354
AAG30857.1	LINRRTPRVDGQPMYGMDAQIEEVRVFEGTEELPGDPDMMRYVDKYGQLQTKML	354
BAH36774.1	LINRRTPRVDGQPMYGMDAQIEEVRVFEGTEELPGDPDMMRYVDRYGQLQTKML	354
BAH36780.1	LINRRTPRVDGQPMYGMDAQIEEVRVFEGTEELPGDPDMMRYVDRYGQLQTKML	354
BAH36822.1	LINRRTPRVDGQPMYGMDAQIEEVRVFEGTEELPGDPDMMRYVDRYGQLQTKML	354
BAH36834.1	LINRRTPRVDGQPMYGMDAQIEEVRVFEGTEELPGDPDMMRYVDRYGQLQTKML	354
BAH36840.1	LINRRTPRVDGQPMYGMDAQIEEVRVFEGTEELPGDPDMMRYVDRYGQLQTKML	354
BAH36846.1	LINRRTPRVDGQPMYGMDAQIEEVRVFEGTEELPGDPDMMRYVDRYGQLQTKML	354
BAH36816.1	LINRRTPRVDGQPMYGMDAQIEEVRVFEGTEELPGDPDMMRYVDRYGQLQTKML	354
BAE45444.1	LINRRTPRVDGQPMYGMDAQIEEVRVFEGTEELPGDPDMMRYVDRYGQLQTKML	354
AAT09819.1	LINRRTPRVDGQPMYGMDAQIEEVRVFEGTEELPGDPDMMRYVDRYGQLQTKML	354
BAC03029.1	LINRRTPRVDGQPMYGMDAQIEEVRVFEGTEELPGDPDMMRYVDRYGQLQTKML	354
BAC03095.1	LINRRTPRVDGQPMYGMDAQIEEVRVFEGTEELPGDPDMMRYVDRYGQLQTKML	354
AAM69560.1	LINRRTPRVDGQPMYGMDAQIEEVRVFEGTEELPGDPDMMRYVDRYGQLQTKML	354
AAM69566.1	LINRRTPRVDGQPMYGMDAQIEEVRVFEGTEELPGDPDMMRYVDRYGQLQTKML	354
AAM69572.1	LINRRTPKVDGQPMYGMDAQIEEVRVFEGTEELPGDPDMMRYVDRYGQLQTKML	354
AAB84311.1	LINRRTPKVDGQPMYGMDAQIEEVRVFEGTEELPGDPDMMRYVDRYGQLQTKML	354
BAD06150.1	LINRRTPRVDGQPMYGMDAQIEEVRVFEGTEELPGDPDMMRYVDRYGQLQTKML	354
AYM55581.1	LINRRTPRVDGQPMYGMDAQIEEVRVFEGTEELPGDPDMMRYVDRYGQLQTKML	354
AYM55588.1	LINRRTPRVDGQPMYGMDAQIEEVRVFEGTEELPGDPDMMRYVDRYGQLQTKML	354
AYM55589.1	LINRRTPRVDGQPMYGMDAQIEEVRVFEGTEELPGDPDMMRYVDRYGQLQTKML	354
AYM55591.1	LINRRTPRVDGQPMYGMDAQIEEVRVFEGTEELPGDPDMMRYVDRYGQLQTKML	354
AYM55592.1	LINRRTPRVDGQPMYGMDAQIEEVRVFEGTEELPGDPDMMRYVDRYGQLQTKML	354
AYM55593.1	LINRRTPRVDGQPMYGMDAQIEEVRVFEGTEELPGDPDMMRYVDRYGQLQTKML	354
AYM55595.1	LINRRTPRVDGQPMYGMDAQIEEVRVFEGTEELPGDPDMMRYVDRYGQLQTKML	354
AYM55597.1	LINRRTPRVDGQPMYGMDAQIEEVRVFEGTEELPGDPDMMRYVDRYGQLQTKML	354
AYM55598.1	LINRRTPRVDGQPMYGMDAQIEEVRVFEGTEELPGDPDMMRYVDRYGQLQTKML	354
AYM55599.1	LINRRTPRVDGQPMYGMDAQIEEVRVFEGTEELPGDPDMMRYVDRYGQLQTKML	354
AYM55600.1	LINRRTPRVDGQPMYGMDAQIEEVRVFEGTEELPGDPDMMRYVDRYGQLQTKML	354
AYM55601.1	LINRRTPRVDGQPMYGMDAQIEEVRVFEGTEELPGDPDMMRYVDRYGQLQTKML	354
AYM55602.1	LINRRTPRVDGQPMYGMDAQIEEVRVFEGTEELPGDPDMMRYVDRYGQLQTKML	354
AYM55603.1	LINRRTPRVDGQPMYGMDAQIEEVRVFEGTEELPGDPDMMRYVDRYGQLQTKML	354
CCW72483.1	LINRRTPRVDGQPMYGMDAQIEEVRVFEGTEELPGDPDMMRYVDRYGQLQTKML	354
CCW72485.1	LINRRTPRVDGQPMYGMDAQIEEVRVFEGTEELPGDPDMMRYVDRYGQLQTKML	354
AFR56669.1	LINRRTPRVDGQPMYGMDAQIEEVRVFEGTEELPGDPDMMRYVDRYGQLQTKML	354
BAH36768.1	LINRRTPRVDGQPMYGMDAQIEEVRVFEGTEELPGDPDMMRYVDRYGQLQTKML	354
BAH36786.1	LINRRTPRVDGQPMYGMDAQIEEVRVFEGTEELPGDPDMMRYVDRYGQLQTKML	354
BAH36792.1	LINRRTPRVDGQPMYGMDAQIEEVRVFEGTEELPGDPDMMRYVDRYGQLQTKML	354
BAH36798.1	LINRRTPRVDGQPMYGMDAQIEEVRVFEGTEELPGDPDMMRYVDRYGQLQTKML	354
BAH36804.1	LINRRTPRVDGQPMYGMDAQIEEVRVFEGTEELPGDPDMMRYVDRYGQLQTKML	354
BAH36810.1	LINRRTPRVDGQPMYGMDAQIEEVRVFEGTEELPGDPDMMRYVDRYGQLQTKML	354
BAH36828.1	LINRRTPRVDGQPMYGMDAQIEEVRVFEGTEELPGDPDMMRYVDRYGQLQTKML	354
BAH36852.1	LINRRTPRVDGQPMYGMDAQIEEVRVFEGTEELPGDPDMMRYVDRYGQLQTKML	354
BAH36858.1	LINRRTPRVDGQPMYGMDAQIEEVRVFEGTEELPGDPDMMRYVDRYGQLQTKML	354
BAE75814.1	LINRRTPRVDGQPMYGMDAQIEEVRVFEGTEELPGDPDMMRYVDRYGQLQTKML	354
BAE75820.1	LINRRTPRVDGQPMYGMDAQIEEVRVFEGTEELPGDPDMMRYVDRYGQLQTKML	354
BAE75826.1	LINRRTPRVDGQPMYGMDAQIEEVRVFEGTEELPGDPDMMRYVDRYGQLQTKML	354
BAE75832.1	LINRRTPRVDGQPMYGMDAQIEEVRVFEGTEELPGDPDMMRYVDRYGQLQTKML	354

```
BAE75838.1        LINRRTPRVDGQPMYGMDAQIEEVRVFEGTEELPGDPDMMRYVDRYGQLQTKML  354
BAE45360.1        LINRRTPRVDGQPMYGMDAQIEEVRVFEGTEELPGDPDMMRYVDRYGQLQTKML  354
BAE45372.1        LINRRTPRVDGQPMYGMDAQIEEVRVFEGTEELPGDPDMMRYVDRYGQLQTKML  354
BAE45378.1        LINRRTPRVDGQPMYGMDAQIEEVRVFEGTEELPGDPDMMRYVDRYGQLQTKML  354
BAE45384.1        LINRRTPRVDGQPMYGMDAQIEEVRVFEGTEELPGDPDMMRYVDRYGQLQTKML  354
BAE45390.1        LINRRTPRVDGQPMYGMDAQIEEVRVFEGTEELPGDPDMMRYVDRYGQLQTKML  354
BAE45396.1        LINRRTPRVDGQPMYGMDAQIEEVRVFEGTEELPGDPDMMRYVDRYGQLQTKML  354
BAE45402.1        LINRRTPRVDGQPMYGMDAQIEEVRVFEGTEELPGDPDMMRYVDRYGQLQTKML  354
BAE45408.1        LINRRTPRVDGQPMYGMDAQIEEVRVFEGTEELPGDPDMMRYVDRYGQLQTKML  354
BAE45414.1        LINRRTPRVDGQPMYGMDAQIEEVRVFEGTEELPGDPDMMRYVDRYGQLQTKML  354
BAE45420.1        LINRRTPRVDGQPMYGMDAQIEEVRVFEGTEELPGDPDMMRYVDRYGQLQTKML  354
BAE45426.1        LINRRTPRVDGQPMYGMDAQIEEVRVFEGTEELPGDPDMMRYVDRYGQLQTKML  354
BAE45432.1        LINRRTPRVDGQPMYGMDAQIEEVRVFEGTEELPGDPDMMRYVDRYGQLQTKML  354
BAE45438.1        LINRRTPRVDGQPMYGMDAQIEEVRVFEGTEELPGDPDMMRYVDRYGQLQTKML  354
BAD06018.1        LINRRTPRVDGQPMYGMDAQIEEVRVFEGTEELPGDPDMMRYVDRYGQLQTKML  354
BAD06024.1        LINRRTPRVDGQPMYGMDAQIEEVRVFEGTEELPGDPDMMRYVDRYGQLQTKML  354
BAD06030.1        LINRRTPRVDGQPMYGMDAQIEEVRVFEGTEELPGDPDMMRYVDRYGQLQTKML  354
BAD06042.1        LINRRTPRVDGQPMYGMDAQIEEVRVFEGTEELPGDPDMMRYVDRYGQLQTKML  354
BAD06048.1        LINRRTPRVDGQPMYGMDAQIEEVRVFEGTEELPGDPDMMRYVDRYGQLQTKML  354
BAD06054.1        LINRRTPRVDGQPMYGMDAQIEEVRVFEGTEELPGDPDMMRYVDRYGQLQTKML  354
BAD06084.1        LINRRTPRVDGQPMYGMDAQIEEVRVFEGTEELPGDPDMMRYVDRYGQLQTKML  354
BAD06090.1        LINRRTPRVDGQPMYGMDAQIEEVRVFEGTEELPGDPDMMRYVDRYGQLQTKML  354
BAD06096.1        LINRRTPRVDGQPMYGMDAQIEEVRVFEGTEELPGDPDMMRYVDRYGQLQTKML  354
BAD06102.1        LINRRTPRVDGQPMYGMDAQIEEVRVFEGTEELPGDPDMMRYVDRYGQLQTKML  354
BAD06108.1        LINRRTPRVDGQPMYGMDAQIEEVRVFEGTEELPGDPDMMRYVDRYGQLQTKML  354
BAD06120.1        LINRRTPRVDGQPMYGMDAQIEEVRVFEGTEELPGDPDMMRYVDRYGQLQTKML  354
BAC15695.1        LINRRTPRVDGQPMYGMDAQIEEVRVFEGTEELPGDPDMMRYVDRYGQLQTKML  354
BAC15725.1        LINRRTPRVDGQPMYGMDAQIEEVRVFEGTEELPGDPDMMRYVDRYGQLQTKML  354
BAC02951.1        LINRRTPRVDGQPMYGMDAQIEEVRVFEGTEELPGDPDMMRYVDRYGQLQTKML  354
BAC02957.1        LINRRTPRVDGQPMYGMDAQIEEVRVFEGTEELPGDPDMMRYVDRYGQLQTKML  354
BAC02963.1        LINRRTPRVDGQPMYGMDAQIEEVRVFEGTEELPGDPDMMRYVDRYGQLQTKML  354
BAC02969.1        LINRRTPRVDGQPMYGMDAQIEEVRVFEGTEELPGDPDMMRYVDRYGQLQTKML  354
BAC02975.1        LINRRTPRVDGQPMYGMDAQIEEVRVFEGTEELPGDPDMMRYVDRYGQLQTKML  354
BAC02981.1        LINRRTPRVDGQPMYGMDAQIEEVRVFEGTEELPGDPDMMRYVDRYGQLQTKML  354
BAC02987.1        LINRRTPRVDGQPMYGMDAQIEEVRVFEGTEELPGDPDMMRYVDRYGQLQTKML  354
BAC02993.1        LINRRTPRVDGQPMYGMDAQIEEVRVFEGTEELPGDPDMMRYVDRYGQLQTKML  354
BAC02999.1        LINRRTPRVDGQPMYGMDAQIEEVRVFEGTEELPGDPDMMRYVDRYGQLQTKML  354
BAC03005.1        LINRRTPRVDGQPMYGMDAQIEEVRVFEGTEELPGDPDMMRYVDRYGQLQTKML  354
BAC03011.1        LINRRTPRVDGQPMYGMDAQIEEVRVFEGTEELPGDPDMMRYVDRYGQLQTKML  354
BAC03017.1        LINRRTPRVDGQPMYGMDAQIEEVRVFEGTEELPGDPDMMRYVDRYGQLQTKML  354
BAC03023.1        LINRRTPRVDGQPMYGMDAQIEEVRVFEGTEELPGDPDMMRYVDRYGQLQTKML  354
BAC03035.1        LINRRTPRVDGQPMYGMDAQIEEVRVFEGTEELPGDPDMMRYVDRYGQLQTKML  354
BAC03041.1        LINRRTPRVDGQPMYGMDAQIEEVRVFEGTEELPGDPDMMRYVDRYGQLQTKML  354
BAC03047.1        LINRRTPRVDGQPMYGMDAQIEEVRVFEGTEELPGDPDMMRYVDRYGQLQTKML  354
BAC03053.1        LINRRTPRVDGQPMYGMDAQIEEVRVFEGTEELPGDPDMMRYVDRYGQLQTKML  354
BAC03059.1        LINRRTPRVDGQPMYGMDAQIEEVRVFEGTEELPGDPDMMRYVDRYGQLQTKML  354
BAC03071.1        LINRRTPRVDGQPMYGMDAQIEEVRVFEGTEELPGDPDMMRYVDRYGQLQTKML  354
BAC03077.1        LINRRTPRVDGQPMYGMDAQIEEVRVFEGTEELPGDPDMMRYVDRYGQLQTKML  354
BAC03083.1        LINRRTPRVDGQPMYGMDAQIEEVRVFEGTEELPGDPDMMRYVDRYGQLQTKML  354
BAC03089.1        LINRRTPRVDGQPMYGMDAQIEEVRVFEGTEELPGDPDMMRYVDRYGQLQTKML  354
AAM69542.1        LINRRTPRVDGQPMYGMDAQIEEVRVFEGTEELPGDPDMMRYVDRYGQLQTKML  354
AAM69548.1        LINRRTPRVDGQPMYGMDAQIEEVRVFEGTEELPGDPDMMRYVDRYGQLQTKML  354
AAM69554.1        LINRRTPRVDGQPMYGMDAQIEEVRVFEGTEELPGDPDMMRYVDRYGQLQTKML  354
AAM69619.1        LINRRTPRVDGQPMYGMDAQIEEVRVFEGTEELPGDPDMMRYVDRYGQLQTKML  354
BAB68977.1        LINRRTPRVDGQPMYGMDAQIEEVRVFEGTEELPGDPDMMRYVDRYGQLQTKML  354
BAB68983.1        LINRRTPRVDGQPMYGMDAQIEEVRVFEGTEELPGDPDMMRYVDRYGQLQTKML  354
BAB68989.1        LINRRTPRVDGQPMYGMDAQIEEVRVFEGTEELPGDPDMMRYVDRYGQLQTKML  354
BAB68995.1        LINRRTPRVDGQPMYGMDAQIEEVRVFEGTEELPGDPDMMRYVDRYGQLQTKML  354
AAK98000.1        LINRRTPRVDGQPMYGMDAQIEEVRVFEGTEELPGDPDMMRYVDRYGQLQTKML  354
AAK98006.1        LINRRTPRVDGQPMYGMDAQIEEVRVFEGTEELPGDPDMMRYVDRYGQLQTKML  354
AAK98010.1        LINRRTPRVDGQPMYGMDAQIEEVRVFEGTEELPGDPDMMRYVDRYGQLQTKML  354
AAK98018.1        LINRRTPRVDGQPMYGMDAQIEEVRVFEGTEELPGDPDMMRYVDRYGQLQTKML  354
AAK98024.1        LINRRTPRVDGQPMYGMDAQIEEVRVFEGTEELPGDPDMMRYVDRYGQLQTKML  354
AAK98030.1        LINRRTPRVDGQPMYGMDAQIEEVRVFEGTEELPGDPDMMRYVDRYGQLQTKML  354
```

```
AAK98036.1        LINRRTPRVDGQPMYGMDAQIEEVRVFEGTEELPGDPDMMRYVDRYGQLQTKML  354
AAK70258.1        LINRRTPRVDGQPMYGMDAQIEEVRVFEGTEELPGDPDMMRYVDRYGQLQTKML  354
AAK70264.1        LINRRTPRVDGQPMYGMDAQIEEVRVFEGTEELPGDPDMMRYVDRYGQLQTKML  354
AAG53896.1        LINRRTPRVDGQPMYGMDAQIEEVRVFEGTEELPGDPDMMRYVDRYGQLQTKML  354
BAA01962.1        LINRRTPRVDGQPMYGMDAQIEEVRVFEGTEELPGDPDMMRYVDRYGQLQTKML  354
ALF06256.1        LINRRTPRVDGQPMYGMDAQVEEVRVFEGTEELPGDPDMMRYVDRYGQLQT---  351
BAD06060.1        LINRRTPRVDGQPMYGMDAQIEEVRVFEGTEELPGDPDMMRYVDRYGQLQTKML  354
BAD06072.1        LINRRTPRVDGQPMYGMDAQIEEVRVFEGTEELPGDPDMMRYVDRYGQLQTKML  354
BAD06156.1        LINRRTPRVDGQPMYGMDAQIEEVRVFEGTEELPGDPDMMRYVDRYGQLQTKML  354
BAD06174.1        LINRRTPRVDGQPMYGMDAQIEEVRVFEGTEELPGDPDMMRYVDRYGQLQTKML  354
AAN85449.1        LINRRTPRVDGQPMYGMDAQIEEVRVFEGTEELPGDPDMMRYVDRYGQLQTKML  354
AAN85455.1        LINRRTPRVDGQPMYGMDAQIEEVRVFEGTEELPGDPDMMRYVDRYGQLQTKML  354
AAK98042.1        LINRRTPRVDGQPMYGMDAQIEEVRVFEGTEELPGDPDMMRYVDRYGQLQTKML  354
BAD06036.1        LINRRTPRVDGQPMYGMDAQIEEVRVFEGTEELPGDPDMMRYVDRYGQLQTKML  354
BCP65063.1        LINRRTPRVDGQPMYGMDAQVEEVRVFEGTEELPGDPDMMRYVDRYGQLQTKML  354
BCP56477.1        LINRRTPRVDGQPMYGMDAQVEEVRVFEGTEELPGDPDMMRYVDRYGQLQTKML  354
BAV69373.1        LINRRTPRVDGQPMYGMDAQVEEVRVFEGTEELPGDPDMMRYVDRYGQLQTKML  354
BAE00153.1        LINRRTPRVDGQPMYGMDAQVEEVRVFEGTEELPGDPDMMRYVDRYGQLQTKML  354
BAC22906.1        LINRRTPRVDGQPMYGMDAQVEEVRVFEGTEELPGDPDMMRYVDRYGQLQTKML  354
BAA05639.1        LINRRTPRVDGQPMYGMDAQVEEVRVFEGTEELPGDPDMMRYVDRYGQLQTKML  354
BAB11728.1        LINRRTPRVDGQPMYGMDAQVEEVRVFEGTEELPGDPDMMRYVDRYGQLQTKML  354
BAA05637.1        LINRRTPRVDGQPMYGMDAQVEEVRVFEGTEELPGDPDMMRYVDRYGQLQTKML  354
BAA01969.1        LINRRTPRVDGQPMYGMDAQVEEVRVFEGTEELPGDPDMMRYVDRYGQLQTKML  354
BAE00159.1        LINRRTPRVDGQPMYGMDAQVEEVRVFEGTEELPGDPDMMRYVDRYGQLQTKML  354
BAE00165.1        LINRRTPRVDGQPMYGMDAQVEEVRVFEGTEELPGDPDMMRYVDRYGQLQTKML  354
BAE00171.1        LINRRTPRVDGQPMYGMDAQVEEVRVFEGTEELPGDPDMMRYVDRYGQLQTKML  354
ALF06268.1        LINRRTPRVDGQPMYGMDAQVEEVRVFEGTEELPGDPDMMRYVDRYGQLQT---  351
AYM55586.1        LINRRTPRVDGQPMYVLDAQIEEVRVCEGTEELPGDPDMMRYVDRYGQLQTKML  354
AYM55585.1        LINRRTPRVDGQPMYGMDAQIEEVRVFEGTEELPGDPDMMRYVDRYGQLQTKML  354
AYM55587.1        LINRRTPRVDGQPMYGMDAQIEEVRVFEGTEGIPGDPDMMRYVDRYGQLQTKML  354
AYM55594.1        LINRRTPRVDGQPMYGMYAQIEEVRVFEGTEELPGDPDMMRYVDRYGQLQTKML  354
QBQ65470.1        LINRRTPRVDGQPMYGMDAQVEEVRVFEGTEKLPGDPDLMRYVDRYGQLQTKML  354
CCW72501.1        LINRRTPRVDGQPMYGMDXQVEEVRXFEGTEELPGDPDMMRYVDRYGQLQTKML  354
CCW72524.1        LINRRTPRVDGQPMYGMDAQVEEVRXFEGTEELPGDPDMMRYVDRYGQLQTKML  354
QWT77116.1        LINRRTPRVDGQPMYGMDAQVEEVRVFEGTEELPGDPDMMRYVDRYGQLQTKML  354
WLD48046.1        LINRRTPRVDGQPMYGMDAQVEEVRVFEGTEELPGDPDMMRYVDRYGQLQTKML  354
WLD48052.1        LINRRTPRVDGQPMYGMDAQVEEVRVFEGTEELPGDPDMMRYVDRYGQLQTKML  354
AAK70288.1        LINRRTPRVDGQPMYGMDAQVEEVRVFEGTEELPGDPDMMRYVDRYGQLQTKML  354
AAK70270.1        LINRRTPRVDGQPMYGMDAQVEEVRVFEGTEELPGDPDMMRYVDRYGQLQTKML  354
BAD21277.2        LINRRTPRVDGQPMYGMDAQVEEVRVFEGTEELPGDPDMMRYVDRYGQLQTKML  354
CCW72498.1        LINRRTPRVDGQPMYGMDAQVEEVRVFEGTEELPGDPDMMRYVDRYGQLQTKML  354
UPN63085.1        LINRRTPRVDGQPMYGMDAQVEEVRVFEGTEELPGDPDMMRYVDRYGQLQTKML  354
UPN63090.1        LINRRTPRVDGQPMYGMDAQVEEVRVFEGTEELPGDPDMMRYVDRYGQLQTKML  354
QWT77134.1        LINRRTPRVDGQPMYGMDAQVEEVRVFEGTEELPGDPDMMRYVDRYGQLQTKML  354
WLD48070.1        LINRRTPRVDGQPMYGMDAQVEEVRVFEGTEELPGDPDMMRYVDRYGQLQTKML  354
AAK70282.1        LINRRTPRVDGQPMYGMDAQVEEVRVFEGTEELPGDPDMMRYVDRYGQLQTKML  354
WLD48058.1        LINRRTPRVDGQPMYGMDAQVEEVRVFEGTEELPGDPDMMRYVDRYGQLQTKML  354
WLD48076.1        LINRRTPRVDGQPMYGMDAQVEEVRVFEGTEELPGDPDMMRYVDRYGQLQTKML  354
UQK62667.1        LINRRTPRVDGQPMYGMDAQVEEVRVFEGTEELPGDPDMMRYVDRYGQLQTKML  354
CCW72480.1        LINRRTPRVDGQPMYGMDAQVEEVRVFEGTEELPGDPDMMRYVDRYGQLQTKML  354
CCW72513.1        LINRRTPRVDGQPMYGMDAQVEEVRVFEGTEELPGDPDMMRYVDRYGQLQTKML  354
AAM69614.1        LINRRTPRVDGQPMYGMDAQVEEVRVFEGTEELPGDPDMMRYVDRYGQLQTKML  354
AAK70318.1        LINRRTPRVDGQPMYGMDAQVEEVRVFEGTEELPGDPDMMRYVDRYGQLQTKML  354
AAK70324.1        LINRRTPRVDGQPMYGMDAQVEEVRVFEGTEELPGDPDMMRYVDRYGQLQTKML  354
AAT09837.1        LINRRTPRVDGQPMYGMDAQIEEVRVFEGTEQLPGDPDMMRYVDRYGQLQTKML  354
AAT09831.1        LINRRTPRVDGQPMYGMDAQIEEVRVFEGTEQLPGDPDMMRYVDRYGQLQTKML  354
AAT09825.1        LINRRTPRVDGQPMYGMDAQIEEVRVFEGTEQLPGDPDMMRYVDRYGQLQTKML  354
AAR89271.1        LINRRTPRVDGQPMYGMDAQIEEVRVFEGTEQLPGDPDMMRYVDRYGQLQTKML  354
AAR89211.1        LINRRTPRVDGQPMYGMDAQIEEVRVFEGTEQLPGDPDMMRYVDRYGQLQTKML  354
BAE02956.1        LINRRTPRVDGQPMYGMDAQIEEVRVFEGTEQLPGDPDMMRYVDRYGQLQTKML  354
ACF75876.1        LINRRTPRVDGQPMYGMDAQIEEVRVFEGTEQLPGDPDMMRYVDRYGQLQTKML  354
QBM11924.1        LINRRTPRVDGQPMYGMDAQIKKVRVFEGTEQLPGDPDMMRYVDKYGQLQTKML  354
QBM11858.1        LINRRTPRVDGQPMYGMDAQIEEVKVFEGTEELPGDPDLMRYVDQYGQLQTKML  354
QBQ04203.1        LINRRTPRVDGQPMYGMDAQIEKVRVFEGTEQLPGDPDMMRYVDKYGQLQTKML  354
```

QBQ65473.1	LINRRTPRVDGQPMYGMDAQIEEVRFFEGTEQLPGDPDMMRYVDQYGQLQTKML	354
QBQ65472.1	LINRRTPRVDGQPMYGMDAQIEEVRVFEGTEQLPGDPDMMRYVDRYGQLQTKML	354
QBM11920.1	LINRRTPRVDGQPMYGMDAQIEEVRVFEGTEQLPGDPDLMRYVDQYGQLQTKML	354
QAA95693.1	LINRRTPRVDGQPMYGMDAQIEEVRVFEGTEQLPGDPDLMRYVDQYGQLQTKML	354
QBX89122.1	LINRRTPRVDGQPMYGMDAQIEEVRVFEGTEQLPGDPDLMRYVDRYGQLQTKML	354
QID57946.1	LINRRTPRVDGQPMYGMDAQIEEVRVFEGTEQLPGDPDMMRYVDKYGQLQTKML	354
QID57952.1	LINRRTPRVDGQPMYGMDAQIEEVRVFEGTEQLPGDPDMMRYVDKYGQLQTKML	354
QID57958.1	LINRRTPRVDGQPMYGMDAQIEEVRVFEGTEQLPGDPDMMRYVDKYGQLQTKML	354
QID57964.1	LINRRTPRVDGQPMYGMDAQIEEVRVFEGTEQLPGDPDMMRYVDKYGQLQTKML	354
QID57970.1	LINRRTPRVDGQPMYGMDAQIEEVRVFEGTEQLPGDPDMMRYVDKYGQLQTKML	354
QID57976.1	LINRRTPRVDGQPMYGMDAQIEEVRVFEGTEQLPGDPDMMRYVDKYGQLQTKML	354
QCH41110.1	LINRRTPRVDGQPMYGMDAQIEEVRVFEGTEQLPGDPDMMRYVDKYGQLQTKML	354
QBX89120.1	LINRRTPRVDGQPMYGMDAQIEEVRVFEGTEQLPGDPDMMRYVDKYGQLQTKML	354
QBX89121.1	LINRRTPRVDGQPMYGMDAQIEEVRVFEGTEQLPGDPDMMRYVDKYGQLQTKML	354
QBX89124.1	LINRRTPRVDGQPMYGMDAQIEEVRVFEGTEQLPGDPDMMRYVDKYGQLQTKML	354
QBQ65471.1	LINRRTPRVDGQPMYGMDAQIEEVRVFEGTEQLPGDPDMMRYVDKYGQLQTKML	354
QBQ65474.1	LINRRTPRVDGQPMYGMDAQIEEVRVFEGTEQLPGDPDMMRYVDKYGQLQTKML	354
QBQ65476.1	LINRRTPRVDGQPMYGMDAQIEEVRVFEGTEQLPGDPDMMRYVDKYGQLQTKML	354
QBQ04201.1	LINRRTPRVDGQPMYGMDAQIEEVRVFEGTEQLPGDPDMMRYVDKYGQLQTKML	354
QBM11921.1	LINRRTPRVDGQPMYGMDAQIEEVRVFEGTEQLPGDPDMMRYVDKYGQLQTKML	354
QBM11922.1	LINRRTPRVDGQPMYGMDAQIEEVRVFEGTEQLPGDPDMMRYVDKYGQLQTKML	354
CDJ79845.1	LINRRTPRVDGQPMYGMDAQIEEVRVFEGTEQLPGDPDMMRYVDKYGQLQTKML	354
BAE02932.1	LINRRTPRVDGQPMYGMDAQIEEVRVFEGTEQLPGDPDMMRYVDKYGQLQTKML	354
BAE02938.1	LINRRTPRVDGQPMYGMDAQIEEVRVFEGTEQLPGDPDMMRYVDKYGQLQTKML	354
BAE02950.1	LINRRTPRVDGQPMYGMDAQIEEVRVFEGTEQLPGDPDMMRYVDKYGQLQTKML	354
BAE03004.1	LINRRTPRVDGQPMYGMDAQIEEVRVFEGTEQLPGDPDMMRYVDKYGQLQTKML	354
BAE03118.1	LINRRTPRVDGQPMYGMDAQIEEVRVFEGTEQLPGDPDMMRYVDKYGQLQTKML	354
BAE03124.1	LINRRTPRVDGQPMYGMDAQIEEVRVFEGTEQLPGDPDMMRYVDKYGQLQTKML	354
BAE03130.1	LINRRTPRVDGQPMYGMDAQIEEVRVFEGTEQLPGDPDMMRYVDKYGQLQTKML	354
BAE03136.1	LINRRTPRVDGQPMYGMDAQIEEVRVFEGTEQLPGDPDMMRYVDKYGQLQTKML	354
BAE03142.1	LINRRTPRVDGQPMYGMDAQIEEVRVFEGTEQLPGDPDMMRYVDKYGQLQTKML	354
BAE03148.1	LINRRTPRVDGQPMYGMDAQIEEVRVFEGTEQLPGDPDMMRYVDKYGQLQTKML	354
BAE03154.1	LINRRTPRVDGQPMYGMDAQIEEVRVFEGTEQLPGDPDMMRYVDKYGQLQTKML	354
QBQ04200.1	LINRRTPRVDGQPMYGMDAQIEEVRVFEGTEQLPGDPDLMRYVDKYGQLQTKML	354
UYL83839.1	LINRRTPRVDGQPMYGMDAQIEEVRVFEGTEQLPGDPDMMRYVDRYGQLQTKML	354
ALF06262.1	LINRRTPRVDGQPMYGMDAQIEEVRVFEGTEQLPGDPDMMRYVDRYGQLQTK--	352
ABH10586.1	LINRRTPRVDGQPMYGMDAQIEEVRVFEGTEQLPGDPDMMRYVDRYGQLQTKML	354
BAE02890.1	LINRRTPRVDGQPMYGMDAQIEEVRVFEGTEQLPGDPDMMRYVDRYGQLQTKML	354
BAE02896.1	LINRRTPRVDGQPMYGMDAQIEEVRVFEGTEQLPGDPDMMRYVDRYGQLQTKML	354
BAE02902.1	LINRRTPRVDGQPMYGMDAQIEEVRVFEGTEQLPGDPDMMRYVDRYGQLQTKML	354
BAE02914.1	LINRRTPRVDGQPMYGMDAQIEEVRVFEGTEQLPGDPDMMRYVDRYGQLQTKML	354
BAE02920.1	LINRRTPRVDGQPMYGMDAQIEEVRVFEGTEQLPGDPDMMRYVDRYGQLQTKML	354
BAE02944.1	LINRRTPRVDGQPMYGMDAQIEEVRVFEGTEQLPGDPDMMRYVDRYGQLQTKML	354
BAE02962.1	LINRRTPRVDGQPMYGMDAQIEEVRVFEGTEQLPGDPDMMRYVDRYGQLQTKML	354
BAE02968.1	LINRRTPRVDGQPMYGMDAQIEEVRVFEGTEQLPGDPDMMRYVDRYGQLQTKML	354
BAE02974.1	LINRRTPRVDGQPMYGMDAQIEEVRVFEGTEQLPGDPDMMRYVDRYGQLQTKML	354
BAE02980.1	LINRRTPRVDGQPMYGMDAQIEEVRVFEGTEQLPGDPDMMRYVDRYGQLQTKML	354
BAE02986.1	LINRRTPRVDGQPMYGMDAQIEEVRVFEGTEQLPGDPDMMRYVDRYGQLQTKML	354
BAE02992.1	LINRRTPRVDGQPMYGMDAQIEEVRVFEGTEQLPGDPDMMRYVDRYGQLQTKML	354
BAE02998.1	LINRRTPRVDGQPMYGMDAQIEEVRVFEGTEQLPGDPDMMRYVDRYGQLQTKML	354
BAE03010.1	LINRRTPRVDGQPMYGMDAQIEEVRVFEGTEQLPGDPDMMRYVDRYGQLQTKML	354
BAE03016.1	LINRRTPRVDGQPMYGMDAQIEEVRVFEGTEQLPGDPDMMRYVDRYGQLQTKML	354
BAE03022.1	LINRRTPRVDGQPMYGMDAQIEEVRVFEGTEQLPGDPDMMRYVDRYGQLQTKML	354
BAE03028.1	LINRRTPRVDGQPMYGMDAQIEEVRVFEGTEQLPGDPDMMRYVDRYGQLQTKML	354
BAE03034.1	LINRRTPRVDGQPMYGMDAQIEEVRVFEGTEQLPGDPDMMRYVDRYGQLQTKML	354
BAE03040.1	LINRRTPRVDGQPMYGMDAQIEEVRVFEGTEQLPGDPDMMRYVDRYGQLQTKML	354
BAE03046.1	LINRRTPRVDGQPMYGMDAQIEEVRVFEGTEQLPGDPDMMRYVDRYGQLQTKML	354
BAE03052.1	LINRRTPRVDGQPMYGMDAQIEEVRVFEGTEQLPGDPDMMRYVDRYGQLQTKML	354
BAE03058.1	LINRRTPRVDGQPMYGMDAQIEEVRVFEGTEQLPGDPDMMRYVDRYGQLQTKML	354
BAE03064.1	LINRRTPRVDGQPMYGMDAQIEEVRVFEGTEQLPGDPDMMRYVDRYGQLQTKML	354
BAE03070.1	LINRRTPRVDGQPMYGMDAQIEEVRVFEGTEQLPGDPDMMRYVDRYGQLQTKML	354
BAE03076.1	LINRRTPRVDGQPMYGMDAQIEEVRVFEGTEQLPGDPDMMRYVDRYGQLQTKML	354
BAE03082.1	LINRRTPRVDGQPMYGMDAQIEEVRVFEGTEQLPGDPDMMRYVDRYGQLQTKML	354
BAE03088.1	LINRRTPRVDGQPMYGMDAQIEEVRVFEGTEQLPGDPDMMRYVDRYGQLQTKML	354

```
BAE03094.1       LINRRTPRVDGQPMYGMDAQIEEVRVFEGTEQLPGDPDMMRYVDRYGQLQTKML  354
BAE03100.1       LINRRTPRVDGQPMYGMDAQIEEVRVFEGTEQLPGDPDMMRYVDRYGQLQTKML  354
BAE03106.1       LINRRTPRVDGQPMYGMDAQIEEVRVFEGTEQLPGDPDMMRYVDRYGQLQTKML  354
BAE03112.1       LINRRTPRVDGQPMYGMDAQIEEVRVFEGTEQLPGDPDMMRYVDRYGQLQTKML  354
BAE03160.1       LINRRTPRVDGQPMYGMDAQIEEVRVFEGTEQLPGDPDMMRYVDRYGQLQTKML  354
BAE03166.1       LINRRTPRVDGQPMYGMDAQIEEVRVFEGTEQLPGDPDMMRYVDRYGQLQTKML  354
AAQ88264.1       LINRRTPRVDGQPMYGMDAQIEEVRVFEGTEQLPGDPDMMRYVDRYGQLQTKML  354
AAQ88270.1       LINRRTPRVDGQPMYGMDAQIEEVRVFEGTEQLPGDPDMMRYVDRYGQLQTKML  354
AAR89187.1       LINRRTPRVDGQPMYGMDAQIEEVRVFEGTEQLPGDPDMMRYVDRYGQLQTKML  354
AAR89193.1       LINRRTPRVDGQPMYGMDAQIEEVRVFEGTEQLPGDPDMMRYVDRYGQLQTKML  354
AAR89199.1       LINRRTPRVDGQPMYGMDAQIEEVRVFEGTEQLPGDPDMMRYVDRYGQLQTKML  354
AAR89205.1       LINRRTPRVDGQPMYGMDAQIEEVRVFEGTEQLPGDPDMMRYVDRYGQLQTKML  354
AAR89217.1       LINRRTPRVDGQPMYGMDAQIEEVRVFEGTEQLPGDPDMMRYVDRYGQLQTKML  354
AAR89223.1       LINRRTPRVDGQPMYGMDAQIEEVRVFEGTEQLPGDPDMMRYVDRYGQLQTKML  354
AAR89229.1       LINRRTPRVDGQPMYGMDAQIEEVRVFEGTEQLPGDPDMMRYVDRYGQLQTKML  354
AAR89235.1       LINRRTPRVDGQPMYGMDAQIEEVRVFEGTEQLPGDPDMMRYVDRYGQLQTKML  354
AAR89241.1       LINRRTPRVDGQPMYGMDAQIEEVRVFEGTEQLPGDPDMMRYVDRYGQLQTKML  354
AAR89247.1       LINRRTPRVDGQPMYGMDAQIEEVRVFEGTEQLPGDPDMMRYVDRYGQLQTKML  354
AAR89253.1       LINRRTPRVDGQPMYGMDAQIEEVRVFEGTEQLPGDPDMMRYVDRYGQLQTKML  354
AAR32743.1       LINRRTPRVDGQPMYGMDAQIEEVRVFEGTEQLPGDPDMMRYVDRYGQLQTKML  354
AAR89265.1       LINRRTPRVDGQPMYGMDAQIEEVRVFEGTEQLPGDPDMMRYVDRYGQLQTKML  354
AAR89277.1       LINRRTPRVDGQPMYGMDAQIEEVRVFEGTEQLPGDPDMMRYVDRYGQLQTKML  354
AAR89283.1       LINRRTPRVDGQPMYGMDAQIEEVRVFEGTEQLPGDPDMMRYVDRYGQLQTKML  354
AAR02463.1       LINRRTPRVDGQPMYGMDAQIEEVRVFEGTEQLPGDPDMMRYVDRYGQLQTKML  354
AAR06661.1       LINRRTPRVDGQPMYGMDAQIEEVRVFEGTEQLPGDPDMMRYVDRYGQLQTKML  354
AAR13659.1       LINRRTPRVDGQPMYGMDAQIEEVRVFEGTEQLPGDPDMMRYVDRYGQLQTKML  354
AAR02457.1       LINRRTPRVDGQPMYGMDAQIEEVRVFEGTEQLPGDPDMMRYVDRYGQLQTKML  354
BAB68785.1       LINRRTPRVDGQPMYGMDAQIEEVRVFEGTEQLPGDPDMMRYVDRYGQLQTKML  354
AAK28472.1       LINRRTPRVDGQPMYGMDAQIEEVRVFEGTEQLPGDPDMMRYVDRYGQLQTKML  354
AAG34667.1       LINRRTPRVDGQPMYGMDAQIEEVRVFEGTEQLPGDPDMMRYVDRYGQLQTKML  354
AAG34673.1       LINRRTPRVDGQPMYGMDAQIEEVRVFEGTEQLPGDPDMMRYVDRYGQLQTKML  354
AAB41705.1       LINRRTPRVDGQPMYGMDAQIEEVRVFEGTEQLPGDPDMMRYVDRYGQLQTKML  354
AAB41711.1       LINRRTPRVDGQPMYGMDAQIEEVRVFEGTEQLPGDPDMMRYVDRYGQLQTKML  354
AAB41717.1       LINRRTPRVDGQPMYGMDAQIEEVRVFEGTEQLPGDPDMMRYVDRYGQLQTKML  354
BAE02926.1       LINRRTPRVDGQPMYGMDAQIEEVRVFEGTEQLPGDPDMMRYVDKYGQLQTKML  354
                  :* *** ::****: :  :  :*:  *.** :* **:: ** *   :
```
