# Supplementary material for: The Conserved YPX3L Motif in the BK Polyomavirus VP1 Protein Is Important for Viral Particle Assembly but Not for Its Secretion into Extracellular Vesicles
Source: Viruses. 2024 Jul 13;16(7):1124. doi: 10.3390/v16071124 (PMC11281352; doi:10.3390/v16071124)
Supplement: Supplementary file 1 [file viruses-16-01124-s001.zip › HPyV 03 alignment.pdf]

CLUSTAL O(1.2.4) multiple sequence alignment

```
ACF20217.1          MSCTACRPQKRLTRPRSQVPRVQTLATEVKKGGVEVLAAVPLSEETEFKVELFVKPVIGN	60
ACR43493.1          MSCTPCRPQKRLTRPRSQVPRVQTLATEVKKGGVEVLAAVPLSEETEFKVELFVKPVIGN	60
ACB12021.1          MSCTPCRPQKRLTRPRSQVPRVQTLATEVKKGGVEVLAAVPLSEETEFKVELFVKPVIGN	60
ABR68677.1          MSCTPCRPQKRLTRPRSQVPRVQTLATEVKKGGVEVLAAVPLSEETEFKVELFVKPVIGN	60
WIV69149.1          MSCTPCRPQKRLTRPRSQVPRVQTLATEVKKGGVEVLAAVPLSEETEFKVELFVKPVIGN	60
WIV69154.1          MSCTPCRPQKRLTRPRSQVPRVQTLATEVKKGGVEVLAAVPLSEETEFKVELFVKPVIGN	60
QCQ73650.1          MSCTPCRPQKRLTRPRSQVPRVQTLATEVKKGGVEVLAAVPLSEETEFKVELFVKPVIGN	60
AIN39530.1          MSCTPCRPQKRLTRPRSQVPRVQTLATEVKKGGVEVLAAVPLSEETEFKVELFVKPVIGN	60
ABR68687.1          MSCTPCRPQKRLTRPRSQVPRVQTLATEVKKGGVEVLAAVPLSEETEFKVELFVKPVIGN	60
YP_001111258.1      MSCTPCRPQKRLTRPRSQVPRVQTLATEVKKGGVEVLAAVPLSEETEFKVELFVKPVIGN	60
WIV69159.1          MSCTPCRPQKRLTRPRSQVPRVQTLATEVKKGGVEVLAAVPLSEETEFKVELFVKPVIGN	60
P0DOI3.1            MSCTPCRPQKRLTRPRSQVPRVQTLATEVKKGGVEVLAAVPLSEETEFKVELFVKPVIGN	60
P0DOI4.1            MSCTPCRPQKRLTRPRSQVPRVQTLATEVKKGGVEVLAAVPLSEETEFKVELFVKPVIGN	60
ANY58913.1          MSCTPCRPQKRLTRPRSQVPRVQTLATEVKKGGVEVLAAVPLSEETEFKVELFVKPVIGN	60
AHB32975.1          MSCTPCRPQKRLTRPRSQVPRVQTLATEVKKGGVEVLAAVPLSEETEFKVELFVKPVIGN	60
ACR43492.1          MSCTPCRPQKRLTRPRSQVPRVQTLATEVKKGGVEVLAAVPLSEETEFKVELFVKPVIGN	60
ACR43494.1          MSCTPCRPQKRLTRPRSQVPRVQTLATEVKKGGVEVLAAVPLSEETEFKVELFVKPVIGN	60
ACR43495.1          MSCTPCRPQKRLTRPRSQVPRVQTLATEVKKGGVEVLAAVPLSEETEFKVELFVKPVIGN	60
ACM92035.1          MSCTPCRPQKRLTRPRSQVPRVQTLATEVKKGGVEVLAAVPLSEETEFKVELFVKPVIGN	60
ACB12026.1          MSCTPCRPQKRLTRPRSQVPRVQTLATEVKKGGVEVLAAVPLSEETEFKVELFVKPVIGN	60
ABR68682.1          MSCTPCRPQKRLTRPRSQVPRVQTLATEVKKGGVEVLAAVPLSEETEFKVELFVKPVIGN	60
ABN09917.1          MSCTPCRPQKRLTRPRSQVPRVQTLATEVKKGGVEVLAAVPLSEETEFKVELFVKPVIGN	60
ABN09927.1          MSCTPCRPQKRLTRPRSQVPRVQTLATEVKKGGVEVLAAVPLSEETEFKVELFVKPVIGN	60
BEP33024.1          MSCTPCRPQKRLTRPRSQVPRVQTLATEVKKGGVEVLAAVPLSEETEFKVELFVKPVIGN	60
P0DOI2.1            MSCTPCRPQKRLTRPRSQVPRVQTLATEVKKGGVEVLAAVPLSEETEFKVELFVKPVIGN	60
ABN09922.1          MSCTPCRPQKRLTRPRSQVPRVQTLATEVKKGGVEVLAAVPLSEETEFKVELFVKPVIGN	60
                    **** *******************************************************

ACF20217.1          TTAAQDGREPTPHYWSISSAIHDKESGSSIKVEETPDADTTVCYSLAEIAPPDIPNQVSE	120
ACR43493.1          TTAAQDGREPTPHYWSISSAIHDKESGSSIKVEETPDADTTVCYSLAEIAPPDIPNQVSE	120
ACB12021.1          TTAAQDGREPTPHYWSISSAIHDKESGSSIKVEETPDADTTVCYSLAEIAPPDIPNQVSE	120
ABR68677.1          TTAAQDGREPTPHYWSISSAIHDKESGSSIKVEETPDADTTVCYSLAEIAPPDIPNQVSE	120
WIV69149.1          TTAAQDGREPTPHYWSISSAIHDKESGSSIKVEETPDADTTVCYSLAEIAPPDIPNQVSE	120
WIV69154.1          TTAAQDGREPTPHYWSISSAIHDKESGSSIKVEETPDADTTVCYSLAEIAPPDIPNQVSE	120
QCQ73650.1          TTAAQDGREPTPHYWSISSAIHDKESGSSIKVEETPDADTTVCYSLAEIAPPDIPNQVSE	120
AIN39530.1          TTAAQDGREPTPHYWSISSAIHDKESGSSIKVEETPDADTTVCYSLAEIAPPDIPNQVSE	120
ABR68687.1          TTAAQDGREPTPHYWSISSAIHDKESGSSIKVEETPDADTTVCYSLAEIAPPDIPNQVSE	120
YP_001111258.1      TTAAQDGREPTPHYWSISSAIHDKESGSSIKVEETPDADTTVCYSLAEIAPPDIPNQVSE	120
WIV69159.1          TTAAQDGREPTPHYWSISSAIHDKESGSSIKVEETPDADTTVCYSLAEIAPPDIPNQVSE	120
P0DOI3.1            TTAAQDGREPTPHYWSISSAIHDKESGSSIKVEETPDADTTVCYSLAEIAPPDIPNQVSE	120
P0DOI4.1            TTAAQDGREPTPHYWSISSAIHDKESGSSIKVEETPDADTTVCYSLAEIAPPDIPNQVSE	120
ANY58913.1          TTAAQDGREPTPHYWSISSAIHDKESGSSIKVEETPDADTTVCYSLAEIAPPDIPNQVSE	120
AHB32975.1          TTAAQDGREPTPHYWSISSAIHDKESGSSIKVEETPDADTTVCYSLAEIAPPDIPNQVSE	120
ACR43492.1          TTAAQDGREPTPHYWSISSAIHDKESGSSIKVEETPDADTTVCYSLAEIAPPDIPNQVSE	120
ACR43494.1          TTAAQDGREPTPHYWSISSAIHDKESGSSIKVEETPDADTTVCYSLAEIAPPDIPNQVSE	120
ACR43495.1          TTAAQDGREPTPHYWSISSAIHDKESGSSIKVEETPDADTTVCYSLAEIAPPDIPNQVSE	120
ACM92035.1          TTAAQDGREPTPHYWSISSAIHDKESGSSIKVEETPDADTTVCYSLAEIAPPDIPNQVSE	120
ACB12026.1          TTAAQDGREPTPHYWSISSAIHDKESGSSIKVEETPDADTTVCYSLAEIAPPDIPNQVSE	120
ABR68682.1          TTAAQDGREPTPHYWSISSAIHDKESGSSIKVEETPDADTTVCYSLAEIAPPDIPNQVSE	120
ABN09917.1          TTAAQDGREPTPHYWSISSAIHDKESGSSIKVEETPDADTTVCYSLAEIAPPDIPNQVSE	120
ABN09927.1          TTAAQDGREPTPHYWSISSAIHDKESGSSIKVEETPDADTTVCYSLAEIAPPDIPNQVSE	120
BEP33024.1          TTAAQDGREPTPHYWSISSAIHDKESGSSIKVEETPDADTTVCYSLAEIAPPDIPNQVSE	120
P0DOI2.1            TTAAQDGREPTPHYWSISSAIHDKESGSSIKVEETPDADTTVCYSLAEIAPPDIPNQVSE	120
ABN09922.1          TTAAQDGREPTPHYWSISSAIHDKESGSSIKVEETPDADTTVCYSLAEIAPPDIPNQVSE	120
                    ************************************************************

ACF20217.1          CDMKVWELYRMETELLVVPLVNALGNTNGVVHGLAGTQLYFWAVGGQPLDVVGVTPTDKY	180
ACR43493.1          CDMKVWELYRMETELLVVPLVNALGNTNGVVHGLAGTQLYFWAVGGQPLDVVGVTPTDKY	180
ACB12021.1          CDMKVWELYRMETELLVVPLVNALGNTNGVVHGLAGTQLYFWAVGGQPLDVVGVTPTDKY	180
ABR68677.1          CDMKVWELYRMETELLVVPLVNALGNTNGVVHGLAGTQLYFWAVGGQPLDVVGVTPTDKY	180
WIV69149.1          CDMKVWELYRMETELLVVPLVNALGNTNGVVHGLAGTQLYFWAVGGQPLDVVGVTPTDKY	180
WIV69154.1          CDMKVWELYRMETELLVVPLVNALGNTNGVVHGLAGTQLYFWAVGGQPLDVVGVTPTDKY	180
QCQ73650.1          CDMKVWELYRMETELLVVPLVNALGNTNGVVHGLAGTQLYFWAVGGQPLDVVGVTPTDKY	180
AIN39530.1          CDMKVWELYRMETELLVVPLVNALGNTNGVVHGLAGTQLYFWAVGGQPLDVVGVTPTDKY	180
```

```
ABR68687.1          CDMKVWELYRMETELLVVPLVNALGNTNGVVHGLAGTQLYFWAVGGQPLDVVGVTPTDKY   180
YP_001111258.1      CDMKVWELYRMETELLVVPLVNALGNTNGVVHGLAGTQLYFWAVGGQPLDVVGVTPTDKY   180
WIV69159.1          CDMKVWELYRMETELLVVPLVNALGNTNGVVHGLAGTQLYFWAVGGQPLDVVGVTPTDKY   180
P0DOI3.1            CDMKVWELYRMETELLVVPLVNALGNTNGVVHGLAGTQLYFWAVGGQPLDVVGVTPTDKY   180
P0DOI4.1            CDMKVWELYRMETELLVVPLVNALGNTNGVVHGLAGTQLYFWAVGGQPLDVVGVTPTDKY   180
ANY58913.1          CDMKVWELYRMETELLVVPLVNALGNTNGVVHGLAGTQLYFWAVGGQPLDVVGVTPTDKY   180
AHB32975.1          CDMKVWELYRMETELLVVPLVNALGNTNGVVHGLAGTQLYFWAVGGQPLDVVGVTPTDKY   180
ACR43492.1          CDMKVWELYRMETELLVVPLVNALGNTNGVVHGLAGTQLYFWAVGGQPLDVVGVTPTDKY   180
ACR43494.1          CDMKVWELYRMETELLVVPLVNALGNTNGVVHGLAGTQLYFWAVGGQPLDVVGVTPTDKY   180
ACR43495.1          CDMKVWELYRMETELLVVPLVNALGNTNGVVHGLAGTQLYFWAVGGQPLDVVGVTPTDKY   180
ACM92035.1          CDMKVWELYRMETELLVVPLVNALGNTNGVVHGLAGTQLYFWAVGGQPLDVVGVTPTDKY   180
ACB12026.1          CDMKVWELYRMETELLVVPLVNALGNTNGVVHGLAGTQLYFWAVGGQPLDVVGVTPTDKY   180
ABR68682.1          CDMKVWELYRMETELLVVPLVNALGNTNGVVHGLAGTQLYFWAVGGQPLDVVGVTPTDKY   180
ABN09917.1          CDMKVWELYRMETELLVVPLVNALGNTNGVVHGLAGTQLYFWAVGGQPLDVVGVTPTDKY   180
ABN09927.1          CDMKVWELYRMETELLVVPLVNALGNTNGVVHGLAGTQLYFWAVGGQPLDVVGVTPTDKY   180
BEP33024.1          CDMKVWELYRMETELLVVPLVNALGNTNGVVHGLAGTQLYFWAVGGQPLDVVGVTPTDKY   180
P0DOI2.1            CDMKVWELYRMETELLVVPLVNALGNTNGVVHGLAGTQLYFWAVGGQPLDVVGVTPTDKY   180
ABN09922.1          CDMKVWELYRMETELLVVPLVNALGNTNGVVHGLAGTQLYFWAVGGQPLDVVGVTPTDKY   180
                    ************************************************************

ACF20217.1          KGPTTYTINPPGDPRTLHVYNSNTPKAKVTSERYSVESWAPDPSRNDNCRYFGRVVGGAA   240
ACR43493.1          RGPTTYTINPPGDPRTLHVYNSNTPKAKVTSERYSVESWAPDPSRNDNCRYFGRVVGGAA   240
ACB12021.1          RGPTTYTINPPGDPRTLHVYNSNTPKAKVTSERYSVESWAPDPSRNDNCRYFGRVVGGAA   240
ABR68677.1          RGPTTYTINPPGDPRTLHVYNSNTPKAKVTSERYSVESWAPDPSRNDNCRYFGRVVGGAA   240
WIV69149.1          KGPTTYTINPPGDPRTLHVYNSNTPKAKVTSERYSVESWAPDPSRNDNCRYFGRVVGGAA   240
WIV69154.1          KGPTTYTINPPGDPRTLHVYNSNTPKAKVTSERYSVESWAPDPSRNDNCRYFGRVVGGAA   240
QCQ73650.1          KGPTTYTINPPGDPRTLHVYNSNTPKAKVTSERYSVESWAPDPSRNDNCRYFGRVVGGAA   240
AIN39530.1          KGPTTYTINPPGDPRTLHVYNSNTPKAKVTSERYSVESWAPDPSRNDNCRYFGRVVGGAA   240
ABR68687.1          KGPTTYTINPPGDPRTLHVYNSNTPKAKVTSERYSVESWAPDPSRNDNCRYFGRVVGGAA   240
YP_001111258.1      KGPTTYTINPPGDPRTLHVYNSNTPKAKVTSERYSVESWAPDPSRNDNCRYFGRVVGGAA   240
WIV69159.1          KGPTTYTINPPGDPRTLHVYNSNTPKAKVTSERYSVESWAPDPSRNDNCRYFGRVVGGAA   240
P0DOI3.1            KGPTTYTINPPGDPRTLHVYNSNTPKAKVTSERYSVESWAPDPSRNDNCRYFGRVVGGAA   240
P0DOI4.1            KGPTTYTINPPGDPRTLHVYNSNTPKAKVTSERYSVESWAPDPSRNDNCRYFGRVVGGAA   240
ANY58913.1          KGPTTYTINPPGDPRTLHVYNSNTPKAKVTSERYSVESWAPDPSRNDNCRYFGRVVGGAA   240
AHB32975.1          KGPTTYTINPPGDPRTLHVYNSNTPKAKVTSERYSVESWAPDPSRNDNCRYFGRVVGGAA   240
ACR43492.1          KGPTTYTINPPGDPRTLHVYNSNTPKAKVTSERYSVESWAPDPSRNDNCRYFGRVVGGAA   240
ACR43494.1          KGPTTYTINPPGDPRTLHVYNSNTPKAKVTSERYSVESWAPDPSRNDNCRYFGRVVGGAA   240
ACR43495.1          KGPTTYTINPPGDPRTLHVYNSNTPKAKVTSERYSVESWAPDPSRNDNCRYFGRVVGGAA   240
ACM92035.1          KGPTTYTINPPGDPRTLHVYNSNTPKAKVTSERYSVESWAPDPSRNDNCRYFGRVVGGAA   240
ACB12026.1          KGPTTYTINPPGDPRTLHVYNSNTPKAKVTSERYSVESWAPDPSRNDNCRYFGRVVGGAA   240
ABR68682.1          KGPTTYTINPPGDPRTLHVYNSNTPKAKVTSERYSVESWAPDPSRNDNCRYFGRVVGGAA   240
ABN09917.1          KGPTTYTINPPGDPRTLHVYNSNTPKAKVTSERYSVESWAPDPSRNDNCRYFGRVVGGAA   240
ABN09927.1          KGPTTYTINPPGDPRTLHVYNSNTPKAKVTSERYSVESWAPDPSRNDNCRYFGRVVGGAA   240
BEP33024.1          KGPTTYTINPPGDPRTLHVYNSNTPKAKVTSERYSVESWAPDPSRNDNCRYFGRVVGGAA   240
P0DOI2.1            KGPTTYTINPPGDPRTLHVYNSNTPKAKVTSERYSVESWAPDPSRNDNCRYFGRVVGGAA   240
ABN09922.1          KGPTTYTINPPGDPRTLHVYNSNTPKAKVTSERYSVESWAPDPSRNDNCRYFGRVVGGAA   240
                    :***********************************************************

ACF20217.1          TPPVVSYGNNSTIPLLDENGIGILCLQGRLYITCADMLGTANSRIHTPMARFFRLHFRQR   300
ACR43493.1          TPPVVSYGNNSTIPLLDENGIGILCLQGRLYITCADMLGTANSRIHTPMARFFRLHFRQR   300
ACB12021.1          TPPVVSYGNNSTIPLLDENGIGILCLQGRLYITCADMLGTANSRIHTPMARFFRLHFRQR   300
ABR68677.1          TPPVVSYGNNSTIPLLDENGIGILCLQGRLYITCADMLGTANSRIHTPMARFFRLHFRQR   300
WIV69149.1          TPPVVSYGNNSTIPLLDENGIGILCLHGRLYITCADMLGTANSRIHTPMARFFRLHFRQR   300
WIV69154.1          TPPVVSYGNNSTIPLLDENGIGILCLHGRLYITCADMLGTANSRIHTPMARFFRLHFRQR   300
QCQ73650.1          TPPVVSYGNNSTIPLLDENGIGILCLHGRLYITCADMLGTANSRIHTPMARFFRLHFRQR   300
AIN39530.1          TPPVVSYGNNSTIPLLDENGIGILCLHGRLYITCADMLGTANSRIHTPMARFFRLHFRQR   300
ABR68687.1          TPPVVSYGNNSTIPLLDENGIGILCLHGRLYITCADMLGTANSRIHTPMARFFRLHFRQR   300
YP_001111258.1      TPPVVSYGNNSTIPLLDENGIGILCLQGRLYITCADMLGTANSRIHTPMARFFRLHFRQR   300
WIV69159.1          TPPVVSYGNNSTIPLLDENGIGILCLQGRLYITCADMLGTANSRIHTPMARFFRLHFRQR   300
P0DOI3.1            TPPVVSYGNNSTIPLLDENGIGILCLQGRLYITCADMLGTANSRIHTPMARFFRLHFRQR   300
P0DOI4.1            TPPVVSYGNNSTIPLLDENGIGILCLQGRLYITCADMLGTANSRIHTPMARFFRLHFRQR   300
ANY58913.1          TPPVVSYGNNSTIPLLDENGIGILCLQGRLYITCADMLGTANSRIHTPMARFFRLHFRQR   300
AHB32975.1          TPPVVSYGNNSTIPLLDENGIGILCLQGRLYITCADMLGTANSRIHTPMARFFRLHFRQR   300
ACR43492.1          TPPVVSYGNNSTIPLLDENGIGILCLQGRLYITCADMLGTANSRIHTPMARFFRLHFRQR   300
ACR43494.1          TPPVVSYGNNSTIPLLDENGIGILCLQGRLYITCADMLGTANSRIHTPMARFFRLHFRQR   300
ACR43495.1          TPPVVSYGNNSTIPLLDENGIGILCLQGRLYITCADMLGTANSRIHTPMARFFRLHFRQR   300
ACM92035.1          TPPVVSYGNNSTIPLLDENGIGILCLQGRLYITCADMLGTANSRIHTPMARFFRLHFRQR   300
```

```
ACB12026.1          TPPVVSYGNNSTIPLLDENGIGILCLQGRLYITCADMLGTANSRIHTPMARFFRLHFRQR   300
ABR68682.1          TPPVVSYGNNSTIPLLDENGIGILCLQGRLYITCADMLGTANSRIHTPMARFFRLHFRQR   300
ABN09917.1          TPPVVSYGNNSTIPLLDENGIGILCLQGRLYITCADMLGTANSRIHTPMARFFRLHFRQR   300
ABN09927.1          TPPVVSYGNNSTIPLLDENGIGILCLQGRLYITCADMLGTANSRIHTPMARFFRLHFRQR   300
BEP33024.1          TPPVVSYGNNSTIPLLDENGIGILCLQGRLYITCADMLGTANSRIHTPMARFFRLHFRQR   300
P0DOI2.1            TPPVVSYGNNSTIPLLDENGIGILCLQGRLYITCADMLGTANSRIHTPMARFFRLHFRQR   300
ABN09922.1          TPPVVSYGNNSTIPLLDENGIGILCLQGRLYITCADMLGTANSRIHTPMARFFRLHFRQR   300
                    **************************:*********************************

ACF20217.1          RVKNPFTMNVLYKQVFNRPTETVDAQVGVTEVTMVEEIGPLPPSIQTTLPTSVNLTQLPR   360
ACR43493.1          RVKNPFTMNVLYKQVFNRPTETVDAQVGVTEVTMVEEIGPLPPSIQTTLPTSVNLTQLPR   360
ACB12021.1          RVKNPFTMNVLYKQVFNRPTETVDAQVGVTEVTMVEEIGPLPPSIQTTLPTSVNLTQLPR   360
ABR68677.1          RVKNPFTMNVLYKQVFNRPTETVDAQVGVTEVTMVEEIGPLPPSIQTTLPTSVNLTQLPR   360
WIV69149.1          RVKNPFTMNVLYKQVFNRPTETVDAQVGVTEVTMVEEIGPLPPSIQTTLPTSVNLTQLPR   360
WIV69154.1          RVKNPFTMNVLYKQVFNRPTETVDAQVGVTEVTMVEEIGPLPPSIQTTLPTSVNLTQLPR   360
QCQ73650.1          RVKNPFTMNVLYKQVFNRPTETVDAQVGVTEVTMVEEIGPLPPSIQTTLPTSVNLTQLPR   360
AIN39530.1          RVKNPFTMNVLYKQVFNRPTETVDAQVGVTEVTMVEEIGPLPPSIQTTLPTSVNLTQLPR   360
ABR68687.1          RVKNPFTMNVLYKQVFNRPTETVDAQVGVTEVTMVEEIGPLPPSIQTTLPTSVNLTQLPR   360
YP_001111258.1      RVKNPFTMNVLYKQVFNRPTETVDAQVGVTEVTMVEEIGPLPPSIQTTLPTSVNLTQLPR   360
WIV69159.1          RVKNPFTMNVLYKQVFNRPTETVDAQVGVTEVTMVEEIGPLPPSIQTTLPTSVNLTQLPR   360
P0DOI3.1            RVKNPFTMNVLYKQVFNRPTETVDAQVGVTEVTMVEEIGPLPPSIQTTLPTSVNLTQLPR   360
P0DOI4.1            RVKNPFTMNVLYKQVFNRPTETVDAQVGVTEVTMVEEIGPLPPSIQTTLPTSVNLTQLPR   360
ANY58913.1          RVKNPFTMNVLYKQVFNRPTETVDAQVGVTEVTMVEEIGPLPPSIQTTLPTSVNLTQLPR   360
AHB32975.1          RVKNPFTMNVLYKQVFNRPTETVDAQVGVTEVTMVEEIGPLPPSIQTTLPTSVNLTQLPR   360
ACR43492.1          RVKNPFTMNVLYKQVFNRPTETVDAQVGVTEVTMVEEIGPLPPSIQTTLPTSVNLTQLPR   360
ACR43494.1          RVKNPFTMNVLYKQVFNRPTETVDAQVGVTEVTMVEEIGPLPPSIQTTLPTSVNLTQLPR   360
ACR43495.1          RVKNPFTMNVLYKQVFNRPTETVDAQVGVTEVTMVEEIGPLPPSIQTTLPTSVNLTQLPR   360
ACM92035.1          RVKNPFTMNVLYKQVFNRPTETVDAQVGVTEVTMVEEIGPLPPSIQTTLPTSVNLTQLPR   360
ACB12026.1          RVKNPFTMNVLYKQVFNRPTETVDAQVGVTEVTMVEEIGPLPPSIQTTLPTSVNLTQLPR   360
ABR68682.1          RVKNPFTMNVLYKQVFNRPTETVDAQVGVTEVTMVEEIGPLPPSIQTTLPTSVNLTQLPR   360
ABN09917.1          RVKNPFTMNVLYKQVFNRPTETVDAQVGVTEVTMVEEIGPLPPSIQTTLPTSVNLTQLPR   360
ABN09927.1          RVKNPFTMNVLYKQVFNRPTETVDAQVGVTEVTMVEEIGPLPPSIQTTLPTSVNLTQLPR   360
BEP33024.1          RVKNPFTMNVLYKQVFNRPTETVDAQVGVTEVTMVEEIGPLPPSIQTTLPTSVNLTQLPR   360
P0DOI2.1            RVKNPFTMNVLYKQVFNRPTETVDAQVGVTEVTMVEEIGPLPPSIQTTLPTSVNLTQLPR   360
ABN09922.1          RVKNPFTMNVLYKQVFNRPTETVDAQVGVTEVTMVEEIGPLPPSIQTTLPTSVNLTQLPR   360
                    ************************************************************

ACF20217.1          TVTLQSQAPLLNTQLNSK   378
ACR43493.1          TVTLQSQAPLLNTQQNSK   378
ACB12021.1          TVTLQSQAPLLNTQQNSK   378
ABR68677.1          TVTLQSQAPLLNTQQNSK   378
WIV69149.1          TVTLQSQAPLLNTQQNSK   378
WIV69154.1          TVTLQSQAPLLNTQQNSK   378
QCQ73650.1          TVTLQSQAPLLNTQQNSK   378
AIN39530.1          TVTLQSQAPLLNTQQNSK   378
ABR68687.1          TVTLQSQAPLLNTQQNSK   378
YP_001111258.1      TVTLQSQAPLLNTQQNSK   378
WIV69159.1          TVTLQSQAPLLNTQQNSK   378
P0DOI3.1            TVTLQSQAPLLNTQQNSK   378
P0DOI4.1            TVTLQSQAPLLNTQQNSK   378
ANY58913.1          TVTLQSQAPLLNTQQNSK   378
AHB32975.1          TVTLQSQAPLLNTQQNSK   378
ACR43492.1          TVTLQSQAPLLNTQQNSK   378
ACR43494.1          TVTLQSQAPLLNTQQNSK   378
ACR43495.1          TVTLQSQAPLLNTQQNSK   378
ACM92035.1          TVTLQSQAPLLNTQQNSK   378
ACB12026.1          TVTLQSQAPLLNTQQNSK   378
ABR68682.1          TVTLQSQAPLLNTQQNSK   378
ABN09917.1          TVTLQSQAPLLNTQQNSK   378
ABN09927.1          TVTLQSQAPLLNTQQNSK   378
BEP33024.1          TVTLQSQTPLLNTQQNSK   378
P0DOI2.1            TVTLQSQTPLLNTQQNSK   378
ABN09922.1          TVTLQSQTPLLNTQQNSK   378
                    *******:****** ***
```
