# Supplementary material for: The Conserved YPX3L Motif in the BK Polyomavirus VP1 Protein Is Important for Viral Particle Assembly but Not for Its Secretion into Extracellular Vesicles
Source: Viruses. 2024 Jul 13;16(7):1124. doi: 10.3390/v16071124 (PMC11281352; doi:10.3390/v16071124)
Supplement: Supplementary file 1 [file viruses-16-01124-s001.zip › HPyV 04 alignment.pdf]

CLUSTAL O(1.2.4) multiple sequence alignment

```
ADD51146.1          MACTAKPACTAKPGRSPRSQPTRLQSLPKQVRKGGVDVLAAVPLSEETEFQVELFVKPVI   60
ADD51151.1          MACTAKPACTAKPGRSPRSQPTRLQSLPKQVRKGGVDVLAAVPLSEETEFQVELFVKPVI   60
ADD51156.1          MACTAKPACTAKPGRSPRSQPTRLQSLPKQVRKGGVDVLAAVPLSEETEFQVELFVKPVI   60
WIV69164.1          MACTAKAACTAKPGRSPRSQPTRLQSLPKQVRKGGVDVLSAVPLSEETEFKVELFVKPVI   60
ARX17310.1          MACTAKAACTAKPGRSPRSQPTRLQSLPKQVRKGGVDVLSAVPLSEETEFKVELFVKPVI   60
ARQ15101.1          MACTAKAACTAKPGRSPRSQPTRLQSLPKQVRKGGVDVLSAVPLSEETEFKVELFVKPVI   60
ARQ15131.1          MACTAKAACTAKPGRSPRSQPTRLQSLPKQVRKGGVDVLSAVPLSEETEFKVELFVKPVI   60
ARQ15046.1          MACTAKAACTAKPGRSPRSQPTRLQSLPKQVRKGGVDVLSAVPLSEETEFKVELFVKPVI   60
ARQ15091.1          MACTAKAACTAKPGRSPRSQPTRLQSLPKQVRKGGVDVLSAVPLSEETEFKVELFVKPVI   60
ARQ15126.1          MACTAKAACTAKPGRSPRSQPTRLQSLPKQVRKGGVDVLSAVPLSEETEFKVELFVKPVI   60
AXU41991.1          MACTAKAACTAKPGRSPRSQPTRLQSLPKQVRKGGVDVLSAVPLSEETEFQVELFVKPVI   60
AXU41996.1          MACTAKAACTAKPGRSPRSQPTRLQSLPKQVRKGGVDVLSAVPLSEETEFQVELFVKPVI   60
AXU42001.1          MACTAKAACTAKPGRSPRSQPTRLQSLPKQVRKGGVDVLSAVPLSEETEFQVELFVKPVI   60
AMS36878.1          MACTAKAACTAKPGRSPRSQPTRLQSLPKQVRKGGVDVLSAVPLSEETEFQVELFVKPVI   60
ADD50941.1          MACTAKAACTAKPGRSPRSQPTRLQSLPKQVRKGGVDVLSAVPLSEETEFQVELFVKPVI   60
ACF19290.1          MACTAKAACTAKPGRSPRSQPTRLQSLPKQVRKGGVDVLSAVPLSEETEFQVELFVKPVI   60
UJY54021.1          MACTAKAACTAKPGRSPRSQPTRLQSLPKQVRKGGVDVLSAVPLSEETEFKVELFVKPVI   60
ADD50921.1          MACTAKAACTAKPGRSPRSQPTRLQSLPKQVRKGGVDVLSAVPLSEETEFKVELFVKPVI   60
ADD50926.1          MACTAKAACTAKPGRSPRSQPTRLQSLPKQVRKGGVDVLSAVPLSEETEFKVELFVKPVI   60
ADD50931.1          MACTAKAACTAKPGRSPRSQPTRLQSLPKQVRKGGVDVLSAVPLSEETEFKVELFVKPVI   60
ADD50936.1          MACTAKAACTAKPGRSPRSQPTRLQSLPKQVRKGGVDVLSAVPLSEETEFQVELFVKPVI   60
ACF19275.1          MACTAKAACTAKPGRSPRSQPTRLQSLPKQVRKGGVDVLSAVPLSEETEFQVELFVKPVI   60
ACX47164.1          MACTAKPACTAKPGRSPRSQPTRVQSLPKQVRKGGVDVLAAVPLSEETEFKVELFVKPVI   60
ACP27651.1          MACTAKPACTAKPGRSPRSQPTRVQSLPKQVRKGGVDVLAAVPLSEETESKVELFVKPVI   60
AID07487.1          MACTAKPACTAKPGRSPRSQPTRVQSLPKQVRKGGVDVLAAVPLSEETEFKVELFVKPVI   60
ACX47144.1          MACTAKPACTAKPGRSPRSQPTRVQSLPKQVRKGGVDVLAAVPLSEETEFKVELFVKPVI   60
ARX17280.1          MACTAKPACTAKPGRSPRSQPTRVQSLPKQVRKGGVDVLAAVPLSEETEFKVELFVKPVI   60
ADX89640.1          MACTAKPACTAKPGRSPRSQPTRVQSLPKQVRKGGVDVLAAVPLSEETEFKVELFVKPVI   60
ACX47139.1          MACTAKPACTAKPGRSPRSQPTRVQSLPKQVRKGGVDVLAAVPLSEETEFKVELFVKPVI   60
ARQ15036.1          MACTAKPACTAKPGRSPRSQPTRVQSLPKQVRKGGVDVLAAVPLSEETEFKVELFVKPVI   60
ARQ14997.1          MACTAKPACTAKPGRSPRSQPTRVQSLPKQVRKGGVDVLAAVPLSEETEFKVELFVKPVI   60
ARQ15002.1          MACTAKPACTAKPGRSPRSQPTRVQSLPKQVRKGGVDVLAAVPLSEETEFKVELFVKPVI   60
ARQ14967.1          MACTAKPACTAKPGRSPRSQPTRVQSLPKQVRKGGVDVLAAVPLSEETEFKVELFVKPVI   60
ARQ15007.1          MACTAKPACTAKPGRSPRSQPTRVQSLPKQVRKGGVDVLAAVPLSEETEFKVELFVKPVI   60
ARQ15011.1          MACTAKPACTAKPGRSPRSQPTRVQSLPKQVRKGGVDVLAAVPLSEETEFKVELFVKPVI   60
ARQ15021.1          MACTAKPACTAKPGRSPRSQPTRVQSLPKQVRKGGVDVLAAVPLSEETEFKVELFVKPVI   60
ARQ15031.1          MACTAKPACTAKPGRSPRSQPTRVQSLPKQVRKGGVDVLAAVPLSEETEFKVELFVKPVI   60
ARQ15026.1          MACTAKPACTAKPGRSPRSQPTRVQSLPKQVRKGGVDVLAAVPLSEETEFKVELFVKPVI   60
ARQ15081.1          MACTAKPACTAKPGRSPRSQPTRVQSLPKQVRKGGVDVLAAVPLSEETEFKVELFVKPVI   60
AIW01035.1          MACTAKPACTAKPGRSPRSQPTRVQSLPKQVRKGGVDVLAAVPLSEETEFKVELFVKPVI   60
ADD51091.1          MACTAKPACTAKPGRSPRSQPTRVQSLPKQVRKGGVDVLAAVPLSEETQFKVELFVKPVI   60
ADD51116.1          MACTAKPACTAKPGRSPRSQPTRVQSLPKQVRKGGVDVLAAVPLSEETEFKVELFVKPVI   60
YP_001285487.1      MACTAKPACTAKPGRSPRSQPTRVQSLPKQVRKGGVDVLAAVPLSEETEFKVELFVKPVI   60
BEJ44995.1          MACTAKPACTAKPGRSPRSQPTRVQSLPKQVRKGGVDVLAAVPLSEETEFKVELFVKPVI   60
QQX23397.1          MACTAKPACTAKPGRSPRSQPTRVQSLPKQVRKGGVDVLAAVPLSEETEFKVELFVKPVI   60
QCQ73655.1          MACTAKPACTAKPGRSPRSQPTRVQSLPKQVRKGGVDVLAAVPLSEETEFKVELFVKPVI   60
QCQ73660.1          MACTAKPACTAKPGRSPRSQPTRVQSLPKQVRKGGVDVLAAVPLSEETEFKVELFVKPVI   60
QCQ73665.1          MACTAKPACTAKPGRSPRSQPTRVQSLPKQVRKGGVDVLAAVPLSEETEFKVELFVKPVI   60
AXU41951.1          MACTAKPACTAKPGRSPRSQPTRVQSLPKQVRKGGVDVLAAVPLSEETEFKVELFVKPVI   60
AXU41956.1          MACTAKPACTAKPGRSPRSQPTRVQSLPKQVRKGGVDVLAAVPLSEETEFKVELFVKPVI   60
AXU41961.1          MACTAKPACTAKPGRSPRSQPTRVQSLPKQVRKGGVDVLAAVPLSEETEFKVELFVKPVI   60
AXU41966.1          MACTAKPACTAKPGRSPRSQPTRVQSLPKQVRKGGVDVLAAVPLSEETEFKVELFVKPVI   60
AXU41971.1          MACTAKPACTAKPGRSPRSQPTRVQSLPKQVRKGGVDVLAAVPLSEETEFKVELFVKPVI   60
AXU41976.1          MACTAKPACTAKPGRSPRSQPTRVQSLPKQVRKGGVDVLAAVPLSEETEFKVELFVKPVI   60
AXU41981.1          MACTAKPACTAKPGRSPRSQPTRVQSLPKQVRKGGVDVLAAVPLSEETEFKVELFVKPVI   60
AXU41986.1          MACTAKPACTAKPGRSPRSQPTRVQSLPKQVRKGGVDVLAAVPLSEETEFKVELFVKPVI   60
ARQ14987.1          MACTAKPACTAKPGRSPRSQPTRVQSLPKQVRKGGVDVLAAVPLSEETEFKVELFVKPVI   60
ARQ15041.1          MACTAKPACTAKPGRSPRSQPTRVQSLPKQVRKGGVDVLAAVPLSEETEFKVELFVKPVI   60
ARQ15051.1          MACTAKPACTAKPGRSPRSQPTRVQSLPKQVRKGGVDVLAAVPLSEETEFKVELFVKPVI   60
ARQ15106.1          MACTAKPACTAKPGRSPRSQPTRVQSLPKQVRKGGVDVLAAVPLSEETEFKVELFVKPVI   60
ARQ15111.1          MACTAKPACTAKPGRSPRSQPTRVQSLPKQVRKGGVDVLAAVPLSEETEFKVELFVKPVI   60
AVK92965.1          MACTAKPACTAKPGRSPRSQPTRVQSLPKQVRKGGVDVLAAVPLSEETEFKVELFVKPVI   60
ARX17290.1          MACTAKPACTAKPGRSPRSQPTRVQSLPKQVRKGGVDVLAAVPLSEETEFKVELFVKPVI   60
ARX17315.1          MACTAKPACTAKPGRSPRSQPTRVQSLPKQVRKGGVDVLAAVPLSEETEFKVELFVKPVI   60
```

ARX17320.1	MACTAKPACTAKPGRSPRSQPTRVQSLPKQVRKGGVDVLAAVPLSEETEFKVELFVKPVI	60
ARX17330.1	MACTAKPACTAKPGRSPRSQPTRVQSLPKQVRKGGVDVLAAVPLSEETEFKVELFVKPVI	60
ARX17335.1	MACTAKPACTAKPGRSPRSQPTRVQSLPKQVRKGGVDVLAAVPLSEETEFKVELFVKPVI	60
AOW41301.1	MACTAKPACTAKPGRSPRSQPTRVQSLPKQVRKGGVDVLAAVPLSEETEFKVELFVKPVI	60
AMO25748.1	MACTAKPACTAKPGRSPRSQPTRVQSLPKQVRKGGVDVLAAVPLSEETEFKVELFVKPVI	60
AJP06396.1	MACTAKPACTAKPGRSPRSQPTRVQSLPKQVRKGGVDVLAAVPLSEETEFKVELFVKPVI	60
AHB32990.1	MACTAKPACTAKPGRSPRSQPTRVQSLPKQVRKGGVDVLAAVPLSEETEFKVELFVKPVI	60
AHB33000.1	MACTAKPACTAKPGRSPRSQPTRVQSLPKQVRKGGVDVLAAVPLSEETEFKVELFVKPVI	60
ADD50946.1	MACTAKPACTAKPGRSPRSQPTRVQSLPKQVRKGGVDVLAAVPLSEETEFKVELFVKPVI	60
ADD50951.1	MACTAKPACTAKPGRSPRSQPTRVQSLPKQVRKGGVDVLAAVPLSEETEFKVELFVKPVI	60
ADD50956.1	MACTAKPACTAKPGRSPRSQPTRVQSLPKQVRKGGVDVLAAVPLSEETEFKVELFVKPVI	60
ADD50961.1	MACTAKPACTAKPGRSPRSQPTRVQSLPKQVRKGGVDVLAAVPLSEETEFKVELFVKPVI	60
ADD50966.1	MACTAKPACTAKPGRSPRSQPTRVQSLPKQVRKGGVDVLAAVPLSEETEFKVELFVKPVI	60
ADD50971.1	MACTAKPACTAKPGRSPRSQPTRVQSLPKQVRKGGVDVLAAVPLSEETEFKVELFVKPVI	60
ADD50976.1	MACTAKPACTAKPGRSPRSQPTRVQSLPKQVRKGGVDVLAAVPLSEETEFKVELFVKPVI	60
ADD50986.1	MACTAKPACTAKPGRSPRSQPTRVQSLPKQVRKGGVDVLAAVPLSEETEFKVELFVKPVI	60
ADD50991.1	MACTAKPACTAKPGRSPRSQPTRVQSLPKQVRKGGVDVLAAVPLSEETEFKVELFVKPVI	60
ADD51001.1	MACTAKPACTAKPGRSPRSQPTRVQSLPKQVRKGGVDVLAAVPLSEETEFKVELFVKPVI	60
ADD51006.1	MACTAKPACTAKPGRSPRSQPTRVQSLPKQVRKGGVDVLAAVPLSEETEFKVELFVKPVI	60
ADD51011.1	MACTAKPACTAKPGRSPRSQPTRVQSLPKQVRKGGVDVLAAVPLSEETEFKVELFVKPVI	60
ADD51016.1	MACTAKPACTAKPGRSPRSQPTRVQSLPKQVRKGGVDVLAAVPLSEETEFKVELFVKPVI	60
ADD51026.1	MACTAKPACTAKPGRSPRSQPTRVQSLPKQVRKGGVDVLAAVPLSEETEFKVELFVKPVI	60
ADD51031.1	MACTAKPACTAKPGRSPRSQPTRVQSLPKQVRKGGVDVLAAVPLSEETEFKVELFVKPVI	60
ADD51036.1	MACTAKPACTAKPGRSPRSQPTRVQSLPKQVRKGGVDVLAAVPLSEETEFKVELFVKPVI	60
ADD51041.1	MACTAKPACTAKPGRSPRSQPTRVQSLPKQVRKGGVDVLAAVPLSEETEFKVELFVKPVI	60
ADD51046.1	MACTAKPACTAKPGRSPRSQPTRVQSLPKQVRKGGVDVLAAVPLSEETEFKVELFVKPVI	60
ADD51051.1	MACTAKPACTAKPGRSPRSQPTRVQSLPKQVRKGGVDVLAAVPLSEETEFKVELFVKPVI	60
ADD51056.1	MACTAKPACTAKPGRSPRSQPTRVQSLPKQVRKGGVDVLAAVPLSEETEFKVELFVKPVI	60
ADD51061.1	MACTAKPACTAKPGRSPRSQPTRVQSLPKQVRKGGVDVLAAVPLSEETEFKVELFVKPVI	60
ADD51066.1	MACTAKPACTAKPGRSPRSQPTRVQSLPKQVRKGGVDVLAAVPLSEETEFKVELFVKPVI	60
ADD51071.1	MACTAKPACTAKPGRSPRSQPTRVQSLPKQVRKGGVDVLAAVPLSEETEFKVELFVKPVI	60
ADD51076.1	MACTAKPACTAKPGRSPRSQPTRVQSLPKQVRKGGVDVLAAVPLSEETEFKVELFVKPVI	60
ADD51081.1	MACTAKPACTAKPGRSPRSQPTRVQSLPKQVRKGGVDVLAAVPLSEETEFKVELFVKPVI	60
ADD51086.1	MACTAKPACTAKPGRSPRSQPTRVQSLPKQVRKGGVDVLAAVPLSEETEFKVELFVKPVI	60
ADD51096.1	MACTAKPACTAKPGRSPRSQPTRVQSLPKQVRKGGVDVLAAVPLSEETEFKVELFVKPVI	60
ADD51101.1	MACTAKPACTAKPGRSPRSQPTRVQSLPKQVRKGGVDVLAAVPLSEETEFKVELFVKPVI	60
ADD51106.1	MACTAKPACTAKPGRSPRSQPTRVQSLPKQVRKGGVDVLAAVPLSEETEFKVELFVKPVI	60
ADD51121.1	MACTAKPACTAKPGRSPRSQPTRVQSLPKQVRKGGVDVLAAVPLSEETEFKVELFVKPVI	60
ADD51126.1	MACTAKPACTAKPGRSPRSQPTRVQSLPKQVRKGGVDVLAAVPLSEETEFKVELFVKPVI	60
ADD51131.1	MACTAKPACTAKPGRSPRSQPTRVQSLPKQVRKGGVDVLAAVPLSEETEFKVELFVKPVI	60
ADD51136.1	MACTAKPACTAKPGRSPRSQPTRVQSLPKQVRKGGVDVLAAVPLSEETEFKVELFVKPVI	60
ADD51141.1	MACTAKPACTAKPGRSPRSQPTRVQSLPKQVRKGGVDVLAAVPLSEETEFKVELFVKPVI	60
ACW84416.1	MACTAKPACTAKPGRSPRSQPTRVQSLPKQVRKGGVDVLAAVPLSEETEFKVELFVKPVI	60
ACX47149.1	MACTAKPACTAKPGRSPRSQPTRVQSLPKQVRKGGVDVLAAVPLSEETEFKVELFVKPVI	60
ACX47154.1	MACTAKPACTAKPGRSPRSQPTRVQSLPKQVRKGGVDVLAAVPLSEETEFKVELFVKPVI	60
ACR43496.1	MACTAKPACTAKPGRSPRSQPTRVQSLPKQVRKGGVDVLAAVPLSEETEFKVELFVKPVI	60
ACR43497.1	MACTAKPACTAKPGRSPRSQPTRVQSLPKQVRKGGVDVLAAVPLSEETEFKVELFVKPVI	60
ACR43499.1	MACTAKPACTAKPGRSPRSQPTRVQSLPKQVRKGGVDVLAAVPLSEETEFKVELFVKPVI	60
ACF19270.1	MACTAKPACTAKPGRSPRSQPTRVQSLPKQVRKGGVDVLAAVPLSEETEFKVELFVKPVI	60
ACF19285.1	MACTAKPACTAKPGRSPRSQPTRVQSLPKQVRKGGVDVLAAVPLSEETEFKVELFVKPVI	60
ACB12031.1	MACTAKPACTAKPGRSPRSQPTRVQSLPKQVRKGGVDVLAAVPLSEETEFKVELFVKPVI	60
ABY26549.1	MACTAKPACTAKPGRSPRSQPTRVQSLPKQVRKGGVDVLAAVPLSEETEFKVELFVKPVI	60
A5HBD5.1	MACTAKPACTAKPGRSPRSQPTRVQSLPKQVRKGGVDVLAAVPLSEETEFKVELFVKPVI	60
ABQ09289.1	MACTAKPACTAKPGRSPRSQPTRVQSLPKQVRKGGVDVLAAVPLSEETEFKVELFVKPVI	60
ABQ09296.1	MACTAKPACTAKPGRSPRSQPTRVQSLPKQVRKGGVDVLAAVPLSEETEFKVELFVKPVI	60
ABQ09301.1	MACTAKPACTAKPGRSPRSQPTRVQSLPKQVRKGGVDVLAAVPLSEETEFKVELFVKPVI	60
ABQ09306.1	MACTAKPACTAKPGRSPRSQPTRVQSLPKQVRKGGVDVLAAVPLSEETEFKVELFVKPVI	60
ABQ09311.1	MACTAKPACTAKPGRSPRSQPTRVQSLPKQVRKGGVDVLAAVPLSEETEFKVELFVKPVI	60
ABQ09316.1	MACTAKPACTAKPGRSPRSQPTRVQSLPKQVRKGGVDVLAAVPLSEETEFKVELFVKPVI	60
QQX23402.1	MACTAKPACTAKPGRSPRSQPTRVQSLPKQVRKGGVDVLAAVPLSEETEFKVELFVKPVI	60
QJR84015.1	MACTAKPACTAKPGRSPRSQPTRVQSLPKQVRKGGVDVLAAVPLSEETEFKVELFVKPVI	60
QJR84020.1	MACTAKPACTAKPGRSPRSQPTRVQSLPKQVRKGGVDVLAAVPLSEETEFKVELFVKPVI	60
ARQ14962.1	MACTAKPACTAKPGRSPRSQPTRVQSLPKQVRKGGVDVLAAVPLSEETEFKVELFVKPVI	60
ARQ14972.1	MACTAKPACTAKPGRSPRSQPTRVQSLPKQVRKGGVDVLAAVPLSEETEFKVELFVKPVI	60
ARQ14977.1	MACTAKPACTAKPGRSPRSQPTRVQSLPKQVRKGGVDVLAAVPLSEETEFKVELFVKPVI	60
ARQ14982.1	MACTAKPACTAKPGRSPRSQPTRVQSLPKQVRKGGVDVLAAVPLSEETEFKVELFVKPVI	60
ARQ14992.1	MACTAKPACTAKPGRSPRSQPTRVQSLPKQVRKGGVDVLAAVPLSEETEFKVELFVKPVI	60

```
ARQ15056.1          MACTAKPACTAKPGRSPRSQPTRVQSLPKQVRKGGVDVLAAVPLSEETEFKVELFVKPVI   60
ARQ15061.1          MACTAKPACTAKPGRSPRSQPTRVQSLPKQVRKGGVDVLAAVPLSEETEFKVELFVKPVI   60
ARQ15066.1          MACTAKPACTAKPGRSPRSQPTRVQSLPKQVRKGGVDVLAAVPLSEETEFKVELFVKPVI   60
ARQ15071.1          MACTAKPACTAKPGRSPRSQPTRVQSLPKQVRKGGVDVLAAVPLSEETEFKVELFVKPVI   60
ARQ15076.1          MACTAKPACTAKPGRSPRSQPTRVQSLPKQVRKGGVDVLAAVPLSEETEFKVELFVKPVI   60
ARQ15086.1          MACTAKPACTAKPGRSPRSQPTRVQSLPKQVRKGGVDVLAAVPLSEETEFKVELFVKPVI   60
ARQ15096.1          MACTAKPACTAKPGRSPRSQPTRVQSLPKQVRKGGVDVLAAVPLSEETEFKVELFVKPVI   60
ARQ15116.1          MACTAKPACTAKPGRSPRSQPTRVQSLPKQVRKGGVDVLAAVPLSEETEFKVELFVKPVI   60
ARX17285.1          MACTAKPACTAKPGRSPRSQPTRVQSLPKQVRKGGVDVLAAVPLSEETEFKVELFVKPVI   60
ARX17295.1          MACTAKPACTAKPGRSPRSQPTRVQSLPKQVRKGGVDVLAAVPLSEETEFKVELFVKPVI   60
ARX17300.1          MACTAKPACTAKPGRSPRSQPTRVQSLPKQVRKGGVDVLAAVPLSEETEFKVELFVKPVI   60
ARX17305.1          MACTAKPACTAKPGRSPRSQPTRVQSLPKQVRKGGVDVLAAVPLSEETEFKVELFVKPVI   60
ARX17325.1          MACTAKPACTAKPGRSPRSQPTRVQSLPKQVRKGGVDVLAAVPLSEETEFKVELFVKPVI   60
AHB33010.1          MACTAKPACTAKPGRSPRSQPTRVQSLPKQVRKGGVDVLAAVPLSEETEFKVELFVKPVI   60
AHB33015.1          MACTAKPACTAKPGRSPRSQPTRVQSLPKQVRKGGVDVLAAVPLSEETEFKVELFVKPVI   60
ACX47159.1          MACTAKPACTAKPGRSPRSQPTRVQSLPKQVRKGGVDVLAAVPLSEETEFKVELFVKPVI   60
ACP27656.1          MACTAKPACTAKPGRSPRSQPTRVQSLPKQVRKGGVDVLAAVPLSEETEFKVELFVKPVI   60
ARQ15016.1          MACTAKPACTAKPGRSPRSQPTRVQSLPKQVRKGGVDVLAAVPLSEETEFKVELFVKPVI   60
ARQ15121.1          MACTAKPACTAKPGRSPRSQPARVQSLPKQVRKGGVDVLAAVPLSEETEFKVELFVKPVI   60
AHB32985.1          MACTAKPACTAKPGRSPRSQPARVQSLPKQVRKGGVDVLAAVPLSEETEFKVELFVKPVI   60
AHB32995.1          MACTAKPACTAKPGRSPRSQPARVQSLPKQVRKGGVDVLAAVPLSEETEFKVELFVKPVI   60
AHB33005.1          MACTAKPACTAKPGRSPRSQPARVQSLPKQVRKGGVDVLAAVPLSEETEFKVELFVKPVI   60
ADD50981.1          MACTAKPACTPKPGRSPRSQPTRVQSLPKQVRKGGVDVLAAVPLSEETEFKVELFVKPVI   60
ADD50996.1          MACTAKPACTPKPGRSPRSQPTRVQSLPKQVRKGGVDVLAAVPLSEETEFKVELFVKPVI   60
ADD51021.1          MACTAKPACTPKPGRSPRSQPTRVQSLPKQVRKGGVDVLAAVPLSEETEFKVELFVKPVI   60
ADD51111.1          MACTAKPACTPKPGRSPRSQPTRVQSLPKQVRKGGVDVLAAVPLSEETEFKVELFVKPVI   60
ACR43498.1          MACTAKPACTPKPGRSPRSQPTRVQSLPKQVRKGGVDVLAAVPLSEETEFKVELFVKPVI   60
ACF19280.1          MACTAKPACTAKPGRSPRSQPTRVQSLPKQVRKGGVDVLAAVPLSEETEFKVELFVKPVI   60
ACB12036.1          MACTAKPACTAKPGRSPRSQPTRVQSLPKQVRKGGVDVLAAVPLSEETEFKVELFVKPVI   60
                    ****** *** **********:*:***************:********: :*********

ADD51146.1          GNAEGTTPHYWSISSPLKAAEAANVTPDADTTVCYSLSQVAPPDIPNQVSECDMLIWELY   120
ADD51151.1          GNAEGTTPHYWSISSPLKAAEAANVTPDADTTVCYSLSQVAPPDIPNQVSECDMLIWELY   120
ADD51156.1          GNAEGTTPHYWSISSPLKAAEAANVTPDADTTVCYSLSQVAPPDIPNQVSECDMLIWELY   120
WIV69164.1          GNAEGTTPHYWSISSPLKAAETANVTPDADTTVCYSLSQVAPPDIPNQVSECDMLIWELY   120
ARX17310.1          GNAEGTTPHYWSISSPLKAAETANVTPDADTTVCYSLSQVAPPDIPNQVSECDMLIWELY   120
ARQ15101.1          GNAEGTTPHYWSISSPLKAAETANVTPDADTTVCYSLSQVAPPDIPNQVSECDMLIWELY   120
ARQ15131.1          GNAEGTTPHYWSISSPLKAAEAANVTPDADTTVCYSLSQVAPPDIPNQVSECDMLIWELY   120
ARQ15046.1          GNAEGTTPHYWSISSPLKAAEAANVTPDADTTVCYSLSQVAPPDIPNQVSECDMLIWELY   120
ARQ15091.1          GNAEGTTPHYWSISSPLKAAEAANVTPDADTTVCYSLSQVAPPDIPNQVSECDMLIWELY   120
ARQ15126.1          GNAEGTTPHYWSISSPLKAAEAANVTPDADTTVCYSLSQVAPPDIPNQVSECDMLIWELY   120
AXU41991.1          GNAEGTTPHYWSISSPLKAAEAANVTPDADTTVCYSLSQVAPPDIPNQVSECDMLIWELY   120
AXU41996.1          GNAEGTTPHYWSISSPLKAAEAANVTPDADTTVCYSLSQVAPPDIPNQVSECDMLIWELY   120
AXU42001.1          GNAEGTTPHYWSISSPLKAAEAANVTPDADTTVCYSLSQVAPPDIPNQVSECDMLIWELY   120
AMS36878.1          GNAEGTTPHYWSISSPLKAAEAANVTPDADTTVCYSLSQVAPPDIPNQVSECDMLIWELY   120
ADD50941.1          GNAEGTTPHYWSISSPLKAAEAANVTPDADTTVCYSLSQVAPPDIPNQVSECDMLIWELY   120
ACF19290.1          GNAEGTTPHYWSISSPLKAAEAANVTPDADTTVCYSLSQVAPPDIPNQVSECDMLIWELY   120
UJY54021.1          GNAEGTTPHYWSISSPLKAAEAANVTPDADTTVCYSLSQVAPPDIPNQVSECDMLIWELY   120
ADD50921.1          GNAEGTTPHYWSISSPLKAAEAANVTPDADTTVCYSLSQVAPPDIPNQVSECDMLIWELY   120
ADD50926.1          GNAEGTTPHYWSISSPLKAAEAANVTPDADTTVCYSLSQVAPPDIPNQVSECDMLIWELY   120
ADD50931.1          GNAEGTTPHYWSISSPLKAAEAANVTPDADTTVCYSLSQVAPPDIPNQVSECDMLIWELY   120
ADD50936.1          GNAEGTTPHYWSISSPLKAAEAANVTPDADTTVCYSLSQVAPPDIPNQVSECDMLIWELY   120
ACF19275.1          GNAEGTTPHYWSISSPLKAAEAANVTPDADTTVCYSLSQVAPPDIPNQVSECDMLIWELY   120
ACX47164.1          GNAEGTTPHYWSISSPLKTAEAANVTPDADTTVCYSLSQVAPPDIPNQVSECDMLIWELY   120
ACP27651.1          GNAEGTTPHYWSISSPLKTAETANVTPDADTTVCYSLSQVVPPDIPNQVSECDMLIWELY   120
AID07487.1          GNAEGTTPHYWSISSPLKTAEAANVTPDADTTVCYSLSQVAPPDIPNQVSECDMLIWELY   120
ACX47144.1          GNAEGTTPHYWSISSPLKTAETANVTPDADTTVCYSLSQVAPPDIPNQVSECDMIIWELY   120
ARX17280.1          GNAEGTTPHYWSISSPLKTAETANVTPDADTTVCYSLSQVAPPDIPNQVSECDMLIWELY   120
ADX89640.1          GNAEGTTPHYWSISSPLKTAETANVTPDADTTVCYSLSQVAPPDIPNQVSECDMLIWELY   120
ACX47139.1          GNAEGTTPHYWSISSPLKTAETANVTPDADTTVCYSLSQVAPPDIPNQVSECDMLIWELY   120
ARQ15036.1          GNAEGTTPHYWSISSPLKTAKTANVTPDADTTVCYSLSQVAPPDIPNQVSECDMLIWELY   120
ARQ14997.1          GNAEGTTPHYWSISSPLKGAEAANVTPDADTTVCYSLSQVAPPDIPNQVSECDMLIWELY   120
ARQ15002.1          GNAEGTTPHYWSISSPLKGAEAANVTPDADTTVCYSLSQVAPPDIPNQVSECDMLIWELY   120
ARQ14967.1          GNAEGTTPHYWSISSPLKTAETANVTPDADTTVCYSLSQVAPPDIPNQVSECDMLIWELY   120
ARQ15007.1          GNAEGTTPHYWSISSPLKTAETANVTPDADTTVCYSLSQVAPPDIPNQVSECDMLIWELY   120
ARQ15011.1          GNAEGTTPHYWSISSPLKTAETANVTPDADTTVCYSLSQVAPPDIPNQVSECDMLIWELY   120
ARQ15021.1          GNAEGTTPHYWSISSPLKTAETANVTPDADTTVCYSLSQVAPPDIPNQVSECDMLIWELY   120
```

ARQ15031.1	GNAEGTTPHYWSISSPLKTAETANVTPDADTTVCYSLSQVAPPDIPNQVSECDMLIWELY	120
ARQ15026.1	GNAEGTTPHYWSISSPLKTAETANVTPDADTTVCYSLSQVAPPDIPNQVSECDMLIWELY	120
ARQ15081.1	GNAEGTTPHYGSISSPLKTAETANVTPDADTTVCYSLSQVAPPDIPNQVSECDMLIWELY	120
AIW01035.1	GNAEGTTPHYWSISSPLKTAETANVTPDADTTVCYSLSQVAPPDIPNQVSECDMLIWELY	120
ADD51091.1	GNAEGTTPHYWSISSPLKTAETANVTPDADTTVCYSLSQVAPPDIPNQVSECDMLIWELY	120
ADD51116.1	GNAEGTTPHYWSISSPLKTAETANVTPDADTTVCYSLSQVAPPDIPNQVSECDMLIWELY	120
YP_001285487.1	GNAEGTTPHYWSISSPLKTAEAANVTPDADTTVCYSLSQVAPPDIPNQVSECDMLIWELY	120
BEJ44995.1	GNAEGTTPHYWSISSPLKTAEAANVTPDADTTVCYSLSQVAPPDIPNQVSECDMLIWELY	120
QQX23397.1	GNAEGTTPHYWSISSPLKTAEAANVTPDADTTVCYSLSQVAPPDIPNQVSECDMLIWELY	120
QCQ73655.1	GNAEGTTPHYWSISSPLKTAEAANVTPDADTTVCYSLSQVAPPDIPNQVSECDMLIWELY	120
QCQ73660.1	GNAEGTTPHYWSISSPLKTAEAANVTPDADTTVCYSLSQVAPPDIPNQVSECDMLIWELY	120
QCQ73665.1	GNAEGTTPHYWSISSPLKTAEAANVTPDADTTVCYSLSQVAPPDIPNQVSECDMLIWELY	120
AXU41951.1	GNAEGTTPHYWSISSPLKTAEAANVTPDADTTVCYSLSQVAPPDIPNQVSECDMLIWELY	120
AXU41956.1	GNAEGTTPHYWSISSPLKTAEAANVTPDADTTVCYSLSQVAPPDIPNQVSECDMLIWELY	120
AXU41961.1	GNAEGTTPHYWSISSPLKTAEAANVTPDADTTVCYSLSQVAPPDIPNQVSECDMLIWELY	120
AXU41966.1	GNAEGTTPHYWSISSPLKTAEAANVTPDADTTVCYSLSQVAPPDIPNQVSECDMLIWELY	120
AXU41971.1	GNAEGTTPHYWSISSPLKTAEAANVTPDADTTVCYSLSQVAPPDIPNQVSECDMLIWELY	120
AXU41976.1	GNAEGTTPHYWSISSPLKTAEAANVTPDADTTVCYSLSQVAPPDIPNQVSECDMLIWELY	120
AXU41981.1	GNAEGTTPHYWSISSPLKTAEAANVTPDADTTVCYSLSQVAPPDIPNQVSECDMLIWELY	120
AXU41986.1	GNAEGTTPHYWSISSPLKTAEAANVTPDADTTVCYSLSQVAPPDIPNQVSECDMLIWELY	120
ARQ14987.1	GNAEGTTPHYWSISSPLKTAEAANVTPDADTTVCYSLSQVAPPDIPNQVSECDMLIWELY	120
ARQ15041.1	GNAEGTTPHYWSISSPLKTAEAANVTPDADTTVCYSLSQVAPPDIPNQVSECDMLIWELY	120
ARQ15051.1	GNAEGTTPHYWSISSPLKTAEAANVTPDADTTVCYSLSQVAPPDIPNQVSECDMLIWELY	120
ARQ15106.1	GNAEGTTPHYWSISSPLKTAEAANVTPDADTTVCYSLSQVAPPDIPNQVSECDMLIWELY	120
ARQ15111.1	GNAEGTTPHYWSISSPLKTAEAANVTPDADTTVCYSLSQVAPPDIPNQVSECDMLIWELY	120
AVK92965.1	GNAEGTTPHYWSISSPLKTAEAANVTPDADTTVCYSLSQVAPPDIPNQVSECDMLIWELY	120
ARX17290.1	GNAEGTTPHYWSISSPLKTAEAANVTPDADTTVCYSLSQVAPPDIPNQVSECDMLIWELY	120
ARX17315.1	GNAEGTTPHYWSISSPLKTAEAANVTPDADTTVCYSLSQVAPPDIPNQVSECDMLIWELY	120
ARX17320.1	GNAEGTTPHYWSISSPLKTAEAANVTPDADTTVCYSLSQVAPPDIPNQVSECDMLIWELY	120
ARX17330.1	GNAEGTTPHYWSISSPLKTAEAANVTPDADTTVCYSLSQVAPPDIPNQVSECDMLIWELY	120
ARX17335.1	GNAEGTTPHYWSISSPLKTAEAANVTPDADTTVCYSLSQVAPPDIPNQVSECDMLIWELY	120
AOW41301.1	GNAEGTTPHYWSISSPLKTAEAANVTPDADTTVCYSLSQVAPPDIPNQVSECDMLIWELY	120
AMO25748.1	GNAEGTTPHYWSISSPLKTAEAANVTPDADTTVCYSLSQVAPPDIPNQVSECDMLIWELY	120
AJP06396.1	GNAEGTTPHYWSISSPLKTAEAANVTPDADTTVCYSLSQVAPPDIPNQVSECDMLIWELY	120
AHB32990.1	GNAEGTTPHYWSISSPLKTAEAANVTPDADTTVCYSLSQVAPPDIPNQVSECDMLIWELY	120
AHB33000.1	GNAEGTTPHYWSISSPLKTAEAANVTPDADTTVCYSLSQVAPPDIPNQVSECDMLIWELY	120
ADD50946.1	GNAEGTTPHYWSISSPLKTAEAANVTPDADTTVCYSLSQVAPPDIPNQVSECDMLIWELY	120
ADD50951.1	GNAEGTTPHYWSISSPLKTAEAANVTPDADTTVCYSLSQVAPPDIPNQVSECDMLIWELY	120
ADD50956.1	GNAEGTTPHYWSISSPLKTAEAANVTPDADTTVCYSLSQVAPPDIPNQVSECDMLIWELY	120
ADD50961.1	GNAEGTTPHYWSISSPLKTAEAANVTPDADTTVCYSLSQVAPPDIPNQVSECDMLIWELY	120
ADD50966.1	GNAEGTTPHYWSISSPLKTAEAANVTPDADTTVCYSLSQVAPPDIPNQVSECDMLIWELY	120
ADD50971.1	GNAEGTTPHYWSISSPLKTAEAANVTPDADTTVCYSLSQVAPPDIPNQVSECDMLIWELY	120
ADD50976.1	GNAEGTTPHYWSISSPLKTAEAANVTPDADTTVCYSLSQVAPPDIPNQVSECDMLIWELY	120
ADD50986.1	GNAEGTTPHYWSISSPLKTAEAANVTPDADTTVCYSLSQVAPPDIPNQVSECDMLIWELY	120
ADD50991.1	GNAEGTTPHYWSISSPLKTAEAANVTPDADTTVCYSLSQVAPPDIPNQVSECDMLIWELY	120
ADD51001.1	GNAEGTTPHYWSISSPLKTAEAANVTPDADTTVCYSLSQVAPPDIPNQVSECDMLIWELY	120
ADD51006.1	GNAEGTTPHYWSISSPLKTAEAANVTPDADTTVCYSLSQVAPPDIPNQVSECDMLIWELY	120
ADD51011.1	GNAEGTTPHYWSISSPLKTAEAANVTPDADTTVCYSLSQVAPPDIPNQVSECDMLIWELY	120
ADD51016.1	GNAEGTTPHYWSISSPLKTAEAANVTPDADTTVCYSLSQVAPPDIPNQVSECDMLIWELY	120
ADD51026.1	GNAEGTTPHYWSISSPLKTAEAANVTPDADTTVCYSLSQVAPPDIPNQVSECDMLIWELY	120
ADD51031.1	GNAEGTTPHYWSISSPLKTAEAANVTPDADTTVCYSLSQVAPPDIPNQVSECDMLIWELY	120
ADD51036.1	GNAEGTTPHYWSISSPLKTAEAANVTPDADTTVCYSLSQVAPPDIPNQVSECDMLIWELY	120
ADD51041.1	GNAEGTTPHYWSISSPLKTAEAANVTPDADTTVCYSLSQVAPPDIPNQVSECDMLIWELY	120
ADD51046.1	GNAEGTTPHYWSISSPLKTAEAANVTPDADTTVCYSLSQVAPPDIPNQVSECDMLIWELY	120
ADD51051.1	GNAEGTTPHYWSISSPLKTAEAANVTPDADTTVCYSLSQVAPPDIPNQVSECDMLIWELY	120
ADD51056.1	GNAEGTTPHYWSISSPLKTAEAANVTPDADTTVCYSLSQVAPPDIPNQVSECDMLIWELY	120
ADD51061.1	GNAEGTTPHYWSISSPLKTAEAANVTPDADTTVCYSLSQVAPPDIPNQVSECDMLIWELY	120
ADD51066.1	GNAEGTTPHYWSISSPLKTAEAANVTPDADTTVCYSLSQVAPPDIPNQVSECDMLIWELY	120
ADD51071.1	GNAEGTTPHYWSISSPLKTAEAANVTPDADTTVCYSLSQVAPPDIPNQVSECDMLIWELY	120
ADD51076.1	GNAEGTTPHYWSISSPLKTAEAANVTPDADTTVCYSLSQVAPPDIPNQVSECDMLIWELY	120
ADD51081.1	GNAEGTTPHYWSISSPLKTAEAANVTPDADTTVCYSLSQVAPPDIPNQVSECDMLIWELY	120
ADD51086.1	GNAEGTTPHYWSISSPLKTAEAANVTPDADTTVCYSLSQVAPPDIPNQVSECDMLIWELY	120
ADD51096.1	GNAEGTTPHYWSISSPLKTAEAANVTPDADTTVCYSLSQVAPPDIPNQVSECDMLIWELY	120
ADD51101.1	GNAEGTTPHYWSISSPLKTAEAANVTPDADTTVCYSLSQVAPPDIPNQVSECDMLIWELY	120
ADD51106.1	GNAEGTTPHYWSISSPLKTAEAANVTPDADTTVCYSLSQVAPPDIPNQVSECDMLIWELY	120
ADD51121.1	GNAEGTTPHYWSISSPLKTAEAANVTPDADTTVCYSLSQVAPPDIPNQVSECDMLIWELY	120
ADD51126.1	GNAEGTTPHYWSISSPLKTAEAANVTPDADTTVCYSLSQVAPPDIPNQVSECDMLIWELY	120

```
ADD51131.1          GNAEGTTPHYWSISSPLKTAEAANVTPDADTTVCYSLSQVAPPDIPNQVSECDMLIWELY  120
ADD51136.1          GNAEGTTPHYWSISSPLKTAEAANVTPDADTTVCYSLSQVAPPDIPNQVSECDMLIWELY  120
ADD51141.1          GNAEGTTPHYWSISSPLKTAEAANVTPDADTTVCYSLSQVAPPDIPNQVSECDMLIWELY  120
ACW84416.1          GNAEGTTPHYWSISSPLKTAEAANVTPDADTTVCYSLSQVAPPDIPNQVSECDMLIWELY  120
ACX47149.1          GNAEGTTPHYWSISSPLKTAEAANVTPDADTTVCYSLSQVAPPDIPNQVSECDMLIWELY  120
ACX47154.1          GNAEGTTPHYWSISSPLKTAEAANVTPDADTTVCYSLSQVAPPDIPNQVSECDMLIWELY  120
ACR43496.1          GNAEGTTPHYWSISSPLKTAEAANVTPDADTTVCYSLSQVAPPDIPNQVSECDMLIWELY  120
ACR43497.1          GNAEGTTPHYWSISSPLKTAEAANVTPDADTTVCYSLSQVAPPDIPNQVSECDMLIWELY  120
ACR43499.1          GNAEGTTPHYWSISSPLKTAEAANVTPDADTTVCYSLSQVAPPDIPNQVSECDMLIWELY  120
ACF19270.1          GNAEGTTPHYWSISSPLKTAEAANVTPDADTTVCYSLSQVAPPDIPNQVSECDMLIWELY  120
ACF19285.1          GNAEGTTPHYWSISSPLKTAEAANVTPDADTTVCYSLSQVAPPDIPNQVSECDMLIWELY  120
ACB12031.1          GNAEGTTPHYWSISSPLKTAEAANVTPDADTTVCYSLSQVAPPDIPNQVSECDMLIWELY  120
ABY26549.1          GNAEGTTPHYWSISSPLKTAEAANVTPDADTTVCYSLSQVAPPDIPNQVSECDMLIWELY  120
A5HBD5.1            GNAEGTTPHYWSISSPLKTAEAANVTPDADTTVCYSLSQVAPPDIPNQVSECDMLIWELY  120
ABQ09289.1          GNAEGTTPHYWSISSPLKTAEAANVTPDADTTVCYSLSQVAPPDIPNQVSECDMLIWELY  120
ABQ09296.1          GNAEGTTPHYWSISSPLKTAEAANVTPDADTTVCYSLSQVAPPDIPNQVSECDMLIWELY  120
ABQ09301.1          GNAEGTTPHYWSISSPLKTAEAANVTPDADTTVCYSLSQVAPPDIPNQVSECDMLIWELY  120
ABQ09306.1          GNAEGTTPHYWSISSPLKTAEAANVTPDADTTVCYSLSQVAPPDIPNQVSECDMLIWELY  120
ABQ09311.1          GNAEGTTPHYWSISSPLKTAEAANVTPDADTTVCYSLSQVAPPDIPNQVSECDMLIWELY  120
ABQ09316.1          GNAEGTTPHYWSISSPLKTAEAANVTPDADTTVCYSLSQVAPPDIPNQVSECDMLIWELY  120
QQX23402.1          GNAEGTTPHYWSISSPLKTAETANVTPDADTTVCYSLSQVAPPDIPNQVSECDMLIWELY  120
QJR84015.1          GNAEGTTPHYWSISSPLKTAETANVTPDADTTVCYSLSQVAPPDIPNQVSECDMLIWELY  120
QJR84020.1          GNAEGTTPHYWSISSPLKTAETANVTPDADTTVCYSLSQVAPPDIPNQVSECDMLIWELY  120
ARQ14962.1          GNAEGTTPHYWSISSPLKTAETANVTPDADTTVCYSLSQVAPPDIPNQVSECDMLIWELY  120
ARQ14972.1          GNAEGTTPHYWSISSPLKTAETANVTPDADTTVCYSLSQVAPPDIPNQVSECDMLIWELY  120
ARQ14977.1          GNAEGTTPHYWSISSPLKTAETANVTPDADTTVCYSLSQVAPPDIPNQVSECDMLIWELY  120
ARQ14982.1          GNAEGTTPHYWSISSPLKTAETANVTPDADTTVCYSLSQVAPPDIPNQVSECDMLIWELY  120
ARQ14992.1          GNAEGTTPHYWSISSPLKTAETANVTPDADTTVCYSLSQVAPPDIPNQVSECDMLIWELY  120
ARQ15056.1          GNAEGTTPHYWSISSPLKTAETANVTPDADTTVCYSLSQVAPPDIPNQVSECDMLIWELY  120
ARQ15061.1          GNAEGTTPHYWSISSPLKTAETANVTPDADTTVCYSLSQVAPPDIPNQVSECDMLIWELY  120
ARQ15066.1          GNAEGTTPHYWSISSPLKTAETANVTPDADTTVCYSLSQVAPPDIPNQVSECDMLIWELY  120
ARQ15071.1          GNAEGTTPHYWSISSPLKTAETANVTPDADTTVCYSLSQVAPPDIPNQVSECDMLIWELY  120
ARQ15076.1          GNAEGTTPHYWSISSPLKTAETANVTPDADTTVCYSLSQVAPPDIPNQVSECDMLIWELY  120
ARQ15086.1          GNAEGTTPHYWSISSPLKTAETANVTPDADTTVCYSLSQVAPPDIPNQVSECDMLIWELY  120
ARQ15096.1          GNAEGTTPHYWSISSPLKTAETANVTPDADTTVCYSLSQVAPPDIPNQVSECDMLIWELY  120
ARQ15116.1          GNAEGTTPHYWSISSPLKTAETANVTPDADTTVCYSLSQVAPPDIPNQVSECDMLIWELY  120
ARX17285.1          GNAEGTTPHYWSISSPLKTAETANVTPDADTTVCYSLSQVAPPDIPNQVSECDMLIWELY  120
ARX17295.1          GNAEGTTPHYWSISSPLKTAETANVTPDADTTVCYSLSQVAPPDIPNQVSECDMLIWELY  120
ARX17300.1          GNAEGTTPHYWSISSPLKTAETANVTPDADTTVCYSLSQVAPPDIPNQVSECDMLIWELY  120
ARX17305.1          GNAEGTTPHYWSISSPLKTAETANVTPDADTTVCYSLSQVAPPDIPNQVSECDMLIWELY  120
ARX17325.1          GNAEGTTPHYWSISSPLKTAETANVTPDADTTVCYSLSQVAPPDIPNQVSECDMLIWELY  120
AHB33010.1          GNAEGTTPHYWSISSPLKTAETANVTPDADTTVCYSLSQVAPPDIPNQVSECDMLIWELY  120
AHB33015.1          GNAEGTTPHYWSISSPLKTAETANVTPDADTTVCYSLSQVAPPDIPNQVSECDMLIWELY  120
ACX47159.1          GNAEGTTPHYWSISSPLKTAETANVTPDADTTVCYSLSQVAPPDIPNQVSECDMLIWELY  120
ACP27656.1          GNAEGTTPHYWSISSPLKTAETANVTPDADTTVCYSLSQVAPPDIPNQVSECDMLIWELY  120
ARQ15016.1          GNAEGTTPHYWSISSPLKTAEAANVTPDADTTVCYSLSQVAPPDIPNQVSECDMLIWELY  120
ARQ15121.1          GNAEGTTPHYWSISSPLKTAEAANVTPDADTTVCYSLSQVAPPDIPNQVSECDMLIWELY  120
AHB32985.1          GNAEGTTPHYWSISSPLKTAEAANVTPDADTTVCYSLSQVAPPDIPNQVSECDMLIWELY  120
AHB32995.1          GNAEGTTPHYWSISSPLKTAEAANVTPDADTTVCYSLSQVAPPDIPNQVSECDMLIWELY  120
AHB33005.1          GNAEGTTPHYWSISSPLKTAEAANVTPDADTTVCYSLSQVAPPDIPNQVSECDMLIWELY  120
ADD50981.1          GNAEGTTPHYWSISSPLKTAEAANVTPDADTTVCYSLSQVAPPDIPNQVSECDMLIWELY  120
ADD50996.1          GNAEGTTPHYWSISSPLKTAEAANVTPDADTTVCYSLSQVAPPDIPNQVSECDMLIWELY  120
ADD51021.1          GNAEGTTPHYWSISSPLKTAEAANVTPDADTTVCYSLSQVAPPDIPNQVSECDMLIWELY  120
ADD51111.1          GNAEGTTPHYWSISSPLKTAEAANVTPDADTTVCYSLSQVAPPDIPNQVSECDMLIWELY  120
ACR43498.1          GNAEGTTPHYWSISSPLKTAEAANVTPDADTTVCYSLSQVAPPDIPNQVSECDMLIWELY  120
ACF19280.1          GNAEGTTPHYWSISSPLKTAEAANVTPDADTTVCYSLSQVAPPDIPNQVSECDMLIWELY  120
ACB12036.1          GNAEGTTPHYWSISSPLKGAEAANVTPDADTTVCYSLSQVAPPDIPNQVSECDMLIWELY  120
                    ********** ******* *::******************.*************:*****

ADD51146.1          RMETEVLVLPVLNAGILNTGGVGGIAGPQLYFWAVGGQPLDVLGLAPTEKYKGPTQYTVN  180
ADD51151.1          RMETEVLVLPVLNAGILNTGGVGGIAGPQLYFWAVGGQPLDVLGLAPTEKYKGPTQYTVN  180
ADD51156.1          RMETEVLVLPVLNAGILNTGGVGGIAGPQLYFWAVGGQPLDVLGLAPTEKYKGPTQYTVN  180
WIV69164.1          RMETEVLVVPFLNAGILNTGGVGGIAGPQLYFWAVGGQPLDVLGLAPTEKYKGPAQYTVN  180
ARX17310.1          RMETEVLVVPFLNAGILNTGGVGGIAGPQLYFWAVGGQPLDVLGLAPTEKYKGPAQYTVN  180
ARQ15101.1          RMETEVLVVPFLNAGILTTGGVGGIAGPQLYFWAVGGQPLDVLGLAPTEKYKGPAQYTVN  180
ARQ15131.1          RMETEVLVLPFLNAGVLNTGGVGGIAGPQLYFWAVGGQPLDVLGLAPTEKYKGPAQYTVN  180
ARQ15046.1          RMETEVLVLPFLNAGVLTTGGVGGIAGPQLYFWAVGGQPLDVLGLAPTEKYKGPAQYTVN  180
```

ARQ15091.1	RMETEVLVLPFLNAGVLTTGGVGGIAGPQLYFWAVGGQPLDVLGLAPTEKYKGPAQYTVN	180
ARQ15126.1	RMETEVLVLPFLNAGVLTTGGVGGIAGPQLYFWAVGGQPLDVLGLAPTEKYKGPAQYTVN	180
AXU41991.1	RMETEVLVLPFLNAGVLNTGGVGGIAGPQLYFWAVGGQPLDVLGLAPTEKYKGPAQYTVN	180
AXU41996.1	RMETEVLVLPFLNAGVLNTGGVGGIAGPQLYFWAVGGQPLDVLGLAPTEKYKGPAQYTVN	180
AXU42001.1	RMETEVLVLPFLNAGVLNTGGVGGIAGPQLYFWAVGGQPLDVLGLAPTEKYKGPAQYTVN	180
AMS36878.1	RMETEVLVLPFLNAGVLNTGGVGGIAGPQLYFWAVGGQPLDVLGLAPTEKYKGPAQYTVN	180
ADD50941.1	RMETEVLVLPFLNAGVLNTGGVGGIAGPQLYFWAVGGQPLDVLGLAPTEKYKGPAQYTVN	180
ACF19290.1	RMETEVLVLPFLNAGVLNTGGVGGIAGPQLYFWAVGGQPLDVLGLAPTEKYKGPAQYTVN	180
UJY54021.1	RMETEVLVLPFLNAGVLNTGGVGGIAGPQLYFWAVGGQPLDVLGIAPTEKYKGPAQYTVN	180
ADD50921.1	RMETEVLVLPFLNAGVLNTGGVGGIAGPQLYFWAVGGQPLDVLGIAPTEKYKGPAQYTVN	180
ADD50926.1	RMETEVLVLPFLNAGVLNTGGVGGIAGPQLYFWAVGGQPLDVLGIAPTEKYKGPAQYTVN	180
ADD50931.1	RMETEVLVLPFLNAGVLNTGGVGGIAGPQLYFWAVGGQPLDVLGIAPTEKYKGPAQYTVN	180
ADD50936.1	RMETEVLVLPFLNAGVLNTGGVGGIAGPQLYFWAVGGQPLDVLGIAPTEKYKGPAQYTVN	180
ACF19275.1	RMETEVLVLPFLNAGVLNTGGVGGIAGPQLYFWAVGGQPLDVLGIAPTEKYKGPAQYTVN	180
ACX47164.1	RMETEVLVLPVLNAGILTTGGVGGIAGPQLYFWAVGGQPLDVLGLAPTEKYKGPAQYTVN	180
ACP27651.1	RMETEVLVLPVLNAGILTTGGVGGIAGPQLYFWAVGGQPLDVLGLAPTEKYKGPAQYTIN	180
AID07487.1	RMETEVLVLPVLNAGILNTGGVGGIAGPQLYFWAVGGQPLDVLGLAPTEKYKGPAQYTVN	180
ACX47144.1	RMETEVLVLPVLNAGILTTGGVGGIAGPQLYFWAVGGQPLDVLGLAPTEKYKGPAQYTVN	180
ARX17280.1	RMETEVLVLPVLNAGILTTGGVGGIAGPQLYFWAVGGQPLDVLGLAPTEKYKGPAQYTVN	180
ADX89640.1	RMETEVLVLPVLNAGILTTGGVGGIAGPQLYFWAVGGQPLDVLGLAPTEKYKGPAQYTVN	180
ACX47139.1	RMETEVLVLPVLNAGILTTGGVGGIAGPQLYFWAVGGQPLDVLGLAPTEKYKGPAQYTVN	180
ARQ15036.1	RMETEVLVLPVLNAGILTTGGVGGIAGPQLYFWAVGGQPLDVLGLAPTEKYKGPAQYTVN	180
ARQ14997.1	RMETEVLVLPVLNAGILTTGGVGGIAGPQLYFWAVGGQPLDVLGLAPTEKYKGPAQYTVN	180
ARQ15002.1	RMETEVLVLPVLNAGILTTGGVGGIAGPQLYFWAVGGQPLDVLGLAPTEKYKGPAQYTVN	180
ARQ14967.1	RMETEVLVLPVLNAGILTTGGVGGIAGPQLYFWAVGGQPLDVLGLAPTEKYKGPAQYTVN	180
ARQ15007.1	RMETEVLVLPVLNAGILTTGGVGGIAGPQLYFWAVGGQPLDVLGLAPTEKYKGPAQYTVN	180
ARQ15011.1	RMETEVLVLPVLNAGILTTGGVGGIAGPQLYFWAVGGQPLDVLGLAPTEKYKGPAQYTVN	180
ARQ15021.1	RMETEVLVLPVLNAGILTTGGVGGIAGPQLYFWAVGGQPLDVLGLAPTEKYKGPAQYTVN	180
ARQ15031.1	RMETEVLVLPVLNAGILTTGGVGGIAGPQLYFWAVGGQPLDVLGLAPTEKYKGPAQYTVN	180
ARQ15026.1	RMETEVLVLPVLNAGILTTGGVGGIAGPQLYFWAVGGQPLDVLGLAPTEKYKGPAQYTVN	180
ARQ15081.1	RMETEVLVLPVLNAGILTTGGVGGIAGPQLYFWAVGGQPLDVLGLAPTEKYKGPAQYTVN	180
AIW01035.1	RMETEVLVLPVLNAGILTTGGVGGIAGPQLYFWAVGGQPLDVLGLAPTEKYKGPAQYTVN	180
ADD51091.1	RMETEVLVLPVLNAGILTTGGVGGIAGPQLYFWAVGGQPLDVLGLAPTEKYKGPAQYTVN	180
ADD51116.1	RMETEVLVLPVLNAGILTTGGVGGIAGPQLYFWAVGGQPLDVLGLAPTEKYKGPAQYTVN	180
YP_001285487.1	RMETEVLVLPVLNAGILTTGGVGGIAGPQLYFWAVGGQPLDVLGLAPTEKYKGPAQYTVN	180
BEJ44995.1	RMETEVLVLPVLNAGILTTGGVGGIAGPQLYFWAVGGQPLDVLGLAPTEKYKGPAQYTVN	180
QQX23397.1	RMETEVLVLPVLNAGILTTGGVGGIAGPQLYFWAVGGQPLDVLGLAPTEKYKGPAQYTVN	180
QCQ73655.1	RMETEVLVLPVLNAGILTTGGVGGIAGPQLYFWAVGGQPLDVLGLAPTEKYKGPAQYTVN	180
QCQ73660.1	RMETEVLVLPVLNAGILTTGGVGGIAGPQLYFWAVGGQPLDVLGLAPTEKYKGPAQYTVN	180
QCQ73665.1	RMETEVLVLPVLNAGILTTGGVGGIAGPQLYFWAVGGQPLDVLGLAPTEKYKGPAQYTVN	180
AXU41951.1	RMETEVLVLPVLNAGILTTGGVGGIAGPQLYFWAVGGQPLDVLGLAPTEKYKGPAQYTVN	180
AXU41956.1	RMETEVLVLPVLNAGILTTGGVGGIAGPQLYFWAVGGQPLDVLGLAPTEKYKGPAQYTVN	180
AXU41961.1	RMETEVLVLPVLNAGILTTGGVGGIAGPQLYFWAVGGQPLDVLGLAPTEKYKGPAQYTVN	180
AXU41966.1	RMETEVLVLPVLNAGILTTGGVGGIAGPQLYFWAVGGQPLDVLGLAPTEKYKGPAQYTVN	180
AXU41971.1	RMETEVLVLPVLNAGILTTGGVGGIAGPQLYFWAVGGQPLDVLGLAPTEKYKGPAQYTVN	180
AXU41976.1	RMETEVLVLPVLNAGILTTGGVGGIAGPQLYFWAVGGQPLDVLGLAPTEKYKGPAQYTVN	180
AXU41981.1	RMETEVLVLPVLNAGILTTGGVGGIAGPQLYFWAVGGQPLDVLGLAPTEKYKGPAQYTVN	180
AXU41986.1	RMETEVLVLPVLNAGILTTGGVGGIAGPQLYFWAVGGQPLDVLGLAPTEKYKGPAQYTVN	180
ARQ14987.1	RMETEVLVLPVLNAGILTTGGVGGIAGPQLYFWAVGGQPLDVLGLAPTEKYKGPAQYTVN	180
ARQ15041.1	RMETEVLVLPVLNAGILTTGGVGGIAGPQLYFWAVGGQPLDVLGLAPTEKYKGPAQYTVN	180
ARQ15051.1	RMETEVLVLPVLNAGILTTGGVGGIAGPQLYFWAVGGQPLDVLGLAPTEKYKGPAQYTVN	180
ARQ15106.1	RMETEVLVLPVLNAGILTTGGVGGIAGPQLYFWAVGGQPLDVLGLAPTEKYKGPAQYTVN	180
ARQ15111.1	RMETEVLVLPVLNAGILTTGGVGGIAGPQLYFWAVGGQPLDVLGLAPTEKYKGPAQYTVN	180
AVK92965.1	RMETEVLVLPVLNAGILTTGGVGGIAGPQLYFWAVGGQPLDVLGLAPTEKYKGPAQYTVN	180
ARX17290.1	RMETEVLVLPVLNAGILTTGGVGGIAGPQLYFWAVGGQPLDVLGLAPTEKYKGPAQYTVN	180
ARX17315.1	RMETEVLVLPVLNAGILTTGGVGGIAGPQLYFWAVGGQPLDVLGLAPTEKYKGPAQYTVN	180
ARX17320.1	RMETEVLVLPVLNAGILTTGGVGGIAGPQLYFWAVGGQPLDVLGLAPTEKYKGPAQYTVN	180
ARX17330.1	RMETEVLVLPVLNAGILTTGGVGGIAGPQLYFWAVGGQPLDVLGLAPTEKYKGPAQYTVN	180
ARX17335.1	RMETEVLVLPVLNAGILTTGGVGGIAGPQLYFWAVGGQPLDVLGLAPTEKYKGPAQYTVN	180
AOW41301.1	RMETEVLVLPVLNAGILTTGGVGGIAGPQLYFWAVGGQPLDVLGLAPTEKYKGPAQYTVN	180
AMO25748.1	RMETEVLVLPVLNAGILTTGGVGGIAGPQLYFWAVGGQPLDVLGLAPTEKYKGPAQYTVN	180
AJP06396.1	RMETEVLVLPVLNAGILTTGGVGGIAGPQLYFWAVGGQPLDVLGLAPTEKYKGPAQYTVN	180
AHB32990.1	RMETEVLVLPVLNAGILTTGGVGGIAGPQLYFWAVGGQPLDVLGLAPTEKYKGPAQYTVN	180
AHB33000.1	RMETEVLVLPVLNAGILTTGGVGGIAGPQLYFWAVGGQPLDVLGLAPTEKYKGPAQYTVN	180
ADD50946.1	RMETEVLVLPVLNAGILTTGGVGGIAGPQLYFWAVGGQPLDVLGLAPTEKYKGPAQYTVN	180
ADD50951.1	RMETEVLVLPVLNAGILTTGGVGGIAGPQLYFWAVGGQPLDVLGLAPTEKYKGPAQYTVN	180
ADD50956.1	RMETEVLVLPVLNAGILTTGGVGGIAGPQLYFWAVGGQPLDVLGLAPTEKYKGPAQYTVN	180

ADD50961.1	RMETEVLVLPVLNAGILTTGGVGGIAGPQLYFWAVGGQPLDVLGLAPTEKYKGPAQYTVN	180
ADD50966.1	RMETEVLVLPVLNAGILTTGGVGGIAGPQLYFWAVGGQPLDVLGLAPTEKYKGPAQYTVN	180
ADD50971.1	RMETEVLVLPVLNAGILTTGGVGGIAGPQLYFWAVGGQPLDVLGLAPTEKYKGPAQYTVN	180
ADD50976.1	RMETEVLVLPVLNAGILTTGGVGGIAGPQLYFWAVGGQPLDVLGLAPTEKYKGPAQYTVN	180
ADD50986.1	RMETEVLVLPVLNAGILTTGGVGGIAGPQLYFWAVGGQPLDVLGLAPTEKYKGPAQYTVN	180
ADD50991.1	RMETEVLVLPVLNAGILTTGGVGGIAGPQLYFWAVGGQPLDVLGLAPTEKYKGPAQYTVN	180
ADD51001.1	RMETEVLVLPVLNAGILTTGGVGGIAGPQLYFWAVGGQPLDVLGLAPTEKYKGPAQYTVN	180
ADD51006.1	RMETEVLVLPVLNAGILTTGGVGGIAGPQLYFWAVGGQPLDVLGLAPTEKYKGPAQYTVN	180
ADD51011.1	RMETEVLVLPVLNAGILTTGGVGGIAGPQLYFWAVGGQPLDVLGLAPTEKYKGPAQYTVN	180
ADD51016.1	RMETEVLVLPVLNAGILTTGGVGGIAGPQLYFWAVGGQPLDVLGLAPTEKYKGPAQYTVN	180
ADD51026.1	RMETEVLVLPVLNAGILTTGGVGGIAGPQLYFWAVGGQPLDVLGLAPTEKYKGPAQYTVN	180
ADD51031.1	RMETEVLVLPVLNAGILTTGGVGGIAGPQLYFWAVGGQPLDVLGLAPTEKYKGPAQYTVN	180
ADD51036.1	RMETEVLVLPVLNAGILTTGGVGGIAGPQLYFWAVGGQPLDVLGLAPTEKYKGPAQYTVN	180
ADD51041.1	RMETEVLVLPVLNAGILTTGGVGGIAGPQLYFWAVGGQPLDVLGLAPTEKYKGPAQYTVN	180
ADD51046.1	RMETEVLVLPVLNAGILTTGGVGGIAGPQLYFWAVGGQPLDVLGLAPTEKYKGPAQYTVN	180
ADD51051.1	RMETEVLVLPVLNAGILTTGGVGGIAGPQLYFWAVGGQPLDVLGLAPTEKYKGPAQYTVN	180
ADD51056.1	RMETEVLVLPVLNAGILTTGGVGGIAGPQLYFWAVGGQPLDVLGLAPTEKYKGPAQYTVN	180
ADD51061.1	RMETEVLVLPVLNAGILTTGGVGGIAGPQLYFWAVGGQPLDVLGLAPTEKYKGPAQYTVN	180
ADD51066.1	RMETEVLVLPVLNAGILTTGGVGGIAGPQLYFWAVGGQPLDVLGLAPTEKYKGPAQYTVN	180
ADD51071.1	RMETEVLVLPVLNAGILTTGGVGGIAGPQLYFWAVGGQPLDVLGLAPTEKYKGPAQYTVN	180
ADD51076.1	RMETEVLVLPVLNAGILTTGGVGGIAGPQLYFWAVGGQPLDVLGLAPTEKYKGPAQYTVN	180
ADD51081.1	RMETEVLVLPVLNAGILTTGGVGGIAGPQLYFWAVGGQPLDVLGLAPTEKYKGPAQYTVN	180
ADD51086.1	RMETEVLVLPVLNAGILTTGGVGGIAGPQLYFWAVGGQPLDVLGLAPTEKYKGPAQYTVN	180
ADD51096.1	RMETEVLVLPVLNAGILTTGGVGGIAGPQLYFWAVGGQPLDVLGLAPTEKYKGPAQYTVN	180
ADD51101.1	RMETEVLVLPVLNAGILTTGGVGGIAGPQLYFWAVGGQPLDVLGLAPTEKYKGPAQYTVN	180
ADD51106.1	RMETEVLVLPVLNAGILTTGGVGGIAGPQLYFWAVGGQPLDVLGLAPTEKYKGPAQYTVN	180
ADD51121.1	RMETEVLVLPVLNAGILTTGGVGGIAGPQLYFWAVGGQPLDVLGLAPTEKYKGPAQYTVN	180
ADD51126.1	RMETEVLVLPVLNAGILTTGGVGGIAGPQLYFWAVGGQPLDVLGLAPTEKYKGPAQYTVN	180
ADD51131.1	RMETEVLVLPVLNAGILTTGGVGGIAGPQLYFWAVGGQPLDVLGLAPTEKYKGPAQYTVN	180
ADD51136.1	RMETEVLVLPVLNAGILTTGGVGGIAGPQLYFWAVGGQPLDVLGLAPTEKYKGPAQYTVN	180
ADD51141.1	RMETEVLVLPVLNAGILTTGGVGGIAGPQLYFWAVGGQPLDVLGLAPTEKYKGPAQYTVN	180
ACW84416.1	RMETEVLVLPVLNAGILTTGGVGGIAGPQLYFWAVGGQPLDVLGLAPTEKYKGPAQYTVN	180
ACX47149.1	RMETEVLVLPVLNAGILTTGGVGGIAGPQLYFWAVGGQPLDVLGLAPTEKYKGPAQYTVN	180
ACX47154.1	RMETEVLVLPVLNAGILTTGGVGGIAGPQLYFWAVGGQPLDVLGLAPTEKYKGPAQYTVN	180
ACR43496.1	RMETEVLVLPVLNAGILTTGGVGGIAGPQLYFWAVGGQPLDVLGLAPTEKYKGPAQYTVN	180
ACR43497.1	RMETEVLVLPVLNAGILTTGGVGGIAGPQLYFWAVGGQPLDVLGLAPTEKYKGPAQYTVN	180
ACR43499.1	RMETEVLVLPVLNAGILTTGGVGGIAGPQLYFWAVGGQPLDVLGLAPTEKYKGPAQYTVN	180
ACF19270.1	RMETEVLVLPVLNAGILTTGGVGGIAGPQLYFWAVGGQPLDVLGLAPTEKYKGPAQYTVN	180
ACF19285.1	RMETEVLVLPVLNAGILTTGGVGGIAGPQLYFWAVGGQPLDVLGLAPTEKYKGPAQYTVN	180
ACB12031.1	RMETEVLVLPVLNAGILTTGGVGGIAGPQLYFWAVGGQPLDVLGLAPTEKYKGPAQYTVN	180
ABY26549.1	RMETEVLVLPVLNAGILTTGGVGGIAGPQLYFWAVGGQPLDVLGLAPTEKYKGPAQYTVN	180
A5HBD5.1	RMETEVLVLPVLNAGILTTGGVGGIAGPQLYFWAVGGQPLDVLGLAPTEKYKGPAQYTVN	180
ABQ09289.1	RMETEVLVLPVLNAGILTTGGVGGIAGPQLYFWAVGGQPLDVLGLAPTEKYKGPAQYTVN	180
ABQ09296.1	RMETEVLVLPVLNAGILTTGGVGGIAGPQLYFWAVGGQPLDVLGLAPTEKYKGPAQYTVN	180
ABQ09301.1	RMETEVLVLPVLNAGILTTGGVGGIAGPQLYFWAVGGQPLDVLGLAPTEKYKGPAQYTVN	180
ABQ09306.1	RMETEVLVLPVLNAGILTTGGVGGIAGPQLYFWAVGGQPLDVLGLAPTEKYKGPAQYTVN	180
ABQ09311.1	RMETEVLVLPVLNAGILTTGGVGGIAGPQLYFWAVGGQPLDVLGLAPTEKYKGPAQYTVN	180
ABQ09316.1	RMETEVLVLPVLNAGILTTGGVGGIAGPQLYFWAVGGQPLDVLGLAPTEKYKGPAQYTVN	180
QQX23402.1	RMETEVLVLPVLNAGILTTGGVGGIAGPQLYFWAVGGQPLDVLGLAPTEKYKGPAQYTVN	180
QJR84015.1	RMETEVLVLPVLNAGILTTGGVGGIAGPQLYFWAVGGQPLDVLGLAPTEKYKGPAQYTVN	180
QJR84020.1	RMETEVLVLPVLNAGILTTGGVGGIAGPQLYFWAVGGQPLDVLGLAPTEKYKGPAQYTVN	180
ARQ14962.1	RMETEVLVLPVLNAGILTTGGVGGIAGPQLYFWAVGGQPLDVLGLAPTEKYKGPAQYTVN	180
ARQ14972.1	RMETEVLVLPVLNAGILTTGGVGGIAGPQLYFWAVGGQPLDVLGLAPTEKYKGPAQYTVN	180
ARQ14977.1	RMETEVLVLPVLNAGILTTGGVGGIAGPQLYFWAVGGQPLDVLGLAPTEKYKGPAQYTVN	180
ARQ14982.1	RMETEVLVLPVLNAGILTTGGVGGIAGPQLYFWAVGGQPLDVLGLAPTEKYKGPAQYTVN	180
ARQ14992.1	RMETEVLVLPVLNAGILTTGGVGGIAGPQLYFWAVGGQPLDVLGLAPTEKYKGPAQYTVN	180
ARQ15056.1	RMETEVLVLPVLNAGILTTGGVGGIAGPQLYFWAVGGQPLDVLGLAPTEKYKGPAQYTVN	180
ARQ15061.1	RMETEVLVLPVLNAGILTTGGVGGIAGPQLYFWAVGGQPLDVLGLAPTEKYKGPAQYTVN	180
ARQ15066.1	RMETEVLVLPVLNAGILTTGGVGGIAGPQLYFWAVGGQPLDVLGLAPTEKYKGPAQYTVN	180
ARQ15071.1	RMETEVLVLPVLNAGILTTGGVGGIAGPQLYFWAVGGQPLDVLGLAPTEKYKGPAQYTVN	180
ARQ15076.1	RMETEVLVLPVLNAGILTTGGVGGIAGPQLYFWAVGGQPLDVLGLAPTEKYKGPAQYTVN	180
ARQ15086.1	RMETEVLVLPVLNAGILTTGGVGGIAGPQLYFWAVGGQPLDVLGLAPTEKYKGPAQYTVN	180
ARQ15096.1	RMETEVLVLPVLNAGILTTGGVGGIAGPQLYFWAVGGQPLDVLGLAPTEKYKGPAQYTVN	180
ARQ15116.1	RMETEVLVLPVLNAGILTTGGVGGIAGPQLYFWAVGGQPLDVLGLAPTEKYKGPAQYTVN	180
ARX17285.1	RMETEVLVLPVLNAGILTTGGVGGIAGPQLYFWAVGGQPLDVLGLAPTEKYKGPAQYTVN	180
ARX17295.1	RMETEVLVLPVLNAGILTTGGVGGIAGPQLYFWAVGGQPLDVLGLAPTEKYKGPAQYTVN	180
ARX17300.1	RMETEVLVLPVLNAGILTTGGVGGIAGPQLYFWAVGGQPLDVLGLAPTEKYKGPAQYTVN	180

```
ARX17305.1          RMETEVLVLPVLNAGILTTGGVGGIAGPQLYFWAVGGQPLDVLGLAPTEKYKGPAQYTVN  180
ARX17325.1          RMETEVLVLPVLNAGILTTGGVGGIAGPQLYFWAVGGQPLDVLGLAPTEKYKGPAQYTVN  180
AHB33010.1          RMETEVLVLPVLNAGILTTGGVGGIAGPQLYFWAVGGQPLDVLGLAPTEKYKGPAQYTVN  180
AHB33015.1          RMETEVLVLPVLNAGILTTGGVGGIAGPQLYFWAVGGQPLDVLGLAPTEKYKGPAQYTVN  180
ACX47159.1          RMETEVLVLPVLNAGILTTGGVGGIAGPQLYFWAVGGQPLDVLGLAPTEKYKGPAQYTVN  180
ACP27656.1          RMETEVLVLPVLNAGILTTGGVGGIAGPQLYFWAVGGQPLDVLGLAPTEKYKGPAQYTVN  180
ARQ15016.1          RMETEVLVLPVLNAGILTTGGVGGIAGPQLYFWAVGGQPLDVLGLAPTEKYKGPAQYTVN  180
ARQ15121.1          RMETEVLVLPVLNAGILTTGGVGGIAGPQLYFWAVGGQPLDVLGLAPTEKYKGPAQYTVN  180
AHB32985.1          RMETEVLVLPVLNAGILTTGGVGGIAGPQLYFWAVGGQPLDVLGLAPTEKYKGPAQYTVN  180
AHB32995.1          RMETEVLVLPVLNAGILTTGGVGGIAGPQLYFWAVGGQPLDVLGLAPTEKYKGPAQYTVN  180
AHB33005.1          RMETEVLVLPVLNAGILTTGGVGGIAGPQLYFWAVGGQPLDVLGLAPTEKYKGPAQYTVN  180
ADD50981.1          RMETEVLVLPVLNAGILTTGGVGGIAGPQLYFWAVGGQPLDVLGLAPTEKYKGPAQYTVN  180
ADD50996.1          RMETEVLVLPVLNAGILTTGGVGGIAGPQLYFWAVGGQPLDVLGLAPTEKYKGPAQYTVN  180
ADD51021.1          RMETEVLVLPVLNAGILTTGGVGGIAGPQLYFWAVGGQPLDVLGLAPTEKYKGPAQYTVN  180
ADD51111.1          RMETEVLVLPVLNAGILTTGGVGGIAGPQLYFWAVGGQPLDVLGLAPTEKYKGPAQYTVN  180
ACR43498.1          RMETEVLVLPVLNAGILTTGGVGGIAGPQLYFWAVGGQPLDVLGLAPTEKYKGPAQYTVN  180
ACF19280.1          RMETEVLVLPVLNAGILTTGGVGGIAGPQLYFWAVGGQPLDVLGLAPTEKYKGPAQYTVN  180
ACB12036.1          RMETEVLVLPVLNAGILTTGGVGGIAGPQLYFWAVGGQPLDVLGLAPTEKYKGPAQYTVN  180
                    ********:*.****:*.**************************:*********:***:*

ADD51146.1          PKTNGTVPHVYSSSETPRARVTNEKYSIESWVADPSRNDNCRYFGRMVGGAATPPVVSFS  240
ADD51151.1          PKTNGTVPHVYSSSETPRARVTNEKYSIESWVADPSRNDNCRYFGRMVGGAATPPVVSFS  240
ADD51156.1          PKTNGTVPHVYSSSETPRARVTNEKYSIESWVADPSRNDNCRYFGRMVGGAATPPVVSFS  240
WIV69164.1          PKTNGTVPHVYSSSETPRARVTNEKYSIESWVADPSRNDNCRYFGRMVGGAATPPVVSFS  240
ARX17310.1          PKTNGTVPHVYSSSETPRARVTNEKYSIESWVADPSRNDNCRYFGRMVGGAATPPVVSFS  240
ARQ15101.1          PKTNGTVPHVYSSSETPRARVTNEKYSIESWVADPSRNDNCRYFGRMVGGAATPPVVSFS  240
ARQ15131.1          PKTNGTVPHVYSSSETPRARVTNEKYSIESWVADPSRNDNCRYFGRMVGGAATPPVVSFS  240
ARQ15046.1          PKTNGTVPHVYSSSDTPRARVTNEKYSIESWVADPSRNDNCRYFGRMVGGAATPPVVSFS  240
ARQ15091.1          PKTNGTVPHVYSSSDTPRARVTNEKYSIESWVADPSRNDNCRYFGRMVGGAATPPVVSFS  240
ARQ15126.1          PKTNGTVPHVYSSSDTPRARVTNEKYSIESWVADPSRNDNCRYFGRMVGGAATPPVVSFS  240
AXU41991.1          PKTNGTVPHVYSSSDTPRARVTNEKYSIESWVADPSRNDNCRYFGRMVGGAATPPVVSFS  240
AXU41996.1          PKTNGTVPHVYSSSDTPRARVTNEKYSIESWVADPSRNDNCRYFGRMVGGAATPPVVSFS  240
AXU42001.1          PKTNGTVPHVYSSSDTPRARVTNEKYSIESWVADPSRNDNCRYFGRMVGGAATPPVVSFS  240
AMS36878.1          PKTNGTVPHVYSSSDTPRARVTNEKYSIESWVADPSRNDNCRYFGRMVGGAATPPVVSFS  240
ADD50941.1          PKTNGTVPHVYSSSDTPRARVTNEKYSIESWVADPSRNDNCRYFGRMVGGAATPPVVSFS  240
ACF19290.1          PKTNGTVPHVYSSSDTPRARVTNEKYSIESWVADPSRNDNCRYFGRMVGGAATPPVVSFS  240
UJY54021.1          PKTNGTVPHVYSSSDTPRARVTNEKYSIESWVADPSRNDNCRYFGRMVGGAATPPVVSFS  240
ADD50921.1          PKTNGTVPHVYSSSDTPRARVTNEKYSIESWVADPSRNDNCRYFGRMVGGAATPPVVSFS  240
ADD50926.1          PKTNGTVPHVYSSSDTPRARVTNEKYSIESWVADPSRNDNCRYFGRMVGGAATPPVVSFS  240
ADD50931.1          PKTNGTVPHVYSSSDTPRARVTNEKYSIESWVADPSRNDNCRYFGRMVGGAATPPVVSFS  240
ADD50936.1          PKTNGTVPHVYSSSDTPRARVTNEKYSIESWVADPSRNDNCRYFGRMVGGAATPPVVSFS  240
ACF19275.1          PKTNGTVPHVYSSSDTPRARVTNEKYSIESWVADPSRNDNCRYFGRMVGGAATPPVVSFS  240
ACX47164.1          PKTNGTVPHVYSSSETPRARVTNEKYSIESWVADPSRNDNCRYFGRVVGGAATPPVVSFS  240
ACP27651.1          PKTNGTVPHVYSSSETPRARVTNEKYSIESWVADPSRNDNCRYFGRMVGGAATPPVVSFS  240
AID07487.1          PKTNGTVPHVYSSSQTPRARVTNEKYSIESWVADPSRNDNCRYFGRMVGGAATPPVVSFS  240
ACX47144.1          PKTNGTVPHVYSSSETPRARVTNEKYSIESWVADPSRNDNCRYFGRMVGGAATPPVVSFS  240
ARX17280.1          PKTNGTVPHVYSSSETPRARVTNEKYSIESWVADPSRNDNCRYFGRMVGGAATPPVVSFS  240
ADX89640.1          PKTNGTVPHVYSSSETPRARVTNEKYSIESWVADPSRNDNCRYFGRMVGGAATPPVVSFS  240
ACX47139.1          PKTNGTVPHVYSSSETPRARVTNEKYSIESWVADPSRNDNCRYFGRMVGGAATPPVVSFS  240
ARQ15036.1          PKTNGTVPHVYSSSETPRARVTNEKYSIESWVADPSRNDNCRYFGRMVGGAATPPVVSFS  240
ARQ14997.1          PKTNGTVPHVYSSSETPRARVTNEKYSIESWVADPSRNDNCRYFGRMVGGAATPPVVSFS  240
ARQ15002.1          PKTNGTVPHVYSSSETPRARVTNEKYSIESWVADPSRNDNCRYFGRMVGGAATPPVVSFS  240
ARQ14967.1          PKTNGTVPHVYSSSETPRARVTNEKYSIESWVADPSRNDNCRYFGRMVGGAATPPVVSFS  240
ARQ15007.1          PKTNGTVPHVYSSSETPRARVTNEKYSIESWVADPSRNDNCRYFGRMVGGAATPPVVSFS  240
ARQ15011.1          PKTNGTVPHVYSSSETPRARVTNEKYSIESWVADPSRNDNCRYFGRMVGGAATPPVVSFS  240
ARQ15021.1          PKTNGTVPHVYSSSETPRARVTNEKYSIESWVADPSRNDNCRYFGRMVGGAATPPVVSFS  240
ARQ15031.1          PKTNGTVPHVYSSSETPRARVTNEKYSIESWVADPSRNDNCRYFGRMVGGAATPPVVSFS  240
ARQ15026.1          PKTNGTVPHVYSSSETPRARVTNEKYSIESWVADPSRNDNCRYFGRMVGGAATPPVVSFS  240
ARQ15081.1          PKTNGTVPHVYSSSETPRARVTNEKYSIESWVADPSRNDNCRYFGRMVGGAATPPVVSFS  240
AIW01035.1          PKTNGTVPHVYSSSETPRARVTNEKYSIESWVADPSRNDNCRYFGRMVGGAATPPVVSFS  240
ADD51091.1          PKTNGTVPHVYSSSETPRARVTNEKYSIESWVADPSRNDNCRYFGRMVGGAATPPVVSFS  240
ADD51116.1          PKTNGTVPHVYSSSETPRARVTNEKYSIESWVADPSRNDNCRYFGRMVGGAATPPVVSFS  240
YP_001285487.1      PKTNGTVPHVYSSSETPRARVTNEKYSIESWVADPSRNDNCRYFGRMVGGAATPPVVSFS  240
BEJ44995.1          PKTNGTVPHVYSSSETPRARVTNEKYSIESWVADPSRNDNCRYFGRMVGGAATPPVVSFS  240
QQX23397.1          PKTNGTVPHVYSSSETPRARVTNEKYSIESWVADPSRNDNCRYFGRMVGGAATPPVVSFS  240
QCQ73655.1          PKTNGTVPHVYSSSETPRARVTNEKYSIESWVADPSRNDNCRYFGRMVGGAATPPVVSFS  240
QCQ73660.1          PKTNGTVPHVYSSSETPRARVTNEKYSIESWVADPSRNDNCRYFGRMVGGAATPPVVSFS  240
```

QCQ73665.1	PKTNGTVPHVYSSSETPRARVTNEKYSIESWVADPSRNDNCRYFGRMVGGAATPPVVSFS	240
AXU41951.1	PKTNGTVPHVYSSSETPRARVTNEKYSIESWVADPSRNDNCRYFGRMVGGAATPPVVSFS	240
AXU41956.1	PKTNGTVPHVYSSSETPRARVTNEKYSIESWVADPSRNDNCRYFGRMVGGAATPPVVSFS	240
AXU41961.1	PKTNGTVPHVYSSSETPRARVTNEKYSIESWVADPSRNDNCRYFGRMVGGAATPPVVSFS	240
AXU41966.1	PKTNGTVPHVYSSSETPRARVTNEKYSIESWVADPSRNDNCRYFGRMVGGAATPPVVSFS	240
AXU41971.1	PKTNGTVPHVYSSSETPRARVTNEKYSIESWVADPSRNDNCRYFGRMVGGAATPPVVSFS	240
AXU41976.1	PKTNGTVPHVYSSSETPRARVTNEKYSIESWVADPSRNDNCRYFGRMVGGAATPPVVSFS	240
AXU41981.1	PKTNGTVPHVYSSSETPRARVTNEKYSIESWVADPSRNDNCRYFGRMVGGAATPPVVSFS	240
AXU41986.1	PKTNGTVPHVYSSSETPRARVTNEKYSIESWVADPSRNDNCRYFGRMVGGAATPPVVSFS	240
ARQ14987.1	PKTNGTVPHVYSSSETPRARVTNEKYSIESWVADPSRNDNCRYFGRMVGGAATPPVVSFS	240
ARQ15041.1	PKTNGTVPHVYSSSETPRARVTNEKYSIESWVADPSRNDNCRYFGRMVGGAATPPVVSFS	240
ARQ15051.1	PKTNGTVPHVYSSSETPRARVTNEKYSIESWVADPSRNDNCRYFGRMVGGAATPPVVSFS	240
ARQ15106.1	PKTNGTVPHVYSSSETPRARVTNEKYSIESWVADPSRNDNCRYFGRMVGGAATPPVVSFS	240
ARQ15111.1	PKTNGTVPHVYSSSETPRARVTNEKYSIESWVADPSRNDNCRYFGRMVGGAATPPVVSFS	240
AVK92965.1	PKTNGTVPHVYSSSETPRARVTNEKYSIESWVADPSRNDNCRYFGRMVGGAATPPVVSFS	240
ARX17290.1	PKTNGTVPHVYSSSETPRARVTNEKYSIESWVADPSRNDNCRYFGRMVGGAATPPVVSFS	240
ARX17315.1	PKTNGTVPHVYSSSETPRARVTNEKYSIESWVADPSRNDNCRYFGRMVGGAATPPVVSFS	240
ARX17320.1	PKTNGTVPHVYSSSETPRARVTNEKYSIESWVADPSRNDNCRYFGRMVGGAATPPVVSFS	240
ARX17330.1	PKTNGTVPHVYSSSETPRARVTNEKYSIESWVADPSRNDNCRYFGRMVGGAATPPVVSFS	240
ARX17335.1	PKTNGTVPHVYSSSETPRARVTNEKYSIESWVADPSRNDNCRYFGRMVGGAATPPVVSFS	240
AOW41301.1	PKTNGTVPHVYSSSETPRARVTNEKYSIESWVADPSRNDNCRYFGRMVGGAATPPVVSFS	240
AMO25748.1	PKTNGTVPHVYSSSETPRARVTNEKYSIESWVADPSRNDNCRYFGRMVGGAATPPVVSFS	240
AJP06396.1	PKTNGTVPHVYSSSETPRARVTNEKYSIESWVADPSRNDNCRYFGRMVGGAATPPVVSFS	240
AHB32990.1	PKTNGTVPHVYSSSETPRARVTNEKYSIESWVADPSRNDNCRYFGRMVGGAATPPVVSFS	240
AHB33000.1	PKTNGTVPHVYSSSETPRARVTNEKYSIESWVADPSRNDNCRYFGRMVGGAATPPVVSFS	240
ADD50946.1	PKTNGTVPHVYSSSETPRARVTNEKYSIESWVADPSRNDNCRYFGRMVGGAATPPVVSFS	240
ADD50951.1	PKTNGTVPHVYSSSETPRARVTNEKYSIESWVADPSRNDNCRYFGRMVGGAATPPVVSFS	240
ADD50956.1	PKTNGTVPHVYSSSETPRARVTNEKYSIESWVADPSRNDNCRYFGRMVGGAATPPVVSFS	240
ADD50961.1	PKTNGTVPHVYSSSETPRARVTNEKYSIESWVADPSRNDNCRYFGRMVGGAATPPVVSFS	240
ADD50966.1	PKTNGTVPHVYSSSETPRARVTNEKYSIESWVADPSRNDNCRYFGRMVGGAATPPVVSFS	240
ADD50971.1	PKTNGTVPHVYSSSETPRARVTNEKYSIESWVADPSRNDNCRYFGRMVGGAATPPVVSFS	240
ADD50976.1	PKTNGTVPHVYSSSETPRARVTNEKYSIESWVADPSRNDNCRYFGRMVGGAATPPVVSFS	240
ADD50986.1	PKTNGTVPHVYSSSETPRARVTNEKYSIESWVADPSRNDNCRYFGRMVGGAATPPVVSFS	240
ADD50991.1	PKTNGTVPHVYSSSETPRARVTNEKYSIESWVADPSRNDNCRYFGRMVGGAATPPVVSFS	240
ADD51001.1	PKTNGTVPHVYSSSETPRARVTNEKYSIESWVADPSRNDNCRYFGRMVGGAATPPVVSFS	240
ADD51006.1	PKTNGTVPHVYSSSETPRARVTNEKYSIESWVADPSRNDNCRYFGRMVGGAATPPVVSFS	240
ADD51011.1	PKTNGTVPHVYSSSETPRARVTNEKYSIESWVADPSRNDNCRYFGRMVGGAATPPVVSFS	240
ADD51016.1	PKTNGTVPHVYSSSETPRARVTNEKYSIESWVADPSRNDNCRYFGRMVGGAATPPVVSFS	240
ADD51026.1	PKTNGTVPHVYSSSETPRARVTNEKYSIESWVADPSRNDNCRYFGRMVGGAATPPVVSFS	240
ADD51031.1	PKTNGTVPHVYSSSETPRARVTNEKYSIESWVADPSRNDNCRYFGRMVGGAATPPVVSFS	240
ADD51036.1	PKTNGTVPHVYSSSETPRARVTNEKYSIESWVADPSRNDNCRYFGRMVGGAATPPVVSFS	240
ADD51041.1	PKTNGTVPHVYSSSETPRARVTNEKYSIESWVADPSRNDNCRYFGRMVGGAATPPVVSFS	240
ADD51046.1	PKTNGTVPHVYSSSETPRARVTNEKYSIESWVADPSRNDNCRYFGRMVGGAATPPVVSFS	240
ADD51051.1	PKTNGTVPHVYSSSETPRARVTNEKYSIESWVADPSRNDNCRYFGRMVGGAATPPVVSFS	240
ADD51056.1	PKTNGTVPHVYSSSETPRARVTNEKYSIESWVADPSRNDNCRYFGRMVGGAATPPVVSFS	240
ADD51061.1	PKTNGTVPHVYSSSETPRARVTNEKYSIESWVADPSRNDNCRYFGRMVGGAATPPVVSFS	240
ADD51066.1	PKTNGTVPHVYSSSETPRARVTNEKYSIESWVADPSRNDNCRYFGRMVGGAATPPVVSFS	240
ADD51071.1	PKTNGTVPHVYSSSETPRARVTNEKYSIESWVADPSRNDNCRYFGRMVGGAATPPVVSFS	240
ADD51076.1	PKTNGTVPHVYSSSETPRARVTNEKYSIESWVADPSRNDNCRYFGRMVGGAATPPVVSFS	240
ADD51081.1	PKTNGTVPHVYSSSETPRARVTNEKYSIESWVADPSRNDNCRYFGRMVGGAATPPVVSFS	240
ADD51086.1	PKTNGTVPHVYSSSETPRARVTNEKYSIESWVADPSRNDNCRYFGRMVGGAATPPVVSFS	240
ADD51096.1	PKTNGTVPHVYSSSETPRARVTNEKYSIESWVADPSRNDNCRYFGRMVGGAATPPVVSFS	240
ADD51101.1	PKTNGTVPHVYSSSETPRARVTNEKYSIESWVADPSRNDNCRYFGRMVGGAATPPVVSFS	240
ADD51106.1	PKTNGTVPHVYSSSETPRARVTNEKYSIESWVADPSRNDNCRYFGRMVGGAATPPVVSFS	240
ADD51121.1	PKTNGTVPHVYSSSETPRARVTNEKYSIESWVADPSRNDNCRYFGRMVGGAATPPVVSFS	240
ADD51126.1	PKTNGTVPHVYSSSETPRARVTNEKYSIESWVADPSRNDNCRYFGRMVGGAATPPVVSFS	240
ADD51131.1	PKTNGTVPHVYSSSETPRARVTNEKYSIESWVADPSRNDNCRYFGRMVGGAATPPVVSFS	240
ADD51136.1	PKTNGTVPHVYSSSETPRARVTNEKYSIESWVADPSRNDNCRYFGRMVGGAATPPVVSFS	240
ADD51141.1	PKTNGTVPHVYSSSETPRARVTNEKYSIESWVADPSRNDNCRYFGRMVGGAATPPVVSFS	240
ACW84416.1	PKTNGTVPHVYSSSETPRARVTNEKYSIESWVADPSRNDNCRYFGRMVGGAATPPVVSFS	240
ACX47149.1	PKTNGTVPHVYSSSETPRARVTNEKYSIESWVADPSRNDNCRYFGRMVGGAATPPVVSFS	240
ACX47154.1	PKTNGTVPHVYSSSETPRARVTNEKYSIESWVADPSRNDNCRYFGRMVGGAATPPVVSFS	240
ACR43496.1	PKTNGTVPHVYSSSETPRARVTNEKYSIESWVADPSRNDNCRYFGRMVGGAATPPVVSFS	240
ACR43497.1	PKTNGTVPHVYSSSETPRARVTNEKYSIESWVADPSRNDNCRYFGRMVGGAATPPVVSFS	240
ACR43499.1	PKTNGTVPHVYSSSETPRARVTNEKYSIESWVADPSRNDNCRYFGRMVGGAATPPVVSFS	240
ACF19270.1	PKTNGTVPHVYSSSETPRARVTNEKYSIESWVADPSRNDNCRYFGRMVGGAATPPVVSFS	240
ACF19285.1	PKTNGTVPHVYSSSETPRARVTNEKYSIESWVADPSRNDNCRYFGRMVGGAATPPVVSFS	240

```
ACB12031.1          PKTNGTVPHVYSSSETPRARVTNEKYSIESWVADPSRNDNCRYFGRMVGGAATPPVVSFS   240
ABY26549.1          PKTNGTVPHVYSSSETPRARVTNEKYSIESWVADPSRNDNCRYFGRMVGGAATPPVVSFS   240
A5HBD5.1            PKTNGTVPHVYSSSETPRARVTNEKYSIESWVADPSRNDNCRYFGRMVGGAATPPVVSFS   240
ABQ09289.1          PKTNGTVPHVYSSSETPRARVTNEKYSIESWVADPSRNDNCRYFGRMVGGAATPPVVSFS   240
ABQ09296.1          PKTNGTVPHVYSSSETPRARVTNEKYSIESWVADPSRNDNCRYFGRMVGGAATPPVVSFS   240
ABQ09301.1          PKTNGTVPHVYSSSETPRARVTNEKYSIESWVADPSRNDNCRYFGRMVGGAATPPVVSFS   240
ABQ09306.1          PKTNGTVPHVYSSSETPRARVTNEKYSIESWVADPSRNDNCRYFGRMVGGAATPPVVSFS   240
ABQ09311.1          PKTNGTVPHVYSSSETPRARVTNEKYSIESWVADPSRNDNCRYFGRMVGGAATPPVVSFS   240
ABQ09316.1          PKTNGTVPHVYSSSETPRARVTNEKYSIESWVADPSRNDNCRYFGRMVGGAATPPVVSFS   240
QQX23402.1          PKTNGTVPHVYSSSETPRARVTNEKYSIESWVADPSRNDNCRYFGRMVGGAATPPVVSFS   240
QJR84015.1          PKTNGTVPHVYSSSETPRARVTNEKYSIESWVADPSRNDNCRYFGRMVGGAATPPVVSFS   240
QJR84020.1          PKTNGTVPHVYSSSETPRARVTNEKYSIESWVADPSRNDNCRYFGRMVGGAATPPVVSFS   240
ARQ14962.1          PKTNGTVPHVYSSSETPRARVTNEKYSIESWVADPSRNDNCRYFGRMVGGAATPPVVSFS   240
ARQ14972.1          PKTNGTVPHVYSSSETPRARVTNEKYSIESWVADPSRNDNCRYFGRMVGGAATPPVVSFS   240
ARQ14977.1          PKTNGTVPHVYSSSETPRARVTNEKYSIESWVADPSRNDNCRYFGRMVGGAATPPVVSFS   240
ARQ14982.1          PKTNGTVPHVYSSSETPRARVTNEKYSIESWVADPSRNDNCRYFGRMVGGAATPPVVSFS   240
ARQ14992.1          PKTNGTVPHVYSSSETPRARVTNEKYSIESWVADPSRNDNCRYFGRMVGGAATPPVVSFS   240
ARQ15056.1          PKTNGTVPHVYSSSETPRARVTNEKYSIESWVADPSRNDNCRYFGRMVGGAATPPVVSFS   240
ARQ15061.1          PKTNGTVPHVYSSSETPRARVTNEKYSIESWVADPSRNDNCRYFGRMVGGAATPPVVSFS   240
ARQ15066.1          PKTNGTVPHVYSSSETPRARVTNEKYSIESWVADPSRNDNCRYFGRMVGGAATPPVVSFS   240
ARQ15071.1          PKTNGTVPHVYSSSETPRARVTNEKYSIESWVADPSRNDNCRYFGRMVGGAATPPVVSFS   240
ARQ15076.1          PKTNGTVPHVYSSSETPRARVTNEKYSIESWVADPSRNDNCRYFGRMVGGAATPPVVSFS   240
ARQ15086.1          PKTNGTVPHVYSSSETPRARVTNEKYSIESWVADPSRNDNCRYFGRMVGGAATPPVVSFS   240
ARQ15096.1          PKTNGTVPHVYSSSETPRARVTNEKYSIESWVADPSRNDNCRYFGRMVGGAATPPVVSFS   240
ARQ15116.1          PKTNGTVPHVYSSSETPRARVTNEKYSIESWVADPSRNDNCRYFGRMVGGAATPPVVSFS   240
ARX17285.1          PKTNGTVPHVYSSSETPRARVTNEKYSIESWVADPSRNDNCRYFGRMVGGAATPPVVSFS   240
ARX17295.1          PKTNGTVPHVYSSSETPRARVTNEKYSIESWVADPSRNDNCRYFGRMVGGAATPPVVSFS   240
ARX17300.1          PKTNGTVPHVYSSSETPRARVTNEKYSIESWVADPSRNDNCRYFGRMVGGAATPPVVSFS   240
ARX17305.1          PKTNGTVPHVYSSSETPRARVTNEKYSIESWVADPSRNDNCRYFGRMVGGAATPPVVSFS   240
ARX17325.1          PKTNGTVPHVYSSSETPRARVTNEKYSIESWVADPSRNDNCRYFGRMVGGAATPPVVSFS   240
AHB33010.1          PKTNGTVPHVYSSSETPRARVTNEKYSIESWVADPSRNDNCRYFGRMVGGAATPPVVSFS   240
AHB33015.1          PKTNGTVPHVYSSSETPRARVTNEKYSIESWVADPSRNDNCRYFGRMVGGAATPPVVSFS   240
ACX47159.1          PKTNGTVPHVYSSSETPRARVTNEKYSIESWVADPSRNDNCRYFGRMVGGAATPPVVSFS   240
ACP27656.1          PKTNGTVPHVYSSSETPRARVTNEKYSIESWVADPSRNDNCRYFGRMVGGAATPPVVSFS   240
ARQ15016.1          PKTNGTVPHVYSSSETPRARVTNEKYSIESWVADPSRNDNCRYFGRMVGGAATPPVVSFS   240
ARQ15121.1          PKTNGTVPHVYSSSETPRARVTNEKYSIESWVADPSRNDNCRYFGRMVGGAATPPVVSFS   240
AHB32985.1          PKTNGTVPHVYSSSETPRARVTNEKYSIESWVADPSRNDNCRYFGRMVGGAATPPVVSFS   240
AHB32995.1          PKTNGTVPHVYSSSETPRARVTNEKYSIESWVADPSRNDNCRYFGRMVGGAATPPVVSFS   240
AHB33005.1          PKTNGTVPHVYSSSETPRARVTNEKYSIESWVADPSRNDNCRYFGRMVGGAATPPVVSFS   240
ADD50981.1          PKTNGTVPHVYSSSETPRARVTNEKYSIESWVADPSRNDNCRYFGRMVGGAATPPVVSFS   240
ADD50996.1          PKTNGTVPHVYSSSETPRARVTNEKYSIESWVADPSRNDNCRYFGRMVGGAATPPVVSFS   240
ADD51021.1          PKTNGTVPHVYSSSETPRARVTNEKYSIESWVADPSRNDNCRYFGRMVGGAATPPVVSFS   240
ADD51111.1          PKTNGTVPHVYSSSETPRARVTNEKYSIESWVADPSRNDNCRYFGRMVGGAATPPVVSFS   240
ACR43498.1          PKTNGTVPHVYSSSETPRARVTNEKYSIESWVADPSRNDNCRYFGRMVGGAATPPVVSFS   240
ACF19280.1          PKTNGTVPHVYSSSETPRARVTNEKYSIESWVADPSRNDNCRYFGRMVGGAATPPVVSFS   240
ACB12036.1          PKTNGTVPHVYSSSETPRARVTNEKYSIESWVADPSRNDNCRYFGRMVGGAATPPVVSFS   240
                    **************:*******************************:*************

ADD51146.1          NNSTIPLLDENGIGILCLQGRLYITCADLLGVNKNRVHTGLSRFFRLHFRQRRVRNPYTI   300
ADD51151.1          NNSTIPLLDENGIGILCLQGRLYITCADLLGVNKNRVHTGLSRFFRLHFRQRRVRNPYTI   300
ADD51156.1          NNSTIPLLDENGIGILCLQGRLYITCADLLGVNKNRVHTGLSRFFRLHFRQRRVRNPYTI   300
WIV69164.1          NNSTIPLLDENGIGILCLQGRLYITCADLLGVNKNRVHTGLSRFFRLHFRQRRVRNPYTI   300
ARX17310.1          NNSTIPLLDENGIGILCLQGRLYITCADLLGVNKNRVHTGLSRFFRLHFRQRRVRNPYTI   300
ARQ15101.1          NNSTIPLLDENGIGILCLQGRLYITCADLLGVNKNRVHTGLSRFFRLHFRQRRVRNPYTI   300
ARQ15131.1          NNSTIPLLDENGIGILCLQGRLYITCADLLGVNKNRVHTGLSRFFRLHFRQRRVRNPYTI   300
ARQ15046.1          NNSTIPLLDENGIGILCLQGRLYITCADLLGVNKNRVHTGLSRFFRLHFRQRRVRNPYTI   300
ARQ15091.1          NNSTIPLLDENGIGILCLQGRLYITCADLLGVNKNRVHTGLSRFFRLHFRQRRVRNPYTI   300
ARQ15126.1          NNSTIPLLDENGIGILCLQGRLYITCADLLGVNKNRVHTGLSRFFRLHFRQRRVRNPYTI   300
AXU41991.1          NNSTIPLLDENGIGILCLQGRLYITCADLLGVNKNRVHTGLSRFFRLHFRQRRVRNPYTI   300
AXU41996.1          NNSTIPLLDENGIGILCLQGRLYITCADLLGVNKNRVHTGLSRFFRLHFRQRRVRNPYTI   300
AXU42001.1          NNSTIPLLDENGIGILCLQGRLYITCADLLGVNKNRVHTGLSRFFRLHFRQRRVRNPYTI   300
AMS36878.1          NNSTIPLLDENGIGILCLQGRLYITCADLLGVNKNRVHTGLSRFFRLHFRQRRVRNPYTI   300
ADD50941.1          NNSTIPLLDENGIGILCLQGRLYITCADLLGVNKNRVHTGLSRFFRLHFRQRRVRNPYTI   300
ACF19290.1          NNSTIPLLDENGIGILCLQGRLYITCADLLGVNKNRVHTGLSRFFRLHFRQRRVRNPYTI   300
UJY54021.1          NNSTIPLLDENGIGILCLQGRLYITCADLLGVNKNRVHTGLSRFFRLHFRQRRVRNPYTI   300
ADD50921.1          NNSTIPLLDENGIGILCLQGRLYITCADLLGVNKNRVHTGLSRFFRLHFRQRRVRNPYTI   300
ADD50926.1          NNSTIPLLDENGIGILCLQGRLYITCADLLGVNKNRVHTGLSRFFRLHFRQRRVRNPYTI   300
```

ADD50931.1	NNSTIPLLDENGIGILCLQGRLYITCADLLGVNKNRVHTGLSRFFRLHFRQRRVRNPYTI	300
ADD50936.1	NNSTIPLLDENGIGILCLQGRLYITCADLLGVNKNRVHTGLSRFFRLHFRQRRVRNPYTI	300
ACF19275.1	NNSTIPLLDENGIGILCLQGRLYITCADLLGVNKNRVHTGLSRFFRLHFRQRRVRNPYTI	300
ACX47164.1	NNSTIPLLDENGIGILCLQGRLYITCADLLGVNKNGVHTGLSRFFRLHFRQRRVRNPYTI	300
ACP27651.1	NNSTIPLLDENGIGILCLQGRLYITCADLLGVNKNRVHTGLSRFFRLHFRQRRVRNPYTI	300
AID07487.1	NNSTIPLLDENGIGILCLQGRLYITCADLLGVNKNRVHTGLSRFFRLHFRQRRVRNPYTI	300
ACX47144.1	NNSTIPLLDENGIGILCLQXRLYITCADLLGVNKNRVHTGLSRFFRLHFRQRRVRNPYTI	300
ARX17280.1	NNSTIPLLDENGIGILCLQGRLYITCADLLGVSKNRVHTGLSRFFRLHFRQRRVRNPYTI	300
ADX89640.1	NNSTIPLLDENGIGILCLQGRLYITCADLLGVSKNRVHTGLSRFFRLHFRQRRVRNPYTI	300
ACX47139.1	NNSTIPLLDENGIGILCLQGRLYITCADLLGVSKNRVHTGLSRFFRLHFRRRRVRNPYTI	300
ARQ15036.1	NNSTIPLLDENGIGILCLQGRLYITCADLLGVNKNRVHTGLSRFFRLHFRQRRVRNPYTI	300
ARQ14997.1	NNSTIPLLDENGIGILCLQGRLYITCADLLGVNKNRVHTGLSRFFRLHFRQRRVRNPYTI	300
ARQ15002.1	NNSTIPLLDENGIGILCLQGRLYITCADLLGVNKNRVHTGLSRFFRLHFRQRRVRNPYTI	300
ARQ14967.1	NNSTIPLLDENGIGILCLQGRLYITCADLLGVNKNRVHTGLSRFFRLHFRQRRVRNPYTI	300
ARQ15007.1	NNSTIPLLDENGIGILCLQGRLYITCADLLGVNKNRVHTGLSRFFRLHFRQRRVRNPYTI	300
ARQ15011.1	NNSTIPLLDENGIGILCLQGRLYITCADLLGVNKNRVHTGLSRFFRLHFRQRRVRNPYTI	300
ARQ15021.1	NNSTIPLLDENGIGILCLQGRLYITCADLLGVNKNRVHTGLSRFFRLHFRQRRVRNPYTI	300
ARQ15031.1	NNSTIPLLDENGIGILCLQGRLYITCADLLGVNKNRVHTGLSRFFRLHFRQRRVRNPYTI	300
ARQ15026.1	NNSTIPLLDENGIGILCLQGRLYITCADLLGVNKNRVHTGLSRFFRLHFRQRRVRNPYTI	300
ARQ15081.1	NNSTIPLLDENGIGILCLQGRLYITCADLLGVNKNRVHTGLSRFFRLHFRQRRVRNPYTI	300
AIW01035.1	NNSTIPLLDENGIGILCLQGRLYITCADLLGVNKNRVHTGLSRFFRLHFRQRRVRNPYTI	300
ADD51091.1	NNSTIPLLDENGIGILCLQGRLYITCADLLGVNKNRVHTGLSRFFRLHFRQRRVRNPYTI	300
ADD51116.1	NNSTIPLLDENGIGILCLQGRLYITCADLLGVNKNRVHTGLSRFFRLHFRQRRVRNPYTI	300
YP_001285487.1	NNSTIPLLDENGIGILCLQGRLYITCADLLGVNKNRVHTGLSRFFRLHFRQRRVRNPYTI	300
BEJ44995.1	NNSTIPLLDENGIGILCLQGRLYITCADLLGVNKNRVHTGLSRFFRLHFRQRRVRNPYTI	300
QQX23397.1	NNSTIPLLDENGIGILCLQGRLYITCADLLGVNKNRVHTGLSRFFRLHFRQRRVRNPYTI	300
QCQ73655.1	NNSTIPLLDENGIGILCLQGRLYITCADLLGVNKNRVHTGLSRFFRLHFRQRRVRNPYTI	300
QCQ73660.1	NNSTIPLLDENGIGILCLQGRLYITCADLLGVNKNRVHTGLSRFFRLHFRQRRVRNPYTI	300
QCQ73665.1	NNSTIPLLDENGIGILCLQGRLYITCADLLGVNKNRVHTGLSRFFRLHFRQRRVRNPYTI	300
AXU41951.1	NNSTIPLLDENGIGILCLQGRLYITCADLLGVNKNRVHTGLSRFFRLHFRQRRVRNPYTI	300
AXU41956.1	NNSTIPLLDENGIGILCLQGRLYITCADLLGVNKNRVHTGLSRFFRLHFRQRRVRNPYTI	300
AXU41961.1	NNSTIPLLDENGIGILCLQGRLYITCADLLGVNKNRVHTGLSRFFRLHFRQRRVRNPYTI	300
AXU41966.1	NNSTIPLLDENGIGILCLQGRLYITCADLLGVNKNRVHTGLSRFFRLHFRQRRVRNPYTI	300
AXU41971.1	NNSTIPLLDENGIGILCLQGRLYITCADLLGVNKNRVHTGLSRFFRLHFRQRRVRNPYTI	300
AXU41976.1	NNSTIPLLDENGIGILCLQGRLYITCADLLGVNKNRVHTGLSRFFRLHFRQRRVRNPYTI	300
AXU41981.1	NNSTIPLLDENGIGILCLQGRLYITCADLLGVNKNRVHTGLSRFFRLHFRQRRVRNPYTI	300
AXU41986.1	NNSTIPLLDENGIGILCLQGRLYITCADLLGVNKNRVHTGLSRFFRLHFRQRRVRNPYTI	300
ARQ14987.1	NNSTIPLLDENGIGILCLQGRLYITCADLLGVNKNRVHTGLSRFFRLHFRQRRVRNPYTI	300
ARQ15041.1	NNSTIPLLDENGIGILCLQGRLYITCADLLGVNKNRVHTGLSRFFRLHFRQRRVRNPYTI	300
ARQ15051.1	NNSTIPLLDENGIGILCLQGRLYITCADLLGVNKNRVHTGLSRFFRLHFRQRRVRNPYTI	300
ARQ15106.1	NNSTIPLLDENGIGILCLQGRLYITCADLLGVNKNRVHTGLSRFFRLHFRQRRVRNPYTI	300
ARQ15111.1	NNSTIPLLDENGIGILCLQGRLYITCADLLGVNKNRVHTGLSRFFRLHFRQRRVRNPYTI	300
AVK92965.1	NNSTIPLLDENGIGILCLQGRLYITCADLLGVNKNRVHTGLSRFFRLHFRQRRVRNPYTI	300
ARX17290.1	NNSTIPLLDENGIGILCLQGRLYITCADLLGVNKNRVHTGLSRFFRLHFRQRRVRNPYTI	300
ARX17315.1	NNSTIPLLDENGIGILCLQGRLYITCADLLGVNKNRVHTGLSRFFRLHFRQRRVRNPYTI	300
ARX17320.1	NNSTIPLLDENGIGILCLQGRLYITCADLLGVNKNRVHTGLSRFFRLHFRQRRVRNPYTI	300
ARX17330.1	NNSTIPLLDENGIGILCLQGRLYITCADLLGVNKNRVHTGLSRFFRLHFRQRRVRNPYTI	300
ARX17335.1	NNSTIPLLDENGIGILCLQGRLYITCADLLGVNKNRVHTGLSRFFRLHFRQRRVRNPYTI	300
AOW41301.1	NNSTIPLLDENGIGILCLQGRLYITCADLLGVNKNRVHTGLSRFFRLHFRQRRVRNPYTI	300
AMO25748.1	NNSTIPLLDENGIGILCLQGRLYITCADLLGVNKNRVHTGLSRFFRLHFRQRRVRNPYTI	300
AJP06396.1	NNSTIPLLDENGIGILCLQGRLYITCADLLGVNKNRVHTGLSRFFRLHFRQRRVRNPYTI	300
AHB32990.1	NNSTIPLLDENGIGILCLQGRLYITCADLLGVNKNRVHTGLSRFFRLHFRQRRVRNPYTI	300
AHB33000.1	NNSTIPLLDENGIGILCLQGRLYITCADLLGVNKNRVHTGLSRFFRLHFRQRRVRNPYTI	300
ADD50946.1	NNSTIPLLDENGIGILCLQGRLYITCADLLGVNKNRVHTGLSRFFRLHFRQRRVRNPYTI	300
ADD50951.1	NNSTIPLLDENGIGILCLQGRLYITCADLLGVNKNRVHTGLSRFFRLHFRQRRVRNPYTI	300
ADD50956.1	NNSTIPLLDENGIGILCLQGRLYITCADLLGVNKNRVHTGLSRFFRLHFRQRRVRNPYTI	300
ADD50961.1	NNSTIPLLDENGIGILCLQGRLYITCADLLGVNKNRVHTGLSRFFRLHFRQRRVRNPYTI	300
ADD50966.1	NNSTIPLLDENGIGILCLQGRLYITCADLLGVNKNRVHTGLSRFFRLHFRQRRVRNPYTI	300
ADD50971.1	NNSTIPLLDENGIGILCLQGRLYITCADLLGVNKNRVHTGLSRFFRLHFRQRRVRNPYTI	300
ADD50976.1	NNSTIPLLDENGIGILCLQGRLYITCADLLGVNKNRVHTGLSRFFRLHFRQRRVRNPYTI	300
ADD50986.1	NNSTIPLLDENGIGILCLQGRLYITCADLLGVNKNRVHTGLSRFFRLHFRQRRVRNPYTI	300
ADD50991.1	NNSTIPLLDENGIGILCLQGRLYITCADLLGVNKNRVHTGLSRFFRLHFRQRRVRNPYTI	300
ADD51001.1	NNSTIPLLDENGIGILCLQGRLYITCADLLGVNKNRVHTGLSRFFRLHFRQRRVRNPYTI	300
ADD51006.1	NNSTIPLLDENGIGILCLQGRLYITCADLLGVNKNRVHTGLSRFFRLHFRQRRVRNPYTI	300
ADD51011.1	NNSTIPLLDENGIGILCLQGRLYITCADLLGVNKNRVHTGLSRFFRLHFRQRRVRNPYTI	300
ADD51016.1	NNSTIPLLDENGIGILCLQGRLYITCADLLGVNKNRVHTGLSRFFRLHFRQRRVRNPYTI	300
ADD51026.1	NNSTIPLLDENGIGILCLQGRLYITCADLLGVNKNRVHTGLSRFFRLHFRQRRVRNPYTI	300

ADD51031.1	NNSTIPLLDENGIGILCLQGRLYITCADLLGVNKNRVHTGLSRFFRLHFRQRRVRNPYTI	300
ADD51036.1	NNSTIPLLDENGIGILCLQGRLYITCADLLGVNKNRVHTGLSRFFRLHFRQRRVRNPYTI	300
ADD51041.1	NNSTIPLLDENGIGILCLQGRLYITCADLLGVNKNRVHTGLSRFFRLHFRQRRVRNPYTI	300
ADD51046.1	NNSTIPLLDENGIGILCLQGRLYITCADLLGVNKNRVHTGLSRFFRLHFRQRRVRNPYTI	300
ADD51051.1	NNSTIPLLDENGIGILCLQGRLYITCADLLGVNKNRVHTGLSRFFRLHFRQRRVRNPYTI	300
ADD51056.1	NNSTIPLLDENGIGILCLQGRLYITCADLLGVNKNRVHTGLSRFFRLHFRQRRVRNPYTI	300
ADD51061.1	NNSTIPLLDENGIGILCLQGRLYITCADLLGVNKNRVHTGLSRFFRLHFRQRRVRNPYTI	300
ADD51066.1	NNSTIPLLDENGIGILCLQGRLYITCADLLGVNKNRVHTGLSRFFRLHFRQRRVRNPYTI	300
ADD51071.1	NNSTIPLLDENGIGILCLQGRLYITCADLLGVNKNRVHTGLSRFFRLHFRQRRVRNPYTI	300
ADD51076.1	NNSTIPLLDENGIGILCLQGRLYITCADLLGVNKNRVHTGLSRFFRLHFRQRRVRNPYTI	300
ADD51081.1	NNSTIPLLDENGIGILCLQGRLYITCADLLGVNKNRVHTGLSRFFRLHFRQRRVRNPYTI	300
ADD51086.1	NNSTIPLLDENGIGILCLQGRLYITCADLLGVNKNRVHTGLSRFFRLHFRQRRVRNPYTI	300
ADD51096.1	NNSTIPLLDENGIGILCLQGRLYITCADLLGVNKNRVHTGLSRFFRLHFRQRRVRNPYTI	300
ADD51101.1	NNSTIPLLDENGIGILCLQGRLYITCADLLGVNKNRVHTGLSRFFRLHFRQRRVRNPYTI	300
ADD51106.1	NNSTIPLLDENGIGILCLQGRLYITCADLLGVNKNRVHTGLSRFFRLHFRQRRVRNPYTI	300
ADD51121.1	NNSTIPLLDENGIGILCLQGRLYITCADLLGVNKNRVHTGLSRFFRLHFRQRRVRNPYTI	300
ADD51126.1	NNSTIPLLDENGIGILCLQGRLYITCADLLGVNKNRVHTGLSRFFRLHFRQRRVRNPYTI	300
ADD51131.1	NNSTIPLLDENGIGILCLQGRLYITCADLLGVNKNRVHTGLSRFFRLHFRQRRVRNPYTI	300
ADD51136.1	NNSTIPLLDENGIGILCLQGRLYITCADLLGVNKNRVHTGLSRFFRLHFRQRRVRNPYTI	300
ADD51141.1	NNSTIPLLDENGIGILCLQGRLYITCADLLGVNKNRVHTGLSRFFRLHFRQRRVRNPYTI	300
ACW84416.1	NNSTIPLLDENGIGILCLQGRLYITCADLLGVNKNRVHTGLSRFFRLHFRQRRVRNPYTI	300
ACX47149.1	NNSTIPLLDENGIGILCLQGRLYITCADLLGVNKNRVHTGLSRFFRLHFRQRRVRNPYTI	300
ACX47154.1	NNSTIPLLDENGIGILCLQGRLYITCADLLGVNKNRVHTGLSRFFRLHFRQRRVRNPYTI	300
ACR43496.1	NNSTIPLLDENGIGILCLQGRLYITCADLLGVNKNRVHTGLSRFFRLHFRQRRVRNPYTI	300
ACR43497.1	NNSTIPLLDENGIGILCLQGRLYITCADLLGVNKNRVHTGLSRFFRLHFRQRRVRNPYTI	300
ACR43499.1	NNSTIPLLDENGIGILCLQGRLYITCADLLGVNKNRVHTGLSRFFRLHFRQRRVRNPYTI	300
ACF19270.1	NNSTIPLLDENGIGILCLQGRLYITCADLLGVNKNRVHTGLSRFFRLHFRQRRVRNPYTI	300
ACF19285.1	NNSTIPLLDENGIGILCLQGRLYITCADLLGVNKNRVHTGLSRFFRLHFRQRRVRNPYTI	300
ACB12031.1	NNSTIPLLDENGIGILCLQGRLYITCADLLGVNKNRVHTGLSRFFRLHFRQRRVRNPYTI	300
ABY26549.1	NNSTIPLLDENGIGILCLQGRLYITCADLLGVNKNRVHTGLSRFFRLHFRQRRVRNPYTI	300
A5HBD5.1	NNSTIPLLDENGIGILCLQGRLYITCADLLGVNKNRVHTGLSRFFRLHFRQRRVRNPYTI	300
ABQ09289.1	NNSTIPLLDENGIGILCLQGRLYITCADLLGVNKNRVHTGLSRFFRLHFRQRRVRNPYTI	300
ABQ09296.1	NNSTIPLLDENGIGILCLQGRLYITCADLLGVNKNRVHTGLSRFFRLHFRQRRVRNPYTI	300
ABQ09301.1	NNSTIPLLDENGIGILCLQGRLYITCADLLGVNKNRVHTGLSRFFRLHFRQRRVRNPYTI	300
ABQ09306.1	NNSTIPLLDENGIGILCLQGRLYITCADLLGVNKNRVHTGLSRFFRLHFRQRRVRNPYTI	300
ABQ09311.1	NNSTIPLLDENGIGILCLQGRLYITCADLLGVNKNRVHTGLSRFFRLHFRQRRVRNPYTI	300
ABQ09316.1	NNSTIPLLDENGIGILCLQGRLYITCADLLGVNKNRVHTGLSRFFRLHFRQRRVRNPYTI	300
QQX23402.1	NNSTIPLLDENGIGILCLQGRLYITCADLLGVNKNRVHTGLSRFFRLHFRQRRVRNPYTI	300
QJR84015.1	NNSTIPLLDENGIGILCLQGRLYITCADLLGVNKNRVHTGLSRFFRLHFRQRRVRNPYTI	300
QJR84020.1	NNSTIPLLDENGIGILCLQGRLYITCADLLGVNKNRVHTGLSRFFRLHFRQRRVRNPYTI	300
ARQ14962.1	NNSTIPLLDENGIGILCLQGRLYITCADLLGVNKNRVHTGLSRFFRLHFRQRRVRNPYTI	300
ARQ14972.1	NNSTIPLLDENGIGILCLQGRLYITCADLLGVNKNRVHTGLSRFFRLHFRQRRVRNPYTI	300
ARQ14977.1	NNSTIPLLDENGIGILCLQGRLYITCADLLGVNKNRVHTGLSRFFRLHFRQRRVRNPYTI	300
ARQ14982.1	NNSTIPLLDENGIGILCLQGRLYITCADLLGVNKNRVHTGLSRFFRLHFRQRRVRNPYTI	300
ARQ14992.1	NNSTIPLLDENGIGILCLQGRLYITCADLLGVNKNRVHTGLSRFFRLHFRQRRVRNPYTI	300
ARQ15056.1	NNSTIPLLDENGIGILCLQGRLYITCADLLGVNKNRVHTGLSRFFRLHFRQRRVRNPYTI	300
ARQ15061.1	NNSTIPLLDENGIGILCLQGRLYITCADLLGVNKNRVHTGLSRFFRLHFRQRRVRNPYTI	300
ARQ15066.1	NNSTIPLLDENGIGILCLQGRLYITCADLLGVNKNRVHTGLSRFFRLHFRQRRVRNPYTI	300
ARQ15071.1	NNSTIPLLDENGIGILCLQGRLYITCADLLGVNKNRVHTGLSRFFRLHFRQRRVRNPYTI	300
ARQ15076.1	NNSTIPLLDENGIGILCLQGRLYITCADLLGVNKNRVHTGLSRFFRLHFRQRRVRNPYTI	300
ARQ15086.1	NNSTIPLLDENGIGILCLQGRLYITCADLLGVNKNRVHTGLSRFFRLHFRQRRVRNPYTI	300
ARQ15096.1	NNSTIPLLDENGIGILCLQGRLYITCADLLGVNKNRVHTGLSRFFRLHFRQRRVRNPYTI	300
ARQ15116.1	NNSTIPLLDENGIGILCLQGRLYITCADLLGVNKNRVHTGLSRFFRLHFRQRRVRNPYTI	300
ARX17285.1	NNSTIPLLDENGIGILCLQGRLYITCADLLGVNKNRVHTGLSRFFRLHFRQRRVRNPYTI	300
ARX17295.1	NNSTIPLLDENGIGILCLQGRLYITCADLLGVNKNRVHTGLSRFFRLHFRQRRVRNPYTI	300
ARX17300.1	NNSTIPLLDENGIGILCLQGRLYITCADLLGVNKNRVHTGLSRFFRLHFRQRRVRNPYTI	300
ARX17305.1	NNSTIPLLDENGIGILCLQGRLYITCADLLGVNKNRVHTGLSRFFRLHFRQRRVRNPYTI	300
ARX17325.1	NNSTIPLLDENGIGILCLQGRLYITCADLLGVNKNRVHTGLSRFFRLHFRQRRVRNPYTI	300
AHB33010.1	NNSTIPLLDENGIGILCLQGRLYITCADLLGVNKNRVHTGLSRFFRLHFRQRRVRNPYTI	300
AHB33015.1	NNSTIPLLDENGIGILCLQGRLYITCADLLGVNKNRVHTGLSRFFRLHFRQRRVRNPYTI	300
ACX47159.1	NNSTIPLLDENGIGILCLQGRLYITCADLLGVNKNRVHTGLSRFFRLHFRQRRVRNPYTI	300
ACP27656.1	NNSTIPLLDENGIGILCLQGRLYITCADLLGVNKNRVHTGLSRFFRLHFRQRRVRNPYTI	300
ARQ15016.1	NNSTIPLLDENGIGILCLQGRLYITCADLLGVNKNRVHTGLSRFFRLHFRQRRVRNPYTI	300
ARQ15121.1	NNSTIPLLDENGIGILCLQGRLYITCADLLGVNKNRVHTGLSRFFRLHFRQRRVRNPYTI	300
AHB32985.1	NNSTIPLLDENGIGILCLQGRLYITCADLLGVNKNRVHTGLSRFFRLHFRQRRVRNPYTI	300
AHB32995.1	NNSTIPLLDENGIGILCLQGRLYITCADLLGVNKNRVHTGLSRFFRLHFRQRRVRNPYTI	300
AHB33005.1	NNSTIPLLDENGIGILCLQGRLYITCADLLGVNKNRVHTGLSRFFRLHFRQRRVRNPYTI	300

```
ADD50981.1          NNSTIPLLDENGIGILCLQGRLYITCADLLGVNKNRVHTGLSRFFRLHFRQRRVRNPYTI   300
ADD50996.1          NNSTIPLLDENGIGILCLQGRLYITCADLLGVNKNRVHTGLSRFFRLHFRQRRVRNPYTI   300
ADD51021.1          NNSTIPLLDENGIGILCLQGRLYITCADLLGVNKNRVHTGLSRFFRLHFRQRRVRNPYTI   300
ADD51111.1          NNSTIPLLDENGIGILCLQGRLYITCADLLGVNKNRVHTGLSRFFRLHFRQRRVRNPYTI   300
ACR43498.1          NNSTIPLLDENGIGILCLQGRLYITCADLLGVNKNRVHTGLSRFFRLHFRQRRVRNPYTI   300
ACF19280.1          NNSTIPLLDENGIGILCLQGRLYITCADLLGVNKNRVHTGLSRFFRLHFRQRRVRNPYTI   300
ACB12036.1          NNSTIPLLDENGIGILCLQGRLYITCADLLGVNKNRVHTGLSRFFRLHFRQRRVRNPYTI   300
                    ******************* ************.** **************:*********

ADD51146.1          NLLYKQVFNKPADDISGQLQVTEVTMTEETGPLPPTVEGNIGVPTTTNLSHLPATVTLQA   360
ADD51151.1          NLLYKQVFNKPADDISGQLQVTEVTMTEETGPLPPTVEGNIGVPTTTNLSHLPATVTLQA   360
ADD51156.1          NLLYKQVFNKPADDISGQLQVTEVTMTEETGPLPPTVEGNIGVPTTTNLSHLPATVTLQA   360
WIV69164.1          NLLYKQVFNKPADDISGQLQVTEVTMTEETGPLPPTVEGNIGVPTTTNLSHLPATVTLQA   360
ARX17310.1          NLLYKQVFNKPADDISGQLQVTEVTMTEETGPLPPTVEGNIGVPTTTNLSHLPATVTLQA   360
ARQ15101.1          NLLYKQVFNKPADDISGQLQVTEVTMTEETGPLPPTVEGNIGVPTTTNLSHLPATVTLQA   360
ARQ15131.1          NLLYKQVFNKPADDISGQLQVTEVTMTEETGPLPPTVEGNIGVPTTTNLSHLPATVTLQA   360
ARQ15046.1          NLLYKQVFNKPADDISGQLQVTEVTMTEETGPLPPTVEGNIGVPTTTNLSHLPATVTLQA   360
ARQ15091.1          NLLYKQVFNKPADDISGQLQVTEVTMTEETGPLPPTVEGNIGVPTTTNLSHLPATVTLQA   360
ARQ15126.1          NLLYKQVFNKPADDISGQLQVTEVTMTEETGPLPPTVEGNIGVPTTTNLSHLPATVTLQA   360
AXU41991.1          NLLYKQVFNKPADDISGQLQVTEVTMTEETGPLPPTVEGNIGVPTTTNLSHLPATVTLQA   360
AXU41996.1          NLLYKQVFNKPADDISGQLQVTEVTMTEETGPLPPTVEGNIGVPTTTNLSHLPATVTLQA   360
AXU42001.1          NLLYKQVFNKPADDISGQLQVTEVTMTEETGPLPPTVEGNIGVPTTTNLSHLPATVTLQA   360
AMS36878.1          NLLYKQVFNKPADDISGQLQVTEVTMTEETGPLPPTVEGNIGVPTTTNLSHLPATVTLQA   360
ADD50941.1          NLLYKQVFNKPADDISGQLQVTEVTMTEETGPLPPTVEGNIGVPTTTNLSHLPATVTLQA   360
ACF19290.1          NLLYKQVFNKPADDISGQLQVTEVTMTEETGPLPPTVEGNIGVPTTTNLSHLPATVTLQA   360
UJY54021.1          NLLYKQVFNKPADDISGQLQVTEVTMTEETGPLPPTVEGNIGVPTTTNLSHLPATVTLQA   360
ADD50921.1          NLLYKQVFNKPADDISGQLQVTEVTMTEETGPLPPTVEGNIGVPTTTNLSHLPATVTLQA   360
ADD50926.1          NLLYKQVFNKPADDISGQLQVTEVTMTEETGPLPPTVEGNIGVPTTTNLSHLPATVTLQA   360
ADD50931.1          NLLYKQVFNKPADDISGQLQVTEVTMTEETGPLPPTVEGNIGVPTTTNLSHLPATVTLQA   360
ADD50936.1          NLLYKQVFNKPADDISGQLQVTEVTMTEETGPLPPTVEGNIGVPTTTNLSHLPATVTLQA   360
ACF19275.1          NLLYKQVFNKPADDISGQLQVTEVTMTEETGPLPPTVEGNIGVPTTTNLSHLPATVTLQA   360
ACX47164.1          NLLYKQAFNKPADDISGQLQVTEVTMTEETGPLPPTVEGNVGVPTTSNLSHLPATVTLQA   360
ACP27651.1          NLLYKQVFNKPADDISGQLQVTEVTMTEETGPLPPTVEGNVGVPTTTNLSHLPATVSLQA   360
AID07487.1          NLLYKQVFNKPADDISGQLQVTEVTMTEETGPLPPTVEGNVGVPTTSNLSHLPATVTLQA   360
ACX47144.1          NLLYKQVFNKPADDISGQLQVTEVTMTEETGPLPPTVEGNVGVPTTSNLSHLPATVTLQA   360
ARX17280.1          NLLYKQVFNKPADDISGQLQVTEVTMTEETGPLPPTVEGNVGVPTTSNLSHLPATVTLQA   360
ADX89640.1          NLLYKQVFNKPADDISGQLQVTEVTMTEETGPLPPTVEGNVGVPTTSNLSHLPATVTLQA   360
ACX47139.1          NLLYKQVFNKPADDISGQLQVTEVTMTEETGPLPPTVEGNVGVPTTSNLSHLPATVTLQA   360
ARQ15036.1          NLLYKQVFNKPADDISGQLQVTEVTMTEETGPLPPTVEGNIGVPTTTNLSHLPATVTLQA   360
ARQ14997.1          NLLYKQVFNKPADDISGQLQVTEVTMTEETGPLPPTVEGNIGVPTTSNLSHLPATVTLQA   360
ARQ15002.1          NLLYKQVFNKPADDISGQLQVTEVTMTEETGPLPPTVEGNIGVPTTSNLSHLPATVTLQA   360
ARQ14967.1          NLLYKQVFNKPADDISGQLQVTEVTMTEETGPLPPTVEGNVGVPTTTNLSHLPATVTLQA   360
ARQ15007.1          NLLYKQVFNKPADDISGQLQVTEVTMTEETGPLPPTVEGNVGVPTTTNLSHLPATVTLQA   360
ARQ15011.1          NLLYKQVFNKPADDISGQLQVTEVTMTEETGPLPPTVEGNVGVPTTTNLSHLPATVTLQA   360
ARQ15021.1          NLLYKQVFNKPADDISGQLQVTEVTMTEETGPLPPTVEGNVGVPTTTNLSHLPATVTLQA   360
ARQ15031.1          NLLYKQVFNKPADDISGQLQVTEVTMTEETGPLPPTVEGNVGVPTTTNLSHLPATVTLQA   360
ARQ15026.1          NLLYKQVFNKPADDISGQLQVTEVTMTEETGPLPPTVEGNVGVPTTXNLSHLPATVTLQA   360
ARQ15081.1          NLLYKQVFNKPADDISGQLQVTEVTMTEETGPLPPTVEGNVGVPTTSNLSHLPATVTLQA   360
AIW01035.1          NLLYKQVFNKPADDISGQLQVTEVTMTEETGPLPPTVEGNVDVPTTSNLSHLPATVTLQA   360
ADD51091.1          NLLYKQVFNKPADDISGQLQVTEVTMTEETGPLPPTVEGNVGVPTTSNLSHLPATVTLQA   360
ADD51116.1          NLLYKQVFNKPADDISGQLQVTEVTMTEETGPLPPTVEGNIGVPTTSNLSHLPATVTLQA   360
YP_001285487.1      NLLYKQVFNKPADDISGQLQVTEVTMTEETGPLPPTVEGNVGVPTTSNLSHLPATVTLQA   360
BEJ44995.1          NLLYKQVFNKPADDISGQLQVTEVTMTEETGPLPPTVEGNVGVPTTSNLSHLPATVTLQA   360
QQX23397.1          NLLYKQVFNKPADDISGQLQVTEVTMTEETGPLPPTVEGNVGVPTTSNLSHLPATVTLQA   360
QCQ73655.1          NLLYKQVFNKPADDISGQLQVTEVTMTEETGPLPPTVEGNVGVPTTSNLSHLPATVTLQA   360
QCQ73660.1          NLLYKQVFNKPADDISGQLQVTEVTMTEETGPLPPTVEGNVGVPTTSNLSHLPATVTLQA   360
QCQ73665.1          NLLYKQVFNKPADDISGQLQVTEVTMTEETGPLPPTVEGNVGVPTTSNLSHLPATVTLQA   360
AXU41951.1          NLLYKQVFNKPADDISGQLQVTEVTMTEETGPLPPTVEGNVGVPTTSNLSHLPATVTLQA   360
AXU41956.1          NLLYKQVFNKPADDISGQLQVTEVTMTEETGPLPPTVEGNVGVPTTSNLSHLPATVTLQA   360
AXU41961.1          NLLYKQVFNKPADDISGQLQVTEVTMTEETGPLPPTVEGNVGVPTTSNLSHLPATVTLQA   360
AXU41966.1          NLLYKQVFNKPADDISGQLQVTEVTMTEETGPLPPTVEGNVGVPTTSNLSHLPATVTLQA   360
AXU41971.1          NLLYKQVFNKPADDISGQLQVTEVTMTEETGPLPPTVEGNVGVPTTSNLSHLPATVTLQA   360
AXU41976.1          NLLYKQVFNKPADDISGQLQVTEVTMTEETGPLPPTVEGNVGVPTTSNLSHLPATVTLQA   360
AXU41981.1          NLLYKQVFNKPADDISGQLQVTEVTMTEETGPLPPTVEGNVGVPTTSNLSHLPATVTLQA   360
AXU41986.1          NLLYKQVFNKPADDISGQLQVTEVTMTEETGPLPPTVEGNVGVPTTSNLSHLPATVTLQA   360
ARQ14987.1          NLLYKQVFNKPADDISGQLQVTEVTMTEETGPLPPTVEGNVGVPTTSNLSHLPATVTLQA   360
ARQ15041.1          NLLYKQVFNKPADDISGQLQVTEVTMTEETGPLPPTVEGNVGVPTTSNLSHLPATVTLQA   360
```

ARQ15051.1	NLLYKQVFNKPADDISGQLQVTEVTMTEETGPLPPTVEGNVGVPTTSNLSHLPATVTLQA	360
ARQ15106.1	NLLYKQVFNKPADDISGQLQVTEVTMTEETGPLPPTVEGNVGVPTTSNLSHLPATVTLQA	360
ARQ15111.1	NLLYKQVFNKPADDISGQLQVTEVTMTEETGPLPPTVEGNVGVPTTSNLSHLPATVTLQA	360
AVK92965.1	NLLYKQVFNKPADDISGQLQVTEVTMTEETGPLPPTVEGNVGVPTTSNLSHLPATVTLQA	360
ARX17290.1	NLLYKQVFNKPADDISGQLQVTEVTMTEETGPLPPTVEGNVGVPTTSNLSHLPATVTLQA	360
ARX17315.1	NLLYKQVFNKPADDISGQLQVTEVTMTEETGPLPPTVEGNVGVPTTSNLSHLPATVTLQA	360
ARX17320.1	NLLYKQVFNKPADDISGQLQVTEVTMTEETGPLPPTVEGNVGVPTTSNLSHLPATVTLQA	360
ARX17330.1	NLLYKQVFNKPADDISGQLQVTEVTMTEETGPLPPTVEGNVGVPTTSNLSHLPATVTLQA	360
ARX17335.1	NLLYKQVFNKPADDISGQLQVTEVTMTEETGPLPPTVEGNVGVPTTSNLSHLPATVTLQA	360
AOW41301.1	NLLYKQVFNKPADDISGQLQVTEVTMTEETGPLPPTVEGNVGVPTTSNLSHLPATVTLQA	360
AMO25748.1	NLLYKQVFNKPADDISGQLQVTEVTMTEETGPLPPTVEGNVGVPTTSNLSHLPATVTLQA	360
AJP06396.1	NLLYKQVFNKPADDISGQLQVTEVTMTEETGPLPPTVEGNVGVPTTSNLSHLPATVTLQA	360
AHB32990.1	NLLYKQVFNKPADDISGQLQVTEVTMTEETGPLPPTVEGNVGVPTTSNLSHLPATVTLQA	360
AHB33000.1	NLLYKQVFNKPADDISGQLQVTEVTMTEETGPLPPTVEGNVGVPTTSNLSHLPATVTLQA	360
ADD50946.1	NLLYKQVFNKPADDISGQLQVTEVTMTEETGPLPPTVEGNVGVPTTSNLSHLPATVTLQA	360
ADD50951.1	NLLYKQVFNKPADDISGQLQVTEVTMTEETGPLPPTVEGNVGVPTTSNLSHLPATVTLQA	360
ADD50956.1	NLLYKQVFNKPADDISGQLQVTEVTMTEETGPLPPTVEGNVGVPTTSNLSHLPATVTLQA	360
ADD50961.1	NLLYKQVFNKPADDISGQLQVTEVTMTEETGPLPPTVEGNVGVPTTSNLSHLPATVTLQA	360
ADD50966.1	NLLYKQVFNKPADDISGQLQVTEVTMTEETGPLPPTVEGNVGVPTTSNLSHLPATVTLQA	360
ADD50971.1	NLLYKQVFNKPADDISGQLQVTEVTMTEETGPLPPTVEGNVGVPTTSNLSHLPATVTLQA	360
ADD50976.1	NLLYKQVFNKPADDISGQLQVTEVTMTEETGPLPPTVEGNVGVPTTSNLSHLPATVTLQA	360
ADD50986.1	NLLYKQVFNKPADDISGQLQVTEVTMTEETGPLPPTVEGNVGVPTTSNLSHLPATVTLQA	360
ADD50991.1	NLLYKQVFNKPADDISGQLQVTEVTMTEETGPLPPTVEGNVGVPTTSNLSHLPATVTLQA	360
ADD51001.1	NLLYKQVFNKPADDISGQLQVTEVTMTEETGPLPPTVEGNVGVPTTSNLSHLPATVTLQA	360
ADD51006.1	NLLYKQVFNKPADDISGQLQVTEVTMTEETGPLPPTVEGNVGVPTTSNLSHLPATVTLQA	360
ADD51011.1	NLLYKQVFNKPADDISGQLQVTEVTMTEETGPLPPTVEGNVGVPTTSNLSHLPATVTLQA	360
ADD51016.1	NLLYKQVFNKPADDISGQLQVTEVTMTEETGPLPPTVEGNVGVPTTSNLSHLPATVTLQA	360
ADD51026.1	NLLYKQVFNKPADDISGQLQVTEVTMTEETGPLPPTVEGNVGVPTTSNLSHLPATVTLQA	360
ADD51031.1	NLLYKQVFNKPADDISGQLQVTEVTMTEETGPLPPTVEGNVGVPTTSNLSHLPATVTLQA	360
ADD51036.1	NLLYKQVFNKPADDISGQLQVTEVTMTEETGPLPPTVEGNVGVPTTSNLSHLPATVTLQA	360
ADD51041.1	NLLYKQVFNKPADDISGQLQVTEVTMTEETGPLPPTVEGNVGVPTTSNLSHLPATVTLQA	360
ADD51046.1	NLLYKQVFNKPADDISGQLQVTEVTMTEETGPLPPTVEGNVGVPTTSNLSHLPATVTLQA	360
ADD51051.1	NLLYKQVFNKPADDISGQLQVTEVTMTEETGPLPPTVEGNVGVPTTSNLSHLPATVTLQA	360
ADD51056.1	NLLYKQVFNKPADDISGQLQVTEVTMTEETGPLPPTVEGNVGVPTTSNLSHLPATVTLQA	360
ADD51061.1	NLLYKQVFNKPADDISGQLQVTEVTMTEETGPLPPTVEGNVGVPTTSNLSHLPATVTLQA	360
ADD51066.1	NLLYKQVFNKPADDISGQLQVTEVTMTEETGPLPPTVEGNVGVPTTSNLSHLPATVTLQA	360
ADD51071.1	NLLYKQVFNKPADDISGQLQVTEVTMTEETGPLPPTVEGNVGVPTTSNLSHLPATVTLQA	360
ADD51076.1	NLLYKQVFNKPADDISGQLQVTEVTMTEETGPLPPTVEGNVGVPTTSNLSHLPATVTLQA	360
ADD51081.1	NLLYKQVFNKPADDISGQLQVTEVTMTEETGPLPPTVEGNVGVPTTSNLSHLPATVTLQA	360
ADD51086.1	NLLYKQVFNKPADDISGQLQVTEVTMTEETGPLPPTVEGNVGVPTTSNLSHLPATVTLQA	360
ADD51096.1	NLLYKQVFNKPADDISGQLQVTEVTMTEETGPLPPTVEGNVGVPTTSNLSHLPATVTLQA	360
ADD51101.1	NLLYKQVFNKPADDISGQLQVTEVTMTEETGPLPPTVEGNVGVPTTSNLSHLPATVTLQA	360
ADD51106.1	NLLYKQVFNKPADDISGQLQVTEVTMTEETGPLPPTVEGNVGVPTTSNLSHLPATVTLQA	360
ADD51121.1	NLLYKQVFNKPADDISGQLQVTEVTMTEETGPLPPTVEGNVGVPTTSNLSHLPATVTLQA	360
ADD51126.1	NLLYKQVFNKPADDISGQLQVTEVTMTEETGPLPPTVEGNVGVPTTSNLSHLPATVTLQA	360
ADD51131.1	NLLYKQVFNKPADDISGQLQVTEVTMTEETGPLPPTVEGNVGVPTTSNLSHLPATVTLQA	360
ADD51136.1	NLLYKQVFNKPADDISGQLQVTEVTMTEETGPLPPTVEGNVGVPTTSNLSHLPATVTLQA	360
ADD51141.1	NLLYKQVFNKPADDISGQLQVTEVTMTEETGPLPPTVEGNVGVPTTSNLSHLPATVTLQA	360
ACW84416.1	NLLYKQVFNKPADDISGQLQVTEVTMTEETGPLPPTVEGNVGVPTTSNLSHLPATVTLQA	360
ACX47149.1	NLLYKQVFNKPADDISGQLQVTEVTMTEETGPLPPTVEGNVGVPTTSNLSHLPATVTLQA	360
ACX47154.1	NLLYKQVFNKPADDISGQLQVTEVTMTEETGPLPPTVEGNVGVPTTSNLSHLPATVTLQA	360
ACR43496.1	NLLYKQVFNKPADDISGQLQVTEVTMTEETGPLPPTVEGNVGVPTTSNLSHLPATVTLQA	360
ACR43497.1	NLLYKQVFNKPADDISGQLQVTEVTMTEETGPLPPTVEGNVGVPTTSNLSHLPATVTLQA	360
ACR43499.1	NLLYKQVFNKPADDISGQLQVTEVTMTEETGPLPPTVEGNVGVPTTSNLSHLPATVTLQA	360
ACF19270.1	NLLYKQVFNKPADDISGQLQVTEVTMTEETGPLPPTVEGNVGVPTTSNLSHLPATVTLQA	360
ACF19285.1	NLLYKQVFNKPADDISGQLQVTEVTMTEETGPLPPTVEGNVGVPTTSNLSHLPATVTLQA	360
ACB12031.1	NLLYKQVFNKPADDISGQLQVTEVTMTEETGPLPPTVEGNVGVPTTSNLSHLPATVTLQA	360
ABY26549.1	NLLYKQVFNKPADDISGQLQVTEVTMTEETGPLPPTVEGNVGVPTTSNLSHLPATVTLQA	360
A5HBD5.1	NLLYKQVFNKPADDISGQLQVTEVTMTEETGPLPPTVEGNVGVPTTSNLSHLPATVTLQA	360
ABQ09289.1	NLLYKQVFNKPADDISGQLQVTEVTMTEETGPLPPTVEGNVGVPTTSNLSHLPATVTLQA	360
ABQ09296.1	NLLYKQVFNKPADDISGQLQVTEVTMTEETGPLPPTVEGNVGVPTTSNLSHLPATVTLQA	360
ABQ09301.1	NLLYKQVFNKPADDISGQLQVTEVTMTEETGPLPPTVEGNVGVPTTSNLSHLPATVTLQA	360
ABQ09306.1	NLLYKQVFNKPADDISGQLQVTEVTMTEETGPLPPTVEGNVGVPTTSNLSHLPATVTLQA	360
ABQ09311.1	NLLYKQVFNKPADDISGQLQVTEVTMTEETGPLPPTVEGNVGVPTTSNLSHLPATVTLQA	360
ABQ09316.1	NLLYKQVFNKPADDISGQLQVTEVTMTEETGPLPPTVEGNVGVPTTSNLSHLPATVTLQA	360
QQX23402.1	NLLYKQVFNKPADDISGQLQVTEVTMTEETGPLPPTVEGNVGVPTTSNLSHLPATVTLQA	360
QJR84015.1	NLLYKQVFNKPADDISGQLQVTEVTMTEETGPLPPTVEGNVGVPTTSNLSHLPATVTLQA	360

```
QJR84020.1          NLLYKQVFNKPADDISGQLQVTEVTMTEETGPLPPTVEGNVGVPTTSNLSHLPATVTLQA   360
ARQ14962.1          NLLYKQVFNKPADDISGQLQVTEVTMTEETGPLPPTVEGNVGVPTTSNLSHLPATVTLQA   360
ARQ14972.1          NLLYKQVFNKPADDISGQLQVTEVTMTEETGPLPPTVEGNVGVPTTSNLSHLPATVTLQA   360
ARQ14977.1          NLLYKQVFNKPADDISGQLQVTEVTMTEETGPLPPTVEGNVGVPTTSNLSHLPATVTLQA   360
ARQ14982.1          NLLYKQVFNKPADDISGQLQVTEVTMTEETGPLPPTVEGNVGVPTTSNLSHLPATVTLQA   360
ARQ14992.1          NLLYKQVFNKPADDISGQLQVTEVTMTEETGPLPPTVEGNVGVPTTSNLSHLPATVTLQA   360
ARQ15056.1          NLLYKQVFNKPADDISGQLQVTEVTMTEETGPLPPTVEGNVGVPTTSNLSHLPATVTLQA   360
ARQ15061.1          NLLYKQVFNKPADDISGQLQVTEVTMTEETGPLPPTVEGNVGVPTTSNLSHLPATVTLQA   360
ARQ15066.1          NLLYKQVFNKPADDISGQLQVTEVTMTEETGPLPPTVEGNVGVPTTSNLSHLPATVTLQA   360
ARQ15071.1          NLLYKQVFNKPADDISGQLQVTEVTMTEETGPLPPTVEGNVGVPTTSNLSHLPATVTLQA   360
ARQ15076.1          NLLYKQVFNKPADDISGQLQVTEVTMTEETGPLPPTVEGNVGVPTTSNLSHLPATVTLQA   360
ARQ15086.1          NLLYKQVFNKPADDISGQLQVTEVTMTEETGPLPPTVEGNVGVPTTSNLSHLPATVTLQA   360
ARQ15096.1          NLLYKQVFNKPADDISGQLQVTEVTMTEETGPLPPTVEGNVGVPTTSNLSHLPATVTLQA   360
ARQ15116.1          NLLYKQVFNKPADDISGQLQVTEVTMTEETGPLPPTVEGNVGVPTTSNLSHLPATVTLQA   360
ARX17285.1          NLLYKQVFNKPADDISGQLQVTEVTMTEETGPLPPTVEGNVGVPTTSNLSHLPATVTLQA   360
ARX17295.1          NLLYKQVFNKPADDISGQLQVTEVTMTEETGPLPPTVEGNVGVPTTSNLSHLPATVTLQA   360
ARX17300.1          NLLYKQVFNKPADDISGQLQVTEVTMTEETGPLPPTVEGNVGVPTTSNLSHLPATVTLQA   360
ARX17305.1          NLLYKQVFNKPADDISGQLQVTEVTMTEETGPLPPTVEGNVGVPTTSNLSHLPATVTLQA   360
ARX17325.1          NLLYKQVFNKPADDISGQLQVTEVTMTEETGPLPPTVEGNVGVPTTSNLSHLPATVTLQA   360
AHB33010.1          NLLYKQVFNKPADDISGQLQVTEVTMTEETGPLPPTVEGNVGVPTTSNLSHLPATVTLQA   360
AHB33015.1          NLLYKQVFNKPADDISGQLQVTEVTMTEETGPLPPTVEGNVGVPTTSNLSHLPATVTLQA   360
ACX47159.1          NLLYKQVFNKPADDISGQLQVTEVTMTEETGPLPPTVEGNVGVPTTSNLSHLPATVTLQA   360
ACP27656.1          NLLYKQVFNKPADDISGQLQVTEVTMTEETGPLPPTVEGNVGVPTTSNLSHLPATVTLQA   360
ARQ15016.1          NLLYKQVFNKPADDISGQLQVTEVTMTEETGPLPPTVEGNVGVPTTTNLSHLPATVTLQA   360
ARQ15121.1          NLLYKQVFNKPADDISGQLQVTEVTMTEETGPLPPTVEGNVGVPTTSNLSHLPATVTLQA   360
AHB32985.1          NLLYKQVFNKPADDISGQLQVTEVTMTEETGPLPPTVEGNVGVPTTSNLSHLPATVTLQA   360
AHB32995.1          NLLYKQVFNKPADDISGQLQVTEVTMTEETGPLPPTVEGNVGVPTTSNLSHLPATVTLQA   360
AHB33005.1          NLLYKQVFNKPADDISGQLQVTEVTMTEETGPLPPTVEGNVGVPTTSNLSHLPATVTLQA   360
ADD50981.1          NLLYKQVFNKPADDISGQLQVTEVTMTEETGPLPPTVEGNVGVPTTSNLSHLPATVTLQA   360
ADD50996.1          NLLYKQVFNKPADDISGQLQVTEVTMTEETGPLPPTVEGNVGVPTTSNLSHLPATVTLQA   360
ADD51021.1          NLLYKQVFNKPADDISGQLQVTEVTMTEETGPLPPTVEGNVGVPTTSNLSHLPATVTLQA   360
ADD51111.1          NLLYKQVFNKPADDISGQLQVTEVTMTEETGPLPPTVEGNVGVPTTSNLSHLPATVTLQA   360
ACR43498.1          NLLYKQVFNKPADDISGQLQVTEVTMTEETGPLPPTVEGNVGVPTTSNLSHLPATVTLQA   360
ACF19280.1          NLLYKQVFNKPADDISGQLQVTEVTMTEETGPLPPTVEGNVGVPTTSNLSYLPATVTLQA   360
ACB12036.1          NLLYKQVFNKPADDISGQLQVTEVTMTEETGPLPPTVEGNVGVPTTSNLSHLPATVTLQA   360
                    ******.*********************************:.**** ***:*****:***

ADD51146.1          TGPILNTQG 369
ADD51151.1          TGPILNTQG 369
ADD51156.1          TGPILNTQG 369
WIV69164.1          TGPILNTQG 369
ARX17310.1          TGPILNTQG 369
ARQ15101.1          TGPILNTQG 369
ARQ15131.1          TGPILNTQG 369
ARQ15046.1          TGPILNTQG 369
ARQ15091.1          TGPILNTQG 369
ARQ15126.1          TGPILNTQG 369
AXU41991.1          TGPILNTQG 369
AXU41996.1          TGPILNTQG 369
AXU42001.1          TGPILNTQG 369
AMS36878.1          TGPILNTQG 369
ADD50941.1          TGPILNTQG 369
ACF19290.1          TGPILNTQG 369
UJY54021.1          TGPILNTQG 369
ADD50921.1          TGPILNTQG 369
ADD50926.1          TGPILNTQG 369
ADD50931.1          TGPILNTQG 369
ADD50936.1          TGPILNTQG 369
ACF19275.1          TGPILNTQG 369
ACX47164.1          TGPILNTQG 369
ACP27651.1          TGPILNTQG 369
AID07487.1          TGPILNTQG 369
ACX47144.1          TGPILNTQG 369
ARX17280.1          TGPILNTQG 369
ADX89640.1          TGPILNTQG 369
ACX47139.1          TGPILNTQG 369
ARQ15036.1          TGPILNTQG 369
```

ARQ14997.1	TGPILNTQG	369
ARQ15002.1	TGPILNTQG	369
ARQ14967.1	TGPILNTQG	369
ARQ15007.1	TGPILNTQG	369
ARQ15011.1	TGPILNTQG	369
ARQ15021.1	TGPILNTQG	369
ARQ15031.1	TGPILNTQG	369
ARQ15026.1	TGPILNTQG	369
ARQ15081.1	TGPILNTQG	369
AIW01035.1	TGPILNTQG	369
ADD51091.1	TGPILNTQG	369
ADD51116.1	TGPILNTQG	369
YP_001285487.1	TGPILNTQG	369
BEJ44995.1	TGPILNTQG	369
QQX23397.1	TGPILNTQG	369
QCQ73655.1	TGPILNTQG	369
QCQ73660.1	TGPILNTQG	369
QCQ73665.1	TGPILNTQG	369
AXU41951.1	TGPILNTQG	369
AXU41956.1	TGPILNTQG	369
AXU41961.1	TGPILNTQG	369
AXU41966.1	TGPILNTQG	369
AXU41971.1	TGPILNTQG	369
AXU41976.1	TGPILNTQG	369
AXU41981.1	TGPILNTQG	369
AXU41986.1	TGPILNTQG	369
ARQ14987.1	TGPILNTQG	369
ARQ15041.1	TGPILNTQG	369
ARQ15051.1	TGPILNTQG	369
ARQ15106.1	TGPILNTQG	369
ARQ15111.1	TGPILNTQG	369
AVK92965.1	TGPILNTQG	369
ARX17290.1	TGPILNTQG	369
ARX17315.1	TGPILNTQG	369
ARX17320.1	TGPILNTQG	369
ARX17330.1	TGPILNTQG	369
ARX17335.1	TGPILNTQG	369
AOW41301.1	TGPILNTQG	369
AMO25748.1	TGPILNTQG	369
AJP06396.1	TGPILNTQG	369
AHB32990.1	TGPILNTQG	369
AHB33000.1	TGPILNTQG	369
ADD50946.1	TGPILNTQG	369
ADD50951.1	TGPILNTQG	369
ADD50956.1	TGPILNTQG	369
ADD50961.1	TGPILNTQG	369
ADD50966.1	TGPILNTQG	369
ADD50971.1	TGPILNTQG	369
ADD50976.1	TGPILNTQG	369
ADD50986.1	TGPILNTQG	369
ADD50991.1	TGPILNTQG	369
ADD51001.1	TGPILNTQG	369
ADD51006.1	TGPILNTQG	369
ADD51011.1	TGPILNTQG	369
ADD51016.1	TGPILNTQG	369
ADD51026.1	TGPILNTQG	369
ADD51031.1	TGPILNTQG	369
ADD51036.1	TGPILNTQG	369
ADD51041.1	TGPILNTQG	369
ADD51046.1	TGPILNTQG	369
ADD51051.1	TGPILNTQG	369
ADD51056.1	TGPILNTQG	369
ADD51061.1	TGPILNTQG	369
ADD51066.1	TGPILNTQG	369
ADD51071.1	TGPILNTQG	369
ADD51076.1	TGPILNTQG	369
ADD51081.1	TGPILNTQG	369

ADD51086.1	TGPILNTQG	369
ADD51096.1	TGPILNTQG	369
ADD51101.1	TGPILNTQG	369
ADD51106.1	TGPILNTQG	369
ADD51121.1	TGPILNTQG	369
ADD51126.1	TGPILNTQG	369
ADD51131.1	TGPILNTQG	369
ADD51136.1	TGPILNTQG	369
ADD51141.1	TGPILNTQG	369
ACW84416.1	TGPILNTQG	369
ACX47149.1	TGPILNTQG	369
ACX47154.1	TGPILNTQG	369
ACR43496.1	TGPILNTQG	369
ACR43497.1	TGPILNTQG	369
ACR43499.1	TGPILNTQG	369
ACF19270.1	TGPILNTQG	369
ACF19285.1	TGPILNTQG	369
ACB12031.1	TGPILNTQG	369
ABY26549.1	TGPILNTQG	369
A5HBD5.1	TGPILNTQG	369
ABQ09289.1	TGPILNTQG	369
ABQ09296.1	TGPILNTQG	369
ABQ09301.1	TGPILNTQG	369
ABQ09306.1	TGPILNTQG	369
ABQ09311.1	TGPILNTQG	369
ABQ09316.1	TGPILNTQG	369
QQX23402.1	TGPILNTQG	369
QJR84015.1	TGPILNTQG	369
QJR84020.1	TGPILNTQG	369
ARQ14962.1	TGPILNTQG	369
ARQ14972.1	TGPILNTQG	369
ARQ14977.1	TGPILNTQG	369
ARQ14982.1	TGPILNTQG	369
ARQ14992.1	TGPILNTQG	369
ARQ15056.1	TGPILNTQG	369
ARQ15061.1	TGPILNTQG	369
ARQ15066.1	TGPILNTQG	369
ARQ15071.1	TGPILNTQG	369
ARQ15076.1	TGPILNTQG	369
ARQ15086.1	TGPILNTQG	369
ARQ15096.1	TGPILNTQG	369
ARQ15116.1	TGPILNTQG	369
ARX17285.1	TGPILNTQG	369
ARX17295.1	TGPILNTQG	369
ARX17300.1	TGPILNTQG	369
ARX17305.1	TGPILNTQG	369
ARX17325.1	TGPILNTQG	369
AHB33010.1	TGPILNTQG	369
AHB33015.1	TGPILNTQG	369
ACX47159.1	TGPILNTQG	369
ACP27656.1	TGPILNTQG	369
ARQ15016.1	TGPILNTQG	369
ARQ15121.1	TGPILNTQG	369
AHB32985.1	TGPILNTQG	369
AHB32995.1	TGPILNTQG	369
AHB33005.1	TGPILNTQG	369
ADD50981.1	TGPILNTQG	369
ADD50996.1	TGPILNTQG	369
ADD51021.1	TGPILNTQG	369
ADD51111.1	TGPILNTQG	369
ACR43498.1	TGPILNTQG	369
ACF19280.1	TGPILNTQG	369
ACB12036.1	TGPILNTQG	369
	*********	
