# Supplementary material for: The Conserved YPX3L Motif in the BK Polyomavirus VP1 Protein Is Important for Viral Particle Assembly but Not for Its Secretion into Extracellular Vesicles
Source: Viruses. 2024 Jul 13;16(7):1124. doi: 10.3390/v16071124 (PMC11281352; doi:10.3390/v16071124)
Supplement: Supplementary file 1 [file viruses-16-01124-s001.zip › HPyV 05 alignment.pdf]

CLUSTAL O(1.2.4) multiple sequence alignment

```
ABY65888.1          MAPKRKASSTCKTPKRQCIPKPGCCPNVASVPKLLVKGGVEVLSVVTGEDSITQIELYLN   60
QXF78584.1          MAPKRKASSTCKTPKRQCIPKPGCCPNVASVPKLLVKGGVEVLSVVTGEDSITQIELYLN   60
QXF78586.1          MAPKRKASSTCKTPKRQCIPKPGCCPNVASVPKLLVKGGVEVLSVVTGEDSITQIELYLN   60
ANF28692.1          MAPKRKASSTCKTPKRQCIPKPGCCPNVASVPKLLVKGGVEVLSVVTGEDSITQIELYLN   60
AHW79948.1          MAPKRKASSTCKTTKRQCIPKPGCCPNVASVPKLLVKGGVEVLSVVTGEDSITQIELYLN   60
AWG42111.1          MAPKRKASSTCKTPKRQCIPKPGCCPNVASVPKLLVKGGVEVLSVVTGEDSITQIELYLN   60
AWG42117.1          MAPKRKASSTCKTPKRQCIPKPGCCPNVASVPKLLVKGGVEVLSVVTGEDSITQIELYLN   60
ANF28693.1          MAPKRKASSTCKTPKRQCIPKPGCCPNVASVPKLLVKGGVEVLSVVTGEDSITQIELYLN   60
ANF28694.1          MAPKRKASSTCKTPKRQCIPKPGCCPNVASVPKLLVKGGVEVLSVVTGEDSITQIELYLN   60
ANF28695.1          MAPKRKASSTCKTPKRQCIPKPGCCPNVASVPKLLVKGGVEVLSVVTGEDSITQIELYLN   60
ANF28698.1          MAPKRKASSTCKTPKRQCIPKPGCCPNVASVPKLLVKGGVEVLSVVTGEDSITQIELYLN   60
CAR95366.1          MAPKRKASSTCKTPKRQCIPKPGCCPNVASVPKLLVKGGVEVLSVVTGEDSITQIELYLN   60
AFM84435.1          MAPKRKASSTCKTPKRQCIPKPGCCPNIASVPKLLVKGGVEVLSVVTGEDSITQIELYLN   60
AFM84450.1          MAPKRKASSTCKTPKRQCIPKPGCCPNIASVPKLLVKGGVEVLSVVTGEDSITQIELYLN   60
AFM84455.1          MAPKRKASSTCKTPKRQCIPKPGCCPNIASVPKLLVKGGVEVLSVVTGEDSITQIELYLN   60
ANF28697.1          MAPKRKASSTCKTPKRQCIPKPGCCPNVASVPKLLVKGGVEVLSVVTGEDSITQIELYLN   60
ACN44192.1          -APKRKASSTCKTPKRQCIPKPGCCPNVASVPKLLVKGGVEVLSVVTGEDSITQIELYLN   59
ADE45355.1          MAPKRKASSTCKTPKRQCIPKPGCCPNVASVPKLLVKGGVEVLSVVTGEDSITQIELYLN   60
ADE45359.1          MAPKRKASSTCKTPKRQCIPKPGCCPNVASVPKLLVKGGVEVLSVVTGEDSITQIELYLN   60
ADE45351.1          MAPKRKASSTCKTPKRQCIPKPGCCPNVASVPKLLVKGGVEVLSVVTGEDSITQIELYLN   60
ADE45347.1          MAPKRKASSTCKTPKRQCIPKPGCCPNVASVPKLLVKGGVEVLSVVTGEDSITQIELYLN   60
AEM01087.1          MAPKRKASSTCKTPKRQCIPKPGCCPNVASVPKLLVKGGVEVLSVVTGEDSITQIELYLN   60
AFC36083.1          MAPKRKASSTCKTPKRQCIPKPGCCPNVASVPKLLVKGGVEVLSVVTGEDSITQIELYLN   60
AFM84460.1          MAPKRKASSTCKTPKRQCLPKPGCCPNVASVPKLLVKGGVEVLSVVTGEDSITQIELYLN   60
AHW79944.1          MAPKRKASSTCKTPKRQCVPKPGCCPNVASVPKLLVKGGVEVLSVVTGEDSITQIELYLN   60
BBJ26583.1          MAPKRKASSTCKTPKRQCISKPGCCPNVASVPKLLVKGGVEVLSVVTGEDSITQIELYLN   60
BBJ26586.1          MAPKRKASSTCKTPKRQCISKPGCCPNVASVPKLLVKGGVEVLSVVTGEDSITQIELYLN   60
APA21079.1          MAPKRKASSTCKTPKRQCISKPGCCPNVASVPKLLVKGGVEVLSVVTGEDSITQIELYLN   60
ADN95996.1          MAPKRKASSTCKTPKRQCISKPGCCPNVASVPKLLVKGGVEVLSVVTGEDSITQIELYLN   60
YP_009111420.1      MAPKRKASSTCKTPKRQCIPKPGCCPNVASVPKLLVKGGVEVLSVVTGEDSITQIELYLN   60
UQK62677.1          MAPKRKASSTCKTPKRQCIPKPGCCPNVASVPKLLVKGGVEVLSVVTGEDSITQIELYLN   60
AWG42121.1          MAPKRKASSTCKTPKRQCIPKPGCCPNVASVPKLLVKGGVEVLSVVTGEDSITQIELYLN   60
AWG42124.1          MAPKRKASSTCKTPKRQCIPKPGCCPNVASVPKLLVKGGVEVLSVVTGEDSITQIELYLN   60
AWG42134.1          MAPKRKASSTCKTPKRQCIPKPGCCPNVASVPKLLVKGGVEVLSVVTGEDSITQIELYLN   60
AWG42140.1          MAPKRKASSTCKTPKRQCIPKPGCCPNVASVPKLLVKGGVEVLSVVTGEDSITQIELYLN   60
AQM73644.1          MAPKRKASSTCKTPKRQCIPKPGCCPNVASVPKLLVKGGVEVLSVVTGEDSITQIELYLN   60
BAV01229.1          MAPKRKASSTCKTPKRQCIPKPGCCPNVASVPKLLVKGGVEVLSVVTGEDSITQIELYLN   60
AKK25202.1          MAPKRKASSTCKTPKRQCIPKPGCCPNVASVPKLLVKGGVEVLSVVTGEDSITQIELYLN   60
AHN49701.1          MAPKRKASSTCKTPKRQCIPKPGCCPNVASVPKLLVKGGVEVLSVVTGEDSITQIELYLN   60
AHN49705.1          MAPKRKASSTCKTPKRQCIPKPGCCPNVASVPKLLVKGGVEVLSVVTGEDSITQIELYLN   60
AHN49709.1          MAPKRKASSTCKTPKRQCIPKPGCCPNVASVPKLLVKGGVEVLSVVTGEDSITQIELYLN   60
AHW79940.1          MAPKRKASSTCKTPKRQCIPKPGCCPNVASVPKLLVKGGVEVLSVVTGEDSITQIELYLN   60
AHN13648.1          MAPKRKASSTCKTPKRQCIPKPGCCPNVASVPKLLVKGGVEVLSVVTGEDSITQIELYLN   60
BAN78699.1          MAPKRKASSTCKTPKRQCIPKPGCCPNVASVPKLLVKGGVEVLSVVTGEDSITQIELYLN   60
AGA03918.1          MAPKRKASSTCKTPKRQCIPKPGCCPNVASVPKLLVKGGVEVLSVVTGEDSITQIELYLN   60
AFM84440.1          MAPKRKASSTCKTPKRQCIPKPGCCPNVASVPKLLVKGGVEVLSVVTGEDSITQIELYLN   60
AFM84445.1          MAPKRKASSTCKTPKRQCIPKPGCCPNVASVPKLLVKGGVEVLSVVTGEDSITQIELYLN   60
AFC36077.1          MAPKRKASSTCKTPKRQCIPKPGCCPNVASVPKLLVKGGVEVLSVVTGEDSITQIELYLN   60
AFC36088.1          MAPKRKASSTCKTPKRQCIPKPGCCPNVASVPKLLVKGGVEVLSVVTGEDSITQIELYLN   60
AFC36093.1          MAPKRKASSTCKTPKRQCIPKPGCCPNVASVPKLLVKGGVEVLSVVTGEDSITQIELYLN   60
AEM01095.1          MAPKRKASSTCKTPKRQCIPKPGCCPNVASVPKLLVKGGVEVLSVVTGEDSITQIELYLN   60
AEM01098.1          MAPKRKASSTCKTPKRQCIPKPGCCPNVASVPKLLVKGGVEVLSVVTGEDSITQIELYLN   60
ADE45363.1          MAPKRKASSTCKTPKRQCIPKPGCCPNVASVPKLLVKGGVEVLSVVTGEDSITQIELYLN   60
ADE45375.1          MAPKRKASSTCKTPKRQCIPKPGCCPNVASVPKLLVKGGVEVLSVVTGEDSITQIELYLN   60
ADE45379.1          MAPKRKASSTCKTPKRQCIPKPGCCPNVASVPKLLVKGGVEVLSVVTGEDSITQIELYLN   60
ADE45387.1          MAPKRKASSTCKTPKRQCIPKPGCCPNVASVPKLLVKGGVEVLSVVTGEDSITQIELYLN   60
ADE45391.1          MAPKRKASSTCKTPKRQCIPKPGCCPNVASVPKLLVKGGVEVLSVVTGEDSITQIELYLN   60
ADE45395.1          MAPKRKASSTCKTPKRQCIPKPGCCPNVASVPKLLVKGGVEVLSVVTGEDSITQIELYLN   60
ADE45403.1          MAPKRKASSTCKTPKRQCIPKPGCCPNVASVPKLLVKGGVEVLSVVTGEDSITQIELYLN   60
ADE45407.1          MAPKRKASSTCKTPKRQCIPKPGCCPNVASVPKLLVKGGVEVLSVVTGEDSITQIELYLN   60
ADE45411.1          MAPKRKASSTCKTPKRQCIPKPGCCPNVASVPKLLVKGGVEVLSVVTGEDSITQIELYLN   60
ADE45415.1          MAPKRKASSTCKTPKRQCIPKPGCCPNVASVPKLLVKGGVEVLSVVTGEDSITQIELYLN   60
ADE45419.1          MAPKRKASSTCKTPKRQCIPKPGCCPNVASVPKLLVKGGVEVLSVVTGEDSITQIELYLN   60
CAR95364.1          MAPKRKASSTCKTPKRQCIPKPGCCPNVASVPKLLVKGGVEVLSVVTGEDSITQIELYLN   60
```

```
CAR95367.1          MAPKRKASSTCKTPKRQCIPKPGCCPNVASVPKLLVKGGVEVLSVVTGEDSITQIELYLN  60
CAR95368.1          MAPKRKASSTCKTPKRQCIPKPGCCPNVASVPKLLVKGGVEVLSVVTGEDSITQIELYLN  60
CAR95369.1          MAPKRKASSTCKTPKRQCIPKPGCCPNVASVPKLLVKGGVEVLSVVTGEDSITQIELYLN  60
ACI25319.1          MAPKRKASSTCKTPKRQCIPKPGCCPNVASVPKLLVKGGVEVLSVVTGEDSITQIELYLN  60
UQK62672.1          MAPKRKASSTCKTPKRQCIPKPGCCPNVASVPKLLVKGGVEVLSVVTGEDSITQIELYLN  60
UQK62681.1          MAPKRKASSTCKTPKRQCIPKPGCCPNVASVPKLLVKGGVEVLSVVTGEDSITQIELYLN  60
UQK62685.1          MAPKRKASSTCKTPKRQCIPKPGCCPNVASVPKLLVKGGVEVLSVVTGEDSITQIELYLN  60
UQK62690.1          MAPKRKASSTCKTPKRQCIPKPGCCPNVASVPKLLVKGGVEVLSVVTGEDSITQIELYLN  60
UQK62693.1          MAPKRKASSTCKTPKRQCIPKPGCCPNVASVPKLLVKGGVEVLSVVTGEDSITQIELYLN  60
ANF28696.1          MAPKRKASSTCKTPKRQCIPKPGCCPNVASVPKLLVKGGVEVLSVVTGEDSITQIELYLN  60
ANF28699.1          MAPKRKASSTCKTPKRQCIPKPGCCPNVASVPKLLVKGGVEVLSVVTGEDSITQIELYLN  60
AEM01083.1          MAPKRKASSTCKTPKRQCIPKPGCCPNVASVPKLLVKGGVEVLSVVTGEDSITQIELYLN  60
ADE45343.1          MAPKRKASSTCKTPKRQCIPKPGCCPNVASVPKLLVKGGVEVLSVVTGEDSITQIELYLN  60
ADE45367.1          MAPKRKASSTCKTPKRQCIPKPGCCPNVASVPKLLVKGGVEVLSVVTGEDSITQIELYLN  60
ADE45371.1          MAPKRKASSTCKTPKRQCIPKPGCCPNVASVPKLLVKGGVEVLSVVTGEDSITQIELYLN  60
ADE45399.1          MAPKRKASSTCKTPKRQCIPKPGCCPNVASVPKLLVKGGVEVLSVVTGEDSITQIELYLN  60
CAR95365.1          MAPKRKASSTCKTPKRQCIPKPGCCPNVASVPKLLVKGGVEVLSVVTGEDSITQIELYLN  60
ABY65893.1          MAPKRKASSTCKTPKRQCIPKPGCCPNVASVPKLLVKGGVEVLSVVTGEDSITQIELYLN  60
BAN78700.1          MAPKRKASSTCKTPKRQCISKSGCCPNVASVPKLLVKGGVEVLSVVTGEDSITQIELYLN  60
AHB32980.1          MAPKRKASSTCKTPKRQCISKSGCCPNVASVPKLLVKGGVEVLSVVTGEDSITQIELYLN  60
BAV01218.1          MAPKRKASSTCKTPKRQCIPKPGCCPNVASVPKLLVKGGVEVLSVVTGEDSITQIELYLN  60
BAV01220.1          MAPKRKASSTCKTPKRQCIPKPGCCPNVASVPKLLVKGGVEVLSVVTGEDSITQIELYLN  60
BAV01214.1          MAPKRKASSTCKTPKRQCISKSGCCPNVASVPKLLVKGGVEVLSVVTGEDSITQIELYLN  60
BAV01216.1          MAPKRKASSTCKTPKRQCIPKSGCCPNVASVPKLLVKGGVEVLSVVTGEDSITQIELYLN  60
BBJ26577.1          MAPKRKASSTCKTPKRQCISKSGCCPNVASVPKLLVKGGVEVLSVVTGEDSITQIELYLN  60
WKF20931.1          MAPKRKASSTCKTPKRQCIPKSGCCPNVASVPKLLVKGGVEVLSVVTGEDSITQIELYLN  60
BBJ26589.1          MAPKRKASSTCKTPKRQCIPKSGCCPNVASVPKLLVKGGVEVLSVVTGEDSITQIELYLN  60
BBJ26592.1          MAPKRKASSTCKTPKRQCIPKSGCCPNVASVPKLLVKGGVEVLSVVTGEDSITQIELYLN  60
BBJ26601.1          MAPKRKASSTCKTPKRQCIPKSGCCPNVASVPKLLVKGGVEVLSVVTGEDSITQIELYLN  60
BAV01209.1          MAPKRKASSTCKTPKRQCIPKSGCCPNVASVPKLLVKGGVEVLSVVTGEDSITQIELYLN  60
BAV01211.1          MAPKRKASSTCKTPKRQCIPKSGCCPNVASVPKLLVKGGVEVLSVVTGEDSITQIELYLN  60
BAV01212.1          MAPKRKASSTCKTPKRQCIPKSGCCPNVASVPKLLVKGGVEVLSVVTGEDSITQIELYLN  60
BAV01213.1          MAPKRKASSTCKTPKRQCIPKSGCCPNVASVPKLLVKGGVEVLSVVTGEDSITQIELYLN  60
BAV01215.1          MAPKRKASSTCKTPKRQCIPKSGCCPNVASVPKLLVKGGVEVLSVVTGEDSITQIELYLN  60
BAV01217.1          MAPKRKASSTCKTPKRQCIPKSGCCPNVASVPKLLVKGGVEVLSVVTGEDSITQIELYLN  60
BAV01219.1          MAPKRKASSTCKTPKRQCIPKSGCCPNVASVPKLLVKGGVEVLSVVTGEDSITQIELYLN  60
BAV01221.1          MAPKRKASSTCKTPKRQCIPKSGCCPNVASVPKLLVKGGVEVLSVVTGEDSITQIELYLN  60
BAV01225.1          MAPKRKASSTCKTPKRQCIPKSGCCPNVASVPKLLVKGGVEVLSVVTGEDSITQIELYLN  60
BAV01226.1          MAPKRKASSTCKTPKRQCIPKSGCCPNVASVPKLLVKGGVEVLSVVTGEDSITQIELYLN  60
BAV01227.1          MAPKRKASSTCKTPKRQCIPKSGCCPNVASVPKLLVKGGVEVLSVVTGEDSITQIELYLN  60
BAV01228.1          MAPKRKASSTCKTPKRQCIPKSGCCPNVASVPKLLVKGGVEVLSVVTGEDSITQIELYLN  60
BAN78690.1          MAPKRKASSTCKTPKRQCIPKSGCCPNVASVPKLLVKGGVEVLSVVTGEDSITQIELYLN  60
BAN78691.1          MAPKRKASSTCKTPKRQCIPKSGCCPNVASVPKLLVKGGVEVLSVVTGEDSITQIELYLN  60
BAN78696.1          MAPKRKASSTCKTPKRQCIPKSGCCPNVASVPKLLVKGGVEVLSVVTGEDSITQIELYLN  60
BAN78697.1          MAPKRKASSTCKTPKRQCIPKSGCCPNVASVPKLLVKGGVEVLSVVTGEDSITQIELYLN  60
BAN78698.1          MAPKRKASSTCKTPKRQCIPKSGCCPNVASVPKLLVKGGVEVLSVVTGEDSITQIELYLN  60
ADE45383.1          MAPKRKASSTCKTPKRQCIPKSGCCPNVASVPKLLVKGGVEVLSVVTGEDSITQIELYLN  60
ACL31697.1          MAPKRKASSTCKTPKRQCIPKSGCCPNVASVPKLLVKGGVEVLSVVTGEDSITQIELYLN  60
QBR98152.1          MAPKRKASSTCKTPKRQCISKSGCCPNVASVPKLLVKGGVEVLSVVTGEDSITQIELYLN  60
BBJ26574.1          MAPKRKASSTCKTPKRQCISKSGCCPNVASVPKLLVKGGVEVLSVVTGEDSITQIELYLN  60
BBJ26580.1          MAPKRKASSTCKTPKRQCISKSGCCPNVASVPKLLVKGGVEVLSVVTGEDSITQIELYLN  60
BBJ26595.1          MAPKRKASSTCKTPKRQCISKSGCCPNVASVPKLLVKGGVEVLSVVTGEDSITQIELYLN  60
BBJ26598.1          MAPKRKASSTCKTPKRQCISKSGCCPNVASVPKLLVKGGVEVLSVVTGEDSITQIELYLN  60
BAV01210.1          MAPKRKASSTCKTPKRQCISKSGCCPNVASVPKLLVKGGVEVLSVVTGEDSITQIELYLN  60
BAV01222.1          MAPKRKASSTCKTPKRQCISKSGCCPNVASVPKLLVKGGVEVLSVVTGEDSITQIELYLN  60
BAV01223.1          MAPKRKASSTCKTPKRQCISKSGCCPNVASVPKLLVKGGVEVLSVVTGEDSITQIELYLN  60
BAV01224.1          MAPKRKASSTCKTPKRQCISKSGCCPNVASVPKLLVKGGVEVLSVVTGEDSITQIELYLN  60
BAV01230.1          MAPKRKASSTCKTPKRQCISKSGCCPNVASVPKLLVKGGVEVLSVVTGEDSITQIELYLN  60
BAN78692.1          MAPKRKASSTCKTPKRQCISKSGCCPNVASVPKLLVKGGVEVLSVVTGEDSITQIELYLN  60
BAN78693.1          MAPKRKASSTCKTPKRQCISKSGCCPNVASVPKLLVKGGVEVLSVVTGEDSITQIELYLN  60
BAN78694.1          MAPKRKASSTCKTPKRQCISKSGCCPNVASVPKLLVKGGVEVLSVVTGEDSITQIELYLN  60
                     ************** ****: * *****:************************************

ABY65888.1          PRMGVNSPDLPTTSNWYTYTYDLQPKGSSPDQPIKENLPAYSVARVSLPMLNEDITCDTL  120
QXF78584.1          PRMGVNSPDLPTISKWYTYTYDLQPKGSSPDQPIKENLPAYSVARVSLPMLNEDITCDTL  120
QXF78586.1          PRMGVNSPDLPTISKWYTYTYDLQPKGSSPDQPIKENLPAYSVARVSLPMLNEDITCDTL  120
ANF28692.1          PRMGVNSPDLPTISKWYTYTYDLQPKGSSPDQPIKENLPAYSVARVSLPMLNEDITCDTL  120
```

AHW79948.1	PRMGVNSPDLPTISKWYTYTYDLQPKGSSPDQPIKENLPAYSVARVSLPMLNEDITCDTL	120
AWG42111.1	PRMGVNSPDLPTVSKWYTYTYDLQPKGSSPDQPIKENLPAYSVARVSLPMLNEDITCDTL	120
AWG42117.1	PRMGVNSPDLPTVSKWYTYTYDLQPKGSSPDQPIKENLPAYSVARVSLPMLNEDITCDTL	120
ANF28693.1	PRMGVNSPDLPTVSKWYTYTYDLQPKGSSPDQPIKENLPAYSVARVSLPMLNEDITCDTL	120
ANF28694.1	PRMGVNSPDLPTVSKWYTYTYDLQPKGSSPDQPIKENLPAYSVARVSLPMLNEDITCDTL	120
ANF28695.1	PRMGVNSPDLPTTSNWYTYTYDLQPKGSSPDQPIKENLPAYSVARVSLPMLNEDITCDTL	120
ANF28698.1	PRMGVNSPDLPTTSNWYTYTYDLQPKGSSPDQPIKENLPAYSVARVSLPMLNEDITCDTL	120
CAR95366.1	PRMGVNSPDLPTTSNWYTYTYDLQPKGSSPDQPIKENLPAYSVARVSLPMLNEDITCDTL	120
AFM84435.1	PRMGVNSPDLPTTSNWYTYTYDLQPKGSSPDQPIKENLPAYSVARVSLPMLNEDITCDTL	120
AFM84450.1	PRMGVNSPDLPTTSNWYTYTYDLQPKGSSPDQPIKENLPAYSVARVSLPMLNEDITCDTL	120
AFM84455.1	PRMGVNSPDLPTTSNWYTYTYDLQPKGSSPDQPIKENLPAYSVARVSLPMLNEDITCDTL	120
ANF28697.1	PRMGVNSPDLPTTSNWYTYTYDLQPKGSSPDQPIKENLPAYSVARVSLPMLNEDITCDTL	120
ACN44192.1	PRMGVNSPDLPTTSNWYTYTYDLQPKGSSPDQPIKENLPAYSVARVSLPMLNEDITCDTL	119
ADE45355.1	PRMGVNSPDLPTTSNWYTYTYDLQPKGSSPDQPIKENLPAYSVARVSLPMLNEDITCDTL	120
ADE45359.1	PRMGVNSPDLPTTSNWYTYTYDLQPKGSSPDQPIKENLPAYSVARVSLPMLNEDITCDTL	120
ADE45351.1	PRMGVNSPDLPTTSNWYTYTYDLQPKGSSPDQPIKENLPAYSVARVSLPMLNEDITCDTL	120
ADE45347.1	PRMGVNSPDLPTTSNWYTYTYDLQPKGSSPDQPIKENLPAYSVARVSLPMLNEDITCDTL	120
AEM01087.1	PRMGVNSPDLPTTSNWYTYTYDLQPKGSSPDQPIKENLPAYSVARVSLPMLNEDITCDTL	120
AFC36083.1	PRMGVNSPDLPTTSNWYTYTYDLQPKGSSPDQPIKENLPAYSVARVSLPMLNEDITCDTL	120
AFM84460.1	PRMGVNSPDLPTTSNWYTYTYDLQPKGSSPDQPIKENLPAYSVARVSLPMLNEDITCDTL	120
AHW79944.1	PRMGVNSPDLPTTSNWYTYTYDLQPKGSSPDQPIKENLPAYSVARVSLPMLNEDITCDTL	120
BBJ26583.1	PRMGVNSPDLPTTSNWYTYTYDLQPKGSSPDQPIKENLPAYSVARVSLPMLNEDITCDTL	120
BBJ26586.1	PRMGVNSPDLPTTSNWYTYTYDLQPKGSSPDQPIKENLPAYSVARVSLPMLNEDITCDTL	120
APA21079.1	PRMGVNSPDLPTTSNWYTYTYDLQPKGSSPDQPIKENLPAYSVARVSLPMLNEDITCDTL	120
ADN95996.1	PRMGVNSPDLPTTSNWYTYTYDLQPKGSSPDQPIKENLPAYSVARVSLPMLNEDITCDTL	120
YP_009111420.1	PRMGVNSPDLPTTSNWYTYTYDLQPKGSSPDQPIKENLPAYSVARVSLPMLNEDITCDTL	120
UQK62677.1	PRMGVNSPDLPTTSNWYTYTYDLQPKGSSPDQPIKENLPAYSVARVSLPMLNEDITCDTL	120
AWG42121.1	PRMGVNSPDLPTTSNWYTYTYDLQPKGSSPDQPIKENLPAYSVARVSLPMLNEDITCDTL	120
AWG42124.1	PRMGVNSPDLPTTSNWYTYTYDLQPKGSSPDQPIKENLPAYSVARVSLPMLNEDITCDTL	120
AWG42134.1	PRMGVNSPDLPTTSNWYTYTYDLQPKGSSPDQPIKENLPAYSVARVSLPMLNEDITCDTL	120
AWG42140.1	PRMGVNSPDLPTTSNWYTYTYDLQPKGSSPDQPIKENLPAYSVARVSLPMLNEDITCDTL	120
AQM73644.1	PRMGVNSPDLPTTSNWYTYTYDLQPKGSSPDQPIKENLPAYSVARVSLPMLNEDITCDTL	120
BAV01229.1	PRMGVNSPDLPTTSNWYTYTYDLQPKGSSPDQPIKENLPAYSVARVSLPMLNEDITCDTL	120
AKK25202.1	PRMGVNSPDLPTTSNWYTYTYDLQPKGSSPDQPIKENLPAYSVARVSLPMLNEDITCDTL	120
AHN49701.1	PRMGVNSPDLPTTSNWYTYTYDLQPKGSSPDQPIKENLPAYSVARVSLPMLNEDITCDTL	120
AHN49705.1	PRMGVNSPDLPTTSNWYTYTYDLQPKGSSPDQPIKENLPAYSVARVSLPMLNEDITCDTL	120
AHN49709.1	PRMGVNSPDLPTTSNWYTYTYDLQPKGSSPDQPIKENLPAYSVARVSLPMLNEDITCDTL	120
AHW79940.1	PRMGVNSPDLPTTSNWYTYTYDLQPKGSSPDQPIKENLPAYSVARVSLPMLNEDITCDTL	120
AHN13648.1	PRMGVNSPDLPTTSNWYTYTYDLQPKGSSPDQPIKENLPAYSVARVSLPMLNEDITCDTL	120
BAN78699.1	PRMGVNSPDLPTTSNWYTYTYDLQPKGSSPDQPIKENLPAYSVARVSLPMLNEDITCDTL	120
AGA03918.1	PRMGVNSPDLPTTSNWYTYTYDLQPKGSSPDQPIKENLPAYSVARVSLPMLNEDITCDTL	120
AFM84440.1	PRMGVNSPDLPTTSNWYTYTYDLQPKGSSPDQPIKENLPAYSVARVSLPMLNEDITCDTL	120
AFM84445.1	PRMGVNSPDLPTTSNWYTYTYDLQPKGSSPDQPIKENLPAYSVARVSLPMLNEDITCDTL	120
AFC36077.1	PRMGVNSPDLPTTSNWYTYTYDLQPKGSSPDQPIKENLPAYSVARVSLPMLNEDITCDTL	120
AFC36088.1	PRMGVNSPDLPTTSNWYTYTYDLQPKGSSPDQPIKENLPAYSVARVSLPMLNEDITCDTL	120
AFC36093.1	PRMGVNSPDLPTTSNWYTYTYDLQPKGSSPDQPIKENLPAYSVARVSLPMLNEDITCDTL	120
AEM01095.1	PRMGVNSPDLPTTSNWYTYTYDLQPKGSSPDQPIKENLPAYSVARVSLPMLNEDITCDTL	120
AEM01098.1	PRMGVNSPDLPTTSNWYTYTYDLQPKGSSPDQPIKENLPAYSVARVSLPMLNEDITCDTL	120
ADE45363.1	PRMGVNSPDLPTTSNWYTYTYDLQPKGSSPDQPIKENLPAYSVARVSLPMLNEDITCDTL	120
ADE45375.1	PRMGVNSPDLPTTSNWYTYTYDLQPKGSSPDQPIKENLPAYSVARVSLPMLNEDITCDTL	120
ADE45379.1	PRMGVNSPDLPTTSNWYTYTYDLQPKGSSPDQPIKENLPAYSVARVSLPMLNEDITCDTL	120
ADE45387.1	PRMGVNSPDLPTTSNWYTYTYDLQPKGSSPDQPIKENLPAYSVARVSLPMLNEDITCDTL	120
ADE45391.1	PRMGVNSPDLPTTSNWYTYTYDLQPKGSSPDQPIKENLPAYSVARVSLPMLNEDITCDTL	120
ADE45395.1	PRMGVNSPDLPTTSNWYTYTYDLQPKGSSPDQPIKENLPAYSVARVSLPMLNEDITCDTL	120
ADE45403.1	PRMGVNSPDLPTTSNWYTYTYDLQPKGSSPDQPIKENLPAYSVARVSLPMLNEDITCDTL	120
ADE45407.1	PRMGVNSPDLPTTSNWYTYTYDLQPKGSSPDQPIKENLPAYSVARVSLPMLNEDITCDTL	120
ADE45411.1	PRMGVNSPDLPTTSNWYTYTYDLQPKGSSPDQPIKENLPAYSVARVSLPMLNEDITCDTL	120
ADE45415.1	PRMGVNSPDLPTTSNWYTYTYDLQPKGSSPDQPIKENLPAYSVARVSLPMLNEDITCDTL	120
ADE45419.1	PRMGVNSPDLPTTSNWYTYTYDLQPKGSSPDQPIKENLPAYSVARVSLPMLNEDITCDTL	120
CAR95364.1	PRMGVNSPDLPTTSNWYTYTYDLQPKGSSPDQPIKENLPAYSVARVSLPMLNEDITCDTL	120
CAR95367.1	PRMGVNSPDLPTTSNWYTYTYDLQPKGSSPDQPIKENLPAYSVARVSLPMLNEDITCDTL	120
CAR95368.1	PRMGVNSPDLPTTSNWYTYTYDLQPKGSSPDQPIKENLPAYSVARVSLPMLNEDITCDTL	120
CAR95369.1	PRMGVNSPDLPTTSNWYTYTYDLQPKGSSPDQPIKENLPAYSVARVSLPMLNEDITCDTL	120
ACI25319.1	PRMGVNSPDLPTTSNWYTYTYDLQPKGSSPDQPIKENLPAYSVARVSLPMLNEDITCDTL	120
UQK62672.1	PRMGVNSPDLPTTSNWYTYTYDLQPKGSSPDQPIKENLPAYSVARVSLPMLNEDITCDTL	120
UQK62681.1	PRMGVNSPDLPTTSNWYTYTYDLQPKGSSPDQPIKENLPAYSVARVSLPMLNEDITCDTL	120
UQK62685.1	PRMGVNSPDLPTTSNWYTYTYDLQPKGSSPDQPIKENLPAYSVARVSLPMLNEDITCDTL	120

```
UQK62690.1          PRMGVNSPDLPTTSNWYTYTYDLQPKGSSPDQPIKENLPAYSVARVSLPMLNEDITCDTL   120
UQK62693.1          PRMGVNSPDLPTTSNWYTYTYDLQPKGSSPDQPIKENLPAYSVARVSLPMLNEDITCDTL   120
ANF28696.1          PRMGVNSPDLPTTSNWYTYTYDLQPKGSSPDQPIKENLPAYSVARVSLPMLNEDITCDTL   120
ANF28699.1          PRMGVNSPDLPTTSNWYTYTYDLQPKGSSPDQPIKENLPAYSVARVSLPMLNEDITCDTL   120
AEM01083.1          PRMGVNSPDLPTTSNWYTYTYDLQPKGSSPDQPIKENLPAYSVARVSLPMLNEDITCDTL   120
ADE45343.1          PRMGVNSPDLPTTSNWYTYTYDLQPKGSSPDQPIKENLPAYSVARVSLPMLNEDITCDTL   120
ADE45367.1          PRMGVNSPDLPTTSNWYTYTYDLQPKGSSPDQPIKENLPAYSVARVSLPMLNEDITCDTL   120
ADE45371.1          PRMGVNSPDLPTTSNWYTYTYDLQPKGSSPDQPIKENLPAYSVARVSLPMLNEDITCDTL   120
ADE45399.1          PRMGVNSPDLPTTSNWYTYTYDLQPKGSSPDQPIKENLPAYSVARVSLPMLNEDITCDTL   120
CAR95365.1          PRMGVNSPDLPTTSNWYTYTYDLQPKGSSPDQPIKENLPAYSVARVSLPMLNEDITCDTL   120
ABY65893.1          PRMGVNSPDLPTTSNWYTYTYDLQPKGSSPDQPIKENLPAYSVARVSLPMLNEDITCDTL   120
BAN78700.1          PRMGVNSPDLPTTSNWYTYTYDLQPKGSSPDQPIKENLPAYSVARVSLPMLNEDITCDTL   120
AHB32980.1          PRMGVNSPDLPTTSNWYTYTYDLQPKGSSPDQPIKENLPAYSVARVSLPMLNEDITCDTL   120
BAV01218.1          PRMGVNSPDLPTTSNWYTYTYDLQPKGSSPDQPIKENLPAYSVARVSLPMLNEDITCDTL   120
BAV01220.1          PRMGVNSPDLPTTSNWYTYTYDLQPKGSSPDQPIKENLPAYSVARVSLPMLNEDITCDTL   120
BAV01214.1          PRMVVNSPDLPTASNWYTYTYDLQPKGSSPDQPIKENLPAYSVARVSLPMLNEDITCDTL   120
BAV01216.1          PRMGVNSPDLPTTSNWYTYTYDLQPKGSSPDQPIKENLPAYSVARVSLPMLNEDITCDTL   120
BBJ26577.1          PRMGVNSPDLPTTSNWYTYTYDLQPKGSSPDQPIKENLPAYSVARVSLPMLNEDITCDTL   120
WKF20931.1          PRMGVNSPDLPTTSNWYTYTYDLQPKGSSPDQPIKENLPAYSVARVSLPMLNEDITCDTL   120
BBJ26589.1          PRMGVNSPDLPTTSNWYTYTYDLQPKGSSPDQPIKENLPAYSVARVSLPMLNEDITCDTL   120
BBJ26592.1          PRMGVNSPDLPTTSNWYTYTYDLQPKGSSPDQPIKENLPAYSVARVSLPMLNEDITCDTL   120
BBJ26601.1          PRMGVNSPDLPTTSNWYTYTYDLQPKGSSPDQPIKENLPAYSVARVSLPMLNEDITCDTL   120
BAV01209.1          PRMGVNSPDLPTTSNWYTYTYDLQPKGSSPDQPIKENLPAYSVARVSLPMLNEDITCDTL   120
BAV01211.1          PRMGVNSPDLPTTSNWYTYTYDLQPKGSSPDQPIKENLPAYSVARVSLPMLNEDITCDTL   120
BAV01212.1          PRMGVNSPDLPTTSNWYTYTYDLQPKGSSPDQPIKENLPAYSVARVSLPMLNEDITCDTL   120
BAV01213.1          PRMGVNSPDLPTTSNWYTYTYDLQPKGSSPDQPIKENLPAYSVARVSLPMLNEDITCDTL   120
BAV01215.1          PRMGVNSPDLPTTSNWYTYTYDLQPKGSSPDQPIKENLPAYSVARVSLPMLNEDITCDTL   120
BAV01217.1          PRMGVNSPDLPTTSNWYTYTYDLQPKGSSPDQPIKENLPAYSVARVSLPMLNEDITCDTL   120
BAV01219.1          PRMGVNSPDLPTTSNWYTYTYDLQPKGSSPDQPIKENLPAYSVARVSLPMLNEDITCDTL   120
BAV01221.1          PRMGVNSPDLPTTSNWYTYTYDLQPKGSSPDQPIKENLPAYSVARVSLPMLNEDITCDTL   120
BAV01225.1          PRMGVNSPDLPTTSNWYTYTYDLQPKGSSPDQPIKENLPAYSVARVSLPMLNEDITCDTL   120
BAV01226.1          PRMGVNSPDLPTTSNWYTYTYDLQPKGSSPDQPIKENLPAYSVARVSLPMLNEDITCDTL   120
BAV01227.1          PRMGVNSPDLPTTSNWYTYTYDLQPKGSSPDQPIKENLPAYSVARVSLPMLNEDITCDTL   120
BAV01228.1          PRMGVNSPDLPTTSNWYTYTYDLQPKGSSPDQPIKENLPAYSVARVSLPMLNEDITCDTL   120
BAN78690.1          PRMGVNSPDLPTTSNWYTYTYDLQPKGSSPDQPIKENLPAYSVARVSLPMLNEDITCDTL   120
BAN78691.1          PRMGVNSPDLPTTSNWYTYTYDLQPKGSSPDQPIKENLPAYSVARVSLPMLNEDITCDTL   120
BAN78696.1          PRMGVNSPDLPTTSNWYTYTYDLQPKGSSPDQPIKENLPAYSVARVSLPMLNEDITCDTL   120
BAN78697.1          PRMGVNSPDLPTTSNWYTYTYDLQPKGSSPDQPIKENLPAYSVARVSLPMLNEDITCDTL   120
BAN78698.1          PRMGVNSPDLPTTSNWYTYTYDLQPKGSSPDQPIKENLPAYSVARVSLPMLNEDITCDTL   120
ADE45383.1          PRMGVNSPDLPTTSNWYTYTYDLQPKGSSPDQPIKENLPAYSVARVSLPMLNEDITCDTL   120
ACL31697.1          PRMGVNSPDLPTTSNWYTYTYDLQPKGSSPDQPIKENLPAYSVARVSLPMLNEDITCDTL   120
QBR98152.1          PRMGVNSPDLPTTSNWYTYTYDLQPKGSSPDQPIKENLPAYSVARVSLPMLNEDITCDTL   120
BBJ26574.1          PRMGVNSPDLPTTSNWYTYTYDLQPKGSSPDQPIKENLPAYSVARVSLPMLNEDITCDTL   120
BBJ26580.1          PRMGVNSPDLPTTSNWYTYTYDLQPKGSSPDQPIKENLPAYSVARVSLPMLNEDITCDTL   120
BBJ26595.1          PRMGVNSPDLPTTSNWYTYTYDLQPKGSSPDQPIKENLPAYSVARVSLPMLNEDITCDTL   120
BBJ26598.1          PRMGVNSPDLPTTSNWYTYTYDLQPKGSSPDQPIKENLPAYSVARVSLPMLNEDITCDTL   120
BAV01210.1          PRMGVNSPDLPTTSNWYTYTYDLQPKGSSPDQPIKENLPAYSVARVSLPMLNEDITCDTL   120
BAV01222.1          PRMGVNSPDLPTTSNWYTYTYDLQPKGSSPDQPIKENLPAYSVARVSLPMLNEDITCDTL   120
BAV01223.1          PRMGVNSPDLPTTSNWYTYTYDLQPKGSSPDQPIKENLPAYSVARVSLPMLNEDITCDTL   120
BAV01224.1          PRMGVNSPDLPTTSNWYTYTYDLQPKGSSPDQPIKENLPAYSVARVSLPMLNEDITCDTL   120
BAV01230.1          PRMGVNSPDLPTTSNWYTYTYDLQPKGSSPDQPIKENLPAYSVARVSLPMLNEDITCDTL   120
BAN78692.1          PRMGVNSPDLPTTSNWYTYTYDLQPKGSSPDQPIKENLPAYSVARVSLPMLNEDITCDTL   120
BAN78693.1          PRMGVNSPDLPTTSNWYTYTYDLQPKGSSPDQPIKENLPAYSVARVSLPMLNEDITCDTL   120
BAN78694.1          PRMGVNSPDLPTTSNWYTYTYDLQPKGSSPDQPIKENLPAYSVARVSLPMLNEDITCDTL   120
                    *** ******** *:*********************************************

ABY65888.1          QMWEAISVKTEVVGISSLINVHYWDMKRVHDYGAGIPVSGVNYHMFAIGGEPLDLQGLVL   180
QXF78584.1          QMWEAISVKTEVVGISSLINVHYWDMKRVHDYGAGIPVSGVNYHMFAIGGEPLDLQGLVL   180
QXF78586.1          QMWEAISVKTEVVGISSLINVHYWDMKRVHDYGAGIPVSGVNYHMFAIGGEPLDLQGLVL   180
ANF28692.1          QMWEAISVKTEVVGISSLINVHYWDMKRVHDYGAGIPVSGVNYHMFAIGGEPLDLQGLVL   180
AHW79948.1          QMWEAISVKTEVVGISSLINVHYWDMKRVHDYGAGIPVSGVNYHMFAIGGEPLDLQGLVL   180
AWG42111.1          QMWEAISVKTEVVGISSLINVHYWDMKRVHDYGAGIPVSGVNYHMFAIGGELLDLQGLVL   180
AWG42117.1          QMWEAISVKTEVVGISSLINVHYWDMKRVHDYGAGIPVSGVNYHMFAIGGELLDLQGLVL   180
ANF28693.1          QMWEAISVKTEVVGISSLINVHYWDMKRVHDYGAGIPVSGVNYHMFAIGGEPLDLQGLVL   180
ANF28694.1          QMWEAISVKTEVVGISSLINVHYWDMKRVHDYGAGIPVSGVNYHMFAIGGEPLDLQGLVL   180
ANF28695.1          QMWEAISVKTEVVGISSLINVHYWDMKRVHDYGAGIPVSGVNYHMFAIGGEPLDLQGLVL   180
ANF28698.1          QMWEAISVKTEVVGISSLINVHYWDMKRVHDYGAGIPVSGVNYHMFAIGGEPLDLQGLVL   180
```

CAR95366.1	QMWEAISVKTEVVGISSLINVHYWDMKRVHDYGAGIPVSGVNYHMFAIGGEPLDLQGLVL	180
AFM84435.1	QMWEAISVKTEVVGISSLINVHYWDMKRVHDYGAGIPVSGVNYHMFAIGGEPLDLQGLVL	180
AFM84450.1	QMWEAISVKTEVVGISSLINVHYWDMKRVHDYGAGIPVSGVNYHMFAIGGEPLDLQGLVL	180
AFM84455.1	QMWEAISVKTEVVGISSLINVHYWDMKRVHDYGAGIPVSGVNYHMFAIGGEPLDLQGLVL	180
ANF28697.1	QMWEAISVKTEVVGISSLINVHYWDMKRVHDYGAGIPVSGVNYHMFAIGGEPLDLQGLVL	180
ACN44192.1	QMWEAISVKTEVVGISSLINVHYWDMKRVHDYGAGIPVSGVNYHMFAIGGEPLDLQGLVL	179
ADE45355.1	QMWEAISVKTEVVGISSLINVHYWDMKRVHDYGAGIPVSGVNYHMFAIGGEPLDLQGLVL	180
ADE45359.1	QMWEAISVKTEVVGISSLINVHYWDMKRVHDYGAGIPVSGVNYHMFAIGGEPLDLQGLVL	180
ADE45351.1	QMWEAISVKTEVVGISSLINVHYWDMKRVHDYGAGIPVSGVNYHMFAIGGEPLDLQGLVL	180
ADE45347.1	QMWEAISVKTEVVGISSLINVHYWDMKRVHDYGAGIPVSGVNYHMFAIGGEPLDLQGLVL	180
AEM01087.1	QMWEAISVKTEVVGISSLINVHYWDMKRVHDYGAGIPVSGVNYHMFAIGGEPLDLQGLVL	180
AFC36083.1	QMWEAISVKTEVVGISSLINVHYWDMKRVHDYGAGIPVSGVNYHMFAIGGEPLDLQGLVL	180
AFM84460.1	QMWEAISVKTEVVGISSLINVHYWDMKRVHDYGAGIPVSGVNYHMFAIGGEPLDLQGLVL	180
AHW79944.1	QMWEAISVKTEVVGISSLINVHYWDMKRVHDYGAGIPVSGVNYHMFAIGGEPLDLQGLVL	180
BBJ26583.1	QMWEAISVKTEVVGISSLINVHYWDMKRVHDYGAGIPVSGVNYHMFAIGGEPLDLQGLVL	180
BBJ26586.1	QMWEAISVKTEVVGISSLINVHYWDMKRVHDYGAGIPVSGVNYHMFAIGGEPLDLQGLVL	180
APA21079.1	QMWEAISVKTEVVGISSLINVHYWDMKRVHDYGAGIPVSGVNYHMFAIGGEPLDLQGLVL	180
ADN95996.1	QMWEAISVKTEVVGISSLINVHYWDMKRVHDYGAGIPVSGVNYHMFAIGGEPLDLQGLVL	180
YP_009111420.1	QMWEAISVKTEVVGISSLINVHYWDMKRVHDYGAGIPVSGVNYHMFAIGGEPLDLQGLVL	180
UQK62677.1	QMWEAISVKTEVVGISSLINVHYWDMKRVHDYGAGIPVSGVNYHMFAIGGEPLDLQGLVL	180
AWG42121.1	QMWEAISVKTEVVGISSLINVHYWDMKRVHDYGAGIPVSGVNYHMFAIGGEPLDLQGLVL	180
AWG42124.1	QMWEAISVKTEVVGISSLINVHYWDMKRVHDYGAGIPVSGVNYHMFAIGGEPLDLQGLVL	180
AWG42134.1	QMWEAISVKTEVVGISSLINVHYWDMKRVHDYGAGIPVSGVNYHMFAIGGEPLDLQGLVL	180
AWG42140.1	QMWEAISVKTEVVGISSLINVHYWDMKRVHDYGAGIPVSGVNYHMFAIGGEPLDLQGLVL	180
AQM73644.1	QMWEAISVKTEVVGISSLINVHYWDMKRVHDYGAGIPVSGVNYHMFAIGGEPLDLQGLVL	180
BAV01229.1	QMWEAISVKTEVVGISSLINVHYWDMKRVHDYGAGIPVSGVNYHMFAIGGEPLDLQGLVL	180
AKK25202.1	QMWEAISVKTEVVGISSLINVHYWDMKRVHDYGAGIPVSGVNYHMFAIGGEPLDLQGLVL	180
AHN49701.1	QMWEAISVKTEVVGISSLINVHYWDMKRVHDYGAGIPVSGVNYHMFAIGGEPLDLQGLVL	180
AHN49705.1	QMWEAISVKTEVVGISSLINVHYWDMKRVHDYGAGIPVSGVNYHMFAIGGEPLDLQGLVL	180
AHN49709.1	QMWEAISVKTEVVGISSLINVHYWDMKRVHDYGAGIPVSGVNYHMFAIGGEPLDLQGLVL	180
AHW79940.1	QMWEAISVKTEVVGISSLINVHYWDMKRVHDYGAGIPVSGVNYHMFAIGGEPLDLQGLVL	180
AHN13648.1	QMWEAISVKTEVVGISSLINVHYWDMKRVHDYGAGIPVSGVNYHMFAIGGEPLDLQGLVL	180
BAN78699.1	QMWEAISVKTEVVGISSLINVHYWDMKRVHDYGAGIPVSGVNYHMFAIGGEPLDLQGLVL	180
AGA03918.1	QMWEAISVKTEVVGISSLINVHYWDMKRVHDYGAGIPVSGVNYHMFAIGGEPLDLQGLVL	180
AFM84440.1	QMWEAISVKTEVVGISSLINVHYWDMKRVHDYGAGIPVSGVNYHMFAIGGEPLDLQGLVL	180
AFM84445.1	QMWEAISVKTEVVGISSLINVHYWDMKRVHDYGAGIPVSGVNYHMFAIGGEPLDLQGLVL	180
AFC36077.1	QMWEAISVKTEVVGISSLINVHYWDMKRVHDYGAGIPVSGVNYHMFAIGGEPLDLQGLVL	180
AFC36088.1	QMWEAISVKTEVVGISSLINVHYWDMKRVHDYGAGIPVSGVNYHMFAIGGEPLDLQGLVL	180
AFC36093.1	QMWEAISVKTEVVGISSLINVHYWDMKRVHDYGAGIPVSGVNYHMFAIGGEPLDLQGLVL	180
AEM01095.1	QMWEAISVKTEVVGISSLINVHYWDMKRVHDYGAGIPVSGVNYHMFAIGGEPLDLQGLVL	180
AEM01098.1	QMWEAISVKTEVVGISSLINVHYWDMKRVHDYGAGIPVSGVNYHMFAIGGEPLDLQGLVL	180
ADE45363.1	QMWEAISVKTEVVGISSLINVHYWDMKRVHDYGAGIPVSGVNYHMFAIGGEPLDLQGLVL	180
ADE45375.1	QMWEAISVKTEVVGISSLINVHYWDMKRVHDYGAGIPVSGVNYHMFAIGGEPLDLQGLVL	180
ADE45379.1	QMWEAISVKTEVVGISSLINVHYWDMKRVHDYGAGIPVSGVNYHMFAIGGEPLDLQGLVL	180
ADE45387.1	QMWEAISVKTEVVGISSLINVHYWDMKRVHDYGAGIPVSGVNYHMFAIGGEPLDLQGLVL	180
ADE45391.1	QMWEAISVKTEVVGISSLINVHYWDMKRVHDYGAGIPVSGVNYHMFAIGGEPLDLQGLVL	180
ADE45395.1	QMWEAISVKTEVVGISSLINVHYWDMKRVHDYGAGIPVSGVNYHMFAIGGEPLDLQGLVL	180
ADE45403.1	QMWEAISVKTEVVGISSLINVHYWDMKRVHDYGAGIPVSGVNYHMFAIGGEPLDLQGLVL	180
ADE45407.1	QMWEAISVKTEVVGISSLINVHYWDMKRVHDYGAGIPVSGVNYHMFAIGGEPLDLQGLVL	180
ADE45411.1	QMWEAISVKTEVVGISSLINVHYWDMKRVHDYGAGIPVSGVNYHMFAIGGEPLDLQGLVL	180
ADE45415.1	QMWEAISVKTEVVGISSLINVHYWDMKRVHDYGAGIPVSGVNYHMFAIGGEPLDLQGLVL	180
ADE45419.1	QMWEAISVKTEVVGISSLINVHYWDMKRVHDYGAGIPVSGVNYHMFAIGGEPLDLQGLVL	180
CAR95364.1	QMWEAISVKTEVVGISSLINVHYWDMKRVHDYGAGIPVSGVNYHMFAIGGEPLDLQGLVL	180
CAR95367.1	QMWEAISVKTEVVGISSLINVHYWDMKRVHDYGAGIPVSGVNYHMFAIGGEPLDLQGLVL	180
CAR95368.1	QMWEAISVKTEVVGISSLINVHYWDMKRVHDYGAGIPVSGVNYHMFAIGGEPLDLQGLVL	180
CAR95369.1	QMWEAISVKTEVVGISSLINVHYWDMKRVHDYGAGIPVSGVNYHMFAIGGEPLDLQGLVL	180
ACI25319.1	QMWEAISVKTEVVGISSLINVHYWDMKRVHDYGAGIPVSGVNYHMFAIGGEPLDLQGLVL	180
UQK62672.1	QMWEAISVKTEVVGISSLINVHYWDMKRVHDYGAGIPVSGVNYHMFAIGGEPLDLQGLVL	180
UQK62681.1	QMWEAISVKTEVVGISSLINVHYWDMKRVHDYGAGIPVSGVNYHMFAIGGEPLDLQGLVL	180
UQK62685.1	QMWEAISVKTEVVGISSLINVHYWDMKRVHDYGAGIPVSGVNYHMFAIGGEPLDLQGLVL	180
UQK62690.1	QMWEAISVKTEVVGISSLINVHYWDMKRVHDYGAGIPVSGVNYHMFAIGGEPLDLQGLVL	180
UQK62693.1	QMWEAISVKTEVVGISSLINVHYWDMKRVHDYGAGIPVSGVNYHMFAIGGEPLDLQGLVL	180
ANF28696.1	QMWEAISVKTEVVGISSLINVHYWDMKRVHDYGAGIPVSGVNYHMFAIGGEPLDLQGLVL	180
ANF28699.1	QMWEAISVKTEVVGISSLINVHYWDMKRVHDYGAGIPVSGVNYHMFAIGGEPLDLQGLVL	180
AEM01083.1	QMWEAISVKTEVVGISSLINVHYWDMKRVHDYGAGIPVSGVNYHMFAIGGEPLDLQGLVL	180
ADE45343.1	QMWEAISVKTEVVGISSLINVHYWDMKRVHDYGAGIPVSGVNYHMFAIGGEPLDLQGLVL	180
ADE45367.1	QMWEAISVKTEVVGISSLINVHYWDMKRVHDYGAGIPVSGVNYHMFAIGGEPLDLQGLVL	180

```
ADE45371.1          QMWEAISVKTEVVGISSLINVHYWDMKRVHDYGAGIPVSGVNYHMFAIGGEPLDLQGLVL   180
ADE45399.1          QMWEAISVKTEVVGISSLINVHYWDMKRVHDYGAGIPVSGVNYHMFAIGGEPLDLQGLVL   180
CAR95365.1          QMWEAISVKTEVVGISSLINVHYWDMKRVHDYGAGIPVSGVNYHMFAIGGEPLDLQGLVL   180
ABY65893.1          QMWEAISVKTEVVGISSLINVHYWDMKRVHDYGAGIPVSGVNYHMFAIGGEPLDLQGLVL   180
BAN78700.1          QMWEAISVKTEVVGISSLINVHYWDMKRVHDYGAGIPVSGVNYHMFAIGGEPLDLQGLVL   180
AHB32980.1          QMWEAISVKTEVVGISSLINVHYWDMKRVHDYGAGIPVSGVNYHMFAIGGEPLDLQGLVL   180
BAV01218.1          QMWEAISVKTEVVGISSLINVHYWDMKRVHDYGAGIPVSGVNYHMFAIGGEPLDLQGLVL   180
BAV01220.1          QMWEAISVKTEVVGISSLINVHYWDMKRVHDYGAGIPVSGVNYHMFAIGGEPLDLQGLVL   180
BAV01214.1          QMWEAISVKTEVVGISSLINVHYWDMKRVHDYGAGIPVSGVNYHMFAIGGEPLDLQGLVL   180
BAV01216.1          QMWEAISVKTEVVGISSLINVHYWDMKRVHDYGAGIPVSGVNYHMFAIGGEPLDLQGLVL   180
BBJ26577.1          QMWEAISVKTEVVGISSLINVHYWDMKRVHDYGAGIPVSGVNYHMFAIGGEPLDLQGLVL   180
WKF20931.1          QMWEAISVKTEVVGISSLINVHYWDMKRVHDYGAGIPVSGVNYHMFAIGGEPLDLQGLVL   180
BBJ26589.1          QMWEAISVKTEVVGISSLINVHYWDMKRVHDYGAGIPVSGVNYHMFAIGGEPLDLQGLVL   180
BBJ26592.1          QMWEAISVKTEVVGISSLINVHYWDMKRVHDYGAGIPVSGVNYHMFAIGGEPLDLQGLVL   180
BBJ26601.1          QMWEAISVKTEVVGISSLINVHYWDMKRVHDYGAGIPVSGVNYHMFAIGGEPLDLQGLVL   180
BAV01209.1          QMWEAISVKTEVVGISSLINVHYWDMKRVHDYGAGIPVSGVNYHMFAIGGEPLDLQGLVL   180
BAV01211.1          QMWEAISVKTEVVGISSLINVHYWDMKRVHDYGAGIPVSGVNYHMFAIGGEPLDLQGLVL   180
BAV01212.1          QMWEAISVKTEVVGISSLINVHYWDMKRVHDYGAGIPVSGVNYHMFAIGGEPLDLQGLVL   180
BAV01213.1          QMWEAISVKTEVVGISSLINVHYWDMKRVHDYGAGIPVSGVNYHMFAIGGEPLDLQGLVL   180
BAV01215.1          QMWEAISVKTEVVGISSLINVHYWDMKRVHDYGAGIPVSGVNYHMFAIGGEPLDLQGLVL   180
BAV01217.1          QMWEAISVKTEVVGISSLINVHYWDMKRVHDYGAGIPVSGVNYHMFAIGGEPLDLQGLVL   180
BAV01219.1          QMWEAISVKTEVVGISSLINVHYWDMKRVHDYGAGIPVSGVNYHMFAIGGEPLDLQGLVL   180
BAV01221.1          QMWEAISVKTEVVGISSLINVHYWDMKRVHDYGAGIPVSGVNYHMFAIGGEPLDLQGLVL   180
BAV01225.1          QMWEAISVKTEVVGISSLINVHYWDMKRVHDYGAGIPVSGVNYHMFAIGGEPLDLQGLVL   180
BAV01226.1          QMWEAISVKTEVVGISSLINVHYWDMKRVHDYGAGIPVSGVNYHMFAIGGEPLDLQGLVL   180
BAV01227.1          QMWEAISVKTEVVGISSLINVHYWDMKRVHDYGAGIPVSGVNYHMFAIGGEPLDLQGLVL   180
BAV01228.1          QMWEAISVKTEVVGISSLINVHYWDMKRVHDYGAGIPVSGVNYHMFAIGGEPLDLQGLVL   180
BAN78690.1          QMWEAISVKTEVVGISSLINVHYWDMKRVHDYGAGIPVSGVNYHMFAIGGEPLDLQGLVL   180
BAN78691.1          QMWEAISVKTEVVGISSLINVHYWDMKRVHDYGAGIPVSGVNYHMFAIGGEPLDLQGLVL   180
BAN78696.1          QMWEAISVKTEVVGISSLINVHYWDMKRVHDYGAGIPVSGVNYHMFAIGGEPLDLQGLVL   180
BAN78697.1          QMWEAISVKTEVVGISSLINVHYWDMKRVHDYGAGIPVSGVNYHMFAIGGEPLDLQGLVL   180
BAN78698.1          QMWEAISVKTEVVGISSLINVHYWDMKRVHDYGAGIPVSGVNYHMFAIGGEPLDLQGLVL   180
ADE45383.1          QMWEAISVKTEVVGISSLINVHYWDMKRVHDYGAGIPVSGVNYHMFAIGGEPLDLQGLVL   180
ACL31697.1          QMWEAISVKTEVVGISSLINVHYWDMKRVHDYGAGIPVSGVNYHMFAIGGEPLDLQGLVL   180
QBR98152.1          QMWEAISVKTEVVGISSLINVHYWDMKRVHDYGAGIPVSGVNYHMFAIGGEPLDLQGLVL   180
BBJ26574.1          QMWEAISVKTEVVGISSLINVHYWDMKRVHDYGAGIPVSGVNYHMFAIGGEPLDLQGLVL   180
BBJ26580.1          QMWEAISVKTEVVGISSLINVHYWDMKRVHDYGAGIPVSGVNYHMFAIGGEPLDLQGLVL   180
BBJ26595.1          QMWEAISVKTEVVGISSLINVHYWDMKRVHDYGAGIPVSGVNYHMFAIGGEPLDLQGLVL   180
BBJ26598.1          QMWEAISVKTEVVGISSLINVHYWDMKRVHDYGAGIPVSGVNYHMFAIGGEPLDLQGLVL   180
BAV01210.1          QMWEAISVKTEVVGISSLINVHYWDMKRVHDYGAGIPVSGVNYHMFAIGGEPLDLQGLVL   180
BAV01222.1          QMWEAISVKTEVVGISSLINVHYWDMKRVHDYGAGIPVSGVNYHMFAIGGEPLDLQGLVL   180
BAV01223.1          QMWEAISVKTEVVGISSLINVHYWDMKRVHDYGAGIPVSGVNYHMFAIGGEPLDLQGLVL   180
BAV01224.1          QMWEAISVKTEVVGISSLINVHYWDMKRVHDYGAGIPVSGVNYHMFAIGGEPLDLQGLVL   180
BAV01230.1          QMWEAISVKTEVVGISSLINVHYWDMKRVHDYGAGIPVSGVNYHMFAIGGEPLDLQGLVL   180
BAN78692.1          QMWEAISVKTEVVGISSLINVHYWDMKRVHDYGAGIPVSGVNYHMFAIGGEPLDLQGLVL   180
BAN78693.1          QMWEAISVKTEVVGISSLINVHYWDMKRVHDYGAGIPVSGVNYHMFAIGGEPLDLQGLVL   180
BAN78694.1          QMWEAISVKTEVVGISSLINVHYWDMKRVHDYGAGIPVSGVNYHMFAIGGEPLDLQGLVL   180
                    *************************************************** ********

ABY65888.1          DYQTEYPKTTNGGPITIETVLGRKMTPKNQGLDPQAKAKLDKDGNYPIEVWCPDPSKNEN   240
QXF78584.1          DYQTEYPQTTNGGPITIETVLGRKMTPKNQGLDPQAKAKLDKDGNYPVEVWCPDPSKNEN   240
QXF78586.1          DYQTEYPQTTNGGPITIETVLGRKMTPKNQGLDPQAKAKLDKDGNYPVEVWCPDPSKNEN   240
ANF28692.1          DYQTEYPQTTNGGPITIETVLGRKMTPKNQGLDPQAKAKLDKDGNYPVEVWCPDPSKNEN   240
AHW79948.1          DYQTEYPQTTNGGPITIETVLGRKMTPKNQGLDPQAKAKLDKDGNYPVEVWCPDPSKNEN   240
AWG42111.1          DYQTEYPQTTNGGPITIETVLGRKMTPKNQGLDPQAKAKLDKDGNYPVEVWCPDPSKNEN   240
AWG42117.1          DYQTEYPQTTNGGPITIETVLGRKMTPKNQGLDPQAKAKLDKDGNYPVEVWCPDPSKNEN   240
ANF28693.1          DYQTEYPQTTNGGPITIETVLGRKMTPKNQGLDPQAKAKLDKDGNYPVEVWCPDPSKNEN   240
ANF28694.1          DYQTEYPQTTNGGPITIETVLGRKMTPKNQGLDPQAKAKLDKDGNYPVEVWCPDPSKNEN   240
ANF28695.1          DYQTEYPKTTNGGPITIETVLGRKMTPKNQGLDPQAKAKLDKDGNYPIEVWCPDPSKNEN   240
ANF28698.1          DYQTEYPKTTNGGPITIETVLGRKMTPKNQGLDPQAKAKLDKDGNYPIEVWCPDPSKNEN   240
CAR95366.1          GYQTEYPKTTNGGPITIETVLGRKMTPKNQGLDPQAKAKLDKDGNYPIEVWCPDPSKNEN   240
AFM84435.1          DYQTEYPKTTNGGPITIETVLGRKMTPKNQGLDPQAKAKLDKDGNYPIEVWCPDPSKNEN   240
AFM84450.1          DYQTEYPKTTNGGPITIETVLGRKMTPKNQGLDPQAKAKLDKDGNYPIEVWCPDPSKNEN   240
AFM84455.1          DYQTEYPKTTNGGPITIETVLGRKMTPKNQGLDPQAKAKLDKDGNYPIEVWCPDPSKNEN   240
ANF28697.1          DYQTQYPKTTNGGPITIETILGRKMTPKNQGLDPQAKAKLDKDGNYPIEVWCPDPSKNEN   240
ACN44192.1          DYQTQYPKTTNGGPITIETVLGRKMTPKNQGLDPQAKAKLDKDGNYPIEVWCPDPSKNEN   239
ADE45355.1          DYQTEYPKTTNGGPITIETILGRKMTPKNQGLDPQAKAKLDKDGNYPIEVWCPDPSKNEN   240
```

ADE45359.1	DYQTEYPKTTNGGPITIETILGRKMTPKNQGLDPQAKAKLDKDGNYPIEVWCPDPSKNEN	240
ADE45351.1	DYQTEYPKTTNGGPITIETVLGRKMTPKNQGLDPQAKAKLDKDGNYPIEVWCPDPSKNEN	240
ADE45347.1	DYQTEYPKTTNGGPITIETVLGRKMTPKNQGLDPQAKAKLDKDGNYPIEVWCPDPSKNEN	240
AEM01087.1	DYQTEYPKTTNGGPITIETVLGRKMTPKNQGLDPQAKAKLDKDGNYPIEVWCPDPSKNEN	240
AFC36083.1	DYQTEYPKTTNGGPITIETVLGRKMTPKNQGLDPQAKAKLDKDGNYPIEVWCPHPSKNEN	240
AFM84460.1	DYQTQYPKTTNGGPITIETVLGRKMTPKNQGLDPQAKAKLDKDGNYPIEVWCPDPSKNEN	240
AHW79944.1	DYQTQYPKTTNGGPITIETVLGRKMTPKNQGLDPQAKAKLDKDGNYPIEVWCPDPSKNEN	240
BBJ26583.1	DYQTEYPKTTNGGPITIETVLGRKMTPKNQGLDPQAKAKLDKDGNYPIEVWCPDPSKNEN	240
BBJ26586.1	DYQTEYPKTTNGGPITIETVLGRKMTPKNQGLDPQAKAKLDKDGNYPIEVWCPDPSKNEN	240
APA21079.1	DYQTEYPKTTNGGPITIETVLGRKMTPKNQGLDPQAKAKLDKDGNYPIEVWCPDPSKNEN	240
ADN95996.1	DYQTEYPKTTNGGPITIETVLGRKMTPKNQGLDPQAKAKLDKDGNYPIEVWCPDPSKNEN	240
YP_009111420.1	DYQTEYPKTTNGGPITIETVLGRKMTPKNQGLDPQAKAKLDKDGNYPIEVWCPDPSKNEN	240
UQK62677.1	DYQTEYPKTTNGGPITIETVLGRKMTPKNQGLDPQAKAKLDKDGNYPIEVWCPDPSKNEN	240
AWG42121.1	DYQTEYPKTTNGGPITIETVLGRKMTPKNQGLDPQAKAKLDKDGNYPIEVWCPDPSKNEN	240
AWG42124.1	DYQTEYPKTTNGGPITIETVLGRKMTPKNQGLDPQAKAKLDKDGNYPIEVWCPDPSKNEN	240
AWG42134.1	DYQTEYPKTTNGGPITIETVLGRKMTPKNQGLDPQAKAKLDKDGNYPIEVWCPDPSKNEN	240
AWG42140.1	DYQTEYPKTTNGGPITIETVLGRKMTPKNQGLDPQAKAKLDKDGNYPIEVWCPDPSKNEN	240
AQM73644.1	DYQTEYPKTTNGGPITIETVLGRKMTPKNQGLDPQAKAKLDKDGNYPIEVWCPDPSKNEN	240
BAV01229.1	DYQTEYPKTTNGGPITIETVLGRKMTPKNQGLDPQAKAKLDKDGNYPIEVWCPDPSKNEN	240
AKK25202.1	DYQTEYPKTTNGGPITIETVLGRKMTPKNQGLDPQAKAKLDKDGNYPIEVWCPDPSKNEN	240
AHN49701.1	DYQTEYPKTTNGGPITIETVLGRKMTPKNQGLDPQAKAKLDKDGNYPIEVWCPDPSKNEN	240
AHN49705.1	DYQTEYPKTTNGGPITIETVLGRKMTPKNQGLDPQAKAKLDKDGNYPIEVWCPDPSKNEN	240
AHN49709.1	DYQTEYPKTTNGGPITIETVLGRKMTPKNQGLDPQAKAKLDKDGNYPIEVWCPDPSKNEN	240
AHW79940.1	DYQTEYPKTTNGGPITIETVLGRKMTPKNQGLDPQAKAKLDKDGNYPIEVWCPDPSKNEN	240
AHN13648.1	DYQTEYPKTTNGGPITIETVLGRKMTPKNQGLDPQAKAKLDKDGNYPIEVWCPDPSKNEN	240
BAN78699.1	DYQTEYPKTTNGGPITIETVLGRKMTPKNQGLDPQAKAKLDKDGNYPIEVWCPDPSKNEN	240
AGA03918.1	DYQTEYPKTTNGGPITIETVLGRKMTPKNQGLDPQAKAKLDKDGNYPIEVWCPDPSKNEN	240
AFM84440.1	DYQTEYPKTTNGGPITIETVLGRKMTPKNQGLDPQAKAKLDKDGNYPIEVWCPDPSKNEN	240
AFM84445.1	DYQTEYPKTTNGGPITIETVLGRKMTPKNQGLDPQAKAKLDKDGNYPIEVWCPDPSKNEN	240
AFC36077.1	DYQTEYPKTTNGGPITIETVLGRKMTPKNQGLDPQAKAKLDKDGNYPIEVWCPDPSKNEN	240
AFC36088.1	DYQTEYPKTTNGGPITIETVLGRKMTPKNQGLDPQAKAKLDKDGNYPIEVWCPDPSKNEN	240
AFC36093.1	DYQTEYPKTTNGGPITIETVLGRKMTPKNQGLDPQAKAKLDKDGNYPIEVWCPDPSKNEN	240
AEM01095.1	DYQTEYPKTTNGGPITIETVLGRKMTPKNQGLDPQAKAKLDKDGNYPIEVWCPDPSKNEN	240
AEM01098.1	DYQTEYPKTTNGGPITIETVLGRKMTPKNQGLDPQAKAKLDKDGNYPIEVWCPDPSKNEN	240
ADE45363.1	DYQTEYPKTTNGGPITIETVLGRKMTPKNQGLDPQAKAKLDKDGNYPIEVWCPDPSKNEN	240
ADE45375.1	DYQTEYPKTTNGGPITIETVLGRKMTPKNQGLDPQAKAKLDKDGNYPIEVWCPDPSKNEN	240
ADE45379.1	DYQTEYPKTTNGGPITIETVLGRKMTPKNQGLDPQAKAKLDKDGNYPIEVWCPDPSKNEN	240
ADE45387.1	DYQTEYPKTTNGGPITIETVLGRKMTPKNQGLDPQAKAKLDKDGNYPIEVWCPDPSKNEN	240
ADE45391.1	DYQTEYPKTTNGGPITIETVLGRKMTPKNQGLDPQAKAKLDKDGNYPIEVWCPDPSKNEN	240
ADE45395.1	DYQTEYPKTTNGGPITIETVLGRKMTPKNQGLDPQAKAKLDKDGNYPIEVWCPDPSKNEN	240
ADE45403.1	DYQTEYPKTTNGGPITIETVLGRKMTPKNQGLDPQAKAKLDKDGNYPIEVWCPDPSKNEN	240
ADE45407.1	DYQTEYPKTTNGGPITIETVLGRKMTPKNQGLDPQAKAKLDKDGNYPIEVWCPDPSKNEN	240
ADE45411.1	DYQTEYPKTTNGGPITIETVLGRKMTPKNQGLDPQAKAKLDKDGNYPIEVWCPDPSKNEN	240
ADE45415.1	DYQTEYPKTTNGGPITIETVLGRKMTPKNQGLDPQAKAKLDKDGNYPIEVWCPDPSKNEN	240
ADE45419.1	DYQTEYPKTTNGGPITIETVLGRKMTPKNQGLDPQAKAKLDKDGNYPIEVWCPDPSKNEN	240
CAR95364.1	DYQTEYPKTTNGGPITIETVLGRKMTPKNQGLDPQAKAKLDKDGNYPIEVWCPDPSKNEN	240
CAR95367.1	DYQTEYPKTTNGGPITIETVLGRKMTPKNQGLDPQAKAKLDKDGNYPIEVWCPDPSKNEN	240
CAR95368.1	DYQTEYPKTTNGGPITIETVLGRKMTPKNQGLDPQAKAKLDKDGNYPIEVWCPDPSKNEN	240
CAR95369.1	DYQTEYPKTTNGGPITIETVLGRKMTPKNQGLDPQAKAKLDKDGNYPIEVWCPDPSKNEN	240
ACI25319.1	DYQTEYPKTTNGGPITIETVLGRKMTPKNQGLDPQAKAKLDKDGNYPIEVWCPDPSKNEN	240
UQK62672.1	DYQTQYPKTTNGGPITIETVLGRKMTPKNQGLDPQAKAKLDKDGNYPIEVWCPDPSKNEN	240
UQK62681.1	DYQTQYPKTTNGGPITIETVLGRKMTPKNQGLDPQAKAKLDKDGNYPIEVWCPDPSKNEN	240
UQK62685.1	DYQTQYPKTTNGGPITIETVLGRKMTPKNQGLDPQAKAKLDKDGNYPIEVWCPDPSKNEN	240
UQK62690.1	DYQTQYPKTTNGGPITIETVLGRKMTPKNQGLDPQAKAKLDKDGNYPIEVWCPDPSKNEN	240
UQK62693.1	DYQTQYPKTTNGGPITIETVLGRKMTPKNQGLDPQAKAKLDKDGNYPIEVWCPDPSKNEN	240
ANF28696.1	DYQTQYPKTTNGGPITIETVLGRKMTPKNQGLDPQAKAKLDKDGNYPIEVWCPDPSKNEN	240
ANF28699.1	DYQTQYPKTTNGGPITIETVLGRKMTPKNQGLDPQAKAKLDKDGNYPIEVWCPDPSKNEN	240
AEM01083.1	DYQTQYPKTTNGGPITIETVLGRKMTPKNQGLDPQAKAKLDKDGNYPIEVWCPDPSKNEN	240
ADE45343.1	DYQTQYPKTTNGGPITIETVLGRKMTPKNQGLDPQAKAKLDKDGNYPIEVWCPDPSKNEN	240
ADE45367.1	DYQTQYPKTTNGGPITIETVLGRKMTPKNQGLDPQAKAKLDKDGNYPIEVWCPDPSKNEN	240
ADE45371.1	DYQTQYPKTTNGGPITIETVLGRKMTPKNQGLDPQAKAKLDKDGNYPIEVWCPDPSKNEN	240
ADE45399.1	DYQTQYPKTTNGGPITIETVLGRKMTPKNQGLDPQAKAKLDKDGNYPIEVWCPDPSKNEN	240
CAR95365.1	DYQTQYPKTTNGGPITIETVLGRKMTPKNQGLDPQAKAKLDKDGNYPIEVWCPDPSKNEN	240
ABY65893.1	DYQTQYPKTTNGGPITIETVLGRKMTPKNQGLDPQAKAKLDKDGNYPIEVWCPDPSKNEN	240
BAN78700.1	DYQTEYPKTTNGGPITIETVLGRKMTPKNQGLDPQAKAKLDKDGNYPIEVWCPDPSKNEN	240
AHB32980.1	DYQTEYPKTTNGGPITIETVLGRKMTPKNQGLDPQAKAKLDKDGNYPIEVWCPDPSKNEN	240
BAV01218.1	DYQTEYPKTTNGGPITIETVLGRKMTPKNQGLDPQAKAKLDKDGNYPIEVWCPDPSKNEN	240

```
BAV01220.1          DYQTEYPKTTNGGPITIETVLGRKMTPKNQGLDPQAKAKLDKDGNYPIEVWCPDPSKNEN   240
BAV01214.1          DYQTEYPKTTNGGPITIETVLGRKMTPKNQGLDPQAKAKLDKDGNYPIEVWCPDPSKNEN   240
BAV01216.1          DYQTEYPKTTNGGPITIETVLGRKMTPKNQGLDPQAKAKLDKDGNYPIEVWCPDPSKNEN   240
BBJ26577.1          DYQTEYPKTTNGGPITIETVLGRKMTPKNQGLDPQAKAKLDKDGNYPIEVWCPDPSKNEN   240
WKF20931.1          DYQTEYPKTTNGGPITIETVLGRKMTPKNQGLDPQAKAKLDKDGNYPIEVWCPDPSKNEN   240
BBJ26589.1          DYQTEYPKTTNGGPITIETVLGRKMTPKNQGLDPQAKAKLDKDGNYPIEVWCPDPSKNEN   240
BBJ26592.1          DYQTEYPKTTNGGPITIETVLGRKMTPKNQGLDPQAKAKLDKDGNYPIEVWCPDPSKNEN   240
BBJ26601.1          DYQTEYPKTTNGGPITIETVLGRKMTPKNQGLDPQAKAKLDKDGNYPIEVWCPDPSKNEN   240
BAV01209.1          DYQTEYPKTTNGGPITIETVLGRKMTPKNQGLDPQAKAKLDKDGNYPIEVWCPDPSKNEN   240
BAV01211.1          DYQTEYPKTTNGGPITIETVLGRKMTPKNQGLDPQAKAKLDKDGNYPIEVWCPDPSKNEN   240
BAV01212.1          DYQTEYPKTTNGGPITIETVLGRKMTPKNQGLDPQAKAKLDKDGNYPIEVWCPDPSKNEN   240
BAV01213.1          DYQTEYPKTTNGGPITIETVLGRKMTPKNQGLDPQAKAKLDKDGNYPIEVWCPDPSKNEN   240
BAV01215.1          DYQTEYPKTTNGGPITIETVLGRKMTPKNQGLDPQAKAKLDKDGNYPIEVWCPDPSKNEN   240
BAV01217.1          DYQTEYPKTTNGGPITIETVLGRKMTPKNQGLDPQAKAKLDKDGNYPIEVWCPDPSKNEN   240
BAV01219.1          DYQTEYPKTTNGGPITIETVLGRKMTPKNQGLDPQAKAKLDKDGNYPIEVWCPDPSKNEN   240
BAV01221.1          DYQTEYPKTTNGGPITIETVLGRKMTPKNQGLDPQAKAKLDKDGNYPIEVWCPDPSKNEN   240
BAV01225.1          DYQTEYPKTTNGGPITIETVLGRKMTPKNQGLDPQAKAKLDKDGNYPIEVWCPDPSKNEN   240
BAV01226.1          DYQTEYPKTTNGGPITIETVLGRKMTPKNQGLDPQAKAKLDKDGNYPIEVWCPDPSKNEN   240
BAV01227.1          DYQTEYPKTTNGGPITIETVLGRKMTPKNQGLDPQAKAKLDKDGNYPIEVWCPDPSKNEN   240
BAV01228.1          DYQTEYPKTTNGGPITIETVLGRKMTPKNQGLDPQAKAKLDKDGNYPIEVWCPDPSKNEN   240
BAN78690.1          DYQTEYPKTTNGGPITIETVLGRKMTPKNQGLDPQAKAKLDKDGNYPIEVWCPDPSKNEN   240
BAN78691.1          DYQTEYPKTTNGGPITIETVLGRKMTPKNQGLDPQAKAKLDKDGNYPIEVWCPDPSKNEN   240
BAN78696.1          DYQTEYPKTTNGGPITIETVLGRKMTPKNQGLDPQAKAKLDKDGNYPIEVWCPDPSKNEN   240
BAN78697.1          DYQTEYPKTTNGGPITIETVLGRKMTPKNQGLDPQAKAKLDKDGNYPIEVWCPDPSKNEN   240
BAN78698.1          DYQTEYPKTTNGGPITIETVLGRKMTPKNQGLDPQAKAKLDKDGNYPIEVWCPDPSKNEN   240
ADE45383.1          DYQTEYPKTTNGGPITIETVLGRKMTPKNQGLDPQAKAKLDKDGNYPIEVWCPDPSKNEN   240
ACL31697.1          DYQTEYPKTTNGGPITIETVLGRKMTPKNQGLDPQAKAKLDKDGNYPIEVWCPDPSKNEN   240
QBR98152.1          DYQTEYPKTTNGGPITIETVLGRKMTPKNQGLDPQAKAKLDKDGNYPIEVWCPDPSKNEN   240
BBJ26574.1          DYQTEYPKTTNGGPITIETVLGRKMTPKNQGLDPQAKAKLDKDGNYPIEVWCPDPSKNEN   240
BBJ26580.1          DYQTEYPKTTNGGPITIETVLGRKMTPKNQGLDPQAKAKLDKDGNYPIEVWCPDPSKNEN   240
BBJ26595.1          DYQTEYPKTTNGGPITIETVLGRKMTPKNQGLDPQAKAKLDKDGNYPIEVWCPDPSKNEN   240
BBJ26598.1          DYQTEYPKTTNGGPITIETVLGRKMTPKNQGLDPQAKAKLDKDGNYPIEVWCPDPSKNEN   240
BAV01210.1          DYQTEYPKTTNGGPITIETVLGRKMTPKNQGLDPQAKAKLDKDGNYPIEVWCPDPSKNEN   240
BAV01222.1          DYQTEYPKTTNGGPITIETVLGRKMTPKNQGLDPQAKAKLDKDGNYPIEVWCPDPSKNEN   240
BAV01223.1          DYQTEYPKTTNGGPITIETVLGRKMTPKNQGLDPQAKAKLDKDGNYPIEVWCPDPSKNEN   240
BAV01224.1          DYQTEYPKTTNGGPITIETVLGRKMTPKNQGLDPQAKAKLDKDGNYPIEVWCPDPSKNEN   240
BAV01230.1          DYQTEYPKTTNGGPITIETVLGRKMTPKNQGLDPQAKAKLDKDGNYPIEVWCPDPSKNEN   240
BAN78692.1          DYQTEYPKTTNGGPITIETVLGRKMTPKNQGLDPQAKAKLDKDGNYPIEVWCPDPSKNEN   240
BAN78693.1          DYQTEYPKTTNGGPITIETVLGRKMTPKNQGLDPQAKAKLDKDGNYPIEVWCPDPSKNEN   240
BAN78694.1          DYQTEYPKTTNGGPITIETVLGRKMTPKNQGLDPQAKAKLDKDGNYPIEVWCPDPSKNEN   240
                    .***:**:***********:***************************:*****.******

ABY65888.1          SRYYGSIQTGSQTPTVLQFSNTLTTVLLDENGVGPLCKGDGLFISCAHIVGFLFKTSGKM   300
QXF78584.1          SRYYGSIQTGSQTPTVLQFSNTLTTVLLDENGVGPLCKGDGLFISCADIVGFLFKTSGKM   300
QXF78586.1          SRYYGSIQTGSQTPTVLQFSNTLTTVLLDENGVGPLCKGDGLFISCADIVGFLFKTSGKM   300
ANF28692.1          SRYYGSIQTGSQTPTVLQFSNTLTTVLLDENGVGPLCKGDGLFISCADIVGFLFKTSGKM   300
AHW79948.1          SRYYGSIQTGSQTPTVLQFSNTLTTVLLDENGVGPLCKGDGLFISCADIVGFLFKTSGKM   300
AWG42111.1          SRYYGSIQTGSQTPTVLQFSNTLTTVLLDENGVGPLCKGDGLFISCADIVGFLFKTSGKM   300
AWG42117.1          SRYYGSIQTGSQTPTVLQFSNTLTTVLLDENGVGPLCKGDGLFISCADIVGFLFKTSGKM   300
ANF28693.1          SRYYGSIQTGSQTPTVLQFSNTLTTVLLDENGVGPLCKGDGLFISCADIVGFLFKTSGKM   300
ANF28694.1          SRYYGSIQTGSQTPTVLQFSNTLTTVLLDENGVGPLCKGDGLFISCADIVGFLFKTSGKM   300
ANF28695.1          SRYYGSIQTGSQTPTVLQFSNTLTTVLLDENGVGPLCKGDGLFISCAHIVGFLFKTSGKM   300
ANF28698.1          SRYYGSIQTGSQTPTVLQFSNTLTTVLLDENGVGPLCKGDGLFISCAHIVGFLFKTSGKM   300
CAR95366.1          SRYYGSIQTGSQTPTVLQFSNTLTTVLLDENGVGPLCKGDGLFISCADIVGFLFKTSGKM   300
AFM84435.1          SRYYGSIQTGSQTPTVLQFSNTLTTVLLDENGVGPLCKGDGLFISCADIVGFLFKTSGKM   300
AFM84450.1          SRYYGSIQTGSQTPTVLQFSNTLTTVLLDENGVGPLCKGDGLFISCADIVGFLFKTSGKM   300
AFM84455.1          SRYYGSIQTGSQTPTVLQFSNTLTTVLLDENGVGPLCKGDGLFISCADIVGFLFKTSGKM   300
ANF28697.1          SRYYGSIQTGSQTPTVLQFSNTLTTVLLDENGVGPLCKGDGLFISCADIVGFLFKTSGKK   300
ACN44192.1          SRYYGSIQTGSQTPTVLQFSNTLTTVLLDENGVGPLCKGDGLFISCADIVGFLFKTSGKM   299
ADE45355.1          SRYYGSIQTGSQTPTVLQFSNTLTTVLLDENGVGPLCKGDGLFISCADIVGFLFKTSGKM   300
ADE45359.1          SRYYGSIQTGSQTPTVLQFSNTLTTVLLDENGVGPLCKGDGLFISCADIVGFLFKTSGKM   300
ADE45351.1          SRYYGSIQTGSQTPTVLQFSNTLTTVLLDENGVGPLCKGDGLFISCADIVGFLFKTSGKM   300
ADE45347.1          SRYYGSIQTGSQTPIVLQFSNTLTTVLLDENGVGPLCKGDGLFISCADIVGFLFKTSGKM   300
AEM01087.1          SRYYGSIQTGSQTPTVLQFSNTLTTVLLDENGVGPLCKGDGLFISCADIVGFLFKTSGKM   300
AFC36083.1          SRYYGSIQTGSQTPTVLQFSNTLTTVLLDENGVGPLCKGDGLFISCADIVGFLFKTSGKM   300
AFM84460.1          SRYYGSIQTGSQTPTVLQFSNTLTTVLLDENGVGPLCKGDGLFISCADIVGFLFKTSGKM   300
AHW79944.1          SRYYGSIQTGSQTPTVLQFSNTLTTVLLDENGVGPLCKGDGLFISCADIVGFLFKTSGKM   300
```

```
BBJ26583.1          SRYYGSIQTGSQTPTVLQFSNTLTTVLLDENGVGPLCKGDGLFISCADIVGFLFKTSGKM   300
BBJ26586.1          SRYYGSIQTGSQTPTVLQFSNTLTTVLLDENGVGPLCKGDGLFISCADIVGFLFKTSGKM   300
APA21079.1          SRYYGSIQTGSQTPTVLQFSNTLTTVLLDENGVGPLCKGDGLFISCADIVGFLFKTSGKM   300
ADN95996.1          SRYYGSIQTGSQTPTVLQFSNTLTTVLLDENGVGPLCKGDGLFISCADIVGFLFKTSGKM   300
YP_009111420.1      SRYYGSIQTGSQTPTVLQFSNTLTTVLLDENGVGPLCKGDGLFISCADIVGFLFKTSGKM   300
UQK62677.1          SRYYGSIQTGSQTPTVLQFSNTLTTVLLDENGVGPLCKGDGLFISCADIVGFLFKTSGKM   300
AWG42121.1          SRYYGSIQTGSQTPTVLQFSNTLTTVLLDENGVGPLCKGDGLFISCADIVGFLFKTSGKM   300
AWG42124.1          SRYYGSIQTGSQTPTVLQFSNTLTTVLLDENGVGPLCKGDGLFISCADIVGFLFKTSGKM   300
AWG42134.1          SRYYGSIQTGSQTPTVLQFSNTLTTVLLDENGVGPLCKGDGLFISCADIVGFLFKTSGKM   300
AWG42140.1          SRYYGSIQTGSQTPTVLQFSNTLTTVLLDENGVGPLCKGDGLFISCADIVGFLFKTSGKM   300
AQM73644.1          SRYYGSIQTGSQTPTVLQFSNTLTTVLLDENGVGPLCKGDGLFISCADIVGFLFKTSGKM   300
BAV01229.1          SRYYGSIQTGSQTPTVLQFSNTLTTVLLDENGVGPLCKGDGLFISCADIVGFLFKTSGKM   300
AKK25202.1          SRYYGSIQTGSQTPTVLQFSNTLTTVLLDENGVGPLCKGDGLFISCADIVGFLFKTSGKM   300
AHN49701.1          SRYYGSIQTGSQTPTVLQFSNTLTTVLLDENGVGPLCKGDGLFISCADIVGFLFKTSGKM   300
AHN49705.1          SRYYGSIQTGSQTPTVLQFSNTLTTVLLDENGVGPLCKGDGLFISCADIVGFLFKTSGKM   300
AHN49709.1          SRYYGSIQTGSQTPTVLQFSNTLTTVLLDENGVGPLCKGDGLFISCADIVGFLFKTSGKM   300
AHW79940.1          SRYYGSIQTGSQTPTVLQFSNTLTTVLLDENGVGPLCKGDGLFISCADIVGFLFKTSGKM   300
AHN13648.1          SRYYGSIQTGSQTPTVLQFSNTLTTVLLDENGVGPLCKGDGLFISCADIVGFLFKTSGKM   300
BAN78699.1          SRYYGSIQTGSQTPTVLQFSNTLTTVLLDENGVGPLCKGDGLFISCADIVGFLFKTSGKM   300
AGA03918.1          SRYYGSIQTGSQTPTVLQFSNTLTTVLLDENGVGPLCKGDGLFISCADIVGFLFKTSGKM   300
AFM84440.1          SRYYGSIQTGSQTPTVLQFSNTLTTVLLDENGVGPLCKGDGLFISCADIVGFLFKTSGKM   300
AFM84445.1          SRYYGSIQTGSQTPTVLQFSNTLTTVLLDENGVGPLCKGDGLFISCADIVGFLFKTSGKM   300
AFC36077.1          SRYYGSIQTGSQTPTVLQFSNTLTTVLLDENGVGPLCKGDGLFISCADIVGFLFKTSGKM   300
AFC36088.1          SRYYGSIQTGSQTPTVLQFSNTLTTVLLDENGVGPLCKGDGLFISCADIVGFLFKTSGKM   300
AFC36093.1          SRYYGSIQTGSQTPTVLQFSNTLTTVLLDENGVGPLCKGDGLFISCADIVGFLFKTSGKM   300
AEM01095.1          SRYYGSIQTGSQTPTVLQFSNTLTTVLLDENGVGPLCKGDGLFISCADIVGFLFKTSGKM   300
AEM01098.1          SRYYGSIQTGSQTPTVLQFSNTLTTVLLDENGVGPLCKGDGLFISCADIVGFLFKTSGKM   300
ADE45363.1          SRYYGSIQTGSQTPTVLQFSNTLTTVLLDENGVGPLCKGDGLFISCADIVGFLFKTSGKM   300
ADE45375.1          SRYYGSIQTGSQTPTVLQFSNTLTTVLLDENGVGPLCKGDGLFISCADIVGFLFKTSGKM   300
ADE45379.1          SRYYGSIQTGSQTPTVLQFSNTLTTVLLDENGVGPLCKGDGLFISCADIVGFLFKTSGKM   300
ADE45387.1          SRYYGSIQTGSQTPTVLQFSNTLTTVLLDENGVGPLCKGDGLFISCADIVGFLFKTSGKM   300
ADE45391.1          SRYYGSIQTGSQTPTVLQFSNTLTTVLLDENGVGPLCKGDGLFISCADIVGFLFKTSGKM   300
ADE45395.1          SRYYGSIQTGSQTPTVLQFSNTLTTVLLDENGVGPLCKGDGLFISCADIVGFLFKTSGKM   300
ADE45403.1          SRYYGSIQTGSQTPTVLQFSNTLTTVLLDENGVGPLCKGDGLFISCADIVGFLFKTSGKM   300
ADE45407.1          SRYYGSIQTGSQTPTVLQFSNTLTTVLLDENGVGPLCKGDGLFISCADIVGFLFKTSGKM   300
ADE45411.1          SRYYGSIQTGSQTPTVLQFSNTLTTVLLDENGVGPLCKGDGLFISCADIVGFLFKTSGKM   300
ADE45415.1          SRYYGSIQTGSQTPTVLQFSNTLTTVLLDENGVGPLCKGDGLFISCADIVGFLFKTSGKM   300
ADE45419.1          SRYYGSIQTGSQTPTVLQFSNTLTTVLLDENGVGPLCKGDGLFISCADIVGFLFKTSGKM   300
CAR95364.1          SRYYGSIQTGSQTPTVLQFSNTLTTVLLDENGVGPLCKGDGLFISCADIVGFLFKTSGKM   300
CAR95367.1          SRYYGSIQTGSQTPTVLQFSNTLTTVLLDENGVGPLCKGDGLFISCADIVGFLFKTSGKM   300
CAR95368.1          SRYYGSIQTGSQTPTVLQFSNTLTTVLLDENGVGPLCKGDGLFISCADIVGFLFKTSGKM   300
CAR95369.1          SRYYGSIQTGSQTPTVLQFSNTLTTVLLDENGVGPLCKGDGLFISCADIVGFLFKTSGKM   300
ACI25319.1          SRYYGSIQTGSQTPTVLQFSNTLTTVLLDENGVGPLCKGDGLFISCADIVGFLFKTSGKM   300
UQK62672.1          SRYYGSIQTGSQTPTVLQFSNTLTTVLLDENGVGPLCKGDGLFISCADIVGFLFKTSGKM   300
UQK62681.1          SRYYGSIQTGSQTPTVLQFSNTLTTVLLDENGVGPLCKGDGLFISCADIVGFLFKTSGKM   300
UQK62685.1          SRYYGSIQTGSQTPTVLQFSNTLTTVLLDENGVGPLCKGDGLFISCADIVGFLFKTSGKM   300
UQK62690.1          SRYYGSIQTGSQTPTVLQFSNTLTTVLLDENGVGPLCKGDGLFISCADIVGFLFKTSGKM   300
UQK62693.1          SRYYGSIQTGSQTPTVLQFSNTLTTVLLDENGVGPLCKGDGLFISCADIVGFLFKTSGKM   300
ANF28696.1          SRYYGSIQTGSQTPTVLQFSNTLTTVLLDENGVGPLCKGDGLFISCADIVGFLFKTSGKM   300
ANF28699.1          SRYYGSIQTGSQTPTVLQFSNTLTTVLLDENGVGPLCKGDGLFISCADIVGFLFKTSGKM   300
AEM01083.1          SRYYGSIQTGSQTPTVLQFSNTLTTVLLDENGVGPLCKGDGLFISCADIVGFLFKTSGKM   300
ADE45343.1          SRYYGSIQTGSQTPTVLQFSNTLTTVLLDENGVGPLCKGDGLFISCADIVGFLFKTSGKM   300
ADE45367.1          SRYYGSIQTGSQTPTVLQFSNTLTTVLLDENGVGPLCKGDGLFISCADIVGFLFKTSGKM   300
ADE45371.1          SRYYGSIQTGSQTPTVLQFSNTLTTVLLDENGVGPLCKGDGLFISCADIVGFLFKTSGKM   300
ADE45399.1          SRYYGSIQTGSQTPTVLQFSNTLTTVLLDENGVGPLCKGDGLFISCADIVGFLFKTSGKM   300
CAR95365.1          SRYYGSIQTGSQTPTVLQFSNTLTTVLLDENGVGPLCKGDGLFISCADIVGFLFKTSGKM   300
ABY65893.1          SRYYGSIQTGSQTPTVLQFSNTLTTVLLDENGVGPLCKGDGLFISCADIVGFLFKTSGKM   300
BAN78700.1          SRYYGSIQTGSQTPTVLQFSNTLTTVLLDENGVGPLCKGDGLFISCADIVGFLFKTSGKM   300
AHB32980.1          SRYYGSIQTGSQTPTVLQFSNTLTTVLLDENGVGPLCKGDGLFISCADIVGFLFKTSGKM   300
BAV01218.1          SRYYGSIQTGSQTPTVLQFSNTLTTVLLDENGVGPLCKGDGLFISCADIVGFLFKTSGKM   300
BAV01220.1          SRYYGSIQTGSQTPTVLQFSNTLTTVLLDENGVGPLCKGDGLFISCADIVGFLFKTSGKM   300
BAV01214.1          SRYYGSIQTGSQTPTVLQFSNTLTTVLLDENGVGPLCKGDGLFISCADIVGFLFKTSGKM   300
BAV01216.1          SRYYGSIQTGSQTPTVLQFSNTLTTVLLDENGVGPLCKGDGLFISCADIVGFLFKTSGKM   300
BBJ26577.1          SRYYGSIQTGSQTPTVLQFSNTLTTVLLDENGVGPLCKGDGLFISCAHIVGFLFKTSGKM   300
WKF20931.1          SRYYGSIQTGSQTPTVLQFSNTLTTVLLDENGVGPLCKGDGLFISCADIVGFLFKTSGKM   300
BBJ26589.1          SRYYGSIQTGSQTPTVLQFSNTLTTVLLDENGVGPLCKGDGLFISCADIVGFLFKTSGKM   300
BBJ26592.1          SRYYGSIQTGSQTPTVLQFSNTLTTVLLDENGVGPLCKGDGLFISCADIVGFLFKTSGKM   300
```

```
BBJ26601.1          SRYYGSIQTGSQTPTVLQFSNTLTTVLLDENGVGPLCKGDGLFISCADIVGFLFKTSGKM  300
BAV01209.1          SRYYGSIQTGSQTPTVLQFSNTLTTVLLDENGVGPLCKGDGLFISCADIVGFLFKTSGKM  300
BAV01211.1          SRYYGSIQTGSQTPTVLQFSNTLTTVLLDENGVGPLCKGDGLFISCADIVGFLFKTSGKM  300
BAV01212.1          SRYYGSIQTGSQTPTVLQFSNTLTTVLLDENGVGPLCKGDGLFISCADIVGFLFKTSGKM  300
BAV01213.1          SRYYGSIQTGSQTPTVLQFSNTLTTVLLDENGVGPLCKGDGLFISCADIVGFLFKTSGKM  300
BAV01215.1          SRYYGSIQTGSQTPTVLQFSNTLTTVLLDENGVGPLCKGDGLFISCADIVGFLFKTSGKM  300
BAV01217.1          SRYYGSIQTGSQTPTVLQFSNTLTTVLLDENGVGPLCKGDGLFISCADIVGFLFKTSGKM  300
BAV01219.1          SRYYGSIQTGSQTPTVLQFSNTLTTVLLDENGVGPLCKGDGLFISCADIVGFLFKTSGKM  300
BAV01221.1          SRYYGSIQTGSQTPTVLQFSNTLTTVLLDENGVGPLCKGDGLFISCADIVGFLFKTSGKM  300
BAV01225.1          SRYYGSIQTGSQTPTVLQFSNTLTTVLLDENGVGPLCKGDGLFISCADIVGFLFKTSGKM  300
BAV01226.1          SRYYGSIQTGSQTPTVLQFSNTLTTVLLDENGVGPLCKGDGLFISCADIVGFLFKTSGKM  300
BAV01227.1          SRYYGSIQTGSQTPTVLQFSNTLTTVLLDENGVGPLCKGDGLFISCADIVGFLFKTSGKM  300
BAV01228.1          SRYYGSIQTGSQTPTVLQFSNTLTTVLLDENGVGPLCKGDGLFISCADIVGFLFKTSGKM  300
BAN78690.1          SRYYGSIQTGSQTPTVLQFSNTLTTVLLDENGVGPLCKGDGLFISCADIVGFLFKTSGKM  300
BAN78691.1          SRYYGSIQTGSQTPTVLQFSNTLTTVLLDENGVGPLCKGDGLFISCADIVGFLFKTSGKM  300
BAN78696.1          SRYYGSIQTGSQTPTVLQFSNTLTTVLLDENGVGPLCKGDGLFISCADIVGFLFKTSGKM  300
BAN78697.1          SRYYGSIQTGSQTPTVLQFSNTLTTVLLDENGVGPLCKGDGLFISCADIVGFLFKTSGKM  300
BAN78698.1          SRYYGSIQTGSQTPTVLQFSNTLTTVLLDENGVGPLCKGDGLFISCADIVGFLFKTSGKM  300
ADE45383.1          SRYYGSIQTGSQTPTVLQFSNTLTTVLLDENGVGPLCKGDGLFISCADIVGFLFKTSGKM  300
ACL31697.1          SRYYGSIQTGSQTPTVLQFSNTLTTVLLDENGVGPLCKGDGLFISCADIVGFLFKTSGKM  300
QBR98152.1          SRYYGSIQTGSQTPTVLQFSNTLTTVLLDENGVGPLCKGDGLFISCADIVGFLFKTSGKM  300
BBJ26574.1          SRYYGSIQTGSQTPTVLQFSNTLTTVLLDENGVGPLCKGDGLFISCADIVGFLFKTSGKM  300
BBJ26580.1          SRYYGSIQTGSQTPTVLQFSNTLTTVLLDENGVGPLCKGDGLFISCADIVGFLFKTSGKM  300
BBJ26595.1          SRYYGSIQTGSQTPTVLQFSNTLTTVLLDENGVGPLCKGDGLFISCADIVGFLFKTSGKM  300
BBJ26598.1          SRYYGSIQTGSQTPTVLQFSNTLTTVLLDENGVGPLCKGDGLFISCADIVGFLFKTSGKM  300
BAV01210.1          SRYYGSIQTGSQTPTVLQFSNTLTTVLLDENGVGPLCKGDGLFISCADIVGFLFKTSGKM  300
BAV01222.1          SRYYGSIQTGSQTPTVLQFSNTLTTVLLDENGVGPLCKGDGLFISCADIVGFLFKTSGKM  300
BAV01223.1          SRYYGSIQTGSQTPTVLQFSNTLTTVLLDENGVGPLCKGDGLFISCADIVGFLFKTSGKM  300
BAV01224.1          SRYYGSIQTGSQTPTVLQFSNTLTTVLLDENGVGPLCKGDGLFISCADIVGFLFKTSGKM  300
BAV01230.1          SRYYGSIQTGSQTPTVLQFSNTLTTVLLDENGVGPLCKGDGLFISCADIVGFLFKTSGKM  300
BAN78692.1          SRYYGSIQTGSQTPTVLQFSNTLTTVLLDENGVGPLCKGDGLFISCADIVGFLFKTSGKM  300
BAN78693.1          SRYYGSIQTGSQTPTVLQFSNTLTTVLLDENGVGPLCKGDGLFISCADIVGFLFKTSGKM  300
BAN78694.1          SRYYGSIQTGSQTPTVLQFSNTLTTVLLDENGVGPLCKGDGLFISCADIVGFLFKTSGKM  300
                    ************** ********************************.***********

ABY65888.1          ALHGLPRYFNVTLRKIWVKNPYPVVNLINSLFSNLMPKVSGQPMEGKDNQVEEVRIYEGS  360
QXF78584.1          ALHGLPRYFNVTLRKRWVKNPYPVVNLINSLFSNLMPKVSGQPMEGKDNQVEEVRIYEGS  360
QXF78586.1          ALHGLPRYFNVTLRKRWVKNPYPVVNLINSLFSNLMPKVSGQPMEGKDNQVEEVRIYEGS  360
ANF28692.1          ALHGLPRYFNVTLRKRWVKNPYPVVNLINSLFSNLMPKVSGQPMEGKDNQVEEVRIYEGS  360
AHW79948.1          ALHGLPRYFNVTLRKRWVKNPYPVVNLINSLFSNLMPKVSGQPMEGKDNQVEEVRIYEGS  360
AWG42111.1          ALHGLPRYFNVTLRKRWVKNPYPVVNLINSLFSNLMPKVSGQPMEGKDNQVEEVRIYEGS  360
AWG42117.1          ALHGLPRYFNVTLRKRWVKNPYPVVNLINSLFSNLMPKVSGQPMEGKDNQVEEVRIYEGS  360
ANF28693.1          ALHGLPRYFNVTLRKRWVKNPYPVVNLINSLFSNLMPKVSGQPMEGKDNQVEEVRIYEGS  360
ANF28694.1          ALHGLPRYFNVTLRKRWVKNPYPVVNLINSLFSNLMPKVSGQPMEGKDNQVEEVRIYEGS  360
ANF28695.1          ALHGLPRYFNVTLRKRWVKNPYPVVNLINSLFSNLMPKVSGQPMEGKDNQVEEVRIYEGS  360
ANF28698.1          ALHGLPRYFNVTLRKRWVKNPYPVVNLINSLFSNLMPKVSGQPMEGKDNQVEEVRIYEGS  360
CAR95366.1          ALHGLPRYFNVTLRKRWVKNPYPVVNLINSLFSNLMPKVSGQPMEGKDNQVEEVRIYEGS  360
AFM84435.1          ALHGLPRYFNVTLRKRWVKNPYPVVNLINSLFSNLMPKVSGQPMEGKDNQVEEVRIYEGS  360
AFM84450.1          ALHGLPRYFNVTLRKRWVKNPYPVVNLINSLFSNLMPKVSGQPMEGKDNQVEEVRIYEGS  360
AFM84455.1          ALHGLPRYFNVTLRKRWVKNPYPVVNLINSLFSNLMPKVSGQPMEGKDNQVEEVRIYEGS  360
ANF28697.1          ALHGLPRYFNVTLRKRWVKNPYPVVNLINSLFSNLMPKVSGQPMEGKDNQVEEVRIYEGS  360
ACN44192.1          ALHGLPRYFNVTLRKRWVKNPYPVVNLINSLFSNLMPKVSGQPMEGKDNQVEEVRIYEGS  359
ADE45355.1          ALHGLPRYFNVTLRKRWVKNPYPVVNLINSLFSNLMPKVSGQPMEGKDNQVEEVRIYEGS  360
ADE45359.1          ALHGLPRYFNVTLRKRWVKNPYPVVNLINSLFSNLMPKVSGQPMEGKDNQVEEVRIYEGS  360
ADE45351.1          ALHGLPRYFNVTLRKRWVKNPYPVVNLINSLFSNLMPKVSGQPMEGKDNQVEEVRIYEGS  360
ADE45347.1          ALHGLPRYFNVTLRKRWVKNPYPVVNLINSLFSNLMPKVSGQPMEGKDNQVEEVRIYEGS  360
AEM01087.1          ALHGLPRYFNVTLRKRWVKNPYPVVNLINSLFSNLMPKVSGQPMEGKDNQVEEVRIYEGS  360
AFC36083.1          ALHGLPRYFNVTLRKRWVKNPYPVVNLINSLFSNLMPKVSGQPMEGKDNQVEEVRIYEGS  360
AFM84460.1          ALHGLPRYFNVTLRKRWVKNPYPVVNLINSLFSNLMPKVSGQPMEGKDNQVEEVRIYEGS  360
AHW79944.1          ALHGLPRYFNVTLRKRWVKNPYPVVNLINSLFSNLMPKVSGQPMEGKDNQVEEVRIYEGS  360
BBJ26583.1          ALHGLPRYFNVTLRKRWVKNPYPVVNLINSLFSNLMPKVSGQPMEGKDNQVEEVRIYEGS  360
BBJ26586.1          ALHGLPRYFNVTLRKRWVKNPYPVVNLINSLFSNLMPKVSGQPMEGKDNQVEEVRIYEGS  360
APA21079.1          ALHGLPRYFNVTLRKRWVKNPYPVVNLINSLFSNLMPKVSGQPMEGKDNQVEEVRIYEGS  360
ADN95996.1          ALHGLPRYFNVTLRKRWVKNPYPVVNLINSLFSNLMPKVSGQPMEGKDNQVEEVRIYEGS  360
YP_009111420.1      ALHGLPRYFNVTLRKRWVKNPYPVVNLINSLFSNLMPKVSGQPMEGKDNQVEEVRIYEGS  360
UQK62677.1          ALHGLPRYFNVTLRKRWVKNPYPVVNLINSLFSNLMPKVSGQPMEGKDNQVEEVRIYEGS  360
AWG42121.1          ALHGLPRYFNVTLRKRWVKNPYPVVNLINSLFSNLMPKVSGQPMEGKDNQVEEVRIYEGS  360
```

AWG42124.1	ALHGLPRYFNVTLRKRWVKNPYPVVNLINSLFSNLMPKVSGQPMEGKDNQVEEVRIYEGS	360
AWG42134.1	ALHGLPRYFNVTLRKRWVKNPYPVVNLINSLFSNLMPKVSGQPMEGKDNQVEEVRIYEGS	360
AWG42140.1	ALHGLPRYFNVTLRKRWVKNPYPVVNLINSLFSNLMPKVSGQPMEGKDNQVEEVRIYEGS	360
AQM73644.1	ALHGLPRYFNVTLRKRWVKNPYPVVNLINSLFSNLMPKVSGQPMEGKDNQVEEVRIYEGS	360
BAV01229.1	ALHGLPRYFNVTLRKRWVKNPYPVVNLINSLFSNLMPKVSGQPMEGKDNQVEEVRIYEGS	360
AKK25202.1	ALHGLPRYFNVTLRKRWVKNPYPVVNLINSLFSNLMPKVSGQPMEGKDNQVEEVRIYEGS	360
AHN49701.1	ALHGLPRYFNVTLRKRWVKNPYPVVNLINSLFSNLMPKVSGQPMEGKDNQVEEVRIYEGS	360
AHN49705.1	ALHGLPRYFNVTLRKRWVKNPYPVVNLINSLFSNLMPKVSGQPMEGKDNQVEEVRIYEGS	360
AHN49709.1	ALHGLPRYFNVTLRKRWVKNPYPVVNLINSLFSNLMPKVSGQPMEGKDNQVEEVRIYEGS	360
AHW79940.1	ALHGLPRYFNVTLRKRWVKNPYPVVNLINSLFSNLMPKVSGQPMEGKDNQVEEVRIYEGS	360
AHN13648.1	ALHGLPRYFNVTLRKRWVKNPYPVVNLINSLFSNLMPKVSGQPMEGKDNQVEEVRIYEGS	360
BAN78699.1	ALHGLPRYFNVTLRKRWVKNPYPVVNLINSLFSNLMPKVSGQPMEGKDNQVEEVRIYEGS	360
AGA03918.1	ALHGLPRYFNVTLRKRWVKNPYPVVNLINSLFSNLMPKVSGQPMEGKDNQVEEVRIYEGS	360
AFM84440.1	ALHGLPRYFNVTLRKRWVKNPYPVVNLINSLFSNLMPKVSGQPMEGKDNQVEEVRIYEGS	360
AFM84445.1	ALHGLPRYFNVTLRKRWVKNPYPVVNLINSLFSNLMPKVSGQPMEGKDNQVEEVRIYEGS	360
AFC36077.1	ALHGLPRYFNVTLRKRWVKNPYPVVNLINSLFSNLMPKVSGQPMEGKDNQVEEVRIYEGS	360
AFC36088.1	ALHGLPRYFNVTLRKRWVKNPYPVVNLINSLFSNLMPKVSGQPMEGKDNQVEEVRIYEGS	360
AFC36093.1	ALHGLPRYFNVTLRKRWVKNPYPVVNLINSLFSNLMPKVSGQPMEGKDNQVEEVRIYEGS	360
AEM01095.1	ALHGLPRYFNVTLRKRWVKNPYPVVNLINSLFSNLMPKVSGQPMEGKDNQVEEVRIYEGS	360
AEM01098.1	ALHGLPRYFNVTLRKRWVKNPYPVVNLINSLFSNLMPKVSGQPMEGKDNQVEEVRIYEGS	360
ADE45363.1	ALHGLPRYFNVTLRKRWVKNPYPVVNLINSLFSNLMPKVSGQPMEGKDNQVEEVRIYEGS	360
ADE45375.1	ALHGLPRYFNVTLRKRWVKNPYPVVNLINSLFSNLMPKVSGQPMEGKDNQVEEVRIYEGS	360
ADE45379.1	ALHGLPRYFNVTLRKRWVKNPYPVVNLINSLFSNLMPKVSGQPMEGKDNQVEEVRIYEGS	360
ADE45387.1	ALHGLPRYFNVTLRKRWVKNPYPVVNLINSLFSNLMPKVSGQPMEGKDNQVEEVRIYEGS	360
ADE45391.1	ALHGLPRYFNVTLRKRWVKNPYPVVNLINSLFSNLMPKVSGQPMEGKDNQVEEVRIYEGS	360
ADE45395.1	ALHGLPRYFNVTLRKRWVKNPYPVVNLINSLFSNLMPKVSGQPMEGKDNQVEEVRIYEGS	360
ADE45403.1	ALHGLPRYFNVTLRKRWVKNPYPVVNLINSLFSNLMPKVSGQPMEGKDNQVEEVRIYEGS	360
ADE45407.1	ALHGLPRYFNVTLRKRWVKNPYPVVNLINSLFSNLMPKVSGQPMEGKDNQVEEVRIYEGS	360
ADE45411.1	ALHGLPRYFNVTLRKRWVKNPYPVVNLINSLFSNLMPKVSGQPMEGKDNQVEEVRIYEGS	360
ADE45415.1	ALHGLPRYFNVTLRKRWVKNPYPVVNLINSLFSNLMPKVSGQPMEGKDNQVEEVRIYEGS	360
ADE45419.1	ALHGLPRYFNVTLRKRWVKNPYPVVNLINSLFSNLMPKVSGQPMEGKDNQVEEVRIYEGS	360
CAR95364.1	ALHGLPRYFNVTLRKRWVKNPYPVVNLINSLFSNLMPKVSGQPMEGKDNQVEEVRIYEGS	360
CAR95367.1	ALHGLPRYFNVTLRKRWVKNPYPVVNLINSLFSNLMPKVSGQPMEGKDNQVEEVRIYEGS	360
CAR95368.1	ALHGLPRYFNVTLRKRWVKNPYPVVNLINSLFSNLMPKVSGQPMEGKDNQVEEVRIYEGS	360
CAR95369.1	ALHGLPRYFNVTLRKRWVKNPYPVVNLINSLFSNLMPKVSGQPMEGKDNQVEEVRIYEGS	360
ACI25319.1	ALHGLPRYFNVTLRKRWVKNPYPVVNLINSLFSNLMPKVSGQPMEGKDNQVEEVRIYEGS	360
UQK62672.1	ALHGLPRYFNVTLRKRWVKNPYPVVNLINSLFSNLMPKVSGQPMEGKDNQVEEVRIYEGS	360
UQK62681.1	ALHGLPRYFNVTLRKRWVKNPYPVVNLINSLFSNLMPKVSGQPMEGKDNQVEEVRIYEGS	360
UQK62685.1	ALHGLPRYFNVTLRKRWVKNPYPVVNLINSLFSNLMPKVSGQPMEGKDNQVEEVRIYEGS	360
UQK62690.1	ALHGLPRYFNVTLRKRWVKNPYPVVNLINSLFSNLMPKVSGQPMEGKDNQVEEVRIYEGS	360
UQK62693.1	ALHGLPRYFNVTLRKRWVKNPYPVVNLINSLFSNLMPKVSGQPMEGKDNQVEEVRIYEGS	360
ANF28696.1	ALHGLPRYFNVTLRKRWVKNPYPVVNLINSLFSNLMPKVSGQPMEGKDNQVEEVRIYEGS	360
ANF28699.1	ALHGLPRYFNVTLRKRWVKNPYPVVNLINSLFSNLMPKVSGQPMEGKDNQVEEVRIYEGS	360
AEM01083.1	ALHGLPRYFNVTLRKRWVKNPYPVVNLINSLFSNLMPKVSGQPMEGKDNQVEEVRIYEGS	360
ADE45343.1	ALHGLPRYFNVTLRKRWVKNPYPVVNLINSLFSNLMPKVSGQPMEGKDNQVEEVRIYEGS	360
ADE45367.1	ALHGLPRYFNVTLRKRWVKNPYPVVNLINSLFSNLMPKVSGQPMEGKDNQVEEVRIYEGS	360
ADE45371.1	ALHGLPRYFNVTLRKRWVKNPYPVVNLINSLFSNLMPKVSGQPMEGKDNQVEEVRIYEGS	360
ADE45399.1	ALHGLPRYFNVTLRKRWVKNPYPVVNLINSLFSNLMPKVSGQPMEGKDNQVEEVRIYEGS	360
CAR95365.1	ALHGLPRYFNVTLRKRWVKNPYPVVNLINSLFSNLMPKVSGQPMEGKDNQVEEVRIYEGS	360
ABY65893.1	ALHGLPRYFNVTLRKRWVKNPYPVVNLINSLFSNLMPKVSGQPMEGKDNQVEEVRIYEGS	360
BAN78700.1	ALHGLPRYFNVTLRKRWVKNPYPVVNLINSLFSNLMPKVSGQPMEGEDNQAEEVRIYEGS	360
AHB32980.1	ALHGLPRYFNVTLRKRWVKNPYPVVNLINSLFSNLMPKVSGQPMEGKDNQVEEVRIYEGS	360
BAV01218.1	ALHGLPRYFNVTLRKRWVKNPYPVVNLINSLFSNLMPKVSGQPMEGKDNQVEEVRIYEGS	360
BAV01220.1	ALHGLPRYFNVTLRKRWVKNPYPVVNLINSLFSNLMPKVSGQPMEGKDNQVEEVRIYEGS	360
BAV01214.1	ALHGLPRYFNVTLRKRWVKNPYPVVNLINSLFSNLMPKVSGQPMEGKDNQVEEVRIYEGS	360
BAV01216.1	ALHGLPRYFNVTLRKRWVKNPYPVVNLINSLFSNLMPKVSGQPMEGKDNQVEEVRIYEGS	360
BBJ26577.1	ALHGLPRYFNVTLRKRWVKNPYPVVNLINSLFSNLMPKVSGQPMEGKDNQVEEVRIYEGS	360
WKF20931.1	ALHGLPRYFNVTLRKRWVKNPYPVVNLINSLFSNLMPKVSGQPMEGKDNQVEEVRIYEGS	360
BBJ26589.1	ALHGLPRYFNVTLRKRWVKNPYPVVNLINSLFSNLMPKVSGQPMEGKDNQVEEVRIYEGS	360
BBJ26592.1	ALHGLPRYFNVTLRKRWVKNPYPVVNLINSLFSNLMPKVSGQPMEGKDNQVEEVRIYEGS	360
BBJ26601.1	ALHGLPRYFNVTLRKRWVKNPYPVVNLINSLFSNLMPKVSGQPMEGKDNQVEEVRIYEGS	360
BAV01209.1	ALHGLPRYFNVTLRKRWVKNPYPVVNLINSLFSNLMPKVSGQPMEGKDNQVEEVRIYEGS	360
BAV01211.1	ALHGLPRYFNVTLRKRWVKNPYPVVNLINSLFSNLMPKVSGQPMEGKDNQVEEVRIYEGS	360
BAV01212.1	ALHGLPRYFNVTLRKRWVKNPYPVVNLINSLFSNLMPKVSGQPMEGKDNQVEEVRIYEGS	360
BAV01213.1	ALHGLPRYFNVTLRKRWVKNPYPVVNLINSLFSNLMPKVSGQPMEGKDNQVEEVRIYEGS	360
BAV01215.1	ALHGLPRYFNVTLRKRWVKNPYPVVNLINSLFSNLMPKVSGQPMEGKDNQVEEVRIYEGS	360
BAV01217.1	ALHGLPRYFNVTLRKRWVKNPYPVVNLINSLFSNLMPKVSGQPMEGKDNQVEEVRIYEGS	360

```
BAV01219.1          ALHGLPRYFNVTLRKRWVKNPYPVVNLINSLFSNLMPKVSGQPMEGKDNQVEEVRIYEGS   360
BAV01221.1          ALHGLPRYFNVTLRKRWVKNPYPVVNLINSLFSNLMPKVSGQPMEGKDNQVEEVRIYEGS   360
BAV01225.1          ALHGLPRYFNVTLRKRWVKNPYPVVNLINSLFSNLMPKVSGQPMEGKDNQVEEVRIYEGS   360
BAV01226.1          ALHGLPRYFNVTLRKRWVKNPYPVVNLINSLFSNLMPKVSGQPMEGKDNQVEEVRIYEGS   360
BAV01227.1          ALHGLPRYFNVTLRKRWVKNPYPVVNLINSLFSNLMPKVSGQPMEGKDNQVEEVRIYEGS   360
BAV01228.1          ALHGLPRYFNVTLRKRWVKNPYPVVNLINSLFSNLMPKVSGQPMEGKDNQVEEVRIYEGS   360
BAN78690.1          ALHGLPRYFNVTLRKRWVKNPYPVVNLINSLFSNLMPKVSGQPMEGKDNQVEEVRIYEGS   360
BAN78691.1          ALHGLPRYFNVTLRKRWVKNPYPVVNLINSLFSNLMPKVSGQPMEGKDNQVEEVRIYEGS   360
BAN78696.1          ALHGLPRYFNVTLRKRWVKNPYPVVNLINSLFSNLMPKVSGQPMEGKDNQVEEVRIYEGS   360
BAN78697.1          ALHGLPRYFNVTLRKRWVKNPYPVVNLINSLFSNLMPKVSGQPMEGKDNQVEEVRIYEGS   360
BAN78698.1          ALHGLPRYFNVTLRKRWVKNPYPVVNLINSLFSNLMPKVSGQPMEGKDNQVEEVRIYEGS   360
ADE45383.1          ALHGLPRYFNVTLRKRWVKNPYPVVNLINSLFSNLMPKVSGQPMEGKDNQVEEVRIYEGS   360
ACL31697.1          ALHGLPRYFNVTLRKRWVKNPYPVVNLINSLFSNLMPKVSGQPMEGKDNQVEEVRIYEGS   360
QBR98152.1          ALHGLPRYFNVTLRKRWVKNPYPVVNLINSLFSNLMPKVSGQPMEGKDNQVEEVRIYEGS   360
BBJ26574.1          ALHGLPRYFNVTLRKRWVKNPYPVVNLINSLFSNLMPKVSGQPMEGKDNQVEEVRIYEGS   360
BBJ26580.1          ALHGLPRYFNVTLRKRWVKNPYPVVNLINSLFSNLMPKVSGQPMEGKDNQVEEVRIYEGS   360
BBJ26595.1          ALHGLPRYFNVTLRKRWVKNPYPVVNLINSLFSNLMPKVSGQPMEGKDNQVEEVRIYEGS   360
BBJ26598.1          ALHGLPRYFNVTLRKRWVKNPYPVVNLINSLFSNLMPKVSGQPMEGKDNQVEEVRIYEGS   360
BAV01210.1          ALHGLPRYFNVTLRKRWVKNPYPVVNLINSLFSNLMPKVSGQPMEGKDNQVEEVRIYEGS   360
BAV01222.1          ALHGLPRYFNVTLRKRWVKNPYPVVNLINSLFSNLMPKVSGQPMEGKDNQVEEVRIYEGS   360
BAV01223.1          ALHGLPRYFNVTLRKRWVKNPYPVVNLINSLFSNLMPKVSGQPMEGKDNQVEEVRIYEGS   360
BAV01224.1          ALHGLPRYFNVTLRKRWVKNPYPVVNLINSLFSNLMPKVSGQPMEGKDNQVEEVRIYEGS   360
BAV01230.1          ALHGLPRYFNVTLRKRWVKNPYPVVNLINSLFSNLMPKVSGQPMEGKDNQVEEVRIYEGS   360
BAN78692.1          ALHGLPRYFNVTLRKRWVKNPYPVVNLINSLFSNLMPKVSGQPMEGKDNQVEEVRIYEGS   360
BAN78693.1          ALHGLPRYFNVTLRKRWVKNPYPVVNLINSLFSNLMPKVSGQPMEGKDNQVEEVRIYEGS   360
BAN78694.1          ALHGLPRYFNVTLRKRWVKNPYPVVNLINSLFSNLMPKVSGQPMEGKDNQVEEVRIYEGS   360
                    *************** ******************************:***.*********

ABY65888.1          EQLPGNPDIVRFLDKFGQEKTVYPKPSVAPAAVTFQSNQQDKGKAPLKGPQKASQKESQT   420
QXF78584.1          EQLPGDPDIVRFLDKFGQEKTVYPKPSVAPAAVTFQSNQQDKSKAPLKGPQKASQKESQT   420
QXF78586.1          EQLPGDPDIVRFLDKFGQEKTVYPKPSVAPAAVTFQSNQQDKSKAPLKGPQKASQKESQT   420
ANF28692.1          EQLPGDPDIVRFLDKFGQEKTVYPKPSVAPAAVTFQSNQQDKSKAPLKGPQKASQKESQT   420
AHW79948.1          EQLPGDPDIVRFLDKFGQEKTVYPKPSVAPAAVTFQSNQQDKSKAPLKGPQKASQKESQT   420
AWG42111.1          EQLPGDPDIVRFLDKFGQEKTVYPKPSVAPAAVTFQSNQQDKSKAPLKGPQKASQKENQT   420
AWG42117.1          EQLPGDPDIVRFLDKFGQEKTVYPKPSVAPAAVTFQSNQQDKSKAPLKGPQKASQKENQT   420
ANF28693.1          EQLPGDPDIVRFLDKFGQEKTVYPKPSVAPAAVTFQSNQQDKSKAPLKGPQKASQKERQT   420
ANF28694.1          EQLPGDPDIVRFLDKFGQEKTVYPKPSVAPAAVTFQSNQQDKSKAPLKGPQKASQKERQT   420
ANF28695.1          EQLPGDPDIVRFLDKFGQEKTVYPKPSVAPAAVTFQSNQQDKGKAPLKGPQKASQKENQT   420
ANF28698.1          EQLPGDPDIVRFLDKFGQEKTVYPKPSVAPAAVTFQSNQQDKGKAPLKGPQKASQKESQT   420
CAR95366.1          EPLPGDPDIVRFLDKFGQEKTVYPKPSVAPAAVTFQSNQQDKGKAPLKGPQKASQKESQT   420
AFM84435.1          EQLPGDPDIVRFLDKFGQEKTVYPKPSVAPAAVTFQSNQEDKGKAPLKGPQKASQKESQT   420
AFM84450.1          EQLPGDPDIVRFLDKFGQEKTVYPKPSVAPAAVTFQSNQEDKGKAPLKGPQKASQKESQT   420
AFM84455.1          EQLPGDPDIVRFLDKFGQEKTVYPKPSVAPAAVTFQSNQEDKGKAPLKGPQKASQKESQT   420
ANF28697.1          EQLPGDPDIVRFLDKFGQEKTVYPKPSVAPAAVTFQSNQQDKGKAPLKGPQKASQKESQT   420
ACN44192.1          EQLPGDPDIVRFLDKFGQEKTVYPKPSVAPAAVTFQSNQQDKGKAPLKGPQKASQKESQT   419
ADE45355.1          EQLPGDPDIVRFLDKFGQEKTVYPKPSVAPAAVTFQSNQQDKGKAPLKGPQKASQKESQT   420
ADE45359.1          EQLPGDPDIVRFLDKFGQEKTVYPKPSVAPAAVTFQSNQQDKGKAPLKGPQKASQKESQT   420
ADE45351.1          EQLPGDPDIVRFLDKFGQEKTVYPKPSVAPAAVTFQSNQQDKGKAPLKGPQQASQKESQT   420
ADE45347.1          EQLPGDPDIVRFLDKFGQEKTVYPKPSVAPAAVTFQSNQQDKGKAPLKGPQKASQKESQT   420
AEM01087.1          EQLPGDPDIVRFLDKFGQEKTVYPKPSVAPAAVTFQSNQQDKGKAPLKGPQKASQKESQT   420
AFC36083.1          EQLPGDPDIVRFLDKFGQEKTVYPKPSVAPAAVTFQSNQQDKGKAPLKGPQKASQKESQT   420
AFM84460.1          EQLPGDPDIVRFLDKFGQEKTVYPKPSVAPAAVTFQSNQQDKGKAPLKGPQKASQKESQT   420
AHW79944.1          EQLPGDPDIVRFLDKFGQEKTVYPKPSVAPAAVTFQSNQQDKGKAPLKGPQKASQKESQT   420
BBJ26583.1          EQLPGDPDIVRFLDKFGQEKTVYPKPSVAPAAVTFQSNQQDKGKAPLKGPQKASQKESQT   420
BBJ26586.1          EQLPGDPDIVRFLDKFGQEKTVYPKPSVAPAAVTFQSNQQDKGKAPLKGPQKASQKESQT   420
APA21079.1          EQLPGDPDIVRFLDKFGQEKTVYPKPSVAPAAVTFQSNQQDKGKAPLKGPQKASQKESQT   420
ADN95996.1          EQLPGDPDIVRFLDKFGQEKTVYPKPSVAPAAVTFQSNQQDKGKAPLKGPQKASQKESQT   420
YP_009111420.1      EQLPGDPDIVRFLDKFGQEKTVYPKPSVAPAAVTFQSNQQDKGKAPLKGPQKASQKESQT   420
UQK62677.1          EQLPGDPDIVRFLDKFGQEKTVYPKPSVAPAAVTFQSNQQDKGKAPLKGPQKASQKESQT   420
AWG42121.1          EQLPGDPDIVRFLDKFGQEKTVYPKPSVAPAAVTFQSNQQDKGKAPLKGPQKASQKESQT   420
AWG42124.1          EQLPGDPDIVRFLDKFGQEKTVYPKPSVAPAAVTFQSNQQDKGKAPLKGPQKASQKESQT   420
AWG42134.1          EQLPGDPDIVRFLDKFGQEKTVYPKPSVAPAAVTFQSNQQDKGKAPLKGPQKASQKESQT   420
AWG42140.1          EQLPGDPDIVRFLDKFGQEKTVYPKPSVAPAAVTFQSNQQDKGKAPLKGPQKASQKESQT   420
AQM73644.1          EQLPGDPDIVRFLDKFGQEKTVYPKPSVAPAAVTFQSNQQDKGKAPLKGPQKASQKESQT   420
BAV01229.1          EQLPGDPDIVRFLDKFGQEKTVYPKPSVAPAAVTFQSNQQDKGKAPLKGPQKASQKESQT   420
AKK25202.1          EQLPGDPDIVRFLDKFGQEKTVYPKPSVAPAAVTFQSNQQDKGKAPLKGPQKASQKESQT   420
AHN49701.1          EQLPGDPDIVRFLDKFGQEKTVYPKPSVAPAAVTFQSNQQDKGKAPLKGPQKASQKESQT   420
```

```
AHN49705.1          EQLPGDPDIVRFLDKFGQEKTVYPKPSVAPAAVTFQSNQQDKGKAPLKGPQKASQKESQT   420
AHN49709.1          EQLPGDPDIVRFLDKFGQEKTVYPKPSVAPAAVTFQSNQQDKGKAPLKGPQKASQKESQT   420
AHW79940.1          EQLPGDPDIVRFLDKFGQEKTVYPKPSVAPAAVTFQSNQQDKGKAPLKGPQKASQKESQT   420
AHN13648.1          EQLPGDPDIVRFLDKFGQEKTVYPKPSVAPAAVTFQSNQQDKGKAPLKGPQKASQKESQT   420
BAN78699.1          EQLPGDPDIVRFLDKFGQEKTVYPKPSVAPAAVTFQSNQQDKGKAPLKGPQKASQKESQT   420
AGA03918.1          EQLPGDPDIVRFLDKFGQEKTVYPKPSVAPAAVTFQSNQQDKGKAPLKGPQKASQKESQT   420
AFM84440.1          EQLPGDPDIVRFLDKFGQEKTVYPKPSVAPAAVTFQSNQQDKGKAPLKGPQKASQKESQT   420
AFM84445.1          EQLPGDPDIVRFLDKFGQEKTVYPKPSVAPAAVTFQSNQQDKGKAPLKGPQKASQKESQT   420
AFC36077.1          EQLPGDPDIVRFLDKFGQEKTVYPKPSVAPAAVTFQSNQQDKGKAPLKGPQKASQKESQT   420
AFC36088.1          EQLPGDPDIVRFLDKFGQEKTVYPKPSVAPAAVTFQSNQQDKGKAPLKGPQKASQKESQT   420
AFC36093.1          EQLPGDPDIVRFLDKFGQEKTVYPKPSVAPAAVTFQSNQQDKGKAPLKGPQKASQKESQT   420
AEM01095.1          EQLPGDPDIVRFLDKFGQEKTVYPKPSVAPAAVTFQSNQQDKGKAPLKGPQKASQKESQT   420
AEM01098.1          EQLPGDPDIVRFLDKFGQEKTVYPKPSVAPAAVTFQSNQQDKGKAPLKGPQKASQKESQT   420
ADE45363.1          EQLPGDPDIVRFLDKFGQEKTVYPKPSVAPAAVTFQSNQQDKGKAPLKGPQKASQKESQT   420
ADE45375.1          EQLPGDPDIVRFLDKFGQEKTVYPKPSVAPAAVTFQSNQQDKGKAPLKGPQKASQKESQT   420
ADE45379.1          EQLPGDPDIVRFLDKFGQEKTVYPKPSVAPAAVTFQSNQQDKGKAPLKGPQKASQKESQT   420
ADE45387.1          EQLPGDPDIVRFLDKFGQEKTVYPKPSVAPAAVTFQSNQQDKGKAPLKGPQKASQKESQT   420
ADE45391.1          EQLPGDPDIVRFLDKFGQEKTVYPKPSVAPAAVTFQSNQQDKGKAPLKGPQKASQKESQT   420
ADE45395.1          EQLPGDPDIVRFLDKFGQEKTVYPKPSVAPAAVTFQSNQQDKGKAPLKGPQKASQKESQT   420
ADE45403.1          EQLPGDPDIVRFLDKFGQEKTVYPKPSVAPAAVTFQSNQQDKGKAPLKGPQKASQKESQT   420
ADE45407.1          EQLPGDPDIVRFLDKFGQEKTVYPKPSVAPAAVTFQSNQQDKGKAPLKGPQKASQKESQT   420
ADE45411.1          EQLPGDPDIVRFLDKFGQEKTVYPKPSVAPAAVTFQSNQQDKGKAPLKGPQKASQKESQT   420
ADE45415.1          EQLPGDPDIVRFLDKFGQEKTVYPKPSVAPAAVTFQSNQQDKGKAPLKGPQKASQKESQT   420
ADE45419.1          EQLPGDPDIVRFLDKFGQEKTVYPKPSVAPAAVTFQSNQQDKGKAPLKGPQKASQKESQT   420
CAR95364.1          EQLPGDPDIVRFLDKFGQEKTVYPKPSVAPAAVTFQSNQQDKGKAPLKGPQKASQKESQT   420
CAR95367.1          EQLPGDPDIVRFLDKFGQEKTVYPKPSVAPAAVTFQSNQQDKGKAPLKGPQKASQKESQT   420
CAR95368.1          EQLPGDPDIVRFLDKFGQEKTVYPKPSVAPAAVTFQSNQQDKGKAPLKGPQKASQKESQT   420
CAR95369.1          EQLPGDPDIVRFLDKFGQEKTVYPKPSVAPAAVTFQSNQQDKGKAPLKGPQKASQKESQT   420
ACI25319.1          EQLPGDPDIVRFLDKFGQEKTVYPKPSVAPAAVTFQSNQQDKGKAPLKGPQKASQKESQT   420
UQK62672.1          EQLPGDPDIVRFLDKFGQEKTVYPKPSVAPAAVTFQSNQQDKGKAPLKGPQKASQKESQT   420
UQK62681.1          EQLPGDPDIVRFLDKFGQEKTVYPKPSVAPAAVTFQSNQQDKGKAPLKGPQKASQKESQT   420
UQK62685.1          EQLPGDPDIVRFLDKFGQEKTVYPKPSVAPAAVTFQSNQQDKGKAPLKGPQKASQKESQT   420
UQK62690.1          EQLPGDPDIVRFLDKFGQEKTVYPKPSVAPAAVTFQSNQQDKGKAPLKGPQKASQKESQT   420
UQK62693.1          EQLPGDPDIVRFLDKFGQEKTVYPKPSVAPAAVTFQSNQQDKGKAPLKGPQKASQKESQT   420
ANF28696.1          EQLPGDPDIVRFLDKFGQEKTVYPKPSVAPAAVTFQSNQQDKGKAPLKGPQKASQKESQT   420
ANF28699.1          EQLPGDPDIVRFLDKFGQEKTVYPKPSVAPAAVTFQSNQQDKGKAPLKGPQKASQKESQT   420
AEM01083.1          EQLPGDPDIVRFLDKFGQEKTVYPKPSVAPAAVTFQSNQQDKGKAPLKGPQKASQKESQT   420
ADE45343.1          EQLPGDPDIVRFLDKFGQEKTVYPKPSVAPAAVTFQSNQQDKGKAPLKGPQKASQKESQT   420
ADE45367.1          EQLPGDPDIVRFLDKFGQEKTVYPKPSVAPAAVTFQSNQQDKGKAPLKGPQKASQKESQT   420
ADE45371.1          EQLPGDPDIVRFLDKFGQEKTVYPKPSVAPAAVTFQSNQQDKGKAPLKGPQKASQKESQT   420
ADE45399.1          EQLPGDPDIVRFLDKFGQEKTVYPKPSVAPAAVTFQSNQQDKGKAPLKGPQKASQKESQT   420
CAR95365.1          EQLPGDPDIVRFLDKFGQEKTVYPKPSVAPAAVTFQSNQQDKGKAPLKGPQKASQKESQT   420
ABY65893.1          EQLPGDPDIVRFLDKFGQEKTVYPKPSVAPAAVTFQSNQQDKGKAPLKGPQKASQKESQT   420
BAN78700.1          EQLPGDPDIVRFLDKFGQEKTVYPKPSVAPAAVTFQSNQQDKGKAPLKGPQKASQKESQT   420
AHB32980.1          EQLPGDPDIVRFLDKFGQEKTVYPKSSVAPAAVTFQSNQQDKGKAPLKGPQKPSQKESQT   420
BAV01218.1          EQLPGDPDIVRFLDKFGQEKTVYPKLSVAPAAATFQSNQQDKGKAPLKGPQKASQKESQT   420
BAV01220.1          EQLPGDPDIVRFLDKFGQEKTVYPKLSVAPAAATFQSNQQDKGKAPLKGPQKASQKESQT   420
BAV01214.1          EQLPGDPDIVRFLDKFGQEKTVYPKLSVAPAAVTFQSNQQDKGKAPLKGPQKASQKESQT   420
BAV01216.1          EQLPGDPDIVRFLDKFGQEKTVYPKLSVAPAAVTFQSNQQEKGKAPLKGPQKASQKESQT   420
BBJ26577.1          EQLPGDPDIVRFLDKFGQEKTVYPKLSVAPAAVTFQSNQQDKGKAPLKGPQKASQKESQT   420
WKF20931.1          EQLPGDPDIVRFLDKFGQEKTVYPKLSVAPAAVTFQSNQQDKGKAPLKGPQKASQKESQT   420
BBJ26589.1          EQLPGDPDIVRFLDKFGQEKTVYPKLSVAPAAVTFQSNQQDKGKAPLKGPQKASQKESQT   420
BBJ26592.1          EQLPGDPDIVRFLDKFGQEKTVYPKLSVAPAAVTFQSNQQDKGKAPLKGPQKASQKESQT   420
BBJ26601.1          EQLPGDPDIVRFLDKFGQEKTVYPKLSVAPAAVTFQSNQQDKGKAPLKGPQKASQKESQT   420
BAV01209.1          EQLPGDPDIVRFLDKFGQEKTVYPKLSVAPAAVTFQSNQQDKGKAPLKGPQKASQKESQT   420
BAV01211.1          EQLPGDPDIVRFLDKFGQEKTVYPKLSVAPAAVTFQSNQQDKGKAPLKGPQKASQKESQT   420
BAV01212.1          EQLPGDPDIVRFLDKFGQEKTVYPKLSVAPAAVTFQSNQQDKGKAPLKGPQKASQKESQT   420
BAV01213.1          EQLPGDPDIVRFLDKFGQEKTVYPKLSVAPAAVTFQSNQQDKGKAPLKGPQKASQKESQT   420
BAV01215.1          EQLPGDPDIVRFLDKFGQEKTVYPKLSVAPAAVTFQSNQQDKGKAPLKGPQKASQKESQT   420
BAV01217.1          EQLPGDPDIVRFLDKFGQEKTVYPKLSVAPAAVTFQSNQQDKGKAPLKGPQKASQKESQT   420
BAV01219.1          EQLPGDPDIVRFLDKFGQEKTVYPKLSVAPAAVTFQSNQQDKGKAPLKGPQKASQKESQT   420
BAV01221.1          EQLPGDPDIVRFLDKFGQEKTVYPKLSVAPAAVTFQSNQQDKGKAPLKGPQKASQKESQT   420
BAV01225.1          EQLPGDPDIVRFLDKFGQEKTVYPKLSVAPAAVTFQSNQQDKGKAPLKGPQKASQKESQT   420
BAV01226.1          EQLPGDPDIVRFLDKFGQEKTVYPKLSVAPAAVTFQSNQQDKGKAPLKGPQKASQKESQT   420
BAV01227.1          EQLPGDPDIVRFLDKFGQEKTVYPKLSVAPAAVTFQSNQQDKGKAPLKGPQKASQKESQT   420
BAV01228.1          EQLPGDPDIVRFLDKFGQEKTVYPKLSVAPAAVTFQSNQQDKGKAPLKGPQKASQKESQT   420
BAN78690.1          EQLPGDPDIVRFLDKFGQEKTVYPKLSVAPAAVTFQSNQQDKGKAPLKGPQKASQKESQT   420
```

```
BAN78691.1          EQLPGDPDIVRFLDKFGQEKTVYPKLSVAPAAVTFQSNQQDKGKAPLKGPQKASQKESQT  420
BAN78696.1          EQLPGDPDIVRFLDKFGQEKTVYPKLSVAPAAVTFQSNQQDKGKAPLKGPQKASQKESQT  420
BAN78697.1          EQLPGDPDIVRFLDKFGQEKTVYPKLSVAPAAVTFQSNQQDKGKAPLKGPQKASQKESQT  420
BAN78698.1          EQLPGDPDIVRFLDKFGQEKTVYPKLSVAPAAVTFQSNQQDKGKAPLKGPQKASQKESQT  420
ADE45383.1          EQLPGDPDIVRFLDKFGQEKTVYPKLSVAPAAVTFQSNQQDKGKAPLKGPQKASQKESQT  420
ACL31697.1          EQLPGDPDIVRFLDKFGQEKTVYPKLSVAPAAVTFQSNQQDKGKAPLKGPQKASQKESQT  420
QBR98152.1          EQLPGDPDIVRFLDKFGQEKTVYPKLSVAPAAVTFQSNQQDKGKAPLKGPQKASQKESQT  420
BBJ26574.1          EQLPGDPDIVRFLDKFGQEKTVYPKLSVAPAAVTFQSNQQDKGKAPLKGPQKASQKESQT  420
BBJ26580.1          EQLPGDPDIVRFLDKFGQEKTVYPKLSVAPAAVTFQSNQQDKGKAPLKGPQKASQKESQT  420
BBJ26595.1          EQLPGDPDIVRFLDKFGQEKTVYPKLSVAPAAVTFQSNQQDKGKAPLKGPQKASQKESQT  420
BBJ26598.1          EQLPGDPDIVRFLDKFGQEKTVYPKLSVAPAAVTFQSNQQDKGKAPLKGPQKASQKESQT  420
BAV01210.1          EQLPGDPDIVRFLDKFGQEKTVYPKLSVAPAAVTFQSNQQDKGKAPLKGPQKASQKESQT  420
BAV01222.1          EQLPGDPDIVRFLDKFGQEKTVYPKLSVAPAAVTFQSNQQDKGKAPLKGPQKASQKESQT  420
BAV01223.1          EQLPGDPDIVRFLDKFGQEKTVYPKLSVAPAAVTFQSNQQDKGKAPLKGPQKASQKESQT  420
BAV01224.1          EQLPGDPDIVRFLDKFGQEKTVYPKLSVAPAAVTFQSNQQDKGKAPLKGPQKASQKESQT  420
BAV01230.1          EQLPGDPDIVRFLDKFGQEKTVYPKLSVAPAAVTFQSNQQDKGKAPLKGPQKASQKESQT  420
BAN78692.1          EQLPGDPDIVRFLDKFGQEKTVYPKLSVAPAAVTFQSNQQDKGKAPLKGPQKASQKESQT  420
BAN78693.1          EQLPGDPDIVRFLDKFGQEKTVYPKLSVAPAAVTFQSNQQDKGKAPLKGPQKASQKESQT  420
BAN78694.1          EQLPGDPDIVRFLDKFGQEKTVYPKLSVAPAAVTFQSNQQDKGKAPLKGPQKASQKESQT  420
                    * ***:******************* ******.******::*.********: **** **

ABY65888.1          QQL 423
QXF78584.1          QEL 423
QXF78586.1          QEL 423
ANF28692.1          QEL 423
AHW79948.1          QEL 423
AWG42111.1          QEL 423
AWG42117.1          QEL 423
ANF28693.1          QEL 423
ANF28694.1          QEL 423
ANF28695.1          QEL 423
ANF28698.1          QEL 423
CAR95366.1          QEL 423
AFM84435.1          QEL 423
AFM84450.1          QEL 423
AFM84455.1          QEL 423
ANF28697.1          QEL 423
ACN44192.1          QQL 422
ADE45355.1          QEL 423
ADE45359.1          QEL 423
ADE45351.1          QEL 423
ADE45347.1          QEL 423
AEM01087.1          QQL 423
AFC36083.1          QEL 423
AFM84460.1          QEL 423
AHW79944.1          QEL 423
BBJ26583.1          QEL 423
BBJ26586.1          QEL 423
APA21079.1          QEL 423
ADN95996.1          QEL 423
YP_009111420.1      QEL 423
UQK62677.1          QEL 423
AWG42121.1          QEL 423
AWG42124.1          QEL 423
AWG42134.1          QEL 423
AWG42140.1          QEL 423
AQM73644.1          QEL 423
BAV01229.1          QEL 423
AKK25202.1          QEL 423
AHN49701.1          QEL 423
AHN49705.1          QEL 423
AHN49709.1          QEL 423
AHW79940.1          QEL 423
AHN13648.1          QEL 423
BAN78699.1          QEL 423
AGA03918.1          QEL 423
AFM84440.1          QEL 423
```

AFM84445.1	QEL 423
AFC36077.1	QEL 423
AFC36088.1	QEL 423
AFC36093.1	QEL 423
AEM01095.1	QEL 423
AEM01098.1	QEL 423
ADE45363.1	QEL 423
ADE45375.1	QEL 423
ADE45379.1	QEL 423
ADE45387.1	QEL 423
ADE45391.1	QEL 423
ADE45395.1	QEL 423
ADE45403.1	QEL 423
ADE45407.1	QEL 423
ADE45411.1	QEL 423
ADE45415.1	QEL 423
ADE45419.1	QEL 423
CAR95364.1	QEL 423
CAR95367.1	QEL 423
CAR95368.1	QEL 423
CAR95369.1	QEL 423
ACI25319.1	QEL 423
UQK62672.1	QEL 423
UQK62681.1	QEL 423
UQK62685.1	QEL 423
UQK62690.1	QEL 423
UQK62693.1	QEL 423
ANF28696.1	QEL 423
ANF28699.1	QEL 423
AEM01083.1	QEL 423
ADE45343.1	QEL 423
ADE45367.1	QEL 423
ADE45371.1	QEL 423
ADE45399.1	QEL 423
CAR95365.1	QEL 423
ABY65893.1	QEL 423
BAN78700.1	QEL 423
AHB32980.1	QEL 423
BAV01218.1	QEL 423
BAV01220.1	QEL 423
BAV01214.1	QEL 423
BAV01216.1	QEL 423
BBJ26577.1	QEL 423
WKF20931.1	QEL 423
BBJ26589.1	QEL 423
BBJ26592.1	QEL 423
BBJ26601.1	QEL 423
BAV01209.1	QEL 423
BAV01211.1	QEL 423
BAV01212.1	QEL 423
BAV01213.1	QEL 423
BAV01215.1	QEL 423
BAV01217.1	QEL 423
BAV01219.1	QEL 423
BAV01221.1	QEL 423
BAV01225.1	QEL 423
BAV01226.1	QEL 423
BAV01227.1	QEL 423
BAV01228.1	QEL 423
BAN78690.1	QEL 423
BAN78691.1	QEL 423
BAN78696.1	QEL 423
BAN78697.1	QEL 423
BAN78698.1	QEL 423
ADE45383.1	QEL 423
ACL31697.1	QEL 423
QBR98152.1	QEL 423

```
BBJ26574.1          QEL 423
BBJ26580.1          QEL 423
BBJ26595.1          QEL 423
BBJ26598.1          QEL 423
BAV01210.1          QEL 423
BAV01222.1          QEL 423
BAV01223.1          QEL 423
BAV01224.1          QEL 423
BAV01230.1          QEL 423
BAN78692.1          QEL 423
BAN78693.1          QEL 423
BAN78694.1          QEL 423
                    *:*
```
