# Supplementary material for: The Conserved YPX3L Motif in the BK Polyomavirus VP1 Protein Is Important for Viral Particle Assembly but Not for Its Secretion into Extracellular Vesicles
Source: Viruses. 2024 Jul 13;16(7):1124. doi: 10.3390/v16071124 (PMC11281352; doi:10.3390/v16071124)
Supplement: Supplementary file 1 [file viruses-16-01124-s001.zip › HPyV 06 alignment.pdf]

CLUSTAL O(1.2.4) multiple sequence alignment

```
AQM73635.1          MPCHRKGNGPIQKLPRVIKKGGVEVMETVPLSEDTIYKVEAILLPNFASGSNTAVYQSRG   60
AWG42085.1          MPCHRKGSGPIQKLPRVIKKGGVEVLETVPLSEDTVYKVEAILLPNFASGSNTAVYQSRG   60
AMQ36137.1          MPCHRKGNGPIQKLPRVIKKGGVEVLETVPLSEDTVYKVEAILLPNFASGSNTAVYQSRG   60
APC92530.1          MPCHRKGNGPIQKLPRVIKKGGVEVLETVPLSEDTVYKVEAILLPNFASGSNTAVYQSRG   60
APC92535.1          MPCHRKGNGPIQKLPRVIKKGGVEVLETVPLSEDTVYKVEAILLPNFASGSNTAVYQSRG   60
AKE33247.1          MPCHRKGNGPIQKLSRVIKKGGVEVLETVPLSEDTIYKVEAILLPNFASGSNTAVYQSRG   60
AMQ77265.1          MPCHRKGNGPIQKLPRVIKKGGVEVLETVPLSEDTIYKVEAILLPNFASGSNTAVYQSRG   60
AWG42094.1          MPCHRKGNGPIQKLPRVIKKGGVEVMETVPLSEDTIYKVEAILLPNFASGSNTAVYQSRG   60
ADE45449.1          MPCHRKGNGPIQKLPRVIKKGGVEVMETVPLSEDTIYKVEAILLPNFASGSNTAVYQSRG   60
YP_003848918.1      MPCHRKGNGPIQKLPRVIKKGGVEVMETVPLSEDTIYKVEAILLPNFASGSNTAVYQSRG   60
UQK62457.1          MPCHRKGNGPIQKLPRVIKKGGVEVMETVPLSEDTIYKVEAILLPNFASGSNTAVYQSRG   60
AIY25480.1          MPCHRKGNGPIQKLPRVIKKGGVEVMETVPLSEDTIYKVEAILLPNFASGSNTAVYQSRG   60
ADE45424.1          MPCHRKGNGPIQKLPRVIKKGGVEVMETVPLSEDTIYKVEAILLPNFASGSNTAVYQSRG   60
ADE45429.1          MPCHRKGNGPIQKLPRVIKKGGVEVMETVPLSEDTIYKVEAILLPNFASGSNTAVYQSRG   60
ADE45434.1          MPCHRKGNGPIQKLPRVIKKGGVEVMETVPLSEDTIYKVEAILLPNFASGSNTAVYQSRG   60
ADE45439.1          MPCHRKGNGPIQKLPRVIKKGGVEVMETVPLSEDTIYKVEAILLPNFASGSNTAVYQSRG   60
ADE45444.1          MPCHRKGNGPIQKLPRVIKKGGVEVMETVPLSEDTIYKVEAILLPNFASGSNTAVYQSRG   60
AWG42099.1          MPCHRKGNGPIQKLPRVIKKGGVEVMETVPLSEDTIYKVEAILLPNFASGSNTAVYQSRG   60
                    *******.****** **********:*********:************************

AQM73635.1          APYTFTDTTLDAGSSLCYTLAVVNLPEIPEALCDDTLLVWEAFRVETELIFTPQVGSAGY   120
AWG42085.1          APYTFTD-TLDAGSSLCYTLAVVNLPEIPEALCDDTLLVWEAFRVETELIFTPQVGSAGY   119
AMQ36137.1          APYTFTD-TLDAGSSLCYTLAVVNLPEIPEALCDDTLLVWEAFRVETELIFTPQVGSAGY   119
APC92530.1          APYTFTD-TLDAGSSLCYTLAVVNLPEIPEALCDDTLLVWEAFRVETELIFTPQVGSAGY   119
APC92535.1          APYTFTD-TLDAGSSLCYTLAVVNLPEIPEALCDDTLLVWEAFRVETELIFTPQVGSAGY   119
AKE33247.1          APYTFTD-TLDAGSSLCYTLAVVNLPEIPEALCDDTLLVWEAFRVETELIFTPQVGSAGY   119
AMQ77265.1          APYTFTD-TLDAGSSLCYTLAVVNLPEIPEALCDDTLLVWEAFRVETELIFTPQVGSAGY   119
AWG42094.1          APYTFTD-TLDAGSSLCYTLAVVNLPEIPEALCDDTLLVWEAFRVETELIFTPQVGSAGY   119
ADE45449.1          APYTFTD-TLDAGSSLCYTLAVVNLPEIPEALCDDTLLVWEAFRVETELIFTPQLGSAGY   119
YP_003848918.1      APYTFTD-TLDAGSSLCYTLAVVNLPEIPEALCDDTLLVWEAFRVETELIFTPQVGSAGY   119
UQK62457.1          APYTFTD-TLDAGSSLCYTLAVVNLPEIPEALCDDTLLVWEAFRVETELIFTPQVGSAGY   119
AIY25480.1          APYTFTD-TLDAGSSLCYTLAVVNLPEIPEALCDDTLLVWEAFRVETELIFTPQVGSAGY   119
ADE45424.1          APYTFTD-TLDAGSSLCYTLAVVNLPEIPEALCDDTLLVWEAFRVETELIFTPQVGSAGY   119
ADE45429.1          APYTFTD-TLDAGSSLCYTLAVVNLPEIPEALCDDTLLVWEAFRVETELIFTPQVGSAGY   119
ADE45434.1          APYTFTD-TLDAGSSLCYTLAVVNLPEIPEALCDDTLLVWEAFRVETELIFTPQVGSAGY   119
ADE45439.1          APYTFTD-TLDAGSSLCYTLAVVNLPEIPEALCDDTLLVWEAFRVETELIFTPQVGSAGY   119
ADE45444.1          APYTFTD-TLDAGSSLCYTLAVVNLPEIPEALCDDTLLVWEAFRVETELIFTPQVGSAGY   119
AWG42099.1          APYTFTD-TLDAGSSLCYTLAVVNLPEIPEALCDDTLLVWEAFRVETELIFTPQVGSAGY   119
                    ******* **********************************************:*****

AQM73635.1          IKAQGTPAGVEGSQMYFWACGGSPLDVIGINPDPERMNVAAGLEGPSKENQPSVAGIKAT   180
AWG42085.1          IRAQGTPAGVEGSQMYFWACGGSPLDVIGINPDPERMNVAAGLEGPSKENQPSVAGIKAT   179
AMQ36137.1          IRAQGTPAGVEGSQMYFWACGGSPLDVIGINPDPERMNVAAGLEGPSKENQPSVAGIKAT   179
APC92530.1          IRAQGTPAGVEGSQMYFWACGGSPLDVIGINPDPERMNVAAGLEGPSKENQPSVAGIKAT   179
APC92535.1          IRAQGTPAGVEGSQMYFWACGGSPLDVIGINPDPERMNVAAGLEGPSKENQPSVAGIKAT   179
AKE33247.1          IRAQGTPAGVEGSQMYFWACGGSPLDVIGINPDPERMNVAAGLEGPSKENQPSVAGIKAT   179
AMQ77265.1          IRAQGTPAGVEGSQMYFWACGGSPLDVIGINPDPERMNVAAGLEGPSKENQPSVAGIKAT   179
AWG42094.1          IRAQGTPAGVEGSQMYFWACGGSPLGVIGINPDPERMNVAAGLEGPSKENQPSVAGIKAT   179
ADE45449.1          IRAQGTPAGVEGSQMYFWACGGSPLDVIGINPDPERMNVAAGLEGPSKENQPSVAGIKAT   179
YP_003848918.1      IRAQGTPAGVEGSQMYFWACGGSPLDVIGINPDPERMNVAAGLEGPSKENQPSVAGIKAT   179
UQK62457.1          IRAQGTPAGVEGSQMYFWACGGSPLDVIGINPDPERMNVAAGLEGPSKENQPSVAGIKAT   179
AIY25480.1          IRAQGTPAGVEGSQMYFWACGGSPLDVIGINPDPERMNVAAGLEGPSKENQPSVAGIKAT   179
ADE45424.1          IRAQGTPAGVEGSQMYFWACGGSPLDVIGINPDPERMNVAAGLEGPSKENQPSVAGIKAT   179
ADE45429.1          IRAQGTPAGVEGSQMYFWACGGSPLDVIGINPDPERMNVAAGLEGPSKENQPSVAGIKAT   179
ADE45434.1          IRAQGTPAGVEGSQMYFWACGGSPLDVIGINPDPERMNVAAGLEGPSKENQPSVAGIKAT   179
ADE45439.1          IRAQGTPAGVEGSQMYFWACGGSPLDVIGINPDPERMNVAAGLEGPSKENQPSVAGIKAT   179
ADE45444.1          IRAQGTPAGVEGSQMYFWACGGSPLDVIGINPDPERMNVAAGLEGPSKENQPSVAGIKAT   179
AWG42099.1          IRAQGTPAGVEGSQMYFWACGGSPLDVIGINPDPERMNVAAGLEGPSKENQPSVAGIKAT   179
                    *:***********************.**********************************

AQM73635.1          RKQVTAANFPIEIWSADPTRNENCRYFGRIVGGSVTPPVVSFGNQSTTPLVDENGVGILC   240
AWG42085.1          RKQVTAANFPIEIWSADPTRNENCRYFGRIVGGSVTPPVVSFGNQSTTPLVDENGVGILC   239
AMQ36137.1          RKQVTAANFPIEIWSADPTRNENCRYFGRIVGGSVTPPVVSFGNQSTTPLVDENGVGILC   239
APC92530.1          RKQVTAANFPIEIWSADPTRNENCRYFGRIVGGSVTPPVVSFGNQSTTPLVDENGVGILC   239
```

```
APC92535.1          RKQVTAANFPIEIWSADPTRNENCRYFGRIVGGSVTPPVVSFGNQSTTPLVDENGVGILC  239
AKE33247.1          RKQVTAANFPIEIWSADPTRNENCRYFGRIVGGSVTPPVVSFGNQSTTPLVDENGVGILC  239
AMQ77265.1          RKQVTAANFPIEIWSADPTRNENCRYFGRIVGGSVTPPVVSFGNQSTTPLVDENGVGILC  239
AWG42094.1          RKQVTAANFPIEIWSADPTRNENCRYFGRIVGGSVTPPVVSFGNQSTTPLVDENGVGILC  239
ADE45449.1          RKQVTAANFPIEIWSADPTRNENCRYFGRIVGGSVTPPVVSFGNQSTTPLVDENGVGILC  239
YP_003848918.1      RKQVTAANFPIEIWSADPTRNENCRYFGRIVGGSVTPPVVSFGNQSTTPLVDENGVGILC  239
UQK62457.1          RKQVTAANFPIEIWSADPTRNENCRYFGRIVGGSVTPPVVSFGNQSTTPLVDENGVGILC  239
AIY25480.1          RKQVTAANFPIEIWSADPTRNENCRYFGRIVGGSVTPPVVSFGNQSTTPLVDENGVGILC  239
ADE45424.1          RKQVTAANFPIEIWSADPTRNENCRYFGRIVGGSVTPPVVSFGNQSTTPLVDENGVGILC  239
ADE45429.1          RKQVTAANFPIEIWSADPTRNENCRYFGRIVGGSVTPPVVSFGNQSTTPLVDENGVGILC  239
ADE45434.1          RKQVTAANFPIEIWSADPTRNENCRYFGRIVGGSVTPPVVSFGNQSTTPLVDENGVGILC  239
ADE45439.1          RKQVTAANFPIEIWSADPTRNENCRYFGRIVGGSVTPPVVSFGNQSTTPLVDENGVGILC  239
ADE45444.1          RKQVTAANFPIEIWSADPTRNENCRYFGRIVGGSVTPPVVSFGNQSTTPLVDENGVGILC  239
AWG42099.1          RKQVTAANFPIEIWSADPTRNENCRYFGRIVGGSVTPPVVSFGNQSTTPLVDENGVGILC  239
                    ************************************************************

AQM73635.1          LFGAIYLTSADMLGMVGYAGNPTLSDAYSQQRSVQAAFGRFFRVHFRQRRVKHPYTVDMM  300
AWG42085.1          LYGAIYLTSADMLGMVGYAGNPTLSDAYSQQRTVQAAFGRFFRVHFRQRRVKHPYTVDMM  299
AMQ36137.1          LYGAIYLTSADMLGMVGYAGNPTLSDAYSQQRTVQAAFGRFFRVHFRQRRVKHPYTVDMM  299
APC92530.1          LFGAIYLTSADMLGMVGYAGNPTLSDAYSQQRTVQAAFGRFFRVHFRQRRVKHPYTVDMM  299
APC92535.1          LFGAIYLTSADMLGMVGYAGNPTLSDAYSQQRSVQAAFGRFFRVHFRQRRVKHPYTVDMM  299
AKE33247.1          LFGAIYLTSADMLGMVGYAGNPTLSDAYSQQRSVQAAFGRFFRVHFRQRRVKHPYTVDMM  299
AMQ77265.1          LFGAIYLTSADMLGMVGYAGNPTLSDAYSQQRSIQAAFGRFFRVHFRQRRVKHPYTVDMM  299
AWG42094.1          LFGAIYLTSADMLGMVGHAGNPTLSDAYSQQRSVQAAFGRFFRVHFRQRRVKHPYTVDMM  299
ADE45449.1          LFGAIYLTSADMLGMVGYAGNPTLSDAYSQQRSVQAAFGRFFRVHFRQRRVKHPYTVDMM  299
YP_003848918.1      LFGAIYLTSADMLGMVGYAGNPTLSDAYSQQRSVQAAFGRFFRVHFRQRRVKHPYTVDMM  299
UQK62457.1          LFGAIYLTSADMLGMVGYAGNPTLSDAYSQQRSVQAAFGRFFRVHFRQRRVKHPYTVDMM  299
AIY25480.1          LFGAIYLTSADMLGMVGYAGNPTLSDAYSQQRSVQAAFGRFFRVHFRQRRVKHPYTVDMM  299
ADE45424.1          LFGAIYLTSADMLGMVGYAGNPTLSDAYSQQRSVQAAFGRFFRVHFRQRRVKHPYTVDMM  299
ADE45429.1          LFGAIYLTSADMLGMVGYAGNPTLSDAYSQQRSVQAAFGRFFRVHFRQRRVKHPYTVDMM  299
ADE45434.1          LFGAIYLTSADMLGMVGYAGNPTLSDAYSQQRSVQAAFGRFFRVHFRQRRVKHPYTVDMM  299
ADE45439.1          LFGAIYLTSADMLGMVGYAGNPTLSDAYSQQRSVQAAFGRFFRVHFRQRRVKHPYTVDMM  299
ADE45444.1          LFGAIYLTSADMLGMVGYAGNPTLSDAYSQQRSVQAAFGRFFRVHFRQRRVKHPYTVDMM  299
AWG42099.1          LFGAIYLTSADMLGMVGYAGNPTLSDAYSQQRSVQAAFGRFFRVHFRQRRVKHPYTVDMM  299
                    *:***************:**************::**************************

AQM73635.1          FRQFLQPQKPQVQGTQPNAVQEVVMEQMQPSILPTTLEGAIGYSPSTKFILQNGELIYPS  360
AWG42085.1          FRQFLQPQKPQVQGTQPNAVQEVVMEQMQPSILPTTLEGAIGYSPSTKFILQNGELIYPS  359
AMQ36137.1          FRQFLQPQKPQVQGTQPNAVQEVVMEQMQPSILPTTLEGAIGYSPSTKFILQNGELIYPS  359
APC92530.1          FRQFLQPQKPQVQGTQPNAVQEVVMEQMQPSILPTTLEGAIGYSPSTKFILQNGELIYPS  359
APC92535.1          FRQFLQPQKPQVQGTQPNAVQEVVMEQMQPSILPTTLEGAIGYSPSTKFILQNGELIYPS  359
AKE33247.1          FRQFLQPQKPQVQGIQPNAVQEVVMEQMQPSILPTTLEGAIGYSPSTKFILQNGELIYPS  359
AMQ77265.1          FRQFLQPQKPQVQGTQPNAVQEVVMEQIQPSILPTTLEGAIGYSPSTKFILQNGELIYPS  359
AWG42094.1          FRQFLQPQKPQVQGTQPNAVQEVVMEQMQPSLLPTTLEGAIGYSPSTKFILQNGELIYPS  359
ADE45449.1          FRQFLQPQKPQVQGTQPNAVQEVVMEQMQPSILPTTLEGAIGYSPSTKFILQNGELIYPS  359
YP_003848918.1      FRQFLQPQKPQVQGTQPNAVQEVVMEQMQPSILPTTLEGAIGYSPSTKFILQNGELIYPS  359
UQK62457.1          FRQFLQPQKPQVQGTQPNAVQEVVMEQMQPSILPTTLEGAIGYSPSTKFILQNGELIYPS  359
AIY25480.1          FRQFLQPQKPQVQGTQPNAVQEVVMEQMQPSILPTTLEGAIGYSPSTKFILQNGELIYPS  359
ADE45424.1          FRQFLQPQKPQVQGTQPNAVQEVVMEQMQPSILPTTLEGAIGYSPSTKFILQNGELIYPS  359
ADE45429.1          FRQFLQPQKPQVQGTQPNAVQEVVMEQMQPSILPTTLEGAIGYSPSTKFILQNGELIYPS  359
ADE45434.1          FRQFLQPQKPQVQGTQPNAVQEVVMEQMQPSILPTTLEGAIGYSPSTKFILQNGELIYPS  359
ADE45439.1          FRQFLQPQKPQVQGTQPNAVQEVVMEQMQPSILPTTLEGAIGYSPSTKFILQNGELIYPS  359
ADE45444.1          FRQFLQPQKPQVQGTQPNAVQEVVMEQMQPSILPTTLEGAIGYSPSTKFILQNGELIYPS  359
AWG42099.1          FRQFLQPQKPQVQGTQPNAVQEVVMEQMQPSLLPTTLEGAIGYSPSTKFILQNGELIYPS  359
                    ************** ************:***:****************************

AQM73635.1          STVAAGAANLFGPPVQKQTSKEPSKGEL     388
AWG42085.1          STVAAGAANLFGPPVEKQTSKEPSKGEL     387
AMQ36137.1          STVAAGAANLFGPPVEKQTSKEPSKGEL     387
APC92530.1          STVAAGAANLFGPPVEKQTSKEPSKGEL     387
APC92535.1          STVAAGAANLFGPPVEKQTSKEPSKGEL     387
AKE33247.1          STVAAGAANLFGPPVEKQTSKEPSKGEL     387
AMQ77265.1          STVAAGAANLFGPPVEKQTSKEPSKGEL     387
AWG42094.1          STVAAGAANLFGPPVEKQTSKEPSKGEL     387
ADE45449.1          STVAAGAANLFGPPVEKQTSKEPSKGEL     387
YP_003848918.1      STVAAGAANLFGPPVEKQTSKEPSKGEL     387
UQK62457.1          STVAAGAANLFGPPVEKQTSKEPSKGEL     387
```

```
AIY25480.1          STVAAGAANLFGPPVEKQTSKEPSKGEL     387
ADE45424.1          STVAAGAANLFGPPVEKQTSKEPSKGEL     387
ADE45429.1          STVAAGAANLFGPPVEKQTSKEPSKGEL     387
ADE45434.1          STVAAGAANLFGPPVEKQTSKEPSKGEL     387
ADE45439.1          STVAAGAANLFGPPVEKQTSKEPSKGEL     387
ADE45444.1          STVAAGAANLFGPPVEKQTSKEPSKGEL     387
AWG42099.1          STVAAGAANLFGPPVEKQTSKEPSKGEL     387
                    ***************:************
```
