# Supplementary material for: The Conserved YPX3L Motif in the BK Polyomavirus VP1 Protein Is Important for Viral Particle Assembly but Not for Its Secretion into Extracellular Vesicles
Source: Viruses. 2024 Jul 13;16(7):1124. doi: 10.3390/v16071124 (PMC11281352; doi:10.3390/v16071124)
Supplement: Supplementary file 1 [file viruses-16-01124-s001.zip › HPyV 07 alignment.pdf]

CLUSTAL O(1.2.4) multiple sequence alignment

```
AXF48237.1          MPCQRKGNGPPQKLPRVIRKGGVEVLDTVPLTEDTVYKVEAVLLPNFGKGATTGNFQSRG   60
QJD09036.1          MPCQRKGNGPPQKLPRVIXKGGVEVLDTVPLTEDTQYKVEAVLLPNFGKAATTGNFQSRG   60
QJD09021.1          MPCQRKGNGPPQKLPRVIRKGGVEVLDTVPLTEDTQYKVEAVLLPNFGKAATTGNFQSRG   60
QJD09016.1          MPCQXKGNGPPQKLPRVIRKGGVEVLDTVPLTEDTQYKVEAVLLPNFGKAATTGNFQSRG   60
QJD09011.1          MPCQRKGNGPPQKLPRVIXKGGVEVLDTVPLTEDTQYKVEAVLLPNFGKAATTGNFQSRG   60
QJD09032.1          MPCQRKGNGPPQKLPRVIXKGGVEVLDTVPLTEDTQYKVEAVLLPNFGKAATTGNFQSRG   60
QJD09026.1          MPCQRKGNGPPQKLPRVIRKGGVEVLDTVPLTEDTQYKVEAVLLPNFGKAATTGNFQSRG   60
AIP98377.1          MPCQRKGNGPPQKLPRVIRKGGVEVLDTVPLTEDTQYKVEAVLLPNFGKAATTGNFQSRG   60
ADE45474.1          MPCQRKGNGPPQKLPRVIRKGGVEVLDTVPLTEDTQYKVEAVLLPNFGKAATTGNFQSRG   60
YP_003848923.1      MPCQRKGNGPTQKLPRVIRKGGVEVLDTVPLTEETQYKVEAVLLPNFGKAATTGNFQSRG   60
ADE45464.1          MPCQRKGNGPTQKLPRVIRKGGVEVLDTVPLTEETQYKVEAVLLPNFGKAATTGNFQSRG   60
ADE45469.1          MPCQRKGNGPTQKLPRVIRKGGVEVLDTVPLTEETQYKVEAVLLPNFGKAATTGNFQSRG   60
AQM73640.1          MPCQRKGNGPTQKLPRVIRKGGVEVLDTVPLTEDTQYKVEAVLLPNFGKAATTGNFQSRG   60
ADE45479.1          MPCQRKGNGPTQKLSRVIRKGGVEVLDTVPLTEDTQYKVEAVLLPNFGKAATTGNFQSRG   60
AIP98372.1          MPCQRKGNGPTQKLSRVIRKGGVEVLDTVPLTEDTQYKVEAVLLPNFGKAATTGNFQSRG   60
ADE45454.1          MPCQRKGNGPTQKLSRVIKKGGVEVLDTVPLTEDTQYKVEAVLLPNFGKAATTGNFQSRG   60
ADE45459.1          MPCQRKGNGPTQKLSRVIKKGGVEVLDTVPLTEDTQYKVEAVLLPNFGKAATTGNFQSRG   60
                    **** ***** *** *** **************:* *************.**********

AXF48237.1          LPYPMSDTLGPGAALCYSVAVINLPEIPDAMCEDTMIVWEAYRLETELLFAPQMASSGYQ   120
QJD09036.1          LPYTMSDTLGPGAALCYSVAVINLPEIPDAMCEDTMIVWEAYRLETELLFAPQMASSGYQ   120
QJD09021.1          LPYTMSDTLGPGAALCYSVAVINLPEIPDAMCEDTMIVWEAYRLETELLFAPQMASSGYQ   120
QJD09016.1          LPYTMSDTLGPGAALCYSVAVINLPEIPDAMCEDTMIVWEAYRLETELLFAPQMASSGYQ   120
QJD09011.1          LPYTMSDTLGPGAALCYSVAVINLPEIPDAMCEDTMIVWEAYRLETELLFAPQMASSGYQ   120
QJD09032.1          LPYTMSDTLGPGAALCYSVAVINLPEIPDAMCEDTMIVWEAYRLETELLFAPQMASSGYQ   120
QJD09026.1          LPYTMSDTLGPGAALCYSVAVINLPEIPDAMCEDTMIVWEAYRLETELLFAPQMASSGYQ   120
AIP98377.1          LPYTMSDTLGPGAALCYSVAVINLPEIPDAMCEDTMIVWEAYRLETELLFAPQMASSGYQ   120
ADE45474.1          LPYTMSDTLGPGAALCYSVAVINLPEIPDAMCEDTMIVWEAYRLETELLFAPQMASSGYQ   120
YP_003848923.1      LPYPMSDTLGPGAALCYSVAVINLPEIPDAMCEDTMIVWEAYRLETELLFAPQMASSGYQ   120
ADE45464.1          LPYPMSDTLGPGAALCYSVAVINLPEIPDAMCEDTMIVWEAYRLETELLFAPQMASSGYQ   120
ADE45469.1          LPYPMSDTLGPGAALCYSVAVINLPEIPDAMCEDTMIVWEAYRLETELLFAPQMASSGYQ   120
AQM73640.1          LPYPMSDTLGPGAALCYSVAVINLPEIPDAMCEDTMIVWEAYRLETELLFAPQMASSGYQ   120
ADE45479.1          LPYTMSDTLGPGAALCYSVAVINLPEIPDAMCEDTMIVWEAYRLETELLFAPQMASSGYQ   120
AIP98372.1          LPYTMSDTLGPGAALCYSVAVINLPEIPDAMCEDTMIVWEAYRLETELLFAPQMASSGYQ   120
ADE45454.1          LPYTMSDTLGPGAALCYSVAVINLPEIPDAMCEDTMIVWEAYRLETELLFAPQMASSGYQ   120
ADE45459.1          LPYTMSDTLGPGAALCYSVAVINLPEIPDAMCEDTMIVWEAYRLETELLFAPQMASSGYQ   120
                    *** ********************************************************

AXF48237.1          RANGTLAGIEGSQLYFWACGGGPLDVIGINPDPERLKVNEALEVPGNTDVASLQALRKQV   180
QJD09036.1          RANGTLAGIEGTQLYFWACGGGPLDVIGINPDPERLKVNXALEGPGNSDVASLQALRKQV   180
QJD09021.1          RANGTLAGIEGTQLYFWACGGGPLDVIGINPDPERLKVNXALEGPGNSDVASLQALRKQV   180
QJD09016.1          RANGTLAGIEGTQLYFWACGGGPLDVIGINPDPERLKVNEALEGPGNSDVASLQALRKQV   180
QJD09011.1          RANGTLAGIEGTQLYFWACGGGPLDVIGINPDPERLKVNEALEGPGNSDVASLQALRKQV   180
QJD09032.1          RANGTLAGIEGTQLYFWACGGGPLDVIGINPDPERLKVNEALEGPGNSDVASLQALRKQV   180
QJD09026.1          RANGTLAGIEGTQLYFWACGGGPLDVIGINPDPERLKVNEALEGPGNSDVASLQALRKQV   180
AIP98377.1          RANGTLAGIEGTQLYFWACGGGPLDVIGINPDPERLKVNEALEGPGNSDVASLQALRKQV   180
ADE45474.1          RANGTLAGIEGTQLYFWACGGGPLDVIGINPDPERLKVNEALEGPGNSDVASLQALRKQV   180
YP_003848923.1      RANGTLAGTEGSQLYFWACGGGPLDVIGINPDPERLKVNEALEGPGNTDVASLQALRKQV   180
ADE45464.1          RANGTLAGTEGSQLYFWACGGGPLDVIGINPDPERLKVNEALEGPGNTDVASLQALRKQV   180
ADE45469.1          RANGTLAGTEGSQLYFWACGGGPLDVIGINPDPERLKVNEALEGPGNTDVASLQALRKQV   180
AQM73640.1          RANGTLAGTEGSQLYFWACGGGPLDVIGINPDPERLKVNEALEGPGNTDVASLQALRKQV   180
ADE45479.1          RANGTLAGIEGTQLYFWACGGGPLDVIGINPDPERLKVNEALEGPGNSDVASLQALRKQV   180
AIP98372.1          RANGTLAGIEGTQLYFWACGGGPLDVIGINPDPERLKVNEALEGPGNSDVASLQALRKQV   180
ADE45454.1          RANGTLAGIEGTQLYFWACGGGPLDVIGINPDPERLKVNEALEGPGNSDVASLQALRKQV   180
ADE45459.1          RANGTLAGIEGTQLYFWACGGGPLDVIGINPDPDRLKVNEALEGPGNSDVASLQALRKQV   180
                    ******** **:*********************:***** *** ***:************

AXF48237.1          NAANFPVELWVADPTKNDNTRYFGRVVGGGVTPPVVSYGNQSTTPLIDENGVGILCSFGS   240
QJD09036.1          NAANFPVELWVADPTKNDNTRYFGRVVGGGVTPPVVSYGNQSTTPLIDENGVGILCTFGS   240
QJD09021.1          NAANFPVELWVADPTKNDNTRYFGRVVGGGVTPPVVSYGNQSTTPLIDENGVGILCTFGS   240
QJD09016.1          NAANFPVELWVADPTKNDNTRYFGRVVGGGVTPPVVSYGNQSTTPLIDENGVGILCTFGS   240
QJD09011.1          NAANFPVELWVADPTKNDNTRYFGRVVGGGVTPPVVSYGNQSTTPLIDENGVGILCTFGS   240
QJD09032.1          NAANFPVELWVADPTKNDNTRYFGRVVGGGVTPPVVSYGNQSTTPLIDENGVGILCTFGS   240
QJD09026.1          NAANFPVELWVADPTKNDNTRYFGRVVGGGVTPPVVSYGNQSTTPLIDENGVGILCTFGS   240
```

```
AIP98377.1           NAANFPVELWVADPTKNDNTRYFGRVVGGGVTPPVVSYGNQSTTPLIDENGVGILCTFGS  240
ADE45474.1           NAANFPVELWVADPTKNDNTRYFGRVVGGGVTPPVVSYGNQSTTPLIDENGVGILCTFGS  240
YP_003848923.1       NAANFPVELWVADPTKNDNTRYFGRVVGGGVTPPVVSYGNQSTTPLIDENGVGILCTFGS  240
ADE45464.1           NAANFPVELWVADPTKNDNTRYFGRVVGGGVTPPVVSYGNQSTTPLIDENGVGILCTFGS  240
ADE45469.1           NAANFPVELWVADPTKNDNTRYFGRVVGGGVTPPVVSYGNQSTTPLIDENGVGILCTFGS  240
AQM73640.1           NAANFPVELWVADPTKNDNTRYFGRVVGGGVTPPVVSYGNQSTTPLIDENGVGILCTFGS  240
ADE45479.1           NAANFPVELWVADPTKNDNTRYFGRVVGGGVTPPVVSYGNQSTTPLIDENGVGILCTFGS  240
AIP98372.1           NAANFPVELWVADPTKNDNTRYFGRVVGGGVTPPVVSYGNQSTTPLIDENGVGILCSFGS  240
ADE45454.1           NAANFPVELWVADPTKNDNTRYFGRVVGGGVTPPVVSYGNQSTTPLIDENGVGILCTFGS  240
ADE45459.1           NAANFPVELWVADPTKNDNTRYFGRVVGGGVTPPVVSYGNQSTTPLIDENGVGILCTFGS  240
                     ********************************************************:***

AXF48237.1           VYLTSADMIGMTGLPGLPTLSADYSNQRTVQAGYGRFFRVHCRQRRIKHPYTVDMMFRQF  300
QJD09036.1           VYLTSADMVGMTGLPGLPTLSADYSNQRTVQAGYGRFFRVHCRQRRIKHPYTVDMMFRQF  300
QJD09021.1           VYLTSADMVGMTGLPGLPTLSADYSNQRTVQAGYGRFFRVHCRQRRIKHPYTVDMMFRQF  300
QJD09016.1           VYLTSADMVGMTGLPGLPTLSADYSNQRTVQAGYGRFFRVHCRQRRIKHPYTVDMMFRQF  300
QJD09011.1           VYLTSADMVGMTGLPGLPTLSADYSNQRTVQAGYGRFFRVHCRQRRIKHPYTVDMMFRQF  300
QJD09032.1           VYLTSADMVGMTGLPGLPTLSADYSNQRTVQAGYGRFFRVHCRQRRIKHPYTVDMMFRQF  300
QJD09026.1           VYLTSADMVGMTGLPGLPTLSADYSNQRTVQAGYGRFFRVHCRQRRIKHPYTVDMMFRQF  300
AIP98377.1           VYLTSADMVGMTGLPGLPTLSADYSNQRTVQAGYGRFFRVHCRQRRIKHPYTVDMMFRQF  300
ADE45474.1           VYLTSADMVGMTGLPGLPTLSADYSNQRTVQAGYGRFFRVHCRQRRIKHPYTVDMMFRQF  300
YP_003848923.1       VYLTSADMVGMTGLPGLPTLSADYSNQRTVQAGYGRFFRVHCRQRRIKHPYTVDMMFRQF  300
ADE45464.1           VYLTSADMVGMTGLPGLPTLSADYSNQRTVQAGYGRFFRVHCRQRRIKHPYTVDMMFRQF  300
ADE45469.1           VYLTSADMVGMTGLPGLPTLSADYSNQRTVQAGYGRFFRVHCRQRRIKHPYTVDMMFRQF  300
AQM73640.1           VYLTSADMVGMTGLPGLPTLSADYSNQRTVQAGYGRFFRVHCRQRRIKHPYTVDMMFRQF  300
ADE45479.1           VYLTSADMVGMTGLPGLPTLSADYSNQRTVQAGYGRFFRVHCRQRRIKHPYTVDMMFRQF  300
AIP98372.1           VYLTSADMVGMTGLPGLPTLSADYSNQRTVQAGYGRFFRVHCRQRRIKHPYTVDMMFRQF  300
ADE45454.1           VYLTSADMVGMTGLPGLPTLSADYSNQRTVQAGYGRFFRVHCRQRRIKHPYTVDMMFRQF  300
ADE45459.1           VYLTSADMVGMTGLPGLPTLSADYSNQRTVQAGYGRFFRVHCRQRRIKHPYTVDMMFRQF  300
                     ********:***************************************************

AXF48237.1           LQPQKPQVQGQQPAAVQEVTMEQMQPATIPPTVEGGLGFAPTSKFLLQNGELIYPSSNAA  360
QJD09036.1           LQPQKPQVQGQQPAAVQEVTMEQMQPGTIPPTVEGGLGFAPTSKFLLQNGELIYPSSNAA  360
QJD09021.1           LQPQKPQVQGQQPAAVQEVTMEQMQPGTIPPTVEGGLGFAPTSKFLLQNGELIYPSSNAA  360
QJD09016.1           LQPQKPQVQGQQPAAVQEVTMEQMQPGTIPPTVEGGLGFAPTSKFLLQNGELIYPSSNAA  360
QJD09011.1           LQPQKPQVQGQQPAAVQEVTMEQMQPGTIPPTVEGGLGFAPTSKFLLQNGELIYPSSNAA  360
QJD09032.1           LQPQKPQVQGQQPAAVQEVTMEQMQPGTIPPTVEGGLGFAPTSKFLLQNGELIYPSSNAA  360
QJD09026.1           LQPQKPQVQGQQPAAVQEVTMEQMQPGTIPPTVEGGLGFAPTSKFLLQNGELIYPSSNAA  360
AIP98377.1           LQPQKPQVQGQQPAAVQEVTMEQMQPATIPPTVEGGLGFAPTSKFLIQNGELIYPSSNAA  360
ADE45474.1           LQPQKPQVQGQQPAAVQEVTMEQMQPATIPPTVEGGLGFAPTSKFLLQNGELIYPSSNAA  360
YP_003848923.1       LQPQKPQVQGQQPAAVQEVTMEQMQPATIPPTVEGGLGFAPTSKFLIQNGELIYPSSNAA  360
ADE45464.1           LQPQKPQVQGQQPAAVQEVTMEQMQPATIPPTVEGGLGFAPTSKFLIQNGELIYPSSNAA  360
ADE45469.1           LQPQKPQVQGQQPAAVQEVTMEQMQPATIPPTVEGGLGFAPTSKFLIQNGELIYPSSNAA  360
AQM73640.1           LQPQKPQVQGQQPAAVQEVTMEQMQPATIPPTVEGGLGFAPTSKFLIQNGELIYPSSNAA  360
ADE45479.1           LQPQKPQVQGQQPAAVQEVTMEQMQPATIPPTVEGGLGFAPTSKFLIQNGELIYPSSNAA  360
AIP98372.1           LQPQKPQVQGQQPAAVQEVTMEQMQPATIPPTVEGGLGFAPTSKFLLQNGELIYPSSNAA  360
ADE45454.1           LQPQKPQVQGQQPAAVQEVTMEQMQPATIPPTVEGGLGFAPTSKFLLQNGELIYPSSNAA  360
ADE45459.1           LQPQKPQVQGQQPAAVQEVTMEQMQPATIPPTVEGGLGFAPTSKFLLQNGELIYPSSNAA  360
                     **************************.*******************:*************

AXF48237.1           AAAAKISVAPKKNTDNKKEL 380
QJD09036.1           AAAAKISVAPKKNTDKKEL- 379
QJD09021.1           AAAAKISVAPKKNTDKKEL- 379
QJD09016.1           AAAAKISVAPKKNTDKKEL- 379
QJD09011.1           AAAAKISVAPKKNTDKKEL- 379
QJD09032.1           AAAAKISVAPKKNTDKKEL- 379
QJD09026.1           AAAAKISVAPKKNTDKKEL- 379
AIP98377.1           AAAAKISVAPKKNTDKKEL- 379
ADE45474.1           AAAAKISVAPKKNTDKKEL- 379
YP_003848923.1       AAAAKISVAPKKNTDNKKEL 380
ADE45464.1           AAAAKISVAPKKNTDNKKEL 380
ADE45469.1           AAAAKISVAPKKNTDNKKEL 380
AQM73640.1           AAAAKISVAPKKNTDNKKEL 380
ADE45479.1           AAAAKISVAPKKNTDNKKEL 380
AIP98372.1           AAAAKISVAPKKNTDNKKEL 380
ADE45454.1           AAAAKISVAPKKNTDNKKEL 380
ADE45459.1           AAAAKISVAPKKNTDNKKEL 380
```

***************:*:
