# Supplementary material for: The Conserved YPX3L Motif in the BK Polyomavirus VP1 Protein Is Important for Viral Particle Assembly but Not for Its Secretion into Extracellular Vesicles
Source: Viruses. 2024 Jul 13;16(7):1124. doi: 10.3390/v16071124 (PMC11281352; doi:10.3390/v16071124)
Supplement: Supplementary file 1 [file viruses-16-01124-s001.zip › HPyV 08 alignment.pdf]

CLUSTAL O(1.2.4) multiple sequence alignment

```
YP_003800006.1      MAPKRKGEGCARKCPTKTCPTPKPVPKLIMKGNIEVLNLVTGPDSITTIELYLNTRMGQN    60
BBK03547.1          MAPKRKGEGCARKCPTKTCPTPKPVPKLIMKGNIEVLNLVTGPDSITTIELYLNTRMGQN    60
AXN56770.1          MAPKRKGEGCARKCPTKTCPTPKPVPKLIMKGNIEVLNLVTGPDSITTIELYLNTRMGQN    60
APD77417.1          MAPKRKGEGCARKCPTKTCPTPKPVPKLIMKGNIEVLNLVTGPDSITTIELYLNTRMGQN    60
AKE33242.1          MAPKRKGEGCARKCPTKTCPTPKPVPKLIMKGNIEVLNLVTGPDSITTIELYLNTRMGQN    60
AHF96120.1          MAPKRKGEGCARKCPTKTCPTPKPVPKLIMKGNIEVLNLVTGPDSITTIELYLNTRMGQN    60
AHF96126.1          MAPKRKGEGCARKCPTKTCPTPKPVPKLIMKGNIEVLNLVTGPDSITTIELYLNTRMGQN    60
AHF96132.1          MAPKRKGEGCARKCPTKTCPTPKPVPKLIMKGNIEVLNLVTGPDSITTIELYLNTRMGQN    60
AHF96138.1          MAPKRKGEGCARKCPTKTCPTPKPVPKLIMKGNIEVLNLVTGPDSITTIELYLNTRMGQN    60
AHF96144.1          MAPKRKGEGCARKCPTKTCPTPKPVPKLIMKGNIEVLNLVTGPDSITTIELYLNTRMGQN    60
AHF96150.1          MAPKRKGEGCARKCPTKTCPTPKPVPKLIMKGNIEVLNLVTGPDSITTIELYLNTRMGQN    60
AHF96156.1          MAPKRKGEGCARKCPTKTCPTPKPVPKLIMKGNIEVLNLVTGPDSITTIELYLNTRMGQN    60
AHF96162.1          MAPKRKGEGCARKCPTKTCPTPKPVPKLIMKGNIEVLNLVTGPDSITTIELYLNTRMGQN    60
AHF96168.1          MAPKRKGEGCARKCPTKTCPTPKPVPKLIMKGNIEVLNLVTGPDSITTIELYLNTRMGQN    60
AHF96174.1          MAPKRKGEGCARKCPTKTCPTPKPVPKLIMKGNIEVLNLVTGPDSITTIELYLNTRMGQN    60
AHF96180.1          MAPKRKGEGCARKCPTKTCPTPKPVPKLIMKGNIEVLNLVTGPDSITTIELYLNTRMGQN    60
AFN02451.1          MAPKRKGEGCARKCPTKTCPTPKPVPKLIMKGNIEVLNLVTGPDSITTIELYLNTRMGQN    60
ADK12664.1          MAPKRKGEGCARKCPTKTCPTPKPVPKLIMKGNIEVLNLVTGPDSITTIELYLNTRMGQN    60
AVK92959.1          MAPKRKGEGCARKCPTKTCPTPKPVPKLIMKGNIEVLNLVTGPDSITTIELYLNTRMGQN    60
AOA60181.1          MAPKRKGEGCARKCPTKTCPTPKPVPKLIMKGNIEVLNLVTGPDSITTIELYLNTRMGQN    60
AOA60186.1          MAPKRKGEGCARKCPTKTCPTPKPVPKLIMKGNIEVLNLVTGPDSITTIELYLNTRMGQN    60
AOA60191.1          MAPKRKGEGCARKCPTKTCPTPKPVPKLIMKGNIEVLNLVTGPDSITTIELYLNTRMGQN    60
AOA60196.1          MAPKRKGEGCARKCPTKTCPTPKPVPKLIMKGNIEVLNLVTGPDSITTIELYLNTRMGQN    60
AID54934.1          MAPKRKGEGCARKCPTKTCPTPKPVPKLIMKGNIEVLNLVTGPDSITTIELYLNTRMGQN    60
BAO09094.1          MAPKRKGEGCARKCPTKTCPTPKPVPKLIMKGNIEVLNLVTGPDSITTIELYLNTRMGQN    60
                    ************************************************************

YP_003800006.1      DESKDNYGYSEKVTVANSSDQDKPTSGEIPTYSTARINLPMLNEDLTCNTLTMWEAVSVK    120
BBK03547.1          DESKDNYGYSEKVTVANSSDQDKPTSGEIPTYSTARINLPMLNEDLTCNTLTMWEAVSVK    120
AXN56770.1          DESKDNYGYSEKVTVANSSDQDKPTSGEIPTYSTARINLPMLNEDLTCNTLTMWEAVSVK    120
APD77417.1          DESKDNYGYSEKVTVANSSDQDKPTSGEIPTYSTARINLPMLNEDLTCNTLTMWEAVSVK    120
AKE33242.1          DESKDNYGYSEKVTVANSSDQDKPTSGEIPTYSTARINLPMLNEDLTCNTLTMWEAVSVK    120
AHF96120.1          DESKDNYGYSEKVTVANSSDQDKPTSGEIPTYSTARINLPMLNEDLTCNTLTMWEAVSVK    120
AHF96126.1          DESKDNYGYSEKVTVANSSDQDKPTSGEIPTYSTARINLPMLNEDLTCNTLTMWEAVSVK    120
AHF96132.1          DESKDNYGYSEKVTVANSSDQDKPTSGEIPTYSTARINLPMLNEDLTCNTLTMWEAVSVK    120
AHF96138.1          DESKDNYGYSEKVTVANSSDQDKPTSGEIPTYSTARINLPMLNEDLTCNTLTMWEAVSVK    120
AHF96144.1          DESKDNYGYSEKVTVANSSDQDKPTSGEIPTYSTARINLPMLNEDLTCNTLTMWEAVSVK    120
AHF96150.1          DESKDNYGYSEKVTVANSSDQDKPTSGEIPTYSTARINLPMLNEDLTCNTLTMWEAVSVK    120
AHF96156.1          DESKDNYGYSEKVTVANSSDQDKPTSGEIPTYSTARINLPMLNEDLTCNTLTMWEAVSVK    120
AHF96162.1          DESKDNYGYSEKVTVANSSDQDKPTSGEIPTYSTARINLPMLNEDLTCNTLTMWEAVSVK    120
AHF96168.1          DESKDNYGYSEKVTVANSSDQDKPTSGEIPTYSTARINLPMLNEDLTCNTLTMWEAVSVK    120
AHF96174.1          DESKDNYGYSEKVTVANSSDQDKPTSGEIPTYSTARINLPMLNEDLTCNTLTMWEAVSVK    120
AHF96180.1          DESKDNYGYSEKVTVANSSDQDKPTSGEIPTYSTARINLPMLNEDLTCNTLTMWEAVSVK    120
AFN02451.1          DESKDNYGYSEKVTVANSSDQDKPTSGEIPTYSTARINLPMLNEDLTCNTLTMWEAVSVK    120
ADK12664.1          DESKDNYGYSEKVTVANSSDQDKPTSGEIPTYSTARINLPMLNEDLTCNTLTMWEAVSVK    120
AVK92959.1          DESKENYGYSEKVTVANSSDQDKPTSGEIPTYSTARINLPMLNEDLTCNTLTMWEAVSVK    120
AOA60181.1          DESKENYGYSEKVTVANSSDQDKPTSGEIPTYSTARINLPMLNEDLTCNTLTMWEAVSVK    120
AOA60186.1          DESKENYGYSEKVTVANSSDQDKPTSGEIPTYSTARINLPMLNEDLTCNTLTMWEAVSVK    120
AOA60191.1          DESKENYGYSEKVTVANSSDQDKPTSGEIPTYSTARINLPMLNEDLTCNTLTMWEAVSVK    120
AOA60196.1          DESKENYGYSEKVTVANSSDQDKPTSGEIPTYSTARINLPMLNEDLTCNTLTMWEAVSVK    120
AID54934.1          DESKENYGYSEKVTVANSSDQDKPTSGEIPTYSTARINLPMLNEDLTCNTLTMWEAVSVK    120
BAO09094.1          DESKENYGYSEKVTVANSSDQDKPTSGEIPTYSTARINLPMLNEDLTCNTLTMWEAVSVK    120
                    ****:*******************************************************

YP_003800006.1      TEVVGVSSLVNVHMATKRMYDDKGIGFPVEGMNFHMFAVGGEPLELQFLTGNYRTDYSAN    180
BBK03547.1          TEVVGVSSLVNVHMATKRMYDDKGIGFPVEGMNFHMFAVGGEPLELQFLTGNYRTDYSAN    180
AXN56770.1          TEVVGVSSLVNVHMATKRMYDDKGIGFPVEGMNFHMFAVGGEPLELQFLTGNYRTDYSAN    180
APD77417.1          TEVVGVSSLVNVHMATKRMYDDKGIGFPVEGMNFHMFAVGGEPLELQFLTGNYRTDYSAN    180
AKE33242.1          TEVVGVSSLVNVHMATKRMYDDKGIGFPVEGMNFHMFAVGGEPLELQFLTGNYRTDYSAN    180
AHF96120.1          TEVVGVSSLVNVHMATKRMYDDKGIGFPVEGMNFHMFAVGGEPLELQFLTGNYRTDYSAN    180
AHF96126.1          TEVVGVSSLVNVHMATKRMYDDKGIGFPVEGMNFHMFAVGGEPLELQFLTGNYRTDYSAN    180
AHF96132.1          TEVVGVSSLVNVHMATKRMYDDKGIGFPVEGMNFHMFAVGGEPLELQFLTGNYRTDYSAN    180
AHF96138.1          TEVVGVSSLVNVHMATKRMYDDKGIGFPVEGMNFHMFAVGGEPLELQFLTGNYRTDYSAN    180
AHF96144.1          TEVVGVSSLVNVHMATKRMYDDKGIGFPVEGMNFHMFAVGGEPLELQFLTGNYRTDYSAN    180
```

```
AHF96150.1          TEVVGVSSLVNVHMATKRMYDDKGIGFPVEGMNFHMFAVGGEPLELQFLTGNYRTDYSAN  180
AHF96156.1          TEVVGVSSLVNVHMATKRMYDDKGIGFPVEGMNFHMFAVGGEPLELQFLTGNYRTDYSAN  180
AHF96162.1          TEVVGVSSLVNVHMATKRMYDDKGIGFPVEGMNFHMFAVGGEPLELQFLTGNYRTDYSAN  180
AHF96168.1          TEVVGVSSLVNVHMATKRMYDDKGIGFPVEGMNFHMFAVGGEPLELQFLTGNYRTDYSAN  180
AHF96174.1          TEVVGVSSLVNVHMATKRMYDDKGIGFPVEGMNFHMFAVGGEPLELQFLTGNYRTDYSAN  180
AHF96180.1          TEVVGVSSLVNVHMATKRMYDDKGIGFPVEGMNFHMFAVGGEPLELQFLTGNYRTDYSAN  180
AFN02451.1          TEVVGVSSLVNVHMATKRMYDDKGIGFPVEGMNFHMFAVGGEPLELQFLTGNYRTDYSAN  180
ADK12664.1          TEVVGVSSLVNVHMATKRMYDDKGIGFPVEGMNFHMFAVGGEPLELQFLTGNYRTDYSAN  180
AVK92959.1          TEVVGVSSLVNVHMATKRMYDDKGIGFPVEGMNFHMFAVGGEPLELQFLTGNYRTDYSAN  180
AOA60181.1          TEVVGVSSLVNVHMATKRMYDDKGIGFPVEGMNFHMFAVGGEPLELQFLTGNYRTDYSAN  180
AOA60186.1          TEVVGVSSLVNVHMATKRMYDDKGIGFPVEGMNFHMFAVGGEPLELQFLTGNYRTDYSAN  180
AOA60191.1          TEVVGVSSLVNVHMATKRMYDDKGIGFPVEGMNFHMFAVGGEPLELQFLTGNYRTDYSAN  180
AOA60196.1          TEVVGVSSLVNVHMATKRMYDDKGIGFPVEGMNFHMFAVGGEPLELQFLTGNYRTDYSAN  180
AID54934.1          TEVVGVSSLVNVHMATKRMYDDKGIGFPVEGMNFHMFAVGGEPLELQFLTGNYRTDYSAN  180
BAO09094.1          TEVVGVSSLVNVHMATKRMYDDKGIGFPVEGMNFHMFAVGGEPLELQFLTGNYRTDYSAN  180
                    ************************************************************

YP_003800006.1      DKLVVPPIKHQSTQGLNPHYKQKLTKDGAFPVECWCPDPSKNENTRYYGSYTGGQSTPPV  240
BBK03547.1          DKLVVPPIKHQSTQGLNPHYKQKLTKDGAFPVECWCPDPSKNENTRYYGSYTGGQSTPPV  240
AXN56770.1          DKLVVPPIKHQSTQGLNPHYKQKLTKDGAFPVECWCPDPSKNENTRYYGSYTGGQSTPPV  240
APD77417.1          DKLVVPPIKHQSTQGLNPHYKQKLTKDGAFPVECWCPDPSKNENTRYYGSYTGGQSTPPV  240
AKE33242.1          DKLVVPPIKHQSTQGLNPHYKQKLTKDGAFPVECWCPDPSKNENTRYYGSYTGGQSTPPV  240
AHF96120.1          DKLVVPPIKHQSTQGLNPHYKQKLTKDGAFPVECWCPDPSKNENTRYYGSYTGGQSTPPV  240
AHF96126.1          DKLVVPPIKHQSTQGLNPHYKQKLTKDGAFPVECWCPDPSKNENTRYYGSYTGGQSTPPV  240
AHF96132.1          DKLVVPPIKHQSTQGLNPHYKQKLTKDGAFPVECWCPDPSKNENTRYYGSYTGGQSTPPV  240
AHF96138.1          DKLVVPPIKHQSTQGLNPHYKQKLTKDGAFPVECWCPDPSKNENTRYYGSYTGGQSTPPV  240
AHF96144.1          DKLVVPPIKHQSTQGLNPHYKQKLTKDGAFPVECWCPDPSKNENTRYYGSYTGGQSTPPV  240
AHF96150.1          DKLVVPPIKHQSTQGLNPHYKQKLTKDGAFPVECWCPDPSKNENTRYYGSYTGGQSTPPV  240
AHF96156.1          DKLVVPPIKHQSTQGLNPHYKQKLTKDGAFPVECWCPDPSKNENTRYYGSYTGGQSTPPV  240
AHF96162.1          DKLVVPPIKHQSTQGLNPHYKQKLTKDGAFPVECWCPDPSKNENTRYYGSYTGGQSTPPV  240
AHF96168.1          DKLVVPPIKHQSTQGLNPHYKQKLTKDGAFPVECWCPDPSKNENTRYYGSYTGGQSTPPV  240
AHF96174.1          DKLVVPPIKHQSTQGLNPHYKQKLTKDGAFPVECWCPDPSKNENTRYYGSYTGGQSTPPV  240
AHF96180.1          DKLVVPPIKHQSTQGLNPHYKQKLTKDGAFPVECWCPDPSKNENTRYYGSYTGGQSTPPV  240
AFN02451.1          DKLVVPPIKHQSTQGLNPHYKQKLTKDGAFPVECWCPDPSKNENTRYYGSYTGGQSTPPV  240
ADK12664.1          DKLVVPPIKHQSTQGLNPHYKQKLTKDGAFPVECWCPDPSKNENTRYYGSYTGGQSTPPV  240
AVK92959.1          DKLVVPPIKHQSTQGLNPHYKQKLTKDGAFPVECWCPDPSKNENTRYYGSYTGGQSTPPV  240
AOA60181.1          DKLVVPPIKHQSTQGLNPHYKQKLTKDGAFPVECWCPDPSKNENTRYYGSYTGGQSTPPV  240
AOA60186.1          DKLVVPPIKHQSTQGLNPHYKQKLTKDGAFPVECWCPDPSKNENTRYYGSYTGGQSTPPV  240
AOA60191.1          DKLVVPPIKHQSTQGLNPHYKQKLTKDGAFPVECWCPDPSKNENTRYYGSYTGGQSTPPV  240
AOA60196.1          DKLVVPPIKHQSTQGLNPHYKQKLTKDGAFPVECWCPDPSKNENTRYYGSYTGGQSTPPV  240
AID54934.1          DKLVVPPIKHQSTQGLNPHYKQKLTKDGAFPVECWCPDPSKNENTRYYGSYTGGQSTPPV  240
BAO09094.1          DKLVVPPIKHQSTQGLNPHYKQKLTKDGAFPVECWCPDPSKNENTRYYGSYTGGQSTPPV  240
                    ************************************************************

YP_003800006.1      LQFTNTVTTVLLDENGVGPLCKGDGLYVSCCDIVGFLVGKDGDMQYRGLPRYFNILLRKR  300
BBK03547.1          LQFTNTVTTVLLDENGVGPLCKGDGLYVSCCDIVGFLVGKDGDMQYRGLPRYFNILLRKR  300
AXN56770.1          LQFTNTVTTVLLDENGVGPLCKGDGLYVSCCDIVGFLVGKDGDMQYRGLPRYFNILLRKR  300
APD77417.1          LQFTNTVTTVLLDENGVGPLCKGDGLYVSCCDIVGFLVGKDGDMQYRGLPRYFNILLRKR  300
AKE33242.1          LQFTNTVTTVLLDENGVGPLCKGDGLYVSCCDIVGFLVGKDGDMQYRGLPRYFNILLRKR  300
AHF96120.1          LQFTNTVTTVLLDENGVGPLCKGDGLYVSCCDIVGFLVGKDGDMQYRGLPRYFNILLRKR  300
AHF96126.1          LQFTNTVTTVLLDENGVGPLCKGDGLYVSCCDIVGFLVGKDGDMQYRGLPRYFNILLRKR  300
AHF96132.1          LQFTNTVTTVLLDENGVGPLCKGDGLYVSCCDIVGFLVGKDGDMQYRGLPRYFNILLRKR  300
AHF96138.1          LQFTNTVTTVLLDENGVGPLCKGDGLYVSCCDIVGFLVGKDGDMQYRGLPRYFNILLRKR  300
AHF96144.1          LQFTNTVTTVLLDENGVGPLCKGDGLYVSCCDIVGFLVGKDGDMQYRGLPRYFNILLRKR  300
AHF96150.1          LQFTNTVTTVLLDENGVGPLCKGDGLYVSCCDIVGFLVGKDGDMQYRGLPRYFNILLRKR  300
AHF96156.1          LQFTNTVTTVLLDENGVGPLCKGDGLYVSCCDIVGFLVGKDGDMQYRGLPRYFNILLRKR  300
AHF96162.1          LQFTNTVTTVLLDENGVGPLCKGDGLYVSCCDIVGFLVGKDGDMQYRGLPRYFNILLRKR  300
AHF96168.1          LQFTNTVTTVLLDENGVGPLCKGDGLYVSCCDIVGFLVGKDGDMQYRGLPRYFNILLRKR  300
AHF96174.1          LQFTNTVTTVLLDENGVGPLCKGDGLYVSCCDIVGFLVGKDGDMQYRGLPRYFNILLRKR  300
AHF96180.1          LQFTNTVTTVLLDENGVGPLCKGDGLYVSCCDIVGFLVGKDGDMQYRGLPRYFNILLRKR  300
AFN02451.1          LQFTNTVTTVLLDENGVGPLCKGDGLYVSCCDIVGFLVGKDGDMQYRGLPRYFNILLRKR  300
ADK12664.1          LQFTNTVTTVLLDENGVGPLCKGDGLYVSCCDIVGFLVGKDGDMQYRGLPRYFNILLRKR  300
AVK92959.1          LQFTNTVTTVLLDENGVGPLCKGDGLYVSCCDIVGFLVGKDGDMQYRGLPRYFNILLRKR  300
AOA60181.1          LQFTNTVTTVLLDENGVGPLCKGDGLYVSCCDIVGFLVGKDGDMQYRGLPRYFNILLRKR  300
AOA60186.1          LQFTNTVTTVLLDENGVGPLCKGDGLYVSCCDIVGFLVGKDGDMQYRGLPRYFNILLRKR  300
AOA60191.1          LQFTNTVTTVLLDENGVGPLCKGDGLYVSCCDIVGFLVGKDGDMQYRGLPRYFNILLRKR  300
AOA60196.1          LQFTNTVTTVLLDENGVGPLCKGDGLYVSCCDIVGFLVGKDGDMQYRGLPRYFNILLRKR  300
```

```
AID54934.1          LQFTNTVTTVLLDENGVGPLCKGDGLYVSCCDIVGFLVGKDGDMQYRGLPRYFNILLRKR   300
BAO09094.1          LQFTNTVTTVLLDENGVGPLCKGDGLYVSCCDIVGFLVGKDGDMQYRGLPRYFNILLRKR   300
                    ************************************************************

YP_003800006.1      TVRNPYPVSSLLNNLFTGLMPAVQGQPMDNGLSTQVEEVRVYDGTEGLPGDPDMVRYIDK   360
BBK03547.1          TVRNPYPVSSLLNNLFTGLMPAVQGQPMDNGLSTQVEEVRVYDGTEGLPGDPDMVRYIDK   360
AXN56770.1          TVRNPYPVSSLLNNLFTGLMPAVQGQPMDNGLSTQVEEVRVYDGTEGLPGDPDMVRYIDK   360
APD77417.1          TVRNPYPVSSLLNNLFTGLMPAVQGQPMDNGLSTQVEEVRVYDGTEGLPGDPDMVRYIDK   360
AKE33242.1          TVRNPYPVSSLLNNLFTGLMPAVQGQPMDNGLSTQVEEVRVYDGTEGLPGDPDMVRYIDK   360
AHF96120.1          TVRNPYPVSSLLNNLFTGLMPAVQGQPMDNGLSTQVEEVRVYDGTEGLPGDPDMVRYIDK   360
AHF96126.1          TVRNPYPVSSLLNNLFTGLMPAVQGQPMDNGLSTQVEEVRVYDGTEGLPGDPDMVRYIDK   360
AHF96132.1          TVRNPYPVSSLLNNLFTGLMPAVQGQPMDNGLSTQVEEVRVYDGTEGLPGDPDMVRYIDK   360
AHF96138.1          TVRNPYPVSSLLNNLFTGLMPAVQGQPMDNGLSTQVEEVRVYDGTEGLPGDPDMVRYIDK   360
AHF96144.1          TVRNPYPVSSLLNNLFTGLMPAVQGQPMDNGLSTQVEEVRVYDGTEGLPGDPDMVRYIDK   360
AHF96150.1          TVRNPYPVSSLLNNLFTGLMPAVQGQPMDNGLSTQVEEVRVYDGTEGLPGDPDMVRYIDK   360
AHF96156.1          TVRNPYPVSSLLNNLFTGLMPAVQGQPMDNGLSTQVEEVRVYDGTEGLPGDPDMVRYIDK   360
AHF96162.1          TVRNPYPVSSLLNNLFTGLMPAVQGQPMDNGLSTQVEEVRVYDGTEGLPGDPDMVRYIDK   360
AHF96168.1          TVRNPYPVSSLLNNLFTGLMPAVQGQPMDNGLSTQVEEVRVYDGTEGLPGDPDMVRYIDK   360
AHF96174.1          TVRNPYPVSSLLNNLFTGLMPAVQGQPMDNGLSTQVEEVRVYDGTEGLPGDPDMVRYIDK   360
AHF96180.1          TVRNPYPVSSLLNNLFTGLMPAVQGQPMDNGLSTQVEEVRVYDGTEGLPGDPDMVRYIDK   360
AFN02451.1          TVRNPYPVSSLLNNLFTGLMPAVQGQPMDNGLSTQVEEVRVYDGTEGLPGDPDMVRYIDK   360
ADK12664.1          TVRNPYPVSSLLNNLFTGLMPAVQGQPMDNGLSTQVEEVRVYDGTEGLPGDPDMVRYIDK   360
AVK92959.1          TVRNPYPVSSLLNNLFTGLMPAVQGQPMDNGLSTQVEEVRVYDGTEGLPGDPDMVRYIDK   360
AOA60181.1          TVRNPYPVSSLLNNLFTGLMPAVQGQPMDNGLSTQVEEVRVYDGTEGLPGDPDMVRYIDK   360
AOA60186.1          TVRNPYPVSSLLNNLFTGLMPAVQGQPMDNGLSTQVEEVRVYDGTEGLPGDPDMVRYIDK   360
AOA60191.1          TVRNPYPVSSLLNNLFTGLMPAVQGQPMDNGLSTQVEEVRVYDGTEGLPGDPDMVRYIDK   360
AOA60196.1          TVRNPYPVSSLLNNLFTGLMPAVQGQPMDNGLSTQVEEVRVYDGTEGLPGDPDMVRYIDK   360
AID54934.1          TVRNPYPVSSLLNNLFTGLMPAVQGQPMDNGLSTQVEEVRVYDGTEGLPGDPDMVRYIDK   360
BAO09094.1          TVRNPYPVSSLLNNLFTGLMPAVQGQPMDNGLSTQVEEVRVYDGTEGLPGDPDMVRYIDK   360
                    ************************************************************

YP_003800006.1      FGQDKTRPPFPARLY 375
BBK03547.1          FGQDKTRPPFPARLY 375
AXN56770.1          FGQDKTRPPFPARLY 375
APD77417.1          FGQDKTRPPFPARLY 375
AKE33242.1          FGQDKTRPPFPARLY 375
AHF96120.1          FGQDKTRPPFPARLY 375
AHF96126.1          FGQDKTRPPFPARLY 375
AHF96132.1          FGQDKTRPPFPARLY 375
AHF96138.1          FGQDKTRPPFPARLY 375
AHF96144.1          FGQDKTRPPFPARLY 375
AHF96150.1          FGQDKTRPPFPARLY 375
AHF96156.1          FGQDKTRPPFPARLY 375
AHF96162.1          FGQDKTRPPFPARLY 375
AHF96168.1          FGQDKTRPPFPARLY 375
AHF96174.1          FGQDKTRPPFPARLY 375
AHF96180.1          FGQDKTRPPFPARLY 375
AFN02451.1          FGQDKTRPPFPARLY 375
ADK12664.1          FGQDKTRPPFPARLY 375
AVK92959.1          FGQDKTRPPFPARLY 375
AOA60181.1          FGQDKTRPPFPARLY 375
AOA60186.1          FGQDKTRPPFPARLY 375
AOA60191.1          FGQDKTRPPFPARLY 375
AOA60196.1          FGQDKTRPPFPARLY 375
AID54934.1          FGQDKTRPPFPARLY 375
BAO09094.1          FGQDKTRPPFPARLY 375
                    ***************
```
