# Supplementary material for: The Conserved YPX3L Motif in the BK Polyomavirus VP1 Protein Is Important for Viral Particle Assembly but Not for Its Secretion into Extracellular Vesicles
Source: Viruses. 2024 Jul 13;16(7):1124. doi: 10.3390/v16071124 (PMC11281352; doi:10.3390/v16071124)
Supplement: Supplementary file 1 [file viruses-16-01124-s001.zip › HPyV 09 alignment.pdf]

CLUSTAL O(1.2.4) multiple sequence alignment

```
YP_004243705.1      MAPQRKRQECGACPVKKTCPTPAPVPKLLVKGGVEVLEVRTGPDAITQIEAYLNPRMGNN   60
UMW88369.1          MAPQRKRQECGACPVKKTCPTPAPVPKLLVKGGVEVLEVRTGPDAITQIEAYLNPRMGNN   60
QWS67644.1          MAPQRKRQECGACPVKKTCPTPAPVPKLLVKGGVEVLEVRTGPDAITQIEAYLNPRMGNN   60
QWS67649.1          MAPQRKRQECGACPVKKTCPTPAPVPKLLVKGGVEVLEVRTGPDAITQIEAYLNPRMGNN   60
QCB66181.1          MAPQRKRQECGACPVKKTCPTPAPVPKLLVKGGVEVLEVRTGPDAITQIEAYLNPRMGNN   60
AGO04554.1          MAPQRKRQECGACPVKKTCPTPAPVPKLLVKGGVEVLEVRTGPDAITQIEAYLNPRMGNN   60
CBZ41798.1          MAPQRKRQECGACPVKKTCPTPAPVPKLLVKGGVEVLEVRTGPDAITQIEAYLNPRMGNN   60
ADV15630.1          MAPQRKRQECGACPVKKTCPTPAPVPKLLVKGGVEVLEVRTGPDAITQIEAYLNPRMGNN   60
                    ************************************************************

YP_004243705.1      NPTDELYGYSADINVASSKASDNPNATTLPTYSVAVIKLPMLNEDMTCDTLLMWEAVSVK   120
UMW88369.1          NPTDELYGYSADINVASSKASDNPNATTLPTYSVAVIKLPMLNEDMTCDTLLMWEAVSVK   120
QWS67644.1          NPTDELYGYSADINVASSKASDNPNATTLPTYSVAVIKLPMLNEDMTCDTLLMWEAVSVK   120
QWS67649.1          NPTDELYGYSADINVASSKASDNPNATTLPTYSVAVIKLPMLNEDMTCDTLLMWEAVSVK   120
QCB66181.1          NPTDELYGYSADINVASSKASDNPNATTLPTYSVAVIKLPMLNEDMTCDTLLMWEAVSVK   120
AGO04554.1          NPTDELYGYSADINVASSKASDNPNATTLPTYSVAVIKLPMLNEDMTCDTLLMWEAVSVK   120
CBZ41798.1          NPTDELYGYSADINVASSKASDNPNATTLPTYSVAVIKLPMLNEDMTCDTLLMWEAVSVK   120
ADV15630.1          NPTDELYGYSADINVASSKASDNPNATTLPTYSVAVIKLPMLNEDMTCDTLLMWEAVSVK   120
                    ************************************************************

YP_004243705.1      TEVMGISSLVNLHQGGKYIYGSSSGTIPVQGTTLHMFSVGGEPLELQGLVASSTTTYPTD   180
UMW88369.1          TEVMGISSLVNLHQGGKYIYGSSSGTIPVQGTTLHMFSVGGEPLELQGLVASSTTTYPTD   180
QWS67644.1          TEVMGISSLVNLHQGGKYIYGSSSGTIPVQGTTLHMFSVGGEPLELQGLVASSTTTYPTD   180
QWS67649.1          TEVMGISSLVNLHQGGKYIYGSSSGTIPVQGTTLHMFSVGGEPLELQGLVASSTTTYPTD   180
QCB66181.1          TEVMGISSLVNLHQGGKYIYGSSSGTIPVQGTTLHMFSVGGEPLELQGLVASSTTTYPTD   180
AGO04554.1          TEVMGISSLVNLHQGGKYIYGSSSGTIPVQGTTLHMFSVGGEPLELQGLVASSTTTYPTD   180
CBZ41798.1          TEVMGISSLVNLHQGGKYIYGSSSGTIPVQGTTLHMFSVGGEPLELQGLVASSTTTYPTD   180
ADV15630.1          TEVMGISSLVNLHQGGKYIYGSSSGTIPVQGTTLHMFSVGGEPLELQGLVASSTTTYPTD   180
                    ************************************************************

YP_004243705.1      MVTIKNMKPVNQALDPNAKALLDKDGKYPVEVWSPDPSKNENTRYYGSFTGGATTPPVMQ   240
UMW88369.1          MVTIKNMKPVNQALDPNAKALLDKDGKYPVEVWSPDPSKNENTRYYGSFTGGATTPPVMQ   240
QWS67644.1          MVTIKNMKPVNQALDPNAKALLDKDGKYPVEVWSPDPSKNENTRYYGSFTGGATTPPVMQ   240
QWS67649.1          MVTIKNMKPVNQALDPNAKALLDKDGKYPVEVWSPDPSKNENTRYYGSFTGGATTPPVMQ   240
QCB66181.1          MVTIKNMKPVNQALDPNAKALLDKDGKYPVEVWSPDPSKNENTRYYGSFTGGATTPPVMQ   240
AGO04554.1          MVTIKNMKPVNQALDPNAKALLDKDGKYPVEVWSPDPSKNENTRYYGSFTGGATTPPVMQ   240
CBZ41798.1          MVTIKNMKPVNQALDPNAKALLDKDGKYPVEVWSPDPSKNENTRYYGSFTGGATTPPVMQ   240
ADV15630.1          MVTIKNMKPVNQALDPNAKALLDKDGKYPVEVWSPDPSKNENTRYYGSFTGGATTPPVMQ   240
                    ************************************************************

YP_004243705.1      FTNSVTTVLLDENGVGPLCKGDKLFLSAVDIVGIHTNYSESQNWRGLPRYFNVTLRKRVV   300
UMW88369.1          FTNSVTTVLLDENGVGPLCKGDKLFLSAVDIVGIHTNYSESQNWRGLPRYFNVTLRKRVV   300
QWS67644.1          FTNSVTTVLLDENGVGPLCKGDKLFLSAVDIVGIHTNYSESQNWRGLPRYFNVTLRKRVV   300
QWS67649.1          FTNSVTTVLLDENGVGPLCKGDKLFLSAVDIVGIHTNYSESQNWRGLPRYFNVTLRKRVV   300
QCB66181.1          FTNSVTTVLLDENGVGPLCKGDKLFLSAVDIVGIHTNYSESQNWRGLPRYFNVTLRKRVV   300
AGO04554.1          FTNSVTTVLLDENGVGPLCKGDKLFLSAVDIVGIHTNYSESQNWRGLPRYFNVTLRKRVV   300
CBZ41798.1          FTNSVTTVLLDENGVGPLCKGDKLFLSAVDIVGIHTNYSESQNWRGLPRYFNVTLRKRVV   300
ADV15630.1          FTNSVTTVLLDENGVGPLCKGDKLFLSAVDIVGIHTNYSESQNWRGLPRYFNVTLRKRVV   300
                    ************************************************************

YP_004243705.1      KNPYPVSSLLNSLFSGLMPQIQGQPMEGTSGQVEEVRIYQGTEGLPGDPDLDRYVDKFCQ   360
UMW88369.1          KNPYPVSSLLNSLFSGLMPQIQGQPMEGTSGQVEEVRIYQGTEGLPGDPDLDRYVDKFCQ   360
QWS67644.1          KNPYPVSSLLNSLFSGLMPQIQGQPMEGTSGQVEEVRIYQGTEGLPGDPDLDRYVDKFCQ   360
QWS67649.1          KNPYPVSSLLNSLFSGLMPQIQGQPMEGTSGQVEEVRIYQGTEGLPGDPDLDRYVDKFCQ   360
QCB66181.1          KNPYPVSSLLNSLFSGLMPQIQGQPMEGTSGQVEEVRIYQGTEGLPGDPDLDRYVDKFCQ   360
AGO04554.1          KNPYPVSSLLNSLFSGLMPQIQGQPMEGTSGQVEEVRIYQGTEGLPGDPDLDRYVDKFCQ   360
CBZ41798.1          KNPYPVSSLLNSLFSGLMPQIQGQPMEGTSGQVEEVRIYQGTEGLPGDPDLDRYVDKFCQ   360
ADV15630.1          KNPYPVSSLLNSLFSGLMPQIQGQPMEGTSGQVEEVRIYQGTEGLPGDPDLDRYVDKFCQ   360
                    ************************************************************

YP_004243705.1      NQTVPPRSNDQ     371
UMW88369.1          NQTVPPRSNDQ     371
QWS67644.1          NQTVPPRSNDQ     371
QWS67649.1          NQTVPPRSNDQ     371
```

```
QCB66181.1          NQTVPPRSNDQ    371
AGO04554.1          NQTVPPRSNDQ    371
CBZ41798.1          NQTVPPRSNDQ    371
ADV15630.1          NQTVPPRSNDQ    371
                    ***********
```
