# Supplementary material for: The Conserved YPX3L Motif in the BK Polyomavirus VP1 Protein Is Important for Viral Particle Assembly but Not for Its Secretion into Extracellular Vesicles
Source: Viruses. 2024 Jul 13;16(7):1124. doi: 10.3390/v16071124 (PMC11281352; doi:10.3390/v16071124)
Supplement: Supplementary file 1 [file viruses-16-01124-s001.zip › HPyV 10 alignment.pdf]

CLUSTAL O(1.2.4) multiple sequence alignment

```
AHB33020.1          MPPKRKTVCTKTVCTRAEAPNKKVCEKPSCSRSCRMSCNKCPCIPCPVPTKVPRIVSKGG	60
ASU87489.1          MPPKRKTVCTKTVCTRAEAPNKKVCEKPTCSRSCRMSCNKCPCIPCPVPTKVPRIVSKGG	60
AHF70972.1          MPPKRKTVCTKTVCTRAEAPNKKVCEKPTCSRSCRMSCNKCPCIPCPVPTKVPRIVSKGG	60
AGL07627.1          MPPKRKTVCTKTVCTRAEAPNKKVCEKPTCSRSCRMSCNKCPCIPCPVPTKVPRIVSKGG	60
AGL07632.1          MPPKRKTVCTKTVCTRAEAPNKKVCEKPTCSRSCRMSCNKCPCIPCPVPTKVPRIVSKGG	60
AGL07637.1          MPPKRKTVCTKTVCTRAEAPNKKVCEKPTCSRSCRMSCNKCPCIPCPVPTKVPRIVSKGG	60
AGL07642.1          MPPKRKTVCTKTVCTRAEAPNKKVCEKPTCSRSCRMSCNKCPCIPCPVPTKVPRIVSKGG	60
AGL07647.1          MPPKRKTVCTKTVCTRAEAPNKKVCEKPTCSRSCRMSCNKCPCIPCPVPTKVPRIVSKGG	60
AGL07657.1          MPPKRKTVCTKTVCTRAEAPNKKVCEKPTCSRSCRMSCNKCPCIPCPVPTKVPRIVSKGG	60
AGL07662.1          MPPKRKTVCTKTVCTRAEAPNKKVCEKPTCSRSCRMSCNKCPCIPCPVPTKVPRIVSKGG	60
AGL07667.1          MPPKRKTVCTKTVCTRAEAPNKKVCEKPTCSRSCRMSCNKCPCIPCPVPTKVPRIVSKGG	60
AFN43006.1          MPPKRKTVCTKTVCTRAEAPNKKVCEKPTCSRSCRMSCNKCPCIPCPVPTKVPRIVSKGG	60
AFN02459.1          MPPKRKTVCTKTVCTRAEAPNKKVCEKPTCSRSCRMSCNKCPCIPCPVPTKVPRIVSKGG	60
AGL07652.1          MPPKRKTVCTKTVCTRAEAPNKKVCEKPTCSRSCRMSCNKCPGIPCPVPTKVPRIVSKGG	60
AHB33045.1          MPTKRKTVCTKTVCTRAEAPNKKVCEKPSCSRSCRMSCNKCPCIPCPTPTKVPRIVSKGG	60
YP_006495788.1      MPTKRKTVCTKTVCTRADAPNKKVCEKPSCSRSCRMSCNKCPCIPCPVPTKVPRIVSKGG	60
WHU50308.1          MPTKRKTVCTKTVCTRADAPNKKVCEKPSCSRSCRMSCNKCPCIPCPVPTKVPRIVSKGG	60
AFN02454.1          MPTKRKTVCTKTVCTRADAPNKKVCEKPSCSRSCRMSCNKCPCIPCPVPTKVPRIVSKGG	60
AHB33025.1          MPTKRKTVCTKTVCTRAEAPNKKVCQKPSCSRSCRMSCNKCPCIPCPVPTKVPRIVSKGG	60
ASU87490.1          MPTKRKTVCTKTVCTRAEAPNKKVCQKVSCSRSCRMSCNKCPCIPCPVPTKVPRIVSKGG	60
ASU87491.1          MPTKRKTVCTKTVCTRAEAPNKKVCQKASCSRSCRMSCNKCPCIPCPVPTKVPRIVSKGG	60
ASU87492.1          MPTKRKTVCTKTVCTRAEAPNKKVCQKPSCSRSCRMSCNKCPCIPCPVPTKVPRIVSKGG	60
AHB33030.1          MPTKRKTVCTKTVCTRAEAPNKKVCQKPSCSRSCRMSCNKCPCIPCPVPTKVPRIVSKGG	60
AHB33035.1          MPTKRKTVCTKTVCTRAEAPNKKVCQKPSCSRSCRMSCNKCPCIPCPVPTKVPRIVSKGG	60
AHB33040.1          MPTKRKTVCTKTVCTRAEAPNKKVCQKPSCSRSCRMSCNKCPCIPCPVPTKVPRIVSKGG	60
WHU50311.1          MPTKRKTVCTKTVCTRAEAPNKKVCEKPSCSRSCRMSCNKCPSIPCPVPTKVPRIVSKGG	60
AKI28601.1          MPTKRKTVCTKTVCTRSEAPNKKMCEKPSCSRSCRMSCNKCPCIPCPVPSKVPRIVSKGG	60
                    ** *************::*****:*:* :************* ****.*:**********

AHB33020.1          IDVLNIIPGPDTTMTVEVILQPRMGNDVKTNKWYGYSDPITVTNTPNINQLPTYSTAKIK	120
ASU87489.1          IEVLNIIPGPDTTMTVEVILQPRMGNDVKTNKWYGYSDPITVTNTPGINQLPTYSTAKIK	120
AHF70972.1          IEVLNIIPGPDTTMTVEVILQPRMGNDVKTNKWYGYSDPITVTNTPGINQLPTYSTAKIK	120
AGL07627.1          IEVLNIIPGPDTTMTVEVILQPRMGNDVKTNKWYGYSDPITVTNTPGINQLPTYSTAKIK	120
AGL07632.1          IEVLNIIPGPDTTMTVEVILQPRMGNDVKTNKWYGYSDPITVTNTPGINQLPTYSTAKIK	120
AGL07637.1          IEVLNIIPGPDTTMTVEVILQPRMGNDVKTNKWYGYSDPITVTNTPGINQLPTYSTAKIK	120
AGL07642.1          IEVLNIIPGPDTTMTVEVILQPRMGNDVKTNKWYGYSDPITVTNTPGINQLPTYSTAKIK	120
AGL07647.1          IEVLNIIPGPDTTMTVEVILQPRMGNDVKTNKWYGYSDPITVTNTPGINQLPTYSTAKIK	120
AGL07657.1          IEVLNIIPGPDTTMTVEVILQPRMGNDVKTNKWYGYSDPITVTNTPGINQLPTYSTAKIK	120
AGL07662.1          IEVLNIIPGPDTTMTVEVILQPRMGNDVKTNKWYGYSDPITVTNTPGINQLPTYSTAKIK	120
AGL07667.1          IEVLNIIPGPDTTMTVEVILQPRMGNDVKTNKWYGYSDPITVTNTPGINQLPTYSTAKIK	120
AFN43006.1          IEVLNIIPGPDTTMTVEVILQPRMGNDVKTNKWYGYSDPITVTNTPGINQLPTYSTAKIK	120
AFN02459.1          IEVLNIIPGPDTTMTVEVILQPRMGNDVKTNKWYGYSDPITVTNTPGINQLPTYSTAKIK	120
AGL07652.1          IEVLNIIPGPDTTMTVEVILQPRMGNDVKTNKWYGYSDPITVTNTPGINQLPTYSTAKIK	120
AHB33045.1          IDVLNIIPGPDTTMTVEVILQPRMGNDVKTNKWYGYSDPITVTNTPTINQLPTYSTAKIK	120
YP_006495788.1      IDVLNIIPGPDTTMTVEVILQPRMGNDVKTNKWYGYSDPITVTNTPTINQLPTYSTAKIK	120
WHU50308.1          IDVLNIIPGPDTTMTVEVILQPRMGNDVKTNKWYGYSDPITVTNTPTINQLPTYSTAKIK	120
AFN02454.1          IDVLNIIPGPDTTMTVEVILQPRMGNDVKTNKWYGYSDPITVTNTPTINQLPTYSTAKIK	120
AHB33025.1          IDVLNIIPGPDTTMTVEVILQPRMGNDVKTNKWYGYSDPITVTNTPNINQLPTYSTAKIK	120
ASU87490.1          IDVLNIIPGPDTTMTVEVILQPRMGNDVKTNKWYGYSDPITVTNTPTINQLPTYSTAKIK	120
ASU87491.1          IDVLNIIPGPDTTMTVEVILQPRMGNDVKTNKWYGYSDPITVTNTPTINQLPTYSTAKIK	120
ASU87492.1          IDVLNIIPGPDTTMTVEVILQPRMGNDVKTNKWYGYSDPITVTNTPTINQLPTYSTAKIK	120
AHB33030.1          IDVLNIIPGPDTTMTVEVILQPRMGNDVKTNKWYGYSDPITVTNTPTINQLPTYSTAKIK	120
AHB33035.1          IDVLNIIPGPDTTMTVEVILQPRMGNDVKTNKWYGYSDPITVTNTPTINQLPTYSTAKIK	120
AHB33040.1          IDVLNIIPGPDTTMTVEVILQPRMGNDVKTNKWYGYSDPITVTNTPTINQLPTYSTAKIK	120
WHU50311.1          IDVLNIIPGPDTTMTVEVILQPRMGNDVKTNKWYGYSDPITVTNNPLINQLPTYSTAKIK	120
AKI28601.1          IDVLNIIPGPDTTMTVEVILQPRMGNDVKTNKWYGYSDPITVSNNPPINQLPTYSTAKIK	120
                    *:****************************************:*.* *************

AHB33020.1          LPPLNEDMTCENLYMWEAVVLKTELIGVTSLITLHTPGVADPATAPALPVEGVSFHFFAV	180
ASU87489.1          LPPLNEDMTCENLYMWEAVVLKTELIGVTSLITLHTPGVADPATAPALPVEGVSFHFFAV	180
AHF70972.1          LPPLNEDMTCENLYMWEAVVLKTELIGVTSLITLHTPGVADPATAPALPVEGVSFHFFAV	180
AGL07627.1          LPPLNEDMTCENLYMWEAVVLKTELIGVTSLITLHTPGVADPATAPALPVEGVSFHFFAV	180
AGL07632.1          LPPLNEDMTCENLYMWEAVVLKTELIGVTSLITLHTPGVADPATAPALPVEGVSFHFFAV	180
AGL07637.1          LPPLNEDMTCENLYMWEAVVLKTELIGVTSLITLHTPGVADPATAPALPVEGVSFHFFAV	180
```

```
AGL07642.1          LPPLNEDMTCENLYMWEAVVLKTELIGVTSLITLHTPGVADPATAPALPVEGVSFHFFAV  180
AGL07647.1          LPPLNEDMTCENLYMWEAVVLKTELIGVTSLITLHTPGVADPATAPALPVEGVSFHFFAV  180
AGL07657.1          LPPLNEDMTCENLYMWEAVVLKTELIGVTSLITLHTPGVADPATAPALPVEGVSFHFFAV  180
AGL07662.1          LPPLNEDMTCENLYMWEAVVLKTELIGVTSLITLHTPGVADPATAPALPVEGVSFHFFAV  180
AGL07667.1          LPPLNEDMTCENLYMWEAVVLKTELIGVTSLITLHTPGVADPATAPALPVEGVSFHFFAV  180
AFN43006.1          LPPLNEDMTCENLYMWEAVVLKTELIGVTSLITLHTPGVADPATAPALPVEGVSFHFFAV  180
AFN02459.1          LPPLNEDMTCENLYMWEAVVLKTELIGVTSLITLHTPGVADPATAPALPVEGVSFHFFAV  180
AGL07652.1          LPPLNEDMTCENLYMWEAVVLKTELIGVTSLITLHTPGVADPATAPALPVEGVSFHFFAV  180
AHB33045.1          LPPLNEDMTCENLYMWEAVVLKTELIGVTSLITLHTPGVADPATAPALPVEGVSFHFFAV  180
YP_006495788.1      LPPLNEDMTCENLYMWEAVVLKTELIGVTSLITLHTPGVADPATAPALPVEGVSFHFFAV  180
WHU50308.1          LPPLNEDMTCENLYMWEAVVLKTELIGVTSLITLHTPGVADPATAPALPVEGVSFHFFAV  180
AFN02454.1          LPPLNEDMTCENLYMWEAVVLKTELIGVTSLITLHTPGVADPATAPALPVEGVSFHFFAV  180
AHB33025.1          LPALNEDMTCENLYMWEAVVLKTELIGVTSLITLHTPGVADPATAPALPVEGVSFHFFAV  180
ASU87490.1          LPALNEDMTCENLYMWEAVVLKTELIGVTSLITLHTPGVADPATAPALPVEGVSFHFFAV  180
ASU87491.1          LPALNEDMTCENLYMWEAVVLKTELIGVTSLITLHTPGVADPATAPALPVEGVSFHFFAV  180
ASU87492.1          LPALNEDMTCENLYMWEAVVLKTELIGVTSLITLHTPGVADPATAPALPVEGVSFHFFAV  180
AHB33030.1          LPALNEDMTCENLYMWEAVVLKTELIGVTSLITLHTPGVADPATAPALPVEGVSFHFFAV  180
AHB33035.1          LPALNEDMTCENLYMWEAVVLKTELIGVTSLITLHTPGVADPATAPALPVEGVSFHFFAV  180
AHB33040.1          LPALNEDMTCENLYMWEAVVLKTELIGVTSLITLHTPGVADPATAPALPVEGVSFHFFAV  180
WHU50311.1          LPPLNEDMTCENLYMWEAVVLKTELIGVTSLLTLHTPGVADPATAPALPVEGVSFHFFAV  180
AKI28601.1          LPSLNEDMTCENLYMWEAVVLKTELIGVTSLITLHTPGVADPATAPALPVEGVSFHFFAV  180
                    ** ****************************:****************************

AHB33020.1          GGQPLDLQYCVPAADIVYPDGTGSFASSGGTQQTYDASFKAILDRDGYYPVEAWAPDPVK  240
ASU87489.1          GGQPLDLQYCVPAADIVYPDGTGSFASSGGTQQTYDASFKAILDRDGYYPVEAWAPDPVK  240
AHF70972.1          GGQPLDLQYCVPAADIVYPDGTGSFASSGGTQQTYDASFKAILDRDGYYPVEAWAPDPVK  240
AGL07627.1          GGQPLDLQYCVPAADIVYPDGTGSFASSGGTQQTYDASFKAILDRDGYYPVEAWAPDPVK  240
AGL07632.1          GGQPLDLQYCVPAADIVYPDGTGSFASSGGTQQTYDASFKAILDRDGYYPVEAWAPDPVK  240
AGL07637.1          GGQPLDLQYCVPAADIVYPDGTGSFASSGGTQQTYDASFKAILDRDGYYPVEAWAPDPVK  240
AGL07642.1          GGQPLDLQYCVPAADIVYPDGTGSFASSGGTQQTYDASFKAILDRDGYYPVEAWAPDPVK  240
AGL07647.1          GGQPLDLQYCVPAADIVYPDGTGSFASSGGTQQTYDASFKAILDRDGYYPVEAWAPDPVK  240
AGL07657.1          GGQPLDLQYCVPAADIVYPDGTGSFASSGGTQQTYDASFKAILDRDGYYPVEAWAPDPVK  240
AGL07662.1          GGQPLDLQYCVPAADIVYPDGTGSFASSGGTQQTYDASFKAILDRDGYYPVEAWAPDPVK  240
AGL07667.1          GGQPLDLQYCVPAADIVYPDGTGSFASSGGTQQTYDASFKAILDRDGYYPVEAWAPDPVK  240
AFN43006.1          GGQPLDLQYCVPAADIVYPDGTGSFASSGGTQQTYDASFKAILDRDGYYPVEAWAPDPVK  240
AFN02459.1          GGQPLDLQYCVPAADIVYPDGTGSFASSGGTQQTYDASFKAILDRDGYYPVEAWAPDPVK  240
AGL07652.1          GGQPLDLQYCVPAADIVYPDGTGSFASSGGTQQTYDASFKAILDRDGYYPVEAWAPDPVK  240
AHB33045.1          GGQPLDLQYCVPAADNVYPDGTGSFASSGGTQQTYDASFKAILDRDGYYPVEAWAPDPVK  240
YP_006495788.1      GGQPLDLQYCVPAADIVYPDGTGSFASSGGTQQTYDASLKAILDRDGYYPVEAWGPDPVK  240
WHU50308.1          GGQPLDLQYCVPAADIVYPDGTGSFASSGGTQQTYDASLKAILDRDGYYPVEAWGPDPVK  240
AFN02454.1          GGQPLDLQYCVPAADIVYPDGTGSFASSGGTQQTYDASLKAILDRDGYYPVEAWGPDPVK  240
AHB33025.1          GGQPLDLQYCVPAADIVYPDGTGSFASSGGTQQTYDASFKAILDRDGYYPVEAWAPDPVK  240
ASU87490.1          GGQPLDLQYCVPAADIVYPDGTGSFASSGGTQQTYDASFKAILDRDGYYPVEAWAPDPVK  240
ASU87491.1          GGQPLDLQYCVPAADIVYPDGTGSFASSGGTQQTYDASFKAILDRDGYYPVEAWAPDPVK  240
ASU87492.1          GGQPLDLQYCVPAADIVYPDGTGSFASSGGTQQTYDASFKAILDRDGYYPVEAWAPDPVK  240
AHB33030.1          GGQPLDLQYCVPAADIVYPDGTGSFASSGGTQQTYDASFKAILDRDGYYPVEAWAPDPVK  240
AHB33035.1          GGQPLDLQYCVPAADIVYPDGTGSFASSGGTQQTYDASFKAILDRDGYYPVEAWAPDPVK  240
AHB33040.1          GGQPLDLQYCVPAADIVYPDGTGSFASSGGTQQTYDASFKAILDRDGYYPVEAWAPDPVK  240
WHU50311.1          GGQPLDLQYCVPTGEIVYPDGTGSFASSGGTQQTYDASFKAILDRDGYYPVEAWAPDPVK  240
AKI28601.1          GGQPLDLQYCVPTGDIVYPDGTGSFASSGGTQQTYDASFKAILDRDGYYPVEAWAPDPVK  240
                    ************:.: **********************:***************.*****

AHB33020.1          NDNSRYYGTVTGGTTTPPVLSTTNAVSTVLLDDRGVGPLCKGDGLYVTAVDICGVFQMPD  300
ASU87489.1          NDNSRYYGTVTGGTTTPPVLSTTNAVSTVLLDDRGVGPLCKGDGLYVTAVDICGVFQMPD  300
AHF70972.1          NDNSRYYGTVTGGTTTPPVLSTTNAVSTVLLDDRGVGPLCKGDGLYVTAVDICGVFQMPD  300
AGL07627.1          NDNSRYYGTVTGGTTTPPVLSTTNAVSTVLLDDRGVGPLCKGDGLYVTAVDICGVFQMPD  300
AGL07632.1          NDNSRYYGTVTGGTTTPPVLSTTNAVSTVLLDDRGVGPLCKGDGLYVTAVDICGVFQMPD  300
AGL07637.1          NDNSRYYGTVTGGTTTPPVLSTTNAVSTVLLDDRGVGPLCKGDGLYVTAVDICGVFQMPD  300
AGL07642.1          NDNSRYYGTVTGGTTTPPVLSTTNAVSTVLLDDRGVGPLCKGDGLYVTAVDICGVFQMPD  300
AGL07647.1          NDNSRYYGTVTGGTTTPPVLSTTNAVSTVLLDDRGVGPLCKGDGLYVTAVDICGVFQMPD  300
AGL07657.1          NDNSRYYGTVTGGTTTPPVLSTTNAVSTVLLDDRGVGPLCKGDGLYVTAVDICGVFQMPD  300
AGL07662.1          NDNSRYYGTVTGGTTTPPVLSTTNAVSTVLLDDRGVGPLCKGDGLYVTAVDICGVFQMPD  300
AGL07667.1          NDNSRYYGTVTGGTTTPPVLSTTNAVSTVLLDDRGVGPLCKGDGLYVTAVDICGVFQMPD  300
AFN43006.1          NDNSRYYGTVTGGTTTPPVLSTTNAVSTVLLDDRGVGPLCKGDGLYVTAVDICGVFQMPD  300
AFN02459.1          NDNSRYYGTVTGGTTTPPVLSTTNAVSTVLLDDRGVGPLCKGDGLYVTAVDICGVFQMPD  300
AGL07652.1          NDNSRYYGTVTGGTTTPPVLSTTNAVSTVLLDDRGVGPLCKGDGLYVTAVDICGVFQMPD  300
AHB33045.1          NDNSRYYGTVTGGTTTPPVLTTTNAVSTVLLDDRGVGPLCKGDGLYVTAVDICGVFQMPD  300
```

```
YP_006495788.1      NDNSRYYGTVTGGTTTPPVLSTTNAVSTVLLDDRGVGPLCKGDGLYVTAVDICGVFQMPD   300
WHU50308.1          NDNSRYYGTVTGGTTTPPVLSTTNAVSTVLLDDRGVGPLCKGDGLYVTAVDICGVFQMPD   300
AFN02454.1          NDNSRYYGTVTGGTTTPPVLSTTNAVSTVLLDDRGVGPLCKGDGLYVTAVDICGVFQMPD   300
AHB33025.1          NDNSRYYGTVTGGTTTPPVLSTTNAVSTVLLDDRGVGPLCKGDGLYVTAVDICGVFQMPD   300
ASU87490.1          NDNSRYYGTVTGGTTTPPVLSTTNAVSTVLLDDRGVGPLCKGDGLYVTAVDICGVFQMPD   300
ASU87491.1          NDNSRYYGTVTGGTTTPPVLSTTNAVSTVLLDDRGVGPLCKGDGLYVTAVDICGVFQMPD   300
ASU87492.1          NDNSRYYGTVTGGTTTPPVLSTTNAVSTVLLDDRGVGPLCKGDGLYVTAVDICGVFQMPD   300
AHB33030.1          NDNSRYYGTVTGGTTTPPVLSTTNAVSTVLLDDRGVGPLCKGDGLYVTAVDICGVFQMPD   300
AHB33035.1          NDNSRYYGTVTGGTTTPPVLSTTNAVSTVLLDDRGVGPLCKGDGLYVTAVDICGVFQMPD   300
AHB33040.1          NDNSRYYGTVTGGTTTPPVLSTTNAVSTVLLDDRGVGPLCKGDGLYVTAVDICGVFQMPD   300
WHU50311.1          NDNSRYYGTVTGGTTTPPVLTTTNAVSTVLLDDRGVGPLCKGDGLYVTAVDICGVFQTAE   300
AKI28601.1          NDNSRYYGTVTGGTTTPPVLTTTNAVSTVLLDDRGVGPLCKGDGLYVTAVDICGVFQTAD   300
                    ********************:************************************  :

AHB33020.1          NTRRHRGLARYFQVQLRQRAVRNPYPVNSLLNSLLTKQIPSIDGQPMDGTDNQVQDVTVF   360
ASU87489.1          NTRRHRGLARYFQVQLRQRAVRNPYPVNSLLNSLLTKQIPSIDGQPMDGTDNQVQDVTVF   360
AHF70972.1          NTRRHRGLARYFQVQLRQRAVRNPYPVNSLLNSLLTKQIPSIDGQPMDGTDNQVQDVTVF   360
AGL07627.1          NTRRHRGLARYFQVQLRQRAVRNPYPVNSLLNSLLTKQIPSIDGQPMDGTDNQVQDVTVF   360
AGL07632.1          NTRRHRGLARYFQVQLRQRAVRNPYPVNSLLNSLLTKQIPSIDGQPMDGTDNQVQDVTVF   360
AGL07637.1          NTRRHRGLARYFQVQLRQRAVRNPYPVNSLLNSLLTKQIPSIDGQPMDGTDNQVQDVTVF   360
AGL07642.1          NTRRHRGLARYFQVQLRQRAVRNPYPVNSLLNSLLTKQIPSIDGQPMDGTDNQVQDVTVF   360
AGL07647.1          NTRRHRGLARYFQVQLRQRAVRNPYPVNSLLNSLLTKQIPSIDGQPMDGTDNQVQDVTVF   360
AGL07657.1          NTRRHRGLARYFQVQLRQRAVRNPYPVNSLLNSLLTKQIPSIDGQPMDGTDNQVQDVTVF   360
AGL07662.1          NTRRHRGLARYFQVQLRQRAVRNPYPVNSLLNSLLTKQIPSIDGQPMDGTDNQVQDVTVF   360
AGL07667.1          NTRRHRGLARYFQVQLRQRAVRNPYPVNSLLNSLLTKQIPSIDGQPMDGTDNQVQDVTVF   360
AFN43006.1          NTRRHRGLARYFQVQLRQRAVRNPYPVNSLLNSLLTKQIPSIDGQPMDGTDNQVQDVTVF   360
AFN02459.1          NTRRHRGLARYFQVQLRQRAVRNPYPVNSLLNSLLTKQIPSIDGQPMDGTDNQVQDVTVF   360
AGL07652.1          NTRRHRGLARYFQVQLRQRAVRNPYPVNSLLNSLLTKQIPSIDGQPMDGTDNQVQDVTVF   360
AHB33045.1          NTRRHRGLARYFQVQLRQRAVRNPYPVNSLLNSLLTKQIPTIDGQPMDGTDNQVQDVTVF   360
YP_006495788.1      NTRRHRGLARYFQVQLRQRAVRNPYPVNSLLNSLLTKQIPSIDGQPMDGTDNQVQDVTVF   360
WHU50308.1          NTRRHRGLARYFQVQLRQRAVRNPYPVNSLLNSLLTKQIPSIDGQPMDGTDNQVQDVTVF   360
AFN02454.1          NTRRHRGLARYFQVQLRQRAVRNPYPVNSLLNSLLTKQIPSIDGQPMDGTDNQVQDVTVF   360
AHB33025.1          NTRRHRGLARYFQVQLRQRAVRNPYPVNSLLNSLLTKQIPTIDGQPMDGTDNQVQDVTVF   360
ASU87490.1          NTRRHRGLARYFQVQLRQRAVRNPYPVNSLLNSLLTKQIPTIDGQPMDGTDNQVQDVTVF   360
ASU87491.1          NTRRHRGLARYFQVQLRQRAVRNPYPVNSLLNSLLTKQIPTIDGQPMDGTDNQVQDVTVF   360
ASU87492.1          NTRRHRGLARYFQVQLRQRAVRNPYPVNSLLNSLLTKQIPTIDGQPMDGTDNQVQDVTVF   360
AHB33030.1          NTRRHRGLARYFQVQLRQRAVRNPYPVNSLLNSLLTKQIPTIDGQPMDGTDNQVQDVTVF   360
AHB33035.1          NTRRHRGLARYFQVQLRQRAVRNPYPVNSLLNSLLTKQIPTIDGQPMDGTDNQVQDVTVF   360
AHB33040.1          NTRRHRGLARYFQVQLRQRAVRNPYPVNSLLNSLLTKQIPTIDGQPMDGTDNQVQDVTVF   360
WHU50311.1          NTRRYRGLARYFQVQLRQRAVRNPYPVNSLLNSLLTKQIPSIDGQPMDGTDNQVQDVTVF   360
AKI28601.1          NTRRYRGLARYFQVQLRQRAVRNPYPVNSLLNSLLTKQIPSIDGQPMDGTDNQVQDVTVF   360
                    ****:***********************************:*******************

AHB33020.1          QGTEPLPGDPTLTRHMDLRCCPGTPVTDMPSDDTQTPVAPAAR   403
ASU87489.1          QGTEPLPGDPTLTRHMDLRCCPGTPVTDMPSDDTPTPVAPAAR   403
AHF70972.1          QGTEPLPGDPTLTRHMDLRCCPGTPVTDMPSDDTPTPVAPAAR   403
AGL07627.1          QGTEPLPGDPTLTRHMDLRCCPGTPVTDMPSDDTPTPVAPAAR   403
AGL07632.1          QGTEPLPGDPTLTRHMDLRCCPGTPVTDMPSDDTPTPVAPAAR   403
AGL07637.1          QGTEPLPGDPTLTRHMDLRCCPGTPVTDMPSDDTPTPVAPAAR   403
AGL07642.1          QGTEPLPGDPTLTRHMDLRCCPGTPVTDMPSDDTPTPVAPAAR   403
AGL07647.1          QGTEPLPGDPTLTRHMDLRCCPGTPVTDMPSDDTPTPVAPAAR   403
AGL07657.1          QGTEPLPGDPTLTRHMDLRCCPGTPVTDMPSDDTPTPVAPAAR   403
AGL07662.1          QGTEPLPGDPTLTRHMDLRCCPGTPVTDMPSDDTPTPVAPAAR   403
AGL07667.1          QGTEPLPGDPTLTRHMDLRCCPGTPVTDMPSDDTPTPVAPAAR   403
AFN43006.1          QGTEPLPGDPTLTRHMDLRCCPGTPVTDMPSDDTPTPVAPAAR   403
AFN02459.1          QGTEPLPGDPTLTRHMDLRCCPGTPVTDMPSDDTPTPVAPAAR   403
AGL07652.1          QGTEPLPGDPTLTRHMDLRCCPGTPVTDMPSDDTPTPVAPAAR   403
AHB33045.1          QGTEPLPGDPTLTRHMDLRCCPGTPVTDMPSDDTPTPVAPAAR   403
YP_006495788.1      QGTEPLPGDPTLTRHMDLRCCPGTPVTDMPSDDTETPVAPAAR   403
WHU50308.1          QGTEPLPGDPTLTRHMDLRCCPGTPVTDMPSDDTETPVAPAAR   403
AFN02454.1          QGTEPLPGDPTLTRHMDLRCCPGTPVTDMPSDDTETPVAPAAR   403
AHB33025.1          QGTEPLPGDPTLTRHMDLRCCPGTPVTDMPSDDSETPVAPAAR   403
ASU87490.1          QGTEPLPGDPTLTRHMDLRCCPGTPVTDMPSDDTETPVAPAAR   403
ASU87491.1          QGTEPLPGDPTLTRHMDLRCCPGTPVTDMPSDDTETPVAPAAR   403
ASU87492.1          QGTEPLPGDPTLTRHMDLRCCPGTPVTDMPSDDTETPVAPAAR   403
AHB33030.1          QGTEPLPGDPTLTRHMDLRCCPGTPVTDMPSDDTETPVAPAAR   403
AHB33035.1          QGTEPLPGDPTLTRHMDLRCCPGTPVTDMPSDDTETPVAPAAR   403
```

```
AHB33040.1          QGTEPLPGDPTLTRHMDLRCCPGTPVTDMPSDDTETPVAPAAR  403
WHU50311.1          QGTEPLPGDPTLTRHMDLRCCPGTPVTDMPSDDTPTPVAPAAR  403
AKI28601.1          QGTEPLPGDPTLTRHMDLRCCPGTPVTDMPSDDTPTPVAPAAR  403
                    **********************************:********
```
