# Supplementary material for: The Conserved YPX3L Motif in the BK Polyomavirus VP1 Protein Is Important for Viral Particle Assembly but Not for Its Secretion into Extracellular Vesicles
Source: Viruses. 2024 Jul 13;16(7):1124. doi: 10.3390/v16071124 (PMC11281352; doi:10.3390/v16071124)
Supplement: Supplementary file 1 [file viruses-16-01124-s001.zip › HPyV 11 alignment.pdf]

CLUSTAL O(1.2.4) multiple sequence alignment

```
WYC28921.1          MAPKRKSRCTSQTCPMDTCNPRRRRCEYDPCQKTVCPPSKPLPPPTCVPRIIAKGGMEVL   60
WYC28927.1          MAPKRKSRCTSQTCPMDTCNPRRRRCEYDPCQKTVCPPSKPLPPPTCVPRIIAKGGMEVL   60
AIW82378.1          MAPKRKSRCTSQTCPMDTCNPRRRRCEYDPCQKTVCPPSKPLPPPTCVPRIIAKGGMEVL   60
AIC33526.1          MAPKRKSRCTSQTCPMDTCNPRRRRCEYDPCQKTVCPPSKPLPPPTCVPRIIAKGGMEVL   60
AGV79295.1          MAPKRKSRCTSQTCPMDTCNPRRRRCEYDPCQKTVCPPSKPLPPPTCVPRIIAKGGMEVL   60
AMQ77270.1          MAPKRKSRCTSQTCPMDTCNPRRRRCEYDPCQKSLCPPPKPLPPPSCVPRIIAKGGMEVL   60
AGC03175.1          MAPKRKSRCTSQTCPMDTCNPRRRRCEYDPCQKSLCPPPKPLPPPTCVPRIIAKGGMEVL   60
YP_007354884.1      MAPKRKSRCTSQTCPMDTCNPRRRRCEYDPCQKSVCPPPKPLPPPTCVPRIIAKGGMEVL   60
AGC03169.1          MAPKRKSRCTSQTCPMDTCNPRRRRCEYDPCQKSVCPPPKPLPPPTCVPRIIAKGGMEVL   60
AIC33519.1          MAPKRKSRCTSQTCPMDTCNPRRRRCEYDPCQKSVCPPPKPLPPPTCVPRIIAKGGMEVL   60
                    *********************************::*** ******:**************

WYC28921.1          DIQVGPDAVLTVEAVLQPRMGNNDPTSQWYGFSDPVTVTAAPTAPSLPTYSFAKISLPPL   120
WYC28927.1          DIQVGPDAVLTVEAVLQPRMGNNDPTSQWYGFSDPVTVTAAPTAPSLPTYSFAKISLPPL   120
AIW82378.1          DIQVGPDAVLTVEAVLQPRMGNNDPTSQWYGFSDPVTVTATPTAPSLPTYSFAKISLPPL   120
AIC33526.1          DIQVGPDAVLTVEAVLQPRMGNNDPTSQWYGFSDPVTVTATPTAPSLPTYSFAKISLPPL   120
AGV79295.1          DIQVGPDAVLTVEAVLQPRMGNNDPTSQWYGFSDPVTVTATPTAPSLPTYSFAKISLPPL   120
AMQ77270.1          DIQVGPDAVLTVEAVLQPRMGNNDPTSAWYGFSDPVTVTATPAAPTLPTYSFAKINLPPL   120
AGC03175.1          DIQVGPDAVLTVEAVLQPRMGNNDPTSAWYGFSDPVTVTATPAAPTLPTYSFAKINLPPL   120
YP_007354884.1      DIQVGPDAVLTVEAVLQPRMGNNDPTSAWYGFSDPVTVTATPAAPTLPTYSFAKINLPPL   120
AGC03169.1          DIQVGPDAVLTVEAVLQPRMGNNDPTSAWYGFSDPVTVTATPAAPTLPTYSFAKINLPPL   120
AIC33519.1          DIQVGPDAVLTVEAVLQPRMGNNDPTSAWYGFSDPVTVTATPAAPTLPTYSFAKISLPPL   120
                    *************************** ************:*:**:*********.****

WYC28921.1          NENLTCDTLTLWEAVSLKTSLLGISVLISGHTPGTPPADRAPTNLIEGPAFHFYSVSGQP   180
WYC28927.1          NENLTCDTLTLWEAVSLKTSLLGISVLISGHTPGTPPADRAPTNLIEGPAFHFYSVSGQP   180
AIW82378.1          NENLTCDTLLLWEAVSLKTSLLGISVLISGHTPGTPPADRAPTNLIEGPTFHFYSVSGQP   180
AIC33526.1          NENLTCDTLLLWEAVSLKTSLLGISVLISGHTPGTPPADRAPTNLIEGPAFHFYSVSGQP   180
AGV79295.1          NENLTCDTLLLWEAVSLKTSLLGISVLISGHTPGTPPADRAPTNLIEGPAFHFYSVSGQP   180
AMQ77270.1          NENLTCDTLTLWEAVSLKTSILGISVLISGHTPGTPPADRAPANLIEGPSFHFYSVSGQP   180
AGC03175.1          NENLTCDTLTLWEAVSLKTSILGISVLISGHTPGTPPADRAPANLIEGPSFHFYSVSGQP   180
YP_007354884.1      NENLTCDTLTLWEAVSLKTSILGISVLISGHTPGTPPADRAPGTLIEGPSFHFYSVSGQP   180
AGC03169.1          NENLTCDTLTLWEAVSLKTSILGISVLISGHTPGTPPADRAPGTLIEGPSFHFYSVSGQP   180
AIC33519.1          NENLTCDTLTLWEAVSLKTSILGISVLISGHTPGTPPADRAPGNLIEGPSFHFYSVSGQP   180
                    ********* **********:******************** .*****:**********

WYC28921.1          LDLQYCTPNISVQYPETMGRFQNFTGVQQTYDSTYKAVLDKDGHYPVEAWFPDPFKNENG   240
WYC28927.1          LDLQYCTPNISVQYPETMGRFQNFTGVQQTYDSTYKAVLDKDGHYPVEAWFPDPFKNENG   240
AIW82378.1          LDLQYCTPNISVQYPETMGRFQNFTGVQQTYDSTYKAVLDKDGHYPVEAWFPDPFKNENG   240
AIC33526.1          LDLQYCTPNISVQYPETMGRFQNFTGVQQTYDSTYKAVLDKDGHYPVEAWFPDPFKNENG   240
AGV79295.1          LDLQYCTPNISVQYPETMGRFQNFTGVQQTYDSTYKAVLDKDGHYPVEAWFPDPFKNENG   240
AMQ77270.1          LDLQYCAPVITVQYPETMGRFQNFTGVQQTYDSTYKGVLDKDGHYPVEAWFPDPFKNENG   240
AGC03175.1          LDLQYCAPVITVQYPETMGRFQNFTGVQQTYDSTYKGVLDKDGHYPVEAWFPDPFKNENG   240
YP_007354884.1      LDLQYCAPVITVQYPETMGRFQNFTGVQQTYDSTYKGVLDKDGHYPVEAWFPDPFKNENG   240
AGC03169.1          LDLQYCAPVITVQYPETMGRFQNFTGVQQTYDSTYKGVLDKDGHYPVEAWFPDPFKNENG   240
AIC33519.1          LDLQYCAPVITVQYPETMGRFQNFTGVQQTYDSTYKGVLDKDGHYPVEAWFPDPFKNENG   240
                    ******:* *:*************************.***********************

WYC28921.1          RYFCTLTGGSTTPPVLNATNSVTTVLLDERGVGILCRGDGLYLGSADICGYFQTDTVNQK   300
WYC28927.1          RYFCTLTGGSTTPPVLNATNSVTTVLLDERGVGILCRGDGLYLGSADICGYFQTDTVNQK   300
AIW82378.1          RYFCTLTGGSTTPPVLNSTNSVTTVLLDERGVGILCRGDGLYLGSADICGYFQTDTVNQK   300
AIC33526.1          RYFCTLTGGSTTPPVLNSTNSVTTVLLDERGVGILCRGDGLYLGSADICGYFQTDTVNQK   300
AGV79295.1          RYFCTLTGGSTTPPVLNSTNSVTTVLLDERGVGILCRGDGLYLGSADICGYFQTDTVNQK   300
AMQ77270.1          RYFCTLTGGATTPPVLNSTNSVSTVLLDERGVGILCRGDGLYLGSADICGYFQTDTVNEK   300
AGC03175.1          RYFCTLTGGATTPPVLNSTNSVSTVLLDERGVGILCRGDGLYLGSADICGYFQTDTVNEK   300
YP_007354884.1      RYFCTLTGGATTPPVLNATNSVSTVLLDERGVGILCRGDGLYLGSADICGYFQTDTVNEK   300
AGC03169.1          RYFCTLTGGATTPPVLNATNSVSTVLLDERGVGILCRGDGLYLGSADICGYFQTDTVNEK   300
AIC33519.1          RYFCTLTGGATTPPVLNATNSVSTVLLDERGVGILCRGDGLYLGSADICGYFQTDTVNEK   300
                    *********:*******:****:***********************************:*

WYC28921.1          RHRGLARHFSVTLRQRNVRNPYPLNTLLSSLLTAQMPRVSGQPMQGSSSQMEEATITDGT   360
WYC28927.1          RHRGLARHFSVTLRQRNVRNPYPLNTLLSSLLTAQMPRVSGQPMQGSSSQMEEATITDGT   360
AIW82378.1          RHRGLARHFSVTLRQRNVRNPYPLNTLLSSLLTAQMPRVSGQPMQGSSSQMEEATITDGT   360
AIC33526.1          RHRGLARHFSVTLRQRNVRNPYPLNTLLSSLLTAQMPRVSGQPMQGSSSQMEEATITDGT   360
```

```
AGV79295.1          RHRGLARHFSVTLRQRNVRNPYPLNTLLSSLLTAQMPRVSGQPMQGSSSQMEEATITDGT   360
AMQ77270.1          RLRGLARHFSVTLRQRNVRNPYPLNTLLSSLLTAQMPRVSGQPMQGSSSQMEEATITDGS   360
AGC03175.1          RLRGLARHFSVTLRQRNVRNPYPLNTLLSSLLTAQMPRVSGQPMQGSSSQMEEATITDGS   360
YP_007354884.1      RLRGLARHFSVTLRQRNVRNPYPLNTLLSSLLTAQMPRVSGQPMQGSSSQMEEATITDGS   360
AGC03169.1          RLRGLARHFSVTLRQRNVRNPYPLNTLLSSLLTAQMPRVSGQPMQGSSSQMEEATITDGS   360
AIC33519.1          RLRGLARHFSVTLRQRNVRNPYPLNTLLSSLLTAQMPRVSGQPMQGSSSQMEEATITDGT   360
                    * *********************************************************:

WYC28921.1          EPLPGDPTLKRTLDARCCQGCPVAVNPLNPPPKDADPTDGA    401
WYC28927.1          EPLPGDPTLKRTLDARCCQGCPVAVNPLNPPPKDADPTDGA    401
AIW82378.1          EPLPGDPTLKRTLDARCCQGCPVAVNPLNPPPKDADPTDGA    401
AIC33526.1          EPLPGDPTLKRTLDARCCQGCPVAVNPLNPPPKDADPTDGA    401
AGV79295.1          EPLPGDPTLKRTLDARCCQGCPVAVNPLNPPPKDADPTDGA    401
AMQ77270.1          EPLPGDPTLRRTLDARCCQGCPVAVNPLNPPPKDADPTDGA    401
AGC03175.1          EPLPGDPTLRRTLDARCCQGCPVAVNPLNPPPKDADPTDGA    401
YP_007354884.1      EPLPGDPTLRRTLDARCCQGCPVAVNPLNPPPKDADPTDGA    401
AGC03169.1          EPLPGDPTLRRTLDARCCQGCPVAVNPLNPPPKDADPTDGA    401
AIC33519.1          EPLPGDPTLRRTLDARCCQGCPVAVNPLNPPPKDADPTDGA    401
                    *********:*******************************
```
