# Supplementary material for: The Conserved YPX3L Motif in the BK Polyomavirus VP1 Protein Is Important for Viral Particle Assembly but Not for Its Secretion into Extracellular Vesicles
Source: Viruses. 2024 Jul 13;16(7):1124. doi: 10.3390/v16071124 (PMC11281352; doi:10.3390/v16071124)
Supplement: Supplementary file 1 [file viruses-16-01124-s001.zip › HPyV 12 alignment.pdf]

CLUSTAL O(1.2.4) multiple sequence alignment

```
YP_007684355.2      MAPKRKTTCSSKKTCPQPSSVPKLIIKGGIEVLDVKTGDDSITQIEAFLNPRMGVNDETN   60
AGH58115.2          MAPKRKTTCSSKKTCPQPSSVPKLIIKGGIEVLDVKTGDDSITQIEAFLNPRMGVNDETN   60
                    ************************************************************

YP_007684355.2      TWYGFSEQVTVATARETDRPPKEQMPYYSCARIPLPLLNEDMTCNTLLMWEAVSVKTEVI   120
AGH58115.2          TWYGFSEQVTVATARETDRPPKEQMPYYSCARIPLPLLNEDMTCNTLLMWEAVSVKTEVI   120
                    ************************************************************

YP_007684355.2      GSNTLMNVHDYMTRTDNGVGHPVVGSTYHMFAVGGEPLDLQGIQQSHLVQYPEGLIVPKS   180
AGH58115.2          GSNTLMNVHDYMTRTDNGVGHPVVGSTYHMFAVGGEPLDLQGIQQSHLVQYPEGLIVPKS   180
                    ************************************************************

YP_007684355.2      VTDVTAKIQCLDPSAKAKLDKDGKYPIETWSPDPSRNENTRYFGNYYGGLTTPPVLTFTN   240
AGH58115.2          VTDVTAKIQCLDPSAKAKLDKDGKYPIETWSPDPSRNENTRYFGNYYGGLTTPPVLTFTN   240
                    ************************************************************

YP_007684355.2      TVTTILLDENGVGPLCKGDGLFLSCCDVMGWFTAGSGTHQRFRGLPRYFNVQLRKRAVRN   300
AGH58115.2          TVTTILLDENGVGPLCKGDGLFLSCCDVMGWFTAGSGTHQRFRGLPRYFNVQLRKRAVRN   300
                    ************************************************************

YP_007684355.2      PYPVSALLTSLFTNMMPRMSGQPMIGNKSQVEEVRVYEGLEQLPGDPDMERHIDEFGQEI   360
AGH58115.2          PYPVSALLTSLFTNMMPRMSGQPMIGNKSQVEEVRVYEGLEQLPGDPDMERHIDEFGQEI   360
                    ************************************************************

YP_007684355.2      TPVP      364
AGH58115.2          TPVP      364
                    ****
```
