# Supplementary material for: The Conserved YPX3L Motif in the BK Polyomavirus VP1 Protein Is Important for Viral Particle Assembly but Not for Its Secretion into Extracellular Vesicles
Source: Viruses. 2024 Jul 13;16(7):1124. doi: 10.3390/v16071124 (PMC11281352; doi:10.3390/v16071124)
Supplement: Supplementary file 1 [file viruses-16-01124-s001.zip › HPyV 13 alignment.pdf]

```
CLUSTAL O(1.2.4) multiple sequence alignment


YP_009030020.1      MAPPRKRPRCAPPSKVTCVPKKCPIPTPVPKLLVKGGVEVLNIITGPDATTEIELWLEPR   60
AHZ11645.1          MAPPRKRPRCAPPSKVTCVPKKCPIPTPVPKLLVKGGVEVLNIITGPDATTEIELWLEPR   60
                    ************************************************************

YP_009030020.1      MGVNAPTGDRKEWYGYSEVIHHADGYDNNLLSVQMPQYSCARVQLPMLNTDMTCETLMMW   120
AHZ11645.1          MGVNAPTGDRKEWYGYSEVIHHADGYDNNLLSVQMPQYSCARVQLPMLNTDMTCETLMMW   120
                    ************************************************************

YP_009030020.1      EAVSCKTEVVGIGSLISVHLLEAKMEAGPNSDGPSRPIEGMNYHMFAVGGEPLDLQGIES   180
AHZ11645.1          EAVSCKTEVVGIGSLISVHLLEAKMEAGPNSDGPSRPIEGMNYHMFAVGGEPLDLQGIES   180
                    ************************************************************

YP_009030020.1      NGQTKYATAIPAKSIHPNDIAKLPEEDKAQLQGLVPKAKAKLDKDGFYPVEEWSPDPSRN   240
AHZ11645.1          NGQTKYATAIPAKSIHPNDIAKLPEEDKAQLQGLVPKAKAKLDKDGFYPVEEWSPDPSRN   240
                    ************************************************************

YP_009030020.1      ENSRYYGSFVGGLQTPPNLQFTNAVSTVLLDENGVGPLCKGDGLFVSCADICGVLVKADN   300
AHZ11645.1          ENSRYYGSFVGGLQTPPNLQFTNAVSTVLLDENGVGPLCKGDGLFVSCADICGVLVKADN   300
                    ************************************************************

YP_009030020.1      EAIRYRGLPRYFKVTLRKRAVKNPYPITSLLGSLFTGLMPKMDGQPMSGPDAQVEEVRIY   360
AHZ11645.1          EAIRYRGLPRYFKVTLRKRAVKNPYPITSLLGSLFTGLMPKMDGQPMSGPDAQVEEVRIY   360
                    ************************************************************

YP_009030020.1      QGKEGLPADPDMKRYIDQFGQEQTPTPTPAAPAAVAALMEKWKEMYSEEHKFDQIHKWGF   420
AHZ11645.1          QGKEGLPADPDMKRYIDQFGQEQTPTPTPAAPAAVAALMEKWKEMYSEEHKFDQIHKWGF   420
                    ************************************************************

YP_009030020.1      SYPGYLFTEKESAKQKPREVQMPQKTQTQGTEVLTEQNLITEEYTTSASPPPSLGGTTDL   480
AHZ11645.1          SYPGYLFTEKESAKQKPREVQMPQKTQTQGTEVLTEQNLITEEYTTSASPPPSLGGTTDL   480
                    ************************************************************

YP_009030020.1      QKLPGTESV 489
AHZ11645.1          QKLPGTESV 489
                    *********
```
