# Supplementary material for: The Conserved YPX3L Motif in the BK Polyomavirus VP1 Protein Is Important for Viral Particle Assembly but Not for Its Secretion into Extracellular Vesicles
Source: Viruses. 2024 Jul 13;16(7):1124. doi: 10.3390/v16071124 (PMC11281352; doi:10.3390/v16071124)
Supplement: Supplementary file 1 [file viruses-16-01124-s001.zip › SV40 alignment.pdf]

CLUSTAL O(1.2.4) multiple sequence alignment

```
CBL79142.1        MKMAPAKRKGSCPGAAPKKPKEPVQVPKLVIEGGIEVLGVKTGVDSFTEVECFLNPQMGN  60
ABB29880.1        MKMAPAKRKGSCPGAAPKKPKEPVQVPKLVIKGGIEVLGVKTGVDSFTEVECFLNPQMGN  60
ABG45891.1        MKMAPTKRKGSCPGAAPKKPKEPVQVPKLVIKGGIEVLGVKTGVDSFTEVECFLNPQMGN  60
AAK29047.1        MKMAPTKRKGSCPGAAPKKPKEPVQVPKLVIKGGIEVLGVKTGVDSFTEVECFLNPQMGN  60
AAK29053.1        MKMAPTKRKGSCPGAAPKKPKEPVQVPKLVIKGGIEVLGVKTGVDSFTEVECFLNPQMGN  60
AAS45614.1        MKMAPTKRKGSCPGAAPKKPKEPVQVPKLVIKGGIEVLGVKTGVDSFTEVECFLNPQMGN  60
AAS16356.1        MKMAPTKRKGSCPGAAPKKPKEPVQVPKLVIKGGIEVLGVKTGVDSFTEVECFLNPQMGN  60
0509266A          ---APTKRKGSCPGAAPKKPKEPVEVPKLVIKGGIEVLGVKTGVDSFTEVECFLNPQMGN  57
P03087.2          --MAPTKRKGSCPGAAPKKPKEPVQVPKLVIKGGIEVLGVKTGVDSFTEVECFLNPQMGN  58
ABU86054.1        MKMAPTKRKGSCPGAAPKKPKEPVQVPKLVIKGGIEVLGVKTGVDSFTEVECFLNPQMGN  60
ABU62643.1        MKMAPTKRKGSCPGAAPKKPKEPVQVPKLVIKGGIEVLGVKTGVDSFTEVECFLNPQMGN  60
NP_043126.1       MKMAPTKRKGSCPGAAPKKPKEPVQVPKLVIKGGIEVLGVKTGVDSFTEVECFLNPQMGN  60
ABU62649.1        MKMAPTKRKGSCPGAAPKKPKEPVQVPKLVIKGGIEVLGVKTGVDSFTEVECFLNPQMGN  60
ABU86048.1        MKMAPTKRKGSCPGAAPKKPKEPVQVPKLVIKGGIEVLGVKTGVDSFTEVECFLNPQMGN  60
ABU86060.1        MKMAPTKRKGSCPGAAPKKPKEPVQVPKLVIKGGIEVLGVKTGVDSFTEVECFLNPQMGN  60
ABU86072.1        MKMAPTKRKGSCPGAAPKKPKEPVQVPKLVIKGGIEVLGVKTGVDSFTEVECFLNPQMGN  60
ABU86096.1        MKMAPTKRKGSCPGAAPKKPKEPVQVPKLVIKGGIEVLGVKTGVDSFTEVECFLNPQMGN  60
AAG39201.1        MKMAPTKRKGSCPGAAPKKPKEPVQVPKLVIKGGIEVLGVKTGVDSFTEVECFLNPQMGN  60
AAG39213.1        MKMAPTKRKGSCPGAAPKKPKEPVQVPKLVIKGGIEVLGVKTGVDSFTEVECFLNPQMGN  60
AAB59923.1        MKMAPTKRKGSCPGAAPKKPKEPVQVPKLVIKGGIEVLGVKTGVDSFTEVECFLNPQMGN  60
ABU86042.1        MKMAPTKRKGSCPGAAPKKPKEPVQVPKLVIKGGIEVLGVKTGVDSFTEVECFLNPQMGN  60
ABU86066.1        MKMAPTKRKGSCPGAAPKKPKEPVQVPKLVIKGGIEVLGVKTGVDSFTEVECFLNPQMGN  60
ABU86078.1        MKMAPTKRKGSCPGAAPKKPKEPVQVPKLVIKGGIEVLGVKTGVDSFTEVECFLNPQMGN  60
ABU86084.1        MKMAPTKRKGSCPGAAPKKPKEPVQVPKLVIKGGIEVLGVKTGVDSFTEVECFLNPQMGN  60
ABU86090.1        MKMAPTKRKGSCPGAAPKKPKEPVQVPKLVIKGGIEVLGVKTGVDSFTEVECFLNPQMGN  60
AAP30059.1        MKMAPTKRKGSCPGAAPKKPKEPVQVPKLVIKGGIEVLGVKTGVDSFTEVECFLNPQMGN  60
AAP30065.1        MKMAPTKRKGSCPGAAPKKPKEPVQVPKLVIKGGIEVLGVKTGVDSFTEVECFLNPQMGN  60
AAM77806.1        MKMAPTKRKGSCPGAAPKKPKEPVQVPKLVIKGGIEVLGVKTGVDSFTEVECFLNPQMGN  60
AAK19524.1        MKMAPTKRKGSCPGAAPKKPKEPVQVPKLVIKGGIEVLGVKTGVDSFTEVECFLNPQMGN  60
AAK01717.1        MKMAPTKRKGSCPGAAPKKPKEPVQVPKLVIKGGIEVLGVKTGVDSFTEVECFLNPQMGN  60
AAG39207.1        MKMAPTKRKGSCPGAAPKKPKEPVQVPKLVIKGGIEVLGVKTGVDSFTEVECFLNPQMGN  60
AAF28269.1        MKMAPTKRKGSCPGAAPKKPKEPVQVPKLVIKGGIEVLGVKTGVDSFTEVECFLNPQMGN  60
AAD43798.1        MKMAPTKRKGSCPGAAPKKPKEPVQVPKLVIKGGIEVLGVKTGVDSFTEVECFLNPQMGN  60
AAD43804.1        MKMAPTKRKGSCPGAAPKKPKEPVQVPKLVIKGGIEVLGVKTGVDSFTEVECFLNPQMGN  60
AAD38992.1        MKMAPTKRKGSCPGAAPKKPKEPVQVPKLVIKGGIEVLGVKTGVDSFTEVECFLNPQMGN  60
AAD39000.1        MKMAPTKRKGSCPGAAPKKPKEPVQVPKLVIKGGIEVLGVKTGVDSFTEVECFLNPQMGN  60
AAD38845.1        MKMAPTKRKGSCPGAAPKKPKEPVQVPKLVIKGGIEVLGVKTGVDSFTEVECFLNPQMGN  60
AAC59343.1        MKMAPTKRKGSCPGAAPKKPKEPVQVPKLVIKGGIEVLGVKTGVDSFTEVECFLNPQMGN  60
                     **:******************:******:****************************

CBL79142.1        PDEHQKGLSKSLAAEKQFTDDSPEKDQLPCYSVARIPLPNLNEDLTCGNILMWEAVTVKT  120
ABB29880.1        PDEHQKGLSKSLAAEKQFTDDSPEKDQLPCYSVARIPLPNLNEDLTCGNILMWEAVTVKT  120
ABG45891.1        PDEHQKGLSKSVAAEKQFTDDSPDKDQLPCYSVARIPLPNLNEDLTCGNILMWEAVTVKT  120
AAK29047.1        PDEHQKGLSKSLAAEKKFTDDSPDKDQLPCYSVARIPLPNLNEDLTCGNILMWEAVTVKT  120
AAK29053.1        PDEHQKGLSKSLAAEKKFTDDSPDKDQLPCYSVARIPLPNLNEDLTCGNILMWEAVTVKT  120
AAS45614.1        PDEHQKGLSKSLAAEKQFTNDSPDKDQLPCYSVARIPLPNLNEDLTCGNILMWEAVTVKT  120
AAS16356.1        PDEHQKGLSKSLAAEKQFTNDSPDKDQLPCYSVARIPLPNLNEDLTCGNILMWEAVTVKT  120
0509266A          PDEHQKGLSKSLAAEKQFTDDSPDKEQLPCYSVARIPLPNLNEDLTCGNILMWEAVTVKT  117
P03087.2          PDEHQKGLSKSLAAEKQFTDDSPDKEQLPCYSVARIPLPNINEDLTCGNILMWEAVTVKT  118
ABU86054.1        PDEHQKGLSKSLAAEKQFTDDSPDKDQLPCYSVARIPLPNLNEDLTCGNILMWEAVTVKT  120
ABU62643.1        PDEHQKGLSKSLAAEKQFTDDSPDKDQLPCYSVARIPLPNLNEDLTCGNILMWEAVTVKT  120
NP_043126.1       PDEHQKGLSKSLAAEKQFTDDSPDKEQLPCYSVARIPLPNLNEDLTCGNILMWEAVTVKT  120
ABU62649.1        PDEHQKGLSKSLAAEKQFTDDSPDKEQLPCYSVARIPLPNLNEDLTCGNILMWEAVTVKT  120
ABU86048.1        PDEHQKGLSKSLAAEKQFTDDSPDKEQLPCYSVARIPLPNLNEDLTCGNILMWEAVTVKT  120
ABU86060.1        PDEHQKGLSKSLAAEKQFTDDSPDKEQLPCYSVARIPLPNLNEDLTCGNILMWEAVTVKT  120
ABU86072.1        PDEHQKGLSKSLAAEKQFTDDSPDKEQLPCYSVARIPLPNLNEDLTCGNILMWEAVTVKT  120
ABU86096.1        PDEHQKGLSKSLAAEKQFTDDSPDKEQLPCYSVARIPLPNLNEDLTCGNILMWEAVTVKT  120
AAG39201.1        PDEHQKGLSKSLAAEKQFTDDSPDKEQLPCYSVARIPLPNLNEDLTCGNILMWEAVTVKT  120
AAG39213.1        PDEHQKGLSKSLAAEKQFTDDSPDKEQLPCYSVARIPLPNLNEDLTCGNILMWEAVTVKT  120
AAB59923.1        PDEHQKGLSKSLAAEKQFTDDSPDKEQLPCYSVARIPLPNLNEDLTCGNILMWEAVTVKT  120
ABU86042.1        PDEHQKGLSKSLAAEKQFTDDSPDKDQLPCYSVARIPLPNLNEDLTCGNILMWEAVTVKT  120
```

```
ABU86066.1          PDEHQKGLSKSLAAEKQFTDDSPDKDQLPCYSVARIPLPNLNEDLTCGNILMWEAVTVKT 120
ABU86078.1          PDEHQKGLSKSLAAEKQFTDDSPDKDQLPCYSVARIPLPNLNEDLTCGNILMWEAVTVKT 120
ABU86084.1          PDEHQKGLSKSLAAEKQFTDDSPDKDQLPCYSVARIPLPNLNEDLTCGNILMWEAVTVKT 120
ABU86090.1          PDEHQKGLSKSLAAEKQFTDDSPDKDQLPCYSVARIPLPNLNEDLTCGNILMWEAVTVKT 120
AAP30059.1          PDEHQKGLSKSLAAEKQFTDDSPDKDQLPCYSVARIPLPNLNEDLTCGNILMWEAVTVKT 120
AAP30065.1          PDEHQKGLSKSLAAEKQFTDDSPDKDQLPCYSVARIPLPNLNEDLTCGNILMWEAVTVKT 120
AAM77806.1          PDEHQKGLSKSLAAEKQFTDDSPDKDQLPCYSVARIPLPNLNEDLTCGNILMWEAVTVKT 120
AAK19524.1          PDEHQKGLSKSLAAEKQFTDDSPDKDQLPCYSVARIPLPNLNEDLTCGNILMWEAVTVKT 120
AAK01717.1          PDEHQKGLSKSLAAEKQFTDDSPDKDQLPCYSVARIPLPNLNEDLTCGNILMWEAVTVKT 120
AAG39207.1          PDEHQKGLSKSLAAEKQFTDDSPDKDQLPCYSVARIPLPNLNEDLTCGNILMWEAVTVKT 120
AAF28269.1          PDEHQKGLSKSLAAEKQFTDDSPDKDQLPCYSVARIPLPNLNEDLTCGNILMWEAVTVKT 120
AAD43798.1          PDEHQKGLSKSLAAEKQFTDDSPDKDQLPCYSVARIPLPNLNEDLTCGNILMWEAVTVKT 120
AAD43804.1          PDEHQKGLSKSLAAEKQFTDDSPDKDQLPCYSVARIPLPNLNEDLTCGNILMWEAVTVKT 120
AAD38992.1          PDEHQKGLSKSLAAEKQFTDDSPDKDQLPCYSVARIPLPNLNEDLTCGNILMWEAVTVKT 120
AAD39000.1          PDEHQKGLSKSLAAEKQFTDDSPDKDQLPCYSVARIPLPNLNEDLTCGNILMWEAVTVKT 120
AAD38845.1          PDEHQKGLSKSLAAEKQFTDDSPDKDQLPCYSVARIPLPNLNEDLTCGNILMWEAVTVKT 120
AAC59343.1          PDEHQKGLSKSLAAEKQFTDDSPDKDQLPCYSVARIPLPNLNEDLTCGNILMWEAVTVKT 120
                    ***********:****:**:***:*:**************:*******************

CBL79142.1          EVIGVTCMLNLHSGTQKTHENGAGKPIQGSNFHFFAIGGEPLELQGVLANYRTKYPALTV 180
ABB29880.1          EVIGVTCMLNLHSGTQKTHENGAGKPIQGSNFHFFAIGGEPLELQGVLANYRTKYPALTV 180
ABG45891.1          EVIGVTAMLNLHSGTQKTHENGAGKPIQGSNFHFFAVVGEPLELQGVLANCSTKYPAQTV 180
AAK29047.1          EVIGVTAMLNLHSGTQKTHENGAGKPIQGSNFHFFAVGGEPLELQGVLANYRTKYPAQTV 180
AAK29053.1          EVIGVTAMLNLHSGTQKTHENGAGKPIQGSNFHFFAVGGEPLELQGVLANYRTKYPAQTV 180
AAS45614.1          EVIGVTAMLNLHSGTQKTHENGAGKPIQGSNFQFFAVGGEPLELQGVLANYRTKYPAQTV 180
AAS16356.1          EVIGVTAMLNLHSGTQKTHENGAGKPIQGSNFQFFAVGGEPLELQGVLANYRTKYPAQTV 180
0509266A            EVIGVTAMLNLHSGTQKTHENGAGKPIQGSNFHFFAVGGEPLELQGVLANYRTKYPAQTV 177
P03087.2            EVIGVTAMLNLHSGTQKTHENGAGKPIQGSNFHFFAVGGEPLELQGVLANYRTKYPAQTV 178
ABU86054.1          EVIGVTAMLNLHSGTQKTHENGAGKPIQGSNFHFFAVGGEPLELQGVLANYRTKYPAQTV 180
ABU62643.1          EVIGVTAMLNLHSGTQKTYENGAGKPIQGSNFHFFAVGGEPLELQGVLANYRTKYPAQTV 180
NP_043126.1         EVIGVTAMLNLHSGTQKTHENGAGKPIQGSNFHFFAVGGEPLELQGVLANYRTKYPAQTV 180
ABU62649.1          EVIGVTAMLNLHSGTQKTHENGAGKPIQGSNFHFFAVGGEPLELQGVLANYRTKYPAQTV 180
ABU86048.1          EVIGVTAMLNLHSGTQKTHENGAGKPIQGSNFHFFAVGGEPLELQGVLANYRTKYPAQTV 180
ABU86060.1          EVIGVTAMLNLHSGTQKTHENGAGKPIQGSNFHFFAVGGEPLELQGVLANYRTKYPAQTV 180
ABU86072.1          EVIGVTAMLNLHSGTQKTHENGAGKPIQGSNFHFFAVGGEPLELQGVLANYRTKYPAQTV 180
ABU86096.1          EVIGVTAMLNLHSGTQKTHENGAGKPIQGSNFHFFAVGGEPLELQGVLANYRTKYPAQTV 180
AAG39201.1          EVIGVTAMLNLHSGTQKTHENGAGKPIQGSNFHFFAVGGEPLELQGVLANYRTKYPAQTV 180
AAG39213.1          EVIGVTAMLNLHSGTQKTHENGAGKPIQGSNFHFFAVGGEPLELQGVLANYRTKYPAQTV 180
AAB59923.1          EVIGVTAMLNLHSGTQKTHENGAGKPIQGSNFHFFAVGGEPLELQGVLANYRTKYPAQTV 180
ABU86042.1          EVIGVTAMLNLHSGTQKTHENGAGKPIQGSNFHFFAVGGEPLELQGVLANYRTKYPAQTV 180
ABU86066.1          EVIGVTAMLNLHSGTQKTHENGAGKPIQGSNFHFFAVGGEPLELQGVLANYRTKYPAQTV 180
ABU86078.1          EVIGVTAMLNLHSGTQKTHENGAGKPIQGSNFHFFAVGGEPLELQGVLANYRTKYPAQTV 180
ABU86084.1          EVIGVTAMLNLHSGTQKTHENGAGKPIQGSNFHFFAVGGEPLELQGVLANYRTKYPAQTV 180
ABU86090.1          EVIGVTAMLNLHSGTQKTHENGAGKPIQGSNFHFFAVGGEPLELQGVLANYRTKYPAQTV 180
AAP30059.1          EVIGVTAMLNLHSGTQKTHENGAGKPIQGSNFHFFAVGGEPLELQGVLANYRTKYPAQTV 180
AAP30065.1          EVIGVTAMLNLHSGTQKTHENGAGKPIQGSNFHFFAVGGEPLELQGVLANYRTKYPAQTV 180
AAM77806.1          EVIGVTAMLNLHSGTQKTHENGAGKPIQGSNFHFFAVGGEPLELQGVLANYRTKYPAQTV 180
AAK19524.1          EVIGVTAMLNLHSGTQKTHENGAGKPIQGSNFHFFAVGGEPLELQGVLANYRTKYPAQTV 180
AAK01717.1          EVIGVTAMLNLHSGTQKTHENGAGKPIQGSNFHFFAVGGEPLELQGVLANYRTKYPAQTV 180
AAG39207.1          EVIGVTAMLNLHSGTQKTHENGAGKPIQGSNFHFFAVGGEPLELQGVLANYRTKYPAQTV 180
AAF28269.1          EVIGVTAMLNLHSGTQKTHENGAGKPIQGSNFHFFAVGGEPLELQGVLANYRTKYPAQTV 180
AAD43798.1          EVIGVTAMLNLHSGTQKTHENGAGKPIQGSNFHFFAVGGEPLELQGVLANYRTKYPAQTV 180
AAD43804.1          EVIGVTAMLNLHSGTQKTHENGAGKPIQGSNFHFFAVGGEPLELQGVLANYRTKYPAQTV 180
AAD38992.1          EVIGVTAMLNLHSGTQKTHENGAGKPIQGSNFHFFAVGGEPLELQGVLANYRTKYPAQTV 180
AAD39000.1          EVIGVTAMLNLHSGTQKTHENGAGKPIQGSNFHFFAVGGEPLELQGVLANYRTKYPAQTV 180
AAD38845.1          EVIGVTAMLNLHSGTQKTHENGAGKPIQGSNFHFFAVGGEPLELQGVLANYRTKYPAQTV 180
AAC59343.1          EVIGVTAMLNLHSGTQKTHENGAGKPIQGSNFHFFAVGGEPLELQGVLANYRTKYPAQTV 180
                    ******.***********:*************:***: ************  ***** **

CBL79142.1          TPKNATSDSQQMNTDHKAVLDKDNAYPIECWVPDPSKNENTRYFGTYTGGENVPPVLHIT 240
ABB29880.1          TPKNATSDSQQMNTDHKAVLDKDNAYPIECWVPDPSKNENTRYFGTYTGGENVPPVLHIT 240
ABG45891.1          TPKNATVDSQQMNTDHKAVLDKDNAYPVECWVPDPSKNENTRYFGTYTGGENVPPVLHIT 240
AAK29047.1          TPKNATFDSQQMNTDHKAVLDKDNAYPVECWVPDPSKNENTRYFGTYTGGENVPPVLHIT 240
AAK29053.1          TPKNATFDSQQMNTDHKAVLDKDNAYPVECWVPDPSKNENTRYFGTYTGGENVPPVLHIT 240
```

```
AAS45614.1       TPKNATVDSQQMNTDHKAVLDKDNAYPVECWVPDPSKNENTRYFGTYTGGENVPPVLHIT  240
AAS16356.1       TPKNATVDSQQMNTDHKAVLDKDNAYPVECWVPDPSKNENTRYFGTYTGGENVPPVLHIT  240
0509266A         TPKNATVDSQQMNTDHKAVLDKDNAYPVECWVPDPSKNENTRYFGTYTGGENVPPVLHIT  237
P03087.2         TPKNATVDSQQMNTDHKAVLDKDNAYPVECWVPDPSKNENTRYFGTYTGGENVPPVLHIT  238
ABU86054.1       TPKNATVDSQQMNTDHKAVLDKDNAYPVECWVPDPSKNENTRYFGTYTGGENVPPVLHIT  240
ABU62643.1       TPKNATVDSQQMNTDHKAVLDKDNAYPVECWVPDPSKNENTRYFGTYTGGENVPPVLHIT  240
NP_043126.1      TPKNATVDSQQMNTDHKAVLDKDNAYPVECWVPDPSKNENTRYFGTYTGGENVPPVLHIT  240
ABU62649.1       TPKNATVDSQQMNTDHKAVLDKDNAYPVECWVPDPSKNENTRYFGTYTGGENVPPVLHIT  240
ABU86048.1       TPKNATVDSQQMNTDHKAVLDKDNAYPVECWVPDPSKNENTRYFGTYTGGENVPPVLHIT  240
ABU86060.1       TPKNATVDSQQMNTDHKAVLDKDNAYPVECWVPDPSKNENTRYFGTYTGGENVPPVLHIT  240
ABU86072.1       TPKNATVDSQQMNTDHKAVLDKDNAYPVECWVPDPSKNENTRYFGTYTGGENVPPVLHIT  240
ABU86096.1       TPKNATVDSQQMNTDHKAVLDKDNAYPVECWVPDPSKNENTRYFGTYTGGENVPPVLHIT  240
AAG39201.1       TPKNATVDSQQMNTDHKAVLDKDNAYPVECWVPDPSKNENTRYFGTYTGGENVPPVLHIT  240
AAG39213.1       TPKNATVDSQQMNTDHKAVLDKDNAYPVECWVPDPSKNENTRYFGTYTGGENVPPVLHIT  240
AAB59923.1       TPKNATVDSQQMNTDHKAVLDKDNAYPVECWVPDPSKNENTRYFGTYTGGENVPPVLHIT  240
ABU86042.1       TPKNATVDSQQMNTDHKAVLDKDNAYPVECWVPDPSKNENTRYFGTYTGGENVPPVLHIT  240
ABU86066.1       TPKNATVDSQQMNTDHKAVLDKDNAYPVECWVPDPSKNENTRYFGTYTGGENVPPVLHIT  240
ABU86078.1       TPKNATVDSQQMNTDHKAVLDKDNAYPVECWVPDPSKNENTRYFGTYTGGENVPPVLHIT  240
ABU86084.1       TPKNATVDSQQMNTDHKAVLDKDNAYPVECWVPDPSKNENTRYFGTYTGGENVPPVLHIT  240
ABU86090.1       TPKNATVDSQQMNTDHKAVLDKDNAYPVECWVPDPSKNENTRYFGTYTGGENVPPVLHIT  240
AAP30059.1       TPKNATVDSQQMNTDHKAVLDKDNAYPVECWVPDPSKNENTRYFGTYTGGENVPPVLHIT  240
AAP30065.1       TPKNATVDSQQMNTDHKAVLDKDNAYPVECWVPDPSKNENTRYFGTYTGGENVPPVLHIT  240
AAM77806.1       TPKNATVDSQQMNTDHKAVLDKDNAYPVECWVPDPSKNENTRYFGTYTGGENVPPVLHIT  240
AAK19524.1       TPKNATVDSQQMNTDHKAVLDKDNAYPVECWVPDPSKNENTRYFGTYTGGENVPPVLHIT  240
AAK01717.1       TPKNATVDSQQMNTDHKAVLDKDNAYPVECWVPDPSKNENTRYFGTYTGGENVPPVLHIT  240
AAG39207.1       TPKNATVDSQQMNTDHKAVLDKDNAYPVECWVPDPSKNENTRYFGTYTGGENVPPVLHIT  240
AAF28269.1       TPKNATVDSQQMNTDHKAVLDKDNAYPVECWVPDPSKNENTRYFGTYTGGENVPPVLHIT  240
AAD43798.1       TPKNATVDSQQMNTDHKAVLDKDNAYPVECWVPDPSKNENTRYFGTYTGGENVPPVLHIT  240
AAD43804.1       TPKNATVDSQQMNTDHKAVLDKDNAYPVECWVPDPSKNENTRYFGTYTGGENVPPVLHIT  240
AAD38992.1       TPKNATVDSQQMNTDHKAVLDKDNAYPVECWVPDPSKNENTRYFGTYTGGENVPPVLHIT  240
AAD39000.1       TPKNATVDSQQMNTDHKAVLDKDNAYPVECWVPDPSKNENTRYFGTYTGGENVPPVLHIT  240
AAD38845.1       TPKNATVDSQQMNTDHKAVLDKDNAYPVECWVPDPSKNENTRYFGTYTGGENVPPVLHIT  240
AAC59343.1       TPKNATVDSQQMNTDHKAVLDKDNAYPVECWVPDPSKNENTRYFGTYTGGENVPPVLHIT  240
                 ****** ********************:********************************

CBL79142.1       NTATTVLLDEQGAGPLCKADSLYVSAADICGLFTNTSGTQQWKGLPRYFKITLRKRSVKN  300
ABB29880.1       NTATTVLLDEQGVGPLCKADSLYVSAADICGLFTNTSGTQQWKGLPRYFKITLRKRSVKN  300
ABG45891.1       NTATTVLLDEQGVGPLCKADSLYVSAVDICGLFTNTSGTQQWKGLPRYFKITLRKRSVKN  300
AAK29047.1       NTATTVLLDEQGVGPLCKADSLYVSAVDICGLFTNTSGTQQWKGLPRYFKITLRKRSVKN  300
AAK29053.1       NTATTVLLDEQGVGPLCKADSLYVSAVDICGLFTNTSGTQQWKGLPRYFKITLRKRSVKN  300
AAS45614.1       NTATTVLLDEQGVGPLCKADSLYVSAVDICGLFTNTSGTQQWKGLPRYFKITLRKRSVKN  300
AAS16356.1       NTATTVLLDEQGVGPLCKADSLYVSAVDICGLFTNTSGTQQWKGLPRYFKITLRKRSVKN  300
0509266A         NTATTVLLDEQGVGPLCKADSLYVSAVDICGLFTNTSGTQQWKGLPRYFKITLRKRSVKN  297
P03087.2         NTATTVLLDEQGVGPLCKADSLYVSAVDICGLFTNTSGTQQWKGLPRYFKITLRKRSVKN  298
ABU86054.1       NTATTVLLDEQGVGPLYKADSLYVSAVDICGLFTNTSGTQQWKGLPRYFKITLRKRSVKN  300
ABU62643.1       NTATTVLLDEQGVGPLCKADSLYVSAVDICGLFTNTSGTQQWKGLPRYFKITLRKRSVKN  300
NP_043126.1      NTATTVLLDEQGVGPLCKADSLYVSAVDICGLFTNTSGTQQWKGLPRYFKITLRKRSVKN  300
ABU62649.1       NTATTVLLDEQGVGPLCKADSLYVSAVDICGLFTNTSGTQQWKGLPRYFKITLRKRSVKN  300
ABU86048.1       NTATTVLLDEQGVGPLCKADSLYVSAVDICGLFTNTSGTQQWKGLPRYFKITLRKRSVKN  300
ABU86060.1       NTATTVLLDEQGVGPLCKADSLYVSAVDICGLFTNTSGTQQWKGLPRYFKITLRKRSVKN  300
ABU86072.1       NTATTVLLDEQGVGPLCKADSLYVSAVDICGLFTNTSGTQQWKGLPRYFKITLRKRSVKN  300
ABU86096.1       NTATTVLLDEQGVGPLCKADSLYVSAVDICGLFTNTSGTQQWKGLPRYFKITLRKRSVKN  300
AAG39201.1       NTATTVLLDEQGVGPLCKADSLYVSAVDICGLFTNTSGTQQWKGLPRYFKITLRKRSVKN  300
AAG39213.1       NTATTVLLDEQGVGPLCKADSLYVSAVDICGLFTNTSGTQQWKGLPRYFKITLRKRSVKN  300
AAB59923.1       NTATTVLLDEQGVGPLCKADSLYVSAVDICGLFTNTSGTQQWKGLPRYFKITLRKRSVKN  300
ABU86042.1       NTATTVLLDEQGVGPLCKADSLYVSAVDICGLFTNTSGTQQWKGLPRYFKITLRKRSVKN  300
ABU86066.1       NTATTVLLDEQGVGPLCKADSLYVSAVDICGLFTNTSGTQQWKGLPRYFKITLRKRSVKN  300
ABU86078.1       NTATTVLLDEQGVGPLCKADSLYVSAVDICGLFTNTSGTQQWKGLPRYFKITLRKRSVKN  300
ABU86084.1       NTATTVLLDEQGVGPLCKADSLYVSAVDICGLFTNTSGTQQWKGLPRYFKITLRKRSVKN  300
ABU86090.1       NTATTVLLDEQGVGPLCKADSLYVSAVDICGLFTNTSGTQQWKGLPRYFKITLRKRSVKN  300
AAP30059.1       NTATTVLLDEQGVGPLCKADSLYVSAVDICGLFTNTSGTQQWKGLPRYFKITLRKRSVKN  300
AAP30065.1       NTATTVLLDEQGVGPLCKADSLYVSAVDICGLFTNTSGTQQWKGLPRYFKITLRKRSVKN  300
AAM77806.1       NTATTVLLDEQGVGPLCKADSLYVSAVDICGLFTNTSGTQQWKGLPRYFKITLRKRSVKN  300
AAK19524.1       NTATTVLLDEQGVGPLCKADSLYVSAVDICGLFTNTSGTQQWKGLPRYFKITLRKRSVKN  300
```

```
AAK01717.1       NTATTVLLDEQGVGPLCKADSLYVSAVDICGLFTNTSGTQQWKGLPRYFKITLRKRSVKN 300
AAG39207.1       NTATTVLLDEQGVGPLCKADSLYVSAVDICGLFTNTSGTQQWKGLPRYFKITLRKRSVKN 300
AAF28269.1       NTATTVLLDEQGVGPLCKADSLYVSAVDICGLFTNTSGTQQWKGLPRYFKITLRKRSVKN 300
AAD43798.1       NTATTVLLDEQGVGPLCKADSLYVSAVDICGLFTNTSGTQQWKGLPRYFKITLRKRSVKN 300
AAD43804.1       NTATTVLLDEQGVGPLCKADSLYVSAVDICGLFTNTSGTQQWKGLPRYFKITLRKRSVKN 300
AAD38992.1       NTATTVLLDEQGVGPLCKADSLYVSAVDICGLFTNTSGTQQWKGLPRYFKITLRKRSVKN 300
AAD39000.1       NTATTVLLDEQGVGPLCKADSLYVSAVDICGLFTNTSGTQQWKGLPRYFKITLRKRSVKN 300
AAD38845.1       NTATTVLLDEQGVGPLCKADSLYVSAVDICGLFTNTSGTQQWKGLPRYFKITLRKRSVKN 300
AAC59343.1       NTATTVLLDEQGVGPLCKADSLYVSAVDICGLFTNTSGTQQWKGLPRYFKITLRKRSVKN 300
                 ************.*** *********.*********************************

CBL79142.1       PYPISFLLSDLINRRTQRVDGQPMIGMSSQVEEVRVYEDTEELPGDPDMIRYIDEFGQTT 360
ABB29880.1       PYPISFLLSDLINRRTQRVDGQPMIGMSSQVEEVRVYEDTEELPGDPDMIRYIDEFGQTT 360
ABG45891.1       PYPISFLLSDLINRRTQRVDGQPMIGMSSQVEEVRVYEDTEELPGDPDMIRYIDEFGQTT 360
AAK29047.1       PYPISFLLSDLINRRTQRVDGQPMIGMSSQVEEVRVYEDTEELPGDPDMIRYIDEFGQTT 360
AAK29053.1       PYPISFLLSDLINRRTQRVDGQPMIGMSSQVEEVRVYEDTEELPGDPDMIRYIDEFGQTT 360
AAS45614.1       PYPISFLLSDLINRRTQRVDGQPMIGMSSQVEEVRVYEDTEELPGDPDMIRYIDEFGQTT 360
AAS16356.1       PYPISFLLSDLINRRTQRVDGQPMIGMSSQVEEVRVYEDTEELPGDPDMIRYIDEFGQTT 360
0509266A         PYPISFLLSDLINRRTQRVDGQPMIGMSSQVEEVRVYEDTEELPGDPDMIRYIDEFGQTT 357
P03087.2         PYPISFLLSDLINRRTQRVDGQPMIGMSSQVEEVRVYEDTEELPGDPDMIRYIDEFGQTT 358
ABU86054.1       PYPISFLLSDLINRRTQRVDGQPMIGMSSQVEEVRVYEDTEELPGDPDMIRYIDEFGQTT 360
ABU62643.1       PYPISFLLSDLINRRTQRVDGQPMIGMSSQVEEVRVYEDTEELPGDPDMIRYIDEFGQTT 360
NP_043126.1      PYPISFLLSDLINRRTQRVDGQPMIGMSSQVEEVRVYEDTEELPGDPDMIRYIDEFGQTT 360
ABU62649.1       PYPISFLLSDLINRRTQRVDGQPMIGMSSQVEEVRVYEDTEELPGDPDMIRYIDEFGQTT 360
ABU86048.1       PYPISFLLSDLINRRTQRVDGQPMIGMSSQVEEVRVYEDTEELPGDPDMIRYIDEFGQTT 360
ABU86060.1       PYPISFLLSDLINRRTQRVDGQPMIGMSSQVEEVRVYEDTEELPGDPDMIRYIDEFGQTT 360
ABU86072.1       PYPISFLLSDLINRRTQRVDGQPMIGMSSQVEEVRVYEDTEELPGDPDMIRYIDEFGQTT 360
ABU86096.1       PYPISFLLSDLINRRTQRVDGQPMIGMSSQVEEVRVYEDTEELPGDPDMIRYIDEFGQTT 360
AAG39201.1       PYPISFLLSDLINRRTQRVDGQPMIGMSSQVEEVRVYEDTEELPGDPDMIRYIDEFGQTT 360
AAG39213.1       PYPISFLLSDLINRRTQRVDGQPMIGMSSQVEEVRVYEDTEELPGDPDMIRYIDEFGQTT 360
AAB59923.1       PYPISFLLSDLINRRTQRVDGQPMIGMSSQVEEVRVYEDTEELPGDPDMIRYIDEFGQTT 360
ABU86042.1       PYPISFLLSDLINRRTQRVDGQPMIGMSSQVEEVRVYEDTEELPGDPDMIRYIDEFGQTT 360
ABU86066.1       PYPISFLLSDLINRRTQRVDGQPMIGMSSQVEEVRVYEDTEELPGDPDMIRYIDEFGQTT 360
ABU86078.1       PYPISFLLSDLINRRTQRVDGQPMIGMSSQVEEVRVYEDTEELPGDPDMIRYIDEFGQTT 360
ABU86084.1       PYPISFLLSDLINRRTQRVDGQPMIGMSSQVEEVRVYEDTEELPGDPDMIRYIDEFGQTT 360
ABU86090.1       PYPISFLLSDLINRRTQRVDGQPMIGMSSQVEEVRVYEDTEELPGDPDMIRYIDEFGQTT 360
AAP30059.1       PYPISFLLSDLINRRTQRVDGQPMIGMSSQVEEVRVYEDTEELPGDPDMIRYIDEFGQTT 360
AAP30065.1       PYPISFLLSDLINRRTQRVDGQPMIGMSSQVEEVRVYEDTEELPGDPDMIRYIDEFGQTT 360
AAM77806.1       PYPISFLLSDLINRRTQRVDGQPMIGMSSQVEEVRVYEDTEELPGDPDMIRYIDEFGQTT 360
AAK19524.1       PYPISFLLSDLINRRTQRVDGQPMIGMSSQVEEVRVYEDTEELPGDPDMIRYIDEFGQTT 360
AAK01717.1       PYPISFLLSDLINRRTQRVDGQPMIGMSSQVEEVRVYEDTEELPGDPDMIRYIDEFGQTT 360
AAG39207.1       PYPISFLLSDLINRRTQRVDGQPMIGMSSQVEEVRVYEDTEELPGDPDMIRYIDEFGQTT 360
AAF28269.1       PYPISFLLSDLINRRTQRVDGQPMIGMSSQVEEVRVYEDTEELPGDPDMIRYIDEFGQTT 360
AAD43798.1       PYPISFLLSDLINRRTQRVDGQPMIGMSSQVEEVRVYEDTEELPGDPDMIRYIDEFGQTT 360
AAD43804.1       PYPISFLLSDLINRRTQRVDGQPMIGMSSQVEEVRVYEDTEELPGDPDMIRYIDEFGQTT 360
AAD38992.1       PYPISFLLSDLINRRTQRVDGQPMIGMSSQVEEVRVYEDTEELPGDPDMIRYIDEFGQTT 360
AAD39000.1       PYPISFLLSDLINRRTQRVDGQPMIGMSSQVEEVRVYEDTEELPGDPDMIRYIDEFGQTT 360
AAD38845.1       PYPISFLLSDLINRRTQRVDGQPMIGMSSQVEEVRVYEDTEELPGDPDMIRYIDEFGQTT 360
AAC59343.1       PYPISFLLSDLINRRTQRVDGQPMIGMSSQVEEVRVYEDTEELPGDPDMIRYIDEFGQTT 360
                 ************************************************************

CBL79142.1       TRMQ 364
ABB29880.1       TRMQ 364
ABG45891.1       TRMQ 364
AAK29047.1       TRMQ 364
AAK29053.1       TRMQ 364
AAS45614.1       TRMQ 364
AAS16356.1       TRMQ 364
0509266A         TRMQ 361
P03087.2         TRMQ 362
ABU86054.1       TRMQ 364
ABU62643.1       TRMQ 364
NP_043126.1      TRMQ 364
ABU62649.1       TRMQ 364
```

```
ABU86048.1         TRMQ  364
ABU86060.1         TRMQ  364
ABU86072.1         TRMQ  364
ABU86096.1         TRMQ  364
AAG39201.1         TRMQ  364
AAG39213.1         TRMQ  364
AAB59923.1         TRMQ  364
ABU86042.1         TRMQ  364
ABU86066.1         TRMQ  364
ABU86078.1         TRMQ  364
ABU86084.1         TRMQ  364
ABU86090.1         TRMQ  364
AAP30059.1         TRMQ  364
AAP30065.1         TRMQ  364
AAM77806.1         TRMQ  364
AAK19524.1         TRMQ  364
AAK01717.1         TRMQ  364
AAG39207.1         TRMQ  364
AAF28269.1         TRMQ  364
AAD43798.1         TRMQ  364
AAD43804.1         TRMQ  364
AAD38992.1         TRMQ  364
AAD39000.1         TRMQ  364
AAD38845.1         TRMQ  364
AAC59343.1         TRMQ  364
                   ****
```
